# Comparison of ozone measurement methods in biomass burning smoke: an evaluation under field and laboratory conditions

**DOI:** 10.5194/amt-14-1783-2021

**Published:** 2021-03-04

**Authors:** Russell W. Long, Andrew Whitehill, Andrew Habel, Shawn Urbanski, Hannah Halliday, Maribel Colón, Surender Kaushik, Matthew S. Landis

**Affiliations:** 1 Center for Environmental Measurement and Modeling, Office of Research and Development, United States Environmental Protection Agency, Research Triangle Park, North Carolina, United States of America; 2 Jacobs Technology Inc., Research Triangle Park, North Carolina, United States of America; 3 U.S. Forest Service, Rocky Mountain Research Station, Missoula, Montana, United States of America

## Abstract

In recent years wildland fires in the United States have had significant impacts on local and regional air quality and negative human health outcomes. Although the primary health concerns from wildland fires come from fine particulate matter (PM_2.5_), large increases in ozone (O_3_) have been observed downwind of wildland fire plumes (DeBell et al., 2004; Bytnerowicz et al., 2010; Preisler et al., 2010; Jaffe et al., 2012; Bytnerowicz et al., 2013; Jaffe et al., 2013; Lu et al., 2016; Lindaas et al., 2017; McClure and Jaffe, 2018; Liu et al., 2018; Baylon et al., 2018; Buysse et al., 2019). Conditions generated in and around wildland fire plumes, including the presence of interfering chemical species, can make the accurate measurement of O_3_ concentrations using the ultraviolet (UV) photometric method challenging if not impossible. UV photometric method instruments are prone to interferences by volatile organic compounds (VOCs) that are present at high concentrations in wildland fire smoke. Four different O_3_ measurement methodologies were deployed in a mobile sampling platform downwind of active prescribed grassland fire lines in Kansas and Oregon and during controlled chamber burns at the United States Forest Service, Rocky Mountain Research Station Fire Sciences Laboratory in Missoula, Montana. We demonstrate that the Federal Reference Method (FRM) nitric oxide (NO) chemiluminescence monitors and Federal Equivalent Method (FEM) gas-phase (NO) chemical scrubber UV photometric O_3_ monitors are relatively interference-free, even in near-field combustion plumes. In contrast, FEM UV photometric O_3_ monitors using solid-phase catalytic scrubbers show positive artifacts that are positively correlated with carbon monoxide (CO) and total gas-phase hydrocarbon (THC), two indicator species of biomass burning. Of the two catalytic scrubber UV photometric methods evaluated, the instruments that included a Nafion® tube dryer in the sample introduction system had artifacts an order of magnitude smaller than the instrument with no humidity correction. We hypothesize that Nafion®-permeating VOCs (such as aromatic hydrocarbons) could be a significant source of interference for catalytic scrubber UV photometric O_3_ monitors and that the inclusion of a Nafion® tube dryer assists with the mitigation of these interferences. The chemiluminescence FRM method is highly recommended for accurate measurements of O_3_ in wildland fire plume studies and at regulatory ambient monitoring sites frequently impacted by wildland fire smoke.

## 1 Introduction

Ground-level ozone (O_3_) is a secondary air pollutant generated from the photochemical interactions of nitrogen oxides (NO_*x*_) and volatile organic compounds (VOCs). The most robust methods for O_3_ measurements are based on chemiluminescence reactions with ethylene (ET-CL, for ethylene chemiluminescence) or nitric oxide (NO-CL, for nitric oxide chemiluminescence) (Long et al., 2014). The overall reaction mechanism for ET-CL generally proceeds as detailed in Eqs. (1–2):

(1) $${\text{C}}_{2}{\text{H}}_{4}+{\text{O}}_{3}\to {\text{H}}_{2}{\text{CO}}^{+}+\text{other}\;\text{products},$$

(2) $${\text{H}}_{2}{\text{CO}}^{*}\to {\text{H}}_{2}\text{CO}+hv.$$

The reaction generates electronically activated formaldehyde (H_2_CO*) which luminesces in the high-ultraviolet (UV) to visible portion of the spectrum (380–550 nm) and vibrationally activated hydroxide ions which luminesce in the visible light to the low-infrared (IR) portion of the spectrum (550–800 nm). The number of photons emitted during the reaction is directly proportional to the amount of O_3_ present and is counted by a photomultiplier tube (PMT), with its response centered at 440 nm. Then the count is converted to O_3_ concentration. The ET-CL method requires a constant supply of ethylene for continuous operation. NO-chemiluminescence analyzers measure O_3_ concentrations using the principle that the dry, gas-phase reaction between NO and O_3_ generates nitrogen dioxide in an electronically excited state (NO_2_*) and oxygen (O_2_) (Ollison et al., 2013; Boylan et al., 2014). As each unstable NO_2_* molecule returns to a lower energy state (NO_2_), it emits a photon (hv). The reaction causes luminescence in a broadband spectrum ranging from visible light to infrared light (approximately 590–2800 nm). The two-step gas-phase reaction proceeds as detailed in Eqs. (3–4):

(3) $$\text{NO}+{\text{O}}_{3}\to {\text{NO}}_{2}^{*}+{\text{O}}_{2},$$

(4) $${\text{NO}}_{2}^{*}\to {\text{NO}}_{2}+hv.$$

The ET-CL method is no longer used nor produced commercially and has been replaced by the NO-CL method. Similar to the ET-CL method, the NO-CL method requires a constant supply of gas, in this case NO, for continuous operation. Both the ET-CL and NO-CL methods are subject to slight interferences by water vapor. However, these potential interferences can be eliminated through the use of a Nafion®-based dryer or equivalent sample water vapor treatment system. The ET-CL method was promulgated as the Federal Reference Method (FRM) for measuring O_3_ in the atmosphere in 1971, and the NO-CL method was promulgated as the FRM in 2015 (U.S. EPA, 2015).

While the chemiluminescence method for measuring O_3_ is technically robust and free of analytical artifacts (Long et al., 2014), it is not widely used in the United States. Instead, Federal Equivalent Methods (FEM) based upon UV photometry are employed at the majority of O_3_ regulatory monitoring locations. According to July 2020 data from the United States Environmental Protection Agency (EPA) Air Quality System (AQS) database, the UV photometric method represents 99% of the roughly 1200 instruments deployed in network monitoring for O_3_ National Ambient Air Quality Standard (NAAQS) attainment. UV photometric methods for O_3_ are generally considered easier to deploy and operate and in most cases do not require external compressed gases for operation. UV photometric analyzers determine O_3_ concentrations by quantitatively measuring the attenuation of light due to absorption by O_3_ present in an absorption cell at the specific wavelength of 254 nm (Parrish and Fehsenfeld, 2000; Williams et al., 2006). The O_3_ concentration is determined through a two-step process in which the light intensity passing through the sample air (*I*) is compared with the light intensity passing through similar sample air from which all O_3_ is first removed (*I*_0_). The ratio of these two light intensity values (*I/I*_0_) provides the measure of the light absorbed at 254 nm, and the O_3_ concentration in the sample is then determined through the use of the Beer–Lambert law as given in Eq. (5):

(5) $$I/I_{0}=e^{-KLC}\left(C=1/KL\ln\left[I/I_{0}\right]\right),$$

where *L* is the length of the absorption cell (cm), *C* is the O_3_ concentration (ppm), and *K* is the absorption cross section of O_3_ at 254 nm at standard atmospheric temperature and pressure conditions (308 atm^−1^ cm^−1^). Photometric monitors generally use mercury vapor lamps as the UV light source, with optical filters to attenuate lamp output at wavelengths other than 254 nm.

Air for the reference cell measurement (*I*_0_) is typically obtained by passing the ambient air sample stream through a catalytic scrubber containing manganese dioxide (MnO_2_), hopcalite (a mixture of Cu, Mn, and Ag oxides), heated silver wool, or another solid state material to “scrub” only O_3_ from the sample air while preserving all other substances in the sample air that potentially absorb at 254 nm (e.g., elemental gaseous mercury [Hg^0^], hydrogen, sulfide [H_2_S], VOCs) so that their effects are canceled in the differential *I/I*_0_ measurement. The integrity of the O_3_ reference scrubber is critical and may allow measurement interferences if it does not perform adequately. Similarly, any tendency of the scrubber to fail to effectively remove all O_3_ from the reference sample will result in a measurement bias. In addition to O_3_, catalytic scrubbers have been shown to remove UV-active VOCs (Kleindienst et al., 1993), creating the potential for positive artifacts in O_3_ measurements when the efficiency of this VOC removal is impacted.

Although FEM-designated UV photometric instruments are accurate under most ambient conditions, locations with high VOC concentrations can produce significant analytical artifacts. Smoke-plume-impacted locations and measurements downwind from wildland fires are a particular concern; O_3_ measurements of up to 320 ppb were observed in a smoke plume in western Oregon using a Dasibi 1003AH UV photometric O_3_ monitor (Huntzicker and Johnson, 1979), which also showed a correlation between apparent O_3_ and aerosol concentrations (*b*_scat_, a combustion plume indicator in this case). O_3_ measurements from UV photometric instruments exceeding 1500 ppb at night (22:00–05:00) were observed in Fort McMurray, Alberta, during smoke impacts from the 2016 Horse River Fire, which were positively correlated with NO and non-methane hydrocarbons (Landis et al., 2018). Follow-up pyrolysis experiments demonstrated that ET-CL instruments do not show a similar response to biomass burning smoke (Huntzicker and Johnson, 1979). Photochemical chamber experiments comparing the O_3_ response of UV (Dasibi model 1003AH, Dasibi model 1008AH, and Thermo model 49) and ET-CL (Bendix model 8002 and Monitor Labs model 8410) mixtures show negligible differences for irradiated paraffin–NO_*x*_ and olefin–NO_*x*_ mixtures but do show a positive UV interference in mixtures with toluene and other aromatics present (Kleindienst et al., 1993). Laboratory studies comparing the response of UV (Thermo model 49, Horiba APOA-370, and 2B Tech model 202) and ET-CL (Bendix) instruments showed a positive interference for *o*-nitrophenol, naphthalene, and *p*-tolualdehyde for the UV instruments but not the ET-CL instruments (Grosjean and Harrison, 1985; Spicer et al., 2010). Additionally, during the Mexico City Metropolitan Area (MCMA-2003) field campaign a mobile laboratory using an FEM-designated UV photometric O_3_ monitor (unheated MnO_2_ scrubber, Thermo 49 series) showed a large positive O_3_ interference (~ 400 ppb) associated with PM_2.5_ and polyaromatic hydrocarbons (PAHs) when following some diesel vehicles (Dunlea et al., 2006). Although not compared to a chemiluminescence instrument, those high O_3_ values are unlikely real considering the high concurrent NO concentrations (in some cases >1000 ppb). The authors of this study attributed the interference to fine particles, based on the correlation with PM_2.5_ and the lack of a correlation with gas-phase organic species measured by the proton-transfer-reaction mass spectrometer (PTR-MS; Dunlea et al., 2006).

In addition to interferences from the presence of aromatic VOCs and semi-volatile PAHs, water vapor (relative humidity) issues have also been observed with older-generation FRM- and FEM-designated chemiluminescence and UV photometric O_3_ instruments, respectively (Kleindienst et al., 1993; Leston et al., 2005; Wilson and Birks, 2006). As such, Nafion® tube dryers are regularly incorporated into some newer-generation chemiluminescence and UV photometric O_3_ monitors in an attempt to mitigate the humidity-related measurement artifacts.

A recently introduced variation in the UV photometric method, known as the “scrubberless” UV photometric (SL-UV) method (Ollison et al., 2013), specifies removal of O_3_ from the sample air for the reference by a gas-phase reaction with NO rather than using a conventional solid-state catalytic scrubber. The NO gas-phase chemical scrubber reacts with O_3_ much faster and more selectively than with other potential interfering compounds and is very effective at removing the O_3_ without affecting other interfering compounds that may be present in ambient air. The differential UV measurement can then effectively reduce interferences to an insignificant level. Similar to NO-CL, the SL-UV method requires a continuous supply of compressed NO or nitrous oxide (N_2_O) (which the instrument converts to NO) to serve as the scrubber gas.

In this study, we investigate UV photometric FEM instrument O_3_ measurement interferences in fresh biomass burning smoke plumes from prescribed grassland fires and during controlled burn experiments in a large-scale combustion chamber. We directly compare NO-CL FRM O_3_ measurements to several FEM-designated UV photometric technologies, including a gas-phase scrubber and catalytic scrubbers with and without Nafion® tube dryer systems. Based on the results from the measurements, we assess the magnitude of the observed artifacts for different technologies and under various smoke conditions and provide suggestions for potential mitigation of the interferences.

## 2 Methods

### 2.1 Overview of methods evaluated

In this study we compared the measurement results from six different commercially available FRM- and FEM-designated O_3_ instruments operated in ambient or chamber-generated biomass burning smoke. All instruments were operated according to their FRM or FEM designation. The six instruments differed by measurement principle (chemiluminescence versus UV photometric) and by sample treatment configuration (scrubber material, presence of dryer, etc.). For interference-free O_3_ measurements, we utilized the newly designated FRM NO-CL method (U.S. EPA, 2015). For the UV photometric methods, we compared both catalytic scrubber and “scrubberless” (gas-phase chemical scrubber) technologies, with the scrubberless monitor using a NO chemical scrubber. Finally, within the catalytic scrubber UV photometric category, we compared instruments with and without Nafion® tube dryer systems. The operation principle and designations (FRM vs. FEM) for the analyzers under investigation are summarized in Table 1 and described in Sect. 2.1.1–2.1.4. These analyzers were operated immediately downwind of fresh biomass burning plumes over 8d of prescribed fires in grassland ecosystems in Oregon and Kansas and during laboratory-based studies at the U.S. Forest Service’s (USFS) combustion facility at the Fire Sciences Laboratory (FSL) in Missoula, Montana. The grassland fire fuels consisted primarily of mixed native prairie tallgrass of varying moisture content. A total of 7 of the 8d of prescribed burning were conducted in the tallgrass prairie ecosystem of central Kansas (4d in March of 2017 and 3d in November of 2017). The additional burn day was conducted at the Sycan Marsh in central Oregon (October of 2017). Laboratory-based chamber burns at the FSL were conducted during April 2018 and again during April 2019. Fuels for the laboratory based chamber burns consisted of ponderosa pine needles and fine woody debris. Details of the individual studies are provided in Sect. 2.2–2.6.

#### 2.1.1 NO chemiluminescence

The FRM O_3_ measurement method was the Teledyne API (San Diego, CA, USA) model T265 chemiluminescence monitor (TAPI T265), which utilizes a NO-CL measurement principle. These NO-CL O_3_ analyzers have been shown to be free of interferences (Long et al., 2014) and have been used as a reference method in other O_3_ comparison studies (Williams et al., 2006; Landis et al., 2021). Although there is a known water vapor interference with chemiluminescence technology (Kleindienst et al., 1993), the TAPI T265 uses a Nafion® tube dryer system to remove water vapor from the air prior to making the measurement, thus eliminating any humidity-related effects. Like the ET-CL technologies (Kleindienst et al., 1993), the NO-CL analyzers have no documented VOC interferences. Manufacturer-provided performance specifications for the NO-CL-based TAPI T265 are given in Table S1.

#### 2.1.2 Catalytic scrubber UV photometric

For this study the UV photometric method with no humidity correction was represented by the Thermo Scientific (Franklin, MA, USA) model 49i (Thermo 49i), which is a dual-cell instrument with a manganese oxide (MnO_2_) catalytic scrubber, referred to as UV-C. Nafion®-based humidity systems or dryers have been employed within photometric O_3_ monitors with catalytic scrubbers before the measurement cell, offering a reduction in relative humidity interferences and artifacts (Wilson and Birks, 2006). Manufacturer-provided performance specifications for the UV-C-based Thermo 49i are given in Table S1.

The UV photometric with a Nafion® humidity conditioning system was represented in this study by a 2B Technologies (Boulder, CO, USA) model 205 (2B 205) O_3_ monitor. The 2B 205 utilized a dual-cell design where sample air and scrubbed air are measured simultaneously. The 2B 205 uses a hopcalite (CuO*/*MnO_2_) catalytic scrubber to remove O_3_ from the reference stream. This instrument will be referred to as UV-C-H. Manufacturer-provided performance specifications for the UV-C-H-based 2B 205 are given in Table S1.

#### 2.1.3 Scrubberless UV photometric

For comparison with the NO-CL, UV-C, and UV-C-H methodologies, a scrubberless UV (SL-UV) photometric analyzer with a gas-phase (NO) chemical scrubber was employed (Ollison et al., 2013; Johnson et al., 2014). The addition of NO gas to the reference stream selectively scrubs O_3_ while not significantly affecting interfering VOC species, resulting in an interference-free O_3_ determination. Inclusion of this instrument in the study allows evaluation of the impact of the UV method in general (compared with chemiluminescence) versus the influence of specific scrubber technologies. The SL-UV method is represented by the 2B Technologies model 211 scrubberless ozone monitor (2B 211). The model 2B 211 requires a continuous supply of compressed NO or nitrous oxide (N_2_O) (which the instrument converts to NO). The SL-UV method also utilizes a Nafion®-based sample humidity conditioning system to eliminate any humidity effects. The SL-UV instrument was not used in the October or November 2017 burns due to the lack of the necessary reagent gas (nitrous oxide, N_2_O) needed to run the instrument. Manufacturer-provided performance specifications for the SL-UV-based 2B 211 are given in Table S1.

#### 2.1.4 Heated graphite scrubber UV photometric

During the final phase of laboratory-based burning, a 2B Technologies model 211-G UV photometric analyzer (2B 211-G) was operated for comparison to the monitors detailed in Sect. 2.1.1–2.1.3. The 2B 211-G differs from the 2B 211 in that it employs a heated graphite scrubber to remove O_3_ from the reference sample stream (*I*_0_) (Turnipseed et al., 2017). The 2B 211-G utilizes the same Nafion®-based sample humidity conditioning system as employed in the 2B 211. For the purposes of this paper the UV photometric method employing the heated graphite scrubber is referred to as UV-G. Manufacturer-provided performance specifications for the UV-G-based 2B 211-G are given in Table S1.

### 2.2 Prescribed fire burn mobile sampling platform

During the prescribed fire grass burns, all study instrumentation (analyzers, data acquisition systems, and peripheral systems) were mounted in portable instrument racks and installed inside an enclosed EPA 4×4 vehicle (Whitehill et al., 2019). The instruments were connected via perfluoroalkoxy alkane (PFA) Teflon® tubing (0.64 cm diameter) to PFA Teflon® filter packs loaded with 47 mm, 5 μm pore size pressure-drop-equivalent Millipore (Burlington, MA, USA) Omnipore® polytetrafluoroethylene (PTFE) filter membranes which were (i) mounted to a rooftop sampling platform during spring 2017 sampling or (ii) connected to a cross-linked Teflon®-coated high-flow manifold mounted on the inside roof of the truck compartment during fall 2017 sampling. The truck was positioned downwind of active biomass burning plumes, usually within meters to hundreds of meters of the active fire line, and positioned so that the trailer was downwind of the sample inlets (to avoid interferences from generator exhaust). In addition to the O_3_ analyzers under investigation, additional monitors were also operated for the determination of carbon monoxide (CO), NO, NO_2_, total oxides of nitrogen (NO_*x*_ =NO+NO_2_), and total hydrocarbons (THCs, to approximate VOC concentrations). The operation principle and designation (FRM vs. FEM) information for the additional analyzers deployed in this study are summarized in Table 2. Data from all instruments were recorded on an Envidas Ultimate data acquisition system.

All instruments were calibrated with multipoint calibrations before and after each sampling day. All pre- and post-calibrations met our quality performance objectives of ±10% and linearity of *r*^2^ ≥ 0.99. For the O_3_ analyzers under investigation, field and laboratory calibrations were performed using a Teledyne API model T700U dynamic dilution calibrator with a NIST traceable O_3_ photometer and O_3_ generation system. Zero air for the calibrator was supplied by a Teledyne API model T701H zero-air generator. Calibrations for NO, NO_2_, NO_*x*_, and CO were performed using the same calibrator and zero air generator utilizing a certified EPA protocol tri-blend (CO, NO, SO_2_) gas cylinder (Airgas). Per the manufacturer-provided operator’s manual, calibrations for THC were performed using the T700U calibrator and a certified EPA methane–propane gas cylinder (Airgas). FID response factors for organic compounds can vary significantly based upon factors such as carbon number and compound class (Tong and Karasek, 1984). The carbon numbers for methane and propane vary by a factor of 3 and the FID response factors for those compounds may also vary by a similar amount. In addition, the complex mixture of hydrocarbons found in smoke will have large variations in carbon number and FID response factors. As such, the results obtained with the THC analyzer are an approximation of THC (and VOC) concentrations in smoke. In addition, for THC calibrations, the T701H zero-air generator was replaced with scientific-grade zero-air compressed gas cylinders (Airgas).

### 2.3 Kansas prescribed burns, March 2017

Biomass burning plumes were sampled over 4d of prescribed burns (15–17 and 20 March 2017) on the Konza Prairie Long Term Ecological Research (LTER) site outside of Manhattan, Kansas. The fuels for this series of burns consisted of mixed native prairie tallgrass of varying moisture content. Over the 4d period, a total of 13 burns were conducted and sampled.

### 2.4 Oregon prescribed burns, October 2017

A single 10h day of prescribed grassland burning was measured at the Sycan Marsh Preserve in central Oregon on 11 October 2017. Fuels for the Sycan Marsh burn consisted of mixed native prairie tallgrass of varying moisture content.

### 2.5 Kansas prescribed burns, November 2017

Biomass burning plumes were sampled during a single day of prescribed burning (10 November 2017) on the Konza Prairie LTER site outside of Manhattan, Kansas, and on 2 additional days (13 and 15 November 2017) at the Tallgrass Prairie National Preserve outside Strong City, Kansas. Fuels for the November 2017 burns consisted of mixed native prairie tallgrass of varying moisture content. During the 10 November sampling, two separate burns were conducted. Three burns were conducted over the 2d at Tallgrass Prairie National Preserve.

### 2.6 USFS Missoula burn chamber burns 2018, 2019

Laboratory-based studies were performed at the US Forest Service’s combustion testing facility at the FSL in Missoula, Montana, by EPA and USFS personnel. These static chamber burns occurred in the spring of 2018 (16–24 April 2018, 33 burns; Landis et al., 2021) and again in the spring of 2019 (15–26 April 2019, 31 burns). The main combustion chamber is a square room with internal dimensions of 12.4m×12.4m×19.6m high and a total volume of 3000 m^3^ and has been described previously (Bertschi et al., 2003; Christian et al., 2004; Yokelson et al., 1996; Landis et al., 2021). During the combustion chamber studies, the facility was fitted with identical instrumentation racks, calibration systems, systems for sampling of test atmosphere, and data acquisition systems as those described in Sect. 2.2. All instrumentation was housed in an observation room immediately adjacent to the combustion chamber with PFA inlet lines extending through the wall into the chamber. All inlet lines contained an identical filter pack and filter assembly described in Sect. 2.2 to protect inlet lines and the analyzers from particulate contamination. Fuel beds consisting of ponderosa pine needles and mixed woody debris were prepared and placed in the middle of the chamber. The amount and moisture content of the fuels were varied to generate different flaming/smoldering conditions during the burns. During the chamber burns the combustion room was sealed and the fuel bed was ignited. Two large circulation fans on the chamber walls and one on the ceiling facilitated mixing and assured homogeneous conditions during the burn periods (Landis et al., 2021). In general, chamber relative humidity (RH) values were below 50%, facilitating dry burning conditions.

## 3 Results and discussion

### 3.1 Results from ozone measurements in prescribed grassland fire plumes

**Figure 1.**
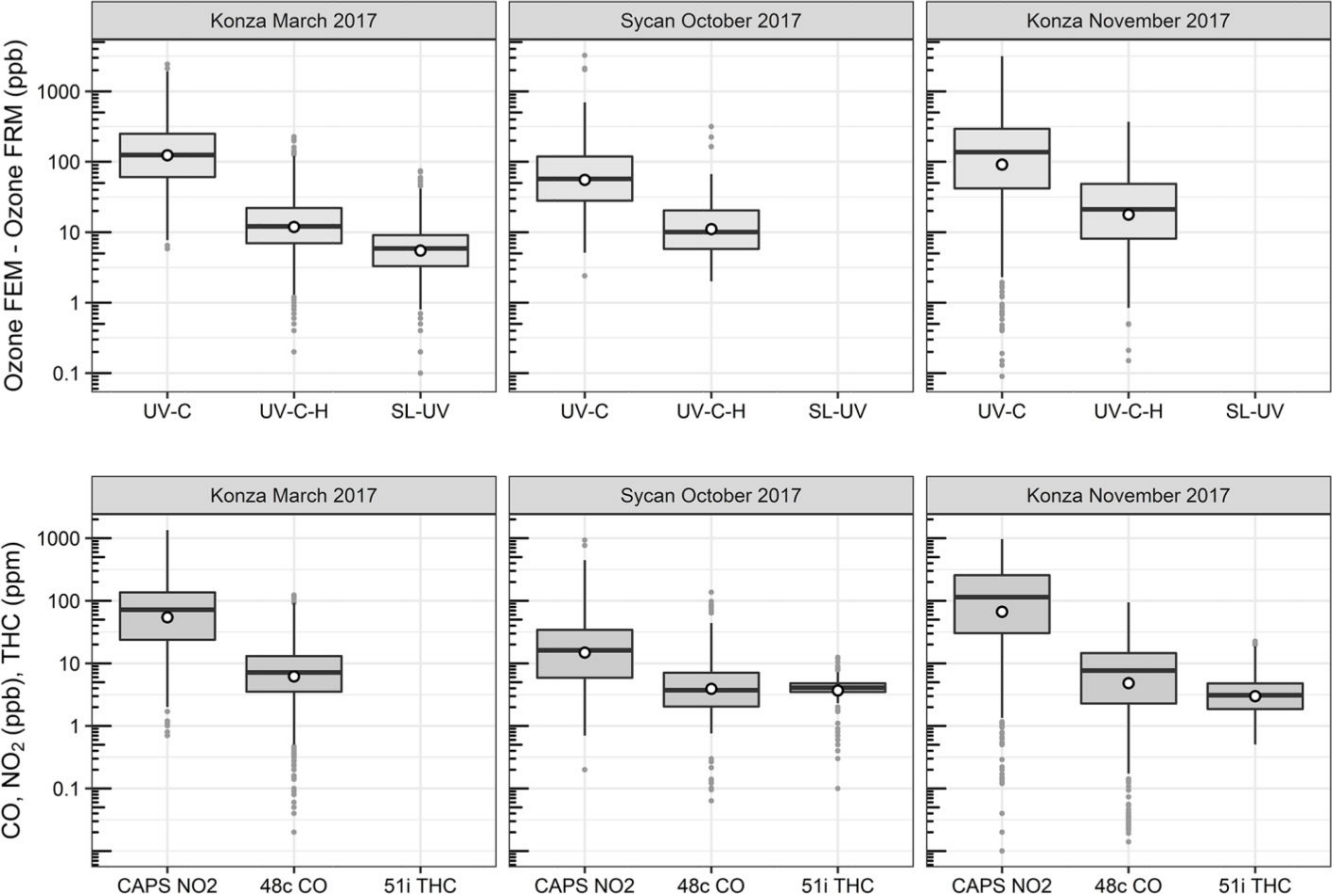
Ozone concentration differences between FEM instruments and the FRM instrument (FEM−FRM) and the measured NO_2_, CO, and total hydrocarbons (THCs) during the three 2017 wildfire deployments. All measurements included are within-smoke-only measurements, and show both the elevated smoke tracers (NO_2_, CO, THC) and the persistent elevation of the FEM O_3_ measurements. The box-and-whisker plots indicate the 25th, 50th, and 75th quartiles, with the whiskers extending to 1.5 times the inner quartile range. The open dots indicate the mean values for each instrument within smoke.

**Figure 2.**
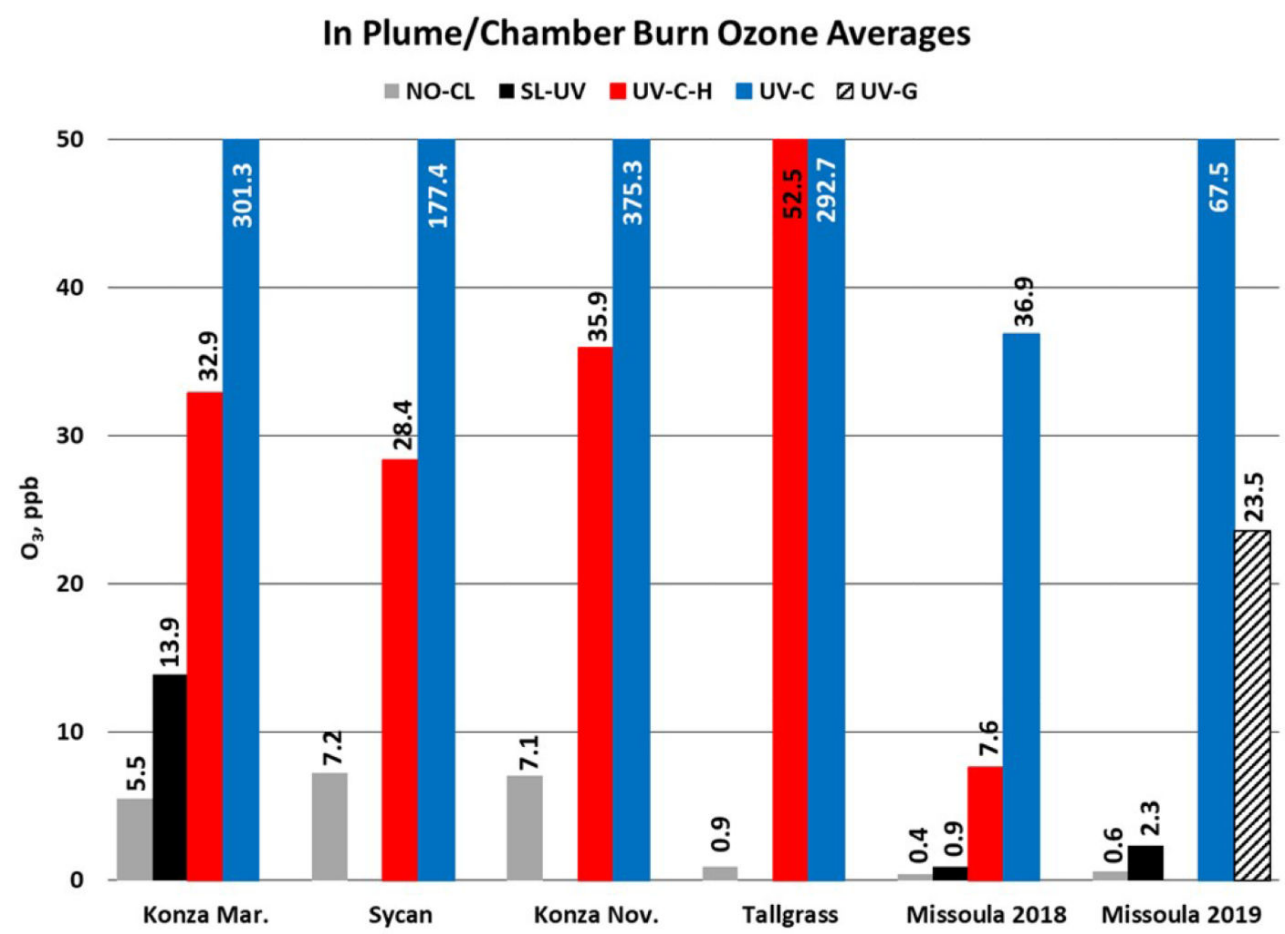
In-plume O_3_ concentration averages from the 2017 prescribed grassland burns and the 2018 and 2019 Missoula chamber burns. For the 2017 grassland burning periods, CO concentration results (≥ 1 ppm) were used as an indicator of when in-smoke sampling was occurring.

O_3_ measurement results from the Oregon and Kansas prescribed grassland fire studies are shown as the difference between the FEM and FRM in Fig. 1, and 1min average time series plots for the studies are presented in Supplement Figs. S1–S3. There were significant differences in the measurement results obtained from the different O_3_ monitors operated during the prescribed fires. The UV-C instrument (Thermo 49i) consistently showed large increases in O_3_ concentration readings in fresh biomass burning plumes, with measurements exceeding the FRM measurement by 2–3 ppm. The O_3_ exceedances were generally correlated in time with CO and THC (biomass burning indicators) and NO_2_. These correlations will be discussed separately. The UV-C-H instrument (2B 205) also showed increased readings in smoke plumes (also correlated with CO, THC, and NO_2_), but with absolute measurements roughly an order of magnitude smaller than the UV-C instruments. The NO-CL (T265) instrument results showed the opposite behavior, with reductions in O_3_ readings inversely correlated with increases in NO_2_ concentrations, as expected from general O_3_ titration by NO (NO+O_3_ → NO_2_+O_2_). For the March 2017 measurements the SL-UV instrument (2B 211) produced readings roughly comparable with the NO-CL monitor, but with substantially more noise on a minute-to-minute timescale. The “in-plume” average O_3_ concentrations from the four prescribed grassland burning periods are shown in Fig. 2. For the purposes of this comparison, CO measurements were used as an indicator of when sampling occurred in plume. In addition, ambient RH values were generally below 50% indicating that the spring and fall 2017 prescribed burns were conducted under dry conditions.

### 3.2 Results from ozone measurements in USFS chamber burns

**Figure 3.**
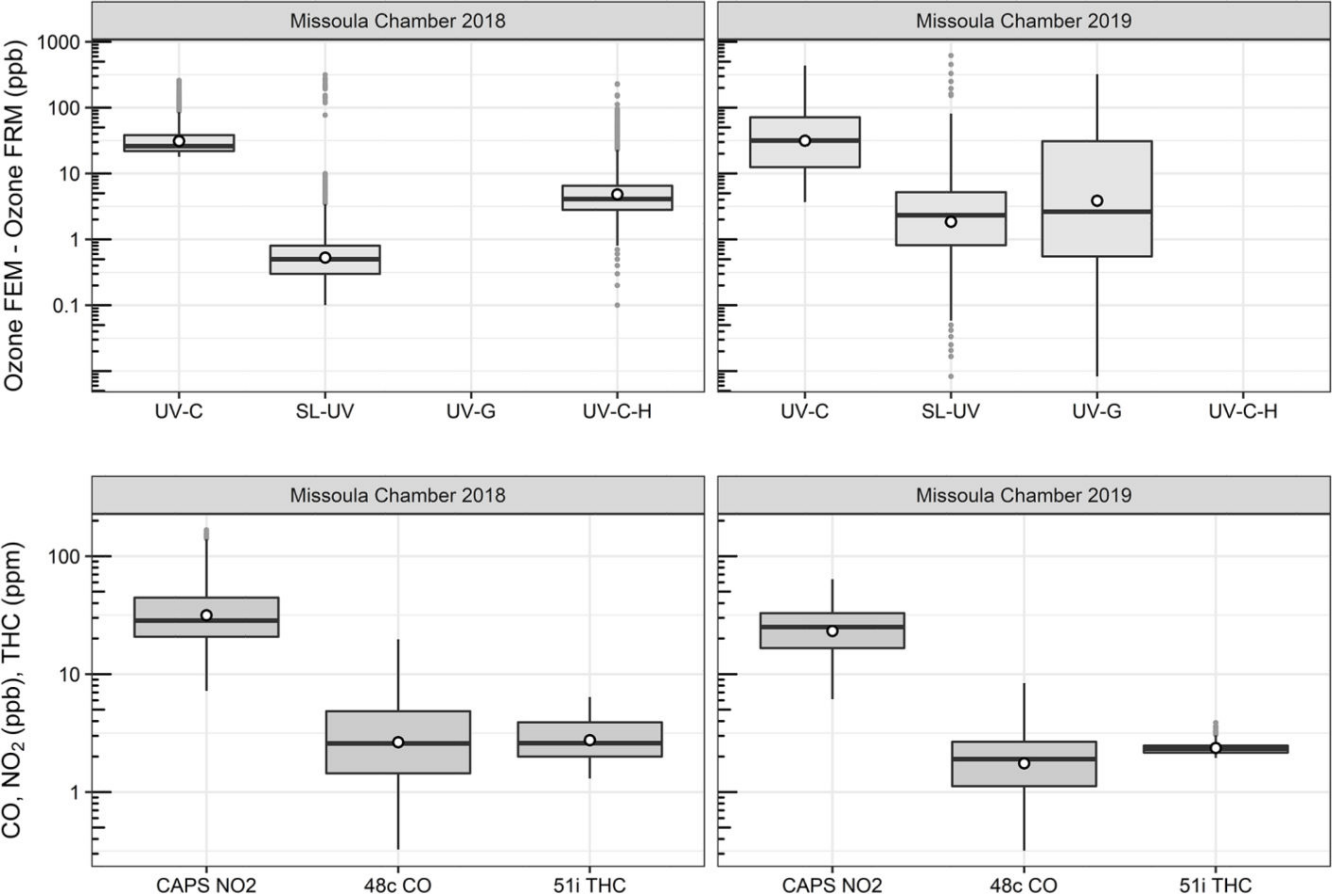
Differences between the FEM and FRM instrument concentrations (FEM−FRM) and NO_2_, CO, and total hydrocarbon (THC) concentrations during the 2018 and 2019 Missoula chamber studies. All measurements included are within-smoke-only measurements and show both the elevated smoke tracers (NO_2_, CO, THC) and the persistent elevation of the FEM O_3_ measurements compared to the FRM. The box-and-whisker plots indicate the 25th, 50th, and 75th quartiles, with the whiskers extending to 1.5 times the inner quartile range. The open dots indicate the mean values for each instrument within smoke.

**Figure 4.**
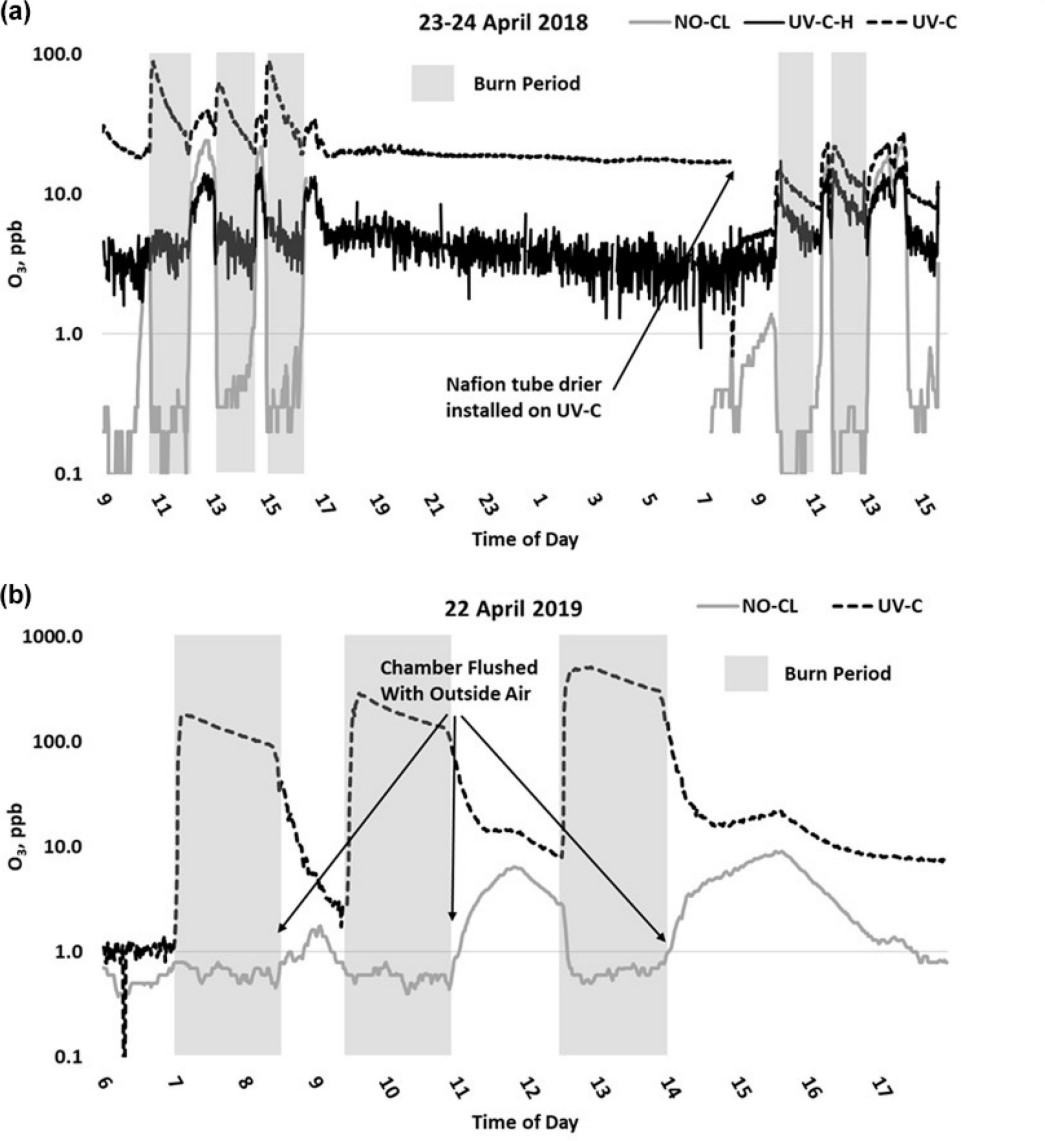
Time series example of USFS chamber burn O_3_ results from the NO-CL, UV-C, and UV-C-H (2018 only) from 23–24 April 2018 (**a**) and 22 April 2019 (**b**). O_3_ concentrations are displayed on a logarithmic scale. The post-burn calibration checks on 23 April 2018 revealed a +8% bias in the NO-CL method and a −2% bias in the UV-C-H method. These biases were evident during the chamber flush periods on that day. Each analyzer was re-zeroed and spanned, resulting in the elimination of the bias between the two methods as observed in the results from the subsequent day (24 April 2018).

O_3_ measurement results from the 2018 and 2019 USFS chamber burn studies are shown in Fig. 3. Time series plots of the chamber study data are included in Supplement Figs. S4 and S5. Figure 4 gives a more detailed view of UV-C and NO-CL O_3_ results (2d from 2018 and 1d from 2019) during the chamber burns. In contrast to the prescribed grassland burns, the Missoula chamber burns employed differing fuel types (ponderosa pine needles and fine woody debris) that are more typical of fuels consumed during western US forest fires. In addition, the concentrations of pollutants generated and observed during the chamber studies were approximately an order of magnitude smaller than those observed during the prescribed grassland fires. For reference, maximum PM_2.5_ concentrations observed during the prescribed fires were in excess of 50 mg m^−3^ while maximum chamber PM_2.5_ concentrations were less than 2 mg m^−3^. Regardless of these differences, there were still significant (order of magnitude or more) differences in the measurement results between the different FEM O_3_ instruments operated during both the 2018 and 2019 chamber studies. The NO-CL method showed identical trends to those observed during the grassland burns in that its measured O_3_ concentrations dropped to near zero during the active burning periods as indicated in Fig. 4 (active burning periods shaded in grey). The only periods when significant O_3_ concentrations were measured by the NO-CL method were when outside air was brought in to flush the chamber in between burns. The post-burn calibration checks on 23 April 2018 revealed a +8% bias in the NO-CL method and a −2% bias in the UV-C-H method. These biases were evident during the chamber flush periods on that day. Each analyzer was re-zeroed and spanned, resulting in the elimination of the bias between the two methods as observed in the results from the subsequent day (24 April 2018). No other calibration corrections were made during the 2018 and 2019 chamber studies. As in the grassland fire plumes, the UV-C method showed increased O_3_ concentration (positive analytical artifact) readings that were correlated in time with CO and NO_2_; see Supplement Figs. S9 and S10. Similarly, the UV-C-H instrument also showed increased positive analytical artifacts during the chamber burns but with absolute measurement values about an order of magnitude smaller than the UV-C instruments. The SL-UV method gave similar results to the NO-CL method during both the 2018 and 2019 chamber burns. Newly added during the 2019 burns, the UV-G method (2B 211-G) gave mixed results: at times it provided similar results compared to the NO-CL and SL-UV methods, and at others it provided results in line with those provided by the UV-C method. See Supplement Fig. S5 for the 2019 chamber burn time series plot. The burn average O_3_ concentrations from the 2018 and 2019 chamber burns are presented in Fig. 2.

During the 2018 chamber burns the UV-C results were biased high by 15–20 ppb even during non-burn (i.e., overnight) periods as evident in Figs. 4 (top panel) and S4. The initial hypothesis was that the bias was associated with high chamber backgrounds of interfering species due to years of heavy burning in the chamber. However, it was later discovered during a subsequent summer–fall 2018 ambient air study in North Carolina in the absence of smoke that sampling heavy smoke plumes during the fall 2017 prescribed grassland burns followed by subsequent storage of the UV-C analyzer irreversibly damaged the MnO_2_ scrubber in the UV-C instrument. It is hypothesized that the damage resulted in the scrubber removing some of the interfering species in addition to ozone, preventing them from being subtracted off as background in the reference measurement and subsequent detection as ozone (positive bias) during the measurement cycle. The effect of the bias was observed mainly when sampling ambient and chamber air and not readily observed during routine calibration checks (zeroes and spans) except for an increase in the time required to obtain stable zero and span values. The bias was not observed during any of the 2017 prescribed grassland burns. During the summer–fall 2018 North Carolina study and prior to the start of the 2019 chamber burns, a new MnO_2_ scrubber was installed and resulted in a significant and immediate reduction of the observed high bias, shown in Figs. 4 (bottom panel) and S5.

### 3.3 Methodological influence on ozone measurements in biomass burning smoke

As discussed in Sect. 3.1 and 3.2, there are large (order-of-magnitude level) differences in O_3_ concentration measurement results obtained from the FRM (NO-CL) and the FEM UV photometric with catalytic scrubber (UV-C) O_3_ methods. The extremely low O_3_ concentrations measured by the NO-CL instrument are consistent with O_3_ depletion in the presence of high NO_*x*_ concentrations (up to parts-per-million levels) observed in the grass burning plumes and during chamber burns. The reaction between NO and O_3_ is rapid and occurs on the timescales of seconds to minutes. As a result, high NO in the fresh biomass combustion plumes will efficiently titrate out O_3_, leading to near-field depletion within the plumes relative to background concentrations. There was no sign of a positive interference in the NO-CL monitors, and it remains the most robust and accurate routine method for O_3_ measurement in fresh and downwind biomass burning plumes.

In contrast with the NO-CL FRM instrument results, the UV-C FEM results showed substantial increases in reported O_3_ concentrations in the fresh biomass burning plumes. There is no known pathway for direct O_3_ emission from biomass burning, and the proximity (meters to hundreds of meters) and timescales (travel time of seconds to minutes from the combustion source to measurement) involved are too short for the usual NO_*x*_–VOC photochemistry to produce secondary O_3_. Further, since the FSL chamber interior is not exposed to sunlight, photochemistry would not have been active in the Missoula laboratory burns. For the purposes of this work, the positive analytical artifact in the UV-C method, termed ΔO_3(UV-C)_, is estimated using Eq. (6) as the difference between UV-C and the NO-CL O_3_ concentration measurement results for the same time period:

(6) $$\Delta {\text{O}}_{3(\text{UV}–\text{C})}=\text{UV}–\text{C}-\text{NO}–\text{CL}.$$

**Figure 5.**
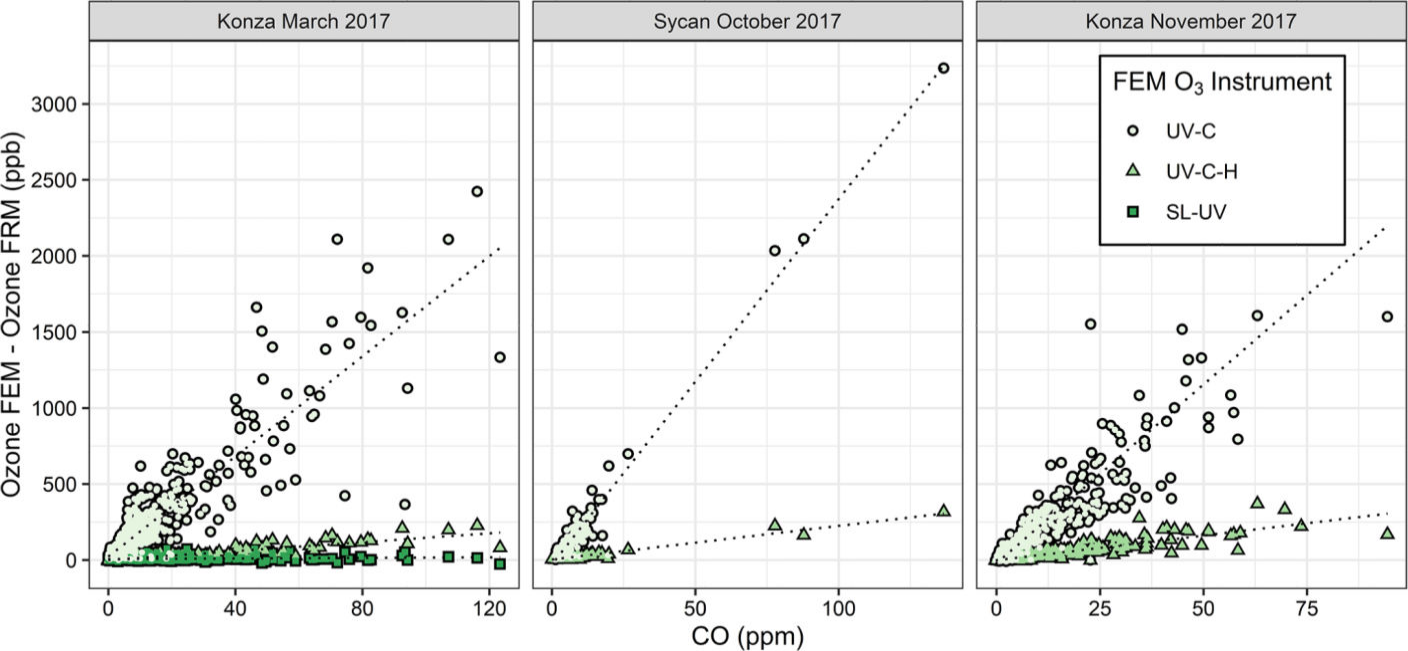
Scatter plots between FEM and FRM O_3_ differences and the CO measurements within the grassland fire smoke plumes. The FEM measurements are differentiated by color and shape. The SL-UV method was only run during the Konza March 2017 measurements.

Figure 5 shows in-plume regressions between ΔO_3(UV-C)_ and the FRM measurement and CO for the three measured prescribed grassland burns in 2017 (Supplement Fig. S6 shows the time series of ΔO_3(UV-C)_ and CO). Figures 5 and S6 show good correlations within the smoke plumes. The average and maximum values of ΔO_3(UV-C)_ are summarized in Table 3. It is hypothesized that the large “O_3_” measurement observed in the UV-C method results from a positive interference or artifact, likely linked to VOC emissions in the grassland burn plumes. VOCs are emitted in higher concentrations from the smoldering phase of combustion, which is also characterized by large CO emissions (Yokelson et al., 1996, 1997), so a correlation between CO and O_3_ artifacts would support the hypothesis of a VOC-linked interference for the UV-C instruments. This is also consistent with observed VOC interferences in previous studies (Grosjean and Harrison, 1985; Kleindienst et al., 1993; Spicer et al., 2010) and observed following fireworks (Fiedrich et al., 2017; Xu et al., 2018).

The presence of a Nafion®-based humidity conditioning system (Nafion® tube dryer) significantly reduced the magnitude of the observed artifact as evident by comparing the UV-C and UV-C-H results shown in Figs. 1–3 and S1–S5. As with the UV-C method, the artifact in the UV-C-H method, ΔO_3(UV-C-H)_, is calculated using Eq. (7) as the difference between UV-C-H and the NO-CL O_3_ concentration measurement results for the same time period:

(7) $$△O_{3(\mathrm{UV}–C–H)}=UV–C–H-NO–CL.$$

The addition of the Nafion®-based humidity conditioning system reduces the magnitude of the ΔO_3(UV-C-H)_ artifact by approximately an order of magnitude compared with the UV-C method. This is further illustrated in the 2018 chamber burns, where prior to beginning the final burn day on 24 April 2018, a Nafion® tube dryer (PermaPure, MD Monotube Dryer Series) was installed in the UV-C method (Thermo 49i), in effect converting it to a UV-C-H method. As shown in Figs. 4 and S4, the addition of the Nafion® tube dryer significantly reduced the ΔO_3(UV-C)_ artifact to a point comparable with that observed in the UV-C-H method (2B 205). A possible explanation for this effect is presented and discussed in Sect. 3.5. In addition, the previously described bias related to the damaged MnO_2_ scrubber was also reduced upon addition of the Nafion® dryer to the UV-C method.

For the March 2017 Konza Prairie study (Fig. 1) and the 2018 and 2019 USFS chamber studies (Fig. 3) the SL-UV instrument concentration results were comparable to, although noisier and slightly higher than, the NO-CL reference instrument. On numerous occasions during the prescribed and chamber burns, the SL-UV instrument shows short (i.e., 1min data point) positive or negative excursions that are not also observed in the NO-CL results. In addition, these excursions are not correlated with changes in CO concentrations. Because the SL-UV is a dual-cell instrument that measures O_3_ by comparing the absorbance of two cells, it is critical in highly dynamic environments (such as during this study) that both cells be measuring the same air at the same time. A slight difference in flow rates or residence times between the two pathways (or a delay in one pathway relative to the other) will cause short-term variability in the difference between the two cells. Although this does not pose an issue for longer time averaging (i.e., hourly data) under stable conditions, the dynamic nature of biomass burning plumes (i.e., changing on the order of seconds) and short time averages (i.e., minute) can create issues (noise) for the SL-UV method.

Significant analytical artifacts were observed for FEM UV photometric O_3_ instruments with (UV-C-H) and without (UV-C) Nafion®-based humidity conditioning systems, where it appears that the dual effect of ambient humidity fluctuations and VOC interferences caused large positive overmeasurement of in-smoke O_3_ concentrations. Chemiluminescence monitors are highly specific to O_3_ and have long been known to be free of VOC interferences (Long et al., 2014; U.S. EPA, 2015). However, studies have shown that the chemiluminescence method can be impacted by changes in relative humidity (Kleindienst et al., 1993). As such, upon promulgation in 2015, the new NO-CL FRM regulatory text requires a humidity correction–dryer system to eliminate the potential water vapor interference. As configured from the manufacturer, the NO-CL-based Teledyne-API model T265 instrument operated during this comparative study employs Nafion® drying technologies to reduce or eliminate the water vapor interferences. The use of a chemical (NO) scrubber for UV photometric instruments (such as the 2B Technologies model 211) is very specific to O_3_ and shows a much better response than the catalytic scrubber instruments, performing almost as well as the NO-CL FRM, and has significant potential as a low-interference O_3_ method. Of the catalytic scrubber photometric instruments, those with Nafion®-based humidity equilibration (2B Technologies model 205) perform significantly better than those without (Thermo 49 series).

In areas highly impacted by smoke or for studies focusing on biomass burning plumes, the use of a NO-CL FRM instrument is highly recommended as it was found to be essentially interference-free. These instruments are anchored to absolute O_3_ concentrations through the use of certified O_3_ calibration sources, many of which are based on UV photometry. The newest generation of commercially available NO-CL FRM instruments, including that used here (the Teledyne T265), have a built-in drying system to correct for the humidity artifacts that affected earlier-generation chemiluminescence instruments (Kleindienst et al., 1993), making remaining interferences negligible compared to other technologies.

The gas-phase chemical scrubber UV instrument (2B 211) did not perform as well as the FRM under the prescribed grassland burns or chamber experimental conditions tested here, with the high-time-resolution (1 min) data showing a much higher degree of variability than the NO-CL FRM instrument. We hypothesize that the main factor driving this divergence between this method and the NO-CL FRM is the dual-cell differential configuration of the instrument, which is not conducive to rapidly changing concentrations in O_3_ or other absorbing gases, such as VOCs.

In smoke-impacted monitoring situations where the use of a UV photometric instrument is still preferred or required, the choice of a monitor with humidity equilibration provides a significant analytical improvement over those monitors without the humidity corrections. In the absence of an instrument with a Nafion® tube dryer and in non-regulatory applications, a dryer can be installed before the inlet or measurement cells to reduce the interference, as was demonstrated on the final day of the 2018 Missoula chamber burns. This will have the added benefit of reducing positive biases from humidity and reducing equilibration time for calibrations (especially when switching from high-humidity ambient air to dry calibration gases).

### 3.4 Magnitude of ozone artifact in fresh biomass burning plumes relative to markers of combustion

It is difficult to estimate an absolute magnitude or correct for the observed O_3_ analytical artifact since primary emissions from biomass combustion are highly variable and depend upon the fuel loading, fuel type and condition, phase of the fire, and the burn conditions (Yokelson et al., 1996, 1997). However, assuming the interference is driven primarily by VOCs, the artifact should be correlated with the excess CO (ΔCO=CO_plume_–CO_background_). Because CO_background_ during the prescribed grassland burns was below 200 ppb (relative to typical conditions of >2 ppm in the plume), ΔCO is estimated as the total measured CO concentration. A simplified view of biomass combustion assumes an approximate linear combination of two dominant emission phases, flaming combustion (characterized by emission of highly oxidized compounds, such as CO_2_, NO_*x*_, and SO_2_) and smoldering combustion (characterized by emission of reduced or mixed oxidation state compounds, such as CO, CH_4_, NH_3_, H_2_S, and most VOCs) (Yokelson et al., 1996, 1997). Because the majority of VOCs are in a reduced or mixed oxidation state, they tend to co-emit with CO during smoldering combustion, and the VOC concentrations tend to be highly correlated with CO in fresh biomass burning plumes (Yokelson et al., 1996). Scatter plots comparing the FEM instrument artifacts (ΔO_3(UV-C)_) and CO for the three prescribed grassland burning periods are shown in Fig. 5. Regression statistics of the comparison of ΔO_3(UV-C)_ and ΔO_3(UV-C-H)_ with CO and THC for grassland burns are given in Table 4. The magnitude of the artifact (estimated by the slope of the regression line of the CO vs. ΔO_3_ comparison), in parts per billion of apparent O_3_ per part per million of CO, ranges between 16–24 ppb ppm^−1^ for the UV-C instrument and 1.5–3 ppb ppm^−1^ for the instrument with humidity correction (UV-C-H). It is important to point out that CO, in and of itself, is not considered to be an interfering species in the UV photometric determination of O_3_ in that CO absorbs in the infrared (IR). The slight differences in the magnitude of the artifacts (fitted regression slopes) along with the low uncertainty (standard errors) values indicate that the magnitude of the artifact may be influenced by local conditions that make each burn unique. Such conditions might include meteorological conditions, fuel composition, fuel moisture content, and time spent in combustion phase (flaming vs. smoldering). Similar to CO, THCs and NO_2_ are indicative of combustion processes and are correlated with ΔO_3_ as given in Table 4 and Figs. S7 and S8. In terms of THC, the magnitude of the artifact, in parts per billion of apparent O_3_ per part per million THC, is significantly higher at ~ 88 ppb ppm^−1^ for the UV-C instrument and ~ 13 ppb ppm^−1^ for the UV-C-H instrument. Both the prescribed grassland and Missoula chamber burns resulted in what would be considered high PM concentrations (2–50 mg m^−3^). These high PM concentrations, however, are not considered to be interfering due to the presence of the inline particle filter assemblies described in Sect. 2.2 and 2.6.

Since the CO concentrations (from upwind fires) observed at most stationary sites from fire plumes are usually on the order of 1 ppm to greater than 10 ppm (Landis et al., 2018), it is reasonable to assume that O_3_ artifacts in the range of 15 ppb to greater than 250 ppb can be observed when employing a UV-C method. Similarly, O_3_ artifacts in the range of 1.5 to above 30 ppb might be observed at smoke-impacted sites monitoring with UV-C-H methods. As such, Nafion®-based humidity conditioning systems are highly recommended for use if employing UV photometric methodology for monitoring O_3_ in areas impacted by wildfires or prescribed burns. As stated previously and as seen in Fig. 3 and Table 3, O_3_ artifacts were observed during the Missoula chamber 2018 and 2019 burns in both the UV-C and UV-C-H methods, although reduced compared to the prescribed grassland burns. The presence and magnitude of the O_3_ artifact strongly suggest that smoke generated from fuels typical of forests in the western United States also result in a measurement interference in UV photometric methods. Since downwind O_3_ production in biomass burning plumes is a significant issue in fire-impacted regions, having reliable, interference-free methods is critical for assessing the contribution of wildland fires to ambient O_3_ levels.

**Figure 6.**
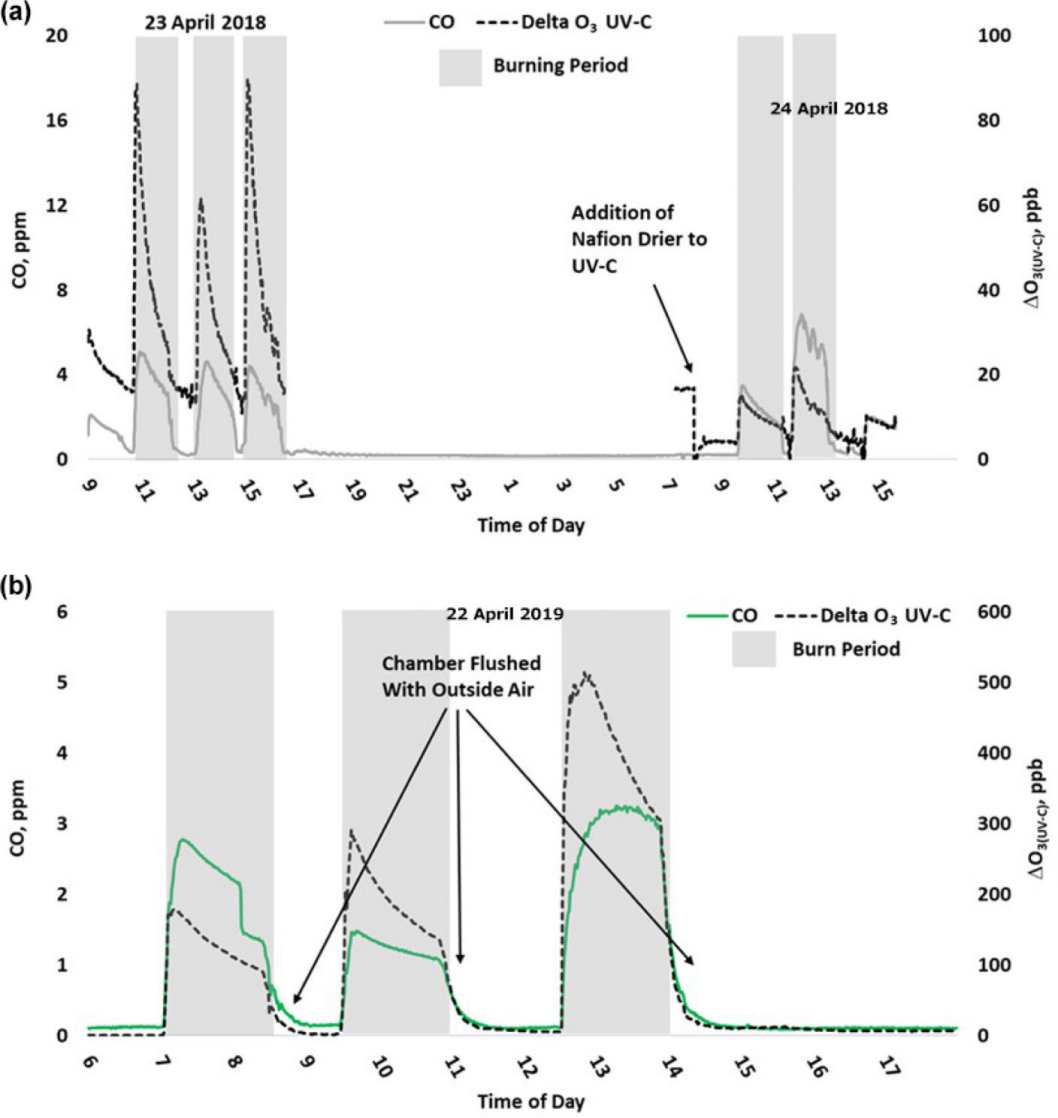
Time series example of USFS chamber burn ΔO_3_(UV-C) and CO concentration results from 23–24 April 2018 (**a**) and 22 April 2019 (**b**).

Figure 6 gives a detailed time series view of ΔO_3(UV-C)_ and CO from 2 burn days from 2018 and a single day during 2019. As indicated, ΔO_3(UV-C)_ and CO appear to be correlated in time, but when performing linear regression comparisons of ΔO_3(UV-C)_ and CO during each year’s chamber burns as a whole, correlations tend to be poor. We suspect the positive O_3_ bias is driven by one or more VOCs (likely oxygenated VOCs). In fresh smoke the excess concentrations of individual VOCs (Δ*X*) and VOC sums (ΔVOC) tend to be highly correlated with ΔCO (Yokelson et al., 1999; Gilman et al. 2015). The emission ratios of individual VOCs to CO (Δ*X/*ΔCO) can vary considerably with combustion conditions such as fuel type and condition (e.g., moisture content and decay state); fuel bed properties, such as bulk density; and the relative mix of flaming and smoldering combustion (Gilman et al., 2015; Koss et al., 2018). Additionally, the response of Δ*X/*ΔCO to burn conditions varies among VOCs. When each burn is considered individually or in groups with similar conditions, the correlations between ΔO_3_, CO, and THC are enhanced. An example of this behavior is shown in Fig. S10. For the chamber burns the magnitude of the ozone artifacts in parts per billion of apparent O_3_ per part per million of CO, ranges between 6–210 ppb ppm^−1^ for the individual burns. *R*^2^ and standard error values were consistent with those observed during the prescribed burns (see Table 4). The lack of a consistent relationship between the O_3_ artifact and ΔCO across all FSL chamber burns, while observing a good correlation for individual burns, likely reflects the variable response of artifact-producing emission(s) to the different combustion conditions of the burns.

One interesting observation from the data obtained from both the prescribed grassland and chamber burns is the order of magnitude difference in the average and maximum O_3_ artifact between the UV-C and the UV-C-H instruments as shown in Table 3. Considering that the prescribed grassland and chamber burns were conducted under dry (RH <50%) conditions, the size of the difference (as large as hundreds of ppb) cannot be explained purely by the previously observed relative humidity effects on measurements (Leston et al., 2005; Wilson et al., 2006), suggesting that the Nafion® dryer is directly impacting the concentrations of other interferents in the sample stream.

### 3.5 Potential reason for lower artifacts with methods employing Nafion®-based humidity equilibration

Nafion® is a sulfonated tetrafluoroethylene polymer that is highly permeable to water but shows little permeability to many other organic and inorganic species (Mauritz and Moore, 2004). As a result, Nafion®-based drying systems are often used as part of sample preparation or conditioning systems in analytical chemistry to remove water vapor from sample streams prior to sample analysis. Nafion® membranes were introduced to some O_3_ monitors as a method to address humidity effects observed in UV-C O_3_ monitors, particularly when there are rapid changes in relative humidity level (Wilson and Birks, 2006). Humidity can affect the transmission of the UV light through the detection cell, and catalytic O_3_ scrubbers can modulate the water vapor in the scrubbed channel by acting as a temporary reservoir, resulting in significant positive or negative O_3_ interferences during rapid swings in relative humidity (Wilson et al., 2006). Adding a Nafion®-based equilibration dryer immediately prior to the measurement cells reduces this water vapor interference without affecting O_3_ concentrations and thus significantly reduces the humidity artifacts in UV photometric O_3_ instruments.

Despite the high selectivity of Nafion® to water vapor, it does demonstrate partial to complete permeability to various VOC or semivolatile organic compounds. Nafion® membranes are highly permeable to alcohols, amines, ketones, and some water-soluble ethers (Baker, 1974), as well as some biogenic oxygenated compounds (Burns et al., 1983). In addition, Nafion® membranes have been shown to catalyze the decomposition and rearrangement of monoterpene compounds (Burns et al., 1983). Systematic study of Nafion® permeability and reactivity for polar and oxygenated compounds has been limited, with most users of Nafion® membranes basing their use on operational testing and confirmation for specific applications.

The significant (order of magnitude) reduction in the O_3_ artifact with the addition of a Nafion®-based dryer to the UV-C suggests that the Nafion® dryer is directly impacting the major interfering species, which was hypothesized to be VOCs emitted during combustion processes. The species that are responsible for most of the O_3_ artifact in UV-C O_3_ instruments would have to be permeable through Nafion® membranes or reactive with Nafion® membranes; would have to be scrubbed by solid-phase, catalytic O_3_ scrubbers (such as MnO_2_ or hopcalite); and would have a significant absorption cross section around 254 nm. The absorption cross section of O_3_ around 254 nm is on the order of 10^−17^ cm^2^ molec.^−1^ (Molina and Molina, 1986), which means species with absorptions around 10^−17^ cm^2^ molec.^−1^ at 254 nm would be potential interfering species. As a class, aromatic VOCs and specifically oxygenated aromatic species (and other polar-derivatized species) absorb strongly in this region of the UV spectrum, and their potential permeability through Nafion® membranes results in them being likely compounds to interfere in UV-C instruments. As an example, aromatic aldehydes such as *o*-tolualdehyde and *p*-tolualdehyde absorb around 5×10^−18^ cm^2^ molec.^−1^ and 4×10^−18^ cm^2^ molec.^−1^, respectively (Etzkorn et al., 1999). Both 2,4-dimethylbenzaldehyde and 2,6-dimethylbenzaldehyde have absorption cross sections above 10^−17^ cm^2^ molec.^−1^ at 254 nm (El Dib et al., 2008). Baker (1974) found 75% of benzaldehyde was removed by a Nafion® membrane, meaning that the Nafion® permeability of tolualdehydes and dimethylbenzaldehydes is also likely to be high. In addition, benzaldehyde was almost quantitatively removed by several commercial catalytic O_3_ scrubbers, including the Thermo 49i MnO_2_ catalytic scrubber (Kleindienst et al., 1993), so similar aldehydes are likely to behave in a similar manner. Therefore, substituted aromatic aldehyde species are one class of compounds that fit the necessary criteria for causing the interference on the UV-C while having a reduced interference on the UV-C-H instrument. Future work examining the potential interferences from different species (or classes of species) on a speciesor class-specific basis are required to confirm this potential mechanism and suggest others.

## 4 Implications

Wildland fires (wildfires and prescribed fires) emit significant amounts of VOCs and NO_*x*_, two important precursors in the photochemical formation of tropospheric O_3_. Therefore, it is not surprising that large increases in O_3_ are routinely reported at ambient monitoring sites downwind from wildland fires (DeBell et al., 2004; Bytnerowicz et al., 2010; Preisler et al., 2010; Jaffe and Wigder, 2012; Bytnerowicz et al., 2013; Jaffe et al., 2013; Lu et al., 2016; Lindaas et al., 2017; Baylon et al., 2018; Liu et al., 2018; McClure and Jaffe, 2018). For example, Buysse et al. (2019) examined regulatory air monitoring data from 18 cities over a 5-year period and found that July–September exceedances of NAAQS for O_3_ were far more common on days with known wildland fire smoke impacts (4.6%) than those without (<0.1%). However, the results of this study suggest caution when interpreting UV photometric method O_3_ measurements under conditions of wildfire smoke impact due to the significant positive artifacts that were observed. The analytical artifacts were also shown to be positively correlated with widely used markers of combustion such as CO and THC suggesting that the artifacts arise from photometric measurement interferences by VOCs and further complicate the interpretation of smoke-impacted UV photometric O_3_ data. As described in Sect. 3.4, it is reasonable to assume that O_3_ artifacts in the range of a few parts per billion to greater than 250 ppb in addition to actual photochemically formed O_3_ can be observed when employing UV photometric methods at sites downwind from fires.

A detailed example of observed artifacts in the UV photometric method occurred during the 2016 Fort McMurray Horse River wildfire in Alberta, Canada, where elevated O_3_ concentrations were observed at multiple community-based air monitoring sites utilizing UV-C instruments in the vicinity of the fire (Landis et al., 2018). Reported O_3_ concentrations reached maximum hourly concentrations in excess of 1500 ppb using UV-C methods at night (between 22:00 and 5:00 local) in the absence of photochemistry and were positively correlated with the combustion markers NO and non-methane hydrocarbons (NMHCs). Peaks in O_3_ concentration are expected to be negatively correlated with peaks in NO concentration as it rapidly titrates O_3_ to NO_2_, and the authors hypothesized that UV photometric measurement artifacts may have been responsible for the unexpected observations.

The findings from this research effort and the observations from ambient studies (Landis et al., 2018; Akagi et al., 2012) raise concerns that routine regulatory monitoring and wildland fire research study O_3_ measurements utilizing UV photometric FEM instruments may be reporting positive measurement artifacts as O_3_ during smoke-impacted events. Some studies have hypothesized that rapid photochemical processing was responsible for elevated O_3_ concentrations reported in downwind wildfire plumes (Liu et al., 2017). Since downwind O_3_ production in biomass burning plumes is a significant issue in fire-impacted regions, having reliable, interference-free methods is critical for assessing the contribution of wildland fires to ambient O_3_ levels and developing and validating accurate deterministic air quality models. Air quality researchers and environmental regulators are strongly encouraged to utilize NO-CL FRM O_3_ instruments in areas routinely impacted by wildland fire smoke.

## 5 Conclusions

In this study, we compare two different O_3_ measurement methods (chemiluminescence and UV photometry) in fresh biomass burning plumes from prescribed grassland fires and during controlled chamber burns. Within the UV photometry category, we look at two different technologies, one using a gas-phase chemical scrubber (NO) and the second using solid phase catalysts to scrub O_3_ from analytical reference channels. Among the UV photometric instruments employing solid phase catalytic scrubbers, we evaluated and compared methods that include a Nafion®-based humidity equilibration system with those that do not.

The NO-CL method (recently promulgated as the O_3_ FRM) performed well even in fresh plumes, whereas the UV photometric methods displayed varying degrees of positive measurement artifacts. The UV photometric method employing the dynamic NO gas-phase scrubber performed comparably with the NO-CL method but was not well suited to the rapidly varying concentrations of VOCs in the smoke plumes. The catalytic scrubber photometric methods demonstrated positive analytical artifacts that were correlated with CO and THC concentrations (both biomass burning plume indicators). There was a significant difference between the catalytic scrubber UV instruments with and without Nafion®-based humidity correction, with the dryer system reducing the positive O_3_ artifact by an order of magnitude compared with the UV photometric method employing no humidity correction. The observed reduction in artifacts cannot be attributed only to elimination of the relative humidity and water vapor interferences and likely results from post-scrubber equilibration or reaction of Nafion®-permeable VOCs prior to the measurement cell. The results of this study strongly suggest that careful consideration be given to employed measurement methods when monitoring O_3_ concentrations in regions where impacts from biomass burning routinely occur due to the significant impact of potential measurement interferences. In addition to consideration of operating methods containing Nafion®-based humidity condition systems, attention should be focused on the scrubbers employed by UV photometric methods and the adverse effects that operation in smoke may have on those scrubbers. Further research is being conducted to evaluate the magnitude of the artifact in the UV photometric method at routine monitoring sites that are often impacted by wildland fire smoke events under the EPA Mobile Ambient Smoke Investigation Capability (MASIC) program (U.S. EPA, 2019).

## Supplementary Material

Supplement1

## Figures and Tables

**Table 1.** Ozone measurement methods investigated. N/A – Not applicable

Name	Manufacturer	Model	Method	Scrubber	Cells	Humidity correction	Deployment*
U.S. EPA Federal Reference Methods (FRM)							
NO-CL	Teledyne API	T-265	CL (NO)	N/A	1	Nafion^®^-based (dryer)	K1, S, K2, T, M1, M2
U.S. EPA Federal Equivalent Methods (FEM)							
UV-C	Thermo Scientific	49i	UV (254 nm)	Catalyst (MnO_2_)	2	None	K1, S, K2, T, M1, M2
UV-C-H	2B Technologies	205	UV (254 nm)	Catalyst (hopcalite)	2	Nafion^®^-based (equilibration)	K1, S, K2, T, M1
SL-UV	2B Technologies	211	UV (254 nm)	Gas chemical (NO)	2	Nafion^®^-based (equilibration)	K1, M1, M2
UV-G	2B Technologies	211-G	UV (254 nm)	Heated graphite	2	Nafion^®^-based (equilibration)	M2

* K1 – Konza Prairie March 2017; S – Sycan Marsh, October 2017; K2 – Konza Prairie November 2017; T – tallgrass prairie November 2017; M1 – Missoula chamber April 2018; M2 – Missoula chamber April 2019.

**Table 2.** Additional measurement methods operated during the present study.

Pollutant	Manufacturer	Model	Method	FRM/FEM	Deployment^f^

CO	Teledyne API	48C	NDIR^a^	FRM	K1, S, K2, T, M1, M2
NO_2_	Teledyne API	T500U	CAPS^b^	FEM	K1, S, K2, T, M1, M2
NO, NO_2_, NO*_x_*	Thermo Scientific	42C	CL (O_3_)^c^	FRM	K1, K2, T, M1
NO, NO_2_, NO*_x_*	Teledyne API	T200/T201^e^	CL (O_3_)	FRM	M1, M2
THC	Thermo Scientific	51i	FID^d^	NA	K2, T, M1, M2

a Non-dispersive infrared absorption.

b Cavity-attenuated phase shift.

c Ozone chemiluminescence.

d Flame ionization detector.

e The Teledyne API model T201 is not a designated FRM or FEM; however it employs the same operating principle as the FRM-designated model T200.

f K1 – Konza Prairie March 2017; S – Sycan Marsh October 2017; K2 – Konza Prairie November 2017; T – tallgrass prairie November 2017; M1 – Missoula chamber April 2018; M2 – Missoula chamber April 2019.

**Table 3.** Ozone artifact (ΔO_3_) averages, maximum values, and CO, NO_2_, and THC averages from the prescribed fire and USFS chamber burns as measured by the UV-C, UV-C-H, and UV-G instruments.

Study	ΔO_3_ avg. (ppb)	ΔO_3_ max (ppb)	CO avg. (ppm)	NO_2_ avg. (ppb)	THC avg. (ppm)

ΔO_3(UV-C)_					

Mar. 2017 Konza Prairie (KS)	295.8	2423.7	15.8	147.3	–
Oct. 2017 Sycan Marsh (OR)	170.2	3235.5	8.5	60.5	2.7
Nov. 2017 Konza & tallgrass prairies (KS)	330.0	3156	14.1	156.9	4.0
Apr. 2018 USFS chamber (MT)	36.5	309.6	3.8	35.6	2.8
Apr. 2019 USFS chamber (MT)	66.9	530.9	2.1	31.7	4.8

ΔO_3(UV-C-H)_					

Mar. 2017 Konza Prairie (KS)	42.8	227.1	15.8	147.3	–
Oct. 2017 Sycan Marsh (OR)	21.1	316.4	8.5	60.5	2.7
Nov. 2017 Konza & tallgrass prairies (KS)	40.2	369.0	14.1	156.9	4.0
Apr. 2018 USFS chamber (MT)	7.2	136.8	3.8	35.6	2.8

ΔO_3(UV-G)_					

Apr. 2019 USFS chamber (MT)	22.9	376.8	2.1	31.7	4.8

ΔO_3(SL-UV)_					

Mar. 2017 Konza Prairie (KS)	8.3	74.2	15.8	147.3	–
Apr. 2018 USFS chamber (MT)	0.5	11.5	3.8	35.6	2.8
Apr. 2019 USFS chamber (MT)	1.7	32.1	2.1	31.7	4.8

**Table 4.** Regression statistics for the ozone artifact (ΔO_3_) versus CO and THC for UV photometric instruments without (UV-C) and with (UV-C-H) a Nafion^®^-based humidity equilibration system during the 2017 prescribed grassland burns.

Study	Slope (ppb/ppm)	Intercept (ppb)	*r*^2^	*n*

ΔO_3(UV-C)_ vs. CO				

Mar. 2017 Konza Prairie (KS)	16.46 (±0.34)^a^	18.53 (± 6.72)^b^	0.79	653
Oct. 2017 Sycan Marsh (OR)	24.02 (±0.25)	−28.05 (±2.73)	0.96	295
Nov. 2017 Konza & tallgrass prairies (KS)	23.51 (±0.73)	−20.8 (±13.03)	0.74	461

ΔO_3(UV-C)_ vs. THC				

Nov. 2017 Konza & tallgrass prairies (KS)	87.14 (±3.74)	−85.36 (±18.63)	0.59	461

ΔO_3(UV-C-H)_ vs. CO				

Mar. 2017 Konza Prairie (KS)	1.46 (±0.04)	0.87 (±1.03)	0.80	163
Oct. 2017 Sycan Marsh (OR)	2.21 (±0.05)	3.44 (±0.54)	0.88	296
Nov. 2017 Konza & tallgrass prairies (KS)	3.24 (±0.09)	−1.17 (±1.67)	0.77	461

ΔO_3(UV-C-H)_ vs. THC				

Nov. 2017 Konza & tallgrass prairies (KS)	13.27 (±0.39)	−14.53 (±1.92)	0.75	461

THC vs. CO				

Nov. 2017 Konza & tallgrass prairies (KS)	0.21 (±0.004)	1.55 (±0.08)	0.79	461

a Standard error or uncertainty of the linear regression slope in parts per billion per part per million.

b Standard error or uncertainty of the linear regression intercept in parts per billion.
