# Supplementary material for: Comparison of ozone measurement methods in biomass burning smoke: an evaluation under field and laboratory conditions
Source: Atmos Meas Tech. Author manuscript; Available in PMC 2022 Mar 4. (PMC8128704; doi:10.5194/amt-14-1783-2021)
Supplement: Supplement1 [file NIHMS1689672-supplement-Supplement1.pdf]

Tables.

Table S1. Ozone method manufacturer provided performance specifications.

Figures.

Figure S1. Time series of O_3, CO, and NO_2 for the four March 2017 Kansas burn days.

Figure S2. Time series of O_3, CO, and NO_2 for the October 2017 Sycan Marsh (Oregon) burn day.

Figure S3. Time series of O_3, CO, and NO_2 for the November 2017 Kansas burns. The November 10 burn occurred at Konza Prairie and the November 13 and 15 burns were conducted at Tallgrass Prairie.

Figure S4. Time series of O_3, CO, and NO_2 for the 2018 USFS chamber burns in Missoula, MT.

Figure S5. Time series of O_3, CO, and NO_2 for the 2019 USFS chamber burns in Missoula, MT.

Figure S6. Time series of the UV-C positive O_3 analytical artifact ($\Delta O_{3(UV\text{-}C)}$) and CO comparison from the three prescribed burning periods. Top-Konza Prairie (KS) March 2017; middle-Sycan Marsh (OR) October 2017; and bottom-Konza Praire and Tallgrass Prairie (KS) November 2017.

Figure S7. Scatter plots between O_3 differences and the THC measurements within the grassland fires smoke plumes.

Figure S8. Scatter plots between ΔO_3 and the CAPS NO_2 measurements within the grassland fires smoke plumes.

Figure S9. Scatter plots between FRM and FEM O_3 differences and CO, NO_2, and THC for all in-plume (burn period only) measurements made during the 2018 and 2019 Missoula Fire Chamber studies. Observation points have been colored by the O_3 instrument. Over all observations there is little correlation between the O_3 instrument differences, but straight line structures within the overall scatters indicate that individual burn events measured in the chamber have good correlations with distinct ratios.

Figure S10. Scatter plots between FRM and FEM O_3 differences and CO, NO_2, and THC for measurements collected in-plume during a single burn event during the 2018 and 2019 Missoula Fire Chamber campaigns. Observation points have been colored by the O_3 instrument. In Figure S9, the regressions had low overall correlation over all in-smoke points, individual burn events measured in the chamber have good correlations with distinct ratios.

Data.

Meta data
Konza Prairie March 2017
Sycan Marsh October 2017
Konza Prairie November 2017
Tallgrass Prairie November 2017
USFS Missoula Chamber 2018
USFS Missoula Chamber 2019

Table S1. Ozone method manufacturer provided performance specifications.

Performance Parameter	TAPI T265 (NO-CL)	Thermo 49i (UV-C)	2B 205 (UV-C-H)	2B 211 (SL-UV)	2B 211-G (UV-G)
**Sample Flow Rate**	500 cc/min ±10%	1 L/min	1.8 L/min	2 L/min	2 L/min
**Range**	0-2000 ppb	0 – 200 ppm	0 – 250 ppm	0-2000 ppb	0 – 500 ppb
**Noise**[1]	< 0.15 ppb	< 0.25 ppb	NA[2]	NA[2]	NA[2]
**LDL**[3]	< 0.3 ppb	0.5 ppb	2.0 ppb	1.0 ppb	1.0 ppb
**Zero drift**[4]	< 0.5 ppb	< 1.0 ppb	< 1.0 ppb	< 1.0 ppb	< 1.0 ppb
**Span drift**[5]	< 0.5%	< 1.0%	< 1.0%	< 0.5%	< 0.5%
**Precision**[6]	< 0.5%	< 1%	< 2%	< 1%	< 1%

[1]Noise is determined as the standard deviation of a series of measurements of a constant concentration about the mean and is expressed in concentration units.
[2]Values not specified by manufacturer
[3]Lower detectable limit
[4]Zero drift is the change in measurement response to zero pollutant concentration over a 24-hour period of continuous unadjusted operation.
[5]Span drift is the percent change in measurement response to an up-scale (80% of the upper range level) pollutant concentration over a 24-hour period of continuous unadjusted operation.
[6]Precision is the variation about the mean of repeated measurements of the same pollutant concentration, denoted as the standard deviation expressed as a percentage of the upper range limit.

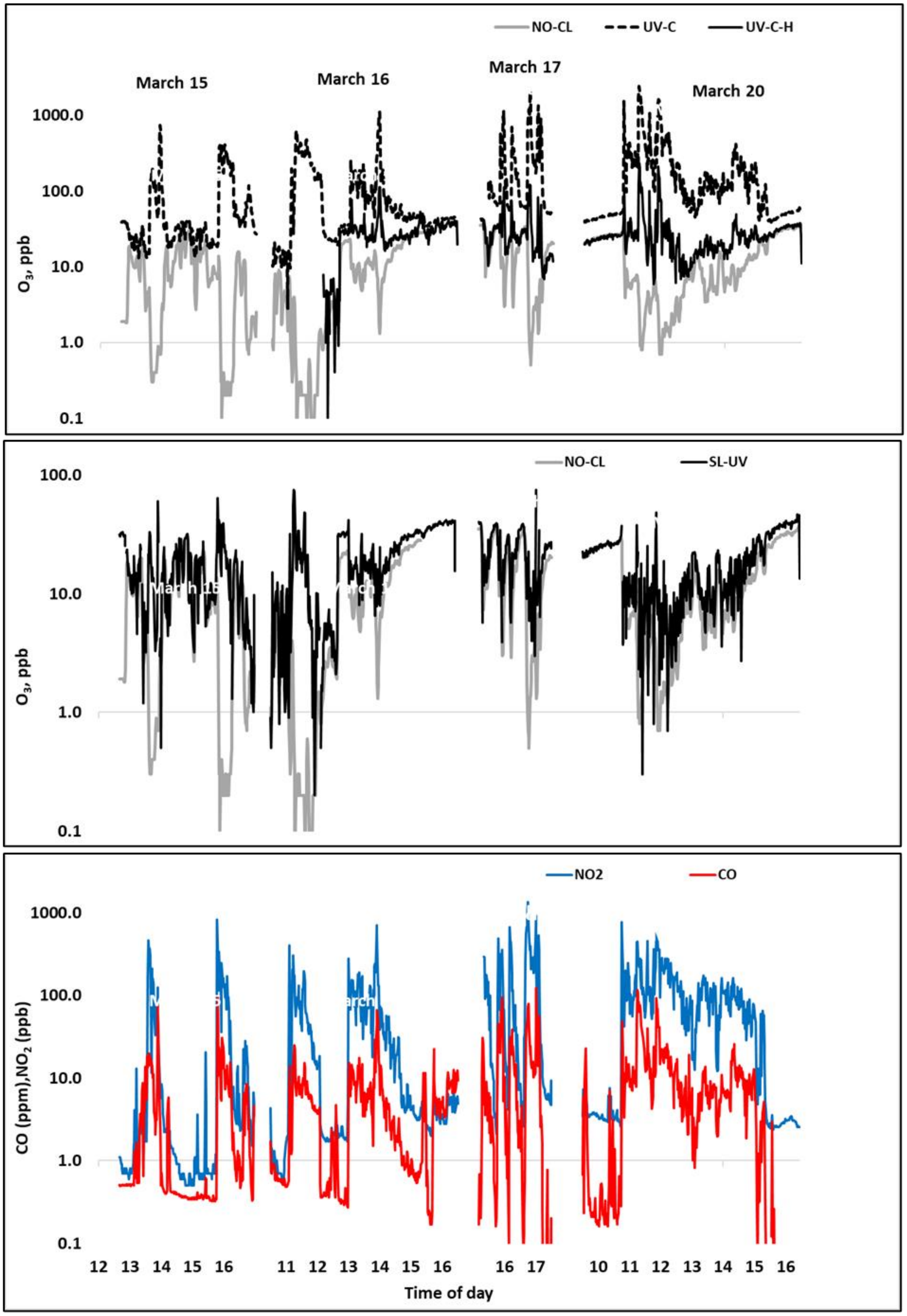

Figure S1. Time series of O_3, CO, and NO_2 for the four March 2017 Kansas burn days. All concentrations are displayed in a logarithmic scale.

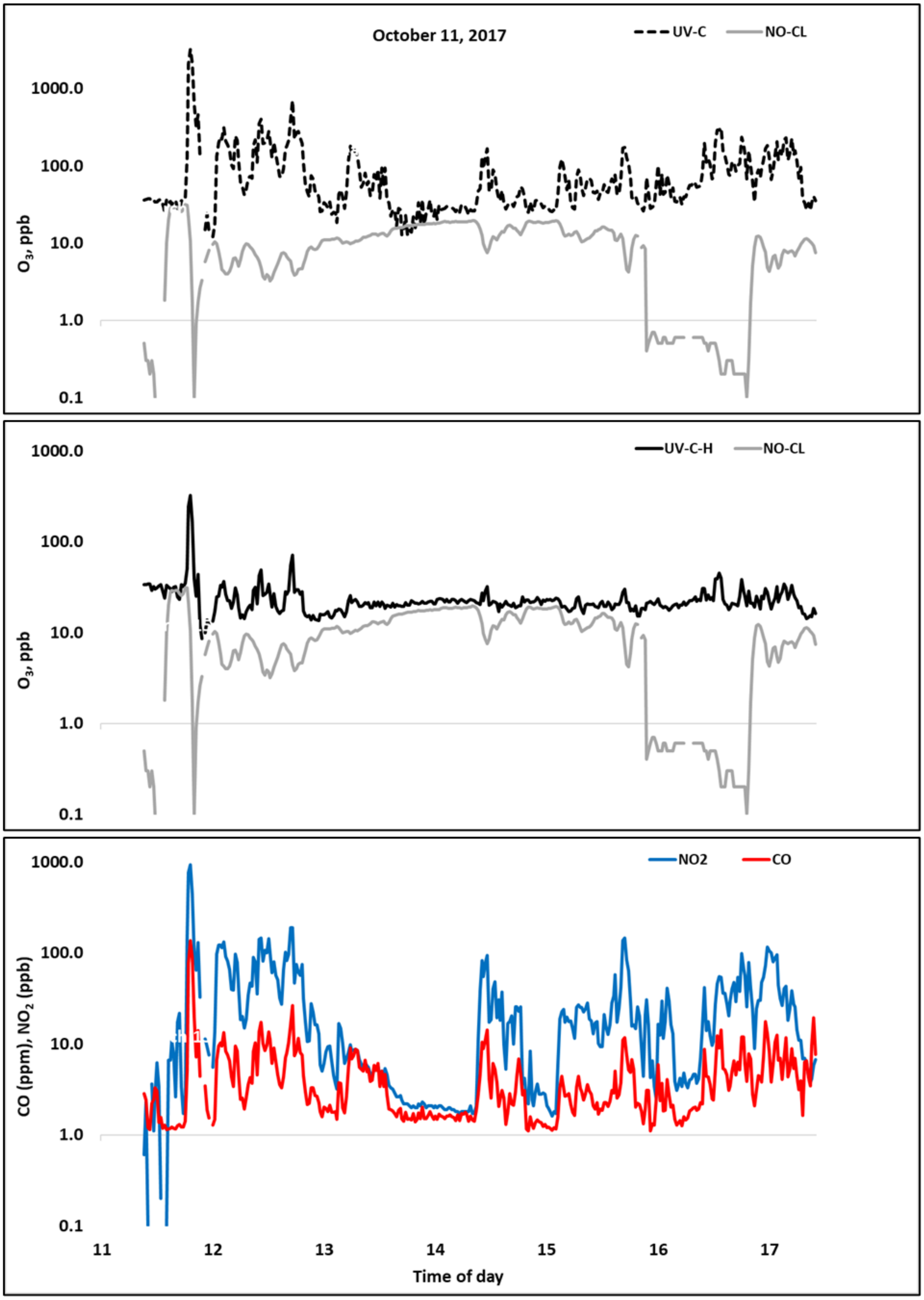

Figure S2. Time series of O_3, CO, and NO_2 for the October 2017 Sycan Marsh (Oregon) burn day. All concentrations are displayed in a logarithmic scale.

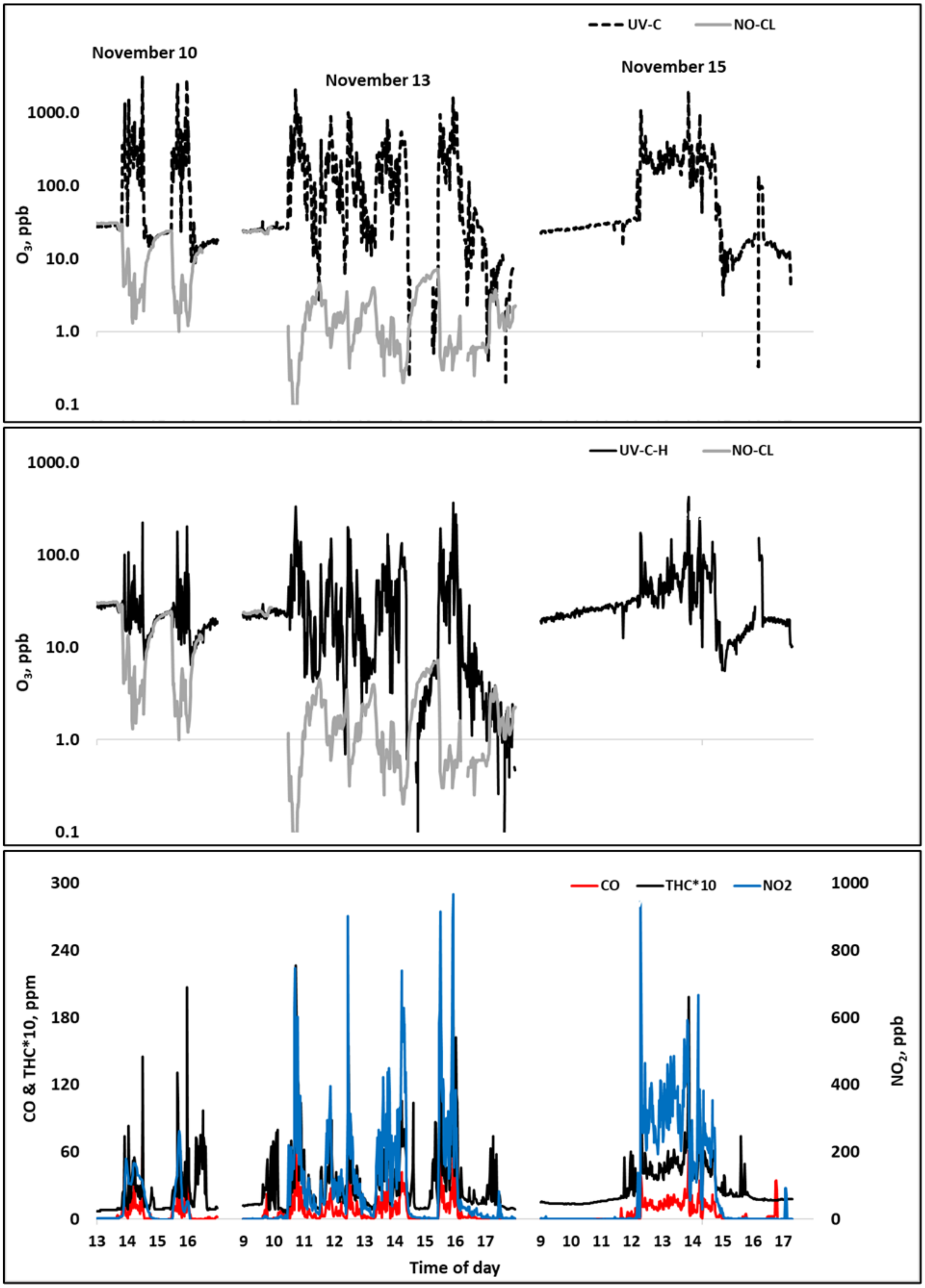

Figure S3. Time series of O_3, CO, and NO_2 for the November 2017 Kansas burns. The November 10 burn occurred at Konza Prairie and the November 13 and 15 burns were conducted at Tallgrass Prairie. Ozone concentrations are displayed in a logarithmic scale.

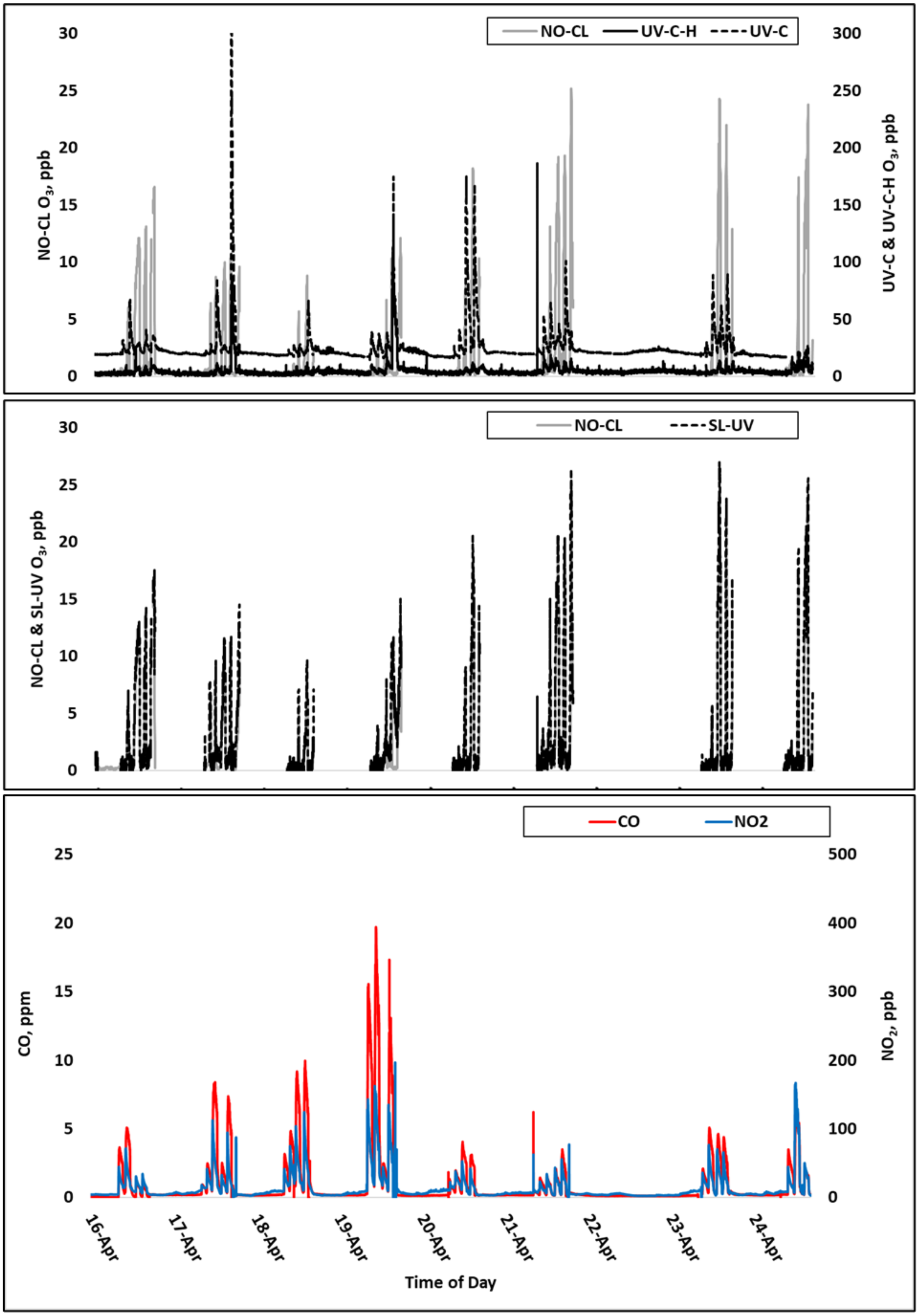

Figure S4. Time series of O_3, CO, and NO_2 for the 2018 USFS chamber burns in Missoula, MT.

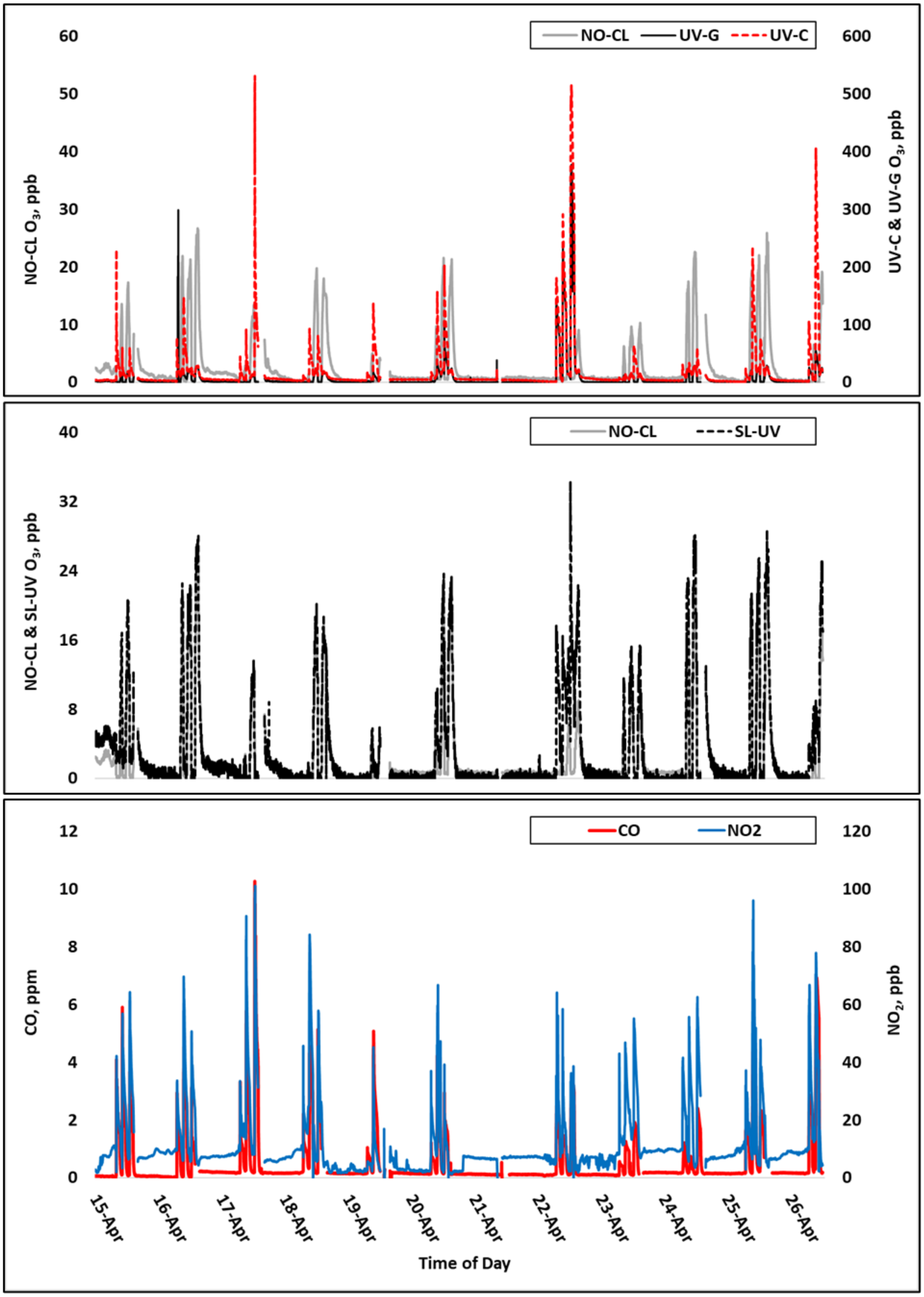

Figure S5. Time series of O_3, CO, and NO_2 for the 2019 USFS chamber burns in Missoula, MT.

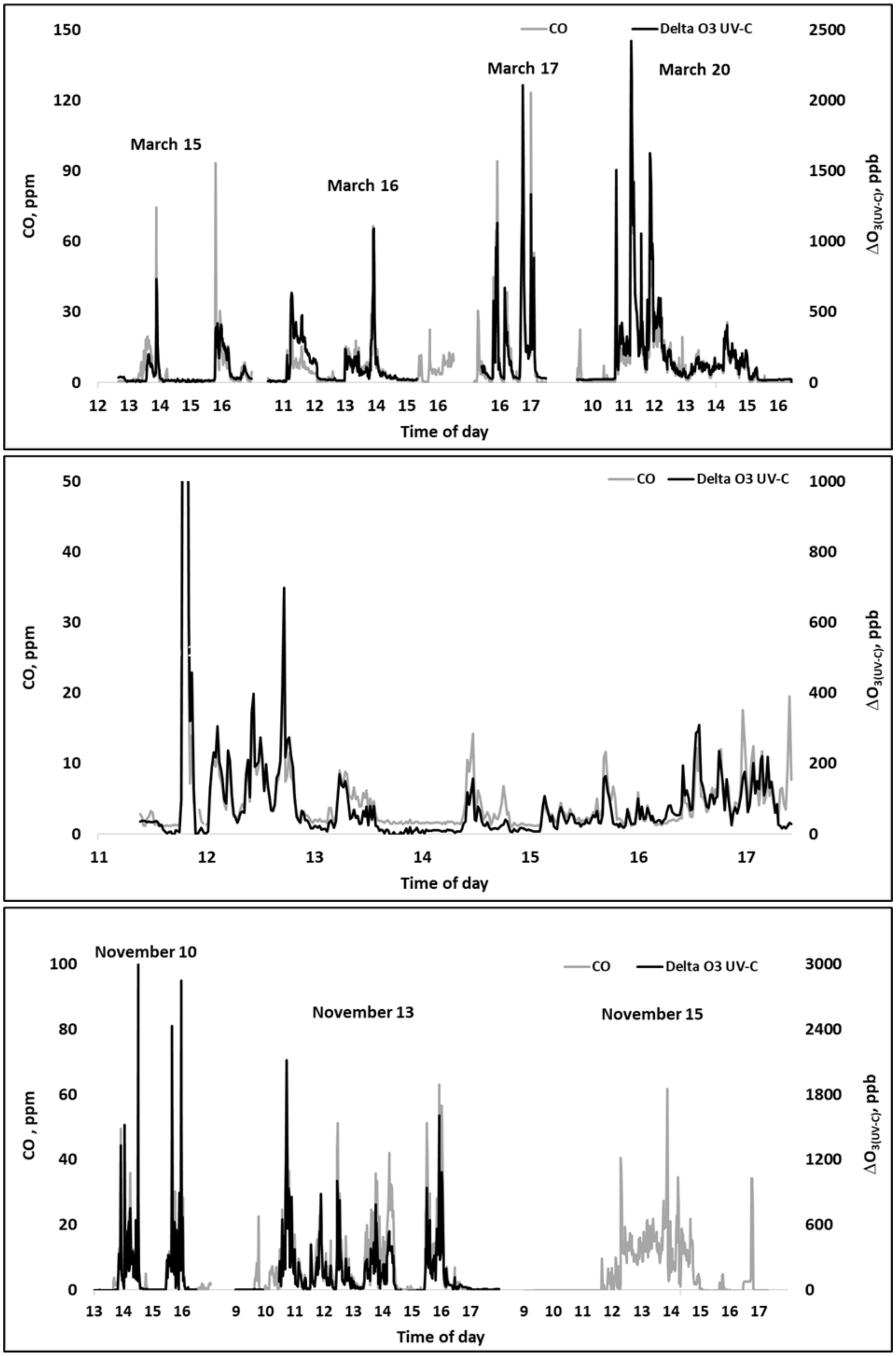

Figure S6. Time series of the UV-C positive O_3 analytical artifact ($\Delta O_{3(UV\text{-}C)}$) and CO comparison from the three prescribed burning periods. Top-Konza Prairie (KS) March 2017; middle-Sycan Marsh (OR) October 2017; and bottom-Konza Praire and Tallgrass Prairie (KS) November 2017.

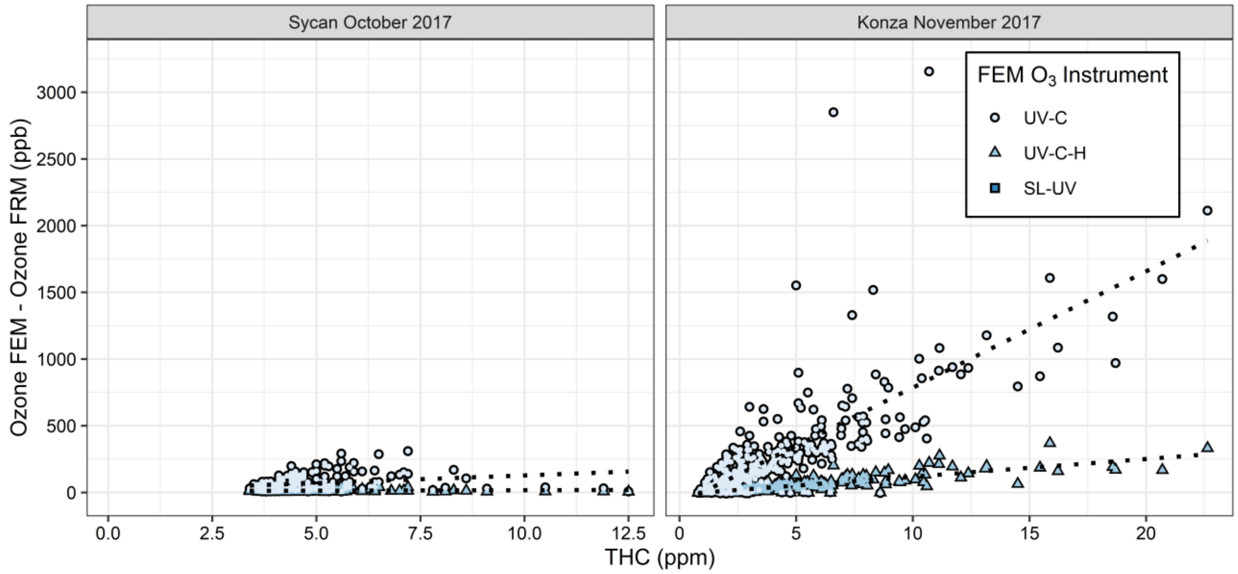

Figure S7. Scatter plots between Ozone differences and the THC measurements within the grassland fires smoke plumes.

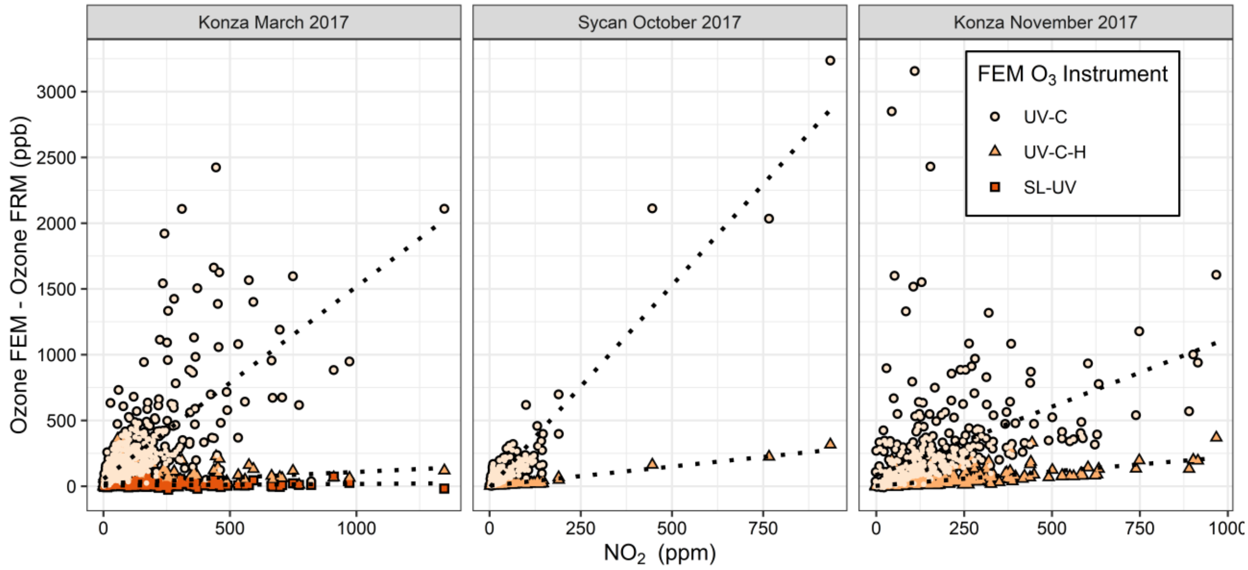

Figure S8. Scatter plots between ΔO_3 and the CAPS NO_2 measurements within the grassland fires smoke plumes.

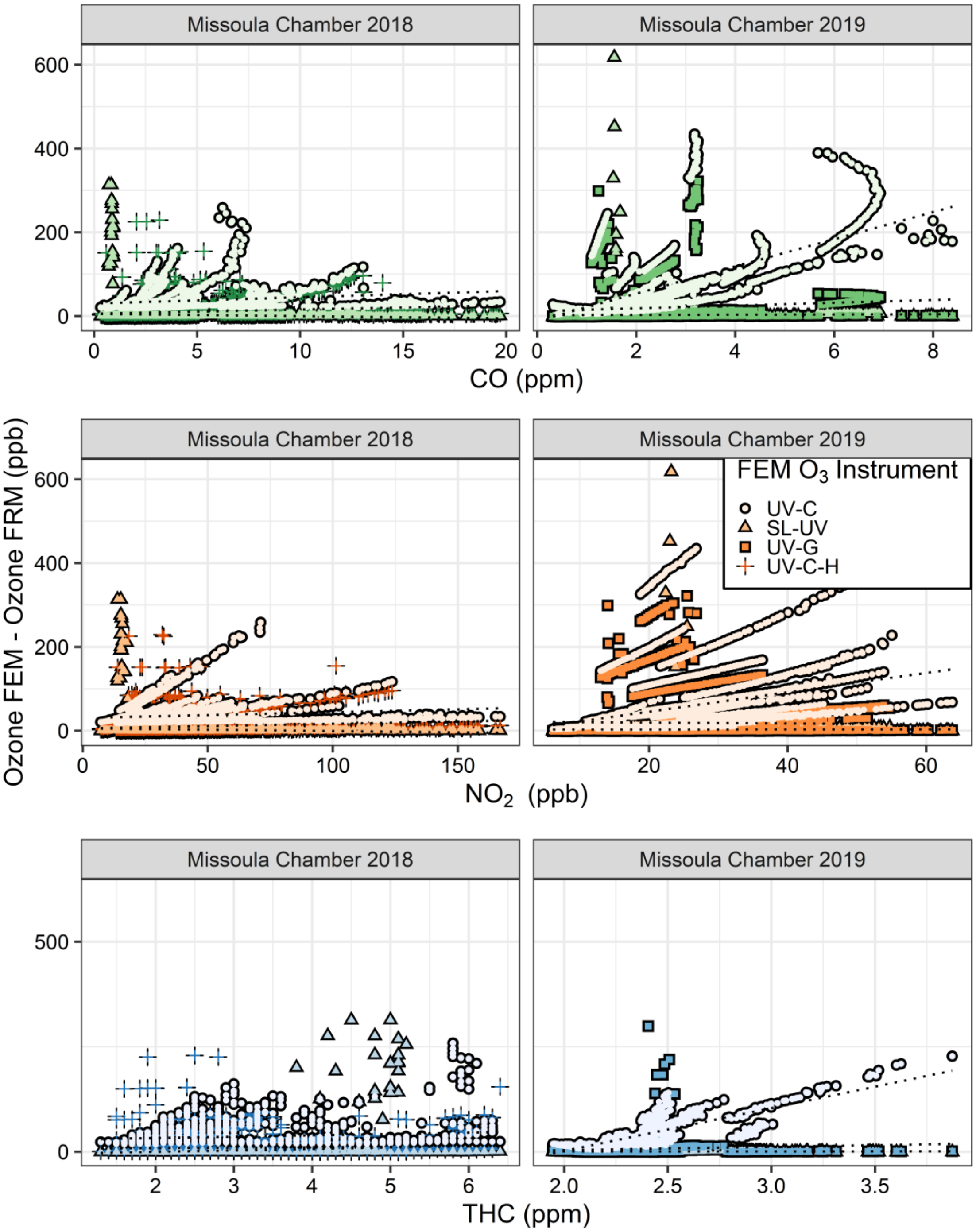

Figure S9. Scatter plots between FRM and FEM O_3 differences and CO, NO_2, and THC for all in-plume (burn period only) measurements made during the 2018 and 2019 Missoula Fire Chamber studies. Observation points have been colored by the O_3 instrument. Over all observations there is little correlation between the O_3 instrument differences, but straight line structures within the overall scatters indicate that individual burn events measured in the chamber have good correlations with distinct ratios.

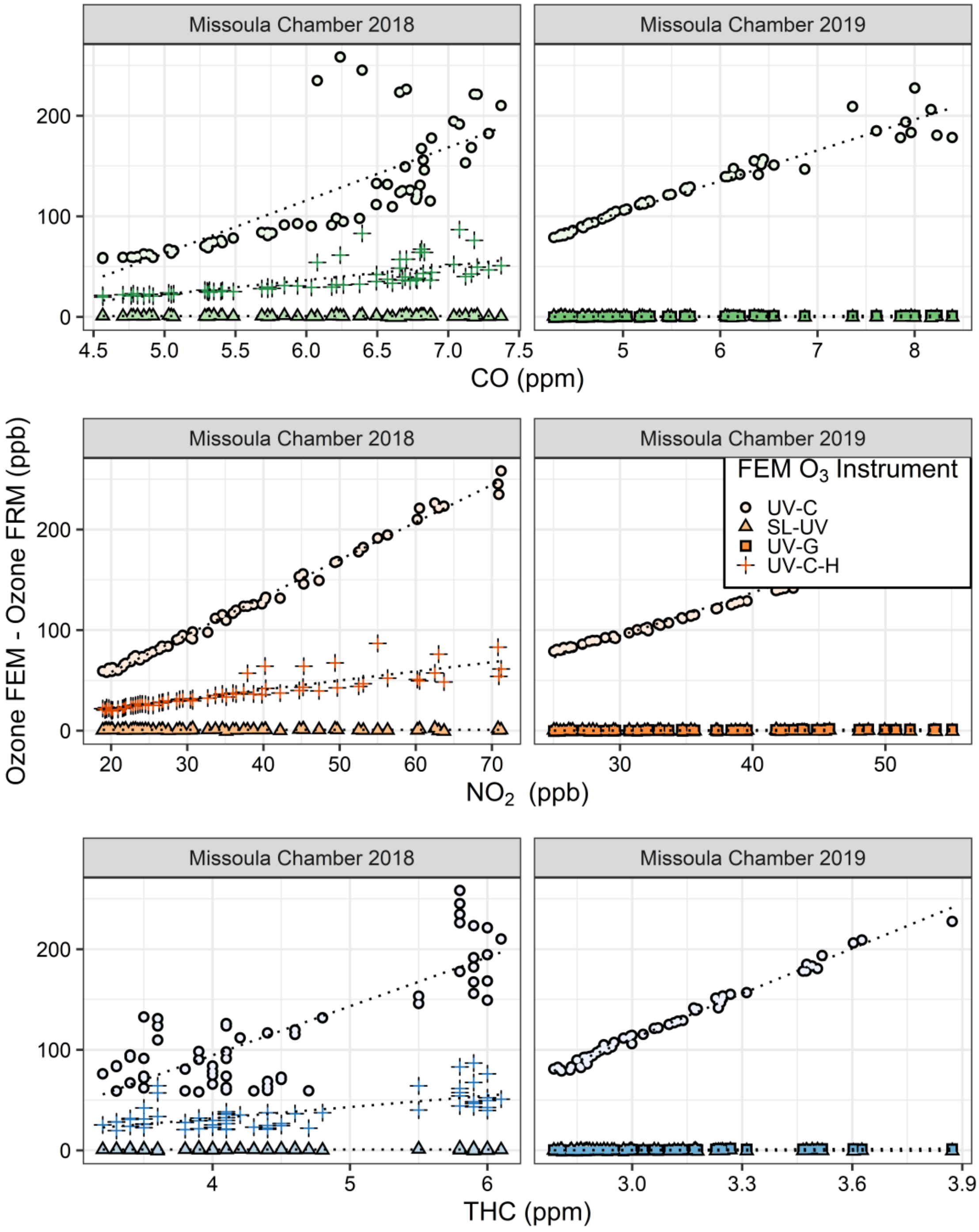

Figure S10. Scatter plots between FRM and FEM O_3 differences and CO, NO_2, and THC for measurements collected in-plume during a single burn event during the 2018 and 2019 Missoula Fire Chamber campaigns. Observation points have been colored by the O_3 instrument. In Figure S9, the regressions had low overall correlation over all in-smoke points, but individual burn events measured in the chamber have good correlations with distinct ratios.

Variable	Instrument	Units/Format	Comments
Date&Time	---	MM/DD/YYYY HH:MM	
O3 NO-CL	Teledyne API Model T265 Ozone FRM Analyzer	ppb	FRM - NO Chemiluminescence
O3 SL-UV	2B Technologies Model 211 Ozone FEM Analyzer	ppb	FEM - UV Photometric
O3 UV-C-H	2B Technologies Model 205 Ozone FEM Analyzer	ppb	FEM - UV Photometric
O3 UV-C	Thermo Environmental Model 49C/49i Ozone FEM Analyzer	ppb	FEM - UV Photometric
O3 UV-G	2B Technologies Model 211-G Ozone FEM Analyzer	ppb	FEM - UV Photometric
NO2-C	Teledyne API Model T500U Nitrogen Dioxide FEM Analyzer	ppb	FEM - Cavity Attenuated Phase Shift
NO	Teledyne API Model T200 Nitrogen Oxide FRM Analyzer	ppb	FRM - O3 Chemiluminescence
CO	Thermo Environmental Model 48C Carbon Monoxide FRM Analyzer	ppm	FRM - NDIR
THC	Thermo Environmental Model 51i Total Hydrocarbon Analyzer	ppm	

FRM Federal Reference Method; FEM - Federal Equivalent Method

ppb - part per billion; ppm - part per million

Teledyne API - San Diego, CA; 2B Technologies - Boulder, CO; Thermo Environmental - Franklin, MA

Study	Locations	Dates
Konza Mar 2017	Konza Prairie Long Term Ecological Research site near Manhattan, KS	March 15-20, 2017
Sycan Oct 2017	Sycan Marsh Preserve in central Oregon	October 11, 2017
Konza Nov 2017	Konza Prairie Long Term Ecological Research site near Manhattan, KS	November 10, 2017
Tallgrass Nov 2017	Tall Grass Prairie National Preserve near Strong City, KS	November 13-15, 2017
Missoula Lab 2018	Burn chamber at USFS Fire Sciences Laboratory in Missoula, MT	April 16-24, 2018
Missoula Lab 2019	Burn chamber at USFS Fire Sciences Laboratory in Missoula, MT	April 15-26, 2019

Konza Prairie March 2017

datetime	O3 NO-CL	O3 SL-UV	O3 UV-C-H	O3 UV-C	NO2-C	CO
3/15/2017 12:40	1.9	31.7		39	1.1	0.51
3/15/2017 12:41	1.9	30.3		38.7	1.1	0.49
3/15/2017 12:42	1.9	32.5		39.7	1	0.5
3/15/2017 12:43	1.9	32.6		39.5	0.9	0.5
3/15/2017 12:44	1.9			39.4	0.9	0.5
3/15/2017 12:45	1.9	32.7		39.5	0.8	0.51
3/15/2017 12:46	1.9	33.1		39.3	0.7	0.51
3/15/2017 12:47	1.9	31.7		38.7	0.7	0.51
3/15/2017 12:48	1.9	30.3		37.7	0.8	0.5
3/15/2017 12:49	1.8	30.6		37.1	0.8	0.5
3/15/2017 12:50	1.8	31.3		41	0.7	0.51
3/15/2017 12:51	2.3	25.2		34	0.8	0.5
3/15/2017 12:52	7.1			32.1	0.8	0.5
3/15/2017 12:53	12	22.2		30.5	0.8	0.52
3/15/2017 12:54	17.8	23.6		31.8	0.7	0.5
3/15/2017 12:55	18.8	18.2		25.8	0.7	0.51
3/15/2017 12:56	16.9	14.3		21.9	0.7	0.5
3/15/2017 12:57	14.8	17.9		25.6	0.7	0.52
3/15/2017 12:58	13.4	15.7		23.1	0.6	0.51
3/15/2017 12:59	13.5	14.8		24.8	0.7	0.51
3/15/2017 13:00	12.9	14.5		20.2	0.8	0.52
3/15/2017 13:01	11.6	10.8		18	0.8	0.49
3/15/2017 13:02	10.8	12.8		21.1	0.7	0.52
3/15/2017 13:03	9.9	12.4		20.9	0.7	0.51
3/15/2017 13:04	9.8	10		16.6	0.8	0.51
3/15/2017 13:05	9.7	14.5		24.6	0.8	0.51
3/15/2017 13:06	9.6	10.3		16.9	0.9	0.53
3/15/2017 13:07	9.8	12.3		21.7	1.6	0.5
3/15/2017 13:08	12.2	20.7		28.2	0.9	0.53
3/15/2017 13:09	13.6	13.6		21.3	4.1	1.28
3/15/2017 13:10	14	13.3		20.9	2.9	1.33
3/15/2017 13:11	12.4	15		22.1	1.5	0.62
3/15/2017 13:12	11.5	11.2		18.7	13	1.66
3/15/2017 13:13	9.2	4.6		12.9	1.4	0.9
3/15/2017 13:14	7.5	9		16.6	1	0.54
3/15/2017 13:15	6.4	11.4		18.8	1	0.54
3/15/2017 13:16	10.2	17.6		26	0.9	0.54
3/15/2017 13:17	13.2	17.9		25.7	0.8	0.54
3/15/2017 13:18	16.4	19.9		28	2.3	2.06
3/15/2017 13:19	15.6	12.7		23.3	3.4	3.67
3/15/2017 13:20	17.4	25.8		36	2.1	2.32
3/15/2017 13:21	19.9	23.5		31.5	2.7	3.68

3/15/2017 13:22	19.9		19.5	2.5	3.18
3/15/2017 13:23	15.9	12	22.4	2.8	6.96
3/15/2017 13:24	9.1	4.4	21.9	2.8	8.74
3/15/2017 13:25	5.4	1.2	16	0.7	0.68
3/15/2017 13:26	2.6	1.4	14.3	1	0.65
3/15/2017 13:27	3	8.4	15.8	2.8	3.69
3/15/2017 13:28	3.9	5	13.9	2.8	3.98
3/15/2017 13:29	4.6	3.5	13	6.9	13.61
3/15/2017 13:30	3.1	3.2	12.7	9.8	9.94
3/15/2017 13:31	3.4	7.1	13.5	1.7	5.38
3/15/2017 13:32	4.5	6.5	15.3	1.7	5.55
3/15/2017 13:33	5.3	5.2	15	10.3	18.25
3/15/2017 13:34	4.3	4.7	28.5	170.5	13.41
3/15/2017 13:35	2.4	21.7	125.7	461.7	19.43
3/15/2017 13:36	0.9	18.3	136.4	321.6	19.65
3/15/2017 13:37	0.4	23.5	148.1	353.1	14.2
3/15/2017 13:38	0.3	33	198.7	366.5	18.03
3/15/2017 13:39	0.3	30.6	197.4	306.6	17.99
3/15/2017 13:40	0.3	19	155.9	68.2	14.88
3/15/2017 13:41	0.3	14.6	129.7	11.6	15.75
3/15/2017 13:42	0.4	11.9	110.8	209.1	11.98
3/15/2017 13:43	0.4	16.7	137.3	158.9	14.19
3/15/2017 13:44	0.4	11	110.7	159.1	9.95
3/15/2017 13:45	0.4	13.5	125.5	142	11.02
3/15/2017 13:46	0.4	8.1	89.4	70	3.85
3/15/2017 13:47	0.4	4.9	66.4	46.2	3.52
3/15/2017 13:48	0.5	3.3	53.8	32.9	2.9
3/15/2017 13:49	0.7	3.3	43.5	55.1	2.08
3/15/2017 13:50	0.9	4	50.2	47.2	3.5
3/15/2017 13:51	0.8	4	50	74.1	3.11
3/15/2017 13:52	0.7	5.1	66	37.5	8.21
3/15/2017 13:53	0.7	53.2	423.7	125.5	74.51
3/15/2017 13:54	0.7	60.3	732.1	59.8	57.18
3/15/2017 13:55	0.7	27.3	634	28	25.78
3/15/2017 13:56	0.9	-6.9	259.4	15.2	9.42
3/15/2017 13:57	1.6	3	158.6	9.6	4.17
3/15/2017 13:58	2.8	4.2	108	7.7	2.41
3/15/2017 13:59	3.3	2	87.6	5.7	0.62
3/15/2017 14:00	2.7	0.5	70.9	6.6	0.5
3/15/2017 14:01	3.9	12.5	33.2	8.4	0.71
3/15/2017 14:02	6.7	15	32.5	7.1	0.51
3/15/2017 14:03	10	11.4	31.6	3.7	0.47
3/15/2017 14:04	12	20.3	37.6	3.2	0.45
3/15/2017 14:05	15.3	24.6	36.4	2.5	0.42

3/15/2017 14:06	17.8	21.4	37.8	2.2	0.43
3/15/2017 14:07	19.2	16.3	25.1	2.2	0.41
3/15/2017 14:08	14.6	6.9	18.2	2.4	0.4
3/15/2017 14:09	10.5	7.8	18.7	2.2	0.4
3/15/2017 14:10	6.5	6.2	18	2.3	0.41
3/15/2017 14:11	7	11.8	23.3	2.3	0.44
3/15/2017 14:12	7.6	7	18.3	3.2	3.27
3/15/2017 14:13	7.3	4.4	18.3	4	4.85
3/15/2017 14:14	6.5	7.9	20.7	4.2	5.8
3/15/2017 14:15	5.4	7	19.7	2.3	1.68
3/15/2017 14:16	5.6	5.4	18.4	2.3	1.17
3/15/2017 14:17	6.6	12.7	24.5	1.8	0.62
3/15/2017 14:18	9	13.3	23.9	1.5	0.43
3/15/2017 14:19	10.8	11.7	24.2	1.5	0.42
3/15/2017 14:20	11.9	14.7	24.6	1.4	0.41
3/15/2017 14:21	11.1	10.6	22.2	1.4	0.42
3/15/2017 14:22	11.7	15.1	26.5	1.4	0.41
3/15/2017 14:23	12.6	18.4	29.5	1.3	0.41
3/15/2017 14:24	15.4	20.2	33.1	1.2	0.42
3/15/2017 14:25	17.5	21	31.8	1.2	0.4
3/15/2017 14:26	18.9	22.7	33.2	1.2	0.42
3/15/2017 14:27	19	19.7	30.2	1.1	0.41
3/15/2017 14:28	19.3	22.4	34	1.1	0.39
3/15/2017 14:29	20.8	26.4	36.1	1	0.41
3/15/2017 14:30	22.2	23.3	34.6	0.9	0.39
3/15/2017 14:31	20.3	12.4	22.5	0.9	0.39
3/15/2017 14:32	15.1	11.1	22.1	0.9	0.39
3/15/2017 14:33	10.5	10.7	22	0.9	0.39
3/15/2017 14:34	9.5	13.7	25.3	0.9	0.38
3/15/2017 14:35	14.5	29.2	38.5	0.7	0.39
3/15/2017 14:36	20.5	28.6	39	0.7	0.37
3/15/2017 14:37	25.8	28.5	39	0.6	0.38
3/15/2017 14:38	27.2	29	38.7	0.6	0.38
3/15/2017 14:39	25.1	18.7	29.2	0.6	0.36
3/15/2017 14:40	22.3	21.1	31.5	0.7	0.37
3/15/2017 14:41	18.5	16.6	25.7	0.6	0.37
3/15/2017 14:42	15.6	12.5	22.8	0.6	0.36
3/15/2017 14:43	13.3	14.5	25.8	0.7	0.36
3/15/2017 14:44	15.7	28	38.1	0.6	0.37
3/15/2017 14:45	21.1	29.4	39.3	0.6	0.36
3/15/2017 14:46	26.5	29.1	39.6	0.6	0.36
3/15/2017 14:47	28.1	29.8	39	0.5	0.37
3/15/2017 14:48	28.4	30	40.5	0.5	0.36
3/15/2017 14:49	29.1	31.7	41.1	0.5	0.36

3/15/2017 14:50	29.9	31.9	42.5	0.5	0.36
3/15/2017 14:51	30.2	28	37.5	0.5	0.35
3/15/2017 14:52	26.4	16.5	26.1	0.5	0.34
3/15/2017 14:53	19.1	8.8	19.2	0.5	0.34
3/15/2017 14:54	11.6	8.3	19.4	0.7	0.35
3/15/2017 14:55	11	22.2	32.2	0.7	0.34
3/15/2017 14:56	15	21.8	30.2	0.6	0.35
3/15/2017 14:57	18.8	20.2	29.5	0.6	0.35
3/15/2017 14:58	18.2	13.5	22.5	0.5	0.34
3/15/2017 14:59	13.5	7	16.3	0.5	0.34
3/15/2017 15:00	8.6	5	14.4	0.5	0.36
3/15/2017 15:01	4.9	4.1	14.1	0.6	0.35
3/15/2017 15:02	3.3	3.6	13.5	0.5	0.34
3/15/2017 15:03	2.7	3.2	13.2	0.7	0.36
3/15/2017 15:04	3.8	10.5	21.1	1	0.35
3/15/2017 15:05	8.3	19.3	29.7	1.1	0.34
3/15/2017 15:06	13.7	20.5	31.2	1	0.36
3/15/2017 15:07	18.7	21.1	31.1	0.7	0.35
3/15/2017 15:08	17	11.2	22.8	1	0.34
3/15/2017 15:09	15.2	15.2	26.9	0.8	0.35
3/15/2017 15:10	13.2	14.2	24.2	3.6	0.41
3/15/2017 15:11	11.5	5.6	16.9	0.8	0.36
3/15/2017 15:12	11.8	19.3	31.2	0.7	0.35
3/15/2017 15:13	13.8	19.8	29.9	0.6	0.36
3/15/2017 15:14	15.2	10.1	21.7	0.7	0.35
3/15/2017 15:15	15.7	18.4	29.5	0.7	0.37
3/15/2017 15:16	14.8	20.3	31	0.6	0.37
3/15/2017 15:17	17	19.2	29.6	0.7	0.39
3/15/2017 15:18	17.8	17.6	28.3	0.6	0.36
3/15/2017 15:19	17.4	20.7	31.6	0.7	0.38
3/15/2017 15:20	18.7	24.1	35.4	0.6	0.38
3/15/2017 15:21	22.1	27.4	38.7	0.6	0.34
3/15/2017 15:22	22.6	16.8	28.8	0.6	0.36
3/15/2017 15:23	19.8	12.6	24.3	0.6	0.37
3/15/2017 15:24	14	9.6	21.7	2.4	0.38
3/15/2017 15:25	9.2	5.3	18.2	20.4	0.62
3/15/2017 15:26	6.5	6.9	19.9	4.3	0.6
3/15/2017 15:27	5.4	7.7	19.7	0.8	0.35
3/15/2017 15:28	6	7.8	19.6	0.7	0.35
3/15/2017 15:29	6	6.3	17.3	0.8	0.36
3/15/2017 15:30	6.1	8	19	0.7	0.37
3/15/2017 15:31	6.4	10.7	20.6	0.7	0.35
3/15/2017 15:32	7.1	7.4	17.9	0.7	0.35
3/15/2017 15:33	7	7.6	18.1	0.7	0.36

3/15/2017 15:34	6.7	9.3	19.3	0.7	0.33
3/15/2017 15:35	8.1	12.3	23.2	0.7	0.33
3/15/2017 15:36	9.8	11.8	22	0.7	0.33
3/15/2017 15:37	10.1	9.2	19.7	0.6	0.33
3/15/2017 15:38	9.1	9.3	20.1	0.7	0.32
3/15/2017 15:39	8	8.8	19.2	0.7	0.33
3/15/2017 15:40	8	10.2	20.3	0.9	0.38
3/15/2017 15:41	8.1	9.5	20	0.7	0.32
3/15/2017 15:42	8.1	8.7	19.9	0.7	0.34
3/15/2017 15:43	8	8.1	18.7	0.7	0.32
3/15/2017 15:44	6.9	8.2	18.5	0.8	0.32
3/15/2017 15:45	7.3	9.6	20.1	1.2	0.35
3/15/2017 15:46	9.7	19.6	28.6	1.1	0.34
3/15/2017 15:47	13.7	23.6	51.3	821.3	4.73
3/15/2017 15:48	11.7	64	377.2	437.1	93.39
3/15/2017 15:49	6.7	35.8	400.1	164.1	37.68
3/15/2017 15:50	1.2	22.3	250.3	115	10.65
3/15/2017 15:51	0.3	41.7	321.1	340.3	23.66
3/15/2017 15:52	0.3	38.7	420.1	221.4	19.98
3/15/2017 15:53	0.1	22.6	309.1	138.4	15.13
3/15/2017 15:54	0.2	16.6	205.5	107.6	5.69
3/15/2017 15:55	0.3	14	177.8	35.7	5.33
3/15/2017 15:56	0.4	19.2	171.4	246	13.04
3/15/2017 15:57	0.3	38.4	333.7	197.1	30.46
3/15/2017 15:58	0.2	38.3	383.2	159.9	26
3/15/2017 15:59	0.2	39.1	410.7	162	23.68
3/15/2017 16:00	0.2	29.8	374	125.1	17.39
3/15/2017 16:01	0.2	24.6	303.7	131.8	13.04
3/15/2017 16:02	0.2	25.8	311.8	122.7	12.32
3/15/2017 16:03	0.2	22.1	257.4	94.9	7.35
3/15/2017 16:04	0.3	22.9	278.4	135.4	10.17
3/15/2017 16:05	0.3	16.2	236.8	67	8.6
3/15/2017 16:06	0.2	16.9	226.3	122.4	13.85
3/15/2017 16:07	0.2	19.9	256.8	169.7	14.23
3/15/2017 16:08	0.2	17.6	225.3	95.5	9.6
3/15/2017 16:09	0.2	15.2	205.3	95.3	8.12
3/15/2017 16:10	0.2	11.4	159.3	70.7	6.52
3/15/2017 16:11	0.3	8.9	174.3	19.3	5.89
3/15/2017 16:12	0.3	10.9	160.6	49.4	7.48
3/15/2017 16:13	0.3	19.4	249.7	48	15.55
3/15/2017 16:14	0.4	3.8	209.9	19.9	11.26
3/15/2017 16:15	0.5	0	124.3	8.8	5.63
3/15/2017 16:16	0.9	1.3	69.7	6.7	2.62
3/15/2017 16:17	2.3	8.3	46.5	5.6	1.33

3/15/2017 16:18	4	9.4	52.5	7.2	1.52
3/15/2017 16:19	5.8	12.7	66.8	7.8	2.16
3/15/2017 16:20	8.1	17.6	45.6	4.2	1.22
3/15/2017 16:21	10.8	19.4	46.1	4.1	1.12
3/15/2017 16:22	13.4	20.5	48	3.3	0.77
3/15/2017 16:23	15.1	23.1	47.9	3	0.73
3/15/2017 16:24	15.8	18.7	44.2	3	0.71
3/15/2017 16:25	15.8	19.7	43.2	2.8	0.57
3/15/2017 16:26	15.2	18.1	46.7	11.5	0.77
3/15/2017 16:27	13.1	10.7	39.8	13.9	1.3
3/15/2017 16:28	9.8	4.6	32	2.9	1.04
3/15/2017 16:29	5.8	6.4	35.6	2.7	0.78
3/15/2017 16:30	4.2	7.5	41.1	6.4	1.16
3/15/2017 16:31	4.5	8.8	37.3	2.9	0.97
3/15/2017 16:32	6.1	11.6	35.5	2.3	0.47
3/15/2017 16:33	8.1	14	36.4	2.4	0.52
3/15/2017 16:34	9.6	15.2	41.2	2.9	0.8
3/15/2017 16:35	11.8	18.7	45.4	2.9	0.57
3/15/2017 16:36	12.3	14.6	42.2	2.8	0.83
3/15/2017 16:37	11.5	6.2	34	4	0.59
3/15/2017 16:38	7.5	4.1	46.1	22.2	4.46
3/15/2017 16:39	3.8	5.5	70.2	16.7	6.61
3/15/2017 16:40	1.5	3.6	65.9	8.6	2.02
3/15/2017 16:41	1	3.4	60.2	28	6.21
3/15/2017 16:42	0.8	3.6	69.8	15.6	5.3
3/15/2017 16:43	0.8	5.9	83.7	19.7	7.48
3/15/2017 16:44	0.7	9.2	118.4	24.6	8.38
3/15/2017 16:45	0.9	6.6	107.5	16.4	8.39
3/15/2017 16:46	1	5.8	92.9	13.8	4.8
3/15/2017 16:47	1.1	4.4	87.7	7.1	5.66
3/15/2017 16:48	1.1	3.8	71.2	7.8	2.38
3/15/2017 16:49	1.5	4.9	66.5	3.7	3.23
3/15/2017 16:50	1.9	3.4	53.8	3	1.11
3/15/2017 16:51	2.2	3.5	52	3.1	1.03
3/15/2017 16:52	2.2	3	42.1	2.1	0.57
3/15/2017 16:53	2.2	3.6	37.9	1.8	0.55
3/15/2017 16:54	2.1	1.2	33.4	1.9	0.41
3/15/2017 16:55	2	2.8	31.4	1.5	0.33
3/15/2017 16:56	1.7	1.2	29.1	1.6	0.34
3/15/2017 16:57	1.4	1	28.1	6.7	2.63
3/15/2017 16:58	1.2	2.1	27.5	4.8	4.36
3/15/2017 16:59	2.5	9.8	27.1	3.5	4.58

3/16/2017 10:30	1.1	0.9	10.2	4.3	1.71
3/16/2017 10:31	0.9	0.5	10.6	1.3	0.71
3/16/2017 10:32	0.8	1	10.5	1.4	0.7
3/16/2017 10:33	2	7.4	19	1	0.8
3/16/2017 10:34	6	15.1	20.9	0.9	0.83
3/16/2017 10:35	8	5	12.9	0.9	0.9
3/16/2017 10:36	7.2	2	10.3	0.9	0.91
3/16/2017 10:37	3.8	3.7	12.6	1.3	0.9
3/16/2017 10:38	2.4	3.2	12.9	1	0.79
3/16/2017 10:39	3.6	7.3	15.2	1.1	0.6
3/16/2017 10:40	5.2	8.9	16.4	0.6	0.58
3/16/2017 10:41	7.4	10.5	17.6	0.9	0.64
3/16/2017 10:42	8.4	7.2	15	1	0.7
3/16/2017 10:43	9	12.5	18.3	0.8	0.6
3/16/2017 10:44	8.5	8.3	16.5	0.7	0.57
3/16/2017 10:45	8.3	7.7	14.5	0.7	0.57
3/16/2017 10:46	6	2.2	9.7	0.7	0.61
3/16/2017 10:47	3.6	0.8	9.7	0.7	0.6
3/16/2017 10:48	1.7	3.5	10.4	0.7	0.6
3/16/2017 10:49	1.8	4.8	11.6	0.7	0.56
3/16/2017 10:50	3.2	10.5	16.3	0.7	0.56
3/16/2017 10:51	4.2	6.2	12.1	0.7	0.55
3/16/2017 10:52	4	2	10.6	0.6	0.6
3/16/2017 10:53	4.8	11.7	16.5	0.6	0.52
3/16/2017 10:54	4.3	4.2	11.9	0.6	0.51
3/16/2017 10:55	4.6	3.8	12	0.6	0.52
3/16/2017 10:56	3.6	6.7	13.7	0.8	0.51
3/16/2017 10:57	3.2	1.6	9.9	1	0.61
3/16/2017 10:58	2.6	1	9.1	1.2	0.53
3/16/2017 10:59	1.4	1.3	2.8	1.5	0.48
3/16/2017 11:00	2.2	9.4	17.1	1.7	0.5
3/16/2017 11:01	5.1	11	17.4	1.8	0.48
3/16/2017 11:02	7.5	9.2	14.6	1.9	0.49
3/16/2017 11:03	6.6	2.4	9.8	2	0.5
3/16/2017 11:04	3.7	1.4	9.5	2	0.54
3/16/2017 11:05	1.3	0.9	10.5	23	0.57
3/16/2017 11:06	0.7	9.8	49.6	405.7	8.44
3/16/2017 11:07	0.5	31.8	195.8	58.9	13.9
3/16/2017 11:08	0.3	15.6	139.6	8.6	3.17
3/16/2017 11:09	0.4	3.2	52.3	5	1.36
3/16/2017 11:10	1.5	5.8	27.2	3.9	1.2
3/16/2017 11:11	3.8	11	29.7	70.8	1.36
3/16/2017 11:12	4	14.6	79.1	208.2	7.43
3/16/2017 11:13	3.1	24.8	149.8	129.4	9.18

3/16/2017 11:14	1	56.6	304.1	304.3	12.13
3/16/2017 11:15	0.5	74.7	587.2	173.5	18.44
3/16/2017 11:16	0.3	72.3	636	175.2	24.76
3/16/2017 11:17	0.1	45.2	608.3	78.9	21.61
3/16/2017 11:18	0.1	26.7	473.2	55.8	7.67
3/16/2017 11:19	0.2	16.8	384.9	71.9	6.29
3/16/2017 11:20	0.2	16.3	331.4	69.4	6.07
3/16/2017 11:21	0.2	15.8	315.4	80	8.92
3/16/2017 11:22	0.2	22.7	332.6	96.5	7.87
3/16/2017 11:23	0.3	34.1	401.2	84.8	8.23
3/16/2017 11:24	0.3	32.9	430.3	130.5	10.39
3/16/2017 11:25	0.3	34	416.9	86.4	9.68
3/16/2017 11:26	0.2	19.5	329.8	68.9	6.39
3/16/2017 11:27	0.2	24	332.1	84.4	8.38
3/16/2017 11:28	0.2	18	287	45.3	6.53
3/16/2017 11:29	0.2	22.1	313.5	44.6	6.6
3/16/2017 11:30	0.2	15.2	282	37.1	5.98
3/16/2017 11:31	0.2	17.1	278.3	69	5.81
3/16/2017 11:32	0.2	19.1	298.7	120.4	8.89
3/16/2017 11:33	0.2	19.9	301.3	115.8	9.97
3/16/2017 11:34	0.2	31.3	346.4	196.8	8.31
3/16/2017 11:35	0.2	48.1	468.5	175.8	15.28
3/16/2017 11:36	0.1	47.6	478.5	139.6	12.93
3/16/2017 11:37	0.1	23.6	381.3	75.5	9.21
3/16/2017 11:38	0.2	11.4	285	60.8	6.35
3/16/2017 11:39	0.2	17.2	302.9	60.5	6.61
3/16/2017 11:40	0.4	20.2	322.4	54.4	9.09
3/16/2017 11:41	0.6	21.4	320.5	45.8	7.13
3/16/2017 11:42	0.5	11.5	268.8	46.8	5.92
3/16/2017 11:43	0.3	7.5	238.1	37.1	6.8
3/16/2017 11:44	0.1	5.7	215.9	34	5.57
3/16/2017 11:45	0.1	9.5	235	38.2	6.28
3/16/2017 11:46	0.1	5.5	209.8	44	6.21
3/16/2017 11:47	0.1	5.3	200.1	37.7	6.19
3/16/2017 11:48	0.1	6	205.3	36.7	5.18
3/16/2017 11:49	0.1	5	191.4	31.6	6.06
3/16/2017 11:50	0.1	5.2	199.3	25.8	5.15
3/16/2017 11:51	0.1	0.9	170.3	16.8	4.28
3/16/2017 11:52	0.2	-0.2	140	26.5	4.39
3/16/2017 11:53	0.2	3.3	149.9	22.7	4.37
3/16/2017 11:54	0.2	1.6	146.4	19.6	4.06
3/16/2017 11:55	0.2	0.2	142.2	15.7	4.63
3/16/2017 11:56	0.2	0.8	132.4	15.2	3.92
3/16/2017 11:57	0.2	1.6	136.3	14.5	4.12

3/16/2017 11:58	0.5	10.1		172.3	16	4.08
3/16/2017 11:59	0.9	2.9		157.2	12.8	3.87
3/16/2017 12:00	1.3	6.4		149.3	13.4	3.81
3/16/2017 12:01	1.4	10		164.1	14.2	3.67
3/16/2017 12:02	1.5	6.1		163.3	14.7	4.07
3/16/2017 12:03	1.5	4.1		133	14	3.9
3/16/2017 12:04	1.3	5.2		141.3	18.5	4.36
3/16/2017 12:05	1	-1.7		96.4	3.7	1.94
3/16/2017 12:06	0.8	-1.9		49.5	2.8	0.52
3/16/2017 12:07	0.8	0.5		37.2	2.4	0.36
3/16/2017 12:08	1	1.7	7.9	30.1	2.2	0.37
3/16/2017 12:09	1.1	1.5	4	28.7	2.1	0.36
3/16/2017 12:10	1.2	2.4	-1.4	27.3	2	0.38
3/16/2017 12:11	1.3	2.3	-5	27.1	1.9	0.38
3/16/2017 12:12	1.6	3.8	-2.2	23.8	1.9	0.37
3/16/2017 12:13	2	4.1	1	24.4	1.9	0.41
3/16/2017 12:14	2.4	5.6	4.9	23.1	1.8	0.38
3/16/2017 12:15	2.7	4.5	4.8	22.5	1.8	0.38
3/16/2017 12:16	2.6	4.5	0.6	23.2	1.7	0.37
3/16/2017 12:17	2.6	5.6	0.1	23.1	1.7	0.37
3/16/2017 12:18	2.5	4.1	1.8	22.6	1.8	0.43
3/16/2017 12:19	2.4	4.2	4.6	23.6	1.9	0.51
3/16/2017 12:20	2.4	4.6	1.3	23.4	1.8	0.56
3/16/2017 12:21	2.6	6.2	-4.4	23	1.7	0.38
3/16/2017 12:22	3.1	7.4	4.3	22.6	1.7	0.38
3/16/2017 12:23	3.4	6.2	6.7	22.5	1.8	0.36
3/16/2017 12:24	3.5	5.2	6.7	21.7	1.8	0.62
3/16/2017 12:25	3.3	4.4	5.3	22.7	1.8	0.57
3/16/2017 12:26	3.2	5.5	4.8	22.2	1.8	0.44
3/16/2017 12:27	3	4.3	7	21.1	2.5	2.4
3/16/2017 12:28	2.8	3.1	3.9	22.4	1.9	1.52
3/16/2017 12:29	2.5	2.9	2.7	21.8	2	1.82
3/16/2017 12:30	2.4	3.8	0.4	21.8	2	2.15
3/16/2017 12:31	2.6	4.8	1.9	23.7	2	1.32
3/16/2017 12:32	2.5	3.5	1.7	21.5	2.2	0.81
3/16/2017 12:33	2.4	2.7	3.7	19.6	2.1	0.45
3/16/2017 12:34	2.2	3.1	5	18.8	2.1	0.36
3/16/2017 12:35	2.1	3.3	4.4	19	2.2	0.44
3/16/2017 12:36	2	2.1	3.3	20.6	3.3	4.69
3/16/2017 12:37	1.9	2.5	0.9	19.8	2.6	2.29
3/16/2017 12:38	1.9	3.9	3.5	21.7	2.7	2.07
3/16/2017 12:39	4.6	22.8	3.6	34.5	2.1	0.67
3/16/2017 12:40	9.9	30.6	20.9	33.8	2	0.69
3/16/2017 12:41	15.6	31.8	29.1	35.4	1.9	0.38

3/16/2017 12:42	19	31.9	28.3	35.2	2	0.33
3/16/2017 12:43	19.7	31.2	29	34.4	2.1	0.32
3/16/2017 12:44	19.9	30.5	29.4	33.2	2.1	0.33
3/16/2017 12:45	19.9	30.7	29.1	33.9	2.1	0.3
3/16/2017 12:46	20.2	31.6	30.3	34.5	2	0.34
3/16/2017 12:47	20.6	31.7	29.9	34.1	2.6	0.33
3/16/2017 12:48	21.1	32.9	26.9	34.9	2.6	0.33
3/16/2017 12:49	21.6	33.3	30.3	36.3	2.1	0.32
3/16/2017 12:50	22	33	31.5	35.6	1.9	0.31
3/16/2017 12:51	22.1	32	27.7	35.1	1.9	0.29
3/16/2017 12:52	22	32.1	28.5	34.6	1.9	0.3
3/16/2017 12:53	21.7	30.2	28.9	34.1	1.8	0.3
3/16/2017 12:54	21.5	31.2	29.5	34	1.9	0.47
3/16/2017 12:55	21.6	32.2	26	35.4	1.9	0.36
3/16/2017 12:56	22.1	32.6	32	36.2	1.9	0.31
3/16/2017 12:57	22.7	33.4	29.3	35.6	1.7	0.28
3/16/2017 12:58	23.1	32.4	27.6	35.9	1.7	0.28
3/16/2017 12:59	23.2	31.3	29.4	34.1	4.8	0.27
3/16/2017 13:00	18.9	42	29.4	257.3	279.9	6.95
3/16/2017 13:01	13.8	12.2	55.5	237.4	125.5	15.23
3/16/2017 13:02	9.4	18.8	77	137.1	78.7	8.63
3/16/2017 13:03	9.8	18.9	32.8	101.9	68	6.02
3/16/2017 13:04	10.8	17.6	28.3	105	87.8	7.36
3/16/2017 13:05	10.7	17.8	27.7	135	112	7.27
3/16/2017 13:06	8.9	13.2	29.1	192.1	153.3	14.51
3/16/2017 13:07	7.5	14.3	34.4	181.6	150	12.78
3/16/2017 13:08	6.4	13.2	35.2	170.7	129.9	12.66
3/16/2017 13:09	6.2	13.7	35.3	186.3	150.6	12.63
3/16/2017 13:10	6.4	15.9	33.9	158.4	101	11.07
3/16/2017 13:11	7.8	17.3	33.5	122.6	73.9	8.2
3/16/2017 13:12	9.2	16.3	29.7	170.6	112.9	8.26
3/16/2017 13:13	9.8	17.6	27.8	116.2	69.8	9.06
3/16/2017 13:14	10.5	20.3	25.7	82	43.2	5.03
3/16/2017 13:15	10.8	17.7	24.4	113.1	74.3	5.72
3/16/2017 13:16	11.1	17.6	24.8	109.5	72.6	7.59
3/16/2017 13:17	9.8	14.5	27.7	154.4	120	8.49
3/16/2017 13:18	7.9	12.4	29.9	187.7	178.6	14.09
3/16/2017 13:19	6.4	11.8	34.6	124.6	128.3	12.75
3/16/2017 13:20	5.5	14.6	31.2	170.6	189.3	9.87
3/16/2017 13:21	4.9	8	29.6	192.3	189.1	17.62
3/16/2017 13:22	4.8	11.4	34.6	148.6	136.4	11.88
3/16/2017 13:23	5.3	13.8	31.4	104.6	77.5	7.79
3/16/2017 13:24	8	25	27.1	81.8	48.4	5.16
3/16/2017 13:25	9.2	32	27.7	224.4	151.7	9.19

3/16/2017 13:26	7.8	8.9	49.7	187.8	169.3	14.76
3/16/2017 13:27	6.4	12.1	39.1	121.7	84.6	9.83
3/16/2017 13:28	7.9	21.3	32.7	58	29.7	3.46
3/16/2017 13:29	10.3	17.8	23.3	97.5	64.8	5.44
3/16/2017 13:30	11.4	16.1	22.7	103.7	75.8	6.67
3/16/2017 13:31	10.9	17.3	23.7	82.7	83	5.97
3/16/2017 13:32	12.1	21.1	18.2	65.7	79.8	3.45
3/16/2017 13:33	12.3	15.6	24.9	89.8	72.2	6.49
3/16/2017 13:34	12.9	18.7	23.3	69.4	44.3	4.06
3/16/2017 13:35	11.6	16.8	21.7	83.4	63	5.91
3/16/2017 13:36	12.2	17.9	24.8	87.6	92.7	5.07
3/16/2017 13:37	11	15.8	25	83	75.1	7.37
3/16/2017 13:38	10.6	15	26.3	75.7	88.7	5.68
3/16/2017 13:39	10.3	18.4	22.4	83.7	69.3	6.29
3/16/2017 13:40	11.4	19.4	22.5	88.4	96.7	5.64
3/16/2017 13:41	11	16.2	24	121.1	132.1	7.91
3/16/2017 13:42	10.1	13.4	36.7	110.2	127.2	8.79
3/16/2017 13:43	9.3	16.3	21.4	83.7	83.3	6.75
3/16/2017 13:44	11.3	22.4	22.8	55.1	34.7	4.63
3/16/2017 13:45	13.3	19.4	21.4	71.3	66.7	3.68
3/16/2017 13:46	15.1	24.2	21.6	41.8	17.9	3.41
3/16/2017 13:47	16.4	26.4	21.7	68.5	46.5	1.52
3/16/2017 13:48	14.9	13.4	27.1	162.5	119.1	9.81
3/16/2017 13:49	13.2	15.7	31.8	98.4	98.6	7.59
3/16/2017 13:50	9.3	15.7	29.3	256.2	227.5	10.5
3/16/2017 13:51	7.5	6.5	34.4	310.8	243.4	29.04
3/16/2017 13:52	6.9	17.7	42.8	310	189.2	11.69
3/16/2017 13:53	5.3	8.1	45.6	566.7	342.9	31.87
3/16/2017 13:54	4.1	12.6	65.2	679.1	706.4	44.1
3/16/2017 13:55	1.8	7.8	85.7	1081.9	533	66.58
3/16/2017 13:56	1.3	16.1	111.8	1094	251.4	56.17
3/16/2017 13:57	1.7	-2.4	111.1	456.4	130.9	49.78
3/16/2017 13:58	2.4	8.2	57.4	312.4	130.7	16.33
3/16/2017 13:59	3.6	9.6	35.5	133.3	46.8	13.89
3/16/2017 14:00	5.6	24.8	24.7	148.3	38.4	6.19
3/16/2017 14:01	6.8	7.9	40.9	168.4	73.1	8.42
3/16/2017 14:02	7.1	11.4	33	189.7	57.8	14.27
3/16/2017 14:03	6.3	9.9	27	142.8	69.9	12.75
3/16/2017 14:04	6.3	11.6	22.5	106.5	62	7.71
3/16/2017 14:05	7.1	14.8	16.9	82.2	40.4	7.15
3/16/2017 14:06	8.4	15.7	17.8	86.5	45.6	5.36
3/16/2017 14:07	9.4	15.6	16.1	85.8	34.9	6.14
3/16/2017 14:08	10.5	17.4	21.3	64.9	32.4	4.8
3/16/2017 14:09	11.1	17.4	17.8	54.7	29	3.32

3/16/2017 14:10	11.2	14.7	17.4	77.8	35.9	5.14
3/16/2017 14:11	11.4	19	18.6	60.5	21.6	3.91
3/16/2017 14:12	12.3	20.2	18.6	63	27.9	2.3
3/16/2017 14:13	12.3	15.2	20.6	86	56.5	5.66
3/16/2017 14:14	12.2	17.2	20.7	79.2	44.6	4.99
3/16/2017 14:15	11.5	18.6	22.9	70.2	39.8	4.22
3/16/2017 14:16	12.2	17.5	22.3	76.6	38.2	4.46
3/16/2017 14:17	11.8	15.7	20.8	77.1	55.6	4.88
3/16/2017 14:18	11.9	19.9	19	59.4	26	3.95
3/16/2017 14:19	12.8	19.7	23.7	57.1	34.6	3.48
3/16/2017 14:20	14.1	20	23.2	56.9	35.8	2.32
3/16/2017 14:21	14.9	24.2	23.3	47.4	13.1	2.6
3/16/2017 14:22	16.1	23.9	22.3	50.3	20	1.81
3/16/2017 14:23	16.6	22.4	25.5	59.5	20.9	2.88
3/16/2017 14:24	17.2	23.6	24.2	57.4	17.6	2.57
3/16/2017 14:25	16.7	22.2	24.5	57.7	25.1	3.08
3/16/2017 14:26	17	23.8	26	53.2	21.6	2.36
3/16/2017 14:27	16.5	20.5	24.8	65.8	30.3	3.46
3/16/2017 14:28	17.4	27.2	23.1	44.6	8.9	1.87
3/16/2017 14:29	18.1	25.9	24.1	52.1	13.3	1.33
3/16/2017 14:30	19.1	25.2	22.7	50.9	14.1	1.82
3/16/2017 14:31	18.6	22.6	24.3	53.2	18.2	2.01
3/16/2017 14:32	17.8	23.2	22.3	48.1	19.1	2
3/16/2017 14:33	17.9	25.6	23.1	43.9	19.2	1.82
3/16/2017 14:34	18.7	24.7	24.2	48.1	21.6	1.67
3/16/2017 14:35	19.1	25.7	26.8	54.2	26.3	2.42
3/16/2017 14:36	18.6	22.2	29.2	63.6	31.1	2.37
3/16/2017 14:37	17.4	21.4	30.9	65.6	30.7	3.25
3/16/2017 14:38	17.7	26.5	24.7	49.1	10.2	2.03
3/16/2017 14:39	18.8	26.5	23.6	52.8	9.9	1.52
3/16/2017 14:40	20.3	26.4	23.2	50.8	11.3	1.71
3/16/2017 14:41	21.2	28.8	23.2	44.7	5.8	1.17
3/16/2017 14:42	21.8	29.3	25	41.2	5.1	1.17
3/16/2017 14:43	23	30	25.7	41.2	4.4	0.88
3/16/2017 14:44	23.2	29.3	23.4	45.4	5.8	0.97
3/16/2017 14:45	23.2	28.5	23.1	46	6.1	1.11
3/16/2017 14:46	23.2	29.2	26.1	43.4	5.6	0.98
3/16/2017 14:47	23.2	28.9	25.2	45.4	5.1	1.14
3/16/2017 14:48	23.9	31.1	25.3	39.8	4.1	0.77
3/16/2017 14:49	24.1	30.6	27.4	45.5	3.8	0.81
3/16/2017 14:50	24.5	30	26.1	43.6	4.2	0.9
3/16/2017 14:51	24.5	30	23.7	44.2	4.1	0.84
3/16/2017 14:52	24.2	29.6	22	47.4	5	0.91
3/16/2017 14:53	23.7	28.3	23.4	51	8.4	1.29

3/16/2017 14:54	23.4	28.8	20	47.5	8.2	1.15
3/16/2017 14:55	24	32.1	25.9	39.4	4.4	0.73
3/16/2017 14:56	25	30.6	27.7	42.4	5.2	0.65
3/16/2017 14:57	25.6	31.1	28.7	42.7	5.5	0.69
3/16/2017 14:58	25.5	31	24.6	44.2	5.4	0.74
3/16/2017 14:59	25.3	30.5	30.2	43.5	6.1	0.88
3/16/2017 15:00	25.4	31.3	28.1	46.2	4.1	0.69
3/16/2017 15:01	25.1	30.5	27.5	50.7	4.3	1.04
3/16/2017 15:02	25.1	30.8	29.7	47	4.3	0.95
3/16/2017 15:03	24.9	31	27.1	44.4	4.1	0.71
3/16/2017 15:04	25.2	31.4	32.7	43.8	4.6	0.78
3/16/2017 15:05	25.4	30.4	28.1	44	4	0.68
3/16/2017 15:06	25.7	32	25	44.3	3.6	0.63
3/16/2017 15:07	26.1	32.1	26.3	45.1	3.6	0.67
3/16/2017 15:08	26.8	33.1	25.2	43.9	3.4	0.58
3/16/2017 15:09	26.9	32.2	28.8	47.4	3.5	0.71
3/16/2017 15:10	27.3	34.3	31.4	44.1	3.4	0.62
3/16/2017 15:11	27.5	33.5	34.1	43.9	3.2	0.54
3/16/2017 15:12	28	34.9	36.1	43	3.1	0.54
3/16/2017 15:13	28.2	34.1	43.5	47	3.2	0.61
3/16/2017 15:14	28.3	32.2	53	46.2	3.3	0.73
3/16/2017 15:15	28.2	34.3	48.1	46.4	3.4	0.72
3/16/2017 15:16	28.2	33	46.9	45.3	3.1	0.63
3/16/2017 15:17	28.4	34.5	37.5	45	3.2	0.7
3/16/2017 15:18	28.2	33	44.4	47.2	3.6	0.71
3/16/2017 15:19	28	33	32.5	52.4	4.4	0.9
3/16/2017 15:20		32.7	38.3	48.9	4.2	0.92
3/16/2017 15:21		31.5	33.3	44.9	5.2	0.98
3/16/2017 15:22		30.5	32.4	39.1	3.6	1.03
3/16/2017 15:23		31.8	28.1	35.1	3.2	4.77
3/16/2017 15:24		29.8	27.6	34	4.4	11.47
3/16/2017 15:25		30.4	25.9	33.3	3.5	3.14
3/16/2017 15:26		32.6	23.8	34.3	3.2	2.98
3/16/2017 15:27		33	24.8	34.3	3.3	5.4
3/16/2017 15:28		32.7	28.8	34.5	4.1	11.57
3/16/2017 15:29		34.5	31.5	34.7	2.7	3.7
3/16/2017 15:30		34.1	19.7	34.8	2.4	0.49
3/16/2017 15:31		35.8	23.6	36.9	2.7	0.76
3/16/2017 15:32		35.9	31.3	37.2	2.5	0.47
3/16/2017 15:33		36.3	28.8	37.7	2.5	0.24
3/16/2017 15:34		35.6	26.1	37.7	2.5	0.22
3/16/2017 15:35		37.1	32.9	38.3	2.3	0.25
3/16/2017 15:36		37	34	39	2.5	0.23
3/16/2017 15:37		35.9	30.5	37.5	2.1	0.17

3/16/2017 15:38	36	32.5	37.3	2	0.19
3/16/2017 15:39	34.5	28.4	37.1	2	0.17
3/16/2017 15:40	35.3	27.3	37.8	2.1	0.17
3/16/2017 15:41	35.7	28.2	38.3	2.2	0.24
3/16/2017 15:42	36.2	28.1	38.7	3.6	3.5
3/16/2017 15:43	38.1	29.2	40.7	4.2	7.94
3/16/2017 15:44	36.5	35.3	40.7	6.2	22.43
3/16/2017 15:45	36.6	34.3	39.6	4.9	9.68
3/16/2017 15:46	37.1	38.3	40.4	4	6.5
3/16/2017 15:47	38.6	39.2	41.4	3.6	5.17
3/16/2017 15:48	37.8	44.2	41.1	4	5.8
3/16/2017 15:49	38.2	35.5	41.2	3.1	3.57
3/16/2017 15:50	38.2	36.2	41.8	3.5	4.52
3/16/2017 15:51	37.5	33.6	41.7	3.7	4.68
3/16/2017 15:52	38.5	35.4	42.2	4	5.56
3/16/2017 15:53	39.6	28.2	43.3	3.7	4.85
3/16/2017 15:54	40.2	36.8	43	3.8	5.63
3/16/2017 15:55	40.6	39.4	43.7	2.9	3.48
3/16/2017 15:56	40	38.9	43.4	2.8	3.24
3/16/2017 15:57	38.9	40.6	43.1	3.1	3.79
3/16/2017 15:58	38.6	32.3	42.7	3.6	5.13
3/16/2017 15:59	39.2	31.3	42.8	3.2	4.37
3/16/2017 16:00	38.7	33.8	42.7	3.2	4.73
3/16/2017 16:01	37.9	34.6	42.2	3.7	5.95
3/16/2017 16:02	38.9	31.6	42.8	3.2	4.7
3/16/2017 16:03	39.1	33.5	43.5	3.1	4.17
3/16/2017 16:04	39.7	33.8	43.8	2.8	3.32
3/16/2017 16:05	40.5	37.1	43.8	3	4.01
3/16/2017 16:06	40.7	37.6	43.8	2.9	4.08
3/16/2017 16:07	41.2	37.7	44.2	3.1	3.63
3/16/2017 16:08	42	39.3	44.9	2.8	3.52
3/16/2017 16:09	40.8	41.2	44.7	3.3	4.88
3/16/2017 16:10	39.6	36.7	43.7	5.2	9.16
3/16/2017 16:11	37.8	35.5	42.2	6.4	11.75
3/16/2017 16:12	39.5	33.7	43.3	5.7	9.97
3/16/2017 16:13	40.9	34.7	43.9	5.2	8.3
3/16/2017 16:14	40.3	37.8	44.2	4.7	7.44
3/16/2017 16:15	39.9	39.2	43.6	4.9	8.15
3/16/2017 16:16	40.8	38.5	44.1	5.2	8.76
3/16/2017 16:17	38.7	35.6	43.1	5.9	8.3
3/16/2017 16:18	40.5	37.9	43.7	5.2	9.31
3/16/2017 16:19	42	39.4	45	4.2	7.62
3/16/2017 16:20	40.8	40	45.2	3.9	6.57
3/16/2017 16:21	40.6	40.5	44.4	5.1	8.88

3/16/2017 16:22		39.7	35.2	44.3	6.7	12.63
3/16/2017 16:23		40.7	34.4	44.4	5.8	11.32
3/16/2017 16:24		41	36.4	44.7	5.7	11.56
3/16/2017 16:25		15.5	39.7	-45.5	3.8	6.68
3/16/2017 16:26		-0.6	19.7	-61.7	4.2	7.19
3/16/2017 16:27		-0.2	0	-50.5	4.6	8.07
3/16/2017 16:28		-0.5	-2	-43.5	5.5	10.33
3/16/2017 16:29		-0.3	-0.4	-36.5	6	12.31
3/16/2017 16:30		-0.1	1.2	-30.1	4.9	9.25

3/17/2017 15:10	35	40.2	42.9			0.17
3/17/2017 15:11	35.4	38.6	42.9			0.68
3/17/2017 15:12	35.4	39.4	40.5			0.51
3/17/2017 15:13	35.3	39.9	42.3			0.28
3/17/2017 15:14	35.6	39.6	43.1			0.2
3/17/2017 15:15	35.9	37	41.3			0.56
3/17/2017 15:16	33.5	24	31			2.99
3/17/2017 15:17	28.6	26.8	41.1			10.73
3/17/2017 15:18	18.7	5.7	36.3			30.53
3/17/2017 15:19	11.6		31.6			25.87
3/17/2017 15:20	7.3	11.7	20.3		294.9	12.98
3/17/2017 15:21	10.6	21.4	22.7		290.1	4.69
3/17/2017 15:22	13.9	18.8	24.1		91.6	7.46
3/17/2017 15:23	16.1	19.1	22.5		92.6	3.53
3/17/2017 15:24	15.4	12.8	16.8		160.1	4.48
3/17/2017 15:25	13.2	12.4	10.2	126.4	177.2	8.94
3/17/2017 15:26	11	14.1	13.4	105.6	153.4	6.39
3/17/2017 15:27	11.2	20.6	19.3	79.8	94.3	3.03
3/17/2017 15:28	13.2	18.6	18.3	101.4	144.9	3.91
3/17/2017 15:29	16.9	28.5	28.8	136	76.1	4.64
3/17/2017 15:30	18.2	16.3	22.9	134.2	102.3	6.53
3/17/2017 15:31	21.3	28.4	26.8	89.7	38.1	2.14
3/17/2017 15:32	20.5	20.4	19.5	90.6	99.3	5.94
3/17/2017 15:33	22.7	29.9	28.4	82.3	14.4	1.65
3/17/2017 15:34	23.4	30.4	30	81.7	13.5	2.94
3/17/2017 15:35	27.5	31.7	30.6	92.1	15.1	3.44
3/17/2017 15:36	29	33.1	33.6	81.3	14.6	3.24
3/17/2017 15:37	30.3	33	34.6	81.1	11.9	2.41
3/17/2017 15:38	30.9	34.6	31.4	71.9	9.9	1.73
3/17/2017 15:39	31.7	34.9	32.4	72.9	9.1	1.41
3/17/2017 15:40	33.1	37.9	34.4	68.2	4.2	0.58
3/17/2017 15:41	34.1	36.3	33.2	68.5	3.3	0.47
3/17/2017 15:42	35	37.2	32.2	62.4	3.7	0.34

3/17/2017 15:43	35.3	38.6	36.1	62.2	2.6	0.16
3/17/2017 15:44	36	39.2	35.7	64	2.1	0.2
3/17/2017 15:45	36.9	37.8	33.2	62.6	2.2	0.59
3/17/2017 15:46	37.1	38.6	34.9	63.2	3.5	5.16
3/17/2017 15:47	34.2	25.6	28.4	68.8	98.5	19.87
3/17/2017 15:48	25.4	29.6	48.8	603.4	489.7	44.86
3/17/2017 15:49	15.3	13.1	28	280.9	164.2	34.64
3/17/2017 15:50	9.6	20.1	28.6	195.8	204.6	32.17
3/17/2017 15:51	10.7	18.4	24.9	149.7	66.4	21.66
3/17/2017 15:52	13.2	34.5	37.1	363.6	313.1	20.15
3/17/2017 15:53	11.4	13.6	73.9	970.3	254.5	64.83
3/17/2017 15:54	8.6	10.2	50.4	790.7	285.8	52
3/17/2017 15:55	4.7	3.9	111.2	1134.7	358.1	94.23
3/17/2017 15:56	3	-1.8	45	493.6	170.7	54.31
3/17/2017 15:57	3.1	9.8	18.5	215.8	77.1	16.47
3/17/2017 15:58	6.6	19.3	14.4	100.3	21.8	5.24
3/17/2017 15:59	9.4	12.3	15.6	149.4	66.9	7.94
3/17/2017 16:00	11.6	17.6	15.8	112.7	27.9	10.37
3/17/2017 16:01	13.2	21.4	15.8	76.7	16.7	3.12
3/17/2017 16:02	15.1	21.8	14.8	63.5	14.4	1.19
3/17/2017 16:03	18.1	25.7	18.7	62.8	12	4.22
3/17/2017 16:04	19.8	25.7	17.9	58.7	13.6	2.67
3/17/2017 16:05	21.6	28.6	22.1	56.2	9.6	0.61
3/17/2017 16:06	23.3	30.7	23.5	55.7	4.5	0.94
3/17/2017 16:07	25.2	31.4	24.7	56.2	4.1	0.28
3/17/2017 16:08	26.1	30	21.5	53.8	8.7	0.09
3/17/2017 16:09	25.9	29.3	22.5	59.9	20.9	1.19
3/17/2017 16:10	20.2	16.5	70.3	692.7	668.8	24.16
3/17/2017 16:11	12.5	6.7	44.2	493.8	463.7	31.03
3/17/2017 16:12	5	5.7	26.8	373.9	429.3	23.87
3/17/2017 16:13	2.9	12.1	24.1	286.6	298.5	22.03
3/17/2017 16:14	5	22.2	39.1	363.7	179.4	38.51
3/17/2017 16:15	8.4	11.1	25.5	267.7	113.1	19.16
3/17/2017 16:16	10.4	17.9	28	272.3	116.4	24.61
3/17/2017 16:17	11.8	20.7	23.1	200.7	55.6	19.44
3/17/2017 16:18	13.2	21.7	24.7	181.2	37	14.1
3/17/2017 16:19	15.9	20.8	20.9	140.8	40.9	10.74
3/17/2017 16:20	17.3	23.8	26.1	165.7	75.9	13.54
3/17/2017 16:21	16.9	19.9	22.5	172.2	41.6	10.27
3/17/2017 16:22	15.5	19.9	24.7	163.3	41.6	14.63
3/17/2017 16:23	17.1	29.2	20.9	97.2	15.8	4.06
3/17/2017 16:24	18.9	23.1	19.3	100.9	33.7	5.23
3/17/2017 16:25	21.9	28.6	22.1	73.4	17.9	2.24
3/17/2017 16:26	22.5	29.2	20.3	72.7	11.6	2

3/17/2017 16:27	25	33.9	24.1	62.1	7.2	1.43
3/17/2017 16:28	27.2	34.9	25.7	64.3	8	3.02
3/17/2017 16:29	28.9	34.4	27.8	63.2	8.2	2.44
3/17/2017 16:30	29.7	34.7	31.2	65.8	8.5	2.14
3/17/2017 16:31	30.1	35.7	27.6	69	6	1.28
3/17/2017 16:32	31.1	35.1	27.8	64.8	4.5	2.38
3/17/2017 16:33	31.5	37.4	30.8	61.4	5.2	4.65
3/17/2017 16:34	32.5	38.2	28.8	61.1	2.9	0.43
3/17/2017 16:35	33.3	38.6	31.6	62.3	2.7	0.15
3/17/2017 16:36	34.2	36.9	28.8	61.8	2.6	0.08
3/17/2017 16:37	33.9	36.3	31.2	59.5	5.8	0.14
3/17/2017 16:38	32.4	33	28.2	59.1	30.5	0.23
3/17/2017 16:39	30.1	28.7	24.1	69.6	34.8	0.55
3/17/2017 16:40	24.4	19.1	22.9	221.2	260.5	7.32
3/17/2017 16:41	15.4	20.2	65.4	657.5	559.2	28.31
3/17/2017 16:42	6.7	9.1	85.4	962.6	664.5	43.36
3/17/2017 16:43	1.7	-3.6	70.7	1191.6	697.2	48.76
3/17/2017 16:44	1	26.8	64.8	949.2	972.5	45.54
3/17/2017 16:45	0.7	-17	119.9	2110.2	1347.4	72.1
3/17/2017 16:46	0.6	22.8	117.6	1596.7	749.1	79.52
3/17/2017 16:47	0.5	5.9	38.3	717.3	487.2	37.69
3/17/2017 16:48	0.8	-0.8	29	471.4	361.7	24.87
3/17/2017 16:49	1.3	11.1	23.7	338.5	340.9	19.25
3/17/2017 16:50	1.4	7.8	23.9	335.2	397.8	24.29
3/17/2017 16:51	1.9	10.1	11.8	219.2	206.2	15.2
3/17/2017 16:52	2.5	4	14.6	240.2	271.6	13.26
3/17/2017 16:53	3	4	14.4	242.4	256.2	14.31
3/17/2017 16:54	3	10.2	13.2	216.7	240	12.69
3/17/2017 16:55	2.7	5.5	16	263	286.7	15.79
3/17/2017 16:56	3.2	15.6	16	171.4	187.5	10.24
3/17/2017 16:57	3.6	9.5	17.9	206.7	212	11.74
3/17/2017 16:58	4	3	15.6	209.8	298.5	11.01
3/17/2017 16:59	3.5	12.4	16.4	239.2	449.4	11.66
3/17/2017 17:00	2.3	75.1	68.7	885.5	910.4	46.11
3/17/2017 17:01	1.3	-25.5	82	1334.3	256.1	123.33
3/17/2017 17:02	1.5	-4.3	27.6	528.8	100.3	58.96
3/17/2017 17:03	3.3	8	13.2	236.1	49.3	21.5
3/17/2017 17:04	5.6	12.5	20.3	329.9	61.7	13.97
3/17/2017 17:05	6.8	14.2	25.3	376.1	532.3	19.87
3/17/2017 17:06	5.8	34.5	65.8	889.7	340.1	55.21
3/17/2017 17:07	3.7	-8.3	61.2	665.3	219	49.47
3/17/2017 17:08	3.4	6.4	11.8	193.1	52.8	12.83
3/17/2017 17:09	5.5	15.5	8.7	92.5	22.4	2.57
3/17/2017 17:10	8.3	14.8	7.3	82	25.1	3.51

3/17/2017 17:11	10.1	15.2	8.5	75.3	18.4	2.43
3/17/2017 17:12	11	17.9	6.9	71.1	14.9	1.55
3/17/2017 17:13	12.3	20.4	8.1	55.2	10.5	0.24
3/17/2017 17:14	14	21.3	10.4	53	8.5	0.04
3/17/2017 17:15	15.2	21.1	8.3	52.6	7.8	0.02
3/17/2017 17:16	15.4	19.7	8.9	53.1	7.5	0.05
3/17/2017 17:17	15.7	22.7	12	53	7.2	0.02
3/17/2017 17:18	16.3	24.3	12.8	52.9	6.7	0.03
3/17/2017 17:19	17.8	26.3	12.4	51.1	6	0.01
3/17/2017 17:20	18.7	23.5	13.4	51.4	5.6	0.01
3/17/2017 17:21	19.3	24.5	12.2	51.8	5.5	0.2
3/17/2017 17:22	19.1	24.2	13	51.3	5.5	0.78
3/17/2017 17:23	19.3	25.8	15	50.4	5.5	0.03
3/17/2017 17:24	19.3	25	14.2	50.1	6.4	0.02
3/17/2017 17:25	19.8	25.6	14.8	51.6	6	0.01
3/17/2017 17:26	20.3	27	13.8	51.6	5.3	0.02
3/17/2017 17:27	20.9	25.9	14.8	51.4	5.2	0.01
3/17/2017 17:28	20.9	25.3	13.8	50.1	4.8	0.02
3/17/2017 17:29	20.3	24.1	11.8	47.8	9.3	0.2
3/17/2017 17:30	20.2	27.2	12.2	50.6	4.7	0.02

3/20/2017 9:30	20.7	22.7	19.8	40.2	3.463	0.67
3/20/2017 9:31	20.1	20.3	20.7	38.5	7.337	5.99
3/20/2017 9:32	20	21.5	20.3	38.8	3.553	0.37
3/20/2017 9:33	20.2	21.1	20.7	39.8	3.373	0.23
3/20/2017 9:34	20.6	20.2	21.5	40.8	4.544	8.38
3/20/2017 9:35	20.4	22.2	22.5	41.2	6.616	14.31
3/20/2017 9:36	20.2	20.6	22.5	41.2	8.959	22.6
3/20/2017 9:37	20.4	23.2	20.7	40.3	5.445	6.02
3/20/2017 9:38	21	21.9	21.6	40	4.454	3.18
3/20/2017 9:39	21.1	20.5	20.7	38.7	3.914	2
3/20/2017 9:40	21	21.7	19.3	39.5	3.643	0.97
3/20/2017 9:41	20.9	21.9	21.5	40.3	3.553	0.51
3/20/2017 9:42	21.3	22.7	21.2	41.1	3.373	0.29
3/20/2017 9:43	22.1	23.1	21.4	40.7	3.463	0.35
3/20/2017 9:44	22.3	22.2	22.2	40.6	3.643	0.29
3/20/2017 9:45	22.7	24.1	23.3	41.9	3.643	0.25
3/20/2017 9:46	23.2	23.8	23	42.5	3.643	0.24
3/20/2017 9:47	23.8	24	22	42.4	3.643	0.21
3/20/2017 9:48	24	23.2	22.8	42.4	3.643	0.22
3/20/2017 9:49	23.7	22.8	22.2	42.4	3.823	0.23
3/20/2017 9:50	23.5	22.9	22.4	41.6	3.733	0.21
3/20/2017 9:51	23.1	22	22.1	41.5	3.823	0.24

3/20/2017 9:52	23.1	23.9	22.3	41.7	3.823	0.21
3/20/2017 9:53	23.4	24.6	22.7	43.2	3.643	0.25
3/20/2017 9:54	24	24.6	23.6	42.3	3.643	0.35
3/20/2017 9:55	24.5	24	23.8	42.4	3.643	0.18
3/20/2017 9:56	24.3	22.9	22.5	42	3.733	0.18
3/20/2017 9:57	24.1	24.9	24	43.9	3.463	0.19
3/20/2017 9:58	24.1	24.4	23.1	43.4	3.463	0.17
3/20/2017 9:59	24.3	24.5	24.1	43.8	3.553	0.18
3/20/2017 10:00	24.4	23.9	23.1	44.9	3.463	0.19
3/20/2017 10:01	24.3	25.1	23.7	45	3.283	0.16
3/20/2017 10:02	24.7	25.4	25	45.9	3.193	0.19
3/20/2017 10:03	25.1	25.8	23.8	45.5	3.193	0.19
3/20/2017 10:04	25.5	24.7	24.7	45.9	3.283	0.37
3/20/2017 10:05	25.6	26.4	24.7	46.7	3.283	0.52
3/20/2017 10:06	25.6	25.3	24.9	46.1	3.463	0.53
3/20/2017 10:07	25.5	24.5	23.5	45.1	3.553	0.44
3/20/2017 10:08	25.3	25.4	25.5	46.3	3.643	0.31
3/20/2017 10:09	25.3	25.3	25	45.9	3.193	0.22
3/20/2017 10:10	25.6	25.9	23.7	47	3.103	0.25
3/20/2017 10:11	25.8	25.3	24.6	46.8	3.103	0.2
3/20/2017 10:12	25.8	25.7	24.3	45.8	3.193	0.17
3/20/2017 10:13	25.6	25.4	23.3	46.5	3.103	0.33
3/20/2017 10:14	25.9	26.3	25.9	47.5	3.013	0.19
3/20/2017 10:15	26.2	25.9	25.6	47.5	3.013	0.23
3/20/2017 10:16	26.4	26.6	24.9	47	3.103	0.22
3/20/2017 10:17	26.4	26.7	26.2	47.9	3.013	0.17
3/20/2017 10:18	26.8	28	26.6	49.3	2.923	0.16
3/20/2017 10:19	27.1	26.8	25.9	47.8	6.076	0.32
3/20/2017 10:20	27.1	26.6	25.8	49.6	3.193	0.26
3/20/2017 10:21	26.5	25.6	24.8	48.2	3.553	0.55
3/20/2017 10:22	25.7	25.3	25.5	50.4	7.517	7.08
3/20/2017 10:23	25.6	26.9	26.5	47.5	4.364	0.38
3/20/2017 10:24	25.3	24.5	23	45.6	5.986	1.42
3/20/2017 10:25	25.8	27	26.1	48.6	4.364	0.31
3/20/2017 10:26	25.8	26	25.4	48.3	3.193	0.28
3/20/2017 10:27	26.1	26	25.7	49.6	3.643	1.21
3/20/2017 10:28	26	26	26.6	49.2	4.004	3.09
3/20/2017 10:29	25.8	25.6	26.1	49.4	3.283	0.31
3/20/2017 10:30	25.9	26.3	26.7	49.1	3.373	0.2
3/20/2017 10:31	26.3	26.5	26.8	50.5	3.103	0.2
3/20/2017 10:32	26.6	26.5	26.4	48.9	3.283	1.37
3/20/2017 10:33	26.7	26.7	24.9	50.2	3.283	0.26
3/20/2017 10:34	26.5	26.2	26.1	50	3.463	0.21
3/20/2017 10:35	26.4	26.2	25.8	49.4	3.823	0.2

3/20/2017 10:36	26.6	26.9	26.3	49.9	4.094	0.17
3/20/2017 10:37	27	27.4	26.1	50.5	3.013	0.19
3/20/2017 10:38	27.1	26.1	25.4	49.9	3.283	0.21
3/20/2017 10:39	27	27.6	25.8	50.9	3.013	0.23
3/20/2017 10:40	27.2	28.3	26	50.8	2.923	0.65
3/20/2017 10:41	27.5	27.9	26.2	51.4	2.923	0.39
3/20/2017 10:42	28.2	29.3	27.5	52.4	2.742	0.39
3/20/2017 10:43	28.8	30.1	28.7	53.4	2.652	0.35
3/20/2017 10:44	29.6	30.4	28.8	53.8	2.562	0.28
3/20/2017 10:45	27.3	37.5	38	644.7	773.193	10.15
3/20/2017 10:46	18.2	-1.3	95.4	1523.5	371.391	48.38
3/20/2017 10:47	9.4	3.7	39.4	623	217.247	19.5
3/20/2017 10:48	3.9	12.6	30.5	266.2	187.427	13.07
3/20/2017 10:49	5.6	10.7	17.3	124.8	53.823	7.65
3/20/2017 10:50	8.2	14	14.7	97.8	41.932	3.3
3/20/2017 10:51	9.4	7.9	20.5	229.5	135.986	7.56
3/20/2017 10:52	8.5	7.6	23.8	284.5	199.409	11.43
3/20/2017 10:53	6.6	9.2	30.7	409.4	152.022	10.84
3/20/2017 10:54	5.4	4.2	38.5	400.2	161.391	17.73
3/20/2017 10:55	5.8	13.7	25.3	280	85.535	8.98
3/20/2017 10:56	6	8.1	30.9	302.3	91.121	10.26
3/20/2017 10:57	6.1	13.3	38.8	430.4	246.076	13.35
3/20/2017 10:58	5.5	9.2	43.6	362.2	173.733	14.61
3/20/2017 10:59	5.1	7.8	34.3	272.3	109.95	11.74
3/20/2017 11:00	5.3	15	39.3	322.8	169.319	12.2
3/20/2017 11:01	6.2	9.8	24.3	202.9	59.499	7.82
3/20/2017 11:02	6.7	11.2	28.5	279.5	102.562	8.9
3/20/2017 11:03	6.7	9.4	27.5	246.9	92.742	9.67
3/20/2017 11:04	6.7	11.8	21.9	196	86.436	7.12
3/20/2017 11:05	6.8	8.2	24.3	220.6	59.229	7.06
3/20/2017 11:06	7	14.3	23.7	278.2	55.715	8.51
3/20/2017 11:07	6.5	11.1	30.2	333	94.184	13.7
3/20/2017 11:08	6.6	7.7	23.9	214.8	87.157	9.43
3/20/2017 11:09	6.9	10.9	26.9	274.8	113.193	11.16
3/20/2017 11:10	7.7	11	24.8	212.7	103.013	10.05
3/20/2017 11:11	7.7	14.7	23.6	264.6	145.265	8.52
3/20/2017 11:12	6.6	7.1	31.3	307.8	177.697	12.26
3/20/2017 11:13	5.8	8.3	22.2	197.1	98.058	7.88
3/20/2017 11:14	4.6	37.8	125.9	1666.7	436.166	46.68
3/20/2017 11:15	3.6	16.9	230.7	2427.3	445.175	116.18
3/20/2017 11:16	2	22.4	199.9	2110	310.4	107.12
3/20/2017 11:17	0.9	-0.2	136.8	1921.4	241.301	81.71
3/20/2017 11:18	0.9	2.3	127.7	1542.9	234.905	82.72
3/20/2017 11:19	0.9	13.4	94.9	1114.6	222.923	63.37

3/20/2017 11:20	0.8	5.6	112.6	1424.6	279.589	75.88
3/20/2017 11:21	0.8	2	80.7	944.7	160.31	64.08
3/20/2017 11:22	1	18	50.8	681.1	119.95	41.95
3/20/2017 11:23	1.3	8.3	49.3	627.4	268.508	42.9
3/20/2017 11:24	1.5	7.2	41.8	571.7	131.751	37.75
3/20/2017 11:25	1.7	0.3	37	519.1	103.193	33.96
3/20/2017 11:26	2	7.6	34.8	490.5	124.184	30.5
3/20/2017 11:27	2.4	4.7	26.9	398.3	103.103	26.37
3/20/2017 11:28	2.9	13.5	20.1	333.8	64.004	21.53
3/20/2017 11:29	3.2	4.6	24.7	373.6	92.562	23.2
3/20/2017 11:30	3.7	7.2	12.7	190.1	53.733	15.22
3/20/2017 11:31	4.2	9.9	10.4	216	65.895	11.88
3/20/2017 11:32	4.7	9.6	13.6	254.5	94.995	13.26
3/20/2017 11:33	4.5	9.1	19.6	254.8	209.499	12.55
3/20/2017 11:34	3.6	25.4	48.6	701.5	424.094	20.29
3/20/2017 11:35	2.5	7	100.5	1060.2	455.355	40.1
3/20/2017 11:36	2.4	1.4	28.7	355.4	103.193	18.06
3/20/2017 11:37	2.6	8.3	34	437.2	110.31	19.69
3/20/2017 11:38	3.3	4.3	25	292	113.733	15.45
3/20/2017 11:39	3.6	7.2	14	196.5	84.905	10.06
3/20/2017 11:40	3.9	11.3	17	248.6	126.526	9.9
3/20/2017 11:41	4	8.2	19.6	256.3	121.211	13.01
3/20/2017 11:42	4.4	11.2	9.4	126.1	59.859	7.41
3/20/2017 11:43	5.2	12.1	5.9	102.6	51.661	4.22
3/20/2017 11:44	6.3	10.7	6.8	109.5	65.715	4.86
3/20/2017 11:45	6.6	9.1	7	98.3	60.58	6.02
3/20/2017 11:46	6.5	17.4	20.3	328.3	164.995	7.72
3/20/2017 11:47	4.9	0.8	56.3	594.6	362.652	23.98
3/20/2017 11:48	4.1	10.5	24.4	235.8	102.112	14.35
3/20/2017 11:49	3.6	9.8	21.2	266.7	181.301	10.43
3/20/2017 11:50	4.2	29.3	27.5	422	213.373	11.95
3/20/2017 11:51	3.4	48.6	134.2	1404.7	592.923	51.67
3/20/2017 11:52	2.2	31.6	210.8	1628.3	458.148	92.59
3/20/2017 11:53	1.1	10.2	161.9	1567.5	574.905	70.45
3/20/2017 11:54	0.7	6.7	145.4	1386.4	452.382	68.45
3/20/2017 11:55	0.7	8.2	73	875.4	347.517	41.6
3/20/2017 11:56	0.7	6	77.7	984.3	364.094	40.51
3/20/2017 11:57	0.7	10.7	71.5	863.2	353.914	41.5
3/20/2017 11:58	0.7	1.7	53.3	624	277.337	34.86
3/20/2017 11:59	1.1	4.1	14.5	292.9	94.634	14.77
3/20/2017 12:00	1.4	7.2	33.1	498.9	252.652	20.6
3/20/2017 12:01	1.5	4.4	24.5	381.3	220.04	18.94
3/20/2017 12:02	1.2	2.3	37.3	467.4	285.085	22.24
3/20/2017 12:03	1.2	8.3	29.8	416.7	244.004	20.11

3/20/2017 12:04	1.3	4	28.8	383.6	199.859	19.85
3/20/2017 12:05	1.5	2.9	28	393.7	218.328	16.28
3/20/2017 12:06	1.8	18.9	23.6	460.4	183.283	15.14
3/20/2017 12:07	1.8	-1.4	39.7	519.5	203.463	22.23
3/20/2017 12:08	1.6	11.7	40.4	601.7	276.706	23.11
3/20/2017 12:09	1.4	4.6	37.9	507.6	205.625	21.9
3/20/2017 12:10	1.5	-4.9	23.9	424.6	235.535	17.74
3/20/2017 12:11	1.6	9.9	22	397.6	228.778	17.48
3/20/2017 12:12	1.5	4.7	42.8	597.1	277.968	25.7
3/20/2017 12:13	1.3	0.7	29.3	384.7	209.769	21.82
3/20/2017 12:14	1.2	2.3	29.8	459.3	275.445	21.23
3/20/2017 12:15	1.5	2.3	13.2	247.3	139.319	12.95
3/20/2017 12:16	1.8	3.9	13.9	248.3	156.796	13.13
3/20/2017 12:17	2.2	4.7	16.2	232.1	187.787	13.4
3/20/2017 12:18	2.4	7.9	11.7	163	125.085	10.48
3/20/2017 12:19	2.2	5.8	27.7	268.7	246.166	18.04
3/20/2017 12:20	2	6.2	17.5	198.4	173.103	13.36
3/20/2017 12:21	1.7	2.5	13.6	162.5	184.995	11.88
3/20/2017 12:22	1.8	2.4	12.8	153.2	171.841	11.19
3/20/2017 12:23	1.9	6.8	13.1	178.5	171.571	10.87
3/20/2017 12:24	2.1	3.7	13	143.7	121.841	9.76
3/20/2017 12:25	3.1	10.2	6.1	97.3	67.968	4.33
3/20/2017 12:26	3.7	6.3	13	166.5	122.742	8.31
3/20/2017 12:27	4	8.4	19.4	231.2	166.256	11.97
3/20/2017 12:28	3.2	4.1	18.5	189.1	154.184	12.59
3/20/2017 12:29	2.6	2.7	24.1	235.6	172.202	15.01
3/20/2017 12:30	2.3	5.5	18.3	198.4	172.832	12.32
3/20/2017 12:31	2.1	9.3	21.1	227.4	190.58	13.57
3/20/2017 12:32	1.9	-0.7	27.2	212.2	164.184	16.88
3/20/2017 12:33	2.5	8.3	8.6	128	109.319	7.14
3/20/2017 12:34	2.8	4.4	12.8	139.9	122.022	9.48
3/20/2017 12:35	3.9	7.6	6.9	99.8	117.607	5.72
3/20/2017 12:36	3.9	8.8	11.3	144.5	128.508	7.28
3/20/2017 12:37	4	4.7	12.2	126	124.634	8.55
3/20/2017 12:38	4.2	12.1	7.6	92.4	96.436	4.56
3/20/2017 12:39	4	5.8	13.7	142	147.247	8.92
3/20/2017 12:40	4.1	6.5	14.5	123.4	104.995	8.23
3/20/2017 12:41	4.6	11	7.4	68.4	45.085	2.97
3/20/2017 12:42	5.8	13.6	9.4	85.3	64.995	3.54
3/20/2017 12:43	6.6	6.3	11.2	106.3	111.211	5.92
3/20/2017 12:44	6.6	11.8	8.1	75.6	65.715	3.37
3/20/2017 12:45	6.3	9.3	11.7	99.3	72.652	4.86
3/20/2017 12:46	7.3	15.1	10.5	63	39.409	7.5
3/20/2017 12:47	8.1	12.8	7.6	69.3	50.13	6.53

3/20/2017 12:48	8.9	14.1	8.5	58.5	32.652	10.57
3/20/2017 12:49	8.7	10	7.4	77.3	65.445	3.47
3/20/2017 12:50	7.6	8.7	8.8	88.5	92.652	4.22
3/20/2017 12:51	6.8	12.8	9.3	76.4	58.418	3.59
3/20/2017 12:52	7.5	16.3	9	55.2	43.283	2.08
3/20/2017 12:53	9.3	15.7	9.1	52.6	37.157	2.09
3/20/2017 12:54	11	17.9	13.2	52.9	38.778	8.4
3/20/2017 12:55	10.9	12.3	11.8	54.6	44.004	19.24
3/20/2017 12:56	9.7	10.3	9.9	70.5	71.481	3.59
3/20/2017 12:57	7.5	7.4	11.3	102.2	99.679	6.04
3/20/2017 12:58	6.4	12.1	11.6	67.3	60.13	3.79
3/20/2017 12:59	7.1	13.1	11.3	72	66.076	3.61
3/20/2017 13:00	8.1	9.5	11.3	82.2	86.256	7.5
3/20/2017 13:01	9.8	21	14.5	51.2	16.616	3.11
3/20/2017 13:02	11.8	22.2	14.8	44.8	20.941	1.01
3/20/2017 13:03	14.2	22	15.6	51.3	17.247	3.32
3/20/2017 13:04	15.5	21.8	16.8	50	16.166	2.37
3/20/2017 13:05	15.6	22.4	15.1	45.4	12.562	0.82
3/20/2017 13:06	15.6	21.1	14.6	55	24.995	1.6
3/20/2017 13:07	14.5	16.4	13	56.8	38.959	2.52
3/20/2017 13:08	12.9	15.5	13.8	66	35.445	2.45
3/20/2017 13:09	12.1	19.2	15.5	62.6	51.661	1.44
3/20/2017 13:10	11.9	16.8	17	82.9	74.724	2.9
3/20/2017 13:11	11.4	14.5	14.7	85.3	78.868	3.59
3/20/2017 13:12	9.5	10	21.5	143.7	140.67	6.45
3/20/2017 13:13	9.2	14.9	16.5	75.5	52.292	2.97
3/20/2017 13:14	9.2	13	14.3	67.4	49.319	3.04
3/20/2017 13:15	9.6	10.1	16	100.1	101.121	4.73
3/20/2017 13:16	7.7	9	16.9	114.8	116.616	6.66
3/20/2017 13:17	6.6	10.9	15.8	118.6	121.571	6.54
3/20/2017 13:18	6	11.2	15	93.6	92.652	5.37
3/20/2017 13:19	6.9	10.8	13.3	87.8	87.607	4.04
3/20/2017 13:20	6.4	8	15.2	124.4	143.283	6.73
3/20/2017 13:21	4.9	7.4	20.6	149.1	156.977	11.33
3/20/2017 13:22	3.4	8	23.5	175.8	174.814	12.17
3/20/2017 13:23	3.4	7.4	18.4	136.8	133.733	8.81
3/20/2017 13:24	4	9.3	20.5	143.1	136.526	9.09
3/20/2017 13:25	4.1	5.5	26.3	185.8	158.598	12.46
3/20/2017 13:26	3.5	4.7	25.6	183.6	161.841	13.8
3/20/2017 13:27	4.1	10.5	14.2	100.3	86.346	6.1
3/20/2017 13:28	4.8	7.9	16.4	120.3	101.301	7.26
3/20/2017 13:29	5.2	7.1	18.3	136.1	143.013	8.13
3/20/2017 13:30	4.9	8	16.5	123.1	118.598	7.15
3/20/2017 13:31	6.2	16.5	20.3	113.1	69.049	4.87

3/20/2017 13:32	8	13.4	24.8	155.4	85.895	6.74
3/20/2017 13:33	10.3	17.3	19	90	42.923	4.27
3/20/2017 13:34	10.5	10.9	16.2	93.7	71.751	4.39
3/20/2017 13:35	8.5	6.9	19.8	146.1	122.112	9.2
3/20/2017 13:36	6.2	7.2	17.4	120.2	102.112	9.23
3/20/2017 13:37	4.7	8.9	14.9	99.2	84.094	6.41
3/20/2017 13:38	5.4	8.9	13	91.5	83.643	5.68
3/20/2017 13:39	5.5	7.3	14.3	110.7	118.328	5.95
3/20/2017 13:40	4.7	6.8	17.7	137.7	106.346	7.92
3/20/2017 13:41	5.3	11.5	19.4	118.6	58.328	5.76
3/20/2017 13:42	5.5	6.9	18	140.5	140.85	8.34
3/20/2017 13:43	5.4	5.3	15.5	122.1	123.914	8.32
3/20/2017 13:44	4.3	7.1	15.2	109.8	113.463	7.45
3/20/2017 13:45	4.3	9.9	16.3	125.6	128.778	7.16
3/20/2017 13:46	5.9	13.4	21.6	121.9	63.553	7.72
3/20/2017 13:47	8.7	20.2	19.1	82.9	21.481	3.31
3/20/2017 13:48	12.2	21.8	19.3	72.1	13.553	2.17
3/20/2017 13:49	15	21.2	19.8	67.6	12.022	1.95
3/20/2017 13:50	15.9	23.1	19	77.4	12.382	2.01
3/20/2017 13:51	15.5	18.2	19.1	104.7	57.157	4.04
3/20/2017 13:52	14.1	18.8	22.6	112.4	55.265	5.45
3/20/2017 13:53	13.7	21.3	21.3	88.4	38.058	3.45
3/20/2017 13:54	12.8	12.3	20.7	127.5	76.616	5.47
3/20/2017 13:55	12.1	15.7	18	90.1	53.373	5.06
3/20/2017 13:56	10.1	9.6	16.3	125.8	126.706	6.23
3/20/2017 13:57	7.3	3.6	20.4	179.5	163.193	10.82
3/20/2017 13:58	5.6	8.7	12.6	99.7	106.076	7.74
3/20/2017 13:59	4.6	10.3	14.3	114	112.292	6.63
3/20/2017 14:00	5.5	8.7	15.8	120.1	113.193	7.49
3/20/2017 14:01	6	10	15.2	122.5	110.67	6.82
3/20/2017 14:02	6.6	13.3	16.8	117.2	104.364	6.48
3/20/2017 14:03	7.2	9.6	15.6	115.4	106.706	6.05
3/20/2017 14:04	7.2	9.3	12.6	103.5	104.094	6.26
3/20/2017 14:05	6.6	10	16.6	139.5	116.977	6.88
3/20/2017 14:06	6.2	12.2	15.1	102	78.058	5.69
3/20/2017 14:07	6.6	9	16.4	118	99.95	5.79
3/20/2017 14:08	6.6	7.2	17	127.9	128.778	7.11
3/20/2017 14:09	6.2	9.2	15.4	124.5	142.562	6.66
3/20/2017 14:10	5.8	9.9	18.3	121.6	127.517	6.98
3/20/2017 14:11	7.6	15.1	19.8	117.1	65.085	5.86
3/20/2017 14:12	8.7	9.7	18.5	132.9	94.094	6.62
3/20/2017 14:13	9.8	18.2	17.3	97.2	60.76	4.34
3/20/2017 14:14	10.6	20.4	20.6	95.8	55.625	3.93
3/20/2017 14:15	10.5	19.5	32.6	307.8	129.859	13.63

3/20/2017 14:16	9.9	12	38	323.3	94.724	15.98
3/20/2017 14:17	8.2	6.6	30.1	241	89.499	14.21
3/20/2017 14:18	6.9	14.2	39.3	368.7	141.571	21.06
3/20/2017 14:19	5.7	7	41.3	329.4	161.121	22.47
3/20/2017 14:20	4.8	6	34.8	300.9	126.706	16.25
3/20/2017 14:21	4.9	13	49.1	412.3	135.895	25.59
3/20/2017 14:22	5.6	10.2	37.8	341.9	95.175	19.32
3/20/2017 14:23	6.3	19.3	30	268.2	98.058	14.31
3/20/2017 14:24	7.7	15.4	26.7	210.4	85.986	10.19
3/20/2017 14:25	9.2	19.6	26.3	197.9	84.544	9.27
3/20/2017 14:26	9.4	12.3	28.1	232.1	76.796	13.36
3/20/2017 14:27	9	9.9	19.8	151.7	106.256	7.87
3/20/2017 14:28	7.7	13.3	24.2	216.2	126.436	11.81
3/20/2017 14:29	7.5	7.2	20.5	179.8	103.733	8.97
3/20/2017 14:30	7.4	20.7	24.3	190	67.787	9.56
3/20/2017 14:31	8.9	21.3	31.1	286	71.301	14.31
3/20/2017 14:32	9.3	11	27.3	204.6	71.751	12.59
3/20/2017 14:33	9.5	10	27.3	200.1	112.112	10.86
3/20/2017 14:34	9.6	19	25.8	226.2	72.832	10.41
3/20/2017 14:35	8.9	2.7	29	233	121.121	13.18
3/20/2017 14:36	9	15.6	25.6	185.5	76.616	9.9
3/20/2017 14:37	9.3	14.6	29.9	240	81.841	11.95
3/20/2017 14:38	10.2	15.2	25.6	203.6	71.661	9.42
3/20/2017 14:39	10.8	14.2	19.9	110.5	45.895	8.19
3/20/2017 14:40	11.2	20.6	23.6	175.1	85.355	7.31
3/20/2017 14:41	10.7	12.1	20.5	133.2	52.382	9.6
3/20/2017 14:42	12.3	22.5	18.4	104.6	39.049	3.49
3/20/2017 14:43	11.7	13.4	23.5	163.3	67.877	9.25
3/20/2017 14:44	12.9	21	17.9	76.9	31.301	3.36
3/20/2017 14:45	13.3	21.1	17.9	68.7	28.418	1.87
3/20/2017 14:46	14.6	24.3	20.4	112	45.265	4.14
3/20/2017 14:47	14.1	17.3	25.7	191.4	83.103	9.35
3/20/2017 14:48	12.9	18.1	21.5	129.9	45.355	7.25
3/20/2017 14:49	12.2	19.6	23.6	133.2	52.292	6.37
3/20/2017 14:50	12.7	21.3	24.5	144.5	57.877	6.84
3/20/2017 14:51	12.3	14.4	31.3	221.2	89.229	11.02
3/20/2017 14:52	12.2	16.5	25.6	145.8	49.679	7.77
3/20/2017 14:53	12.3	16.9	24.7	140.4	56.706	7.02
3/20/2017 14:54	13.4	22.8	24.3	119.2	31.841	6.18
3/20/2017 14:55	14.1	18.6	22.9	125.4	37.427	5.45
3/20/2017 14:56	14.7	22.5	26.2	159.6	52.202	7.21
3/20/2017 14:57	14.3	17.2	29	185.9	53.103	10.1
3/20/2017 14:58	13.8	28.8	34.6	241.8	79.589	10.83
3/20/2017 14:59	12.9	18.5	34.2	228.8	76.166	12.81

3/20/2017 15:00	12.9	17.4	29.4	200.7	63.283	10.55
3/20/2017 15:01	13.4	20.8	22.7	123.7	50.76	5.8
3/20/2017 15:02	14.2	25.6	22.7	121.1	54.094	5.28
3/20/2017 15:03	15.1	21.5	26.1	119.1	47.607	5.31
3/20/2017 15:04	16.3	25.5	21.5	63.4	12.112	2.12
3/20/2017 15:05	18.4	28.8	21.2	44.1	6.526	0.24
3/20/2017 15:06	20.6	29.9	21.8	40.9	4.814	0.06
3/20/2017 15:07	21.6	27.6	23.1	66.7	12.923	1.22
3/20/2017 15:08	21.4	28.2	21.3	50.6	11.301	0.93
3/20/2017 15:09	21.9	31.5	24	46.8	7.427	0.31
3/20/2017 15:10	22.4	29.6	23.4	46.1	6.256	0.32
3/20/2017 15:11	22.3	25.8	23.7	60.5	6.346	1.51
3/20/2017 15:12	20.6	20.8	24.8	85.1	59.409	3.01
3/20/2017 15:13	19	28	23.4	62.3	15.895	1.71
3/20/2017 15:14	19.2	26.6	21.5	57.4	10.58	0.96
3/20/2017 15:15	19.6	24.9	23.4	84.5	46.256	2.37
3/20/2017 15:16	18.9	22.9	26.9	109.8	65.265	3.79
3/20/2017 15:17	17.1	21.8	26.3	109.6	48.688	4.34
3/20/2017 15:18	15.2	17.4	24.8	122.1	57.337	5.14
3/20/2017 15:19	14.7	18.3	22.2	86.9	23.193	3.39
3/20/2017 15:20	15	22.2	21.8	67.8	14.544	2.34
3/20/2017 15:21	17.4	28	22.7	43.5	5.175	0.34
3/20/2017 15:22	20.4	30.7	24.6	41.5	4.184	0.1
3/20/2017 15:23	23.2	32.9	26.3	41	3.193	-0.03
3/20/2017 15:24	24.8	32.5	25.9	40.3	3.013	-0.04
3/20/2017 15:25	25.6	33.2	26.7	42.1	2.832	-0.05
3/20/2017 15:26	25.9	32.5	28.4	41.9	2.742	-0.04
3/20/2017 15:27	26.1	33.4	27.8	41.4	2.652	-0.05
3/20/2017 15:28	25.9	31.1	27.3	41	2.562	-0.01
3/20/2017 15:29	25.2	28.7	25.2	39	2.832	0.69
3/20/2017 15:30	24.6	32.4	26.5	40.8	2.652	-0.02
3/20/2017 15:31	24.5	31	26.8	40.3	2.472	-0.02
3/20/2017 15:32	24.8	30.4	25.4	39.9	2.472	0
3/20/2017 15:33	24.6	29.5	24.6	38.9	2.742	0.12
3/20/2017 15:34	24.8	33.6	27.4	41.9	3.553	2.95
3/20/2017 15:35	25.9	35.1	29.4	42.7	2.472	0.12
3/20/2017 15:36	27.2	32.2	28.7	42.2	2.382	0.11
3/20/2017 15:37	27.5	33.4	29.5	42.1	2.562	0.06
3/20/2017 15:38	27.4	32.5	29.8	43	2.652	0.26
3/20/2017 15:39	27.7	36.2	31.5	44.4	2.562	0.03
3/20/2017 15:40	28.3	35.4	30.9	44.5	2.562	-0.04
3/20/2017 15:41	29	35.8	29.9	44.7	2.652	-0.05
3/20/2017 15:42	29.2	35.2	30	45.1	2.652	-0.05
3/20/2017 15:43	29.2	35.4	29.2	45.6	2.562	-0.06

3/20/2017 15:44	29.4	37.1	30.1	46.1	2.562	-0.07
3/20/2017 15:45	29.8	36.4	31.4	46.9	2.562	-0.06
3/20/2017 15:46	30	36.1	31.1	45.9	2.562	-0.08
3/20/2017 15:47	29.9	35.4	31.4	45.5	2.562	-0.07
3/20/2017 15:48	29.8	38	32.5	47.3	2.562	-0.09
3/20/2017 15:49	30.3	38.6	32.8	48.5	2.652	-0.08
3/20/2017 15:50	31.2	38.5	32.6	48.6	2.652	-0.09
3/20/2017 15:51	31.5	37.1	32.2	48.3	2.742	-0.07
3/20/2017 15:52	31.5	37.8	33	48.8	2.832	-0.07
3/20/2017 15:53	31.2	37.8	33.3	49.2	2.832	-0.06
3/20/2017 15:54	31.2	38	34.4	49.2	2.832	-0.05
3/20/2017 15:55	31.3	36.5	33.7	48.5	2.832	-0.06
3/20/2017 15:56	31.1	37.4	32	48.7	2.923	-0.05
3/20/2017 15:57	31.5	39.6	33.6	50.6	2.832	-0.07
3/20/2017 15:58	31.9	38.8	35.3	50.8	2.923	-0.05
3/20/2017 15:59	32	36	33.9	49.2	3.013	-0.05
3/20/2017 16:00	31.4	37.8	33.5	49.9	3.193	-0.03
3/20/2017 16:01	31.5	41	35.5	53.2	3.103	-0.05
3/20/2017 16:02	32.4	39.9	36	53.2	3.103	-0.04
3/20/2017 16:03	33.3	39.3	34.4	53	3.013	-0.05
3/20/2017 16:04	33.4	39.1	35.2	53.1	3.103	-0.05
3/20/2017 16:05	33.1	39.2	34.7	51.7	3.103	-0.04
3/20/2017 16:06	32.8	38.3	33.9	51.2	3.103	-0.04
3/20/2017 16:07	32.5	39.1	34.6	51.5	3.103	-0.05
3/20/2017 16:08	32.3	38.4	34.1	51.7	3.283	-0.05
3/20/2017 16:09	32.4	39.7	35.8	53.3	3.373	-0.04
3/20/2017 16:10	32.2	37.5	33.5	51.2	3.463	-0.04
3/20/2017 16:11	31.5	35.4	32	50.7	3.463	-0.03
3/20/2017 16:12	30.8	37.1	32.6	51.3	3.463	-0.03
3/20/2017 16:13	31.4	40.5	35.4	54	3.103	-0.04
3/20/2017 16:14	32.5	39.1	36	53.8	3.013	-0.04
3/20/2017 16:15	33.1	39.6	34	53.5	3.103	-0.05
3/20/2017 16:16	33	39.8	35.2	54.4	3.013	-0.04
3/20/2017 16:17	32.9	39.6	35.8	54.3	3.013	-0.02
3/20/2017 16:18	33.3	39.7	34.1	54.8	3.013	0.01
3/20/2017 16:19	33.7	41.8	36.7	56	2.923	-0.04
3/20/2017 16:20	33.9	40.6	36.2	54.8	2.923	-0.03
3/20/2017 16:21	34.1	40.1	34.6	54.5	2.742	-0.03
3/20/2017 16:22	34.1	41.9	35.8	55.6	2.652	-0.05
3/20/2017 16:23	35	46.5	34.2	60.2	2.562	-0.05
3/20/2017 16:24	36.3	42.6	38	56.6	2.652	-0.05
3/20/2017 16:25	36.5	42.7	36	54	2.562	-0.05
3/20/2017 16:26	37.2	46.3	25.6	58.4	2.562	-0.06
3/20/2017 16:27	33.1	13.4	11	-6.6	2.562	-0.04

Sycan Marsh October 2017

Date&Time	O3 NO-CL	O3 UV-C-H	O3 UV-C	NO2-C	CO	THC
10/11/2017 11:00 AM						
10/11/2017 11:01 AM						
10/11/2017 11:02 AM						
10/11/2017 11:03 AM						
10/11/2017 11:04 AM						
10/11/2017 11:05 AM						
10/11/2017 11:06 AM						
10/11/2017 11:07 AM						
10/11/2017 11:08 AM						
10/11/2017 11:09 AM						
10/11/2017 11:10 AM						
10/11/2017 11:11 AM						
10/11/2017 11:12 AM						
10/11/2017 11:13 AM						
10/11/2017 11:14 AM						
10/11/2017 11:15 AM						
10/11/2017 11:16 AM						
10/11/2017 11:17 AM						
10/11/2017 11:18 AM						
10/11/2017 11:19 AM						
10/11/2017 11:20 AM						
10/11/2017 11:21 AM						
10/11/2017 11:22 AM						
10/11/2017 11:23 AM	0.5	33.9	36.1	0.6	2.8183	
10/11/2017 11:24 AM	0.3	33.5	36.6	2.3	2.416	
10/11/2017 11:25 AM	0.3	34.3	37.3	0.1	1.1891	
10/11/2017 11:26 AM	0.2	34.4	37.1	-0.2	1.1347	
10/11/2017 11:27 AM	0.3	29.2	34.6	3.6	2.2095	
10/11/2017 11:28 AM	0.2	32.3	36	1.1	2.4692	
10/11/2017 11:29 AM	0.1	30.1	33.9	2.7	3.2681	
10/11/2017 11:30 AM	0	31.2	33.9	6.2	3.0266	
10/11/2017 11:31 AM	0	32.5	36	3.4	1.3601	
10/11/2017 11:32 AM	0	33.5	36.4	0.2	1.564	
10/11/2017 11:33 AM	-0.1	28.7	32.2	0	1.1725	
10/11/2017 11:34 AM	1.8	23.9	25.4	0	1.2848	
10/11/2017 11:35 AM	9.9	33	36.4	0.1	1.1489	
10/11/2017 11:36 AM	19.9	31.6	32.9	6.6	1.1559	
10/11/2017 11:37 AM	28.4	30.8	34.8	6.6	1.1693	
10/11/2017 11:38 AM	29.2	28.4	31.2	10.8	1.2138	
10/11/2017 11:39 AM	28.6	31.9	34	7.8	1.1735	
10/11/2017 11:40 AM	29	32.2	35.7	2.6	1.1578	
10/11/2017 11:41 AM	29.3	24.9	28.2	17.3	1.2497	
10/11/2017 11:42 AM	26.8	23.4	26.6	21.9	1.2893	
10/11/2017 11:43 AM	25.4	33.8	35.1	3.4	1.2222	
10/11/2017 11:44 AM	27.1	32.1	35.3	1.7	1.2234	

10/11/2017 11:45 AM	31.3	34.7	36.4	1.9	1.4073
10/11/2017 11:46 AM	30.9	51	128.3	143.6	4.9936
10/11/2017 11:47 AM	21.5	245.8	2055.7	766	77.7558
10/11/2017 11:48 AM	10.5	326.9	3246	934	136.5833
10/11/2017 11:49 AM	1.7	165	2113.8	446	87.8125
10/11/2017 11:50 AM	0.1	39	618.7	100.1	19.85
10/11/2017 11:51 AM	0.9	25.2	321.7	64.4	7.1284
10/11/2017 11:52 AM	1.7	43.9	460.1	130.6	14.0049
10/11/2017 11:53 AM	2.6	12.1	131.2	32.8	4.4147
10/11/2017 11:54 AM	3.3	8.6			
10/11/2017 11:55 AM					
10/11/2017 11:56 AM	5.7	10	14.9	11.4	3.4701
10/11/2017 11:57 AM	6.8	13.7	23.9	9.4	1.8881
10/11/2017 11:58 AM	8	12.2	14.1	7.6	1.519
10/11/2017 11:59 AM					
10/11/2017 12:00 PM	9.6	13.3	12	5.5	1.2752
10/11/2017 12:01 PM	10.3	16.9	17.4	13.6	1.4731
10/11/2017 12:02 PM	9.8	24.8	131.6	96.7	6.6728
10/11/2017 12:03 PM	7.8	25.2	197.1	122.6	9.5108
10/11/2017 12:04 PM	5.8	33.1	237.8	121.8	10.3646
10/11/2017 12:05 PM	4.5	31.6	222.5	113.8	9.5393
10/11/2017 12:06 PM	4.3	36.4	309.5	132.9	13.3217
10/11/2017 12:07 PM	4	26.3	206.7	90.5	8.2848
10/11/2017 12:08 PM	4	22.3	184.6	82	7.5048
10/11/2017 12:09 PM	4.3	22.5	159.4	65.7	6.4805
10/11/2017 12:10 PM	5	18.5	102.4	39.6	4.3428
10/11/2017 12:11 PM	6.3	21.6	92.5	38.8	3.4236
10/11/2017 12:12 PM	6.4	31.2	243.2	98	9.5188
10/11/2017 12:13 PM	5.7	26	212.1	79.4	8.2818
10/11/2017 12:14 PM	5	19.2	117.5	41.7	4.6045
10/11/2017 12:15 PM	6	14.4	61.6	18.5	2.444
10/11/2017 12:16 PM	7.7	15.7	53.7	20.1	2.5137
10/11/2017 12:17 PM	9.1	14.2	41.6	14.9	1.9327
10/11/2017 12:18 PM	9.7	16.9	49.8	18.9	2.59
10/11/2017 12:19 PM	9.5	18.9	69.1	34.2	3.5635
10/11/2017 12:20 PM	8.8	19.9	72.5	47.1	4.2318
10/11/2017 12:21 PM	8.3	18	68.7	43.4	3.6713
10/11/2017 12:22 PM	7.9	29.4	178.2	89.7	8.1842
10/11/2017 12:23 PM	7.1	31.9	217.4	79.9	10.2964
10/11/2017 12:24 PM	6.5	20.7	110.9	35.5	4.5813
10/11/2017 12:25 PM	5.8	42.4	349.4	141.8	13.6263
10/11/2017 12:26 PM	4.8	49.4	402.9	146.6	17.4133
10/11/2017 12:27 PM	3.7	25.4	194.7	81.1	10.1483
10/11/2017 12:28 PM	3.4	26.6	198.9	106.7	8.4143
10/11/2017 12:29 PM	3.9	28	209.2	100.9	9.661
10/11/2017 12:30 PM	3.7	34.1	276.9	144	13.6092
10/11/2017 12:31 PM	3.2	27	211.1	99.8	11.1924

10/11/2017 12:32 PM	3.5	19.2	125.9	60.9	6.0871	
10/11/2017 12:33 PM	4.1	27.1	202.3	80.3	9.0158	
10/11/2017 12:34 PM	4.7	19.4	140	57	6.4057	
10/11/2017 12:35 PM	5.4	17.4	103.7	50.5	4.5381	
10/11/2017 12:36 PM	6.3	16.2	72.9	32.3	3.5916	
10/11/2017 12:37 PM	7.3	17.4	71.9	27.5	3.2943	
10/11/2017 12:38 PM	7.5	17.7	91.1	65.7	4.3066	
10/11/2017 12:39 PM	7.2	28.1	166.9	101.9	7.0822	
10/11/2017 12:40 PM	6.6	26.3	178.5	83.4	7.888	
10/11/2017 12:41 PM	6.2	29.4	212.7	97.4	10.3983	
10/11/2017 12:42 PM	5.8	54.7	403.8	189.6	16.3577	
10/11/2017 12:43 PM	4.4	71.5	702.2	189.3	26.5842	
10/11/2017 12:44 PM	3.8	27.9	220.3	46.7	7.4525	
10/11/2017 12:45 PM	4	29.7	269.3	75.2	8.3148	
10/11/2017 12:46 PM	4.5	29.7	278.8	62.2	11.4478	
10/11/2017 12:47 PM	4.6	26.5	228	56.9	8.0989	
10/11/2017 12:48 PM	4.6	28.2	194.7	74.6	7.5148	
10/11/2017 12:49 PM	5.3	17.3	108.1	31.7	4.4514	
10/11/2017 12:50 PM	6.3	15.5	56.5	17.6	2.9852	
10/11/2017 12:51 PM	7.7	14.9	45.8	10.6	2.1508	
10/11/2017 12:52 PM	8.5	15.5	39.4	10.8	2.2286	
10/11/2017 12:53 PM	8.8	13.7	74.8	27.6	3.2973	
10/11/2017 12:54 PM	8.4	15.5	68	22.4	3.2766	
10/11/2017 12:55 PM	8.2	14	46.9	16.2	2.6874	
10/11/2017 12:56 PM	8.3	13.7	46.7	16.2	2.6742	
10/11/2017 12:57 PM	9	13.6	38.9	12.9	2.2578	
10/11/2017 12:58 PM	9.7	16	25.4	5.9	1.8139	
10/11/2017 12:59 PM	10.7	15.8	25.9	5	1.5759	
10/11/2017 1:00 PM	11	15.8	33.3	6.1	2.0154	
10/11/2017 1:01 PM	11	16.3	31.4	8.2	1.9621	
10/11/2017 1:02 PM	10.9	17.1	28.3	9.4	1.7935	
10/11/2017 1:03 PM	10.9	15	33.9	9.5	2.1345	
10/11/2017 1:04 PM	11.1	14.5	23.6	6.4	1.7792	
10/11/2017 1:05 PM	11.1	15.5	25.1	4.5	1.7657	
10/11/2017 1:06 PM	11.4	17.5	24.6	3.6	1.8064	
10/11/2017 1:07 PM	11.7	17.4	18.2	3.2	1.4803	
10/11/2017 1:08 PM	11.4	18.3	42.3	16.8	3.7469	
10/11/2017 1:09 PM	10.7	18	48.9	14.7	3.6977	
10/11/2017 1:10 PM	10	16.5	38.8	10.1	1.9965	
10/11/2017 1:11 PM	10.1	15	28.1	6.1	1.7436	7.8
10/11/2017 1:12 PM	10.3	17.1	46	7	2.681	8.1
10/11/2017 1:13 PM	10.2	22.5	115.2	8	5.4648	8.6
10/11/2017 1:14 PM	9.8	25.5	178.9	9.8	9.0407	8.3
10/11/2017 1:15 PM	9.9	21.4	149	8.3	6.8069	7.2
10/11/2017 1:16 PM	10.2	22.4	142.9	8.6	7.3873	7
10/11/2017 1:17 PM	10.6	23.2	161	8.7	8.8053	7.1
10/11/2017 1:18 PM	10.5	21.9	132.3	8.5	8.4585	6.8

10/11/2017 1:19 PM	10.8	19.5	87.5	6.7	5.4929	6.4
10/11/2017 1:20 PM	11.2	19.5	52.8	6	5.2861	6.4
10/11/2017 1:21 PM	11.8	20	59.5	5.3	4.9833	5.7
10/11/2017 1:22 PM	11.9	21.4	80.7	6.2	6.6523	5.7
10/11/2017 1:23 PM	12	20.7	57.2	5.7	4.9066	5.4
10/11/2017 1:24 PM	12.2	21.5	62.5	5.5	5.2608	5.4
10/11/2017 1:25 PM	12.6	18.7	41	4.7	3.8849	5.2
10/11/2017 1:26 PM	13.1	18.7	43.2	4.5	4.0098	5.1
10/11/2017 1:27 PM	13.1	21.6	77.7	5.4	5.7944	5.4
10/11/2017 1:28 PM	12.9	19.8	62.7	5.5	5.0835	5.3
10/11/2017 1:29 PM	12.6	21.8	90.1	5.7	6.1411	5.4
10/11/2017 1:30 PM	12.9	19.3	37.7	4.5	3.4213	5
10/11/2017 1:31 PM	13.2	21.6	92.8	5	5.05	5.1
10/11/2017 1:32 PM	13.3	20.3	54	4.7	3.4199	5
10/11/2017 1:33 PM	12.7	18.4	92.6	5.4	4.6584	5.1
10/11/2017 1:34 PM	12.4	20	35.3	4.6	2.7583	4.6
10/11/2017 1:35 PM	13	19.8	33.3	3.5	1.9347	4.5
10/11/2017 1:36 PM	14.2	19.1	22.1	3.3	1.8878	4.4
10/11/2017 1:37 PM	15.1	21.1	26.9	3.2	1.8297	4.4
10/11/2017 1:38 PM	15.4	19.1	26.3	2.9	1.7243	4.3
10/11/2017 1:39 PM	15.6	20.3	16.6	2.6	1.5885	4.3
10/11/2017 1:40 PM	15.9	19.6	27.9	2.6	1.846	4.3
10/11/2017 1:41 PM	15.9	19.8	27	2.7	1.9626	4.3
10/11/2017 1:42 PM	15.9	19.5	12.8	2.4	1.4978	4.2
10/11/2017 1:43 PM	16.2	19.5	14.9	2.2	1.4062	4.1
10/11/2017 1:44 PM	16.6	21.3	22.6	2.2	1.7671	4.1
10/11/2017 1:45 PM	16.8	20.1	12.5	2.2	1.4888	4.1
10/11/2017 1:46 PM	16.7	20.1	14.7	2.1	1.4958	4
10/11/2017 1:47 PM	16.8	22.6	13.9	2	1.4521	4
10/11/2017 1:48 PM	16.9	21.9	21.9	2.1	1.7233	4
10/11/2017 1:49 PM	17.1	20.9	15.9	2	1.3973	4
10/11/2017 1:50 PM	17.4	20.9	23.4	2	1.4893	4
10/11/2017 1:51 PM	17.5	20.8	33.4	2.2	1.7521	4
10/11/2017 1:52 PM	17.4	21.2	17.9	2.1	1.5045	4
10/11/2017 1:53 PM	17.3	22.2	36.8	2.3	2.0297	4.1
10/11/2017 1:54 PM	17.4	21.2	17.3	2.2	1.4998	4
10/11/2017 1:55 PM	17.6	21.1	24.7	2	1.5006	4
10/11/2017 1:56 PM	17.7	21.8	27.5	2.1	1.6283	4
10/11/2017 1:57 PM	17.8	22.2	35.7	2.1	1.7921	4
10/11/2017 1:58 PM	17.7	22	22.5	2.1	1.5938	4
10/11/2017 1:59 PM	17.9	20.6	22.1	2	1.486	4
10/11/2017 2:00 PM	17.8	20.8	28.1	2.1	1.6022	4.1
10/11/2017 2:01 PM	18	23.1	20.4	2	1.4844	4.1
10/11/2017 2:02 PM	18	23.6	31	2.1	1.695	4.1
10/11/2017 2:03 PM	18.5	23.6	27.1	2.1	1.6092	4.1
10/11/2017 2:04 PM	18.7	23.2	27.3	2	1.5792	4
10/11/2017 2:05 PM	18.9	21.7	28	1.9	1.5011	4

10/11/2017 2:06 PM	18.8	23.3	29.5	1.9	1.6173	4.1
10/11/2017 2:07 PM	18.6	21.8	28.6	2	1.6069	4.1
10/11/2017 2:08 PM	18.4	21.8	28.6	2	1.6643	4.1
10/11/2017 2:09 PM	18.6	23.1	28.5	1.9	1.5783	4
10/11/2017 2:10 PM	18.8	23.4	27.5	1.9	1.5499	4
10/11/2017 2:11 PM	19	22.6	25.5	1.8	1.4224	4
10/11/2017 2:12 PM	18.7	21.1	28	1.8	1.6569	4.1
10/11/2017 2:13 PM	18.8	22.4	25	1.7	1.431	4
10/11/2017 2:14 PM	18.6	23.3	31.3	1.8	1.7247	4
10/11/2017 2:15 PM	18.7	22.5	29.3	1.8	1.6899	4
10/11/2017 2:16 PM	18.7	21.6	28.9	1.8	1.7668	4
10/11/2017 2:17 PM	18.8	21.5	26.7	1.8	1.6743	4
10/11/2017 2:18 PM	19	22.5	23	1.8	1.4001	3.9
10/11/2017 2:19 PM	19.1	23.3	26.8	2.1	1.6089	3.8
10/11/2017 2:20 PM	19.6	22.9	26.1	1.7	1.4593	3.8
10/11/2017 2:21 PM	19.4	22.3	25.8	1.7	1.3993	3.8
10/11/2017 2:22 PM	18.7	21.4	31.3	2.1	1.8666	3.8
10/11/2017 2:23 PM	17.5	21.2	46.8	11	2.805	4.1
10/11/2017 2:24 PM	15.3	20.3	50.6	38.3	4.5913	4.3
10/11/2017 2:25 PM	12.5	27.4	131.1	83.2	10.5325	5.2
10/11/2017 2:26 PM	9.8	22.4	92.2	55	8.8313	5.2
10/11/2017 2:27 PM	8.4	28.8	127.7	77.8	11.0443	5.5
10/11/2017 2:28 PM	7.5	32.2	164.6	93.6	14.2692	6.1
10/11/2017 2:29 PM	8.5	20.2	48.2	17.2	4.1423	4.9
10/11/2017 2:30 PM	10.6	20.6	47.1	18.6	2.5281	4.2
10/11/2017 2:31 PM	12	22.2	88.8	40.2	6.139	4.8
10/11/2017 2:32 PM	11.7	21.7	77.9	48.1	5.3112	4.7
10/11/2017 2:33 PM	10.9	20.9	48.6	19.5	3.7968	4.4
10/11/2017 2:34 PM	12.2	17	37.3	30.4	2.0654	4.1
10/11/2017 2:35 PM	13	20.7	35.2	18.1	2.4643	4.1
10/11/2017 2:36 PM	14.1	19.1	47.5	37.2	3.1025	4.1
10/11/2017 2:37 PM	13.7	20.5	32.3	9.8	2.5373	4.1
10/11/2017 2:38 PM	15.6	22	27.7	5.3	1.2904	3.8
10/11/2017 2:39 PM	16.7	20.5	30.8	16.7	1.7642	3.8
10/11/2017 2:40 PM	17.1	20.1	32.3	17.6	2.8283	4
10/11/2017 2:41 PM	16.5	19.4	33	20.4	2.1761	3.9
10/11/2017 2:42 PM	15.6	21	29.5	10	2.1073	3.9
10/11/2017 2:43 PM	15.6	18.9	34.6	25.4	3.0023	3.9
10/11/2017 2:44 PM	14.6	19.2	39.8	25.7	4.318	4.1
10/11/2017 2:45 PM	13.8	19.7	40.7	22.9	6.821	4.2
10/11/2017 2:46 PM	12.8	19.8	54.1	25.5	4.8471	4.8
10/11/2017 2:47 PM	14.2	24.8	50	3.6	2.7351	4.8
10/11/2017 2:48 PM	16.1	21.7	39.3	4.2	3.0579	5.2
10/11/2017 2:49 PM	18.3	23	25.8	2.5	1.1591	5.3
10/11/2017 2:50 PM	19.1	23	25.1	2.2	1.0949	5.6
10/11/2017 2:51 PM	19.1	21.5	33.8	8.3	1.5705	5.2
10/11/2017 2:52 PM	18.6	21.6	26.9	2.4	1.2838	4.1

10/11/2017 2:53 PM	18.3	21.7	26	2.1	1.1689	3.9
10/11/2017 2:54 PM	18.5	22.5	35.1	3	1.3563	4
10/11/2017 2:55 PM	18.8	24.2	36.2	3.6	1.4628	4
10/11/2017 2:56 PM	18.6	22.9	34.4	2.8	1.3894	4
10/11/2017 2:57 PM	18.4	20.4	31.7	2.8	1.3563	3.9
10/11/2017 2:58 PM	18.1	22.9	28.5	2.9	1.2422	3.9
10/11/2017 2:59 PM	18.2	22.1	30	2.8	1.3062	3.8
10/11/2017 3:00 PM	18.3	23.2	28.1	2.1	1.1925	3.8
10/11/2017 3:01 PM	18.4	23	27.9	2	1.2264	3.8
10/11/2017 3:02 PM	18.6	23.1	25.7	1.8	1.1703	3.7
10/11/2017 3:03 PM	18.9	22.6	25.6	1.6	1.1305	3.7
10/11/2017 3:04 PM	19.2	22.7	26.7	1.8	1.1923	4
10/11/2017 3:05 PM	19.3	24.1	26.4	1.7	1.1674	4.8
10/11/2017 3:06 PM	19.2	23.6	42	5.9	1.582	4.3
10/11/2017 3:07 PM	17.8	22	95.8	18.7	3.2873	4.4
10/11/2017 3:08 PM	15.4	19	122.5	22.4	4.4212	4.5
10/11/2017 3:09 PM	12.9	19.4	93.1	24	3.2518	4.2
10/11/2017 3:10 PM	12.3	19.4	72.6	17.2	2.6657	4
10/11/2017 3:11 PM	12.6	18.2	48.4	19.7	2.6835	4
10/11/2017 3:12 PM	13.1	17	65	17.5	2.8545	4
10/11/2017 3:13 PM	12.5	17.3	33	17.9	2.1537	3.8
10/11/2017 3:14 PM	12.8	18.4	28.3	14.7	1.78	3.6
10/11/2017 3:15 PM	13.7	18.8	27.3	11.6	1.6188	4
10/11/2017 3:16 PM	14.1	23.7	70.1	25.3	3.3607	4.2
10/11/2017 3:17 PM	12.7	24.9	88.6	27.1	4.4095	4.5
10/11/2017 3:18 PM	11.2	19.8	71.7	25	3.2487	4.1
10/11/2017 3:19 PM	10.3	17.9	51.9	25.1	2.9612	4.1
10/11/2017 3:20 PM	10.6	16.1	41.1	22.4	2.394	3.8
10/11/2017 3:21 PM	11	20.1	51.7	24.2	2.843	3.9
10/11/2017 3:22 PM	11.4	20.8	67.9	28.4	3.5637	4.1
10/11/2017 3:23 PM	11.5	20.3	68.7	18.7	3.0283	4.5
10/11/2017 3:24 PM	12.2	20.3	64	17.9	2.778	5.2
10/11/2017 3:25 PM	13.1	20.7	45.2	13	2.072	3.9
10/11/2017 3:26 PM	14.4	19.3	44.3	16.9	1.9343	3.7
10/11/2017 3:27 PM	14.4	19	46.5	17.6	2.2637	3.8
10/11/2017 3:28 PM	14.9	18.9	36.7	10.1	1.6857	3.7
10/11/2017 3:29 PM	15.1	21	43.9	9.4	1.9123	3.7
10/11/2017 3:30 PM	16.2	22.1	44	9.3	1.8492	3.7
10/11/2017 3:31 PM	16.2	19.3	56.6	20.9	2.257	3.8
10/11/2017 3:32 PM	15.4	19.3	46.5	15.2	2.269	3.8
10/11/2017 3:33 PM	14.8	21.9	45.5	12.8	2.0353	3.7
10/11/2017 3:34 PM	14.7	20.5	56.3	16.1	2.3195	3.8
10/11/2017 3:35 PM	14.5	17.9	67	28.1	3.065	4
10/11/2017 3:36 PM	13.4	18.7	60.9	33.5	2.885	3.9
10/11/2017 3:37 PM	11	20.6	76.3	57.4	5.0503	4.5
10/11/2017 3:38 PM	10.7	20.5	42.4	17.2	2.5275	4.2
10/11/2017 3:39 PM	11.8	20.3	42.5	24.3	2.754	3.8

10/11/2017 3:40 PM	12.9	22.4	67.1	41.9	3.7305	4.2
10/11/2017 3:41 PM	11.3	27.9	168	137.2	10.9052	5.9
10/11/2017 3:42 PM	7.2	30.2	170.9	145.8	11.67	6.4
10/11/2017 3:43 PM	4.5	21.3	127.2	84.2	7.503	5.7
10/11/2017 3:44 PM	4.2	21.4	105.7	64	5.2177	5
10/11/2017 3:45 PM	5.9	17.3	72.1	29.5	4.8435	4.5
10/11/2017 3:46 PM	8.5	18.2	38.5	15.7	6.6822	4.2
10/11/2017 3:47 PM	11.1	17.2	37.3	16.3	5.1807	4.6
10/11/2017 3:48 PM	12.5	19.6	40.5	14.8	2.0008	4.8
10/11/2017 3:49 PM	12.3	15.3	32.4	25.5	2.2133	4.8
10/11/2017 3:50 PM		15.3	30.5	19.9	2.1418	4.8
10/11/2017 3:51 PM	8.7	18.5	29.1	9.1	1.829	4.7
10/11/2017 3:52 PM	9.3	18.6	26.1	4.4	1.3278	4.5
10/11/2017 3:53 PM	8.3	17.8	32.6	21.2	1.9363	5.4
10/11/2017 3:54 PM	0.4	21.2	70.7	30.5	3.059	4.8
10/11/2017 3:55 PM	0.5	21.5	43.9	16	3.0807	4.1
10/11/2017 3:56 PM	0.6	22.2	27.9	4.3	1.0998	3.5
10/11/2017 3:57 PM	0.7	20.9	28.9	6.5	1.3255	3.5
10/11/2017 3:58 PM	0.7	20.3	30.6	3.4	1.2812	3.4
10/11/2017 3:59 PM	0.6	21.9	56.4	2.5	2.4338	3.7
10/11/2017 4:00 PM	0.5	23.5	101.1	16.5	5.9345	4.4
10/11/2017 4:01 PM	0.5	20	42.5	12.5	2.3635	3.8
10/11/2017 4:02 PM	0.5	19.4	78.4	29.7	3.7123	3.9
10/11/2017 4:03 PM	0.6	18.7	42.6	11.5	1.8388	3.8
10/11/2017 4:04 PM	0.6	19.4	44.8	17.7	1.856	3.6
10/11/2017 4:05 PM	0.5	18.8	75.4	40.8	4.0955	4.1
10/11/2017 4:06 PM	0.5	17.7	58.2	29.8	2.7563	3.8
10/11/2017 4:07 PM	0.5	18.4	39.5	16.3	2.0202	3.7
10/11/2017 4:08 PM	0.5	19.7	52	13	2.115	3.6
10/11/2017 4:09 PM	0.6	20.3	34.5	3.9	1.4757	3.5
10/11/2017 4:10 PM	0.6	20.7	33.7	3.1	1.272	3.5
10/11/2017 4:11 PM	0.6	18.9	38.5	3.6	1.3643	3.5
10/11/2017 4:12 PM	0.6	20	39.8	4.2	1.4282	3.5
10/11/2017 4:13 PM	0.6	20.2	31.1	3.4	1.2618	3.6
10/11/2017 4:14 PM	0.6	21.7	39.6	4.8	1.59	5.8
10/11/2017 4:15 PM	-4.1	20.1	39.6	3.7	1.4562	6.3
10/11/2017 4:16 PM	-13.6	20.9	47.9	3.3	1.5212	5.8
10/11/2017 4:17 PM	-22.5	22.5	56.1	4	1.837	6.5
10/11/2017 4:18 PM	-24.8	22.3	55.9	4.1	1.8877	5.8
10/11/2017 4:19 PM	0.6	23.3	63.1	4.6	1.9997	4.6
10/11/2017 4:20 PM	0.6	24.9	57.9	4.6	2.0248	3.8
10/11/2017 4:21 PM	0.6	22.1	52.7	3.7	1.8733	3.7
10/11/2017 4:22 PM	0.6	23.2	53.6	4.1	1.9222	3.6
10/11/2017 4:23 PM	0.6	22.5	54.5	7.1	2.2675	3.7
10/11/2017 4:24 PM	0.6	30.8	82	15.9	2.187	3.8
10/11/2017 4:25 PM	0.5	30.7	195.3	41.5	8.672	5.3
10/11/2017 4:26 PM	0.5	23	110.5	13.9	4.3998	4.7

10/11/2017 4:27 PM	0.4	26.1	124.8	17.5	4.338	4.3
10/11/2017 4:28 PM	0.5	23.1	102.5	12.4	3.5742	4.1
10/11/2017 4:29 PM	0.5	24.5	68.1	9.7	2.5105	3.9
10/11/2017 4:30 PM	0.5	22.4	71.5	10.6	2.4222	3.7
10/11/2017 4:31 PM	0.5	38.8	197.8	27.2	5.1888	4.4
10/11/2017 4:32 PM	0.4	39.2	287.1	46.3	12.3005	6.5
10/11/2017 4:33 PM	0.3	45	291.2	54.9	8.9608	5.6
10/11/2017 4:34 PM	0.2	39.8	309.2	54.4	14.2797	7.2
10/11/2017 4:35 PM	0.2	23.1	152.9	36	5.318	4.9
10/11/2017 4:36 PM	0.2	20.6	133.7	36	5.1865	4.5
10/11/2017 4:37 PM	0.3	19.9	105.7	27.7	4.5832	4.2
10/11/2017 4:38 PM	0.3	17.8	77.3	20.3	3.08	3.8
10/11/2017 4:39 PM	0.3	18.7	74.6	29.5	3.4562	3.9
10/11/2017 4:40 PM	0.3	21.2	111.8	40.4	5.0047	4.1
10/11/2017 4:41 PM	0.2	20.8	112	47.9	6.558	4.5
10/11/2017 4:42 PM	0.2	20.5	84.5	29.3	4.5275	4.1
10/11/2017 4:43 PM	0.2	19.7	100	53.9	6.333	4.4
10/11/2017 4:44 PM	0.2	25.3	105.9	38	5.2165	4.5
10/11/2017 4:45 PM	0.2	38.6	234.8	99.3	11.8207	5.6
10/11/2017 4:46 PM	0.2	28.5	188.4	68.5	11.983	5.8
10/11/2017 4:47 PM	0.2	25	142.9	56.7	6.1403	4.4
10/11/2017 4:48 PM	0.1	19.7	67.4	25.4	3.8887	4.1
10/11/2017 4:49 PM	0.3	28.9	156.2	79.1	7.7822	4.5
10/11/2017 4:50 PM	1.7	20.9	98.9	41.1	6.8312	4.6
10/11/2017 4:51 PM	5.1	21	63	24.1	2.8053	3.6
10/11/2017 4:52 PM	8.8	19	34.2	8.9	2.0998	3.4
10/11/2017 4:53 PM	12	25.3	73.6	24.2	3.0552	3.5
10/11/2017 4:54 PM	12.4	22.1	88.3	30.1	4.4607	3.9
10/11/2017 4:55 PM	11.8	18.2	69.8	30.3	4.3152	3.8
10/11/2017 4:56 PM	10.7	25.3	90.9	47.8	3.8192	3.7
10/11/2017 4:57 PM	8.9	23.7	117.8	54.6	7.1308	4.4
10/11/2017 4:58 PM	7.6	30.7	167.2	73.2	17.6167	5
10/11/2017 4:59 PM	4.8	27.9	181.1	116.5	12.9717	5.5
10/11/2017 5:00 PM	4.3	22.2	126.9	106.4	8.1912	4.7
10/11/2017 5:01 PM	5.3	18.2	66.7	100.2	3.796	3.8
10/11/2017 5:02 PM	6.7	21.4	88.4	80.3	5.97	3.9
10/11/2017 5:03 PM	6.9	24	140.5	88.5	10.383	4.7
10/11/2017 5:04 PM	5.4	32.2	206.2	96.3	12.4227	5.2
10/11/2017 5:05 PM	4.7	21.9	120.2	44.4	6.6033	4.6
10/11/2017 5:06 PM	5.2	25.4	155.9	31.7	6.9735	4.7
10/11/2017 5:07 PM	7.1	28.3	134	26.5	4.0483	4.1
10/11/2017 5:08 PM	8	34.2	218.4	40.2	6.965	5
10/11/2017 5:09 PM	7.8	30.9	228.7	43.1	11.7845	5.9
10/11/2017 5:10 PM	7.5	23.2	116.6	18.2	4.367	4.4
10/11/2017 5:11 PM	7.8	27	143.9	24.4	5.2305	4.4
10/11/2017 5:12 PM	7.9	33.3	226.8	38.4	8.836	5.2
10/11/2017 5:13 PM	7.5	26.4	137.4	27.4	5.3827	4.6

10/11/2017 5:14 PM	6.8	24.4	155.7	25.8	6.9639	4.9
10/11/2017 5:15 PM	7.8	22.6	114.8	14.2	4.4056	4.1
10/11/2017 5:16 PM	8.6	18.8	68.3	10.9	3.1368	3.8
10/11/2017 5:17 PM	9.6	19.4	94.5	10.9	3.9112	4.5
10/11/2017 5:18 PM	10.3	16.2	36.4	6.8	1.6265	4.7
10/11/2017 5:19 PM	11.1	16	33.4	6.9	6.3105	4.4
10/11/2017 5:20 PM	11.4	14.3	27	6.2	6.5414	4.3
10/11/2017 5:21 PM	10.9	14.7	32.8	4.5	4.2706	4.6
10/11/2017 5:22 PM	10.3	15.4	26.3	3.8	3.4488	4.2
10/11/2017 5:23 PM	9.9	14.9	33	4.1	9.6464	4.9
10/11/2017 5:24 PM	9.3	18.4	40.4	5.8	19.5483	5.7
10/11/2017 5:25 PM	7.4	16.1	35.5	6.7	7.7216	6.1

Konza Prairie November 2017

Date&Time	O3 NO-CL	O3 UV-C-H	O3 UV-C	NO2-C	NO	CO	THC
11/10/2017 12:00 AM	30.8	29.3	28.8	-0.1	0.6	-0.141	-1.5
11/10/2017 12:01 AM	30.9	29.9	28.3	-0.1	1.1	-0.135	-1.5
11/10/2017 12:02 AM	30.9	29.1	28.6	0	1.1	-0.142	-1.5
11/10/2017 12:03 AM	30.9	29.3	28.6	0.2	1.3	-0.149	-1.5
11/10/2017 12:04 AM	30.9	29.5	28.7	0.2	1.2	-0.141	-1.5
11/10/2017 12:05 AM	31	29.1	29.1	0.2	0.6	-0.135	-1.5
11/10/2017 12:06 AM	31.2	28.4	28.5	0.1	1.1	-0.141	-1.5
11/10/2017 12:07 AM	31	28.6	28.4	0	1	-0.145	-1.5
11/10/2017 12:08 AM	30.9	29.9	28.3	-0.1	0.9	-0.139	-1.5
11/10/2017 12:09 AM	30.7	29.8	28.9	-0.1	0.8	-0.135	-1.5
11/10/2017 12:10 AM	30.9	27.9	28.5	-0.2	0.8	-0.145	-1.5
11/10/2017 12:11 AM	31.1	27.8	28.8	-0.2	0.6	-0.142	-1.5
11/10/2017 12:12 AM	31.3	30.4	28.8	-0.2	0.9	-0.141	-1.5
11/10/2017 12:13 AM	31.1	28.7	28.3	-0.2	2.1	-0.136	-1.5
11/10/2017 12:14 AM	30.9	29.5	28.5	-0.1	1.7	-0.148	-1.5
11/10/2017 12:15 AM	30.7	28.6	28.6	0	0.4	-0.142	-1.5
11/10/2017 12:16 AM	30.7	28.9	28.5	-0.1	0.5	-0.134	-1.5
11/10/2017 12:17 AM	30.9	28.7	28.8	-0.1	0.5	-0.137	-1.5
11/10/2017 12:18 AM	30.9	29.1	28.7	-0.1	0.6	-0.147	-1.5
11/10/2017 12:19 AM	30.9	30.3	28.5	-0.2	1	-0.138	-1.5
11/10/2017 12:20 AM	30.8	29	28.3	-0.2	1	-0.134	-1.5
11/10/2017 12:21 AM	30.9	31.3	28.7	-0.3	0.4	-0.140	-1.5
11/10/2017 12:22 AM	30.9	29.2	28.5	-0.3	1.4	-0.147	-1.5
11/10/2017 12:23 AM	31	30.1	28.7	-0.3	0.5	-0.137	-1.5
11/10/2017 12:24 AM	30.8	29.3	28.5	-0.3	0.8	-0.137	-1.5
11/10/2017 12:25 AM	30.8	27.1	28.5	-0.3	0.9	-0.147	-1.5
11/10/2017 12:26 AM	30.6	28.6	28.5	-0.2	0.6	-0.137	-1.5
11/10/2017 12:27 AM	30.5	29.7	28.2	-0.2	0.7	-0.132	-1.5
11/10/2017 12:28 AM	30.5	29.1	28.3	-0.2	0.7	-0.144	-1.5
11/10/2017 12:29 AM	30.7	28.9	28.5	-0.2	0.6	-0.150	-1.5
11/10/2017 12:30 AM	30.8	28.7	28.2	-0.2	1	-0.140	-1.5
11/10/2017 12:31 AM	30.9	29.4	28.5	-0.2	0.5	-0.139	-1.5
11/10/2017 12:32 AM	30.7	30.7	28.5	-0.2	0.7	-0.150	-1.5
11/10/2017 12:33 AM	30.6	28.7	28	-0.2	1	-0.141	-1.5
11/10/2017 12:34 AM	30.3	28.4	27.9	-0.3	1.3	-0.134	-1.5
11/10/2017 12:35 AM	30.2	29.2	28.7	-0.3	0.5	-0.142	-1.5
11/10/2017 12:36 AM	30.3	28.6	28.3	-0.3	1.2	-0.147	-1.5
11/10/2017 12:37 AM	30.6	29	28	-0.3	1	-0.136	-1.5
11/10/2017 12:38 AM	30.5	28.1	28	-0.3	0.9	-0.134	-1.5
11/10/2017 12:39 AM	30.4	29.8	28	-0.3	0.6	-0.143	-1.5
11/10/2017 12:40 AM	30.3	29.5	27.8	-0.2	1.6	-0.142	-1.5
11/10/2017 12:41 AM	30.3	27.5	28.1	-0.2	0.6	-0.132	-1.5
11/10/2017 12:42 AM	30.4	29.7	28.3	-0.3	0.6	-0.132	-1.5
11/10/2017 12:43 AM	30.4	28.9	28	-0.3	1	-0.145	-1.5
11/10/2017 12:44 AM	30.6	28.3	28.2	-0.3	1	-0.139	-1.5

11/10/2017 12:45 AM	30.4	28.3	28	-0.3	0.8	-0.138	-1.5
11/10/2017 12:46 AM	30.6	30.5	28.3	-0.2	0.9	-0.142	-1.5
11/10/2017 12:47 AM	30.6	29.3	28.6	-0.1	0.2	-0.145	-1.5
11/10/2017 12:48 AM	30.8	28.8	28.4	-0.1	0.8	-0.140	-1.5
11/10/2017 12:49 AM	30.7	31.1	28.5	-0.1	0.3	-0.131	-1.5
11/10/2017 12:50 AM	30.7	30.3	28.5	-0.1	0.9	-0.139	-1.5
11/10/2017 12:51 AM	30.8	29.5	28.6	-0.2	0.7	-0.148	-1.5
11/10/2017 12:52 AM	30.8	30.7	28.7	-0.2	0.7	-0.143	-1.5
11/10/2017 12:53 AM	30.8	29.7	28.3	-0.3	0.9	-0.134	-1.5
11/10/2017 12:54 AM	30.7	29.6	28.6	-0.3	0.9	-0.146	-1.5
11/10/2017 12:55 AM	30.7	29.6	28.6	-0.3	0.5	-0.151	-1.5
11/10/2017 12:56 AM	30.7	29.5	28.6	-0.3	0.6	-0.132	-1.5
11/10/2017 12:57 AM	30.7	29.2	28.4	-0.3	0.5	-0.136	-1.5
11/10/2017 12:58 AM	30.7	29.3	28.7	-0.3	0.7	-0.149	-1.5
11/10/2017 12:59 AM	30.9	28.6	28.6	-0.4	0.7	-0.142	-1.5
11/10/2017 1:00 AM	30.9	30.5	28.4	-0.4	0.8	-0.123	-1.5
11/10/2017 1:01 AM	31.1	29.5	28.7	-0.4	0.5	-0.140	-1.5
11/10/2017 1:02 AM	31.1	29.3	28.5	-0.4	0.9	-0.148	-1.5
11/10/2017 1:03 AM	31.1	29.9	28.8	-0.5	0.7	-0.134	-1.5
11/10/2017 1:04 AM	31.1	30	29	-0.5	0.3	-0.136	-1.5
11/10/2017 1:05 AM	31.1	28.6	28.3	-0.5	0.6	-0.145	-1.5
11/10/2017 1:06 AM	31	28.5	28.4	-0.5	0.6	-0.141	-1.5
11/10/2017 1:07 AM	30.9	27.2	28.5	-0.5	0.4	-0.135	-1.5
11/10/2017 1:08 AM	30.9	28.4	28.5	-0.5	0.3	-0.140	-1.5
11/10/2017 1:09 AM	31	28.9	28.2	-0.6	0.8	-0.148	-1.5
11/10/2017 1:10 AM	31	29.7	28.5	-0.5	0.6	-0.141	-1.5
11/10/2017 1:11 AM	31	28.5	28.2	-0.5	0.9	-0.128	-1.5
11/10/2017 1:12 AM	30.9	29.2	28.2	-0.5	0.6	-0.132	-1.5
11/10/2017 1:13 AM	30.8	29.8	28.6	-0.5	1.2	-0.143	-1.5
11/10/2017 1:14 AM	30.9	29.1	28.6	-0.5	0.2	-0.134	-1.5
11/10/2017 1:15 AM	30.9	27.4	28.2	-0.5	1	-0.136	-1.5
11/10/2017 1:16 AM	30.9	27.3	28.3	-0.5	0.7	-0.141	-1.5
11/10/2017 1:17 AM	30.6	28.9	28.2	-0.5	0.6	-0.139	-1.5
11/10/2017 1:18 AM	30.5	29	28.2	-0.5	0.7	-0.131	-1.5
11/10/2017 1:19 AM	30.6	28.6	28.2	-0.5	0.9	-0.128	-1.5
11/10/2017 1:20 AM	30.6	27.6	28.3	-0.5	0.8	-0.142	-1.5
11/10/2017 1:21 AM	30.5	28.8	27.7	-0.5	1.4	-0.138	-1.5
11/10/2017 1:22 AM	30.4	28.7	28.2	-0.4	0.5	-0.126	-1.5
11/10/2017 1:23 AM	30.3	29.4	27.8	-0.4	0.5	-0.134	-1.5
11/10/2017 1:24 AM	30.3	28.6	27.8	-0.4	0.9	-0.138	-1.5
11/10/2017 1:25 AM	30.3	27.1	27.8	-0.3	0.4	-0.133	-1.5
11/10/2017 1:26 AM	30.3	28.9	28	-0.4	0.5	-0.121	-1.5
11/10/2017 1:27 AM	30.4	28.9	27.9	-0.3	0.3	-0.157	-1.5
11/10/2017 1:28 AM	30.6	28.9	28.4	-0.3	0.3	-0.144	-1.5
11/10/2017 1:29 AM	30.7	28.3	28.5	-0.4	0.3	-0.133	-1.5
11/10/2017 1:30 AM	30.6	28.6	28	-0.4	0.6	-0.128	-1.5
11/10/2017 1:31 AM	30.5	27.9	28	-0.4	0.8	-0.150	-1.5

11/10/2017 1:32 AM	30.4	28.8	28.2	-0.4	0.3	-0.137	-1.5
11/10/2017 1:33 AM	30.3	27.4	27.5	-0.4	0.7	-0.125	-1.5
11/10/2017 1:34 AM	30.4	29.5	28.1	-0.4	0.4	-0.132	-1.5
11/10/2017 1:35 AM	30.3	27.5	27.9	-0.4	0.7	-0.136	-1.5
11/10/2017 1:36 AM	30.5	27.7	27.9	-0.4	0.6	-0.133	-1.5
11/10/2017 1:37 AM	30.5	29.1	28	-0.4	0.5	-0.126	-1.5
11/10/2017 1:38 AM	30.3	28.1	27.5	-0.4	1.2	-0.139	-1.5
11/10/2017 1:39 AM	30.1	27.9	27.8	-0.4	0.5	-0.134	-1.5
11/10/2017 1:40 AM	30.1	27.7	27.9	-0.4	0.3	-0.128	-1.5
11/10/2017 1:41 AM	30.3	28.5	28	-0.4	0.4	-0.130	-1.5
11/10/2017 1:42 AM	30.4	28.5	28.1	-0.4	0.1	-0.143	-1.5
11/10/2017 1:43 AM	30.3	29.3	27.9	-0.4	0.8	-0.131	-1.5
11/10/2017 1:44 AM	30.1	25.9	27.5	-0.4	1.2	-0.124	-1.5
11/10/2017 1:45 AM	30	28.2	28	-0.4	0.5	-0.133	-1.5
11/10/2017 1:46 AM	30.1	28.2	27.7	-0.4	0.5	-0.136	-1.5
11/10/2017 1:47 AM	30.2	29.5	28.1	-0.4	0.4	-0.132	-1.5
11/10/2017 1:48 AM	30.3	28.3	27.7	-0.4	0.7	-0.126	-1.5
11/10/2017 1:49 AM	30.1	28.3	27.8	-0.4	0.3	-0.129	-1.5
11/10/2017 1:50 AM	30	27.2	27.9	-0.4	0.2	-0.143	-1.5
11/10/2017 1:51 AM	30.1	28.5	27.9	-0.4	0.5	-0.137	-1.5
11/10/2017 1:52 AM	30.1	27.9	27.5	-0.4	1.1	-0.129	-1.5
11/10/2017 1:53 AM	30.1	27.4	27.7	-0.4	0.7	-0.130	-1.5
11/10/2017 1:54 AM	29.9	28.2	27.6	-0.4	0.4	-0.137	-1.5
11/10/2017 1:55 AM	29.9	26.8	27.5	-0.4	0.8	-0.134	-1.5
11/10/2017 1:56 AM	29.9	30.3	27.7	-0.4	0.2	-0.129	-1.5
11/10/2017 1:57 AM	29.8	28.2	27.4	-0.4	0.7	-0.131	-1.5
11/10/2017 1:58 AM	29.9	29	27.8	-0.4	0.4	-0.134	-1.5
11/10/2017 1:59 AM	29.9	28.3	27.9	-0.4	0.4	-0.129	-1.5
11/10/2017 2:00 AM	30	28.5	27.4	-0.5	1.1	-0.130	-1.5
11/10/2017 2:01 AM	29.9	27.8	27.2	-0.5	1.1	-0.137	-1.5
11/10/2017 2:02 AM	29.5	28.1	27.3	-0.5	0.6	-0.133	-1.5
11/10/2017 2:03 AM	29.5	28	27.2	-0.5	0.6	-0.123	-1.5
11/10/2017 2:04 AM	29.5	28.3	27.2	-0.5	0.9	-0.134	-1.5
11/10/2017 2:05 AM	29.6	26.6	27.5	-0.5	0.9	-0.136	-1.5
11/10/2017 2:06 AM	29.6	26.9	27.2	-0.5	0.5	-0.124	-1.5
11/10/2017 2:07 AM	29.6	27.9	27.5	-0.5	0.5	-0.124	-1.5
11/10/2017 2:08 AM	29.8	28.1	27.4	-0.4	0.4	-0.136	-1.5
11/10/2017 2:09 AM	29.7	26.6	26.8	-0.4	1.2	-0.133	-1.5
11/10/2017 2:10 AM	29.6	27.9	27.2	-0.3	0.6	-0.125	-1.5
11/10/2017 2:11 AM	29.4	27.5	26.9	-0.3	1.9	-0.131	-1.5
11/10/2017 2:12 AM	29.2	27.7	27.1	-0.2	2.1	-0.133	-1.5
11/10/2017 2:13 AM	29.3	26.8	27	-0.2	0.6	-0.126	-1.5
11/10/2017 2:14 AM	29.4	28.2	27.6	-0.2	0.6	-0.121	-1.5
11/10/2017 2:15 AM	29.6	27.6	27.2	-0.2	0.5	-0.129	-1.5
11/10/2017 2:16 AM	29.7	28	27.1	-0.3	1.1	-0.130	-1.5
11/10/2017 2:17 AM	29.5	28.5	27.1	-0.3	1	-0.126	-1.5
11/10/2017 2:18 AM	29.5	27.6	27.4	-0.3	0.6	-0.118	-1.5

11/10/2017 2:19 AM	29.4	28.8	26.8	-0.3	1.9	-0.130	-1.5
11/10/2017 2:20 AM	29.3	28.1	27.1	-0.3	1.6	-0.130	-1.5
11/10/2017 2:21 AM	29	26.5	26.9	-0.3	1.6	-0.122	-1.5
11/10/2017 2:22 AM	29	28.3	26.9	-0.3	0.9	-0.124	-1.5
11/10/2017 2:23 AM	29.1	27.3	27	-0.2	0.8	-0.133	-1.5
11/10/2017 2:24 AM	29.2	28.4	27.1	-0.2	0.7	-0.122	-1.5
11/10/2017 2:25 AM	29.3	29.6	27	-0.1	1.1	-0.118	-1.5
11/10/2017 2:26 AM	29.3	28	27.1	-0.1	1.1	-0.130	-1.5
11/10/2017 2:27 AM	29.3	26.6	27.3	-0.1	0.8	-0.134	-1.5
11/10/2017 2:28 AM	29.3	27.7	26.8	-0.2	0.6	-0.124	-1.5
11/10/2017 2:29 AM	29.3	28.1	27.1	-0.2	1.3	-0.122	-1.5
11/10/2017 2:30 AM	29.2	26.5	27.2	-0.1	1.1	-0.129	-1.5
11/10/2017 2:31 AM	29.2	28.2	27.2	-0.1	0.8	-0.134	-1.5
11/10/2017 2:32 AM	29.2	28.7	26.9	0.1	1.1	-0.120	-1.5
11/10/2017 2:33 AM	29.4	28.3	27.6	0.1	0.3	-0.120	-1.5
11/10/2017 2:34 AM	29.6	27.2	27.6	0	0.2	-0.133	-1.5
11/10/2017 2:35 AM	29.8	28.6	27.3	-0.1	0.4	-0.126	-1.5
11/10/2017 2:36 AM	29.7	28.5	26.9	-0.3	0.9	-0.130	-1.5
11/10/2017 2:37 AM	29.5	27.4	27.2	-0.4	0.7	-0.130	-1.5
11/10/2017 2:38 AM	29.5	27.6	27.2	-0.4	0.3	-0.128	-1.5
11/10/2017 2:39 AM	29.5	29	27.4	-0.5	0.5	-0.130	-1.5
11/10/2017 2:40 AM	29.6	28.3	27.1	-0.5	0.6	-0.127	-1.5
11/10/2017 2:41 AM	29.5	28.8	27.1	-0.5	0.6	-0.121	-1.5
11/10/2017 2:42 AM	29.6	27.4	27.4	-0.5	0.6	-0.122	-1.5
11/10/2017 2:43 AM	29.6	28.3	27	-0.5	1.3	-0.131	-1.5
11/10/2017 2:44 AM	29.5	28	27	-0.5	2.1	-0.123	-1.5
11/10/2017 2:45 AM	29.3	27.1	26.8	-0.5	0.4	-0.125	-1.5
11/10/2017 2:46 AM	29.2	27.8	27	-0.4	0.4	-0.133	-1.5
11/10/2017 2:47 AM	29.4	29.4	27.2	-0.3	0.4	-0.141	-1.5
11/10/2017 2:48 AM	29.6	26.9	27.2	-0.3	0.6	-0.120	-1.5
11/10/2017 2:49 AM	29.8	28.7	27.4	-0.4	0.1	-0.119	-1.5
11/10/2017 2:50 AM	29.7	28.8	27.3	-0.4	0.7	-0.131	-1.5
11/10/2017 2:51 AM	29.6	29.1	27.2	-0.5	0.6	-0.128	-1.5
11/10/2017 2:52 AM	29.7	28.1	27.5	-0.6	0.3	-0.128	-1.5
11/10/2017 2:53 AM	29.9	28.3	27.5	-0.6	0.5	-0.125	-1.5
11/10/2017 2:54 AM	29.8	28.9	27.2	-0.7	0.3	-0.129	-1.5
11/10/2017 2:55 AM	29.7	27.5	27.5	-0.8	0.5	-0.131	-1.5
11/10/2017 2:56 AM	29.6	29.9	27.2	-0.8	0.6	-0.124	-1.5
11/10/2017 2:57 AM	29.4	27.2	27	-0.8	0.6	-0.130	-1.5
11/10/2017 2:58 AM	29.2	28.3	27.1	-0.9	1.1	-0.131	-1.5
11/10/2017 2:59 AM	29.1	26.6	26.9	-0.8	0.4	-0.128	-1.5
11/10/2017 3:00 AM	29	27.2	27.1	-0.8	0.9	-0.121	-1.5
11/10/2017 3:01 AM	29.2	29.1	27.2	-0.8	0.3	-0.127	-1.5
11/10/2017 3:02 AM	29.3	26.6	27.4	-0.8	0.4	-0.129	-1.5
11/10/2017 3:03 AM	29.5	27.7	27.2	-0.8	0.6	-0.117	-1.5
11/10/2017 3:04 AM	29.5	28.1	27.2	-0.8	1	-0.120	-1.5
11/10/2017 3:05 AM	29.4	26.4	27.2	-0.8	0.7	-0.130	-1.5

11/10/2017 3:06 AM	29.4	26.1	27.3	-0.8	0.9	-0.132	-1.5
11/10/2017 3:07 AM	29.5	27.1	27.3	-0.8	0.6	-0.123	-1.5
11/10/2017 3:08 AM	29.6	27.6	27.3	-0.8	0.3	-0.122	-1.5
11/10/2017 3:09 AM	29.6	27.6	27.3	-0.8	0.9	-0.130	-1.5
11/10/2017 3:10 AM	29.6	29.2	27.3	-0.7	0.7	-0.130	-1.5
11/10/2017 3:11 AM	29.6	28.9	27.3	-0.7	0.7	-0.118	-1.5
11/10/2017 3:12 AM	29.6	28.2	27.4	-0.7	0.6	-0.125	-1.5
11/10/2017 3:13 AM	29.5	28.7	26.9	-0.7	0.5	-0.132	-1.5
11/10/2017 3:14 AM	29.5	27.9	27.1	-0.7	1.1	-0.124	-1.5
11/10/2017 3:15 AM	29.5	27.7	27.2	-0.5	0.7	-0.116	-1.5
11/10/2017 3:16 AM	29.7	28.5	27.4	-0.4	0.2	-0.126	-1.5
11/10/2017 3:17 AM	29.7	28.3	27.3	-0.3	0.4	-0.128	-1.5
11/10/2017 3:18 AM	29.8	28	27.2	-0.3	0.9	-0.121	-1.5
11/10/2017 3:19 AM	29.6	27.8	27.2	-0.4	1.2	-0.113	-1.5
11/10/2017 3:20 AM	29.3	28.7	26.8	-0.4	0.9	-0.128	-1.5
11/10/2017 3:21 AM	29.3	28.1	27.2	-0.4	0.5	-0.136	-1.5
11/10/2017 3:22 AM	29.6	27.3	27.3	-0.4	0.5	-0.123	-1.5
11/10/2017 3:23 AM	29.7	29.3	27.5	-0.5	0.5	-0.134	-1.5
11/10/2017 3:24 AM	29.8	28.2	27.3	-0.5	0.9	-0.132	-1.5
11/10/2017 3:25 AM	29.8	26.9	27.4	-0.5	0.5	-0.124	-1.5
11/10/2017 3:26 AM	29.7	27.4	27.3	-0.5	0.4	-0.115	-1.5
11/10/2017 3:27 AM	29.6	27.9	27.4	-0.6	0.7	-0.128	-1.5
11/10/2017 3:28 AM	29.6	27.2	27.4	-0.6	0.3	-0.125	-1.5
11/10/2017 3:29 AM	29.7	26.5	27.2	-0.6	0.4	-0.124	-1.5
11/10/2017 3:30 AM	29.6	25.8	27.3	-0.6	0.6	-0.120	-1.5
11/10/2017 3:31 AM	29.7	27.6	27.1	-0.6	0.7	-0.126	-1.5
11/10/2017 3:32 AM	29.5	28.1	26.9	-0.6	0.4	-0.132	-1.5
11/10/2017 3:33 AM	29.4	28.1	26.9	-0.5	0.6	-0.119	-1.5
11/10/2017 3:34 AM	29.4	28.3	27.1	-0.4	0.6	-0.113	-1.5
11/10/2017 3:35 AM	29.5	27.4	27	-0.4	0.8	-0.133	-1.5
11/10/2017 3:36 AM	29.5	26.8	27	-0.4	1	-0.128	-1.5
11/10/2017 3:37 AM	29.3	26.7	27.1	-0.4	0.9	-0.120	-1.5
11/10/2017 3:38 AM	29.2	27	26.8	-0.3	0.5	-0.126	-1.5
11/10/2017 3:39 AM	29.2	28.2	27.1	-0.3	0.3	-0.123	-1.5
11/10/2017 3:40 AM	29.2	27.7	26.6	-0.3	1.1	-0.115	-1.5
11/10/2017 3:41 AM	29.1	28.1	26.7	-0.2	1	-0.121	-1.5
11/10/2017 3:42 AM	29	27.8	27	-0.1	0.7	-0.124	-1.5
11/10/2017 3:43 AM	28.9	25.9	26.5	-0.1	0.8	-0.126	-1.5
11/10/2017 3:44 AM	29.1	27.3	27	-0.1	0.4	-0.118	-1.5
11/10/2017 3:45 AM	29.3	27.4	27.1	-0.1	0.5	-0.123	-1.5
11/10/2017 3:46 AM	29.4	28.8	27	-0.1	0.4	-0.133	-1.5
11/10/2017 3:47 AM	29.5	27.2	27	-0.2	0.7	-0.119	-1.5
11/10/2017 3:48 AM	29.4	26.8	27.1	-0.2	0.5	-0.130	-1.5
11/10/2017 3:49 AM	29.2	27.6	26.8	-0.2	0.7	-0.131	-1.5
11/10/2017 3:50 AM	29.2	27.3	27.1	-0.2	0.7	-0.126	-1.5
11/10/2017 3:51 AM	29.2	27.7	26.6	-0.2	0.8	-0.120	-1.5
11/10/2017 3:52 AM	29.2	27.3	26.8	-0.2	0.7	-0.127	-1.5

11/10/2017 3:53 AM	29.1	28.7	27	-0.2	0.5	-0.128	-1.5
11/10/2017 3:54 AM	29.1	27.5	26.5	-0.2	1.1	-0.125	-1.5
11/10/2017 3:55 AM	29	29.2	26.9	-0.2	0.5	-0.122	-1.5
11/10/2017 3:56 AM	29.1	28.2	26.9	-0.3	0.2	-0.133	-1.5
11/10/2017 3:57 AM	29.2	27.3	27.1	-0.3	0.3	-0.126	-1.5
11/10/2017 3:58 AM	29.4	27.8	27.1	-0.3	0.4	-0.122	-1.5
11/10/2017 3:59 AM	29.4	28.1	26.8	-0.3	0.4	-0.121	-1.5
11/10/2017 4:00 AM	29.5	26.9	27.1	-0.4	0.6	-0.131	-1.5
11/10/2017 4:01 AM	29.5	26.8	26.9	-0.4	0.5	-0.129	-1.5
11/10/2017 4:02 AM	29.5	28.6	27.2	-0.4	0.5	-0.122	-1.5
11/10/2017 4:03 AM	29.5	27.1	27.1	-0.4	0.3	-0.123	-1.5
11/10/2017 4:04 AM	29.5	29	26.8	-0.4	1.5	-0.133	-1.5
11/10/2017 4:05 AM	29.5	27.2	27	-0.3	0.8	-0.122	-1.5
11/10/2017 4:06 AM	29.2	26	26.7	-0.3	2	-0.119	-1.5
11/10/2017 4:07 AM	29	26.2	26.7	-0.3	0.9	-0.132	-1.5
11/10/2017 4:08 AM	29	27.8	26.8	-0.2	0.5	-0.121	-1.5
11/10/2017 4:09 AM	29.2	27.6	26.9	-0.2	0.7	-0.124	-1.5
11/10/2017 4:10 AM	29.3	26.8	26.6	-0.1	0.6	-0.112	-1.5
11/10/2017 4:11 AM	29.3	28	26.8	-0.1	0.5	-0.126	-1.5
11/10/2017 4:12 AM	29.2	27.2	26.9	-0.1	1	-0.129	-1.5
11/10/2017 4:13 AM	29.1	25.8	26.7	-0.1	1.8	-0.122	-1.5
11/10/2017 4:14 AM	29	28.3	26.7	0.3	1.2	-0.122	-1.5
11/10/2017 4:15 AM	28.9	29.8	26.8	0.5	0.7	-0.130	-1.5
11/10/2017 4:16 AM	29.1	27.1	27	0.6	0.5	-0.120	-1.5
11/10/2017 4:17 AM	29.1	26.8	27.1	0.5	0.3	-0.120	-1.5
11/10/2017 4:18 AM	29.2	26.8	26.8	0.4	1	-0.125	-1.5
11/10/2017 4:19 AM	29.1	28	27	0.2	0.9	-0.133	-1.5
11/10/2017 4:20 AM	29.1	27.7	27.2	0.1	0.6	-0.127	-1.5
11/10/2017 4:21 AM	29.2	27.3	26.8	0.1	0.5	-0.120	-1.5
11/10/2017 4:22 AM	29.2	27.9	26.6	0	0.4	-0.121	-1.5
11/10/2017 4:23 AM	29.3	28.3	26.8	0	0.2	-0.134	-1.5
11/10/2017 4:24 AM	29.3	28.7	26.7	-0.1	0.7	-0.119	-1.5
11/10/2017 4:25 AM	29.2	28.2	27	-0.1	0.7	-0.123	-1.5
11/10/2017 4:26 AM	29.1	24.5	27	-0.2	0.7	-0.135	-1.5
11/10/2017 4:27 AM	29	26.3	26.9	-0.2	0.8	-0.127	-1.5
11/10/2017 4:28 AM	29	27.3	26.6	-0.2	0.8	-0.124	-1.5
11/10/2017 4:29 AM	29	26.6	26.8	0	1	-0.128	-1.5
11/10/2017 4:30 AM	29	27	26.6	0	0.4	-0.134	-1.5
11/10/2017 4:31 AM	29	26.7	26.4	0	1.2	-0.124	-1.5
11/10/2017 4:32 AM	29	26.9	26.8	0	0.5	-0.120	-1.5
11/10/2017 4:33 AM	29	27.5	26.6	0	0.6	-0.131	-1.5
11/10/2017 4:34 AM	29	27.8	26.6	0	0.3	-0.126	-1.5
11/10/2017 4:35 AM	28.8	27.6	26.2	-0.1	1.4	-0.105	-1.5
11/10/2017 4:36 AM	28.6	27.7	26.4	0	1.1	-0.128	-1.5
11/10/2017 4:37 AM	28.7	26.7	26.6	0.3	0.3	-0.130	-1.5
11/10/2017 4:38 AM	28.9	27.6	26.6	0.4	0.5	-0.121	-1.5
11/10/2017 4:39 AM	29.1	26.2	26.3	0.4	0.7	-0.116	-1.5

11/10/2017 4:40 AM	28.8	26.1	26.3	0.3	1.5	-0.124	-1.5
11/10/2017 4:41 AM	28.1	25.2	24.8	0.2	6.2	-0.128	-1.5
11/10/2017 4:42 AM	27.7	25.9	26.4	0.3	1.9	-0.123	-1.5
11/10/2017 4:43 AM	27.9	26.9	26.5	0.4	0.4	-0.124	-1.5
11/10/2017 4:44 AM	28.5	27.1	26.7	0.5	1.1	-0.131	-1.5
11/10/2017 4:45 AM	28.9	26.8	26.6	0.5	1	-0.130	-1.5
11/10/2017 4:46 AM	29	27.2	26.6	0.5	0.6	-0.124	-1.5
11/10/2017 4:47 AM	29	27.6	26.7	0.4	0.6	-0.121	-1.5
11/10/2017 4:48 AM	29.2	27.3	26.6	0.3	0.4	-0.134	-1.5
11/10/2017 4:49 AM	29.3	28.1	26.6	0.2	0.3	-0.126	-1.5
11/10/2017 4:50 AM	29.2	27.2	26.6	0.1	0.6	-0.119	-1.5
11/10/2017 4:51 AM	29.1	28	26.7	0.1	1	-0.131	-1.5
11/10/2017 4:52 AM	29.2	27.8	26.7	0	0.7	-0.131	-1.5
11/10/2017 4:53 AM	29.2	28.3	27.1	0	0.8	-0.125	-1.5
11/10/2017 4:54 AM	29.2	28.9	26.8	0	0.6	-0.124	-1.5
11/10/2017 4:55 AM	29.1	27.3	26.7	-0.1	0.6	-0.130	-1.5
11/10/2017 4:56 AM	29	25.3	26.3	-0.1	0.9	-0.125	-1.5
11/10/2017 4:57 AM	28.8	26.8	25.9	-0.1	2.7	-0.118	-1.5
11/10/2017 4:58 AM	28.4	25.3	25.5	0	2.8	-0.125	-1.5
11/10/2017 4:59 AM	28.2	26.4	26.2	0.1	1.2	-0.130	-1.5
11/10/2017 5:00 AM	28.3	28	26.7	0.3	0.8	-0.127	-1.5
11/10/2017 5:01 AM	28.7	27.8	26.6	0.3	0.8	-0.120	-1.5
11/10/2017 5:02 AM	28.9	27.3	26.6	0.4	0.8	-0.124	-1.5
11/10/2017 5:03 AM	29	28.3	26.4	0.4	1.6	-0.134	-1.5
11/10/2017 5:04 AM	28.8	27.1	26.3	0.4	1.5	-0.129	-1.5
11/10/2017 5:05 AM	28.5	26	26.1	0.4	1.3	-0.125	-1.5
11/10/2017 5:06 AM	28.4	27.6	26.5	0.4	0.6	-0.126	-1.5
11/10/2017 5:07 AM	28.5	27.3	26.4	0.4	0.5	-0.131	-1.5
11/10/2017 5:08 AM	28.7	28	26.7	0.4	0.5	-0.126	-1.5
11/10/2017 5:09 AM	28.9	28.3	26.5	0.3	0.8	-0.123	-1.5
11/10/2017 5:10 AM	29	28	26.7	0.3	0.2	-0.127	-1.5
11/10/2017 5:11 AM	29.1	26.9	26.8	0.3	0.5	-0.136	-1.5
11/10/2017 5:12 AM	29.1	27.9	26.6	0.2	0.4	-0.123	-1.5
11/10/2017 5:13 AM	29.1	27.6	26.8	0.2	0.3	-0.126	-1.5
11/10/2017 5:14 AM	29.2	26.9	26.7	0.2	0.3	-0.132	-1.5
11/10/2017 5:15 AM	29.2	28.6	26.2	0.1	0.4	-0.127	-1.5
11/10/2017 5:16 AM	29.2	27.5	26.7	0.1	0.7	-0.122	-1.5
11/10/2017 5:17 AM	29	26.6	26.6	0.1	0.5	-0.134	-1.5
11/10/2017 5:18 AM	29	25.7	26.4	0.1	0.6	-0.132	-1.5
11/10/2017 5:19 AM	29	27.5	26.3	0.1	0.6	-0.123	-1.5
11/10/2017 5:20 AM	28.8	25.8	25.9	0	1.2	-0.124	-1.5
11/10/2017 5:21 AM	28.6	26.5	26.2	0.1	0.4	-0.137	-1.5
11/10/2017 5:22 AM	28.5	26.9	26.4	0.2	0.5	-0.134	-1.5
11/10/2017 5:23 AM	28.8	27.6	26.1	0.2	0.6	-0.117	-1.5
11/10/2017 5:24 AM	28.9	26.3	26.7	0.2	0.3	-0.126	-1.5
11/10/2017 5:25 AM	28.8	27	26.3	0.2	0.6	-0.132	-1.5
11/10/2017 5:26 AM	28.7	27.7	26.4	0.1	0.4	-0.133	-1.5

11/10/2017 5:27 AM	28.7	27.1	26.3	0.1	0.5	-0.119	-1.5
11/10/2017 5:28 AM	28.8	27.8	26.2	0	1.3	-0.128	-1.5
11/10/2017 5:29 AM	28.7	26.3	26.4	0	0.4	-0.132	-1.5
11/10/2017 5:30 AM	28.6	27.9	26.3	0	0.9	-0.133	-1.5
11/10/2017 5:31 AM	28.6	26.6	25.9	0	1.5	-0.127	-1.5
11/10/2017 5:32 AM	28.5	27.7	26.3	0	1	-0.136	-1.5
11/10/2017 5:33 AM	28.4	25.9	26	0	0.8	-0.141	-1.5
11/10/2017 5:34 AM	28.3	25.9	25.8	0	0.3	-0.117	-1.5
11/10/2017 5:35 AM	28.4	27.5	26.1	0	0.7	-0.129	-1.5
11/10/2017 5:36 AM	28.5	27.9	26.2	-0.1	0.8	-0.138	-1.5
11/10/2017 5:37 AM	28.7	26.9	26.1	0	1	-0.122	-1.5
11/10/2017 5:38 AM	28.8	26.9	26.5	0	0.3	-0.125	-1.5
11/10/2017 5:39 AM	28.9	26.1	26.4	0	0.4	-0.137	-1.5
11/10/2017 5:40 AM	28.9	26.4	26.5	-0.1	0.7	-0.132	-1.5
11/10/2017 5:41 AM	28.8	26.9	26.3	-0.1	0.3	-0.128	-1.5
11/10/2017 5:42 AM	28.7	26.3	26.3	-0.1	0.2	-0.130	-1.5
11/10/2017 5:43 AM	28.6	26.3	26.2	-0.2	0.5	-0.132	-1.5
11/10/2017 5:44 AM	28.5	27.4	25.9	-0.2	0.4	-0.136	-1.5
11/10/2017 5:45 AM	28.4	27.2	26.2	-0.2	0.5	-0.129	-1.5
11/10/2017 5:46 AM	28.4	25.7	26.2	-0.2	0.3	-0.123	-1.5
11/10/2017 5:47 AM	28.3	27.2	26.2	-0.2	0.7	-0.133	-1.5
11/10/2017 5:48 AM	28.2	25.3	25.7	-0.2	1.4	-0.130	-1.5
11/10/2017 5:49 AM	27.9	26.3	25.5	-0.2	1.5	-0.129	-1.5
11/10/2017 5:50 AM	27.9	25.6	26.1	-0.1	0.9	-0.127	-1.5
11/10/2017 5:51 AM	28.1	25.8	26	0	0.9	-0.134	-1.5
11/10/2017 5:52 AM	28.3	25	26.1	0	0.3	-0.138	-1.5
11/10/2017 5:53 AM	28.4	26.3	26	0	0.7	-0.128	-1.5
11/10/2017 5:54 AM	28.4	26.7	26.1	0	0.6	-0.124	-1.5
11/10/2017 5:55 AM	28.4	27.6	26.3	0	0.8	-0.142	-1.5
11/10/2017 5:56 AM	28.4	24.3	26.1	0	0.6	-0.133	-1.5
11/10/2017 5:57 AM	28.5	25.2	26.3	0	0.4	-0.124	-1.5
11/10/2017 5:58 AM	28.5	26.8	26.3	0	0.5	-0.132	-1.5
11/10/2017 5:59 AM	28.5	26.1	26.2	-0.1	0.5	-0.133	-1.5
11/10/2017 6:00 AM	28.4	26.6	26.3	-0.1	0.8	-0.132	-1.5
11/10/2017 6:01 AM	28.4	26.3	26	-0.1	0.8	-0.120	-1.5
11/10/2017 6:02 AM	28.3	25.8	25.8	-0.1	1.5	-0.136	-1.5
11/10/2017 6:03 AM	28.2	26.2	25.6	-0.1	1.8	-0.136	-1.5
11/10/2017 6:04 AM	28.1	25	25.2	0	2.6	-0.129	-1.5
11/10/2017 6:05 AM	27.4	25.3	24.3	0.1	6.4	-0.125	-1.5
11/10/2017 6:06 AM	27.1	25.2	24.9	0.4	3.7	-0.132	-1.5
11/10/2017 6:07 AM	26.7	23.5	24.6	0.6	5	-0.135	-1.5
11/10/2017 6:08 AM	27.1	27	25.5	0.7	1.7	-0.128	-1.5
11/10/2017 6:09 AM	27.2	26	25.3	0.8	0.7	-0.133	-1.5
11/10/2017 6:10 AM	27.6	25.5	25.6	1	0.7	-0.127	-1.5
11/10/2017 6:11 AM	27.9	26.1	25.9	1	0.5	-0.130	-1.5
11/10/2017 6:12 AM	28.4	28.1	26	1	0.4	-0.129	-1.5
11/10/2017 6:13 AM	28.6	25.7	25.9	0.9	0.6	-0.128	-1.5

11/10/2017 6:14 AM	28.4	25.2	25.7	0.8	1	-0.133	-1.5
11/10/2017 6:15 AM	28.2	26.8	26	0.7	0.3	-0.134	-1.5
11/10/2017 6:16 AM	28.2	25.2	26	0.6	0.3	-0.124	-1.5
11/10/2017 6:17 AM	28.3	26.5	25.8	0.5	0.7	-0.134	-1.5
11/10/2017 6:18 AM	28.3	25.5	25.9	0.5	0.5	-0.147	-1.5
11/10/2017 6:19 AM	28.3	27.6	26.1	0.4	0.6	-0.124	-1.5
11/10/2017 6:20 AM	28.5	25.5	26.2	0.3	0.8	-0.130	-1.5
11/10/2017 6:21 AM	28.5	27.3	26	0.2	0.6	-0.131	-1.5
11/10/2017 6:22 AM	28.4	26.4	25.8	0.2	0.5	-0.141	-1.5
11/10/2017 6:23 AM	28.2	26.8	26.1	0.2	0.4	-0.129	-1.5
11/10/2017 6:24 AM	28.1	26.2	25.5	0.1	1	-0.132	-1.5
11/10/2017 6:25 AM	28	26.5	25.7	0.1	0.7	-0.134	-1.5
11/10/2017 6:26 AM	28	24.5	25.6	0.3	0.3	-0.140	-1.5
11/10/2017 6:27 AM	27.9	26.3	25.6	0.3	0.4	-0.129	-1.5
11/10/2017 6:28 AM	28	26.6	25.8	0.3	0.3	-0.124	-1.5
11/10/2017 6:29 AM	27.9	24.7	25.4	0.3	1	-0.136	-1.5
11/10/2017 6:30 AM	27.8	24.4	25.2	0.2	1	-0.132	-1.5
11/10/2017 6:31 AM	27.6	24.8	25.4	0.1	0.7	-0.129	-1.5
11/10/2017 6:32 AM	27.6	25	25.6	0.1	0.7	-0.131	-1.5
11/10/2017 6:33 AM	27.5	26.7	25.2	0.2	2.3	-0.138	-1.5
11/10/2017 6:34 AM	27.3	24.8	24.9	0.2	3.7	-0.131	-1.5
11/10/2017 6:35 AM	27	25.9	24.9	0.2	1.2	-0.125	-1.5
11/10/2017 6:36 AM	27.1	25.8	25.4	0.3	0.7	-0.138	-1.5
11/10/2017 6:37 AM	27.4	25.5	25.3	0.7	1	-0.136	-1.5
11/10/2017 6:38 AM	27.6	25.5	25.5	0.6	0.3	-0.132	-1.5
11/10/2017 6:39 AM	27.7	27.5	25.2	0.5	0.9	-0.134	-1.5
11/10/2017 6:40 AM	27.8	25.2	25.4	0.4	0.6	-0.137	-1.5
11/10/2017 6:41 AM	27.8	27	25.6	0.4	0.3	-0.129	-1.5
11/10/2017 6:42 AM	27.8	25.7	25.4	0.3	0.5	-0.135	-1.5
11/10/2017 6:43 AM	27.7	25.2	25.6	0.2	0.8	-0.137	-1.5
11/10/2017 6:44 AM	27.8	26.8	25.7	0.3	0.4	-0.131	-1.5
11/10/2017 6:45 AM	27.8	27.2	25.6	0.3	0.4	-0.127	-1.5
11/10/2017 6:46 AM	27.8	25.6	25.4	0.2	0.6	-0.134	-1.5
11/10/2017 6:47 AM	27.7	27.2	25.6	0.2	0.5	-0.138	-1.5
11/10/2017 6:48 AM	27.6	26.3	25.4	0.2	0.5	-0.131	-1.5
11/10/2017 6:49 AM	27.6	25.7	25.5	0.1	0.4	-0.136	-1.5
11/10/2017 6:50 AM	27.6	25.7	25.7	0.1	0.8	-0.133	-1.5
11/10/2017 6:51 AM	27.7	25.8	25.3	0	0.9	-0.139	-1.5
11/10/2017 6:52 AM	27.5	25.4	25	0	0.9	-0.135	-1.5
11/10/2017 6:53 AM	27.3	26.2	25.2	0.1	0.6	-0.129	-1.5
11/10/2017 6:54 AM	27.1	26.3	25.2	0.1	0.4	-0.136	-1.5
11/10/2017 6:55 AM	27.1	25.6	25.1	0.1	0.8	-0.136	-1.5
11/10/2017 6:56 AM	27.3	24.9	25.5	0	1	-0.132	-1.5
11/10/2017 6:57 AM	27.3	25.6	25.1	0	0.4	-0.130	-1.5
11/10/2017 6:58 AM	27.4	25.3	25.2	0	0.5	-0.139	-1.5
11/10/2017 6:59 AM	27.4	26.8	24.7	0.1	1.2	-0.140	-1.5
11/10/2017 7:00 AM	27.3	24.1	24.9	0.1	1	-0.131	-1.5

11/10/2017 7:01 AM	27.3	24.6	25.1	0.1	0.4	-0.129	-1.5
11/10/2017 7:02 AM	27.4	25.2	24.9	0.1	0.6	-0.139	-1.5
11/10/2017 7:03 AM	27.4	25.6	24.8	0.1	0.9	-0.133	-1.5
11/10/2017 7:04 AM	27.2	26.2	24.9	0.2	0.9	-0.122	-1.5
11/10/2017 7:05 AM	27.3	26.4	25.2	0.3	0.5	-0.129	-1.5
11/10/2017 7:06 AM	27.4	26.2	25	0.3	0.6	-0.137	-1.5
11/10/2017 7:07 AM	27.5	25.4	25.2	0.3	1.5	-0.132	-1.5
11/10/2017 7:08 AM	27.5	26.2	25.1	0.3	0.6	-0.129	-1.5
11/10/2017 7:09 AM	27.4	25.9	25.4	0.3	0.7	-0.143	-1.5
11/10/2017 7:10 AM	27.6	24.9	25.1	0.3	0.6	-0.138	-1.5
11/10/2017 7:11 AM	27.6	27	25.2	0.3	0.7	-0.133	-1.5
11/10/2017 7:12 AM	27.7	26.1	25.3	0.3	0.5	-0.128	-1.5
11/10/2017 7:13 AM	27.4	24.1	24.1	0.5	4.7	-0.130	-1.5
11/10/2017 7:14 AM	26.9	25.4	24.7	0.5	2.7	-0.139	-1.5
11/10/2017 7:15 AM	26.7	24.6	25.3	0.7	0.9	-0.132	-1.5
11/10/2017 7:16 AM	26.9	24.8	25.3	0.7	0.5	-0.131	-1.5
11/10/2017 7:17 AM	27.6	25.6	25.6	0.7	0.6	-0.130	-1.5
11/10/2017 7:18 AM	27.8	25.5	25.3	0.7	1.6	-0.135	-1.5
11/10/2017 7:19 AM	27.8	26.9	25.7	0.7	0.5	-0.139	-1.5
11/10/2017 7:20 AM	27.9	25.4	25.4	0.7	0.4	-0.127	-1.5
11/10/2017 7:21 AM	27.9	25.8	25.7	0.7	0.3	-0.130	-1.5
11/10/2017 7:22 AM	27.8	26.4	25.4	0.7	0.5	-0.141	-1.5
11/10/2017 7:23 AM	27.8	24.6	25.6	0.7	0.5	-0.134	-1.5
11/10/2017 7:24 AM	27.7	25.5	25.5	0.8	0.6	-0.130	-1.5
11/10/2017 7:25 AM	27.8	25.1	25.7	0.8	0.4	-0.138	-1.5
11/10/2017 7:26 AM	27.8	25.5	25.3	0.7	0.8	-0.141	-1.5
11/10/2017 7:27 AM	27.8	25.7	25.7	0.7	0.7	-0.131	-1.5
11/10/2017 7:28 AM	27.8	25.8	25.5	0.7	0.8	-0.131	-1.5
11/10/2017 7:29 AM	27.8	25.7	25.8	0.6	0.7	-0.141	-1.5
11/10/2017 7:30 AM	27.8	26.4	25.6	0.6	0.9	-0.134	-1.5
11/10/2017 7:31 AM	27.8	27.2	25.4	0.6	1.4	-0.129	-1.5
11/10/2017 7:32 AM	27.7	25	25.6	0.6	1.9	-0.136	-1.5
11/10/2017 7:33 AM	27.6	24.8	25.2	0.6	2.4	-0.142	-1.5
11/10/2017 7:34 AM	27.2	24.2	24.7	0.7	5.6	-0.138	-1.5
11/10/2017 7:35 AM	27	27.2	25.2	0.7	1.7	-0.130	-1.5
11/10/2017 7:36 AM	27.1	26.3	24.9	0.8	1.5	-0.134	-1.5
11/10/2017 7:37 AM	27.4	25.5	25.3	0.8	2.7	-0.137	-1.5
11/10/2017 7:38 AM	27.3	24.9	24.8	0.7	5.4	-0.128	-1.5
11/10/2017 7:39 AM	27	25.6	24.5	0.8	5.4	-0.133	-1.5
11/10/2017 7:40 AM	26.6	24.7	24.7	1	3.6	-0.140	-1.5
11/10/2017 7:41 AM	26.5	25.1	25.1	1.2	2.4	-0.135	-1.5
11/10/2017 7:42 AM	26.6	25	24.7	1.4	2.3	-0.127	-1.5
11/10/2017 7:43 AM	27	25.2	24.9	1.5	2.8	-0.134	-1.5
11/10/2017 7:44 AM	27	24.3	24.8	1.6	3.2	-0.139	-1.5
11/10/2017 7:45 AM	27	26.4	24.9	1.7	2.3	-0.134	-1.5
11/10/2017 7:46 AM	27.1	26.8	25.3	1.8	0.6	-0.129	-1.5
11/10/2017 7:47 AM	27.3	26.4	25.4	1.8	0.9	-0.133	-1.5

11/10/2017 7:48 AM	27.5	25.4	25.2	1.8	0.8	-0.139	-1.5
11/10/2017 7:49 AM	27.4	24.4	24.5	1.8	5.4	-0.130	-1.5
11/10/2017 7:50 AM	27.2	24.8	25.2	1.7	4.8	-0.128	-1.5
11/10/2017 7:51 AM	26.9	24.4	24.3	1.7	5.7	-0.136	-1.5
11/10/2017 7:52 AM	26.4	24	24.2	1.7	6.2	-0.142	-1.5
11/10/2017 7:53 AM	26.3	26.4	24.6	1.8	1.9	-0.134	-1.5
11/10/2017 7:54 AM	26.3	23.8	24.6	1.9	3.2	-0.133	-1.5
11/10/2017 7:55 AM	26.9	25.7	25.3	2.1	1	-0.142	-1.5
11/10/2017 7:56 AM	27.3	24.9	24.9	2.2	2.1	-0.141	-1.5
11/10/2017 7:57 AM	27.1	24.6	24.1	2.2	5.6	-0.131	-1.5
11/10/2017 7:58 AM	26.7	25.6	24.4	2.2	5.3	-0.135	-1.5
11/10/2017 7:59 AM	26.5	24.8	25.1	2.2	2.3	-0.142	-1.5
11/10/2017 8:00 AM	26.7	25.6	25.1	2.2	1.3	-0.139	-1.5
11/10/2017 8:01 AM	27.2	26.5	24.7	2.2	2.2	-0.128	-1.5
11/10/2017 8:02 AM	27.2	26.6	25.5	2.3	2.1	-0.133	-1.5
11/10/2017 8:03 AM	27.5	25.1	25.8	2.3	1	-0.144	-1.5
11/10/2017 8:04 AM	27.6	26.3	25.7	2.2	1.3	-0.136	-1.5
11/10/2017 8:05 AM	27.8	25.4	25.2	2.1	2.2	-0.129	-1.5
11/10/2017 8:06 AM	27.7	28.1	25.3	2.1	1.4	-0.138	-1.5
11/10/2017 8:07 AM	27.7	27.6	25.5	2	0.6	-0.140	-1.5
11/10/2017 8:08 AM	27.7	25.1	25.2	1.9	2.6	-0.137	-1.5
11/10/2017 8:09 AM	27.5	24	25.3	1.8	6.7	-0.136	-1.5
11/10/2017 8:10 AM	27.3	26.5	25.3	1.8	2.9	-0.139	-1.5
11/10/2017 8:11 AM	27.2	26.4	25.3	1.9	2.2	-0.142	-1.5
11/10/2017 8:12 AM	27.4	25.9	25.8	1.9	0.6	-0.134	-1.5
11/10/2017 8:13 AM	27.8	26.6	25.8	1.9	1.4	-0.131	-1.5
11/10/2017 8:14 AM	28.1	25.3	25.8	1.9	0.9	-0.143	-1.5
11/10/2017 8:15 AM	28.2	25.8	25.9	1.9	1.1	-0.138	-1.5
11/10/2017 8:16 AM	28	25	24.7	1.8	6.7	-0.135	-1.5
11/10/2017 8:17 AM	27.5	25.3	24.7	1.7	7	-0.143	-1.5
11/10/2017 8:18 AM	26.6	25.7	23.8	1.7	8.5	-0.146	-1.5
11/10/2017 8:19 AM	26.3	25.4	25	1.8	4.2	-0.137	-1.5
11/10/2017 8:20 AM	26.1	24.2	23.8	1.9	6.3	-0.134	-1.5
11/10/2017 8:21 AM	25.6	22.9	23.7	2.1	9.1	-0.142	-1.5
11/10/2017 8:22 AM	25.5	24.2	25.2	2.3	2.2	-0.142	-1.5
11/10/2017 8:23 AM	25.5	21.7	23.7	2.3	9.2	-0.145	-1.5
11/10/2017 8:24 AM	25.2	21.7	20.5	2.1	15.9	-0.135	-1.5
11/10/2017 8:25 AM	24.7	24.3	18.1	1.7	4.6	-0.144	-1.5
11/10/2017 8:26 AM	24.4	24	17	0.4	7.6	-0.143	-1.5
11/10/2017 8:27 AM	25.1	24.4	16.7	-0.9	5.4	-0.141	-1.5
11/10/2017 8:28 AM	25.2	23.1	15.8	-1.9	6.6	-0.141	-1.5
11/10/2017 8:29 AM	25.4	22.8	16.5	-2.5	4.4	-0.151	-1.5
11/10/2017 8:30 AM	25.3	24.2	17	-2.8	7.9	-0.142	-1.5
11/10/2017 8:31 AM	25.2	24.3	16.2	-2.9	9.3	-0.138	-1.5
11/10/2017 8:32 AM	25	23.6	15.9	-3	12.3	-0.157	-1.5
11/10/2017 8:33 AM	24.6	23.9	16.2	-0.9	10	-0.144	39.4
11/10/2017 8:34 AM	24.9	24.3	16.2	0	10	-0.137	101.5

11/10/2017 8:35 AM	24.7	24.4	21.3	0	8.5	-0.141	79.7
11/10/2017 8:36 AM	25.4	24	23.7	0.2	2.6	-0.144	58.2
11/10/2017 8:37 AM	25.9	25.5	24.6	0.2	5.1	-0.137	40.3
11/10/2017 8:38 AM	26.2	23.5	24.6	0.3	0.8	-0.138	26.9
11/10/2017 8:39 AM	25.4	24.2	24.7	2.1	0.5	-0.147	17.7
11/10/2017 8:40 AM	24	24.1	24.2	2.7	0.7	-0.144	11.8
11/10/2017 8:41 AM	22.8	24.3	23.6	3	0.6	-0.146	7.6
11/10/2017 8:42 AM	21.9	23.8	22.8	3.3	106.6	-0.137	4.8
11/10/2017 8:43 AM	21.6	24.5	24.3	4	96.2	-0.136	3.2
11/10/2017 8:44 AM	21.4	24.2	23.9	4.5	77.6	-0.144	2.7
11/10/2017 8:45 AM	21.3	22.7	23.8	4.7	76.9	-0.134	2.4
11/10/2017 8:46 AM	21.3	24.3	24.4	4.7	76.9	-0.135	2.1
11/10/2017 8:47 AM	21.3	23.1	23.8	4.6	77.4	-0.138	2
11/10/2017 8:48 AM	21.7	24.7	23.7	4.4	79.6	-0.147	1.8
11/10/2017 8:49 AM	21.8	24.1	23.9	4.2	82.4	-0.132	1.8
11/10/2017 8:50 AM	21.3	23	22.9	4	84.8	-0.129	1.7
11/10/2017 8:51 AM	20.9	25.2	23.4	4.1	86.8	-0.142	1.6
11/10/2017 8:52 AM	20.8	25.6	23.8	4.2	89.9	-0.133	1.6
11/10/2017 8:53 AM	21.1	20.9	23.5	4.2	92.3	-0.138	1.6
11/10/2017 8:54 AM	21.5	24.5	24.7	4.2	93.5	-0.140	1.5
11/10/2017 8:55 AM	21.9	23.2	23.9	4.2	95.2	-0.145	1.5
11/10/2017 8:56 AM	21.9	22.8	23	4.5	96.7	-0.144	1.4
11/10/2017 8:57 AM	21.8	22.8	23.5	5.5	97.8	-0.136	1.4
11/10/2017 8:58 AM	21.4	26.7	24.3	6.5	98.1	-0.128	1.4
11/10/2017 8:59 AM	21.7	22.1	24.2	6.9	98.8	-0.142	1.4
11/10/2017 9:00 AM	21.8	23.5	24.2	7.8	99.9	-0.139	1.4
11/10/2017 9:01 AM	22	25.9	24.9	8	87.2	-0.136	1.3
11/10/2017 9:02 AM	22.5	24	24.5	7.4	16.3	-0.138	1.3
11/10/2017 9:03 AM	23.1	24.2	24.9	6.5	21.9	-0.143	1.2
11/10/2017 9:04 AM	23.7	24.3	24.9	5.6	25.3	-0.144	1.2
11/10/2017 9:05 AM	24	23.8	24.6	5.1	28.2	-0.128	1.2
11/10/2017 9:06 AM	23.8	24.9	24.6	5.2	29.5	-0.143	1.1
11/10/2017 9:07 AM	23.6	24.1	24.9	5	30.3	-0.144	1.1
11/10/2017 9:08 AM	23	23.7	24.1	4.6	29.8	-0.135	1.2
11/10/2017 9:09 AM	23.5	25.4	23	6.4	10.9	-0.137	1.2
11/10/2017 9:10 AM	24.5	24.9	21.6	31.3	5.8	-0.148	1.1
11/10/2017 9:11 AM	25.8	25	20.2	53.5	3.3	-0.151	1.1
11/10/2017 9:12 AM	26.6	25.4	19.9	64.2	5.1	-0.145	1.1
11/10/2017 9:13 AM	26.6	24.1	19.6	65.9	4	-0.146	1.1
11/10/2017 9:14 AM	26.5	23.6	19.7	66.5	3.1	-0.153	1.2
11/10/2017 9:15 AM	26.3	23.1	19.5	66.9	9.3	-0.138	1.2
11/10/2017 9:16 AM	26.5	25.1	20	67.1	3.2	-0.141	1.2
11/10/2017 9:17 AM	26.6	23.7	19.8	67	5.9	-0.148	1.3
11/10/2017 9:18 AM	26.4	25.3	20.1	67.1	4.9	-0.147	1.2
11/10/2017 9:19 AM	26.4	26.5	20	71	3.6	-0.139	1.3
11/10/2017 9:20 AM	26.2	25.7	20.2	79.1	4.3	-0.137	1.3
11/10/2017 9:21 AM	26.4	25.7	19.5	56.3	8	-0.145	1.3

11/10/2017 9:22 AM	26.3	25.3	23.3	40.1	4.2	10.892	1.3
11/10/2017 9:23 AM	26.5	26	25.1	27.8	6.1	33.362	1.3
11/10/2017 9:24 AM	27.1	26	25.7	22.4	4.9	33.438	1.2
11/10/2017 9:25 AM	27.2	26.4	25.3	16.4	12.3	33.423	1.2
11/10/2017 9:26 AM	27.5	25.8	26.8	12.3	6.3	33.460	1.2
11/10/2017 9:27 AM	27.8	26.7	26.9	9.7	2.9	33.453	1.1
11/10/2017 9:28 AM	28	25	25	7.9	4.7	30.698	1.1
11/10/2017 9:29 AM	27.6	25.8	25.1	6.7	2.8	4.323	1.1
11/10/2017 9:30 AM	26.7	25.1	24.9	5.9	5.8	0.657	1.1
11/10/2017 9:31 AM	26.7	25.3	26.2	6	3.7	-0.142	1.1
11/10/2017 9:32 AM	27	25.2	25.5	6.2	4.9	-0.144	1.1
11/10/2017 9:33 AM	27.1	24.9	25.1	6	6.9	-0.132	1.1
11/10/2017 9:34 AM	26.7	23.4	24.1	5.5	3.3	-0.142	1.1
11/10/2017 9:35 AM	26.3	23	24.9	4.9	4.4	-0.146	1.1
11/10/2017 9:36 AM	26	22.2	23.7	4.3	4.8	-0.143	1.1
11/10/2017 9:37 AM	25.9	26.3	24.9	3.8	5.6	-0.135	1.1
11/10/2017 9:38 AM	26.1	25.6	25.1	3.7	6.2	-0.143	1.1
11/10/2017 9:39 AM	26.2	23.6	24	3.7	8.7	-0.142	1.1
11/10/2017 9:40 AM	26.5	25.9	25.7	3.3	4.4	-0.137	1.1
11/10/2017 9:41 AM	26.7	27.5	26.9	3.6	5.9	-0.129	1.1
11/10/2017 9:42 AM	27.8	26.9	27.2	3.7	1.7	-0.149	1
11/10/2017 9:43 AM	28.6	26.7	27.2	3.6	2.2	-0.137	1
11/10/2017 9:44 AM	29.1	28.1	27.3	3.4	3.3	-0.133	0.9
11/10/2017 9:45 AM	29.1	27.5	27.4	3.1	3.5	-0.149	0.9
11/10/2017 9:46 AM	29.1	27.2	27.5	2.8	1.9	-0.144	0.8
11/10/2017 9:47 AM	29.3	27.5	27.8	2.6	3.5	-0.140	0.8
11/10/2017 9:48 AM	29.3	30.1	27.6	2.4	2.9	-0.132	0.8
11/10/2017 9:49 AM	29.6	27.8	28	2.3	1.4	-0.145	0.7
11/10/2017 9:50 AM	29.3	27	27.4	2.5	1.8	-0.144	0.7
11/10/2017 9:51 AM	29.2	28.8	27.3	2.9	4.6	-0.138	0.7
11/10/2017 9:52 AM	29	29.4	27.7	3	3.1	-0.135	0.7
11/10/2017 9:53 AM	29	26	27.3	3.1	7	-0.137	0.6
11/10/2017 9:54 AM	29	27.9	28	3.1	3.2	-0.126	0.6
11/10/2017 9:55 AM	28.9	27.5	26.8	3	1.4	-0.132	0.6
11/10/2017 9:56 AM	28.6	23.4	25.2	2.8	6.5	-0.141	0.7
11/10/2017 9:57 AM	28.2	25.3	25.1	2.7	4.7	-0.133	0.7
11/10/2017 9:58 AM	27.7	25	25.2	2.6	3.2	-0.126	0.7
11/10/2017 9:59 AM	27.2	24.3	24	2.6	4	-0.134	0.7
11/10/2017 10:00 AM	27.2	24.9	24.4	2.6	5.2	-0.132	0.7
11/10/2017 10:01 AM	27.2	25	25.1	2.8	3.5	-0.124	0.7
11/10/2017 10:02 AM	27.4	24.6	24.8	3	3.9	-0.129	0.8
11/10/2017 10:03 AM	27.7	26.5	25.8	3	1.5	-0.133	0.8
11/10/2017 10:04 AM	28	25.2	26	2.9	3.2	-0.127	0.8
11/10/2017 10:05 AM	28.1	26.7	25.6	2.8	3.7	-0.121	0.8
11/10/2017 10:06 AM	28	25.2	25.2	2.7	4.5	-0.138	0.8
11/10/2017 10:07 AM	27.6	24.8	24.7	2.7	3.4	-0.136	0.9
11/10/2017 10:08 AM	27.5	26.2	25.7	2.9	3.7	-0.130	0.9

11/10/2017 10:09 AM	27.8	26.5	26.2	3.1	2	-0.132	0.8
11/10/2017 10:10 AM	28.2	25.8	26.5	3.1	2.2	-0.139	0.8
11/10/2017 10:11 AM	28.5	26.3	26.4	3.1	5.5	-0.131	0.8
11/10/2017 10:12 AM	28.6	28.1	26.7	3.8	5.9	-0.132	0.8
11/10/2017 10:13 AM	28.6	24.2	25.3	5.1	4.3	-0.129	0.8
11/10/2017 10:14 AM	28.5	26.9	25.5	5.4	1.6	-0.135	0.8
11/10/2017 10:15 AM	28.3	25.5	24.6	5.3	2.3	-0.131	0.8
11/10/2017 10:16 AM	28	25.2	25.5	4.8	3	-0.124	0.8
11/10/2017 10:17 AM	27.6	24.3	24.6	4.3	7.1	-0.135	0.8
11/10/2017 10:18 AM	27.5	25.2	25.3	4.1	2.4	-0.122	0.8
11/10/2017 10:19 AM	27.5	24.2	24.6	4.2	5.7	-0.123	0.8
11/10/2017 10:20 AM	27.3	26.1	25.3	4.2	4.7	-0.135	0.9
11/10/2017 10:21 AM	27.1	24	24.1	4	3	-0.132	0.9
11/10/2017 10:22 AM	26.7	23.6	24.2	4.1	7.6	-0.122	0.9
11/10/2017 10:23 AM	26.7	24.5	24	4.5	12.2	-0.131	0.9
11/10/2017 10:24 AM	26.1	24.2	22.9	4.5	4.4	-0.107	0.9
11/10/2017 10:25 AM	26	24.8	23.9	4.3	2.5	-0.112	0.9
11/10/2017 10:26 AM	26.1	26.6	24.5	4.1	4.9	-0.122	0.9
11/10/2017 10:27 AM	27.1	28.2	26.5	3.9	1.9	-0.138	0.9
11/10/2017 10:28 AM	28	26.7	25.8	3.6	5.7	-0.132	0.9
11/10/2017 10:29 AM	28.2	25.1	24.9	3.5	10	-0.124	0.9
11/10/2017 10:30 AM	28.1	28.2	25.5	3.4	5.8	-0.129	0.9
11/10/2017 10:31 AM	27.9	27	25.5	3.2	3.8	-0.131	0.9
11/10/2017 10:32 AM	28.3	26.3	26.5	3.2	3.3	-0.122	0.9
11/10/2017 10:33 AM	28.8	26.2	27.1	3.6	1.7	-0.131	0.8
11/10/2017 10:34 AM	29.1	26.1	27	3.8	2.6	-0.134	0.8
11/10/2017 10:35 AM	29.1	27.1	26.8	3.6	3.7	-0.127	0.8
11/10/2017 10:36 AM	28.8	25.4	25.2	3.3	5	-0.122	0.8
11/10/2017 10:37 AM	28.2	26.7	25.2	3.1	4.4	-0.137	0.8
11/10/2017 10:38 AM	27.9	26.1	25.6	3	1.3	-0.132	0.8
11/10/2017 10:39 AM	27.5	24.3	24.6	3	4.7	-0.129	0.8
11/10/2017 10:40 AM	27.7	24.4	25.6	3	1.9	-0.130	0.8
11/10/2017 10:41 AM	27.6	24.3	25	3	6.1	-0.141	0.8
11/10/2017 10:42 AM	27.5	24.3	24.8	3.1	2.9	-0.134	0.9
11/10/2017 10:43 AM	27.6	28.6	26	3.4	3.3	-0.126	0.8
11/10/2017 10:44 AM	27.8	26.4	25.7	3.6	5.6	-0.140	0.8
11/10/2017 10:45 AM	28.1	27	26	3.7	4.5	-0.134	0.8
11/10/2017 10:46 AM	28.1	28	25.4	3.5	7.3	-0.127	0.9
11/10/2017 10:47 AM	28.1	26.6	25.9	3.3	2.7	-0.127	0.9
11/10/2017 10:48 AM	28.4	26.9	25.9	3.1	2.4	-0.142	0.8
11/10/2017 10:49 AM	28.4	26.1	25.7	2.9	2	-0.130	0.8
11/10/2017 10:50 AM	28.1	25.4	26.2	2.8	6.4	-0.127	0.9
11/10/2017 10:51 AM	27.9	25.8	26.8	3.2	5.8	-0.136	0.9
11/10/2017 10:52 AM	28	25.1	26.8	4.5	8.4	-0.131	0.8
11/10/2017 10:53 AM	28.4	25.6	27.4	5.4	2.3	-0.133	1
11/10/2017 10:54 AM	28.7	26.1	27.4	5.4	2.2	-0.138	0.9
11/10/2017 10:55 AM	28.9	26.6	26.5	4.9	3.8	-0.144	1.7

11/10/2017 10:56 AM	28.7	25.3	26.6	4.3	6.6	-0.128	11
11/10/2017 10:57 AM	28.2	23.7	26.7	3.6	9.5	-0.130	14.2
11/10/2017 10:58 AM	28.3	26.6	27.3	3.1	2.6	-0.143	14.3
11/10/2017 10:59 AM	27.9	23.6	24.5	2.7	11.3	0.006	14.3
11/10/2017 11:00 AM	26.6	24.1	26	2.9	10	-0.031	10.2
11/10/2017 11:01 AM	26.8	27.5	27.5	4.2	1.8	-0.133	1
11/10/2017 11:02 AM	27.8	28.5	27.9	5.1	2.1	-0.135	0.6
11/10/2017 11:03 AM	29.8	27.9	27.8	5.3	1.9	-0.125	0.6
11/10/2017 11:04 AM	29.9	27.4	27.9	4.9	1.2	-0.141	0.6
11/10/2017 11:05 AM	29.7	27.3	27.8	4.3	1.8	-0.131	0.6
11/10/2017 11:06 AM	29.8	27.9	28.2	3.7	1.4	-0.129	0.6
11/10/2017 11:07 AM	30.1	29.2	28.4	3.4	0.5	-0.134	0.6
11/10/2017 11:08 AM	30.4	27.4	27.8	3.1	2.4	-0.127	0.6
11/10/2017 11:09 AM	30	30.5	36.2	2.9	7.7	0.787	1.2
11/10/2017 11:10 AM	29.4	28.9	26.9	2.7	4.4	0.577	0.9
11/10/2017 11:11 AM	28.9	27.2	27.1	2.8	4.8	0.523	0.7
11/10/2017 11:12 AM	29.3	28.3	27.4	2.9	5	0.617	0.7
11/10/2017 11:13 AM	29.4	27.4	27.6	3.1	5.8	0.720	0.7
11/10/2017 11:14 AM	29.2	26.6	30.1	3.3	10.9	1.018	1.1
11/10/2017 11:15 AM	29	26.8	26.5	3.6	6.6	0.313	1.1
11/10/2017 11:16 AM	29.3	29.4	27.8	4	3.3	0.488	0.9
11/10/2017 11:17 AM	29.6	28.3	27	4.6	7.1	1.247	0.8
11/10/2017 11:18 AM	29.1	26.1	26	5.2	18.1	-0.045	0.8
11/10/2017 11:19 AM	28.1	28.4	27.1	5.9	10.4	-0.043	0.8
11/10/2017 11:20 AM	27.8	26.5	26.6	6.6	10	0.012	0.8
11/10/2017 11:21 AM	28.3	28.6	28.1	7.1	3.6	-0.115	0.8
11/10/2017 11:22 AM	29.2	26.7	28.2	7.7	3.3	0.866	0.8
11/10/2017 11:23 AM	29.1	25	29.5	7.9	6	2.939	0.8
11/10/2017 11:24 AM	29.1	27.9	28.2	8	3.8	0.284	0.7
11/10/2017 11:25 AM	29.1	28.5	27.7	7.9	1.1	-0.032	0.7
11/10/2017 11:26 AM	29.6	26.3	27.1	7.6	2.7	0.150	0.7
11/10/2017 11:27 AM	28.2	23.1	25	7	22.5	5.054	0.9
11/10/2017 11:28 AM	27.5	28.6	27.2	6.6	2.1	0.489	0.8
11/10/2017 11:29 AM	27.6	27.5	27.7	6.7	1.2	-0.012	0.6
11/10/2017 11:30 AM	29.6	28	27.8	6.6	1.1	0.157	0.6
11/10/2017 11:31 AM	30.6	29.7	28.2	6.4	1	0.297	0.7
11/10/2017 11:32 AM	30.7	27.7	27.7	6	0.9	0.018	0.7
11/10/2017 11:33 AM	30.3	28.2	27.4	5.7	3.8	1.151	0.7
11/10/2017 11:34 AM	30.1	26.3	27.1	5.3	1.3	0.083	1.2
11/10/2017 11:35 AM	29.9	27.7	27.8	5.1	0.6	0.031	1.2
11/10/2017 11:36 AM	30.2	28.1	27.7	4.9	0.8	0.028	1.1
11/10/2017 11:37 AM	30.2	28.2	27.4	4.8	4.4	1.842	1.1
11/10/2017 11:38 AM	30.1	29	27.9	4.8	2.4	0.669	1.2
11/10/2017 11:39 AM	29.9	26.2	27.9	4.9	4.2	1.571	1.2
11/10/2017 11:40 AM	30.3	29.1	27.9	4.9	0.8	0.036	0.8
11/10/2017 11:41 AM	30.5	28.3	27.7	4.9	0.7	0.019	0.7
11/10/2017 11:42 AM	30.8	28.3	28.2	4.5	0.9	0.022	0.7

11/10/2017 11:43 AM	30.9	28.9	27.9	4.2	0.3	-0.106	0.7
11/10/2017 11:44 AM	30.8	28.4	27.6	4.1	0.3	-0.106	0.7
11/10/2017 11:45 AM	30.6	28.3	27.8	4	0.7	0.044	0.9
11/10/2017 11:46 AM	30.4	27.1	27.7	3.9	0.6	-0.115	1.4
11/10/2017 11:47 AM	30.4	27.4	28.5	3.8	0.1	-0.105	1.1
11/10/2017 11:48 AM	30.6	27.9	27.7	3.7	0.2	-0.129	1
11/10/2017 11:49 AM	30.8	28.1	27.8	3.6	0.3	-0.113	0.7
11/10/2017 11:50 AM	31	30	27.6	3.5	0.2	-0.127	3.3
11/10/2017 11:51 AM	31	28.3	28.1	3.4	0.2	-0.130	6.5
11/10/2017 11:52 AM	31.1	29.2	25.5	3.3	0.1	-0.121	9
11/10/2017 11:53 AM	30.7	27.8	24.3	3.2	0.1	0.053	7.3
11/10/2017 11:54 AM	30.6	27.7	26.6	3.1	-0.1	-0.091	8.3
11/10/2017 11:55 AM	30.4	25.4	24.2	2.9	0.3	-0.052	8.4
11/10/2017 11:56 AM	29.1	25.2	25.1	2.9	0.2	-0.033	6.3
11/10/2017 11:57 AM	29	27.7	27.6	2.8	0.4	-0.132	3.1
11/10/2017 11:58 AM	29.3	28.7	27.8	2.6	0.3	-0.136	5.6
11/10/2017 11:59 AM	30.8	28	28.7	2.5	0.4	-0.126	4
11/10/2017 12:00 PM	30.8	28.1	27.8	2.4	0.4	-0.136	3
11/10/2017 12:01 PM	30.5	28.1	28	2.3	0.4	-0.134	2.2
11/10/2017 12:02 PM	30.4	28.4	27.9	2.2	0.2	-0.141	0.9
11/10/2017 12:03 PM	30.5	27.7	27.8	2.1	0.3	-0.142	0.7
11/10/2017 12:04 PM	30.4	27.8	27.4	2	0.1	-0.136	0.7
11/10/2017 12:05 PM	30.3	27.8	27.6	2	0.3	-0.130	0.7
11/10/2017 12:06 PM	30.3	27.6	27.3	2	0.4	-0.129	0.8
11/10/2017 12:07 PM	30.3	27.2	27.7	1.9	0.2	-0.152	0.8
11/10/2017 12:08 PM	30.3	28.8	24.4	1.9	0.5	-0.137	0.7
11/10/2017 12:09 PM	30	29.6	22.1	1.9	0.4	-0.129	0.6
11/10/2017 12:10 PM	29.9	27.3	25.9	2	0.3	-0.137	0.7
11/10/2017 12:11 PM	29.7	26.8	27.2	2	0.3	-0.148	0.8
11/10/2017 12:12 PM	29.8	26.5	26.9	2.1	0.5	-0.143	0.8
11/10/2017 12:13 PM	30	26.4	27.8	2.1	0.3	-0.137	0.7
11/10/2017 12:14 PM	30.2	29.3	27.7	2.1	0.4	-0.155	0.8
11/10/2017 12:15 PM	30.3	28.7	27.5	2.1	0.2	-0.148	0.7
11/10/2017 12:16 PM	30.4	27.9	27.5	2.1	0.2	-0.150	0.6
11/10/2017 12:17 PM	30.3	26.6	27.7	2	0.3	-0.134	0.8
11/10/2017 12:18 PM	30.2	28.3	27.5	2	0.2	-0.145	0.7
11/10/2017 12:19 PM	30.2	28.3	27.8	1.9	0.4	-0.151	0.7
11/10/2017 12:20 PM	30.2	27.7	27.8	1.9	0.4	-0.144	0.6
11/10/2017 12:21 PM	30.4	27.1	28.1	1.9	0.2	-0.145	0.7
11/10/2017 12:22 PM	30.4	28.8	27.3	1.9	0.3	-0.155	0.7
11/10/2017 12:23 PM	30.4	29.3	26.8	1.9	0.4	-0.150	0.8
11/10/2017 12:24 PM	30.1	27.8	27.2	1.9	0.7	-0.150	0.7
11/10/2017 12:25 PM	30	29.2	27.8	2	0.6	-0.149	0.7
11/10/2017 12:26 PM	30.2	28.7	27.8	2	0.3	-0.158	0.7
11/10/2017 12:27 PM	30.5	26.1	27.5	2	0.6	-0.160	0.7
11/10/2017 12:28 PM	30.7	26.6	28.1	2.1	0.4	-0.154	0.7
11/10/2017 12:29 PM	30.6	27.2	27.8	2.1	0.6	-0.154	0.7

11/10/2017 12:30 PM	30.6	28.6	27.8	2.1	0.2	-0.163	0.7
11/10/2017 12:31 PM	30.5	29.2	27.8	2.1	0.5	-0.155	0.7
11/10/2017 12:32 PM	30.5	28.5	27.6	2.1	0.6	-0.157	0.7
11/10/2017 12:33 PM	30.6	27.9	27.4	2.1	0.5	-0.155	0.7
11/10/2017 12:34 PM	30.6	29.7	27.8	2.1	0.3	-0.159	0.8
11/10/2017 12:35 PM	30.6	27.7	27.5	2.1	0.5	-0.165	0.7
11/10/2017 12:36 PM	30.4	28.8	27.1	2.1	0.9	-0.148	0.8
11/10/2017 12:37 PM	30.4	27.2	27.8	2.2	0.7	-0.156	0.8
11/10/2017 12:38 PM	30.3	28.1	27.6	2.2	0.6	-0.163	0.8
11/10/2017 12:39 PM	30.6	29.6	27.5	2.2	0.6	-0.156	0.8
11/10/2017 12:40 PM	30.6	27.8	27.8	2.2	0.4	-0.148	0.8
11/10/2017 12:41 PM	30.7	29.1	28	2.3	0.4	-0.162	0.8
11/10/2017 12:42 PM	30.8	28.1	27.8	2.3	0.5	-0.163	0.8
11/10/2017 12:43 PM	30.8	29.4	27.9	2.2	0.3	-0.154	0.8
11/10/2017 12:44 PM	30.8	29.1	28.2	2	0.6	-0.158	0.8
11/10/2017 12:45 PM	30.8	30.4	28.4	2	0.4	-0.172	0.8
11/10/2017 12:46 PM	30.8	29.1	28.3	1.9	0.6	-0.167	0.7
11/10/2017 12:47 PM	30.8	28.7	28.9	1.8	0.5	-0.150	0.7
11/10/2017 12:48 PM	30.9	28.8	28.5	1.7	0.4	-0.167	0.7
11/10/2017 12:49 PM	30.9	29.8	28.4	1.6	0.4	-0.169	0.6
11/10/2017 12:50 PM	30.9	28.6	28.2	1.5	0.3	-0.163	0.7
11/10/2017 12:51 PM	30.7	29.1	28.2	1.4	0.7	-0.156	0.6
11/10/2017 12:52 PM	30.7	29.5	28.1	1.3	0.6	-0.169	0.7
11/10/2017 12:53 PM	30.6	29.2	28.1	1.3	0.5	-0.169	0.7
11/10/2017 12:54 PM	30.7	28.3	28.3	1.2	0.3	-0.160	0.7
11/10/2017 12:55 PM	30.6	28.6	27.8	1.3	0.5	-0.154	0.7
11/10/2017 12:56 PM	30.6	28.4	28.3	1.3	0.7	-0.166	0.7
11/10/2017 12:57 PM	30.7	29.7	27.8	1.6	0.5	-0.165	0.7
11/10/2017 12:58 PM	30.7	28.6	27.5	1.9	0.3	-0.153	0.7
11/10/2017 12:59 PM	30.7	29.8	27.8	2.3	0.5	-0.158	0.7
11/10/2017 1:00 PM	30.5	27.4	27.4	2.6	0.6	-0.166	0.7
11/10/2017 1:01 PM	30.2	27.2	27.2	2.8	1	-0.160	0.7
11/10/2017 1:02 PM	30	27.7	27.2	3	0.7	-0.153	0.7
11/10/2017 1:03 PM	29.8	28.8	27.4	3.1	0.6	-0.154	0.7
11/10/2017 1:04 PM	30	26.8	27.2	3.1	0.4	-0.160	0.7
11/10/2017 1:05 PM	30	27.8	27.4	3.1	0.4	-0.159	0.7
11/10/2017 1:06 PM	30	29	27.8	3	0.5	-0.152	0.7
11/10/2017 1:07 PM	30.2	29.8	28	2.9	0.3	-0.150	0.8
11/10/2017 1:08 PM	30.6	29	28.1	2.8	0.7	-0.156	0.8
11/10/2017 1:09 PM	30.5	25.8	27.3	2.7	0.6	-0.159	0.8
11/10/2017 1:10 PM	30.1	29.4	27.2	2.7	0.8	-0.151	0.8
11/10/2017 1:11 PM	29.9	30	28.4	2.6	0.6	-0.155	0.8
11/10/2017 1:12 PM	30.1	28.4	28.2	2.5	0.5	-0.164	0.8
11/10/2017 1:13 PM	30.5	29.6	28.6	2.5	0.4	-0.151	0.8
11/10/2017 1:14 PM	30.7	28	27.4	2.4	0.8	-0.151	0.8
11/10/2017 1:15 PM	30.4	26.7	27.6	2.4	0.8	-0.155	0.8
11/10/2017 1:16 PM	30.2	30.1	27.3	2.3	0.7	-0.160	0.8

11/10/2017 1:17 PM	30	29.3	27.6	2.3	0.7	-0.151	0.8
11/10/2017 1:18 PM	29.9	28.6	27.4	2.3	0.6	-0.150	0.8
11/10/2017 1:19 PM	30	29.8	27.5	2.3	0.6	-0.158	0.8
11/10/2017 1:20 PM	30	27.9	27.7	2.3	0.5	-0.164	0.8
11/10/2017 1:21 PM	30.2	27.8	28	2.3	0.6	-0.156	0.8
11/10/2017 1:22 PM	30.3	28.1	27.9	2.3	0.4	-0.157	0.8
11/10/2017 1:23 PM	30.4	28.6	28.1	2.4	0.2	-0.166	0.8
11/10/2017 1:24 PM	30.7	28.1	28.1	2.4	0.3	-0.166	0.8
11/10/2017 1:25 PM	30.7	28	28.1	2.4	0.7	-0.152	0.8
11/10/2017 1:26 PM	30.7	29.1	27.9	2.3	0.5	-0.160	0.8
11/10/2017 1:27 PM	30.6	28.2	28.5	2.3	0.4	-0.162	0.8
11/10/2017 1:28 PM	30.7	28.7	28.1	2.3	0.3	-0.167	0.8
11/10/2017 1:29 PM	30.8	28.4	27.8	2.2	0.5	-0.157	0.8
11/10/2017 1:30 PM	30.8	30.3	28.3	2.2	0.5	-0.165	0.8
11/10/2017 1:31 PM	30.9	29.5	28.2	2.1	0.7	-0.164	0.8
11/10/2017 1:32 PM	31	29.5	28.2	2.1	0.3	-0.168	0.8
11/10/2017 1:33 PM	31	30.6	28	2.1	0.5	-0.153	0.8
11/10/2017 1:34 PM	30.9	28.2	27.8	2.1	0.4	-0.169	0.8
11/10/2017 1:35 PM	30.8	30.6	28.2	2.1	0.7	-0.164	0.8
11/10/2017 1:36 PM	30.9	28.9	28.5	2.1	0.7	-0.156	0.8
11/10/2017 1:37 PM	30.9	29.2	28.6	2.1	0.5	-0.150	0.8
11/10/2017 1:38 PM	31	29.1	28.5	2	0.6	-0.138	0.8
11/10/2017 1:39 PM	31	28.2	28.2	2	0.9	-0.120	0.8
11/10/2017 1:40 PM	30.7	29.3	27.9	2	3.1	2.263	0.9
11/10/2017 1:41 PM	29.8	26.1	27.3	2.1	8.2	3.750	1
11/10/2017 1:42 PM	28.6	24.8	25.8	2.2	22.8	1.689	0.9
11/10/2017 1:43 PM	26.7	25.2	25.2	2.2	32.6	1.328	0.9
11/10/2017 1:44 PM	24.9	23.7	24.8	2.8	40.4	2.290	1
11/10/2017 1:45 PM	24	25.6	25.9	4.5	25.9	1.420	0.9
11/10/2017 1:46 PM	25	28.4	27.3	5.9	13	1.888	0.9
11/10/2017 1:47 PM	27	28	27.9	7.2	5.4	2.306	1
11/10/2017 1:48 PM	28.6	26.8	27.6	8	6.3	1.650	0.9
11/10/2017 1:49 PM	29.3	29.8	28.2	8.3	5.8	3.000	0.9
11/10/2017 1:50 PM	27.8	26.2	179.9	8.4	131.2	6.448	1.3
11/10/2017 1:51 PM	19.6	32	354.7	9.2	613.7	12.675	2.3
11/10/2017 1:52 PM	10.6	24.7	274.2	21.9	696.6	13.044	2.5
11/10/2017 1:53 PM	4.1	19.6	201.8	44.6	462.2	8.786	1.8
11/10/2017 1:54 PM	4.6	22.5	195.5	63.3	375.5	7.749	1.6
11/10/2017 1:55 PM	4.7	101.3	1334	84.4	1086.6	49.528	7.4
11/10/2017 1:56 PM	4.5	15.7	158.5	136.2	270.5	7.370	2.8
11/10/2017 1:57 PM	4.5	15	105.6	172.3	183.7	3.188	1.2
11/10/2017 1:58 PM	6.6	15.9	91.6	179.4	155.8	3.577	1.2
11/10/2017 1:59 PM	8.2	15.1	101.1	171.7	183.1	3.269	1.2
11/10/2017 2:00 PM	10.1	17.1	63.8	155.2	93.6	1.729	1.1
11/10/2017 2:01 PM	12.4	21.3	27	135.2	39.6	0.692	0.9
11/10/2017 2:02 PM	13.6	50.3	470.2	120	989.7	15.497	2.6
11/10/2017 2:03 PM	10	106.6	1527.5	105.7	828	44.838	8.3

11/10/2017 2:04 PM	5.7	16.2	166	105	322.8	8.698	4.2
11/10/2017 2:05 PM	3.2	13.9	125.2	105.1	180.1	3.892	1.5
11/10/2017 2:06 PM	4.5	17.8	225.1	105.3	356.9	7.089	1.8
11/10/2017 2:07 PM	4.7	16	211.4	103.9	472	7.767	1.9
11/10/2017 2:08 PM	3.7	26.9	289.4	105.5	554.5	8.873	2
11/10/2017 2:09 PM	2.5	45.2	533.9	110.9	851.6	18.154	3.6
11/10/2017 2:10 PM	1.6	50.7	551.7	122.9	917.3	18.354	4.2
11/10/2017 2:11 PM	1.3	52	635	132.7	599.8	23.837	5.2
11/10/2017 2:12 PM	2.8	20	174	153.5	61.8	9.625	3.2
11/10/2017 2:13 PM	5	26.3	200.3	166.1	74	15.500	3.1
11/10/2017 2:14 PM	6.2	76.6	755.2	166.8	462.6	35.899	5.5
11/10/2017 2:15 PM	4.7	31.9	387	161.4	721.6	19.945	5.1
11/10/2017 2:16 PM	2.7	18.8	237.2	164.3	362.8	9.727	2.7
11/10/2017 2:17 PM	1.5	25.2	279.1	160.4	535.8	12.360	2.9
11/10/2017 2:18 PM	2.1	25.6	315.3	156.5	423.3	10.503	2.5
11/10/2017 2:19 PM	2.7	33.6	360.8	151.6	367.6	12.680	2.9
11/10/2017 2:20 PM	2.7	27.5	321.5	136.4	501.6	12.212	3
11/10/2017 2:21 PM	2.6	18.8	117.9	130.9	348.4	9.604	2.5
11/10/2017 2:22 PM	2.5	21.6	276.7	131.2	371.2	7.918	2
11/10/2017 2:23 PM	2.6	15.8	249.9	130.3	391.1	7.677	2
11/10/2017 2:24 PM	2.6	17.5	148.5	126.2	402.1	8.076	2
11/10/2017 2:25 PM	2.9	14.5	100.6	123.2	180.5	4.555	1.8
11/10/2017 2:26 PM	3.8	35.9	644.7	121.4	307.3	15.705	3
11/10/2017 2:27 PM	3.8	18.5	235.2	119	450.7	8.775	2.1
11/10/2017 2:28 PM	3.1	18.8	227.2	119.3	428.2	9.608	2
11/10/2017 2:29 PM	3	16.7	82.7	117.4	81.4	7.456	2.3
11/10/2017 2:30 PM	4.4	225.1	3160.4	109.6	196.7	73.616	10.7
11/10/2017 2:31 PM	4.1	67.7	798.7	102.7	365.1	58.299	14.5
11/10/2017 2:32 PM	3.2	14.8	167	94.4	189.6	7.059	3.1
11/10/2017 2:33 PM	1.9	10.3	86.7	88	116.8	3.428	1.8
11/10/2017 2:34 PM	3.6	7.3	17.3	80.3	17.1	0.285	1.2
11/10/2017 2:35 PM	5.6	8.8	25.7	71.7	12.8	0.347	1.1
11/10/2017 2:36 PM	7.3	9.7	28.1	62.5	6.9	0.390	1.2
11/10/2017 2:37 PM	8.2	11.9	21.8	53.9	5.3	0.351	1.1
11/10/2017 2:38 PM	8.7	10.9	21.2	45.4	3.9	0.232	1.1
11/10/2017 2:39 PM	9.2	10.4	19.5	39.9	5.5	0.618	1.1
11/10/2017 2:40 PM	9.7	11.3	20.5	32.9	3.3	0.225	1
11/10/2017 2:41 PM	10.1	13.4	18.7	27	2.7	0.072	1
11/10/2017 2:42 PM	10.8	13.3	14.7	22.1	2.4	-0.054	1
11/10/2017 2:43 PM	11.7	13.2	14.2	19	1.7	-0.114	0.9
11/10/2017 2:44 PM	12.5	13.8	19.3	18.9	2.4	0.235	1.2
11/10/2017 2:45 PM	13.1	13.6	21.2	12.2	9.4	0.518	1.3
11/10/2017 2:46 PM	13.1	14.2	18.3	7.2	12.5	4.951	1.2
11/10/2017 2:47 PM	13.4	13.6	17.7	6.1	2.5	0.753	1.7
11/10/2017 2:48 PM	13.9	16.1	14.8	5	1.4	0.045	2.5
11/10/2017 2:49 PM	14.1	12.9	20.8	4.1	3	0.810	2.8
11/10/2017 2:50 PM	14.3	15.7	15.6	3.5	1.5	-0.144	1.9

11/10/2017 2:51 PM	14.9	15.8	15.2	2.9	1.3	-0.171	1.8
11/10/2017 2:52 PM	16.3	17.9	16.9	2.3	1.3	-0.181	1.9
11/10/2017 2:53 PM	17.3	19	18.2	1.8	1.2	-0.194	1.3
11/10/2017 2:54 PM	18	18.7	18.2	1.4	1	-0.200	1.1
11/10/2017 2:55 PM	18.6	20	18.4	1	1.1	-0.188	0.9
11/10/2017 2:56 PM	18.9	19.2	18.9	0.6	1.4	-0.187	0.9
11/10/2017 2:57 PM	19.2	19.8	19.4	0.3	1.1	-0.195	0.9
11/10/2017 2:58 PM	19.4	21.9	19.6	0	1.3	-0.185	0.9
11/10/2017 2:59 PM	19.7	21.7	20	-0.2	1.5	-0.186	0.9
11/10/2017 3:00 PM	19.8	20	20	-0.4	1.1	-0.195	0.9
11/10/2017 3:01 PM	20.1	20.5	20.4	-0.6	0.9	-0.199	0.9
11/10/2017 3:02 PM	20.4	20.8	20.8	-0.7	1.2	-0.189	0.9
11/10/2017 3:03 PM	20.7	21.8	20.9	-0.8	1.2	-0.114	0.9
11/10/2017 3:04 PM	21.1	20.6	21	-0.9	1	-0.181	0.9
11/10/2017 3:05 PM	21.3	22.4	21.1	-1	0.9	-0.195	0.9
11/10/2017 3:06 PM	21.4	20.8	21.2	-1.1	1.3	-0.195	0.9
11/10/2017 3:07 PM	21.6	20.8	21.7	-1.1	1.1	-0.202	0.9
11/10/2017 3:08 PM	21.8	22.3	22	-1.2	1	-0.202	0.9
11/10/2017 3:09 PM	22	20.9	22	-1.3	1.1	-0.198	0.9
11/10/2017 3:10 PM	22.2	22.7	22	-1.3	1.1	-0.192	0.9
11/10/2017 3:11 PM	22.3	22.5	22	-1.4	1	-0.202	1
11/10/2017 3:12 PM	22.4	20.9	21.7	-1.4	1.2	-0.162	1
11/10/2017 3:13 PM	22.5	21.9	22.2	-1.4	1.4	-0.168	0.9
11/10/2017 3:14 PM	22.6	22.2	22.3	-1.4	1	-0.199	1
11/10/2017 3:15 PM	22.8	23.8	22.6	-1.4	1	-0.204	1
11/10/2017 3:16 PM	23.1	23.1	22.3	-1.4	1.2	-0.203	1
11/10/2017 3:17 PM	23.4	23.6	22.5	-1.4	1	-0.197	1
11/10/2017 3:18 PM	23.4	22.7	22.2	-1.4	1.2	-0.207	1
11/10/2017 3:19 PM	23.5	24.3	22.7	-1.4	1.2	-0.182	1
11/10/2017 3:20 PM	23.5	23.6	22.8	-1.4	1.3	-0.198	1
11/10/2017 3:21 PM	23.6	23.8	22.8	-1.4	1.3	-0.201	1
11/10/2017 3:22 PM	23.6	23.5	22.3	-1.4	3.8	-0.124	1
11/10/2017 3:23 PM	23.4	24.3	22.9	-1.4	4.1	-0.005	1
11/10/2017 3:24 PM	23.6	23.3	23.3	-1.4	1.1	-0.185	1
11/10/2017 3:25 PM	23.8	24.9	23.5	-1.3	1.6	-0.036	0.9
11/10/2017 3:26 PM	24.3	23.2	23.3	-1.2	1.2	-0.210	1
11/10/2017 3:27 PM	24.3	22.2	23.2	-1.1	1.3	-0.199	1
11/10/2017 3:28 PM	22.4	20.9	131	-1.1	87.3	1.707	1.3
11/10/2017 3:29 PM	16.2	26.7	285.8	0	269.8	6.496	2.1
11/10/2017 3:30 PM	9	25.5	292.9	10.6	361.3	9.157	2.6
11/10/2017 3:31 PM	3.9	16.6	113.4	25.6	296.4	5.361	2.2
11/10/2017 3:32 PM	4.1	30.2	324.3	40.6	356.9	9.597	2.6
11/10/2017 3:33 PM	3.6	27.8	322.6	59.2	473.7	10.776	2.9
11/10/2017 3:34 PM	3	25.4	263.5	74	569.6	11.813	2.9
11/10/2017 3:35 PM	1.8	23.3	223.2	87.1	434.7	9.414	2.6
11/10/2017 3:36 PM	2.3	21.2	231.3	101	463.1	7.699	2.2
11/10/2017 3:37 PM	2.4	22.3	263.3	114.7	596.8	10.476	2.4

11/10/2017 3:38 PM	2.5	16.6	171	124.8	405.2	6.877	2
11/10/2017 3:39 PM	2.6	131.8	1554.1	129.2	684.5	22.749	5
11/10/2017 3:40 PM	2.1	180.3	2431.8	154.2	884.4	58.878	13.1
11/10/2017 3:41 PM	1.6	47.4	405.4	207.5	358.6	42.267	10.6
11/10/2017 3:42 PM	1	12.2	179.6	247	292.8	6.585	2.6
11/10/2017 3:43 PM	1.5	13.5	164.6	262.2	292.7	4.991	1.9
11/10/2017 3:44 PM	1.9	14.3	142.9	247.9	323.4	5.778	2.1
11/10/2017 3:45 PM	2.1	52.2	626.6	247.1	384.5	13.160	3.6
11/10/2017 3:46 PM	1.8	54.6	542.8	185.6	413	30.302	6.1
11/10/2017 3:47 PM	2.8	10.7	22.8	107.8	50.5	4.598	3.4
11/10/2017 3:48 PM	4.8	17.6	70.8	81.7	27.6	1.294	2
11/10/2017 3:49 PM	5.9	46	555.2	60.7	316.2	18.462	4.2
11/10/2017 3:50 PM	4.9	16.3	126.7	40.6	387.5	8.571	2.7
11/10/2017 3:51 PM	3.9	31.5	326.9	28.5	299.7	7.842	2.4
11/10/2017 3:52 PM	2.7	27.5	349.1	23.5	637.9	14.021	3.2
11/10/2017 3:53 PM	4	11.1	-5.5	21.9	94.2	2.329	2
11/10/2017 3:54 PM	4.2	34.9	429.5	20.8	352.4	10.122	3
11/10/2017 3:55 PM	4.4	64.7	901.3	29.4	648.9	25.589	5.1
11/10/2017 3:56 PM	3	16.8	206.1	36.2	415.6	10.016	3.1
11/10/2017 3:57 PM	1.9	14.3	180.1	41.8	347.9	5.795	1.7
11/10/2017 3:58 PM	1.8	203.3	2851.4	44.2	543.7	40.157	6.6
11/10/2017 3:59 PM	1.6	169.4	1601.1	52.3	419.4	94.413	20.7
11/10/2017 4:00 PM	1.2	22.8	281.2	54.3	112.5	12.942	5.1
11/10/2017 4:01 PM	1.3	62.5	670.1	49.8	118.6	25.170	5.1
11/10/2017 4:02 PM	1.4	36.5	345.3	39.8	189.5	28.237	6.3
11/10/2017 4:03 PM	1.6	15.7	83.4	27.7	78.7	10.425	3.5
11/10/2017 4:04 PM	2.1	15.2	119	13	31.4	5.431	2.4
11/10/2017 4:05 PM	3	9.2	40.4	-1.9	20.8	3.922	2.3
11/10/2017 4:06 PM	4.2	6.4	21.6		5.9	0.913	1.4
11/10/2017 4:07 PM	5.1	7.3	9.1		13.3	0.466	1.5
11/10/2017 4:08 PM	5.9	10.1	10.5		7	0.248	1.7
11/10/2017 4:09 PM	6.7	7.9	11.7		2.5	0.127	1.2
11/10/2017 4:10 PM	7.5	10	11.9		2.4	0.027	1.1
11/10/2017 4:11 PM	8.4	9.9	20.7		2.5	0.121	1.1
11/10/2017 4:12 PM	8.7	10.6	31		2.8	0.345	1.1
11/10/2017 4:13 PM	8.9	11.5	21.1		2.4	0.258	1.5
11/10/2017 4:14 PM	9.5	11.1	9.1		1.3	-0.116	2.2
11/10/2017 4:15 PM	10.3	10.9	8.8		1.6	-0.022	3
11/10/2017 4:16 PM	10.9	9.8	11.4		2.1	0.152	7.3
11/10/2017 4:17 PM	11.4	13.1	14.9		1.5	-0.075	6.8
11/10/2017 4:18 PM	11.7	12.8	11.4		1.5	-0.171	7.2
11/10/2017 4:19 PM	11.9	10.7	12		1.3	-0.195	5.8
11/10/2017 4:20 PM	11.8	11	13.1		1.4	-0.144	3.1
11/10/2017 4:21 PM	11.9	13.1	12.1		1.4	-0.224	6.3
11/10/2017 4:22 PM	12.2	15.2	12.4		1.9	-0.046	6.5
11/10/2017 4:23 PM	13	13.1	12.9		1.6	-0.186	4
11/10/2017 4:24 PM	13.5	13.6	14.3		1.8	0.080	5.7

11/10/2017 4:25 PM	13.8	14.5	13.7	1.4	-0.145	7.5
11/10/2017 4:26 PM	13.4	12.2	15.2	1.6	-0.075	6.1
11/10/2017 4:27 PM	13.4	14.9	13.6	1.2	-0.189	6.5
11/10/2017 4:28 PM	12.5	14.6	14.2	1.2	-0.158	6.6
11/10/2017 4:29 PM	12.1	14	13.5	3.1	0.345	4.5
11/10/2017 4:30 PM		16	15.1	1.5	-0.225	9.7
11/10/2017 4:31 PM		11.2	17.7	1.5	-0.191	4.5
11/10/2017 4:32 PM		16	15.3	1.4	-0.214	7.3
11/10/2017 4:33 PM		17.6	14.6	1.5	-0.202	6.6
11/10/2017 4:34 PM		16.9	15.3	2.6	0.700	2.3
11/10/2017 4:35 PM		16.7	15.8	1.2	-0.227	2.2
11/10/2017 4:36 PM		16.3	14.7	1	-0.223	6.5
11/10/2017 4:37 PM		16.4	16.1	3.5	0.883	2.2
11/10/2017 4:38 PM		16.3	15.9	2.3	-0.135	1
11/10/2017 4:39 PM		16.9	15.6	9.3	-0.206	0.8
11/10/2017 4:40 PM		17.1	16.9	1.9	0.029	0.8
11/10/2017 4:41 PM		17.7	16.2	7.1	1.954	0.8
11/10/2017 4:42 PM		17.7	16.8	4.7	0.480	0.9
11/10/2017 4:43 PM		18.5	16.7	5.1	1.656	1
11/10/2017 4:44 PM		18.6	17	4.6	0.867	0.9
11/10/2017 4:45 PM		15.6	15.6	13	-0.053	0.9
11/10/2017 4:46 PM		20	17.7	1.7	-0.201	0.9
11/10/2017 4:47 PM		18.3	17.3	2.2	-0.014	0.9
11/10/2017 4:48 PM		17.8	17.3	3.1	0.624	0.9
11/10/2017 4:49 PM		20.8	17.7	2.2	0.157	0.9
11/10/2017 4:50 PM		18	18	2.2	0.033	0.9
11/10/2017 4:51 PM		18.5	18.1	1.6	-0.110	0.9
11/10/2017 4:52 PM		18	16.5	5.5	0.184	0.9
11/10/2017 4:53 PM		20	18	2.3	-0.154	0.9
11/10/2017 4:54 PM		17.3	17.4	2.9	0.152	0.9
11/10/2017 4:55 PM		20.4	17.2	6.5	1.565	0.9
11/10/2017 4:56 PM		18	16.7	12.5	1.854	1.1
11/10/2017 4:57 PM		18.1	16.1	12.5	2.164	1.1
11/10/2017 4:58 PM		19	18	6.1	1.974	1
11/10/2017 4:59 PM		18.1	17.8	6.2	1.580	1
11/10/2017 5:00 PM		18.6	15.9	10.9	0.476	0.9
11/10/2017 5:01 PM	17.7	19.5	17.2	9.9	2.094	1
11/10/2017 5:02 PM	17.8	18.2	17.1	5.9	1.568	1
11/10/2017 5:03 PM	18.2	20	17.8	6.9	1.366	0.9
11/10/2017 5:04 PM	18.9	18.8	18	3.6	1.055	0.9
11/10/2017 5:05 PM	19	19.3	17.5	6.3	1.735	1
11/10/2017 5:06 PM	19.1	19.1	18.1	3	0.587	1.1
11/10/2017 5:07 PM	18.9	19.4	17.4	3.8	0.437	1
11/10/2017 5:08 PM	19	19.3	17.9	4.1	0.686	1
11/10/2017 5:09 PM	19.1	18.8	18.3	2.4	0.106	1
11/10/2017 5:10 PM	19.4	19.3	18.5	5.9	1.050	1
11/10/2017 5:11 PM	19.8	19.7	18.8	2.7	0.157	1

11/10/2017 5:12 PM	19.9	20.8	18.9	1.9	-0.121	1
11/10/2017 5:13 PM	18.3	15.2	16.6	59.6	25.851	1.3
11/10/2017 5:14 PM	15.2	16.7	13.4	49.3	17.082	1.2
11/10/2017 5:15 PM	13.8	17	14.9	12.4	5.068	1
11/10/2017 5:16 PM	14.7	18.3	16.6	4.7	0.420	1
11/10/2017 5:17 PM	17.5	19.5	17.6	2.3	-0.091	1
11/10/2017 5:18 PM	18.6	18.2	18	1.8	-0.171	1
11/10/2017 5:19 PM	19.2	18.8	17.6	2.2	-0.208	2.3
11/10/2017 5:20 PM	19.3	20	18.4	2.5	-0.222	2.3
11/10/2017 5:21 PM	19.3	23.1	19.8	2.7	-0.219	4.1
11/10/2017 5:22 PM	19	30.1	24.5	3.5	-0.227	10.8
11/10/2017 5:23 PM	18.7	23.4	19.1	2.8	-0.233	4.5
11/10/2017 5:24 PM	18.4	20.9	18.2	3.4	-0.219	2.6
11/10/2017 5:25 PM	17.9	16.1	14.1	8.4	-0.212	1.9
11/10/2017 5:26 PM	16.5	14.2	13.7	18.3	-0.143	1.3
11/10/2017 5:27 PM	15.1	15.3	15.1	14.4	-0.148	1.1
11/10/2017 5:28 PM	14.7	14.9	13.6	13.9	-0.176	1
11/10/2017 5:29 PM	15	15.1	14	6.6	-0.206	1
11/10/2017 5:30 PM	15.4	17.1	17.5	5.3	-0.065	1.3
11/10/2017 5:31 PM	15.5	16	16.2	10.9	-0.010	1.5
11/10/2017 5:32 PM	15.6	14	15	7.5	-0.162	1.2
11/10/2017 5:33 PM	15.1	13.8	14	5.1	-0.195	1.1
11/10/2017 5:34 PM	14.7	14.9	15	9.7	-0.212	1.1
11/10/2017 5:35 PM	14.5	13.8	15.2	8.7	-0.215	1
11/10/2017 5:36 PM	15.1	16.1	17.4	10.4	-0.214	1.2
11/10/2017 5:37 PM	15.6	16.9	16.8	5.5	-0.223	1.4
11/10/2017 5:38 PM	15.7	14.6	16.5	3.6	-0.213	1.1
11/10/2017 5:39 PM	15.8	14.8	16.2	7	-0.224	1
11/10/2017 5:40 PM	16	17.3	17	13.7	-0.234	0.9
11/10/2017 5:41 PM	16.4	15.8	16.4	6	-0.169	0.9
11/10/2017 5:42 PM	16.4	14.8	16.9	4.9	-0.128	0.9
11/10/2017 5:43 PM	15.8	14.3	14.6	4.2	-0.158	0.9
11/10/2017 5:44 PM	15.3	15.7	15.5	6.1	-0.231	0.8
11/10/2017 5:45 PM	15.5	14.6	15.9	9.5	-0.242	0.8
11/10/2017 5:46 PM	16.1	16.5	16.5	7.4	-0.244	0.8
11/10/2017 5:47 PM	16.5	14	15.6	5.4	-0.254	0.8
11/10/2017 5:48 PM	16.1	15.6	15.5	8.7	-0.251	0.7
11/10/2017 5:49 PM	15.6	15.9	15.7	6.2	-0.254	0.7
11/10/2017 5:50 PM	15.2	15.6	15.1	9.4	-0.252	0.7
11/10/2017 5:51 PM	14.9	13.5	14	17.1	-0.261	0.7
11/10/2017 5:52 PM	14.7	15.3	14	2	-0.254	0.7
11/10/2017 5:53 PM	14.7	13	14.1	1.7	-0.256	0.6
11/10/2017 5:54 PM	15.1	15.8	15.1	1.8	-0.257	0.6
11/10/2017 5:55 PM	15.5	14	15	1.6	-0.254	0.6
11/10/2017 5:56 PM	15.9	15.4	15.1	1.3	-0.245	0.5
11/10/2017 5:57 PM	16.1	13.8	14.7	1.6	-0.250	0.5
11/10/2017 5:58 PM	16.1	15.2	14.8	1.5	-0.255	0.5

11/10/2017 5:59 PM	15.9	14.8	14.4	1.5	-0.249	0.4
11/10/2017 6:00 PM	15.5	14.2	14.2	1.8	-0.247	0.5
11/10/2017 6:01 PM	14.5	12.5	11.9	2.2	-0.237	0.5
11/10/2017 6:02 PM	12.6	6.5	8.8	2.7	-0.241	0.5
11/10/2017 6:03 PM	9.9	5.1	6.9	3	-0.249	0.5
11/10/2017 6:04 PM	7.2	1.6	5.9	3.6	-0.237	0.5
11/10/2017 6:05 PM	5	2.3	5.2	3.8	-0.228	0.5
11/10/2017 6:06 PM	3.7	0.3	5	4.1	-0.233	0.5
11/10/2017 6:07 PM	2.9	2.9	5.1	4.4	-0.232	0.5
11/10/2017 6:08 PM	2.5	2.3	5.2	4.7	-0.220	0.6
11/10/2017 6:09 PM	3	8.4	10.5	4.2	-0.221	0.5
11/10/2017 6:10 PM	7	15.3	16.7	1.8	-0.232	-1
11/10/2017 6:11 PM	11.5	13.4	13.8	2	-0.227	-1.5
11/10/2017 6:12 PM	13.6	7.8	9.7	2.5	-0.219	-1.5
11/10/2017 6:13 PM	11.4	4.5	7.7	3.1	-0.224	-1.5
11/10/2017 6:14 PM	7.7	3.4	6.5	3.2	-0.224	-1.5
11/10/2017 6:15 PM	5.3	2.9	5.7	3.8	-0.222	-1.5
11/10/2017 6:16 PM	3.9	2.8	5.4	4.4	-0.209	-1.5
11/10/2017 6:17 PM	3.1	2.5	5.4	4.6	-0.213	-1.5
11/10/2017 6:18 PM	2.5	1.8	5.2	4.6	-0.209	-1.5
11/10/2017 6:19 PM	2.2	0.8	5.2	4.8	-0.201	-1.5
11/10/2017 6:20 PM	2	0.6	5.3	5.1	-0.209	-1.5
11/10/2017 6:21 PM	1.9	1.3	5.4	5.2	-0.217	-1.5
11/10/2017 6:22 PM	1.9	2	5.2	5.4	-0.208	-1.5
11/10/2017 6:23 PM	1.9	1	5.6	5.6	-0.201	-1.5
11/10/2017 6:24 PM	1.9	1.1	5.5	5.7	-0.204	-1.5
11/10/2017 6:25 PM	2	1.5	5.7	6	-0.206	-1.5
11/10/2017 6:26 PM	2	1.3	5.5	6.4	-0.196	-1.5
11/10/2017 6:27 PM	2	2	6	6.4	-0.199	-1.5
11/10/2017 6:28 PM	2	0.9	5.7	6.4	-0.203	-1.5
11/10/2017 6:29 PM	2	2.2	6.1	6.5	-0.200	-1.5
11/10/2017 6:30 PM	2.2	1.7	6.2	6.2	-0.199	-1.5
11/10/2017 6:31 PM	2.3	1.5	6.3	6.1	-0.203	-1.5
11/10/2017 6:32 PM	2.3	2	6.4	5.8	-0.220	-1.5
11/10/2017 6:33 PM	2.3	0.7	5.8	5.9	-0.204	-1.5
11/10/2017 6:34 PM	2.3	1.9	6.5	5.9	-0.205	-1.5
11/10/2017 6:35 PM	2.3	3.1	6.5	5.9	-0.216	-1.5
11/10/2017 6:36 PM	2.4	2.4	6.1	5.6	-0.213	-1.5
11/10/2017 6:37 PM	2.4	0.7	5.8	5.6	-0.210	-1.5
11/10/2017 6:38 PM	2.2	2.1	5.8	5.8	-0.209	-1.5
11/10/2017 6:39 PM	2	1.3	6	5.5	-0.213	-1.5
11/10/2017 6:40 PM	2	1.9	6.4	5.5	-0.218	-1.5
11/10/2017 6:41 PM	2.1	0.6	6.3	5.6	-0.208	-1.5
11/10/2017 6:42 PM	2.2	2	6.5	6	-0.216	-1.5
11/10/2017 6:43 PM	2.4	1.9	6.4	5.6	-0.224	-1.5
11/10/2017 6:44 PM	2.4	2.8	6.6	5.7	-0.219	-1.5
11/10/2017 6:45 PM	2.5	1.2	6.5	5.7	-0.216	-1.5

11/10/2017 6:46 PM	2.4	0.6	6.5	5.7	-0.220	-1.5
11/10/2017 6:47 PM	2.3	0	6.4	5.7	-0.225	-1.5
11/10/2017 6:48 PM	2.3	2.3	6.5	5.5	-0.215	-1.5
11/10/2017 6:49 PM	2.3	0.1	6.2	5.5	-0.218	-1.5
11/10/2017 6:50 PM	2.5	2.1	6.7	5.4	-0.225	-1.5
11/10/2017 6:51 PM	2.6	1.8	7	5.3	-0.225	-1.5
11/10/2017 6:52 PM	2.6	2.2	6.7	5.5	-0.216	-1.5
11/10/2017 6:53 PM	2.7	2.4	6.6	5.4	-0.227	-1.5
11/10/2017 6:54 PM	2.6	1.6	6.7	5.4	-0.227	-1.5
11/10/2017 6:55 PM	2.6	2.4	6.9	5.3	-0.221	-1.5
11/10/2017 6:56 PM	2.7	2.7	7	5	-0.229	-1.5
11/10/2017 6:57 PM	2.7	3.4	6.6	5.3	-0.232	-1.5
11/10/2017 6:58 PM	2.7	2.8	6.8	5.2	-0.225	-1.5
11/10/2017 6:59 PM	2.6	0.5	6.7	5.4	-0.219	-1.5
11/10/2017 7:00 PM	2.6	2.7	6.8	5.4	-0.229	-1.5
11/10/2017 7:01 PM	2.5	0.8	6.7	5.1	-0.228	-1.5
11/10/2017 7:02 PM	2.4	2.2	6.8	5.2	-0.218	-1.5
11/10/2017 7:03 PM	2.6	2.1	7.1	5.1	-0.231	-1.5
11/10/2017 7:04 PM	2.8	1.9	7.4	5.1	-0.236	-1.5
11/10/2017 7:05 PM	3.1	2	7.4	4.7	-0.226	-1.5
11/10/2017 7:06 PM	3.3	2.7	7.1	4.8	-0.229	-1.5
11/10/2017 7:07 PM	3.3	2.7	7.3	4.9	-0.233	-1.5
11/10/2017 7:08 PM	3.2	1.7	7.2	5.1	-0.232	-1.5
11/10/2017 7:09 PM	3.3	2.2	7.3	4.7	-0.227	-1.5
11/10/2017 7:10 PM	3.3	1.8	7.1	4.8	-0.228	-1.5
11/10/2017 7:11 PM	3.3	1.4	7.2	4.9	-0.240	-1.5
11/10/2017 7:12 PM	3.2	2	7	5	-0.231	-1.5
11/10/2017 7:13 PM	3	1.8	7	4.9	-0.229	-1.5
11/10/2017 7:14 PM	3	1.3	6.9	4.9	-0.235	-1.5
11/10/2017 7:15 PM	3.1	2.1	7.4	4.7	-0.238	-1.5
11/10/2017 7:16 PM	3.2	3.5	7.7	4.8	-0.233	-1.5
11/10/2017 7:17 PM	3.4	2.2	7.4	4.5	-0.227	-1.5
11/10/2017 7:18 PM	3.6	0.7	7.4	4.6	-0.233	-1.5
11/10/2017 7:19 PM	3.6	3	7.1	4.7	-0.235	-1.5
11/10/2017 7:20 PM	3.4	2.1	7.1	4.4	-0.227	-1.5
11/10/2017 7:21 PM	3.2	2.3	6.6	4.7	-0.222	-1.5
11/10/2017 7:22 PM	3.1	1.2	7	4.9	-0.235	-1.5
11/10/2017 7:23 PM	3	2.4	7.1	4.8	-0.234	-1.5
11/10/2017 7:24 PM	3.1	1.6	7.1	4.8	-0.228	-1.5
11/10/2017 7:25 PM	3	2.9	6.9	4.7	-0.229	-1.5
11/10/2017 7:26 PM	3	2.5	7.3	5.1	-0.237	-1.5
11/10/2017 7:27 PM	3	2.5	7.1	4.8	-0.230	-1.5
11/10/2017 7:28 PM	3.1	2.5	6.7	4.7	-0.227	-1.5
11/10/2017 7:29 PM	3.1	2.4	7.1	4.8	-0.238	-1.5
11/10/2017 7:30 PM	3	1.9	7.2	4.6	-0.234	-1.5
11/10/2017 7:31 PM	3.1	3.8	7.5	4.9	-0.227	-1.5
11/10/2017 7:32 PM	3.2	2.1	7.1	4.6	-0.230	-1.5

11/10/2017 7:33 PM	3.4	2.8	7.2		4.6	-0.237	-1.5
11/10/2017 7:34 PM	3.4	2.9	7.2		4.6	-0.233	-1.5
11/10/2017 7:35 PM	3.4	2.4	7		4.7	-0.225	-1.5
11/10/2017 7:36 PM	3.3	2.3	7.2		4.9	-0.236	-1.5
11/10/2017 7:37 PM	3.3	1.7	7.6		4.6	-0.233	-1.5
11/10/2017 7:38 PM	3.4	1.6	7.1		4.7	-0.236	-1.5
11/10/2017 7:39 PM	3.5	3.2	7.3		4.5	-0.230	-1.5
11/10/2017 7:40 PM	3.4	3.4	7.2		4.4	-0.248	-1.5
11/10/2017 7:41 PM	3.4	3	7.5		4.3	-0.232	-1.5
11/10/2017 7:42 PM	3.5	4.5	7.9		4.3	-0.228	-1.5
11/10/2017 7:43 PM	3.8	3.5	7.8		4.1	-0.239	-1.5
11/10/2017 7:44 PM	4	2	8.1		4	-0.240	-1.5
11/10/2017 7:45 PM	3.9	2.6	7.5		4.2	-0.235	-1.5
11/10/2017 7:46 PM	3.8	3.4	7.4		4.4	-0.236	-1.5
11/10/2017 7:47 PM	3.6	3.2	7.2		4.7	-0.236	-1.5
11/10/2017 7:48 PM	3.4	3.2	7		4.4	-0.241	-1.5
11/10/2017 7:49 PM	3.3	3	7.5		4.7	-0.231	-1.5
11/10/2017 7:50 PM	3.2	2	7.2		4.4	-0.236	-1.5
11/10/2017 7:51 PM	3.3	2.4	7.3	1.8	4.8	-0.235	-1.5
11/10/2017 7:52 PM	3.4	2.8	7.3	1.8	4.4	-0.243	-1.5
11/10/2017 7:53 PM	3.4	1.7	7.3	1.8	4.5	-0.241	-1.5
11/10/2017 7:54 PM	3.4	2.9	7.4	1.9	4.6	-0.231	-1.5
11/10/2017 7:55 PM	3.4	2.9	7.2	1.9	4.5	-0.242	-1.5
11/10/2017 7:56 PM	3.3	1.5	6.9	1.8	4.7	-0.243	-1.5
11/10/2017 7:57 PM	3.1	1.8	6.8	1.8	5	-0.231	-1.5
11/10/2017 7:58 PM	2.9	2.1	6.8	1.8	4.9	-0.233	-1.5
11/10/2017 7:59 PM	2.8	1	6.8	1.8	4.9	-0.242	-1.5
11/10/2017 8:00 PM	2.8	2.6	6.8	1.8	5	-0.238	-1.5
11/10/2017 8:01 PM	2.9	3.4	7.1	1.8	5	-0.229	-1.5
11/10/2017 8:02 PM	2.9	1.7	6.9	1.8	5	-0.233	-1.5
11/10/2017 8:03 PM	3	1.5	6.9	1.8	4.8	-0.248	-1.5
11/10/2017 8:04 PM	3.1	3.2	7.3	1.8	4.6	-0.235	-1.5
11/10/2017 8:05 PM	3.2	1	7.2	1.8	4.8	-0.233	-1.5
11/10/2017 8:06 PM	3.2	1.7	7.1	1.8	4.8	-0.236	-1.5
11/10/2017 8:07 PM	3.2	3.3	7.3	1.8	4.6	-0.251	-1.5
11/10/2017 8:08 PM	3.2	1.9	7.1	1.8	4.6	-0.236	-1.5
11/10/2017 8:09 PM	3.2	1.8	7	1.7	5	-0.237	-1.5
11/10/2017 8:10 PM	3.2	2.8	7.1	1.7	4.9	-0.243	-1.5
11/10/2017 8:11 PM	3.1	1.5	7	1.8	4.9	-0.234	-1.5
11/10/2017 8:12 PM	3.1	1.9	7.1	1.8	5	-0.232	-1.5
11/10/2017 8:13 PM	3.1	2.2	7.2	1.7	5	-0.237	-1.5
11/10/2017 8:14 PM	3.2	2.8	7.1	1.7	5	-0.243	-1.5
11/10/2017 8:15 PM	3.4	5.1	7.5	1.7	4.7	-0.234	-1.5
11/10/2017 8:16 PM	3.4	2.6	7.3	1.8	4.8	-0.233	-1.5
11/10/2017 8:17 PM	3.4	1.9	7.5	1.7	4.5	-0.243	-1.5
11/10/2017 8:18 PM	3.3	3.2	7.1	1.7	4.6	-0.243	-1.5
11/10/2017 8:19 PM	3.1	1.7	6.7	1.7	5	-0.230	-1.5

11/10/2017 8:20 PM	3	3.3	6.5	1.7	5.1	-0.234	-1.5
11/10/2017 8:21 PM	2.8	1.8	6.8	1.7	5	-0.238	-1.5
11/10/2017 8:22 PM	2.9	2.7	6.9	1.7	4.9	-0.240	-1.5
11/10/2017 8:23 PM	2.9	2.4	7	1.7	5.1	-0.232	-1.5
11/10/2017 8:24 PM	3.1	2.7	7.2	1.7	4.9	-0.234	-1.5
11/10/2017 8:25 PM	3.2	1.6	7.4	1.7	4.8	-0.241	-1.5
11/10/2017 8:26 PM	3.3	2	7.1	1.7	4.9	-0.240	-1.5
11/10/2017 8:27 PM	3.3	2.4	7.6	1.7	4.9	-0.232	-1.5
11/10/2017 8:28 PM	3.3	2	7.2	1.7	4.7	-0.240	-1.5
11/10/2017 8:29 PM	3.3	2.5	7.3	1.7	4.7	-0.242	-1.5
11/10/2017 8:30 PM	3.3	2.7	7.4	1.7	4.8	-0.234	-1.5
11/10/2017 8:31 PM	3.4	2.1	7.5	1.7	4.8	-0.238	-1.5
11/10/2017 8:32 PM	3.5	3.2	7.1	1.7	4.6	-0.247	-1.5
11/10/2017 8:33 PM	3.7	4.1	7.8	1.7	4.7	-0.230	-1.5
11/10/2017 8:34 PM	3.7	2.9	7.2	1.7	4.6	-0.233	-1.5
11/10/2017 8:35 PM	3.7	2.6	7.4	1.7	4.7	-0.247	-1.5
11/10/2017 8:36 PM	3.5	2.4	7.4	1.7	5	-0.239	-1.5
11/10/2017 8:37 PM	3.5	1.9	7.9	1.7	4.8	-0.233	-1.5
11/10/2017 8:38 PM	3.6	3	7.8	1.7	4.5	-0.236	-1.5
11/10/2017 8:39 PM	3.7	3.4	7.7	1.7	4.6	-0.247	-1.5
11/10/2017 8:40 PM	3.7	2.3	7.6	1.7	4.7	-0.236	-1.5
11/10/2017 8:41 PM	3.7	3.4	7.6	1.7	4.4	-0.235	-1.5
11/10/2017 8:42 PM	3.7	2.1	7.5	1.7	4.4	-0.246	-1.5
11/10/2017 8:43 PM	3.7	3.8	7.1	1.7	4.6	-0.238	-1.5
11/10/2017 8:44 PM	3.7	1.8	7.8	1.7	4.8	-0.236	-1.5
11/10/2017 8:45 PM	3.6	3.9	7.7	1.7	4.5	-0.237	-1.5
11/10/2017 8:46 PM	3.7	3.1	7.5	1.7	4.4	-0.245	-1.5
11/10/2017 8:47 PM	3.8	2.2	7.4	1.7	4.5	-0.241	-1.5
11/10/2017 8:48 PM	3.7	0.3	7.1	1.7	4.7	-0.234	-1.5
11/10/2017 8:49 PM	3.5	2.5	7.4	1.7	4.4	-0.248	-1.5
11/10/2017 8:50 PM	3.5	3.5	7.3	1.7	4.6	-0.246	-1.5
11/10/2017 8:51 PM	3.5	2.1	7.8	1.7	4.5	-0.234	-1.5
11/10/2017 8:52 PM	3.7	2.9	7.7	1.9	4.6	-0.239	-1.5
11/10/2017 8:53 PM	3.7	3.9	7.5	2.1	4.6	-0.248	-1.5
11/10/2017 8:54 PM	3.7	3.7	7.4	2	4.7	-0.238	-1.5
11/10/2017 8:55 PM	3.7	1.6	7.4	2	5	-0.235	-1.5
11/10/2017 8:56 PM	3.6	4	7.6	2	4.5	-0.243	-1.5
11/10/2017 8:57 PM	3.5	2.4	7.5	2	4.6	-0.244	-1.5
11/10/2017 8:58 PM	3.6	2.9	7.6	2	4.7	-0.236	-1.5
11/10/2017 8:59 PM	3.6	3.1	7.5	2	4.7	-0.248	-1.5
11/10/2017 9:00 PM	3.8	3.6	7.7	2	4.7	-0.247	-1.5
11/10/2017 9:01 PM	3.8	3.3	7.6	2	4.9	-0.241	-1.5
11/10/2017 9:02 PM	3.9	3.6	7.8	2.1	4.9	-0.231	-1.5
11/10/2017 9:03 PM	4	3.5	7.8	2.1	5.1	-0.235	-1.5
11/10/2017 9:04 PM	4.1	4	7.8	2.2	5.2	-0.246	-1.5
11/10/2017 9:05 PM	4.2	5.4	8.2	2.3	5.4	-0.241	-1.5
11/10/2017 9:06 PM	4.1	1.8	7.5	2.4	5.4	-0.237	-1.5

11/10/2017 9:07 PM	4.1	3.3	7.5	2.4	5.7	-0.249	-1.5
11/10/2017 9:08 PM	3.9	2.1	7.5	2.5	5.7	-0.248	-1.5
11/10/2017 9:09 PM	3.8	2.7	7.7	2.6	5.4	-0.234	-1.5
11/10/2017 9:10 PM	3.9	3.9	8.1	2.6	4.7	-0.245	-1.5
11/10/2017 9:11 PM	4.1	3.2	7.9	2.6	4.8	-0.254	-1.5
11/10/2017 9:12 PM	4.2	3.1	7.6	2.7	5	-0.241	-1.5
11/10/2017 9:13 PM	4	3.3	7.4	2.6	4.8	-0.240	-1.5
11/10/2017 9:14 PM	3.7	3.4	7.3	2.6	4.8	-0.248	-1.5
11/10/2017 9:15 PM	3.6	2.3	7.3	2.5	4.7	-0.240	-1.5
11/10/2017 9:16 PM	3.5	2.2	7.4	2.5	4.9	-0.239	-1.5
11/10/2017 9:17 PM	3.4	3.7	7.3	2.4	4.7	-0.242	-1.5
11/10/2017 9:18 PM	3.4	1	7.4	2.4	4.8	-0.253	-1.5
11/10/2017 9:19 PM	3.6	3.6	7.3	2.3	4.6	-0.240	-1.5
11/10/2017 9:20 PM	3.6	2.6	7.4	2.3	4.4	-0.228	-1.5
11/10/2017 9:21 PM	3.6	3.4	7.7	2.3	4.6	-0.249	-1.5
11/10/2017 9:22 PM	3.7	3.3	7.8	2.2	4.4	-0.239	-1.5
11/10/2017 9:23 PM	3.7	4.6	7.5	2.2	4.6	-0.234	-1.5
11/10/2017 9:24 PM	3.8	2.7	7.7	2.2	4.6	-0.244	-1.5
11/10/2017 9:25 PM	3.8	2.7	7.5	2.2	4.3	-0.245	-1.5
11/10/2017 9:26 PM	3.9	2.7	7.8	2.2	4.8	-0.242	-1.5
11/10/2017 9:27 PM	3.9	2.4	7.5	2.2	5	-0.243	-1.5
11/10/2017 9:28 PM	3.9	3.3	7.5	2.2	5.3	-0.251	-1.5
11/10/2017 9:29 PM	3.9	3	7.7	2.2	5.4	-0.243	-1.5
11/10/2017 9:30 PM	3.9	3.4	7.6	2.3	5.4	-0.233	-1.5
11/10/2017 9:31 PM	3.9	3.5	7.5	2.4	5.2	-0.237	-1.5
11/10/2017 9:32 PM	3.9	3	7.8	2.5	5.2	-0.242	-1.5
11/10/2017 9:33 PM	4	2.8	8.1	2.5	5.3	-0.244	-1.5
11/10/2017 9:34 PM	4	3.2	7.9	2.6	5	-0.233	-1.5
11/10/2017 9:35 PM	4	1.4	7.4	2.6	5	-0.255	-1.5
11/10/2017 9:36 PM	3.9	1.7	7.3	2.6	4.7	-0.244	-1.5
11/10/2017 9:37 PM	3.7	2.8	7.6	2.6	5.1	-0.234	-1.5
11/10/2017 9:38 PM	3.7	4.8	7.7	2.6	4.8	-0.253	-1.5
11/10/2017 9:39 PM	3.9	2.6	7.8	2.5	4.6	-0.251	-1.5
11/10/2017 9:40 PM	4.1	2.9	7.7	2.5	4.6	-0.242	-1.5
11/10/2017 9:41 PM	4.1	3.3	7.4	2.4	4.6	-0.234	-1.5
11/10/2017 9:42 PM	4	2.7	7.8	2.4	4.4	-0.251	-1.5
11/10/2017 9:43 PM	4	3.3	7.8	2.4	4.2	-0.245	-1.5
11/10/2017 9:44 PM	4.1	3.8	7.7	2.3	4.6	-0.235	-1.5
11/10/2017 9:45 PM	4.1	3.2	7.6	2.3	4.4	-0.246	-1.5
11/10/2017 9:46 PM	4.1	2.9	7.9	2.3	4.4	-0.248	-1.5
11/10/2017 9:47 PM	4.1	2.8	7.9	2.2	4.5	-0.243	-1.5
11/10/2017 9:48 PM	4.2	3.7	7.8	2.2	4.5	-0.242	-1.5
11/10/2017 9:49 PM	4.3	4.6	7.8	2.2	4.5	-0.250	-1.5
11/10/2017 9:50 PM	4.3	4.3	7.8	2.2	4.4	-0.247	-1.5
11/10/2017 9:51 PM	4.4	2.7	7.7	2.2	4.7	-0.243	-1.5
11/10/2017 9:52 PM	4.3	1.9	7.6	2.2	4.3	-0.241	-1.5
11/10/2017 9:53 PM	4.2	2.5	7.5	2.4	4.3	-0.249	-1.5

11/10/2017 9:54 PM	4.1	3	7.6	2.7	4.5	-0.243	-1.5
11/10/2017 9:55 PM	4.1	3.3	7.5	2.7	4.6	-0.237	-1.5
11/10/2017 9:56 PM	4	3.7	7.2	2.7	5.1	-0.245	-1.5
11/10/2017 9:57 PM	3.8	4	6.9	2.7	5.3	-0.244	-1.5
11/10/2017 9:58 PM	3.7	3.2	7.3	2.8	5	-0.230	-1.5
11/10/2017 9:59 PM	3.7	3.5	7.4	2.8	5.3	-0.245	-1.5
11/10/2017 10:00 PM	3.7	3	7.1	2.9	5.5	-0.251	-1.5
11/10/2017 10:01 PM	3.7	2.7	6.7	3	5.5	-0.237	-1.5
11/10/2017 10:02 PM	3.5	0.9	7	3	5.9	-0.234	-1.5
11/10/2017 10:03 PM	3.5	3	6.8	3.1	6	-0.242	-1.5
11/10/2017 10:04 PM	3.4	2.7	6.8	3.1	5.8	-0.246	-1.5
11/10/2017 10:05 PM	3.4	1.9	6.9	3.2	6	-0.232	-1.5
11/10/2017 10:06 PM	3.4	1.3	6.9	3.2	5.7	-0.239	-1.5
11/10/2017 10:07 PM	3.3	2.5	7.2	3.3	5.4	-0.242	-1.5
11/10/2017 10:08 PM	3.3	3.6	7.2	3.2	5.2	-0.250	-1.5
11/10/2017 10:09 PM	3.4	2.7	7.4	3.2	5.2	-0.237	-1.5
11/10/2017 10:10 PM	3.5	3	7.2	3.2	5	-0.237	-1.5
11/10/2017 10:11 PM	3.6	2.7	7.3	3.1	5	-0.249	-1.5
11/10/2017 10:12 PM	3.7	2.5	7.4	3.1	4.9	-0.244	-1.5
11/10/2017 10:13 PM	3.7	3.7	7.6	3.1	4.9	-0.235	-1.5
11/10/2017 10:14 PM	3.7	2.2	7.2	3	4.7	-0.250	-1.5
11/10/2017 10:15 PM	3.6	3.5	7.4	3	4.8	-0.245	-1.5
11/10/2017 10:16 PM	3.5	1.8	7.2	3	4.9	-0.236	-1.5
11/10/2017 10:17 PM	3.4	1.9	7	2.9	5.1	-0.238	-1.5
11/10/2017 10:18 PM	3.4	2.7	7.1	2.9	5	-0.244	-1.5
11/10/2017 10:19 PM	3.4	2.5	7	2.9	4.8	-0.243	-1.5
11/10/2017 10:20 PM	3.3	1.2	6.9	2.8	5.3	-0.237	-1.5
11/10/2017 10:21 PM	3.3	2.7	7.1	2.8	4.9	-0.240	-1.5
11/10/2017 10:22 PM	3.3	2.7	7	2.8	5	-0.242	-1.5
11/10/2017 10:23 PM	3.4	2.4	7.2	2.8	5.1	-0.237	-1.5
11/10/2017 10:24 PM	3.4	2.3	7.3	2.8	4.9	-0.239	-1.5
11/10/2017 10:25 PM	3.4	2.1	7.1	2.8	5	-0.241	-1.5
11/10/2017 10:26 PM	3.5	0.7	7.2	2.8	5	-0.239	-1.5
11/10/2017 10:27 PM	3.5	2.5	6.9	2.7	5.1	-0.230	-1.5
11/10/2017 10:28 PM	3.5	1.9	7	2.8	5.1	-0.230	-1.5
11/10/2017 10:29 PM	3.5	2.7	7	2.8	5.5	-0.244	-1.5
11/10/2017 10:30 PM	3.4	1.3	7	2.8	5.7	-0.236	-1.5
11/10/2017 10:31 PM	3.3	2.3	7.1	2.9	6	-0.233	-1.5
11/10/2017 10:32 PM	3.2	1.4	6.8	3	6.1	-0.245	-1.5
11/10/2017 10:33 PM	3.1	2	6.8	3.1	6.7	-0.240	-1.5
11/10/2017 10:34 PM	3	2.1	6.5	3.2	6.6	-0.235	-1.5
11/10/2017 10:35 PM	2.9	3.4	6.9	3.3	6.5	-0.239	-1.5
11/10/2017 10:36 PM	2.9	2.4	6.9	3.4	6.4	-0.244	-1.5
11/10/2017 10:37 PM	2.9	1.9	6.9	3.4	6.2	-0.238	-1.5
11/10/2017 10:38 PM	3	1.4	6.7	3.5	6.1	-0.234	-1.5
11/10/2017 10:39 PM	3	2.3	6.7	3.5	6	-0.240	-1.5
11/10/2017 10:40 PM	3	2.5	6.9	3.5	5.8	-0.244	-1.5

11/10/2017 10:41 PM	3	2.9	7.2	3.4	5.5	-0.232	-1.5
11/10/2017 10:42 PM	3	1.1	6.8	3.4	5.5	-0.241	-1.5
11/10/2017 10:43 PM	3.1	1.3	7	3.3	5.5	-0.248	-1.5
11/10/2017 10:44 PM	3.1	2.6	7	3.3	5.4	-0.237	-1.5
11/10/2017 10:45 PM	3.1	1	7	3.2	5.4	-0.235	-1.5
11/10/2017 10:46 PM	3.2	1.2	7	3.1	5.2	-0.240	-1.5
11/10/2017 10:47 PM	3.2	2.9	7.2	3.1	5.3	-0.246	-1.5
11/10/2017 10:48 PM	3.3	3.4	7.3	3.1	5.2	-0.230	-1.5
11/10/2017 10:49 PM	3.3	2.2	7.2	3	5.5	-0.244	-1.5
11/10/2017 10:50 PM	3.3	2.8	7.4	3	5.3	-0.245	-1.5
11/10/2017 10:51 PM	3.3	2.8	6.9	3	5	-0.241	-1.5
11/10/2017 10:52 PM	3.4	3.7	7.2	2.9	5.1	-0.235	-1.5
11/10/2017 10:53 PM	3.6	2.1	7.7	2.9	5	-0.245	-1.5
11/10/2017 10:54 PM	3.8	2.3	7.7	2.9	4.7	-0.244	-1.5
11/10/2017 10:55 PM	3.9	3.5	7.6	2.9	4.9	-0.239	-1.5
11/10/2017 10:56 PM	3.9	2.4	7.9	2.9	4.8	-0.240	-1.5
11/10/2017 10:57 PM	3.9	2.7	7.6	2.8	4.9	-0.252	-1.5
11/10/2017 10:58 PM	3.8	2.6	7.8	2.8	4.6	-0.246	-1.5
11/10/2017 10:59 PM	3.9	4.2	7.7	2.8	4.7	-0.233	-1.5
11/10/2017 11:00 PM	3.9	3.3	7.3	2.8	4.8	-0.244	-1.5
11/10/2017 11:01 PM	3.8	1.3	7.4	2.8	4.9	-0.247	-1.5
11/10/2017 11:02 PM	3.8	2.3	7.3	2.9	5.3	-0.235	-1.5
11/10/2017 11:03 PM	3.7	2.2	7.4	2.9	5.5	-0.245	-1.5
11/10/2017 11:04 PM	3.8	2.8	7.2	3	5.7	-0.247	-1.5
11/10/2017 11:05 PM	3.8	1.5	7.2	3.1	5.8	-0.241	-1.5
11/10/2017 11:06 PM	3.8	2.8	7	3.2	6	-0.238	-1.5
11/10/2017 11:07 PM	3.6	2.1	7	3.3	6.2	-0.245	-1.5
11/10/2017 11:08 PM	3.6	2.1	7.2	3.4	5.4	-0.252	-1.5
11/10/2017 11:09 PM	3.7	2.7	7.6	3.4	5.3	-0.235	-1.5
11/10/2017 11:10 PM	3.8	2.1	7.8	3.4	5.1	-0.237	-1.5
11/10/2017 11:11 PM	3.9	4.1	8	3.4	5.2	-0.249	-1.5
11/10/2017 11:12 PM	4.1	2.7	7.4	3.4	4.8	-0.247	-1.5
11/10/2017 11:13 PM	4.1	2.9	7.8	3.4	4.7	-0.244	-1.5
11/10/2017 11:14 PM	4	2.8	7.3	3.3	4.9	-0.244	-1.5
11/10/2017 11:15 PM	3.8	3.6	7.5	3.3	4.8	-0.249	-1.5
11/10/2017 11:16 PM	3.7	3.1	7.2	3.2	4.8	-0.243	-1.5
11/10/2017 11:17 PM	3.7	2.6	7.8	3.1	4.9	-0.238	-1.5
11/10/2017 11:18 PM	3.9	1.6	8	3.1	4.6	-0.243	-1.5
11/10/2017 11:19 PM	4.1	4.6	7.8	3	4.7	-0.249	-1.5
11/10/2017 11:20 PM	4.2	4.5	8	3	4.6	-0.238	-1.5
11/10/2017 11:21 PM	4.3	2.4	8	2.9	4.5	-0.241	-1.5
11/10/2017 11:22 PM	4.3	3.3	8	2.9	4.2	-0.249	-1.5
11/10/2017 11:23 PM	4.3	3.2	7.7	2.9	4.5	-0.237	-1.5
11/10/2017 11:24 PM	4.3	1.8	7.7	2.9	4.4	-0.238	-1.5
11/10/2017 11:25 PM	4.3	2.2	8.1	2.9	4.4	-0.242	-1.5
11/10/2017 11:26 PM	4.4	3.6	7.3	2.8	4.3	-0.250	-1.5
11/10/2017 11:27 PM	4.4	3.1	7.9	2.8	4.4	-0.238	-1.5

11/10/2017 11:28 PM	4.4	4.1	8.3	2.8	4.8	-0.244	-1.5
11/10/2017 11:29 PM	4.4	3.1	7.7	2.8	5.1	-0.242	-1.5
11/10/2017 11:30 PM	4.3	3.3	7.4	2.9	5.5	-0.236	-1.5
11/10/2017 11:31 PM	4.1	3.6	7.6	2.9	5.6	-0.233	-1.5
11/10/2017 11:32 PM	4	3.9	7.5	3	5.9	-0.245	-1.5
11/10/2017 11:33 PM	4	2.9	7.9	3.1	5.3	-0.247	-1.5
11/10/2017 11:34 PM	4.2	4.1	7.5	3.2	5.8	-0.236	-1.5
11/10/2017 11:35 PM	4.2	2.5	7.5	3.3	5.4	-0.241	-1.5
11/10/2017 11:36 PM	4.2	2.8	7.9	3.3	4.9	-0.245	-1.5
11/10/2017 11:37 PM	4.3	2.7	8	3.4	5.1	-0.244	-1.5
11/10/2017 11:38 PM	4.6	3.8	8.5	3.4	4.5	-0.240	-1.5
11/10/2017 11:39 PM	4.6	3.4	8.2	3.4	4.7	-0.238	-1.5
11/10/2017 11:40 PM	4.6	4.9	7.5	3.3	4.3	-0.248	-1.5
11/10/2017 11:41 PM	4.4	2.9	7.9	3.2	4.8	-0.238	-1.5
11/10/2017 11:42 PM	4.3	3.9	8.1	3.2	4.6	-0.236	-1.5
11/10/2017 11:43 PM	4.3	4	8.4	3.1	4.4	-0.240	-1.5
11/10/2017 11:44 PM	4.5	3.5	8.6	3.1	4.1	-0.248	-1.5
11/10/2017 11:45 PM	4.7	4.2	8.1	3	4.2	-0.242	-1.5
11/10/2017 11:46 PM	4.8	3.9	8.4	3	4.4	-0.238	-1.5
11/10/2017 11:47 PM	4.9	4.4	8.2	2.9	3.9	-0.243	-1.5
11/10/2017 11:48 PM	4.9	4.3	8.7	2.9	3.9	-0.244	-1.5
11/10/2017 11:49 PM	5	2.9	8.8	2.9	3.7	-0.239	-1.5
11/10/2017 11:50 PM	5.1	5.4	9	2.8	3.7	-0.239	-1.5
11/10/2017 11:51 PM	5.4	5	9	2.8	3.9	-0.247	-1.5
11/10/2017 11:52 PM	5.5	5.1	8.9	2.8	3.9	-0.244	-1.5
11/10/2017 11:53 PM	5.6	4.8	8.8	2.7	3.8	-0.238	-1.5
11/10/2017 11:54 PM	5.5	3.9	9.1	2.7	3.7	-0.237	-1.5
11/10/2017 11:55 PM	5.6	5.3	9	2.7	3.7	-0.249	-1.5
11/10/2017 11:56 PM	5.7	4.5	8.9	2.6	3.8	-0.239	-1.5
11/10/2017 11:57 PM	5.7	5.9	9.3	2.6	3.6	-0.236	-1.5
11/10/2017 11:58 PM	5.8	5.9	9.4	2.6	3.5	-0.243	-1.5
11/10/2017 11:59 PM	5.9	4.5	9.1	2.6	3.9	-0.246	-1.5
11/11/2017 12:00 AM	5.9	4.2	8.5	2.6	4.3	-0.236	-1.5
11/11/2017 12:01 AM	5.6	6.1	8.8	2.7	4.4	-0.239	-1.5
11/11/2017 12:02 AM	5.5	4	8.6	2.8	4.5	-0.253	-1.5
11/11/2017 12:03 AM	5.3	3.9	8.4	2.8	4.5	-0.239	-1.5
11/11/2017 12:04 AM	5.3	3.4	8.2	2.9	4.7	-0.238	-1.5
11/11/2017 12:05 AM	5.1	5.8	8.2	3	4.9	-0.244	-1.5
11/11/2017 12:06 AM	5.1	3.7	8.4	3	4.9	-0.247	-1.5
11/11/2017 12:07 AM	5	4.2	8.3	3.1	4.5	-0.240	-1.5
11/11/2017 12:08 AM	5.2	3.3	8.7	3.1	4.2	-0.237	-1.5
11/11/2017 12:09 AM	5.3	5.9	8.6	3.2	4.2	-0.244	-1.5
11/11/2017 12:10 AM	5.6	3.4	9.1	3.1	3.8	-0.240	-1.5
11/11/2017 12:11 AM	5.7	4.2	9	3.1	4.1	-0.239	-1.5
11/11/2017 12:12 AM	5.8	3.8	8.6	3	3.9	-0.241	-1.5
11/11/2017 12:13 AM	5.7	4.9	8.8	3	3.8	-0.252	-1.5
11/11/2017 12:14 AM	5.7	5.5	8.8	2.9	4	-0.243	-1.5

11/11/2017 12:15 AM	5.6	4.1	8.6	2.9	3.9	-0.241	-1.5
11/11/2017 12:16 AM	5.6	4.9	8.6	2.8	3.8	-0.245	-1.5
11/11/2017 12:17 AM	5.5	5	8.3	2.8	4	-0.248	-1.5
11/11/2017 12:18 AM	5.5	4.6	8.6	2.7	3.8	-0.237	-1.5
11/11/2017 12:19 AM	5.6	4.5	8.7	2.7	3.8	-0.243	-1.5
11/11/2017 12:20 AM	5.7	5.3	8.8	2.7	3.9	-0.249	-1.5
11/11/2017 12:21 AM	5.9	5.5	9.1	2.7	3.7	-0.244	-1.5
11/11/2017 12:22 AM	6	6.3	9.2	2.6	3.9	-0.235	-1.5
11/11/2017 12:23 AM	6.1	5.3	8.8	2.6	4	-0.250	-1.5
11/11/2017 12:24 AM	6	4.9	8.6	2.6	3.8	-0.249	-1.5
11/11/2017 12:25 AM	5.9	4.9	8.5	2.6	3.9	-0.236	-1.5
11/11/2017 12:26 AM	5.7	4.4	8.9	2.6	3.8	-0.238	-1.5
11/11/2017 12:27 AM	5.7	5.4	8.6	2.6	4.2	-0.249	-1.5
11/11/2017 12:28 AM	5.7	6	8.8	2.6	4	-0.241	-1.5
11/11/2017 12:29 AM	5.8	5.1	8.6	2.6	4.5	-0.236	-1.5
11/11/2017 12:30 AM	5.9	4.8	8.6	2.6	4.3	-0.233	-1.5
11/11/2017 12:31 AM	5.8	5.3	8.2	2.7	4.6	-0.241	-1.5
11/11/2017 12:32 AM	5.5	3.4	7.7	2.8	4.7	-0.237	-1.5
11/11/2017 12:33 AM	5.2	2.2	7.3	2.9	5	-0.237	-1.5
11/11/2017 12:34 AM	5	2.9	7.8	2.9	5.1	-0.245	-1.5
11/11/2017 12:35 AM	5	3.9	8.3	3	4.5	-0.240	-1.5
11/11/2017 12:36 AM	5.2	5.9	8.1	3.1	4.5	-0.233	-1.5
11/11/2017 12:37 AM	5.4	4.7	8.7	3.1	4.6	-0.236	-1.5
11/11/2017 12:38 AM	5.5	3.8	8.3	3.1	4.3	-0.244	-1.5
11/11/2017 12:39 AM	5.5	3.5	7.8	3.1	3.9	-0.239	-1.5
11/11/2017 12:40 AM	5.4	3.6	7.8	3.1	4.2	-0.236	-1.5
11/11/2017 12:41 AM	5.1	4.7	7.6	3	4.2	-0.243	-1.5
11/11/2017 12:42 AM	5	3.7	7.7	3	4.2	-0.242	-1.5
11/11/2017 12:43 AM	4.9	2.3	7.8	2.9	4.4	-0.232	-1.5
11/11/2017 12:44 AM	4.8	4.1	7.5	2.9	3.9	-0.234	-1.5
11/11/2017 12:45 AM	4.9	5.2	7.7	2.8	4.3	-0.244	-1.5
11/11/2017 12:46 AM	4.8	3.7	7.5	2.8	4.5	-0.246	-1.5
11/11/2017 12:47 AM	4.8	3.1	7.6	2.8	4.4	-0.233	-1.5
11/11/2017 12:48 AM	4.5	2.7	7	2.8	4.5	-0.237	-1.5
11/11/2017 12:49 AM	4.2	2.8	6.6	2.7	4.6	-0.243	-1.5
11/11/2017 12:50 AM	4	4.9	7	2.7	4.5	-0.233	-1.5
11/11/2017 12:51 AM	3.9	2.7	6.8	2.7	4.6	-0.230	-1.5
11/11/2017 12:52 AM	3.9	2.1	6.7	2.7	4.7	-0.242	-1.5
11/11/2017 12:53 AM	3.7	3	6.5	2.7	4.9	-0.246	-1.5
11/11/2017 12:54 AM	3.6	2.4	6.3	2.7	4.8	-0.231	-1.5
11/11/2017 12:55 AM	3.4	3.8	6.4	2.7	5	-0.229	-1.5
11/11/2017 12:56 AM	3.4	0	6.5	2.7	5.3	-0.244	-1.5
11/11/2017 12:57 AM	3.2	2.5	5.9	2.7	5.5	-0.235	-1.5
11/11/2017 12:58 AM	3	2.1	5.7	2.8	5.8	-0.230	-1.5
11/11/2017 12:59 AM	2.7	1.5	5.4	2.8	6.2	-0.241	-1.5
11/11/2017 1:00 AM	2.4	0.7	5.1	2.9	7	-0.237	-1.5
11/11/2017 1:01 AM	2.3	0.4	5.3	3	7.7	-0.229	-1.5

11/11/2017 1:02 AM	2.2	1	5.5	3.2	7.4	-0.240	-1.5
11/11/2017 1:03 AM	2.2	2.8	5.4	3.4	7.4	-0.237	-1.5
11/11/2017 1:04 AM	2.2	0.7	5.5	3.5	7	-0.238	-1.5
11/11/2017 1:05 AM	2.2	0.9	5.3	3.6	7.1	-0.231	-1.5
11/11/2017 1:06 AM	2.3	1.2	5.8	3.6	6.9	-0.238	-1.5
11/11/2017 1:07 AM	2.4	1.6	5.8	3.6	6.8	-0.238	-1.5
11/11/2017 1:08 AM	2.4	0.9	5.5	3.6	6.6	-0.229	-1.5
11/11/2017 1:09 AM	2.3	1.2	5.2	3.6	6.5	-0.228	-1.5
11/11/2017 1:10 AM	2.3	-0.8	5.6	3.5	6.4	-0.244	-1.5
11/11/2017 1:11 AM	2.3	0.5	5.5	3.4	6.7	-0.243	-1.5
11/11/2017 1:12 AM	2.4	0.7	5.4	3.3	6.4	-0.233	-1.5
11/11/2017 1:13 AM	2.3	1.2	5.3	3.3	6.6	-0.232	-1.5
11/11/2017 1:14 AM	2.2	0.9	5.4	3.2	6.5	-0.243	-1.5
11/11/2017 1:15 AM	2.1	0.2	5.2	3.2	6.8	-0.234	-1.5
11/11/2017 1:16 AM	2	0.7	5.3	3.1	6.4	-0.231	-1.5
11/11/2017 1:17 AM	2	-0.2	5.5	3.1	6.3	-0.240	-1.5
11/11/2017 1:18 AM	2.1	1.8	5.5	3	6.5	-0.238	-1.5
11/11/2017 1:19 AM	2.1	2.1	5.4	3	6.7	-0.229	-1.5
11/11/2017 1:20 AM	2.1	0.6	5.3	2.9	6.4	-0.242	-1.5
11/11/2017 1:21 AM	2	-0.1	5.4	2.9	6.4	-0.251	-1.5
11/11/2017 1:22 AM	1.9	0	5.3	2.9	6.7	-0.238	-1.5
11/11/2017 1:23 AM	1.9	0.9	5.4	2.9	6.6	-0.230	-1.5
11/11/2017 1:24 AM	2	1.4	5.3	2.9	6.5	-0.246	-1.5
11/11/2017 1:25 AM	2.1	2.1	5.6	2.8	6.4	-0.244	-1.5
11/11/2017 1:26 AM	2.1	0.3	5.5	2.8	6.5	-0.235	-1.5
11/11/2017 1:27 AM	2.1	0.6	5.5	2.8	6.4	-0.238	-1.5
11/11/2017 1:28 AM	2	0.5	5.3	2.8	6.4	-0.240	-1.5
11/11/2017 1:29 AM	2	-0.1	5.5	2.8	6.7	-0.241	-1.5
11/11/2017 1:30 AM	2	1	5.6	2.7	6.2	-0.236	-1.5
11/11/2017 1:31 AM	2	2	5.4	2.7	6.3	-0.245	-1.5
11/11/2017 1:32 AM	2	0.9	5.7	2.7	6.3	-0.243	-1.5
11/11/2017 1:33 AM	2	0.6	5.3	2.7	6.5	-0.243	-1.5
11/11/2017 1:34 AM	2	1.2	5.7	2.6	6.4	-0.243	-1.5
11/11/2017 1:35 AM	2	0.5	5.3	2.6	6.2	-0.255	-1.5
11/11/2017 1:36 AM	1.9	0.9	5.3	2.5	6.3	-0.240	-1.5
11/11/2017 1:37 AM	1.9	0.7	5.4	2.5	5.9	-0.239	-1.5
11/11/2017 1:38 AM	1.8	0.5	5.2	2.5	6	-0.250	-1.5
11/11/2017 1:39 AM	1.8	1.1	5.4	2.4	6.3	-0.252	-1.5
11/11/2017 1:40 AM	1.8	0.6	5.5	2.4	6.6	-0.240	-1.5
11/11/2017 1:41 AM	1.7	0.3	5.3	2.4	6.8	-0.241	-1.5
11/11/2017 1:42 AM	1.6	0.3	5.3	2.3	6.5	-0.251	-1.5
11/11/2017 1:43 AM	1.5	-0.1	5.3	2.3	6.5	-0.237	-1.5
11/11/2017 1:44 AM	1.5	-0.7	5.3	2.2	6.5	-0.243	-1.5
11/11/2017 1:45 AM	1.5	0.7	5.4	2.2	6.5	-0.251	-1.5
11/11/2017 1:46 AM	1.7	0.6	5.7	2.2	6.7	-0.248	-1.5
11/11/2017 1:47 AM	1.7	0.8	5.4	2.2	6.6	-0.233	-1.5
11/11/2017 1:48 AM	1.8	1.3	5.6	2.2	6.6	-0.248	-1.5

11/11/2017 1:49 AM	1.8	0.7	5.7	2.2	6.5	-0.249	-1.5
11/11/2017 1:50 AM	1.8	0.5	5.7	2.2	6.5	-0.246	-1.5
11/11/2017 1:51 AM	1.9	1.2	5.7	2.2	6.6	-0.246	-1.5
11/11/2017 1:52 AM	1.8	1.8	5.5	2.3	6.7	-0.253	-1.5
11/11/2017 1:53 AM	1.8	1.2	5.5	2.3	6.2	-0.252	-1.5
11/11/2017 1:54 AM	1.7	-0.6	5.4	2.3	6.2	-0.241	-1.5
11/11/2017 1:55 AM	1.7	0.3	5.2	2.3	6.5	-0.256	-1.5
11/11/2017 1:56 AM	1.7	0.1	5.1	2.3	6.3	-0.253	-1.5
11/11/2017 1:57 AM	1.6	-0.3	5.2	2.3	6.8	-0.251	-1.5
11/11/2017 1:58 AM	1.5	0.7	5.1	2.4	6.7	-0.250	-1.5
11/11/2017 1:59 AM	1.4	-0.4	5	2.4	6.7	-0.255	-1.5
11/11/2017 2:00 AM	1.3	0.2	5.3	2.4	6.8	-0.252	-1.5
11/11/2017 2:01 AM	1.3	-0.4	5.4	2.4	6.6	-0.246	-1.5
11/11/2017 2:02 AM	1.3	0.6	5.2	2.4	7	-0.254	-1.5
11/11/2017 2:03 AM	1.2	1.3	5.2	2.4	7.3	-0.252	-1.5
11/11/2017 2:04 AM	1.2	1.2	5.3	2.3	6.9	-0.247	-1.5
11/11/2017 2:05 AM	1.1	0.9	5.3	2.3	6.6	-0.251	-1.5
11/11/2017 2:06 AM	1.1	-1.7	5.1	2.4	6.9	-0.258	-1.5
11/11/2017 2:07 AM	1.2	1.8	5.5	2.4	6.8	-0.249	-1.5
11/11/2017 2:08 AM	1.4	0.9	5.8	2.4	6.9	-0.247	-1.5
11/11/2017 2:09 AM	1.6	1.4	6	2.4	6.5	-0.255	-1.5
11/11/2017 2:10 AM	2	1.3	6.3	2.4	6.5	-0.255	-1.5
11/11/2017 2:11 AM	2.2	1.7	6.2	2.4	6.3	-0.241	-1.5
11/11/2017 2:12 AM	2.3	1.2	6.2	2.4	5.9	-0.256	-1.5
11/11/2017 2:13 AM	2.4	1.4	6.1	2.5	5.9	-0.255	-1.5
11/11/2017 2:14 AM	2.4	2.4	6.1	2.5	5.9	-0.255	-1.5
11/11/2017 2:15 AM	2.3	0.7	6	2.5	5.8	-0.251	-1.5
11/11/2017 2:16 AM	2.2	1.8	5.8	2.5	5.7	-0.257	-1.5
11/11/2017 2:17 AM	2	2.8	6	2.5	5.9	-0.256	-1.5
11/11/2017 2:18 AM	2	0	5.8	2.5	5.9	-0.252	-1.5
11/11/2017 2:19 AM	2.1	1.9	6	2.5	5.8	-0.261	-1.5
11/11/2017 2:20 AM	2.2	1.1	6.2	2.5	5.8	-0.266	-1.5
11/11/2017 2:21 AM	2.3	1.2	5.8	2.5	5.6	-0.251	-1.5
11/11/2017 2:22 AM	2.3	1.1	6	2.4	5.4	-0.256	-1.5
11/11/2017 2:23 AM	2.2	1.3	5.8	2.5	6	-0.265	-1.5
11/11/2017 2:24 AM	2.2	1.4	5.8	2.5	5.9	-0.250	-1.5
11/11/2017 2:25 AM	2.1	-1.3	5.8	2.5	5.8	-0.251	-1.5
11/11/2017 2:26 AM	2	0.9	5.6	2.5	5.8	-0.258	-1.5
11/11/2017 2:27 AM	2	-0.4	5.6	2.5	6	-0.259	-1.5
11/11/2017 2:28 AM	2	0.2	5.7	2.5	6	-0.252	-1.5
11/11/2017 2:29 AM	1.9	2.3	5.7	2.5	6.4	-0.258	-1.5
11/11/2017 2:30 AM	1.8	0.3	5.5	2.5	6.5	-0.260	-1.5
11/11/2017 2:31 AM	1.8	0.2	5.5	2.6	6.7	-0.246	-1.5
11/11/2017 2:32 AM	1.7	1.4	5.5	2.6	6.7	-0.251	-1.5
11/11/2017 2:33 AM	1.7	-0.9	5.7	2.6	6.7	-0.257	-1.5
11/11/2017 2:34 AM	1.6	0.9	5.5	2.6	6.5	-0.248	-1.5
11/11/2017 2:35 AM	1.6	0.1	5.5	2.6	6.7	-0.248	-1.5

11/11/2017 2:36 AM	1.6	0.3	5.6	2.6	6.6	-0.256	-1.5
11/11/2017 2:37 AM	1.5	0.4	5.3	2.6	6.7	-0.254	-1.5
11/11/2017 2:38 AM	1.5	-0.2	5.4	2.6	6.8	-0.246	-1.5
11/11/2017 2:39 AM	1.4	0.3	5.4	2.6	7.3	-0.249	-1.5
11/11/2017 2:40 AM	1.4	0.8	5.2	2.7	7.4	-0.262	-1.5
11/11/2017 2:41 AM	1.3	0	4.9	2.7	7.2	-0.251	-1.5
11/11/2017 2:42 AM	1.3	0.1	5.3	2.8	7.3	-0.248	-1.5
11/11/2017 2:43 AM	1.3	-0.3	5.2	2.9	7.4	-0.255	-1.5
11/11/2017 2:44 AM	1.3	0.5	5.1	2.9	7.3	-0.259	-1.5
11/11/2017 2:45 AM	1.4	0.4	5.5	2.9	7.1	-0.246	-1.5
11/11/2017 2:46 AM	1.5	1.4	5.8	2.9	7.2	-0.251	-1.5
11/11/2017 2:47 AM	1.7	0.8	5.8	2.9	6.8	-0.257	-1.5
11/11/2017 2:48 AM	1.8	1.2	5.4	2.9	7.2	-0.251	-1.5
11/11/2017 2:49 AM	1.8	-0.9	5.8	2.9	7.1	-0.248	-1.5
11/11/2017 2:50 AM	1.8	-0.3	5.4	2.9	7.2	-0.254	-1.5
11/11/2017 2:51 AM	1.7	0	5.2	2.8	7.1	-0.257	-1.5
11/11/2017 2:52 AM	1.6	0.5	5.5	2.8	7.1	-0.247	-1.5
11/11/2017 2:53 AM	1.5	0.3	5.2	2.9	7.1	-0.258	-1.5
11/11/2017 2:54 AM	1.4	-0.1	5.4	2.8	7.4	-0.260	-1.5
11/11/2017 2:55 AM	1.4	0.9	5.6	2.8	7.3	-0.244	-1.5
11/11/2017 2:56 AM	1.5	0.7	5.4	2.7	7.2	-0.252	-1.5
11/11/2017 2:57 AM	1.5	1	5.5	2.7	7.1	-0.269	-1.5
11/11/2017 2:58 AM	1.4	0	5.3	2.7	7.2	-0.265	-1.5
11/11/2017 2:59 AM	1.4	1.9	5.6	2.6	7.1	-0.258	-1.5
11/11/2017 3:00 AM	1.5	0.4	5.7	2.6	7	-0.259	-1.5
11/11/2017 3:01 AM	1.6	0.9	5.7	2.6	6.6	-0.266	-1.5
11/11/2017 3:02 AM	1.6	1	5.6	2.5	7.1	-0.259	-1.5
11/11/2017 3:03 AM	1.7	1.6	5.8	2.5	6.7	-0.248	-1.5
11/11/2017 3:04 AM	1.7	-0.1	5.4	2.5	6.8	-0.262	-1.5
11/11/2017 3:05 AM	1.6	0.8	5.5	2.5	6.7	-0.259	-1.5
11/11/2017 3:06 AM	1.6	0	5.7	2.5	6.7	-0.261	-1.5
11/11/2017 3:07 AM	1.6	-0.3	5.8	2.5	6.9	-0.258	-1.5
11/11/2017 3:08 AM	1.7	1	6	2.5	6.5	-0.265	-1.5
11/11/2017 3:09 AM	1.8	0.5	6.1	2.4	6.7	-0.257	-1.5
11/11/2017 3:10 AM	1.9	1.6	5.9	2.4	6.5	-0.255	-1.5
11/11/2017 3:11 AM	2	1.9	5.7	2.4	6.3	-0.266	-1.5
11/11/2017 3:12 AM	2	0.2	6.1	2.4	6	-0.269	-1.5
11/11/2017 3:13 AM	1.9	1.8	5.9	2.4	6.1	-0.259	-1.5
11/11/2017 3:14 AM	1.9	0.8	5.8	2.4	6.1	-0.262	-1.5
11/11/2017 3:15 AM	1.9	-1.4	5.9	2.3	6.2	-0.267	-1.5
11/11/2017 3:16 AM	1.8	1.9	5.7	2.3	6.1	-0.260	-1.5
11/11/2017 3:17 AM	1.7	0.1	5.7	2.3	6	-0.254	-1.5
11/11/2017 3:18 AM	1.7	2.4	5.7	2.3	5.9	-0.271	-1.5
11/11/2017 3:19 AM	1.8	1	5.9	2.3	6.1	-0.265	-1.5
11/11/2017 3:20 AM	1.9	0.1	5.9	2.3	5.9	-0.260	-1.5
11/11/2017 3:21 AM	1.9	0.6	6.2	2.3	5.9	-0.259	-1.5
11/11/2017 3:22 AM	1.9	0.3	6.1	2.3	5.8	-0.270	-1.5

11/11/2017 3:23 AM	1.9	1.1	6.1	2.3	6.1	-0.262	-1.5
11/11/2017 3:24 AM	1.8	-0.7	5.9	2.3	5.7	-0.263	-1.5
11/11/2017 3:25 AM	1.8	1.7	5.8	2.3	6	-0.267	-1.5
11/11/2017 3:26 AM	1.7	0.6	6.2	2.3	5.8	-0.264	-1.5
11/11/2017 3:27 AM	1.8	0.1	6.1	2.2	5.6	-0.257	-1.5
11/11/2017 3:28 AM	1.8	0.1	6.3	2.2	5.7	-0.261	-1.5
11/11/2017 3:29 AM	1.9	-0.1	6	2.2	5.9	-0.267	-1.5
11/11/2017 3:30 AM	1.9	0.7	6	2.2	5.7	-0.263	-1.5
11/11/2017 3:31 AM	1.9	1.6	5.8	2.2	5.8	-0.260	-1.5
11/11/2017 3:32 AM	1.8	1.8	6	2.2	5.7	-0.268	-1.5
11/11/2017 3:33 AM	1.7	0.1	5.9	2.2	5.9	-0.271	-1.5
11/11/2017 3:34 AM	1.7	0.3	6.2	2.2	6.1	-0.257	-1.5
11/11/2017 3:35 AM	1.7	1.3	5.8	2.2	6.1	-0.268	-1.5
11/11/2017 3:36 AM	1.6	0.4	5.9	2.2	6.2	-0.263	-1.5
11/11/2017 3:37 AM	1.6	-0.5	6	2.2	6.4	-0.258	-1.5
11/11/2017 3:38 AM	1.6	1.3	6.1	2.2	6.4	-0.263	-1.5
11/11/2017 3:39 AM	1.7	-0.4	6	2.2	6.3	-0.268	-1.5
11/11/2017 3:40 AM	1.7	0.5	6.2	2.2	7.2	-0.255	-1.5
11/11/2017 3:41 AM	1.7	0.5	6	2.3	7.3	-0.261	-1.5
11/11/2017 3:42 AM	1.9	2	6.7	2.5	7.4	-0.264	-1.5
11/11/2017 3:43 AM	2.2	3.7	6.9	2.6	7.6	-0.257	-1.5
11/11/2017 3:44 AM	2.5	1.8	6.7	2.7	7.1	-0.261	-1.5
11/11/2017 3:45 AM	2.6	1.3	6.7	2.9	6.7	-0.254	-1.5
11/11/2017 3:46 AM	2.7	1.1	7.2	3	6.4	-0.261	-1.5
11/11/2017 3:47 AM	3	2.3	7.2	3.1	6.5	-0.253	-1.5
11/11/2017 3:48 AM	3.2	3	7.2	3.1	5.8	-0.254	-1.5
11/11/2017 3:49 AM	3.5	1.5	8.1	3.1	5.4	-0.260	-1.5
11/11/2017 3:50 AM	3.8	2.5	7.9	3.1	5	-0.267	-1.5
11/11/2017 3:51 AM	4	6	8.2	3.1	4.9	-0.250	-1.5
11/11/2017 3:52 AM	4.3	3.4	8.2	3.1	4.8	-0.257	-1.5
11/11/2017 3:53 AM	4.3	4.4	8	3	4.7	-0.261	-1.5
11/11/2017 3:54 AM	4.3	4.8	7.6	3	4.6	-0.251	-1.5
11/11/2017 3:55 AM	4.3	4.2	8.4	3	4.5	-0.255	-1.5
11/11/2017 3:56 AM	4.4	3.7	8.3	2.9	4.5	-0.256	-1.5
11/11/2017 3:57 AM	4.6	4.3	8.7	2.9	4.5	-0.263	-1.5
11/11/2017 3:58 AM	4.7	4.5	8.3	2.8	4.3	-0.255	-1.5
11/11/2017 3:59 AM	4.7	4.1	8.5	2.8	4.2	-0.262	-1.5
11/11/2017 4:00 AM	4.6	2.6	8	2.8	4.1	-0.268	-1.5
11/11/2017 4:01 AM	4.6	2.5	8.3	2.8	4.1	-0.255	-1.5
11/11/2017 4:02 AM	4.6	2.9	7.9	2.8	4.2	-0.254	-1.5
11/11/2017 4:03 AM	4.4	3.9	7.6	2.8	4.2	-0.265	-1.5
11/11/2017 4:04 AM	4.2	1.6	7.5	2.7	4.2	-0.258	-1.5
11/11/2017 4:05 AM	4	3.2	7.4	2.7	4.3	-0.255	-1.5
11/11/2017 4:06 AM	3.9	4	7.4	2.7	4.5	-0.261	-1.5
11/11/2017 4:07 AM	3.8	2.2	7.4	2.7	4.1	-0.257	-1.5
11/11/2017 4:08 AM	3.9	4	8	2.6	4.1	-0.246	-1.5
11/11/2017 4:09 AM	4.1	2.6	7.5	2.6	4.3	-0.264	-1.5

11/11/2017 4:10 AM	4.2	2.7	7.7	2.6	4.1	-0.263	-1.5
11/11/2017 4:11 AM	4.1	3.3	7.7	2.6	4.3	-0.256	-1.5
11/11/2017 4:12 AM	4.1	2.1	7.5	2.6	4.5	-0.255	-1.5
11/11/2017 4:13 AM	4.1	2.6	7.4	2.6	4.6	-0.262	-1.5
11/11/2017 4:14 AM	4.1	5.4	7.8	2.6	4.8	-0.261	-1.5
11/11/2017 4:15 AM	4.2	3.1	7.9	2.7	5.2	-0.258	-1.5
11/11/2017 4:16 AM	4.4	1.5	7.8	2.8	5.2	-0.258	-1.5
11/11/2017 4:17 AM	4.3	0.8	7.3	2.8	5.4	-0.265	-1.5
11/11/2017 4:18 AM	4.1	2.4	7.5	2.9	5.5	-0.254	-1.5
11/11/2017 4:19 AM	3.9	3.6	7.3	3	5.5	-0.259	-1.5
11/11/2017 4:20 AM	3.9	3.4	7.6	3.2	5.3	-0.270	-1.5
11/11/2017 4:21 AM	4	3	8	3.2	5.3	-0.257	-1.5
11/11/2017 4:22 AM	4.2	5.2	8	3.3	5	-0.260	-1.5
11/11/2017 4:23 AM	4.5	4.4	8.1	3.3	4.5	-0.268	-1.5
11/11/2017 4:24 AM	4.7	4.4	8.6	3.3	4.6	-0.260	-1.5
11/11/2017 4:25 AM	4.9	5	8.3	3.2	4.4	-0.256	-1.5
11/11/2017 4:26 AM	5.2	4.5	8.8	3.1	4.1	-0.260	-1.5
11/11/2017 4:27 AM	5.2	4.8	8.5	3.1	4.4	-0.273	-1.5
11/11/2017 4:28 AM	5.3	3.7	8.6	3	4	-0.262	-1.5
11/11/2017 4:29 AM	5.2	4.9	8.6	3	3.8	-0.260	-1.5
11/11/2017 4:30 AM	5.3	4.8	9.1	2.9	4	-0.269	-1.5
11/11/2017 4:31 AM	5.4	6	9.1	2.8	3.5	-0.268	-1.5
11/11/2017 4:32 AM	5.6	4.6	8.9	2.8	3.7	-0.262	-1.5
11/11/2017 4:33 AM	5.8	5	9.1	2.7	3.5	-0.271	-1.5
11/11/2017 4:34 AM	5.6	3.5	8.6	2.7	3.7	-0.268	-1.5
11/11/2017 4:35 AM	5.5	4.2	8.6	2.7	3.8	-0.264	-1.5
11/11/2017 4:36 AM	5.4	6.6	9.3	2.6	3.6	-0.263	-1.5
11/11/2017 4:37 AM	5.6	4.8	9.4	2.6	3.9	-0.270	-1.5
11/11/2017 4:38 AM	5.8	4.4	9.3	2.5	3.5	-0.264	-1.5
11/11/2017 4:39 AM	6.1	5.4	9.8	2.5	3.3	-0.272	-1.5
11/11/2017 4:40 AM	6.2	4.5	9.4	2.5	3.5	-0.274	-1.5
11/11/2017 4:41 AM	6.1	6.2	9.3	2.5	3.6	-0.269	-1.5
11/11/2017 4:42 AM	6.3	5.3	10.4	2.5	3.5	-0.260	-1.5
11/11/2017 4:43 AM	6.6	5.8	10.1	2.5	3.6	-0.279	-1.5
11/11/2017 4:44 AM	6.8	4.7	9.4	2.4	3.6	-0.272	-1.5
11/11/2017 4:45 AM	6.5	5.3	9.3	2.4	3.4	-0.266	-1.5
11/11/2017 4:46 AM	6.3	5.8	9.6	2.4	3.6	-0.272	-1.5
11/11/2017 4:47 AM	6.4	6.6	10.2	2.4	3.5	-0.272	-1.5
11/11/2017 4:48 AM	6.7	7.5	10.1	2.4	3.3	-0.265	-1.5
11/11/2017 4:49 AM	7	6.4	10.4	2.4	3.4	-0.274	-1.5
11/11/2017 4:50 AM	7.1	4.2	10.1	2.4	3.7	-0.271	-1.5
11/11/2017 4:51 AM	6.9	5.8	9.5	2.4	3.7	-0.256	-1.5
11/11/2017 4:52 AM	6.9	5.8	9.9	2.4	3.7	-0.269	-1.5
11/11/2017 4:53 AM	6.7	5.6	9.5	2.5	4.5	-0.279	-1.5
11/11/2017 4:54 AM	6.5	4.5	9.5	2.6	4.3	-0.268	-1.5
11/11/2017 4:55 AM	6.5	4.5	9.8	2.7	4.2	-0.263	-1.5
11/11/2017 4:56 AM	6.3	5.6	9.2	2.7	4.7	-0.278	-1.5

11/11/2017 4:57 AM	6.3	5.4	9.4	2.8	4.6	-0.267	-1.5
11/11/2017 4:58 AM	6.2	6.5	9.7	2.9	4.3	-0.263	-1.5
11/11/2017 4:59 AM	6.3	4.4	10.1	2.9	4	-0.284	-1.5
11/11/2017 5:00 AM	6.3	5.9	9.5	2.9	3.9	-0.272	-1.5
11/11/2017 5:01 AM	6.4	5.1	9.6	2.9	3.7	-0.268	-1.5
11/11/2017 5:02 AM	6.4	6.8	9.8	2.9	3.4	-0.283	-1.5
11/11/2017 5:03 AM	6.4	5.8	9.5	2.9	3.6	-0.274	-1.5
11/11/2017 5:04 AM	6.4	4.5	9.8	2.8	3.4	-0.268	-1.5
11/11/2017 5:05 AM	6.6	5.7	10.1	2.7	3.2	-0.277	-1.5
11/11/2017 5:06 AM	6.7	7.8	10.1	2.6	3.5	-0.282	-1.5
11/11/2017 5:07 AM	6.8	5.2	10	2.6	3.2	-0.267	-1.5
11/11/2017 5:08 AM	6.8	5.6	9.8	2.5	3.5	-0.272	-1.5
11/11/2017 5:09 AM	6.8	4.2	9.2	2.5	3.5	-0.282	-1.5
11/11/2017 5:10 AM	6.6	4.7	9.1	2.4	3.4	-0.269	-1.5
11/11/2017 5:11 AM	6.4	4.4	9.8	2.4	3.4	-0.273	-1.5
11/11/2017 5:12 AM	6.4	7.5	9.7	2.3	3.3	-0.283	-1.5
11/11/2017 5:13 AM	6.8	5.8	10.4	2.3	3.2	-0.276	-1.5
11/11/2017 5:14 AM	7	4.7	9.7	2.3	3.2	-0.272	-1.5
11/11/2017 5:15 AM	7	6.5	10	2.3	3.5	-0.278	-1.5
11/11/2017 5:16 AM	7	6.1	10.5	2.3	3.3	-0.280	-1.5
11/11/2017 5:17 AM	7.1	7.1	10.7	2.2	3.6	-0.274	-1.5
11/11/2017 5:18 AM	7.3	6.6	10.7	2.2	3.3	-0.268	-1.5
11/11/2017 5:19 AM	7.5	6	10.8	2.2	3.1	-0.285	-1.5
11/11/2017 5:20 AM	7.5	6.4	10	2.2	3.2	-0.270	-1.5
11/11/2017 5:21 AM	7.4	5.2	10.6	2.2	3.3	-0.271	-1.5
11/11/2017 5:22 AM	7.4	6.3	10.3	2.2	3.5	-0.286	-1.5
11/11/2017 5:23 AM	7.2	5.2	10.2	2.2	3.3	-0.271	-1.5
11/11/2017 5:24 AM	7.2	6.5	10.4	2.2	3.4	-0.275	-1.5
11/11/2017 5:25 AM	7.3	6.6	10.9	2.2	3.3	-0.278	-1.5
11/11/2017 5:26 AM	7.6	6.3	10.3	2.2	3	-0.283	-1.5
11/11/2017 5:27 AM	7.7	5.5	10.7	2.2	3.1	-0.270	-1.5
11/11/2017 5:28 AM	7.7	6.4	10.6	2.2	3.3	-0.275	-1.5
11/11/2017 5:29 AM	7.5	5.1	9.8	2.2	3.3	-0.290	-1.5
11/11/2017 5:30 AM	7.4	7.2	10.3	2.2	3.4	-0.273	-1.5
11/11/2017 5:31 AM	7.2	4.5	10.1	2.2	3.3	-0.272	-1.5
11/11/2017 5:32 AM	7.2	6.1	10.3	2.2	3.3	-0.282	-1.5
11/11/2017 5:33 AM	7.3	6.2	10.7	2.2	3.1	-0.281	-1.5
11/11/2017 5:34 AM	7.5	6.4	11	2.2	3.3	-0.274	-1.5
11/11/2017 5:35 AM	7.6	6.9	10.8	2.2	3.6	-0.278	-1.5
11/11/2017 5:36 AM	7.7	7.4	10.3	2.2	4	-0.281	-1.5
11/11/2017 5:37 AM	7.6	6.9	10.5	2.3	3.9	-0.274	-1.5
11/11/2017 5:38 AM	7.4	5.4	9.9	2.4	4	-0.283	-1.5
11/11/2017 5:39 AM	7.1	6.3	9.9	2.4	4	-0.285	-1.5
11/11/2017 5:40 AM	6.9	6.3	9.6	2.5	4.5	-0.269	-1.5
11/11/2017 5:41 AM	6.7	4.7	9.9	2.6	4.3	-0.276	-1.5
11/11/2017 5:42 AM	6.9	6.4	10.7	2.7	4.1	-0.286	-1.5
11/11/2017 5:43 AM	7.1	8.3	10.6	2.8	3.7	-0.280	-1.5

11/11/2017 5:44 AM	7.3	6.8	10.1	2.8	3.4	-0.275	-1.5
11/11/2017 5:45 AM	7.4	5.5	10.4	2.8	3.6	-0.284	-1.5
11/11/2017 5:46 AM	7.1	6.8	9.6	2.7	3.6	-0.278	-1.5
11/11/2017 5:47 AM	7.1	6.7	10.3	2.7	3.6	-0.271	-1.5
11/11/2017 5:48 AM	7.3	7.2	10.6	2.6	3.2	-0.289	-1.5
11/11/2017 5:49 AM	7.6	7.1	10.7	2.5	3.5	-0.282	-1.5
11/11/2017 5:50 AM	7.8	6.8	10.4	2.5	3.2	-0.268	-1.5
11/11/2017 5:51 AM	7.7	6.3	10.6	2.4	3.2	-0.281	-1.5
11/11/2017 5:52 AM	7.6	4.9	10.6	2.4	3.4	-0.286	-1.5
11/11/2017 5:53 AM	7.6	6.9	10.4	2.3	3.4	-0.281	-1.5
11/11/2017 5:54 AM	7.5	7.8	10.5	2.3	3.4	-0.283	-1.5
11/11/2017 5:55 AM	7.3	5.7	9.8	2.2	3.4	-0.286	-1.5
11/11/2017 5:56 AM	7.2	6.4	10.1	2.2	3.1	-0.276	-1.5
11/11/2017 5:57 AM	7.1	5.5	10	2.2	3.2	-0.279	-1.5
11/11/2017 5:58 AM	7.1	7.5	10.5	2.2	3.4	-0.285	-1.5
11/11/2017 5:59 AM	7.3	5.8	10.6	2.1	3.4	-0.285	-1.5
11/11/2017 6:00 AM	7.5	6.7	10.6	2.1_Zero	3.1	-0.272	-1.5
11/11/2017 6:01 AM	7.8	6.6	11.4	2.1_Zero	3.3	-0.286	-1.5
11/11/2017 6:02 AM	8	7.7	11.2	2.1_Zero	3.2	-0.279	-1.5
11/11/2017 6:03 AM	8.1	7.7	11	2.1_Zero	3	-0.274	-1.5
11/11/2017 6:04 AM	8.1	7.1	11	2.1_Zero	3.3	-0.279	-1.5
11/11/2017 6:05 AM	7.9	6.8	10.4	2.1_Zero	3.3	-0.281	-1.5
11/11/2017 6:06 AM	7.8	7.5	10.8	2.1_Zero	3.1	-0.276	-1.5
11/11/2017 6:07 AM	7.8	7.7	10.9	2.1_Zero	3.1	-0.278	-1.5
11/11/2017 6:08 AM	7.9	7.6	10.3	2.1_Zero	3.5	-0.286	-1.5
11/11/2017 6:09 AM	7.8	5.5	10.7	2.1_Zero	3.3	-0.279	-1.5
11/11/2017 6:10 AM	7.8	7	10.9	2.1_Zero	3	-0.273	-1.5
11/11/2017 6:11 AM	7.8	6.6	10.6	2.1_Zero	3.1	-0.285	-1.5
11/11/2017 6:12 AM	7.7	6.9	10.2	2.1_Zero	3.2	-0.285	-1.5
11/11/2017 6:13 AM	7.5	5.9	10.1	2.1_Zero	3.1	-0.272	-1.5
11/11/2017 6:14 AM	7.3	6.4	10.5	2.1_Zero	3.3	-0.280	-1.5
11/11/2017 6:15 AM	7.3	5.3	10.3	2.1_Span	3.1	-0.284	-1.5
11/11/2017 6:16 AM	7.4	8.1	10.8	2.1_Span	3.4	-0.279	-1.5
11/11/2017 6:17 AM	7.6	7.6	10.6	2.1_Span	3.2	-0.279	-1.5
11/11/2017 6:18 AM	7.7	6.9	10.7	2.1_Span	3.2	-0.282	-1.5
11/11/2017 6:19 AM	7.7	7.2	10.6	2.1_Span	3.2	-0.275	-1.5
11/11/2017 6:20 AM	7.7	7.6	10.8	2.1_Span	3	-0.276	-1.5
11/11/2017 6:21 AM	7.9	7	11.1	2.1_Span	3.4	-0.282	-1.5
11/11/2017 6:22 AM	8	6.3	10.9	2.1_Span	3	-0.274	-1.5
11/11/2017 6:23 AM	8	6.2	10.6	2.1_Span	3.2	-0.279	-1.5
11/11/2017 6:24 AM	7.9	7.3	11.3	2.1_Span	3.2	-0.284	-1.5
11/11/2017 6:25 AM	8	8	11.1	2.1_Span	3.2	-0.283	-1.5
11/11/2017 6:26 AM	8.1	7.8	11.1	2.1_Span	3.5	-0.275	-1.5
11/11/2017 6:27 AM	8.2	6.9	10.9	2.1_Span	3.8	-0.275	-1.5
11/11/2017 6:28 AM	8	6.4	10.2	2.2_Span	4.2	-0.288	-1.5
11/11/2017 6:29 AM	7.7	6.6	10	2.3_Span	4	-0.278	-1.5
11/11/2017 6:30 AM	7.4	6.1	9.9	2.4_Span1	4.2	-0.278	-1.5

11/11/2017 6:31 AM	7.2	6.4	10	2.5_Span1	4.2	-0.286	-1.5
11/11/2017 6:32 AM	7.2	6.7	10.1	2.6_Span1	4.3	-0.282	-1.5
11/11/2017 6:33 AM	7.2	6.7	10.3	2.7_Span1	4.1	-0.275	-1.5
11/11/2017 6:34 AM	7.2	5.4	10.2	2.8_Span1	3.8	-0.280	-1.5
11/11/2017 6:35 AM	7.1	7.4	10.3	2.8_Span1	3.5	-0.289	-1.5
11/11/2017 6:36 AM	7.3	7.3	11.2	2.8_Span1	3.4	-0.277	-1.5
11/11/2017 6:37 AM	7.6	8	10.8	2.8_Span1	3.5	-0.279	-1.5
11/11/2017 6:38 AM	7.9	6.2	10.6	2.7_Span1	3.6	-0.285	-1.5
11/11/2017 6:39 AM	7.8	6.1	10.8	2.6_Span1	3.3	-0.275	-1.5
11/11/2017 6:40 AM	7.8	7	10.9	2.5_Span1	3.2	-0.282	-1.5
11/11/2017 6:41 AM	7.8	5.9	10.6	2.4_Span1	3.2	-0.286	-1.5
11/11/2017 6:42 AM	7.7	6.6	10.4	2.4_Span1	3.2	-0.280	-1.5
11/11/2017 6:43 AM	7.5	7.4	10.9	2.3_Span1	3.3	-0.278	-1.5
11/11/2017 6:44 AM	7.5	6.5	10.6	2.3_Span1	3.2	-0.282	-1.5
11/11/2017 6:45 AM	7.7	9	11.5	2.3_Span2	3	-0.287	-1.5
11/11/2017 6:46 AM	8.1	6.3	11.6	2.2_Span2	3	-0.276	-1.5
11/11/2017 6:47 AM	8.3	6.7	11	2.2_Span2	3	-0.281	-1.5
11/11/2017 6:48 AM	8.5	8.4	10.8	2.2_Span2	3.1	-0.289	-1.5
11/11/2017 6:49 AM	8.2	6.1	10.3	2.2_Span2	3.1	-0.277	-1.5
11/11/2017 6:50 AM	7.8	5.7	9.9	2.1_Span2	3.4	-0.278	-1.5
11/11/2017 6:51 AM	7.4	6.7	9.9	2.1_Span2	3.1	-0.288	-1.5
11/11/2017 6:52 AM	7.2	5.7	10.1	2.1_Span2	3.3	-0.286	-1.5
11/11/2017 6:53 AM	7.2	6.4	10	2.1_Span2	3.5	-0.273	-1.5
11/11/2017 6:54 AM	7.1	5.7	9.5	2.1_Span2	3.7	-0.282	-1.5
11/11/2017 6:55 AM	7	5.2	9.7	2.1_Span2	3.8	-0.293	-1.5
11/11/2017 6:56 AM	7	6.7	9.9	2.1_Span2	3.5	-0.277	-1.5
11/11/2017 6:57 AM	7	6	9.7	2.1_Span2	3.6	-0.282	-1.5
11/11/2017 6:58 AM	7	6.4	9.9	2.1_Span2	3.6	-0.288	-1.5
11/11/2017 6:59 AM	7.1	6	10.2	2.1_Span2	3.2	-0.285	-1.5
11/11/2017 7:00 AM	7.3	7.5	10.5	2.1_Purge	3.2	-0.282	-1.5
11/11/2017 7:01 AM	7.5	7.3	10.4	2.1_Purge	3.3	-0.284	-1.5
11/11/2017 7:02 AM	7.7	7.3	11.1	2.1_Purge	3.1	-0.281	-1.5
11/11/2017 7:03 AM	7.9	7.9	11	2.2_Purge	3.4	-0.279	-1.5
11/11/2017 7:04 AM	8.2	7.5	11.1	2.3_Purge	3	-0.285	-1.5
11/11/2017 7:05 AM	8.4	7.7	11	2.3	3.1	-0.290	-1.5
11/11/2017 7:06 AM	8.4	6.2	10.9	2.3	3.4	-0.279	-1.5
11/11/2017 7:07 AM	8.1	6.9	10.7	2.3	3.3	-0.279	-1.5
11/11/2017 7:08 AM	7.9	6.7	10.5	2.3	3.6	-0.293	-1.5
11/11/2017 7:09 AM	7.8	6.3	10.8	2.3	3.3	-0.282	-1.5
11/11/2017 7:10 AM	7.9	6.6	11	2.2	3.1	-0.278	-1.5
11/11/2017 7:11 AM	8	7.8	10.9	2.2	3.1	-0.290	-1.5
11/11/2017 7:12 AM	8.2	8.2	11.2	2.2	3.1	-0.286	-1.5
11/11/2017 7:13 AM	8.2	6.7	11.2	2.3	3.2	-0.279	-1.5
11/11/2017 7:14 AM	8.5	7.4	11.5	2.2	3.2	-0.283	-1.5
11/11/2017 7:15 AM	8.4	8.2	11	2.2	3.2	-0.291	-1.5
11/11/2017 7:16 AM	8.4	7.1	11	2.2	3.1	-0.280	-1.5
11/11/2017 7:17 AM	8.3	7	11.2	2.2	3.2	-0.278	-1.5

11/11/2017 7:18 AM	8	7.3	10.2	2.2	3.1	-0.288	-1.5
11/11/2017 7:19 AM	7.7	6.1	10.3	2.2	3.6	-0.290	-1.5
11/11/2017 7:20 AM	7.5	6.1	10.1	2.2	3.6	-0.278	-1.5
11/11/2017 7:21 AM	7.3	5.7	9.7	2.3	3.9	-0.284	-1.5
11/11/2017 7:22 AM	7.2	7.7	10.2	2.4	4	-0.287	-1.5
11/11/2017 7:23 AM	7.2	6.4	10.8	2.5	3.8	-0.285	-1.5
11/11/2017 7:24 AM	7.4	7.4	10.4	2.6	4.4	-0.277	-1.5
11/11/2017 7:25 AM	7.6	6.3	10.4	2.7	4.3	-0.290	-1.5
11/11/2017 7:26 AM	7.7	6.9	10.6	2.8	3.9	-0.277	-1.5
11/11/2017 7:27 AM	7.7	5.5	10.8	2.9	3.7	-0.279	-1.5
11/11/2017 7:28 AM	7.9	6.1	10.7	3	3.6	-0.288	-1.5
11/11/2017 7:29 AM	7.8	5.9	9.9	3	3.7	-0.284	-1.5
11/11/2017 7:30 AM	7.5	5.2	9.4	2.9	3.7	-0.281	-1.5
11/11/2017 7:31 AM	7.1	6.6	9.3	2.9	3.3	-0.279	-1.5
11/11/2017 7:32 AM	6.9	5.3	9.9	2.8	3.3	-0.291	-1.5
11/11/2017 7:33 AM	6.9	5.4	9.6	2.7	3.6	-0.279	-1.5
11/11/2017 7:34 AM	6.9	6	9.6	2.7	3.5	-0.285	-1.5
11/11/2017 7:35 AM	6.8	5.7	9.4	2.6	3.5	-0.289	-1.5
11/11/2017 7:36 AM	6.7	5.2	9.3	2.5	3.5	-0.281	-1.5
11/11/2017 7:37 AM	6.7	4.5	9.6	2.5	3.6	-0.282	-1.5
11/11/2017 7:38 AM	6.7	6.5	9.6	2.5	3.8	-0.292	-1.5
11/11/2017 7:39 AM	6.9	6.3	9.9	2.5	3.3	-0.288	-1.5
11/11/2017 7:40 AM	7	6.7	10	2.4	3.3	-0.284	-1.5
11/11/2017 7:41 AM	7.3	5.5	10.1	2.4	3.2	-0.285	-1.5
11/11/2017 7:42 AM	7.3	5.8	10	2.4	3.4	-0.293	-1.5
11/11/2017 7:43 AM	7.4	5.8	10.5	2.4	3.4	-0.282	-1.5
11/11/2017 7:44 AM	7.5	6.5	10.4	2.3	3.2	-0.284	-1.5
11/11/2017 7:45 AM	7.5	6.6	10.2	2.4	3.4	-0.289	-1.5
11/11/2017 7:46 AM	7.6	7.4	11	2.3	3.3	-0.284	-1.5
11/11/2017 7:47 AM	7.8	6.5	10.8	2.3	3.3	-0.279	-1.5
11/11/2017 7:48 AM	7.9	6.5	10	2.3	3.3	-0.290	-1.5
11/11/2017 7:49 AM	7.8	7.3	10.4	2.3	3.3	-0.288	-1.5
11/11/2017 7:50 AM	7.7	7.9	11	2.3	3.1	-0.281	-1.5
11/11/2017 7:51 AM	8	7.5	11	2.3	3.2	-0.292	-1.5
11/11/2017 7:52 AM	8.3	7.7	11.2	2.3	3.3	-0.287	-1.5
11/11/2017 7:53 AM	8.5	7	10.9	2.3	2.8	-0.284	-1.5
11/11/2017 7:54 AM	8.5	8.6	11.3	2.3	3.1	-0.288	-1.5
11/11/2017 7:55 AM	8.4	7.5	10.5	2.3	3.1	-0.295	-1.5
11/11/2017 7:56 AM	8.3	6.4	11	2.3	3.3	-0.278	-1.5
11/11/2017 7:57 AM	8	6.6	10.3	2.3	3.4	-0.289	-1.5
11/11/2017 7:58 AM	7.7	5.3	10.3	2.3	3.4	-0.295	-1.5
11/11/2017 7:59 AM	7.8	5.4	11.1	2.3	3.3	-0.281	-1.5
11/11/2017 8:00 AM	8	5.2	11	2.3	3.4	-0.287	-1.5
11/11/2017 8:01 AM	8.3	6.5	11.2	2.3	3.3	-0.292	-1.5
11/11/2017 8:02 AM	8.6	9.8	11.8	2.3	2.9	-0.284	-1.5
11/11/2017 8:03 AM	9	9.6	12.2	2.3	3	-0.285	-1.5
11/11/2017 8:04 AM	9.3	8.8	11.7	2.3	3.1	-0.297	-1.5

11/11/2017 8:05 AM	9.2	7.4	11.1	2.5	2.8	-0.296	-1.5
11/11/2017 8:06 AM	8.8	6.5	10.6	2.4	3.1	-0.285	-1.5
11/11/2017 8:07 AM	8.3	5.7	9.9	2.4	3.3	-0.289	-1.5
11/11/2017 8:08 AM	7.8	5.9	10	2.4	3.2	-0.295	-1.5
11/11/2017 8:09 AM	7.6	7.3	10.2	2.5	3.3	-0.284	-1.5
11/11/2017 8:10 AM	7.6	6.6	10.6	2.4	3.3	-0.284	-1.5
11/11/2017 8:11 AM	7.8	6.7	10.6	2.4	3.3	-0.300	-1.5
11/11/2017 8:12 AM	8	7.1	10.7	2.5	3.2	-0.282	-1.5
11/11/2017 8:13 AM	8.2	8.7	11.3	2.5	3.2	-0.285	-1.5
11/11/2017 8:14 AM	8.2	7.7	10.5	2.5	3.1	-0.295	-1.5
11/11/2017 8:15 AM	8	6.4	10	2.5	3.2	-0.297	-1.5
11/11/2017 8:16 AM	7.8	5.4	10.1	2.5	3.5	-0.284	-1.5
11/11/2017 8:17 AM	7.6	7.4	10.2	2.5	3.9	-0.289	-1.5
11/11/2017 8:18 AM	7.6	6.3	10.3	2.6	4.1	-0.297	-1.5
11/11/2017 8:19 AM	7.8	8.5	11.1	2.7	4.1	-0.276	-1.5
11/11/2017 8:20 AM	8.1	6.5	10.8	2.8	4	-0.290	-1.5
11/11/2017 8:21 AM	8.3	7.9	10.8	2.9	4.4	-0.292	-1.5
11/11/2017 8:22 AM	8.2	6.3	10.3	3	4.2	-0.285	-1.5
11/11/2017 8:23 AM	8.2	7.1	11.2	3.1	4	-0.285	-1.5
11/11/2017 8:24 AM	8.4	8	11.6	3.2	3.6	-0.296	-1.5
11/11/2017 8:25 AM	8.7	8.9	11.3	3.2	3.5	-0.288	-1.5
11/11/2017 8:26 AM	8.9	8.7	11.3	3.2	3.8	-0.288	-1.5
11/11/2017 8:27 AM	8.8	7.4	11.2	3.2	3.5	-0.296	-1.5
11/11/2017 8:28 AM	8.7	7.2	10.8	3.1	3.3	-0.291	-1.5
11/11/2017 8:29 AM	8.6	7	11.9	3	3.2	-0.283	-1.5
11/11/2017 8:30 AM	8.7	8.1	11.4	3	3.2	-0.294	-1.5
11/11/2017 8:31 AM	8.8	8.7	10.5	2.9	3.5	-0.290	-1.5
11/11/2017 8:32 AM	8.6	6.7	10.6	2.9	3.4	-0.289	-1.5
11/11/2017 8:33 AM	7.8	4.6	9.2	2.8	10.6	-0.285	-1.5
11/11/2017 8:34 AM	7	4.9	8.5	2.9	8.8	-0.300	-1.5
11/11/2017 8:35 AM	6.3	5.2	8.8	3.2	7.5	-0.287	-1.5
11/11/2017 8:36 AM	6.3	6	9.9	3.6	6.1	-0.285	-1.5
11/11/2017 8:37 AM	6.6	7.2	9.6	3.9	5.5	-0.291	-1.5
11/11/2017 8:38 AM	7	7.9	10.2	4.1	5.1	-0.283	-1.5
11/11/2017 8:39 AM	7.4	5.8	10.5	4.1	4.4	-0.279	-1.5
11/11/2017 8:40 AM	7.7	7.5	10.7	4.1	4.1	-0.283	-1.5
11/11/2017 8:41 AM	8	6.9	10.5	4	4	-0.292	-1.5
11/11/2017 8:42 AM	8.1	8	11.2	3.9	3.5	-0.243	-1.5
11/11/2017 8:43 AM	8.1	7.2	10.8	3.7	3.7	-0.226	-1.5
11/11/2017 8:44 AM	8	7	10.2	3.6	3.7	-0.246	-1.5
11/11/2017 8:45 AM	7.8	6.4	10.1	3.5	3.4	-0.246	-1.5
11/11/2017 8:46 AM	7.5	8.3	10.4	3.4	3.5	-0.251	-1.5
11/11/2017 8:47 AM	7.7	7.5	11.3	3.3	3.3	-0.270	-1.5
11/11/2017 8:48 AM	7.9	7.3	11.5	3.2	3.1	-0.265	-1.5
11/11/2017 8:49 AM	8.3	7.9	11.8	3.1	3.2	-0.270	-1.5
11/11/2017 8:50 AM	8.7	8.3	12.4	3	3.3	-0.274	-1.5
11/11/2017 8:51 AM	9.1	8.1	12.1	2.9	3.3	-0.282	-1.5

11/11/2017 8:52 AM	9.4	8	11.9	2.9	3.1	-0.276	-1.5
11/11/2017 8:53 AM	9.2	8.8	11.5	2.8	3.2	-0.279	-1.5
11/11/2017 8:54 AM	8.9	8.9	11.1	2.7	3.6	-0.071	-1.5
11/11/2017 8:55 AM	8.7	8.6	11.6	2.7	4.1	0.112	-1.5
11/11/2017 8:56 AM	8.7	7.7	11.3	2.7	3.8	0.030	-1.5
11/11/2017 8:57 AM	8.7	6.6	11.3	2.7	3.5	-0.024	-1.5
11/11/2017 8:58 AM	8.8	11.8	13.7	2.7	3.2	-0.082	-1.5
11/11/2017 8:59 AM	11.5	22.5	23.9	2.8	1.6	-0.224	-1.5
11/11/2017 9:00 AM	16.3	23.5	25.9	2.7	1.4	-0.288	-1.5
11/11/2017 9:01 AM	21.2	23	24.6	2.6	1.8	-0.295	-1.5
11/11/2017 9:02 AM	23.4	21	23.8	2.5	1.5	-0.286	-1.5
11/11/2017 9:03 AM	23.2	23.7	24.1	2.4	1.8	-0.297	-1.5
11/11/2017 9:04 AM	22.9	20.6	23.1	2.4	1.8	-0.300	-1.5
11/11/2017 9:05 AM	22	17.5	20.5	2.4	2.3	-0.269	-1.5
11/11/2017 9:06 AM	19.6	12	15.4	2.5	8.6	3.488	-1.5
11/11/2017 9:07 AM	15.7	8.6	11.6	2.6	7.8	3.617	-1.5
11/11/2017 9:08 AM	12	9.3	11.2	3.1	6.4	2.501	-1.5
11/11/2017 9:09 AM	9.9	7.5	10.9	3.7	6	1.649	-1.5
11/11/2017 9:10 AM	8.9	5.8	9.3	4.2	5.6	1.380	-1.5
11/11/2017 9:11 AM	8.5	6.8	9.3	4.6	5.3	1.035	-1.5
11/11/2017 9:12 AM	8	7.8	9.6	5	4.4	0.806	-1.5
11/11/2017 9:13 AM	8.1	8.2	10.3	5.1	4	0.584	-1.5
11/11/2017 9:14 AM	8.4	7	10.2	5.1	3.8	0.401	-1.5
11/11/2017 9:15 AM	8.6	8.3	10.4	4.9	3.4	0.282	-1.5
11/11/2017 9:16 AM	8.7	7.7	10.3	4.7	3.2	0.179	-1.5
11/11/2017 9:17 AM	8.8	8.2	10.1	4.4	3.6	0.086	-1.5
11/11/2017 9:18 AM	8.7	7.6	9.6	4.1	3.4	0.028	-1.5
11/11/2017 9:19 AM	8.4	5.5	9.4	3.9	3.3	-0.007	-1.5
11/11/2017 9:20 AM	7.8	5.7	8.9	3.7	3.5	-0.051	-1.5
11/11/2017 9:21 AM	7.5	5.8	9.2	3.6	3.7	-0.091	-1.5
11/11/2017 9:22 AM	7.3	6.8	9	3.5	3.6	-0.121	-1.5
11/11/2017 9:23 AM	7.2	5.8	8.9	3.4	3.4	-0.145	-1.5
11/11/2017 9:24 AM	7.3	6.2	9.6	3.3	3.4	-0.166	-1.5
11/11/2017 9:25 AM	7.7	6.8	10.6	3.2	3.3	-0.193	-1.5
11/11/2017 9:26 AM	8.4	7.5	11.3	3.2	3.4	-0.214	-1.5
11/11/2017 9:27 AM	9.1	8.1	11.6	3.1	3.1	-0.221	-1.5
11/11/2017 9:28 AM	9.6	10.3	11.8	3.1	2.9	-0.248	-1.5
11/11/2017 9:29 AM	10.1	10.8	12	3.1	2.7	-0.259	-1.5
11/11/2017 9:30 AM	10.1	8.8	10.7	3	2.9	-0.253	-1.5
11/11/2017 9:31 AM	9.8	7.9	10.8	3	3	-0.265	-1.5
11/11/2017 9:32 AM	9.3	8.7	10.4	2.9	3.1	-0.270	-1.5
11/11/2017 9:33 AM	8.9	6.8	9.6	2.9	3	-0.247	-1.5
11/11/2017 9:34 AM	8.6	7.6	10.3	2.9	3.4	-0.252	-1.5
11/11/2017 9:35 AM	8.5	7.4	10.3	2.9	3.1	-0.268	-1.5
11/11/2017 9:36 AM	8.6	7.9	10.3	2.9	3.1	-0.266	-1.5
11/11/2017 9:37 AM	8.9	7	11	2.8	3.1	-0.267	-1.5
11/11/2017 9:38 AM	9	8.7	10.6	2.8	3.2	-0.268	-1.5

11/11/2017 9:39 AM	9.1	8.2	10.1	2.8	2.9	-0.285	-1.5
11/11/2017 9:40 AM	9	8	10.4	2.8	2.9	-0.277	-1.5
11/11/2017 9:41 AM	8.9	8	10.2	2.8	2.9	-0.284	-1.5
11/11/2017 9:42 AM	8.7	7.3	10.7	2.8	3.1	-0.280	-1.5
11/11/2017 9:43 AM	8.9	7.3	10.9	2.8	2.8	-0.285	-1.5
11/11/2017 9:44 AM	9.2	8	10.8	2.8	2.9	-0.276	-1.5
11/11/2017 9:45 AM	9.5	8.3	11.3	2.8	2.7	-0.270	-1.5
11/11/2017 9:46 AM	9.6	8.1	10.5	2.8	3	-0.288	-1.5
11/11/2017 9:47 AM	9.2	7.4	10.4	2.8	3	-0.283	-1.5
11/11/2017 9:48 AM	9	7.7	10.1	2.8	2.8	-0.276	-1.5
11/11/2017 9:49 AM	8.8	11.9	12.2	2.8	2.8	-0.278	-1.5
11/11/2017 9:50 AM	11.8	20.7	23	2.8	1.9	-0.288	-1.5
11/11/2017 9:51 AM	16.5	20.9	23.3	2.7	1.2	-0.281	-1.5
11/11/2017 9:52 AM	21.3	21.1	23.7	2.7	1.3	-0.280	-1.5
11/11/2017 9:53 AM	23.3	20.7	23.8	2.6	1.1	-0.294	-1.5
11/11/2017 9:54 AM	23.4	22.9	24	2.5	1.1	-0.293	-1.5
11/11/2017 9:55 AM	23.6	21.7	23.8	2.4	1.2	-0.282	-1.5
11/11/2017 9:56 AM	23.3	21.4	23.1	2.4	1.2	-0.282	-1.5
11/11/2017 9:57 AM	23.4	24.4	24.2	2.3	1.5	-0.292	-1.5
11/11/2017 9:58 AM	23.4	22.8	23.8	2.3	1.2	-0.286	-1.5
11/11/2017 9:59 AM	23.7	21.5	23.5	2.3	1.5	-0.281	-1.5
11/11/2017 10:00 AM	23.4	20.7	23	2.3	1.3	-0.295	-1.5
11/11/2017 10:01 AM	22.8	22.2	22.6	2.3	1.3	-0.282	-1.5
11/11/2017 10:02 AM	22.7	24	23.5	2.3	1.3	-0.285	-1.5
11/11/2017 10:03 AM	23.1	23.9	23.2	2.2	1.3	-0.288	-1.5
11/11/2017 10:04 AM	23.7	22.6	24.3	2.3	1.6	-0.289	-1.5
11/11/2017 10:05 AM	23.8	22.1	23	2.3	1.4	-0.275	-1.5
11/11/2017 10:06 AM	23.6	23	23.3	2.3	1.3	-0.252	-1.5
11/11/2017 10:07 AM	23.8	25.3	24	2.5	1.2	-0.260	-1.5
11/11/2017 10:08 AM	24	21.5	23.7	2.5	1.3	-0.283	-1.5
11/11/2017 10:09 AM	24.3	24	24.3	2.5	1.4	-0.276	-1.5
11/11/2017 10:10 AM	24.4	25.5	24.2	2.5	1.3	-0.268	-1.5
11/11/2017 10:11 AM	24.5	22.7	24.1	2.4	1.3	-0.234	-1.5
11/11/2017 10:12 AM	24.1	37.5	46.6	2.5	11	5.187	-1.5
11/11/2017 10:13 AM	23.2	29.1	27.9	2.7	7.5	2.506	-1.5
11/11/2017 10:14 AM	23	28.4	26.3	3.5	2.3	0.981	-1.5
11/11/2017 10:15 AM	23.1	29	27.7	4.2	9.6	8.647	-1.5
11/11/2017 10:16 AM	22.9	34.6	30.1	4.5	10.9	20.972	-1.5
11/11/2017 10:17 AM	22.4	24.6	20.6	5.1	7.7	13.072	-1.5
11/11/2017 10:18 AM	22.1	22.5	20.5	5.8	8.9	11.159	-1.5
11/11/2017 10:19 AM	21	16.1	15.4	6.5	9.5	11.657	-1.5
11/11/2017 10:20 AM	18.5	14.4	15.1	7.1	9.3	13.289	-1.5
11/11/2017 10:21 AM	15.3	9.4	11.4	7.5	9	14.832	-1.5
11/11/2017 10:22 AM	12.2	8	8.1	7.9	11.1	18.283	-1.5
11/11/2017 10:23 AM	9.4	4.1	6.8	8.5	14.5	22.958	-1.5
11/11/2017 10:24 AM	6.8	7.6	5.5	9.1	14.3	23.257	-1.5
11/11/2017 10:25 AM	5.4	6.6	6	9.8	14.5	23.633	-1.5

11/11/2017 10:26 AM	5.4	11.1	8.5	10.5	13.5	23.193	-1.5
11/11/2017 10:27 AM	9.1	20.5	19.7	11	10.6	16.798	-1.5
11/11/2017 10:28 AM	13.4	16.6	15.6	11.2	7.8	14.430	-1.5
11/11/2017 10:29 AM	17.1	17.6	17.5	10.9	5.9	13.553	-1.5
11/11/2017 10:30 AM	16.7	13.5	13.8	10.5	6.7	14.591	-1.5
11/11/2017 10:31 AM	14.1	12	11.7	9.9	18.5	16.371	-1.5
11/11/2017 10:32 AM	11	9.7	8.5	9.6	14.9	17.752	-1.5
11/11/2017 10:33 AM	8.3	8.3	7.8	9.7	12.9	17.869	-1.5
11/11/2017 10:34 AM	7	6.3	7.6	10.1	10.1	16.270	-1.5
11/11/2017 10:35 AM	6.6	7.9	7.7	10.4	9.3	15.904	-1.5
11/11/2017 10:36 AM	6.6	6.4	7.7	10.6	7.9	14.521	-1.5
11/11/2017 10:37 AM	6.6	8.6	7.1	10.5	7.9	14.289	-1.5
11/11/2017 10:38 AM	6.6	4.4	6.8	10.3	6.9	12.563	-1.3
11/11/2017 10:39 AM	6.3	4.8	6.5	9.9	6.3	11.005	5
11/11/2017 10:40 AM	6.1	6.9	6.4	9.4	5.5	9.481	1.3
11/11/2017 10:41 AM	6.7	11.1	11.9	9	6.9	7.188	-1.5
11/11/2017 10:42 AM	8.7	12.3	15.2	8.7	6.3	3.724	-1.5
11/11/2017 10:43 AM	11.3	14.5	14.8	8.3	6.8	1.944	-1.5
11/11/2017 10:44 AM	13.3	12.2	14.8	8	9.6	0.757	-1.5
11/11/2017 10:45 AM	14	11.5	12.8	8.3	7.8	0.474	-1.5
11/11/2017 10:46 AM	13.9	12.6	14.2	8.7	12.4	0.323	-1.4
11/11/2017 10:47 AM	15	17.9	18	8.8	16.6	-0.104	-1.5
11/11/2017 10:48 AM	15.6	14.2	20.1	8.9	30.1	-0.146	-1.5
11/11/2017 10:49 AM	17.1	18.8	23.2	10.5	9.2	0.479	-1.5
11/11/2017 10:50 AM	18.6	19.6	23.7	11.8	7.5	0.606	-1.5
11/11/2017 10:51 AM	20.7	19.9	24.2	10.8	5.5	0.086	-1.5
11/11/2017 10:52 AM	22.2	16.7	19	9	3.9	0.068	-1.4
11/11/2017 10:53 AM	20	11.3	13.3	7.8	15.8	0.249	-1.4
11/11/2017 10:54 AM	14.6	4.2	4.7	7.9	87.6	0.244	-1.5
11/11/2017 10:55 AM	9.5	6.1	10.5	11.7	37.8	0.157	-1.5
11/11/2017 10:56 AM	6.7	3.4	7.4	15.7	63.8	0.319	-1.5
11/11/2017 10:57 AM	7	7	10.8	17.5	30.8	0.262	-1.5
11/11/2017 10:58 AM	8.1	8.4	11.9	17.8	17.3	0.518	-1.5
11/11/2017 10:59 AM	7.7	0.8	2.8	16.9	202.9	1.050	-1.5
11/11/2017 11:00 AM	6	1.3	4.9	18	95.8	0.871	-1.5
11/11/2017 11:01 AM	4.8	5.9	9.2	19.7	40.8	0.459	-1.5
11/11/2017 11:02 AM	5.9	8.5	12.9	19.6	21.6	0.437	-1.5
11/11/2017 11:03 AM	8.5	11.7	12.8	18.1	13.2	0.624	-1.5
11/11/2017 11:04 AM	10.6	12.5	14.2	15.9	8.8	0.661	-1.5
11/11/2017 11:05 AM	12.3	12.5	15.5	13.6	6.6	0.788	-1.5
11/11/2017 11:06 AM	14.4	16.6	21.1	13.2	4.6	0.085	-1.5
11/11/2017 11:07 AM	15.5	12.8	15.4	12.5	6	0.201	-1.4
11/11/2017 11:08 AM	15.2	9.9	14.8	6.3	12.2	0.243	-1.5
11/11/2017 11:09 AM	13.3	8	11.2	6.3	25.7	0.551	-1.5
11/11/2017 11:10 AM	10.3	6.3	7.7	8.2	44.4	0.941	-1.5
11/11/2017 11:11 AM	8.3	5.3	7.3	11.1	35.1	0.441	-1.5
11/11/2017 11:12 AM	6.8	4.6	7.7	13.2	33.1	0.353	-1.5

11/11/2017 11:13 AM	6.9	5.3	9.3	14.2	30.6	0.218	-1.5
11/11/2017 11:14 AM	7	4.5	8.2	14.2	36.8	0.047	-1.5
11/11/2017 11:15 AM	7.1	5.3	7.8	14.1	31.6	-0.041	-1.5
11/11/2017 11:16 AM	7	5.3	7.1	14.1	35.9	-0.127	-1.4
11/11/2017 11:17 AM	6.6	4.5	5.8	14.1	43.8	-0.193	-1.5
11/11/2017 11:18 AM	6.2	3.8	6.7	14.4	42.2	-0.197	-1.5
11/11/2017 11:19 AM	5.7	2.8	5.4	14.9	48.1	-0.166	-1.5
11/11/2017 11:20 AM	5.3	2.4	5.5	15.3	52	-0.093	-1.4
11/11/2017 11:21 AM	5.1	3.9	4.6	15.1	51	-0.150	-1.1
11/11/2017 11:22 AM	4.3	1	2.6	14.6	55.8	-0.170	-1.1
11/11/2017 11:23 AM	3.2	-1.9	1.6	13.9	47.6	-0.160	-1.4
11/11/2017 11:24 AM	2.1	-0.3	2	13.2	41.6	0.391	-1.5
11/11/2017 11:25 AM	1.7	0.4	2.4	12.7	36.3	3.492	-1.5
11/11/2017 11:26 AM	2	3.7	3.8	12.2	30	5.618	-1.5
11/11/2017 11:27 AM	2.5	0.1	3.6	11.8	25.4	6.905	-1.5
11/11/2017 11:28 AM	2.8	1.7	3.8	11.5	22.8	9.156	-1.5
11/11/2017 11:29 AM	2.9	1.7	3.8	11.3	19.4	8.433	-1.5
11/11/2017 11:30 AM	3.1	1	3.9	11.1	16.4	7.565	-1.5
11/11/2017 11:31 AM	3.3	3.7	4.6	10.8	14.2	7.968	-1.5
11/11/2017 11:32 AM	3.6	4.4	4.9	10.5	12.6	8.572	-1.5
11/11/2017 11:33 AM	4	3.6	5.9	10.1	11.3	10.081	-1.5
11/11/2017 11:34 AM	4.5	4.2	6.2	9.6	10.2	9.334	-1.5
11/11/2017 11:35 AM	5	4.2	6.1	9.3	9.5	8.676	-1.5
11/11/2017 11:36 AM	5.4	3.2	6.9	8.8	7.8	7.896	-1.5
11/11/2017 11:37 AM	5.5	4.8	6.9	8.4	7.5	9.362	-1.5
11/11/2017 11:38 AM	5.6	6.4	6.9	8	7.4	9.848	-1.5
11/11/2017 11:39 AM	6	5.6	7.3	7.7	6.6	10.291	-1.5
11/11/2017 11:40 AM	6.3	5.6	7.6	7.3	6.1	9.191	-1.5
11/11/2017 11:41 AM	6.5	4.9	7.5	7	5.8	9.282	-1.5
11/11/2017 11:42 AM	6.3	3	7.2	6.7	5.5	8.976	-1.5
11/11/2017 11:43 AM	6	4.2	6.2	6.4	5.4	9.221	-1.5
11/11/2017 11:44 AM	5.7	6.5	7.6	6.1	5.6	10.177	-1.5
11/11/2017 11:45 AM	6	4.1	8	6	5.6	10.748	-1.5
11/11/2017 11:46 AM	6.3	5.4	7.2	5.8	6.4	11.370	-1.5
11/11/2017 11:47 AM	6.2	4.4	6.9	5.8	6.5	12.028	-1.5
11/11/2017 11:48 AM	5.7	3.8	6.9	5.9	6.4	12.773	-1.5
11/11/2017 11:49 AM	5.2	4.4	6.5	6	5.9	11.856	-1.5
11/11/2017 11:50 AM	5.1	5	6.6	6	5.8	11.322	-1.5
11/11/2017 11:51 AM	5.1	4.7	6.7	6	6.3	12.961	-1.5
11/11/2017 11:52 AM	5.3	5.8	7.5	6	7.7	13.736	-1.5
11/11/2017 11:53 AM	5.4	3.9	7.2	6.1	7.7	12.908	-1.5
11/11/2017 11:54 AM	5.5	5.1	7.7	6.3	6.9	13.910	-1.5
11/11/2017 11:55 AM	5.8	6.3	7.9	6.5	6	12.278	-1.5
11/11/2017 11:56 AM	6	5.9	8	6.7	6.1	12.499	-1.5
11/11/2017 11:57 AM	6.2	4.8	7.3	6.7	5.7	11.821	-1.5
11/11/2017 11:58 AM	6	3.4	7.3	6.7	5.5	10.634	-1.5
11/11/2017 11:59 AM	5.9	4.4	7.6	6.6	4.9	9.472	-1.5

11/11/2017 12:00 PM	5.9	5	7.5	6.4	4.8	9.387	-1.5
11/11/2017 12:01 PM	5.8	4	7.1	6	4.7	9.283	-1.5
11/11/2017 12:02 PM	5.6	4.5	6.9	5.7	4.9	9.346	-1.5
11/11/2017 12:03 PM	5.6	8.2	10.3	5.4	4.7	9.118	-1.5
11/11/2017 12:04 PM	8.9	14.4	18.3	5.2	4.5	7.863	-1.5
11/11/2017 12:05 PM	12.2	14.7	16.4	5	4.8	9.004	-1.5
11/11/2017 12:06 PM	14.6	10.9	13.5	4.8	4.9	7.840	-1.5
11/11/2017 12:07 PM	13.1	8.7	11.3	4.8	4.1	7.116	-1.5
11/11/2017 12:08 PM	10.9	6.8	9.4	4.8	7.1	6.585	-1.5
11/11/2017 12:09 PM	8	0.7	5.1	4.3	69.4	6.084	-1.3
11/11/2017 12:10 PM	5.4	1.8	5.4	4.5	75.6	4.764	-1
11/11/2017 12:11 PM	4.1	4.3	6.8	5.9	38.6	2.848	-0.9
11/11/2017 12:12 PM	5	6.8	11.8	8.3	21.3	1.497	-1.5
11/11/2017 12:13 PM	7.7	11.2	16.5	10.1	12.3	0.435	-1.5
11/11/2017 12:14 PM	10.7	12.8	16.7	10.1	5.7	0.131	-1.4
11/11/2017 12:15 PM	13.4	13.8	17.5	8.3	4	-0.032	-0.6
11/11/2017 12:16 PM	14.7	12.3	16.3	6.2	11.6	-0.018	-0.9
11/11/2017 12:17 PM	14.9	13.4	17.8	5.3	5.8	-0.099	-1.4
11/11/2017 12:18 PM	14.7	12.9	18.8	5.3	4.4	-0.087	-1.5
11/11/2017 12:19 PM	15.3	14.8	18.7	4.8	3.1	0.208	-1.5
11/11/2017 12:20 PM	16.2	15.1	18.6	3.9	2.1	0.191	-1.5
11/11/2017 12:21 PM	16.7	13.1	18.6	3	2	0.004	-1.5
11/11/2017 12:22 PM	16.9	15.7	17.9	2.3	1.9	1.257	-1.5
11/11/2017 12:23 PM	17.3	18.1	20.9	1.9	2	2.186	-1.4
11/11/2017 12:24 PM	18.1	17.1	20.1	1.7	2	1.328	-1
11/11/2017 12:25 PM	18.6	15.2	19.2	1.6	3.4	0.513	-1.2
11/11/2017 12:26 PM	18.5	16.3	18	1.9	5.5	0.158	-1.4
11/11/2017 12:27 PM	17.5	15.2	15.7	2.5	6.1	0.165	-1.2
11/11/2017 12:28 PM	15.8	10.4	12.8	2.8	9	0.140	-1.2
11/11/2017 12:29 PM	13.5	8.7	10.9	3	6.2	1.559	-1.5
11/11/2017 12:30 PM	11.1	7.9	10.2	3.2	7.1	8.035	-1.5
11/11/2017 12:31 PM	9.3	8.1	9.7	3.6	7.6	14.002	-1.5
11/11/2017 12:32 PM	8.2	6.4	9.4	4.1	8	18.990	-1.5
11/11/2017 12:33 PM	7.5	7.3	8.8	4.8	6.9	18.608	-1.4
11/11/2017 12:34 PM	7.2	5.2	8.9	5.5	6.1	15.413	-1.5
11/11/2017 12:35 PM	7.1	5.5	8.2	6.1	7.2	12.547	-1.5
11/11/2017 12:36 PM	7	3.5	7.8	6.4	6.4	11.398	-1.5
11/11/2017 12:37 PM	6.6	3.4	7.2	6.4	6.1	12.157	-1.5
11/11/2017 12:38 PM	5.8	4.1	6.5	6.4	7.2	12.807	-1.5
11/11/2017 12:39 PM	5.5	8.6	11.2	6.3	7.4	15.090	-1.5
11/11/2017 12:40 PM	7.8	18	18.7	6.4	6.5	28.307	-1.5
11/11/2017 12:41 PM	11.6	19.1	19.8	6.7	4.4	26.983	-1.5
11/11/2017 12:42 PM	15.6	22	22.9	7.2	3.1	14.332	-1.5
11/11/2017 12:43 PM	17	17.8	17.9	7.5	2.4	9.436	-1.5
11/11/2017 12:44 PM	16.5	15.9	15.3	7.1	1.9	6.172	-1.5
11/11/2017 12:45 PM	15.5	14.3	15.5	6.3	1.7	3.940	-1.5
11/11/2017 12:46 PM	15	16.7	16	5.2	1.8	2.216	-1.5

11/11/2017 12:47 PM	15.1	14	15.1	4.1	2.1	1.423	-1.5
11/11/2017 12:48 PM	14.7	11.2	12.9	3.1	2.1	1.230	-1.5
11/11/2017 12:49 PM	13.5	12	12	2.4	2.4	0.951	-1.5
11/11/2017 12:50 PM	11.7	6.1	10.3	1.8	2.6	0.860	-1.5
11/11/2017 12:51 PM	10.2	8	9.6	1.4	2.4	0.764	-1.5
11/11/2017 12:52 PM	9	5.7	9.3	1.2	2.7	0.631	-1.5
11/11/2017 12:53 PM	8.3	6.9	9.3	1	2.7	0.507	-1.5
11/11/2017 12:54 PM	8	7.1	9.1	1	2.7	0.383	-1.5
11/11/2017 12:55 PM	7.7	4.7	8.1	0.9	3	0.340	-1.5
11/11/2017 12:56 PM	7.3	3.2	7.8	0.9	3	0.277	-1.5
11/11/2017 12:57 PM	6.6	5	7.2	0.9	3.3	0.230	-1.5
11/11/2017 12:58 PM	6.1	5.9	7.2	0.9	3.2	0.174	-1.5
11/11/2017 12:59 PM	5.9	4.2	7.4	0.9	3.5	0.126	-1.5
11/11/2017 1:00 PM	5.9	3.2	7.1	1	3.5	0.088	-1.5
11/11/2017 1:01 PM	5.8	3.7	7.1	1	4.4	0.041	-1.5
11/11/2017 1:02 PM	5.8	4.8	7.3	1	4.7	0.012	-1.5
11/11/2017 1:03 PM	5.8	3	7.2	1	5.4	-0.026	-1.5
11/11/2017 1:04 PM	5.8	4.1	6.7	1.1	5.9	-0.057	-1.5
11/11/2017 1:05 PM	5.7	4.5	7.5	1.3	5.4	-0.012	-1.5
11/11/2017 1:06 PM	5.8	4.4	7.9	1.5	5	0.296	-1.5
11/11/2017 1:07 PM	6.2	4.2	8.2	1.7	4.6	0.164	-1.5
11/11/2017 1:08 PM	6.6	4.8	8.7	1.7	4.2	0.090	-1.5
11/11/2017 1:09 PM	6.9	4.3	8.9	1.7	3.9	0.046	-1.5
11/11/2017 1:10 PM	7	4	8.1	1.6	3.8	-0.002	-1.5
11/11/2017 1:11 PM	6.8	4.9	7.8	1.4	3.5	-0.043	-1.5
11/11/2017 1:12 PM	6.5	2.9	8.2	1.3	3.4	-0.061	-1.5
11/11/2017 1:13 PM	6.3	4.1	7.7	1.2	3.4	-0.087	-1.5
11/11/2017 1:14 PM	6.1	4.1	7.7	1.1	3.4	-0.114	-1.5
11/11/2017 1:15 PM	6	2.6	7.4	1	3.5	-0.116	-1.5
11/11/2017 1:16 PM	5.9	4	8.5	0.9	3.6	-0.143	-1.5
11/11/2017 1:17 PM	7.6	11.1	12.8	0.9	2.9	-0.210	-1.5
11/11/2017 1:18 PM	8.9	5.3	10.7	0.8	2.9	-0.207	-1.5
11/11/2017 1:19 PM	9.7	4.3	8.9	0.7	3.2	-0.211	-1.5
11/11/2017 1:20 PM	8.4	4.8	8.4	0.7	3.1	-0.204	-1.5
11/11/2017 1:21 PM	7.3	4.3	7.9	0.6	3.1	-0.216	-1.5
11/11/2017 1:22 PM	6.7	4.3	7.9	0.6	3.6	-0.213	-1.5
11/11/2017 1:23 PM	6.3	3.2	7.5	0.5	3.5	-0.216	-1.5
11/11/2017 1:24 PM	6.1	3.7	7.5	0.5	3.4	-0.245	-1.5
11/11/2017 1:25 PM	5.9	4	7.4	0.5	3.8	-0.236	-1.5
11/11/2017 1:26 PM	5.7	3.1	7.3	0.5	3.5	-0.235	-1.5
11/11/2017 1:27 PM	5.5	4.5	7.4	0.5	3.2	-0.254	-1.5
11/11/2017 1:28 PM	5.5	3.9	7.7	0.5	3.4	-0.244	-1.5
11/11/2017 1:29 PM	5.7	4.9	7.9	0.4	3.7	-0.240	-1.5
11/11/2017 1:30 PM	5.9	2.8	7.3	0.4	3.9	-0.259	-1.5
11/11/2017 1:31 PM	5.6	2.7	6.6	0.5	4.2	-0.249	-1.5
11/11/2017 1:32 PM	5.2	2	7	0.5	4.2	-0.247	-1.5
11/11/2017 1:33 PM	4.9	3.1	7.1	0.5	4.1	-0.263	-1.5

11/11/2017 1:34 PM	5	2.9	7.4	0.5	4.2	-0.267	-1.5
11/11/2017 1:35 PM	5.1	2.2	7.3	0.5	4	-0.259	-1.5
11/11/2017 1:36 PM	5.2	2	6.9	0.5	4.1	-0.259	-1.5
11/11/2017 1:37 PM	5.1	3.4	7.1	0.5	4	-0.274	-1.5
11/11/2017 1:38 PM	5.1	4.2	7.2	0.5	3.6	-0.265	-1.5
11/11/2017 1:39 PM	5.1	3.5	7.3	0.5	3.7	-0.271	-1.5
11/11/2017 1:40 PM	5.2	4.1	7.6	0.5	3.7	-0.283	-1.5
11/11/2017 1:41 PM	7	13.3	17	0.5	2.6	-0.282	-1.5
11/11/2017 1:42 PM	10.8	14.3	17.9	0.4	2.2	-0.284	-1.5
11/11/2017 1:43 PM	14.5	14.4	17.9	0.4	2	-0.294	-1.5
11/11/2017 1:44 PM	16.9	18.4	18.9	0.4	1.6	-0.297	-1.5
11/11/2017 1:45 PM	17.5	15.8	19.1	0.3	1.8	-0.289	-1.5
11/11/2017 1:46 PM	18.1	16.8	18.4	0.2	2	-0.307	-1.5
11/11/2017 1:47 PM	17.4	11.5	14.2	0.1	1.9	-0.303	-1.5
11/11/2017 1:48 PM	15.2	6.7	11.1	0.1	2.3	-0.291	-1.5
11/11/2017 1:49 PM	12	6.8	9.5	0	2.8	-0.296	-1.5
11/11/2017 1:50 PM	9.3	5.7	8.5	0	3.1	-0.307	-1.5
11/11/2017 1:51 PM	7.7	4.9	8.4	0	3.1	-0.292	-1.5
11/11/2017 1:52 PM	7.6	6.1	10.8	0.1	3	-0.293	-1.5
11/11/2017 1:53 PM	7.8	6	8.9	0.1	3	-0.303	-1.5
11/11/2017 1:54 PM	7.8	5.3	8.1	0.1	3	-0.291	-1.5
11/11/2017 1:55 PM	7.1	4.9	7.9	0.1	3.5	-0.293	-1.5
11/11/2017 1:56 PM	6.4	3.8	7.3	0.2	3.5	-0.303	-1.5
11/11/2017 1:57 PM	5.8	2.3	6.5	0.2	3.8	-0.296	-1.5
11/11/2017 1:58 PM	5.2	3.3	6	0.2	4	-0.289	-1.5
11/11/2017 1:59 PM	4.7	1.8	6.2	0.2	4	-0.295	-1.5
11/11/2017 2:00 PM	4.5	2.2	6.4	0.2	4.2	-0.299	-1.5
11/11/2017 2:01 PM	4.5	2.8	7	0.2	4.1	-0.287	-1.5
11/11/2017 2:02 PM	4.7	4.3	7	0.2	4.1	-0.295	-1.5
11/11/2017 2:03 PM	4.9	3.1	6.7	0.3	4.1	-0.297	-1.5
11/11/2017 2:04 PM	4.8	2.4	6.1	0.3	4.3	-0.286	-1.5
11/11/2017 2:05 PM	4.5	2.9	6.3	0.3	4.4	-0.295	-1.5
11/11/2017 2:06 PM	4.2	3.3	6.4	0.3	4.4	-0.295	-1.5
11/11/2017 2:07 PM	4.1	1.8	6.1	0.4	4.4	-0.284	-1.5
11/11/2017 2:08 PM	4.1	3	6.3	0.3	4.3	-0.290	-1.5
11/11/2017 2:09 PM	4.1	1.3	6.4	0.3	4.6	-0.293	-1.5
11/11/2017 2:10 PM	4.2	2.6	6.8	0.3	4.5	-0.293	-1.5
11/11/2017 2:11 PM	4.5	1.6	7.2	0.3	4	-0.292	-1.5
11/11/2017 2:12 PM	5	4.1	8.7	0.2	4.1	-0.298	-1.5
11/11/2017 2:13 PM	8.4	20.7	23.7	0.2	2	-0.298	-1.5
11/11/2017 2:14 PM	14.1	19.9	22.4	0.2	1.4	-0.297	-1.5
11/11/2017 2:15 PM	19.7	20.8	23.2	0.1	1.5	-0.301	-1.5
11/11/2017 2:16 PM	21.8	19	20.7	0	2	-0.313	-1.5
11/11/2017 2:17 PM	20.9	15.9	19.6	-0.2	1.7	-0.304	-1.5
11/11/2017 2:18 PM	19.6	14.8	18.9	-0.3	1.9	-0.306	-1.5
11/11/2017 2:19 PM	18.7	18.5	20	-0.4	1.9	-0.309	-1.5
11/11/2017 2:20 PM	19.1	17.9	21.4	-0.5	1.7	-0.306	-1.5

11/11/2017 2:21 PM	20.1	19.5	22.4	-0.5	1.8	-0.311	-1.5
11/11/2017 2:22 PM	21.5	21.7	23.1	-0.5	2.1	-0.317	-1.5
11/11/2017 2:23 PM	22.4	21	23.9	-0.5	2.4	-0.313	-1.5
11/11/2017 2:24 PM	23	21.7	23.1	-0.5	1.9	-0.124	-1.5
11/11/2017 2:25 PM	22.9	19.5	22	-0.4	4	-0.069	-1.5
11/11/2017 2:26 PM	20.9	15.6	16.4	-0.3	24.2	-0.244	-1.5
11/11/2017 2:27 PM	19	17.2	19.6	0.7	6.9	-0.285	-1.5
11/11/2017 2:28 PM	18.7	21.8	22.5	2	2.1	-0.313	-1.5

Tallgrass Prairie November 2017

Date&Time	O3 NO-CL	O3 UV-C-H	O3 UV-C	NO2-C	NO	CO	THC
11/13/2017 0:00	20.97	18.73	22.01	-0.81	1.32	0.054	1.311
11/13/2017 0:01	20.78	18.79	21.25	-0.63	1.28	0.050	1.319
11/13/2017 0:02	20.80	18.81	20.34	-0.71	1.18	0.060	1.318
11/13/2017 0:03	20.50	17.48	20.79	-0.35	1.73	0.052	1.315
11/13/2017 0:04	19.98	18.08	19.94	-0.31	1.49	0.049	1.320
11/13/2017 0:05	19.80	17.84	20.66	-0.85	1.09	0.061	1.323
11/13/2017 0:06	19.92	18.09	20.88	-0.61	1.30	0.057	1.301
11/13/2017 0:07	20.12	17.58	20.87	-0.65	0.97	0.057	1.301
11/13/2017 0:08	20.57	20.79	22.18	-0.91	0.91	0.063	1.288
11/13/2017 0:09	20.73	19.19	21.60	-0.93	1.03	0.051	1.275
11/13/2017 0:10	20.93	18.79	21.36	-0.93	1.29	0.068	1.297
11/13/2017 0:11	20.53	18.55	20.57	-0.92	1.07	0.054	1.302
11/13/2017 0:12	19.95	17.20	19.98	-0.86	1.05	0.045	1.304
11/13/2017 0:13	19.48	18.86	20.38	-0.92	1.23	0.056	1.294
11/13/2017 0:14	19.60	18.30	20.58	-0.83	1.07	0.060	1.289
11/13/2017 0:15	19.73	18.39	20.29	-0.51	1.42	0.061	1.293
11/13/2017 0:16	19.72	16.98	19.63	-0.03	1.55	0.064	1.305
11/13/2017 0:17	19.30	17.88	19.84	0.65	1.76	0.053	1.315
11/13/2017 0:18	19.02	19.37	19.75	0.20	1.67	0.056	1.303
11/13/2017 0:19	19.00	18.91	20.24	-0.17	1.04	0.060	1.293
11/13/2017 0:20	19.32	19.11	20.39	0.10	1.71	0.055	1.285
11/13/2017 0:21	19.55	18.10	20.08	-0.06	1.23	0.050	1.289
11/13/2017 0:22	19.42	18.25	20.13	0.45	1.81	0.059	1.298
11/13/2017 0:23	19.33	17.72	20.56	0.28	1.54	0.046	1.308
11/13/2017 0:24	19.30	20.15	20.55	-0.27	1.30	0.056	1.307
11/13/2017 0:25	19.72	19.74	20.74	-0.19	1.37	0.061	1.286
11/13/2017 0:26	20.00	18.45	20.95	-0.42	1.20	0.047	1.274
11/13/2017 0:27	20.47	19.38	21.66	-0.86	1.10	0.057	1.284
11/13/2017 0:28	20.52	18.59	20.79	-0.65	1.40	0.054	1.287
11/13/2017 0:29	20.60	20.77	21.66	0.09	1.98	0.048	1.290
11/13/2017 0:30	20.53	19.22	21.00	-0.03	2.04	0.056	1.289
11/13/2017 0:31	20.35	18.92	20.79	0.26	1.52	0.048	1.293
11/13/2017 0:32	20.23	17.31	20.64	-0.65	1.09	0.053	1.295
11/13/2017 0:33	20.05	18.47	20.82	-0.94	1.16	0.059	1.313
11/13/2017 0:34	20.12	19.44	20.80	-0.71	1.31	0.047	1.314
11/13/2017 0:35	20.20	19.98	21.42	-0.31	1.56	0.050	1.294
11/13/2017 0:36	20.65	19.57	22.27	-0.87	1.07	0.056	1.289
11/13/2017 0:37	20.98	18.18	21.18	-0.79	1.03	0.046	1.280
11/13/2017 0:38	20.95	17.68	20.69	-0.49	1.26	0.053	1.260
11/13/2017 0:39	20.35	17.39	20.15	-0.50	1.10	0.056	1.265
11/13/2017 0:40	20.00	19.73	21.03	-0.84	0.91	0.042	1.263
11/13/2017 0:41	20.15	19.43	21.16	-0.72	1.17	0.055	1.278
11/13/2017 0:42	20.47	18.58	21.48	-0.89	1.15	0.052	1.286
11/13/2017 0:43	20.73	19.84	21.54	-0.65	1.52	0.044	1.279
11/13/2017 0:44	20.70	18.66	21.51	-1.03	1.15	0.057	1.286

11/13/2017 0:45	20.67	18.04	21.23	-0.69	1.73	0.045	1.284
11/13/2017 0:46	20.37	18.98	20.67	-0.48	1.42	0.048	1.273
11/13/2017 0:47	20.02	18.48	20.42	-0.60	1.82	0.058	1.278
11/13/2017 0:48	19.73	18.12	20.67	-0.35	1.32	0.043	1.285
11/13/2017 0:49	19.70	17.48	20.64	-0.68	1.43	0.054	1.282
11/13/2017 0:50	19.67	17.72	20.21	-0.60	1.08	0.054	1.280
11/13/2017 0:51	19.70	18.43	20.42	-0.98	0.79	0.036	1.286
11/13/2017 0:52	19.65	18.32	20.20	-0.98	0.98	0.060	1.285
11/13/2017 0:53	19.53	18.28	19.83	-0.98	1.22	0.043	1.281
11/13/2017 0:54	19.27	18.17	20.69	-0.73	1.32	0.048	1.299
11/13/2017 0:55	19.53	19.08	21.31	-0.72	1.40	0.056	1.320
11/13/2017 0:56	19.95	21.23	21.19	-0.09	1.44	0.044	1.302
11/13/2017 0:57	20.65	19.16	21.95	-0.75	1.11	0.055	1.293
11/13/2017 0:58	20.80	20.12	21.33	-1.15	1.00	0.060	1.303
11/13/2017 0:59	20.73	19.28	20.47	0.09	2.39	0.037	1.294
11/13/2017 1:00	20.25	18.63	20.52	0.60	1.72	0.057	1.301
11/13/2017 1:01	19.92	19.73	20.68	-0.75	1.64	0.049	1.299
11/13/2017 1:02	19.97	19.33	21.30	-1.11	0.93	0.048	1.284
11/13/2017 1:03	20.32	19.09	21.13	-1.11	0.91	0.051	1.280
11/13/2017 1:04	20.50	19.71	21.28	-0.43	1.34	0.037	1.278
11/13/2017 1:05	20.58	19.50	21.56	-0.87	1.03	0.048	1.284
11/13/2017 1:06	20.42	19.23	20.56	-0.97	0.87	0.060	1.301
11/13/2017 1:07	20.28	18.40	21.02	-0.79	0.88	0.040	1.317
11/13/2017 1:08	20.03	17.74	20.32	-0.91	1.13	0.048	1.320
11/13/2017 1:09	19.87	18.63	20.48	-0.86	0.85	0.045	1.329
11/13/2017 1:10	19.85	18.58	20.85	-0.84	0.76	0.042	1.332
11/13/2017 1:11	19.90	18.88	20.38	-0.68	0.96	0.047	1.318
11/13/2017 1:12	19.72	18.74	19.73	-0.62	1.44	0.047	1.325
11/13/2017 1:13	19.45	17.56	20.10	-0.68	1.08	0.040	1.323
11/13/2017 1:14	19.22	20.42	20.79	-0.58	1.36	0.051	1.312
11/13/2017 1:15	19.63	17.93	21.60	-0.80	1.05	0.044	1.307
11/13/2017 1:16	19.98	18.08	20.91	-0.70	1.06	0.046	1.313
11/13/2017 1:17	20.30	17.93	21.20	-0.70	0.95	0.054	1.309
11/13/2017 1:18	20.40	20.00	21.76	-0.72	0.69	0.040	1.318
11/13/2017 1:19	20.75	19.07	22.29	-0.69	1.25	0.055	1.311
11/13/2017 1:20	21.00	18.73	21.41	-0.74	1.07	0.048	1.300
11/13/2017 1:21	20.97	18.69	21.84	-0.07	1.39	0.043	1.322
11/13/2017 1:22	20.92	20.73	21.86	-0.06	2.22	0.057	1.338
11/13/2017 1:23	20.97	18.85	21.88	-0.05	2.06	0.046	1.347
11/13/2017 1:24	21.13	18.63	21.63	0.08	1.22	0.043	1.344
11/13/2017 1:25	20.88	19.30	21.21	1.84	3.53	0.058	1.348
11/13/2017 1:26	20.55	19.34	20.75	0.01	1.76	0.041	1.331
11/13/2017 1:27	20.32	18.26	21.29	-0.57	1.11	0.044	1.348
11/13/2017 1:28	20.17	19.21	20.82	-0.99	1.16	0.052	1.354
11/13/2017 1:29	20.12	19.63	20.95	-0.92	0.85	0.036	1.363
11/13/2017 1:30	20.15	18.90	21.08	-0.97	0.81	0.050	1.354
11/13/2017 1:31	20.22	17.63	20.72	-0.92	1.01	0.045	1.344

11/13/2017 1:32	20.30	18.61	20.68	-0.99	0.85	0.042	1.346
11/13/2017 1:33	20.12	18.81	20.45	-1.03	0.80	0.051	1.351
11/13/2017 1:34	19.93	18.57	20.00	-0.70	1.29	0.042	1.344
11/13/2017 1:35	19.70	17.56	20.60	-0.94	1.29	0.046	1.332
11/13/2017 1:36	19.67	17.98	20.17	-0.23	1.64	0.045	1.335
11/13/2017 1:37	19.70	19.13	20.46	-0.20	1.56	0.032	1.336
11/13/2017 1:38	19.72	18.47	20.47	0.32	2.21	0.053	1.355
11/13/2017 1:39	19.72	17.93	20.69	0.26	2.12	0.045	1.319
11/13/2017 1:40	20.05	19.65	21.54	-0.38	1.18	0.045	1.300
11/13/2017 1:41	20.50	19.67	22.05	-1.02	0.87	0.054	1.308
11/13/2017 1:42	20.95	19.83	21.83	-1.04	0.80	0.034	1.313
11/13/2017 1:43	21.25	19.84	22.20	-0.83	1.07	0.054	1.341
11/13/2017 1:44	21.40	20.12	22.62	-1.09	0.96	0.048	1.344
11/13/2017 1:45	21.58	21.43	22.63	-1.13	1.18	0.040	1.332
11/13/2017 1:46	21.70	19.96	22.79	-1.22	1.01	0.050	1.334
11/13/2017 1:47	21.97	20.57	22.63	-1.20	0.97	0.045	1.320
11/13/2017 1:48	21.87	18.90	21.53	-1.24	0.91	0.046	1.338
11/13/2017 1:49	21.53	18.56	21.52	-1.17	0.97	0.051	1.333
11/13/2017 1:50	21.25	20.78	22.26	-1.17	0.83	0.037	1.338
11/13/2017 1:51	21.37	20.15	22.61	-1.17	0.72	0.047	1.329
11/13/2017 1:52	21.85	20.89	23.57	-1.02	1.17	0.048	1.347
11/13/2017 1:53	22.18	21.39	23.46	-0.91	0.94	0.039	1.335
11/13/2017 1:54	22.45	20.88	23.29	-0.91	0.95	0.047	1.309
11/13/2017 1:55	22.60	21.88	23.01	-1.00	0.99	0.042	1.318
11/13/2017 1:56	22.50	20.48	22.75	-0.98	1.18	0.039	1.326
11/13/2017 1:57	22.00	20.31	21.98	-0.93	1.16	0.042	1.324
11/13/2017 1:58	21.53	20.43	21.85	-0.89	1.18	0.034	1.339
11/13/2017 1:59	21.22	19.07	22.05	-1.03	1.00	0.042	1.348
11/13/2017 2:00	21.23	20.10	21.88	-0.84	1.56	0.048	1.329
11/13/2017 2:01	20.97	19.40	21.04	-0.09	2.23	0.039	1.320
11/13/2017 2:02	20.62	18.57	21.21	-0.14	1.99	0.047	1.315
11/13/2017 2:03	20.42	18.64	20.98	0.44	2.46	0.043	1.314
11/13/2017 2:04	20.40	19.47	21.51	-0.41	1.43	0.039	1.316
11/13/2017 2:05	20.53	18.99	20.67	-0.70	1.44	0.048	1.298
11/13/2017 2:06	20.37	18.15	21.00	-0.85	1.44	0.042	1.334
11/13/2017 2:07	20.30	19.03	20.81	-0.75	1.22	0.042	1.350
11/13/2017 2:08	20.15	18.12	20.89	-0.44	1.52	0.056	1.338
11/13/2017 2:09	20.22	19.74	21.43	-0.05	1.90	0.039	1.321
11/13/2017 2:10	20.45	18.77	22.11	-0.70	1.20	0.037	1.315
11/13/2017 2:11	21.10	21.90	22.86	-0.89	1.30	0.043	1.298
11/13/2017 2:12	21.95	21.60	23.83	-0.98	1.15	0.036	1.293
11/13/2017 2:13	22.48	18.79	22.71	-0.88	1.22	0.047	1.267
11/13/2017 2:14	22.35	21.14	22.35	-0.75	1.25	0.045	1.284
11/13/2017 2:15	21.95	20.66	22.67	-0.62	1.15	0.039	1.307
11/13/2017 2:16	21.77	20.17	22.53	-0.65	1.24	0.045	1.302
11/13/2017 2:17	21.82	18.65	22.64	-0.91	1.14	0.039	1.310
11/13/2017 2:18	21.67	20.15	21.62	-0.68	1.32	0.040	1.322

11/13/2017 2:19	21.35	19.44	21.97	-0.38	1.27	0.049	1.330
11/13/2017 2:20	21.38	20.89	22.93	-0.67	1.53	0.033	1.328
11/13/2017 2:21	21.65	19.91	22.80	-0.18	1.89	0.042	1.363
11/13/2017 2:22	21.98	21.46	23.02	-0.66	1.40	0.048	1.368
11/13/2017 2:23	22.12	20.86	23.13	-0.61	1.25	0.036	1.343
11/13/2017 2:24	22.20	20.39	22.89	-0.95	1.05	0.051	1.329
11/13/2017 2:25	22.23	20.03	22.95	-1.02	0.83	0.038	1.325
11/13/2017 2:26	22.30	20.21	23.39	-1.10	0.85	0.036	1.344
11/13/2017 2:27	22.35	21.56	23.57	-0.54	1.49	0.045	1.361
11/13/2017 2:28	22.48	21.48	23.09	-0.96	1.08	0.030	1.357
11/13/2017 2:29	22.18	20.18	22.48	-0.38	2.05	0.037	1.345
11/13/2017 2:30	21.93	20.64	23.04	-0.68	1.38	0.040	1.323
11/13/2017 2:31	21.65	19.76	22.23	-0.56	1.53	0.033	1.320
11/13/2017 2:32	21.55	19.53	22.04	-0.22	1.68	0.045	1.310
11/13/2017 2:33	21.40	19.50	22.13	-0.23	1.40	0.040	1.328
11/13/2017 2:34	21.43	20.84	22.60	-0.71	1.22	0.030	1.326
11/13/2017 2:35	21.58	19.06	22.73	-1.08	1.23	0.038	1.317
11/13/2017 2:36	21.80	21.22	22.31	-0.87	1.51	0.037	1.305
11/13/2017 2:37	21.73	19.91	21.98	-1.04	1.27	0.029	1.322
11/13/2017 2:38	21.48	19.68	22.18	-0.84	1.46	0.037	1.312
11/13/2017 2:39	21.48	20.81	22.85	-0.81	1.15	0.032	1.301
11/13/2017 2:40	21.62	20.43	22.95	-0.39	1.91	0.038	1.302
11/13/2017 2:41	21.87	21.39	22.78	-1.19	0.97	0.049	1.297
11/13/2017 2:42	22.10	21.52	23.77	-1.36	0.99	0.034	1.311
11/13/2017 2:43	22.33	21.24	23.20	-1.13	1.15	0.042	1.311
11/13/2017 2:44	22.50	20.52	23.06	-0.61	1.29	0.035	1.302
11/13/2017 2:45	22.22	20.07	22.05	-0.39	1.85	0.032	1.314
11/13/2017 2:46	21.68	20.48	22.13	-0.84	1.24	0.037	1.302
11/13/2017 2:47	21.43	21.40	22.27	-1.08	1.53	0.034	1.323
11/13/2017 2:48	21.38	20.36	22.45	-0.61	1.35	0.034	1.317
11/13/2017 2:49	21.52	18.75	22.18	-0.70	1.39	0.039	1.300
11/13/2017 2:50	21.43	20.79	22.42	-1.37	1.05	0.033	1.311
11/13/2017 2:51	21.47	20.07	22.05	-1.22	1.15	0.040	1.298
11/13/2017 2:52	21.52	22.06	22.89	-1.38	0.92	0.032	1.280
11/13/2017 2:53	21.90	21.22	23.37	-1.46	0.91	0.025	1.287
11/13/2017 2:54	22.33	20.90	23.88	-1.48	1.02	0.040	1.290
11/13/2017 2:55	22.80	22.76	23.49	-1.48	1.52	0.032	1.289
11/13/2017 2:56	22.90	20.05	23.35	-1.30	1.40	0.029	1.282
11/13/2017 2:57	22.83	21.45	23.49	-1.41	0.90	0.046	1.283
11/13/2017 2:58	22.85	20.88	23.90	-1.32	0.91	0.033	1.306
11/13/2017 2:59	22.68	20.84	23.03	-0.82	1.23	0.034	1.310
11/13/2017 3:00	22.38	20.63	22.53	-0.50	1.11	0.034	1.311
11/13/2017 3:01	22.03	20.34	22.85	-0.33	1.59	0.027	1.287
11/13/2017 3:02	22.13	21.63	23.51	-1.28	1.19	0.047	1.303
11/13/2017 3:03	22.38	20.24	23.61	-1.39	0.93	0.045	1.315
11/13/2017 3:04	22.42	19.81	22.18	-1.34	0.93	0.034	1.326
11/13/2017 3:05	22.05	19.76	22.40	-1.21	1.08	0.038	1.329

11/13/2017 3:06	21.80	21.83	22.15	-0.54	1.87	0.027	1.333
11/13/2017 3:07	21.55	19.98	21.98	-0.72	1.55	0.030	1.314
11/13/2017 3:08	21.48	20.20	21.84	-0.51	1.30	0.044	1.328
11/13/2017 3:09	21.37	19.41	22.02	-0.84	1.50	0.026	1.336
11/13/2017 3:10	21.28	19.11	21.86	0.15	2.45	0.034	1.327
11/13/2017 3:11	21.35	20.35	22.18	-0.22	1.70	0.034	1.330
11/13/2017 3:12	21.43	20.45	21.96	-0.41	1.98	0.023	1.331
11/13/2017 3:13	21.47	20.18	22.11	-0.44	1.61	0.032	1.350
11/13/2017 3:14	21.27	19.68	21.76	-0.26	1.79	0.038	1.336
11/13/2017 3:15	21.15	18.85	21.90	-0.34	1.63	0.035	1.328
11/13/2017 3:16	21.17	20.18	22.49	-1.37	1.09	0.036	1.344
11/13/2017 3:17	21.40	20.22	22.09	-1.25	1.28	0.039	1.336
11/13/2017 3:18	21.45	21.13	22.57	-0.03	1.78	0.028	1.321
11/13/2017 3:19	21.82	21.62	23.28	-1.46	1.13	0.036	1.335
11/13/2017 3:20	22.00	20.71	22.86	-1.48	1.04	0.029	1.333
11/13/2017 3:21	22.03	20.45	21.79	-1.46	0.95	0.033	1.350
11/13/2017 3:22	21.57	19.27	21.21	-1.12	1.53	0.034	1.340
11/13/2017 3:23	21.28	20.85	22.34	-1.36	1.00	0.026	1.302
11/13/2017 3:24	21.25	19.90	22.43	-1.51	1.07	0.034	1.307
11/13/2017 3:25	21.57	20.47	22.51	-1.51	0.91	0.033	1.331
11/13/2017 3:26	21.72	20.13	22.28	-1.55	0.96	0.021	1.330
11/13/2017 3:27	21.82	22.02	22.19	-1.52	0.97	0.034	1.332
11/13/2017 3:28	21.90	21.26	23.14	-1.55	1.01	0.028	1.332
11/13/2017 3:29	22.22	21.58	23.48	-1.57	0.73	0.032	1.321
11/13/2017 3:30	22.70	21.59	24.10	-1.24	1.11	0.042	1.335
11/13/2017 3:31	23.07	22.47	23.91	-1.23	1.45	0.030	1.355
11/13/2017 3:32	23.15	22.06	23.65	-0.75	1.73	0.031	1.366
11/13/2017 3:33	23.07	21.19	23.14	-0.49	1.45	0.038	1.352
11/13/2017 3:34	22.68	21.73	22.99	-0.64	1.89	0.028	1.341
11/13/2017 3:35	22.77	22.84	24.06	-1.35	1.18	0.032	1.341
11/13/2017 3:36	22.83	21.75	23.66	-1.33	1.43	0.032	1.328
11/13/2017 3:37	22.95	21.34	23.43	-0.92	1.60	0.025	1.327
11/13/2017 3:38	22.67	21.13	22.87	-1.01	1.46	0.037	1.348
11/13/2017 3:39	22.43	21.23	23.04	-0.96	1.66	0.027	1.325
11/13/2017 3:40	22.27	21.27	22.74	0.09	2.63	0.031	1.317
11/13/2017 3:41	22.05	19.66	21.93	-1.23	1.30	0.035	1.350
11/13/2017 3:42	21.67	19.57	21.45	-1.50	1.13	0.021	1.338
11/13/2017 3:43	21.33	19.05	22.27	-1.52	0.98	0.033	1.327
11/13/2017 3:44	21.38	21.50	22.93	-1.54	1.18	0.031	1.326
11/13/2017 3:45	21.97	22.15	23.59	-1.56	1.06	0.026	1.324
11/13/2017 3:46	22.50	20.71	23.70	-1.56	1.03	0.033	1.354
11/13/2017 3:47	22.83	22.19	23.96	-1.58	1.24	0.030	1.360
11/13/2017 3:48	23.03	21.38	23.93	-1.57	1.16	0.029	1.340
11/13/2017 3:49	23.00	21.18	23.47	-1.57	1.19	0.032	1.318
11/13/2017 3:50	23.05	21.97	24.35	-1.37	1.25	0.029	1.312
11/13/2017 3:51	23.30	22.87	24.44	-1.32	1.41	0.031	1.335
11/13/2017 3:52	23.38	21.53	23.94	-1.31	1.28	0.033	1.349

11/13/2017 3:53	23.52	21.34	23.87	-1.51	0.87	0.024	1.346
11/13/2017 3:54	23.13	22.25	23.90	-1.54	1.02	0.031	1.346
11/13/2017 3:55	23.15	20.64	23.66	-1.53	1.29	0.034	1.341
11/13/2017 3:56	22.92	21.06	23.04	-1.53	1.14	0.019	1.341
11/13/2017 3:57	22.68	21.43	22.94	-1.55	0.97	0.032	1.342
11/13/2017 3:58	22.42	21.52	22.60	-1.57	1.15	0.032	1.338
11/13/2017 3:59	22.30	20.73	22.76	-1.48	1.32	0.024	1.344
11/13/2017 4:00	22.12	20.58	22.74	-1.56	0.93	0.027	1.328
11/13/2017 4:01	22.17	21.54	23.60	-1.54	1.04	0.026	1.320
11/13/2017 4:02	22.30	20.78	22.93	-1.58	1.10	0.024	1.342
11/13/2017 4:03	22.30	21.65	22.09	-1.54	0.97	0.037	1.337
11/13/2017 4:04	22.05	21.14	22.86	-0.84	1.74	0.022	1.337
11/13/2017 4:05	21.85	18.78	22.45	-0.01	2.57	0.035	1.333
11/13/2017 4:06	21.67	19.64	21.76	0.36	2.70	0.027	1.307
11/13/2017 4:07	21.52	21.28	21.87	-0.68	1.52	0.022	1.303
11/13/2017 4:08	21.45	21.68	22.25	-1.06	1.37	0.030	1.319
11/13/2017 4:09	21.68	20.97	22.74	-0.86	1.35	0.025	1.326
11/13/2017 4:10	22.17	21.50	23.63	-1.32	1.20	0.028	1.321
11/13/2017 4:11	22.65	22.23	23.92	-0.84	1.20	0.033	1.308
11/13/2017 4:12	22.90	21.68	23.30	-1.28	1.19	0.022	1.334
11/13/2017 4:13	22.77	21.92	22.86	-1.22	0.91	0.030	1.353
11/13/2017 4:14	22.35	20.04	21.68	-1.16	1.27	0.031	1.351
11/13/2017 4:15	21.75	20.83	21.46	-0.69	1.43	0.014	1.348
11/13/2017 4:16	21.48	20.51	22.59	-0.73	1.40	0.027	1.357
11/13/2017 4:17	21.62	20.49	22.49	-0.57	1.46	0.020	1.336
11/13/2017 4:18	21.98	20.79	23.38	-0.76	1.27	0.024	1.344
11/13/2017 4:19	22.30	19.98	22.99	-0.67	1.24	0.033	1.365
11/13/2017 4:20	22.32	19.70	22.76	0.11	2.05	0.026	1.375
11/13/2017 4:21	22.25	22.36	22.53	-0.06	1.62	0.026	1.347
11/13/2017 4:22	22.23	20.68	23.26	-0.46	1.52	0.029	1.340
11/13/2017 4:23	22.37	21.91	23.63	0.11	1.71	0.019	1.345
11/13/2017 4:24	22.75	21.08	23.25	-0.90	1.23	0.028	1.338
11/13/2017 4:25	22.68	20.99	23.09	-0.16	1.39	0.025	1.332
11/13/2017 4:26	22.72	21.26	23.67	-1.03	1.08	0.018	1.340
11/13/2017 4:27	22.70	22.48	23.62	-1.15	0.99	0.031	1.312
11/13/2017 4:28	22.83	20.76	23.31	-1.16	1.07	0.027	1.287
11/13/2017 4:29	22.78	20.89	23.34	-0.86	1.33	0.021	1.303
11/13/2017 4:30	22.48	19.81	23.30	-0.90	1.46	0.032	1.309
11/13/2017 4:31	22.30	19.66	22.73	-0.80	1.16	0.017	1.306
11/13/2017 4:32	22.17	20.86	22.98	-0.73	1.33	0.031	1.310
11/13/2017 4:33	22.40	21.96	23.74	-0.93	1.02	0.030	1.313
11/13/2017 4:34	22.55	20.19	22.88	-0.92	1.63	0.019	1.320
11/13/2017 4:35	22.57	19.76	22.77	-0.40	1.53	0.030	1.335
11/13/2017 4:36	22.18	20.59	22.70	-0.96	1.14	0.022	1.318
11/13/2017 4:37	22.07	22.05	23.44	-1.29	1.04	0.019	1.317
11/13/2017 4:38	22.33	22.68	23.78	-1.29	1.08	0.030	1.312
11/13/2017 4:39	22.43	20.92	22.37	-0.10	2.31	0.011	1.339

11/13/2017 4:40	21.92	18.80	20.64	0.81	2.04	0.028	1.345
11/13/2017 4:41	20.98	20.98	22.09	1.20	1.94	0.029	1.341
11/13/2017 4:42	21.22	22.08	24.35	-0.99	1.05	0.011	1.334
11/13/2017 4:43	22.13	21.68	24.29	-1.26	0.77	0.034	1.308
11/13/2017 4:44	23.37	22.66	24.76	-1.22	0.86	0.022	1.317
11/13/2017 4:45	23.43	21.50	23.62	-1.21	0.82	0.012	1.343
11/13/2017 4:46	23.20	20.80	23.45	-1.07	1.27	0.025	1.367
11/13/2017 4:47	23.05	21.73	23.97	-1.09	1.05	0.018	1.352
11/13/2017 4:48	22.92	21.49	23.55	-0.75	1.39	0.017	1.336
11/13/2017 4:49	23.00	20.80	23.57	-0.46	1.13	0.033	1.351
11/13/2017 4:50	22.93	21.37	24.24	-1.00	1.00	0.014	1.344
11/13/2017 4:51	23.10	21.83	23.72	-1.09	0.82	0.027	1.359
11/13/2017 4:52	23.08	21.63	23.79	-1.16	0.73	0.021	1.368
11/13/2017 4:53	23.22	21.44	24.15	-1.17	0.77	0.018	1.353
11/13/2017 4:54	23.22	20.94	23.63	-1.00	1.01	0.029	1.331
11/13/2017 4:55	23.22	21.76	23.42	-0.29	1.72	0.024	1.370
11/13/2017 4:56	22.88	20.35	23.15	0.27	1.38	0.019	1.378
11/13/2017 4:57	22.57	22.31	22.95	0.27	1.24	0.025	1.368
11/13/2017 4:58	22.50	21.02	23.54	0.06	1.52	0.008	1.370
11/13/2017 4:59	22.40	20.63	22.58	0.07	1.71	0.016	1.346
11/13/2017 5:00	22.23	20.24	22.79	-0.05	1.39	0.028	1.357
11/13/2017 5:01	22.08	19.66	23.23	-0.40	1.24	0.009	1.369
11/13/2017 5:02	22.22	21.16	23.44	-0.80	1.18	0.023	1.400
11/13/2017 5:03	22.40	21.83	22.95	-0.82	1.37	0.020	1.408
11/13/2017 5:04	22.47	21.10	23.39	-1.09	1.22	0.010	1.389
11/13/2017 5:05	22.48	21.84	23.03	-1.25	0.97	0.025	1.386
11/13/2017 5:06	22.33	21.16	22.85	-1.00	1.06	0.022	1.382
11/13/2017 5:07	22.27	20.60	22.88	-1.18	0.90	0.019	1.369
11/13/2017 5:08	22.03	21.17	22.67	-1.01	0.92	0.029	1.381
11/13/2017 5:09	22.13	20.04	23.12	-1.09	1.27	0.020	1.390
11/13/2017 5:10	22.25	21.11	23.30	-1.16	0.93	0.017	1.380
11/13/2017 5:11	22.48	21.53	23.29	-1.30	1.04	0.027	1.361
11/13/2017 5:12	22.48	20.83	22.49	-1.29	0.94	0.016	1.374
11/13/2017 5:13	22.28	20.81	22.35	-1.28	0.92	0.023	1.381
11/13/2017 5:14	22.00	21.00	22.67	-1.27	1.06	0.026	1.371
11/13/2017 5:15	21.87	20.02	22.96	-1.20	1.08	0.013	1.380
11/13/2017 5:16	21.82	20.03	21.95	-1.31	0.94	0.030	1.371
11/13/2017 5:17	21.60	21.14	22.28	-1.38	0.72	0.021	1.366
11/13/2017 5:18	21.62	21.86	22.97	-1.40	0.90	0.019	1.355
11/13/2017 5:19	21.93	20.94	22.91	-1.43	0.99	0.033	1.355
11/13/2017 5:20	22.20	20.19	22.38	-1.43	1.00	0.022	1.328
11/13/2017 5:21	22.25	22.08	23.14	-1.38	1.15	0.026	1.322
11/13/2017 5:22	22.37	20.49	23.31	-1.25	1.12	0.025	1.349
11/13/2017 5:23	22.47	20.43	22.38	-0.50	1.88	0.020	1.351
11/13/2017 5:24	22.22	20.58	22.07	-0.50	1.23	0.036	1.344
11/13/2017 5:25	21.58	20.63	21.33	-0.97	0.97	0.023	1.353
11/13/2017 5:26	21.17	19.74	21.67	-1.17	0.99	0.019	1.351

11/13/2017 5:27	21.00	18.47	21.65	-1.13	0.77	0.031	1.360
11/13/2017 5:28	21.00	19.33	21.83	-1.08	0.96	0.015	1.343
11/13/2017 5:29	20.93	18.80	21.44	-0.99	0.92	0.025	1.361
11/13/2017 5:30	20.87	18.99	21.78	-0.43	1.43	0.029	1.362
11/13/2017 5:31	20.77	19.13	21.19	-0.55	0.99	0.020	1.363
11/13/2017 5:32	20.63	20.15	21.43	-0.22	1.31	0.030	1.373
11/13/2017 5:33	20.57	17.58	21.03	-0.01	1.43	0.029	1.378
11/13/2017 5:34	20.27	16.54	20.17	-0.12	1.50	0.019	1.388
11/13/2017 5:35	19.93	18.36	20.54	0.67	2.14	0.024	1.399
11/13/2017 5:36	19.78	19.23	21.23	-0.21	1.26	0.018	1.382
11/13/2017 5:37	20.23	19.73	22.28	-1.06	1.07	0.024	1.361
11/13/2017 5:38	20.68	18.83	21.54	-0.84	1.16	0.033	1.389
11/13/2017 5:39	20.88	19.18	21.51	-0.85	1.23	0.017	1.389
11/13/2017 5:40	20.92	19.18	21.82	-1.13	1.12	0.037	1.392
11/13/2017 5:41	21.20	20.76	22.02	-1.01	1.05	0.026	1.379
11/13/2017 5:42	21.37	19.88	21.91	-0.76	1.64	0.023	1.372
11/13/2017 5:43	21.35	19.91	21.77	-0.80	1.17	0.030	1.381
11/13/2017 5:44	20.88	19.14	21.11	-0.18	2.19	0.019	1.394
11/13/2017 5:45	20.47	17.47	20.58	0.15	1.78	0.029	1.392
11/13/2017 5:46	19.90	18.75	20.30	0.31	1.54	0.027	1.402
11/13/2017 5:47	19.65	17.79	20.23	-0.66	1.15	0.021	1.393
11/13/2017 5:48	19.65	20.29	21.27	-0.32	1.45	0.020	1.403
11/13/2017 5:49	20.33	22.53	23.39	-0.92	0.97	0.026	1.408
11/13/2017 5:50	21.40	22.03	23.85	-1.29	1.02	0.014	1.395
11/13/2017 5:51	22.62	22.23	24.22	-1.34	0.73	0.030	1.400
11/13/2017 5:52	23.18	22.19	23.61	-1.36	0.89	0.019	1.396
11/13/2017 5:53	23.38	23.09	24.50	-1.38	0.93	0.019	1.400
11/13/2017 5:54	23.57	21.57	24.12	-1.31	0.73	0.033	1.427
11/13/2017 5:55	23.30	22.06	23.06	-0.84	1.20	0.009	1.421
11/13/2017 5:56	23.03	21.88	23.80	-0.67	1.58	0.024	1.413
11/13/2017 5:57	22.72	21.52	23.12	-1.13	1.12	0.027	1.409
11/13/2017 5:58	22.75	21.72	23.59	-1.13	1.28	0.018	1.420
11/13/2017 5:59	22.83	20.63	23.08	-0.74	1.49	0.024	1.405
11/13/2017 6:00	22.67	20.83	22.97	-0.96	0.92	0.026	1.406
11/13/2017 6:01	22.70	21.10	23.23	-1.30	0.86	0.013	1.389
11/13/2017 6:02	22.62	20.72	23.68	-1.12	1.27	0.024	1.413
11/13/2017 6:03	22.72	20.51	23.29	-1.37	1.12	0.023	1.408
11/13/2017 6:04	22.70	21.06	23.54	-1.29	1.03	0.017	1.416
11/13/2017 6:05	22.78	20.51	23.13	-1.16	1.02	0.022	1.443
11/13/2017 6:06	22.78	20.93	23.55	-1.33	1.03	0.012	1.434
11/13/2017 6:07	22.90	20.91	23.56	-1.36	0.66	0.016	1.399
11/13/2017 6:08	22.98	21.15	23.75	-1.41	0.98	0.023	1.390
11/13/2017 6:09	23.00	20.57	23.48	-1.40	1.13	0.015	1.391
11/13/2017 6:10	22.83	22.29	23.11	-1.42	0.93	0.015	1.410
11/13/2017 6:11	22.80	21.63	23.51	-1.43	0.91	0.022	1.378
11/13/2017 6:12	22.78	22.34	23.28	-1.43	0.94	0.007	1.365
11/13/2017 6:13	22.78	20.48	23.40	-1.44	0.95	0.023	1.351

11/13/2017 6:14	22.73	21.24	23.17	-1.42	0.84	0.024	1.343
11/13/2017 6:15	22.43	21.28	22.98	-1.39	1.06	0.013	1.347
11/13/2017 6:16	22.28	19.67	23.12	-1.08	1.22	0.029	1.356
11/13/2017 6:17	22.35	21.61	23.83	-1.38	0.88	0.017	1.361
11/13/2017 6:18	22.67	22.07	24.29	-0.97	1.25	0.014	1.346
11/13/2017 6:19	23.18	23.42	24.57	-1.12	1.03	0.029	1.357
11/13/2017 6:20	23.45	21.79	24.19	-1.19	0.98	0.014	1.364
11/13/2017 6:21	23.55	21.60	24.23	-1.24	0.96	0.021	1.340
11/13/2017 6:22	23.40	21.39	23.64	-0.49	2.30	0.021	1.345
11/13/2017 6:23	23.00	22.98	23.63	-0.21	1.81	0.012	1.352
11/13/2017 6:24	22.75	22.05	23.67	-1.03	1.15	0.024	1.347
11/13/2017 6:25	22.75	20.74	24.00	-1.31	0.81	0.015	1.357
11/13/2017 6:26	22.80	22.95	23.38	-1.18	0.70	0.015	1.364
11/13/2017 6:27	22.63	21.34	23.11	-1.04	1.01	0.016	1.359
11/13/2017 6:28	22.18	21.41	22.65	-0.59	1.52	0.017	1.379
11/13/2017 6:29	21.95	20.83	22.52	-0.75	1.51	0.016	1.371
11/13/2017 6:30	21.73	19.73	22.57	-0.96	1.14	0.024	1.375
11/13/2017 6:31	21.93	21.82	23.55	-0.27	1.80	0.010	1.390
11/13/2017 6:32	22.33	22.23	24.25	-0.78	1.14	0.022	1.381
11/13/2017 6:33	22.87	21.54	23.82	-0.76	1.38	0.016	1.398
11/13/2017 6:34	22.97	21.51	22.78	-0.42	1.32	0.009	1.401
11/13/2017 6:35	22.48	20.61	22.29	0.53	1.69	0.025	1.390
11/13/2017 6:36	21.93	20.10	22.50	0.79	1.71	0.020	1.391
11/13/2017 6:37	21.77	20.40	23.02	0.10	1.43	0.014	1.401
11/13/2017 6:38	22.28	22.55	24.65	-0.27	1.31	0.025	1.394
11/13/2017 6:39	23.08	22.30	24.49	-0.07	1.83	0.011	1.387
11/13/2017 6:40	24.00	23.87	25.90	-1.03	0.81	0.012	1.375
11/13/2017 6:41	24.38	21.99	25.04	-0.40	1.78	0.025	1.378
11/13/2017 6:42	24.63	24.32	25.37	-0.93	0.97	0.010	1.373
11/13/2017 6:43	24.50	22.76	24.83	-0.90	1.12	0.014	1.371
11/13/2017 6:44	24.48	22.66	25.01	-0.92	0.96	0.023	1.370
11/13/2017 6:45	24.22	21.03	24.14	-0.61	1.73	0.005	1.363
11/13/2017 6:46	24.08	23.38	25.59	-0.89	1.45	0.018	1.372
11/13/2017 6:47	24.33	23.72	25.57	-1.06	0.87	0.011	1.392
11/13/2017 6:48	24.67	22.50	25.11	-0.77	1.07	0.016	1.392
11/13/2017 6:49	24.62	21.82	24.47	-0.57	1.23	0.022	1.380
11/13/2017 6:50	24.17	23.66	24.64	-0.87	0.98	0.008	1.355
11/13/2017 6:51	23.93	22.15	25.10	-1.04	0.92	0.015	1.345
11/13/2017 6:52	23.82	22.59	23.83	-1.08	0.65	0.022	1.360
11/13/2017 6:53	23.42	20.71	23.22	-0.99	0.70	0.011	1.353
11/13/2017 6:54	22.98	21.13	23.76	-0.93	0.91	0.021	1.356
11/13/2017 6:55	22.75	22.06	24.02	-1.12	0.85	0.017	1.356
11/13/2017 6:56	23.23	21.57	24.84	-1.31	0.96	0.009	1.343
11/13/2017 6:57	23.90	22.37	25.57	-1.45	1.15	0.020	1.359
11/13/2017 6:58	24.40	22.58	25.08	-1.53	0.65	0.015	1.360
11/13/2017 6:59	24.48	21.95	24.78	-1.54	0.87	0.012	1.360
11/13/2017 7:00	24.45	23.38	25.54	-1.54	0.93	0.019	1.368

11/13/2017 7:01	24.60	22.87	25.17	-1.56	0.92	0.007	1.355
11/13/2017 7:02	24.78	23.26	25.50	-0.68	1.82	0.019	1.359
11/13/2017 7:03	24.83	22.89	25.03	-1.28	1.15	0.023	1.349
11/13/2017 7:04	24.68	23.83	24.92	-1.24	1.02	0.013	1.341
11/13/2017 7:05	24.58	22.24	25.14	-0.97	1.16	0.026	1.369
11/13/2017 7:06	24.27	21.93	24.04	-0.48	1.31	0.023	1.378
11/13/2017 7:07	23.93	21.91	24.11	-0.45	1.17	0.012	1.367
11/13/2017 7:08	23.83	23.63	25.21	-0.91	1.09	0.024	1.383
11/13/2017 7:09	24.15	23.92	25.12	-1.29	1.21	0.011	1.379
11/13/2017 7:10	24.65	23.33	25.52	-1.33	1.36	0.012	1.383
11/13/2017 7:11	24.63	23.10	24.88	-0.28	1.97	0.022	1.388
11/13/2017 7:12	24.73	24.27	25.88	-1.21	1.03	0.009	1.401
11/13/2017 7:13	24.73	22.78	25.77	-1.29	0.96	0.014	1.414
11/13/2017 7:14	25.12	24.79	26.02	-1.35	0.89	0.028	1.414
11/13/2017 7:15	25.33	23.07	26.51	-1.25	0.80	0.231	1.457
11/13/2017 7:16	25.63	23.79	25.77	-1.44	0.99	0.020	1.404
11/13/2017 7:17	25.43	22.59	25.32	-1.31	1.13	0.020	1.386
11/13/2017 7:18	24.97	22.29	25.08	-0.40	1.79	0.009	1.393
11/13/2017 7:19	24.42	24.33	24.99	-1.03	1.04	0.021	1.392
11/13/2017 7:20	24.22	22.69	24.55	-1.25	1.11	0.011	1.401
11/13/2017 7:21	23.98	23.26	23.75	-1.31	0.98	0.020	1.385
11/13/2017 7:22	23.68	22.11	23.52	-1.11	1.02	0.022	1.370
11/13/2017 7:23	23.35	21.43	23.91	-0.92	0.92	0.007	1.386
11/13/2017 7:24	23.12	20.20	23.18	-1.13	1.02	0.017	1.383
11/13/2017 7:25	23.17	22.95	24.58	-1.08	0.88	0.024	1.388
11/13/2017 7:26	23.62	22.68	25.23	-1.10	0.78	0.005	1.373
11/13/2017 7:27	24.03	21.09	24.60	-0.83	1.89	0.038	1.356
11/13/2017 7:28	23.18	19.83	21.55	3.16	39.50	0.184	1.380
11/13/2017 7:29	22.32	21.56	24.35	0.00	1.47	0.013	1.366
11/13/2017 7:30	22.10	21.74	24.30	-1.25	1.23	0.021	1.368
11/13/2017 7:31	23.20	23.72	24.93	-1.26	0.86	0.008	1.379
11/13/2017 7:32	23.90	22.13	24.32	-1.34	0.85	0.016	1.361
11/13/2017 7:33	23.98	21.96	24.92	-1.35	0.76	0.016	1.342
11/13/2017 7:34	23.87	22.98	23.98	-1.35	0.69	0.008	1.347
11/13/2017 7:35	23.82	23.71	24.60	-1.36	1.14	0.017	1.339
11/13/2017 7:36	23.88	22.23	24.92	-1.36	0.77	0.016	1.348
11/13/2017 7:37	24.13	23.17	25.09	-1.33	0.84	0.011	1.342
11/13/2017 7:38	24.30	23.11	25.20	-1.27	0.83	0.171	1.356
11/13/2017 7:39	23.92	21.99	24.05	0.01	2.51	1.304	1.505
11/13/2017 7:40	23.35	22.65	23.99	-0.49	1.10	0.303	1.482
11/13/2017 7:41	23.10	22.66	24.01	-0.95	0.88	0.048	1.661
11/13/2017 7:42	23.13	23.01	24.53	-0.60	1.28	0.019	1.672
11/13/2017 7:43	23.38	19.18	24.09	-0.83	1.12	0.041	1.417
11/13/2017 7:44	23.30	24.53	25.03	-0.68	1.38	0.356	1.403
11/13/2017 7:45	23.75	22.57	24.78	-1.17	0.98	0.015	1.368
11/13/2017 7:46	24.02	22.98	24.78	-1.10	1.00	0.009	1.361
11/13/2017 7:47	24.47	23.62	25.09	-1.07	0.82	0.020	1.358

11/13/2017 7:48	24.50	22.99	25.47	-1.12	0.96	0.012	1.323
11/13/2017 7:49	24.57	22.66	24.53	-0.73	1.40	0.008	1.340
11/13/2017 7:50	24.62	23.83	25.18	-0.71	1.49	0.013	1.334
11/13/2017 7:51	24.63	23.53	25.02	-0.86	1.24	0.007	1.330
11/13/2017 7:52	24.55	22.95	25.19	-0.35	1.45	0.015	1.353
11/13/2017 7:53	24.78	24.28	26.50	-0.59	0.93	0.018	1.331
11/13/2017 7:54	25.03	23.54	26.00	-1.13	1.12	0.005	1.319
11/13/2017 7:55	25.45	25.55	26.15	-1.18	1.03	0.014	1.309
11/13/2017 7:56	25.55	24.02	26.18	-0.58	1.83	0.019	1.358
11/13/2017 7:57	25.45	23.92	26.44	-0.20	1.69	0.009	1.371
11/13/2017 7:58	25.53	22.26	26.04	-0.81	1.58	0.025	1.351
11/13/2017 7:59	25.33	22.51	25.49	-0.13	1.31	0.019	1.391
11/13/2017 8:00	25.23	23.80	26.26	-0.21	1.41	0.008	1.388
11/13/2017 8:01	25.02	22.88	25.47	-0.31	1.73	0.017	1.383
11/13/2017 8:02	24.85	22.64	24.37	-0.71	1.53	0.014	1.383
11/13/2017 8:03	24.52	22.74	24.47	-0.91	1.32	0.009	1.393
11/13/2017 8:04	24.10	22.30	24.59	-1.24	1.01	0.015	1.380
11/13/2017 8:05	24.07	22.53	24.95	-0.86	1.35	0.008	1.360
11/13/2017 8:06	24.38	23.82	25.91	-0.84	1.16	0.011	1.318
11/13/2017 8:07	24.83	25.04	26.02	-1.11	1.17	0.013	1.305
11/13/2017 8:08	25.47	24.83	26.72	-1.33	0.96	0.003	1.319
11/13/2017 8:09	25.77	24.35	26.25	-1.32	1.01	0.015	1.339
11/13/2017 8:10	25.87	24.26	26.08	-1.40	1.06	0.009	1.301
11/13/2017 8:11	25.72	24.14	26.08	-1.00	1.14	0.000	1.305
11/13/2017 8:12	25.65	24.03	26.06	-1.58	0.86	0.007	1.321
11/13/2017 8:13	25.28	21.45	24.17	1.34	4.40	0.012	1.246
11/13/2017 8:14	24.50	21.63	23.86	-0.78	2.17	0.000	1.216
11/13/2017 8:15	23.52	20.20	22.88	-1.28	1.34	0.000	1.196
11/13/2017 8:16	22.77	20.63	23.67	-0.62	1.57	0.007	1.219
11/13/2017 8:17	22.97	21.98	24.77	-0.22	2.31	-0.002	1.241
11/13/2017 8:18	23.23	21.89	23.86	3.88	4.23	-0.006	1.238
11/13/2017 8:19	23.47	22.08	23.72	0.83	6.10	0.089	1.229
11/13/2017 8:20	23.32	22.23	25.58	1.41	4.12	0.034	1.167
11/13/2017 8:21	23.60	21.41	24.79	-0.02	1.58	0.000	1.162
11/13/2017 8:22	23.70	20.88	24.02	-0.90	1.60	0.012	1.185
11/13/2017 8:23	23.68	21.23	23.63	-1.10	1.35	0.007	1.171
11/13/2017 8:24	23.22	22.28	23.36	-0.91	1.80	-0.007	1.147
11/13/2017 8:25	22.72	20.10	22.61	-0.88	1.30	0.002	1.152
11/13/2017 8:26	22.25	21.08	22.57	-1.09	1.35	0.002	1.172
11/13/2017 8:27	21.95	20.03	22.95	-0.41	1.58	-0.011	1.198
11/13/2017 8:28	21.98	20.70	23.14	-1.26	1.11	-0.004	1.193
11/13/2017 8:29	22.02	20.07	22.75	-0.39	1.72	0.006	1.153
11/13/2017 8:30	21.80	19.73	22.27	0.48	2.01	-0.003	1.092
11/13/2017 8:31	21.38	18.47	21.75	0.48	1.95	-0.007	1.098
11/13/2017 8:32	20.92	20.47	21.98	1.88	3.33	0.010	1.105
11/13/2017 8:33	21.08	19.77	22.51	0.18	2.03	0.009	1.115
11/13/2017 8:34	21.28	19.43	22.01	0.36	2.17	0.003	1.111

11/13/2017 8:35	21.40	20.51	21.96	0.70	1.65	0.017	1.108
11/13/2017 8:36	21.27	19.15	22.02	0.60	1.82	0.015	1.108
11/13/2017 8:37	21.22	21.50	22.64	0.21	1.27	0.006	1.096
11/13/2017 8:38	21.60	19.58	23.26	0.79	2.20	0.018	1.122
11/13/2017 8:39	22.15	22.28	24.32	1.98	3.92	0.023	1.064
11/13/2017 8:40	22.87	22.31	23.84	0.11	1.83	0.011	1.060
11/13/2017 8:41	23.38	21.43	23.98	-0.55	1.34	0.013	1.058
11/13/2017 8:42	23.50	20.86	23.71	-0.82	1.19	0.023	1.068
11/13/2017 8:43	23.52	20.44	24.45	-0.81	1.20	0.018	1.059
11/13/2017 8:44	23.48	22.00	24.18	-0.60	1.00	0.020	1.075
11/13/2017 8:45	23.53	21.09	23.67	-0.39	1.32	0.026	1.055
11/13/2017 8:46	23.35	21.88	23.40	0.35	1.83	0.018	1.059
11/13/2017 8:47	23.07	21.73	23.37	-0.76	1.08	0.018	1.113
11/13/2017 8:48	22.77	21.02	23.17	-0.48	1.39	0.035	1.137
11/13/2017 8:49	22.77	21.36	23.80	-0.61	1.23	0.026	1.155
11/13/2017 8:50	22.27	15.23	18.15	6.51	39.02	0.525	1.179
11/13/2017 8:51	19.78	18.05	19.95	4.59	35.69	0.323	1.193
11/13/2017 8:52	19.72	21.13	24.60	-0.21	1.34	0.039	1.160
11/13/2017 8:53	20.40	20.08	23.22	-0.36	1.93	0.027	1.175
11/13/2017 8:54	22.45	18.43	22.15	1.68	5.90	0.049	1.171
11/13/2017 8:55	22.48	21.83	23.55	0.37	1.48	0.039	1.248
11/13/2017 8:56	22.30	22.41	23.78	0.76	1.93	0.025	1.247
11/13/2017 8:57	23.07	21.17	24.01	0.61	1.41	0.033	1.222
11/13/2017 8:58	23.37	23.25	24.19	-0.01	1.42	0.044	1.203
11/13/2017 8:59	23.68	23.24	24.26	0.48	2.03	0.027	1.217
11/13/2017 9:00	23.82	22.81	24.19	0.07	0.92	0.035	1.190
11/13/2017 9:01	23.85	21.05	23.93	-0.15	0.79	0.042	1.190
11/13/2017 9:02	23.60	22.04	23.11	-0.14	1.03	0.031	1.170
11/13/2017 9:03	23.35	22.62	23.62	-0.51	0.70	0.034	1.213
11/13/2017 9:04	23.13	21.13	23.19	-0.52	1.03	0.035	1.222
11/13/2017 9:05	23.07	20.86	23.57	-0.02	1.26	0.029	1.235
11/13/2017 9:06	23.20	22.36	23.82	-0.53	0.66	0.039	1.239
11/13/2017 9:07	23.23	20.03	23.23	0.00	1.36	0.041	1.222
11/13/2017 9:08	23.05	21.29	22.87	0.51	1.35	0.034	1.240
11/13/2017 9:09	23.00	21.40	24.05	0.34	1.33	0.029	1.267
11/13/2017 9:10	23.15	21.66	23.86	0.21	1.19	0.042	1.284
11/13/2017 9:11	23.42	21.61	23.68	0.17	1.22	0.031	1.268
11/13/2017 9:12	23.35	21.18	23.82	-0.01	0.87	0.035	1.257
11/13/2017 9:13	23.20	20.24	23.80	-0.08	1.57	0.040	1.250
11/13/2017 9:14	23.05	19.93	22.89	-0.03	1.13	0.028	1.263
11/13/2017 9:15	23.13	22.48	23.80	0.00	1.26	0.040	1.276
11/13/2017 9:16	23.47	22.13	24.83	0.14	1.04	0.039	1.282
11/13/2017 9:17	23.90	21.73	24.16	-0.32	0.99	0.029	1.279
11/13/2017 9:18	24.05	22.12	24.27	0.24	1.81	0.033	1.298
11/13/2017 9:19	23.72	21.73	23.54	1.47	2.41	0.039	1.304
11/13/2017 9:20	23.53	23.12	24.03	1.40	2.11	0.028	1.301
11/13/2017 9:21	23.75	23.89	25.03	0.20	1.32	0.032	1.319

11/13/2017 9:22	24.32	21.59	25.36	-0.38	0.78	0.040	1.319
11/13/2017 9:23	24.85	22.53	25.55	-0.34	0.71	0.029	1.324
11/13/2017 9:24	24.93	22.52	24.70	-0.23	1.04	0.033	1.329
11/13/2017 9:25	24.35	22.08	23.61	0.31	1.21	0.046	1.348
11/13/2017 9:26	23.80	21.13	23.64	0.68	1.72	0.035	1.348
11/13/2017 9:27	23.40	21.70	24.08	0.75	1.58	0.030	1.356
11/13/2017 9:28	23.72	23.71	24.98	-0.19	1.02	0.039	1.334
11/13/2017 9:29	24.28	22.71	24.95	-0.08	1.31	0.034	1.309
11/13/2017 9:30	24.63	23.59	25.04	-0.15	1.12	0.030	1.318
11/13/2017 9:31	24.82	23.63	25.22	0.01	1.64	0.036	1.322
11/13/2017 9:32	24.57	20.72	24.12	0.50	1.80	0.039	1.298
11/13/2017 9:33	24.62	24.30	25.57	-0.10	1.00	0.028	1.310
11/13/2017 9:34	24.68	23.21	24.87	-0.20	1.44	0.040	1.325
11/13/2017 9:35	24.68	21.92	24.61	0.02	1.83	0.036	1.326
11/13/2017 9:36	24.40	21.28	24.00	0.13	1.45	0.027	1.330
11/13/2017 9:37	23.92	21.76	24.01	0.17	1.85	0.042	1.348
11/13/2017 9:38	23.83	26.20	29.02	0.20	2.20	0.406	1.669
11/13/2017 9:39	23.65	29.52	31.72	2.25	4.77	3.056	3.116
11/13/2017 9:40	22.98	23.13	23.46	5.54	9.99	3.773	1.910
11/13/2017 9:41	22.57	23.82	24.00	4.73	9.08	2.959	2.326
11/13/2017 9:42	22.28	20.67	23.49	5.53	15.70	4.665	2.732
11/13/2017 9:43	21.92	20.62	22.68	9.94	24.93	8.269	2.797
11/13/2017 9:44	22.07	23.21	24.48	5.12	7.72	2.991	2.619
11/13/2017 9:45	22.42	20.18	23.14	5.40	10.03	3.151	2.586
11/13/2017 9:46	23.00	21.10	23.85	6.02	7.74	2.810	2.678
11/13/2017 9:47	22.33	20.84	22.16	9.03	31.67	22.672	4.394
11/13/2017 9:48	21.80	22.29	23.44	5.02	7.99	1.271	5.319
11/13/2017 9:49	22.73	24.33	23.99	1.77	1.03	0.139	2.754
11/13/2017 9:50	24.52	25.63	26.26	1.09	0.98	0.035	0.946
11/13/2017 9:51	26.05	23.62	26.63	1.12	1.41	0.258	1.028
11/13/2017 9:52	26.53	25.48	26.96	1.01	0.98	0.054	0.796
11/13/2017 9:53	26.70	23.38	25.94	0.50	0.94	0.031	0.879
11/13/2017 9:54	26.77	23.68	25.44	0.15	0.77	0.046	5.528
11/13/2017 9:55	26.78	23.75	26.93	-0.19	0.74	0.035	6.462
11/13/2017 9:56	26.70	26.12	26.83	-0.51	0.95	0.022	1.653
11/13/2017 9:57		24.93	27.41	-0.71	0.69	0.040	5.970
11/13/2017 9:58		25.13	26.20	-0.81	0.91	0.042	5.784
11/13/2017 9:59		25.22	27.39	-1.03	0.77	0.014	6.941
11/13/2017 10:00		24.35	27.32	-0.71	1.65	0.565	6.886
11/13/2017 10:01		25.85	26.58	1.10	2.66	1.378	1.486
11/13/2017 10:02		26.40	27.40	0.79	0.90	0.042	1.158
11/13/2017 10:03		25.93	27.18	0.54	1.23	0.248	0.885
11/13/2017 10:04		22.02	24.52	3.46	43.58	0.710	4.258
11/13/2017 10:05		22.73	27.11	1.93	10.82	0.173	6.636
11/13/2017 10:06		26.36	28.35	0.22	1.59	0.054	7.764
11/13/2017 10:07		25.43	31.44	-0.60	1.24	0.219	3.691
11/13/2017 10:08		25.53	30.86	-0.85	1.14	0.108	7.998

11/13/2017 10:09		24.86	27.92	0.04	2.43	1.157	5.263
11/13/2017 10:10		22.17	25.74	6.28	11.73	5.610	0.894
11/13/2017 10:11		24.12	26.36	5.06	8.77	4.723	0.928
11/13/2017 10:12		22.94	25.87	6.76	12.49	6.361	0.965
11/13/2017 10:13		23.39	25.59	5.27	10.47	5.660	0.975
11/13/2017 10:14		25.54	26.56	8.95	11.72	6.181	0.988
11/13/2017 10:15		23.21	26.20	10.06	13.36	7.322	0.978
11/13/2017 10:16		23.81	26.44	9.99	10.17	5.153	0.919
11/13/2017 10:17		24.43	26.87	4.86	8.01	3.871	0.810
11/13/2017 10:18		23.93	26.87	4.82	8.78	4.777	0.864
11/13/2017 10:19		24.24	27.15	2.72	5.37	2.622	0.738
11/13/2017 10:20		23.38	27.07	0.53	3.44	1.454	0.628
11/13/2017 10:21		24.44	26.95	0.97	4.81	2.329	0.665
11/13/2017 10:22		22.30	26.52	2.66	8.02	4.055	0.706
11/13/2017 10:23		22.21	26.43	8.41	14.36	6.811	0.804
11/13/2017 10:24		22.16	25.38	11.19	20.06	7.134	0.800
11/13/2017 10:25		22.96	24.80	7.26	14.34	2.033	0.610
11/13/2017 10:26		21.19	24.43	5.21	16.49	0.056	0.531
11/13/2017 10:27		22.93	26.03	0.29	6.96	0.019	0.504
11/13/2017 10:28		23.30	26.02	0.66	6.16	0.024	0.523
11/13/2017 10:29	1.17	26.87	75.76	84.00	61.15	1.637	1.085
11/13/2017 10:30	0.85	45.05	263.68	220.59	254.65	12.480	3.183
11/13/2017 10:31	0.43	27.78	155.93	154.53	216.83	8.237	2.547
11/13/2017 10:32	0.22	18.98	101.47	82.20	131.79	6.226	2.090
11/13/2017 10:33	0.35	15.39	35.26	41.77	37.58	0.857	1.137
11/13/2017 10:34	0.40	51.18	242.92	179.32	166.33	6.099	2.523
11/13/2017 10:35	0.37	100.28	651.79	213.56	189.55	24.715	6.978
11/13/2017 10:36	0.22	41.24	293.04	130.98	166.18	14.602	5.169
11/13/2017 10:37	0.20	21.02	127.02	117.68	131.33	4.870	2.385
11/13/2017 10:38	0.10	53.39	251.11	153.53	172.73	9.468	3.074
11/13/2017 10:39	0.10	72.52	494.26	171.14	275.07	20.281	6.068
11/13/2017 10:40	0.05	33.97	237.18	149.72	224.92	9.950	4.066
11/13/2017 10:41	0.02	32.49	192.48	237.94	252.50	8.042	2.790
11/13/2017 10:42	0.02	131.54	776.77	632.85	609.65	30.125	7.195
11/13/2017 10:43	0.00	196.84	1178.03	747.96	703.30	45.752	13.164
11/13/2017 10:44	0.00	331.58	2112.50	443.29	362.83	69.638	22.634
11/13/2017 10:45	0.00	190.21	1317.91	319.97	310.27	46.378	18.571
11/13/2017 10:46	0.05	75.95	563.49	196.42	204.77	20.831	9.449
11/13/2017 10:47	0.17	132.13	706.82	279.17	293.93	22.977	7.399
11/13/2017 10:48	0.20	143.02	933.25	602.17	510.00	36.435	12.378
11/13/2017 10:49	0.18	110.95	884.92	254.16	239.77	36.241	12.060
11/13/2017 10:50	0.22	40.48	333.02	188.80	211.15	14.424	6.332
11/13/2017 10:51	0.40	22.66	205.31	163.61	221.65	7.804	3.478
11/13/2017 10:52	0.50	29.87	205.24	228.41	284.53	9.257	3.593
11/13/2017 10:53	0.43	95.68	474.62	366.89	438.32	19.836	5.847
11/13/2017 10:54	0.40	138.93	856.48	214.33	142.18	28.600	10.386
11/13/2017 10:55	0.42	61.43	453.15	135.39	121.85	16.997	7.860

11/13/2017 10:56	0.62	43.79	335.35	104.98	89.92	11.189	5.006
11/13/2017 10:57	0.72	42.15	276.78	149.00	142.21	9.873	4.041
11/13/2017 10:58	0.97	21.76	164.75	126.33	82.39	6.881	3.355
11/13/2017 10:59	1.18	21.08	145.15	76.35	62.94	4.875	2.568
11/13/2017 11:00	1.25	65.87	352.25	133.18	120.59	13.415	4.365
11/13/2017 11:01	1.02	62.74	371.85	141.14	199.90	17.043	6.187
11/13/2017 11:02	1.08	4.73	53.23	43.30	36.12	2.402	2.514
11/13/2017 11:03	1.43	6.28	55.85	37.94	39.91	1.757	1.451
11/13/2017 11:04	1.77	11.09	57.97	41.01	63.91	2.699	1.644
11/13/2017 11:05	1.87	14.33	53.80	87.61	72.93	3.779	1.790
11/13/2017 11:06	1.87	5.13	35.12	35.54	35.06	2.805	1.726
11/13/2017 11:07	2.30	8.19	33.79	29.80	18.83	1.444	1.288
11/13/2017 11:08	2.53	51.99	258.45	108.08	98.05	9.522	2.922
11/13/2017 11:09	2.35	39.13	220.08	98.65	86.19	9.030	3.939
11/13/2017 11:10	1.93	38.22	210.38	123.18	101.78	7.793	3.373
11/13/2017 11:11	1.45	25.67	163.24	102.46	116.18	8.583	3.627
11/13/2017 11:12	1.55	22.53	133.63	73.03	40.62	4.107	2.187
11/13/2017 11:13	1.68	24.51	140.47	71.64	56.39	5.517	2.519
11/13/2017 11:14	2.03	14.58	104.04	45.40	33.69	4.831	2.489
11/13/2017 11:15	2.17	11.99	69.93	37.74	18.21	2.783	1.762
11/13/2017 11:16	2.50	6.78	26.64	36.63	18.21	1.897	1.360
11/13/2017 11:17	2.88	4.52	18.85	26.00	14.53	1.446	1.138
11/13/2017 11:18	3.00	5.50	23.48	40.17	28.39	1.425	1.099
11/13/2017 11:19	2.92	4.27	10.23	30.72	39.84	1.284	1.091
11/13/2017 11:20	2.72	11.81	47.82	73.55	103.97	3.163	1.306
11/13/2017 11:21	2.45	20.88	91.74	42.88	71.40	4.647	2.072
11/13/2017 11:22	2.53	21.23	110.89	46.08	47.71	3.672	1.949
11/13/2017 11:23	2.42	15.20	79.77	42.18	47.06	3.326	1.920
11/13/2017 11:24	2.83	5.01	17.58	24.34	18.72	1.016	1.246
11/13/2017 11:25	3.20	4.95	11.96	20.22	13.21	0.508	0.986
11/13/2017 11:26	3.60	4.61	13.92	20.91	16.44	0.670	1.011
11/13/2017 11:27	3.63	5.38	14.68	21.80	20.28	0.841	1.028
11/13/2017 11:28	3.73	4.79	8.46	17.13	12.20	0.537	0.970
11/13/2017 11:29	3.98	4.97	5.92	18.54	13.16	0.621	0.942
11/13/2017 11:30	4.38	5.28	2.59	14.10	8.43	0.405	0.908
11/13/2017 11:31	4.57	5.70	4.07	19.38	21.67	0.731	0.910
11/13/2017 11:32	4.50	9.88	32.80	41.85	55.11	1.906	1.130
11/13/2017 11:33	4.08	14.37	47.49	52.85	43.02	2.352	1.401
11/13/2017 11:34	3.32	78.98	418.38	114.22	145.35	14.191	4.270
11/13/2017 11:35	2.55	34.79	241.89	68.50	76.00	9.816	4.677
11/13/2017 11:36	2.25	14.60	92.62	55.19	70.44	3.408	2.114
11/13/2017 11:37	2.48	11.16	72.98	52.66	52.13	2.289	1.583
11/13/2017 11:38	2.65	14.13	83.23	55.01	72.19	3.374	1.793
11/13/2017 11:39	2.67	9.12	47.39	54.65	76.36	2.308	1.415
11/13/2017 11:40	2.40	8.54	46.20	42.02	52.04	2.151	1.425
11/13/2017 11:41	2.57	10.54	42.34	49.80	63.76	2.146	1.367
11/13/2017 11:42	2.65	8.04	31.55	56.84	97.60	2.941	1.510

11/13/2017 11:43	2.65	24.18	99.49	129.57	197.90	5.581	1.821
11/13/2017 11:44	2.10	30.88	157.56	183.63	443.73	11.394	3.056
11/13/2017 11:45	1.42	50.28	254.80	255.56	449.90	12.994	3.493
11/13/2017 11:46	0.93	38.55	217.11	219.73	556.95	15.427	4.634
11/13/2017 11:47	0.88	53.58	300.78	294.90	559.47	15.310	4.092
11/13/2017 11:48	0.90	21.68	139.42	166.57	445.47	9.797	3.568
11/13/2017 11:49	0.93	38.05	211.39	244.01	489.20	11.498	3.610
11/13/2017 11:50	1.05	35.48	196.53	279.10	280.17	8.653	3.211
11/13/2017 11:51	1.10	73.11	426.20	283.66	428.20	15.521	4.787
11/13/2017 11:52	0.97	89.35	540.34	333.53	422.17	22.915	7.118
11/13/2017 11:53	0.65	74.33	479.27	396.13	442.37	21.219	6.938
11/13/2017 11:54	0.60	150.79	885.20	239.62	231.55	27.586	8.410
11/13/2017 11:55	0.68	82.70	548.74	151.79	159.42	20.572	8.837
11/13/2017 11:56	0.85	53.53	426.13	116.28	117.22	14.888	5.811
11/13/2017 11:57	1.00	36.43	304.80	96.96	90.64	9.912	4.272
11/13/2017 11:58	1.23	10.73	129.65	59.06	52.66	4.578	2.839
11/13/2017 11:59	1.57	10.22	113.84	72.96	44.88	2.678	1.879
11/13/2017 12:00	1.70	18.36	136.90	67.67	75.23	4.974	2.471
11/13/2017 12:01	1.73	20.01	126.80	119.21	88.01	4.715	2.286
11/13/2017 12:02	1.47	13.56	110.01	101.31	103.87	5.235	2.454
11/13/2017 12:03	1.37	31.71	196.65	108.06	91.47	6.532	2.669
11/13/2017 12:04	1.13	48.73	259.40	110.13	113.77	11.301	4.169
11/13/2017 12:05	1.22	22.88	161.07	110.13	133.42	8.017	3.456
11/13/2017 12:06	1.38	12.03	89.67	86.00	90.41	4.421	2.223
11/13/2017 12:07	1.60	4.43	43.27	51.69	74.70	2.935	1.777
11/13/2017 12:08	1.80	10.32	63.04	70.72	71.40	3.029	1.490
11/13/2017 12:09	1.70	17.53	88.18	112.55	174.10	5.861	2.071
11/13/2017 12:10	1.50	24.46	111.67	126.81	211.17	7.337	2.551
11/13/2017 12:11	1.33	6.94	44.09	75.75	117.58	4.241	2.091
11/13/2017 12:12	1.58	8.15	42.36	70.16	119.67	3.265	1.554
11/13/2017 12:13	1.83	34.94	160.60	115.22	137.51	6.754	2.366
11/13/2017 12:14	1.70	43.78	226.90	147.06	280.71	15.162	4.378
11/13/2017 12:15	1.42	14.99	95.86	84.21	155.18	7.371	2.998
11/13/2017 12:16	1.30	17.04	92.66	121.72	206.40	6.122	2.196
11/13/2017 12:17	1.33	13.36	72.69	97.65	161.18	5.088	2.073
11/13/2017 12:18	1.38	5.30	33.28	77.02	136.90	3.866	1.764
11/13/2017 12:19	1.43	5.10	25.34	73.33	120.09	2.882	1.442
11/13/2017 12:20	1.58	3.08	21.49	69.21	107.88	2.694	1.440
11/13/2017 12:21	1.68	1.30	10.79	55.62	81.42	2.003	2.135
11/13/2017 12:22	1.87	0.69	5.98	39.32	44.32	1.227	2.541
11/13/2017 12:23	2.03	2.87	20.01	44.72	67.90	1.820	2.986
11/13/2017 12:24	2.68	1.32	-3.94	16.60	10.86	0.963	2.823
11/13/2017 12:25	3.48	8.00	16.52	50.66	52.22	0.969	2.160
11/13/2017 12:26	3.33	29.23	140.42	190.55	386.56	9.172	3.150
11/13/2017 12:27	2.47	63.94	288.11	330.98	433.28	13.741	4.181
11/13/2017 12:28	1.07	201.03	1002.37	901.53	879.99	42.999	10.278
11/13/2017 12:29	0.53	185.92	870.67	439.82	681.33	51.259	15.454

11/13/2017 12:30	0.32	60.88	442.23	264.15	538.82	24.817	8.830
11/13/2017 12:31	0.37	21.35	186.23	192.94	413.36	10.120	3.837
11/13/2017 12:32	0.50	32.72	207.20	179.42	381.30	8.859	3.233
11/13/2017 12:33	0.60	148.31	829.65	313.49	437.68	29.654	8.783
11/13/2017 12:34	0.60	51.48	387.29	254.11	494.01	24.297	8.048
11/13/2017 12:35	0.57	50.15	295.36	197.85	208.33	9.518	4.131
11/13/2017 12:36	0.55	36.96	253.40	244.35	506.37	15.240	4.990
11/13/2017 12:37	0.65	20.83	153.98	196.01	370.76	9.140	3.275
11/13/2017 12:38	0.68	13.28	97.59	152.74	298.36	6.432	2.528
11/13/2017 12:39	0.75	7.94	75.68	105.90	186.66	4.596	2.150
11/13/2017 12:40	0.90	13.78	87.20	148.28	224.33	5.489	2.054
11/13/2017 12:41	0.92	6.32	52.47	82.14	131.71	3.788	1.996
11/13/2017 12:42	1.10	9.64	62.27	77.21	47.43	3.005	1.707
11/13/2017 12:43	1.37	8.19	50.45	76.19	45.94	2.760	1.697
11/13/2017 12:44	1.48	23.41	132.41	96.38	78.48	5.661	2.255
11/13/2017 12:45	1.35	68.41	291.52	129.70	174.68	16.377	4.772
11/13/2017 12:46	1.02	35.17	170.23	109.37	150.91	9.661	4.294
11/13/2017 12:47	0.97	23.54	135.97	91.53	90.76	7.602	3.358
11/13/2017 12:48	1.40	16.06	85.49	71.26	30.97	4.229	1.878
11/13/2017 12:49	1.50	11.99	93.15	83.34	82.13	6.204	2.563
11/13/2017 12:50	1.42	30.79	156.51	91.69	62.76	6.112	2.505
11/13/2017 12:51	1.00	33.67	206.83	106.19	76.43	9.512	3.622
11/13/2017 12:52	1.12	19.52	123.52	66.41	33.24	5.112	2.614
11/13/2017 12:53	1.23	15.38	98.53	54.04	30.92	4.487	2.342
11/13/2017 12:54	1.52	5.58	48.39	31.17	17.62	2.574	1.784
11/13/2017 12:55	1.97	1.78	16.84	18.16	6.41	0.691	1.179
11/13/2017 12:56	2.38	5.28	21.09	14.97	5.47	0.491	1.090
11/13/2017 12:57	2.45	22.47	111.55	75.04	44.28	4.695	1.919
11/13/2017 12:58	2.05	10.59	54.32	54.92	43.26	3.224	1.971
11/13/2017 12:59	2.07	4.29	19.82	31.66	10.45	1.165	1.288
11/13/2017 13:00	2.10	7.77	40.15	54.65	22.47	1.878	1.286
11/13/2017 13:01	2.03	17.34	81.27	58.80	37.92	3.813	1.875
11/13/2017 13:02	1.82	9.15	47.77	35.03	18.88	2.561	1.734
11/13/2017 13:03	1.90	9.67	45.14	32.52	13.66	1.956	1.494
11/13/2017 13:04	2.22	8.34	45.84	37.41	15.69	2.181	1.482
11/13/2017 13:05	2.40	3.25	12.93	29.40	10.96	0.799	1.211
11/13/2017 13:06	2.37	7.08	36.82	29.40	15.77	1.272	1.200
11/13/2017 13:07	2.27	7.80	31.26	26.42	21.35	2.196	1.471
11/13/2017 13:08	2.33	6.79	35.05	31.70	21.07	1.793	1.343
11/13/2017 13:09	2.37	6.27	25.95	32.83	26.02	1.481	1.300
11/13/2017 13:10	2.57	4.61	17.30	23.54	16.21	1.069	1.220
11/13/2017 13:11	2.63	7.70	23.43	32.28	26.37	1.412	1.200
11/13/2017 13:12	2.67	5.28	16.56	18.93	17.14	1.034	1.173
11/13/2017 13:13	2.88	5.71	19.35	12.12	4.05	0.580	1.109
11/13/2017 13:14	3.15	5.10	10.93	10.59	3.38	0.352	1.009
11/13/2017 13:15	3.58	6.04	28.11	10.31	3.77	0.382	0.889
11/13/2017 13:16	3.78	6.61	21.59	11.74	5.71	0.425	1.108

11/13/2017 13:17	3.88	7.44	26.44	11.37	3.60	0.460	1.081
11/13/2017 13:18	3.93	6.93	24.03	6.90	3.86	0.282	1.028
11/13/2017 13:19	3.95	6.10	17.54	12.25	8.43	0.494	1.028
11/13/2017 13:20	3.48	5.86	15.34	40.41	48.72	1.905	1.293
11/13/2017 13:21	2.90	5.36	14.11	30.66	35.96	1.463	1.291
11/13/2017 13:22	2.45	11.93	43.56	61.71	77.42	2.679	1.376
11/13/2017 13:23	1.92	19.78	88.10	89.58	157.76	5.729	2.136
11/13/2017 13:24	1.52	33.67	158.27	155.72	320.48	9.769	2.767
11/13/2017 13:25	1.05	53.38	229.08	190.48	287.51	10.080	3.340
11/13/2017 13:26	0.88	48.33	263.08	242.90	480.00	17.680	5.107
11/13/2017 13:27	0.82	37.35	185.11	135.23	222.06	8.613	3.113
11/13/2017 13:28	0.77	41.07	210.60	183.02	239.84	9.436	3.363
11/13/2017 13:29	0.70	53.00	284.57	266.77	700.19	19.983	5.122
11/13/2017 13:30	0.75	13.66	104.03	102.58	159.09	5.340	2.709
11/13/2017 13:31	0.98	39.73	192.25	184.26	236.46	8.473	2.617
11/13/2017 13:32	0.98	35.53	191.27	245.87	547.89	15.247	4.133
11/13/2017 13:33	0.75	36.25	184.20	258.63	615.51	14.737	3.861
11/13/2017 13:34	0.45	34.78	174.70	215.42	545.24	14.122	4.040
11/13/2017 13:35	0.93	14.99	80.86	56.57	45.91	1.440	1.948
11/13/2017 13:36	1.20	79.21	383.08	299.17	479.23	17.828	4.576
11/13/2017 13:37	1.15	59.53	294.60	370.81	942.52	24.700	6.270
11/13/2017 13:38	0.55	78.59	329.89	421.77	992.02	23.262	5.478
11/13/2017 13:39	0.25	41.27	244.19	274.69	793.47	20.132	5.687
11/13/2017 13:40	0.47	58.24	288.66	218.17	230.95	8.445	3.416
11/13/2017 13:41	0.57	51.08	299.17	262.24	559.19	18.118	5.511
11/13/2017 13:42	0.63	75.29	435.46	288.75	657.53	23.913	6.120
11/13/2017 13:43	0.63	27.93	148.40	115.77	196.28	6.230	3.591
11/13/2017 13:44	0.65	72.72	364.14	312.70	545.89	19.263	5.455
11/13/2017 13:45	0.78	23.82	166.49	86.43	296.32	7.560	3.794
11/13/2017 13:46	0.72	167.28	786.14	438.09	695.53	35.742	8.949
11/13/2017 13:47	0.63	24.33	215.20	175.83	485.58	15.639	6.095
11/13/2017 13:48	0.65	124.94	621.47	314.55	403.86	21.044	5.743
11/13/2017 13:49	0.50	88.75	474.99	448.89	1029.86	33.523	9.666
11/13/2017 13:50	0.48	52.04	304.14	382.15	771.54	21.397	5.827
11/13/2017 13:51	0.48	3.23	101.01	114.68	294.79	7.561	3.232
11/13/2017 13:52	1.02	-0.10	53.88	53.15	13.13	0.609	1.331
11/13/2017 13:53	1.53	17.14	90.26	90.22	91.05	4.171	1.578
11/13/2017 13:54	1.57	51.84	271.06	249.31	641.67	22.570	4.986
11/13/2017 13:55	1.47	-1.26	17.02	37.84	35.37	2.926	2.145
11/13/2017 13:56	1.53	3.18	35.45	52.28	43.93	2.586	1.475
11/13/2017 13:57	1.52	22.35	105.56	166.14	301.00	7.771	2.186
11/13/2017 13:58	1.13	34.92	152.42	226.14	432.45	11.610	3.236
11/13/2017 13:59	0.57	27.53	136.65	208.73	465.25	12.670	3.584
11/13/2017 14:00	0.45	25.17	109.99	195.15	352.42	9.200	2.874
11/13/2017 14:01	0.50	24.73	125.69	197.05	399.53	10.568	3.047
11/13/2017 14:02	0.50	52.24	239.41	247.17	492.53	16.181	4.459
11/13/2017 14:03	0.50	14.28	89.61	127.98	226.33	6.637	2.866

11/13/2017 14:04	0.63	9.46	55.98	91.11	156.63	4.346	2.053
11/13/2017 14:05	0.75	54.37	237.42	222.36	389.55	13.248	3.547
11/13/2017 14:06	0.70	29.24	157.70	324.50	514.30	14.692	4.356
11/13/2017 14:07	0.55	17.23	98.29	326.35	309.67	7.715	2.683
11/13/2017 14:08	0.50	6.81	53.90	150.54	193.45	4.926	2.112
11/13/2017 14:09	0.67	17.36	87.30	115.80	199.33	5.655	2.119
11/13/2017 14:10	0.67	69.27	308.05	275.93	555.73	17.236	4.460
11/13/2017 14:11	0.52	70.92	339.88	332.46	673.23	21.417	6.103
11/13/2017 14:12	0.30	92.18	429.15	295.86	670.80	24.035	6.925
11/13/2017 14:13	0.28	123.88	524.56	500.93	945.73	30.935	8.006
11/13/2017 14:14	0.30	133.58	540.48	738.56	1761.67	42.067	10.532
11/13/2017 14:15	0.25	84.29	413.95	528.78	1531.70	36.253	9.414
11/13/2017 14:16	0.20	83.69	364.79	587.52	1061.40	24.482	6.494
11/13/2017 14:17	0.20	74.82	362.58	528.35	1287.67	30.250	7.656
11/13/2017 14:18	0.20	89.38	393.21	628.33	1527.50	32.310	7.812
11/13/2017 14:19	0.23	79.02	341.75	550.63	1374.00	31.157	7.806
11/13/2017 14:20	0.30	94.87	404.39	581.31	1314.50	31.355	8.008
11/13/2017 14:21	0.30	53.48	249.94	377.13	919.27	22.155	6.544
11/13/2017 14:22	0.32	67.81	318.05	437.45	1030.57	24.265	6.193
11/13/2017 14:23	0.30	30.75	179.76	259.86	732.48	18.000	5.313
11/13/2017 14:24	0.37	8.07	78.95	115.12	201.07	15.815	3.749
11/13/2017 14:25	0.40	0.61	46.31	78.18	127.76	7.443	2.465
11/13/2017 14:26	0.55	-5.63	42.25	59.74	17.74	5.916	2.380
11/13/2017 14:27	0.70	-5.27	6.74	29.74	6.28	1.995	2.299
11/13/2017 14:28	0.98	-4.82	5.64	23.80	4.89	0.424	1.276
11/13/2017 14:29	1.17	-3.83	0.24	18.82	4.67	0.683	1.154
11/13/2017 14:30	1.30	-4.59	5.02	15.96	4.15	1.315	1.223
11/13/2017 14:31	1.38	-4.90	-6.31	14.06	3.32	0.613	1.753
11/13/2017 14:32	1.58	-3.76	-4.44	15.52	7.83	0.358	2.219
11/13/2017 14:33	1.67	-2.88	-5.70	14.26	12.45	0.521	2.942
11/13/2017 14:34	1.92	-3.32	-10.90	8.94	3.16	0.055	2.986
11/13/2017 14:35	2.20	-3.73	-15.26	7.81	2.12	0.053	3.542
11/13/2017 14:36	2.53	-2.96	-14.53	7.13	2.17	0.050	4.531
11/13/2017 14:37	2.68	-2.31	-13.64	6.57	2.08	0.031	10.399
11/13/2017 14:38	2.62	-2.88	5.29	7.34	3.17	0.495	4.934
11/13/2017 14:39	2.48	-2.14	-8.42	7.07	3.75	0.190	1.491
11/13/2017 14:40	2.30	-2.97	-6.57	6.58	2.67	0.258	1.126
11/13/2017 14:41	2.47	-1.38	-11.73	5.33	2.10	0.043	0.973
11/13/2017 14:42	2.87	-0.17	-11.54	4.95	1.98	0.037	0.935
11/13/2017 14:43	3.42	0.57	-10.96	4.70	2.00	0.025	0.938
11/13/2017 14:44	3.78	0.34	-10.80	4.53	2.08	0.036	0.971
11/13/2017 14:45	4.03	-0.48	-10.29	4.29	1.98	0.028	0.956
11/13/2017 14:46	4.17	0.02	-10.30	3.94	1.97	0.028	0.942
11/13/2017 14:47	4.23	1.50	-10.18	3.85	1.71	0.037	0.940
11/13/2017 14:48	4.30	1.02	-9.14	3.73	1.87	0.018	0.932
11/13/2017 14:49	4.38	1.12	-9.07	3.64	1.92	0.030	0.899
11/13/2017 14:50	4.47	1.98	-8.51	3.56	1.92	0.086	0.920

11/13/2017 14:51	4.58	1.23	-7.64	3.56	2.18	0.939	0.975
11/13/2017 14:52	4.70	2.64	-7.57	3.43	2.01	0.119	0.940
11/13/2017 14:53	4.83	1.50	-6.63	3.30	1.72	0.034	0.916
11/13/2017 14:54	4.95	1.72	-6.48	3.26	1.70	0.020	0.910
11/13/2017 14:55	5.07	1.36	-5.83	3.17	1.96	0.032	0.937
11/13/2017 14:56	5.18	1.43	-5.11	3.12	2.00	0.022	0.945
11/13/2017 14:57	5.33	2.87	-4.50	3.07	1.84	0.027	0.951
11/13/2017 14:58	5.33	1.33	-5.65	4.81	30.94	1.227	0.987
11/13/2017 14:59	4.97	2.98	-5.39	7.91	37.66	0.302	1.006
11/13/2017 15:00	4.87	3.74	-4.13	3.79	14.59	0.207	0.941
11/13/2017 15:01	4.83	2.03	-3.76	5.28	40.35	0.483	1.022
11/13/2017 15:02	5.35	3.30	-3.01	3.05	1.96	0.014	0.972
11/13/2017 15:03	5.57	3.81	-2.88	3.05	2.35	0.038	0.960
11/13/2017 15:04	5.90	3.10	-2.50	3.18	4.23	0.052	0.949
11/13/2017 15:05	5.77	2.73	-2.70	5.01	18.89	0.074	0.954
11/13/2017 15:06	5.80	3.47	-2.15	3.24	2.19	0.039	0.950
11/13/2017 15:07	5.63	2.59	-2.74	3.90	2.36	0.049	0.991
11/13/2017 15:08	5.62	2.36	-2.73	3.92	2.42	0.057	1.132
11/13/2017 15:09	5.37	2.63	-2.17	3.92	2.22	0.055	1.436
11/13/2017 15:10	5.30	4.60	-1.38	3.26	2.03	0.042	2.016
11/13/2017 15:11	5.40	2.98	0.31	2.95	1.85	0.040	2.509
11/13/2017 15:12	5.67	3.37	-0.96	2.75	1.99	0.031	2.277
11/13/2017 15:13	5.88	5.17	0.36	2.55	2.13	0.045	1.863
11/13/2017 15:14	6.13	5.13	-0.42	2.51	2.29	0.034	1.912
11/13/2017 15:15	6.32	4.97	0.64	2.44	2.01	0.020	2.826
11/13/2017 15:16	6.55	6.15	4.39	2.43	1.94	0.026	4.992
11/13/2017 15:17	6.63	4.96	2.86	2.36	1.92	0.039	5.334
11/13/2017 15:18	6.75	7.03	0.50	2.47	3.51	0.725	5.031
11/13/2017 15:19	6.80	5.00	1.93	2.39	2.08	0.174	3.424
11/13/2017 15:20	6.80	4.96	0.95	2.29	2.07	0.044	4.266
11/13/2017 15:21	6.82	6.38	2.06	2.31	2.10	0.031	8.688
11/13/2017 15:22	6.90	5.83	2.12	2.32	1.93	0.036	8.604
11/13/2017 15:23	6.93	4.60	2.68	2.27	1.83	0.034	4.750
11/13/2017 15:24	7.02	5.30	4.02	2.21	1.86	0.026	2.779
11/13/2017 15:25	7.10	5.20	3.19	2.16	2.24	0.073	2.150
11/13/2017 15:26	7.20	5.18	3.50	2.32	2.47	0.200	1.374
11/13/2017 15:27	6.60	27.89	104.13	154.37	171.94	3.818	1.559
11/13/2017 15:28	4.55	42.81	224.95	291.51	577.12	14.152	3.394
11/13/2017 15:29	2.35	81.45	379.06	585.37	1236.72	23.370	5.352
11/13/2017 15:30	0.73	81.75	317.38	621.58	1277.85	25.653	6.062
11/13/2017 15:31	0.45	194.58	939.94	914.85	1699.72	51.213	11.700
11/13/2017 15:32	0.40	97.71	488.88	583.68	1141.05	39.794	10.112
11/13/2017 15:33	0.35	68.36	348.40	490.53	1013.72	26.158	6.527
11/13/2017 15:34	0.30	37.88	236.96	387.49	847.58	19.067	5.091
11/13/2017 15:35	0.33	31.69	201.26	349.85	694.57	15.261	3.893
11/13/2017 15:36	0.38	56.48	260.86	416.33	788.92	19.698	4.255
11/13/2017 15:37	0.30	114.72	642.56	380.88	690.65	29.725	7.106

11/13/2017 15:38	0.38	52.21	347.94	224.71	364.69	22.014	7.362
11/13/2017 15:39	0.50	19.15	150.68	162.69	308.21	9.469	3.701
11/13/2017 15:40	0.60	26.67	159.68	201.93	351.50	9.589	3.281
11/13/2017 15:41	0.60	19.56	129.88	157.45	320.00	9.282	3.386
11/13/2017 15:42	0.60	17.40	107.40	207.05	316.11	6.289	2.470
11/13/2017 15:43	0.52	43.65	199.47	300.47	477.22	12.081	3.589
11/13/2017 15:44	0.55	51.45	284.22	227.08	315.59	14.117	4.624
11/13/2017 15:45	0.47	31.95	227.26	208.29	326.08	11.576	4.054
11/13/2017 15:46	0.50	59.16	314.75	321.98	400.04	14.272	4.227
11/13/2017 15:47	0.60	30.11	173.30	133.28	206.25	8.708	3.707
11/13/2017 15:48	0.78	35.59	240.90	120.29	138.03	11.364	4.031
11/13/2017 15:49	0.87	11.00	88.45	140.60	212.63	5.513	2.603
11/13/2017 15:50	0.83	84.41	342.97	247.10	343.07	12.350	3.791
11/13/2017 15:51	0.70	86.25	523.58	221.22	294.77	24.558	7.836
11/13/2017 15:52	0.67	105.39	565.16	370.61	558.88	28.113	7.675
11/13/2017 15:53	0.57	55.73	323.53	385.83	617.44	18.134	5.727
11/13/2017 15:54	0.45	75.85	357.53	561.35	879.67	20.955	5.649
11/13/2017 15:55	0.30	131.49	570.18	889.76	1167.92	31.239	7.870
11/13/2017 15:56	0.37	369.38	1607.54	967.09	1481.53	63.011	15.877
11/13/2017 15:57	0.40	169.38	970.17	281.15	346.33	57.295	18.691
11/13/2017 15:58	0.48	22.53	279.62	153.41	199.78	15.662	6.499
11/13/2017 15:59	0.55	35.93	304.62	289.86	379.62	10.825	3.837
11/13/2017 16:00	0.60	75.63	360.16	284.03	344.08	11.990	4.623
11/13/2017 16:01	0.60	276.50	1083.21	383.77	332.88	34.493	11.144
11/13/2017 16:02	0.55	159.85	1085.18	263.98	280.39	56.568	16.222
11/13/2017 16:03	0.50	212.67	912.83	271.42	283.31	41.099	11.118
11/13/2017 16:04	0.50	77.35	528.38	126.32	121.38	29.023	10.457
11/13/2017 16:05	0.58	38.14	297.39	75.68	47.98	11.372	5.877
11/13/2017 16:06	0.72	15.80	169.92	49.25	14.64	3.317	3.643
11/13/2017 16:07	0.93	6.33	108.99	38.77	8.08	1.380	2.631
11/13/2017 16:08	1.22	2.13	67.23	30.62	5.03	0.721	2.091
11/13/2017 16:09	1.48	-0.57	45.24	30.14	4.26	0.541	1.801
11/13/2017 16:10	1.62	1.32	33.58	24.60	4.13	0.454	1.775
11/13/2017 16:11	0.58	42.58	198.34	58.66	25.55	5.901	3.268
11/13/2017 16:12	-7.58	24.75	114.01	49.34	42.86	7.105	4.575
11/13/2017 16:13	-16.98	12.13	60.94	40.10	58.23	4.220	3.003
11/13/2017 16:14	-23.57	13.18	53.39	35.41	36.80	2.278	2.387
11/13/2017 16:15	-23.80	9.98	32.56	33.33	33.65	1.924	2.205
11/13/2017 16:16	-23.80	6.12	25.81	34.74	45.69	1.432	1.971
11/13/2017 16:17	-23.80	6.20	22.40	36.40	46.87	1.707	1.920
11/13/2017 16:18	-23.80	5.69	23.44	32.46	47.77	1.739	1.845
11/13/2017 16:19	-23.80	6.65	18.20	32.01	42.23	1.587	1.789
11/13/2017 16:20	-23.80	5.81	17.50	34.02	48.17	1.588	1.757
11/13/2017 16:21	-23.80	6.00	18.16	32.57	45.35	1.647	1.802
11/13/2017 16:22	-23.80	4.60	9.28	26.55	30.74	1.166	1.675
11/13/2017 16:23	-23.80	6.29	14.65	28.57	35.80	1.393	1.727
11/13/2017 16:24		3.71	2.12	26.22	29.98	1.240	1.588

11/13/2017 16:25	0.40	3.78	4.75	28.00	41.98	1.612	1.586
11/13/2017 16:26	0.50	5.54	8.61	36.51	43.13	1.186	1.540
11/13/2017 16:27	0.50	14.48	53.78	35.90	35.08	2.303	1.814
11/13/2017 16:28	0.50	28.59	114.87	36.91	19.03	6.901	2.843
11/13/2017 16:29	0.50	10.13	38.11	21.76	12.10	3.925	2.322
11/13/2017 16:30	0.50	2.89	12.94	14.97	9.38	1.645	1.593
11/13/2017 16:31	0.50	4.75	11.35	20.24	20.76	2.569	1.546
11/13/2017 16:32	0.58	2.30	7.40	15.53	10.53	1.172	1.480
11/13/2017 16:33	0.60	1.10	-1.42	10.83	6.21	1.009	1.325
11/13/2017 16:34	0.60	8.63	29.10	22.45	15.45	1.465	1.566
11/13/2017 16:35	0.63	4.71	13.64	11.87	10.47	2.325	1.540
11/13/2017 16:36	0.60	3.75	11.27	10.98	8.09	2.230	1.447
11/13/2017 16:37	0.60	1.66	31.93	11.03	9.32	1.196	2.606
11/13/2017 16:38	0.25	10.88	49.58	26.28	32.18	2.311	2.068
11/13/2017 16:39	0.38	11.13	48.36	21.29	30.82	1.768	1.902
11/13/2017 16:40	0.57	8.58	46.18	23.04	28.18	1.593	1.861
11/13/2017 16:41	0.60	9.92	44.02	23.17	27.02	1.296	1.792
11/13/2017 16:42	0.60	7.68	42.57	23.23	30.93	1.063	1.747
11/13/2017 16:43	0.57	3.77	36.07	32.33	23.45	0.441	1.592
11/13/2017 16:44	0.60	5.18	29.96	13.26	21.14	0.195	1.471
11/13/2017 16:45	0.60	4.12	24.37	12.61	12.27	0.125	1.371
11/13/2017 16:46	0.60	5.59	25.66	14.26	11.12	0.142	1.359
11/13/2017 16:47	0.60	3.96	26.69	14.58	14.85	0.129	1.360
11/13/2017 16:48	0.60	4.40	27.17	13.03	12.15	0.101	1.377
11/13/2017 16:49	0.60	4.78	27.10	13.98	13.84	0.092	1.384
11/13/2017 16:50	0.60	4.53	27.09	12.12	10.74	0.086	1.386
11/13/2017 16:51	0.60	4.57	26.63	17.02	16.75	0.063	1.388
11/13/2017 16:52	0.60	5.49	26.33	9.57	9.47	0.127	1.385
11/13/2017 16:53	0.60	3.33	24.83	6.04	4.39	0.094	1.694
11/13/2017 16:54	0.63	4.27	18.34	4.83	3.12	0.051	1.572
11/13/2017 16:55	0.70	2.13	6.39	4.66	3.66	0.024	1.938
11/13/2017 16:56	0.68	2.27	14.49	5.16	4.33	0.018	1.887
11/13/2017 16:57	0.60	1.65	18.49	8.21	9.70	0.045	1.397
11/13/2017 16:58	0.60	2.57	16.99	6.15	6.72	0.031	1.532
11/13/2017 16:59	0.60	2.08	13.53	4.77	3.89	0.011	1.713
11/13/2017 17:00	0.60	2.36	16.17	5.88	6.39	0.037	1.373
11/13/2017 17:01	0.55	2.83	16.08	7.10	8.68	0.041	1.315
11/13/2017 17:02	0.52	3.18	8.25	9.06	40.09	0.168	1.609
11/13/2017 17:03	0.60	0.98	0.58	5.07	7.14	-0.002	2.245
11/13/2017 17:04	0.60	1.07	2.79	13.02	6.91	-0.010	3.027
11/13/2017 17:05	0.60	2.03	1.24	4.24	8.46	-0.007	2.451
11/13/2017 17:06	0.67	1.55	0.39	4.03	4.12	-0.020	1.810
11/13/2017 17:07	0.70	1.28	3.62	5.98	9.58	0.018	2.354
11/13/2017 17:08	1.07	4.18	2.86	3.59	2.25	-0.003	2.977
11/13/2017 17:09	1.83	2.30	2.67	3.35	2.17	-0.001	4.174
11/13/2017 17:10	2.68	1.39	3.98	3.32	2.68	0.014	6.314
11/13/2017 17:11	3.15	2.14	3.12	3.26	2.49	-0.009	5.368

11/13/2017 17:12	3.10	1.71	4.38	2.79	2.36	0.008	1.674
11/13/2017 17:13	2.87	2.63	6.79	2.81	2.50	-0.002	1.546
11/13/2017 17:14	2.67	2.88	5.68	2.72	2.52	-0.003	2.632
11/13/2017 17:15	2.60	3.55	6.07	2.64	2.24	-0.005	7.387
11/13/2017 17:16	2.62	1.65	7.21	10.52	12.59	-0.011	4.497
11/13/2017 17:17	2.73	2.99	4.17	2.89	2.80	-0.003	1.603
11/13/2017 17:18	3.07	1.22	2.67	3.24	2.43	-0.006	4.068
11/13/2017 17:19	3.57	3.88	4.52	2.89	1.87	-0.019	5.280
11/13/2017 17:20	3.77	1.22	6.34	3.12	3.14	-0.002	1.253
11/13/2017 17:21	3.28	1.52	4.65	5.02	5.87	0.006	2.718
11/13/2017 17:22	3.07	2.53	2.20	3.04	2.76	0.021	5.797
11/13/2017 17:23	2.85	1.53	4.98	3.18	3.05	-0.002	1.923
11/13/2017 17:24	2.70	2.39	8.25	2.70	4.10	0.002	1.717
11/13/2017 17:25	2.18	1.23	6.10	12.11	5.03	0.122	1.207
11/13/2017 17:26	1.65	0.26	7.32	32.27	23.79	0.095	1.441
11/13/2017 17:27	1.35	-0.64	7.79	81.77	57.80	0.006	2.330
11/13/2017 17:28	1.13	1.88	9.92	12.75	22.89	0.016	2.070
11/13/2017 17:29	1.23	2.45	9.77	3.53	3.20	0.009	1.567
11/13/2017 17:30	1.48	1.80	8.25	43.92	31.10	0.010	1.195
11/13/2017 17:31	1.65	2.53	9.74	8.10	13.94	0.007	1.162
11/13/2017 17:32	1.63	1.28	9.68	14.77	9.64	0.386	1.134
11/13/2017 17:33	1.67	0.93	9.71	3.04	5.45	0.049	1.138
11/13/2017 17:34	1.75	0.99	11.12	5.23	4.82	0.008	1.143
11/13/2017 17:35	1.58	1.10	8.91	3.68	5.76	0.054	1.160
11/13/2017 17:36	1.35	-0.44	-0.10	2.10	2.84	0.008	1.130
11/13/2017 17:37	1.10	0.06	-1.33	1.77	3.13	-0.008	1.086
11/13/2017 17:38	1.02	0.31	-1.65	2.50	4.01	-0.009	1.028
11/13/2017 17:39	1.02	1.19	-2.98	2.98	4.25	0.226	1.018
11/13/2017 17:40	1.13	-0.19	0.20	4.60	5.05	0.126	1.014
11/13/2017 17:41	1.27	1.91	2.04	3.22	6.23	0.019	0.982
11/13/2017 17:42	1.25	0.64	2.62	1.25	2.63	0.009	0.939
11/13/2017 17:43	1.80	2.06	3.12	4.22	3.54	-0.019	0.884
11/13/2017 17:44	2.22	0.93	-0.37	2.69	3.65	-0.032	0.895
11/13/2017 17:45	2.22	1.17	1.20	1.59	2.97	-0.023	0.902
11/13/2017 17:46	1.72	-0.41	1.45	1.04	3.00	-0.027	0.897
11/13/2017 17:47	1.18	0.39	1.80	3.25	4.89	-0.017	0.925
11/13/2017 17:48	1.13	0.83	3.89	2.61	4.15	0.346	0.945
11/13/2017 17:49	1.17	1.67	4.04	1.61	3.82	0.040	0.941
11/13/2017 17:50	1.20	1.59	4.36	2.75	4.89	0.045	0.960
11/13/2017 17:51	1.27	0.92	5.53	2.47	5.03	0.042	0.996
11/13/2017 17:52	1.33	1.49	6.28	2.07	3.85	0.022	0.997
11/13/2017 17:53	1.38	0.83	6.95	8.29	9.15	0.026	0.991
11/13/2017 17:54	1.55	2.43	7.22	5.52	6.74	0.018	0.994
11/13/2017 17:55	1.90	1.82	7.09	3.01	3.38	0.003	0.988
11/13/2017 17:56	2.18	-2.05	7.14	1.44	2.24	0.086	0.952
11/13/2017 17:57	2.22	-1.04	7.25	3.96	4.73	0.128	0.940
11/13/2017 17:58	2.18	0.51	7.51	2.44	3.52	0.090	0.910

11/13/2017 17:59	2.27	0.47	7.36	1.45	2.42	-0.003	0.864
11/13/2017 18:00	2.43	-1.18	6.88	2.03	3.41	0.011	0.846
11/13/2017 18:01	2.22	-1.05	6.59	4.01	5.67	0.012	0.813
11/13/2017 18:02	1.83	-1.96	7.50	6.99	7.97	0.032	0.785
11/13/2017 18:03	1.60	-0.68	7.36	6.29	5.89	0.018	0.767
11/13/2017 18:04	1.77	2.68	6.99	5.58	4.85	-0.003	0.779
11/13/2017 18:05	1.75	0.81	3.07	2.49	4.22	-0.003	0.725
11/13/2017 18:06	1.35	-0.28	2.95	2.68	4.78	0.004	0.701
11/13/2017 18:07	1.02	-1.35	6.32	26.17	10.26	0.025	0.683
11/13/2017 18:08	0.82	-1.36	4.69	53.38	135.31	0.152	0.668
11/13/2017 18:09	0.73	-1.38	2.37	25.43	58.81	0.054	0.665
11/13/2017 18:10	0.35	-1.44	2.17	21.67	51.85	0.041	0.661
11/13/2017 18:11	0.18	-2.06	2.12	20.36	46.18	0.053	0.715
11/13/2017 18:12	0.25	-0.57	1.55	17.37	27.62	0.063	0.702
11/13/2017 18:13	0.58	-0.88	2.59	9.29	30.12	0.046	0.674
11/13/2017 18:14	0.53	-1.29	3.49	8.31	37.95	0.048	0.682
11/13/2017 18:15	0.40	-0.69	3.85	7.15	34.34	0.054	0.692
11/13/2017 18:16	0.53	0.73	3.97	6.06	10.23	0.039	0.699
11/13/2017 18:17	1.17	-0.68	3.82	5.08	8.66	0.036	0.712
11/13/2017 18:18	1.28	-0.93	5.10	4.91	24.22	0.050	0.803
11/13/2017 18:19	0.85	-1.28	5.70	4.48	21.57	0.031	0.770
11/13/2017 18:20	0.20	-2.13	5.85	4.16	19.62	0.035	0.802
11/13/2017 18:21	0.18	-1.01	6.42	3.96	15.51	0.043	0.791
11/13/2017 18:22	0.20	-2.44	6.58	3.96	15.75	0.031	0.795
11/13/2017 18:23	0.27	-2.53	7.46	3.83	15.53	0.046	0.774
11/13/2017 18:24	0.22	0.22	7.82	3.58	16.08	0.042	0.805
11/13/2017 18:25	0.23	-0.74	8.15	3.33	15.46	0.033	0.829
11/13/2017 18:26	0.20	-0.95	8.93	3.06	14.84	0.048	0.867
11/13/2017 18:27	0.23	-2.20	9.46	2.92	14.54	0.034	0.842
11/13/2017 18:28	0.30	0.90	9.63	2.79	12.36	0.046	0.829
11/13/2017 18:29	0.30	-0.22	9.51	2.80	12.92	0.065	0.812
11/13/2017 18:30	0.32	0.38	9.88	2.71	12.54	0.053	0.862
11/13/2017 18:31	0.30	-0.72	9.94	2.61	11.88	0.057	0.841
11/13/2017 18:32	0.30	-0.24	10.28	2.55	12.07	0.067	0.826
11/13/2017 18:33	0.32	-1.98	10.96	2.47	12.20	0.053	0.848
11/13/2017 18:34	0.33	-1.62	11.10	2.37	11.46	0.066	0.847
11/13/2017 18:35	0.40	-0.31	11.44	2.37	10.68	0.069	0.824
11/13/2017 18:36	0.40	-1.48	11.40	2.38	11.87	0.054	0.880
11/13/2017 18:37	0.40	-0.07	11.85	2.36	12.02	0.062	0.903
11/13/2017 18:38	0.33	-1.08	11.94	2.38	11.74	0.047	0.965
11/13/2017 18:39	0.35	-0.07	12.21	2.41	10.09	0.056	0.941
11/13/2017 18:40	0.42	-0.05	12.19	2.43	10.77	0.052	0.919
11/13/2017 18:41	0.40	-1.08	11.96	2.47	10.75	0.040	0.938
11/13/2017 18:42	0.40	0.08	12.51	2.44	10.27	0.053	1.001
11/13/2017 18:43	0.40	-0.35	12.59	2.44	10.56	0.044	1.013
11/13/2017 18:44	0.40	-2.58	12.33	2.44	10.23	0.037	0.997
11/13/2017 18:45	0.40	0.11	12.35	2.45	9.22	0.045	0.942

11/13/2017 18:46	0.40	-0.63	12.95	2.51	9.61	0.050	0.978
11/13/2017 18:47	0.47	-0.93	12.96	2.54	8.42	0.032	0.958
11/13/2017 18:48	0.75	0.43	12.83	2.60	5.17	0.040	0.875
11/13/2017 18:49	0.98	-1.03	12.84	2.63	6.48	0.037	0.895
11/13/2017 18:50	0.98	0.43	12.82	2.72	8.66	0.031	0.968
11/13/2017 18:51	0.72	-1.26	12.80	2.77	8.64	0.039	0.982
11/13/2017 18:52	0.47	-0.23	12.97	2.78	8.70	0.026	0.985
11/13/2017 18:53	0.40	-1.10	13.10	2.78	8.36	0.028	1.007
11/13/2017 18:54	0.47	0.57	12.81	2.81	6.88	0.034	0.952
11/13/2017 18:55	0.50	-0.33	12.81	2.83	7.98	0.024	0.933
11/13/2017 18:56	0.80	2.28	12.72	2.82	4.63	0.035	0.922
11/13/2017 18:57	1.35	0.90	13.28	2.82	3.13	0.031	0.896
11/13/2017 18:58	1.60	0.13	13.29	2.87	5.28	0.022	0.914
11/13/2017 18:59	1.33	-0.06	13.06	2.95	7.57	0.037	1.006
11/13/2017 19:00	0.73	-0.20	13.73	2.88	7.29	0.022	1.002
11/13/2017 19:01	0.40	-0.78	13.70	2.83	7.84	0.030	1.031
11/13/2017 19:02	0.40	-0.40	13.46	2.86	7.69	0.031	1.078
11/13/2017 19:03	0.40	-0.68	13.19	2.95	7.55	0.020	1.129
11/13/2017 19:04	0.40	-0.56	13.29	3.03	7.23	0.022	1.133
11/13/2017 19:05	0.40	-0.05	13.01	3.02	6.91	0.020	1.107
11/13/2017 19:06	0.40	-0.23	12.75	2.97	6.42	0.020	1.032
11/13/2017 19:07	0.40	-1.23	12.98	2.96	6.99	0.030	0.973
11/13/2017 19:08	0.43	-0.61	13.35	2.98	6.80	0.012	0.956
11/13/2017 19:09	0.50	-0.80	13.79	2.99	7.34	0.026	0.984
11/13/2017 19:10	0.47	0.83	14.18	2.91	7.81	0.020	1.062
11/13/2017 19:11	0.47	-0.56	13.94	2.89	7.58	0.023	1.085
11/13/2017 19:12	0.58	1.55	13.99	2.89	5.72	0.031	1.048
11/13/2017 19:13	0.67	-0.58	13.73	2.88	7.43	0.016	1.053
11/13/2017 19:14	0.70	-0.84	14.06	2.63	7.03	0.021	1.026
11/13/2017 19:15	0.48	-1.50	14.31	2.51	7.14	0.028	1.071
11/13/2017 19:16	0.43	0.31	13.96	2.52	7.54	0.021	1.031
11/13/2017 19:17	0.40	-0.12	14.28	2.50	7.68	0.030	1.030
11/13/2017 19:18	0.40	-0.55	13.82	2.51	7.52	0.027	1.117
11/13/2017 19:19	0.42	0.45	13.48	2.59	6.38	0.014	1.132
11/13/2017 19:20	0.43	-0.23	13.59	2.63	6.88	0.026	1.069
11/13/2017 19:21	0.45	1.19	13.56	2.63	6.14	0.017	1.011
11/13/2017 19:22	0.58	1.28	13.55	2.55	5.64	0.026	0.995
11/13/2017 19:23	0.65	0.05	13.86	2.50	6.51	0.033	1.002
11/13/2017 19:24	0.67	1.94	13.92	2.49	6.44	0.012	1.043
11/13/2017 19:25	0.83	0.38	13.38	2.48	4.67	0.028	0.992
11/13/2017 19:26	0.90	-0.58	13.67	2.49	7.18	0.024	1.015
11/13/2017 19:27	0.83	-1.61	13.45	2.50	7.13	0.023	1.090
11/13/2017 19:28	0.48	0.41	13.38	2.53	7.03	0.026	1.132
11/13/2017 19:29	0.40	1.17	13.24	2.59	6.55	0.011	1.193
11/13/2017 19:30	0.33	0.83	12.84	2.64	6.39	0.018	1.217
11/13/2017 19:31	0.45	0.22	11.45	2.59	5.34	0.019	1.192
11/13/2017 19:32	0.92	0.88	9.81	2.29	3.10	0.016	1.047

11/13/2017 19:33	1.28	1.03	11.55	2.43	3.76	0.025	1.003
11/13/2017 19:34	1.78	1.04	10.53	2.28	3.05	0.018	0.979
11/13/2017 19:35	1.82	0.83	12.01	2.31	3.71	0.025	0.979
11/13/2017 19:36	1.78	1.34	11.53	2.38	3.33	0.022	0.965
11/13/2017 19:37	1.75	0.73	10.48	2.32	2.45	0.015	0.948
11/13/2017 19:38	1.62	-0.13	11.55	2.36	4.20	0.025	0.985
11/13/2017 19:39	1.35	-1.09	12.26	2.41	6.10	0.016	1.021
11/13/2017 19:40	0.90	0.35	12.71	2.31	5.46	0.022	0.997
11/13/2017 19:41	0.52	-0.44	12.88	2.25	6.58	0.032	1.040
11/13/2017 19:42	0.57	-0.88	12.70	2.20	5.64	0.019	1.066
11/13/2017 19:43	0.60	-1.08	12.79	2.16	4.58	0.029	1.020
11/13/2017 19:44	0.83	-0.58	13.46	2.15	5.45	0.018	1.025
11/13/2017 19:45	0.83	-2.54	13.63	2.16	6.92	0.019	1.019
11/13/2017 19:46	0.67	0.03	13.94	2.11	8.17	0.030	1.084
11/13/2017 19:47	0.47	-2.38	13.71	2.08	8.36	0.016	1.112
11/13/2017 19:48	0.40	0.35	13.76	2.03	7.22	0.031	1.009
11/13/2017 19:49	0.43	-1.36	13.57	2.06	7.31	0.028	0.982
11/13/2017 19:50	0.60	-1.12	13.52	2.15	6.81	0.026	0.971
11/13/2017 19:51	0.60	-2.53	13.57	2.27	7.21	0.028	0.992
11/13/2017 19:52	0.63	-0.92	13.65	2.28	6.07	0.023	0.966
11/13/2017 19:53	0.60	-0.93	13.56	2.26	5.22	0.030	0.963
11/13/2017 19:54	0.62	-2.13	13.69	2.27	7.32	0.030	1.006
11/13/2017 19:55	0.60	-0.98	13.59	2.25	7.53	0.022	0.993
11/13/2017 19:56	0.55	0.03	13.77	2.20	6.46	0.030	0.979
11/13/2017 19:57	0.60	0.04	13.71	2.16	6.54	0.024	0.980
11/13/2017 19:58	0.57	-1.08	13.94	2.14	8.70	0.035	1.059
11/13/2017 19:59	0.50	-2.03	14.15	2.10	7.94	0.019	1.014
11/13/2017 20:00	0.40	-1.36	14.38	2.12	8.51	0.025	1.005
11/13/2017 20:01	0.40	-2.17	13.96	2.15	6.39	0.033	0.959
11/13/2017 20:02	0.40	-1.78	13.84	2.20	8.56	0.021	1.014
11/13/2017 20:03	0.40	-1.19	14.20	2.24	9.34	0.032	1.051
11/13/2017 20:04	0.35	-1.49	14.51	2.26	9.33	0.030	1.045
11/13/2017 20:05	0.30	-1.95	14.55	2.29	9.95	0.033	1.022
11/13/2017 20:06	0.30	-0.73	14.38	2.32	10.11	0.040	1.017
11/13/2017 20:07	0.30	-2.28	14.34	2.39	10.02	0.024	1.030
11/13/2017 20:08	0.33	-1.88	14.34	2.48	10.11	0.030	1.053
11/13/2017 20:09	0.40	-1.66	14.42	2.54	10.35	0.028	1.046
11/13/2017 20:10	0.40	-2.51	14.51	2.59	10.08	0.034	1.044
11/13/2017 20:11	0.37	-1.53	14.26	2.60	9.90	0.041	1.063
11/13/2017 20:12	0.37	-1.73	14.34	2.63	10.29	0.024	1.041
11/13/2017 20:13	0.40	-1.10	14.27	2.65	10.25	0.039	1.068
11/13/2017 20:14	0.35	-1.97	13.94	2.65	9.87	0.034	1.058
11/13/2017 20:15	0.30	-1.79	14.11	2.47	9.93	0.021	1.050
11/13/2017 20:16	0.30	-1.96	14.35	2.33	9.60	0.033	1.049
11/13/2017 20:17	0.30	-2.42	14.22	2.32	9.69	0.016	1.042
11/13/2017 20:18	0.35	-1.91	14.35	2.25	9.69	0.034	1.031
11/13/2017 20:19	0.37	-1.86	14.49	2.17	10.27	0.027	1.068

11/13/2017 20:20	0.33	-1.85	14.81	2.11	10.16	0.024	1.055
11/13/2017 20:21	0.30	-1.19	14.76	2.10	10.21	0.034	1.072
11/13/2017 20:22	0.30	-1.13	14.68	2.08	10.21	0.016	1.097
11/13/2017 20:23	0.30	-2.19	14.83	2.01	10.82	0.030	1.101
11/13/2017 20:24	0.30	0.43	14.99	1.95	10.82	0.021	1.147
11/13/2017 20:25	0.30	-0.91	15.79	1.90	11.35	0.029	1.189
11/13/2017 20:26	0.30	-1.27	15.90	1.86	11.68	0.038	1.203
11/13/2017 20:27	0.30	0.24	15.63	1.81	11.88	0.020	1.197
11/13/2017 20:28	0.30	0.08	16.14	1.89	12.14	0.029	1.185
11/13/2017 20:29	0.30	-0.08	16.22	3.58	16.13	0.019	1.201
11/13/2017 20:30	0.30	-1.93	16.30	9.65	33.95	0.028	1.216
11/13/2017 20:31	0.30	-0.47	16.24	9.89	33.88	0.035	1.200
11/13/2017 20:32	0.30	-0.65	16.75	19.43	42.97	0.026	1.185
11/13/2017 20:33	0.30	0.46	16.82	34.49	90.32	0.035	1.217
11/13/2017 20:34	0.30	-0.01	16.91	27.83	60.44	0.019	1.232
11/13/2017 20:35	0.30	-0.17	17.14	59.34	108.23	0.043	1.220
11/13/2017 20:36	0.32	-0.24	17.21	25.29	82.75	0.031	1.196
11/13/2017 20:37	0.40	0.10	17.19	32.07	73.85	0.029	1.219
11/13/2017 20:38	0.38	0.86	17.25	36.34	78.95	0.034	1.212
11/13/2017 20:39	0.30	-0.78	17.20	55.50	90.10	0.020	1.213
11/13/2017 20:40	0.30	0.81	17.61	54.29	140.76	0.034	1.236
11/13/2017 20:41	0.30	-0.88	17.31	79.22	123.13	0.028	1.254
11/13/2017 20:42	0.30	-1.51	17.67	78.45	189.32	0.033	1.274
11/13/2017 20:43	0.35	0.04	17.74	78.00	166.46	0.033	1.252
11/13/2017 20:44	0.38	0.51	17.88	31.89	106.46	0.018	1.259
11/13/2017 20:45	0.32	-0.23	17.83	67.68	103.36	0.035	1.285
11/13/2017 20:46	0.30	-0.15	17.66	64.57	154.22	0.025	1.286
11/13/2017 20:47	0.30	-0.48	17.86	69.00	125.09	0.027	1.285
11/13/2017 20:48	0.30	-0.25	17.89	56.91	129.03	0.029	1.282
11/13/2017 20:49	0.30	0.50	17.95	51.51	107.91	0.028	1.284
11/13/2017 20:50	0.30	0.04	17.96	35.36	89.74	0.039	1.280
11/13/2017 20:51	0.30	-0.15	18.32	44.10	82.63	0.024	1.300
11/13/2017 20:52	0.30	0.20	18.21	67.04	117.15	0.034	1.299
11/13/2017 20:53	0.30	1.18	18.40	114.73	184.32	0.028	1.310
11/13/2017 20:54	0.30	-0.29	18.33	76.17	181.60	0.030	1.319
11/13/2017 20:55	0.27	0.89	18.44	77.92	150.82	0.032	1.317
11/13/2017 20:56	0.20	0.28	18.11	42.11	104.12	0.024	1.316
11/13/2017 20:57	0.20	-0.08	18.64	38.70	91.32	0.029	1.321
11/13/2017 20:58	0.20	1.00	18.30	40.96	79.02	0.018	1.313
11/13/2017 20:59	0.20	0.46	18.55	60.98	136.18	0.028	1.344
11/13/2017 21:00	0.20	0.55	18.32	41.62	89.63	0.024	1.356
11/13/2017 21:01	0.20	1.13	18.84	69.36	144.78	0.018	1.348
11/13/2017 21:02	0.20	0.43	18.79	39.61	89.38	0.031	1.347
11/13/2017 21:03	0.20	1.29	18.85	68.83	122.52	0.020	1.383
11/13/2017 21:04	0.20	1.23	18.92	72.95	158.50	0.030	1.382
11/13/2017 21:05	0.20	1.83	18.88	88.92	146.20	0.023	1.379
11/13/2017 21:06	0.28	0.53	19.30	95.16	203.47	0.034	1.375

11/13/2017 21:07	0.30	0.72	18.79	37.82	96.95	0.024	1.364
11/13/2017 21:08	0.30	0.49	19.15	72.50	124.17	0.033	1.362
11/13/2017 21:09	0.30	0.49	18.95	103.29	166.55	0.023	1.348
11/13/2017 21:10	0.30	0.93	19.28	103.29	232.22	0.032	1.357
11/13/2017 21:11	0.30	0.71	19.25	62.52	124.75	0.034	1.379
11/13/2017 21:12	0.30	0.90	19.03	51.68	116.91	0.022	1.393
11/13/2017 21:13	0.33	1.18	18.99	52.88	114.37	0.034	1.387
11/13/2017 21:14	0.38	1.96	19.08	51.59	91.33	0.020	1.396
11/13/2017 21:15	0.37	1.83	19.44	51.59	96.83	0.034	1.446
11/13/2017 21:16	0.30	1.42	19.15	35.53	68.53	0.018	1.419
11/13/2017 21:17	0.30	1.20	19.38	74.28	129.83	0.032	1.436
11/13/2017 21:18	0.30	0.83	19.52	84.89	191.50	0.024	1.441
11/13/2017 21:19	0.30	0.00	19.75	117.89	168.17	0.025	1.424
11/13/2017 21:20	0.30	1.36	19.78	130.10	256.25	0.028	1.411
11/13/2017 21:21	0.30	0.86	20.13	137.27	226.77	0.021	1.386
11/13/2017 21:22	0.30	0.33	19.91	155.76	277.02	0.031	1.416
11/13/2017 21:23	0.30	0.09	19.84	118.96	236.57	0.017	1.407
11/13/2017 21:24	0.30	1.62	19.78	55.58	145.50	0.034	1.421
11/13/2017 21:25	0.25	1.27	19.12	9.86	44.15	0.014	1.427
11/13/2017 21:26	0.20	0.73	19.40	8.08	28.70	0.033	1.453
11/13/2017 21:27	0.20	1.91	18.92	5.86	24.19	0.015	1.462
11/13/2017 21:28	0.20	2.06	18.91	2.55	17.27	0.017	1.444
11/13/2017 21:29	0.20	0.40	19.12	2.14	16.65	0.014	1.422
11/13/2017 21:30	0.20	0.05	18.87	1.79	15.55	0.016	1.402
11/13/2017 21:31	0.23	-0.08	19.02	1.80	15.63	0.015	1.449
11/13/2017 21:32	0.30	1.81	19.37	1.83	15.63	0.016	1.481
11/13/2017 21:33	0.30	1.47	19.21	1.88	15.73	0.020	1.462
11/13/2017 21:34	0.20	0.10	19.64	1.90	16.01	0.010	1.452
11/13/2017 21:35	0.20	0.49	20.28	1.96	16.75	0.020	1.465
11/13/2017 21:36	0.20	1.73	20.17	4.54	24.70	0.005	1.486
11/13/2017 21:37	0.25	0.28	18.80	2.57	33.23	0.020	1.468
11/13/2017 21:38	0.25	1.50	18.85	1.83	14.88	0.012	1.445
11/13/2017 21:39	0.22	1.33	18.89	1.81	14.77	0.022	1.444
11/13/2017 21:40	0.20	0.06	19.11	1.82	14.74	0.014	1.448
11/13/2017 21:41	0.22	-0.86	19.65	1.81	15.29	0.012	1.465
11/13/2017 21:42	0.28	0.76	19.25	1.80	15.27	0.018	1.472
11/13/2017 21:43	0.30	0.82	19.09	1.79	14.76	0.012	1.458
11/13/2017 21:44	0.30	-0.79	20.55	1.79	15.59	0.019	1.470
11/13/2017 21:45	0.30	1.39	19.88	1.84	16.40	0.013	1.467
11/13/2017 21:46	0.27	0.94	19.94	1.82	16.19	0.019	1.453
11/13/2017 21:47	0.20	1.80	20.66	8.43	39.39	0.009	1.443
11/13/2017 21:48	0.22	1.81	20.69	147.60	194.53	0.027	1.458
11/13/2017 21:49	0.27	0.08	20.23	141.91	305.05	0.003	1.480
11/13/2017 21:50	0.30	1.64	20.73	177.22	266.58	0.018	1.473
11/13/2017 21:51	0.30	0.78	21.00	173.83	319.90	0.008	1.475
11/13/2017 21:52	0.30	1.66	20.52	132.47	263.05	0.017	1.466
11/13/2017 21:53	0.30	0.93	21.10	112.35	198.98	0.004	1.457

11/13/2017 21:54	0.30	1.25	20.65	108.90	191.85	0.013	1.494
11/13/2017 21:55	0.30	1.29	21.08	99.24	182.43	0.006	1.507
11/13/2017 21:56	0.23	2.66	21.26	170.44	233.40	0.006	1.529
11/13/2017 21:57	0.28	-0.31	20.08	70.29	143.27	0.012	1.507
11/13/2017 21:58	0.27	0.88	20.09	35.94	79.24	0.001	1.491
11/13/2017 21:59	0.30	0.43	20.25	50.29	79.04	0.009	1.507
11/13/2017 22:00	0.30	1.40	20.70	83.55	148.07	-0.002	1.492
11/13/2017 22:01	0.30	-0.06	21.32	83.76	166.65	0.007	1.463
11/13/2017 22:02	0.30	0.60	20.27	52.61	81.91	-0.002	1.473
11/13/2017 22:03	0.30	0.97	20.60	97.31	130.52	0.009	1.487
11/13/2017 22:04	0.30	0.51	20.57	100.07	173.47	0.004	1.489
11/13/2017 22:05	0.28	-0.03	20.54	60.78	115.67	0.012	1.488
11/13/2017 22:06	0.22	1.53	20.13	38.44	101.80	-0.003	1.484
11/13/2017 22:07	0.20	1.85	20.79	45.27	80.30	0.013	1.510
11/13/2017 22:08	0.20	0.04	19.82	21.83	66.89	-0.001	1.501
11/13/2017 22:09	0.27	1.14	20.21	31.34	55.50	0.005	1.490
11/13/2017 22:10	0.30	-0.91	20.24	34.11	85.88	-0.006	1.498
11/13/2017 22:11	0.30	-0.59	19.95	9.49	40.97	0.011	1.495
11/13/2017 22:12	0.30	0.81	20.23	25.78	54.09	-0.009	1.529
11/13/2017 22:13	0.30	0.38	19.37	7.31	41.55	0.013	1.475
11/13/2017 22:14	0.30	-0.58	19.36	2.14	15.43	-0.015	1.470
11/13/2017 22:15	0.32	-0.50	19.76	2.12	15.41	0.010	1.487
11/13/2017 22:16	0.33	-0.23	19.66	2.12	15.13	-0.003	1.482
11/13/2017 22:17	0.30	-0.18	19.63	1.98	15.45	-0.001	1.520
11/13/2017 22:18	0.30	0.71	19.66	1.79	16.04	-0.011	1.547
11/13/2017 22:19	0.30	-0.16	19.23	1.82	15.75	0.002	1.526
11/13/2017 22:20	0.30	0.43	19.13	1.66	15.19	-0.011	1.486
11/13/2017 22:21	0.30	0.36	19.63	1.62	14.79	-0.001	1.469
11/13/2017 22:22	0.30	-0.55	19.75	1.63	14.61	-0.011	1.462
11/13/2017 22:23	0.25	-1.54	19.51	1.63	14.56	0.006	1.447
11/13/2017 22:24	0.20	0.09	19.39	1.63	14.63	-0.012	1.498
11/13/2017 22:25	0.20	-0.24	19.92	1.65	14.90	0.003	1.500
11/13/2017 22:26	0.20	0.28	20.07	1.64	14.73	-0.013	1.512
11/13/2017 22:27	0.20	0.90	19.24	1.68	14.80	-0.003	1.531
11/13/2017 22:28	0.20	-1.33	19.45	1.69	14.57	-0.012	1.517
11/13/2017 22:29	0.20	-0.33	20.10	1.71	14.45	-0.003	1.510
11/13/2017 22:30	0.20	0.53	19.31	1.70	14.66	-0.017	1.491
11/13/2017 22:31	0.20	0.49	19.65	1.73	14.24	0.002	1.487
11/13/2017 22:32	0.20	0.30	19.67	1.72	14.32	-0.007	1.470
11/13/2017 22:33	0.20	-0.95	19.18	1.73	14.63	-0.003	1.415
11/13/2017 22:34	0.20	0.41	19.64	1.79	14.47	-0.007	1.493
11/13/2017 22:35	0.20	0.47	19.22	1.83	14.53	-0.003	1.506
11/13/2017 22:36	0.20	-0.09	19.42	1.80	14.27	0.001	1.470
11/13/2017 22:37	0.20	0.32	20.25	1.80	14.49	-0.007	1.489
11/13/2017 22:38	0.20	0.18	19.58	1.84	14.62	-0.008	1.506
11/13/2017 22:39	0.20	0.05	19.29	1.83	14.60	-0.012	1.500
11/13/2017 22:40	0.20	0.82	19.86	1.84	14.80	-0.003	1.526

11/13/2017 22:41	0.28	1.16	19.94	1.85	14.63	-0.014	1.536
11/13/2017 22:42	0.30	1.38	19.71	1.85	14.93	-0.006	1.539
11/13/2017 22:43	0.30	0.05	20.21	1.83	15.34	-0.011	1.525
11/13/2017 22:44	0.30	0.28	19.92	1.83	16.05	-0.001	1.535
11/13/2017 22:45	0.30	0.58	19.79	1.80	15.26	-0.012	1.529
11/13/2017 22:46	0.30	0.53	21.04	7.89	23.19	0.010	1.543
11/13/2017 22:47	0.30	-0.24	19.74	8.60	38.42	-0.007	1.555
11/13/2017 22:48	0.37	-0.26	19.51	5.06	22.40	-0.001	1.549
11/13/2017 22:49	0.37	-0.60	19.49	2.62	16.46	-0.017	1.541
11/13/2017 22:50	0.37	-0.04	19.52	2.10	15.32	0.003	1.537
11/13/2017 22:51	0.35	0.93	19.37	1.88	14.81	-0.020	1.556
11/13/2017 22:52	0.30	0.97	19.82	1.89	14.90	-0.007	1.566
11/13/2017 22:53	0.38	0.58	20.52	16.12	45.89	-0.006	1.562
11/13/2017 22:54	0.40	0.69	20.21	24.19	100.69	0.011	1.570
11/13/2017 22:55	0.40	1.69	20.62	45.98	142.82	-0.007	1.580
11/13/2017 22:56	0.32	0.02	20.93	75.07	208.42	0.001	1.596
11/13/2017 22:57	0.35	-0.41	20.38	54.32	186.14	-0.014	1.616
11/13/2017 22:58	0.40	2.00	20.32	115.19	162.53	0.008	1.618
11/13/2017 22:59	0.40	2.33	20.68	127.62	230.80	-0.005	1.586
11/13/2017 23:00	0.40	0.78	20.26	78.18	159.38	0.007	1.599
11/13/2017 23:01	0.38	1.08	20.66	118.56	193.65	-0.009	1.617
11/13/2017 23:02	0.40	1.14	20.39	70.54	161.61	0.005	1.618
11/13/2017 23:03	0.40	0.08	19.91	7.79	46.21	-0.007	1.598
11/13/2017 23:04	0.40	0.08	19.73	22.51	49.27	-0.004	1.601
11/13/2017 23:05	0.32	1.64	19.56	19.27	61.70	-0.001	1.581
11/13/2017 23:06	0.30	0.87	19.88	6.78	22.17	0.003	1.580
11/13/2017 23:07	0.35	0.71	19.88	21.90	49.80	-0.020	1.612
11/13/2017 23:08	0.40	0.27	19.92	33.00	88.48	0.000	1.609
11/13/2017 23:09	0.40	0.68	19.84	17.86	68.39	-0.008	1.590
11/13/2017 23:10	0.38	1.16	19.92	25.24	61.32	0.001	1.598
11/13/2017 23:11	0.30	0.69	19.71	29.81	90.90	-0.010	1.630
11/13/2017 23:12	0.30	0.25	19.73	20.90	81.99	0.005	1.629
11/13/2017 23:13	0.30	1.64	19.98	9.53	37.67	-0.010	1.637
11/13/2017 23:14	0.30	0.62	20.38	31.45	66.47	-0.004	1.642
11/13/2017 23:15	0.30	0.51	20.17	60.67	100.29	-0.011	1.604
11/13/2017 23:16	0.30	1.33	21.02	13.87	148.04	-0.001	1.597
11/13/2017 23:17	0.25	1.35	20.88	12.04	316.98	-0.001	1.637
11/13/2017 23:18	0.25	0.79	20.44	58.88	154.43	0.005	1.664
11/13/2017 23:19	0.30	1.78	21.03	128.63	210.92	-0.001	1.652
11/13/2017 23:20	0.30	0.98	21.02	90.73	161.29	-0.009	1.642
11/13/2017 23:21	0.30	1.08	20.62	133.32	166.58	0.002	1.644
11/13/2017 23:22	0.30	0.57	20.03	59.52	164.53	-0.009	1.669
11/13/2017 23:23	0.25	1.28	20.73	67.97	82.24	0.004	1.660
11/13/2017 23:24	0.20	0.88	20.09	57.02	128.04	-0.011	1.637
11/13/2017 23:25	0.20	0.21	19.81	12.88	35.88	0.002	1.633
11/13/2017 23:26	0.23	1.11	20.00	36.85	73.42	-0.013	1.652
11/13/2017 23:27	0.30	1.53	19.91	37.43	72.69	0.006	1.637

11/13/2017 23:28	0.30	0.40	19.96	49.62	126.69	-0.008	1.646
11/13/2017 23:29	0.25	1.70	19.46	7.42	43.38	0.003	1.641
11/13/2017 23:30	0.20	1.42	18.94	3.26	19.63	-0.009	1.646
11/13/2017 23:31	0.20	0.34	19.01	1.78	15.98	0.003	1.626
11/13/2017 23:32	0.20	1.18	19.52	1.66	15.73	-0.010	1.634
11/13/2017 23:33	0.20	1.13	19.10	1.67	15.45	-0.002	1.656
11/13/2017 23:34	0.20	0.15	19.73	1.66	15.70	-0.009	1.642
11/13/2017 23:35	0.20	1.44	19.66	1.67	15.62	-0.006	1.656
11/13/2017 23:36	0.20	1.39	19.75	1.73	16.15	-0.007	1.680
11/13/2017 23:37	0.20	1.45	19.29	1.68	15.90	-0.004	1.680
11/13/2017 23:38	0.20	-0.30	20.03	1.67	15.85	-0.004	1.695
11/13/2017 23:39	0.20	0.88	19.74	2.02	18.24	-0.004	1.700
11/13/2017 23:40	0.20	0.96	19.93	2.96	23.75	-0.003	1.706
11/13/2017 23:41	0.28	0.14	19.92	8.45	60.94	-0.014	1.701
11/13/2017 23:42	0.28	0.39	18.99	4.22	29.88	-0.004	1.648
11/13/2017 23:43	0.30	0.04	19.67	1.71	15.79	-0.012	1.658
11/13/2017 23:44	0.22	-1.73	19.03	1.73	15.68	-0.002	1.638
11/13/2017 23:45	0.20	1.18	19.74	1.66	14.76	-0.016	1.641
11/13/2017 23:46	0.22	1.38	20.36	2.15	19.95	-0.005	1.678
11/13/2017 23:47	0.27	1.40	19.50	3.62	34.80	-0.011	1.669
11/13/2017 23:48	0.27	-0.73	19.39	2.31	21.47	-0.004	1.647
11/13/2017 23:49	0.20	-0.34	19.13	2.67	28.30	-0.015	1.653
11/13/2017 23:50	0.20	-1.79	18.57	1.73	14.97	-0.002	1.563
11/13/2017 23:51	0.23	0.81	18.71	1.64	14.07	-0.007	1.522
11/13/2017 23:52	0.30	1.44	18.64	1.72	13.98	-0.001	1.623
11/13/2017 23:53	0.30	-2.53	18.60	1.69	14.15	-0.012	1.599
11/13/2017 23:54	0.20	-1.81	18.34	1.69	13.81	-0.010	1.562
11/13/2017 23:55	0.20	-1.31	18.70	1.69	14.04	-0.004	1.609
11/13/2017 23:56	0.20	-1.88	18.32	1.68	13.82	-0.012	1.580
11/13/2017 23:57	0.22	-0.99	17.99	1.69	13.25	-0.003	1.487
11/13/2017 23:58	0.20	1.58	18.45	1.71	13.30	-0.012	1.582
11/13/2017 23:59	0.20	-0.06	18.60	1.73	13.26	-0.001	1.621

11/15/2017 0:00	9.85	8.35	11.86	1.03	2.88		2.394
11/15/2017 0:01	9.33	7.22	10.70	1.00	3.04		2.398
11/15/2017 0:02	9.05	8.71	12.06	1.68	3.55		2.384
11/15/2017 0:03	8.82	8.43	11.56	3.79	4.50		2.388
11/15/2017 0:04	8.83	9.00	11.31	2.31	3.87		2.387
11/15/2017 0:05	8.82	7.19	11.15	0.87	2.98		2.392
11/15/2017 0:06	8.72	7.79	10.92	0.92	2.94		2.395
11/15/2017 0:07	8.42	7.03	10.02	1.34	3.45		2.417
11/15/2017 0:08	7.95	6.53	10.04	1.09	3.39		2.407
11/15/2017 0:09	7.63	7.13	10.19	2.87	4.86		2.402
11/15/2017 0:10	7.58	7.41	10.54	1.11	3.31		2.397
11/15/2017 0:11	7.80	7.25	10.74	0.84	3.34		2.412
11/15/2017 0:12	7.97	6.63	10.60	1.17	3.69		2.400

11/15/2017 0:13	7.97	6.45	10.58	1.42	3.95	2.402
11/15/2017 0:14	7.73	7.03	10.16	3.14	4.97	2.397
11/15/2017 0:15	7.58	7.28	10.33	1.88	3.85	2.406
11/15/2017 0:16	7.55	6.70	10.55	1.12	3.67	2.405
11/15/2017 0:17	7.72	5.62	10.75	1.16	3.67	2.409
11/15/2017 0:18	8.00	7.88	11.56	0.90	3.52	2.393
11/15/2017 0:19	8.10	5.82	10.18	1.57	4.65	2.426
11/15/2017 0:20	7.87	7.31	10.80	4.57	6.50	2.443
11/15/2017 0:21	7.65	6.93	11.61	3.07	4.56	2.430
11/15/2017 0:22	7.77	6.95	11.22	3.20	5.68	2.434
11/15/2017 0:23	8.03	7.00	10.98	5.68	6.89	2.453
11/15/2017 0:24	8.07	8.00	11.03	8.32	9.44	2.446
11/15/2017 0:25	8.18	7.81	12.22	1.00	4.27	2.424
11/15/2017 0:26	8.50	7.78	11.70	0.95	4.07	2.431
11/15/2017 0:27	8.72	7.08	11.56	3.14	5.13	2.449
11/15/2017 0:28	8.43	6.69	10.28	3.28	6.52	2.453
11/15/2017 0:29	8.07	7.03	11.85	3.62	5.35	2.444
11/15/2017 0:30	8.08	8.49	11.75	0.94	4.08	2.441
11/15/2017 0:31	8.42	9.32	11.53	2.09	4.94	2.453
11/15/2017 0:32	8.83	8.38	11.81	1.28	3.76	2.456
11/15/2017 0:33	8.75	6.96	11.48	0.73	3.90	2.471
11/15/2017 0:34	8.72	8.16	11.51	0.72	3.92	2.474
11/15/2017 0:35	8.58	6.44	11.39	0.74	4.09	2.469
11/15/2017 0:36	8.25	6.47	9.90	0.85	4.20	2.447
11/15/2017 0:37	7.78	6.52	10.14	1.45	4.89	2.439
11/15/2017 0:38	7.52	7.45	10.82	1.12	4.58	2.454
11/15/2017 0:39	7.75	7.48	11.33	1.56	5.01	2.457
11/15/2017 0:40	8.02	7.42	10.51	2.83	5.77	2.445
11/15/2017 0:41	8.02	7.98	11.44	3.65	6.04	2.457
11/15/2017 0:42	8.00	6.67	10.64	1.24	5.23	2.480
11/15/2017 0:43	7.85	6.50	10.95	1.95	5.73	2.459
11/15/2017 0:44	7.83	7.37	10.78	1.66	4.65	2.464
11/15/2017 0:45	7.90	7.22	11.10	1.64	4.39	2.475
11/15/2017 0:46	8.02	5.53	10.13	1.45	4.72	2.464
11/15/2017 0:47	7.82	6.49	10.39	1.11	4.93	2.443
11/15/2017 0:48	7.52	6.84	10.79	1.03	4.75	2.455
11/15/2017 0:49	7.55	6.65	10.39	1.12	5.10	2.461
11/15/2017 0:50	7.65	6.16	10.22	1.06	4.94	2.451
11/15/2017 0:51	7.67	5.56	10.08	0.98	4.72	2.433
11/15/2017 0:52	7.35	5.66	9.20	0.85	4.66	2.451
11/15/2017 0:53	6.98	6.21	9.47	0.87	4.82	2.471
11/15/2017 0:54	6.85	6.40	10.44	0.95	4.84	2.473
11/15/2017 0:55	7.37	7.48	11.36	1.04	4.85	2.475
11/15/2017 0:56	8.00	7.85	11.59	1.07	4.83	2.470
11/15/2017 0:57	8.50	8.68	12.07	1.10	5.02	2.448
11/15/2017 0:58	8.95	9.43	13.74	1.26	5.00	2.468
11/15/2017 0:59	9.75	10.63	14.45	1.40	4.80	2.464

11/15/2017 1:00	10.60	11.66	14.71	1.38	4.72	2.449
11/15/2017 1:01	11.42	12.14	15.79	1.29	4.85	2.430
11/15/2017 1:02	12.15	12.13	16.05	1.23	4.80	2.432
11/15/2017 1:03	12.37	11.60	14.70	1.18	5.06	2.421
11/15/2017 1:04	12.30	10.58	15.02	1.13	4.82	2.414
11/15/2017 1:05	11.83	10.92	15.30	1.08	5.32	2.440
11/15/2017 1:06	11.70	11.03	14.90	1.05	5.03	2.443
11/15/2017 1:07	11.73	11.28	15.32	0.97	5.04	2.445
11/15/2017 1:08	11.93	11.60	16.08	0.95	5.24	2.445
11/15/2017 1:09	12.08	12.27	15.37	0.90	5.05	2.435
11/15/2017 1:10	12.37	12.02	15.79	0.93	5.00	2.424
11/15/2017 1:11	12.25	10.38	15.27	0.87	4.96	2.415
11/15/2017 1:12	12.30	12.35	15.88	0.88	5.07	2.432
11/15/2017 1:13	12.30	13.39	16.06	0.92	4.95	2.410
11/15/2017 1:14	12.17	10.76	15.00	0.87	5.02	2.410
11/15/2017 1:15	12.02	12.76	15.00	0.89	5.00	2.414
11/15/2017 1:16	11.58	10.80	14.38	0.87	5.10	2.417
11/15/2017 1:17	11.42	10.08	14.67	0.85	5.21	2.403
11/15/2017 1:18	11.38	11.61	14.81	0.90	5.31	2.423
11/15/2017 1:19	11.58	10.98	15.60	0.92	5.16	2.421
11/15/2017 1:20	11.90	10.73	15.22	0.98	5.04	2.434
11/15/2017 1:21	12.03	11.16	14.79	1.18	5.49	2.450
11/15/2017 1:22	11.82	9.88	14.35	1.81	5.72	2.440
11/15/2017 1:23	11.38	10.71	14.77	2.53	6.18	2.442
11/15/2017 1:24	11.07	9.64	13.98	4.11	7.29	2.447
11/15/2017 1:25	10.83	9.30	14.17	2.56	6.08	2.449
11/15/2017 1:26	10.88	9.14	14.67	1.41	5.79	2.445
11/15/2017 1:27	10.85	10.98	14.63	0.94	5.01	2.434
11/15/2017 1:28	10.62	7.91	13.00	0.98	5.82	2.433
11/15/2017 1:29	9.75	7.13	11.83	0.77	5.12	2.452
11/15/2017 1:30	8.77	8.12	11.17	1.06	5.78	2.461
11/15/2017 1:31	8.07	7.13	11.27	0.89	5.77	2.448
11/15/2017 1:32	7.87	8.17	11.32	1.55	5.69	2.436
11/15/2017 1:33	7.75	8.33	11.27	1.90	6.61	2.444
11/15/2017 1:34	8.02	8.84	12.36	1.14	5.54	2.438
11/15/2017 1:35	8.38	7.46	12.17	1.22	5.82	2.447
11/15/2017 1:36	8.67	8.89	11.91	1.05	5.31	2.461
11/15/2017 1:37	8.52	7.08	10.66	0.52	5.45	2.446
11/15/2017 1:38	7.72	6.11	9.46	0.94	5.99	2.445
11/15/2017 1:39	7.03	4.83	9.48	1.27	5.99	2.448
11/15/2017 1:40	6.48	6.73	10.07	0.64	5.42	2.452
11/15/2017 1:41	6.70	6.83	10.08	0.56	5.28	2.465
11/15/2017 1:42	6.70	5.43	9.74	2.76	7.23	2.472
11/15/2017 1:43	6.65	5.46	9.67	2.03	5.88	2.460
11/15/2017 1:44	6.50	6.43	9.49	0.90	5.73	2.452
11/15/2017 1:45	6.77	5.85	10.92	1.61	6.13	2.428
11/15/2017 1:46	7.30	7.72	11.11	1.63	5.36	2.438

11/15/2017 1:47	7.75	6.72	10.98	2.44	7.39	2.447
11/15/2017 1:48	8.02	7.06	10.29	0.73	5.47	2.446
11/15/2017 1:49	7.83	6.16	10.33	0.66	5.66	2.443
11/15/2017 1:50	7.52	5.38	9.46	0.72	5.40	2.459
11/15/2017 1:51	6.95	4.77	8.80	1.04	5.48	2.465
11/15/2017 1:52	6.58	4.65	9.32	0.79	5.56	2.468
11/15/2017 1:53	6.63	6.70	10.98	0.94	5.78	2.484
11/15/2017 1:54	7.40	7.47	10.75	0.64	5.43	2.450
11/15/2017 1:55	7.95	7.40	10.48	0.64	5.69	2.450
11/15/2017 1:56	8.10	5.96	10.21	0.62	5.68	2.470
11/15/2017 1:57	7.70	6.98	9.58	0.64	5.66	2.475
11/15/2017 1:58	7.40	5.43	8.89	0.62	5.89	2.467
11/15/2017 1:59	6.60	4.96	8.97	0.56	5.69	2.453
11/15/2017 2:00	6.03	6.63	8.28	0.50	5.98	2.443
11/15/2017 2:01	4.58	4.15	8.71	0.48	5.87	2.457
11/15/2017 2:02	2.78	5.12	9.68	0.50	5.72	2.448
11/15/2017 2:03	1.55	6.24	9.67	0.53	5.67	2.459
11/15/2017 2:04	1.13	7.62	10.32	0.55	5.71	2.474
11/15/2017 2:05	1.10	8.60	11.04	0.54	5.77	2.494
11/15/2017 2:06	1.10	8.88	12.46	0.64	5.70	2.482
11/15/2017 2:07	1.15	9.72	12.72	0.67	5.45	2.484
11/15/2017 2:08	1.23	8.55	12.53	0.64	5.71	2.481
11/15/2017 2:09	1.30	8.92	12.38	0.69	5.90	2.466
11/15/2017 2:10	1.33	9.55	13.02	0.64	5.57	2.457
11/15/2017 2:11	1.40	8.78	12.38	0.62	5.76	2.451
11/15/2017 2:12	1.32	7.68	10.52	0.60	5.57	2.473
11/15/2017 2:13	1.23	8.64	11.51	0.55	5.83	2.472
11/15/2017 2:14	1.22	9.33	12.69	0.57	5.57	2.473
11/15/2017 2:15	1.30	9.08	12.50	0.55	5.61	2.487
11/15/2017 2:16	1.30	8.06	12.04	0.51	5.73	2.489
11/15/2017 2:17	1.25	8.99	12.53	0.48	5.61	2.481
11/15/2017 2:18	1.20	7.10	11.40	0.46	5.66	2.475
11/15/2017 2:19	1.20	6.02	10.59	0.43	5.98	2.486
11/15/2017 2:20	1.20	6.15	10.61	0.44	5.97	2.498
11/15/2017 2:21	1.10	8.66	10.68	0.37	5.64	2.494
11/15/2017 2:22	1.10	6.03	10.27	0.40	6.08	2.503
11/15/2017 2:23	1.05	7.13	10.34	0.39	5.96	2.505
11/15/2017 2:24	1.08	6.63	10.09	0.37	5.99	2.484
11/15/2017 2:25	1.10	7.55	10.01	0.33	5.97	2.482
11/15/2017 2:26	1.10	6.21	10.23	0.32	5.83	2.496
11/15/2017 2:27	1.10	6.95	10.44	0.34	5.88	2.490
11/15/2017 2:28	1.10	7.93	11.29	0.36	6.14	2.465
11/15/2017 2:29	1.97	8.56	11.56	0.43	5.99	2.475
11/15/2017 2:30	4.63	8.01	11.38	0.42	6.03	2.479
11/15/2017 2:31	7.30	8.40	11.44	0.43	6.12	2.486
11/15/2017 2:32	9.07	8.69	11.14	0.44	6.01	2.499
11/15/2017 2:33	9.15	9.28	11.38	0.39	6.08	2.504

11/15/2017 2:34	9.30	8.83	11.09	0.40	6.24	2.499
11/15/2017 2:35	9.08	7.58	9.55	0.40	6.42	2.501
11/15/2017 2:36	8.65	6.42	9.18	0.35	6.26	2.505
11/15/2017 2:37	8.23	6.80	9.56	0.33	6.06	2.496
11/15/2017 2:38	7.92	5.53	8.33	0.31	5.86	2.507
11/15/2017 2:39	7.50	4.73	7.35	0.61	6.80	2.503
11/15/2017 2:40	6.80	5.24	7.20	3.22	8.20	2.527
11/15/2017 2:41	6.28	5.83	7.54	1.61	7.35	2.530
11/15/2017 2:42	6.48	5.44	7.52	1.45	7.13	2.512
11/15/2017 2:43	6.40	4.63	6.62	3.16	7.72	2.531
11/15/2017 2:44	6.65	7.46	7.72	0.58	6.50	2.515
11/15/2017 2:45	6.70	6.60	7.76	0.50	6.04	2.508
11/15/2017 2:46	7.07	5.48	7.01	0.95	6.70	2.524
11/15/2017 2:47	6.92	5.64	6.20	1.17	6.62	2.511
11/15/2017 2:48	6.22	4.63	5.94	1.25	7.62	2.530
11/15/2017 2:49	5.72	4.26	4.89	0.89	6.36	2.530
11/15/2017 2:50	5.32	2.77	4.42	0.68	6.57	2.535
11/15/2017 2:51	5.25	5.94	5.16	0.71	6.66	2.547
11/15/2017 2:52	5.37	4.65	4.09	1.39	7.11	2.546
11/15/2017 2:53	5.28	3.71	3.27	1.58	7.54	2.549
11/15/2017 2:54	5.03	3.26	1.73	0.92	6.79	2.556
11/15/2017 2:55	4.83	4.75	2.71	0.76	6.72	2.553
11/15/2017 2:56	4.90	2.18	3.80	1.74	7.92	2.551
11/15/2017 2:57	5.30	6.08	7.17	2.41	7.71	2.542
11/15/2017 2:58	5.90	6.48	7.34	0.73	6.50	2.530
11/15/2017 2:59	6.23	3.68	6.85	0.76	6.77	2.519
11/15/2017 3:00	6.25	4.85	7.15	0.73	6.99	2.532
11/15/2017 3:01	6.03	5.70	7.24	0.72	7.00	2.550
11/15/2017 3:02	5.82	5.15	6.89	1.58	7.80	2.566
11/15/2017 3:03	5.92	4.43	7.51	0.56	6.85	2.559
11/15/2017 3:04	5.87	5.57	7.33	0.49	7.23	2.552
11/15/2017 3:05	6.00	4.69	7.42	2.28	8.70	2.556
11/15/2017 3:06	5.80	5.19	7.15	2.07	7.27	2.565
11/15/2017 3:07	-2.18	3.69	6.35	2.01	7.72	2.559
11/15/2017 3:08	-14.52	3.66	6.22	1.23	7.70	2.567
11/15/2017 3:09	-25.68	3.49	5.61	2.13	8.17	2.571
11/15/2017 3:10	-27.60	3.07	4.83	0.85	7.33	2.570
11/15/2017 3:11	-27.60	4.12	4.15	0.53	7.36	2.564
11/15/2017 3:12	-27.60	5.21	4.37	0.53	7.15	2.570
11/15/2017 3:13	-27.60	3.91	4.14	0.74	8.00	2.586
11/15/2017 3:14	-27.60	3.44	5.22	3.75	8.24	2.591
11/15/2017 3:15	-27.60	5.61	5.75	1.87	8.79	2.585
11/15/2017 3:16	-27.60	4.16	6.02	0.70	7.57	2.582
11/15/2017 3:17	-27.60	2.99	5.67	1.90	8.54	2.597
11/15/2017 3:18	-27.60	5.58	6.15	1.46	7.87	2.583
11/15/2017 3:19	-27.60	4.12	6.95	0.56	7.65	2.578
11/15/2017 3:20	-27.60	4.11	7.20	0.57	7.59	2.585

11/15/2017 3:21	-27.60	5.42	6.83	0.62	7.56	2.586
11/15/2017 3:22	-27.60	4.90	6.68	0.75	7.94	2.601
11/15/2017 3:23	-27.60	2.22	5.98	3.52	8.29	2.603
11/15/2017 3:24	-27.60	2.40	5.51	3.53	10.12	2.624
11/15/2017 3:25	-27.60	4.33	6.68	1.39	8.21	2.621
11/15/2017 3:26	-27.60	5.05	6.78	0.96	7.81	2.601
11/15/2017 3:27	-27.60	4.10	6.51	0.73	7.86	2.600
11/15/2017 3:28	-27.60	4.19	6.58	0.67	7.81	2.597
11/15/2017 3:29	-27.60	4.39	6.57	0.68	7.85	2.595
11/15/2017 3:30	-27.60	3.65	6.47	0.72	8.04	2.606
11/15/2017 3:31	-27.60	4.66	6.69	0.67	7.79	2.621
11/15/2017 3:32	-27.60	3.48	6.56	0.67	7.95	2.610
11/15/2017 3:33	-27.60	3.48	6.03	1.05	8.78	2.617
11/15/2017 3:34	-27.60	3.96	6.20	1.72	8.94	2.636
11/15/2017 3:35	-27.60	5.13	7.37	1.37	8.10	2.624
11/15/2017 3:36		4.77	6.57	2.86	9.24	2.627
11/15/2017 3:37		4.79	6.28	2.14	9.09	2.625
11/15/2017 3:38		4.49	6.48	1.17	8.47	2.598
11/15/2017 3:39		5.14	7.38	0.89	8.12	2.587
11/15/2017 3:40		5.81	8.43	0.93	8.29	2.598
11/15/2017 3:41		5.75	8.25	0.91	8.23	2.591
11/15/2017 3:42		5.87	7.84	0.89	8.08	2.592
11/15/2017 3:43		5.02	7.21	0.87	8.31	2.606
11/15/2017 3:44		5.12	6.92	0.98	8.53	2.619
11/15/2017 3:45		4.64	6.12	1.65	9.10	2.626
11/15/2017 3:46		5.04	6.08	1.51	8.27	2.609
11/15/2017 3:47		6.16	7.51	0.91	8.40	2.609
11/15/2017 3:48		4.60	7.36	1.00	8.23	2.617
11/15/2017 3:49		5.98	7.59	1.02	8.96	2.602
11/15/2017 3:50		4.05	6.83	1.71	8.18	2.601
11/15/2017 3:51		5.50	8.40	1.51	8.07	2.619
11/15/2017 3:52		5.64	8.42	1.51	8.35	2.598
11/15/2017 3:53		5.18	7.73	1.71	8.08	2.617
11/15/2017 3:54		5.19	6.64	1.90	8.33	2.611
11/15/2017 3:55		6.51	7.70	1.96	8.13	2.618
11/15/2017 3:56		5.21	8.09	2.06	8.01	2.604
11/15/2017 3:57		5.08	7.67	2.31	8.13	2.613
11/15/2017 3:58		6.08	7.70	2.59	8.00	2.622
11/15/2017 3:59		5.13	7.41	2.70	8.36	2.616
11/15/2017 4:00		6.17	7.44	2.71	8.63	2.602
11/15/2017 4:01		6.48	8.53	2.60	8.23	2.615
11/15/2017 4:02		6.06	8.60	2.22	8.63	2.616
11/15/2017 4:03		7.14	10.04	1.50	8.35	2.612
11/15/2017 4:04		7.94	10.70	1.17	8.37	2.617
11/15/2017 4:05		9.09	12.06	1.49	8.57	2.620
11/15/2017 4:06		11.27	13.11	0.85	8.38	2.617
11/15/2017 4:07		10.35	12.30	0.55	8.18	2.608

11/15/2017 4:08	9.23	12.21	0.46	8.50	2.602
11/15/2017 4:09	9.83	12.43	0.44	8.46	2.611
11/15/2017 4:10	10.78	12.79	0.47	8.78	2.609
11/15/2017 4:11	9.38	13.08	0.42	8.38	2.597
11/15/2017 4:12	10.44	13.20	0.39	8.38	2.612
11/15/2017 4:13	10.02	12.56	0.34	8.44	2.627
11/15/2017 4:14	8.93	12.40	0.32	8.45	2.624
11/15/2017 4:15	9.61	12.07	0.32	8.54	2.614
11/15/2017 4:16	9.96	11.65	0.31	8.31	2.604
11/15/2017 4:17	9.56	11.19	0.27	8.61	2.598
11/15/2017 4:18	7.51	10.55	0.59	8.80	2.593
11/15/2017 4:19	6.78	10.18	0.85	8.73	2.588
11/15/2017 4:20	6.37	9.97	0.49	8.53	2.586
11/15/2017 4:21	6.59	9.03	0.62	8.47	2.586
11/15/2017 4:22	6.57	9.17	0.82	8.45	2.623
11/15/2017 4:23	7.15	8.47	1.00	8.63	2.624
11/15/2017 4:24	6.53	8.19	0.97	8.42	2.625
11/15/2017 4:25	5.98	8.32	0.90	8.39	2.607
11/15/2017 4:26	7.14	9.12	0.87	8.53	2.609
11/15/2017 4:27	7.87	8.98	0.89	8.31	2.618
11/15/2017 4:28	5.34	8.69	0.84	7.95	2.597
11/15/2017 4:29	7.55	8.74	0.87	8.31	2.587
11/15/2017 4:30	7.49	8.90	0.83	8.23	2.604
11/15/2017 4:31	7.23	8.95	0.92	8.29	2.628
11/15/2017 4:32	8.13	10.48	0.89	8.01	2.628
11/15/2017 4:33	9.52	11.17	1.37	8.15	2.620
11/15/2017 4:34	8.64	11.22	0.71	8.29	2.616
11/15/2017 4:35	8.68	10.98	0.55	8.18	2.630
11/15/2017 4:36	10.45	11.26	1.08	8.34	2.614
11/15/2017 4:37	10.04	11.57	0.88	8.14	2.611
11/15/2017 4:38	9.36	11.77	0.98	8.41	2.624
11/15/2017 4:39	10.04	11.71	0.79	8.38	2.599
11/15/2017 4:40	9.27	11.60	1.10	8.36	2.585
11/15/2017 4:41	9.34	11.97	0.83	8.01	2.580
11/15/2017 4:42	9.50	11.84	1.29	8.03	2.577
11/15/2017 4:43	9.38	11.62	0.63	7.77	2.559
11/15/2017 4:44	10.06	10.81	0.54	7.78	2.555
11/15/2017 4:45	10.16	10.08	0.64	7.92	2.531
11/15/2017 4:46	9.31	9.91	0.87	7.83	2.544
11/15/2017 4:47	9.86	11.88	0.45	7.61	2.535
11/15/2017 4:48	9.24	11.28	0.20	7.62	2.554
11/15/2017 4:49	8.21	11.12	0.22	7.39	2.526
11/15/2017 4:50	9.75	11.21	0.22	7.20	2.528
11/15/2017 4:51	8.87	10.44	0.31	7.17	2.508
11/15/2017 4:52	8.69	9.42	0.38	7.06	2.509
11/15/2017 4:53	9.63	7.73	0.73	7.14	2.488
11/15/2017 4:54	7.87	9.37	0.70	6.95	2.493

11/15/2017 4:55	8.01	9.92	1.45	7.12	2.494
11/15/2017 4:56	8.78	10.14	2.72	7.56	2.488
11/15/2017 4:57	8.83	9.77	2.35	7.21	2.451
11/15/2017 4:58	9.22	10.25	2.34	7.52	2.479
11/15/2017 4:59	7.12	10.73	3.60	7.40	2.476
11/15/2017 5:00	8.25	10.76	4.04	7.54	2.457
11/15/2017 5:01	7.92	11.43	2.37	7.03	2.447
11/15/2017 5:02	7.07	11.39	2.05	6.69	2.460
11/15/2017 5:03	7.33	10.83	7.02	7.33	2.457
11/15/2017 5:04	8.27	11.94	3.10	6.95	2.449
11/15/2017 5:05	7.91	12.24	1.74	6.34	2.436
11/15/2017 5:06	10.13	13.12	1.67	6.11	2.435
11/15/2017 5:07	8.76	12.95	2.77	6.62	2.413
11/15/2017 5:08	7.14	12.43	3.11	6.52	2.425
11/15/2017 5:09	8.23	12.51	1.56	5.95	2.403
11/15/2017 5:10	9.90	13.06	1.85	6.41	2.410
11/15/2017 5:11	8.53	12.98	2.00	6.22	2.375
11/15/2017 5:12	9.58	13.37	2.19	5.59	2.369
11/15/2017 5:13	10.48	13.81	2.80	6.23	2.381
11/15/2017 5:14	9.79	13.22	1.62	5.72	2.385
11/15/2017 5:15	11.05	13.85	2.27	5.49	2.377
11/15/2017 5:16	11.20	15.09	2.12	5.32	2.361
11/15/2017 5:17	10.68	14.03	2.25	5.18	2.343
11/15/2017 5:18	9.73	14.14	2.69	5.76	2.343
11/15/2017 5:19	9.59	13.71	4.63	5.65	2.359
11/15/2017 5:20	10.39	13.36	6.25	6.73	2.339
11/15/2017 5:21	8.13	13.72	1.69	5.03	2.353
11/15/2017 5:22	9.34	13.67	1.85	4.99	2.357
11/15/2017 5:23	8.63	13.72	1.43	4.81	2.362
11/15/2017 5:24	8.86	13.31	2.38	5.02	2.323
11/15/2017 5:25	8.88	13.45	1.46	4.69	2.333
11/15/2017 5:26	9.15	12.87	1.61	5.02	2.331
11/15/2017 5:27	10.21	13.12	2.65	4.81	2.321
11/15/2017 5:28	10.12	12.77	2.85	4.75	2.320
11/15/2017 5:29	9.78	13.24	3.67	4.87	2.308
11/15/2017 5:30	9.61	13.88	6.22	5.43	2.297
11/15/2017 5:31	8.77	13.39	4.72	4.64	2.296
11/15/2017 5:32	9.40	12.21	13.09	6.63	2.274
11/15/2017 5:33	10.21	12.92	3.48	4.59	2.281
11/15/2017 5:34	9.54	13.42	4.23	4.16	2.279
11/15/2017 5:35	10.73	13.44	8.94	5.66	2.261
11/15/2017 5:36	10.59	13.44	0.58	3.32	2.271
11/15/2017 5:37	8.62	12.92	1.08	3.71	2.250
11/15/2017 5:38	10.23	13.75	1.25	3.57	2.244
11/15/2017 5:39	9.54	13.10	2.29	3.67	2.249
11/15/2017 5:40	10.85	13.19	12.98	6.01	2.254
11/15/2017 5:41	9.31	13.37	1.87	3.68	2.234

11/15/2017 5:42	8.89	12.75	1.88	3.77	2.221
11/15/2017 5:43	10.47	13.53	1.53	3.30	2.248
11/15/2017 5:44	10.05	13.04	3.97	3.52	2.247
11/15/2017 5:45	10.37	13.14	4.52	4.42	2.226
11/15/2017 5:46	9.37	12.71	15.31	7.13	2.222
11/15/2017 5:47	10.15	13.09	3.51	3.87	2.218
11/15/2017 5:48	10.48	13.21	1.31	2.63	2.220
11/15/2017 5:49	9.70	12.69	2.38	3.18	2.193
11/15/2017 5:50	9.95	13.68	3.43	2.63	2.188
11/15/2017 5:51	10.54	13.42	3.43	2.62	2.181
11/15/2017 5:52	8.08	12.28	2.99	2.48	2.174
11/15/2017 5:53	8.11	13.39	4.10	3.21	2.165
11/15/2017 5:54	10.38	13.14	2.34	2.15	2.160
11/15/2017 5:55	9.73	13.66	2.12	2.31	2.153
11/15/2017 5:56	11.05	13.74	1.77	2.10	2.160
11/15/2017 5:57	11.69	14.30	1.19	2.06	2.170
11/15/2017 5:58	9.22	13.58	1.86	2.02	2.159
11/15/2017 5:59	11.17	14.21	5.98	1.95	2.159
11/15/2017 6:00	10.96	13.72	5.33	3.44	2.149
11/15/2017 6:01	10.00	14.28	2.75	2.19	2.129
11/15/2017 6:02	10.23	14.39	2.98	1.71	2.127
11/15/2017 6:03	10.18	13.79	3.12	2.22	2.125
11/15/2017 6:04	10.76	13.82	2.54	1.85	2.132
11/15/2017 6:05	11.42	14.35	1.28	1.29	2.150
11/15/2017 6:06	12.52	14.06	4.49	1.77	2.156
11/15/2017 6:07	11.28	14.42	1.39	1.47	2.127
11/15/2017 6:08	11.13	14.75	2.29	1.40	2.120
11/15/2017 6:09	11.86	14.23	1.13	1.11	2.105
11/15/2017 6:10	11.07	14.77	1.44	1.04	2.124
11/15/2017 6:11	12.10	14.31	3.05	1.52	2.109
11/15/2017 6:12	11.93	15.13	4.95	1.53	2.106
11/15/2017 6:13	11.15	15.20	2.78	1.22	2.103
11/15/2017 6:14	11.58	15.37	2.17	1.00	2.072
11/15/2017 6:15	11.29	14.29	1.86	0.94	2.073
11/15/2017 6:16	11.83	14.96	1.34	0.96	2.089
11/15/2017 6:17	12.48	14.18	0.66	0.55	2.102
11/15/2017 6:18	9.98	14.81	1.69	1.05	2.073
11/15/2017 6:19	11.54	14.90	1.90	0.51	2.070
11/15/2017 6:20	13.16	15.19	1.60	0.49	2.072
11/15/2017 6:21	11.53	14.89	1.20	0.68	2.068
11/15/2017 6:22	12.53	14.78	0.85	0.46	2.077
11/15/2017 6:23	11.71	14.74	1.17	0.74	2.062
11/15/2017 6:24	10.17	14.71	4.50	0.96	2.069
11/15/2017 6:25	11.20	14.55	3.91	1.22	2.087
11/15/2017 6:26	13.08	14.91	4.06	1.18	2.066
11/15/2017 6:27	12.11	14.83	2.61	0.95	2.056
11/15/2017 6:28	12.62	15.14	1.69	0.67	2.061

11/15/2017 6:29	12.73	15.42	2.01	1.02	2.066
11/15/2017 6:30	12.05	15.62	2.38	0.67	2.041
11/15/2017 6:31	10.73	15.41	2.42	0.72	2.031
11/15/2017 6:32	12.14	15.78	0.91	0.43	2.032
11/15/2017 6:33	13.24	15.67	1.61	0.78	2.051
11/15/2017 6:34	13.08	15.39	2.51	0.79	2.040
11/15/2017 6:35	10.19	14.90	1.89	0.97	2.032
11/15/2017 6:36	11.47	15.28	3.33	0.87	2.035
11/15/2017 6:37	10.99	15.40	2.59	0.79	2.021
11/15/2017 6:38	12.08	14.88	1.88	0.60	2.018
11/15/2017 6:39	12.01	14.84	1.45	0.66	2.010
11/15/2017 6:40	11.87	14.58	0.23	0.49	2.009
11/15/2017 6:41	11.76	14.53	0.51	0.48	2.011
11/15/2017 6:42	9.78	14.22	0.87	0.60	2.016
11/15/2017 6:43	12.17	13.86	0.78	0.51	2.032
11/15/2017 6:44	11.65	14.60	0.99	0.58	2.026
11/15/2017 6:45	11.23	15.07	0.42	0.58	2.029
11/15/2017 6:46	12.53	15.76	0.81	0.60	2.023
11/15/2017 6:47	12.92	15.27	0.79	0.46	2.011
11/15/2017 6:48	11.98	15.00	2.04	0.60	2.018
11/15/2017 6:49	12.86	15.56	3.57	0.91	2.027
11/15/2017 6:50	12.78	15.17	2.31	0.69	2.020
11/15/2017 6:51	13.26	15.34	1.69	0.68	2.000
11/15/2017 6:52	12.38	15.96	1.69	0.91	1.993
11/15/2017 6:53	14.68	15.87	2.20	0.65	1.994
11/15/2017 6:54	12.20	15.89	2.71	0.76	2.001
11/15/2017 6:55	14.14	15.61	1.20	0.33	1.987
11/15/2017 6:56	13.28	16.37	1.88	0.63	1.986
11/15/2017 6:57	12.09	15.23	1.18	0.31	1.991
11/15/2017 6:58	11.81	15.16	1.45	0.69	1.988
11/15/2017 6:59	13.14	16.01	1.29	0.72	1.985
11/15/2017 7:00	12.57	15.31	1.88	0.83	1.997
11/15/2017 7:01	12.09	15.32	3.61	1.06	1.978
11/15/2017 7:02	12.31	15.87	8.08	2.06	1.986
11/15/2017 7:03	14.29	16.71	2.39	0.90	1.976
11/15/2017 7:04	14.42	17.29	1.25	0.28	1.959
11/15/2017 7:05	14.10	17.33	0.03	0.33	1.959
11/15/2017 7:06	12.99	17.15	0.37	0.33	1.961
11/15/2017 7:07	13.48	17.44	0.72	0.27	1.962
11/15/2017 7:08	12.90	16.77	0.73	0.30	1.952
11/15/2017 7:09	14.67	17.05	1.11	0.75	1.966
11/15/2017 7:10	14.45	16.96	0.96	0.49	1.962
11/15/2017 7:11	13.93	16.57	2.54	0.60	1.969
11/15/2017 7:12	13.94	16.64	1.09	0.49	1.953
11/15/2017 7:13	14.08	16.98	0.59	0.33	1.945
11/15/2017 7:14	12.95	16.86	0.97	0.22	1.957
11/15/2017 7:15	13.08	17.07	0.76	0.37	1.957

11/15/2017 7:16	15.64	17.07	0.38	0.06	1.952
11/15/2017 7:17	14.45	17.37	0.52	0.05	1.947
11/15/2017 7:18	11.77	17.25	0.62	0.19	1.948
11/15/2017 7:19	14.10	17.19	0.39	0.26	1.933
11/15/2017 7:20	12.92	17.12	0.17	0.12	1.917
11/15/2017 7:21	14.42	16.81	0.60	0.38	1.938
11/15/2017 7:22	13.29	16.45	0.33	0.09	1.947
11/15/2017 7:23	14.06	16.57	-0.01	0.15	1.945
11/15/2017 7:24	13.52	16.87	0.57	0.37	1.951
11/15/2017 7:25	13.91	16.45	1.09	0.33	1.946
11/15/2017 7:26	13.41	16.17	1.12	0.38	1.949
11/15/2017 7:27	14.73	16.65	1.14	0.31	1.944
11/15/2017 7:28	12.94	16.81	0.89	0.40	1.947
11/15/2017 7:29	13.36	16.40	0.54	0.42	1.945
11/15/2017 7:30	13.93	17.05	0.38	-0.03	1.925
11/15/2017 7:31	14.06	17.28	0.19	0.30	1.935
11/15/2017 7:32	12.93	16.70	0.24	-0.03	1.947
11/15/2017 7:33	14.31	16.57	0.11	0.14	1.950
11/15/2017 7:34	14.35	16.54	0.67	0.07	1.951
11/15/2017 7:35	12.21	16.16	0.29	0.45	1.956
11/15/2017 7:36	12.75	15.92	0.31	0.49	1.970
11/15/2017 7:37	13.21	16.44	0.75	0.53	1.952
11/15/2017 7:38	14.56	16.99	0.51	0.27	1.930
11/15/2017 7:39	14.88	17.08	-0.06	-0.16	1.947
11/15/2017 7:40	15.61	17.35	-0.17	-0.17	1.936
11/15/2017 7:41	13.35	17.03	-0.34	0.23	1.940
11/15/2017 7:42	13.23	16.68	-0.27	-0.04	1.953
11/15/2017 7:43	14.10	17.17	0.01	0.08	1.962
11/15/2017 7:44	14.53	16.90	0.31	0.28	1.972
11/15/2017 7:45	13.89	17.08	0.17	0.14	1.947
11/15/2017 7:46	13.73	17.14	-0.05	0.30	1.954
11/15/2017 7:47	12.53	16.28	-0.36	0.14	1.943
11/15/2017 7:48	12.79	16.33	-0.27	-0.17	1.953
11/15/2017 7:49	13.88	16.54	-0.02	0.32	1.960
11/15/2017 7:50	13.50	16.53	-0.11	0.33	1.962
11/15/2017 7:51	13.48	16.63	-0.33	-0.01	1.955
11/15/2017 7:52	12.08	16.07	-0.32	0.33	1.957
11/15/2017 7:53	13.01	15.68	-0.32	0.32	1.966
11/15/2017 7:54	13.62	15.74	0.24	0.18	1.987
11/15/2017 7:55	12.45	16.02	1.31	0.20	1.976
11/15/2017 7:56	13.23	16.06	0.92	0.28	1.982
11/15/2017 7:57	12.27	16.39	0.29	0.11	1.986
11/15/2017 7:58	12.56	15.46	0.81	0.19	1.987
11/15/2017 7:59	13.68	15.73	0.68	0.49	2.001
11/15/2017 8:00	13.13	16.13	0.58	0.21	2.003
11/15/2017 8:01	12.13	15.54	0.99	0.41	1.991
11/15/2017 8:02	12.41	15.97	0.99	0.10	1.974

11/15/2017 8:03		13.19	16.44	1.21	0.21	1.975
11/15/2017 8:04		14.12	16.85	0.89	0.09	1.995
11/15/2017 8:05		15.25	17.52	0.04	0.00	2.006
11/15/2017 8:06		15.01	17.17	0.64	0.27	2.013
11/15/2017 8:07		14.44	17.30	1.05	0.20	2.016
11/15/2017 8:08		13.80	17.67	0.56	0.22	2.009
11/15/2017 8:09		15.12	17.41	-0.08	0.06	2.014
11/15/2017 8:10		14.60	17.33	-0.16	-0.02	2.018
11/15/2017 8:11		14.84	17.29	0.90	0.14	2.017
11/15/2017 8:12		14.68	17.27	0.41	-0.05	2.013
11/15/2017 8:13		13.20	17.36	0.39	0.20	2.025
11/15/2017 8:14		13.86	17.04	-0.02	0.28	2.019
11/15/2017 8:15		13.82	17.39	0.69	0.19	2.031
11/15/2017 8:16		14.93	17.30	0.89	0.19	2.028
11/15/2017 8:17		15.04	17.85	1.09	0.17	2.036
11/15/2017 8:18		14.94	18.31	-0.29	0.07	2.033
11/15/2017 8:19		15.89	17.82	-0.32	0.08	2.033
11/15/2017 8:20		15.98	18.23	-0.24	-0.18	2.042
11/15/2017 8:21		16.14	18.77	-0.23	-0.06	2.046
11/15/2017 8:22		15.10	19.08	-0.24	0.13	2.019
11/15/2017 8:23		16.80	19.40	-0.13	0.10	2.013
11/15/2017 8:24		16.13	19.17	-0.24	0.04	2.022
11/15/2017 8:25		16.42	19.80	-0.30	-0.01	2.015
11/15/2017 8:26		18.00	19.53	-0.23	0.23	2.015
11/15/2017 8:27		16.40	19.19	-0.25	0.03	2.032
11/15/2017 8:28		16.24	19.22	-0.12	0.05	2.038
11/15/2017 8:29		16.24	19.58	-0.21	0.15	2.036
11/15/2017 8:30		16.74	19.84	-0.04	0.03	2.043
11/15/2017 8:31		17.65	19.93	-0.21	0.19	2.045
11/15/2017 8:32		17.89	20.36	0.00	-0.09	2.019
11/15/2017 8:33		17.96	20.26	0.02	0.04	2.012
11/15/2017 8:34	-1.30	17.20	20.49	-0.27	0.03	1.942
11/15/2017 8:35	-1.02	15.87	20.62	-0.21	0.01	1.871
11/15/2017 8:36	-0.95	18.23	20.69	-0.08	0.05	1.842
11/15/2017 8:37	-1.22	18.51	20.49	-0.22	0.02	1.813
11/15/2017 8:38	-1.47	16.99	20.72	-0.34	-0.02	1.783
11/15/2017 8:39	-1.55	16.75	21.03	-0.16	0.10	1.761
11/15/2017 8:40	-1.60	17.10	20.48	1.93	0.94	1.761
11/15/2017 8:41	-1.60	17.33	20.75	4.79	1.24	1.778
11/15/2017 8:42	-1.60	17.45	20.99	1.32	0.90	1.739
11/15/2017 8:43		17.79	20.70	4.69	0.44	1.752
11/15/2017 8:44	-1.38	17.48	21.31	5.79	0.96	1.723
11/15/2017 8:45	-1.12	17.18	21.17	0.28	0.54	1.686
11/15/2017 8:46	-1.10	17.23	20.93	0.00	0.41	1.681
11/15/2017 8:47		17.75	21.11	0.87	0.73	1.639
11/15/2017 8:48		17.53	21.01	3.89	1.00	1.600
11/15/2017 8:49	-1.60	18.15	20.70	0.84	0.48	1.586

11/15/2017 8:50	-1.40	19.28	20.93	2.69	0.61	1.585
11/15/2017 8:51	-1.30	-19.77	21.87	2.64	0.59	1.562
11/15/2017 8:52	-1.37	18.88	21.66	1.35	0.44	1.552
11/15/2017 8:53		17.08	21.30	1.56	0.52	1.569
11/15/2017 8:54	-1.80	17.54	20.84	1.64	0.93	1.566
11/15/2017 8:55	-1.80	17.53	21.49	1.61	0.41	1.542
11/15/2017 8:56		18.50	21.14	0.08	0.42	1.535
11/15/2017 8:57	-1.58	18.62	21.56	0.81	0.65	1.533
11/15/2017 8:58	-1.62	17.49	21.94	0.83	0.73	1.537
11/15/2017 8:59	-1.77	18.78	22.11	1.67	1.20	1.533
11/15/2017 9:00	-2.00	19.26	22.13	3.05	0.73	1.518
11/15/2017 9:01	-2.25	18.05	22.47	2.49	0.92	1.545
11/15/2017 9:02	-2.38	19.73	23.21	2.61	0.96	1.545
11/15/2017 9:03	-2.40	20.27	23.63	1.72	0.56	1.537
11/15/2017 9:04	-2.40	20.13	23.44	0.33	0.61	1.517
11/15/2017 9:05		20.58	23.59	-0.08	0.12	1.499
11/15/2017 9:06		20.64	23.69	1.63	0.84	1.504
11/15/2017 9:07		20.58	23.58	0.22	0.24	1.481
11/15/2017 9:08		19.31	23.48	0.52	0.54	1.485
11/15/2017 9:09		18.89	22.16	3.56	0.47	1.465
11/15/2017 9:10		18.76	22.00	7.45	11.49	1.456
11/15/2017 9:11		20.99	23.26	0.39	0.91	1.433
11/15/2017 9:12		20.87	24.18	0.12	0.28	1.435
11/15/2017 9:13		19.85	24.07	0.18	0.42	1.436
11/15/2017 9:14		20.95	23.63	0.39	0.59	1.431
11/15/2017 9:15		20.75	24.12	0.03	0.00	1.421
11/15/2017 9:16		20.67	24.18	-0.16	0.10	1.413
11/15/2017 9:17		20.41	23.76	-0.26	0.14	1.411
11/15/2017 9:18		20.68	24.32	-0.21	0.23	1.401
11/15/2017 9:19		20.73	23.26	-0.15	0.21	1.405
11/15/2017 9:20		21.22	23.60	-0.15	0.12	1.403
11/15/2017 9:21		19.22	23.25	-0.11	0.26	1.403
11/15/2017 9:22		21.47	24.27	-0.05	0.35	1.406
11/15/2017 9:23		20.42	24.37	0.12	0.34	1.403
11/15/2017 9:24		20.94	23.97	0.22	0.33	1.400
11/15/2017 9:25		20.12	23.70	-0.03	0.12	1.387
11/15/2017 9:26		19.02	23.51	-0.07	0.32	1.384
11/15/2017 9:27		21.24	24.40	-0.10	0.00	1.390
11/15/2017 9:28		20.38	23.96	-0.08	-0.03	1.392
11/15/2017 9:29		21.33	24.40	-0.11	-0.12	1.387
11/15/2017 9:30		20.48	24.62	-0.11	0.06	1.384
11/15/2017 9:31		22.00	24.80	-0.14	-0.06	1.389
11/15/2017 9:32		23.45	25.03	-0.08	-0.08	1.387
11/15/2017 9:33		21.32	25.09	-0.01	-0.25	1.375
11/15/2017 9:34		22.51	25.00	-0.14	-0.16	1.370
11/15/2017 9:35		23.06	25.09	-0.17	-0.10	1.369
11/15/2017 9:36		20.33	24.35	-0.09	-0.18	1.364

11/15/2017 9:37	20.55	24.40	-0.15	-0.13	1.384
11/15/2017 9:38	20.46	24.39	-0.19	-0.21	1.379
11/15/2017 9:39	22.03	24.39	-0.15	-0.15	1.381
11/15/2017 9:40	23.29	25.32	-0.15	-0.25	1.382
11/15/2017 9:41	23.25	25.67	-0.17	-0.29	1.388
11/15/2017 9:42	21.08	25.20	-0.16	-0.21	1.394
11/15/2017 9:43	23.11	25.30	-0.11	-0.14	1.389
11/15/2017 9:44	20.07	25.03	-0.09	-0.31	1.376
11/15/2017 9:45	21.43	24.13	-0.04	-0.22	1.371
11/15/2017 9:46	21.70	23.58	-0.09	-0.42	1.319
11/15/2017 9:47	22.17	23.68	-0.12	-0.44	1.319
11/15/2017 9:48	21.36	23.95	-0.11	-0.16	1.373
11/15/2017 9:49	21.73	24.31	-0.14	-0.48	1.370
11/15/2017 9:50	21.78	25.19	-0.16	-0.18	1.364
11/15/2017 9:51	22.31	24.54	-0.18	-0.34	1.347
11/15/2017 9:52	22.87	24.86	-0.22	-0.70	1.364
11/15/2017 9:53	22.24	25.35	-0.20	-0.26	1.363
11/15/2017 9:54	22.68	24.84	-0.20	-0.13	1.361
11/15/2017 9:55	21.18	24.74	-0.20	0.33	1.377
11/15/2017 9:56	21.78	24.89	-0.16	0.66	1.370
11/15/2017 9:57	21.75	25.00	0.01	-0.34	1.357
11/15/2017 9:58	21.60	25.36	-0.08	-0.60	1.354
11/15/2017 9:59	21.11	25.30	-0.15	-0.56	1.349
11/15/2017 10:00	22.08	24.96	-0.11	-0.67	1.353
11/15/2017 10:01	23.28	25.42	-0.10	-0.54	1.371
11/15/2017 10:02	22.81	25.51	-0.10	-0.54	1.385
11/15/2017 10:03	23.93	25.52	-0.11	-0.74	1.373
11/15/2017 10:04	23.79	26.22	-0.14	-0.63	1.372
11/15/2017 10:05	22.74	26.30	-0.14	-0.61	1.369
11/15/2017 10:06	21.81	25.66	-0.16	-0.56	1.363
11/15/2017 10:07	21.05	25.20	0.02	-0.31	1.363
11/15/2017 10:08	24.95	26.06	0.00	-0.59	1.355
11/15/2017 10:09	22.64	26.43	-0.12	-0.72	1.340
11/15/2017 10:10	23.54	25.84	-0.12	-0.77	1.351
11/15/2017 10:11	26.05	27.24	-0.22	-0.84	1.360
11/15/2017 10:12	24.79	28.43	-0.19	-0.77	1.347
11/15/2017 10:13	23.85	27.26	-0.15	-0.69	1.344
11/15/2017 10:14	24.88	26.74	-0.13	-0.71	1.344
11/15/2017 10:15	23.19	26.18	0.04	-0.40	1.337
11/15/2017 10:16	21.74	25.47	0.68	0.26	1.332
11/15/2017 10:17	22.34	26.11	1.38	0.91	1.341
11/15/2017 10:18	24.86	25.80	0.14	-0.44	1.337
11/15/2017 10:19	22.99	25.64	-0.11	-0.95	1.310
11/15/2017 10:20	23.14	26.51	-0.16	-0.68	1.320
11/15/2017 10:21	23.76	26.26	-0.17	-0.78	1.343
11/15/2017 10:22	23.27	26.56	-0.27	-0.63	1.344
11/15/2017 10:23	25.47	25.89	0.23	-0.03	1.342

11/15/2017 10:24	24.92	27.19	1.29	0.44	1.370
11/15/2017 10:25	24.50	27.92	1.18	-0.21	1.376
11/15/2017 10:26	26.92	28.04	0.49	-0.59	1.370
11/15/2017 10:27	25.16	28.08	-0.01	-0.64	1.368
11/15/2017 10:28	24.20	26.85	0.64	0.99	1.377
11/15/2017 10:29	24.10	26.48	1.36	0.46	1.394
11/15/2017 10:30	24.87	27.42	0.60	-0.45	1.381
11/15/2017 10:31	25.08	27.29	-0.04	-0.51	1.372
11/15/2017 10:32	26.23	27.90	-0.14	-0.51	1.372
11/15/2017 10:33	24.28	27.37	-0.05	-0.61	1.382
11/15/2017 10:34	24.04	26.39	0.31	0.25	1.388
11/15/2017 10:35	26.18	27.78	0.01	-0.76	1.391
11/15/2017 10:36	25.42	27.93	-0.35	-0.72	1.391
11/15/2017 10:37	26.11	28.79	-0.42	-0.76	1.395
11/15/2017 10:38	25.98	29.15	-0.39	-1.04	1.423
11/15/2017 10:39	25.11	28.64	-0.40	-1.17	1.436
11/15/2017 10:40	27.03	28.32	-0.43	-0.90	1.445
11/15/2017 10:41	27.29	28.53	-0.45	-1.02	1.452
11/15/2017 10:42	26.62	28.90	-0.49	-1.14	1.464
11/15/2017 10:43	25.32	28.79	-0.45	-1.08	1.447
11/15/2017 10:44	25.38	28.36	-0.47	-1.12	1.487
11/15/2017 10:45	26.45	28.68	-0.52	-1.30	1.493
11/15/2017 10:46	24.84	28.56	-0.52	-1.11	1.512
11/15/2017 10:47	24.78	28.50	-0.42	-0.04	1.518
11/15/2017 10:48	25.12	27.93	-0.15	-0.44	1.521
11/15/2017 10:49	26.68	28.69	-0.55	-1.35	1.524
11/15/2017 10:50	26.78	28.98	-0.51	-1.17	1.531
11/15/2017 10:51	26.28	28.85	-0.56	-1.11	1.524
11/15/2017 10:52	26.48	29.13	-0.52	-0.99	1.535
11/15/2017 10:53	25.88	29.17	-0.54	-1.16	1.552
11/15/2017 10:54	26.71	29.37	-0.52	-1.02	1.558
11/15/2017 10:55	24.83	29.44	-0.54	-1.01	1.566
11/15/2017 10:56	26.12	28.85	-0.54	-1.16	1.578
11/15/2017 10:57	25.38	29.04	-0.54	-1.03	1.557
11/15/2017 10:58	25.69	28.55	-0.47	-1.16	1.565
11/15/2017 10:59	25.73	28.21	-0.45	-0.91	1.591
11/15/2017 11:00	24.88	28.51	-0.52	-0.93	1.597
11/15/2017 11:01	27.07	29.19	-0.55	-1.14	1.610
11/15/2017 11:02	26.28	29.41	-0.31	-0.83	1.617
11/15/2017 11:03	25.90	29.43	-0.38	-1.25	1.607
11/15/2017 11:04	26.78	29.34	-0.45	-1.02	1.609
11/15/2017 11:05	27.61	29.41	-0.40	-1.06	1.626
11/15/2017 11:06	27.68	30.01	-0.49	-1.08	1.618
11/15/2017 11:07	26.53	30.19	-0.53	-0.93	1.625
11/15/2017 11:08	27.47	30.23	-0.52	-1.05	1.631
11/15/2017 11:09	28.54	29.88	-0.43	-0.82	1.640
11/15/2017 11:10	26.66	29.79	-0.47	-0.87	1.633

11/15/2017 11:11	27.05	29.54	-0.33	-0.65	1.659
11/15/2017 11:12	28.68	30.82	-0.38	-0.87	1.659
11/15/2017 11:13	27.23	30.90	-0.50	-0.92	1.680
11/15/2017 11:14	26.36	30.28	-0.24	-0.68	1.716
11/15/2017 11:15	28.26	30.55	-0.18	-0.60	1.705
11/15/2017 11:16	29.52	31.18	-0.48	-0.97	1.706
11/15/2017 11:17	28.28	31.09	-0.28	-0.52	1.722
11/15/2017 11:18	28.03	31.00	-0.42	-0.81	1.737
11/15/2017 11:19	28.23	30.69	-0.10	-0.66	1.725
11/15/2017 11:20	27.95	30.57	-0.13	-0.53	1.728
11/15/2017 11:21	26.66	30.36	-0.36	-0.87	1.747
11/15/2017 11:22	27.78	29.40	-0.21	-0.72	1.759
11/15/2017 11:23	26.27	29.19	-0.03	-0.44	1.760
11/15/2017 11:24	26.87	28.90	0.03	-0.70	1.765
11/15/2017 11:25	24.37	28.80	0.09	-0.43	1.774
11/15/2017 11:26	23.53	24.50	5.53	15.43	1.787
11/15/2017 11:27	26.45	27.46	4.16	4.33	1.791
11/15/2017 11:28	25.88	27.96	3.90	3.61	1.795
11/15/2017 11:29	29.79	31.37	0.38	-0.54	1.778
11/15/2017 11:30	30.15	32.06	-0.45	-1.19	1.786
11/15/2017 11:31	29.07	31.11	-0.09	1.23	1.803
11/15/2017 11:32	27.80	31.06	-0.23	-0.54	1.808
11/15/2017 11:33	27.38	30.96	-0.21	-0.63	1.803
11/15/2017 11:34	29.08	31.37	-0.55	-0.66	1.836
11/15/2017 11:35	29.06	31.39	-0.48	-0.43	1.836
11/15/2017 11:36	29.35	31.87	0.14	-0.31	1.838
11/15/2017 11:37	29.10	32.30	-0.23	-0.66	1.861
11/15/2017 11:38	28.93	32.57	-0.44	-0.68	1.863
11/15/2017 11:39	29.86	31.48	2.50	5.87	2.050
11/15/2017 11:40	28.88	31.74	1.37	2.78	2.045
11/15/2017 11:41	30.37	31.85	-0.11	0.01	1.931
11/15/2017 11:42	28.29	29.47	-0.42	-0.83	1.903
11/15/2017 11:43	12.51	14.63	2.49	4.10	1.994
11/15/2017 11:44	26.29	26.16	1.34	-0.23	1.923
11/15/2017 11:45	23.92	27.62	-0.02	-0.64	2.046
11/15/2017 11:46	27.43	28.14	-0.49	-0.56	5.495
11/15/2017 11:47	26.37	27.45	-0.47	-0.88	2.866
11/15/2017 11:48	28.83	30.67	-0.69	-0.91	2.339
11/15/2017 11:49	28.62	30.89	-0.77	-0.83	1.923
11/15/2017 11:50	24.78	30.07	-0.80	-0.67	1.908
11/15/2017 11:51	31.26	32.05	0.12	0.50	2.455
11/15/2017 11:52	29.40	32.62	2.92	2.62	2.757
11/15/2017 11:53	31.18	32.52	0.62	0.17	2.052
11/15/2017 11:54	31.77	33.49	1.65	1.23	2.153
11/15/2017 11:55	29.99	33.76	1.79	1.20	2.349
11/15/2017 11:56	30.51	33.12	2.64	1.27	2.580
11/15/2017 11:57	29.98	32.77	2.61	0.00	5.080

11/15/2017 11:58	32.44	32.81	2.31	-0.69	6.012
11/15/2017 11:59	30.83	33.52	-0.28	-0.62	3.980
11/15/2017 12:00	31.20	34.31	2.76	2.27	2.662
11/15/2017 12:01	34.00	34.74	0.95	-0.26	3.550
11/15/2017 12:02	34.03	34.77	-0.65	-0.74	5.661
11/15/2017 12:03	31.76	35.65	-0.66	-0.50	2.653
11/15/2017 12:04	31.71	34.83	-0.47	-0.45	2.143
11/15/2017 12:05	34.12	36.23	1.15	0.87	2.096
11/15/2017 12:06	33.38	35.67	2.27	0.96	2.651
11/15/2017 12:07	32.68	36.00	2.32	1.29	4.557
11/15/2017 12:08	32.73	34.43	4.90	4.07	4.227
11/15/2017 12:09	32.63	34.64	4.94	2.83	3.069
11/15/2017 12:10	31.90	33.48	1.16	-0.12	2.471
11/15/2017 12:11	35.09	68.90	46.77	24.34	3.300
11/15/2017 12:12	35.93	161.23	138.45	294.63	3.386
11/15/2017 12:13	27.20	58.70	35.79	46.54	2.529
11/15/2017 12:14	27.94	65.28	29.20	33.08	2.257
11/15/2017 12:15	27.93	33.05	4.74	2.84	2.004
11/15/2017 12:16	29.74	31.99	3.69	3.20	1.928
11/15/2017 12:17	46.68	164.39	148.17	131.70	2.413
11/15/2017 12:18	173.58	1081.30	946.39	1503.08	8.539
11/15/2017 12:19	159.55	937.70	703.31	1155.68	9.685
11/15/2017 12:20	87.67	563.30	457.18	814.38	8.016
11/15/2017 12:21	42.70	259.22	266.25	507.38	4.741
11/15/2017 12:22	42.47	239.12	280.81	533.68	4.178
11/15/2017 12:23	34.80	195.78	256.71	540.82	4.069
11/15/2017 12:24	40.09	217.80	277.74	635.66	4.113
11/15/2017 12:25	38.76	239.52	337.52	520.72	4.061
11/15/2017 12:26	65.68	329.02	296.38	421.68	4.514
11/15/2017 12:27	83.26	468.93	465.01	679.45	5.929
11/15/2017 12:28	63.72	361.52	257.40	387.57	5.432
11/15/2017 12:29	37.54	224.03	204.37	349.21	4.351
11/15/2017 12:30	38.72	200.08	211.95	358.14	3.596
11/15/2017 12:31	52.02	268.30	324.37	656.68	4.405
11/15/2017 12:32	44.86	248.18	303.65	666.15	4.561
11/15/2017 12:33	35.26	188.00	256.92	549.50	4.083
11/15/2017 12:34	43.34	238.05	270.46	515.05	4.129
11/15/2017 12:35	37.98	210.38	305.17	636.49	4.031
11/15/2017 12:36	43.83	219.25	325.60	713.13	3.938
11/15/2017 12:37	51.42	257.97	390.67	810.39	5.104
11/15/2017 12:38	57.67	285.95	366.76	711.43	4.567
11/15/2017 12:39	51.43	266.83	404.55	822.54	4.808
11/15/2017 12:40	46.33	233.97	368.21	855.13	4.697
11/15/2017 12:41	29.79	161.35	289.58	587.07	3.867
11/15/2017 12:42	40.76	198.70	337.68	762.63	3.861
11/15/2017 12:43	32.63	172.48	281.74	676.61	3.971
11/15/2017 12:44	32.35	164.48	248.99	565.46	3.757

11/15/2017 12:45	27.54	146.77	227.15	467.28	3.537
11/15/2017 12:46	35.67	176.27	276.46	632.24	3.606
11/15/2017 12:47	35.51	183.53	266.87	558.75	3.817
11/15/2017 12:48	31.50	160.87	230.34	527.58	3.985
11/15/2017 12:49	33.73	171.88	235.22	410.25	3.752
11/15/2017 12:50	29.69	150.80	226.12	456.29	3.623
11/15/2017 12:51	29.04	139.73	218.26	421.34	3.531
11/15/2017 12:52	26.72	136.40	193.93	394.88	3.482
11/15/2017 12:53	37.40	162.95	235.57	485.99	3.615
11/15/2017 12:54	56.07	296.08	353.49	766.19	4.483
11/15/2017 12:55	54.83	301.32	336.89	806.60	4.986
11/15/2017 12:56	37.61	169.87	272.64	490.52	4.140
11/15/2017 12:57	61.33	294.55	413.73	828.43	4.873
11/15/2017 12:58	40.51	189.75	409.96	690.41	4.285
11/15/2017 12:59	50.72	257.27	406.39	714.33	4.550
11/15/2017 13:00	49.69	239.78	326.45	641.16	4.522
11/15/2017 13:01	39.37	212.35	306.98	634.96	4.294
11/15/2017 13:02	26.84	139.67	229.26	520.08	3.657
11/15/2017 13:03	30.82	151.15	253.04	463.60	3.533
11/15/2017 13:04	47.13	235.90	335.55	582.26	3.998
11/15/2017 13:05	50.26	261.62	403.01	950.20	5.044
11/15/2017 13:06	38.38	192.50	314.13	643.89	4.232
11/15/2017 13:07	44.15	212.27	357.27	701.89	4.203
11/15/2017 13:08	49.82	265.92	384.31	850.83	4.701
11/15/2017 13:09	40.06	197.58	294.98	579.02	4.253
11/15/2017 13:10	43.12	199.27	271.49	585.39	4.367
11/15/2017 13:11	92.40	395.45	460.65	838.58	5.485
11/15/2017 13:12	62.98	316.38	416.57	955.48	6.080
11/15/2017 13:13	47.86	243.47	346.36	756.71	5.114
11/15/2017 13:14	36.83	165.35	266.12	491.78	4.102
11/15/2017 13:15	44.33	222.53	336.30	797.51	4.555
11/15/2017 13:16	38.91	176.03	286.72	663.42	4.491
11/15/2017 13:17	74.88	347.33	483.50	960.63	5.224
11/15/2017 13:18	51.41	214.42	374.69	911.27	5.383
11/15/2017 13:19	148.52	313.85	436.43	960.10	5.431
11/15/2017 13:20	58.62	271.78	390.88	921.40	5.249
11/15/2017 13:21	55.02	245.87	380.32	846.52	5.174
11/15/2017 13:22	42.69	193.43	306.61	668.02	4.584
11/15/2017 13:23	74.12	306.55	462.10	942.35	5.278
11/15/2017 13:24	78.45	364.37	486.19	1160.90	6.185
11/15/2017 13:25	59.46	259.50	370.05	925.58	5.713
11/15/2017 13:26	57.21	253.10	370.10	958.00	5.261
11/15/2017 13:27	55.43	253.57	359.87	821.93	5.137
11/15/2017 13:28	55.72	238.62	373.49	782.41	4.959
11/15/2017 13:29	59.07	254.45	381.19	805.43	5.234
11/15/2017 13:30	63.43	263.70	377.34	909.38	5.396
11/15/2017 13:31	58.61	251.97	394.96	855.96	5.577

11/15/2017 13:32	49.88	205.53	310.08	683.13	4.982
11/15/2017 13:33	45.02	198.40	255.77	535.70	4.612
11/15/2017 13:34	45.62	196.32	286.31	605.38	4.737
11/15/2017 13:35	40.38	180.97	267.01	557.53	4.322
11/15/2017 13:36	51.18	211.95	340.84	689.94	4.844
11/15/2017 13:37	35.03	142.43	252.78	535.13	4.406
11/15/2017 13:38	30.88	139.82	203.09	449.47	3.974
11/15/2017 13:39	41.92	180.23	249.26	459.27	4.210
11/15/2017 13:40	54.98	227.88	307.96	654.70	4.917
11/15/2017 13:41	45.38	194.08	262.05	522.31	4.768
11/15/2017 13:42	71.15	281.45	384.31	904.45	5.171
11/15/2017 13:43	55.54	227.53	334.05	659.33	4.907
11/15/2017 13:44	97.63	384.25	534.36	1003.35	6.274
11/15/2017 13:45	104.96	438.33	473.44	1114.07	7.735
11/15/2017 13:46	71.07	303.03	393.45	944.39	6.468
11/15/2017 13:47	75.39	303.00	403.37	872.43	5.926
11/15/2017 13:48	88.61	344.23	409.21	816.57	6.288
11/15/2017 13:49	95.02	402.72	465.52	1003.74	6.891
11/15/2017 13:50	77.90	321.87	470.18	1066.43	6.353
11/15/2017 13:51	106.04	418.32	591.75	1263.00	6.568
11/15/2017 13:52	360.08	1482.25	576.73	876.30	11.208
11/15/2017 13:53	423.03	2007.50	375.53	347.88	19.833
11/15/2017 13:54	111.72	497.83	142.39	130.36	11.106
11/15/2017 13:55	44.60	192.08	93.99	82.40	5.362
11/15/2017 13:56	19.77	95.72	55.66	48.82	3.858
11/15/2017 13:57	17.88	95.56	66.33	78.77	3.581
11/15/2017 13:58	43.64	161.10	163.25	280.85	3.731
11/15/2017 13:59	99.58	354.58	295.00	959.18	6.539
11/15/2017 14:00	72.52	280.60	295.00	774.93	5.992
11/15/2017 14:01	25.46	97.62	114.56	196.83	4.103
11/15/2017 14:02	36.37	136.89	133.09	230.87	3.844
11/15/2017 14:03	64.49	257.75	171.21	315.03	4.779
11/15/2017 14:04	35.49	139.47	170.72	350.15	4.102
11/15/2017 14:05	39.69	162.12	167.42	330.57	4.155
11/15/2017 14:06	30.44	125.16	144.38	309.18	3.635
11/15/2017 14:07	39.08	145.97	143.18	282.03	3.894
11/15/2017 14:08	27.72	118.92	108.41	267.92	3.786
11/15/2017 14:09	31.48	111.85	114.87	193.73	3.455
11/15/2017 14:10	29.63	116.15	167.20	325.45	3.601
11/15/2017 14:11	85.91	330.97	450.28	1051.88	4.557
11/15/2017 14:12	120.13	393.62	667.65	1367.62	5.843
11/15/2017 14:13	52.53	216.50	196.76	473.80	6.158
11/15/2017 14:14	137.28	469.37	280.28	272.58	5.225
11/15/2017 14:15	250.69	953.02	386.74	323.63	9.821
11/15/2017 14:16	154.93	605.47	275.10	311.63	8.567
11/15/2017 14:17	54.29	218.72	109.26	139.51	5.916
11/15/2017 14:18	20.65	81.84	57.27	43.64	3.259

11/15/2017 14:19	30.87	129.67	76.96	59.43	3.615
11/15/2017 14:20	10.12	41.66	28.98	33.56	2.875
11/15/2017 14:21	59.01	202.70	174.11	282.09	3.957
11/15/2017 14:22	100.73	311.12	383.15	669.85	5.704
11/15/2017 14:23	100.36	359.20	294.31	633.68	6.074
11/15/2017 14:24	85.92	268.60	259.73	538.75	6.078
11/15/2017 14:25	84.63	280.10	285.64	675.20	5.844
11/15/2017 14:26	72.88	219.43	219.54	434.22	5.048
11/15/2017 14:27	71.72	252.85	256.51	570.92	5.529
11/15/2017 14:28	71.16	217.33	248.17	456.78	4.724
11/15/2017 14:29	76.44	266.08	242.26	491.87	5.263
11/15/2017 14:30	55.88	189.32	210.89	432.63	4.691
11/15/2017 14:31	61.42	206.02	205.70	397.35	4.410
11/15/2017 14:32	57.22	186.72	219.96	463.25	4.537
11/15/2017 14:33	55.73	179.95	196.79	408.42	4.218
11/15/2017 14:34	64.43	218.38	241.35	519.05	4.674
11/15/2017 14:35	51.04	181.10	205.48	398.62	4.296
11/15/2017 14:36	39.23	127.85	155.50	316.57	3.776
11/15/2017 14:37	49.50	158.55	178.13	349.95	3.805
11/15/2017 14:38	51.38	164.58	208.74	476.97	4.196
11/15/2017 14:39	54.77	172.25	183.60	367.48	3.959
11/15/2017 14:40	137.47	385.70	353.89	807.55	6.147
11/15/2017 14:41	104.71	328.98	284.94	608.60	6.110
11/15/2017 14:42	86.68	271.25	260.34	577.52	5.210
11/15/2017 14:43	89.51	284.53	244.11	479.05	5.381
11/15/2017 14:44	101.92	295.47	272.25	502.85	5.393
11/15/2017 14:45	65.99	216.17	202.93	446.02	5.037
11/15/2017 14:46	36.57	132.33	136.33	246.57	3.898
11/15/2017 14:47	21.63	81.22	89.78	127.98	3.173
11/15/2017 14:48	9.24	34.33	52.82	68.89	2.690
11/15/2017 14:49	10.78	37.95	58.83	94.23	2.572
11/15/2017 14:50	19.77	61.24	62.22	68.36	2.726
11/15/2017 14:51	17.83	50.63	50.93	47.89	2.828
11/15/2017 14:52	14.00	38.36	47.07	35.95	2.663
11/15/2017 14:53	18.82	47.56	36.62	29.42	2.755
11/15/2017 14:54	18.88	54.72	39.76	26.52	2.773
11/15/2017 14:55	11.68	35.20	32.21	41.32	2.790
11/15/2017 14:56	7.93	15.54	25.58	41.72	2.517
11/15/2017 14:57	6.89	9.40	16.99	18.97	2.446
11/15/2017 14:58	10.59	27.09	33.12	13.71	2.763
11/15/2017 14:59	7.99	17.96	20.77	14.99	3.237
11/15/2017 15:00	5.66	6.57	19.71	5.05	3.282
11/15/2017 15:01	5.93	3.20	19.71	1.65	2.199
11/15/2017 15:02	5.79	4.53	5.61	1.53	2.127
11/15/2017 15:03	5.80	5.10	3.40	1.18	2.556
11/15/2017 15:04	5.63	12.95	4.77	3.51	3.905
11/15/2017 15:05	5.53	5.65	4.59	1.60	3.757

11/15/2017 15:06	7.13	8.04	3.29	1.24	2.069
11/15/2017 15:07	7.96	8.83	3.07	1.61	2.144
11/15/2017 15:08	9.22	12.18	3.30	1.74	2.275
11/15/2017 15:09	9.25	8.64	3.28	1.90	2.212
11/15/2017 15:10	9.51	6.65	3.07	1.73	2.141
11/15/2017 15:11	11.08	12.22	3.86	2.34	2.153
11/15/2017 15:12	11.80	11.99	2.49	1.24	2.323
11/15/2017 15:13	10.88	11.22	2.20	1.26	2.199
11/15/2017 15:14	11.73	12.55	2.14	1.34	2.320
11/15/2017 15:15	12.08	13.41	2.10	1.45	2.375
11/15/2017 15:16	11.18	11.73	1.93	1.31	2.639
11/15/2017 15:17	10.91	11.28	1.80	1.16	2.401
11/15/2017 15:18	11.03	9.75	1.61	1.36	2.355
11/15/2017 15:19	11.26	10.82	1.54	1.40	2.263
11/15/2017 15:20	10.97	7.04	1.34	0.84	2.280
11/15/2017 15:21	10.32	9.41	1.26	0.95	2.035
11/15/2017 15:22	10.45	8.06	1.22	1.15	2.004
11/15/2017 15:23	11.03	8.29	1.06	0.73	2.209
11/15/2017 15:24	11.90	9.44	1.05	0.91	2.121
11/15/2017 15:25	9.48	9.07	1.03	1.07	2.005
11/15/2017 15:26	10.52	9.27	1.02	0.77	2.050
11/15/2017 15:27	8.42	10.09	1.32	0.96	2.367
11/15/2017 15:28	11.14	9.73	1.05	1.00	2.183
11/15/2017 15:29	13.02	10.20	0.99	0.81	2.022
11/15/2017 15:30	11.96	9.30	1.02	0.75	2.005
11/15/2017 15:31	13.63	9.51	1.25	1.85	1.993
11/15/2017 15:32	13.36	9.66	2.33	3.72	2.008
11/15/2017 15:33	11.97	10.04	1.91	2.32	2.007
11/15/2017 15:34	12.87	11.45	1.34	1.20	2.046
11/15/2017 15:35	11.23	9.93	3.21	14.25	2.459
11/15/2017 15:36	13.20	11.67	1.36	1.18	5.366
11/15/2017 15:37	12.53	12.66	0.90	1.04	7.414
11/15/2017 15:38	12.05	11.98	0.86	0.89	4.123
11/15/2017 15:39	13.90	11.97	0.84	0.98	2.029
11/15/2017 15:40	14.13	13.80	2.04	2.57	2.202
11/15/2017 15:41	13.38	14.51	1.43	1.19	2.110
11/15/2017 15:42	12.72	14.31	0.82	0.65	2.304
11/15/2017 15:43	14.73	15.87	0.75	0.79	3.182
11/15/2017 15:44	15.12	15.39	0.81	0.80	4.921
11/15/2017 15:45	13.92	16.62	0.77	1.01	2.891
11/15/2017 15:46	15.91	17.41	2.55	3.71	2.196
11/15/2017 15:47	14.66	16.35	4.64	4.69	2.186
11/15/2017 15:48	17.28	16.49	1.45	1.30	2.172
11/15/2017 15:49	16.85	17.25	1.67	1.28	2.424
11/15/2017 15:50	15.68	17.43	0.95	1.03	2.223
11/15/2017 15:51	14.91	17.42	0.61	0.85	2.158
11/15/2017 15:52	16.63	18.05	0.45	0.80	2.111

11/15/2017 15:53	15.08	16.76	0.37	0.96	2.058
11/15/2017 15:54	15.65	16.45	0.39	1.07	2.007
11/15/2017 15:55	16.51	16.64	0.40	1.12	2.016
11/15/2017 15:56	18.59	17.29	0.43	1.14	1.988
11/15/2017 15:57	17.97	18.27	0.25	0.95	1.935
11/15/2017 15:58	18.08	17.67	0.20	1.10	1.891
11/15/2017 15:59	18.89	17.40	0.25	1.15	1.883
11/15/2017 16:00	15.73	14.69	0.24	0.69	1.899
11/15/2017 16:01	18.16	15.26	0.25	0.86	1.875
11/15/2017 16:02	23.68	15.28	0.25	0.99	1.803
11/15/2017 16:03	19.98	17.53	0.17	0.86	1.826
11/15/2017 16:04	27.51	21.85	0.12	1.23	1.829
11/15/2017 16:05	27.67	21.15	0.15	1.13	1.785
11/15/2017 16:06	-0.36	-17.28	0.09	1.34	1.780
11/15/2017 16:07	-2.27	-10.69	0.10	1.26	1.748
11/15/2017 16:08	-1.73	-3.40	0.11	1.29	1.767
11/15/2017 16:09	-3.11	-1.23	0.11	1.21	1.762
11/15/2017 16:10	-2.04	0.33	0.08	1.47	1.743
11/15/2017 16:11	-1.53	0.33	0.40	1.53	1.759
11/15/2017 16:12	151.43	134.27	0.30	1.25	1.744
11/15/2017 16:13	86.77	87.84	0.13	1.28	1.723
11/15/2017 16:14	89.22	89.45	0.06	1.16	1.722
11/15/2017 16:15	93.15	94.14	0.05	1.12	1.720
11/15/2017 16:16	97.53	97.17	0.00	1.26	1.757
11/15/2017 16:17	97.69	97.27	0.03	1.19	1.733
11/15/2017 16:18	99.16	96.67	0.03	1.48	1.754
11/15/2017 16:19	89.33	93.12	0.10	1.29	1.773
11/15/2017 16:20	16.86	31.64	0.01	1.05	1.711
11/15/2017 16:21	19.57	26.73	-0.01	1.04	1.690
11/15/2017 16:22	18.55	19.44	0.04	1.16	1.684
11/15/2017 16:23	20.92	17.53	0.06	1.09	1.765
11/15/2017 16:24	20.99	16.54	0.22	1.13	1.861
11/15/2017 16:25	18.88	15.55	0.09	1.12	1.705
11/15/2017 16:26	19.98	15.34	0.02	1.19	1.709
11/15/2017 16:27	20.38	15.83	-0.05	1.14	1.723
11/15/2017 16:28	-28.25	16.28	-0.04	1.18	1.707
11/15/2017 16:29	19.93	15.42	-0.05	1.10	1.707
11/15/2017 16:30	20.47	16.63	-0.06	1.10	1.695
11/15/2017 16:31	19.44	16.48	0.03	1.14	1.699
11/15/2017 16:32	20.89	16.46	-0.01	1.10	1.697
11/15/2017 16:33	20.42	16.06	0.01	1.06	1.684
11/15/2017 16:34	19.01	15.36	-0.03	1.07	1.693
11/15/2017 16:35	20.84	16.44	-0.03	1.05	1.708
11/15/2017 16:36	19.71	17.05	0.03	1.04	1.723
11/15/2017 16:37	19.64	15.74	0.17	1.87	1.712
11/15/2017 16:38	21.03	15.08	2.40	4.87	1.672
11/15/2017 16:39	19.34	14.89	0.46	1.46	1.657

11/15/2017 16:40	18.83	14.27	0.08	1.32	1.653
11/15/2017 16:41	19.64	14.81	0.01	0.94	1.659
11/15/2017 16:42	20.53	15.84	0.00	0.89	1.661
11/15/2017 16:43	20.84	16.55	-0.03	1.00	1.690
11/15/2017 16:44	19.84	15.40	-0.07	1.03	1.682
11/15/2017 16:45	19.64	14.47	-0.07	0.97	1.649
11/15/2017 16:46	18.35	13.26	-0.13	1.05	1.683
11/15/2017 16:47	19.83	14.49	0.06	1.02	1.692
11/15/2017 16:48	20.01	14.60	-0.05	0.80	1.658
11/15/2017 16:49	19.40	14.17	-0.05	0.88	1.673
11/15/2017 16:50	18.89	12.18	0.15	1.06	1.703
11/15/2017 16:51	20.34	10.71	-0.52	1.01	1.727
11/15/2017 16:52	18.88	9.53	-0.56	0.44	1.733
11/15/2017 16:53	18.33	10.23	-0.57	0.43	1.714
11/15/2017 16:54	18.94	11.23	-0.54	16.89	1.726
11/15/2017 16:55	19.86	12.09	-0.35	96.81	1.759
11/15/2017 16:56	18.92	12.74	-0.17	96.73	1.772
11/15/2017 16:57	19.06	13.71	-0.03	96.67	1.764
11/15/2017 16:58	19.74	13.05	0.06	96.85	1.762
11/15/2017 16:59	17.90	12.52	0.11	96.57	1.762
11/15/2017 17:00	18.81	12.82	0.11	96.40	1.752
11/15/2017 17:01	18.00	11.69	0.09	96.89	1.731
11/15/2017 17:02	19.34	11.74	0.10	76.58	1.734
11/15/2017 17:03	18.76	11.84	0.10	12.35	1.731
11/15/2017 17:04	18.75	10.81	61.84	8.02	1.746
11/15/2017 17:05	18.91	11.34	92.92	8.64	1.758
11/15/2017 17:06	17.03	10.33	92.85	8.61	1.743
11/15/2017 17:07	17.22	10.67	71.80	8.25	1.755
11/15/2017 17:08	19.87	11.16	0.30	1.39	1.775
11/15/2017 17:09	18.82	11.55	0.01	0.60	1.780
11/15/2017 17:10	16.96	11.03	-0.01	0.79	1.804
11/15/2017 17:11	18.70	11.80	-0.03	0.69	1.809
11/15/2017 17:12	19.87	11.50	-0.04	1.03	1.798
11/15/2017 17:13	19.82	12.28	-0.03	0.76	1.809
11/15/2017 17:14	15.54	8.57	0.01	0.83	1.816
11/15/2017 17:15	10.78	5.30	-0.01	0.66	1.764
11/15/2017 17:16	10.63	4.44	-0.02	0.67	1.765
11/15/2017 17:17	10.07	5.41	-0.06	0.70	1.777

USFS Missoula Chamber 2018

Date&Time	O3 NO-CL	O3 SL-UV	O3 UV-C-H	O3 UV-C	NO2-C	NO	CO	THC
4/16/2018 0:00	0.2	0.8	1.6	19.4	4.2	4.4	0.0417	3.6
4/16/2018 0:01	0.2	1.5	3.6	19.1	4.2	4.4	0.0502	3.9
4/16/2018 0:02	0.2	0.6	2.8	18.9	4.2	4.4	0.0462	4.2
4/16/2018 0:03	0.1	0.5	2.2	19.1	4.2	4.4	0.047	4.3
4/16/2018 0:04	0.1	1.4	1.3	19.2	4.2	4.4	0.0591	4.5
4/16/2018 0:05	0.1	1	1.2	19.5	4.2	4.4	0.0417	4.6
4/16/2018 0:06	0.2	1	1.9	19.4	4.2	4.4	0.0602	4.6
4/16/2018 0:07	0.2	0.8	3.1	19.2	4.2	4.4	0.0391	4.5
4/16/2018 0:08	0.2	0.7	2.1	19.3	4.1	4.4	0.0472	4.5
4/16/2018 0:09	0.2	0.9	2.4	19.6	4.2	4.4	0.05	4.4
4/16/2018 0:10	0.2	0.7	2	19.2	4.2	4.4	0.044	4.5
4/16/2018 0:11	0.2	0.9	2.6	19.3	4.2	4.4	0.0542	4.2
4/16/2018 0:12	0.2	0.9	1.5	19.4	4.2	4.4	0.0408	2.5
4/16/2018 0:13	0.2	0.4	2.5	19.2	4.2	4.4	0.0526	2.1
4/16/2018 0:14	0.2	1.2	2.3	19	4.2	4.4	0.0501	2.3
4/16/2018 0:15	0.2	1.2	1.9	19.2	4.2	4.4	0.0367	2.5
4/16/2018 0:16	0.1	0.6	1.5	19.2	4.2	4.4	0.0502	2.7
4/16/2018 0:17	0.1	0.3	2	19.2	4.1	4.4	0.042	2.9
4/16/2018 0:18	0.1	0.9	2.7	19.1	4.1	4.4	0.0527	3.1
4/16/2018 0:19	0.1	0.6	2.5	19.4	4.2	4.4	0.0483	3.2
4/16/2018 0:20	0.1	0.4	2.8	19.2	4.1	4.4	0.0435	3.5
4/16/2018 0:21	0.1	1.3	1.3	19.2	4	4.4	0.0531	3.6
4/16/2018 0:22	0.1	1.6	2.3	19.2	4.1	4.4	0.0491	3.7
4/16/2018 0:23	0.1	0.5	3.4	19.3	4.1	4.4	0.0469	3.8
4/16/2018 0:24	0	1	1.6	19.3	4.2	4.4	0.0516	3.9
4/16/2018 0:25	0	1.2	2.4	19.1	4.1	4.4	0.0384	4
4/16/2018 0:26	0.1	1.5	3.2	19.1	4.1	4.4	0.0528	4
4/16/2018 0:27	0.1	0.7	3	19.5	4.2	4.4	0.0436	4.1
4/16/2018 0:28	0.1	0.9	1.5	19.3	4.1	4.4	0.0486	4.1
4/16/2018 0:29	0.1	0.8	1.9	19.1	4	4.4	0.0495	4
4/16/2018 0:30	0.1	1	1.5	19.2	4.1	4.4	0.0365	4
4/16/2018 0:31	0.1	0.6	2	19.4	4.1	4.4	0.0551	4
4/16/2018 0:32	0.2	0	1.8	19.2	4.1	4.4	0.0394	4.1
4/16/2018 0:33	0.2	1	1.7	19.3	4.1	4.5	0.0443	4.1
4/16/2018 0:34	0.2	1	0.9	19.1	4.1	4.4	0.0553	4.1
4/16/2018 0:35	0.3	1	3.1	19.1	4.2	4.4	0.041	4.1
4/16/2018 0:36	0.3	0.9	2.9	19.1	4.1	4.5	0.0505	4
4/16/2018 0:37	0.2	1	1.6	19.4	4.2	4.4	0.0464	4
4/16/2018 0:38	0.2	0.9	2.1	19.3	4.1	4.4	0.0463	4.1
4/16/2018 0:39	0.2	0.9	2.6	19.3	4.1	4.4	0.0483	4.1
4/16/2018 0:40	0.2	1	2.6	19.1	4.1	4.4	0.0403	4.1
4/16/2018 0:41	0.2	1	2	19.1	4.1	4.4	0.0508	4.2
4/16/2018 0:42	0.2	0.8	3.2	19.3	4.1	4.4	0.0386	4.2
4/16/2018 0:43	0.2	0.6	3.2	19.2	4.1	4.5	0.0533	4.3
4/16/2018 0:44	0.2	1.6	2.6	19.3	4.2	4.4	0.0424	4.4

4/16/2018 0:45	0.3	0.6	1.4	19.2	4.1	4.4	0.0448	4.4
4/16/2018 0:46	0.3	0.2	2.4	19.4	4.1	4.4	0.0494	4.4
4/16/2018 0:47	0.3	0.9	1.8	19.4	4.1	4.5	0.0363	4.5
4/16/2018 0:48	0.3	0.5	0.6	19.1	4.2	4.5	0.0522	4.6
4/16/2018 0:49	0.3	0.4	1.7	19.1	4.1	4.5	0.0411	4.6
4/16/2018 0:50	0.2	0.6	2	19.4	4.2	4.4	0.0434	4.5
4/16/2018 0:51	0.2	1.1	2.4	19.6	4.3	4.4	0.0425	4.5
4/16/2018 0:52	0.2	-0.4	3.4	19.2	4.2	4.4	0.0389	4.6
4/16/2018 0:53	0.2	-3.2	3.3	19	4.2	4.4	0.0465	4.6
4/16/2018 0:54	0.2	-7.7	1.2	19.1	4.3	4.4	0.041	4.6
4/16/2018 0:55	0.2	-9.2	1.3	19.1	4.2	4.4	0.0502	4.6
4/16/2018 0:56	0.2	-9.5	1.4	19.3	4.1	4.4	0.0413	4.7
4/16/2018 0:57	0.2	-10	3	19	4.2	4.4	0.0467	4.7
4/16/2018 0:58	0.3	-10.5	2.2	19.1	4.2	4.4	0.0424	4.7
4/16/2018 0:59	0.2	-10.9	2.4	19.3	4.2	4.4	0.0381	4.8
4/16/2018 1:00	0.3	-10.5	3	19.2	4.2	4.4	0.0546	4.9
4/16/2018 1:01	0.2	-10.9	2.9	19.2	4.2	4.4	0.0451	4.8
4/16/2018 1:02	0.3	-10.9	1.7	19.3	4.2	4.4	0.0388	4.7
4/16/2018 1:03	0.3	-10.5	1.9	19.3	4.2	4.4	0.0473	4.7
4/16/2018 1:04	0.2	-11.3	2.8	19.2	4.2	4.4	0.0368	4.6
4/16/2018 1:05	0.2	-11	1.8	19	4.2	4.4	0.0471	4.7
4/16/2018 1:06	0.2	-11.2	2.8	19	4.2	4.5	0.0429	4.7
4/16/2018 1:07	0.2	-10.9	1.6	19.3	4.2	4.5	0.0374	4
4/16/2018 1:08	0.2	-10.6	2	19.2	4.2	4.5	0.0502	2.7
4/16/2018 1:09	0.3	-11.9	3.1	19.1	4.3	4.5	0.0369	2.8
4/16/2018 1:10	0.3	-11.5	2.1	19.1	4.3	4.5	0.0473	3.1
4/16/2018 1:11	0.2	-10.9	2.2	19.1	4.2	4.5	0.0489	3.2
4/16/2018 1:12	0.2	-11.2	3.4	19	4.3	4.5	0.0417	3.3
4/16/2018 1:13	0.2	-11.8	2.2	19.2	4.3	4.5	0.053	3.4
4/16/2018 1:14	0.2	-11.1	2	19.3	4.2	4.5	0.0366	3.4
4/16/2018 1:15	0.2	-11.4	1.8	19.3	4.4	4.5	0.0524	3.4
4/16/2018 1:16	0.2	-11.2	2.5	19.3	4.4	4.4	0.0429	3.3
4/16/2018 1:17	0.3	-11.4	1.8	19.2	4.3	4.5	0.045	3.2
4/16/2018 1:18	0.3	-10.7	1.4	19.2	4.3	4.5	0.0499	2.9
4/16/2018 1:19	0.3	-10.5	1.7	19	4.3	4.5	0.0313	1.8
4/16/2018 1:20	0.2	-11.4	1.5	19.3	4.3	4.5	0.0494	1.6
4/16/2018 1:21	0.2	-11.3	3.5	19.4	4.5	4.4	0.0383	1.7
4/16/2018 1:22	0.2	-11.6	2.9	19.2	4.5	4.4	0.046	1.7
4/16/2018 1:23	0.2	-11.2	1.7	19.4	4.6	4.4	0.0501	1.7
4/16/2018 1:24	0.2	-10.8	2.5	19.4	4.6	4.4	0.0363	1.8
4/16/2018 1:25	0.2	-11.5	2.5	19.4	4.6	4.4	0.0476	1.8
4/16/2018 1:26	0.2	-10.9	2.1	19.4	4.7	4.3	0.0492	1.9
4/16/2018 1:27	0.2	-10.8	2.3	19.4	4.6	4.3	0.0365	1.9
4/16/2018 1:28	0.2	-11	1.5	19.1	4.5	4.3	0.0485	1.9
4/16/2018 1:29	0.2	-11.7	0	19.2	4.4	4.3	0.0393	2
4/16/2018 1:30	0.2	-10.8	2.9	19.3	4.4	4.3	0.0492	2
4/16/2018 1:31	0.2	-11.1	3.5	19	4.4	4.3	0.0498	2

4/16/2018 1:32	0.2	-11.3	2.3	19.4	4.4	4.3	0.0405	2
4/16/2018 1:33	0.2	-11.1	2	19.3	4.4	4.3	0.0514	2.1
4/16/2018 1:34	0.2	-11.4	3.5	19	4.3	4.3	0.0386	2.3
4/16/2018 1:35	0.2	-10.6	3	19.1	4.3	4.4	0.0498	2.3
4/16/2018 1:36	0.2	-10.4	2.7	19.2	4.4	4.4	0.045	2.3
4/16/2018 1:37	0.1	-10.7	2.4	19.3	4.4	4.4	0.0386	2.4
4/16/2018 1:38	0.2	-10.1	2.9	19.2	4.5	4.4	0.0517	2.5
4/16/2018 1:39	0.2	-10.9	1.7	19.2	4.6	4.4	0.0359	2.7
4/16/2018 1:40	0.2	-11.3	2.6	19.5	4.6	4.3	0.0463	2.8
4/16/2018 1:41	0.2	-10.9	3.8	19.2	4.5	4.3	0.0421	2.9
4/16/2018 1:42	0.1	-10.4	3.9	19.2	4.5	4.3	0.0387	2.7
4/16/2018 1:43	0.1	-10.4	2.3	19.2	4.5	4.3	0.0491	2.5
4/16/2018 1:44	0.1	-10.8	2.7	19	4.5	4.3	0.0309	2.6
4/16/2018 1:45	0.1	-10.9	2	19.1	4.4	4.3	0.0511	2.7
4/16/2018 1:46	0.1	-10.9	1.9	19.4	4.5	4.3	0.0372	2.7
4/16/2018 1:47	0.1	-10.4	4.1	19.2	4.5	4.3	0.0488	2.8
4/16/2018 1:48	0	-11	1.5	19.2	4.5	4.3	0.0415	3
4/16/2018 1:49	0	-11	2.1	19.3	4.5	4.3	0.0332	3.1
4/16/2018 1:50	0.1	-10.6	2	19.2	4.5	4.3	0.0535	3.3
4/16/2018 1:51	0.1	-10.5	3.7	19.1	4.4	4.3	0.0339	3.4
4/16/2018 1:52	0.1	-10.9	1.3	19.2	4.4	4.3	0.048	3.4
4/16/2018 1:53	0	-10.3	1.9	19.3	4.5	4.3	0.0357	3.6
4/16/2018 1:54	0	-10.4	1.7	19.1	4.5	4.3	0.0393	3.6
4/16/2018 1:55	0	-10.4	2.2	19.2	4.4	4.3	0.0422	3.6
4/16/2018 1:56	0	-10.6	1.8	19.2	4.5	4.3	0.0315	3.7
4/16/2018 1:57	0.1	-10.7	3	19.3	4.4	4.3	0.0489	3.7
4/16/2018 1:58	0.1	-10.8	1.8	19.2	4.4	4.3	0.0343	3.8
4/16/2018 1:59	0	-10.6	2.8	19.2	4.5	4.3	0.0443	3.8
4/16/2018 2:00	0	-10.3	3.1	19.4	4.5	4.3	0.0414	3.9
4/16/2018 2:01	0	-10.4	3.9	19.2	4.4	4.3	0.0323	3.9
4/16/2018 2:02	0.1	-10.7	3.2	19.2	4.3	4.3	0.0469	3.9
4/16/2018 2:03	0.1	-10.3	3	18.9	4.3	4.3	0.0365	4
4/16/2018 2:04	0.1	-10.2	2	19.2	4.3	4.4	0.045	4.1
4/16/2018 2:05	0.1	-10	2.4	18.9	4.3	4.4	0.0505	3.9
4/16/2018 2:06	0.1	-9.6	1.9	19.1	4.3	4.4	0.0353	3.8
4/16/2018 2:07	0.1	-9.5	3.7	19.3	4.3	4.4	0.0489	3.9
4/16/2018 2:08	0.1	-10.1	1.4	19.5	4.3	4.4	0.0265	4
4/16/2018 2:09	0.1	-10.4	3	19.3	4.3	4.4	0.0387	4
4/16/2018 2:10	0.1	-10.6	2.8	19.3	4.3	4.4	0.0427	4
4/16/2018 2:11	0.1	-10.8	1.7	19.3	4.3	4.4	0.0384	4.1
4/16/2018 2:12	0.1	-10.5	1.7	19.3	4.3	4.4	0.0481	4.2
4/16/2018 2:13	0.1	-10.3	1	18.8	4.3	4.4	0.036	4.2
4/16/2018 2:14	0.1	-10	1.6	19.1	4.3	4.4	0.0486	4.2
4/16/2018 2:15	0.1	-10.8	3.1	19.3	4.3	4.4	0.0431	4.3
4/16/2018 2:16	0.1	-10.2	1.9	18.9	4.3	4.4	0.0362	4.3
4/16/2018 2:17	0.1	-11.2	2.1	19.1	4.3	4.4	0.0484	4.3
4/16/2018 2:18	0.1	-10.1	2.6	19.2	4.3	4.4	0.0351	4.3

4/16/2018 2:19	0.1	-10.3	2.6	19.2	4.3	4.3	0.0446	3.9
4/16/2018 2:20	0.1	-9.7	1.5	18.9	4.2	4.3	0.0381	3.6
4/16/2018 2:21	0.1	-9.1	2.2	18.9	4.3	4.3	0.0393	3.8
4/16/2018 2:22	0.2	-9.6	2.4	19.3	4.3	4.3	0.0449	3.8
4/16/2018 2:23	0.1	-9.9	2	19	4.3	4.3	0.0342	4
4/16/2018 2:24	0	-10.6	2.7	19.1	4.3	4.3	0.0566	4.1
4/16/2018 2:25	-0.1	-10.9	2.4	19.2	4.3	4.3	0.0425	4.2
4/16/2018 2:26	-0.1	-9.8	3.2	19.3	4.3	4.3	0.0467	4.2
4/16/2018 2:27	-0.1	-9.9	2.1	19.4	4.2	4.3	0.0404	4.2
4/16/2018 2:28	-0.1	-10.2	2.3	19.2	4.2	4.3	0.0366	4.2
4/16/2018 2:29	-0.1	-9.3	3	19.2	4.2	4.3	0.0421	4.2
4/16/2018 2:30	0	-10.2	3.3	19.1	4.2	4.3	0.033	4.2
4/16/2018 2:31	0	-10	2	19.1	4.2	4.3	0.0415	4.1
4/16/2018 2:32	0	-10.1	2.1	19	4.2	4.3	0.0437	4.1
4/16/2018 2:33	-0.1	-10	2	19.4	4.2	4.3	0.0362	4.1
4/16/2018 2:34	-0.1	-9.9	1.9	19	4.2	4.3	0.0415	4.1
4/16/2018 2:35	-0.1	-10.2	1.9	19	4.2	4.3	0.038	4.1
4/16/2018 2:36	-0.1	-9.7	2	19.1	4.2	4.3	0.0385	4
4/16/2018 2:37	0	-9.4	2.3	19.3	4.1	4.3	0.0407	4
4/16/2018 2:38	0	-10.1	1.6	19.1	4.2	4.3	0.0368	3.4
4/16/2018 2:39	0	-10.3	2	19.3	4.1	4.3	0.0424	3.1
4/16/2018 2:40	0	-9.3	3.2	19.2	4.2	4.3	0.0363	3.2
4/16/2018 2:41	0	-9.4	1.9	19.1	4.1	4.3	0.039	3.3
4/16/2018 2:42	0.1	-9.5	2.4	19.1	4.1	4.3	0.0389	3.3
4/16/2018 2:43	0.2	-9.6	2.9	19.4	4.2	4.3	0.037	3.5
4/16/2018 2:44	0.2	-10.3	1.6	19	4.2	4.3	0.0366	3.8
4/16/2018 2:45	0.2	-9.7	2.4	19.2	4.1	4.3	0.0291	3.9
4/16/2018 2:46	0.2	-9.4	2	19.3	4.1	4.3	0.0443	3.9
4/16/2018 2:47	0.2	-9	1.8	19.2	4	4.3	0.0393	3.9
4/16/2018 2:48	0.2	-9.7		19	4.1	4.3	0.0359	4
4/16/2018 2:49	0.2	-9.4		19.3	4	4.3	0.0448	4
4/16/2018 2:50	0.2	-9.3		19.3	4.1	4.3	0.033	4
4/16/2018 2:51	0.1	-9.5	8.3	19.1	4.1	4.3	0.0481	4
4/16/2018 2:52	0.1	-9.7		19.2	4.2	4.3	0.0308	4.1
4/16/2018 2:53	0.1	-9.4		19	4	4.3	0.0516	4.1
4/16/2018 2:54	0.2	-9.6	3.1	19.1	4.1	4.3	0.0294	4
4/16/2018 2:55	0.2	-10.2	1.8	19.3	4.1	4.3	0.0304	4
4/16/2018 2:56	0.2	-9.9	3.7	19.1	4.1	4.3	0.0476	3.9
4/16/2018 2:57	0.1	-9.5	1.5	19.3	4.1	4.3	0.0249	4
4/16/2018 2:58	0.1	-9.7	2.9	19.1	4	4.2	0.0471	4
4/16/2018 2:59	0.2	-9.3	2.9	19.1	4	4.3	0.0361	4.1
4/16/2018 3:00	0.1	-10.1	2.1	19	4.1	4.3	0.038	4
4/16/2018 3:01	0.2	-10	2	19.3	4.1	4.3	0.0464	4.1
4/16/2018 3:02	0.1	-9.9	1.8	19.1	4.1	4.3	0.0278	4.1
4/16/2018 3:03	0.2	-8.8	3.6	19.1	4.1	4.3	0.0493	4.1
4/16/2018 3:04	0.2	-9.4	2.1	19.1	4.1	4.2	0.0267	4.1
4/16/2018 3:05	0.2	-8.7	1.9	19	4	4.2	0.0446	4.1

4/16/2018 3:06	0.2	-9.3	1.5	19.1	4.1	4.2	0.0438	4.1
4/16/2018 3:07	0.2	-9.2	1.8	19.1	4.1	4.2	0.0329	4.2
4/16/2018 3:08	0.2	-9.1	2.3	19.1	4.1	4.2	0.0443	4.2
4/16/2018 3:09	0.2	-9.8	2.2	19.1	4	4.2	0.0317	4.3
4/16/2018 3:10	0.2	-9.3	1.7	19.2	4.1	4.2	0.0454	4.3
4/16/2018 3:11	0.2	-8.8	3.1	19.3	4	4.2	0.0361	4.2
4/16/2018 3:12	0.3	-9.5	1.2	19.1	4.1	4.2	0.0353	4.2
4/16/2018 3:13	0.3	-9.3	1.1	19.1	4	4.2	0.0406	4.2
4/16/2018 3:14	0.3	-9.2	1.7	19.2	4.1	4.2	0.0251	4.1
4/16/2018 3:15	0.3	-9.3	1.9	19.3	4.1	4.2	0.0351	4.2
4/16/2018 3:16	0.3	-9.1	2.5	19	4.1	4.2	0.0417	4.4
4/16/2018 3:17	0.3	-10	2.4	19.3	4	4.2	0.0327	4.4
4/16/2018 3:18	0.3	-9.2	3.7	19.4	4	4.2	0.042	4.4
4/16/2018 3:19	0.3	-9.2	1.4	19.6	4	4.2	0.0317	4.5
4/16/2018 3:20	0.2	-8.7	3	19.2	4	4.2	0.0471	4.5
4/16/2018 3:21	0.2	-10	1.6	19.3	4	4.2	0.0373	4.5
4/16/2018 3:22	0.3	-9.6	1.3	19.1	4	4.2	0.0347	4.5
4/16/2018 3:23	0.2	-9.2	2.7	19.3	4	4.2	0.0437	4.5
4/16/2018 3:24	0.2	-8.9	2.6	19.2	4	4.2	0.0254	4.5
4/16/2018 3:25	0.2	-9	2.6	19.2	3.9	4.2	0.0447	4.5
4/16/2018 3:26	0.2	-9.4	2.1	19.1	3.9	4.2	0.0319	4.5
4/16/2018 3:27	0.3	-9.1	3.2	19.2	4	4.2	0.0396	4.5
4/16/2018 3:28	0.3	-9	2.2	19	4	4.2	0.0396	4.5
4/16/2018 3:29	0.2	-9.2	1.3	19.4	4	4.2	0.0357	4.5
4/16/2018 3:30	0.2	-8.9	3.2	19.1	4	4.2	0.0441	4.5
4/16/2018 3:31	0.2	-9.5	3.4	19.2	3.9	4.2	0.0327	4.6
4/16/2018 3:32	0.2	-9.2	1.9	19	3.9	4.2	0.0402	4.5
4/16/2018 3:33	0.2	-9.2	3.6	19.2	3.9	4.2	0.0376	4.5
4/16/2018 3:34	0.2	-8.4	3.1	19.3	3.9	4.2	0.048	4.4
4/16/2018 3:35	0.2	-8.5	1.7	19.4	3.9	4.2	0.0438	4.5
4/16/2018 3:36	0.2	-8.7	2.8	19.1	3.9	4.2	0.033	4.6
4/16/2018 3:37	0.2	-9.2	2.2	19	4	4.2	0.0435	4.7
4/16/2018 3:38	0.2	-8.5	2	19.4	3.9	4.2	0.0281	4.8
4/16/2018 3:39	0.2	-8.8	2.1	19.1	3.9	4.2	0.0376	4.7
4/16/2018 3:40	0.2	-8.3	3	19.3	3.9	4.2	0.0311	4.8
4/16/2018 3:41	0.2	-8.3	2	19.1	3.9	4.2	0.0385	4.8
4/16/2018 3:42	0.2	-8.5	1.5	19.4	3.9	4.2	0.0428	4.7
4/16/2018 3:43	0.2	-8.8	3.3	19.3	3.9	4.2	0.0347	4.7
4/16/2018 3:44	0.2	-8.5	3.5	19	3.9	4.2	0.0537	4.6
4/16/2018 3:45	0.2	-8.9	3.3	19.2	3.9	4.2	0.025	4.7
4/16/2018 3:46	0.2	-9.4	2.7	19.4	3.9	4.2	0.0425	4.8
4/16/2018 3:47	0.2	-8.4	1.9	19.1	3.9	4.2	0.0352	4.8
4/16/2018 3:48	0.3	-8.8	2.1	19.2	3.9	4.2	0.0408	4.7
4/16/2018 3:49	0.3	-9.1	2.4	19.4	3.9	4.1	0.0436	4.7
4/16/2018 3:50	0.2	-8.5	1.8	19.2	3.9	4.1	0.03	4.8
4/16/2018 3:51	0.2	-9.3	3.1	19.5	3.9	4.1	0.0454	4.8
4/16/2018 3:52	0.2	-8.9	2.4	19.4	3.8	4.1	0.0277	4.9

4/16/2018 3:53	0.2	-8.8	3.2	19.3	3.9	4.1	0.0348	4.8
4/16/2018 3:54	0.2	-8.3	2.4	19	3.9	4.1	0.0401	4.9
4/16/2018 3:55	0.2	-8.5	1.9	19	3.9	4.1	0.0282	5
4/16/2018 3:56	0.2	-8.7	1.3	19.1	3.9	4.1	0.043	5
4/16/2018 3:57	0.2	-8.3	3.5	19.4	3.9	4.2	0.0307	5.1
4/16/2018 3:58	0.2	-8.1	1.5	19.3	3.9	4.2	0.0412	5
4/16/2018 3:59	0.2	-8.6	2.5	19	3.8	4.2	0.0356	5
4/16/2018 4:00	0.2	-8.8	2.1	19.1	3.9	4.2	0.0346	5.1
4/16/2018 4:01	0.2	-8.4	2.1	19.1	3.8	4.1	0.0435	5.1
4/16/2018 4:02	0.2	-8.4	1.3	19.3	3.8	4.1	0.0317	4.8
4/16/2018 4:03	0.2	-8	2.2	19.1	3.8	4.2	0.0446	5
4/16/2018 4:04	0.2	-8.9	2.6	19	3.8	4.1	0.0267	5.1
4/16/2018 4:05	0.2	-8.9	2.5	19.1	3.8	4.1	0.04	5.1
4/16/2018 4:06	0.2	-8.4	1.7	19.2	3.9	4.1	0.0402	5
4/16/2018 4:07	0.1	-9.1	0.8	19.2	3.9	4.1	0.0309	4.9
4/16/2018 4:08	0.1	-8.6	2.8	19.5	3.9	4.1	0.0454	4.9
4/16/2018 4:09	0.1	-8.4	0.8	19.3	3.9	4.1	0.0206	5
4/16/2018 4:10	0.1	-8.6	3	19.2	3.9	4.1	0.0416	5
4/16/2018 4:11	0.1	-8.3	2.5	19.3	3.8	4.1	0.0238	5.1
4/16/2018 4:12	0.1	-8.2	2.9	19.2	3.8	4.1	0.0302	5.1
4/16/2018 4:13	0.2	-8.8	2.3	19.3	3.8	4.1	0.0366	5.1
4/16/2018 4:14	0.2	-7.9	4.5	19.3	3.8	4.1	0.0319	5
4/16/2018 4:15	0.2	-8.4	1.7	19.4	3.8	4.1	0.0456	4
4/16/2018 4:16	0.1	-8.4	2.5	19.5	3.9	4.1	0.025	4
4/16/2018 4:17	0.1	-8.7	1.7	19.3	3.9	4.1	0.0407	4.3
4/16/2018 4:18	0.1	-8.4	2.8	19.5	3.9	4.1	0.0307	4.5
4/16/2018 4:19	0.1	-8.2	3.2	19.3	3.8	4.1	0.0382	4.7
4/16/2018 4:20	0.2	-8.6	2.2	19.4	3.8	4.1	0.0385	4.8
4/16/2018 4:21	0.1	-8.3	2.9	19.2	3.9	4.1	0.0208	4.8
4/16/2018 4:22	0.1	-8.9	3	19.3	3.8	4.1	0.0398	4.8
4/16/2018 4:23	0.1	-7.9	2.9	19.2	3.8	4.1	0.0259	4.9
4/16/2018 4:24	0.2	-8.5	2.3	19.5	3.9	4.1	0.0392	4.9
4/16/2018 4:25	0.2	-7.7	2.7	19.4	3.8	4	0.0363	5
4/16/2018 4:26	0.2	-8.5	2.2	19.3	3.9	4	0.0309	5
4/16/2018 4:27	0.2	-8.6	2.7	19.3	3.9	4	0.0357	4.9
4/16/2018 4:28	0.3	-7.9	2.9	19.3	3.9	4	0.0229	4.9
4/16/2018 4:29	0.3	-8.4	2.7	19.4	3.8	4	0.0334	5
4/16/2018 4:30	0.3	-8.7	2.2	19.3	3.8	4	0.0251	4.9
4/16/2018 4:31	0.2	-8.8	1.5	19.4	3.8	4	0.0309	4.5
4/16/2018 4:32	0.2	-8.3	2.9	19.2	3.9	3.9	0.0365	4.4
4/16/2018 4:33	0.2	-8.8	3	19.6	3.8	4	0.0273	4.4
4/16/2018 4:34	0.2	-8	1.8	19.4	3.8	4	0.0365	4.3
4/16/2018 4:35	0.2	-8.2	2	19.1	3.8	4	0.0224	4.2
4/16/2018 4:36	0.2	-7.9	1.7	19.8	3.8	4	0.0402	4.1
4/16/2018 4:37	0.2	-7.5	2.1	19.4	3.9	4	0.0319	4.1
4/16/2018 4:38	0.2	-8.3	2.3	19.4	3.9	4	0.0318	4.2
4/16/2018 4:39	0.2	-7.5	3.2	19.1	3.8	4	0.0358	4.3

4/16/2018 4:40	0.2	-7.7	3.5	19.4	3.9	4	0.0273	4.4
4/16/2018 4:41	0.1	-7.8	2.3	19.6	3.9	4	0.0326	4.4
4/16/2018 4:42	0.1	-8.5	2.1	19.3	3.8	4	0.0216	4.4
4/16/2018 4:43	0.1	-8	0.7	19.5	3.8	4	0.0286	4.4
4/16/2018 4:44	0.1	-8.2	1.7	19.2	3.8	4	0.03	4.5
4/16/2018 4:45	0.1	-7.4	0.8	19.5	3.9	4	0.029	4.5
4/16/2018 4:46	0.1	-8.1	2.2	19.5	3.8	4	0.0356	4.5
4/16/2018 4:47	0.1	-8.1	2.6	19.3	3.8	4	0.0153	4.5
4/16/2018 4:48	0.1	-7.7	2.8	19.6	3.9	3.9	0.0326	4.5
4/16/2018 4:49	0.1	-7.6	2	19.4	3.9	3.9	0.0219	4.5
4/16/2018 4:50	0.1	-7.4	0.7	19.4	3.9	3.9	0.0262	4.5
4/16/2018 4:51	0.2	-8	3.3	19.4	3.8	3.9	0.0379	4.5
4/16/2018 4:52	0.2	-8.3	3.6	19.7	3.9	3.9	0.02	4.6
4/16/2018 4:53	0.2	-8.1	4.3	19.1	3.9	3.9	0.0306	4.7
4/16/2018 4:54	0.3	-7.8	2.1	19.4	3.9	3.9	0.021	4.8
4/16/2018 4:55	0.3	-7.8	1.9	19.6	3.9	3.9	0.0374	4.8
4/16/2018 4:56	0.3	-7.9	2.7	19.6	3.9	3.9	0.0293	4.9
4/16/2018 4:57	0.3	-8	2.9	19.7	3.9	3.9	0.0253	4.8
4/16/2018 4:58	0.2	-8	2.2	19.7	3.9	3.9	0.0338	4.8
4/16/2018 4:59	0.2	-7.6	2.5	19.6	4	3.9	0.0174	4.7
4/16/2018 5:00	0.2	-8.3	2	19.3	3.9	3.8	0.035	4.7
4/16/2018 5:01	0.2	-7.4	2.3	19.3	3.9	3.8	0.0248	4.8
4/16/2018 5:02	0.2	-7.3	2	19.8	3.9	3.8	0.0278	4.6
4/16/2018 5:03	0.2	-7.9	2.1	19.4	3.9	3.8	0.0259	4.5
4/16/2018 5:04	0.2	-7.8	3.9	19.7	3.9	3.8	0.0198	3.9
4/16/2018 5:05	0.2	-7.8	3.1	19.6	4	3.8	0.0322	4.2
4/16/2018 5:06	0.2	-8	2.3	19.6	4	3.8	0.0229	4.5
4/16/2018 5:07	0.2	-7.9	2.6	19.4	4	3.8	0.0328	4.6
4/16/2018 5:08	0.2	-7.8	3.6	19.6	4	3.8	0.0219	4.8
4/16/2018 5:09	0.2	-8	2	19.5	4	3.8	0.0238	4.8
4/16/2018 5:10	0.2	-7.8	2.1	19.5	4	3.8	0.0327	4.8
4/16/2018 5:11	0.2	-8.4	2.6	19.6	4	3.8	0.0208	4.8
4/16/2018 5:12	0.2	-8.5	3.5	19.4	4.1	3.8	0.0329	4.8
4/16/2018 5:13	0.2	-7.9	3.1	19.4	4	3.8	0.0182	4.9
4/16/2018 5:14	0.1	-7.1	3.1	19.5	4.1	3.8	0.0311	4.9
4/16/2018 5:15	0.1	-7.7	2.4	19.5	4.1	3.8	0.0244	4.9
4/16/2018 5:16	0.1	-7.5	2.9	19.8	4.1	3.8	0.0269	4.8
4/16/2018 5:17	0.1	-7.8	2.3	19.5	4.1	3.8	0.0319	4.8
4/16/2018 5:18	0.2	-7.5	2	19.6	4.2	3.8	0.0226	4.9
4/16/2018 5:19	0.2	-7.7	1.4	19.6	4.2	3.8	0.0375	4.8
4/16/2018 5:20	0.2	-8.2	3.3	19.6	4.2	3.8	0.0216	4.8
4/16/2018 5:21	0.2	-8	2	19.5	4.2	3.7	0.0347	4.8
4/16/2018 5:22	0.1	-7.4	2.4	19.7	4.2	3.7	0.0347	4.8
4/16/2018 5:23	0.1	-7.4	2.7	19.5	4.2	3.7	0.0163	4.8
4/16/2018 5:24	0.1	-8	2.8	19.6	4.2	3.7	0.0226	4.8
4/16/2018 5:25	0.1	-8	1.8	19.8	4.3	3.7	0.0119	4.9
4/16/2018 5:26	0.1	-7.7	4	20.1	4.3	3.7	0.0273	4.9

4/16/2018 5:27	0.1	-7.8	2.7	19.9	4.3	3.7	0.02	4.9
4/16/2018 5:28	0.1	-7.5	2.8	20	4.2	3.7	0.0248	4.9
4/16/2018 5:29	0.1	-6.8	2.9	19.9	4.3	3.7	0.0192	4.8
4/16/2018 5:30	0.1	-6.8	2.7	20	4.3	3.7	0.0244	4.7
4/16/2018 5:31	0.1	-7.7	2	19.8	4.4	3.7	0.0272	4.7
4/16/2018 5:32	0.1	-7.6	2.6	20.1	4.4	3.6	0.0209	4.7
4/16/2018 5:33	0.1	-7.3	2.7	20.4	4.5	3.6	0.0236	4.7
4/16/2018 5:34	0.1	-7.7	1.6	20.2	4.5	3.6	0.0195	4.8
4/16/2018 5:35	0	-8.1	2.3	20.4	4.6	3.6	0.0148	4.7
4/16/2018 5:36	0	-7.5	2	20.4	4.5	3.5	0.0252	4.6
4/16/2018 5:37	0.1	-7.6		20.5	4.6	3.5	0.0029	4.6
4/16/2018 5:38	0.1	-7.8		20.1	4.5	3.5	0.0257	4.5
4/16/2018 5:39	0.1	-7.3	9.4	20.3	4.5	3.5	0.021	4.6
4/16/2018 5:40	0	-6.9		20.1	4.4	3.5	0.0257	4.6
4/16/2018 5:41	0	-7.8	1.9	20	4.4	3.5	0.0171	4.5
4/16/2018 5:42	0	-7.7	6.3	20	4.4	3.5	0.0106	4.5
4/16/2018 5:43	0.1	-7.6		20.1	4.4	3.5	0.0227	4.5
4/16/2018 5:44	0	-7.5	5	20	4.4	3.5	0.0158	4.6
4/16/2018 5:45	0	-7.4		20.2	4.5	3.5	0.0199	4.4
4/16/2018 5:46	0	-7.5	3	19.9	4.5	3.5	0.0215	4.6
4/16/2018 5:47	0	-7.5	2.5	19.7	4.4	3.5	0.0185	4.6
4/16/2018 5:48	0.1	-7.8	2.9	19.8	4.4	3.5	0.0237	4.6
4/16/2018 5:49	0.1	-7.9	3.1	20.1	4.4	3.5	0.0118	4.6
4/16/2018 5:50	0.1	-7.7	1.4	19.9	4.4	3.4	0.0197	3.9
4/16/2018 5:51	0.1	-7.1	1.4	20.1	4.4	3.4	0.0091	2.7
4/16/2018 5:52	0.1	-7.4	3.2	19.7	4.4	3.4	0.0237	3
4/16/2018 5:53	0.1	-7.7	2	20	4.4	3.4	0.0138	3.6
4/16/2018 5:54	0.1	-8.3	2.4	19.8	4.4	3.4	0.0171	3.9
4/16/2018 5:55	0.2	-7.8	2.7	19.9	4.4	3.4	0.023	4.2
4/16/2018 5:56	0.2	-7.5	1.7	19.6	4.4	3.4	0.008	4.3
4/16/2018 5:57	0.2	-7.3	2	19.4	4.4	3.4	0.0225	4.5
4/16/2018 5:58	0.1	-7.7	3.8	20	4.5	3.4	0.0019	4.6
4/16/2018 5:59	0.2	-6.9	2.2	20.1	4.5	3.4	0.0219	4.7
4/16/2018 6:00	0.2	-7.2	1.3	20	4.4	3.4	0.0079	4.6
4/16/2018 6:01	0.2	-8	2	19.8	4.4	3.4	0.0155	4.5
4/16/2018 6:02	0.2	-7.7	3	20.1	4.4	3.4	0.026	4.4
4/16/2018 6:03	0.2	-7.7	2.9	19.8	4.4	3.4	0.0097	4.5
4/16/2018 6:04	0.2	-7.2	3.2	19.9	4.4	3.4	0.019	4.5
4/16/2018 6:05	0.1	-7.6	3.7	19.8	4.4	3.4	0.0069	4.5
4/16/2018 6:06	0.1	-7	2.7	19.8	4.4	3.4	0.0197	4.5
4/16/2018 6:07	0.1	-7.5	2.1	19.7	4.4	3.4	0.0173	4.7
4/16/2018 6:08	0.2	-7.4	3.3	19.7	4.4	3.4	0.0143	4.6
4/16/2018 6:09	0.2	-7.6	3.1	19.7	4.4	3.4	0.0118	4.7
4/16/2018 6:10	0.1	-7.2	1.1	19.6	4.4	3.4	0.0126	4.7
4/16/2018 6:11	0.1	-7.7	1.7	19.6	4.4	3.4	0.027	4.8
4/16/2018 6:12	0.1	-7.4	3.3	19.5	4.3	3.4	0.0138	4.7
4/16/2018 6:13	0.1	-7.5	2.3	19.7	4.3	3.5	0.0186	4.7

4/16/2018 6:14	0.2	-7	1.8	19.5	4.4	3.5	0.0134	4.6
4/16/2018 6:15	0.1	-7.3	2.6	19.6	4.3	3.5	0.021	4.1
4/16/2018 6:16	0.1	-7.6	2.3	19.7	4.3	3.5	0.0182	3.9
4/16/2018 6:17	0.1	-8	2.3	19.5	4.3	3.5	0.0123	3.6
4/16/2018 6:18	0.2	-7.3	3.3	19.8	4.3	3.5	0.0279	3
4/16/2018 6:19	0.2	-7	2.3	19.7	4.3	3.5	0.0054	3.1
4/16/2018 6:20	0.2	-7.6	2.7	19.5	4.3	3.5	0.0289	3.3
4/16/2018 6:21	0.2	-7.1	2.2	19.5	4.3	3.5	0.007	3.5
4/16/2018 6:22	0.2	-7.3	3	19.5	4.3	3.5	0.0242	3.7
4/16/2018 6:23	0.2	-7.9	2.7	19.6	4.3	3.5	0.0224	3.9
4/16/2018 6:24	0.2	-7.2	2.3	19.6	4.3	3.5	0.0108	4
4/16/2018 6:25	0.2	-7.9	1.8	19.9	4.3	3.5	0.0221	4.1
4/16/2018 6:26	0.2	-7.1	2	19.7	4.2	3.5	0.0088	4.2
4/16/2018 6:27	0.2	-7.1	3.6	19.7	4.3	3.5	0.0199	4.3
4/16/2018 6:28	0.2	-6.8	1.1	19.7	4.3	3.5	0.0113	4.3
4/16/2018 6:29	0.2	-7.2	1.7	19.8	4.2	3.5	0.0104	4.4
4/16/2018 6:30	0.2	-7.1	2.2	19.8	4.2	3.5	0.0197	4.4
4/16/2018 6:31	0.2	-7.4	2.1	19.6	4.2	3.5	0.009	4.3
4/16/2018 6:32	0.2	-7.4	3	19.7	4.2	3.5	0.0119	4.2
4/16/2018 6:33	0.2	-7.3	2.3	19.8	4.2	3.5	0.01	4.3
4/16/2018 6:34	0.2	-7.1	3	19.7	4.2	3.5	0.0116	4.3
4/16/2018 6:35	0.2	-7.2	2.8	19.7	4.2	3.5	0.0207	4.3
4/16/2018 6:36	0.2	-7.7	3	19.9	4.2	3.4	0.0035	4.3
4/16/2018 6:37	0.2	-7.4	0.7	19.7	4.2	3.4	0.0223	4.3
4/16/2018 6:38	0.2	-8.8	2.4	19.8	4.2	3.4	0.0069	4.3
4/16/2018 6:39	0.2	-7.6	2	19.7	4.2	3.4	0.0114	4.3
4/16/2018 6:40	0.2	-6.7	4.2	19.9	4.2	3.4	0.0215	4.1
4/16/2018 6:41	0.2	-6.9	2.2	19.7	4.2	3.4	0.0046	4.2
4/16/2018 6:42	0.2	-7	3.7	19.8	4.2	3.4	0.0144	4.4
4/16/2018 6:43	0.2	-7.5	2.2	19.8	4.2	3.4	0.01	4.4
4/16/2018 6:44	0.2	-7.4	3.1	19.7	4.2	3.4	0.0101	4.3
4/16/2018 6:45	0.2	-7.2	2.8	19.7	4.2	3.4	0.0203	4.3
4/16/2018 6:46	0.2	-7.3	2.4	19.7	4.2	3.4	0.0147	4.5
4/16/2018 6:47	0.2	-7.6	2.5	19.5	4.2	3.5	0.0191	4.5
4/16/2018 6:48	0.2	-7.6	1.9	19.4	4.2	3.5	0.0021	4.5
4/16/2018 6:49	0.2	-7.9	2.1	19.5	4.2	3.5	0.0146	4.5
4/16/2018 6:50	0.2	-7.2	2.6	19.5	4.2	3.5	0.0107	4.5
4/16/2018 6:51	0.2	-7.1	2.6	19.6	4.2	3.5	0.0034	4.4
4/16/2018 6:52	0.2	-7.8	2.8	19.6	4.1	3.5	0.0189	4.6
4/16/2018 6:53	0.2	-7.2	2.4	19.6	4.2	3.5	-0.0031	4.6
4/16/2018 6:54	0.2	-7.1	2.8	19.6	4.2	3.5	0.0209	4.7
4/16/2018 6:55	0.2	-7	1.4	19.6	4.1	3.5	0.0083	4.7
4/16/2018 6:56	0.2	-7.7	1.5	19.9	4.2	3.5	0.0108	4.7
4/16/2018 6:57	0.2	-7.8	2.8	19.6	4.2	3.5	0.0194	4.6
4/16/2018 6:58	0.2	-8.1	1.9	19.4	4.2	3.5	0.0058	4.7
4/16/2018 6:59	0.2	-7.1	4.6	19.8	4.1	3.5	0.0223	4.5
4/16/2018 7:00	0.2	-7.4	2.2	19.8	4.1	3.5	0.003	4.6

4/16/2018 7:01	0.2	-7	3.1	19.6	4.1	3.5	0.0161	4.4
4/16/2018 7:02	0.2	-7	2.1	19.5	4.2	3.5	0.015	4.4
4/16/2018 7:03	0.3	-7	2.4	19.6	4.1	3.5	0.0054	4.3
4/16/2018 7:04	0.3	-7.8	2.2	19.5	4.1	3.5	0.0181	4.3
4/16/2018 7:05	0.3	-7.7	2.2	19.7	4.2	3.5	0.0062	4.3
4/16/2018 7:06	0.2	-7.1	2	19.6	4.2	3.5	0.0234	4.4
4/16/2018 7:07	0.2	-7.2	2.6	19.4	4.2	3.5	0.013	4.5
4/16/2018 7:08	0.2	-7.5	2.1	19.7	4.2	3.5	0.0027	3.9
4/16/2018 7:09	0.2	-7.2	2.4	19.6	4.2	3.5	0.0164	4.1
4/16/2018 7:10	0.2	-7.6	1.6	19.6	4.2	3.5	-0.0003	4.1
4/16/2018 7:11	0.2	-7	2.7	19.4	4.2	3.5	0.0129	4
4/16/2018 7:12	0.2	-7.3	2.6	19.7	4.2	3.5	0.0034	4.1
4/16/2018 7:13	0.2	-7.1	3.6	19.7	4.2	3.5	0.0113	4
4/16/2018 7:14	0.2	-7.7	2.7	19.5	4.1	3.5	0.0174	4
4/16/2018 7:15	0.2	-7.5	3.7	19.6	4.1	3.5	0.0053	3.9
4/16/2018 7:16	0.2	-7.4	4	19.4	4.2	3.5	0.0156	3.9
4/16/2018 7:17	0.3	-7.7	1.6	19.3	4.1	3.5	0.0046	3.9
4/16/2018 7:18	0.3	-7.5	2.2	19.5	4.1	3.5	0.0211	3.9
4/16/2018 7:19	0.3	-7.2	2.6	19.4	4.1	3.5	0.0146	3.9
4/16/2018 7:20	0.3	-7	1.6	19.7	4.1	3.5	0.0096	3.9
4/16/2018 7:21	0.2	-7.4	2.7	19.4	4.1	3.5	0.0156	3.9
4/16/2018 7:22	0.2	-7.5	1.3	19.3	4.1	3.5	0.009	3.9
4/16/2018 7:23	0.3	-7.5	2.4	19.7	4.1	3.5	0.0175	3.9
4/16/2018 7:24	0.3	-7.7	2	19.6	4.1	3.5	0.0041	4
4/16/2018 7:25	0.3	-7.6	2	19.6	4.1	3.5	0.0167	3.7
4/16/2018 7:26	0.2	-7.2	3	19.4	4.1	3.5	0.0158	2.7
4/16/2018 7:27	0.3	-7.1	3.2	19.5	4.1	3.5	0.0071	2.4
4/16/2018 7:28	0.3	-7.5	2.4	19.4	4.1	3.6	0.0168	2.4
4/16/2018 7:29	0.2	-7	2	19.5	4.1	3.6	0.0056	2.5
4/16/2018 7:30	0.2	-7	2.2	19.4	4.1	3.6	0.0176	2.5
4/16/2018 7:31	0.2	-131.4	2.7	19.7	4.1	3.6	0.0046	2.6
4/16/2018 7:32	0.3	-16.1	2.4	19.5	4.1	3.6	0.0187	2.4
4/16/2018 7:33	0.5	-1.7	4.2	20.9	4.1	3.6	0.02	1.7
4/16/2018 7:34	0.6	-0.4	2.7	19	4.1	3.6	0.0086	1.7
4/16/2018 7:35	0.7	-0.7	1.5	19.1	4.1	3.6	0.0154	1.7
4/16/2018 7:36	0.7	0.6	2.9	19.5	4.2	3.6	0.0059	1.7
4/16/2018 7:37	0.7	0.4	1.4	19.2	4.1	3.6	0.0131	1.8
4/16/2018 7:38	0.6	-0.2	3.2	19.6	4.1	3.6	0.0088	1.7
4/16/2018 7:39	0.6	0.3	0.9	19.2	4.2	3.6	0.016	1.8
4/16/2018 7:40	0.6	0.5	1.6	19.5	4.1	3.6	0.0211	1.8
4/16/2018 7:41	0.6	0	2.4	19.8	4.1	3.6	0.0069	1.8
4/16/2018 7:42	0.6	0.7	2.9	19.6	4.2	3.6	0.0232	1.9
4/16/2018 7:43	0.6	-0.2	2.1	19.4	4.2	3.6	0.0172	1.8
4/16/2018 7:44	0.6	-0.1	2.3	19.6	4.2	3.6	-0.01	1.8
4/16/2018 7:45	0.6	0.6	2.6	19.7	4.3	3.6	0.0261	1.8
4/16/2018 7:46	0.6	1	3.1	19.1	4.2	3.6	0.0025	1.8
4/16/2018 7:47	0.6	0.7	3.6	19.5	4.3	3.6	0.0209	1.8

4/16/2018 7:48	0.6	0.8	3.5	19.7	4.3	3.6	0.0119	1.9
4/16/2018 7:49	0.6	0.6	1.8	19.3	4.3	3.6	0.0152	1.9
4/16/2018 7:50	0.6	0.4	3	19.3	4.4	3.6	0.0177	1.9
4/16/2018 7:51	0.6	0.5	1.7	19.5	4.3	3.6	0.0124	1.9
4/16/2018 7:52	0.6	-0.4	2.7	19.2	4.4	3.6	0.0204	2
4/16/2018 7:53	0.6	0.8	1.7	19.2	4.4	3.6	0.0109	2
4/16/2018 7:54	0.7	0.4	1.5	19.4	4.4	3.6	0.0125	2
4/16/2018 7:55	0.7	0.4	3.2	19.5	4.4	3.6	0.0202	2
4/16/2018 7:56	0.6	0.9	2.6	19.4	4.4	3.6	0.0043	2.1
4/16/2018 7:57	0.6	0.7	2.2	19.4	4.4	3.6	0.0236	2.1
4/16/2018 7:58	0.6	0.7	2	19.4	4.4	3.6	0.0118	2.1
4/16/2018 7:59	0.6	1.2	3.4	19.3	4.4	3.6	0.0152	2.1
4/16/2018 8:00	0.6	1.1	2.4	19.4	4.5	3.6	0.0227	2.1
4/16/2018 8:01	0.6	1	2.9	19.7	4.4	3.6	0.0117	2.1
4/16/2018 8:02	0.6	1	2.2	20.6	13.2	11.2	0.0729	2.1
4/16/2018 8:03	0.4	0	2.7	20.9	21.6	71.1	0.1758	2.1
4/16/2018 8:04	0.3	0.1	1.9	21.9	31.2	121.7	0.4299	2
4/16/2018 8:05	0.2	0.7	2.2	23.3	37.7	144.8	0.7122	1.8
4/16/2018 8:06	0.2	0	3.8	25.6	40.9	157.7	1.1179	1.9
4/16/2018 8:07	0.2	-0.2	3.3	29.1	40.9	167.1	1.6968	2
4/16/2018 8:08	0.2	0.7	3	30.9	43.2	167.4	2.1313	2.1
4/16/2018 8:09	0.2	-0.2	3.8	31.1	44.7	166.8	2.3557	2.2
4/16/2018 8:10	0.2	0.5	3.9	31.8	43	158.3	2.7775	2.3
4/16/2018 8:11	0.2	0.2	2.4	31.7	43.6	163	3.1293	2.3
4/16/2018 8:12	0.1	0.2	3.4	30.7	41.8	158.2	3.1533	2.4
4/16/2018 8:13	0.1	0.4	2.3	30.9	41.2	158.2	3.4235	2.5
4/16/2018 8:14	0.1	-0.3	2.4	29.9	39.8	150.7	3.4691	2.5
4/16/2018 8:15	0.1	-0.1	1.8	30	39	149.2	3.521	2.6
4/16/2018 8:16	0.1	0.4	2.6	28.6	38	148	3.5038	2.6
4/16/2018 8:17	0.1	0.1	3.5	28.6	36.8	142.2	3.524	2.6
4/16/2018 8:18	0.1	0	2.6	28	36.1	140.4	3.5002	2.7
4/16/2018 8:19	0.1	-0.1	1.8	27.5	34.7	135.5	3.4615	2.7
4/16/2018 8:20	0.1	0.6	3.5	27.5	34.9	139.2	3.5557	2.7
4/16/2018 8:21	0.1	0.5	1.8	27.7	35.3	139.5	3.6496	2.7
4/16/2018 8:22	0.1	0.6	3.3	26.9	33.7	133.8	3.4815	2.8
4/16/2018 8:23	0.1	-0.5	3.1	26.4	33.1	132.9	3.4989	2.9
4/16/2018 8:24	0.2	0.5	3	26	31.9	127.6	3.4057	2.9
4/16/2018 8:25	0.2	0.7	2.6	26.4	32.9	134	3.5833	3
4/16/2018 8:26	0.2	0.7	1.7	25.8	31.6	129.7	3.4373	2.9
4/16/2018 8:27	0.2	0.5	1	25.4	30.6	124.8	3.3383	3
4/16/2018 8:28	0.3	0.2	2.3	25.4	30.5	125.4	3.4243	3.1
4/16/2018 8:29	0.3	-0.3	2.3	24.5	29.4	119.6	3.2408	3.2
4/16/2018 8:30	0.3	-0.1	3.4	24.5	28.2	118.2	3.2067	3.3
4/16/2018 8:31	0.3	0.1	3.2	24.4	28.4	120.6	3.2548	3.2
4/16/2018 8:32	0.3	0	3.7	24.7	29.1	122.6	3.413	3.2
4/16/2018 8:33	0.2	-0.2	4.1	24.1	28.2	118	3.2723	3.3
4/16/2018 8:34	0.2	0.2	2.9	23.9	27.4	112.7	3.186	3.5

4/16/2018 8:35	0.2	0.7	2.8	24.1	27.3	115	3.2716	3.5
4/16/2018 8:36	0.2	-0.1	2.7	23.9	26.8	113.8	3.2018	3.6
4/16/2018 8:37	0.2	0.7	2.8	23.7	26.9	114.3	3.2243	3.5
4/16/2018 8:38	0.2	0.5	3.6	23.7	26.1	113.8	3.1513	3.6
4/16/2018 8:39	0.3	0.9	3.4	23.7	26.6	116.3	3.2361	3.6
4/16/2018 8:40	0.3	0.6	2.6	23.6	26.2	116	3.2177	3.6
4/16/2018 8:41	0.2	0.2	3.1	23.4	26.3	115.9	3.2557	3.5
4/16/2018 8:42	0.2	-0.2	3.6	22.9	25.4	115.3	3.129	3.5
4/16/2018 8:43	0.2	0	4.7	23.3	24.9	109.6	3.0892	3.6
4/16/2018 8:44	0.2	0.9	3.1	22.9	24.4	108.3	3.0003	3.6
4/16/2018 8:45	0.2	0.4	2.2	22.7	23.6	103	2.8955	3.7
4/16/2018 8:46	0.2	0.9	2.4	22.7	23.7	105.2	3.0059	3.7
4/16/2018 8:47	0.2	0.4	3.7	22.5	23.9	106.3	3.0051	3.7
4/16/2018 8:48	0.2	0.2	1.9	22.7	23.1	101.9	2.9192	3.6
4/16/2018 8:49	0.2	1	3.2	22.8	23.4	104.9	3.0018	3.6
4/16/2018 8:50	0.2	0.6	2.6	22.5	23.3	106.2	3.0158	3.5
4/16/2018 8:51	0.2	0.1	1.5	22	22.1	99.4	2.8035	3.5
4/16/2018 8:52	0.2	0.5	2.9	22.2	22.4	101.6	2.8949	3.5
4/16/2018 8:53	0.2	-0.2	2.7	22.2	22	98.3	2.8687	3.5
4/16/2018 8:54	0.2	0.6	3	22.2	22	102	2.8607	3.5
4/16/2018 8:55	0.2	0.3	4.6	22.1	21.6	101.1	2.8423	3.5
4/16/2018 8:56	0.3	0.5		22	20.4	91.3	2.6506	3.5
4/16/2018 8:57	0.3	0.4		23	20.7	94.9	2.734	3.5
4/16/2018 8:58	0.3	0.2	2.1	21.9	20.5	92.4	2.7279	3.5
4/16/2018 8:59	0.2	0.3	6.4	21.9	20.7	95.9	2.7632	3.5
4/16/2018 9:00	0.2	0		21.6	19.7	90.3	2.5987	3.5
4/16/2018 9:01	0.2	0.5	1.3	21.7	20.1	95	2.6816	3.5
4/16/2018 9:02	0.3	0.9	2.3	21.3	19.2	93.9	2.5308	3.4
4/16/2018 9:03	0.3	-0.1	4	22	19.8	93.7	2.6844	3.4
4/16/2018 9:04	0.3	-0.1	2.7	21.6	19.4	93.4	2.6419	3.5
4/16/2018 9:05	0.2	0.3	7.6	21.6	19.4	93.1	2.6537	3.5
4/16/2018 9:06	0.2	-0.2	9.9	21.4	18.9	92.4	2.5838	3.5
4/16/2018 9:07	0.2	0.1	3.8	21.3	18.9	91.9	2.5838	3.5
4/16/2018 9:08	0.2	0.1	1.6	21.1	18.9	85.1	2.4539	3.5
4/16/2018 9:09	0.2	0.8	3.9	21.4	18.6	87.8	2.5166	3.5
4/16/2018 9:10	0.2	0.4	1.8	21.4	18.2	89.5	2.5455	3.5
4/16/2018 9:11	0.2	0.6	2.3	21.4	18.2	88.9	2.5502	3.6
4/16/2018 9:12	0.2	0.7	3.2	21.3	18.1	88.5	2.5367	3.6
4/16/2018 9:13	0.2	0.2	1.9	21	17.5	88.2	2.4148	3.6
4/16/2018 9:14	0.2	0.2	2.3	21.1	17.1	85	2.3523	3.6
4/16/2018 9:15	0.2	0.4	4.1	20.8	17.3	83.3	2.4355	3.7
4/16/2018 9:16	0.2	0.4	2.9	20.6	16.6	79.4	2.2227	3.8
4/16/2018 9:17	0.3	0.5	1.7	20.7	16.1	76	2.1372	3.8
4/16/2018 9:18	0.5	1.9	1.9	20.6	15	60.7	1.6137	3.8
4/16/2018 9:19	0.9	1.7	2.1	20.4	14.2	42.8	1.2885	3.8
4/16/2018 9:20	1.5	2.5	3.1	20.4	14.1	37.5	1.0633	3.8
4/16/2018 9:21	1.9	3	3	21	14.4	30.3	0.9057	3.8

4/16/2018 9:22	2.2	2.7	1.8	20.7	14.8	30.3	1.0024	3.8
4/16/2018 9:23	2.4	3.4	3.1	21	14.7	30.1	0.8522	3.9
4/16/2018 9:24	2.6	2.8	3.1	21.2	15	29.8	0.9039	3.9
4/16/2018 9:25	2.8	2.9	3.4	21.4	15.5	29.4	0.8561	3.9
4/16/2018 9:26	2.8	2.5	2.7	20.8	15.6	28.9	0.8735	3.9
4/16/2018 9:27	2.6	3.4	3.5	21.1	16.5	28.9	0.8857	3.9
4/16/2018 9:28	2.7	3.3	3	21.3	15.8	27.4	0.643	4.4
4/16/2018 9:29	2.7	4	3.4	21	15.9	25.9	0.6548	4.5
4/16/2018 9:30	3.2	4.5	3.6	21.7	15.9	24	0.5302	3.8
4/16/2018 9:31	3.4	3.8	4.3	21.7	16	22.1	0.5447	3.7
4/16/2018 9:32	3.7	4.3	2.4	22	16	20.6	0.5204	3.7
4/16/2018 9:33	3.9	4.1	3.8	21.8	16	18.6	0.5078	3.5
4/16/2018 9:34	4.1	4.5	4.6	22.1	15.8	17.5	0.5013	3.4
4/16/2018 9:35	4.2	4.8	3.6	21.9	15.7	15.3	0.4998	3.5
4/16/2018 9:36	4.3	5.3	4.5	22.5	15.6	14	0.4569	3.6
4/16/2018 9:37	4.5	5.4	3.7	22.4	15.9	12.9	0.4471	3.6
4/16/2018 9:38	4.8	5.7	4	22.8	15.8	12.2	0.44	3.5
4/16/2018 9:39	4.8	5.1	4.1	22.6	15.8	11.6	0.4344	3.5
4/16/2018 9:40	4.9	5.5	3.8	23.2	15.8	10.8	0.3585	3.5
4/16/2018 9:41	5.1	6.4	4.4	23.5	15.5	10.4	0.3733	3.6
4/16/2018 9:42	5.5	6.1	3.1	23.3	15.3	9.6	0.3358	3.7
4/16/2018 9:43	5.6	6.5	4.1	23.7	15.8	8.9	0.36	3.7
4/16/2018 9:44	5.9	6	3.2	23.4	14.9	8.3	0.3352	3.7
4/16/2018 9:45	5.7	5.6	5.1	23.7	16	7.8	0.3404	3.5
4/16/2018 9:46	6	7.1	4.4	24.5	14.4	7.2	0.2677	3.5
4/16/2018 9:47	6	6.1	3.4	23.8	13.9	6.7	0.2795	3.6
4/16/2018 9:48	5.9	4.5	4.5	22.5	15.1	6.4	0.3536	3.6
4/16/2018 9:49	5	4.7	3.2	22	15.6	6.2	0.3448	3.6
4/16/2018 9:50	4.2	4	3.5	21.5	15.8	6	0.3443	3.5
4/16/2018 9:51	3.7	4.4	3.6	21.3	16	5.8	0.3472	3.4
4/16/2018 9:52	3.4	3.1	3.5	21.2	16.6	5.7	0.3497	3.2
4/16/2018 9:53	3.2	2.9	2.4	21	17.5	5.5	0.3682	3.2
4/16/2018 9:54	3	3	3.3	20.9	17.4	5.4	0.358	3.2
4/16/2018 9:55	2.8	2.4	3.1	21	17.5	5.4	0.3676	3.2
4/16/2018 9:56	2.6	2.5	2.7	20.7	17.6	5.3	0.37	3.3
4/16/2018 9:57	2.4	1.8	2.3	20.7	17.5	5.2	0.3563	3.4
4/16/2018 9:58	2.2	2.1	2	20.6	17.3	5.1	0.3701	3.4
4/16/2018 9:59	2.1	2.3	2.9	20.4	17.1	5	0.3571	3.4
4/16/2018 10:00	2	2.4	2.4	20.4	17.3	5	0.3799	3.4
4/16/2018 10:01	1.9	1.8	1.8	20.3	17.2	5.1	0.3745	3.4
4/16/2018 10:02	1.6	1	1.1	22.2	26.4	32.7	0.4653	3.5
4/16/2018 10:03	1.1	0.8	2.4	26.3	47.5	82.7	0.7899	3.5
4/16/2018 10:04	0.6	0.1	2.2	29	57.6	138.7	1.0377	3.5
4/16/2018 10:05	0.3	0.6	3.7	32.9	66.2	159	1.3495	3.5
4/16/2018 10:06	0.2	0.3	3.8	35.4	70.4	162.7	1.6168	3.6
4/16/2018 10:07	0.3	-0.1	3.3	43	70.8	162.9	2.0015	3.6
4/16/2018 10:08	0.3	0.7	3.6	56.3	68.7	169.6	2.5268	3.7

4/16/2018 10:09	0.3	1.2	4.2	63.2	69.9	176.6	3.091	3.9
4/16/2018 10:10	0.3	0.5	4.8	67.6	70.9	176.3	3.462	4
4/16/2018 10:11	0.2	0.1	3.4	64.9	71.6	177.7	3.5968	4.1
4/16/2018 10:12	0.3	0	2.4	64.4	66.2	163	3.6035	4.1
4/16/2018 10:13	0.3	0.4	4.5	66.7	68.7	169.5	4.1733	4.1
4/16/2018 10:14	0.3	0.2	4.2	64.5	66.2	164.7	4.3735	4
4/16/2018 10:15	0.3	0.6	4.5	62.1	64.5	158.5	4.4327	4.2
4/16/2018 10:16	0.2	0.4	4	60.8	63.3	160.8	4.5802	4.4
4/16/2018 10:17	0.2	-0.2	3.1	56	60.3	151.9	4.4443	4.3
4/16/2018 10:18	0.2	0	5.2	55.6	57.8	145.3	4.3755	4.3
4/16/2018 10:19	0.2	0.2	4.5	56.6	58.7	150.3	4.697	4.3
4/16/2018 10:20	0.2	0.1	4.9	53	57.2	148.3	4.717	4.3
4/16/2018 10:21	0.2	-0.5	4.3	51.8	55.1	146.2	4.6888	4.2
4/16/2018 10:22	0.2	0.3	2.8	50.4	54.1	142.2	4.8177	4.3
4/16/2018 10:23	0.2	0.7	3.5	48.7	52.9	141.3	4.7522	4.3
4/16/2018 10:24	0.2	0.4	3.6	46.7	49.9	134.4	4.5548	4.3
4/16/2018 10:25	0.2	0.6	3.3	47.9	51.5	139.1	4.921	4.3
4/16/2018 10:26	0.1	0.1	3.2	47	50.9	138.7	4.9058	4.3
4/16/2018 10:27	0.1	-0.8	3	46.7	50.6	138.6	5.0868	4.3
4/16/2018 10:28	0.1	0.2	4	45	49	136.6	4.955	4.3
4/16/2018 10:29	0.1	0.2	3.7	41.6	46.7	135.9	4.7125	4.3
4/16/2018 10:30	0.1	0.1	3.8	43.1	47.1	133.2	4.9645	4.3
4/16/2018 10:31	0.1	0.3	4.6	41.4	45.8	130.9	4.8558	4.3
4/16/2018 10:32	0.1	1.1	1.6	41	45.4	130.1	4.954	4.3
4/16/2018 10:33	0.2	0.4	3.5	39	44.2	128.4	4.8515	4.2
4/16/2018 10:34	0.2	0.1	2.2	38.8	42.1	123.5	4.7047	4.2
4/16/2018 10:35	0.2	-0.2	4.7	38.8	42.9	126.6	4.8502	4.2
4/16/2018 10:36	0.2	0.8	4.1	37.8	41.7	123.7	4.8243	4.2
4/16/2018 10:37	0.2	1.1	3.5	38.4	42.8	127.2	5.04	4.2
4/16/2018 10:38	0.2	1.3	2.6	35.1	39.9	115.8	4.602	4.2
4/16/2018 10:39	0.2	0.2	4.1	37.7	40.6	121.4	4.9447	4.3
4/16/2018 10:40	0.2	0.2	2	36	40.3	122.3	4.9317	4.3
4/16/2018 10:41	0.2	0.4	4.1	35.8	39.4	122	4.889	4.4
4/16/2018 10:42	0.2	0.4	3.6	34	38.4	118.5	4.7583	4.4
4/16/2018 10:43	0.2	1	3.9	32.8	36	104.9	4.5082	4.3
4/16/2018 10:44	0.2	0	3.5	33.5	37.6	115.5	4.8318	4.4
4/16/2018 10:45	0.2	1	1.5	32.2	34.7	108.1	4.3733	4.4
4/16/2018 10:46	0.2	0.2	3.4	33.5	36.6	115.2	4.8043	4.4
4/16/2018 10:47	0.2	0.4	2.2	31.7	35	113.4	4.5352	4.5
4/16/2018 10:48	0.2	0.5	2.9	31.3	34.2	109.7	4.519	4.4
4/16/2018 10:49	0.2	0.6	1.6	31	33.9	105.7	4.5062	4.3
4/16/2018 10:50	0.2	0.2	3.2	30.2	33.2	100.3	4.3982	4
4/16/2018 10:51	0.2	0.7	3.6	29.9	32.7	104.5	4.4228	4
4/16/2018 10:52	0.2	0.8	3.8	30.3	32.4	103.6	4.4385	4
4/16/2018 10:53	0.2	-0.5	3.4	30.3	32.9	107.5	4.5757	4
4/16/2018 10:54	0.2	0.1	1.8	30.3	32.8	108.2	4.6325	4
4/16/2018 10:55	0.2	0.6	3.8	29.4	31.3	99.8	4.3372	4

4/16/2018 10:56	0.2	0.6	3.9	29.8	31.5	104.2	4.5108	4.1
4/16/2018 10:57	0.2	0.9	3.4	29.7	31.9	104.7	4.5803	3.2
4/16/2018 10:58	0.2	0.2	3.4	28.3	30.3	97.9	4.2858	3.2
4/16/2018 10:59	0.2	0.7	2.1	28.7	30.2	99.4	4.3263	3.5
4/16/2018 11:00	0.2	0.7	3.4	28.4	29.6	97.8	4.2403	3.7
4/16/2018 11:01	0.2	-0.6	2.2	27.9	29.7	97.8	4.2987	3.9
4/16/2018 11:02	0.2	0.4	2.8	27.4	28.2	92.1	4.0313	4
4/16/2018 11:03	0.2	0.5	3.6	27.9	28.4	95.3	4.1598	4.1
4/16/2018 11:04	0.3	0.7	3.3	27	28.1	96.4	4.1072	4
4/16/2018 11:05	0.3	0.5	2.2	27.1	27.3	91.2	4.0593	3.8
4/16/2018 11:06	0.3	0.4	3.2	27.1	27.6	94.2	4.1175	3.7
4/16/2018 11:07	0.3	1	3.4	26.5	27.3	90.6	4.069	3.7
4/16/2018 11:08	0.3	0.2	3.7	27.3	27.4	94.1	4.1592	3.8
4/16/2018 11:09	0.3	0.3	2.9	25.8	26.9	90.4	3.976	3.8
4/16/2018 11:10	0.3	0.4	1.8	25.6	26.9	86.1	3.8193	3.9
4/16/2018 11:11	0.4	0.8	3.1	26.3	26.6	86.7	3.8887	3.9
4/16/2018 11:12	0.4	0.3	3.7	26.5	26.1	91.2	4.038	4
4/16/2018 11:13	0.4	0.3	3.5	25.8	25.4	90.4	3.8395	4
4/16/2018 11:14	0.4	0.9	2.4	25.9	24.9	86.6	3.8031	4
4/16/2018 11:15	0.4	0.2	2.2	25.6	24.8	86.7	3.8421	4
4/16/2018 11:16	0.4	0.7	2.1	26	24.9	88.1	3.9228	4.1
4/16/2018 11:17	0.3	0.6	3.5	25.9	24.8	87.5	3.8982	4.1
4/16/2018 11:18	0.4	0.6	2.5	24.7	24	84.5	3.6995	4.1
4/16/2018 11:19	0.4	0.8	2.4	24.7	23.4	80.2	3.6478	4.1
4/16/2018 11:20	0.4	0.5	3.5	24.3	23.4	80.4	3.6342	4.1
4/16/2018 11:21	0.4	0.4	2.5	24.1	22.5	76.6	3.4314	4
4/16/2018 11:22	0.5	0.3	0.2	24.4	22.7	77.2	3.527	4.1
4/16/2018 11:23	0.5	0.3	1.7	23.2	21.9	77	3.2429	4
4/16/2018 11:24	0.6	0.5	3.2	22.4	20.3	65.8	2.886	4
4/16/2018 11:25	1	3	3	20.2	19.2	44.5	1.897	3.9
4/16/2018 11:26	1.6	3.4	3.9	18.9	16.9	26.8	1.3793	3.7
4/16/2018 11:27	2.2	3.4	3.7	19.1	16.4	22	1.2115	3.8
4/16/2018 11:28	2.5	3.2	3.3	19.7	16.5	20.4	1.2129	3.7
4/16/2018 11:29	2.8	4.2	4.2	19.6	15.7	18.7	1.0471	3.8
4/16/2018 11:30	3	4.2	3.9	20.1	15.4	18.4	0.9839	3.9
4/16/2018 11:31	3.3	4	3.3	20.3	14.8	18	0.9287	4
4/16/2018 11:32	3.5	4.4	2.3	20.5	14.6	17.6	0.8773	4.1
4/16/2018 11:33	3.8	4.8	4	20.8	14.1	16.8	0.8241	4.2
4/16/2018 11:34	3.9	4.3	3	20.8	14.3	16.3	0.8778	4
4/16/2018 11:35	4	5	3.4	21.1	14	15.6	0.7834	3.8
4/16/2018 11:36	4.1	5.3	3.7	21.4	14.1	14.6	0.773	3.7
4/16/2018 11:37	4.3	5.4	3.8	21.2	14.4	13.8	0.7832	3.7
4/16/2018 11:38	4.3	4.3	2.5	21.3	15.3	13.3	0.8381	3.7
4/16/2018 11:39	4.4	6.8	5	22.9	15	12.9	0.8118	3.8
4/16/2018 11:40	5.2	7.6		22.6	14.1	12.3	0.7272	3.8
4/16/2018 11:41	6.1	8.3		23.7	14.2	12	0.6879	3.8
4/16/2018 11:42	7.2	9.1		24.7	13.4	11.3	0.5981	3.8

4/16/2018 11:43	7.5	8.7		24.6	12.9	10.7	0.6239	3.6
4/16/2018 11:44	7.9	8.2		24.5	12.8	10.4	0.6153	3.6
4/16/2018 11:45	7.6	8.6		24.3	13.7	10.1	0.6368	3.6
4/16/2018 11:46	7.6	8.5		24.7	13.2	9.5	0.5341	3.8
4/16/2018 11:47	7.5	7.8	8.2	24.3	13.9	8.9	0.558	3.7
4/16/2018 11:48	7.5	7.8	14.2	24.2	13.8	8.4	0.5743	3.5
4/16/2018 11:49	7.5	8.8	6.7	25.4	13.1	7.9	0.4928	3.6
4/16/2018 11:50	7.8	8.3	11	25.1	12.6	7.5	0.6117	3.7
4/16/2018 11:51	8.2	9	12.6	24.7	12.2	7.2	0.5818	3.8
4/16/2018 11:52	8.2	8.9	12.4	25.2	12.5	6.9	0.5159	3.8
4/16/2018 11:53	8.2	9	25.4	25.4	12.2	6.5	0.4844	3.8
4/16/2018 11:54	8	8.1		24.5	12.5	6.2	0.4643	3
4/16/2018 11:55	7.7	8.3	4.2	24.3	13.4	5.9	0.4657	2.5
4/16/2018 11:56	7.7	9	4.5	25.3	12	5.6	0.4049	2.9
4/16/2018 11:57	7.9	9.6	5.3	25.5	11.4	5.3	0.3928	3.1
4/16/2018 11:58	8.4	9.1	5.1	25.4	11.5	5.1	0.3699	3.3
4/16/2018 11:59	8.4	9.4	5.4	25.4	11.7	4.8	0.3676	3.5
4/16/2018 12:00	8.4	9.9	16.8	25.6	11.4	4.6	0.3527	3.5
4/16/2018 12:01	8.6	9.7	7.5	26	10.9	4.3	0.3124	3.5
4/16/2018 12:02	8.9	9.7	7	26.2	10.4	4.1	0.2995	3.5
4/16/2018 12:03	9.1	10	6.6	26.1	10.3	4	0.2822	3.5
4/16/2018 12:04	9.2	9.6	6.2	25.8	10.4	3.9	0.2803	3.5
4/16/2018 12:05	9.1	9.6	5.9	26.1	10.5	3.8	0.258	3.6
4/16/2018 12:06	9.1	9.2	6.5	26	10.4	3.7	0.2496	3.7
4/16/2018 12:07	9.1	10.1	6	26.4	10.3	3.5	0.2454	3.7
4/16/2018 12:08	9.3	10.4	6	26.3	10.1	3.5	0.2211	3.4
4/16/2018 12:09	9.4	10.9	6	26.6	10	3.4	0.2142	3.2
4/16/2018 12:10	9.5	10.6	8.1	26.4	10	3.3	0.1912	3.3
4/16/2018 12:11	9.6	10.5	5.2	26.6	10	3.2	0.1939	3.4
4/16/2018 12:12	9.6	10.2	6.6	26.4	10	3.1	0.1744	3.5
4/16/2018 12:13	9.6	10.3	6.5	26.7	9.9	3	0.1729	3.4
4/16/2018 12:14	9.7	10	6.8	26.4	9.9	3	0.1664	3.6
4/16/2018 12:15	9.6	10.5	6.3	26.6	9.8	2.9	0.1536	3.6
4/16/2018 12:16	9.8	11.4	6.6	26.8	9.7	2.8	0.1581	3.6
4/16/2018 12:17	9.9	10.9	6.3	26.6	9.6	2.7	0.1381	3.6
4/16/2018 12:18	9.9	11	6.4	26.5	9.6	2.7	0.1491	3.7
4/16/2018 12:19	9.9	11.4	5.8	26.9	9.5	2.6	0.1266	3.9
4/16/2018 12:20	10.1	10.6	6.4	27.2	9.4	2.6	0.1268	3.9
4/16/2018 12:21	10.2	10.8	6.5	27.1	9.3	2.5	0.1255	3.9
4/16/2018 12:22	10.2	10.8	5.3	26.8	9.2	2.5	0.1129	4
4/16/2018 12:23	10.1	11.1	7.9	27.4	9.1	2.5	0.1196	3.8
4/16/2018 12:24	10.3	11.1	7.4	27.5	9	2.5	0.0938	3.5
4/16/2018 12:25	10.5	11.7	8	27.7	8.9	2.5	0.1093	2.6
4/16/2018 12:26	10.7	11.2	7.8	27.9	8.9	2.4	0.0802	2.1
4/16/2018 12:27	10.8	11.4	6.6	27.7	8.9	2.4	0.0944	2.2
4/16/2018 12:28	10.9	11.6	5.8	27.7	8.9	2.4	0.079	2.3
4/16/2018 12:29	11	11.4	7.5	27.8	8.9	2.4	0.0835	2.4

4/16/2018 12:30	10.9	11.4	6.5	27.9	8.8	2.4	0.0898	2.5
4/16/2018 12:31	11	11.5	7.7	27.9	8.7	2.3	0.073	2.6
4/16/2018 12:32	11.1	12.4	6.5	28.2	8.7	2.3	0.0746	2.7
4/16/2018 12:33	11.3	11.6	5.4	28.2	8.6	2.3	0.0595	2.9
4/16/2018 12:34	11.4	12.4	6.3	28.1	8.6	2.3	0.0701	3
4/16/2018 12:35	11.5	12.1	6	28.2	8.6	2.3	0.0616	3.2
4/16/2018 12:36	11.5	12.3	7.5	28.4	8.5	2.3	0.0643	3.2
4/16/2018 12:37	11.5	11.9	5.4	28.4	8.5	2.3	0.0623	3.3
4/16/2018 12:38	11.5	12.2	7.2	28.3	8.5	2.3	0.0508	3.2
4/16/2018 12:39	11.6	12.6	7.7	28.8	8.4	2.3	0.0659	3
4/16/2018 12:40	11.8	12.4	7.9	28.8	8.3	2.3	0.0511	3
4/16/2018 12:41	11.9	12.4	7	28.6	8.3	2.3	0.0595	2.7
4/16/2018 12:42	11.9	12.2	6.9	28.7	8.3	2.2	0.0456	2.5
4/16/2018 12:43	11.8	12.2	7.4	28.9	8.3	2.2	0.0547	2.6
4/16/2018 12:44	12	12.4	8.2	29.1	8.5	2.2	0.044	2.8
4/16/2018 12:45	12.1	12.9	6.7	28.6	8.5	2.2	0.0429	3.1
4/16/2018 12:46	12.1	12.6	7.2	28.6	8.5	2.2	0.0461	3.2
4/16/2018 12:47	12	12.4	7.7	28.6	8.5	2.2	0.0316	3.3
4/16/2018 12:48	12	13	6.9	28.5	8.5	2.2	0.0532	3.3
4/16/2018 12:49	11.9	11.5	7.4	28.8	8.4	2.2	0.0316	3.3
4/16/2018 12:50	11.9	12.1	8.7	29	8.4	2.2	0.0391	3.3
4/16/2018 12:51	11.9	12.8	8.9	28.8	8.4	2.2	0.0362	3.3
4/16/2018 12:52	12	12.2	7.8	29.1	8.3	2.2	0.0292	3.2
4/16/2018 12:53	12	12.3	6.9	28.8	8.3	2.2	0.0282	3.4
4/16/2018 12:54	12	11.7	6.3	28.9	8.3	2.2	0.0245	3.5
4/16/2018 12:55	12.1	12.6	6.5	29.3	8.3	2.2	0.0374	3.5
4/16/2018 12:56	12.1	12.2	7.7	28.5	8.2	2.2	0.0153	3.6
4/16/2018 12:57	11.8	11.2	6.2	27.8	8.1	2.2	0.0287	3.7
4/16/2018 12:58	11.4	11	7.1	28.1	8	2.2	0.0325	3.8
4/16/2018 12:59	11.1	11.6	6.8	27.9	8.1	2.2	0.0256	3.8
4/16/2018 13:00	10.9	11.1	7.2	28	8.1	2.2	0.0349	3.7
4/16/2018 13:01	10.8	10.9	7.2	27.4	8	2.3	0.0288	3.7
4/16/2018 13:02	10.5	10.3	6	27	8	2.3	0.0359	3.7
4/16/2018 13:03	9.4	6.5	4.1	25	13.3	13.1	0.0809	3.9
4/16/2018 13:04	7.3	3.7	3.1	23.8	23.1	28.1	0.2712	3.9
4/16/2018 13:05	4.7	1.8	2.4	23.7	28	34.3	0.411	3.9
4/16/2018 13:06	2.5	1.5	3.1	24.7	30.7	36.8	0.5936	3.9
4/16/2018 13:07	1.2	1.2	2.8	25.3	30.4	36.3	0.7667	3.3
4/16/2018 13:08	0.8	0.8	1.5	25	29	36	0.8203	3.5
4/16/2018 13:09	0.6	0.8	2.2	24.9	29.6	35.9	0.9905	3.7
4/16/2018 13:10	0.5	1	1.8	24.9	29.6	35.8	1.0214	3.7
4/16/2018 13:11	0.4	0.9	2	24.7	29.3	35.6	1.0865	3.7
4/16/2018 13:12	0.3	0.3	0.7	24.9	29.3	35.4	1.1185	3.8
4/16/2018 13:13	0.3	1.2	2.5	24.5	29.2	35.1	1.1215	3.9
4/16/2018 13:14	0.3	0.7	1	24.2	27.8	34.8	1.1573	4.1
4/16/2018 13:15	0.3	0.5	3.2	24.1	27.2	34.3	1.1882	4.1
4/16/2018 13:16	0.3	0.7	2.8	23.8	26.7	34.1	1.163	4.1

4/16/2018 13:17	0.3	0.2	1.3	23.9	26.4	33.7	1.1708	4.2
4/16/2018 13:18	0.3	0.6	2.9	23.5	26.1	33.3	1.1828	4.1
4/16/2018 13:19	0.4	0.5	2.6	23.1	25.3	32.8	1.1677	4.2
4/16/2018 13:20	0.3	0.7	2.5	22.9	25.1	32.3	1.1448	4.3
4/16/2018 13:21	0.4	0.4	3	22.7	24.5	31.8	1.1252	4.2
4/16/2018 13:22	0.3	1.1	2.2	22.9	24.5	31.4	1.1402	4.1
4/16/2018 13:23	0.3	0.6	2.9	22.8	24.3	31.1	1.1275	4
4/16/2018 13:24	0.2	-0.1	1.7	22.6	23.9	30.7	1.1252	4
4/16/2018 13:25	0.2	0.8	2	22.5	23.7	30.4	1.0988	3.8
4/16/2018 13:26	0.2	0.8	1.2	22.5	23.4	30	1.0838	3.7
4/16/2018 13:27	0.2	0.2	2.6	22.4	23	29.6	1.0553	3.8
4/16/2018 13:28	0.3	1	3.8	22.2	22.3	29.3	1.0427	3.4
4/16/2018 13:29	0.3	0.9	1.5	21.9	22.4	28.9	1.0333	3.4
4/16/2018 13:30	0.4	0.9	3.3	22	21.5	28.5	0.9844	3.5
4/16/2018 13:31	0.4	0.5	1.3	21.9	21.9	28.1	1.0275	3.6
4/16/2018 13:32	0.4	1.4	3.5	21.9	21.8	27.8	0.9992	3.6
4/16/2018 13:33	0.3	1	1.5	21.7	21.3	27.4	0.9863	3.7
4/16/2018 13:34	0.3	1.1	2.8	21.9	21.3	27	0.9913	3.7
4/16/2018 13:35	0.3	0.3	2.3	21.8	21.2	26.7	0.9938	3.8
4/16/2018 13:36	0.3	1	3.4	21.5	20.7	26.5	0.956	3.8
4/16/2018 13:37	0.3	0.3	0.9	21.6	20.7	26.3	0.9613	3.7
4/16/2018 13:38	0.3	0.9	3.7	21.6	20.4	26.1	0.9586	3.8
4/16/2018 13:39	0.3	0.4	2.2	21.2	20.3	26	0.9342	3.9
4/16/2018 13:40	0.3	0.8	2.2	21.5	19.9	25.7	0.9236	4
4/16/2018 13:41	0.4	0.9	2.1	21.2	19.4	25.4	0.8836	4
4/16/2018 13:42	0.4	0.2	3	21.3	19.5	25.1	0.9091	4.1
4/16/2018 13:43	0.5	0.6	2.7	21.3	19.3	24.8	0.899	4.2
4/16/2018 13:44	0.4	0.9	1.8	21.1	19.2	24.5	0.891	4.1
4/16/2018 13:45	0.4	0.9	1.3	21.1	19	24.2	0.8879	4.1
4/16/2018 13:46	0.4	1.2	2.3	21.2	19	24	0.884	4.1
4/16/2018 13:47	0.5	1.4	1.9	21.1	18.2	23.6	0.8214	4.1
4/16/2018 13:48	0.6	0.9	2.7	21	18.1	23.3	0.8331	4.1
4/16/2018 13:49	0.6	1	1.7	21.1	18.2	23	0.8424	4.1
4/16/2018 13:50	0.6	0.7	2.7	20.9	18.2	22.8	0.8506	4
4/16/2018 13:51	0.6	0.9	2.2	21	17.7	22.5	0.814	3.5
4/16/2018 13:52	0.7	1	3.2	21	17.3	22.2	0.7884	3.4
4/16/2018 13:53	0.8	0.9	2.1	20.8	17.1	21.9	0.7803	3.6
4/16/2018 13:54	0.7	0.9	2.1	20.7	17.5	21.6	0.8163	3.6
4/16/2018 13:55	0.7	1.5	2.1	20.8	17	21.3	0.7769	3.6
4/16/2018 13:56	0.6	0.2	1.9	20.8	17.2	21.2	0.7978	3.7
4/16/2018 13:57	0.7	0.6	1.6	21.1	16.9	21.1	0.7821	3.9
4/16/2018 13:58	0.7	1	2	20.4	17	20.9	0.7872	4
4/16/2018 13:59	0.6	0.7	1.7	20.6	16.9	20.7	0.7889	3.9
4/16/2018 14:00	0.7	1.2	4	20.8	16.2	20.5	0.7382	4
4/16/2018 14:01	0.7	1	1.1	20.9	16	20.4	0.7352	4
4/16/2018 14:02	0.7	1	2.1	20.7	16.3	20.2	0.7717	4
4/16/2018 14:03	0.6	0.4	2.2	20.7	16.2	20.1	0.7443	4.1

4/16/2018 14:04	0.6	1.1	3.6	20.9	15.8	19.9	0.7281	4
4/16/2018 14:05	0.6	0.7	1.5	20.6	15.8	19.7	0.7155	4
4/16/2018 14:06	0.7	0.8	3.1	20.6	15.4	19.4	0.696	4
4/16/2018 14:07	0.8	0.9	2.5	20.8	15.2	19.2	0.6861	4
4/16/2018 14:08	0.8	0.8	1.6	20.6	15.3	19	0.6941	4
4/16/2018 14:09	0.8	1.1	2.9	20.7	14.8	18.7	0.667	4.1
4/16/2018 14:10	0.9	1.3	2.7	20.5	14.9	18.5	0.6688	4.1
4/16/2018 14:11	0.9	0.7	2.7	20.7	14.9	18.2	0.6828	4
4/16/2018 14:12	0.8	1	2	20.5	15	18	0.6751	4
4/16/2018 14:13	0.8	1.9	2.6	20.6	15	17.6	0.6323	3.9
4/16/2018 14:14	0.9	1.4	2.7	20.8	14.9	17.4	0.6437	3.9
4/16/2018 14:15	1	1.5	2.4	20.2	14.7	17.2	0.6412	3.9
4/16/2018 14:16	0.9	0.5	2.6	20.4	14.7	17	0.6708	3.9
4/16/2018 14:17	0.6	1.5	1.8	19.9	14.8	16.9	0.6578	3.9
4/16/2018 14:18	0.6	0.3	1.9	20.5	14.4	16.8	0.6501	3.9
4/16/2018 14:19	0.6	0.5	3.8	20.2	14.2	16.5	0.628	3.9
4/16/2018 14:20	0.8	1.7	0.8	20.4	14	16.3	0.6047	3.9
4/16/2018 14:21	1.1	1.4	2.2	20.4	13.4	16.1	0.5667	3.9
4/16/2018 14:22	1.8	3.6	4.3	22.5	12.7	15.7	0.4957	3.9
4/16/2018 14:23	3.8	8.3	5.6	24.8	9.9	14.4	0.2855	3.9
4/16/2018 14:24	5.9	8.5	5.4	25.6	9.2	13.4	0.2271	3.7
4/16/2018 14:25	7.8	9	6.1	25.7	8.9	11.9	0.2146	3.6
4/16/2018 14:26	8	7.3	4.9	25.3	8.7	10.4	0.1899	3.7
4/16/2018 14:27	7.8	8.3	5.6	25	8.5	9.1	0.1989	3.8
4/16/2018 14:28	7.7	8.3	6.1	25.1	8.3	7.9	0.1773	3.9
4/16/2018 14:29	7.8	9.1	5.9	25.9	8.2	6.6	0.1658	3.8
4/16/2018 14:30	8.3	8.8	8.3	26.1	8.1	5.3	0.1726	3.8
4/16/2018 14:31	8.7	9.8	6.8	26.7	7.9	4.7	0.1381	4.5
4/16/2018 14:32	9	9.1	5.9	26.2	8.5	4.4	0.1557	4.8
4/16/2018 14:33	9	8.5	5.3	26.2	9.3	4.2	0.1713	3.8
4/16/2018 14:34	8.9	8.8	6.3	26.5	9	4	0.1678	3.8
4/16/2018 14:35	9	10.6	7.9	27	9.1	3.9	0.1525	3.9
4/16/2018 14:36	9.6	10.7	6.9	27.8	8.1	3.7	0.119	3.9
4/16/2018 14:37	10	10.5	5.5	27.2	8	3.6	0.1234	3.8
4/16/2018 14:38	10.4	10.8	7.4	28	7.9	3.4	0.0829	3.6
4/16/2018 14:39	10.6	11.2	5	28.1	7.7	3.3	0.0945	3.6
4/16/2018 14:40	10.9	11.4	6.4	28.1	7.9	3.2	0.108	3.8
4/16/2018 14:41	11.4	12.4	7.4	29.7	7.5	3.1	0.0983	3.9
4/16/2018 14:42	12.1	13.5	7.9	29.7	7.1	3	0.0843	3.8
4/16/2018 14:43	12.3	12.5	7.3	29.6	7.5	2.8	0.0872	3.6
4/16/2018 14:44	12.5	12.8	7.3	29.8	7	2.7	0.0884	3.7
4/16/2018 14:45	12.4	13.1	8	29.5	6.8	2.7	0.0643	3.8
4/16/2018 14:46	12.7	13.5	8	30	6.6	2.6	0.0776	3.9
4/16/2018 14:47	12.9	13.5	9.3	30.3	6.2	2.6	0.0656	3.9
4/16/2018 14:48	12.9	12.7	6.8	29.6	6.5	2.5	0.0736	3.9
4/16/2018 14:49	12.7	12.7	7.9	30.1	6.9	2.4	0.0728	3.9
4/16/2018 14:50	12.8	14.3	8.2	30.6	6.1	2.4	0.0518	3.9

4/16/2018 14:51	13	13.5	7.8	30	6.1	2.3	0.0567	4
4/16/2018 14:52	13.1	12.9	6.5	29.9	6.3	2.3	0.0554	4.1
4/16/2018 14:53	12.8	13.2	8.7	30.1	6.3	2.3	0.071	4
4/16/2018 14:54	12.5	12.9	7.7	29.7	6.3	2.3	0.0512	4.1
4/16/2018 14:55	12.3	11.7	7.8	28.8	6.6	2.3	0.0722	4.1
4/16/2018 14:56	11.9	11.7	5.7	28.7	6.9	2.3	0.0798	4.1
4/16/2018 14:57	10.8	8.4	4.1	27.4	11.5	8	0.1211	4
4/16/2018 14:58	8.6	5.1	4.4	36	23.7	24.7	0.4814	4
4/16/2018 14:59	5.5	1.5	4	40.4	31.4	30.4	0.9323	4.1
4/16/2018 15:00	2.8	2	4	40.5	34	33.7	1.1593	4
4/16/2018 15:01	1.3	1.6	2.6	40	34.3	34.2	1.1983	4.1
4/16/2018 15:02	0.8	0.2	4.2	38.7	33.7	34	1.195	4.1
4/16/2018 15:03	0.6	0.9	2.2	37.6	32.8	33.6	1.1607	4
4/16/2018 15:04	0.4	1	2.1	36.7	32.6	33.1	1.131	3.9
4/16/2018 15:05	0.4	0.9	4.2	35.3	30.7	32.3	1.055	3.9
4/16/2018 15:06	0.5	0.7	1.8	35	30.4	31.6	1.0445	3.8
4/16/2018 15:07	0.6	1.1	3.3	33.1	29	30.7	0.9881	3.8
4/16/2018 15:08	0.7	1.2	2.1	32.8	28.4	29.7	0.9695	3.8
4/16/2018 15:09	0.8	1.2	1.8	32.7	27.8	28.8	0.9583	3.7
4/16/2018 15:10	0.6	0.7	3	32.5	28.7	27.9	0.9934	3.7
4/16/2018 15:11	0.6	1.3	3.7	30.8	27.1	27.1	0.9055	3.7
4/16/2018 15:12	0.6	1.3	2.6	30.6	26.5	26.2	0.907	3.6
4/16/2018 15:13	0.7	1.1	2.3	30.6	26.2	25.6	0.891	3.6
4/16/2018 15:14	0.7	1	3.2	29.9	26.7	25.1	0.8858	3.6
4/16/2018 15:15	0.7	1.1	3.6	29.9	26.7	24.6	0.8935	3.7
4/16/2018 15:16	0.6	0.7	3.3	29.1	25.9	24.2	0.8692	3.8
4/16/2018 15:17	0.7	0.3	3.6	28.7	24.4	23.8	0.837	3.8
4/16/2018 15:18	0.7	0.7	3.5	28.5	24.7	23.3	0.8384	3.9
4/16/2018 15:19	0.7	0.9	2.7	28.5	24.8	22.9	0.8593	3.8
4/16/2018 15:20	0.6	1.9	2.4	28	24.3	22.6	0.8164	3.8
4/16/2018 15:21	0.8	1.6	6.1	27	22.9	22.2	0.7802	3.8
4/16/2018 15:22	0.8	1.1	3.2	27.7	23.5	21.8	0.8102	3.7
4/16/2018 15:23	0.8	0.2	3.5	27	23.5	21.5	0.8022	3.8
4/16/2018 15:24	0.7	0.3	1.6	27	23.5	21	0.8059	3.8
4/16/2018 15:25	0.6	0.7	1.7	26.8	23	20.7	0.7827	4
4/16/2018 15:26	0.7	0.8	3.8	26.1	22.3	20.4	0.7598	4
4/16/2018 15:27	0.9	1.6	1	26	21.4	19.9	0.7358	4
4/16/2018 15:28	0.9	1.2	2.6	25.6	21.9	19.5	0.7493	4.1
4/16/2018 15:29	0.9	0.5	2.4	25.6	20.8	19.1	0.7183	4.1
4/16/2018 15:30	0.9	1.7	2.1	25.3	20.3	18.7	0.6814	4
4/16/2018 15:31	0.9	1.1	2.8	25.7	21.1	18.4	0.7361	4
4/16/2018 15:32	0.9	1.6	2.8	25.1	21.2	18.2	0.7196	3.9
4/16/2018 15:33	0.9	1.5	3.8	24.3	19.7	17.7	0.6563	3.9
4/16/2018 15:34	1.1	0.4	3	24.9	19.2	17.3	0.6615	3.9
4/16/2018 15:35	1.2	1.4	3.2	24.6	20.2	17.1	0.6904	3.9
4/16/2018 15:36	1.1	1.5	2.7	24.3	19.4	16.8	0.6578	3.8
4/16/2018 15:37	1.1	2	2.6	24.3	19.1	16.5	0.6389	3.8

4/16/2018 15:38	1	1	3.6	24.5	19.3	16.3	0.6701	3.7
4/16/2018 15:39	1	0.5	2.8	24.4	19.3	16.2	0.6501	3.8
4/16/2018 15:40	1	1.2	2.6	24	19.1	15.9	0.6438	3.7
4/16/2018 15:41	0.9	0.7	0.8	24	19.4	15.8	0.6587	3.8
4/16/2018 15:42	0.8	1.5	2.6	23.7	19	15.8	0.6563	3.5
4/16/2018 15:43	0.8	1	3.1	23.7	18.6	15.5	0.6315	3.2
4/16/2018 15:44	1	1.2	2.5	23.9	17.5	15.2	0.5805	3.3
4/16/2018 15:45	1.1	1.4	1.7	23.3	18.4	15	0.6396	3.6
4/16/2018 15:46	1.2	1.9	2.8	23.3	17.3	14.8	0.5664	3.8
4/16/2018 15:47	1.1	1.4	2.3	23.3	17.9	14.6	0.5975	3.9
4/16/2018 15:48	1.2	1.3	1.6	23.2	17.5	14.3	0.5924	4
4/16/2018 15:49	1.2	1.6	3	23	17.3	14	0.58	4
4/16/2018 15:50	1.3	1.8	2.3	23.2	16.7	13.7	0.5645	4.1
4/16/2018 15:51	1.2	1.5	2.3	23.2	17.3	13.5	0.5797	4.1
4/16/2018 15:52	1.2	2	1.5	23	17.1	13.4	0.5858	4.1
4/16/2018 15:53	1	1.3	1.9	22.9	17.3	13.3	0.5874	4.1
4/16/2018 15:54	1	1.4	3.4	22.8	16.5	13.1	0.5595	4.2
4/16/2018 15:55	0.9	2.1	2.1	22.7	16.7	12.9	0.5736	4.2
4/16/2018 15:56	1.1	1.8	1.5	22.8	15.5	12.7	0.5067	4.2
4/16/2018 15:57	1.1	1	2.7	22.9	16	12.5	0.5399	4.2
4/16/2018 15:58	1.2	2	2.5	22.4	16.2	12.3	0.5349	4.2
4/16/2018 15:59	1.2	2.2	3.2	22.4	15.1	12	0.4966	4.2
4/16/2018 16:00	1.2	1.3	2.9	22.5	15.7	11.8	0.5295	4.1
4/16/2018 16:01	1.2	1.6	2.7	22.3	15.6	11.6	0.5141	3.4
4/16/2018 16:02	1.1	1.2	2.7	22.3	15	11.4	0.5084	3.3
4/16/2018 16:03	1.1	2	3	22.3	15	11.2	0.4922	3.3
4/16/2018 16:04	1.2	1.9	2.3	22.3	14.9	11	0.4906	3.5
4/16/2018 16:05	1.2	2.1	1.1	22.4	14.9	10.9	0.4968	3.8
4/16/2018 16:06	1.3	2.3	4	22.3	14.7	10.7	0.4724	3.2
4/16/2018 16:07	1.2	1.6	2.9	22.1	14.6	10.6	0.4895	2.2
4/16/2018 16:08	1.3	2.3	2.1	22	14.2	10.4	0.4492	2.4
4/16/2018 16:09	1.2	1.2	4.1	22	14.8	10.3	0.5003	2.8
4/16/2018 16:10	1.4	1.7	3	22.1	13.7	10.2	0.438	3
4/16/2018 16:11	1.4	2.4	3.8	22.1	13.5	10	0.4371	3.1
4/16/2018 16:12	1.6	1.1	1.5	22	13.9	9.8	0.4618	3.1
4/16/2018 16:13	2.2	4.5	4.8	23	11.6	9.5	0.3207	3.2
4/16/2018 16:14	3.7	7.4	7	25.6	10.2	9	0.2742	3.3
4/16/2018 16:15	6.2	10	6.9	26.9	9.8	8.3	0.1858	3.4
4/16/2018 16:16	8	10.8	5.5	27	9.8	7.7	0.1906	3.4
4/16/2018 16:17	9	10	6.2	27.4	8.8	6.9	0.1657	3.6
4/16/2018 16:18	9.2	10.3	6.4	27.7	8.3	6.1	0.1458	3.8
4/16/2018 16:19	9.6	10.9	4.5	27.8	8.3	5.5	0.1529	3.9
4/16/2018 16:20	9.3	8.9	4.7	26.7	8.9	4.8	0.18	4
4/16/2018 16:21	9.3	11.3	5.7	27.7	7.8	4.2	0.135	4.2
4/16/2018 16:22	9.6	11.5	5.5	28.1	7	3.7	0.1132	4.3
4/16/2018 16:23	10.3	11.1	8.2	29.3	6.6	3.4	0.1063	4.2
4/16/2018 16:24	10.9	13.4	15.6	27.5	6.6	3.2	0.1122	3.7

4/16/2018 16:25	12	12.8	12.5	18.2	6.6	3.1	0.1019	2.5
4/16/2018 16:26					6.5	3	0.1086	2.4
4/16/2018 16:27					6.3	2.9	0.0856	2.2
4/16/2018 16:28					6.3	2.8	0.105	2.2
4/16/2018 16:29					6.2	2.6	0.0838	2.2
4/16/2018 16:30					6.2	2.6	0.0872	2.2
4/16/2018 16:31					6	2.6	0.0888	2.1
4/16/2018 16:32					5.8	2.5	0.0682	2.2
4/16/2018 16:33					5.8	2.4	0.0755	2.3
4/16/2018 16:34					6.1	2.4	0.0631	2.4
4/16/2018 16:35					6	2.3	0.0673	2.5
4/16/2018 16:36					5.9	2.2	0.055	2.5
4/16/2018 16:37					6	2.2	0.0517	2.5
4/16/2018 16:38					5.7	2.1	0.0483	2.6
4/16/2018 16:39					5.7	2.1	0.0363	2.6
4/16/2018 16:40					5.6	2	0.0524	2.6
4/16/2018 16:41					5.4	2	0.0378	2.7
4/16/2018 16:42					5.3	2	0.0556	2.7
4/16/2018 16:43					5.4	1.9	0.0376	2.7
4/16/2018 16:44					5.2	1.9	0.0406	2.7
4/16/2018 16:45					5.2	1.9	0.0354	2.7
4/16/2018 16:46					5.2	1.9	0.0324	2.7
4/16/2018 16:47					5.1	1.8	0.0329	2.7
4/16/2018 16:48					5.1	1.8	0.0209	2.7
4/16/2018 16:49					5	1.8	0.0306	2.7
4/16/2018 16:50					4.9	1.8	0.0121	2.7
4/16/2018 16:51					4.9	1.8	0.0229	2.7
4/16/2018 16:52					4.8	1.8	0.0057	2.7
4/16/2018 16:53					4.8	1.8	-0.0852	1.4
4/16/2018 16:54					4.7	1.7	-0.1967	0.1
4/16/2018 16:55	14.3	15.2	7.8	35.4	4.7	1.7	-0.2074	0.2
4/16/2018 16:56	14.5	14.7	8.8	33.6	4.7	1.7	-0.1847	0.2
4/16/2018 16:57	14.7	15.1	8.4	33.3	4.6	1.7	-0.2097	0.2
4/16/2018 16:58	15	15	7.2	32.7	4.6	1.6	-0.1938	0.2
4/16/2018 16:59	15.3	15.8	8.2	32.9	4.5	1.6	-0.2058	0.2
4/16/2018 17:00	15.4	15.8	9.3	32.4	4.4	1.6	-0.0968	0.2
4/16/2018 17:01	15.4	16.7	7.7	32.8	4.3	1.6	0.0117	0.3
4/16/2018 17:02	15.4	15.7	8.6	32.8	4.4	1.6	-0.0015	0.1
4/16/2018 17:03	15.6	15.7	8.1	33.1	4.4	1.6	0.0151	0
4/16/2018 17:04	15.8	16.4	9.2	33.1	4.3	1.6	0.0856	1.1
4/16/2018 17:05	16	16	9.5	33.3	4.3	1.5	0.2038	2.4
4/16/2018 17:06	16	16.6	8.8	33.2	4.2	1.5	0.2073	3
4/16/2018 17:07	15.9	16	10.2	32.8	4.2	1.5	0.2186	3.1
4/16/2018 17:08	15.9	16.8	8.4	33.4	4.2	1.5	0.2125	2.6
4/16/2018 17:09	16.1	16.6	9.2	33.3	4.2	1.5	0.213	2.8
4/16/2018 17:10	16.3	16.4	7.7	33.6	4.2	1.5	0.2146	2.9
4/16/2018 17:11	16.5	16.2	8.3	33.7	4.2	1.5	0.2022	2.6

4/16/2018 17:12	16.5	16.9	9.8	33.8	4.1	1.4	0.2145	2.3
4/16/2018 17:13	16.5	16.6	9.4	33.2	4.1	1.4	0.204	2.8
4/16/2018 17:14	16.5	16.6	8.5	33.7	4.1	1.4	0.2143	2.9
4/16/2018 17:15	16.4	16.4	8.6	33.5	4.1	1.4	0.2096	2.8
4/16/2018 17:16	16.4	16.5	8.5	33.5	4.1	1.4	0.2087	2.9
4/16/2018 17:17	16.4	16.6	8.9	33.9	4.1	1.4	0.2151	3
4/16/2018 17:18	16.6	17.6	8.6	33.7	4.1	1.4	0.1949	3.1
4/16/2018 17:19	16.6	16.7	8.1	33.4	4.1	1.4	0.2055	3.1
4/16/2018 17:20	16	15.4	7.8	33.5	4.1	1.4	0.1988	3.2
4/16/2018 17:21	14.6	8.5	8.8	33.8	4.1	1.4	0.204	3.2
4/16/2018 17:22	12	12.1	9.3	34	4	1.4	0.203	3.2
4/16/2018 17:23	8.4	13.7	8.6	34	4	1.4	0.1946	3.3
4/16/2018 17:24	4.8	14.9	8.9	34.1	4	1.4	0.2069	3.4
4/16/2018 17:25	2.3	13.6	8.8	32.5	4	1.4	0.1976	3.5
4/16/2018 17:26	1.1	12.9	8.8	32.2	4	1.4	0.2063	3.6
4/16/2018 17:27	0.5	12.3	6.6	31.5	3.9	1.5	0.1948	3.7
4/16/2018 17:28	0.2	13.3	9	32.6	4	1.5	0.2082	3.6
4/16/2018 17:29			9	32.1	4	1.5	0.204	3.7
4/16/2018 17:30			8.2	31.8	3.9	1.5	0.1962	3.9
4/16/2018 17:31			6.6	31.3	4	1.5	0.2085	4
4/16/2018 17:32			6	30.7	4	1.6	0.1949	4
4/16/2018 17:33			7.8	30.4	3.9	1.6	0.2097	4.1
4/16/2018 17:34			7.6	30	4	1.6	0.1939	4.2
4/16/2018 17:35			6.6	29.6	4	1.6	0.202	4.2
4/16/2018 17:36			7.8	29.6	4	1.6	0.199	4.2
4/16/2018 17:37			7.8	29.1	4	1.7	0.1958	4.2
4/16/2018 17:38			7	29.2	3.9	1.7	0.2069	4.2
4/16/2018 17:39			6.6	28.8	4	1.8	0.1985	4.2
4/16/2018 17:40			6.4	28.4	4	1.8	0.2095	4.3
4/16/2018 17:41			6.1	28.2	4	1.8	0.1907	4.3
4/16/2018 17:42			5.8	27.7	4	1.8	0.2012	4.2
4/16/2018 17:43			7.1	27.6	4	1.9	0.1981	4.3
4/16/2018 17:44			6.4	27.3	4	1.9	0.2012	4.3
4/16/2018 17:45			6	27	4.1	1.9	0.2001	4.3
4/16/2018 17:46			5.4	27.1	4.1	1.9	0.1866	4.3
4/16/2018 17:47			6.5	26.9	4.1	2	0.2013	4.1
4/16/2018 17:48			4.8	26.7	4.1	2	0.1912	3.6
4/16/2018 17:49			5.4	26.5	4.2	2	0.2035	3.8
4/16/2018 17:50			5.5	26.1	4.3	2	0.2055	3.6
4/16/2018 17:51			4.6	25.8	4.3	2	0.1961	3.7
4/16/2018 17:52			6.3	25.9	4.3	2	0.2101	4
4/16/2018 17:53			6.9	25.1	4.3	2.1	0.1955	4.1
4/16/2018 17:54			5.8	25.2	4.4	2.1	0.2106	4.4
4/16/2018 17:55			5.5	25.6	4.4	2.1	0.1952	4.5
4/16/2018 17:56			5.1	24.9	4.2	2.1	0.2086	4.5
4/16/2018 17:57			4	24	4	2.2	0.203	4.4
4/16/2018 17:58			4.8	24.1	4.1	2.2	0.1962	4.3

4/16/2018 17:59	4.5	24.4	4.3	2.2	0.2148	4.4
4/16/2018 18:00	4	24.7	4.3	2.2	0.1983	4.4
4/16/2018 18:01	5.3	24.6	4.3	2.2	0.2045	4.4
4/16/2018 18:02		24.1	4.2	2.2	0.1921	4.4
4/16/2018 18:03	5.3	24.4	4	2.2	0.202	4.4
4/16/2018 18:04	5	24.6	4	2.3	0.2	4.3
4/16/2018 18:05	4.8	24.4	4	2.2	0.1995	4.3
4/16/2018 18:06	4.6	24.6	4	2.2	0.2084	4.3
4/16/2018 18:07	4.9	24.4	4	2.2	0.1917	4.5
4/16/2018 18:08	3	24.3	3.9	2.3	0.203	4.5
4/16/2018 18:09	6.1	24.6	3.9	2.3	0.1945	4.5
4/16/2018 18:10	5.2	24.3	3.9	2.3	0.2051	4.3
4/16/2018 18:11	5.5	24.3	4	2.3	0.1968	3.5
4/16/2018 18:12		24.6	4	2.2	0.1977	3.2
4/16/2018 18:13		24.5	4.1	2.2	0.206	3.5
4/16/2018 18:14		24.7	4	2.2	0.1923	3.5
4/16/2018 18:15		24.4	4.1	2.2	0.2091	3.5
4/16/2018 18:16		24.8	4.1	2.2	0.1914	3.5
4/16/2018 18:17	5.1	25.3	4.2	2.2	0.2013	3.5
4/16/2018 18:18	4.9	25.4	4.2	2.2	0.1946	3.6
4/16/2018 18:19	5.3	25	4.3	2.1	0.1944	3.6
4/16/2018 18:20	3.1	24.2	4	2.2	0.2033	3.6
4/16/2018 18:21	3.6	23.8	4	2.2	0.1945	3.6
4/16/2018 18:22	4.6	23.6	4	2.2	0.2137	3.8
4/16/2018 18:23	3.1	23.5	4	2.2	0.1907	3.9
4/16/2018 18:24	3.7	23.5	4	2.3	0.2101	4
4/16/2018 18:25	4.6	23.5	4	2.3	0.1944	4
4/16/2018 18:26	5.2	23.5	4	2.3	0.2011	3.9
4/16/2018 18:27	3.9	23.8	4	2.3	0.1966	3.9
4/16/2018 18:28	4.2	23.6	4	2.3	0.2031	4
4/16/2018 18:29	2.8	23.5	4	2.3	0.2011	3.9
4/16/2018 18:30	4.6	23.5	4	2.3	0.1982	3.9
4/16/2018 18:31	3.7	23.7	4	2.3	0.2055	4.1
4/16/2018 18:32	3.1	23.4	3.9	2.3	0.1956	4.2
4/16/2018 18:33	3.8	23.9	4	2.3	0.2113	4
4/16/2018 18:34	4.8	24	4	2.3	0.1848	4.3
4/16/2018 18:35	3.4	24.9	4.1	2.2	0.2024	4.3
4/16/2018 18:36	5.1	24	4	2.3	0.1913	4.4
4/16/2018 18:37	4	23.1	3.9	2.3	0.2025	4.5
4/16/2018 18:38	3.7	23.2	3.9	2.3	0.207	4.6
4/16/2018 18:39	3.6	23.3	4	2.3	0.192	4.6
4/16/2018 18:40	5.9	23.3	4	2.3	0.204	4.6
4/16/2018 18:41	4.1	23.4	3.9	2.3	0.1924	4.6
4/16/2018 18:42	2.5	23.1	3.9	2.4	0.2093	4.5
4/16/2018 18:43	3.4	22.8	3.9	2.4	0.1923	4.2
4/16/2018 18:44	3.5	22.9	3.9	2.4	0.2054	4.2
4/16/2018 18:45	3.8	23.4	3.9	2.4	0.1952	4.1

4/16/2018 18:46	3.9	23.8	3.9	2.4	0.1981	3.1
4/16/2018 18:47	4.6	23.5	3.9	2.4	0.206	3.6
4/16/2018 18:48	3.1	23.7	4	2.4	0.1937	3.9
4/16/2018 18:49	3.7	23.6	3.9	2.4	0.1998	4
4/16/2018 18:50	3.5	23.6	3.9	2.4	0.1898	4
4/16/2018 18:51	4.4	23.5	3.9	2.4	0.2056	4
4/16/2018 18:52	2.9	23	3.9	2.4	0.1909	4.1
4/16/2018 18:53	3.9	22.9	3.9	2.4	0.2012	4.3
4/16/2018 18:54	3.3	22.9	3.9	2.4	0.202	4.3
4/16/2018 18:55	4.4	22.9	3.9	2.4	0.1903	4.3
4/16/2018 18:56	4	23	3.8	2.4	0.2001	4.4
4/16/2018 18:57	4.6	23	3.8	2.4	0.1884	4.4
4/16/2018 18:58	4.5	23	3.8	2.4	0.1942	4.4
4/16/2018 18:59	4.3	23.4	3.8	2.4	0.1877	4.3
4/16/2018 19:00	4.1	23.4	3.8	2.4	0.2007	4.3
4/16/2018 19:01	5.2	22.9	3.8	2.4	0.198	4.5
4/16/2018 19:02	4	23.1	3.8	2.4	0.1882	4.4
4/16/2018 19:03	3	23.3	3.8	2.4	0.1988	4.4
4/16/2018 19:04	3.8	22.7	3.8	2.4	0.1853	4.4
4/16/2018 19:05	3.7	22.9	3.8	2.4	0.1931	4.3
4/16/2018 19:06	5.3	23	3.8	2.4	0.1801	4.3
4/16/2018 19:07	4.1	22.7	3.8	2.4	0.1891	4.4
4/16/2018 19:08	3.8	22.7	3.8	2.4	0.1904	4.4
4/16/2018 19:09	4	22.7	3.8	2.4	0.1808	4.4
4/16/2018 19:10	5	22.7	3.8	2.4	0.1929	4.6
4/16/2018 19:11	3.5	22.7	3.8	2.4	0.1725	4.6
4/16/2018 19:12	3.4	23.3	3.8	2.4	0.181	4.5
4/16/2018 19:13	4	23.2	3.8	2.4	0.1789	3.2
4/16/2018 19:14	4	23.1	3.8	2.4	0.1797	3.6
4/16/2018 19:15	3.8	23.6	3.8	2.4	0.1796	4
4/16/2018 19:16	4	23.4	3.8	2.4	0.1814	4.2
4/16/2018 19:17	3.5	23.3	3.8	2.4	0.1845	4.3
4/16/2018 19:18	5	23.3	3.8	2.4	0.1848	4.5
4/16/2018 19:19	5.4	23.8	3.8	2.4	0.1811	4.5
4/16/2018 19:20	4.6	24.1	3.8	2.3	0.188	4.3
4/16/2018 19:21	4.4	23.9	3.9	2.3	0.18	3.8
4/16/2018 19:22	4	24.8	3.9	2.3	0.1881	3.9
4/16/2018 19:23	3.7	25.3	3.9	2.2	0.1783	4
4/16/2018 19:24	6.4	25.5	3.9	2.2	0.1854	3.9
4/16/2018 19:25	3.7	25	3.9	2.1	0.1744	3.9
4/16/2018 19:26	3.9	23.2	3.8	2.1	0.178	4
4/16/2018 19:27	3.4	22.9	3.8	2.1	0.1919	4.1
4/16/2018 19:28	4.7	22.4	3.8	2.2	0.1738	4.3
4/16/2018 19:29	5.1	22.4	3.8	2.2	0.1938	4.3
4/16/2018 19:30	4.1	22.4	3.7	2.2	0.188	4.3
4/16/2018 19:31	3.7	22.5	3.7	2.3	0.1931	4.3
4/16/2018 19:32	3.6	22.6	3.7	2.3	0.1827	4.3

4/16/2018 19:33	1.7	22.4	3.7	2.4	0.1765	4.5
4/16/2018 19:34	3	22.4	3.7	2.4	0.1907	4.6
4/16/2018 19:35	4.7	22.4	3.7	2.4	0.18	4.6
4/16/2018 19:36	3.1	22.5	3.7	2.4	0.1815	4.7
4/16/2018 19:37	3.8	22.5	3.7	2.4	0.1944	4.6
4/16/2018 19:38	3.5	22.4	3.7	2.4	0.1789	4.4
4/16/2018 19:39	3.9	22.2	3.7	2.4	0.1907	4.6
4/16/2018 19:40	3	22.2	3.6	2.4	0.1818	4.5
4/16/2018 19:41	2.5	22.3	3.6	2.4	0.1946	4.4
4/16/2018 19:42	5.2	22.6	3.6	2.4	0.1868	4.4
4/16/2018 19:43	3.9	22.7	3.6	2.4	0.1819	3.9
4/16/2018 19:44	4.4	23.2	3.6	2.4	0.1884	3.4
4/16/2018 19:45	4.4	23.1	3.6	2.4	0.1703	3.7
4/16/2018 19:46	4.3	23.3	3.6	2.4	0.1875	3.9
4/16/2018 19:47	3.8	23.3	3.6	2.4	0.1806	4
4/16/2018 19:48	3.4	22.8	3.6	2.4	0.1756	4
4/16/2018 19:49	3	23.4	3.6	2.3	0.1979	3.9
4/16/2018 19:50	4.1	23	3.6	2.3	0.174	4
4/16/2018 19:51	2.4	22.3	3.6	2.3	0.1893	4.2
4/16/2018 19:52	4.2	22.3	3.6	2.3	0.1824	4.2
4/16/2018 19:53	3.5	22.4	3.6	2.3	0.1818	4.3
4/16/2018 19:54	3.4	22.5	3.6	2.3	0.1878	4.3
4/16/2018 19:55	3.8	22.4	3.6	2.4	0.1746	4.4
4/16/2018 19:56	3.1	22.4	3.6	2.4	0.1896	4.5
4/16/2018 19:57	4.2	22.9	3.6	2.4	0.1836	4.6
4/16/2018 19:58	3.6	23.2	3.6	2.4	0.1803	4.6
4/16/2018 19:59	2.6	22.4	3.5	2.4	0.1948	4.5
4/16/2018 20:00	3.8	22.4	3.6	2.4	0.1899	4.5
4/16/2018 20:01	3.2	22.3	3.6	2.4	0.1981	4.6
4/16/2018 20:02	3.8	22.4	3.5	2.3	0.1756	4.8
4/16/2018 20:03	5.3	22.3	3.6	2.3	0.1889	4.8
4/16/2018 20:04	4	22.7	3.5	2.3	0.1866	5
4/16/2018 20:05	4.6	22.7	3.6	2.3	0.1798	4.9
4/16/2018 20:06	2.8	22.7	3.6	2.3	0.1953	4.9
4/16/2018 20:07	3.4	22.8	3.6	2.3	0.1762	4.8
4/16/2018 20:08	4.3	22.8	3.5	2.4	0.1948	4.9
4/16/2018 20:09	3.2	23	3.5	2.4	0.1789	4.9
4/16/2018 20:10	3.9	22.8	3.6	2.4	0.1862	4.9
4/16/2018 20:11	4.5	22.9	3.5	2.4	0.1953	5
4/16/2018 20:12	2.9	22.9	3.5	2.4	0.1772	4.9
4/16/2018 20:13	3.9	22.5	3.5	2.3	0.1914	4.7
4/16/2018 20:14	2.8	22.6	3.5	2.3	0.181	4.8
4/16/2018 20:15	3.1	22.8	3.5	2.3	0.1946	4.8
4/16/2018 20:16	3.3	22.6	3.5	2.3	0.1974	5
4/16/2018 20:17	3.7	23.1	3.6	2.3	0.1815	5
4/16/2018 20:18	3.9	22.5	3.5	2.3	0.1901	4.9
4/16/2018 20:19	2.9	22.7	3.5	2.3	0.1818	4.7

4/16/2018 20:20	3.2	22.4	3.5	2.3	0.1881	3.9
4/16/2018 20:21	3.4	22.1	3.5	2.3	0.1833	3.2
4/16/2018 20:22	4.1	22.3	3.5	2.3	0.1879	3.6
4/16/2018 20:23	3	22.2	3.5	2.3	0.196	3.8
4/16/2018 20:24	2.7	22.1	3.5	2.4	0.1827	3.9
4/16/2018 20:25	2	22.2	3.5	2.4	0.1939	3.9
4/16/2018 20:26	3.3	21.9	3.5	2.4	0.186	4.1
4/16/2018 20:27	5	22.3	3.5	2.4	0.1933	4.3
4/16/2018 20:28	3.9	22.1	3.5	2.4	0.196	4.3
4/16/2018 20:29	3.2	22.1	3.5	2.4	0.1859	4.4
4/16/2018 20:30	4.4	22.1	3.5	2.4	0.1969	4.5
4/16/2018 20:31	3.2	21.7	3.5	2.4	0.1799	4.5
4/16/2018 20:32	3.7	22	3.5	2.4	0.1916	4.5
4/16/2018 20:33	3.8	21.9	3.5	2.4	0.1839	4.4
4/16/2018 20:34	4.3	21.9	3.5	2.4	0.1869	4.4
4/16/2018 20:35	3.2	21.6	3.5	2.4	0.1939	4.4
4/16/2018 20:36	3.6	21.8	3.5	2.4	0.1847	4.5
4/16/2018 20:37	4	21.7	3.5	2.4	0.1874	4.5
4/16/2018 20:38	5.7	21.8	3.5	2.4	0.1767	4.6
4/16/2018 20:39	2.8	22.2	3.5	2.4	0.1866	4.6
4/16/2018 20:40	3.4	22.2	3.5	2.4	0.1902	4.7
4/16/2018 20:41	4.3	22.5	3.5	2.4	0.1816	4.8
4/16/2018 20:42	4.3	22.7	3.5	2.4	0.1888	4.8
4/16/2018 20:43	4.4	22.7	3.5	2.4	0.1881	4.8
4/16/2018 20:44	3.1	22.5	3.5	2.4	0.1908	4.7
4/16/2018 20:45	3.9	22.1	3.5	2.4	0.1872	4.6
4/16/2018 20:46	3.8	21.9	3.5	2.3	0.184	4.6
4/16/2018 20:47	3.8	21.7	3.5	2.4	0.1903	4.6
4/16/2018 20:48	3.8	21.9	3.4	2.4	0.1776	4.4
4/16/2018 20:49	4.3	22.1	3.4	2.4	0.1893	4.4
4/16/2018 20:50	3.5	21.8	3.4	2.4	0.1908	4.5
4/16/2018 20:51	4.5	22.3	3.4	2.4	0.1821	4.5
4/16/2018 20:52	2.8	21.8	3.5	2.4	0.188	4.5
4/16/2018 20:53	4.7	21.8	3.4	2.4	0.1781	4.5
4/16/2018 20:54	2.7	21.6	3.4	2.4	0.1937	4.4
4/16/2018 20:55	4	21.6	3.4	2.4	0.1854	4.5
4/16/2018 20:56	3.9	21.5	3.4	2.4	0.1866	4.4
4/16/2018 20:57	3	21.9	3.4	2.4	0.1947	4.5
4/16/2018 20:58	4.2	21.7	3.4	2.4	0.1816	4.5
4/16/2018 20:59	2.6	21.6	3.4	2.4	0.1946	4.5
4/16/2018 21:00	3.4	21.6	3.4	2.4	0.186	4.4
4/16/2018 21:01	3.1	21.7	3.4	2.4	0.1953	4.1
4/16/2018 21:02	4.1	21.6	3.4	2.5	0.1893	2.7
4/16/2018 21:03	2.6	21.8	3.5	2.5	0.1821	2.7
4/16/2018 21:04	3.6	21.9	3.5	2.4	0.1949	3
4/16/2018 21:05	3.1	21.7	3.5	2.4	0.1812	3.3
4/16/2018 21:06	2.9	22	3.5	2.4	0.1904	3.3

4/16/2018 21:07	3.9	22	3.5	2.4	0.1867	3.4
4/16/2018 21:08	3.9	22	3.5	2.4	0.1817	3.5
4/16/2018 21:09	2.9	22	3.6	2.4	0.198	3.5
4/16/2018 21:10	2.9	21.9	3.5	2.4	0.178	3.6
4/16/2018 21:11	3.3	22.2	3.6	2.3	0.19	3.5
4/16/2018 21:12	3.5	22.1	3.6	2.3	0.1862	3.8
4/16/2018 21:13	4.4	21.9	3.6	2.3	0.1842	4
4/16/2018 21:14	3.8	22.1	3.6	2.3	0.1906	4
4/16/2018 21:15	4.9	22	3.6	2.3	0.1813	4.1
4/16/2018 21:16	3.2	21.8	3.7	2.3	0.1994	4.2
4/16/2018 21:17	3.9	22.1	3.6	2.3	0.1847	4.3
4/16/2018 21:18	3.9	21.6	3.6	2.3	0.1861	4.2
4/16/2018 21:19	3	21.5	3.6	2.3	0.1905	4.1
4/16/2018 21:20	3.6	21.6	3.6	2.3	0.1832	4.1
4/16/2018 21:21	3.5	21.4	3.6	2.3	0.1948	4.1
4/16/2018 21:22	3.8	21.5	3.6	2.4	0.1816	4.2
4/16/2018 21:23	3.9	21.5	3.6	2.4	0.1906	4.3
4/16/2018 21:24		21.6	3.6	2.4	0.1928	4.3
4/16/2018 21:25	3.1	21.2	3.6	2.4	0.1829	4.2
4/16/2018 21:26	2.2	21.2	3.6	2.4	0.1931	4.2
4/16/2018 21:27		21.5	3.6	2.4	0.1816	4.2
4/16/2018 21:28		21.6	3.7	2.4	0.1955	4.2
4/16/2018 21:29		21.8	3.7	2.4	0.1866	4.1
4/16/2018 21:30	9.7	21.6	3.7	2.4	0.1844	4.1
4/16/2018 21:31		21.7	3.7	2.4	0.1933	4
4/16/2018 21:32	4.8	21.7	3.8	2.4	0.18	3.9
4/16/2018 21:33	2.9	21.5	3.8	2.4	0.1879	3.8
4/16/2018 21:34	6.4	21.6	3.8	2.4	0.193	3.8
4/16/2018 21:35	3.3	21.4	3.8	2.4	0.1821	4
4/16/2018 21:36	3.7	22	3.8	2.4	0.196	4.3
4/16/2018 21:37	5.2	21.6	3.9	2.4	0.1722	4.3
4/16/2018 21:38	2.4	21.6	3.9	2.4	0.1942	4.2
4/16/2018 21:39	4.4	21.1	3.8	2.4	0.1871	4.2
4/16/2018 21:40	3.1	21.3	3.8	2.4	0.1899	4.3
4/16/2018 21:41	3.3	21.1	3.7	2.4	0.1926	4.4
4/16/2018 21:42	3.4	21	3.8	2.4	0.1825	4.4
4/16/2018 21:43	2.2	21.5	3.9	2.4	0.1978	4.4
4/16/2018 21:44	3.9	21.2	3.9	2.4	0.1783	4.3
4/16/2018 21:45	3.7	21.2	4	2.5	0.2009	4.2
4/16/2018 21:46	3.2	20.8	3.9	2.5	0.1857	4.3
4/16/2018 21:47	4	20.9	4	2.5	0.1907	3.7
4/16/2018 21:48	2.3	20.7	3.9	2.5	0.1931	1.8
4/16/2018 21:49	3.9	20.9	4	2.5	0.1831	1.6
4/16/2018 21:50	3.3	20.8	4.1	2.5	0.1944	1.9
4/16/2018 21:51	2.5	20.7	4	2.5	0.1845	2.1
4/16/2018 21:52	2.9	20.9	4.1	2.5	0.1881	2.3
4/16/2018 21:53	2.2	20.9	4.2	2.5	0.1884	2.4

4/16/2018 21:54	4.2	21	4.3	2.5	0.177	2.6
4/16/2018 21:55	2.8	20.7	4.2	2.5	0.1954	2.7
4/16/2018 21:56	2.6	20.5	4.3	2.5	0.1822	2.8
4/16/2018 21:57	2.9	20.9	4.3	2.5	0.1922	2.9
4/16/2018 21:58	2.6	20.7	4.2	2.5	0.1844	3
4/16/2018 21:59	2.7	20.7	4.4	2.5	0.1913	3.1
4/16/2018 22:00	3.2	20.7	4.3	2.5	0.1999	3.2
4/16/2018 22:01	2.6	20.7	4.3	2.5	0.1824	3.3
4/16/2018 22:02	2	20.8	4.3	2.5	0.1936	3.4
4/16/2018 22:03	2.5	20.3	4.4	2.5	0.1797	3.5
4/16/2018 22:04	2.9	20.7	4.4	2.5	0.1916	3.6
4/16/2018 22:05	2.2	20.5	4.4	2.5	0.1952	3.6
4/16/2018 22:06	3	20.7	4.4	2.5	0.1799	3.6
4/16/2018 22:07	3.2	20.5	4.4	2.5	0.1936	3.6
4/16/2018 22:08	3.1	20.5	4.4	2.5	0.1779	3.6
4/16/2018 22:09	2	20.4	4.4	2.5	0.1918	3.7
4/16/2018 22:10	2.6	20.6	4.4	2.5	0.1913	3.7
4/16/2018 22:11	3	20.7	4.5	2.5	0.1872	3.8
4/16/2018 22:12	2.3	20.5	4.5	2.5	0.1934	3.8
4/16/2018 22:13	2.7	20.6	4.4	2.5	0.179	3.9
4/16/2018 22:14	2.7	20.6	4.5	2.5	0.1968	4
4/16/2018 22:15	2.1	20.6	4.5	2.5	0.1848	4
4/16/2018 22:16	3.3	20.2	4.5	2.5	0.1876	4
4/16/2018 22:17	2.2	20.6	4.5	2.5	0.1879	4.1
4/16/2018 22:18	3.1	20.4	4.5	2.5	0.1844	4.1
4/16/2018 22:19	2.4	20.5	4.5	2.5	0.1955	4.1
4/16/2018 22:20	3.3	20.6	4.4	2.5	0.1867	4.1
4/16/2018 22:21	2.7	20.6	4.5	2.5	0.1932	4.1
4/16/2018 22:22	3.5	20.6	4.5	2.5	0.1865	4.1
4/16/2018 22:23	3.5	20.6	4.5	2.5	0.1841	4.1
4/16/2018 22:24	3.1	20.8	4.6	2.5	0.1926	4.1
4/16/2018 22:25	3.4	21	4.6	2.5	0.1832	4.1
4/16/2018 22:26	3.4	20.8	4.6	2.5	0.1998	4.1
4/16/2018 22:27	3.8	20.8	4.6	2.4	0.1866	4.2
4/16/2018 22:28	2.5	20.7	4.6	2.5	0.1859	4.2
4/16/2018 22:29	2.8	20.5	4.6	2.5	0.1926	4.1
4/16/2018 22:30	3.6	20.7	4.7	2.5	0.1865	4
4/16/2018 22:31	2.8	20.4	4.8	2.5	0.1953	3.9
4/16/2018 22:32	1.9	20.5	4.7	2.5	0.1879	2.4
4/16/2018 22:33	2.8	20.4	4.8	2.5	0.1929	1.8
4/16/2018 22:34	2.6	20.5	4.7	2.5	0.1908	2
4/16/2018 22:35	2.6	20.5	4.7	2.5	0.1767	2.2
4/16/2018 22:36	3.7	20.5	4.8	2.5	0.192	2.4
4/16/2018 22:37	3.1	20.8	4.9	2.5	0.1882	2.6
4/16/2018 22:38	1.8	20.7	5	2.5	0.2022	2.8
4/16/2018 22:39	3.5	20.9	5.1	2.5	0.197	3
4/16/2018 22:40	2.2	20.9	5	2.5	0.1842	3.1

4/16/2018 22:41	3.6	20.8	5	2.5	0.2019	3.2
4/16/2018 22:42	2.1	20.8	5	2.5	0.1881	3.2
4/16/2018 22:43	3.8	20.9	5	2.5	0.1822	3.3
4/16/2018 22:44	4	21	5.2	2.5	0.1932	3.3
4/16/2018 22:45	2.8	20.6	5.1	2.4	0.1886	3.4
4/16/2018 22:46	3.4	20.9	5.1	2.4	0.2012	3.4
4/16/2018 22:47	3.7	20.8	5.2	2.4	0.1804	3.5
4/16/2018 22:48	1.8	21	5.2	2.4	0.2005	3.5
4/16/2018 22:49	3.1	20.9	5.2	2.4	0.1891	3.6
4/16/2018 22:50	3.3	20.9	5.2	2.4	0.188	3.6
4/16/2018 22:51	3.1	21	5.2	2.4	0.1962	3.7
4/16/2018 22:52	4.9	20.8	5.2	2.4	0.1818	3.7
4/16/2018 22:53	2.4	20.8	5.1	2.4	0.199	3.8
4/16/2018 22:54	3.1	20.4	5.1	2.4	0.1873	3.8
4/16/2018 22:55	4.4	20.8	5.3	2.4	0.1828	3.8
4/16/2018 22:56	2.1	20.7	5.3	2.4	0.1979	3.8
4/16/2018 22:57	1.8	20.4	5.1	2.4	0.1823	4
4/16/2018 22:58	1.8	20.8	5.1	2.4	0.1975	4.2
4/16/2018 22:59	4.5	21	5.2	2.4	0.1861	4.4
4/16/2018 23:00	1.5	20.9	5.1	2.4	0.1962	4.4
4/16/2018 23:01	2.1	20.5	5.1	2.4	0.1917	4.5
4/16/2018 23:02	2.2	20.7	5.1	2.4	0.1828	4.5
4/16/2018 23:03	2.6	20.6	5.1	2.4	0.1987	4.5
4/16/2018 23:04	3.5	20.4	5	2.4	0.1854	4.5
4/16/2018 23:05	1.8	20.5	5.1	2.5	0.1945	4.5
4/16/2018 23:06	2.7	20.5	5.1	2.4	0.1862	4.6
4/16/2018 23:07	2	20.5	5	2.5	0.1891	4.6
4/16/2018 23:08	3.6	20.6	5	2.5	0.1918	4.7
4/16/2018 23:09	3.1	20.4	5.1	2.5	0.1824	4.7
4/16/2018 23:10	2.5	20.5	5.1	2.5	0.2003	4.8
4/16/2018 23:11	2.5	20.2	5	2.5	0.1869	4.8
4/16/2018 23:12	1.5	20.3	5.1	2.5	0.1931	4.7
4/16/2018 23:13	3.2	20.2	5.2	2.5	0.1992	4.7
4/16/2018 23:14	3.4	20.5	5.2	2.5	0.1823	4.7
4/16/2018 23:15	1.8	20.2	5.2	2.6	0.1997	4.6
4/16/2018 23:16	3.1	20.3	5.1	2.6	0.1885	4.7
4/16/2018 23:17	2.2	20.5	5.1	2.6	0.1935	4.7
4/16/2018 23:18	2.1	20.3	5.2	2.5	0.1918	4.7
4/16/2018 23:19	1.8	20.4	5.3	2.5	0.1837	4.7
4/16/2018 23:20	2.5	20.3	5.2	2.5	0.1971	4.7
4/16/2018 23:21	2.6	20.3	5.1	2.5	0.1851	4.7
4/16/2018 23:22	1.9	20.2	5.1	2.5	0.2006	4.6
4/16/2018 23:23	2.6	20.2	5.1	2.5	0.1936	4.7
4/16/2018 23:24	2.5	20.2	5.1	2.5	0.1871	4.5
4/16/2018 23:25	2.6	20.1	5.1	2.5	0.1962	4.6
4/16/2018 23:26	3.2	20.1	5.1	2.6	0.1858	4.6
4/16/2018 23:27	2.5	20.1	5.1	2.6	0.1905	4.6

4/16/2018 23:28	0.7	20	5.2	2.6	0.1873	4.6
4/16/2018 23:29	2.3	19.9	5.2	2.6	0.1858	4.7
4/16/2018 23:30	0.4	20	5.1	2.6	0.1968	4.7
4/16/2018 23:31	3.1	20	5.2	2.6	0.1804	4.8
4/16/2018 23:32	2.3	19.9	5.3	2.6	0.1929	4.7
4/16/2018 23:33	2.3	19.9	5.2	2.6	0.1871	4.7
4/16/2018 23:34	2.9	20	5.3	2.6	0.1911	4.7
4/16/2018 23:35	2.8	20.2	5.3	2.6	0.1973	4.8
4/16/2018 23:36	2.8	19.9	5.3	2.6	0.1752	4.9
4/16/2018 23:37	2.3	19.9	5.3	2.6	0.1975	4.8
4/16/2018 23:38	2.9	19.9	5.5	2.6	0.1864	4.7
4/16/2018 23:39	3.6	19.9	5.4	2.6	0.1951	4.7
4/16/2018 23:40	2.7	19.9	5.5	2.6	0.1892	4.2
4/16/2018 23:41	3.3	20	5.4	2.6	0.1857	3.1
4/16/2018 23:42	2.2	19.9	5.5	2.6	0.1922	3.2
4/16/2018 23:43	3	19.8	5.5	2.5	0.1786	3.6
4/16/2018 23:44	1.4	20	5.5	2.5	0.1961	3.7
4/16/2018 23:45	3.1	20.1	5.5	2.6	0.1847	3.8
4/16/2018 23:46	3.6	19.9	5.5	2.6	0.1905	3.9
4/16/2018 23:47	2.5	19.8	5.5	2.6	0.1884	4
4/16/2018 23:48	2.4	20.1	5.7	2.6	0.1775	4.1
4/16/2018 23:49	3.2	19.9	5.7	2.6	0.1939	4.4
4/16/2018 23:50	2.8	19.9	5.6	2.6	0.1814	4.4
4/16/2018 23:51	2.7	19.9	5.7	2.6	0.1914	4.2
4/16/2018 23:52	3.1	19.6	5.7	2.6	0.1934	4.1
4/16/2018 23:53	3.4	19.7	5.7	2.6	0.1883	4.1
4/16/2018 23:54	2.9	19.7	5.7	2.6	0.1948	4.1
4/16/2018 23:55	4.3	19.9	5.7	2.6	0.1782	4.4
4/16/2018 23:56	1.9	19.8	5.7	2.6	0.1903	4.7
4/16/2018 23:57	2.9	19.9	5.8	2.7	0.1912	4.8
4/16/2018 23:58	3.1	19.6	5.8	2.7	0.1881	4.9
4/16/2018 23:59	3	19.7	5.8	2.7	0.1938	5
4/17/2018 0:00	1.8	19.7	5.8	2.7	0.182	5.1
4/17/2018 0:01	1.2	19.6	5.9	2.7	0.1994	5
4/17/2018 0:02	2.6	19.8	5.9	2.7	0.1836	5
4/17/2018 0:03	1.9	19.8	6	2.7	0.1928	5
4/17/2018 0:04	2.5	19.7	6	2.7	0.196	4.8
4/17/2018 0:05	3.9	20.1	6.5	2.6	0.1829	4.2
4/17/2018 0:06	2.2	19.9	6.5	2.6	0.1954	4.1
4/17/2018 0:07	2.9	19.9	6.5	2.6	0.1855	4.4
4/17/2018 0:08	3.3	20	6.6	2.6	0.196	4.7
4/17/2018 0:09	3.7	20	6.6	2.6	0.1912	4.9
4/17/2018 0:10	2.4	20	6.9	2.6	0.1907	4.8
4/17/2018 0:11	2.8	20.1	6.7	2.6	0.1957	4.6
4/17/2018 0:12	2.6	19.8	6.6	2.5	0.1868	4.7
4/17/2018 0:13	3	19.8	6.5	2.5	0.2015	4.6
4/17/2018 0:14	2.4	19.7	6.4	2.5	0.194	4.7

4/17/2018 0:15		19.8	6.3	2.5	0.1913	4.7
4/17/2018 0:16		19.8	6.4	2.5	0.1952	4.7
4/17/2018 0:17		19.8	6.3	2.6	0.1852	4.7
4/17/2018 0:18	1.6	19.9	6.8	2.6	0.2012	4.8
4/17/2018 0:19	1.3	19.7	6.6	2.6	0.1841	4.8
4/17/2018 0:20		19.5	6.3	2.6	0.2003	4.8
4/17/2018 0:21		19.5	6.2	2.6	0.1899	4.8
4/17/2018 0:22		19.3	6.4	2.6	0.1944	4.9
4/17/2018 0:23		19.3	6.3	2.6	0.1981	4.9
4/17/2018 0:24	1.5	19.7	6.3	2.6	0.1897	4.9
4/17/2018 0:25	6.1	19.5	6.3	2.6	0.2004	5
4/17/2018 0:26	1.7	19.7	6.3	2.6	0.1874	5
4/17/2018 0:27	0.5	19.5	6.6	2.7	0.2003	5
4/17/2018 0:28	2.1	19.4	6.4	2.7	0.1934	4.9
4/17/2018 0:29	3.4	19.5	6.4	2.7	0.1819	5
4/17/2018 0:30	2.5	19.5	6.5	2.7	0.1998	5
4/17/2018 0:31	2.5	19.5	6.6	2.7	0.1809	4.9
4/17/2018 0:32	2.2	19.6	6.8	2.7	0.1942	4.9
4/17/2018 0:33	1.9	19.5	6.7	2.7	0.2022	4.9
4/17/2018 0:34	1.8	19.4	6.8	2.7	0.1948	4.8
4/17/2018 0:35	2.7	19.4	6.7	2.7	0.2065	4.9
4/17/2018 0:36	3.1	19.4	7	2.7	0.1704	4.9
4/17/2018 0:37	3	19.4	7.4	2.7	0.2022	4.7
4/17/2018 0:38	1.6	19.3	6.9	2.7	0.1926	4.4
4/17/2018 0:39	2.5	19.6	7.2	2.7	0.1881	4.3
4/17/2018 0:40	2.6	19.4	7.1	2.7	0.2064	4.3
4/17/2018 0:41	2.3	19.4	7.2	2.7	0.1934	4.2
4/17/2018 0:42	3.2	19.8	7.3	2.7	0.2099	4.3
4/17/2018 0:43	2.5	19.6	7.8	2.7	0.2056	4.2
4/17/2018 0:44	2.9	19.5	7.5	2.7	0.1974	4.1
4/17/2018 0:45	1	19.6	7.7	2.7	0.2014	4.2
4/17/2018 0:46	2.7	20.1	7.6	2.7	0.1959	4.1
4/17/2018 0:47	2.2	19.7	7.6	2.6	0.1975	3.3
4/17/2018 0:48	2.4	19.7	7.2	2.6	0.1956	3.5
4/17/2018 0:49	1.5	19.6	7.2	2.6	0.2006	3.8
4/17/2018 0:50	1.5	19.7	7.3	2.6	0.1988	4
4/17/2018 0:51	1.9	19.6	7.1	2.6	0.198	4.1
4/17/2018 0:52	2.2	19.8	7.3	2.6	0.2022	4.2
4/17/2018 0:53	0.4	19.7	7.2	2.6	0.1847	4.3
4/17/2018 0:54	1.9	19.4	6.9	2.6	0.1992	4.3
4/17/2018 0:55	2.3	19.7	6.9	2.7	0.2006	4.3
4/17/2018 0:56	2.4	19.4	6.9	2.7	0.1977	4.3
4/17/2018 0:57	2.6	19.5	6.8	2.7	0.2024	4.3
4/17/2018 0:58	2.4	19.6	6.8	2.7	0.1931	4.4
4/17/2018 0:59	2.7	19.5	6.9	2.7	0.1972	4.4
4/17/2018 1:00	1.9	19.5	7	2.7	0.1935	4.4
4/17/2018 1:01	1.7	19.3	6.9	2.7	0.198	4.3

4/17/2018 1:02	1.3	19.9	7	2.7	0.2032	4.3
4/17/2018 1:03	2.4	19.6	7	2.7	0.1927	4.4
4/17/2018 1:04	2.1	19.8	6.9	2.7	0.2097	4.5
4/17/2018 1:05	1	19.7	6.9	2.7	0.1936	4.2
4/17/2018 1:06	1.5	19.5	6.9	2.7	0.2015	4.3
4/17/2018 1:07	1.1	19.6	6.9	2.7	0.2056	4.2
4/17/2018 1:08	2	19.8	6.9	2.7	0.1931	3.8
4/17/2018 1:09	1.7	19.5	6.9	2.7	0.2071	3.5
4/17/2018 1:10	0.8	19.7	6.9	2.7	0.1911	3.5
4/17/2018 1:11	2.5	19.6	7	2.7	0.1983	3.6
4/17/2018 1:12	2.7	19.6	6.9	2.7	0.2037	3.9
4/17/2018 1:13	2.3	20.1	6.8	2.7	0.196	3.9
4/17/2018 1:14	3.1	19.8	6.8	2.7	0.209	4
4/17/2018 1:15	2.6	19.8	6.8	2.7	0.1879	4.2
4/17/2018 1:16	2.3	19.9	6.8	2.7	0.208	4.2
4/17/2018 1:17	3.4	20.2	6.8	2.7	0.1939	4.2
4/17/2018 1:18	3.2	19.9	6.7	2.7	0.2048	4.1
4/17/2018 1:19	1.8	20	6.7	2.7	0.205	4
4/17/2018 1:20	2.9	19.9	6.7	2.7	0.1937	4
4/17/2018 1:21	2.7	20.2	6.6	2.7	0.2004	3.9
4/17/2018 1:22	3	20.2	6.6	2.7	0.1932	4
4/17/2018 1:23	3.6	20.3	6.6	2.7	0.2018	4
4/17/2018 1:24	3.5	20.6	6.6	2.7	0.1946	3.9
4/17/2018 1:25	1.9	20.8	6.6	2.7	0.1976	3.7
4/17/2018 1:26	2.5	20.4	6.6	2.6	0.2103	3.8
4/17/2018 1:27	2.7	20.8	6.5	2.6	0.1873	3.8
4/17/2018 1:28	3.5	20.8	6.4	2.6	0.2098	3.9
4/17/2018 1:29	2.9	20.6	6.3	2.6	0.1921	3.9
4/17/2018 1:30	2.8	20.8	6.3	2.5	0.2003	3.8
4/17/2018 1:31	3.8	20.5	6.2	2.5	0.2004	3.9
4/17/2018 1:32	2.9	20.6	6.2	2.5	0.1917	3.9
4/17/2018 1:33	3.1	20.8	6.2	2.5	0.2009	3.8
4/17/2018 1:34	3.8	20.2	6.2	2.5	0.1899	3.8
4/17/2018 1:35	2	20.4	6.2	2.5	0.2055	3.8
4/17/2018 1:36	2.1	20.5	6.2	2.5	0.19	3.1
4/17/2018 1:37	2.1	20.2	6.1	2.5	0.2025	2.5
4/17/2018 1:38	4.3	20.4	6.1	2.5	0.1974	3.2
4/17/2018 1:39	2.8	20	6.2	2.6	0.1998	3.4
4/17/2018 1:40	3.4	19.8	6.1	2.6	0.2051	3.5
4/17/2018 1:41	4.1	20.1	6.1	2.6	0.1922	3.7
4/17/2018 1:42	2.8	20.1	6.1	2.6	0.2028	3.7
4/17/2018 1:43	2.1	19.9	6	2.6	0.1963	3.8
4/17/2018 1:44	3.2	20.3	6	2.6	0.1868	3.8
4/17/2018 1:45	3	20.2	6	2.6	0.2008	3.9
4/17/2018 1:46	2.4	20.3	5.9	2.6	0.1898	4
4/17/2018 1:47	2.9	20.2	5.9	2.6	0.2021	4.1
4/17/2018 1:48	2.3	20.4	5.9	2.6	0.1855	4.1

4/17/2018 1:49	3.8	20.4	5.9	2.6	0.1972	4.1
4/17/2018 1:50	2.7	20.5	5.8	2.6	0.1991	4.2
4/17/2018 1:51	2	20.4	5.8	2.6	0.1878	4.3
4/17/2018 1:52	2.2	20.2	5.8	2.6	0.2055	4.2
4/17/2018 1:53	2.6	20.4	5.8	2.6	0.1882	4.1
4/17/2018 1:54	1.8	20.2	5.7	2.6	0.1997	4.1
4/17/2018 1:55	3	20.3	5.6	2.6	0.1987	4.2
4/17/2018 1:56	2.4	20.5	5.6	2.6	0.1924	4.3
4/17/2018 1:57	4.4	20.4	5.6	2.6	0.2015	4.4
4/17/2018 1:58	2.2	20.5	5.5	2.5	0.191	4.5
4/17/2018 1:59	1.3	20.8	5.6	2.5	0.2064	4.6
4/17/2018 2:00	1.5	20.7	5.5	2.5	0.1916	4.6
4/17/2018 2:01	1.7	20.7	5.4	2.5	0.1914	4.6
4/17/2018 2:02	1.6	20.5	5.4	2.5	0.2007	4.6
4/17/2018 2:03	3.4	20.3	5.5	2.5	0.1789	4.6
4/17/2018 2:04	3	20.6	5.4	2.5	0.206	4.7
4/17/2018 2:05	3.2	20.5	5.3	2.5	0.1901	4.8
4/17/2018 2:06	2.7	20.4	5.3	2.5	0.194	4.7
4/17/2018 2:07	2.3	20.6	5.3	2.5	0.195	4.8
4/17/2018 2:08	2.6	20.7	5.2	2.5	0.189	4.9
4/17/2018 2:09	2.9	20.6	5.2	2.5	0.1975	5
4/17/2018 2:10	3.2	20.6	5.2	2.5	0.1806	5
4/17/2018 2:11	2.3	20.5	5.2	2.5	0.2029	5
4/17/2018 2:12	4.6	20.8	5.2	2.5	0.1889	5
4/17/2018 2:13	2.9	20.6	5.1	2.5	0.1939	5.1
4/17/2018 2:14	3.3	20.7	5.1	2.4	0.197	5
4/17/2018 2:15	2	20.5	5.1	2.4	0.1765	5.1
4/17/2018 2:16	3.5	20.6	5.1	2.4	0.1941	5.1
4/17/2018 2:17	4	20.8	5	2.4	0.1786	5.1
4/17/2018 2:18	2.2	20.9	5	2.4	0.1954	5.1
4/17/2018 2:19	2.5	20.3	5	2.4	0.1902	5.1
4/17/2018 2:20	3.7	20.6	5	2.4	0.1803	5.2
4/17/2018 2:21	2.1	20.8	5	2.4	0.1868	5.2
4/17/2018 2:22	4.6	20.9	5	2.4	0.1759	5.3
4/17/2018 2:23	3.1	20.8	4.9	2.4	0.1923	5.2
4/17/2018 2:24	2.5	20.7	4.9	2.4	0.1853	5.1
4/17/2018 2:25	4.3	21.2	4.9	2.4	0.1956	5.1
4/17/2018 2:26	2.7	20.9	4.9	2.4	0.1962	5.1
4/17/2018 2:27	2.3	21.1	4.9	2.4	0.1892	5
4/17/2018 2:28	3.9	21.3	4.9	2.3	0.2061	4.5
4/17/2018 2:29	3.4	20.9	4.9	2.4	0.1879	4.6
4/17/2018 2:30	3.2	20.9	4.9	2.3	0.1989	4.8
4/17/2018 2:31	3.2	20.9	4.9	2.3	0.1947	4.8
4/17/2018 2:32	2.7	20.9	4.9	2.3	0.1915	4.8
4/17/2018 2:33	2.7	20.8	4.9	2.3	0.2023	4.9
4/17/2018 2:34	3.1	20.9	4.9	2.3	0.1798	4.9
4/17/2018 2:35	2.6	20.4	4.9	2.3	0.1997	5

4/17/2018 2:36	1.9	20.5	4.9	2.3	0.1864	5
4/17/2018 2:37	2.9	20.4	4.9	2.3	0.1887	5.2
4/17/2018 2:38	2.4	20.5	4.9	2.3	0.1914	5.2
4/17/2018 2:39	3.1	20.4	4.9	2.4	0.1918	5.2
4/17/2018 2:40	1.7	20.4	4.9	2.4	0.1957	5.1
4/17/2018 2:41	3.3	20.3	4.9	2.4	0.188	5.2
4/17/2018 2:42	1.9	20.4	4.9	2.4	0.1997	5.1
4/17/2018 2:43	3.3	20.4	4.9	2.4	0.1897	5.2
4/17/2018 2:44	2.6	20.2	4.9	2.4	0.1907	5.3
4/17/2018 2:45	2.2	20.1	4.9	2.4	0.2036	5.4
4/17/2018 2:46	3.7	20.4	4.9	2.4	0.1878	5.4
4/17/2018 2:47	2.8	20.4	4.9	2.4	0.1974	5.3
4/17/2018 2:48	3.4	20.2	4.9	2.5	0.1932	5.4
4/17/2018 2:49	2.1	20.1	4.9	2.5	0.1883	5.4
4/17/2018 2:50	3.6	20.3	4.9	2.5	0.2043	5.3
4/17/2018 2:51	2.3	19.9	5	2.5	0.1893	4
4/17/2018 2:52	2.3	20.3	5	2.4	0.1959	3.5
4/17/2018 2:53	1.8	20.1	5	2.5	0.1887	4.1
4/17/2018 2:54	3.4	20.2	5	2.5	0.1884	4.5
4/17/2018 2:55	2.5	20.1	5	2.5	0.1902	4.7
4/17/2018 2:56	2.2	20.1	5	2.5	0.1873	4.7
4/17/2018 2:57	2.4	20	5	2.5	0.2048	4.8
4/17/2018 2:58	2.1	20.4	5	2.5	0.1896	4.9
4/17/2018 2:59	0.7	20	5	2.5	0.1895	4.8
4/17/2018 3:00	1.8	20.1	5.1	2.5	0.1964	4.8
4/17/2018 3:01	2.4	20.3	5.1	2.5	0.1839	4.9
4/17/2018 3:02	1.9	20.2	5.1	2.5	0.1934	5
4/17/2018 3:03	3.5	20.2	5.1	2.5	0.1826	4.9
4/17/2018 3:04	2.6	20.1	5.1	2.4	0.1993	5
4/17/2018 3:05	1.8	20.2	5.1	2.5	0.1934	5
4/17/2018 3:06	2.1	20.1	5.1	2.5	0.1866	5
4/17/2018 3:07	2.3	19.9	5.1	2.5	0.1928	4.9
4/17/2018 3:08	2.5	20	5.1	2.5	0.1879	4.9
4/17/2018 3:09	1.9	19.7	5.1	2.5	0.1955	4.9
4/17/2018 3:10	3.7	20	5.1	2.5	0.1919	5.1
4/17/2018 3:11	3.3	20	5.1	2.5	0.1871	5.2
4/17/2018 3:12	3.7	20.2	5.2	2.5	0.197	5.3
4/17/2018 3:13	2.5	20	5.1	2.5	0.19	5.3
4/17/2018 3:14	3	20	5.2	2.5	0.199	5.4
4/17/2018 3:15	2.7	19.9	5.2	2.5	0.1922	5.4
4/17/2018 3:16	3.1	20	5.3	2.5	0.1958	5.4
4/17/2018 3:17	1.8	20	5.2	2.5	0.1931	5.3
4/17/2018 3:18	2.3	20.4	5.2	2.5	0.1834	5.4
4/17/2018 3:19	2.6	20.2	5.3	2.5	0.201	5.2
4/17/2018 3:20	2.1	20.1	5.2	2.5	0.1883	5.2
4/17/2018 3:21	1.2	20.2	5.2	2.5	0.1902	5.2
4/17/2018 3:22	1.7	20	5.3	2.5	0.2003	5.3

4/17/2018 3:23	2	20.1	5.3	2.5	0.1968	5.2
4/17/2018 3:24	3.6	20.2	5.3	2.4	0.1952	5.3
4/17/2018 3:25	2.3	19.8	5.4	2.4	0.1886	5.3
4/17/2018 3:26	3.4	20	5.3	2.4	0.1879	5.3
4/17/2018 3:27	2.8	20	5.3	2.4	0.1964	5.3
4/17/2018 3:28	3.2	20.1	5.3	2.4	0.1792	5.4
4/17/2018 3:29	1.7	19.9	5.3	2.4	0.197	5.3
4/17/2018 3:30	3	19.6	5.3	2.4	0.1909	5.1
4/17/2018 3:31	3	20	5.4	2.4	0.1882	5.2
4/17/2018 3:32		19.7	5.4	2.4	0.1964	5.4
4/17/2018 3:33		19.7	5.4	2.4	0.1791	5.5
4/17/2018 3:34		19.7	5.4	2.4	0.1986	5.4
4/17/2018 3:35		19.7	5.6	2.5	0.1913	5.5
4/17/2018 3:36		19.8	5.5	2.5	0.1889	5.6
4/17/2018 3:37		19.5	5.6	2.5	0.1995	5.5
4/17/2018 3:38		19.9	5.6	2.5	0.1858	5.5
4/17/2018 3:39		19.9	5.8	2.5	0.1986	5.6
4/17/2018 3:40		19.8	5.8	2.5	0.1869	5.6
4/17/2018 3:41		19.8	5.9	2.5	0.1976	5.5
4/17/2018 3:42		19.8	5.7	2.5	0.1913	5.5
4/17/2018 3:43	3.4	19.7	5.8	2.5	0.1772	4.3
4/17/2018 3:44	3.7	19.7	5.9	2.5	0.1923	3.5
4/17/2018 3:45	7.7	19.6	6	2.5	0.179	3.9
4/17/2018 3:46	3.6	19.6	5.9	2.5	0.1954	4.3
4/17/2018 3:47	3.2	19.6	5.9	2.5	0.1932	4.6
4/17/2018 3:48	3	19.5	6.2	2.5	0.1913	4.8
4/17/2018 3:49	4	19.5	6.3	2.5	0.2032	5
4/17/2018 3:50	1.7	19.7	6.1	2.5	0.1886	5.2
4/17/2018 3:51	3.1	19.6	6.3	2.5	0.1994	5.3
4/17/2018 3:52	3.3	19.6	6.3	2.5	0.189	5.4
4/17/2018 3:53	2.8	19.5	6.3	2.5	0.1939	5.4
4/17/2018 3:54	2.6	19.6	6.4	2.4	0.2008	5.3
4/17/2018 3:55	2.1	19.6	6.4	2.4	0.1763	5.3
4/17/2018 3:56	2.9	19.6	6.5	2.4	0.2009	5.3
4/17/2018 3:57	2.1	19.4	6.3	2.4	0.1866	5.2
4/17/2018 3:58	4.1	19.4	6.3	2.5	0.1891	5.3
4/17/2018 3:59	2.7	19.5	6.3	2.5	0.1915	5.3
4/17/2018 4:00	3.7	19.3	6.3	2.5	0.184	5.4
4/17/2018 4:01	1.1	19.5	6.5	2.5	0.2015	5.3
4/17/2018 4:02	4	19.7	6.5	2.5	0.1846	5.3
4/17/2018 4:03	2.4	19.6	6.6	2.5	0.1963	5.3
4/17/2018 4:04	1.7	19.8	6.6	2.5	0.1958	5.4
4/17/2018 4:05	3.1	19.6	6.6	2.5	0.186	5.5
4/17/2018 4:06	2.4	19.2	6.4	2.5	0.1981	5.5
4/17/2018 4:07	2.1	19.6	6.6	2.5	0.1891	5.4
4/17/2018 4:08	1.7	19.5	6.5	2.5	0.1988	5.4
4/17/2018 4:09	2.5	19.7	6.5	2.5	0.1915	5.4

4/17/2018 4:10	3.5	19.6	6.5	2.5	0.196	5.3
4/17/2018 4:11	2.9	19.6	6.5	2.5	0.1959	4.6
4/17/2018 4:12	2.8	19.5	6.4	2.5	0.184	3.8
4/17/2018 4:13	1.4	19.6	6.6	2.5	0.193	3.2
4/17/2018 4:14	2.2	19.6	6.5	2.5	0.1878	3
4/17/2018 4:15	2.7	19.6	6.5	2.5	0.1944	2.5
4/17/2018 4:16	2	19.7	6.4	2.5	0.1912	2.4
4/17/2018 4:17	2	19.5	6.5	2.5	0.185	2.3
4/17/2018 4:18	2.3	19.5	6.5	2.5	0.2005	2.6
4/17/2018 4:19	1.9	19.9	6.5	2.6	0.1864	2.9
4/17/2018 4:20	2	19.5	6.4	2.6	0.1901	3.1
4/17/2018 4:21	2.3	19.6	6.5	2.5	0.1971	3.3
4/17/2018 4:22	4.1	19.5	6.4	2.6	0.1815	3.5
4/17/2018 4:23	2.4	19.6	6.4	2.6	0.1969	3.6
4/17/2018 4:24	2.7	19.7	6.5	2.6	0.1873	3.8
4/17/2018 4:25	2.9	19.5	6.5	2.6	0.197	3.8
4/17/2018 4:26	1.9	19.6	6.5	2.5	0.1952	4
4/17/2018 4:27	2.7	19.5	6.5	2.5	0.1877	4.1
4/17/2018 4:28	2.4	19.6	6.5	2.5	0.1991	4.3
4/17/2018 4:29	2	19.6	6.5	2.5	0.1873	4.3
4/17/2018 4:30	1.6	19.6	6.5	2.5	0.1984	4.4
4/17/2018 4:31	2.3	19.6	6.5	2.5	0.1908	4.5
4/17/2018 4:32	2.2	19.7	6.5	2.5	0.1848	4.6
4/17/2018 4:33	3	19.7	6.5	2.5	0.1936	4.7
4/17/2018 4:34	1.5	19.8	6.5	2.5	0.1843	4.8
4/17/2018 4:35	4.1	19.4	6.5	2.5	0.1908	4.9
4/17/2018 4:36	3.2	19.7	6.5	2.5	0.1845	5
4/17/2018 4:37	3.1	19.8	6.5	2.5	0.1938	5
4/17/2018 4:38	2.7	19.9	6.5	2.5	0.19	5.1
4/17/2018 4:39	3.5	19.7	6.5	2.5	0.1823	5.1
4/17/2018 4:40	1.7	19.4	6.4	2.5	0.1969	5
4/17/2018 4:41	3.1	19.7	6.4	2.5	0.1799	5.1
4/17/2018 4:42	2.6	19.4	6.4	2.5	0.2001	5.1
4/17/2018 4:43	2	19.7	6.4	2.5	0.1875	5
4/17/2018 4:44	2.8	19.7	6.4	2.5	0.1913	5
4/17/2018 4:45	1.7	19.7	6.5	2.5	0.1954	5
4/17/2018 4:46	2.2	19.7	6.5	2.5	0.1838	5.1
4/17/2018 4:47	2.6	19.7	6.4	2.6	0.1963	5.2
4/17/2018 4:48	3.5	19.8	6.5	2.6	0.1841	5.3
4/17/2018 4:49	2.5	19.7	6.5	2.6	0.1886	5.2
4/17/2018 4:50	1.8	19.6	6.5	2.6	0.1903	5.1
4/17/2018 4:51	1.1	19.7	6.4	2.6	0.1869	5.1
4/17/2018 4:52	1.6	19.5	6.5	2.6	0.1956	5.2
4/17/2018 4:53	3.6	19.6	6.5	2.6	0.1844	5.2
4/17/2018 4:54	2.6	19.6	6.7	2.5	0.1975	5.1
4/17/2018 4:55	2.1	19.6	6.6	2.5	0.1866	5
4/17/2018 4:56	2.1	19.5	6.6	2.5	0.1892	4.9

4/17/2018 4:57	1.6	19.5	6.6	2.5	0.1937	5
4/17/2018 4:58	2.8	19.7	6.6	2.5	0.1795	5.1
4/17/2018 4:59	3.4	19.8	6.7	2.5	0.1969	4.9
4/17/2018 5:00	2.6	19.5	6.6	2.5	0.1862	4.9
4/17/2018 5:01	1.9	19.4	6.7	2.5	0.1846	4.8
4/17/2018 5:02	2.2	19.9	6.9	2.5	0.1922	4.9
4/17/2018 5:03	2.9	19.6	6.8	2.5	0.183	4.8
4/17/2018 5:04	1.8	19.4	6.9	2.5	0.198	4.8
4/17/2018 5:05	3.9	19.7	7	2.6	0.1893	4.8
4/17/2018 5:06	2	19.6	7.1	2.6	0.1912	4.8
4/17/2018 5:07	2.7	19.6	7	2.5	0.1867	4.9
4/17/2018 5:08	3.1	19.5	7	2.6	0.1912	4.8
4/17/2018 5:09	2.3	19.6	7	2.5	0.1925	4.8
4/17/2018 5:10	0.6	19.3	7.1	2.5	0.1836	4.8
4/17/2018 5:11	2.1	19.8	7.3	2.5	0.1922	4.8
4/17/2018 5:12	2.6	19.4	7.2	2.5	0.1889	4.8
4/17/2018 5:13	2.1	19.5	7.1	2.5	0.1795	4.9
4/17/2018 5:14	3	19.7	7	2.5	0.1977	4.9
4/17/2018 5:15	1.1	19.5	7	2.5	0.1792	5
4/17/2018 5:16	3.6	19.3	7.1	2.5	0.2026	5
4/17/2018 5:17	3.2	19.4	7.1	2.5	0.1872	4.9
4/17/2018 5:18	2.8	19.5	7.1	2.5	0.1874	4.8
4/17/2018 5:19	1.1	19.3	7	2.5	0.1908	4.8
4/17/2018 5:20	0.8	19.4	7.1	2.5	0.186	4.9
4/17/2018 5:21	2.3	19.3	7.2	2.5	0.1938	5
4/17/2018 5:22	2.7	19.3	7.1	2.5	0.1866	5.1
4/17/2018 5:23	2.5	19.2	7	2.5	0.178	5
4/17/2018 5:24	1.9	19.6	7.1	2.5	0.2009	5.1
4/17/2018 5:25	2.7	19.8	7.1	2.5	0.1814	5.1
4/17/2018 5:26	3.5	19.5	7.1	2.5	0.1997	5.2
4/17/2018 5:27	4	19.3	7	2.5	0.1835	5.2
4/17/2018 5:28	3	19.4	7.1	2.5	0.1853	5.2
4/17/2018 5:29	2.7	19.5	7	2.5	0.1875	5.2
4/17/2018 5:30	2.5	19.5	7	2.5	0.1741	4.3
4/17/2018 5:31	1.6	19.4	7	2.5	0.1888	3.1
4/17/2018 5:32	2.3	19.5	7	2.5	0.1796	3.1
4/17/2018 5:33	1.5	19.4	7	2.5	0.1824	2.7
4/17/2018 5:34	2.2	19.3	6.9	2.5	0.1902	2.6
4/17/2018 5:35	1.2	19.5	6.9	2.5	0.1741	2.7
4/17/2018 5:36	1.9	19.4	6.9	2.5	0.1938	2.8
4/17/2018 5:37	2.4	19.4	6.9	2.5	0.1875	2.8
4/17/2018 5:38	1.9	19.5	6.9	2.5	0.1911	2.9
4/17/2018 5:39	1.8	19.4	6.9	2.5	0.1885	3.1
4/17/2018 5:40	3	19.2	6.8	2.5	0.1774	3.1
4/17/2018 5:41	3.5	19.4	6.8	2.5	0.1928	3.1
4/17/2018 5:42	2.1	19.4	6.8	2.5	0.1798	3.2
4/17/2018 5:43	2	19.1	6.8	2.5	0.1909	3.3

4/17/2018 5:44	2.7	19.2	6.8	2.5	0.1884	3.4
4/17/2018 5:45	1.8	19.4	6.8	2.5	0.1808	3.5
4/17/2018 5:46	2.2	19.5	6.7	2.5	0.1897	3.5
4/17/2018 5:47	1.7	19.2	6.7	2.5	0.1841	3.7
4/17/2018 5:48	0.7	19.4	6.7	2.5	0.1915	3.7
4/17/2018 5:49	2	19.5	6.7	2.5	0.1872	3.7
4/17/2018 5:50	1.3	19.3	6.7	2.5	0.184	3.6
4/17/2018 5:51	2.7	19.5	6.7	2.5	0.1968	3.6
4/17/2018 5:52	2.3	19.4	6.7	2.5	0.1843	3.5
4/17/2018 5:53	1.9	19.5	6.7	2.5	0.1982	3.7
4/17/2018 5:54	3.2	19.6	6.7	2.5	0.1799	3.8
4/17/2018 5:55	4.8	19.4	6.6	2.5	0.187	3.9
4/17/2018 5:56	2.1	19.2	6.6	2.5	0.1987	4
4/17/2018 5:57	2.7	19.1	6.6	2.5	0.1845	4
4/17/2018 5:58	2.9	19.3	6.6	2.5	0.1966	4.1
4/17/2018 5:59	3.1	19.2	6.6	2.5	0.1791	4.3
4/17/2018 6:00	2	19.3	6.6	2.5	0.1944	4.3
4/17/2018 6:01	1.9	19.5	6.6	2.5	0.1902	4.4
4/17/2018 6:02	2.8	19.2	6.6	2.6	0.1819	4.5
4/17/2018 6:03	2	19.4	6.6	2.6	0.1955	4.5
4/17/2018 6:04	2.4	19.2	6.5	2.6	0.1768	4.5
4/17/2018 6:05	3.5	19.3	6.6	2.6	0.1977	4.6
4/17/2018 6:06	3.4	19.3	6.5	2.6	0.1966	4.7
4/17/2018 6:07	2.1	19.1	6.6	2.6	0.1884	4.8
4/17/2018 6:08	1.1	19.5	6.5	2.5	0.1927	4.5
4/17/2018 6:09	1.9	19.3	6.5	2.5	0.1846	4.5
4/17/2018 6:10	2.8	19.1	6.5	2.5	0.1928	4.5
4/17/2018 6:11	1.5	19.5	6.6	2.6	0.2015	4.6
4/17/2018 6:12	3.1	19.3	6.6	2.6	0.1856	4.7
4/17/2018 6:13	2.6	19.1	6.5	2.6	0.1955	4.8
4/17/2018 6:14	3.5	19.3	6.5	2.5	0.1852	5
4/17/2018 6:15	2.8	19.1	6.5	2.6	0.196	5.1
4/17/2018 6:16	3	19	6.5	2.6	0.1945	5.2
4/17/2018 6:17	1	19.1	6.6	2.6	0.1813	5.2
4/17/2018 6:18	2.2	19.3	6.6	2.6	0.1966	5.2
4/17/2018 6:19	2	19.5	6.7	2.5	0.1858	5.2
4/17/2018 6:20	1.7	19.1	6.8	2.5	0.1965	5.3
4/17/2018 6:21	2.7	19.1	6.7	2.6	0.1952	5.3
4/17/2018 6:22	2.8	19.2	6.8	2.6	0.1926	5.3
4/17/2018 6:23	2	19.1	6.8	2.6	0.2011	5.3
4/17/2018 6:24	3	18.9	6.9	2.6	0.1888	5.3
4/17/2018 6:25	1.1	19.2	7	2.6	0.1985	5.3
4/17/2018 6:26	3.7	19	6.8	2.6	0.1978	5.4
4/17/2018 6:27	2	18.9	6.9	2.6	0.1868	5.4
4/17/2018 6:28	3.8	18.9	7	2.6	0.2043	5.4
4/17/2018 6:29	2	19	7.1	2.6	0.1895	5.5
4/17/2018 6:30		19	7.5	2.6	0.2024	5.5

4/17/2018 6:31		18.9	7.5	2.6	0.1959	5.5
4/17/2018 6:32		18.9	7.5	2.7	0.1944	5.5
4/17/2018 6:33		18.8	7.3	2.7	0.2039	5.5
4/17/2018 6:34		18.9	7.3	2.7	0.195	5.5
4/17/2018 6:35		18.9	7.6	2.7	0.2042	5.6
4/17/2018 6:36		18.7	7.4	2.7	0.1957	5.6
4/17/2018 6:37	2.3	18.8	7.6	2.7	0.2037	5.6
4/17/2018 6:38	1.3	18.7	7.6	2.8	0.2025	5.6
4/17/2018 6:39	2.7	18.7	7.6	2.8	0.1902	5.6
4/17/2018 6:40	1	19	7.7	2.8	0.2066	5.5
4/17/2018 6:41	1.6	18.9	7.8	2.8	0.191	5.6
4/17/2018 6:42	1.6	19	7.6	2.8	0.2064	5.5
4/17/2018 6:43	2.6	18.9	7.7	2.8	0.2013	5.5
4/17/2018 6:44	1	18.6	7.7	2.8	0.1902	5.7
4/17/2018 6:45	1.9	18.6	7.7	2.8	0.2071	5.7
4/17/2018 6:46	2.2	18.7	7.6	2.8	0.1907	5.6
4/17/2018 6:47	1.6	18.5	7.9	2.8	0.2081	5.5
4/17/2018 6:48	2.7	18.9	7.8	2.8	0.2015	5.4
4/17/2018 6:49	3.2	18.7	7.9	2.8	0.2042	5.2
4/17/2018 6:50	1.1	18.8	7.9	2.9	0.203	4.9
4/17/2018 6:51	1.2	18.8	8	2.9	0.197	4.7
4/17/2018 6:52	1	18.8	8.1	2.9	0.2065	4.6
4/17/2018 6:53	2.5	18.8	8	2.9	0.1974	4.9
4/17/2018 6:54	1.8	18.7	8	2.9	0.199	5.2
4/17/2018 6:55	3.1	18.7	8.3	2.9	0.2058	5.3
4/17/2018 6:56	1.5	18.7	8.2	2.9	0.1888	5.4
4/17/2018 6:57	1.6	18.8	8.5	2.9	0.2113	5.5
4/17/2018 6:58	1	18.7	8.4	2.9	0.194	5.6
4/17/2018 6:59	1.9	18.8	8.3	2.9	0.2022	5.7
4/17/2018 7:00	2.1	18.8	8.3	2.9	0.1964	5.7
4/17/2018 7:01	1.6	18.7	8.1	2.9	0.1999	5.8
4/17/2018 7:02	1.5	18.5	8.2	2.9	0.2044	5.8
4/17/2018 7:03	1.6	18.5	8.3	2.9	0.1946	5.8
4/17/2018 7:04	1.6	18.7	8.4	2.9	0.2063	5.7
4/17/2018 7:05	1.7	18.7	8.4	2.9	0.1942	5.7
4/17/2018 7:06	2.3	18.6	8.5	3	0.2013	5.6
4/17/2018 7:07	1.1	18.8	8.6	3	0.2074	5.6
4/17/2018 7:08	-0.1	18.7	8.7	3	0.1974	5.6
4/17/2018 7:09	1.6	18.7	8.8	3	0.2151	5.5
4/17/2018 7:10	1.6	18.6	8.9	3	0.1976	5.5
4/17/2018 7:11	1.4	18.6	8.9	3	0.2114	5.8
4/17/2018 7:12	0.6	18.7	8.9	3	0.2108	5.8
4/17/2018 7:13	2.4	18.6	8.9	3	0.2005	5.7
4/17/2018 7:14	1.8	18.7	9.1	3	0.2175	5.7
4/17/2018 7:15	-0.6	18.7	9.1	3	0.1972	5.7
4/17/2018 7:16	1.7	18.6	9.2	3.1	0.2147	5.7
4/17/2018 7:17	2.3	18.7	9.2	3.1	0.2071	5.7

4/17/2018 7:18			1.5	18.7	9.4	3.1	0.2172	5.6
4/17/2018 7:19			1.6	18.5	9.4	3.1	0.2077	5.6
4/17/2018 7:20			1.5	18.7	9.4	3.1	0.2048	5.4
4/17/2018 7:21			3	18.5	9.5	3.2	0.2125	5.4
4/17/2018 7:22			2	18.6	9.4	3.2	0.2011	5.5
4/17/2018 7:23			1.3	18.6	9.4	3.2	0.2093	5.5
4/17/2018 7:24			2.5	18.4	9.6	3.3	0.2068	5.5
4/17/2018 7:25			0.4	18.4	9.6	3.3	0.2062	5.5
4/17/2018 7:26			2.2	18.5	9.5	3.3	0.2146	5.6
4/17/2018 7:27			1.9	18.3	9.6	3.4	0.2025	5.7
4/17/2018 7:28			2.6	18.6	9.6	3.4	0.2127	5.5
4/17/2018 7:29			0.9	18.6	9.6	3.4	0.2034	5.3
4/17/2018 7:30			2.3	18.4	9.7	3.5	0.2115	5.1
4/17/2018 7:31			2.9	18.5	9.6	3.5	0.2179	5.2
4/17/2018 7:32			1.4	18.1	9.6	3.5	0.1996	5.4
4/17/2018 7:33			1.6	18.6	9.6	3.6	0.2124	5.5
4/17/2018 7:34			2.5	18.6	9.7	3.6	0.2053	5.5
4/17/2018 7:35			0.9	18.5	9.7	3.6	0.2105	5.5
4/17/2018 7:36			0.3	18.7	9.8	3.6	0.2114	5.7
4/17/2018 7:37			2.3	18.7	9.8	3.7	0.2086	5.7
4/17/2018 7:38			1.2	18.5	10	3.7	0.2194	5.6
4/17/2018 7:39			2.4	20.2	10	3.7	0.2041	5.7
4/17/2018 7:40			3	19.9	9.9	3.7	0.2214	5.6
4/17/2018 7:41			3	17.9	9.9	3.7	0.2137	5.5
4/17/2018 7:42			0.1	18.3	9.9	3.8	0.2101	5.5
4/17/2018 7:43			2.7	18.5	9.9	3.8	0.2195	5.5
4/17/2018 7:44	0.4	0.2	0.5	18.6	10.2	3.8	0.203	5.5
4/17/2018 7:45	0.4	0.2	1.5	18.9	10.1	3.8	0.2115	5.4
4/17/2018 7:46	0.5	0	2.1	18.8	10	3.8	0.2042	5.4
4/17/2018 7:47	0.4	0.4	2.5	18.8	10	3.8	0.2129	5.2
4/17/2018 7:48	0.4	0.2	2.4	18.6	10.1	3.8	0.2232	5
4/17/2018 7:49	0.4	0.7	1.7	18.9	9.9	3.8	0.2102	4.8
4/17/2018 7:50	0.5	0.6	1.4	18.8	10	3.8	0.2169	4.7
4/17/2018 7:51	0.5	0.7	2.4	18.7	10	3.8	0.2072	4.7
4/17/2018 7:52	0.5	-0.1	2.6	18.9	10	3.8	0.2165	4.8
4/17/2018 7:53	0.5	0.2	2.6	18.8	9.9	3.8	0.2151	4.7
4/17/2018 7:54	0.6	0.6	0.8	18.6	9.9	3.8	0.2112	4.5
4/17/2018 7:55	0.6	0.2	1	18.7	10	3.8	0.2149	4.4
4/17/2018 7:56	0.6	0.3	1.4	18.7	9.9	3.8	0.213	4.4
4/17/2018 7:57	0.6	0.3	3.4	19	10	3.8	0.211	4.3
4/17/2018 7:58	0.6	0.1	2.8	19	9.9	3.8	0.2226	4.4
4/17/2018 7:59	0.6	3.2	2.1	18.7	9.9	3.8	0.2035	4.5
4/17/2018 8:00	0.6	0.6	1.7	18.6	9.9	3.8	0.2216	4.5
4/17/2018 8:01	0.6	0.7	-0.4	18.7	9.9	3.8	0.2081	4.6
4/17/2018 8:02	0.6	1	2	18.9	9.9	3.7	0.21	4.7
4/17/2018 8:03	0.5	1	2	19.1	10.4	6.4	0.2242	4.8
4/17/2018 8:04	0.5	0.2	2.5	19.9	15.3	25.4	0.3366	4.8

4/17/2018 8:05	0.5	0.7	1.2	20.9	16.4	26.3	0.4222	4.8
4/17/2018 8:06	0.5	0.6	1.6	21.7	17.6	29.4	0.5687	4.8
4/17/2018 8:07	0.4	0.9	3	22.7	18.1	31	0.7024	4.9
4/17/2018 8:08	0.4		2.6	23.7	18.2	31.1	0.8338	4.9
4/17/2018 8:09	0.2		3.3	23.2	18.1	31.3	0.858	4.8
4/17/2018 8:10	0.2		1.3	22.7	17.9	31.3	0.908	4.9
4/17/2018 8:11	0.1		1.2	22.7	17.6	31.3	0.8977	4.9
4/17/2018 8:12	0.2		3.5	22.1	17.6	31.3	0.9124	5
4/17/2018 8:13	0.2		0.9	21.9	17	31	0.834	5
4/17/2018 8:14	0.3		3	21.9	16.8	30.6	0.8732	5.1
4/17/2018 8:15	0.3		2.4	21.7	16.7	30.1	0.8836	5
4/17/2018 8:16	0.3		1.7	21.5	16.6	29.5	0.8818	5
4/17/2018 8:17	0.3		2.4	21.1	16.1	28.6	0.8452	5.1
4/17/2018 8:18	0.4		2.3	21.4	16.3	27.9	0.9024	5
4/17/2018 8:19	0.4		3.1	21.2	16.2	27.4	0.9172	4.8
4/17/2018 8:20	0.3		2.4	21.3	16.2	26.8	0.9353	5
4/17/2018 8:21	0.3		2.4	21.2	16.2	26.5	0.93	5.1
4/17/2018 8:22	0.3		2	21.2	16	26.2	0.9293	5.1
4/17/2018 8:23	0.4		0.7	20.9	15.8	25.9	0.8752	5.2
4/17/2018 8:24	0.5		1.9	20.8	15.6	25.5	0.8545	5.1
4/17/2018 8:25	0.5		1.4	21.1	15.5	25.1	0.8512	5.1
4/17/2018 8:26	0.5		1	20.9	15.6	25	0.8961	5
4/17/2018 8:27	0.5		1.7	20.6	15.5	24.6	0.8819	5.1
4/17/2018 8:28	0.4		1.7	20.7	15.3	24.3	0.839	4.8
4/17/2018 8:29	0.4		0.9	20.5	15.2	23.9	0.8701	4.8
4/17/2018 8:30	0.5		2.2	20.4	15.2	23.5	0.8422	5
4/17/2018 8:31	0.5		2.5	20.6	15.1	23.1	0.8422	4.3
4/17/2018 8:32	0.5		3	20.4	15.2	23	0.8604	4.2
4/17/2018 8:33	0.6		3.2	20.2	15.2	22.9	0.8202	3.8
4/17/2018 8:34	0.6		1.8	20.3	15.2	22.7	0.829	3.9
4/17/2018 8:35	0.6		0.1	20.3	15	22.4	0.8345	4.1
4/17/2018 8:36	0.6		1.6	20.1	14.8	22.3	0.8098	4.2
4/17/2018 8:37	0.6		2	20.4	14.6	21.9	0.7836	4.1
4/17/2018 8:38	0.6		2.8	20.1	14.6	21.6	0.776	4.1
4/17/2018 8:39	0.6		2.2	19.8	14.5	21.4	0.7975	4.3
4/17/2018 8:40	0.6		2.8	20	14.4	21.1	0.7905	4.3
4/17/2018 8:41	0.5		2.7	20.2	14.4	20.8	0.7695	4.4
4/17/2018 8:42	0.5		1.8	20.2	14.3	20.6	0.7588	4.4
4/17/2018 8:43	0.6		2.7	20	14.3	20.3	0.7525	4.4
4/17/2018 8:44	0.6		2.4	20.1	14.2	20	0.7597	4.5
4/17/2018 8:45	0.5		2.3	20	14.3	19.9	0.7866	4.7
4/17/2018 8:46	0.5		1.9	19.9	14.2	19.9	0.7551	4.7
4/17/2018 8:47	0.5		2	20	14.1	19.7	0.7521	4.7
4/17/2018 8:48	0.6		3.3	20	14.1	19.4	0.7154	4.5
4/17/2018 8:49	0.6		2.5	20	14	19.1	0.7348	4.5
4/17/2018 8:50	0.6		2.9	19.8	14	18.8	0.7224	4.5
4/17/2018 8:51	0.6		2.4	20	13.9	18.6	0.709	4.4

4/17/2018 8:52	0.6		3	19.7	13.9	18.5	0.7239	4.5
4/17/2018 8:53	0.5		2.1	19.9	13.9	18.2	0.7153	4.6
4/17/2018 8:54	0.6		2.5	19.7	13.8	17.8	0.6952	4.8
4/17/2018 8:55	0.5		2	19.9	13.8	17.7	0.7196	4.8
4/17/2018 8:56	0.6		1.6	19.8	13.7	17.5	0.6842	4.8
4/17/2018 8:57	0.5		1.2	19.8	13.7	17.4	0.696	4.8
4/17/2018 8:58	0.6		2.6	19.7	13.7	17.3	0.6784	4.7
4/17/2018 8:59	0.5		2	19.7	13.6	17.3	0.7138	4.7
4/17/2018 9:00	0.5		0.7	19.4	13.5	17.3	0.7135	4.7
4/17/2018 9:01	0.5		1.2	19.7	13.5	17.1	0.6809	4.6
4/17/2018 9:02	0.5		2.8	19.5	13.5	17	0.6905	4.7
4/17/2018 9:03	0.5		2.5	19.7	13.5	16.8	0.6518	4.5
4/17/2018 9:04	0.6		1.6	19.7	13.5	16.5	0.6572	4.5
4/17/2018 9:05	0.7		0.4	19.7	13.5	16.4	0.6496	4.5
4/17/2018 9:06	0.7		1.8	19.6	13.4	16.3	0.6761	4.6
4/17/2018 9:07	0.5		2.7	19.4	13.3	16.2	0.6804	4.7
4/17/2018 9:08	0.5		2	19.6	13.3	15.9	0.6293	4.9
4/17/2018 9:09	0.6		2.2	19.6	13.3	15.7	0.663	4.9
4/17/2018 9:10	0.6	0.8	1.7	19.6	13.3	15.6	0.6587	5
4/17/2018 9:11	0.5	1.4	2.2	19.5	13.2	15.5	0.6478	5
4/17/2018 9:12	0.5	2	2	19.5	13.2	15.5	0.6531	5.1
4/17/2018 9:13	0.5	1.2	1.9	19.5	13.2	15.4	0.622	5.2
4/17/2018 9:14	0.6	2.5	0.4	19.5	13.2	15.2	0.6332	4.8
4/17/2018 9:15	0.7	1.1	2.5	19.4	13.1	15	0.6189	4.9
4/17/2018 9:16	0.7	7.5	2.2	19.6	13.1	14.8	0.621	5
4/17/2018 9:17	0.7	7.7	1.3	19.3	13.1	14.6	0.6256	5
4/17/2018 9:18	0.7	7.3	2.1	19.8	13.1	14.4	0.5995	4.8
4/17/2018 9:19	0.8	7.4	3	19.8	13.2	14.1	0.5943	4.8
4/17/2018 9:20	1.4	7.8	3.3	21.2	14	13.6	0.4856	4.9
4/17/2018 9:21	2.3	7.9	3.2	21.3	14.3	12.6	0.4198	4.9
4/17/2018 9:22	3.2	4.1	1.6	21.7	14.1	11.7	0.3953	4.6
4/17/2018 9:23	3.9	4.2	2.1	21.9	14	10.7	0.3675	4.3
4/17/2018 9:24	4.1	5.8	4.6	21.9	13.9	9.6	0.3932	3.7
4/17/2018 9:25	4.2	4.7	2.9	22.1	13.8	8.6	0.3646	4
4/17/2018 9:26	4.3	5.3	2.1	22.5	13.8	7.6	0.36	4.2
4/17/2018 9:27	4.6	5.3	2.4	22.7	13.8	6.5	0.3457	4.4
4/17/2018 9:28	5.1	6.3	2.4	23.5	13.9	5.6	0.3197	4.6
4/17/2018 9:29	5.5	6.5	3.2	23.6	13.9	5.1	0.3212	4.7
4/17/2018 9:30	6.1	6.9	3	23.9	13.9	4.7	0.2985	4.8
4/17/2018 9:31	6.4	6.6	2.4	23.9	14.1	4.4	0.31	5
4/17/2018 9:32	6.3	5.1	4	22.3	14.4	4.2	0.3302	5
4/17/2018 9:33	5.6	5.2	3.2	22.2	15.1	4	0.3353	5
4/17/2018 9:34	4.7	4.4	3.2	22	14.9	3.8	0.3599	4.9
4/17/2018 9:35	4	3.2	2.9	21.3	14.9	14.2	0.364	5
4/17/2018 9:36	3.1	0.9	2.9	20.6	27.5	44.4	0.4369	5
4/17/2018 9:37	1.9	0.9	1.5	21.2	34.9	70.9	0.6278	5
4/17/2018 9:38	0.9	0.7	2.2	22.9	39.3	91.4	0.9329	5

4/17/2018 9:39	0.5	1.2		24.9	41.4	98	1.2313	4.9
4/17/2018 9:40	0.4	0.4		28.2	41.6	101.3	1.7905	4.9
4/17/2018 9:41	0.5	0.1		27.3	39	89.1	1.9148	4.9
4/17/2018 9:42	0.5	0.8		27.6	39.1	95.3	2.2448	5
4/17/2018 9:43	0.4	0.7		27.3	39.3	96.7	2.339	5
4/17/2018 9:44	0.4	0.8	2.3	27.1	38.5	94.6	2.3532	5
4/17/2018 9:45	0.4	0.6	3	26.5	36.6	88.9	2.2305	5
4/17/2018 9:46	0.4	0.5	4.9	26.8	37.2	91.5	2.4433	5.1
4/17/2018 9:47	0.3	0.6		26	37	92.7	2.482	5.1
4/17/2018 9:48	0.3	0.4		25.5	34.9	86.5	2.3052	5.2
4/17/2018 9:49	0.4	0.3	3.4	25.2	33.3	82	2.2538	5
4/17/2018 9:50	0.4	0.4	4.3	24.8	32.5	79.6	2.2338	4.9
4/17/2018 9:51	0.4	-0.1		25	33.4	80.6	2.3817	4.6
4/17/2018 9:52	0.4	0.4	3.7	25	33.7	80.7	2.409	4.4
4/17/2018 9:53	0.3	0.5		24.6	33.1	80.6	2.4218	4.4
4/17/2018 9:54	0.3	0.8	3.4	24.4	32.6	80.3	2.3817	4.5
4/17/2018 9:55	0.3	0.9	3.1	23.7	31.6	79.8	2.3288	4.6
4/17/2018 9:56	0.3	1.3	3.5	24	31.2	76.8	2.3028	4.6
4/17/2018 9:57	0.3	0.4	3.4	23.9	31.6	78.6	2.37	4.7
4/17/2018 9:58	0.4	1	2.4	23.5	30.5	75.7	2.2312	4.6
4/17/2018 9:59	0.4	1	3.5	23.1	28.6	68.5	2.1013	4.5
4/17/2018 10:00	0.4	0.7	2.9	23.2	28.9	68.7	2.1538	4.4
4/17/2018 10:01	0.4	0.9	2.9	23.1	29.2	71.2	2.1802	4.4
4/17/2018 10:02	0.5	0.9	2	22.8	27.3	63.8	2.0397	4.3
4/17/2018 10:03	0.5	1.3	2.9	22.6	27.9	64.1	2.1267	3.7
4/17/2018 10:04	0.5	1.5	2.8	22.5	27.7	64.4	2.1122	4.1
4/17/2018 10:05	0.5	1.2	2.4	22.7	26.7	64.3	2.0275	4.4
4/17/2018 10:06	0.5	0.8	3	22.7	28.1	65.1	2.1845	4.7
4/17/2018 10:07	0.5	1.3	3.5	22.3	27	65.4	2.0922	4.8
4/17/2018 10:08	0.4	1	2.4	22.1	27.2	65.8	2.1292	4.9
4/17/2018 10:09	0.4	0.6	3.8	22.2	26.8	66.1	2.0942	5
4/17/2018 10:10	0.4	0.6	3.2	22.2	26.4	66.2	2.0583	5.1
4/17/2018 10:11	0.4	0.7	3	21.8	25.9	66	2.0098	5.2
4/17/2018 10:12	0.5	0.5	3.6	22.1	25.8	65.6	2.046	5.1
4/17/2018 10:13	0.4	0.3	2.2	21.7	25.4	65.5	1.9843	5
4/17/2018 10:14	0.4	0.5	2.9	21.9	24.9	60.9	1.9483	4.9
4/17/2018 10:15	0.5	0.9	4.1	21.8	24.6	60.2	1.9363	4.8
4/17/2018 10:16	0.5	1.1	1.8	21.9	25.1	62.2	2.0003	4.9
4/17/2018 10:17	0.5	1.3	2.5	21.2	24	57.5	1.9058	5.1
4/17/2018 10:18	0.4	1.2	-0.7	21.8	24.4	57.7	1.9618	5.1
4/17/2018 10:19	0.4	1	2.1	21.5	23.6	57.7	1.86	5.1
4/17/2018 10:20	0.5	1.3	2	21.4	23.1	57.5	1.8177	5
4/17/2018 10:21	0.5	1.4	2.5	21.5	23.3	57.6	1.858	4.9
4/17/2018 10:22	0.6	0.7	2.8	21.3	22.9	57.5	1.809	4.9
4/17/2018 10:23	0.6	1.3	1	21.2	23	57.5	1.8357	4.7
4/17/2018 10:24	0.6	1.2	2.5	20.9	22	57	1.759	3.9
4/17/2018 10:25	0.6	0.5	2	21.1	22.4	56.6	1.8077	3.9

4/17/2018 10:26	0.6	0.9	1.8	21.1	22.1	56.1	1.7818	3.4
4/17/2018 10:27	0.5	0.5	2.7	21.1	21.2	55	1.725	3.1
4/17/2018 10:28	0.5	-0.1	2.4	21.2	21.3	50.2	1.7188	3.3
4/17/2018 10:29	0.5	1.6	2.4	20.9	21.6	52.5	1.7397	3.5
4/17/2018 10:30	0.5	1	1.9	21	21	50.3	1.6818	3.5
4/17/2018 10:31	0.5	1.4	3.7	20.9	20.9	49.4	1.6965	3.6
4/17/2018 10:32	0.6	0.7	2.3	20.9	20.9	49.6	1.6897	3.7
4/17/2018 10:33	0.6	1.4	2.5	20.9	20.4	49.4	1.6465	3.8
4/17/2018 10:34	0.6	1.4	3	20.8	20.5	49.2	1.6705	3.9
4/17/2018 10:35	0.6	0.5	1.3	21	20	49.1	1.6928	3.9
4/17/2018 10:36	0.6	1.4	1.8	20.8	20	49	1.6492	3.9
4/17/2018 10:37	0.7	1.1	2.4	20.7	19.6	48.6	1.5867	3.8
4/17/2018 10:38	0.7	0.7	2.3	20.6	19	48	1.5183	3.8
4/17/2018 10:39	0.7	1.3	5.7	20.7	19.7	47.7	1.6328	3.9
4/17/2018 10:40	0.7	1	2.8	20.8	19.6	47.2	1.6077	4
4/17/2018 10:41	0.6	1.3	2.9	20.8	19.5	47.1	1.614	4
4/17/2018 10:42	0.6	1.2	3	20.7	18.6	46.7	1.513	4
4/17/2018 10:43	0.6	0.7	2.4	20.6	18.5	46.3	1.5145	4.1
4/17/2018 10:44	0.7	0.9	3.4	20.7	18.7	45.6	1.5645	4.2
4/17/2018 10:45	0.6	0.8	1.4	20.6	18.3	45.2	1.4832	4.3
4/17/2018 10:46	0.6	0.9	1.8	20.7	18.9	45.2	1.5808	4.3
4/17/2018 10:47	0.5	0.8	2	20.4	18.5	44.8	1.5083	4.4
4/17/2018 10:48	0.6	0.9	2.2	20.3	17.6	44.1	1.4232	4.3
4/17/2018 10:49	0.8	1	2.1	20.4	17.3	43.2	1.4122	4.3
4/17/2018 10:50	0.8	1	2.4	20.6	17.4	42.4	1.4178	4.4
4/17/2018 10:51	0.7	1.2	2.4	20.4	18	41.9	1.43	4.4
4/17/2018 10:52	0.9	2.1	2.4	21.1	17.1	37.8	1.2938	4.4
4/17/2018 10:53	1.5	4.3	3.2	22.6	15.5	28.9	1.0706	4.6
4/17/2018 10:54	3	6	1.1	23.6	12.8	16.5	0.7905	4.6
4/17/2018 10:55	4.5	6.3	3	23.8	12.3	14.5	0.7128	4.7
4/17/2018 10:56	5.7	7.2	3.8	24.3	11.9	13.1	0.6562	4.7
4/17/2018 10:57	6.2	6.7	4	24.6	11.6	12.7	0.604	4.8
4/17/2018 10:58	6.7	7.7	4.3	25.1	11.5	12.3	0.5802	4.8
4/17/2018 10:59	7.1	8.4	5.1	25.5	11.1	11.8	0.5462	4.8
4/17/2018 11:00	7.5	7.7	3.1	25.3	11	11.1	0.5285	5
4/17/2018 11:01	7.4	8.3	3.9	25.4	11.4	10.6	0.5577	5
4/17/2018 11:02	7.5	7.7	3.6	25.3	11	9.9	0.5121	4.9
4/17/2018 11:03	7.6	8.2	4	25.9	11.2	9.2	0.5141	4.8
4/17/2018 11:04	8.1	8.5	4.9	26.5	10.7	8.4	0.4804	5
4/17/2018 11:05	8.6	9.6	6.2	26.9	10.3	7.8	0.444	5.1
4/17/2018 11:06	8.7	7.9	3.6	24.8	11.1	7.3	0.4767	5.3
4/17/2018 11:07	7.5	4.6	1.5	22.5	15.3	7	0.5932	5.3
4/17/2018 11:08	5.9	5.1	3	22.6	15.7	6.8	0.5833	5.4
4/17/2018 11:09	3.9	1.3	3.6	23.9	42.6	55.1	0.768	5.2
4/17/2018 11:10	2.4	0.9	4.4	26.5	56.8	124.7	1.1052	5.1
4/17/2018 11:11	1	1.3	5.2	29.1	70.1	172.8	1.2757	5.1
4/17/2018 11:12	0.2	1.2	5.3	33.1	83	206.4	1.6257	5.1

4/17/2018 11:13	0.2	1.5	5.9	43.4	94	221.9	2.1972	5.1
4/17/2018 11:14	0.2	1.1	6.2	47.9	96.3	232.2	2.5788	4.8
4/17/2018 11:15	0.3	1.9	6.8	58.5	105.5	229.1	3.2883	4.9
4/17/2018 11:16	0.3	1.6	9.1	66.5	112.5	246.1	3.8663	5.1
4/17/2018 11:17	0.3	1.7	9.7	70.1	111.5	244.1	4.3005	5.2
4/17/2018 11:18	0.2	2.4	9.3	70.8	111.4	240	4.6283	5.3
4/17/2018 11:19	0.2	2.2	7.8	67	103.5	231.4	4.6987	5.4
4/17/2018 11:20	0.3	1.8	7.8	69.3	103.5	223.3	4.9728	5.4
4/17/2018 11:21	0.3	1.6	9	73.6	105.5	220.1	5.4102	5.4
4/17/2018 11:22	0.3	1.9	8.7	73.8	101.9	229.5	5.5845	5.5
4/17/2018 11:23	0.3	2	9	75.7	101.2	225.9	5.8655	5.6
4/17/2018 11:24	0.3	2.1	10.5	81.4	99.9	220.2	6.4305	5.7
4/17/2018 11:25	0.3	1.7	10	79.1	94	217.3	6.414	5.7
4/17/2018 11:26	0.3	1.9	10.1	79.5	94.2	216.4	6.6648	5.9
4/17/2018 11:27	0.3	1.7	11.5	82.8	92.7	216.4	6.9843	5.9
4/17/2018 11:28	0.3	1.3	10	82.6	92	225.4	7.3423	6
4/17/2018 11:29	0.2	1.7	9.7	84.3	92.8	222.4	7.6639	6
4/17/2018 11:30	0.2	1.3	9.4	75.2	85.6	214.5	7.2447	6.2
4/17/2018 11:31	0.2	1.6	8.6	72	82.8	204.7	6.8953	6.1
4/17/2018 11:32	0.3	1.8	9.8	74.1	81.7	203.4	7.3578	6.2
4/17/2018 11:33	0.2	1.7	8.9	74.1	81.4	214.1	7.7595	6.3
4/17/2018 11:34	0.2	2	8.8	70.6	80.1	204.9	7.7	6.2
4/17/2018 11:35	0.2	1	9.2	69.7	77.4	200.5	7.4899	5.9
4/17/2018 11:36	0.2	1.1	8.3	67.9	79.4	199.9	7.7248	5.7
4/17/2018 11:37	0.1	1.3	8.1	68	79.5	189.3	7.8541	5.4
4/17/2018 11:38	0.2	0.9	8.9	65	77.1	198.1	7.8978	5.8
4/17/2018 11:39	0.2	1.4	8.6	68.6	75.7	198	8.2605	5.8
4/17/2018 11:40	0.2	1.5	8	58.4	67.8	187.8	7.5761	5.9
4/17/2018 11:41	0.2	2	5.9	57.8	69.6	176.5	7.568	6
4/17/2018 11:42	0.2	1.8	9.2	61.6	71.2	187.9	8.1764	6.2
4/17/2018 11:43	0.1	1.4	6.6	61.7	70.5	186	8.309	6.3
4/17/2018 11:44	0.1	1.3	7.5	54.9	67.8	179.2	7.9843	6.3
4/17/2018 11:45	0.1	1.7	7.9	53.6	62.7	167.1	7.6228	6.2
4/17/2018 11:46	0.2	1.5	6.8	55.2	63.6	178.1	7.9362	6.3
4/17/2018 11:47	0.2	1	6.8	54.2	64.2	181.4	8.1435	6.3
4/17/2018 11:48	0.1	1.3	6.6	50.4	58.8	164.9	7.4996	6.3
4/17/2018 11:49	0.1	1.4	7.9	50.6	59	166.8	7.7375	6.3
4/17/2018 11:50	0.1	1.5	6.4	48.7	57.4	163.7	7.6392	6.2
4/17/2018 11:51	0.1	1	8	49.6	57.2	168.4	8.0645	6.1
4/17/2018 11:52	0.1	1.4	6.6	49.5	58.2	170.4	8.042	6.1
4/17/2018 11:53	0.1	1.3	6.6	45.9	55.3	162.1	7.7158	6
4/17/2018 11:54	0.2	0.9	7.1	47.4	56.4	162.1	8.1346	6
4/17/2018 11:55	0.2	1.4	5.4	44.9	53.4	157.6	7.6276	6
4/17/2018 11:56	0.1	1.2	6.6	46.3	53	155.4	7.9112	6
4/17/2018 11:57	0.1	0.8	5.8	45	54.2	162	8.1144	6.1
4/17/2018 11:58	0.1	1.6	5.6	44.4	53.3	153.6	8.1602	6.1
4/17/2018 11:59	0.1	2	5.7	44.4	52.6	159.2	8.0926	6.2

4/17/2018 12:00	0.1	1.4	5.9	45.7	53.6	161.3	8.4008	6.3
4/17/2018 12:01	0.1	1.2	6.2	41.3	51.5	154.6	7.9753	6.1
4/17/2018 12:02	0.2	1.1	6.8	43	48.4	143.9	7.7135	6
4/17/2018 12:03	0.2	1.4	4.8	40.9	50	151.8	8.053	6.1
4/17/2018 12:04	0.2	0.9	5.1	40.7	48.4	149.3	7.89	6.1
4/17/2018 12:05	0.2	1.2	3.5	39.3	46.1	144.2	7.504	6
4/17/2018 12:06	0.2	1	5	39.4	45.7	143.9	7.6505	5.8
4/17/2018 12:07	0.2	0.8	5.7	38.9	45.2	143.4	7.6443	5.7
4/17/2018 12:08	0.3	1	6.3	36.8	43.7	137	7.2958	5.8
4/17/2018 12:09	0.3	1.1	5.7	37.7	43	136	7.3348	5.9
4/17/2018 12:10	0.3	0.8	4.6	37.1	42.2	132.9	7.3027	5.7
4/17/2018 12:11	0.3	1	4.4	36.3	41.9	134.2	7.3008	5.2
4/17/2018 12:12	0.3	0.7	4.8	37.6	41.7	137	7.4416	5.4
4/17/2018 12:13	0.3	0.8	5.3	34.9	40.9	131.7	7.2119	4.7
4/17/2018 12:14	0.3	0.8	4.5	36.8	41	130	7.4415	3.7
4/17/2018 12:15	0.3	0.9	5	34.8	40.7	132.2	7.3888	3.7
4/17/2018 12:16	0.3	0.9	4.3	32.6	37.7	119.7	6.7222	3.8
4/17/2018 12:17	0.4	0.8	3	33.6	37.8	118.5	6.9589	4
4/17/2018 12:18	0.4	1.1	5.2	35.3	38.3	125.4	7.2227	4.1
4/17/2018 12:19	0.4	0.7	4.9	33.7	38.3	124.6	7.1375	4.2
4/17/2018 12:20	0.4	1	4.2	31.7	35.8	114.4	6.5664	4.1
4/17/2018 12:21	0.4	0.8	3.5	31.7	34.7	109.3	6.5126	4
4/17/2018 12:22	0.4	0.9	3.8	31.9	35.3	113.9	6.6578	4.2
4/17/2018 12:23	0.4	0.9	4	33.5	35.3	114.3	6.8533	4.1
4/17/2018 12:24	0.4	1.2	4.1	30.8	34.7	115.3	6.6098	4.2
4/17/2018 12:25	0.4	1.2	4.7	31.6	33.9	109.6	6.5943	4.1
4/17/2018 12:26	0.4	1.1	4.4	32.2	34.5	114	6.8133	4.1
4/17/2018 12:27	0.4	1.4	3	30.1	32.8	105.7	6.2849	4
4/17/2018 12:28	0.3	1.1	4.8	30.8	32.5	109.6	6.4459	4.1
4/17/2018 12:29	0.3	0.3	4.9	30.5	32.4	110.8	6.4627	4.2
4/17/2018 12:30	0.3	0.8	2.4	29.2	31.4	104.2	6.2095	4.1
4/17/2018 12:31	0.3	1.1	3.8	28.7	31	101.9	6.1748	4.2
4/17/2018 12:32	0.3	1.2	1.6	28.5	30.2	100.3	5.9991	4.2
4/17/2018 12:33	0.3	1.2	3.6	28.1	29.4	96.6	5.8441	4.2
4/17/2018 12:34	0.3	0.9	3.4	29.6	30.1	104.2	6.1436	4.2
4/17/2018 12:35	0.3	1.2		29.2	30.2	103.6	6.2433	4.3
4/17/2018 12:36	0.3	1.4		27.2	27.9	94.4	5.4743	4.2
4/17/2018 12:37	0.4	1.9		22.3	28.1	88.2	4.4741	4.1
4/17/2018 12:38	0.8	3		20.6	28	37.6	2.6284	4
4/17/2018 12:39	1.3	3.1		21	23.4	28.9	2.3849	4
4/17/2018 12:40	1.7	2.2		20.8	18	28.8	2.3715	4.1
4/17/2018 12:41	1.8	3.5		20.9	17.4	23.1	2.1159	4
4/17/2018 12:42	1.9	3.1		21	17.4	22.2	2.0857	4
4/17/2018 12:43	2	2.7		21	17.1	22.2	2.0502	4
4/17/2018 12:44	2.1	3.8		20.9	16.4	22	1.9085	4
4/17/2018 12:45	2.2	3.6		21	15.8	21.5	1.7932	4.1
4/17/2018 12:46	2.3	4.2		21	15.5	20.9	1.7103	4.1

4/17/2018 12:47	2.6	4.4	9.1	21.2	15.1	20.1	1.6013	4.2
4/17/2018 12:48	2.8	3.7	2.4	21.4	14.9	19.3	1.5463	4.1
4/17/2018 12:49	3	4.3	1.3	21.6	15.1	18.5	1.6071	4.1
4/17/2018 12:50	3	4.5	2.1	21.6	14.6	17.6	1.4863	4
4/17/2018 12:51	3.1	4.5	2.8	21.8	14.2	16.4	1.4613	4
4/17/2018 12:52	3.2	4.5	2.4	21.7	14.6	15.5	1.4843	4.1
4/17/2018 12:53	3.3	5.4	1.2	21.8	13.9	14.7	1.3068	4.1
4/17/2018 12:54	3.5	5.7	1.9	22.3	13.2	13.8	1.2378	4.1
4/17/2018 12:55	3.8	5.4	2.4	22.4	13.1	13.1	1.2268	4.2
4/17/2018 12:56	3.9	4.9	3.3	22.2	13.7	12.5	1.283	4.2
4/17/2018 12:57	3.9	5.2	2.9	22.1	13.6	12	1.2514	4.2
4/17/2018 12:58	3.7	4.4	2.6	21.9	14.4	11.3	1.2317	4.1
4/17/2018 12:59	3.8	5.5	3.1	22.5	14.2	10.8	1.1534	4.3
4/17/2018 13:00	3.9	5.1	1.7	22.8	13	10.3	1.1008	4.3
4/17/2018 13:01	4.1	5.6	2.8	22.8	12.9	9.8	1.1139	4.4
4/17/2018 13:02	4.4	6.4	1.7	22.7	12	9.4	1.0215	4.4
4/17/2018 13:03	4.6	6.3	1.5	22.8	12.1	9	0.9513	4.5
4/17/2018 13:04	4.8	5.8	1.8	23	12	8.5	0.928	4.4
4/17/2018 13:05	5	6.6	1.2	23.8	11.6	8	0.926	4.5
4/17/2018 13:06	5.1	5.8	3.3	23.2	11.9	7.4	0.9109	4.6
4/17/2018 13:07	5.3	7.2	2.8	23.7	11.2	7	0.8583	4.6
4/17/2018 13:08	5.6	7.8	3.8	24.3	10.8	6.6	0.8316	4.7
4/17/2018 13:09	5.9	8	2.1	24.2	10.6	6.1	0.7554	4.6
4/17/2018 13:10	5.9	6.8	1.3	23.8	10.7	5.6	0.784	4.6
4/17/2018 13:11	6.2	8.5	2.7	24.8	9.6	5.2	0.661	4.4
4/17/2018 13:12	6.6	8.9	3.3	25.3	9.2	4.9	0.6381	4.4
4/17/2018 13:13	7	9	1	24.8	9.4	4.5	0.6511	4.5
4/17/2018 13:14	6.9	7.5	1.9	24.7	10.2	4.2	0.6591	4.6
4/17/2018 13:15	6.7	8.2	3.7	24.9	9.7	3.8	0.6316	4.6
4/17/2018 13:16	6.6	8.3	2.7	24.7	9.1	3.5	0.5841	4.7
4/17/2018 13:17	6.8	8.1	2.6	24.8	8.9	3.3	0.5743	4.7
4/17/2018 13:18	7	7.9	2.6	24.9	8.4	3.1	0.5354	4.7
4/17/2018 13:19	7.1	8.8	2.8	25.3	8.5	2.9	0.5289	4.7
4/17/2018 13:20	7.4	9.3	3.4	25.5	7.9	2.8	0.495	4.8
4/17/2018 13:21	7.7	10	2.9	26.2	7.5	2.7	0.4884	4.8
4/17/2018 13:22	8.3	9.3	2.7	26.3	6.9	2.6	0.4474	4.7
4/17/2018 13:23	8.4	9.2	3	26.2	7.3	2.5	0.4532	4.8
4/17/2018 13:24	8.6	10.9	4.1	26.8	6.9	2.4	0.4353	4.8
4/17/2018 13:25	8.8	10.4	3.4	27	6.4	2.3	0.4215	4.9
4/17/2018 13:26	9.3	10.7	4	27	6	2.3	0.3938	4.9
4/17/2018 13:27	9.4	9.1	3.3	26.4	6.5	2.2	0.3999	5
4/17/2018 13:28	9.3	9.7	2.6	26.4	6.4	2.2	0.3794	5
4/17/2018 13:29	9	10.4	2.1	26.3	6.2	2.1	0.3679	5
4/17/2018 13:30	9	10.3	3.4	26.6	6	2	0.3492	5
4/17/2018 13:31	9.1	10.8	2.7	26.7	5.8	2	0.3586	5.1
4/17/2018 13:32	9.2	11.1	3.6	26.9	5.7	1.9	0.332	5.2
4/17/2018 13:33	9.4	11.7	4.5	27.2	5.5	1.8	0.3129	5.2

4/17/2018 13:34	9.7	10.4	2.3	27.3	5.2	1.8	0.3244	5.1
4/17/2018 13:35	9.6	10.6	3.7	26.4	5.5	1.7	0.3182	5.3
4/17/2018 13:36	9.3	8.9	5.7	28.8	5.5	1.7	0.322	5.2
4/17/2018 13:37	9.2	11	7.5	24.9	5.6	1.7	0.3299	5.2
4/17/2018 13:38	9.7	11.4	8.8	26	5.7	1.7	0.3236	5
4/17/2018 13:39	10	10.6	6.7	26.8	5.7	1.7	0.339	4.9
4/17/2018 13:40	10	10.2	7.1	26.7	5.7	1.7	0.3313	5
4/17/2018 13:41	9.7	9.7	6.6	26.9	5.6	1.7	0.3444	5
4/17/2018 13:42	9.3	9.4	7	26.5	5.7	1.8	0.3327	5
4/17/2018 13:43	9	9.6	6.9	26.4	5.7	1.8	0.3481	5.1
4/17/2018 13:44	8.6	8.4	6.9	26	5.7	1.9	0.3481	5.3
4/17/2018 13:45	8.2	8.3	6.3	25.7	5.7	1.9	0.341	5.3
4/17/2018 13:46	7.9	8.6	6.3	25.3	5.7	1.9	0.3618	5.3
4/17/2018 13:47	7.6	7.9	6.5	25.1	5.7	2	0.3418	5.3
4/17/2018 13:48	7.4	7.7	4.8	25.2	5.7	2	0.3543	5.2
4/17/2018 13:49	7.1	7.3	6.7	24.7	5.7	2.1	0.3509	5.1
4/17/2018 13:50	6.4	3.8	4.8	23.6	11.1	14.7	0.4111	5.3
4/17/2018 13:51	5	1.7	3.9	22.1	18.3	37.4	0.4515	5.4
4/17/2018 13:52	3	1.5	3.9	22	24.8	57.5	0.6063	5.4
4/17/2018 13:53	1.4	1.1	5.3	22.6	30.4	76.4	0.9878	5.4
4/17/2018 13:54	0.6	0.6	6.1	23.3	32	79.4	1.28	5.4
4/17/2018 13:55	0.4	0.4	4.4	24.2	32.6	82.5	1.736	5.3
4/17/2018 13:56	0.3	0.6	5.9	24.6	32.8	84.4	1.963	5.3
4/17/2018 13:57	0.3	1	5.2	25.4	31.9	83.9	2.2465	5.2
4/17/2018 13:58	0.3	1.2	5.4	24.7	31.5	83.6	2.3745	5.4
4/17/2018 13:59	0.3	0.1	4.4	24.4	30.5	78	2.4212	5.5
4/17/2018 14:00	0.3	0.4	3.3	24	30.6	78.4	2.5217	5.4
4/17/2018 14:01	0.3	0.6	5.1	24.1	29	72	2.3283	5.5
4/17/2018 14:02	0.4	0.8	4.9	24	29.2	77.4	2.4773	5.3
4/17/2018 14:03	0.4	1.2	4	24	29	75.9	2.4563	5.3
4/17/2018 14:04	0.3	0.9	3.9	23.8	28.6	74.5	2.4605	5.3
4/17/2018 14:05	0.3	0.6	5.7	23.1	27.2	72	2.2925	5.2
4/17/2018 14:06	0.4	0.8	4.7	23.4	26.4	67.5	2.2865	5.1
4/17/2018 14:07	0.4	0.9	4.5	22.9	26.9	67.4	2.3545	5.2
4/17/2018 14:08	0.4	1.3	4.3	23.1	26	66.2	2.2843	5.2
4/17/2018 14:09	0.4	0.6	3.9	22.8	25.7	64.1	2.2863	5.2
4/17/2018 14:10	0.4	1.3	5.6	22.8	25.1	63.9	2.1987	5
4/17/2018 14:11	0.4	1.1	5.6	22.7	24.9	63.8	2.1817	5.2
4/17/2018 14:12	0.5	1	3.6	22.6	24.4	63.3	2.1278	5.3
4/17/2018 14:13	0.4	0.3	4.5	22.7	25	63.2	2.2333	5.4
4/17/2018 14:14	0.4	0.7	6	22.4	24.9	63.2	2.2415	4.9
4/17/2018 14:15	0.3	0.7	4	22.4	24.3	62.9	2.2007	4.9
4/17/2018 14:16	0.3	0.4	4.6	22.4	24.1	62.6	2.166	5
4/17/2018 14:17	0.3	1.2	4.7	22.3	23.9	62.1	2.1632	5.2
4/17/2018 14:18	0.4	1	6	22.3	22.9	61.3	2.0505	5.2
4/17/2018 14:19	0.4	0.3	5.6	22.1	23	60.9	2.0728	5.2
4/17/2018 14:20	0.5	0.9	4	22	22.5	60.1	2.0193	5.4

4/17/2018 14:21	0.5	0.2	4.5	22.2	22.4	59.5	2.0355	5.3
4/17/2018 14:22	0.6	1.2	4.9	21.9	22.2	58.6	2.0035	5.3
4/17/2018 14:23	0.6	0.9	2.8	21.9	21.6	54.4	1.9043	5.3
4/17/2018 14:24	0.6	1.2	3.7	21.9	21.7	54.6	1.9765	5.1
4/17/2018 14:25	0.6	1	5.1	21.9	21	50.6	1.8663	5.3
4/17/2018 14:26	0.6	0.3	3.6	22.1	21.4	52.4	1.9472	5.3
4/17/2018 14:27	0.5	1.1	4.4	21.7	21.5	54.9	1.9755	5.1
4/17/2018 14:28	0.4	0.4	3.1	21.6	21.4	54.8	1.9698	4.5
4/17/2018 14:29	0.4	0.8	4.1	21.5	20.3	54.2	1.816	4.6
4/17/2018 14:30	0.5	1	4.1	21.4	20	53.3	1.8138	4.7
4/17/2018 14:31	0.6	0.9	3.8	21.5	19.9	52.5	1.809	4.8
4/17/2018 14:32	0.6	0.5	5.4	21.6	19.8	51.7	1.788	4.8
4/17/2018 14:33	0.6	0.7	3.1	21.7	19.1	50.8	1.7103	4.6
4/17/2018 14:34	0.6	0.8	4.6	21.6	19.4	50	1.7568	4.5
4/17/2018 14:35	0.6	1.1	3.2	21.7	18.9	46.5	1.7315	4.4
4/17/2018 14:36	0.6	1.1	3.1	21.3	18.6	45.3	1.6813	4.3
4/17/2018 14:37	0.6	1.2	3.1	21.1	18.8	44.5	1.696	4.2
4/17/2018 14:38	0.6	0.6	4.4	21.4	18.6	46.3	1.671	4.2
4/17/2018 14:39	0.6	0.9	3.9	21.2	18.6	45.9	1.6815	4.3
4/17/2018 14:40	0.5	1.3	4.3	21.2	18.7	45.6	1.6947	4.4
4/17/2018 14:41	0.5	1.1	3.5	21.4	18.6	45.2	1.6712	4.5
4/17/2018 14:42	0.5	1.1	5.2	21.1	17.8	44.6	1.6132	4.5
4/17/2018 14:43	0.6	1.6	4.2	20.9	17	43.7	1.5062	4.5
4/17/2018 14:44	0.7	0.9	3.6	21.3	17.4	43	1.5917	4.6
4/17/2018 14:45	0.7	0.6	2.8	21.2	17.4	42.3	1.6007	4.6
4/17/2018 14:46	0.7	1	2.7	21.2	17.2	41.6	1.5367	4.5
4/17/2018 14:47	0.7	1.1	3.4	21	17.2	41	1.5568	4.4
4/17/2018 14:48	0.7	0.9	4.3	21.1	17	40.5	1.5727	4.5
4/17/2018 14:49	0.7	1.3	3.9	20.9	16.7	39.9	1.4975	4.4
4/17/2018 14:50	0.8	0.8	4.4	21	16.3	39.4	1.4907	4.3
4/17/2018 14:51	0.8	1.3	4.2	20.7	16.4	39.1	1.4623	4.3
4/17/2018 14:52	0.8	1.1	3.2	20.9	16.1	38.9	1.4448	4.4
4/17/2018 14:53	0.8	1.2	3.3	20.8	16.1	38.5	1.455	4.5
4/17/2018 14:54	0.8	0.8	2.2	21.1	16.1	38	1.4658	4.6
4/17/2018 14:55	0.8	0.9	3.3	20.7	15.9	37.4	1.4322	4.4
4/17/2018 14:56	0.8	1.2	3.3	20.6	15.9	37	1.4247	4.3
4/17/2018 14:57	0.8	1.7	4.7	20.7	15.6	36.3	1.396	4.2
4/17/2018 14:58	0.8	1	4.4	20.6	15.6	36.1	1.3927	4.2
4/17/2018 14:59	0.8	1.6	5	20.8	15.4	35.8	1.3673	4.1
4/17/2018 15:00	0.9	2	3.2	21	14.9	35.2	1.3067	4.1
4/17/2018 15:01	0.9	1.4	3.7	20.4	14.9	34.6	1.3165	4.2
4/17/2018 15:02	0.9	1.8	2.5	20.6	14.9	33.9	1.3083	4.1
4/17/2018 15:03	0.8	1.1	4.3	20.6	14.9	33.4	1.3133	4.2
4/17/2018 15:04	1	1.2	3.5	20.6	14.2	32.6	1.1902	4
4/17/2018 15:05	1.6	4	4.8	21.1	13.4	30.8	0.9521	4
4/17/2018 15:06	2.6	4	5	21.9	13	18.2	0.8227	3.8
4/17/2018 15:07	3.4	4.8	4.2	22.1	12.8	14.6	0.8028	3.9

4/17/2018 15:08	4.2	6.2	6.2	23.2	11.6	13.6	0.7611	4
4/17/2018 15:09	4.9	6.9	5.1	24.1	10.7	10.7	0.6753	4.1
4/17/2018 15:10	5.9	8.4	5.3	24.6	10.2	10.3	0.5768	4.2
4/17/2018 15:11	6.8	8.5	6.1	25.1	9.9	10	0.5673	4.2
4/17/2018 15:12	7.1	7.9	6.1	24.9	10.2	9.7	0.5665	4.2
4/17/2018 15:13	7.5	8.7	5.8	25.7	9.6	9.3	0.5009	4.1
4/17/2018 15:14	7.9	9.8	6.8	26.1	9.4	8.6	0.4552	4.1
4/17/2018 15:15	8.4	9.5	7.1	26.3	9.1	7.9	0.4494	4.1
4/17/2018 15:16	8.8	10.5	7.1	27	9.2	7.2	0.444	4.3
4/17/2018 15:17	9.4	11.3	7.6	27.4	8.6	6.5	0.3916	4.3
4/17/2018 15:18	9.9	10.2	9.2	27.5	8.4	5.8	0.4076	4.1
4/17/2018 15:19	10	10.6	7.3	27.2	8.6	5.3	0.4066	4.1
4/17/2018 15:20	10	10.8	6	27.4	8.2	4.8	0.3931	4
4/17/2018 15:21	10.1	10.9	7.4	27.3	8.3	4.3	0.3805	3.9
4/17/2018 15:22	10.3	11.2	6.9	27.2	8.2	4	0.3688	4
4/17/2018 15:23	10.1	10.2	7.8	27	8.5	3.9	0.3889	4
4/17/2018 15:24	10	10.4	7.2	27.1	8.8	3.6	0.3562	4
4/17/2018 15:25	10	10.1	5.2	27.4	8.3	3.5	0.3827	3.9
4/17/2018 15:26	10.3	11.8	8.8	28.9	7.9	3.3	0.3274	4
4/17/2018 15:27	10.3	9.5	5.7	26.7	8.1	3.2	0.3777	3.9
4/17/2018 15:28	9.8	8.9	6.3	25.8	8.2	3.1	0.3794	3.9
4/17/2018 15:29	8.8	8.5	6.2	25.6	9	3.1	0.393	3.9
4/17/2018 15:30	8.3	8.1	5.2	25.4	10.5	3	0.4098	4
4/17/2018 15:31	6.5	3.2	13.3	33.7	59.5	82.1	0.696	4.1
4/17/2018 15:32	3.8	2.3	24	81.7	94.5	323.8	1.8976	4.3
4/17/2018 15:33	1.4	3.4	50.6	215.2	84.3	349.9	2.8388	4.6
4/17/2018 15:34	0.3	0.2	61.9	285.7	93.4	387.5	4.0911	5.3
4/17/2018 15:35	0.3	1.5	62.2	297	89.2	391.2	4.4487	5.7
4/17/2018 15:36	0.3	0.1	67.7	309.9	87.3	392.2	5.2723	6
4/17/2018 15:37	0.3	1.8	67.9	300.3	81.9	364.1	5.6317	6.1
4/17/2018 15:38	0.2	0.2	64.5	292.8	79.8	361.3	6.0469	6
4/17/2018 15:39	0.2	0.3	63.5	276.1	77.3	366.6	6.3017	5.7
4/17/2018 15:40	0.2	0.9	61.6	258.5	71.2	346.2	6.2391	5.8
4/17/2018 15:41	0.2	0.5	54.3	235.1	70.9	330.2	6.0773	5.8
4/17/2018 15:42	0.2	2.2	83.2	245.5	70.8	323.2	6.3933	5.8
4/17/2018 15:43	0.2	-0.4	48.6	223.5	63.7	322.5	6.6584	5.9
4/17/2018 15:44	0.2	2.5	57.6	226.5	62.5	293.3	6.7057	5.8
4/17/2018 15:45	0.2	0.1	76.3	221.6	63	321.2	7.1836	6
4/17/2018 15:46	0.3	1.9	49.8	221.5	60.5	324.1	7.2042	6
4/17/2018 15:47	0.3	0.8	51.2	210.4	60.2	318.7	7.3729	6.1
4/17/2018 15:48	0.3	0.7	52.5	194.8	56.3	297.1	7.0388	6
4/17/2018 15:49	0.3	0.7	87.1	191.8	55	300.4	7.0795	5.9
4/17/2018 15:50	0.3	1	44.4	178.1	52.5	295.4	6.8823	5.8
4/17/2018 15:51	0.3	0.6	47	182.5	53.1	290.1	7.2858	5.9
4/17/2018 15:52	0.2	1.9	67.6	167.5	49.4	281.6	6.8116	5.9
4/17/2018 15:53	0.2	1.1	42.8	168.5	49.7	285	7.1633	6
4/17/2018 15:54	0.2	1.1	39.8	149.4	47.3	276.8	6.6967	6

4/17/2018 15:55	0.2	1.8	43.4	156.1	45.2	277.2	6.8235	5.9
4/17/2018 15:56	0.2	1.3	64.3	146.1	45.3	269.9	6.8317	5.5
4/17/2018 15:57	0.2	1.5	40.2	153.4	44.7	261.5	7.1214	5.5
4/17/2018 15:58	0.2	0.3	37.6	131.9	42.2	258.6	6.5715	4.8
4/17/2018 15:59	0.2	1.1	42.3	132.8	40.3	241.1	6.4952	3.5
4/17/2018 16:00	0.2	1.1	64.4	131.1	40.2	239	6.8024	3.6
4/17/2018 16:01	0.2	1	36	126.2	39.8	253.2	6.7308	4.1
4/17/2018 16:02	0.2	1	36.7	125.6	38.7	249	6.6774	4.1
4/17/2018 16:03	0.2	0.9	57.3	123.8	37.9	239.9	6.6598	3.6
4/17/2018 16:04	0.2	0.9	38.5	123.8	37.4	247	6.7804	4.1
4/17/2018 16:05	0.2	0.6	36.6	119.9	36.4	244.3	6.7798	4.6
4/17/2018 16:06	0.2	0.9	37.6	116.9	36	241.8	6.7733	4.4
4/17/2018 16:07	0.2	-0.4	33.7	109.8	35.1	236.2	6.6062	3.6
4/17/2018 16:08	0.2	1.3	35.3	111.9	33.7	229.1	6.4932	4.2
4/17/2018 16:09	0.2	1.7	36.7	115.4	34.6	238.2	6.8736	4.6
4/17/2018 16:10	0.2	1.1	32.6	98	32.7	232	6.3753	4.1
4/17/2018 16:11	0.2	0.7	31	92.8	30.4	200.2	5.9373	3.4
4/17/2018 16:12	0.2	2.3	32.2	98.4	30.7	212.2	6.2123	3.9
4/17/2018 16:13	0.2	1	29.9	91.4	30.7	209.8	6.1788	4.1
4/17/2018 16:14	0.2	1.5	31.4	91.6	29	191.8	5.8453	3.5
4/17/2018 16:15	0.2	0.6	31.9	95	29.4	205.2	6.2606	3.4
4/17/2018 16:16	0.2	0.5	29.5	90.4	28.6	207.4	6.0354	3.9
4/17/2018 16:17	0.2	0.3	29.9	84	27.5	195	5.7277	4
4/17/2018 16:18	0.3	1.2	28.4	84.3	26.7	183.9	5.6857	3.3
4/17/2018 16:19	0.3	0.9	28.6	83.5	26.8	189.2	5.7585	3.3
4/17/2018 16:20	0.2	1	27.8	80.9	26.3	192.6	5.7278	3.8
4/17/2018 16:21	0.2	0.7	25.4	78.5	25.5	178.3	5.4851	4
4/17/2018 16:22	0.2	0.9	25.4	76.3	24.9	168.1	5.3972	3.2
4/17/2018 16:23	0.1	1	26.4	73.7	24.5	172.6	5.4072	3.5
4/17/2018 16:24	0.1	0.8	26.6	73.7	24.1	173.2	5.3172	4.1
4/17/2018 16:25	0.1	0.7	26.7	70.5	23.8	173.3	5.2843	4.5
4/17/2018 16:26	0.1	0.4	25.9	71.9	23.5	178.1	5.3007	3.5
4/17/2018 16:27	0.1	1.5	25.3	74.6	23.2	172.8	5.3479	4
4/17/2018 16:28	0.1	1.3	24.7	72.7	22.9	176.5	5.3129	4.5
4/17/2018 16:29	0.1	0.5	24.8	68.7	22.6	167.7	5.3069	4.4
4/17/2018 16:30	0.1	1.6	23.9	67.1	21.9	166	5.0297	3.4
4/17/2018 16:31	0	0.2	22.9	65.8	21.7	164.7	5.0645	4
4/17/2018 16:32	0	1	22.2	63.3	21.5	159.7	5.0479	4.4
4/17/2018 16:33	0.1	1.1	21.3	60	20.9	166.4	4.809	4.1
4/17/2018 16:34	0.1	1	19.8	59	20.1	146.1	4.5648	3.3
4/17/2018 16:35	0.1	0.7	20.7	62.7	20.2	150.8	4.8538	4.1
4/17/2018 16:36	0	0.8	20.9	59.5	20	153.6	4.7645	4.4
4/17/2018 16:37	0	1.2	21.4	58	19.4	151.6	4.5642	3.9
4/17/2018 16:38	0	1.2	22.4	62	19.7	153.2	4.893	3.5
4/17/2018 16:39	0.1	0.8	23.1	59.1	19.4	156.6	4.7792	4.3
4/17/2018 16:40	0.1	0.6	22.1	59.3	18.9	152.4	4.7072	4.7
4/17/2018 16:41	0.1	1	20.7	59.1	19.2	152.5	4.919	3.8

4/17/2018 16:42	0.1	1	19.3	56.4	19.1	137.9	4.5044	3.6
4/17/2018 16:43	0.1	0.3	18.3	58	19.1	145.7	4.7383	4.4
4/17/2018 16:44	0.2	1	20.2	54	18.2	145	2.8209	4.2
4/17/2018 16:45	0.2	0.9	17.5	48.7	17.4	125.8	0.0488	1.2
4/17/2018 16:46	0.2	1.6	16.4	45.6	17.2	121.2	-0.0216	0.1
4/17/2018 16:47	0.2	0.2	17.9	43.3	16.7	111.7	-0.0357	0.1
4/17/2018 16:48	0.2	0.7	11.6	35.1	15.9	88.1	-0.0308	0.2
4/17/2018 16:49	0.3	1	11	33	16	80.1	-0.0339	0.2
4/17/2018 16:50	0.4	1.9	7.9	21.5	16.2	50.2	-0.0439	0.2
4/17/2018 16:51	0.6	2.4	6.8	19.7	15.4	29.4	-0.0286	0.1
4/17/2018 16:52	0.9	2.5	6.1	20.2	14.8	26.6	-0.044	0.1
4/17/2018 16:53	0.9	2.2	6.8	20.2	14.7	24.1	-0.0307	0.1
4/17/2018 16:54	1	2.4	4.2	20.1	14.6	24	-0.0395	0.1
4/17/2018 16:55	1	3.1	5.7	19.5	14.5	24.1	-0.0361	0.1
4/17/2018 16:56	1	2.3	6.5	19.4	14.1	24.1	-0.0163	0.3
4/17/2018 16:57	1.1	3.2	4.2	18.6	13.3	23.6	0.1614	1.7
4/17/2018 16:58	1.2	2.5	5.2	18.8	13.3	23.1	0.1392	2.1
4/17/2018 16:59	1.2	2.5	5.3	19.4	13.2	22.7	0.1434	2
4/17/2018 17:00	1.2	2.2	4.6	19.3	13.4	22.4	0.1522	2
4/17/2018 17:01	1.3	3	4.5	19.4	13.3	21.8	0.1397	1.9
4/17/2018 17:02	1.4	3.1	3.8	18.4	12.8	20.4	0.1461	1.9
4/17/2018 17:03	1.6	3.9	3.4	18.4	11.7	18.9	0.1494	1.8
4/17/2018 17:04	1.8	3.3	5.7	19.3	12	17.4	0.1546	1.8
4/17/2018 17:05	1.9	3.5	4.5	19.2	6.6	9.2	0.7217	2.1
4/17/2018 17:06	1.9	4	5.8	19.8	0.7	0.6	0.8686	2.3
4/17/2018 17:07	1.9	4.1	5	19.1	0.7	0	0.8704	2.3
4/17/2018 17:08	2	4.4	5.5	19.1	0.6	0.1	0.7476	2.2
4/17/2018 17:09	2.1	3.9	5.2	19.2	0.6	0.1	0.7341	2.2
4/17/2018 17:10	2.2	4.3	4.7	19.1	0.6	0.1	0.66	2.3
4/17/2018 17:11	2.4	4.7	5.2	19.1	0.6	0.1	0.633	2.4
4/17/2018 17:12	2.5	4.5	5.6	19.7	0.6	0.1	0.6554	2.3
4/17/2018 17:13	2.6	5.1	5.5	19.8	0	0	0.6435	2.2
4/17/2018 17:14	2.5	4.4	5	19.9	0	0	0.6539	2.2
4/17/2018 17:15	2.6	5.6	5	20.1	0.8	7.6	0.5881	2.3
4/17/2018 17:16	2.7	5.1	4.8	20.2	8.7	5.1	0.5409	2.5
4/17/2018 17:17	3	5.2	5.9	20.5	10	4.5	0.217	2.6
4/17/2018 17:18	2.9	4.8	5	19.8	10.2	4.3	0.1403	2.7
4/17/2018 17:19	2.8	5	5	19.9	10.2	4.1	0.1493	2.8
4/17/2018 17:20	2.9	5	5.7	20.5	10	4.1	0.1387	2.2
4/17/2018 17:21	3	5	4.2	20.5	10.1	4.1	0.1432	2.5
4/17/2018 17:22	3	5.1	5.1	20.5	8.8	8.2	0.1394	2.8
4/17/2018 17:23	3.1	6.3	4.6	20.6	0.7	85.5	0.1399	2.3
4/17/2018 17:24	3.2	5.6	5.8	20.7	0.3	76.5	0.1445	2.2
4/17/2018 17:25	3.3	5	4.5	20.9	0.8	49	0.1352	2.3
4/17/2018 17:26	3.3	5.4	6.7	20.7	1.6	35.3	0.1462	2.3
4/17/2018 17:27	3.3	5.9	5.3	21	2.3	27.9	0.1453	2.4
4/17/2018 17:28	3.3	5.6	5.8	21.2	2.9	25.6	0.136	2.4

4/17/2018 17:29	3.5	6.8	7.2	20.7	3.7	25.3	0.1473	2.2
4/17/2018 17:30	3.7	5.9	4.9	21	4.2	24.8	0.1296	2.2
4/17/2018 17:31	3.8	6.6	3.5	21	4.7	24.6	0.1918	2
4/17/2018 17:32	3.8	6.1	6.7	21.2	8	16.1	0.3621	1.9
4/17/2018 17:33	3.9	6.2	5.2	21.2	5.3	29.6	0.3557	1.7
4/17/2018 17:34	4	6.8	5.4	21.5	7	36.5	0.3473	1.8
4/17/2018 17:35	4.2	7.1	4.5	21.8	7.9	46.7	0.3264	2
4/17/2018 17:36	4.3	6.9	4.9	26.8	7.8	52.5	0.331	2
4/17/2018 17:37	5	10.5	7	18.8	7.4	56.1	0.3256	2
4/17/2018 17:38	5.9	10	7	22.9	6.9	71.5	0.299	2.2
4/17/2018 17:39	6.9	11.5	8.8	25.3	6.4	77	0.3007	2.2
4/17/2018 17:40	7.4	11.8	6.7	25.8	5.8	82.6	0.2812	2.3
4/17/2018 17:41	7.6	11.8	8.7	25.9	5.2	85.4	0.2881	2.3
4/17/2018 17:42	7.8	12.4	9.3	25.9	4.7	85.9	0.2698	2.3
4/17/2018 17:43	7.9	12.2	8.3	26	4.5	86.5	0.2692	2.4
4/17/2018 17:44	8.1	12.5	7.1	26.1	4.5	87.4	0.2699	2.4
4/17/2018 17:45	8.1	11.7	7.5	26.1	3.8	88.5	0.2487	2.3
4/17/2018 17:46	8.3	12.3	7.3	26.5	3.1	89.8	0.2593	2.3
4/17/2018 17:47	8.5	12.3	7.5	26.8	2.9	90.9	0.2374	2.4
4/17/2018 17:48	8.7	13.7	8.3	27.8	2.7	92.4	0.2377	2.3
4/17/2018 17:49	9.4	14.5	9.8	28.1	2.6	93.9	0.2254	2.4
4/17/2018 17:50	9.6	12.4	9.3	26.9	2.4	94.9	0.2526	2.3
4/17/2018 17:51	9.5	12.2	7.9	26.9	2.3	96	0.2519	2.4
4/17/2018 17:52	8.6	11.6	8.6	25.9	2.2	96.9	0.2323	2.3
4/17/2018 17:53	8.3	10.5	8	25.5	2.1	97.6	0.2462	2.4
4/17/2018 17:54	7.6	9.2	7.1	24.7	2.1	98	0.2302	2.3
4/17/2018 17:55	7.2	9	5.9	24.3	2	98.5	0.2487	2.3
4/17/2018 17:56	6.5	8.3	4.6	23.5	2	98.9	0.2392	2.5
4/17/2018 17:57	6.4	9	5.6	23.9	1.9	99.7	0.2213	2.6
4/17/2018 17:58	6	7	5	22.8	1.7	96.5	0.2473	2.6
4/17/2018 17:59	5.7	6.8	7.1	22.5	8.7	41.7	0.2302	2.4
4/17/2018 18:00			4.7	22.1	68.3	46.3	0.2377	2.1
4/17/2018 18:01			5.5	22.6	85.6	20.1	0.2677	2.3
4/17/2018 18:02			4.2	22.1	87.2	17.6	0.2515	2.6
4/17/2018 18:03			4.6	22.4	87.3	17.9	0.2576	2.7
4/17/2018 18:04			4.7	22.1	87.4	18.1	0.2305	2.7
4/17/2018 18:05			2.2	22.1	87.5	18.1	0.2638	2.7
4/17/2018 18:06			5.4	21.7	79	18.1	0.2437	2.8
4/17/2018 18:07			5.7	21.8	8.7	10.3	0.2462	2.8
4/17/2018 18:08			5.1	21.6	4.4	3	0.1613	2.8
4/17/2018 18:09			5.1	21.6	4.5	2.6	0.1347	2.4
4/17/2018 18:10			6.2	21.8	4.4	2.7	0.147	2.5
4/17/2018 18:11			5.5	21.5	4.3	2.9	0.1363	2.5
4/17/2018 18:12			4.1	21.5	4.4	2.8	0.1545	2.7
4/17/2018 18:13			4.3	21.1	4.3	2.8	0.148	2.8
4/17/2018 18:14			4.9	21	4.2	2.8	0.1469	2.6
4/17/2018 18:15			3.7	21.1	4.3	2.8	0.1393	2.6

4/17/2018 18:16	3.5	21	4.2	2.8	0.1368	2.7
4/17/2018 18:17	4.5	21	4.1	2.8	0.1532	2.7
4/17/2018 18:18	4.2	21	4.1	2.8	0.1371	2.9
4/17/2018 18:19	4.8	21	4.1	2.8	0.2181	3.5
4/17/2018 18:20	4.2	20.7	4.1	2.8	0.2368	3.7
4/17/2018 18:21	3.2	21	4	2.8	0.2232	3.7
4/17/2018 18:22	2.6	21	4.1	2.8	0.2215	3.9
4/17/2018 18:23	3.1	20.7	4.1	2.8	0.201	4
4/17/2018 18:24	4.8	20.8	4.2	2.7	0.2077	3.9
4/17/2018 18:25	5	21	4	2.7	0.2164	4
4/17/2018 18:26	3.2	20.9	4	2.7	0.208	4
4/17/2018 18:27	3.2	20.4	4	2.7	0.2134	4.1
4/17/2018 18:28	4.8	20.8	4	2.7	0.2036	4.1
4/17/2018 18:29	4.8	20.8	4	2.7	0.2156	4.1
4/17/2018 18:30	2.8	20.9	4	2.7	0.2045	4.2
4/17/2018 18:31	4.1	20.8	4	2.7	0.2095	4.2
4/17/2018 18:32	4	20.5	4.1	2.7	0.199	4.1
4/17/2018 18:33	3.5	20.6	4.1	2.7	0.203	4.1
4/17/2018 18:34	3.5	20.8	4.1	2.7	0.2054	4.1
4/17/2018 18:35	4.9	21	4	2.7	0.1942	4
4/17/2018 18:36	5.7	20.2	4.1	2.7	0.2041	4.2
4/17/2018 18:37	3.1	20.3	4.1	2.7	0.1955	4.2
4/17/2018 18:38	3.6	20.4	4.1	2.7	0.1987	4.1
4/17/2018 18:39	3.3	20.5	4.1	2.7	0.2032	4.1
4/17/2018 18:40	4.3	20.7	4.1	2.7	0.1936	3.9
4/17/2018 18:41	4.7	20.5	4.3	2.6	0.1975	3.9
4/17/2018 18:42	3.6	20.7	4.2	2.6	0.1924	3.5
4/17/2018 18:43	5.1	20.9	4.2	2.6	0.2018	3.7
4/17/2018 18:44	4	20.5	4.3	2.5	0.1787	4
4/17/2018 18:45	3.6	20.5	4.3	2.5	0.2025	4
4/17/2018 18:46	3.7	20.5	4.1	2.5	0.1987	4
4/17/2018 18:47	3.1	20.6	3.8	2.5	0.192	3.9
4/17/2018 18:48	3.4	20.7	3.9	2.5	0.2063	4
4/17/2018 18:49	3.4	20.8	4	2.5	0.1806	4
4/17/2018 18:50	2.9	20.4	3.9	2.5	0.1993	3.9
4/17/2018 18:51	3.5	20.5	3.9	2.5	0.1838	3.9
4/17/2018 18:52	3.5	20.5	4.1	2.5	0.1947	3.9
4/17/2018 18:53	2.7	20.5	3.9	2.5	0.2026	3.9
4/17/2018 18:54	3.1	20.2	3.9	2.5	0.1955	3.8
4/17/2018 18:55		20.4	4.1	2.5	0.1931	3.8
4/17/2018 18:56		20.2	3.9	2.4	0.188	3.9
4/17/2018 18:57	3.7	20.3	3.9	2.4	0.1937	3.8
4/17/2018 18:58	4.5	20.5	3.9	2.4	0.1827	3.8
4/17/2018 18:59	3.9	20.5	3.9	2.4	0.1928	3.7
4/17/2018 19:00	4.2	20.8	3.9	2.4	0.1875	3.7
4/17/2018 19:01	3.6	20.4	3.9	2.4	0.1935	2.7
4/17/2018 19:02	2.9	20.6	4	2.4	0.1939	1.4

4/17/2018 19:03	3.9	21	4.1	2.3	0.1778	1.4
4/17/2018 19:04	3.4	20.7	3.9	2.3	0.2018	1.6
4/17/2018 19:05	3.1	20.6	3.9	2.3	0.1746	1.6
4/17/2018 19:06	2.9	20.9	4	2.3	0.1893	1.5
4/17/2018 19:07	4.6	20.8	3.8	2.3	0.1926	1.4
4/17/2018 19:08	4.6	21.1	4	2.3	0.1684	1.4
4/17/2018 19:09	4.1	21.1	4.1	2.3	0.1869	1.4
4/17/2018 19:10	3.4	20.8	4.1	2.3	0.1792	1.4
4/17/2018 19:11	4.5	20.8	4.2	2.3	0.1838	1.4
4/17/2018 19:12	4.3	21	4.3	2.3	0.1715	1.4
4/17/2018 19:13	4.1	21.2	4.3	2.3	0.191	1.4
4/17/2018 19:14	4.3	21.1	4.3	2.3	0.1855	1.4
4/17/2018 19:15	3	21.2	4.2	2.3	0.1831	1.5
4/17/2018 19:16	4.2	21.2	4.2	2.3	0.1958	1.5
4/17/2018 19:17	3.6	21.1	4.3	2.3	0.1749	1.6
4/17/2018 19:18	2.9	20.8	4.1	2.4	0.1921	1.7
4/17/2018 19:19	3	20.8	4.1	2.4	0.1896	1.7
4/17/2018 19:20	5.2	21	4	2.5	0.1901	1.8
4/17/2018 19:21	3.9	21	4.2	2.6	0.1953	2
4/17/2018 19:22	5.1	20.8	4.3	2.6	0.1729	2
4/17/2018 19:23	4.1	20.8	4.1	2.6	0.1963	2
4/17/2018 19:24	3.4	20.8	4.3	2.6	0.1803	2.1
4/17/2018 19:25	2.6	20.8	4.1	2.6	0.1901	2.3
4/17/2018 19:26	3.2	20.7	4.1	2.7	0.1891	2.4
4/17/2018 19:27	3.2	20.6	4.1	2.7	0.1889	2.5
4/17/2018 19:28	3.4	20.8	4.1	2.7	0.1948	2.6
4/17/2018 19:29	2.4	21.2	4	2.7	0.1845	2.7
4/17/2018 19:30	3.7	20.7	4.2	2.7	0.1829	2.5
4/17/2018 19:31	3.7	21	4.1	2.7	0.181	2.6
4/17/2018 19:32	2.5	20.8	4.3	2.7	0.1723	2.4
4/17/2018 19:33	1.6	20.9	4.1	2.7	0.1852	2.4
4/17/2018 19:34	4.5	20.7	4.1	2.6	0.1734	2.3
4/17/2018 19:35	3.2	20.8	4	2.6	0.1938	2.4
4/17/2018 19:36	4.1	20.7	3.9	2.6	0.1864	2.4
4/17/2018 19:37	4.7	20.6	4.1	2.6	0.1749	2.4
4/17/2018 19:38	3.8	20.8	4	2.6	0.1863	2.4
4/17/2018 19:39	2.8	21	4.1	2.6	0.1745	2.5
4/17/2018 19:40	2.4	20.7	4.1	2.6	0.1905	2.5
4/17/2018 19:41	2.9	20.8	4	2.6	0.1731	2.5
4/17/2018 19:42	5.3	20.8	4.1	2.6	0.1735	2.4
4/17/2018 19:43	3	20.8	4.2	2.6	0.182	2.6
4/17/2018 19:44	3.7	20.8	4.2	2.6	0.1755	2.4
4/17/2018 19:45	3.2	20.9	4.2	2.6	0.1823	2.5
4/17/2018 19:46	3.7	20.6	4.2	2.6	0.1671	2.7
4/17/2018 19:47	3.3	20.8	4	2.6	0.1764	2.7
4/17/2018 19:48	3.7	20.8	3.8	2.6	0.1804	2.4
4/17/2018 19:49	3	20.9	3.7	2.6	0.1672	2.8

4/17/2018 19:50	3.6	20.6	3.7	2.6	0.1761	3.1
4/17/2018 19:51	3.4	20.7	3.7	2.6	0.1747	3.2
4/17/2018 19:52	2.3	20.5	3.7	2.6	0.1751	3.2
4/17/2018 19:53	4.1	20.5	3.6	2.6	0.1858	1.8
4/17/2018 19:54	2.7	20.9	3.7	2.6	0.1676	1.6
4/17/2018 19:55	2.8	20.8	3.6	2.7	0.184	2.2
4/17/2018 19:56	2.8	20.7	3.6	2.7	0.1732	2.8
4/17/2018 19:57	2.7	20.8	3.6	2.7	0.1757	3.4
4/17/2018 19:58	4.3	20.7	3.6	2.7	0.1762	3.4
4/17/2018 19:59	1.7	20.4	3.6	2.7	0.1663	3.5
4/17/2018 20:00	3.6	20.9	3.6	2.7	0.1721	3.7
4/17/2018 20:01	1.8	20.5	3.5	2.7	0.182	3.9
4/17/2018 20:02	4.5	20.5	3.6	2.7	0.1665	3.9
4/17/2018 20:03	3.4	20.7	3.6	2.7	0.1826	3.9
4/17/2018 20:04	4.6	20.7	3.6	2.7	0.1662	3.9
4/17/2018 20:05	2.5	20.7	3.5	2.6	0.179	3.9
4/17/2018 20:06	1.1	20.7	3.5	2.7	0.1776	4
4/17/2018 20:07	3.2	20.5	3.5	2.7	0.1696	4.1
4/17/2018 20:08	2.7	20.3	3.5	2.7	0.1768	4.1
4/17/2018 20:09	2.1	20.7	3.5	2.7	0.1632	4.1
4/17/2018 20:10	3.6	20.5	3.5	2.7	0.1803	4.1
4/17/2018 20:11	2.9	20.6	3.5	2.7	0.1743	4.1
4/17/2018 20:12	3.2	20.6	3.5	2.7	0.1653	4.1
4/17/2018 20:13	3.8	20.8	3.5	2.7	0.1805	4.2
4/17/2018 20:14	3.5	20.5	3.5	2.7	0.1581	4.3
4/17/2018 20:15	5.2	20.7	3.6	2.7	0.1653	4.3
4/17/2018 20:16	3.6	20.4	3.5	2.7	0.1764	4.3
4/17/2018 20:17	4	21.1	3.5	2.7	0.166	4.3
4/17/2018 20:18	3.1	20.5	3.5	2.7	0.1699	4.2
4/17/2018 20:19	2.8	20.7	3.5	2.7	0.1662	4.2
4/17/2018 20:20	3.3	20.7	3.5	2.7	0.1697	4
4/17/2018 20:21	3	20.7	3.5	2.7	0.171	4
4/17/2018 20:22	3.6	20.5	3.5	2.7	0.1711	4.1
4/17/2018 20:23	3.8	20.4	3.5	2.7	0.1769	4.1
4/17/2018 20:24	2.4	20.5	3.5	2.7	0.1681	4.2
4/17/2018 20:25	3.5	20.5	3.5	2.7	0.1752	4.3
4/17/2018 20:26	2.9	20.6	3.5	2.7	0.1705	4.4
4/17/2018 20:27	2.4	20.7	3.5	2.7	0.1646	3.9
4/17/2018 20:28	3.3	20.5	3.4	2.7	0.1803	4
4/17/2018 20:29	2.6	20.5	3.5	2.7	0.1625	4.3
4/17/2018 20:30	2.8	20.5	3.5	2.7	0.1735	4.4
4/17/2018 20:31	1.5	20.6	3.5	2.7	0.1743	4.4
4/17/2018 20:32	2.9	20.7	3.5	2.7	0.1625	4.2
4/17/2018 20:33	3.4	20.4	3.5	2.7	0.1749	3.8
4/17/2018 20:34	3.8	20.4	3.5	2.7	0.1659	4.1
4/17/2018 20:35	3.4	20.4	3.5	2.7	0.1693	4.1
4/17/2018 20:36	2.1	20.5	3.5	2.7	0.1719	4.2

4/17/2018 20:37	2.6	20.6	3.4	2.7	0.1593	4.3
4/17/2018 20:38	2.8	20.5	3.5	2.7	0.1754	4.3
4/17/2018 20:39	3.1	20.7	3.5	2.7	0.167	4.4
4/17/2018 20:40	3.3	20.7	3.5	2.7	0.1635	4.5
4/17/2018 20:41	3.9	20.7	3.5	2.7	0.171	4.5
4/17/2018 20:42	2.9	20.4	3.5	2.7	0.1628	4.5
4/17/2018 20:43	3	20.2	3.5	2.6	0.1749	4.6
4/17/2018 20:44	2.1	20.4	3.5	2.6	0.1616	4.8
4/17/2018 20:45	3.2	20.6	3.5	2.6	0.1739	4.9
4/17/2018 20:46	1.7	20.6	3.5	2.7	0.1698	4.9
4/17/2018 20:47	3.2	20.5	3.5	2.7	0.1599	4.8
4/17/2018 20:48	2.8	20.5	3.4	2.7	0.1695	4.8
4/17/2018 20:49	3.6	20.7	3	2.7	0.1705	4.6
4/17/2018 20:50	3.7	20.6	3.1	2.7	0.1621	4.2
4/17/2018 20:51	2.1	20.5	3.1	2.7	0.1675	3.8
4/17/2018 20:52	4.7	20.6	3	2.7	0.1594	3.5
4/17/2018 20:53	3.2	20.5	3.1	2.6	0.1723	3.9
4/17/2018 20:54	2.9	20.3	3.1	2.6	0.1619	4.1
4/17/2018 20:55	3.6	20.7	3	2.6	0.1669	3.8
4/17/2018 20:56	3.2	20.7	3.1	2.6	0.1705	2.9
4/17/2018 20:57	2.7	20.6	3.1	2.6	0.1561	3.3
4/17/2018 20:58	2.9	20.7	3.1	2.6	0.1711	3.7
4/17/2018 20:59	2.2	20.6	3.1	2.6	0.1564	4
4/17/2018 21:00	2.2	20.6	3.1	2.6	0.1702	4.2
4/17/2018 21:01	2.6	20.4	3.1	2.6	0.1675	4.4
4/17/2018 21:02	1.6	20.5	3.1	2.6	0.1567	4.6
4/17/2018 21:03	3.8	20.5	3.1	2.6	0.1663	4.5
4/17/2018 21:04	2.6	20.4	3.1	2.6	0.159	4.3
4/17/2018 21:05	3.2	20.5	3.1	2.6	0.1712	4.4
4/17/2018 21:06	2.2	20.6	3.1	2.6	0.1669	4.6
4/17/2018 21:07	2.4	20.6	3.1	2.6	0.1576	4.6
4/17/2018 21:08	3	20.7	3.1	2.6	0.1622	4.5
4/17/2018 21:09	2.8	20.7	3.1	2.6	0.1629	4.7
4/17/2018 21:10	2.7	20.6	3.1	2.6	0.1555	4.7
4/17/2018 21:11	3.5	20.4	3.1	2.6	0.1721	4.7
4/17/2018 21:12	3.1	20.4	3.1	2.6	0.1546	4.6
4/17/2018 21:13	3.4	20.4	3.1	2.6	0.1667	4.5
4/17/2018 21:14	3.6	20.5	3.1	2.6	0.1618	4.5
4/17/2018 21:15	2.7	20.5	3.1	2.6	0.1628	4.6
4/17/2018 21:16	2.6	20.5	3.1	2.6	0.1766	4.5
4/17/2018 21:17	1.8	20.5	3.1	2.6	0.1571	4.4
4/17/2018 21:18	2.8	20.5	3.1	2.6	0.1675	4.4
4/17/2018 21:19	2.9	20.2	3.1	2.6	0.1656	4.5
4/17/2018 21:20	2.6	20.5	3.1	2.6	0.1528	4.5
4/17/2018 21:21	4.9	20.7	3.1	2.6	0.1638	4.6
4/17/2018 21:22	3	20.9	3.2	2.6	0.153	4.6
4/17/2018 21:23	2.5	20.7	3.1	2.6	0.1624	4.6

4/17/2018 21:24	2.9	20.4	3.1	2.6	0.1632	4.6
4/17/2018 21:25	4	20.8	3.1	2.6	0.1598	4.7
4/17/2018 21:26	2.7	20.6	3.2	2.5	0.1663	4.6
4/17/2018 21:27	3.9	20.3	3.1	2.6	0.1588	4.6
4/17/2018 21:28	3.8	20.5	3.1	2.6	0.1639	4.5
4/17/2018 21:29	2.6	20.4	3.1	2.6	0.1657	4.4
4/17/2018 21:30	3.1	20.6	3.1	2.6	0.1579	4.4
4/17/2018 21:31	4.4	20.5	3.1	2.6	0.1595	4.4
4/17/2018 21:32	2.7	20.7	3.1	2.6	0.1533	4.3
4/17/2018 21:33	4	20.6	3.1	2.6	0.1589	4.3
4/17/2018 21:34	3.2	20.4	3.1	2.6	0.1606	4.2
4/17/2018 21:35	3.4	20.8	3.1	2.6	0.1452	4.2
4/17/2018 21:36	3.2	20.5	3.1	2.6	0.166	4.2
4/17/2018 21:37	4.5	20.4	3.1	2.6	0.1589	4.3
4/17/2018 21:38	2.7	20.4	3.1	2.6	0.1584	4.3
4/17/2018 21:39	2.7	20.5	3.1	2.6	0.1627	4.3
4/17/2018 21:40	2.9	20.4	3.1	2.6	0.1533	4.4
4/17/2018 21:41	3.8	20.2	3.1	2.6	0.1609	4.5
4/17/2018 21:42	3.5	20.4	3.1	2.6	0.1599	4.3
4/17/2018 21:43	3.5	20.5	3.1	2.6	0.1562	4.3
4/17/2018 21:44	3.2	20.5	3.1	2.6	0.1635	4.3
4/17/2018 21:45	1.9	20.7	3.1	2.6	0.1553	4.4
4/17/2018 21:46	4.5	20.4	3.1	2.6	0.1545	4.4
4/17/2018 21:47	2.9	20.5	3.1	2.6	0.1603	4.4
4/17/2018 21:48	3.5	20.4	3.1	2.6	0.1554	4.4
4/17/2018 21:49	4	20.4	3	2.6	0.1621	4.5
4/17/2018 21:50	3.5	20.2	2.7	2.6	0.1486	4.3
4/17/2018 21:51	2.3	20.3	2.6	2.6	0.1565	4.3
4/17/2018 21:52	2.9	20.6	2.6	2.6	0.1655	4.3
4/17/2018 21:53	4.4	20.4	2.6	2.6	0.152	4.4
4/17/2018 21:54	2.9	20.6	2.6	2.6	0.165	4.5
4/17/2018 21:55	2.7	20.6	2.7	2.5	0.1497	4.6
4/17/2018 21:56	2.9	20.6	2.6	2.5	0.1584	4.5
4/17/2018 21:57	2.1	20.3	2.6	2.5	0.1549	4.6
4/17/2018 21:58	2.6	20.6	2.6	2.5	0.157	4.5
4/17/2018 21:59	3.9	20.5	2.6	2.5	0.1596	4.5
4/17/2018 22:00	3.5	20.3	2.6	2.5	0.145	4.5
4/17/2018 22:01	2.8	20.2	2.6	2.5	0.1529	4.5
4/17/2018 22:02	4	20.7	2.6	2.5	0.1618	4.4
4/17/2018 22:03	1.3	20.3	2.6	2.5	0.1425	4.4
4/17/2018 22:04	2.9	20.5	2.6	2.6	0.1588	4.4
4/17/2018 22:05	3.2	20.5	2.6	2.6	0.1479	4.4
4/17/2018 22:06	2	20.2	2.6	2.6	0.1518	4.4
4/17/2018 22:07		20.5	2.6	2.6	0.16	4.3
4/17/2018 22:08		20.5	2.6	2.6	0.1455	4.3
4/17/2018 22:09		20.2	2.5	2.6	0.1712	4.3
4/17/2018 22:10		20.4	2.6	2.6	0.1554	4.4

4/17/2018 22:11		20.4	2.6	2.6	0.1517	4.4
4/17/2018 22:12		20.6	2.6	2.6	0.1606	4.4
4/17/2018 22:13		20.5	2.6	2.6	0.1488	4.3
4/17/2018 22:14		20.5	2.6	2.6	0.1544	4.3
4/17/2018 22:15		20.3	2.6	2.6	0.1551	4.3
4/17/2018 22:16	3.5	20.5	2.6	2.6	0.1467	4.4
4/17/2018 22:17	2.9	20.5	2.6	2.6	0.1586	4.5
4/17/2018 22:18	6.6	20.5	2.6	2.5	0.1489	4.5
4/17/2018 22:19	4.6	20.7	2.6	2.5	0.1578	4.5
4/17/2018 22:20	2.7	20.6	2.6	2.5	0.1445	4.4
4/17/2018 22:21	3.7	20.2	2.6	2.5	0.1556	4.4
4/17/2018 22:22	3.5	20.5	2.6	2.5	0.157	4.5
4/17/2018 22:23	4.3	20.4	2.6	2.5	0.1485	4.6
4/17/2018 22:24	3	20.3	2.5	2.5	0.1602	4.5
4/17/2018 22:25	2.8	20.4	2.5	2.5	0.1421	4.5
4/17/2018 22:26	2.8	20.3	2.5	2.5	0.1507	4.7
4/17/2018 22:27	3	20.6	2.6	2.5	0.1575	4.7
4/17/2018 22:28	3.4	20.5	2.6	2.6	0.1533	4.7
4/17/2018 22:29	3.1	20.5	2.5	2.6	0.1574	4.5
4/17/2018 22:30	3.5	20.2	2.6	2.6	0.1626	4.4
4/17/2018 22:31	4	20.5	2.6	2.6	0.1487	4.4
4/17/2018 22:32	1.6	20.4	2.6	2.6	0.1619	4.5
4/17/2018 22:33	3.5	20.4	2.6	2.6	0.1498	4.5
4/17/2018 22:34	1.9	20.5	2.6	2.5	0.1632	4.5
4/17/2018 22:35	2.2	20.7	2.9	2.5	0.148	4.5
4/17/2018 22:36	4.1	20.6	2.8	2.5	0.1539	4.6
4/17/2018 22:37	2.3	20.6	2.7	2.5	0.1573	4.7
4/17/2018 22:38	2.3	20.4	2.8	2.5	0.1431	4.8
4/17/2018 22:39	2.6	20.4	3	2.5	0.1553	4.8
4/17/2018 22:40	4.3	20.5	2.8	2.5	0.1401	4.8
4/17/2018 22:41	3	20.4	2.8	2.5	0.1518	4.9
4/17/2018 22:42	1.9	20.3	2.9	2.5	0.1616	4.9
4/17/2018 22:43	3.2	20.3	2.9	2.5	0.1397	4.8
4/17/2018 22:44	3	20.2	3	2.5	0.1607	4.5
4/17/2018 22:45	3.4	20.3	3	2.5	0.1448	4.7
4/17/2018 22:46	4	20.4	2.9	2.5	0.1553	4.7
4/17/2018 22:47	1.7	20.3	3.1	2.5	0.1555	4.8
4/17/2018 22:48	2.2	20.4	2.8	2.5	0.1478	4.9
4/17/2018 22:49	1.9	20.1	2.7	2.6	0.1636	5
4/17/2018 22:50	3.4	20	2.7	2.6	0.142	5.1
4/17/2018 22:51	3.4	19.9	2.8	2.5	0.1595	5
4/17/2018 22:52	3.6	20.2	2.9	2.5	0.1514	5.1
4/17/2018 22:53	3.8	19.8	3.2	2.5	0.1461	5.1
4/17/2018 22:54	2.3	19.9	3.7	2.5	0.1591	5
4/17/2018 22:55	2.9	20	3.1	2.5	0.1417	5
4/17/2018 22:56	4.1	19.9	3.1	2.5	0.1563	5
4/17/2018 22:57	3.2	19.9	3.3	2.5	0.1523	5

4/17/2018 22:58	3.5	20.2	3.4	2.4	0.1457	4.7
4/17/2018 22:59	2.6	20	3	2.4	0.1563	4.8
4/17/2018 23:00	3	20.1	3.4	2.5	0.1469	4.9
4/17/2018 23:01	3.6	20.1	3.2	2.5	0.1593	5
4/17/2018 23:02	1.2	20	3.3	2.5	0.1508	4.9
4/17/2018 23:03	3.9	19.8	3.6	2.5	0.1505	4.9
4/17/2018 23:04	2.1	20	3.1	2.5	0.1558	5
4/17/2018 23:05	4.2	19.9	3	2.5	0.1421	5.1
4/17/2018 23:06	2.2	19.9	3.1	2.6	0.1563	5.1
4/17/2018 23:07	3.3	19.9	3.3	2.6	0.1493	5.2
4/17/2018 23:08	3	19.7	3.8	2.6	0.1439	5.2
4/17/2018 23:09	3.2	19.7	4.1	2.5	0.1626	5.2
4/17/2018 23:10	4.2	20.2	3.9	2.5	0.1395	5.3
4/17/2018 23:11	3.2	19.8	4.1	2.5	0.149	5.2
4/17/2018 23:12	4	19.9	3.6	2.5	0.1443	5.1
4/17/2018 23:13	2.5	20	4	2.5	0.1489	5.1
4/17/2018 23:14	2.8	19.9	3.8	2.5	0.151	5.1
4/17/2018 23:15	2.8	19.8	4.4	2.5	0.1477	5.3
4/17/2018 23:16	2.6	19.8	3.8	2.5	0.1589	5.1
4/17/2018 23:17	2.6	19.7	4.1	2.5	0.1471	5
4/17/2018 23:18	2.3	19.9	4.2	2.5	0.1571	4.9
4/17/2018 23:19	3.4	19.7	4.2	2.5	0.1514	5
4/17/2018 23:20	2.4	19.5	3.9	2.5	0.1534	5.1
4/17/2018 23:21	2.4	20	3.7	2.6	0.1573	5.2
4/17/2018 23:22	2	19.9	3.6	2.6	0.1368	5.1
4/17/2018 23:23	3	19.5	3.8	2.6	0.1612	5
4/17/2018 23:24	3.3	19.7	4	2.6	0.1522	5.2
4/17/2018 23:25	2.3	19.6	4.4	2.6	0.1473	5.2
4/17/2018 23:26	2.7	19.5	4.7	2.6	0.1554	5.3
4/17/2018 23:27	2.3	19.6	4.7	2.6	0.1451	5.4
4/17/2018 23:28	3.3	19.8	4.8	2.6	0.1526	5.4
4/17/2018 23:29	0.6	19.7	4.4	2.5	0.1466	5.3
4/17/2018 23:30	2.8	19.9	4.5	2.5	0.1504	5.2
4/17/2018 23:31	3.6	19.9	4.6	2.5	0.1562	5.2
4/17/2018 23:32	3.2	19.8	4.9	2.5	0.1441	5
4/17/2018 23:33	2.7	19.8	4.4	2.5	0.1554	5.1
4/17/2018 23:34	3.2	19.8	4.7	2.5	0.1444	5.1
4/17/2018 23:35	2.5	19.6	4.9	2.5	0.1481	5
4/17/2018 23:36	3.2	19.8	4.8	2.5	0.1551	4.8
4/17/2018 23:37	2.3	19.7	4.5	2.6	0.1468	4.6
4/17/2018 23:38	2.4	19.9	4.2	2.6	0.1614	4.7
4/17/2018 23:39	3.5	19.7	4.3	2.6	0.1512	4.8
4/17/2018 23:40	1.4	19.7	4.6	2.6	0.1505	4.9
4/17/2018 23:41	3.8	19.7	4.9	2.6	0.1554	4.8
4/17/2018 23:42	2.2	19.7	4.9	2.6	0.1461	4.6
4/17/2018 23:43	3.5	19.6	4.9	2.6	0.1613	4.5
4/17/2018 23:44	2.7	19.8	5.1	2.6	0.1468	4.3

4/17/2018 23:45	3.2	19.7	5.1	2.6	0.1572	4.4
4/17/2018 23:46	3.8	19.6	5	2.6	0.1523	4.7
4/17/2018 23:47	2	19.8	4.9	2.6	0.1522	4.8
4/17/2018 23:48	2.3	20	4.7	2.6	0.1596	5
4/17/2018 23:49	4	19.6	4.8	2.6	0.1416	5
4/17/2018 23:50	3	19.6	4.8	2.6	0.1546	5
4/17/2018 23:51	1.2	19.6	4.8	2.6	0.159	5.1
4/17/2018 23:52	3.5	19.8	4.8	2.6	0.1497	5.3
4/17/2018 23:53	2.9	19.6	4.4	2.6	0.1566	5.2
4/17/2018 23:54	3.4	19.6	4.2	2.7	0.1421	5.2
4/17/2018 23:55	3.1	19.7	4.4	2.7	0.1571	5.2
4/17/2018 23:56	2	19.6	4.6	2.7	0.1572	5.1
4/17/2018 23:57	2	19.7	4.9	2.7	0.1493	5
4/17/2018 23:58	2.6	19.9	5.1	2.7	0.1586	4.7
4/17/2018 23:59	3.8	19.6	4.9	2.7	0.1471	4.7
4/18/2018 0:00	2.3	19.7	4.9	2.7	0.1579	4.6
4/18/2018 0:01	3	19.7	4.9	2.7	0.1519	4.5
4/18/2018 0:02	2.5	19.9	4.9	2.6	0.1519	4.6
4/18/2018 0:03	2.3	19.8	4.6	2.6	0.1602	4.5
4/18/2018 0:04	1.6	19.6	4.7	2.6	0.1531	4.6
4/18/2018 0:05	1.9	19.6	4.6	2.6	0.1579	4.7
4/18/2018 0:06	4	19.5	4.5	2.7	0.1576	4.8
4/18/2018 0:07	1.8	19.8	4.5	2.7	0.148	4.8
4/18/2018 0:08	3	19.5	4.5	2.7	0.1646	4.9
4/18/2018 0:09	2.4	19.5	4.5	2.7	0.1497	5.1
4/18/2018 0:10	3.2	19.4	4.5	2.7	0.1561	5.1
4/18/2018 0:11	2.5	19.7	4.6	2.8	0.1513	4.9
4/18/2018 0:12	2.8	19.5	4.9	2.7	0.1494	4.8
4/18/2018 0:13	2.3	19.6	4.8	2.7	0.1591	4.9
4/18/2018 0:14	2.7	19.7	4.9	2.7	0.1492	4.8
4/18/2018 0:15	2.7	19.7	4.9	2.7	0.1575	4.8
4/18/2018 0:16	1.6	19.7	4.7	2.7	0.1623	4.9
4/18/2018 0:17	3	20	4.9	2.7	0.1543	5.1
4/18/2018 0:18	2	19.9	4.8	2.7	0.1607	4.9
4/18/2018 0:19	3	20	4.9	2.7	0.1492	4.9
4/18/2018 0:20	3.9	19.7	4.8	2.6	0.1612	4.9
4/18/2018 0:21	2.9	19.8	4.8	2.6	0.1605	4.8
4/18/2018 0:22	2.6	19.8	4.9	2.6	0.1534	4.8
4/18/2018 0:23	2.9	19.7	4.8	2.7	0.1608	5
4/18/2018 0:24	1.1	19.7	4.9	2.7	0.1505	5
4/18/2018 0:25	3.4	19.9	4.8	2.6	0.1639	5.1
4/18/2018 0:26	2.9	19.7	4.8	2.6	0.1584	5.2
4/18/2018 0:27	2.3	19.8	4.5	2.6	0.1505	5.1
4/18/2018 0:28	2.8	19.5	4.6	2.7	0.1627	2.8
4/18/2018 0:29	2.7	20	4.8	2.7	0.1507	1.3
4/18/2018 0:30	2.3	19.6	5	2.7	0.1626	1.4
4/18/2018 0:31	2.8	19.9	5	2.6	0.16	1.4

4/18/2018 0:32	2.5	19.7	5	2.6	0.1573	1.4
4/18/2018 0:33	2.7	19.6	4.9	2.6	0.163	1.4
4/18/2018 0:34	2.4	19.8	5	2.7	0.1536	1.4
4/18/2018 0:35	3	19.6	4.9	2.6	0.159	1.7
4/18/2018 0:36	2.3	19.7	4.8	2.6	0.1625	2.1
4/18/2018 0:37	3.2	19.8	4.9	2.6	0.1567	2.6
4/18/2018 0:38	1.8	19.7	4.9	2.7	0.1627	3
4/18/2018 0:39	2.7	19.8	4.8	2.7	0.151	2.8
4/18/2018 0:40	3.9	19.8	4.8	2.7	0.1676	2.6
4/18/2018 0:41	1.7	19.6	4.8	2.7	0.1619	3.1
4/18/2018 0:42	2.5	19.8	4.8	2.8	0.1576	3.3
4/18/2018 0:43	2.1	19.7	4.9	2.8	0.1639	3.5
4/18/2018 0:44	1.7	19.4	5	2.8	0.1506	3.7
4/18/2018 0:45	1.8	19.5	5.3	2.8	0.1696	3.9
4/18/2018 0:46	2	19.6	5.1	2.8	0.1576	4
4/18/2018 0:47	3.1	19.7	5.3	2.7	0.1554	4.1
4/18/2018 0:48	2.4	19.6	5.3	2.7	0.1659	4.2
4/18/2018 0:49	2.6	19.8	5.4	2.7	0.1559	4.3
4/18/2018 0:50	4.1	19.4	5.5	2.7	0.157	4.4
4/18/2018 0:51		19.4	5.3	2.7	0.1578	4.5
4/18/2018 0:52		19.6	5.3	2.7	0.1564	4.6
4/18/2018 0:53		19.6	5	2.7	0.1662	4.6
4/18/2018 0:54	4.4	19.4	4.9	2.7	0.1537	4.7
4/18/2018 0:55	1.2	19.2	4.9	2.7	0.1677	4.7
4/18/2018 0:56		19.3	4.9	2.7	0.1566	4.8
4/18/2018 0:57	1.9	19.5	4.9	2.7	0.1542	4.8
4/18/2018 0:58		19.5	5	2.7	0.1717	4.8
4/18/2018 0:59	2.5	19.6	5.3	2.7	0.1552	4.9
4/18/2018 1:00	6.1	19.5	5.4	2.7	0.1663	4.8
4/18/2018 1:01	2.4	19.8	5.4	2.7	0.1575	4.8
4/18/2018 1:02	3.3	19.5	5.7	2.7	0.158	4.9
4/18/2018 1:03	4.1	19.6	5.4	2.7	0.1674	4.8
4/18/2018 1:04	2.8	19.7	5.2	2.7	0.1518	4.8
4/18/2018 1:05		19.7	5.1	2.7	0.1709	4.8
4/18/2018 1:06	4.2	19.5	5.2	2.7	0.1611	4.8
4/18/2018 1:07	3	19.2	5.2	2.7	0.1612	4.8
4/18/2018 1:08	3.4	19.5	5.2	2.7	0.1676	4.8
4/18/2018 1:09	2.4	19.3	5.2	2.8	0.1555	4.8
4/18/2018 1:10	2.6	19.3	5.2	2.8	0.1714	4.9
4/18/2018 1:11	2.8	19.4	5.1	2.8	0.1594	4.9
4/18/2018 1:12	1.9	19.8	5.4	2.8	0.1601	4.9
4/18/2018 1:13	4	19.5	5.7	2.8	0.1626	4.9
4/18/2018 1:14	2	19.3	5.7	2.8	0.1556	4.9
4/18/2018 1:15	3.4	19.5	5.6	2.8	0.1638	4.9
4/18/2018 1:16	2.8	19.7	5.7	2.8	0.1659	5
4/18/2018 1:17	1.8	19.6	5.4	2.8	0.1589	4.9
4/18/2018 1:18	1.6	19.5	5.5	2.8	0.1662	4.8

4/18/2018 1:19	3.1	19.6	5.6	2.8	0.153	4.8
4/18/2018 1:20	1.4	19.6	5.4	2.8	0.1582	4.4
4/18/2018 1:21	2.9	19.8	5.4	2.8	0.1549	3.9
4/18/2018 1:22	2.5	19.5	5.4	2.8	0.1614	3.3
4/18/2018 1:23	3.1	19.3	5.4	2.8	0.1627	2.8
4/18/2018 1:24	3	19.5	5.3	2.8	0.1567	3.1
4/18/2018 1:25	2.3	19.7	5.4	2.8	0.1586	3.3
4/18/2018 1:26	0.9	19.4	5.5	2.8	0.1589	3.4
4/18/2018 1:27	2.9	19.5	5.6	2.8	0.1546	3.5
4/18/2018 1:28	2.5	19.4	5.6	2.8	0.1649	3.8
4/18/2018 1:29	3.6	19.5	5.5	2.8	0.1598	4
4/18/2018 1:30	3	19.5	5.4	2.8	0.1714	4.1
4/18/2018 1:31	3.3	19.6	5.4	2.8	0.1623	4.2
4/18/2018 1:32	2	19.4	5.3	2.8	0.161	4.3
4/18/2018 1:33	1.9	19.5	5.3	2.8	0.1671	4.4
4/18/2018 1:34	2.2	19.5	5.3	2.8	0.1652	4.5
4/18/2018 1:35	1.4	19.5	5.2	2.8	0.1579	4.5
4/18/2018 1:36	3.5	19.6	5.3	2.8	0.168	3.2
4/18/2018 1:37	3.2	19.4	5.3	2.8	0.153	1.6
4/18/2018 1:38	2.1	19.2	5.4	2.8	0.1739	2.2
4/18/2018 1:39	2.6	19.5	5.6	2.8	0.1526	2.4
4/18/2018 1:40	2.7	19.5	5.5	2.8	0.159	2.6
4/18/2018 1:41	2	19.4	5.3	2.8	0.159	3
4/18/2018 1:42	2.7	19.6	5.4	2.8	0.1464	3.2
4/18/2018 1:43	2.9	19.8	5.6	2.7	0.1689	3.3
4/18/2018 1:44	3.6	19.6	5.5	2.7	0.1588	3.3
4/18/2018 1:45	2.6	19.7	5.4	2.7	0.1766	3.4
4/18/2018 1:46	4	19.6	5.3	2.7	0.1631	3.5
4/18/2018 1:47	2.6	19.4	5.3	2.8	0.1547	3.6
4/18/2018 1:48	3.4	19.8	5.2	2.8	0.1663	3.6
4/18/2018 1:49	2.8	19.5	5.2	2.8	0.1477	3.7
4/18/2018 1:50	1.7	19.4	5.2	2.8	0.1586	3.8
4/18/2018 1:51	2.8	19.4	5.2	2.9	0.1615	3.9
4/18/2018 1:52	2.7	19.5	5.2	2.9	0.1524	3.8
4/18/2018 1:53	2	19.4	5.2	2.9	0.1674	3.8
4/18/2018 1:54	2.4	19.3	5.4	2.8	0.154	3.5
4/18/2018 1:55	3.4	19.5	5.5	2.8	0.1652	3.5
4/18/2018 1:56	3	19.6	5.5	2.8	0.1657	3.5
4/18/2018 1:57	2.3	19.3	5.6	2.8	0.1585	3.6
4/18/2018 1:58	3.2	19.5	5.5	2.7	0.1661	3.7
4/18/2018 1:59	1.9	19.2	5.7	2.7	0.1601	3.8
4/18/2018 2:00	3.3	19.4	5.3	2.7	0.1695	3.7
4/18/2018 2:01	1.9	19.5	5.3	2.7	0.1702	3.6
4/18/2018 2:02	2.3	19.5	5.2	2.8	0.158	3.7
4/18/2018 2:03	2.5	19.4	5.2	2.8	0.1683	3.7
4/18/2018 2:04	1.9	19.3	5.2	2.8	0.1601	3.7
4/18/2018 2:05	2.7	19.2	5.3	2.8	0.1603	3.7

4/18/2018 2:06	2.3	19.5	5.7	2.8	0.1694	3.8
4/18/2018 2:07	3.2	19.3	6	2.8	0.1601	3.8
4/18/2018 2:08	3	19.2	6	2.8	0.166	3.8
4/18/2018 2:09	2.6	19.3	5.5	2.8	0.1526	3.9
4/18/2018 2:10	2	19.3	5.6	2.8	0.1613	3.9
4/18/2018 2:11	3.5	19.5	5.8	2.8	0.1694	3.9
4/18/2018 2:12	1.9	19.3	6.1	2.8	0.1571	3.9
4/18/2018 2:13	2.7	19.5	6.1	2.8	0.1717	3.9
4/18/2018 2:14	2.2	19.3	6	2.7	0.1542	3.9
4/18/2018 2:15	2.7	19.6	6	2.7	0.1642	3.9
4/18/2018 2:16	3.2	19.4	5.6	2.7	0.1625	3.9
4/18/2018 2:17	4	19.2	5.5	2.7	0.1583	3.8
4/18/2018 2:18	2.5	19.2	5.5	2.7	0.1667	3.9
4/18/2018 2:19	2.1	19.1	5.8	2.7	0.1585	3.9
4/18/2018 2:20	2.1	19.2	6	2.7	0.162	3.9
4/18/2018 2:21	2.5	19.4	5.8	2.8	0.1652	3.8
4/18/2018 2:22	3.7	19.3	6	2.8	0.1556	3.9
4/18/2018 2:23	3	19.1	6.5	2.8	0.1662	3.9
4/18/2018 2:24	2.1	19.4	5.9	2.8	0.1611	4
4/18/2018 2:25	1.2	19.3	5.8	2.8	0.1583	3.8
4/18/2018 2:26	1.4	19.3	6.1	2.7	0.1643	3.8
4/18/2018 2:27	2.5	19	6	2.7	0.1537	3.9
4/18/2018 2:28	3.8	19.3	5.8	2.7	0.1611	4
4/18/2018 2:29	1.4	19.2	5.6	2.7	0.1584	3.9
4/18/2018 2:30	1.5	19.1	5.7	2.7	0.1611	4
4/18/2018 2:31	2.3	19.1	5.7	2.8	0.1717	4
4/18/2018 2:32	2	19.4	5.8	2.8	0.1589	4
4/18/2018 2:33	2.2	19.2	6.1	2.8	0.1651	3.8
4/18/2018 2:34	1.8	19.2	6.6	2.8	0.1676	3.7
4/18/2018 2:35	3.1	19.4	6.2	2.8	0.1631	3.7
4/18/2018 2:36	2.5	19.1	6.1	2.8	0.1734	3.7
4/18/2018 2:37	0.7	19.1	6.3	2.7	0.1556	3.7
4/18/2018 2:38	2.4	19	5.8	2.7	0.1678	3.8
4/18/2018 2:39	2.6	18.9	6	2.7	0.1606	3.8
4/18/2018 2:40	1.6	19.2	6.1	2.7	0.162	3.9
4/18/2018 2:41	2.8	19	6.3	2.8	0.1779	3.9
4/18/2018 2:42	3.1	18.9	6.9	2.8	0.1561	3.8
4/18/2018 2:43	2.7	18.9	6.1	2.8	0.1693	3.7
4/18/2018 2:44	1.6	19	6.9	2.8	0.1625	3.7
4/18/2018 2:45	1.9	19.1	6.6	2.8	0.1665	3.8
4/18/2018 2:46	2.9	19	6.9	2.8	0.1727	4
4/18/2018 2:47	2.7	18.9	6.5	2.8	0.1613	4.1
4/18/2018 2:48	1.8	19.2	6.9	2.8	0.1725	4
4/18/2018 2:49	2.6	19.2	7.1	2.8	0.1704	4
4/18/2018 2:50	1.4	18.6	7.5	2.8	0.1639	4.1
4/18/2018 2:51	2	19	7.1	2.8	0.1747	4.2
4/18/2018 2:52	2.5	19	7.2	2.8	0.1642	4.3

4/18/2018 2:53	1.6	19	7.1	2.8	0.1615	4.2
4/18/2018 2:54	1.4	19	7.1	2.9	0.1701	4.2
4/18/2018 2:55	0.7	19.2	6.8	2.9	0.1621	4.1
4/18/2018 2:56	2	19.1	6.9	2.9	0.1725	4.2
4/18/2018 2:57	2.2	18.8	7.3	2.9	0.1543	4.2
4/18/2018 2:58	2.2	19	7	2.9	0.1733	4.1
4/18/2018 2:59	2.2	19.1	6.7	2.9	0.1714	4.2
4/18/2018 3:00	1.1	18.7	7.1	2.9	0.1598	4.1
4/18/2018 3:01	1.7	18.9	6.9	2.9	0.1772	4.2
4/18/2018 3:02	2	18.9	6.9	2.9	0.1642	4.4
4/18/2018 3:03	1.4	18.9	7.3	2.9	0.1671	4.3
4/18/2018 3:04	2.6	19	7.2	2.9	0.1731	4.2
4/18/2018 3:05	2.3	19	7.8	2.9	0.1627	4.2
4/18/2018 3:06	2.5	19.1	7.3	2.9	0.1746	4.2
4/18/2018 3:07	1.9	19.1	7.1	2.9	0.1663	4.2
4/18/2018 3:08	2.2	18.9	6.9	2.9	0.1714	4.2
4/18/2018 3:09	1.2	18.9	7.1	2.9	0.1689	4.3
4/18/2018 3:10	2.4	19.1	7.1	2.9	0.1593	4.3
4/18/2018 3:11	1.7	18.9	7.5	2.9	0.1797	4.3
4/18/2018 3:12	2.6	18.9	7	2.9	0.162	4.4
4/18/2018 3:13	1	19	6.9	2.9	0.1651	4.4
4/18/2018 3:14	1.7	19	7.1	2.9	0.1764	4.4
4/18/2018 3:15	1.6	19	6.9	2.9	0.1603	4.4
4/18/2018 3:16	1.9	19.2	7	2.9	0.1718	4.4
4/18/2018 3:17	2	19.1	7	2.9	0.1669	4.4
4/18/2018 3:18	2.1	18.8	6.8	2.9	0.1695	4.4
4/18/2018 3:19	2.1	19	7	2.9	0.1701	4.5
4/18/2018 3:20	2.4	19.1	6.8	2.9	0.1625	4.5
4/18/2018 3:21	2.2	19	6.8	2.9	0.1721	4.5
4/18/2018 3:22	1.9	19.1	7	2.9	0.1665	4.5
4/18/2018 3:23	2.5	19.1	7	2.9	0.1661	4.5
4/18/2018 3:24	3.2	19.1	6.9	2.9	0.1747	4.6
4/18/2018 3:25	1.6	18.8	6.7	3	0.1552	4.6
4/18/2018 3:26	1.6	19	7.1	2.9	0.1712	4.6
4/18/2018 3:27	3.1	19.2	6.7	2.9	0.1633	4.5
4/18/2018 3:28	3.4	19	6.7	2.9	0.1612	4.6
4/18/2018 3:29	1.7	19	7	2.9	0.1774	4.5
4/18/2018 3:30	2.4	19	7.1	2.9	0.1654	4.4
4/18/2018 3:31	1.7	19.1	6.8	2.9	0.1749	4.4
4/18/2018 3:32	1.5	19.2	6.5	2.9	0.1642	4.4
4/18/2018 3:33	2.3	18.9	7	2.9	0.1632	4.4
4/18/2018 3:34	3.2	19.1	7.2	2.9	0.1749	4.5
4/18/2018 3:35	3.1	18.8	7	2.9	0.1653	4.4
4/18/2018 3:36	2.6	18.9	6.7	2.9	0.159	4.5
4/18/2018 3:37	2.9	18.7	6.8	2.9	0.1755	4.4
4/18/2018 3:38	3.6	18.8	7	3	0.1671	4.4
4/18/2018 3:39	2.6	19	6.9	3	0.1713	4.3

4/18/2018 3:40	2.8	19	7.1	3	0.1672	4.3
4/18/2018 3:41	1.1	19	7.2	3	0.1642	4.2
4/18/2018 3:42	1.4	19	7.1	3	0.1741	4.2
4/18/2018 3:43	1.7	18.8	7.2	3.1	0.1583	4.3
4/18/2018 3:44	1.7	19	7	3.1	0.175	4.3
4/18/2018 3:45	3.4	19	7.1	3.1	0.1615	4.3
4/18/2018 3:46	1.6	18.7	7.5	3.1	0.1675	4.4
4/18/2018 3:47	2.1	19	7.5	3.1	0.1728	4.4
4/18/2018 3:48	2.4	18.7	7.6	3.1	0.1621	4.5
4/18/2018 3:49	3.4	18.9	7.3	3.2	0.1757	4.4
4/18/2018 3:50	1.2	18.7	7.7	3.2	0.1749	4.5
4/18/2018 3:51	2.8	18.6	7.8	3.2	0.1716	4.4
4/18/2018 3:52	2.2	18.7	7.7	3.2	0.1754	4.3
4/18/2018 3:53	2.7	18.7	8.1	3.3	0.1648	4.3
4/18/2018 3:54	2.2	18.8	8	3.3	0.1762	4.3
4/18/2018 3:55	2.1	18.8	8	3.3	0.1663	4.3
4/18/2018 3:56	1.5	18.4	8.2	3.4	0.1789	4.3
4/18/2018 3:57	1.6	18.4	8.3	3.4	0.1823	4.3
4/18/2018 3:58	1.5	18.6	7.4	3.4	0.1721	4.2
4/18/2018 3:59	1.6	18.7	7.7	3.4	0.1809	4.1
4/18/2018 4:00	0.7	18.5	8	3.4	0.1751	4.2
4/18/2018 4:01	1.2	18.3	8.1	3.5	0.1752	4.3
4/18/2018 4:02	0.8	18.6	8	3.5	0.1799	4.4
4/18/2018 4:03	1.7	18.6	7.7	3.5	0.1704	4.3
4/18/2018 4:04	1.4	18.8	8	3.5	0.1839	4.3
4/18/2018 4:05	2.2	18.7	7.8	3.6	0.1751	4.4
4/18/2018 4:06	2.5	18.6	8.3	3.6	0.1792	4.4
4/18/2018 4:07	2.4	18.6	8.3	3.7	0.1858	4.4
4/18/2018 4:08	3	18.7	8.1	3.7	0.1721	4.4
4/18/2018 4:09	1.4	18.6	8.3	3.7	0.1814	4.3
4/18/2018 4:10	2.3	18.4	8.6	3.7	0.1783	4.4
4/18/2018 4:11	2.3	18.3	8.9	3.8	0.1716	4.4
4/18/2018 4:12	1.7	18.6	9.1	3.8	0.1892	4.3
4/18/2018 4:13	2.5	18.3	8	3.8	0.1698	4.1
4/18/2018 4:14	0.5	18.6	8.7	3.8	0.1858	3.8
4/18/2018 4:15	2	18.6	8.2	3.8	0.1756	3.8
4/18/2018 4:16	0.8	18.8	8.5	3.8	0.18	3.8
4/18/2018 4:17	2.9	18.8	8.2	3.9	0.1844	3.8
4/18/2018 4:18	3.2	18.9	8.6	3.9	0.1741	3.9
4/18/2018 4:19	2.7	18.5	8.7	3.9	0.1748	4.2
4/18/2018 4:20	2	18.4	8.8	3.9	0.17	4.2
4/18/2018 4:21	1.9	18.4	9	3.9	0.167	4.4
4/18/2018 4:22	2.1	18.5	8.9	3.9	0.1833	4.4
4/18/2018 4:23		18.6	8.9	3.9	0.1742	4.5
4/18/2018 4:24		18.5	8.6	4	0.1791	4.5
4/18/2018 4:25		18.6	8.7	4	0.1892	4.5
4/18/2018 4:26		18.7	8.9	4	0.1713	4.4

4/18/2018 4:27		18.6	8.8	4.1	0.1856	4.5
4/18/2018 4:28	4.5	18.3	9.1	4	0.1766	4.4
4/18/2018 4:29	0.7	18.7	9.1	4	0.1862	4.4
4/18/2018 4:30	2.5	18.5	9.1	4	0.1872	4.4
4/18/2018 4:31	2.2	18.4	9.5	4	0.1828	4.5
4/18/2018 4:32	1.8	18.4	9.4	4	0.1806	4.4
4/18/2018 4:33	2.8	18.6	8.8	4	0.171	4.4
4/18/2018 4:34	0.8	18.5	9	4	0.1855	4.3
4/18/2018 4:35	2.5	18.5	8.8	3.9	0.1822	2.4
4/18/2018 4:36	1.8	18.6	8.8	3.9	0.1748	1.4
4/18/2018 4:37	2.1	18.6	9.2	3.9	0.1887	1.4
4/18/2018 4:38	2.1	18.6	8.8	3.9	0.1745	1.4
4/18/2018 4:39	3.6	18.7	8.5	3.9	0.1764	1.4
4/18/2018 4:40	2.2	18.7	8.9	3.9	0.1835	1.4
4/18/2018 4:41	1.2	18.6	8.9	3.9	0.1794	1.4
4/18/2018 4:42	2.1	18.7	8.9	3.9	0.1803	1.4
4/18/2018 4:43	2.2	18.5	9.2	3.9	0.1787	1.4
4/18/2018 4:44	2.7	18.5	9.1	3.9	0.1835	1.4
4/18/2018 4:45	1.9	18.4	8.8	3.9	0.1844	1.4
4/18/2018 4:46	2	18.5	8.9	3.9	0.1738	1.4
4/18/2018 4:47	3.1	18.7	8.9	3.9	0.1843	1.4
4/18/2018 4:48	3.5	18.6	9.6	3.9	0.1788	1.5
4/18/2018 4:49	2.6	18.7	9.2	3.9	0.1859	1.6
4/18/2018 4:50	1	18.5	8.6	4	0.184	1.5
4/18/2018 4:51	3.4	18.5	8.7	4	0.1795	1.5
4/18/2018 4:52	1.1	18.8	8.8	4.1	0.1843	1.5
4/18/2018 4:53	1.9	18.6	8.8	4.1	0.186	1.6
4/18/2018 4:54	1.5	18.5	9.2	4.1	0.1727	1.8
4/18/2018 4:55	1.5	18.6	9.3	4.1	0.1858	2
4/18/2018 4:56	2.1	18.6	9.3	4.2	0.1746	2.2
4/18/2018 4:57	1.5	18.5	9.2	4.2	0.1821	2.4
4/18/2018 4:58	2.2	18.6	9	4.2	0.1893	2.7
4/18/2018 4:59	2.3	18.5	9.2	4.3	0.1764	2.9
4/18/2018 5:00	2.6	18.6	9.1	4.4	0.1851	3.1
4/18/2018 5:01	1.8	18.6	9.1	4.4	0.1828	3.2
4/18/2018 5:02	1.8	18.4	9.1	4.5	0.1744	3.2
4/18/2018 5:03	2.3	18.4	9.4	4.6	0.1908	3.3
4/18/2018 5:04	2.4	18.5	9.2	4.7	0.1765	3.4
4/18/2018 5:05	1.8	18.3	9.4	4.8	0.1845	3.5
4/18/2018 5:06	1.3	18.4	9.7	4.9	0.1834	3.5
4/18/2018 5:07	1.9	18.3	9.3	4.9	0.1723	3.4
4/18/2018 5:08	1	18.3	9	5	0.1897	3.5
4/18/2018 5:09	0.6	18.5	9.8	5.1	0.1762	3.6
4/18/2018 5:10	1.6	18.5	9.1	5.1	0.1912	3.6
4/18/2018 5:11	1.7	18.1	10.2	5.2	0.184	3.5
4/18/2018 5:12	2.6	18.7	10	5.2	0.1842	3.6
4/18/2018 5:13	1.1	18.4	9.2	5.3	0.1866	3.6

4/18/2018 5:14	3.7	18.6	9.5	5.3	0.1763	3.7
4/18/2018 5:15	2.3	18.5	9.7	5.4	0.1831	3.8
4/18/2018 5:16	1.5	18.8	9.8	5.5	0.1823	3.9
4/18/2018 5:17	1.7	18.5	9.7	5.5	0.1818	4
4/18/2018 5:18	1.1	18.6	9.6	5.5	0.1905	3.9
4/18/2018 5:19	1.5	18.4	10	5.6	0.1787	4.1
4/18/2018 5:20	2.1	18.6	9.9	5.6	0.1809	4
4/18/2018 5:21	3	18.5	9.8	5.7	0.1872	4
4/18/2018 5:22	0.5	18	10.2	5.7	0.1822	4.2
4/18/2018 5:23	0.5	18.3	9.7	5.8	0.1915	4.2
4/18/2018 5:24	0.9	18.6	9.4	5.8	0.1852	4.2
4/18/2018 5:25	2	18.4	9	5.8	0.1869	4.2
4/18/2018 5:26	3.1	18.6	9.6	5.8	0.1908	4.2
4/18/2018 5:27	1.1	18.5	9.6	5.8	0.1751	4.2
4/18/2018 5:28	1.4	18.6	9.7	5.8	0.1949	4.3
4/18/2018 5:29	2.8	18.7	9.2	5.8	0.1817	4.4
4/18/2018 5:30	2.1	18.5	9.4	5.8	0.1867	4.3
4/18/2018 5:31	3	18.4	9.3	5.8	0.1855	4.2
4/18/2018 5:32	1.9	18.5	9.3	5.8	0.1741	4.2
4/18/2018 5:33	1.1	18.6	9.2	5.8	0.19	4.3
4/18/2018 5:34	2.9	18.5	9.2	5.9	0.1845	4.3
4/18/2018 5:35	1.1	18.8	9	5.9	0.1822	4.3
4/18/2018 5:36	3.4	18.7	8.8	5.9	0.1959	4.2
4/18/2018 5:37	2.8	18.5	9.1	5.9	0.1816	4.3
4/18/2018 5:38	1.5	18.6	9.4	5.9	0.1841	4.3
4/18/2018 5:39	2.1	18.6	8.9	5.9	0.1834	4.3
4/18/2018 5:40	2.9	18.6	8.8	5.9	0.1781	4.4
4/18/2018 5:41	2.8	18.8	8.7	6	0.1908	4.3
4/18/2018 5:42	2.3	18.5	9	6	0.1815	4.4
4/18/2018 5:43	2.4	18.6	9.2	6	0.182	4.4
4/18/2018 5:44	1.9	18.7	8.8	6	0.1864	4.4
4/18/2018 5:45	1.9	18.8	8.8	6.1	0.1736	4.4
4/18/2018 5:46	2.6	18.8	8.9	6.1	0.1927	4.4
4/18/2018 5:47	2.7	18.6	8.9	6.1	0.1797	4.3
4/18/2018 5:48	1.1	18.8	8.9	6.2	0.1849	4.2
4/18/2018 5:49	2.3	18.8	8.7	6.2	0.1977	4.2
4/18/2018 5:50	3.1	18.8	8.9	6.3	0.1825	4.3
4/18/2018 5:51	1.3	18.5	8.8	6.3	0.1877	4.2
4/18/2018 5:52	2.3	18.4	9.1	6.4	0.1897	4.3
4/18/2018 5:53	2.8	18.6	9	6.4	0.1846	4.3
4/18/2018 5:54	2.4	18.6	8.9	6.4	0.1998	4.3
4/18/2018 5:55	2.2	18.5	8.6	6.4	0.1801	4.4
4/18/2018 5:56	1.6	18.6	8.8	6.5	0.1938	4.3
4/18/2018 5:57	2.7	18.6	8.8	6.5	0.1864	4.4
4/18/2018 5:58	2.7	18.7	8.8	6.5	0.1853	4.2
4/18/2018 5:59	1.9	18.6	8.8	6.5	0.1888	4.2
4/18/2018 6:00	1.1	18.6	9	6.6	0.1809	4.3

4/18/2018 6:01	1.7	18.6	8.8	6.6	0.1855	4.2
4/18/2018 6:02	1.9	18.7	8.8	6.6	0.1904	4
4/18/2018 6:03	1.3	18.6	8.6	6.7	0.1778	4.1
4/18/2018 6:04	0.4	18.6	9	6.8	0.1993	4.1
4/18/2018 6:05	2.6	18.6	8.6	6.8	0.1838	4.1
4/18/2018 6:06	0.6	18.6	8.6	6.8	0.1828	4.1
4/18/2018 6:07	2.3	18.6	8.7	6.9	0.1983	4.1
4/18/2018 6:08	3.2	18.4	9	6.9	0.1821	4.1
4/18/2018 6:09	2.8	18.5	9	6.9	0.1952	4.2
4/18/2018 6:10	1.8	18.5	9.3	7	0.1962	4.3
4/18/2018 6:11	2.4	18.5	9.5	7	0.1949	4.3
4/18/2018 6:12	1.5	18.3	9.4	7.1	0.1972	4.3
4/18/2018 6:13	1.6	18.9	9.3	7.1	0.1842	4.3
4/18/2018 6:14	1.8	18.3	9.4	7.1	0.1948	4.2
4/18/2018 6:15	2	18.4	9.4	7.2	0.1934	4.2
4/18/2018 6:16	0.9	18.5	9	7.2	0.1938	4.2
4/18/2018 6:17	1.7	18.2	9.5	7.2	0.2042	4.2
4/18/2018 6:18	0.8	18.1	9.6	7.3	0.1865	4.2
4/18/2018 6:19	1.2	18.5	9.7	7.3	0.2095	4.3
4/18/2018 6:20	2.2	18.1	9.9	7.3	0.1981	4.2
4/18/2018 6:21	1.9	18.4	9.5	7.4	0.1906	4.1
4/18/2018 6:22	2.9	18.4	8.8	7.4	0.2056	4.1
4/18/2018 6:23	1.7	18.3	9.4	7.4	0.1981	4.2
4/18/2018 6:24	1.7	18.4	9.7	7.4	0.2042	4.2
4/18/2018 6:25	1.9	18.3	9.1	7.4	0.1933	4.2
4/18/2018 6:26	2.1	18.8	8.8	7.4	0.1882	4.1
4/18/2018 6:27	1.7	18.4	9.2	7.4	0.2022	3.9
4/18/2018 6:28	2.8	18.6	9.1	7.4	0.1873	3.9
4/18/2018 6:29	1.7	18.6	8.9	7.4	0.2036	3.9
4/18/2018 6:30	1.1	18.6	8.7	7.4	0.1887	3.1
4/18/2018 6:31	0.7	18.5	8.8	7.4	0.1934	1.8
4/18/2018 6:32	2.9	18.4	9	7.4	0.2084	1.9
4/18/2018 6:33	0.9	18.7	9.1	7.5	0.1946	2
4/18/2018 6:34	1.8	18.5	9	7.5	0.2105	2
4/18/2018 6:35	2.9	18.3	9.4	7.6	0.1997	2.1
4/18/2018 6:36	1.4	18.4	9.4	7.6	0.1972	2.2
4/18/2018 6:37	1.5	18.6	9.6	7.6	0.2102	2.2
4/18/2018 6:38	2	18.3	9.5	7.6	0.1994	2.3
4/18/2018 6:39	2	18.4	9.4	7.7	0.2047	2.2
4/18/2018 6:40	2	18.7	9.3	7.7	0.1968	2.1
4/18/2018 6:41	1.2	18.7	9.1	7.7	0.1992	2.3
4/18/2018 6:42	0.9	18.4	9.1	7.7	0.2083	2.4
4/18/2018 6:43	0.6	18.4	9	7.8	0.1993	2.3
4/18/2018 6:44	1.2	18.2	9.1	7.8	0.2012	2.3
4/18/2018 6:45	3.7	18.5	9.2	7.9	0.2064	2.2
4/18/2018 6:46	2.1	18.5	8.9	7.9	0.201	2.3
4/18/2018 6:47	1.6	18.3	9.3	7.9	0.2059	2.3

4/18/2018 6:48			3.5	18.7	9.5	7.9	0.1938	2.2
4/18/2018 6:49			1.9	18.4	9.4	8	0.2054	2.3
4/18/2018 6:50			1.9	18.4	9.1	8	0.1953	2.3
4/18/2018 6:51			2.4	18.4	9	8	0.1986	2.4
4/18/2018 6:52			2	18.6	9.1	8	0.2103	2.5
4/18/2018 6:53			1.1	18.6	9.4	8	0.1937	2.6
4/18/2018 6:54			1.6	18.7	9.5	8.1	0.2041	2.4
4/18/2018 6:55			3.3	18.3	9.7	8.1	0.2024	2.4
4/18/2018 6:56			1.4	18.4	9.7	8.1	0.1915	2.5
4/18/2018 6:57			2.5	18.4	9.8	8.1	0.2103	2.5
4/18/2018 6:58			1.1	18.3	9.8	8.1	0.1912	2.5
4/18/2018 6:59			2.7	18.6	9.7	8.2	0.2053	2.4
4/18/2018 7:00				18.5	9.2	8.1	0.2025	2.3
4/18/2018 7:01			1.7	18.7	9	8.2	0.196	2.6
4/18/2018 7:02				18.5	9.2	8.2	0.2067	2.9
4/18/2018 7:03				18.5	9.3	8.2	0.2001	3
4/18/2018 7:04			2.1	18.3	9.4	8.2	0.1951	3.1
4/18/2018 7:05				18.6	9.2	8.2	0.205	3.2
4/18/2018 7:06				18.4	9.2	8.2	0.1889	3.3
4/18/2018 7:07			1.7	18.5	9.4	8.2	0.2038	3.2
4/18/2018 7:08				18.4	9.5	8.2	0.2031	3.1
4/18/2018 7:09			1.4	18.6	9.2	8.2	0.1947	2.9
4/18/2018 7:10			2.6	18.6	9	8.2	0.2058	2.7
4/18/2018 7:11			0.4	18.6	8.9	8.1	0.1936	2.8
4/18/2018 7:12				18.6	8.8	8.1	0.2047	2.9
4/18/2018 7:13			2.7	18.5	8.8	8.1	0.1961	2.9
4/18/2018 7:14			0	18.6	8.7	8.1	0.1961	2.9
4/18/2018 7:15			1.1	18.6	8.7	8.1	0.2086	3
4/18/2018 7:16			1.6	18.6	8.8	8.2	0.1882	3
4/18/2018 7:17			3.3	18.7	8.8	8.1	0.2075	2.8
4/18/2018 7:18				18.4	8.8	8.2	0.1993	2
4/18/2018 7:19				18.4	8.6	8.1	0.1827	1.6
4/18/2018 7:20			9.8	18.8	8.6	8.2	0.2002	1.8
4/18/2018 7:21			2.7	18.7	8.7	8.2	0.2044	1.6
4/18/2018 7:22			8.3	18.5	8.6	8.2	0.2038	0.1
4/18/2018 7:23			2.5	18.7	8.6	8.2	0.2174	0.2
4/18/2018 7:24			1.4	18.4	8.7	8.2	0.1986	0.5
4/18/2018 7:25			2.1	18.6	8.5	8.2	0.2159	0.7
4/18/2018 7:26			2.1	18.8	8.5	8.2	0.2079	0.8
4/18/2018 7:27			2	18.6	8.9	8.2	0.2211	1
4/18/2018 7:28			1.6	18.7	8.7	8.2	0.2252	1.1
4/18/2018 7:29			1	18.5	8.5	8.3	0.2171	1.2
4/18/2018 7:30	0.2	-0.4	2	18.8	8.6	8.3	0.2201	1.3
4/18/2018 7:31	0.2	-0.6	1.4	18.7	8.6	8.3	0.2262	0.6
4/18/2018 7:32	0.2	0	1.4	18.6	8.6	8.3	0.2318	2.2
4/18/2018 7:33	0.2	-0.2	1.8	18.7	8.5	8.4	0.2367	25.2
4/18/2018 7:34	0.2	-0.4	2.8	18.4	8.7	8.4	0.2291	33.6

4/18/2018 7:35	0.2	-0.4	3.4	18.7	8.6	8.4	0.2406	34.1
4/18/2018 7:36	0.2	-1.1	1.5	18.9	8.6	8.5	0.2376	33.9
4/18/2018 7:37	0.2	-0.5	1.5	18.5	8.7	8.6	0.2286	26.8
4/18/2018 7:38	0.2	-0.8	2	18.6	8.8	8.7	0.2483	5.6
4/18/2018 7:39	0.1	-0.9	1.8	18.4	8.7	8.8	0.2469	0
4/18/2018 7:40	0.1	-0.5	1.6	18.8	8.5	8.8	0.2461	-0.5
4/18/2018 7:41	0.2	-0.3	2.5	18.9	8.7	8.9	0.2565	-0.5
4/18/2018 7:42	0.2	0.3	1.9	18.6	8.6	9	0.2384	-0.4
4/18/2018 7:43	0.2	-0.3	1.1	18.7	8.7	9.1	0.2531	-0.3
4/18/2018 7:44	0.2	-0.3	2.2	18.6	8.7	9.2	0.2526	-0.2
4/18/2018 7:45	0.2	-0.2	2.5	19	8.6	9.3	0.2561	-0.2
4/18/2018 7:46	0.2	-0.2	3.5	19	8.6	9.4	0.2571	0
4/18/2018 7:47	0.2	-0.2	1.6	18.7	8.6	9.4	0.2464	-1.2
4/18/2018 7:48	0.2	-0.2	1.7	18.8	8.7	9.6	0.2638	-0.4
4/18/2018 7:49	0.2	-0.2	1.7	18.9	8.7	9.7	0.2582	
4/18/2018 7:50	0.2	-0.2	1.8	18.6	8.8	9.8	0.2518	
4/18/2018 7:51	0.2	-0.3	2.1	18.5	9	9.9	0.2722	
4/18/2018 7:52	0.2	-1	1.5	18.7	8.9	10.1	0.2489	
4/18/2018 7:53	0.2	-0.2	1.6	18.7	8.8	10.2	0.2152	
4/18/2018 7:54	0.2	-0.4	1.2	19.6	10.2	14.9	0.2342	
4/18/2018 7:55	0.2	-0.5	1.9	20.6	17.1	52	0.2848	
4/18/2018 7:56	0.2	0.1	3	21.4	23.7	79.6	0.3912	
4/18/2018 7:57	0.2	-0.2	3.3	22.2	32.6	127.2	0.5792	
4/18/2018 7:58	0.2	-0.6	4.1	22.6	37.6	150.9	0.936	1.4
4/18/2018 7:59	0.2	-0.7	3	22.8	38.5	153.6	1.3816	-0.3
4/18/2018 8:00	0.2	-0.6	3.2	23.3	38.6	152.1	1.8029	-0.8
4/18/2018 8:01	0.2	0.4	1.8	23.3	40.7	154.6	2.2221	-0.8
4/18/2018 8:02	0.2	-0.3	3.3	23.4	41.5	157.4	2.615	-0.2
4/18/2018 8:03	0.2	0.1	1.4	23.2	40.6	156.3	2.7558	1.3
4/18/2018 8:04	0.2	0	3	23	39.7	155.7	2.9468	1.4
4/18/2018 8:05	0.2	0	2.9	22.6	39	151.2	3.0236	1.5
4/18/2018 8:06	0.2	-0.3	2.2	22.5	38.1	145.9	3.1682	1.5
4/18/2018 8:07	0.2	-0.5	3.1	22.2	37.1	144.2	3.1063	1.5
4/18/2018 8:08	0.2	0	2.5	21.9	35.8	140.1	3.0779	1.5
4/18/2018 8:09	0.2	-0.6	2	21.9	35.3	139.6	3.0595	1.5
4/18/2018 8:10	0.2	-0.4	3.2	21.7	34.6	139.3	3.0704	1.5
4/18/2018 8:11	0.2	0.6	2.1	21.6	34.4	139	3.107	1.5
4/18/2018 8:12	0.2	0	4.6	21.6	34.1	138.4	3.0945	1.5
4/18/2018 8:13	0.2	-0.4	3	21.5	33.3	133.9	3.0343	1.6
4/18/2018 8:14	0.2	0	1.4	21.4	32.7	132	3.0216	1.7
4/18/2018 8:15	0.2	-0.1	2.7	21.2	31.9	129.8	2.9412	1.9
4/18/2018 8:16	0.2	0.2	3.2	21.2	31.7	128.9	2.9744	1.9
4/18/2018 8:17	0.2	-0.4	1.5	21.3	31.3	127.2	2.9322	2
4/18/2018 8:18	0.3	-0.2	2.5	21.1	30.8	126.2	2.9358	2.1
4/18/2018 8:19	0.3	0.1	1.2	20.9	30.2	125.8	2.845	2.3
4/18/2018 8:20	0.3	0.5	3.4	21.2	30	125.6	2.8872	2.3
4/18/2018 8:21	0.2	0.1	2.2	20.9	29.4	125.3	2.8338	2.4

4/18/2018 8:22	0.2	0.1	1.7	20.7	29	124.9	2.7843	2.5
4/18/2018 8:23	0.3	-0.1	2.7	20.9	28.4	124.2	2.7467	2.5
4/18/2018 8:24	0.3	0.3	2.2	20.8	28.3	123.6	2.7757	2.7
4/18/2018 8:25	0.3	-0.1	3.1	20.6	28.1	122.9	2.7753	3
4/18/2018 8:26	0.3	0.1	2.5	20.3	27.3	121.5	2.6712	3.1
4/18/2018 8:27	0.2	-0.1	3	20.6	27	120.5	2.6527	3.1
4/18/2018 8:28	0.2	-0.3	2.4	20.4	26.6	111.5	2.5812	3.2
4/18/2018 8:29	0.2	-0.4	1.7	20.4	26.3	111.5	2.5837	3.3
4/18/2018 8:30	0.2	1.2	3.1	20.4	26	112.7	2.568	3
4/18/2018 8:31	0.2	-0.1	2.5	20.3	25.9	112.9	2.5694	3.1
4/18/2018 8:32	0.2	0.3	3	20.3	25.2	112.3	2.487	3.1
4/18/2018 8:33	0.2	-0.6	2.2	20.4	25.2	112	2.5223	3.1
4/18/2018 8:34	0.1	-0.1	1.2	20.3	25.2	111.8	2.5526	3.4
4/18/2018 8:35	0.1	0.4	2.4	20.2	24.8	111.5	2.5068	3.6
4/18/2018 8:36	0.1	-0.2	1.9	20.3	24.6	110.9	2.5183	3.8
4/18/2018 8:37	0.1	-0.1	1.9	20.1	24.3	110.4	2.4708	3.7
4/18/2018 8:38	0.1	0.1	1.6	20.3	23.7	109.7	2.4087	3.3
4/18/2018 8:39	0.1	0.2	2.6	20.2	23.9	109	2.4743	3.6
4/18/2018 8:40	0.1	-0.5	1.8	19.8	23.2	101.4	2.2939	3.8
4/18/2018 8:41	0.1	0	2.4	19.7	22.8	99.6	2.2773	3.7
4/18/2018 8:42	0.1	0.2	1.3	19.9	22.4	95.8	2.1907	3.9
4/18/2018 8:43	0.2	0.5	2.4	19.7	22.3	95.8	2.2235	4
4/18/2018 8:44	0.2	-0.6	0.5	20	22.4	96.6	2.2941	3.9
4/18/2018 8:45	0.2	-0.1	2	19.8	22.3	97.2	2.3116	3.9
4/18/2018 8:46	0.3	-0.3	3.8	19.7	22	97.7	2.3128	3.9
4/18/2018 8:47	0.3	0	3.1	19.8	21.9	98.3	2.3021	3.9
4/18/2018 8:48	0.3	0	2.4	20	21.6	98.5	2.243	3.9
4/18/2018 8:49	0.3	0	1.5	19.8	21.3	98.5	2.2135	4
4/18/2018 8:50	0.3	0.2	1.8	19.8	21	95.8	2.1595	4
4/18/2018 8:51	0.3	0.7	2.2	19.6	20.6	91	2.0742	4
4/18/2018 8:52	0.3	-0.5	3.4	19.6	20.5	92.1	2.1117	3.8
4/18/2018 8:53	0.3	-0.5	1.6	19.5	19.9	86	1.9663	2
4/18/2018 8:54	0.2	-0.3	3.9	19.7	20	87.6	2.0896	1.6
4/18/2018 8:55	0.3	-0.3	1.6	19.7	20	88.2	2.0904	1.7
4/18/2018 8:56	0.2	-0.2	1.9	19.5	19.9	88.8	2.1313	1.8
4/18/2018 8:57	0.2	0.4	3	19.6	19.5	89.3	2.0607	1.9
4/18/2018 8:58	0.2	0.8	1.1	19.6	19.4	89.5	2.0485	1.8
4/18/2018 8:59	0.2	-0.2	1.9	19.7	19.2	89.6	2.0356	1.8
4/18/2018 9:00	0.2	-0.2	2	19.8	19.2	89.9	2.0158	1.8
4/18/2018 9:01	0.1	-0.5	2.3	19.3	19.2	89.1	2.0098	1.8
4/18/2018 9:02	0.1	-0.1	1.8	19.5	18.7	83.3	1.9195	1.8
4/18/2018 9:03	0.1	0.1	1.3	19.3	18.3	84.4	1.9776	1.6
4/18/2018 9:04	0.1	0.3	2.6	19.3	17.6	77.3	1.8423	1.6
4/18/2018 9:05	0.1	0.1	2.5	18.8	17.4	66.8	1.537	1.7
4/18/2018 9:06	0.1	0	1.2	18.8	16.9	65	1.5238	1.7
4/18/2018 9:07	0.1	0.1	3.2	18.9	16.6	59.9	1.5143	1.7
4/18/2018 9:08	0.1	0.2	2.7	18.7	16.3	65.1	1.4793	1.8

4/18/2018 9:09	0.1	-0.1	1.5	18.2	16.8	57.4	1.2553	1.9
4/18/2018 9:10	0.2	-0.2	2	18.3	17.1	44.3	1.0707	1.9
4/18/2018 9:11	0.2	0.7	1.1	18.2	17.6	42.8	1.048	1.9
4/18/2018 9:12	0.3	-0.4	2.8	18.5	18	42.4	1.0195	1.9
4/18/2018 9:13	0.4	0.2	1	18.4	18	37.6	0.9355	1.8
4/18/2018 9:14	0.4	0	3	18.3	17.8	37.1	0.9462	1.8
4/18/2018 9:15	0.5	0.8	2.9	18.1	18	37.6	0.9439	1.9
4/18/2018 9:16	0.5	-0.2	2.4	18.1	18.2	37.3	1.0035	1.8
4/18/2018 9:17	0.4	-0.3	2	18.2	18.4	37.5	0.9598	1.8
4/18/2018 9:18	0.3	0.7	3.4	18	18.4	37.5	0.9172	1.7
4/18/2018 9:19	0.3	0.2	1.2	18.2	18.4	37.1	0.8616	1.6
4/18/2018 9:20	0.4	0.2	1.4	18	18.8	36.4	0.8382	1.6
4/18/2018 9:21	0.4	-0.2	0.9	18.2	18.7	35.7	0.8273	1.7
4/18/2018 9:22	0.5	1.2	0.9	18	18.7	34.7	0.7474	1.7
4/18/2018 9:23	0.5	0.2	1.5	18	18.7	33.3	0.6939	1.7
4/18/2018 9:24	0.5	1.2	1.8	18	18.7	31.2	0.6808	1.7
4/18/2018 9:25	0.6	0.1	2.4	18	18.5	29.3	0.616	1.7
4/18/2018 9:26	0.7	-0.1	1	17.8	18.7	27.2	0.6177	1.6
4/18/2018 9:27	0.7	0.2	2.2	18.2	18.7	25.3	0.6094	1.6
4/18/2018 9:28	0.7	0.7	2	18	18.6	23.7	0.5516	1.6
4/18/2018 9:29	0.7	0.9	0.8	18	18.8	22	0.5348	1.6
4/18/2018 9:30	0.8	0.9	1.7	18.2	18.6	20.3	0.5018	1.7
4/18/2018 9:31	1	1.1	2.7	18.6	17.7	18.8	0.4738	1.6
4/18/2018 9:32	1.1	1.2	2.3	18.2	18	17.6	0.4628	1.7
4/18/2018 9:33	1.1	0.6	1.9	18.4	18.1	16.5	0.4604	1.8
4/18/2018 9:34	1	0.7	1.6	18	18.3	15.6	0.4879	1.7
4/18/2018 9:35	0.8	-0.2	2.3	21.5	21.6	27.5	0.5708	1.8
4/18/2018 9:36	0.6	-0.1	3.2	23.3	43.2	73.9	0.7309	1.8
4/18/2018 9:37	0.4	-0.4	4.7	24.6	55.7	193.2	0.8864	1.9
4/18/2018 9:38	0.2	0.5	3.9	25.2	64.2	247	1.216	1.9
4/18/2018 9:39	0.2	0.1	4.7	25.8	70.3	276	1.6832	2
4/18/2018 9:40	0.2	-0.2	3.6	26.6	73.9	289.5	2.257	1.9
4/18/2018 9:41	0.3	0.5	3.5	27.1	74.8	292	2.8427	1.8
4/18/2018 9:42	0.2	0	4.8	27.2	74.6	290.4	3.2947	1.8
4/18/2018 9:43	0.2	-0.1	3.9	26.6	71.7	271.4	3.465	1.9
4/18/2018 9:44	0.1	0.4	4.1	27	70	272.6	3.8502	1.8
4/18/2018 9:45	0.1	-0.4	3.6	27.1	71	278.2	4.2658	1.8
4/18/2018 9:46	0.1	0	3.7	27.2	70.2	278.3	4.4657	1.8
4/18/2018 9:47	0.2	0.3	3.6	26.2	68.3	271.3	4.5128	1.8
4/18/2018 9:48	0.1	-0.2	2.8	26.4	67.1	267.2	4.6298	1.7
4/18/2018 9:49	0.1	0	2.2	26.3	66.3	266.7	4.781	1.7
4/18/2018 9:50	0.1	-0.6	3.4	25.8	64.8	262.4	4.7537	1.8
4/18/2018 9:51	0.1	0.4	2.8	24.5	61.4	254.7	4.5093	1.8
4/18/2018 9:52	0.1	0.8	2.9	25.5	60.6	255.3	4.6307	1.8
4/18/2018 9:53	0.1	-0.5	3.8	25	61.4	254.7	4.8452	1.8
4/18/2018 9:54	0.1	-0.1	4	24.7	59.6	248.2	4.717	1.8
4/18/2018 9:55	0.1	-0.4	3.9	24.3	57.4	238	4.5747	1.7

4/18/2018 9:56	0.1	-0.4	3.6	24.3	57.3	238.8	4.6235	1.8
4/18/2018 9:57	0.1	0	2.4	23.9	55.2	231.9	4.51	1.8
4/18/2018 9:58	0.1	-0.1	2.1	23.9	54.7	236.2	4.4107	1.8
4/18/2018 9:59	0.1	0.2	4.5	24.2	55	237.7	4.6367	1.6
4/18/2018 10:00	0.1	-0.5	4	23.3	52.3	219.4	4.3163	1.6
4/18/2018 10:01	0	-0.2	2.9	23.8	51.6	227.4	4.5168	1.6
4/18/2018 10:02	0.1	-0.5	3.3	23.7	51.6	231.2	4.631	1.6
4/18/2018 10:03	0.1	-0.2	3.2	23.1	51.5	227.5	4.5482	1.6
4/18/2018 10:04	0.1	-0.2	4.3	23.2	50.1	215.9	4.4162	1.6
4/18/2018 10:05	0.1	-0.1	1.4	23	49.9	218.3	4.4218	1.6
4/18/2018 10:06	0.2	0.1	4	22.8	48.2	213.8	4.3477	1.6
4/18/2018 10:07	0.2	-0.4	2.5	23	48.6	217.1	4.441	1.6
4/18/2018 10:08	0.2	0.3	4	22.3	45.4	207.2	4.1323	1.6
4/18/2018 10:09	0.2	0	2.7	22.8	47.1	213.2	4.4268	1.6
4/18/2018 10:10	0.2	0.2	2.8	22.1	45.3	211.7	4.2538	1.7
4/18/2018 10:11	0.2	0.1	1.9	22.2	44.9	204	4.2035	1.7
4/18/2018 10:12	0.2	0.3	1.1	22.1	43.6	205.4	4.1458	1.8
4/18/2018 10:13	0.2	0	2.3	22.1	44.5	205.9	4.3312	1.8
4/18/2018 10:14	0.2	0.6	2.5	21.8	42.8	206.1	4.164	1.9
4/18/2018 10:15	0.2	-0.8	3.9	22.4	43.2	202.7	4.2817	2
4/18/2018 10:16	0.3	0.5	2.7	22	42.5	198.2	4.2277	2
4/18/2018 10:17	0.3	0.2	2.8	21.4	40.2	190	3.9792	1.9
4/18/2018 10:18	0.3	0.4	1.6	21.5	38.8	180.1	3.8493	1.9
4/18/2018 10:19	0.3	0.7	2.7	21.8	39.8	189.9	4.1048	2
4/18/2018 10:20	0.3	-0.2	8.9	21.4	39	185.7	3.972	2
4/18/2018 10:21	0.3	-0.1		21.5	39.6	191.4	4.1228	1.7
4/18/2018 10:22	0.2	-0.3		21.7	38.6	186.6	4.048	1.6
4/18/2018 10:23	0.2	-0.2		21.5	38.6	188.5	4.0975	1.6
4/18/2018 10:24	0.3	-0.1		21.3	36.9	176.5	3.8953	1.5
4/18/2018 10:25	0.2	0	2.5	21	34.8	163.8	3.6532	1.5
4/18/2018 10:26	0.2	0.4		21	34.6	161.6	3.681	1.5
4/18/2018 10:27	0.2	0.3	2	21.1	35.6	177.7	3.9038	1.5
4/18/2018 10:28	0.2	0.1		20.8	34.3	165.4	3.6487	1.5
4/18/2018 10:29	0.2	-0.6	2.6	21.1	34.4	171	3.795	1.5
4/18/2018 10:30	0.2	0.1	8.9	21.1	34.3	171.3	3.7957	1.5
4/18/2018 10:31	0.2	0.2	4.6	20.4	32.5	159.4	3.5283	1.5
4/18/2018 10:32	0.2	0.2	3.3	20.9	32.4	154	3.5723	1.6
4/18/2018 10:33	0.2	0.1		21	32.7	166	3.709	1.8
4/18/2018 10:34	0.2	0.4		20.8	32.5	164.7	3.6788	2
4/18/2018 10:35	0.2	-0.5		20.7	32	167.6	3.6365	2
4/18/2018 10:36	0.2	0.1	2.9	20.9	31.7	167.6	3.6575	2.3
4/18/2018 10:37	0.1	-0.2	7.1	20.9	31.2	166.7	3.6605	2
4/18/2018 10:38	0.1	-0.2	2	20.5	31	164.8	3.6665	1.5
4/18/2018 10:39	0.1	0	2.8	20.6	30.6	159.4	3.579	1.5
4/18/2018 10:40	0.2	0	2.6	20.2	28.9	143.6	3.2783	1.5
4/18/2018 10:41	0.2	0.5	3.5	20.5	28.8	147.8	3.4072	1.5
4/18/2018 10:42	0.2	0.1	1.9	20	28.6	148.1	3.3815	1.6

4/18/2018 10:43	0.2	0	2.2	20.4	27.8	143.5	3.2923	1.7
4/18/2018 10:44	0.2	0.6	1.3	20.5	27.9	150.2	3.3648	1.7
4/18/2018 10:45	0.2	-0.1	3.5	20.7	28.3	150.1	3.4667	1.6
4/18/2018 10:46	0.2	-0.3	2.8	20.5	28	147.4	3.4473	1.6
4/18/2018 10:47	0.2	0	5	20.5	27.8	149.1	3.4208	1.6
4/18/2018 10:48							0	
4/18/2018 10:49							0	
4/18/2018 10:50	0.2	0	2.9	20.2	26.1	138.6	3.2017	1.5
4/18/2018 10:51	0.5	1.1	2.5	20	23.4	118.9	2.5697	1.6
4/18/2018 10:52	0.8	1.2	2.6	19.7	21.2	94.1	2.2722	1.6
4/18/2018 10:53	1.3	1.7	5	20.2	20.1	69.8	1.8395	1.6
4/18/2018 10:54	1.8	2.2	2.4	19.7	23	70.2	1.7372	1.6
4/18/2018 10:55	1.6	0.7	0.7	17.2	47.7	208.9	1.333	1.5
4/18/2018 10:56	1.2	0.7	1.2	18	40.9	259.8	1.2483	1.5
4/18/2018 10:57	1	2.8	4.4	23	33.2	125.2	1.1078	1.7
4/18/2018 10:58	1.8	4.2	3.2	19.5	30.5	81.4	0.9483	1.9
4/18/2018 10:59	3	4.8	3.8	20.9	29.1	61.8	0.9474	2.2
4/18/2018 11:00	4.2	6.9	4.3	23.5	26.8	50.3	0.8186	2.4
4/18/2018 11:01	5.3	7.1	5.1	25	25.6	44.6	0.8357	2.5
4/18/2018 11:02	5.7	6	3.2	22.9	25.7	46	0.8092	2.8
4/18/2018 11:03	4.9	2.7	2.2	19.1	25.7	51.2	0.9394	2.8
4/18/2018 11:04	3.1	0	2.5	18	27.9	62.1	1.1065	2.2
4/18/2018 11:05	1.7	0.8	3	18.5	28.7	60.3	1.063	1.9
4/18/2018 11:06	0.8	1.2	2.9	18.4	28.3	56.3	1.0097	1.7
4/18/2018 11:07	0.7	0.5	2.4	18.3	27.3	50.9	0.9722	1.8
4/18/2018 11:08	0.7	1	2.4	18.2	27	49.4	0.9046	1.7
4/18/2018 11:09	1.1	2.3	3.2	19	25.1	39.8	0.8051	1.9
4/18/2018 11:10	1.2	1.5	2.9	18.5	24.9	38.8	0.8209	2
4/18/2018 11:11	1.1	0.5	1.4	18	26.2	38.4	0.8123	1.6
4/18/2018 11:12	0.7	0.2	2.1	18	25.9	38.1	0.8379	1.4
4/18/2018 11:13	0.4	0.2	2.3	18	25.7	38	0.8307	1.4
4/18/2018 11:14	0.4	-0.3	1.9	17.7	25.8	38.1	0.8509	1.4
4/18/2018 11:15	0.3	-0.3	2.7	21.6	38.2	54.3	0.9868	1.4
4/18/2018 11:16	0.3	0	2.4	21.2	53.8	186.5	1.0497	1.4
4/18/2018 11:17	0.2	0.2	3.9	22.4	68.3	268.3	1.2165	1.5
4/18/2018 11:18	0.2	0.5	3.3	23.6	83.4	343.1	1.4133	1.5
4/18/2018 11:19	0.2	0.3	3.3	25.1	92	396.6	2.2358	1.6
4/18/2018 11:20	0.2	0.3	2.9	26.7	102.4	438.5	3.4843	1.6
4/18/2018 11:21	0.2	0.6	4.6	27.2	103.9	457.2	4.2243	1.7
4/18/2018 11:22	0.2	0.7	3.7	27.5	101.5	430.3	4.8633	1.7
4/18/2018 11:23	0.2	0.7	1.8	27.9	100.1	432	5.6085	1.7
4/18/2018 11:24	0.2	0.2	2.7	27.8	97	410.3	6.1693	1.7
4/18/2018 11:25	0.2	0.6	5.2	28.4	96.8	415.6	6.9903	1.7
4/18/2018 11:26	0.2	0.4	4.7	27.9	97.8	429.8	7.458	1.7
4/18/2018 11:27	0.2	0.4	5	27.4	91.9	420.3	7.5792	1.7
4/18/2018 11:28	0.2	0.2	3.2	26.5	86.8	407.5	7.7973	1.7
4/18/2018 11:29	0.2	0.6	5.2	26	85.5	376.9	7.8978	1.8

4/18/2018 11:30	0.2	-0.5	4.2	26.1	84.4	384.8	8.1722	1.8
4/18/2018 11:31	0.2	0.2	3.7	26	85.4	393.1	8.6895	1.8
4/18/2018 11:32	0.2	0	3.2	25.5	82.3	400.7	8.4613	1.8
4/18/2018 11:33	0.2	0.3	2.9	25.1	79	369.3	8.2412	1.6
4/18/2018 11:34	0.2	0.1	4.3	25	78.3	347.5	8.405	1.6
4/18/2018 11:35	0.2	0.1	5.4	24.8	77.5	355.7	8.5742	1.6
4/18/2018 11:36	0.1	-0.6	2.6	24.8	76	360.7	8.587	1.6
4/18/2018 11:37	0.1	0.4	3.2	24.5	74.3	360.4	8.5225	1.6
4/18/2018 11:38	0.1	0.4	2.8	24.3	74.4	368.1	8.8457	1.6
4/18/2018 11:39	0.1	0	3.5	24.4	74.9	365.7	9.101	1.6
4/18/2018 11:40	0.1	0	3.5	24.1	71.4	359.3	8.8589	1.6
4/18/2018 11:41	0	0.6	3.2	24.2	72.9	363.8	9.1831	1.6
4/18/2018 11:42	0.1	0.2	3.8	23.8	69.7	360.2	8.9034	1.6
4/18/2018 11:43	0.1	0.4	4.7	23.8	68.8	347.1	8.9027	1.6
4/18/2018 11:44	0.1	0.2	2.9	23.4	68.3	343.8	8.7752	1.6
4/18/2018 11:45	0	0.3	3.5	23.7	66.8	337.4	8.8377	1.7
4/18/2018 11:46	0	0.1	2.7	23.6	67.4	342.1	9.0413	1.7
4/18/2018 11:47	0	0.5	5.3	23	64.9	335.3	8.6199	1.6
4/18/2018 11:48	0	0.5	2.8	23	62.3	316.6	8.3958	1.6
4/18/2018 11:49	0	0.3	3.6	23.3	62.9	316.7	8.6131	1.6
4/18/2018 11:50	0	-0.2	3	23	62.3	324.5	8.5233	1.6
4/18/2018 11:51	0	-0.8	3.5	22.5	59.7	299.4	8.1563	1.6
4/18/2018 11:52	0	0.1	3.3	22.8	58.9	302.6	8.1927	1.6
4/18/2018 11:53	0	-0.2	3	22.5	58	303.8	8.0808	1.6
4/18/2018 11:54	0	0.1	2.9	22	56.7	303.1	7.9874	1.6
4/18/2018 11:55	0	0.2	3.7	22.3	55.6	300.7	8.0137	1.6
4/18/2018 11:56	0	0	2.8	21.8	54.5	293.3	7.7433	1.6
4/18/2018 11:57	0.1	-0.3	3.5	22.2	53.5	272.9	7.8842	1.6
4/18/2018 11:58	0.1	0.4	4.3	22.4	55.5	296.2	8.2201	1.6
4/18/2018 11:59	0.1	0.6	2.1	21.9	52.8	293.5	7.7194	1.6
4/18/2018 12:00	0.1	0.5	4.1	21.9	53.6	298.8	8.0038	1.6
4/18/2018 12:01	0.1	0	2.1	21.7	52.3	291.6	7.7768	1.6
4/18/2018 12:02	0.1	0.7	2.2	22.1	51.5	277.6	7.8307	1.5
4/18/2018 12:03	0.1	0.3	3.4	22.1	51.3	280.4	7.6162	1.6
4/18/2018 12:04	0.1	0.2	3.4	21.6	51.3	276.2	7.3009	1.6
4/18/2018 12:05	0.2	0	3.4	21.5	49.9	258.5	7.101	1.5
4/18/2018 12:06	0.2	-0.4	3.3	21.6	47.1	263	7.2507	1.6
4/18/2018 12:07	0.2	-0.4	1.6	21.8	48.2	276.4	7.623	1.6
4/18/2018 12:08	0.2	0	1.3	21.5	47.9	276.5	7.4859	1.6
4/18/2018 12:09	0.2	0.3	3	21.6	47.6	274.4	7.5278	1.6
4/18/2018 12:10	0.2	-0.4	2.8	21.5	47.4	272.9	7.5119	1.6
4/18/2018 12:11	0.2	0.4	3.6	21.7	46.7	266.6	7.4624	1.6
4/18/2018 12:12	0.2	-0.1	3.5	21.2	46.1	270.7	7.4089	1.6
4/18/2018 12:13	0.2	-0.2	2	21.1	44.6	266.1	7.1682	1.6
4/18/2018 12:14	0.3	-0.3	3.5	20.9	43.2	261.7	6.874	1.6
4/18/2018 12:15	0.3	1.1	2.4	21.2	42.1	237.1	6.8018	1.6
4/18/2018 12:16	0.3	0.3	3	21.4	42.9	252.6	7.0268	1.6

4/18/2018 12:17	0.2	0.2	2.9	21.1	42	256.5	6.9101	1.6
4/18/2018 12:18	0.2	0.1	4.1	20.8	41.5	248.1	6.7638	1.7
4/18/2018 12:19	0.2	0.5	2.6	20.2	38.4	216.8	6.1432	1.6
4/18/2018 12:20	0.3	0.1	2.6	21.1	40.1	231.9	6.7508	1.6
4/18/2018 12:21	0.3	-0.3	2.9	21.1	40.6	245.2	6.7978	1.6
4/18/2018 12:22	0.3	-0.1	2	20.8	39.6	242.3	6.6553	1.7
4/18/2018 12:23	0.3	0.1	2.5	20.7	38	233.2	6.3375	1.7
4/18/2018 12:24	0.2	0.3	2	21.1	38.5	235.4	6.6346	1.7
4/18/2018 12:25	0.2	0.3	2.6	20.8	38.4	235.2	6.6082	1.8
4/18/2018 12:26	0.1	0	1	20.4	37.9	234.8	6.5037	1.8
4/18/2018 12:27	0.1	1	2.9	20.8	36	228	6.1388	1.8
4/18/2018 12:28	0.1	0.2	0.9	20.9	36.7	232.1	6.4688	1.8
4/18/2018 12:29	0.1	-0.1	2.9	20.3	34.4	216.4	6.0726	1.7
4/18/2018 12:30	0.1	0.3	2.3	19.6	30.2	196.8	5.2688	1.8
4/18/2018 12:31	0.1	0.4	2.9	19.2	28.1	175.9	4.8666	1.7
4/18/2018 12:32	0.1	-0.2	2.1	19.1	25.6	156.6	4.5458	1.7
4/18/2018 12:33	0.2	0.5	3.2	18.4	26.3	144.3	4.2275	1.6
4/18/2018 12:34	0.4	0.8	2.1	18.4	24.9	114	3.1996	1.7
4/18/2018 12:35	0.5	1.6	2.5	18.6	24.4	91.1	2.9205	1.8
4/18/2018 12:36	0.7	1	2.6	18.7	23.5	75.7	2.5945	1.7
4/18/2018 12:37	0.8	1.4	1.6	18.7	23.1	66.9	2.3728	1.5
4/18/2018 12:38	1	2.2	5.1	19.1	22.5	59.3	2.0923	1.4
4/18/2018 12:39	1.3	2.2	4	19.3	22.1	48.4	1.8655	1.4
4/18/2018 12:40	1.6	1.9	2.7	19.3	21.9	47	1.8295	1.6
4/18/2018 12:41	1.7	1.9	3.4	19.5	21.7	47.2	1.8168	1.6
4/18/2018 12:42	1.7	1.9	3.3	19.3	21.7	47.1	1.7255	1.6
4/18/2018 12:43	1.8	2.7	3.2	19.8	21.5	46.1	1.6123	1.5
4/18/2018 12:44	1.8	2.5	2.7	19.7	21.5	45.4	1.6418	1.4
4/18/2018 12:45	1.9	2.3	3.4	19.8	21.5	44.8	1.6966	1.5
4/18/2018 12:46	2	2.5	3.5	20.1	20.8	43.5	1.5042	1.5
4/18/2018 12:47	2.3	3	2.9	20.3	20.5	42	1.4515	1.5
4/18/2018 12:48	2.4	2.5	2.8	19.9	20.8	41	1.5443	1.4
4/18/2018 12:49	2.3	3.2	3.5	20.2	20.6	39.4	1.4284	1.5
4/18/2018 12:50	2.2	2.7	3	19.6	21.5	37.9	1.4653	1.5
4/18/2018 12:51	2.4	3.1	3.2	20.3	20.4	36.8	1.2902	1.5
4/18/2018 12:52	2.5	2.4	3.4	20.1	20.7	35.8	1.3818	1.6
4/18/2018 12:53	2.6	2.9	3.4	20.6	20.2	34.1	1.2626	1.6
4/18/2018 12:54	2.6	2.6	2.6	20.3	20.7	32.9	1.2931	1.6
4/18/2018 12:55	2.7	2.3	2.2	20	20.5	32	1.2498	1.5
4/18/2018 12:56	2.6	3.2	2.2	19.9	21	30.8	1.2252	1.5
4/18/2018 12:57	2.4	2.1	2.8	19.6	22	29.6	1.2111	1.5
4/18/2018 12:58	2.3	2.3	2.8	19.9	20.8	28.3	1.1231	1.4
4/18/2018 12:59	2.3	2.9	4.3	20	21.3	26.7	1.1039	1.4
4/18/2018 13:00	2.6	2.4	2.3	19.8	20.2	25.3	1.0208	1.4
4/18/2018 13:01	2.7	3.5	3	19.9	21.3	23.5	0.9877	1.4
4/18/2018 13:02	2.7	2.3	2.8	20.5	20.8	22.2	0.9552	1.4
4/18/2018 13:03	2.6	2.7	3.3	19.8	20.7	20.6	0.9479	1.5

4/18/2018 13:04	2.6	2.3	3.3	20.3	21.7	19	0.8939	1.6
4/18/2018 13:05	2.6	2.7	2.4	20.3	21.7	17.4	0.8267	1.7
4/18/2018 13:06	2.8	3.7	3.6	20.8	21	15.9	0.8065	1.9
4/18/2018 13:07	3.1	3.3	3.5	20.9	19.4	14.5	0.7829	1.9
4/18/2018 13:08	3.3	4.1	3.2	21.2	19.5	13.2	0.7454	2
4/18/2018 13:09	3.5	4		21.9	18.4	12.2	0.6963	1.6
4/18/2018 13:10	3.9	4.6		22.1	17.6	11.1	0.6866	1.4
4/18/2018 13:11	4.2	5.2		21.7	17.8	10.2	0.6604	1.4
4/18/2018 13:12	4.4	5.1	9.1	22.4	17.5	9.2	0.6216	1.4
4/18/2018 13:13	4.6	6.1	4.2	22.8	17	8.4	0.5902	1.4
4/18/2018 13:14	4.9	6.5		23	16.4	7.6	0.5536	1.5
4/18/2018 13:15	5.3	7	4.7	23.3	15.8	6.8	0.5473	1.6
4/18/2018 13:16	5.7	6.8	5.8	23.6	15.5	6.2	0.5139	1.6
4/18/2018 13:17	6	7.1	5.6	24.1	15.1	5.6	0.5049	1.5
4/18/2018 13:18	6.2	7.6		24.3	14.5	5	0.4899	1.4
4/18/2018 13:19	6.5	8.1	5	24.6	13.9	4.6	0.4637	1.4
4/18/2018 13:20	6.8	8.7	5	24.7	13.3	4.2	0.4605	1.4
4/18/2018 13:21	7.1	8.6	5.3	25.1	12.9	3.9	0.4315	1.4
4/18/2018 13:22	7.3	8.9		25.6	12.7	3.7	0.4209	1.4
4/18/2018 13:23	7.7	9.2		25.6	12.4	3.5	0.4025	1.4
4/18/2018 13:24	7.9	9.1		26.2	11.9	3.3	0.3895	1.4
4/18/2018 13:25	8.2	9.4	12.3	26.1	11.4	3.1	0.3887	1.4
4/18/2018 13:26	8.4	9.5	5.1	26.6	10.8	3.1	0.3796	1.3
4/18/2018 13:27	8.7	9.8		26.8	10.1	3	0.3565	1.3
4/18/2018 13:28	8.8	9		25.9	10.5	2.9	0.3499	1.3
4/18/2018 13:29	8.8	9.4		26	10.6	2.8	0.3683	1.3
4/18/2018 13:30	8.4	8.8		25.5	10.8	2.7	0.3737	1.3
4/18/2018 13:31	8	7.7	8.5	25.1	10.9	2.7	0.3616	1.3
4/18/2018 13:32	7.4	7.6	4.4	25	11	2.7	0.3814	1.3
4/18/2018 13:33	7	7.2	4	24.4	11	2.7	0.374	1.3
4/18/2018 13:34	6.6	6.9	4.1	23.8	11.1	2.8	0.3878	1.3
4/18/2018 13:35	6.2	6.3	3.3	23.9	11.1	2.7	0.3909	1.3
4/18/2018 13:36	5.9	6.5	4.9	23.6	11	2.8	0.3897	1.3
4/18/2018 13:37	5.6	5.8	3.4	23	11.1	2.9	0.4104	1.3
4/18/2018 13:38	5.3	5.6	4.3	22.9	11.1	3	0.3983	1.4
4/18/2018 13:39	5	5.4	4.9	22.8	11.1	3	0.4047	1.4
4/18/2018 13:40	4.8	4.2	2.5	22.2	16.3	7.3	0.4247	1.3
4/18/2018 13:41	3.5	0.9	2.4	22.3	50.2	81.7	0.6559	1.4
4/18/2018 13:42	2	0.9	3	26.1	80.9	204.7	1.1827	1.4
4/18/2018 13:43	0.7	0.5	3	30.8	103	304.1	1.7157	1.5
4/18/2018 13:44	0.3	0.5	3.6	39.1	113.2	335.4	2.3752	1.6
4/18/2018 13:45	0.2	0	4.2	48.4	120.9	365.5	3.236	1.7
4/18/2018 13:46	0.2	0.5	3.1	54.9	124.3	387	4.1543	1.8
4/18/2018 13:47	0.2	-0.2	5.4	65.1	123.1	385.9	5.5812	2
4/18/2018 13:48	0.2	0	4.4	64.7	121.9	381.1	6.0292	2.1
4/18/2018 13:49	0.2	0.4	4.9	67	117.6	380.2	6.9063	2.1
4/18/2018 13:50	0.2	0.6	7.5	64.1	113	374.4	7.4285	2.2

4/18/2018 13:51	0.2	-0.6	5.3	63.7	109.6	364.2	7.7868	2.2
4/18/2018 13:52	0.2	0	4.3	63.3	107.4	361.8	8.3997	2.2
4/18/2018 13:53	0.2	-0.5	6	58.7	100.6	351	8.1868	2.2
4/18/2018 13:54	0.2	-0.4	3.9	60.1	103.4	346.1	8.9283	2.2
4/18/2018 13:55	0.2	-0.4	3.5	59.7	102.4	352.2	9.2477	2.2
4/18/2018 13:56	0.1	0.5	5.8	58	99.9	346.3	9.452	2.2
4/18/2018 13:57	0.1	0.1	4.1	56.5	98.1	342.1	9.411	2.2
4/18/2018 13:58	0.1	0	5.1	54.9	95.7	334.9	9.505	2.1
4/18/2018 13:59	0.1	-0.3	3.5	52.7	92.7	322.7	9.3948	2.1
4/18/2018 14:00	0.1	0.3	2.4	47.8	83.6	322.8	8.8717	2.1
4/18/2018 14:01	0.1	0.4	4.8	51.1	89.5	319	9.457	2.1
4/18/2018 14:02	0.1	0.4	4	51	89.8	325.6	9.9623	2.1
4/18/2018 14:03	0.2	0.3	3.3	47.9	86.8	315.9	9.723	2.1
4/18/2018 14:04	0.2	0.3	4	47	84.2	308.9	9.5805	2.1
4/18/2018 14:05	0.2	0.1	3.9	46.4	84.4	308	9.561	2.1
4/18/2018 14:06	0.2	-0.9	3.4	44.1	84.2	303.8	9.1112	2.1
4/18/2018 14:07	0.2	0.2	4.5	43.9	83.6	300.2	9.28	2.1
4/18/2018 14:08	0.2	0.1	4.3	42.8	77.9	287.3	9.1275	2.1
4/18/2018 14:09	0.2	-0.1	3	42	73.8	289.1	8.8305	2
4/18/2018 14:10	0.2	0.4	3.4	42.1	75.9	292.8	9.276	2
4/18/2018 14:11	0.2	0.2	4.3	40	72.5	285.5	8.773	2
4/18/2018 14:12	0.2	0.3	4.4	40.6	73	283.2	8.9707	2
4/18/2018 14:13	0.2	-0.1	3.4	40.4	73	287.2	9.161	2.1
4/18/2018 14:14	0.2	0.2	4.4	38.1	67.6	268.7	8.4122	2.1
4/18/2018 14:15	0.2	0.9	3.7	38.3	69.4	274.2	8.7983	2
4/18/2018 14:16	0.3	-0.1	3.4	38.5	67.2	276	8.5797	2
4/18/2018 14:17	0.3	0.1	3.4	38	67.9	274.7	8.677	2
4/18/2018 14:18	0.3	0	4.4	36.5	64.4	264	8.2145	2
4/18/2018 14:19	0.3	0.5	3.2	37	65.8	263.2	8.6312	2
4/18/2018 14:20	0.3	0	3.5	35.9	64.1	261.6	8.3775	2
4/18/2018 14:21	0.2	-0.1	3.5	35.1	62.4	255.8	8.1593	2
4/18/2018 14:22	0.2	0.2	3.3	35.1	62.7	258.8	8.3578	2
4/18/2018 14:23	0.2	0.9	3.5	34.9	60.2	252.2	8.0565	2
4/18/2018 14:24	0.2	-0.2	3.9	34.8	60.9	254.7	8.324	2
4/18/2018 14:25	0.1	0.5	2.9	33.8	59.2	245.9	8.0977	2
4/18/2018 14:26	0.1	0.3	3.1	31.2	55.9	228.1	7.4878	2
4/18/2018 14:27	0.1	0.2	4.5	32.2	54	219.6	7.4133	2
4/18/2018 14:28	0.1	0.1	1.7	31.6	53.7	229.9	7.3543	2
4/18/2018 14:29	0.1	-0.1	2	32.4	54.8	235.2	7.681	2
4/18/2018 14:30	0.1	0.6	3.1	32.6	56.2	240.7	8.002	2
4/18/2018 14:31	0.1	-0.1	4.6	32.2	54.9	233	7.7547	2.1
4/18/2018 14:32	0.1	0.5	3.9	31.3	53.1	228.5	7.5242	2
4/18/2018 14:33	0.1	0.5	3.7	31.4	52.8	227.4	7.5747	1.9
4/18/2018 14:34	0.2	0.8	4	31.5	52.4	227.6	7.6108	1.9
4/18/2018 14:35	0.2	0.4	2.1	30.4	50.9	223	7.3297	1.9
4/18/2018 14:36	0.2	-0.2	3.2	29.8	48.9	217.1	7.0582	1.9
4/18/2018 14:37	0.2	0.6	3.9	30.2	49.3	216.9	7.2395	1.9

4/18/2018 14:38	0.2	0.1	2.4	29.8	48.7	218.4	7.1488	1.9
4/18/2018 14:39	0.2	-0.3	3.6	30	48.4	215.9	7.234	1.8
4/18/2018 14:40	0.2	0.7	2.2	30	47.7	214.2	7.1445	1.9
4/18/2018 14:41	0.2	0.6	2.8	29.8	47.5	216.3	7.1715	1.9
4/18/2018 14:42	0.2	-0.2	1.1	29.3	46.9	216.7	7.1428	1.9
4/18/2018 14:43	0.2	-0.2	3.5	27.7	44.2	205.7	6.5693	1.8
4/18/2018 14:44	0.2	0.3	5.5	27	42.8	195.9	6.38	1.9
4/18/2018 14:45	0.2	0.6	1.7	27.6	41.2	189.2	6.1935	1.9
4/18/2018 14:46	0.2	0.6	3.5	27.6	42.2	188.6	6.511	1.8
4/18/2018 14:47	0.2	0.1	3.1	26.9	41.2	195.6	6.2567	1.8
4/18/2018 14:48	0.3	0.1	1.9	27.2	41.3	194.6	6.415	1.8
4/18/2018 14:49	0.3	0.4	3.2	27.7	41.1	195.5	6.5465	1.8
4/18/2018 14:50	0.3	0.4	2.2	27.3	41.2	195.7	6.5652	1.8
4/18/2018 14:51	0.4	0.3	4	27	39.7	186.5	6.2663	1.8
4/18/2018 14:52	0.3	0.6	1.1	26.8	40	184.5	6.3125	1.8
4/18/2018 14:53	0.3	0.7	3.3	25.9	38.3	178.4	6.0265	1.7
4/18/2018 14:54	0.3	0.8	2	26.3	37.3	177.6	6.0188	1.8
4/18/2018 14:55	0.3	1.3	2	26.3	37.7	182	6.1602	1.8
4/18/2018 14:56	0.2	0.8	5.1	26.3	37.6	180.1	6.121	1.8
4/18/2018 14:57	0.2	0.5	2.9	26.4	37.2	178.6	4.1372	1.7
4/18/2018 14:58	0.2	0.1	3.4	25.6	36.1	178.2	0.3	1.4
4/18/2018 14:59	0.1	0.3	2.4	26	35.1	173.6	-0.0472	1.4
4/18/2018 15:00	0.2	1.1	3.5	25.5	35.7	172.7	-0.0439	1.4
4/18/2018 15:01	0.2	0.9	2.8	25.6	34.1	171.5	-0.0641	1.4
4/18/2018 15:02	0.2	0.6	3.6	24.5	33.5	150.9	-0.0439	1.4
4/18/2018 15:03	0.3	1.6	1.8	20.3	29.3	115.4	-0.0552	1.4
4/18/2018 15:04	0.5	1.3	1	19.5	25	61.8	-0.0555	1.4
4/18/2018 15:05	0.8	1.3	1.6	19.4	23.2	51.2	-0.0448	1.4
4/18/2018 15:06	0.9	2	2.7	19	22.7	48.4	-0.0567	1.4
4/18/2018 15:07	0.9	1.3	2.9	19.1	22.7	43.5	-0.0463	1.4
4/18/2018 15:08	0.8	1.2	2.1	19.3	22.6	41.8	-0.0607	1.4
4/18/2018 15:09	0.9	2	2	19.1	21	41.2	-0.0322	1.4
4/18/2018 15:10	0.9	1.5	1.6	18.7	20.1	40.4	0.0133	1.4
4/18/2018 15:11	1.1	2.9	2.1	19.1	19.8	39		1.4
4/18/2018 15:12	1.1	1.8	0.8	19.3	20	37.8		1.5
4/18/2018 15:13	1.2	1.7	1.7	19.1	19.8	36.6		1.4
4/18/2018 15:14	1.2	1.5	1.8	19.3	19.1	34.7		1.4
4/18/2018 15:15	1.2	1.9	3	18.9	19.3	33.1		1.4
4/18/2018 15:16	1.3	2.2	2.6	19.3	19.1	31.4		1.5
4/18/2018 15:17	1.4	1.6	1.7	19.3	18.3	29.7		1.5
4/18/2018 15:18	1.5	2.1	2.1	19.7	18.2	28.2		1.4
4/18/2018 15:19	1.6	1.7	3.2	19.6	18	27.2		1.5
4/18/2018 15:20	1.6	2.2	2.6	19.2	17.5	26		1.5
4/18/2018 15:21	1.6	2.4	2.5	19.6	17.3	24.7		1.5
4/18/2018 15:22	1.6	2.1	2.1	19.7	17.1	23.9		1.6
4/18/2018 15:23	1.7	2.2	3.7	19.7	16.3	20	2.678	1.5
4/18/2018 15:24	2.2	7.1	8.1	24.7	16.2	16.5	1.178	1.4

4/18/2018 15:25			16.1	16.6	1.094	1.5
4/18/2018 15:26			15.8	14.8	1.0573	1.5
4/18/2018 15:27			16.1	14.9	1.092	1.5
4/18/2018 15:28			16.2	15	1.1005	1.5
4/18/2018 15:29			15.5	15	1.0049	1.6
4/18/2018 15:30			15.2	14.8	0.9992	1.6
4/18/2018 15:31			14.7	14.5	0.9143	1.5
4/18/2018 15:32			15.1	14.4	0.9983	1.6
4/18/2018 15:33			14.8	14.1	0.9627	1.6
4/18/2018 15:34			14.7	13.8	0.9066	1.6
4/18/2018 15:35			14.5	13.3	0.8977	1.6
4/18/2018 15:36			14.8	12.8	0.928	1.6
4/18/2018 15:37			13.5	12.2	0.8067	1.6
4/18/2018 15:38			13.1	11.5	0.7259	1.6
4/18/2018 15:39			13.6	11	0.8038	1.6
4/18/2018 15:40			13.8	10.6	0.8268	1.6
4/18/2018 15:41			13	10.2	0.747	1.6
4/18/2018 15:42			12.4	9.6	0.7075	1.7
4/18/2018 15:43			12.8	9.2	0.7345	1.6
4/18/2018 15:44			12.9	8.8	0.7631	1.6
4/18/2018 15:45			12.7	8.6	0.7552	1.5
4/18/2018 15:46			12.1	8.5	0.6696	1.6
4/18/2018 15:47			12.2	8.4	0.6982	1.6
4/18/2018 15:48			12	8	0.6668	1.6
4/18/2018 15:49			11.3	7.6	0.6239	1.7
4/18/2018 15:50			11.3	7.4	0.6296	1.8
4/18/2018 15:51			11.2	7.1	0.607	1.8
4/18/2018 15:52			10.9	6.8	0.5937	1.9
4/18/2018 15:53			10.4	6.3	0.5502	2.4
4/18/2018 15:54			10.2	5.9	0.5481	2.7
4/18/2018 15:55			10.2	5.7	0.5528	2.8
4/18/2018 15:56	4.9	27	9.7	5.4	0.5354	2.7
4/18/2018 15:57	3.1	23.7	9.2	5.1	0.4699	3.1
4/18/2018 15:58	5.3	23	9.1	4.9	0.4814	3.2
4/18/2018 15:59	3.8	22.6	9	4.7	0.4887	2.6
4/18/2018 16:00	4.1	21.8	8.8	4.4	0.4578	1.4
4/18/2018 16:01	4.7	23	8.8	4.2	0.4671	1.4
4/18/2018 16:02	5.4	21.9	8.3	4.1	0.4325	1.4
4/18/2018 16:03	4	22	8.1	3.9	0.4337	1.4
4/18/2018 16:04	5.8	21.9	8.1	3.8	0.4338	1.5
4/18/2018 16:05	5.3	22.3	8	3.6	0.4266	1.7
4/18/2018 16:06	5	22.1	8	3.5	0.4343	1.9
4/18/2018 16:07	4.6	22.4	7.8	3.4	0.4132	2.1
4/18/2018 16:08	5.4	22.2	7.7	3.3	0.4127	2.2
4/18/2018 16:09	5.2	22.3	7.7	3.2	0.3711	2
4/18/2018 16:10	5.6	22.7	7.1	3.1	0.3695	1.4
4/18/2018 16:11	5	22.3	7.1	3.1	0.3887	1.3

4/18/2018 16:12	5.6	22.6	6.8	3	0.3558	1.4
4/18/2018 16:13	5.8	22.5	6.5	2.9	0.3497	1.4
4/18/2018 16:14	5.4	23.3	6.4	2.8	0.3405	1.4
4/18/2018 16:15	6	23.4	6.3	2.7	0.3409	1.4
4/18/2018 16:16	5.3	23.7	6.4	2.6	0.3499	1.4
4/18/2018 16:17	4.9	23.3	6.1	2.5	0.3183	1.4
4/18/2018 16:18	5.1	23.5	6.1	2.5	0.3385	1.4
4/18/2018 16:19	6.9	23.1	6	2.4	0.3247	1.4
4/18/2018 16:20	4.9	23.6	5.7	2.3	0.3137	1.4
4/18/2018 16:21	6.4	23.7	5.7	2.3	0.3092	1.4
4/18/2018 16:22	5.3	24.2	5.7	2.3	0.3207	1.4
4/18/2018 16:23	4.4	24.3	5.6	2.3	0.2974	1.4
4/18/2018 16:24	6.5	24.4	5.4	2.2	0.2925	1.5
4/18/2018 16:25	5.8	24.2	5.3	2.2	0.2847	1.6
4/18/2018 16:26	6.1	24.4	5.1	2.1	0.2724	1.6
4/18/2018 16:27	5.8	24.6	5.1	2.1	0.296	1.6
4/18/2018 16:28	4.8	24.3	5	2.1	0.2606	1.7
4/18/2018 16:29	6.7	24.8	5	2.1	0.2887	1.7
4/18/2018 16:30	7.3	25	4.9	2.1	0.2654	1.6
4/18/2018 16:31	5.2	25	4.7	2	0.2602	1.5
4/18/2018 16:32	6.4	25.5	4.7	2	0.2637	1.5
4/18/2018 16:33	7.9	25.2	4.7	1.9	0.2481	1.6
4/18/2018 16:34	6.1	26.4	4.4	1.9	0.2392	1.7
4/18/2018 16:35	6.8	25.3	4.5	1.9	0.2327	1.7
4/18/2018 16:36	4.5	24.8	5.1	1.9	0.2907	1.8
4/18/2018 16:37	7	24.5	5.2	1.9	0.2734	1.8
4/18/2018 16:38	5.8	25.8	4.4	1.9	0.2403	1.8
4/18/2018 16:39	5.7	25.1	4.3	1.9	0.2433	1.8
4/18/2018 16:40	5.8	25.6	4.4	1.9	0.2371	1.8
4/18/2018 16:41	6.4	25.2	4.4	1.9	0.2544	1.9
4/18/2018 16:42	6.8	24.5	4.4	1.9	0.2459	1.9
4/18/2018 16:43	6.9	24.7	4.4	2	0.2509	1.9
4/18/2018 16:44		24.4	4.4	2	0.2416	1.9
4/18/2018 16:45		24.6	4.5	2	0.2598	1.8
4/18/2018 16:46		23.9	4.5	2.1	0.2539	1.9
4/18/2018 16:47		24.1	4.5	2.1	0.2624	1.9
4/18/2018 16:48	4.9	23.9	4.5	2.2	0.2585	2
4/18/2018 16:49	5.9	23.6	4.5	2.2	0.2534	2
4/18/2018 16:50	5.1	23.1	4.6	2.3	0.27	2
4/18/2018 16:51	9.1	23.1	4.5	2.4	0.251	2.1
4/18/2018 16:52	4.6	23.1	4.5	2.4	0.2625	2.1
4/18/2018 16:53	5.7	22.9	4.5	2.5	0.258	2.1
4/18/2018 16:54	4.3	23.1	4.6	2.5	0.2631	2.1
4/18/2018 16:55	5.4	22.6	4.5	2.6	0.2563	2.1
4/18/2018 16:56	4	22.5	4.5	2.6	0.2411	2.1
4/18/2018 16:57	4.5	22.2	4.5	2.6	0.2544	2.2
4/18/2018 16:58	4.1	22.5	4.5	2.6	0.2361	2.2

4/18/2018 16:59	5.1	21.8	4.4	2.6	0.2312	2.2
4/18/2018 17:00	5	22.4	4.3	2.6	0.2236	2.3
4/18/2018 17:01	5	22.5	4.3	2.6	0.2273	2.3
4/18/2018 17:02	5	22.1	4.3	2.5	0.2347	2.3
4/18/2018 17:03	5.3	22.2	4.3	2.5	0.2307	2.3
4/18/2018 17:04	5.4	22.2	4.4	2.5	0.237	2.2
4/18/2018 17:05	2.8	21.8	4.4	2.5	0.227	2.1
4/18/2018 17:06	5	22.1	4.4	2.5	0.2325	2.1
4/18/2018 17:07	4.4	21.7	4.4	2.5	0.2161	2.2
4/18/2018 17:08	4.5	21.1	4.4	2.5	0.2474	2.2
4/18/2018 17:09	5.7	21.2	4.4	2.6	0.2578	2.2
4/18/2018 17:10	5.3	21	4.4	2.6	0.243	2.1
4/18/2018 17:11	4.4	20.9	4.4	2.7	0.2581	2.1
4/18/2018 17:12	3.6	21.1	4.4	2.8	0.24	2.2
4/18/2018 17:13	4.4	20.9	4.3	2.8	0.2565	2.2
4/18/2018 17:14	5.3	21.1	4.3	2.9	0.2416	2
4/18/2018 17:15	4.9	21	4.3	2.9	0.2513	2.1
4/18/2018 17:16	3.5	20.8	4.2	3	0.2435	2.2
4/18/2018 17:17	3.4	21.8	4.1	2.9	0.232	2.2
4/18/2018 17:18	4.1	21.6	4	2.9	0.2332	2.2
4/18/2018 17:19	5.3	22.2	4	2.8	0.2065	2.2
4/18/2018 17:20	4.1	22.4	3.9	2.7	0.2334	2.2
4/18/2018 17:21	4.4	21.7	3.9	2.7	0.2145	2.3
4/18/2018 17:22	5	22	3.9	2.6	0.2239	2.3
4/18/2018 17:23	3.9	21.2	3.9	2.6	0.2272	2.3
4/18/2018 17:24	4.4	20.8	4	2.6	0.2426	2.3
4/18/2018 17:25	2.8	20.8	4	2.6	0.246	2.3
4/18/2018 17:26	3.6	20.3	4.1	2.7	0.2338	2.3
4/18/2018 17:27	4.1	20.3	4.1	2.8	0.2646	2.3
4/18/2018 17:28	3	20.8	4.1	2.9	0.2445	2.4
4/18/2018 17:29	3.9	20.6	4	2.9	0.2463	2.3
4/18/2018 17:30	5.2	20.8	4	3	0.2303	2.4
4/18/2018 17:31	4.4	20.8	4	3.1	0.2358	2.4
4/18/2018 17:32	4.9	20.9	4	3.1	0.2381	2.5
4/18/2018 17:33	4.2	20.8	4	3.1	0.2263	2.5
4/18/2018 17:34	4.4	21.7	3.9	3.1	0.2289	2.4
4/18/2018 17:35	5.1	22	3.8	3	0.2076	2.5
4/18/2018 17:36	5.2	22.1	3.8	2.9	0.2156	2.5
4/18/2018 17:37	5.4	22.3	3.8	2.8	0.2042	2.4
4/18/2018 17:38	4.1	21.9	3.8	2.7	0.2141	2.4
4/18/2018 17:39	4.4	21.1	3.8	2.7	0.2154	2.4
4/18/2018 17:40	2.3	20.3	3.9	2.7	0.2328	2.4
4/18/2018 17:41	4.3	20.3	3.9	2.7	0.2418	2.3
4/18/2018 17:42	4.5	20.2	4	2.7	0.2359	2.3
4/18/2018 17:43	3.3	20.1	4	2.8	0.2435	2.3
4/18/2018 17:44	2.8	19.8	3.9	2.9	0.2328	2.3
4/18/2018 17:45	3.9	20.1	3.9	3	0.2402	2.3

4/18/2018 17:46	4.2	20.2	3.9	3.1	0.2345	2.3
4/18/2018 17:47	4.6	20.5	3.8	3.2	0.2293	2.4
4/18/2018 17:48	3	20.4	3.8	3.2	0.2315	2.4
4/18/2018 17:49	4.2	20.5	3.8	3.2	0.2217	2.3
4/18/2018 17:50	3.9	20.7	3.8	3.2	0.2391	2.2
4/18/2018 17:51	1.8	21.4	3.7	3.2	0.207	2.2
4/18/2018 17:52	4.9	21.6	3.7	3.1	0.2222	2.3
4/18/2018 17:53	3.9	22.4	3.6	3	0.2055	2.3
4/18/2018 17:54	5.4	22	3.6	2.8	0.2069	2.3
4/18/2018 17:55	4.5	22.2	3.6	2.7	0.2083	2.2
4/18/2018 17:56	5	21.5	3.6	2.7	0.201	2.2
4/18/2018 17:57	4.5	20.3	3.6	2.7	0.2456	2.2
4/18/2018 17:58	4.7	20.2	3.7	2.7	0.2205	2.1
4/18/2018 17:59	4.5	20	3.7	2.7	0.241	2
4/18/2018 18:00	1.6	19.9	3.8	2.8	0.2281	2
4/18/2018 18:01	2.7	19.8	3.7	2.9	0.2411	2
4/18/2018 18:02	3.1	19.9	3.7	3.1	0.2382	2
4/18/2018 18:03	2.7	20.2	3.6	3.2	0.2228	2.1
4/18/2018 18:04	2.7	20.6	3.6	3.2	0.2345	2.1
4/18/2018 18:05	3.6	20.3	3.6	3.2	0.2141	2.2
4/18/2018 18:06	3.9	20.4	3.6	3.2	0.2296	2.1
4/18/2018 18:07	4.1	20.4	3.6	3.2	0.2116	2.1
4/18/2018 18:08	4.3	21.7	3.5	3.2	0.2114	2
4/18/2018 18:09	5.4	21.9	3.4	3.1	0.2118	1.9
4/18/2018 18:10	4.6	22.2	3.4	3	0.1943	2
4/18/2018 18:11	5.7	23	3.4	2.8	0.2052	2.1
4/18/2018 18:12	5.2	22.7	3.4	2.7	0.1942	2.2
4/18/2018 18:13	3.6	21.7	3.4	2.7	0.2121	2.3
4/18/2018 18:14	4.3	20.6	3.5	2.7	0.2207	2.3
4/18/2018 18:15	3.9	20.6	3.5	2.7	0.2216	2.3
4/18/2018 18:16	4.7	20	3.6	2.7	0.2271	2.3
4/18/2018 18:17	2.4	19.8	3.6	2.8	0.2263	2.3
4/18/2018 18:18	2.8	19.8	3.6	2.9	0.2369	2.2
4/18/2018 18:19	3.9	20.3	3.6	3.1	0.2167	2.3
4/18/2018 18:20	4	20.1	3.5	3.2	0.2308	2.4
4/18/2018 18:21	3.4	20.4	3.5	3.2	0.2154	2.4
4/18/2018 18:22	1.2	20.3	3.4	3.3	0.2254	2.4
4/18/2018 18:23	4.9	20.2	3.5	3.3	0.2222	2.2
4/18/2018 18:24	4.9	20.4	3.4	3.3	0.2184	2.2
4/18/2018 18:25	3.7	21.9	3.3	3.2	0.2178	2.3
4/18/2018 18:26	5.2	21.9	3.2	3.1	0.1967	2.2
4/18/2018 18:27	4.5	22.6	3.2	3	0.2017	2.3
4/18/2018 18:28	5.3	22.5	3.2	2.8	0.1872	2.4
4/18/2018 18:29	4.4	23	3.1	2.7	0.1959	2.4
4/18/2018 18:30	4.9	21.4	3.2	2.6	0.2036	2.4
4/18/2018 18:31	4	20.7	3.3	2.6	0.2188	2.4
4/18/2018 18:32	3.2	20.4	3.3	2.6	0.2197	2.1

4/18/2018 18:33	3.1	19.8	3.4	2.7	0.2145	2.1
4/18/2018 18:34	2.8	20	3.4	2.8	0.2243	2.1
4/18/2018 18:35	2.4	20.1	3.4	2.9	0.2179	2.1
4/18/2018 18:36	1.7	20.2	3.4	3	0.2267	2
4/18/2018 18:37	3.9	19.9	3.3	3.2	0.2139	2
4/18/2018 18:38	3.1	20.3	3.3	3.3	0.2135	2.1
4/18/2018 18:39	4.2	20.4	3.2	3.3	0.218	2.2
4/18/2018 18:40	3.5	20.5	3.2	3.3	0.2028	2.3
4/18/2018 18:41	4	20.2	3.2	3.3	0.2257	2.3
4/18/2018 18:42	3.5	20.3	3.2	3.2	0.2077	2.4
4/18/2018 18:43	4.7	22.3	3	3.2	0.2068	2.4
4/18/2018 18:44	5.8	22.2	2.9	3.1	0.1966	2.5
4/18/2018 18:45	6.1	23.2	2.9	2.9	0.198	2.5
4/18/2018 18:46	4.5	23	2.8	2.8	0.197	2.5
4/18/2018 18:47	5.4	23	2.9	2.7	0.1919	2.5
4/18/2018 18:48	5.1	21.7	2.9	2.6	0.2021	2.4
4/18/2018 18:49	4.3	20.5	3.1	2.6	0.209	2.4
4/18/2018 18:50	5.5	20.9	3.1	2.6	0.2086	2.3
4/18/2018 18:51	3.5	20	3.1	2.6	0.2101	1.9
4/18/2018 18:52	4.2	19.9	3.2	2.7	0.2126	2.2
4/18/2018 18:53	3.4	19.9	3.1	2.9	0.2172	2.4
4/18/2018 18:54	3.7	20.2	3.1	3	0.2064	2.6
4/18/2018 18:55	3.9	20.2	3.1	3.1	0.2169	2.6
4/18/2018 18:56	3.3	20.5	3.1	3.2	0.2058	2.6
4/18/2018 18:57	3.2	21	3.1	3.2	0.2212	2.6
4/18/2018 18:58	4.1	21	3	3.2	0.2044	2.5
4/18/2018 18:59	5.4	22.2	2.9	3.1	0.1978	2.6
4/18/2018 19:00	5	22.3	2.9	3	0.2	2.5
4/18/2018 19:01	6.8	23	2.8	2.8	0.1788	1.9
4/18/2018 19:02	4.3	23.5	2.8	2.7	0.1989	1.5
4/18/2018 19:03	6.4	24.1	2.8	2.6	0.1758	1.6
4/18/2018 19:04	3.6	22	2.8	2.4	0.1993	1.8
4/18/2018 19:05	4.3	20.9	3	2.5	0.1985	1.9
4/18/2018 19:06	3.4	20.6	3	2.5	0.2121	2
4/18/2018 19:07	1.8	20	3	2.6	0.2121	2.1
4/18/2018 19:08	3.4	19.7	3	2.7	0.2128	2
4/18/2018 19:09	2.7	20	3	2.8	0.2168	2
4/18/2018 19:10	3.9	20	3	3	0.2024	2.1
4/18/2018 19:11	4.2	20.2	3	3.1	0.2181	2.1
4/18/2018 19:12	4.1	20.7	3	3.3	0.1934	2.3
4/18/2018 19:13	4.2	20.3	3	3.3	0.2131	2.3
4/18/2018 19:14	3.3	20.8	3	3.3	0.2112	2.3
4/18/2018 19:15	5.3	22.4	3	3.2	0.1831	2.3
4/18/2018 19:16	4.5	23	2.9	3.1	0.1941	2.4
4/18/2018 19:17	7.6	24.2	2.9	2.9	0.1743	2.5
4/18/2018 19:18	5.2	24.4	2.9	2.7	0.1878	2.5
4/18/2018 19:19	5.8	25.1	3	2.6	0.1863	2.5

4/18/2018 19:20	5	23.3	3	2.5	0.1873	2.5
4/18/2018 19:21		21.4	3	2.5	0.2006	2.5
4/18/2018 19:22	3.5	20.9	3.1	2.4	0.1965	2.4
4/18/2018 19:23	2.9	20.7	3.1	2.5	0.2059	2.5
4/18/2018 19:24		20.3	3.1	2.6	0.1944	2.5
4/18/2018 19:25	3.2	20.2	3.1	2.7	0.2071	2.6
4/18/2018 19:26	2.5	20.5	3.1	2.8	0.2002	2.5
4/18/2018 19:27	3.5	21.3	3.2	2.9	0.1944	2.5
4/18/2018 19:28		21.9	3.3	3	0.1965	2.5
4/18/2018 19:29		21.5	3.3	3	0.1901	2.6
4/18/2018 19:30	5	21.8	3.3	2.9	0.2072	2.6
4/18/2018 19:31	7.8	22.8	3.6	2.9	0.1872	2.5
4/18/2018 19:32		24.3	3.9	2.8	0.1928	2.4
4/18/2018 19:33	5.9	25	3.9	2.6	0.1759	2.3
4/18/2018 19:34		25.5	4.1	2.5	0.1817	2.3
4/18/2018 19:35		23.3	3.9	2.4	0.1859	2.5
4/18/2018 19:36	3.2	21.4	3.4	2.4	0.2044	2.5
4/18/2018 19:37	4.9	21.6	3.4	2.4	0.2113	2.4
4/18/2018 19:38		21	3.4	2.4	0.1959	2.3
4/18/2018 19:39	4.6	20.1	3.3	2.5	0.2139	2.3
4/18/2018 19:40	5.9	20.4	3.3	2.6	0.1945	2.3
4/18/2018 19:41	4.8	20.6	3.3	2.7	0.2124	2.3
4/18/2018 19:42	3.8	20.5	3.2	2.9	0.1988	2.3
4/18/2018 19:43	3.7	20.8	3.3	3	0.2075	2.4
4/18/2018 19:44	3.9	21.6	3.5	3	0.2016	2.3
4/18/2018 19:45		21.4	3.5	3	0.193	2.4
4/18/2018 19:46	4.5	21.5	3.5	3	0.1981	2.3
4/18/2018 19:47	4.5	22	3.4	2.9	0.188	2.3
4/18/2018 19:48	4.8	22.5	3.6	2.9	0.186	2.2
4/18/2018 19:49	6.2	24.2	3.7	2.7	0.1795	2
4/18/2018 19:50	6	25	3.7	2.6	0.1823	2.1
4/18/2018 19:51	7.1	25.8	3.8	2.4	0.1833	2.2
4/18/2018 19:52	8.4	25.6	3.8	2.3	0.1702	2.2
4/18/2018 19:53	4.5	23	3.6	2.2	0.1837	2.2
4/18/2018 19:54	2.1	21.3	3.4	2.3	0.184	2.3
4/18/2018 19:55	3.4	21.4	3.3	2.3	0.1957	2.4
4/18/2018 19:56	4.1	20.8	3.4	2.3	0.1866	2.4
4/18/2018 19:57	4.2	20.8	3.3	2.3	0.1879	2.4
4/18/2018 19:58	2.9	20.6	3.3	2.5	0.1981	2.6
4/18/2018 19:59	4.3	20.6	3.2	2.6	0.1837	2.8
4/18/2018 20:00	4.3	20.9	3.3	2.7	0.1931	2.8
4/18/2018 20:01	5.1	21.3	3.2	2.9	0.1852	2.9
4/18/2018 20:02	4.5	21.5	3.3	2.9	0.1935	2.9
4/18/2018 20:03	4.3	21.4	3.3	2.9	0.1851	2.8
4/18/2018 20:04	5.8	21.8	3.3	2.9	0.196	2.8
4/18/2018 20:05	5.4	22.6	3.3	2.8	0.1873	2.8
4/18/2018 20:06	6.6	24.6	3.4	2.7	0.1724	2.8

4/18/2018 20:07	6.1	25.3	3.4	2.6	0.1792	2.9
4/18/2018 20:08	6.2	25.5	3.4	2.4	0.1668	2.9
4/18/2018 20:09	4.8	23.5	3.3	2.3	0.1816	2.8
4/18/2018 20:10	4	21.5	3.2	2.3	0.1842	2.7
4/18/2018 20:11	4	21.7	3.2	2.3	0.1919	2.4
4/18/2018 20:12	4.6	21	3.3	2.3	0.1924	2.3
4/18/2018 20:13	3.7	20.8	3.3	2.3	0.1838	2.2
4/18/2018 20:14	4.2	20.5	3.3	2.4	0.1964	2.2
4/18/2018 20:15	5.1	20.5	3.3	2.5	0.1839	2.3
4/18/2018 20:16	4.9	21.4	3.3	2.6	0.1911	2.4
4/18/2018 20:17	3.8	21.3	3.3	2.7	0.177	2.4
4/18/2018 20:18	3.7	21.7	3.3	2.8	0.1957	2.4
4/18/2018 20:19	4.4	21.1	3.3	2.8	0.1903	2.4
4/18/2018 20:20	4.7	22.5	3.4	2.7	0.1792	2.4
4/18/2018 20:21	5.4	24.2	3.4	2.7	0.1819	2.4
4/18/2018 20:22	4	24.8	3.4	2.5	0.1633	2.4
4/18/2018 20:23	5.4	24.8	3.4	2.4	0.1828	2.4
4/18/2018 20:24	5.6	23.4	3.4	2.4	0.1742	2.5
4/18/2018 20:25	3.7	21	3.3	2.4	0.1919	2.5
4/18/2018 20:26	4.9	21.7	3.3	2.3	0.1796	2.5
4/18/2018 20:27	5.1	21.3	3.3	2.3	0.1867	2.5
4/18/2018 20:28	3.3	21	3.3	2.3	0.194	2.4
4/18/2018 20:29	3.4	20.8	3.2	2.4	0.1808	2.4
4/18/2018 20:30	5.3	20.9	3.2	2.5	0.1896	2.4
4/18/2018 20:31	3.1	21.1	3.2	2.6	0.1827	2.4
4/18/2018 20:32	4	21.3	3.2	2.7	0.1915	2.4
4/18/2018 20:33	4.2	21.4	3.3	2.7	0.1798	2.3
4/18/2018 20:34	5.1	21.6	3.3	2.7	0.184	2.4
4/18/2018 20:35	3.5	21.6	3.3	2.7	0.1792	2.4
4/18/2018 20:36	3.9	22.1	3.3	2.7	0.1786	2.3
4/18/2018 20:37	5.3	22.5	3.3	2.7	0.1872	2.1
4/18/2018 20:38	4.8	24.5	3.4	2.5	0.1666	2
4/18/2018 20:39	5.3	25.4	3.5	2.4	0.1763	2
4/18/2018 20:40	7.4	25.3	3.6	2.3	0.1683	2
4/18/2018 20:41	4.8	22.6	3.4	2.3	0.1764	2
4/18/2018 20:42	5	21.1	3.2	2.3	0.1843	2.1
4/18/2018 20:43	4.5	21.8	3.4	2.3	0.1802	2
4/18/2018 20:44	3.3	20.9	3.3	2.3	0.1906	1.7
4/18/2018 20:45	4.7	20.9	3.2	2.3	0.1729	1.6
4/18/2018 20:46	5.1	20.8	3.2	2.4	0.1867	1.7
4/18/2018 20:47	2.3	20.7	3.2	2.5	0.1804	1.7
4/18/2018 20:48	4.3	20.9	3.2	2.6	0.1876	1.8
4/18/2018 20:49	4.4	20.9	3.3	2.7	0.1802	1.8
4/18/2018 20:50	3.7	21.3	3.3	2.7	0.1767	1.8
4/18/2018 20:51	4.7	21.6	3.4	2.7	0.1853	1.8
4/18/2018 20:52	3.7	21.9	3.4	2.7	0.1676	1.8
4/18/2018 20:53	5.4	21.9	3.5	2.7	0.1793	1.7

4/18/2018 20:54	5.8	22.2	3.5	2.6	0.1829	1.7
4/18/2018 20:55	4.4	22	3.4	2.6	0.1767	1.7
4/18/2018 20:56	6	21.9	3.5	2.5	0.1806	1.7
4/18/2018 20:57	5	24.5	3.6	2.4	0.1713	1.7
4/18/2018 20:58	5.2	24.4	3.7	2.3	0.1724	1.7
4/18/2018 20:59	4.3	22.5	3.5	2.2	0.1672	1.8
4/18/2018 21:00	5.6	21.5	3.3	2.3	0.1872	1.8
4/18/2018 21:01	3.2	21.2	3.4	2.3	0.1816	1.8
4/18/2018 21:02	4	20.9	3.3	2.3	0.1719	1.8
4/18/2018 21:03	3.2	20.8	3.2	2.4	0.1871	1.8
4/18/2018 21:04	2.5	20.7	3.2	2.4	0.1699	1.9
4/18/2018 21:05	3.6	20.6	3.2	2.5	0.1873	1.9
4/18/2018 21:06	3.9	20.5	3.2	2.5	0.1768	1.9
4/18/2018 21:07	4.2	21.2	3.3	2.6	0.1803	2
4/18/2018 21:08	5.1	21.4	3.3	2.6	0.1731	2
4/18/2018 21:09	4.4	21.8	3.3	2.6	0.168	2
4/18/2018 21:10	4.7	21.8	3.3	2.6	0.1806	2.1
4/18/2018 21:11	4.4	21.5	3.3	2.5	0.1682	2.1
4/18/2018 21:12	4.8	21.7	3.3	2.5	0.1813	2
4/18/2018 21:13	2.8	21.7	3.2	2.4	0.1689	2
4/18/2018 21:14	4.5	20.8	3.3	2.4	0.1797	2
4/18/2018 21:15	4.7	21.1	3.2	2.4	0.1801	2.1
4/18/2018 21:16	5.1	20.7	3.3	2.4	0.1744	2.2
4/18/2018 21:17	3.9	20.7	3.2	2.5	0.182	2.2
4/18/2018 21:18	4.3	20.8	3.2	2.5	0.164	2.1
4/18/2018 21:19	2.9	20.6	3.2	2.5	0.1794	2.1
4/18/2018 21:20	2.7	20.5	3.2	2.5	0.1606	2.2
4/18/2018 21:21	4.3	20.5	3.2	2.6	0.1753	2.2
4/18/2018 21:22	2.8	20.6	3.2	2.6	0.1828	2.2
4/18/2018 21:23	3.5	20.6	3.2	2.6	0.166	2.1
4/18/2018 21:24	5.5	20.8	3.2	2.6	0.1797	2.1
4/18/2018 21:25	5.5	21.3	3.2	2.6	0.1645	2.1
4/18/2018 21:26	3.9	21.4	3.2	2.6	0.1729	2.1
4/18/2018 21:27	4.2	21	3.1	2.6	0.1682	2.2
4/18/2018 21:28	4.3	21.3	3.1	2.5	0.1706	2.1
4/18/2018 21:29	4.8	21.2	3.1	2.5	0.1782	2.1
4/18/2018 21:30	5	21.2	3.1	2.5	0.1652	2
4/18/2018 21:31	4.6	21	3.1	2.5	0.1794	1.6
4/18/2018 21:32	3.5	20.7	3.1	2.5	0.1702	1.7
4/18/2018 21:33	5.1	21.2	3.1	2.5	0.1774	1.9
4/18/2018 21:34	4.1	21.4	3.1	2.5	0.171	2.1
4/18/2018 21:35	3.5	20.7	3.1	2.5	0.1725	2.1
4/18/2018 21:36	4.4	20.1	3.1	2.5	0.1761	2
4/18/2018 21:37	2.5	20.5	3.1	2.5	0.1663	1.9
4/18/2018 21:38	3.4	21	3.1	2.6	0.1815	2
4/18/2018 21:39	4.8	20.5	3.1	2.6	0.1682	2.1
4/18/2018 21:40	2.9	20.6	3	2.6	0.1715	2.1

4/18/2018 21:41	3.2	20.5	3.1	2.6	0.1708	2.2
4/18/2018 21:42	3.2	20.8	3.1	2.6	0.1691	2.2
4/18/2018 21:43	5	21.1	3.1	2.6	0.1768	2.3
4/18/2018 21:44	4.3	21.7	3.1	2.6	0.1583	2.3
4/18/2018 21:45	5.2	21.4	3.1	2.6	0.1773	2.3
4/18/2018 21:46	5.2	21.4	3.1	2.5	0.1665	1.9
4/18/2018 21:47	3.8	21.4	3.2	2.5	0.1684	1.5
4/18/2018 21:48	4	21.3	3.2	2.4	0.1796	1.6
4/18/2018 21:49	4.9	21.4	3.2	2.4	0.1595	1.8
4/18/2018 21:50	4.7	21.2	3.2	2.4	0.1789	2
4/18/2018 21:51	2.8	21.2	3.3	2.4	0.1614	1.4
4/18/2018 21:52	4	20.8	3.2	2.4	0.1751	1.4
4/18/2018 21:53	4.3	20.6	3.2	2.4	0.165	1.4
4/18/2018 21:54	4.3	20.6	3.1	2.4	0.1737	1.5
4/18/2018 21:55	3.5	20.6	3.1	2.4	0.166	1.7
4/18/2018 21:56	4.2	20.9	3.2	2.5	0.1652	1.6
4/18/2018 21:57	3.9	21.1	3.3	2.5	0.1768	1.6
4/18/2018 21:58	4.8	21.2	3.5	2.5	0.1597	1.7
4/18/2018 21:59	3.2	21.1	3.4	2.5	0.1671	1.6
4/18/2018 22:00	3.9	20.9	3.4	2.4	0.1685	1.6
4/18/2018 22:01	4.8	21.1	3.3	2.4	0.1683	1.6
4/18/2018 22:02	4.4	21.5	3.4	2.4	0.1679	1.5
4/18/2018 22:03	4.9	21.5	3.4	2.4	0.1608	1.6
4/18/2018 22:04	4.2	21.6	3.5	2.3	0.1694	1.6
4/18/2018 22:05	5	21.1	3.4	2.3	0.1665	1.6
4/18/2018 22:06	4	21.2	3.3	2.3	0.1705	1.6
4/18/2018 22:07	3.8	21.1	3.3	2.3	0.1671	1.6
4/18/2018 22:08	3.7	20.5	3.2	2.3	0.1683	1.5
4/18/2018 22:09	4	20.8	3.2	2.4	0.1723	1.5
4/18/2018 22:10	4.3	20.6	3.2	2.4	0.1604	1.6
4/18/2018 22:11	3.9	20.6	3.2	2.4	0.171	1.5
4/18/2018 22:12	4.6	21	3.2	2.4	0.159	1.5
4/18/2018 22:13	3.9	20.8	3.1	2.5	0.1752	1.5
4/18/2018 22:14	2.8	20.3	3.1	2.5	0.1715	1.7
4/18/2018 22:15	3.3	20.6	3.1	2.5	0.1595	1.8
4/18/2018 22:16	4.6	20.6	3.2	2.5	0.1714	1.9
4/18/2018 22:17	5.4	21	3.2	2.5	0.1624	2.1
4/18/2018 22:18	4.4	20.9	3.2	2.5	0.1684	2.2
4/18/2018 22:19	3.8	21.2	3.2	2.5	0.1633	2.3
4/18/2018 22:20	3.4	21	3.2	2.5	0.1753	2.4
4/18/2018 22:21	4.8	21	3.2	2.4	0.1718	2.4
4/18/2018 22:22	3.2	20.7	3.1	2.4	0.1647	2.4
4/18/2018 22:23	4.1	21.2	3.1	2.4	0.1716	2.5
4/18/2018 22:24	5	20.6	3.1	2.4	0.1548	2.5
4/18/2018 22:25	3.8	20.6	3.1	2.4	0.1735	2.5
4/18/2018 22:26	2.2	20.5	3.1	2.5	0.1695	2.6
4/18/2018 22:27	3.4	20.2	3.1	2.5	0.1649	2.6

4/18/2018 22:28	4	20.3	3.1	2.5	0.174	2.5
4/18/2018 22:29	4.9	20.5	3.1	2.5	0.1596	2.5
4/18/2018 22:30	4.1	20.5	3.1	2.5	0.1762	2.5
4/18/2018 22:31	5.3	21	3.1	2.5	0.1607	2.6
4/18/2018 22:32	4.8	20.7	3.1	2.5	0.1666	2.5
4/18/2018 22:33	5	20.9	3.1	2.5	0.1714	2.6
4/18/2018 22:34	2.6	20.5	3.1	2.5	0.1592	2.6
4/18/2018 22:35	4	20.4	3.1	2.5	0.1745	2.7
4/18/2018 22:36	4	20.8	3.1	2.5	0.1565	2.7
4/18/2018 22:37	3.6	20.3	3.1	2.5	0.1685	2.7
4/18/2018 22:38	5.5	20.5	3.1	2.5	0.1681	2.7
4/18/2018 22:39	2.8	20.4	3.2	2.5	0.1658	2.6
4/18/2018 22:40	3.1	20	3.1	2.5	0.1689	2.6
4/18/2018 22:41	3.3	20.3	3.2	2.5	0.1554	2.6
4/18/2018 22:42	4.9	20	3.1	2.6	0.1757	2.6
4/18/2018 22:43	3.4	20	3.2	2.6	0.1631	2.7
4/18/2018 22:44	3.6	20.1	3.2	2.6	0.174	2.7
4/18/2018 22:45	4	20.2	3.3	2.6	0.169	2.7
4/18/2018 22:46	2.9	20.2	3.3	2.6	0.163	2.7
4/18/2018 22:47	4.3	20	3.4	2.6	0.1743	2.7
4/18/2018 22:48	4.1	20.4	3.5	2.6	0.1649	2.7
4/18/2018 22:49		20.1	3.4	2.6	0.1757	2.7
4/18/2018 22:50		20.3	3.4	2.6	0.1676	2.7
4/18/2018 22:51	3.9	20.2	3.6	2.6	0.1723	2.7
4/18/2018 22:52	3.3	20.4	3.6	2.5	0.1787	2.7
4/18/2018 22:53		20.5	3.7	2.5	0.1572	2.7
4/18/2018 22:54	8.2	20.2	3.6	2.5	0.1707	2.6
4/18/2018 22:55		20.1	3.6	2.5	0.1599	2.6
4/18/2018 22:56	4.4	20	3.5	2.5	0.1701	2.6
4/18/2018 22:57	10	20	3.4	2.5	0.1696	2.7
4/18/2018 22:58		19.7	3.3	2.5	0.1586	2.7
4/18/2018 22:59	4.3	19.8	3.3	2.5	0.175	2.7
4/18/2018 23:00	6.4	19.7	3.3	2.6	0.1633	2.7
4/18/2018 23:01	4.8	19.9	3.4	2.6	0.1757	2.7
4/18/2018 23:02	10.8	20.1	3.5	2.6	0.1675	2.7
4/18/2018 23:03	3.6	20	3.4	2.6	0.1671	2.7
4/18/2018 23:04	3.9	20	3.4	2.6	0.1643	2.8
4/18/2018 23:05	4.3	20	3.4	2.6	0.165	2.8
4/18/2018 23:06	4.4	20.1	3.4	2.6	0.1658	2.7
4/18/2018 23:07	3.5	19.8	3.4	2.6	0.1607	2.7
4/18/2018 23:08	4	19.9	3.5	2.6	0.1737	2.8
4/18/2018 23:09	3.1	20	3.5	2.6	0.1593	2.8
4/18/2018 23:10	5	20.1	3.5	2.6	0.1652	2.8
4/18/2018 23:11	3.6	19.7	3.5	2.6	0.1695	2.7
4/18/2018 23:12	4.1	19.7	3.5	2.6	0.163	2.5
4/18/2018 23:13	3	20	3.4	2.6	0.1687	2.5
4/18/2018 23:14	2.9	19.8	3.5	2.6	0.1579	2.5

4/18/2018 23:15	3.5	20.2	3.4	2.6	0.1687	2.7
4/18/2018 23:16	4.7	19.9	3.4	2.6	0.168	2.6
4/18/2018 23:17	3.9	20	3.4	2.6	0.1567	2.6
4/18/2018 23:18	3.9	20.1	3.5	2.6	0.1729	2.7
4/18/2018 23:19	2.2	20.1	3.7	2.6	0.1614	2.6
4/18/2018 23:20	4.8	20	3.6	2.6	0.1703	2.5
4/18/2018 23:21	3.3	20.2	3.6	2.5	0.1629	2.7
4/18/2018 23:22	3.3	20	3.6	2.5	0.1699	2.7
4/18/2018 23:23	4.1	19.8	3.6	2.5	0.1702	2.9
4/18/2018 23:24	3.3	20.3	3.5	2.5	0.1645	2.9
4/18/2018 23:25	4.8	20.3	3.6	2.5	0.1708	2.8
4/18/2018 23:26	4.2	20.2	3.5	2.5	0.1569	2.7
4/18/2018 23:27	4	20.1	3.5	2.5	0.1667	2.6
4/18/2018 23:28	4.6	20.1	3.5	2.5	0.1562	2.3
4/18/2018 23:29	4.9	20	3.5	2.5	0.1734	2.3
4/18/2018 23:30	2	20.3	3.5	2.5	0.1701	2.4
4/18/2018 23:31	4.8	20.7	3.5	2.5	0.1531	2.4
4/18/2018 23:32	3.2	19.8	3.5	2.5	0.1808	2.4
4/18/2018 23:33	4.8	20.5	3.5	2.5	0.1506	2.5
4/18/2018 23:34	3.7	20	3.4	2.5	0.1622	2.6
4/18/2018 23:35	2.8	20.1	3.5	2.5	0.1564	2.7
4/18/2018 23:36	5.9	20.2	3.4	2.5	0.1684	2.6
4/18/2018 23:37	4.7	20.4	3.4	2.5	0.1624	2.6
4/18/2018 23:38	4.1	20	3.4	2.5	0.1575	2.5
4/18/2018 23:39	3.3	20.3	3.4	2.5	0.1712	2.6
4/18/2018 23:40	3.6	20.2	3.4	2.5	0.1578	2.8
4/18/2018 23:41	3.7	20.1	3.3	2.5	0.1689	2.9
4/18/2018 23:42	2.2	20.5	3.4	2.5	0.1659	2.9
4/18/2018 23:43	3.2	20	3.4	2.5	0.1609	2.9
4/18/2018 23:44	4.4	19.9	3.3	2.5	0.1733	2.8
4/18/2018 23:45	4.5	19.9	3.3	2.5	0.1606	2.8
4/18/2018 23:46	3.9	20.1	3.3	2.5	0.1768	2.8
4/18/2018 23:47	2.7	20.1	3.3	2.5	0.1614	2.8
4/18/2018 23:48	4.3	20.2	3.3	2.5	0.1595	2.8
4/18/2018 23:49	3.8	20.3	3.3	2.5	0.1686	2.9
4/18/2018 23:50	2.7	20	3.3	2.5	0.155	2.9
4/18/2018 23:51	2	20	3.3	2.5	0.1686	2.9
4/18/2018 23:52	2	20.5	3.3	2.5	0.1623	2.8
4/18/2018 23:53	4.6	20.4	3.3	2.5	0.1673	2.8
4/18/2018 23:54	3	20	3.3	2.5	0.1688	2.9
4/18/2018 23:55	5	20.1	3.3	2.5	0.1601	2.9
4/18/2018 23:56	4.1	20.2	3.3	2.5	0.1764	2.8
4/18/2018 23:57	4.8	20.1	3.3	2.5	0.1551	2.8
4/18/2018 23:58	3.5	20.1	3.3	2.5	0.1692	2.8
4/18/2018 23:59	4.3	20.2	3.3	2.5	0.1723	2.8
4/19/2018 0:00	3.4	20.3	3.3	2.5	0.1613	2.8
4/19/2018 0:01	3.5	20.2	3.3	2.5	0.1663	2.8

4/19/2018 0:02	3.9	20	3.3	2.5	0.1529	2.8
4/19/2018 0:03	4	19.8	3.2	2.5	0.17	2.8
4/19/2018 0:04	4.3	19.8	3.2	2.5	0.1584	2.9
4/19/2018 0:05	3.6	20.1	3.2	2.5	0.1664	2.9
4/19/2018 0:06	3.6	19.9	3.2	2.5	0.1657	3
4/19/2018 0:07	3.8	20	3.2	2.5	0.1613	3
4/19/2018 0:08	3.6	19.8	3.2	2.5	0.1685	3
4/19/2018 0:09	3.6	19.6	3.2	2.6	0.157	2.9
4/19/2018 0:10	3.1	19.7	3.2	2.6	0.1722	2.9
4/19/2018 0:11	3.1	19.9	3.2	2.6	0.1575	2.9
4/19/2018 0:12	3.1	20	3.2	2.6	0.1611	2.8
4/19/2018 0:13	2.6	20	3.2	2.6	0.168	2.8
4/19/2018 0:14	5.7	19.8	3.2	2.6	0.1558	2.8
4/19/2018 0:15	3.4	19.7	3.2	2.6	0.162	2.8
4/19/2018 0:16	5	19.8	3.2	2.6	0.1477	2.9
4/19/2018 0:17	4	19.6	3.2	2.6	0.169	2.9
4/19/2018 0:18	3	19.5	3.2	2.6	0.1586	2.9
4/19/2018 0:19	4.2	19.6	3.3	2.6	0.1632	2.9
4/19/2018 0:20	3.5	19.8	3.3	2.6	0.1657	2.9
4/19/2018 0:21	3.4	19.5	3.3	2.6	0.1595	2.9
4/19/2018 0:22	3.6	19.7	3.4	2.6	0.1699	2.9
4/19/2018 0:23	3.9	19.5	3.3	2.6	0.156	2.8
4/19/2018 0:24	2.6	19.6	3.4	2.6	0.1736	2.9
4/19/2018 0:25	2.9	19.5	3.3	2.6	0.1631	2.8
4/19/2018 0:26	3.5	19.6	3.4	2.6	0.1704	2.7
4/19/2018 0:27	3.3	19.5	3.3	2.6	0.1733	2.8
4/19/2018 0:28	3.6	19.5	3.3	2.6	0.1602	2.8
4/19/2018 0:29	4.6	19.3	3.3	2.6	0.1801	2.8
4/19/2018 0:30	4.1	19.9	3.3	2.6	0.1523	2.7
4/19/2018 0:31	2.8	19.1	3.4	2.6	0.17	2.8
4/19/2018 0:32	5.1	19.6	3.4	2.6	0.1561	2.8
4/19/2018 0:33	3.1	19.6	3.4	2.6	0.1661	2.9
4/19/2018 0:34	3.6	19.2	3.4	2.6	0.1618	2.9
4/19/2018 0:35	4	19.5	3.4	2.6	0.1564	3
4/19/2018 0:36	3.5	19.2	3.4	2.6	0.1712	3
4/19/2018 0:37	2.7	19.3	3.4	2.6	0.1608	2.9
4/19/2018 0:38	2.8	19.4	3.4	2.6	0.1659	2.8
4/19/2018 0:39	3.6	19.5	3.4	2.7	0.1602	2.7
4/19/2018 0:40	2.4	19.4	3.4	2.7	0.1627	2.7
4/19/2018 0:41	2.8	19.1	3.5	2.7	0.1665	2.7
4/19/2018 0:42	3.6	19.3	3.5	2.7	0.1547	2.7
4/19/2018 0:43	2.7	19.4	3.5	2.7	0.1677	2.6
4/19/2018 0:44	2.3	19.2	3.5	2.7	0.1596	2.7
4/19/2018 0:45	2.6	19.4	3.5	2.7	0.1677	2.8
4/19/2018 0:46	4	19.1	3.5	2.7	0.1636	2.9
4/19/2018 0:47	4.3	19.2	3.5	2.7	0.1584	3
4/19/2018 0:48	4.6	18.9	3.5	2.7	0.1679	3

4/19/2018 0:49	4	19.5	3.7	2.7	0.1555	3
4/19/2018 0:50	3.9	19	3.7	2.7	0.1741	3
4/19/2018 0:51	2.7	19.7	3.9	2.7	0.1583	3
4/19/2018 0:52	5.6	19.8	4	2.6	0.1698	2.9
4/19/2018 0:53	4.8	20	4.1	2.6	0.173	3
4/19/2018 0:54	3.2	20	4.1	2.5	0.1616	2.9
4/19/2018 0:55	2.2	19.8	4.2	2.5	0.1734	2.8
4/19/2018 0:56	4.7	19.6	3.9	2.4	0.161	2.7
4/19/2018 0:57	4.1	19.8	3.9	2.4	0.157	2.7
4/19/2018 0:58	3.3	20.8	4.4	2.4	0.1629	2.6
4/19/2018 0:59	5.4	20.8	4.6	2.3	0.1597	2.6
4/19/2018 1:00	4.1	21.1	4.8	2.3	0.1728	2.6
4/19/2018 1:01	5.1	20.1	4.6	2.3	0.1586	2.6
4/19/2018 1:02	4.6	19.3	3.8	2.3	0.1702	2.6
4/19/2018 1:03	3.5	19.6	4	2.3	0.1607	2.7
4/19/2018 1:04	3	19.3	4	2.3	0.1621	2.7
4/19/2018 1:05	3.9	19	3.8	2.4	0.1692	2.7
4/19/2018 1:06	2.8	19	3.7	2.4	0.1598	2.7
4/19/2018 1:07	1.7	19	3.7	2.4	0.1736	2.7
4/19/2018 1:08	4.4	18.9	3.7	2.5	0.159	2.8
4/19/2018 1:09	3.1	19	3.8	2.6	0.1679	2.7
4/19/2018 1:10	4.5	19.2	3.8	2.6	0.1608	2.7
4/19/2018 1:11	4	19.3	3.8	2.6	0.1652	2.7
4/19/2018 1:12	3.8	19.3	3.9	2.7	0.1699	2.8
4/19/2018 1:13	1.8	19	3.9	2.7	0.1596	2.8
4/19/2018 1:14	4	19	3.9	2.7	0.1683	2.8
4/19/2018 1:15	2.8	18.9	3.9	2.6	0.1631	2.9
4/19/2018 1:16	3.6	19.3	4	2.6	0.167	2.9
4/19/2018 1:17	5	19.5	4.2	2.6	0.1549	2.9
4/19/2018 1:18	3	19.5	4.2	2.6	0.165	2.9
4/19/2018 1:19	3.9	18.9	4.2	2.6	0.172	2.9
4/19/2018 1:20	4.2	18.9	4.2	2.6	0.1562	2.9
4/19/2018 1:21	4.2	20.2	4.6	2.6	0.1714	2.8
4/19/2018 1:22	3.8	20.2	5	2.5	0.16	2.9
4/19/2018 1:23	5	21	5.3	2.5	0.1705	2.9
4/19/2018 1:24	3.3	19.6	5	2.4	0.1689	2.8
4/19/2018 1:25	3.6	19.2	4.3	2.4	0.1599	2.9
4/19/2018 1:26	3.1	19	4.3	2.4	0.1702	2.8
4/19/2018 1:27	3.5	18.9	4.3	2.4	0.1562	2.9
4/19/2018 1:28	3.9	18.8	4.1	2.4	0.1753	2.9
4/19/2018 1:29	3.1	18.9	4.1	2.4	0.1608	2.9
4/19/2018 1:30	2.5	18.9	4.1	2.4	0.1677	3
4/19/2018 1:31	4.5	18.9	4.1	2.5	0.1679	2.9
4/19/2018 1:32	4.3	18.7	4	2.6	0.1559	2.9
4/19/2018 1:33	3.9	19	4.1	2.6	0.1708	3
4/19/2018 1:34	3.6	18.6	4.1	2.6	0.1594	2.9
4/19/2018 1:35	2.8	19.1	4.1	2.7	0.1693	2.9

4/19/2018 1:36	3.5	18.9	4.1	2.7	0.1673	2.9
4/19/2018 1:37	5.2	18.6	4.1	2.7	0.1592	2.8
4/19/2018 1:38	2	18.7	4.2	2.7	0.1653	2.9
4/19/2018 1:39	2	18.7	4.3	2.7	0.1595	2.9
4/19/2018 1:40	2.6	18.9	4.4	2.7	0.1687	2.9
4/19/2018 1:41		18.8	4.4	2.7	0.1609	2.9
4/19/2018 1:42		18.8	4.6	2.7	0.1716	2.9
4/19/2018 1:43	5.1	18.6	4.4	2.7	0.1614	3
4/19/2018 1:44		18.7	4.4	2.7	0.1673	3
4/19/2018 1:45	4.6	18.7	4.6	2.7	0.1662	2.9
4/19/2018 1:46		18.6	4.7	2.7	0.1583	2.8
4/19/2018 1:47	4.3	19	4.9	2.7	0.169	2.8
4/19/2018 1:48	3.7	18.5	5.1	2.7	0.1604	2.8
4/19/2018 1:49		18.7	5.3	2.7	0.1689	2.8
4/19/2018 1:50	5.2	18.9	5.1	2.7	0.1667	2.8
4/19/2018 1:51		18.9	5.6	2.6	0.1668	2.8
4/19/2018 1:52	3.9	19.6	5.7	2.6	0.1783	2.7
4/19/2018 1:53	3.3	19.2	6.4	2.6	0.1545	2.7
4/19/2018 1:54	4.3	19.3	6.3	2.5	0.1756	2.8
4/19/2018 1:55	3.6	18.7	4.8	2.5	0.1593	2.9
4/19/2018 1:56	4.3	18.7	5	2.5	0.1665	2.9
4/19/2018 1:57	2.2	18.3	5.2	2.5	0.1684	3
4/19/2018 1:58	4.3	18.4	4.9	2.6	0.1614	3.1
4/19/2018 1:59	3.3	18.8	4.8	2.6	0.1694	3.2
4/19/2018 2:00	3.5	18.6	4.8	2.6	0.1574	3.2
4/19/2018 2:01	3.5	18.4	4.7	2.7	0.1724	3.1
4/19/2018 2:02	2.2	18.5	4.7	2.7	0.162	3
4/19/2018 2:03	2.9	18.5	4.7	2.8	0.161	2.9
4/19/2018 2:04	2.5	18.5	4.8	2.8	0.171	2.9
4/19/2018 2:05	3.9	18.3	4.8	2.8	0.1549	3
4/19/2018 2:06	2.9	18.5	4.9	2.8	0.1697	2.8
4/19/2018 2:07	2.8	18.4	4.8	2.8	0.167	2.9
4/19/2018 2:08	2.9	18.4	4.9	2.8	0.1636	3
4/19/2018 2:09	2.8	18.5	5	2.8	0.1756	3
4/19/2018 2:10	4.2	18.3	5.3	2.8	0.1618	3
4/19/2018 2:11	3.2	18.3	5.2	2.8	0.1782	2.8
4/19/2018 2:12	3.4	18.4	5.2	2.8	0.162	2.3
4/19/2018 2:13	5.3	18.2	5.1	2.8	0.1642	1.7
4/19/2018 2:14	2.5	18.2	5.4	2.8	0.1627	1.9
4/19/2018 2:15	2.5	18.1	5.6	2.9	0.1739	2.1
4/19/2018 2:16	2.3	18.1	5.6	2.8	0.1821	2.3
4/19/2018 2:17	3.4	18.3	5.9	2.8	0.1659	2.3
4/19/2018 2:18	3.7	18.2	6.4	2.8	0.1769	2.2
4/19/2018 2:19	2.6	18.2	6.5	2.8	0.1693	2.2
4/19/2018 2:20	2.2	18.2	6.5	2.8	0.175	2
4/19/2018 2:21	3.5	18.2	7.3	2.8	0.1644	2
4/19/2018 2:22	2.3	18.1	7.5	2.8	0.1638	2.2

4/19/2018 2:23	3.2	18.2	6.3	2.8	0.1744	2.3
4/19/2018 2:24	4	18	6.5	2.8	0.1585	2.3
4/19/2018 2:25	3.2	18	6.9	2.8	0.1711	2.4
4/19/2018 2:26	2.7	17.8	6.7	2.8	0.1712	2.5
4/19/2018 2:27	3.4	18.1	6.1	2.8	0.1571	2.5
4/19/2018 2:28	3	17.9	5.9	2.8	0.1729	2.6
4/19/2018 2:29	2.8	18.1	6	2.8	0.1594	2.6
4/19/2018 2:30	3.8	17.9	5.9	2.9	0.1758	2.6
4/19/2018 2:31	2.4	18.2	5.9	2.9	0.1617	2.5
4/19/2018 2:32	2.4	18	5.8	2.9	0.1753	2.5
4/19/2018 2:33	3.3	17.8	5.9	2.9	0.1768	2.4
4/19/2018 2:34	1.5	18.1	5.9	2.9	0.1601	2.4
4/19/2018 2:35	2.8	18	5.9	2.9	0.1786	2.4
4/19/2018 2:36	3.5	18	5.9	2.9	0.1657	2.4
4/19/2018 2:37	3.7	17.8	5.9	2.9	0.1781	2.4
4/19/2018 2:38	2.4	18	6	2.9	0.1715	2.5
4/19/2018 2:39	2.5	18.1	6.1	2.9	0.1687	2.4
4/19/2018 2:40	3.9	18.1	6	2.9	0.177	2.4
4/19/2018 2:41	3	17.8	5.9	3	0.161	2.5
4/19/2018 2:42	3.1	17.9	6	2.9	0.1817	2.5
4/19/2018 2:43	2.3	18.1	5.9	3	0.1615	2.4
4/19/2018 2:44	3	18.1	6	3	0.1781	2.4
4/19/2018 2:45	2.8	18	6	3	0.1667	2.5
4/19/2018 2:46	2.6	18.1	6	3	0.1713	2.6
4/19/2018 2:47	4	18.1	6	3	0.1767	2.5
4/19/2018 2:48	3.5	18.1	6	3	0.165	2.4
4/19/2018 2:49	2.8	17.9	5.9	3	0.1769	2.1
4/19/2018 2:50	3.6	17.9	5.9	3	0.1628	1.3
4/19/2018 2:51	3.5	18	5.9	3	0.1836	1.3
4/19/2018 2:52	3.7	18.3	6	3	0.1792	1.3
4/19/2018 2:53	3.3	18.3	6	3	0.1692	1.3
4/19/2018 2:54	2.5	18.1	5.9	3	0.1793	1.3
4/19/2018 2:55	3.5	18.2	6	3	0.1702	1.3
4/19/2018 2:56	3	18.2	5.9	3	0.1805	1.4
4/19/2018 2:57	4.3	18	5.9	3	0.1635	1.4
4/19/2018 2:58	3.5	18.4	6	3	0.1753	1.5
4/19/2018 2:59	2.9	18.2	5.9	3	0.1706	1.6
4/19/2018 3:00	2.8	18.1	5.9	3	0.1656	1.6
4/19/2018 3:01	2.7	18.1	5.9	3	0.1736	1.7
4/19/2018 3:02	2.3	18.6	5.8	3	0.1615	1.8
4/19/2018 3:03	2.4	18.6	5.9	2.9	0.1765	1.9
4/19/2018 3:04	5.1	18.7	5.9	2.9	0.1636	1.9
4/19/2018 3:05	2.2	18.9	5.9	2.8	0.1725	1.9
4/19/2018 3:06	3.4	18.8	5.9	2.8	0.1732	1.9
4/19/2018 3:07	3.8	18.7	5.7	2.8	0.1631	1.9
4/19/2018 3:08	3.3	18.5	5.8	2.8	0.1738	1.9
4/19/2018 3:09	4.3	19.2	5.8	2.7	0.1658	2

4/19/2018 3:10	3	18.6	5.8	2.7	0.1755	2
4/19/2018 3:11	2.8	18.4	5.6	2.7	0.1725	2.1
4/19/2018 3:12	4.3	18.5	5.6	2.7	0.1682	2.1
4/19/2018 3:13	3.1	18.5	5.7	2.7	0.175	2.2
4/19/2018 3:14	2.8	18.4	5.6	2.7	0.1604	2.2
4/19/2018 3:15	3.2	18.1	5.5	2.8	0.1774	2.2
4/19/2018 3:16	3	18.3	5.5	2.8	0.1741	2.2
4/19/2018 3:17	2.8	18.2	5.5	2.8	0.1699	2.3
4/19/2018 3:18	2	18.3	5.5	2.9	0.1793	2.3
4/19/2018 3:19	3.2	18.3	5.5	2.9	0.1555	2.4
4/19/2018 3:20	2.7	18	5.5	2.9	0.1738	2.4
4/19/2018 3:21	3.4	18.4	5.5	3	0.1607	2.5
4/19/2018 3:22	2.7	18.4	5.5	3	0.1716	2.5
4/19/2018 3:23	3.2	18.4	5.5	2.9	0.1711	2.5
4/19/2018 3:24	3.9	18.2	5.5	3	0.1726	2.2
4/19/2018 3:25	4.4	18.2	5.5	3	0.1706	2.3
4/19/2018 3:26	4.6	18.1	5.5	3	0.164	2.4
4/19/2018 3:27	3	18.2	5.5	3	0.1773	2.4
4/19/2018 3:28	3.1	18	5.6	3	0.1612	2.5
4/19/2018 3:29	2.4	18.2	5.5	3	0.1678	2.6
4/19/2018 3:30	3.9	18.1	5.6	3	0.1695	2.6
4/19/2018 3:31	2.4	18.3	5.9	3	0.171	2.5
4/19/2018 3:32	4.2	18	5.7	2.9	0.1657	2.5
4/19/2018 3:33	3.5	18.7	5.8	2.9	0.159	2.5
4/19/2018 3:34	4.5	18.3	6	2.9	0.172	2.6
4/19/2018 3:35	2.3	18.5	6.1	2.9	0.1598	2.6
4/19/2018 3:36	2.7	18.3	6.4	2.8	0.1654	2.5
4/19/2018 3:37	2.3	18.3	6.3	2.8	0.1682	2.5
4/19/2018 3:38	3.4	18.3	6.4	2.7	0.1671	2.5
4/19/2018 3:39	4.2	18.2	6.2	2.7	0.1704	2.5
4/19/2018 3:40	3.5	18.1	6.1	2.7	0.163	2.5
4/19/2018 3:41	3.2	18.2	6.2	2.7	0.1682	2.5
4/19/2018 3:42	2.6	18.1	6.2	2.6	0.1718	2.5
4/19/2018 3:43	3	18.2	6.2	2.6	0.1567	2.5
4/19/2018 3:44	2.3	18.1	6.1	2.7	0.1715	2.5
4/19/2018 3:45	1.4	18.1	5.7	2.7	0.1623	2.5
4/19/2018 3:46	2.7	17.8	5.6	2.8	0.1689	2.5
4/19/2018 3:47	2.6	18	5.6	2.8	0.168	2.5
4/19/2018 3:48	4.3	18	5.7	2.9	0.1662	2.5
4/19/2018 3:49	3.7	18.2	5.7	2.9	0.1757	2.6
4/19/2018 3:50	3.2	18	5.7	2.9	0.1582	2.6
4/19/2018 3:51	3.8	18	5.7	3	0.1733	2.5
4/19/2018 3:52	3.8	18.1	5.8	3	0.1605	2.5
4/19/2018 3:53	2.5	17.9	5.8	3	0.1628	2.5
4/19/2018 3:54	5.7	18.1	5.7	3	0.1654	2.5
4/19/2018 3:55	2.3	17.8	5.7	3	0.156	2.6
4/19/2018 3:56	4	18.1	5.7	2.9	0.1702	2.6

4/19/2018 3:57	2.4	18.1	5.8	3	0.1601	2.7
4/19/2018 3:58	2	18	5.9	2.9	0.1647	2.7
4/19/2018 3:59	3.3	17.9	5.9	2.9	0.1671	2.8
4/19/2018 4:00	3.4	18.2	6.1	2.9	0.161	2.8
4/19/2018 4:01	3.4	18	6.2	2.9	0.1719	2.8
4/19/2018 4:02	2.8	17.8	6	2.9	0.1622	2.8
4/19/2018 4:03	4.9	18	6.2	2.9	0.1649	2.7
4/19/2018 4:04	3.6	18.2	6.5	2.9	0.1574	2.8
4/19/2018 4:05	3.7	18.3	6.5	2.8	0.1606	2.7
4/19/2018 4:06	3.4	18.1	6.5	2.8	0.1641	1.9
4/19/2018 4:07	3.3	18.4	6.9	2.8	0.1581	1.9
4/19/2018 4:08	4.1	18.4	7.6	2.7	0.1669	2.2
4/19/2018 4:09	3.8	18	6.9	2.7	0.1534	2.4
4/19/2018 4:10	3.4	17.8	6	2.7	0.1685	2.5
4/19/2018 4:11	4.2	17.8	6.4	2.7	0.157	2.5
4/19/2018 4:12	1.8	17.9	6.2	2.7	0.1657	2.4
4/19/2018 4:13	4.2	17.8	6	2.8	0.1664	1.6
4/19/2018 4:14	2.2	17.9	6	2.8	0.1572	1.6
4/19/2018 4:15	2.7	17.7	6	2.8	0.1684	1.9
4/19/2018 4:16	3.7	17.6	6.2	2.9	0.1617	2.2
4/19/2018 4:17	3.7	17.6	6	2.9	0.1649	2.2
4/19/2018 4:18	4.2	17.7	6.1	2.9	0.1694	2.1
4/19/2018 4:19	2.5	17.9	6.1	2.9	0.1602	1.4
4/19/2018 4:20	2.5	17.7	6.1	2.9	0.1771	1.5
4/19/2018 4:21	2.6	18	6	2.9	0.1586	1.8
4/19/2018 4:22	3.5	18.1	6	2.9	0.1666	1.9
4/19/2018 4:23	2.2	17.8	6.1	2.9	0.1659	2.2
4/19/2018 4:24	2.2	17.8	6.2	3	0.162	2.3
4/19/2018 4:25	5.2	18	6.2	3	0.1704	2.2
4/19/2018 4:26	2.1	17.7	6.3	3	0.1618	2.1
4/19/2018 4:27	3.4	17.8	6.5	3	0.1691	2.3
4/19/2018 4:28	3	17.8	6.5	3	0.1596	2.4
4/19/2018 4:29	3.5	17.6	6.4	3	0.1722	2.3
4/19/2018 4:30	3.4	18	6.4	3	0.1701	2.4
4/19/2018 4:31	0.6	18.1	6.5	2.9	0.1662	2.4
4/19/2018 4:32	3.6	18.1	6.6	2.9	0.1645	2.5
4/19/2018 4:33	3.5	18	6.6	2.9	0.1592	2.4
4/19/2018 4:34	2.4	18.4	6.6	2.8	0.1731	2.5
4/19/2018 4:35	3.3	18.2	6.5	2.8	0.1594	2.5
4/19/2018 4:36	4.3	17.9	6.5	2.8	0.1673	2.6
4/19/2018 4:37	4.4	17.9	6.4	2.8	0.1755	2.6
4/19/2018 4:38	2.1	18.3	6.3	2.7	0.1575	2.6
4/19/2018 4:39	3.2	18.2	6.3	2.7	0.1723	2.7
4/19/2018 4:40	1.3	18	6.2	2.7	0.1663	2.7
4/19/2018 4:41	2.3	17.8	6	2.7	0.1631	2.6
4/19/2018 4:42	3.4	17.8	5.9	2.8	0.1612	2.6
4/19/2018 4:43	1.7	17.7	5.9	2.9	0.1556	2.7

4/19/2018 4:44	2.7	17.9	5.9	2.9	0.1759	2.7
4/19/2018 4:45	2.2	17.8	5.9	2.9	0.1527	2.7
4/19/2018 4:46	2.4	18	5.9	3	0.1747	2.7
4/19/2018 4:47	3	18	5.9	3	0.1612	2.7
4/19/2018 4:48	2.4	18	5.9	3	0.1557	2.7
4/19/2018 4:49	3.8	17.9	5.9	3	0.1693	2.7
4/19/2018 4:50	3.4	17.8	5.8	3	0.1577	2.8
4/19/2018 4:51	4.2	17.8	5.8	3	0.1701	2.8
4/19/2018 4:52	3.4	18	5.8	3	0.1629	2.9
4/19/2018 4:53	2	18.1	5.8	3	0.1557	2.6
4/19/2018 4:54	2.9	17.9	5.9	3	0.1686	2.6
4/19/2018 4:55	3.3	17.9	5.9	3	0.1643	2.7
4/19/2018 4:56	2.1	18.2	5.9	2.9	0.169	2.6
4/19/2018 4:57	2.4	18.2	5.9	2.9	0.156	2.5
4/19/2018 4:58		18.1	6	2.9	0.1664	2.5
4/19/2018 4:59	4.8	18.2	5.9	2.9	0.1641	2.6
4/19/2018 5:00		18.1	6	2.8	0.1542	2.7
4/19/2018 5:01	3.7	18.2	6	2.8	0.1668	2.7
4/19/2018 5:02		17.9	6.1	2.8	0.1507	2.5
4/19/2018 5:03	3.6	18	6.2	2.8	0.1636	2.4
4/19/2018 5:04	2.9	18.1	6.1	2.7	0.1564	2.5
4/19/2018 5:05		18.3	6.2	2.7	0.1561	2.6
4/19/2018 5:06	3.3	18	6.2	2.7	0.1699	2.6
4/19/2018 5:07		17.9	6.1	2.7	0.1527	2.6
4/19/2018 5:08	5	17.8	5.8	2.7	0.1686	2.4
4/19/2018 5:09	2.9	17.7	5.7	2.8	0.1585	2.1
4/19/2018 5:10	1.5	17.7	5.8	2.8	0.1649	2.1
4/19/2018 5:11		17.5	5.8	2.9	0.1683	2.4
4/19/2018 5:12		17.7	5.9	2.9	0.159	2.6
4/19/2018 5:13		17.7	5.8	3	0.1705	2.6
4/19/2018 5:14		17.6	5.9	3	0.155	2.8
4/19/2018 5:15	4.3	17.8	5.9	3.1	0.1664	2.8
4/19/2018 5:16	3.5	17.8	5.9	3.1	0.1722	2.9
4/19/2018 5:17	3.6	17.6	5.9	3.1	0.1517	2.8
4/19/2018 5:18	2.7	17.5	6	3.1	0.1673	2.7
4/19/2018 5:19	2.9	17.9	5.9	3.1	0.1561	2.7
4/19/2018 5:20	3.4	17.5	5.9	3.1	0.1731	2.7
4/19/2018 5:21	3.4	17.6	6.2	3.1	0.1645	2.8
4/19/2018 5:22	3	17.4	6.2	3.1	0.1614	2.8
4/19/2018 5:23	2.5	17.5	6.2	3.1	0.166	2.9
4/19/2018 5:24	2.4	17.6	6.3	3.1	0.1523	2.9
4/19/2018 5:25	2.6	17.4	6.7	3.1	0.1682	3
4/19/2018 5:26	2.3	17.5	6.7	3.1	0.1675	3
4/19/2018 5:27	1.8	17.3	6.8	3.1	0.1635	3
4/19/2018 5:28	1.4	17.7	6.8	3.1	0.1717	3
4/19/2018 5:29	2.7	17.4	7	3.1	0.1573	3
4/19/2018 5:30	2.6	17.2	6.6	3.2	0.1762	2.9

4/19/2018 5:31	4	17.4	6.9	3.2	0.1585	2.9
4/19/2018 5:32	2.1	17.4	6.9	3.2	0.1743	2.8
4/19/2018 5:33	2	17.4	6.9	3.2	0.1691	2.8
4/19/2018 5:34	2.4	17.3	6.6	3.2	0.1685	2.8
4/19/2018 5:35	3.1	17.4	7.2	3.2	0.1762	2.7
4/19/2018 5:36	2.9	17.4	7.2	3.2	0.1617	2.8
4/19/2018 5:37	1.7	17.7	7.3	3.2	0.1745	2.8
4/19/2018 5:38	2.8	17.3	7.5	3.2	0.1722	2.8
4/19/2018 5:39	3.1	17.4	7.5	3.2	0.1698	2.8
4/19/2018 5:40	2.7	17.3	7.5	3.3	0.1795	2.9
4/19/2018 5:41	1.8	17.2	7.8	3.3	0.1665	2.7
4/19/2018 5:42	2.8	17.4	7.6	3.3	0.1835	2.8
4/19/2018 5:43	1.7	16.9	8.1	3.3	0.1732	2.7
4/19/2018 5:44	3.1	17.3	8.1	3.3	0.1811	2.7
4/19/2018 5:45	4.3	17.5	8.2	3.3	0.1731	2.5
4/19/2018 5:46	1.4	17.4	7.9	3.3	0.1746	2.3
4/19/2018 5:47	2.1	17.3	7.7	3.3	0.1787	2.3
4/19/2018 5:48	3.4	17.1	7.1	3.3	0.1672	2.5
4/19/2018 5:49	3.5	17.4	6.8	3.3	0.1778	2.6
4/19/2018 5:50	2.8	17.5	6.9	3.4	0.1734	2.7
4/19/2018 5:51	2.1	17.5	7	3.4	0.1739	2.7
4/19/2018 5:52	1.2	17.4	6.8	3.4	0.1751	2.9
4/19/2018 5:53	1.7	17.3	7.1	3.5	0.1686	2.8
4/19/2018 5:54	3.3	17.5	7.2	3.5	0.1752	2.8
4/19/2018 5:55	1.8	17.2	7.2	3.5	0.1673	2.9
4/19/2018 5:56	2.9	17.2	7.2	3.6	0.1847	3.1
4/19/2018 5:57	2.1	17.2	7.1	3.6	0.1816	3.1
4/19/2018 5:58	2.1	17.3	7.3	3.6	0.1726	3.1
4/19/2018 5:59	2.6	17.2	7.5	3.6	0.1921	3.1
4/19/2018 6:00	1.5	17.3	7.5	3.6	0.1653	3.2
4/19/2018 6:01	2.2	17.4	7.6	3.6	0.181	3
4/19/2018 6:02	2.9	17.2	7.6	3.7	0.1791	2.9
4/19/2018 6:03	1.8	17	7.5	3.7	0.1721	3
4/19/2018 6:04	2	17.1	7.7	3.7	0.1792	3.1
4/19/2018 6:05	3.5	17.1	7.9	3.7	0.1618	3.2
4/19/2018 6:06	3.8	17.1	7.9	3.7	0.1845	3.2
4/19/2018 6:07	1.3	17.1	8.1	3.8	0.1744	3.2
4/19/2018 6:08	2.8	17.1	8	3.8	0.1716	3.2
4/19/2018 6:09	1.8	17.3	8.1	3.8	0.1777	3.2
4/19/2018 6:10	2.4	17	7.9	3.8	0.1713	3.3
4/19/2018 6:11	1.8	17.3	7.7	3.8	0.1914	3.3
4/19/2018 6:12	0.9	17.4	7.8	3.9	0.1773	3.3
4/19/2018 6:13	1.7	17.1	8	3.9	0.176	3.4
4/19/2018 6:14	2.6	16.9	8	3.9	0.1827	3.4
4/19/2018 6:15	2.8	17.1	8	3.9	0.1731	3.4
4/19/2018 6:16	3.6	17	8.3	4	0.1825	3.4
4/19/2018 6:17	3.3	17.2	8.1	4	0.1733	3.4

4/19/2018 6:18	3.2	17.3	8.4	4	0.1755	3.4
4/19/2018 6:19	3.2	17	8.1	4	0.1919	2.8
4/19/2018 6:20	2.1	17.1	8	4.1	0.1763	2.3
4/19/2018 6:21	2.3	17.3	8.1	4.1	0.1852	2.5
4/19/2018 6:22	1.9	17.2	8	4.1	0.1746	2.9
4/19/2018 6:23	2	17.4	8.2	4.1	0.1747	3
4/19/2018 6:24	3.2	17.1	8.2	4.1	0.1854	3
4/19/2018 6:25	3	17	8.2	4.1	0.177	3
4/19/2018 6:26	3	16.9	8.3	4.2	0.1846	3
4/19/2018 6:27	2.4	17.2	8.5	4.2	0.1779	2.9
4/19/2018 6:28	1.9	17.2	8.3	4.2	0.1855	3
4/19/2018 6:29	2.8	17.3	8	4.2	0.1831	3.1
4/19/2018 6:30	2.1	17.1	8	4.2	0.1769	3.1
4/19/2018 6:31	3	17.4	8.1	4.2	0.1869	3.1
4/19/2018 6:32	1.4	17.1	8.2	4.2	0.1753	2.8
4/19/2018 6:33	1.7	17.2	8	4.2	0.184	2
4/19/2018 6:34	2.4	17.1	8	4.2	0.1826	1.8
4/19/2018 6:35	2.9	17.4	8.1	4.2	0.1762	1.7
4/19/2018 6:36	1.3	17.2	8.2	4.2	0.1895	1.6
4/19/2018 6:37	2.8	17.3	8.1	4.2	0.1717	1.8
4/19/2018 6:38	2.9	17.1	8.1	4.2	0.1854	1.7
4/19/2018 6:39	2.4	17.2	8.4	4.2	0.1773	1.6
4/19/2018 6:40	2.7	17.4	8.4	4.2	0.1865	1.5
4/19/2018 6:41	3	17.2	8.3	4.2	0.1754	1.6
4/19/2018 6:42	4.1	17.4	8.2	4.1	0.1713	1.7
4/19/2018 6:43	1.3	17.2	8.4	4.1	0.1817	1.6
4/19/2018 6:44	2.4	17.5	8.3	4.1	0.1798	1.7
4/19/2018 6:45	4	17.3	8.2	4.1	0.1812	1.8
4/19/2018 6:46	2.8	17.4	8	4.1	0.1776	1.7
4/19/2018 6:47	1.5	17.2	8.1	4.1	0.1708	1.7
4/19/2018 6:48	3.2	17.4	8.2	4.1	0.1869	1.8
4/19/2018 6:49	3.1	17.4	8.2	4.1	0.1739	1.8
4/19/2018 6:50	3.3	17.3	8.2	4.1	0.1887	1.9
4/19/2018 6:51	3	17.2	8.2	4.1	0.1793	2
4/19/2018 6:52	2.7	17.4	8.1	4.1	0.1808	2.1
4/19/2018 6:53	3.2	17.5	8.2	4.2	0.1949	2.2
4/19/2018 6:54	4.5	17.2	8.3	4.2	0.1751	2.3
4/19/2018 6:55	1.4	17.3	8.3	4.2	0.1835	2.2
4/19/2018 6:56	2.2	17.3	8.2	4.2	0.1821	2.3
4/19/2018 6:57	2.3	17.2	8.2	4.2	0.1802	2.3
4/19/2018 6:58	1.7	17.3	8.3	4.3	0.1847	2.4
4/19/2018 6:59	2.6	17.3	8.3	4.3	0.1768	2.5
4/19/2018 7:00	2.2	17	8.3	4.3	0.1856	2.5
4/19/2018 7:01	1.5	17.1	8.5	4.3	0.1772	2.5
4/19/2018 7:02	3.2	17.2	8.5	4.4	0.1859	2.5
4/19/2018 7:03	3.7	17.2	8.6	4.4	0.1731	2.5
4/19/2018 7:04	2	17.2	8.5	4.4	0.1784	2.4

4/19/2018 7:05			3	17.2	8.5	4.4	0.1903	2.4
4/19/2018 7:06			3.3	17	8.3	4.4	0.1761	2.4
4/19/2018 7:07			2.1	17.1	8.4	4.5	0.1837	2.5
4/19/2018 7:08			2.2	17.1	8.2	4.5	0.1844	2.6
4/19/2018 7:09			3.7	17.3	8.3	4.5	0.1744	2.5
4/19/2018 7:10			2	17.3	8.5	4.6	0.1856	2.5
4/19/2018 7:11			2.8	17.4	8.4	4.6	0.1757	2.4
4/19/2018 7:12			3.1	17.2	8.6	4.6	0.19	2.3
4/19/2018 7:13			0.9	17	8.6	4.7	0.178	2.3
4/19/2018 7:14			1.1	17.2	8.8	4.7	0.1822	2.3
4/19/2018 7:15			2.3	17.3	8.9	4.8	0.19	2.2
4/19/2018 7:16			2.5	17.3	9	4.8	0.1804	2.1
4/19/2018 7:17			3.5	17.2	8.9	4.9	0.1951	2.1
4/19/2018 7:18			2.6	17.5	8.9	4.9	0.1852	2.2
4/19/2018 7:19			3.1	17.2	8.9	5	0.1835	2.3
4/19/2018 7:20			3.3	17.3	8.9	5	0.1862	2.3
4/19/2018 7:21			2.6	17.2	8.8	5.1	0.177	2.3
4/19/2018 7:22			3.2	17	9	5.2	0.1942	2.3
4/19/2018 7:23			2.8	17.3	9.1	5.2	0.1879	2.3
4/19/2018 7:24			2.7	17.3	8.9	5.3	0.1889	2.1
4/19/2018 7:25			3.6	17.5	8.9	5.3	0.1945	1.8
4/19/2018 7:26			2.1	17.3	8.9	5.3	0.1827	2
4/19/2018 7:27			4.5	17.3	9	5.4	0.2103	1.5
4/19/2018 7:28			2.2	17.3	9.1	5.4	0.2269	1.4
4/19/2018 7:29			4.9	17.1	9.1	5.5	0.2312	1.4
4/19/2018 7:30			3	17.2	8.9	5.5	0.2427	1.4
4/19/2018 7:31			2.3	17.1	9.1	5.5	0.2362	1.4
4/19/2018 7:32			2.3	17	9.4	5.6	0.2404	1.4
4/19/2018 7:33			4.1	20	9.3	5.6	0.231	1.4
4/19/2018 7:34			3.8	14.6	9.1	5.7	0.2388	1.4
4/19/2018 7:35			3.2	15.8	9.3	5.8	0.2457	1.4
4/19/2018 7:36			4	16.9	9.4	5.8	0.2437	1.4
4/19/2018 7:37			3.2	17	9.6	5.9	0.2491	1.4
4/19/2018 7:38			4.3	17	9.4	5.9	0.239	1.4
4/19/2018 7:39			3	17.2	9.5	6	0.2478	1.4
4/19/2018 7:40			2.9	17.1	9.6	6	0.2465	1.4
4/19/2018 7:41			2.9	17.1	9.4	6	0.2305	1.4
4/19/2018 7:42			2.9	17.2	9.4	6	0.2461	1.4
4/19/2018 7:43			2.6	17.1	9.5	6	0.2352	1.4
4/19/2018 7:44	0.2	0.1	1.9	17.2	9.4	6	0.2491	1.4
4/19/2018 7:45	0.1	0.2	2.2	17.2	9.2	6	0.2415	1.4
4/19/2018 7:46	0.1	0.1	1.8	17.3	9.2	6	0.2438	1.4
4/19/2018 7:47	0.1	0	2.8	17.3	9.4	6	0.2542	1.4
4/19/2018 7:48	0.1	0.3	3.4	17.1	9.3	6	0.2387	1.4
4/19/2018 7:49	0.1	0.5	3.1	17	9.3	6	0.2556	1.5
4/19/2018 7:50	0.1	-0.5	4.2	16.9	9.2	6	0.2465	1.4
4/19/2018 7:51	0.1	0	2.4	17	10.3	6.1	0.2459	1.4

4/19/2018 7:52	0.2	-0.2	2.8	17.3	18.6	53.3	0.2462	1.4
4/19/2018 7:53	0.2	-0.4	3.4	17.2	19.9	87.1	0.2432	1.4
4/19/2018 7:54	0.3	-0.2		17	19.6	87	0.2441	1.5
4/19/2018 7:55	0.3	-0.2		17.1	11.9	16.7	0.2557	1.5
4/19/2018 7:56	0.3	0.1		17.1	9.3	6.2	0.2398	1.5
4/19/2018 7:57	0.3	-0.2		17.4	9.4	6.4	0.2383	1.5
4/19/2018 7:58	0.3	0.2	6.7	24.2	24.6	20	0.3684	1.5
4/19/2018 7:59	0.3	0.6		27.9	61.7	136.9	0.6815	1.6
4/19/2018 8:00	0.2	0.4	6.7	28.6	83.6	328.2	0.892	1.6
4/19/2018 8:01	0.3	0.2		30.4	111.2	454.6	1.3373	1.6
4/19/2018 8:02	0.3	0.9	9.1	31.3	128.2	586.9	2.1125	1.6
4/19/2018 8:03	0.3	-0.3	7.4	32.9	122.6	611.8	2.9396	1.6
4/19/2018 8:04	0.3	0.3	9.6	33.7	118.6	528.9	4.1368	1.7
4/19/2018 8:05	0.3	0.6	6.1	33.8	111.8	479.3	5.0083	1.7
4/19/2018 8:06	0.3	0.2	10.8	36.6	116	487.7	6.6066	1.8
4/19/2018 8:07	0.3	-0.6	7.2	36.3	113.5	487.6	7.9257	1.8
4/19/2018 8:08	0.2	0.4	8.5	35.1	112.7	469.7	8.2484	1.8
4/19/2018 8:09	0.2	0.4	7.8	33.8	111.5	470.7	8.775	1.7
4/19/2018 8:10	0.2	0	6.8	34.6	114.2	469.1	10.405	1.7
4/19/2018 8:11	0.2	0.3	7.4	37	126.3	528.1	11.475	1.8
4/19/2018 8:12	0.2	0.3	8.5	39.4	137.3	580.2	14.605	1.9
4/19/2018 8:13	0.2	0.1	7.3	39.3	143	630.4	15.3492	1.9
4/19/2018 8:14	0.2	0.1	7.9	38.1	138.8	625	14.6042	1.9
4/19/2018 8:15	0.2	-0.1	8.9	37.7	137.5	624	14.7217	1.9
4/19/2018 8:16	0.2	0.3	7.6	37.5	134.8	613.4	15.0508	1.9
4/19/2018 8:17	0.1	-0.4	8.7	36.3	134.7	614.5	15.3108	1.9
4/19/2018 8:18	0.1	0.6	7.8	33.7	125.4	608.3	14.4075	2
4/19/2018 8:19	0.1	0.3	7.8	34.1	126.1	591.8	14.9692	2
4/19/2018 8:20	0.1	-0.5	7.2	34.1	123.8	588.2	15.1267	1.8
4/19/2018 8:21	0.1	-0.2	7.2	33.5	122.3	587.5	15.3092	1.8
4/19/2018 8:22	0.1	0.1	7.3	32.9	120.6	584.6	15.5558	2
4/19/2018 8:23	0.1	0.2	6.9	31.2	112.5	550.9	14.4242	2
4/19/2018 8:24	0.1	0	7.4	31.8	114.6	561.9	15.0383	1.8
4/19/2018 8:25	0.1	-0.2	7.2	29.4	108.1	548.5	14.1517	1.9
4/19/2018 8:26	0.1	0.4	7.5	28.8	103.1	509.3	13.0958	2.1
4/19/2018 8:27	0.1	-0.2	8	29.4	101.3	491.9	13.7908	2.1
4/19/2018 8:28	0.1	-0.1	6.2	28.5	100.8	506	13.8258	1.8
4/19/2018 8:29	0.1	0.3	7.6	29.8	103.5	513.8	14.1458	1.9
4/19/2018 8:30	0.1	0.5	5.4	28.9	100.5	523.9	14.5125	2
4/19/2018 8:31	0.1	0.3	6.2	28.2	98.4	510.8	13.8392	1.8
4/19/2018 8:32	0.1	0.8	7.2	28.4	101.2	506.3	14.3317	1.9
4/19/2018 8:33	0.1	0.8	7.2	27.8	96.3	508.2	14.3342	2.1
4/19/2018 8:34	0.1	0.3	6.9	27.3	93.9	504.3	13.7125	2.1
4/19/2018 8:35	0.1	0.6	6.3	27.3	92.4	470.3	13.3933	2.2
4/19/2018 8:36	0.1	-0.8	5.8	27.6	94.8	486.3	14.1775	2.2
4/19/2018 8:37	0.1	0.3	6.1	26.8	92.3	479.6	13.9342	2.2
4/19/2018 8:38	0.1	0.2	6.8	27.1	91.6	481.7	13.99	2.2

4/19/2018 8:39	0.2	0	6.1	26.2	89.4	477	13.5483	2.2
4/19/2018 8:40	0.2	-0.3	6.1	25.5	82.8	447.7	12.66	2.3
4/19/2018 8:41	0.2	0.8	6.8	26.6	87.1	445.2	13.4492	2.2
4/19/2018 8:42	0.1	0	6.4	25.6	85.8	471	13.3958	2.2
4/19/2018 8:43	0.2	0.3	6.5	26.1	87	468.1	13.6542	2.2
4/19/2018 8:44	0.2	0.8	6.5	25.4	84	460.5	13.2425	2.2
4/19/2018 8:45	0.2	0.2	6.8	25.1	79.6	447.5	12.7158	2.2
4/19/2018 8:46	0.2	0.1	6.9	24.7	80.8	449.7	13.1733	2.3
4/19/2018 8:47	0.2	0.6	6.6	25	79	434.2	12.6067	1.9
4/19/2018 8:48	0.3	0.6	5.9	24.4	79	421.9	12.78	1.7
4/19/2018 8:49	0.3	0.5	5.5	24.7	75.6	408.1	12.335	1.8
4/19/2018 8:50	0.3	0.8	7.5	24.6	76.2	410.7	12.5533	1.9
4/19/2018 8:51	0.3	0.7	5.2	24.3	75.7	417.9	12.6642	2
4/19/2018 8:52	0.2	0.4	5.8	24.3	74.5	414.6	12.5417	2.2
4/19/2018 8:53	0.2	0.1	5.4	22.7	66.3	404.8	11.2683	2.4
4/19/2018 8:54	0.2	0.6	5.4	23.1	67.2	374	11.2342	2.5
4/19/2018 8:55	0.2	0.3	6.2	24	69.1	389	11.9408	2.5
4/19/2018 8:56	0.2	1.2	6.2	23.6	70.3	404.6	12.0392	2.6
4/19/2018 8:57	0.2	0.2	5.7	24	70.1	398.3	12.1417	2.6
4/19/2018 8:58	0.2	-0.2	5	22.6	65.3	396	11.4008	2.6
4/19/2018 8:59	0.2	0.5	4.8	22.4	63.2	353.4	10.6117	2.5
4/19/2018 9:00	0.2	-0.1	6.3	23	65.3	387.2	11.4425	2.6
4/19/2018 9:01	0.2	0.3	5	23.3	65.6	381	11.5225	2.5
4/19/2018 9:02	0.2	0.6	5.7	22.6	64.8	379.2	11.335	2.5
4/19/2018 9:03	0.2	0.1	5.7	22.7	62.9	350.6	11.0858	2.6
4/19/2018 9:04	0.2	0.7	4.7	22.5	63.4	375.6	11.2608	2.5
4/19/2018 9:05	0.2	1.1	3.9	21.8	58.6	359.2	10.32	2.5
4/19/2018 9:06	0.2	0	4.4	22.2	57.9	350.1	10.3175	2.5
4/19/2018 9:07	0.2	-0.2	5.9	22.1	58.6	333.6	10.5633	2.6
4/19/2018 9:08	0.2	0.1	5.3	22	59.1	347.5	10.6825	2.6
4/19/2018 9:09	0.3	0.1	4.9	22.1	56.5	335.1	10.1908	2.6
4/19/2018 9:10	0.3	0.3	4.7	21.9	56	329.5	10.2208	2.6
4/19/2018 9:11	0.3	0.3	5	21.3	55.8	349.3	10.1122	2.6
4/19/2018 9:12	0.3	0.2	5.7	21.5	55.7	339.4	10.2093	2.5
4/19/2018 9:13	0.3	0.2	5.1	21.3	53.4	335	9.7735	2.5
4/19/2018 9:14	0.3	0	5	20.7	52.7	332.6	9.6279	2.5
4/19/2018 9:15	0.3	0.4	4.4	21	51.2	319.5	9.3862	2.5
4/19/2018 9:16	0.2	-0.1	4.9	21.3	52.1	309.6	9.6565	2.5
4/19/2018 9:17	0.2	0	4	20.9	50.3	306.3	9.3394	2.5
4/19/2018 9:18	0.2	0.7	5.8	21	51.9	323	9.7379	2.4
4/19/2018 9:19	0.2	0	5.5	21	49.3	300.5	9.1933	2.5
4/19/2018 9:20	0.2	0.2	4.5	21	50.5	311.7	9.5838	2.6
4/19/2018 9:21	0.3	0.5	5.1	20.1	49.7	321.2	9.3516	2.6
4/19/2018 9:22	0.3	0.6	4.1	21	48.3	293.8	9.2313	2.7
4/19/2018 9:23	0.3	0.3	4.6	20.5	47.8	292.9	9.0326	2.8
4/19/2018 9:24	0.3	0.7	4.6	20.4	46.4	288.9	8.7379	2.8
4/19/2018 9:25	0.2	0.2	4.3	20.3	45	264.2	8.3636	2.8

4/19/2018 9:26	0.2	-0.1	4.6	19.8	44.3	268.7	7.9759	2.7
4/19/2018 9:27	0.2	0.6	4.3	18.5	44.3	227.1	6.4286	2.7
4/19/2018 9:28	0.4	1.4	3	18.1	41.2	164.3	4.9053	2.7
4/19/2018 9:29	0.6	0.7	3.5	17.9	31.4	141.6	4.4562	2.7
4/19/2018 9:30	0.7	0.5	4.7	17.9	30.6	134	4.139	2.7
4/19/2018 9:31	0.7	1.1	3	18	30.6	131.8	4.1158	2.6
4/19/2018 9:32	0.8	1	4.1	17.9	30.4	125.3	4.0127	2.6
4/19/2018 9:33	0.8	1	3.1	17.8	30.1	124.1	3.9691	2.5
4/19/2018 9:34	0.9	1	3.3	17.8	28.2	106.4	3.3829	2.5
4/19/2018 9:35	1	1.2	4.6	17.7	26.9	92.9	2.9733	2.5
4/19/2018 9:36	1.1	1.7	3.8	17.8	27	98.9	2.9994	2.5
4/19/2018 9:37	1.2	1.5	2.4	17.6	26.5	88.6	2.9718	2.4
4/19/2018 9:38	1.2	1.5	4.5	18	25.7	80.4	2.5839	2.4
4/19/2018 9:39	1.2	1.1	2	17.7	25.8	77.2	2.7223	2.5
4/19/2018 9:40	1.3	1.6	4.6	17.8	24.4	68.6	2.4175	2.5
4/19/2018 9:41	1.4	2.3	4.3	17.6	23.8	66.9	2.2943	2.5
4/19/2018 9:42	1.6	2.1	4.6	17.9	22.5	53.7	1.8758	2.6
4/19/2018 9:43	1.7	1.8	2.9	17.7	23.2	54.6	2.0558	2.6
4/19/2018 9:44	1.9	2.4	3.5	18.1	21.9	48.2	1.7283	2.6
4/19/2018 9:45	2	2.6	4.1	18.1	21.4	45.1	1.6265	2.5
4/19/2018 9:46	2	1.7	3.3	17.6	22.4	48	1.8238	2.6
4/19/2018 9:47	2	2.9	3.6	18.2	21.8	44.7	1.623	2.7
4/19/2018 9:48	1.9	2	2.2	17.6	22.3	46.8	1.6681	2.7
4/19/2018 9:49	1.7	2	3.1	17.5	22.9	50.4	1.7107	2.6
4/19/2018 9:50	1.7	2.2	3.3	18.2	21	39.4	1.3893	2.5
4/19/2018 9:51	1.9	2.6	3.5	17.9	20.5	37.6	1.3524	2.4
4/19/2018 9:52	2.2	2.3	4.6	18.2	21	35.1	1.2981	2.4
4/19/2018 9:53	2.4	4.1	4.2	18.4	20	29.2	1.1551	2.3
4/19/2018 9:54	2.6	2.3	4.6	18.1	19.3	23.7	1.0615	2.4
4/19/2018 9:55	2.7	2.5	3.8	18.5	19.7	23.9	1.0338	2.3
4/19/2018 9:56	2.7	2.6	3.4	18.1	19.7	23.7	1.02	2.3
4/19/2018 9:57	2.7	3.4	5	18.7	19.1	23.7	0.9631	2.3
4/19/2018 9:58	2.8	2.8	3.6	18.5	19.5	23.4	0.9423	2.4
4/19/2018 9:59	2.7	2	4.1	18.1	19.6	23	0.9323	2.5
4/19/2018 10:00	2.7	3.3	3.9	19.1	18.5	22.4	0.8251	2.5
4/19/2018 10:01	3	3.6	4.6	19	17.8	21.6	0.7862	2.4
4/19/2018 10:02	2.9	2	2.5	17.3	19.1	20.7	0.8527	2.4
4/19/2018 10:03	2.3	1.1	2.6	17	20.7	19.6	0.8876	2.4
4/19/2018 10:04	1.5	1.1	3	20.9	27.8	20.2	1.0023	2.4
4/19/2018 10:05	0.8	0.5	3.2	27.1	63.7	85.7	1.4162	2.5
4/19/2018 10:06	0.5	0	4.3	24.8	76.2	279.5	1.4513	2.5
4/19/2018 10:07	0.2	0.5	5.1	25.3	113.8	417.3	1.695	2.5
4/19/2018 10:08	0.2	0.1	4.5	25.3	122.1	561.5	1.9745	2.6
4/19/2018 10:09	0.2	0.2	6	28.6	150.3	678.4	3.0793	2.7
4/19/2018 10:10	0.2	0.4	5.4	31.7	154.9	714.4	4.2482	2.6
4/19/2018 10:11	0.2	0.3	5.8	35.1	162.4	757.6	5.9052	2.8
4/19/2018 10:12	0.2	0.9	7.4	37.2	162.2	759.6	7.6607	2.8

4/19/2018 10:13	0.1	0.6	7.2	37.3	161.4	731.2	8.479	2.9
4/19/2018 10:14	0.1	0.1	6.9	36.9	151.4	717.7	8.8392	2.9
4/19/2018 10:15	0.1	-0.1	5.6	36.4	146.8	697.2	9.8823	2.9
4/19/2018 10:16	0.1	0.9	5.4	37.6	152.3	691.7	10.7135	2.9
4/19/2018 10:17	0.1	0.5	7.4	34.8	142	650.5	11.53	2.8
4/19/2018 10:18	0.1	0.3	5.6	36.2	150.5	654.9	12.535	2.6
4/19/2018 10:19	0.1	-0.6	6	36.1	155.4	677.7	13.375	2.7
4/19/2018 10:20	0.1	0.8	6.2	35.5	151.8	701.2	14.8517	2.7
4/19/2018 10:21	0.1	0.2	5.6	34.6	148.2	738.7	15.4583	2.7
4/19/2018 10:22	0.1	0.2	4.2	35.9	155.1	751.3	16.985	2.7
4/19/2018 10:23	0.1	0.8	4.6	32.6	141.2	726.1	15.625	2.7
4/19/2018 10:24	0.1	-0.1	5.4	31.4	133.7	671.3	15.43	2.7
4/19/2018 10:25	0.1	0.2	4.4	30.8	128.6	675.1	15.1	2.3
4/19/2018 10:26	0.1	0.2	6.7	32	137.9	685.4	16.6683	2.4
4/19/2018 10:27	0.1	-0.4	5.3	30.1	128.7	692.1	15.7633	2.3
4/19/2018 10:28	0.1	0.2	4.7	29.6	124.3	633.2	14.8967	2.2
4/19/2018 10:29	0.1	0.2	4.9	29.8	124.4	628.1	15.9917	2.3
4/19/2018 10:30	0.1	0.4	5.3	33.6	146.9	669.4	18.4583	2.3
4/19/2018 10:31	0.1	1	6.1	33.9	151.5	757.3	19.7067	2.4
4/19/2018 10:32	0.1	0.6	5.6	33	148.4	752.5	19.4383	2.5
4/19/2018 10:33	0.1	0.3	4.5	33	148.5	754	19.5017	2.5
4/19/2018 10:34	0.1	0.4	6.1	32.5	147.7	754.6	19.575	2.5
4/19/2018 10:35	0.1	0.3	6.7	31.9	143	752.5	19.2167	2.5
4/19/2018 10:36	0.1	0.8	5.8	31.5	142.4	749.8	19.3183	2.6
4/19/2018 10:37	0.1	-0.1	5.2	30.5	138.8	741	18.9383	2.6
4/19/2018 10:38	0.1	0.1	5.5	29.7	133.4	700.8	18.4033	2.6
4/19/2018 10:39	0.1	0.2	5.2	29.5	131.4	687.3	18.3	2.6
4/19/2018 10:40	0.2	0.3	5.1	28.9	126.7	689	18.05	2.6
4/19/2018 10:41	0.2	0.1	4.8	28.7	128.6	677.4	18.07	2.6
4/19/2018 10:42	0.1	0.7	4.9	27.4	117.4	651.4	17.05	2.6
4/19/2018 10:43	0.2	0.3	4.6	27.8	122.1	638.3	17.395	2.6
4/19/2018 10:44	0.2	0.5	5	27.4	120.3	659.7	17.31	2.7
4/19/2018 10:45	0.2	0.2	5.1	27.1	115.4	644.3	17.0367	2.6
4/19/2018 10:46	0.3	1.2	5.4	27.4	118.2	637	17.36	2.7
4/19/2018 10:47	0.3	0.2	5.2	26	111.1	635.9	16.745	2.6
4/19/2018 10:48	0.2	0.4	4.8	26.3	111.5	617	16.365	2.6
4/19/2018 10:49	0.2	0.2	4.6	25.7	108.5	620.9	16.3633	2.6
4/19/2018 10:50	0.2	0.2	4.1	25.1	101.4	608.6	15.6567	2.6
4/19/2018 10:51	0.2	0.8	6	25.3	103.1	588.4	15.6967	2.6
4/19/2018 10:52	0.2	0.3	4.2	24.3	99.5	588.1	15.3883	2.6
4/19/2018 10:53	0.2	0.2	4.9	24.6	98.8	578.6	15.2617	2.7
4/19/2018 10:54	0.2	0.3	5.7	25.4	105.1	562.7	15.87	2.7
4/19/2018 10:55	0.1	0.6	5	25.2	104.4	594.5	16.29	2.6
4/19/2018 10:56	0.1	0	4.9	25	101.4	581	15.9067	2.6
4/19/2018 10:57	0.1	0.4	5.1	24.8	99.1	573.5	15.805	2.6
4/19/2018 10:58	0.1	0.2	5.4	24.9	101.1	577.1	16.105	2
4/19/2018 10:59	0.1	0.6	5.8	23.4	93.8	558.8	15.1733	1.9

4/19/2018 11:00	0.1	0.9	5.5	23.9	93.1	540.4	14.8267	2
4/19/2018 11:01	0.1	0.6	5.2	24.5	94.7	541.5	15.4583	2.1
4/19/2018 11:02	0.1	0.5	4.1	23.9	94.8	557.5	15.3683	2.2
4/19/2018 11:03	0.1	0.4	4.6	23.3	90.1	549.9	14.6683	2.3
4/19/2018 11:04	0.1	0.5	5.3	24.1	92.9	537.8	14.9733	2.3
4/19/2018 11:05	0	0	5.3	23.2	90.4	545.1	14.7483	2.3
4/19/2018 11:06	0.1	0.7	4.6	23.3	88	530.7	14.57	2.2
4/19/2018 11:07	0.1	0.1	4.8	22.2	80.5	492	13.29	2.3
4/19/2018 11:08	0.1	0.2	5.4	22.4	80.3	497.8	13.3533	2.3
4/19/2018 11:09	0.1	0.6	5.4	22	76.3	476.8	13.1317	2.3
4/19/2018 11:10	0.1	0.7	4.9	21.9	75.1	445.9	12.7083	2.2
4/19/2018 11:11	0.1	0.1	4.1	21.5	74.6	437.9	12.8067	2.3
4/19/2018 11:12	0.2	-0.6	3.2	20.9	71	417.4	12.0533	2.3
4/19/2018 11:13	0.2	0.7	3.9	21.1	69	418.5	11.9067	2.2
4/19/2018 11:14	0.2	0.4	4.4	20.8	69	414.8	11.8383	2.3
4/19/2018 11:15	0.2	0.2	4.6	21.1	69.7	409.9	12.05	2.4
4/19/2018 11:16	0.2	0.4	2.9	22.4	73.4	452.3	12.87	2.5
4/19/2018 11:17	0.2	-0.1	3.2	22.5	77.1	485.5	13.56	2.4
4/19/2018 11:18	0.2	0.3		22.5	78.9	494.8	14.0067	2.4
4/19/2018 11:19	0.2	0.9	2.9	22.7	78.9	495.6	14.0317	2.3
4/19/2018 11:20	0.2	1	5.4	22.4	76.4	486.9	13.5983	2.3
4/19/2018 11:21	0.1	0		21.9	74.7	486.1	13.45	2.3
4/19/2018 11:22	0.1	0.3		21.5	73.7	478.7	13.26	2.5
4/19/2018 11:23	0.1	0.7		21.6	71.9	464.9	12.8317	2.5
4/19/2018 11:24	0.1	0.1	4.9	21.7	71.2	449.7	12.67	2.5
4/19/2018 11:25	0.1	0.3	5.1	21.5	70.7	452.2	12.7417	2.5
4/19/2018 11:26	0.1	1	4	21.4	69.5	449.9	12.5967	2.5
4/19/2018 11:27	0.1	0.8	5.1	19.7	62.8	418.2	10.9668	2.5
4/19/2018 11:28	0.3	0.6	4	19.3	53.5	340.9	8.4825	2.6
4/19/2018 11:29	0.3	0.7	4.2	17.8	60.4	330.1	8.92	2.4
4/19/2018 11:30	0.4	0.9	2.5	16.3	60.4	239.4	5.6567	2.3
4/19/2018 11:31	0.3	0.7	3.7	16.4	34.8	144.6	4.379	2.3
4/19/2018 11:32	0.3	0.7	4.3	16.6	34.8	141.8	4.5267	2.5
4/19/2018 11:33	0.4	1	2.9	16.6	34.1	140.3	4.3748	2.4
4/19/2018 11:34	0.4	1.4	3.4	16.2	32.5	117.1	3.8478	2.3
4/19/2018 11:35	0.5	0.9	3.2	16	31.3	102.1	3.4608	2.3
4/19/2018 11:36	0.5	0.6	2.5	16.6	31.3	108.4	3.6335	2.3
4/19/2018 11:37	0.5	0.7	4.3	16.4	31.5	113.1	3.7238	2.3
4/19/2018 11:38	0.5	1.1	2.2	16.5	30.8	104	3.466	2.4
4/19/2018 11:39	0.6	1.6	5.2	16.5	29.5	92.8	2.9438	2.4
4/19/2018 11:40	0.7	1.4	3.1	16.6	29.6	90.5	3.052	2.3
4/19/2018 11:41	0.8	1.1	4.2	16.5	29.5	91.5	3.0602	2.4
4/19/2018 11:42	0.7	1.6	2.8	16.7	29.1	86.4	2.9923	2.4
4/19/2018 11:43	0.8	0.6	3.5	16.5	28.2	76.8	2.5927	2.5
4/19/2018 11:44	0.8	1.5	3.4	16.5	28.3	76.3	2.7055	2.4
4/19/2018 11:45	0.9	0.9	4.9	16.5	28.1	74	2.5965	2.5
4/19/2018 11:46	0.9	1.4	2	16.6	27.7	69.7	2.4977	2.3

4/19/2018 11:47	1	1.2	1.2	16.9	27.4	70.4	2.3578	2.2
4/19/2018 11:48	1	0.6	4	16.7	27.3	70	2.4355	2.3
4/19/2018 11:49	1.1	1.4	3.5	16.9	27	68.9	2.2592	2.4
4/19/2018 11:50	1.1	0.5	3.6	16.7	26.4	58	2.0895	2.2
4/19/2018 11:51	1.1	1.6	3.2	16.8	26.5	56.8	2.1237	2.3
4/19/2018 11:52	1.2	0.9	3.7	16.5	26.1	50.9	1.9368	2.3
4/19/2018 11:53	1.3	1.4	2.9	16.9	25.9	50.2	1.925	2.4
4/19/2018 11:54	1.3	1.6	2.5	17.2	25.8	49	1.8753	2.5
4/19/2018 11:55	1.4	2.1	2.4	16.9	25.4	48.5	1.7437	2.5
4/19/2018 11:56	1.4	2.6	3.5	16.7	25.3	47.8	1.7541	2.5
4/19/2018 11:57	1.5	2.3	2.5	17.1	25.3	47.3	1.7515	2.5
4/19/2018 11:58	1.5	1.5	3.9	17.2	25.3	47	1.8003	2.2
4/19/2018 11:59	1.7	1.8	2.9	17.4	25.2	46.5	1.7582	2.2
4/19/2018 12:00	1.7	2.1	3	17.5	24.7	45.6	1.6921	2.3
4/19/2018 12:01	1.8	2	3.2	17.8	24.4	44.5	1.5655	2.4
4/19/2018 12:02	1.9	2	2.6	17.5	24.1	42.7	1.4773	2.5
4/19/2018 12:03	2	2.6	3.9	17.7	23.9	41.3	1.5068	2.5
4/19/2018 12:04	2.1	2.1	3.5	17.4	23.9	40.5	1.4578	1.7
4/19/2018 12:05	2.1	2.4	2.6	17.6	23.5	39	1.4176	1.6
4/19/2018 12:06	2.2	2.8	4.1	18.1	23.2	37.1	1.3559	1.6
4/19/2018 12:07	2.4	2.7	3.2	18.1	22.8	34.9	1.3148	1.5
4/19/2018 12:08	2.6	2.8	3.4	18.5	22.8	33.5	1.3488	1.4
4/19/2018 12:09	2.6	2.7	5.3	18.5	22.2	31.8	1.2187	1.4
4/19/2018 12:10	2.7	3.2	4.9	18.3	21.9	30.3	1.1956	1.5
4/19/2018 12:11	2.7	2.4	4.3	18.4	21.9	29.3	1.1677	1.6
4/19/2018 12:12	2.7	2.9	3.3	18.5	21.8	27.8	1.133	1.7
4/19/2018 12:13	2.7	3.8	4.4	18.5	21.5	26.2	1.086	1.7
4/19/2018 12:14	2.8	3.4	5.1	18.8	21	24.8	1.0604	1.6
4/19/2018 12:15	3.2	4	4.4	19.5	20	23.8	0.957	1.8
4/19/2018 12:16	3.4	4	3.3	19	20	22.2	0.9602	1.8
4/19/2018 12:17	3.5	3.9	2.2	19	19.9	20.8	0.9199	1.9
4/19/2018 12:18	3.5	4.1	2.8	19.3	19.3	19.9	0.9092	2
4/19/2018 12:19	3.5	3.7	7.4	27.7	19.6	18.9	0.9325	2
4/19/2018 12:20	4	6	6.8	17.3	18.5	17.4	0.7911	1.9
4/19/2018 12:21	4.5	4.8	7.9	19.3	18.8	16.3	0.7832	1.9
4/19/2018 12:22	5.1	6	8.1	22.1	18.1	15.1	0.7299	1.9
4/19/2018 12:23	5.7	8.1	8.6	23.7	17	13.9	0.6707	2
4/19/2018 12:24	6.4	7.1	8.1	23.1	17.3	12.8	0.6445	1.9
4/19/2018 12:25	6.7	6.1	8	22.9	19.1	11.8	0.6835	1.8
4/19/2018 12:26	6.3	5.6	7	21.9	20	10.7	0.6897	1.6
4/19/2018 12:27	5.4	5.3	7.6	21.6	20.7	9.4	0.7034	1.6
4/19/2018 12:28	4.7	4.2	6.5	21.1	21.7	8.6	0.7321	1.5
4/19/2018 12:29	3.8	3.3	8.7	22.7	25.4	16.5	0.7665	1.6
4/19/2018 12:30	2.8	2.3	11.3	23.9	27.6	33	0.8517	1.8
4/19/2018 12:31	1.7	1.4	11.8	26	27.6	43.7	0.9949	1.8
4/19/2018 12:32	0.9	2.2	12.8	28.4	39.6	47.9	1.1763	1.7
4/19/2018 12:33	0.5	1.4	13.1	30.6	41.9	52.4	1.3751	1.9

4/19/2018 12:34	0.3	2.1	14.6	33.5	43.7	55	1.5981	1.8
4/19/2018 12:35	0.2	2.6	16.2	38.7	44.3	55.2	1.9373	2
4/19/2018 12:36	0.2	2.2	16.5	40.2	43.8	55.4	2.2228	2.2
4/19/2018 12:37	0.2	1.5	15.4	38.8	43.1	55.6	2.2835	2
4/19/2018 12:38	0.3	2.3	15.5	38.6	42.9	55.9	2.4848	2
4/19/2018 12:39	0.3	1.9	15.8	37	41.7	55.9	2.4107	2.1
4/19/2018 12:40	0.3	2	13.5	36.4	40.9	55.8	2.4182	2.1
4/19/2018 12:41	0.3	1.6	14.3	36.6	40.8	55.8	2.4894	2
4/19/2018 12:42	0.4	1.6	13	35.1	39.6	55.5	2.4153	2.1
4/19/2018 12:43	0.4	1.6	13.7	35.2	39.1	55.2	2.4402	2.2
4/19/2018 12:44	0.4	1.8	13.3	34.2	38.5	54.6	2.4643	2.2
4/19/2018 12:45	0.4	1.6	13.5	32.9	37.1	53.8	2.3584	2.3
4/19/2018 12:46	0.4	1.3	13.1	33.3	37.6	53.1	2.4767	2.2
4/19/2018 12:47	0.4	1.5	12.5	32.5	36.7	52.3	2.3996	2.1
4/19/2018 12:48	0.4	2.3	12.8	32.9	35.9	51.6	2.3799	2.1
4/19/2018 12:49	0.4	1.2	12.2	31.8	36.6	51.2	2.46	2.1
4/19/2018 12:50	0.5	1.3	11	30.4	34.2	50.3	2.2593	2.2
4/19/2018 12:51	0.4	2.1	11.8	31.2	34.6	49.7	2.37	2.1
4/19/2018 12:52	0.5	1.5	11.8	31	35.2	49.2	2.4116	2.1
4/19/2018 12:53	0.3	1.4	12.8	30.4	34.8	49	2.3996	2.1
4/19/2018 12:54	0.3	1.5	11.9	29.5	34.2	48.7	2.3511	2.1
4/19/2018 12:55	0.3	1.8	12.2	29.7	33	48.1	2.2815	2.1
4/19/2018 12:56	0.4	1.6	12.4	29.2	32.6	47.6	2.28	2.3
4/19/2018 12:57	0.4	1.6	10	29.3	32.7	47.1	2.2945	2.2
4/19/2018 12:58	0.4	1.2	11.6	28.9	32.1	46.6	2.2656	1.9
4/19/2018 12:59	0.4	1.5	10.5	28.5	31.9	46.5	2.2668	1.9
4/19/2018 13:00	0.4	2.1	9.9	27.8	31.2	45.8	2.2027	1.9
4/19/2018 13:01	0.5	1.1	12.2	27.8	30.6	45	2.1908	2
4/19/2018 13:02	0.5	1.2	10.2	27.7	30.5	44.5	2.186	2.2
4/19/2018 13:03	0.5	2.2	10.8	26.8	29.9	43.8	2.134	2.2
4/19/2018 13:04	0.7	2.2	10.9	26.3	27.8	43	1.9713	2
4/19/2018 13:05	0.9	2.1	11.2	26.8	28.2	42.2	2.029	2
4/19/2018 13:06	0.8	1.3	9.7	27.4	29	41.7	2.1224	2.1
4/19/2018 13:07	0.6	0.9	11.3	26.6	29.6	41.2	2.1689	2
4/19/2018 13:08	0.5	1.7	10.9	26.6	28.7	40.7	2.0723	2.1
4/19/2018 13:09	0.4	1.3	11.7	26.6	29	40.5	2.1309	2.1
4/19/2018 13:10	0.4	1.3	10	26.4	28.7	40.2	2.1043	2.1
4/19/2018 13:11	0.4	1.4	11.1	26.1	28.2	39.8	2.0638	2
4/19/2018 13:12	0.4	1.7	10.8	26.1	27.6	39.7	2.0408	2
4/19/2018 13:13	0.5	1.3	10.1	25.2	27.9	39.9	2.052	2.1
4/19/2018 13:14	0.5	1.3	8.8	25.8	27.1	39.8	2.0343	1.9
4/19/2018 13:15	0.4	1.3	8.6	25.4	27.4	39.5	2.0226	2.1
4/19/2018 13:16	0.5	1.8	9	25.1	26.2	39	1.9147	2.3
4/19/2018 13:17	0.5	0.7	10.1	25.5	26.4	38.6	1.9733	2.4
4/19/2018 13:18	0.6	1.2	9.5	24.8	26	38.2	1.9151	2.4
4/19/2018 13:19	0.6	1.2	9	25	25.2	37.6	1.8951	2.4
4/19/2018 13:20	0.6	1.2	9.3	24.3	25.4	37.3	1.8643	2.3

4/19/2018 13:21	0.7	1.6	9.7	24.7	25.3	36.7	1.9028	2.4
4/19/2018 13:22	0.6	1	9.9	24.4	24.6	36.2	1.8302	2.4
4/19/2018 13:23	0.7	1.2	8.5	23.8	24.2	35.5	1.8038	2.4
4/19/2018 13:24	0.8	1.8	8.7	23.9	23.3	34.7	1.736	2.4
4/19/2018 13:25	0.8	2.1	9.8	24.6	23.9	34.3	1.7874	2.5
4/19/2018 13:26	0.8	1.8	8.5	23.8	23.7	33.9	1.7693	2.3
4/19/2018 13:27	0.8	1.5	8	23	22.1	33.2	1.605	2.3
4/19/2018 13:28	1.1	2	7.1	23.5	21.2	32.2	1.5574	2.6
4/19/2018 13:29	1.3	3	9	23.1	21.6	31.6	1.5898	2.5
4/19/2018 13:30	1.4	1.9	7.5	22.9	21	30.7	1.5676	2.6
4/19/2018 13:31	1.2	2.6	9.3	22.8	21.3	29.8	1.5759	2.7
4/19/2018 13:32	1.3	1.6	8.1	22.9	21.3	29.4	1.5123	2.7
4/19/2018 13:33	1.3	2.4	7.1	22.9	21.1	28.9	1.569	2.6
4/19/2018 13:34	1.3	2.7	8.4	22.9	20.4	27.9	1.5227	2.8
4/19/2018 13:35	1	1.7	8.5	23	21.7	27.8	1.6902	2.7
4/19/2018 13:36	0.7	1.1	9.2	23	22.8	28.1	1.7417	2.5
4/19/2018 13:37	0.4	1.2	8.4	23.1	22.5	28.4	1.732	2.5
4/19/2018 13:38	0.3	1.5	8.5	22.9	22.3	28.8	1.7081	2.7
4/19/2018 13:39	0.3	1.2	7.5	22.8	22	29.1	1.6998	2.5
4/19/2018 13:40	0.3	1.3	7.4	23	21.9	29.5	1.6931	2.6
4/19/2018 13:41	0.3	1.1	8.1	22.8	21.5	29.8	1.6585	2.6
4/19/2018 13:42	0.4	1.4	7.6	22.5	21	30.1	1.6248	2.6
4/19/2018 13:43	0.5	1.1	7.8	22.6	20.7	30.1	1.5896	2.2
4/19/2018 13:44	0.6	1.3	8.3	22.3	20.4	29.8	1.5778	2
4/19/2018 13:45	0.6	0.9	7.8	22.5	20.4	29.4	1.576	2.1
4/19/2018 13:46	0.5	0.7	8.5	22.2	20.5	29.2	1.5984	2.1
4/19/2018 13:47	0.8	3.1	8.6	22.7	18.8	27.1	1.3906	2.1
4/19/2018 13:48	2.8	8.7	7.8	24.3	14.4	12.6	0.9063	2.2
4/19/2018 13:49	5.1	7.9	9	24.2	12.6	10	0.7819	2.3
4/19/2018 13:50	7	8.5	7.6	24.2	11.8	8.4	0.6974	2.3
4/19/2018 13:51	7.6	9.3	10	25	11.4	7.5	0.649	2.4
4/19/2018 13:52	8	9.4	8.5	24.8	11.1	7.4	0.6258	2.4
4/19/2018 13:53	8.2	8.8	8.3	24.5	11.1	7.3	0.6196	2.6
4/19/2018 13:54	8.3	9.2	8.1	24.6	10.8	7.1	0.5761	2.5
4/19/2018 13:55	8.3	9.9	8.4	24.8	10.3	6.8	0.5317	2.5
4/19/2018 13:56	8.6	10.6	8.7	25.6	10.1	6.5	0.5031	2.5
4/19/2018 13:57	9.2	10.7	8.9	26	9.6	6.1	0.4666	2.2
4/19/2018 13:58	9.8	11.1	9.7	26.2	9.4	5.7	0.4455	1.5
4/19/2018 13:59	10.3	10.7	7.5	26.3	9.5	5.2	0.4549	1.4
4/19/2018 14:00	10.3	10.5	8.7	25.6	9.6	4.9	0.466	1.4
4/19/2018 14:01	10.3	11.3	8.4	26.7	9.6	4.6	0.4267	1.5
4/19/2018 14:02	10.2	9.6	8.4	25.6	10.2	4.3	0.4778	1.6
4/19/2018 14:03	9.5	7.3	6.8	24.1	13.4	4.1	0.5893	1.5
4/19/2018 14:04	8.4	7.7	7.9	23.9	13.5	4	0.5782	1.5
4/19/2018 14:05	7.2	7.5	7.9	25.3	14.2	7.5	0.5796	1.5
4/19/2018 14:06	5.4	6.4	44	48.4	60.2	79	1.006	1.6
4/19/2018 14:07	3.3	6	69.3	62.9	95.1	246.5	2.0503	1.7

4/19/2018 14:08	1.1	8.7	59.8	79.8	123.6	314	2.6665	1.8
4/19/2018 14:09	0.3	9.7	76.5	88.7	132.1	390.6	3.1437	2
4/19/2018 14:10	0.2	9.5		90.9	135	411	3.6112	2.2
4/19/2018 14:11	0.3	9.3	69.1	86.1	119.6	376.4	3.6752	2.3
4/19/2018 14:12	0.3	9.7	77	96.4	115.1	337.6	4.2588	2.4
4/19/2018 14:13	0.3	10	85.1	105.5	120.8	340.2	5.8887	2.6
4/19/2018 14:14	0.3	10		103.4	117.5	336.3	6.4965	2.7
4/19/2018 14:15	0.3	8.8	71.3	94.6	110.7	335.3	6.6445	2.8
4/19/2018 14:16	0.3	9.1	75.1	91.7	106.6	340.9	7.1158	2.8
4/19/2018 14:17	0.3	9.7	98.7	99.9	108	322.3	7.5628	2.8
4/19/2018 14:18	0.2	9.7	84.2	109.2	117.6	326.1	9.3087	3
4/19/2018 14:19	0.2	10.9	88.1	107.9	115	344.9	9.4312	3
4/19/2018 14:20	0.2	10.6	75.6	112.7	118.4	374.6	10.9133	3.1
4/19/2018 14:21	0.2	10	97.6	107.7	118.5	369.6	10.8983	3.1
4/19/2018 14:22	0.2	11.7		121.1	114.4	378.9	12.0083	3.1
4/19/2018 14:23	0.2	10.6	137	175	114.1	413.3		4.4
4/19/2018 14:24	0.2	10.1	101.3	119.2	123.5	423.3	17.325	3.8
4/19/2018 14:25	0.3	10.2	96.2	117.3	123.7	404.5	13.06	3.4
4/19/2018 14:26	0.3	9.8	95.7	115.1	122.3	401.3	12.6217	3.4
4/19/2018 14:27	0.2	9.6	93.8	111	120.9	395.7	12.4733	3.4
4/19/2018 14:28	0.2	9.8	89.5	106.7	118.1	393.1	12.2867	3.3
4/19/2018 14:29	0.1	9.6	90.8	106.4	116.7	392.5	12.455	3.2
4/19/2018 14:30	0.2	9	87.9	102.1	114.1	378.8	12.275	3.2
4/19/2018 14:31	0.2	8.9	85.8	100.7	112.1	376.5	12.3433	3.2
4/19/2018 14:32	0.2	9.2	82.3	96.5	110.4	364.4	12.035	3.3
4/19/2018 14:33	0.2	8.3	80.7	93.7	110.3	358.9	11.9333	3.3
4/19/2018 14:34	0.1	7.4	77.1	91.9	105.3	344.4	11.6217	3.3
4/19/2018 14:35	0.1	7.8	76.5	90.4	101.5	351.4	11.9383	3.3
4/19/2018 14:36	0.2	7.2	74.7	88.1	100.5	357.7	11.7183	3.3
4/19/2018 14:37	0.2	8	77	89.2	99.3	351.8	11.785	3.1
4/19/2018 14:38	0.2	7.2	74.6	85.4	98.7	344.3	11.8783	3.1
4/19/2018 14:39	0.2	6.9	71	82.8	95.1	336.5	11.6117	3.3
4/19/2018 14:40	0.2	6.6	72.2	81.7	94.6	335.7	11.5983	3.3
4/19/2018 14:41	0.2	6.2	66.2	75.6	91	328.5	11.0767	3.2
4/19/2018 14:42	0.2	5.9	66.2	76.6	86.7	310.7	10.7	3.3
4/19/2018 14:43	0.2	5.3	57.4	65.9	76.9	305.7	9.9592	3.3
4/19/2018 14:44	0.2	6.7	57.8	70.7	76.6	290.4	9.4987	3.3
4/19/2018 14:45	0.2	4.5	53.4	60.2	73	279.8	9.349	3.2
4/19/2018 14:46	0.3	5.2	54.7	65.1	72.6	258.2	9.2123	3.1
4/19/2018 14:47	0.3	5.6	56.5	66.1	73.4	252.7	9.5787	2.8
4/19/2018 14:48	0.3	5.1	52.1	61.5	72.8	258	9.5965	2.8
4/19/2018 14:49	0.3	5.7	53.9	63	70.2	264.4	10.5325	2.9
4/19/2018 14:50	0.3	5.5	57	67.3	71.7	277.2	13.0817	3
4/19/2018 14:51	0.3	5.5	59.1	66.8	76	287.7	10.65	3
4/19/2018 14:52	0.3	5.4	59.2	68.7	77.2	286.4	10.6017	3.1
4/19/2018 14:53	0.3	5.6	59.8	70.4	79.7	304.6	11.1783	3.1
4/19/2018 14:54	0.3	5.6	59.1	68.9	79.3	308.2	11.065	3.2

4/19/2018 14:55	0.3	5.6	57.8	67.7	78.1	307.1	10.93	3.2
4/19/2018 14:56	0.3	5.5	58.1	66.9	76.8	305.5	10.8983	3.2
4/19/2018 14:57	0.3	4.8	56	64.9	74.7	291.1	10.6	3.2
4/19/2018 14:58	0.2	5.5	55.2	64.9	73.7	286.6	10.5683	3.2
4/19/2018 14:59	0.2	5	54.1	62.9	73	284.6	10.4817	3.1
4/19/2018 15:00	0.2	4.5	50.9	62.1	71.2	279.4	10.2492	3
4/19/2018 15:01	0.2	4.9	51.3	61.8	69.8	277.5	10.11	3
4/19/2018 15:02	0.2	4.3	53	60	69.5	279.2	10.1605	2.4
4/19/2018 15:03	0.2	4.5	51.2	59.5	68.6	277.3	10.1125	2.3
4/19/2018 15:04	0.2	4	49.5	58.5	67.5	272.9	10.001	2.4
4/19/2018 15:05	0.2	4.5	45.4	54.7	64.2	263.5	9.4147	2.4
4/19/2018 15:06	0.2	4.8	48	57.8	64.4	263.8	9.6218	2.5
4/19/2018 15:07	0.1	3.5	41.9	48.3	61.3	255.3	8.9272	2.5
4/19/2018 15:08	0.2	3.7	39.7	49.7	56.1	235.1	8.1795	2.5
4/19/2018 15:09	0.2	3.3	40	48.7	56.1	229.5	8.2732	2.6
4/19/2018 15:10	0.2	3.3	38.6	48.9	53.6	222.8	8.0812	2.7
4/19/2018 15:11	0.2	3.6	36.1	44	51.5	215.1	7.6047	2.7
4/19/2018 15:12	0.2	3.2	38.1	45.7	50.7	194.7	7.6095	2.8
4/19/2018 15:13	0.3	4.6	41.3	50	53.4	210.3	8.2177	2.8
4/19/2018 15:14	0.2	3.6	39.4	47.7	54.1	219.5	8.407	2.8
4/19/2018 15:15	0.2	3.5	38.2	46.8	53	226.2	8.5575	2.8
4/19/2018 15:16	0.3	3.7	38.1	47.4	51.1	212.6	8.1747	2.8
4/19/2018 15:17	0.3	4.3	39.3	49.4	52	220	8.6577	2.9
4/19/2018 15:18	0.3	3	39.7	48.8	53	226.2	8.5082	3
4/19/2018 15:19	0.2	3.9	39.2	50.6	54.9	233.9	8.7493	3
4/19/2018 15:20	0.2	3.2	40.1	49.9	55.7	238	8.9063	2.8
4/19/2018 15:21	0.2	3.8	39.3	49.4	55.1	237.8	8.8445	2.3
4/19/2018 15:22	0.2	3	39	48.7	54.1	237.2	8.6798	2.4
4/19/2018 15:23	0.2	3.3	39.1	47.9	53.2	236.4	8.592	2.5
4/19/2018 15:24	0.2	3.3	36.9	47.3	52.4	234.9	8.4888	2.5
4/19/2018 15:25	0.2	3.2	37.7	47	51.9	233.4	8.4893	2.7
4/19/2018 15:26	0.2	3.5	36.3	45.8	51	231.9	8.3488	2.9
4/19/2018 15:27	0.2	2.9	35.4	44.4	49.3	218.2	8.0788	2.9
4/19/2018 15:28	0.2	2.7	34.3	44.8	37.4	206.7	8.073	2.6
4/19/2018 15:29	0.2	2.7	34.1	43.1	7.4	84	7.985	2
4/19/2018 15:30	0.3	3	34.1	43.7	0.1	0.2	7.8168	1.9
4/19/2018 15:31	0.3	2.9	33.1	42.1	-0.3	0.1	7.7517	1.9
4/19/2018 15:32	0.3	2.1	24.7	31.8	-0.3	0.1	6.7175	1.8
4/19/2018 15:33	0.7	4.6	19.6	29.3	-0.3	0.1	4.1258	1.7
4/19/2018 15:34	1.3	3.6	16.5	26.4	-0.3	0.1	3.5428	1.6
4/19/2018 15:35	1.8	4	15.1	26.4	-0.3	0.1	3.0597	1.6
4/19/2018 15:36	2	3.9	13.9	25.7	-0.2	0.1	2.9478	1.6
4/19/2018 15:37	2.1	4.5	14.5	26.1	0	0	2.909	1.6
4/19/2018 15:38	2.2	4.8	13.5	25.3	0.1	0	2.6668	1.6
4/19/2018 15:39	2.3	4.1	15.6	26.4	0.9	73.4	2.7785	1.6
4/19/2018 15:40	2.3	4.6	11.7	24.4	0.3	109.2	2.4987	1.6
4/19/2018 15:41	2.3	3.7	12	24.2	1.1	105.5	2.4903	1.6

4/19/2018 15:42	2.5	4.4	11.4	23.7	1.9	104.3	2.1423	1.5
4/19/2018 15:43	2.6	4.8	10.9	23.7	2.5	104.2	2.0747	1.5
4/19/2018 15:44	2.7	4.3	11.6	23.5	2.8	103.9	2.0485	1.5
4/19/2018 15:45	2.6	4.3	10.3	22.9	2.9	103.7	1.9762	1.5
4/19/2018 15:46	2.8	4.9	9.1	22.9	3	103.5	1.6705	1.5
4/19/2018 15:47	3.2	4.9	10.2	22.7	2.9	103.3	1.538	1.5
4/19/2018 15:48	3.4	5	8.7	22.3	2.8	102.3	1.4805	1.5
4/19/2018 15:49	3.5	5.5	7.5	22.4	2.7	100.7	1.369	1.5
4/19/2018 15:50	3.5	5.5	9.3	22.4	6.6	62.6	1.3482	1.5
4/19/2018 15:51	3.7	4.9	7	22.2	37.3	19.6	1.2152	1.4
4/19/2018 15:52	3.9	6.1	8.1	22.6	52.5	26.9	1.2	1.5
4/19/2018 15:53	4.1	6.6	8.6	22.8	65.4	29.7	1.1165	1.5
4/19/2018 15:54	4.1	5.3	5.9	21.8	72.3	32.2	1.193	1.5
4/19/2018 15:55	4.2	5.9	8	22.2	74.9	33.4	1.076	1.5
4/19/2018 15:56	4.3	6	8.1	22.4	75.5	33.5	1.0548	1.5
4/19/2018 15:57	4.6	7	7	22.7	75.5	33.6	0.9218	1.5
4/19/2018 15:58	5.1	7.5	7.8	22.7	75.5	33.6	0.8461	1.5
4/19/2018 15:59	5.4	7.2	8.8	22.9	75.5	33.7	0.8512	1.5
4/19/2018 16:00	5.5	6.4	7.8	22.9	75.5	33.7	0.8718	1.5
4/19/2018 16:01	5.5	7.2	7.7	23	75.9	33.7	0.7989	1.5
4/19/2018 16:02	5.5	6.3	5.7	22.5	76.6	33.6	0.791	1.5
4/19/2018 16:03	5.3	5.7	8	22.1	76.9	33.5	0.9063	1.5
4/19/2018 16:04	5.2	7.1	6.9	22.7	67.2	33.4	0.744	1.5
4/19/2018 16:05	5.5	8.2	7.9	23.1	196.8	28.6	0.6636	1.5
4/19/2018 16:06	6.2	8.5	7	23.6	0.1	1.5	0.6158	1.5
4/19/2018 16:07	6.7	8.7	7.4	23.8	-0.1	0	0.5762	1.6
4/19/2018 16:08	7.1	9.1	5.9	24	-0.1	0	0.5429	1.5
4/19/2018 16:09	7.3	8.2	6.8	24	-0.1	-0.1	0.5635	1.5
4/19/2018 16:10	7.3	7.8	8.4	23.9	-0.1	-0.1	0.5434	1.6
4/19/2018 16:11	7.2	9	6.6	24.1	-0.1	-0.1	0.512	1.6
4/19/2018 16:12	7	8.7	5.9	23.6	0	4.9	0.5665	1.5
4/19/2018 16:13	6.8	7.9	5.5	23.5	0.4	87.6	0.5351	1.9
4/19/2018 16:14	6.9	9.1	7.1	23.7	0.2	102.4	0.4996	2.5
4/19/2018 16:15	7	8.9	8	23.9	0.3	102.4	0.5019	1.7
4/19/2018 16:16	7.2	8.9	7.1	24.1	0.4	102.7	0.4801	1.7
4/19/2018 16:17	7.5	9.8	6.7	24.6	0.4	102.8	0.4266	2.1
4/19/2018 16:18	7.9	9.9	6.9	24.6	0.4	102.7	0.4045	3.9
4/19/2018 16:19	8.3	10.5	8.4	24.9	0.4	102.7	0.3846	4.8
4/19/2018 16:20	8.6	10.5	8.1	25.1	0.3	101.6	0.3736	2.3
4/19/2018 16:21	8.9	11.7	8.4	25.9	6.3	63.6	0.3365	2
4/19/2018 16:22	9.6	12.3	7.7	26.8	30.9	12.9	0.3104	2
4/19/2018 16:23	10.2	12.2	8.8	26.7	48.8	22.7	0.3076	1.9
4/19/2018 16:24	10.7	13.2	7.7	27.7	57	27.1	0.2821	1.9
4/19/2018 16:25	11.1	13.3	8.2	28.1	63.4	28.7	0.2788	1.8
4/19/2018 16:26	11.6	13.9	8.4	28.4	66.2	29.2	0.2407	1.7
4/19/2018 16:27	12.1	15	8.9	28.4	67.1	29.2	0.2361	1.6
4/19/2018 16:28	12.1	13	7.3	27.6	67.4	29.2	0.2526	1.6

4/19/2018 16:29	11.5	11	8.2	26.5	67.5	29.3	0.262	1.7
4/19/2018 16:30	10.8	10.9	7.1	26.3	67.5	29.3	0.2758	1.7
4/19/2018 16:31	10.1	11.2	8.5	26.8	70	29.3	0.2561	1.7
4/19/2018 16:32	10.4	12.8	7.6	27.4	21.3	18.5	0.2504	1.7
4/19/2018 16:33	10.5	12.7	8.4	27.6	7.7	4	0.2321	1.7
4/19/2018 16:34	10.9	13	8.3	27.7	7.4	2.3	0.2413	1.6
4/19/2018 16:35	10.3	9.7	8.2	27.4	7.4	2.5	0.2305	1.6
4/19/2018 16:36	8.6	9.1	8.2	27.8	7.4	2.6	0.238	1.6
4/19/2018 16:37	6	10.5	9	28.8	7.3	2.6	0.2138	1.6
4/19/2018 16:38	3.4	11.5	8.3	28.6	7.2	2.5	0.2109	1.7
4/19/2018 16:39			7.8	28.8	7.1	2.5	0.2024	1.7
4/19/2018 16:40			8.9	29	7	2.4	0.1953	1.7
4/19/2018 16:41			9	28.6	7.4	2.3	0.1981	1.7
4/19/2018 16:42			9	28	8.5	2.3	0.19	1.7
4/19/2018 16:43			8.8	27.1	8.9	2.3	0.2016	1.7
4/19/2018 16:44			8.3	26	9.2	2.4	0.1921	1.8
4/19/2018 16:45			6.8	26.3	9.5	2.4	0.2001	1.8
4/19/2018 16:46			7.4	26.5	10.1	2.5	0.1721	1.8
4/19/2018 16:47			7.4	26.6	10.5	2.5	0.1886	1.8
4/19/2018 16:48			6.8	26.5	11.2	2.5	0.18	1.8
4/19/2018 16:49			8.2	26.5	11.6	2.5	0.1766	1.8
4/19/2018 16:50			7.6	26.7	11.8	2.5	0.1778	1.7
4/19/2018 16:51			7.2	26.5	12	2.4	0.1686	1.7
4/19/2018 16:52			6.8	26.4	12	2.4	0.1766	1.7
4/19/2018 16:53			7.1	26.2	11.8	2.3	0.1641	1.8
4/19/2018 16:54			6.7	25.9	11.8	2.3	0.1817	1.8
4/19/2018 16:55			6.5	25.1	11.7	2.3	0.1675	1.8
4/19/2018 16:56			8	25.8	11.5	2.3	0.1794	1.9
4/19/2018 16:57			6.3	26	11.4	2.3	0.1652	2
4/19/2018 16:58			6.1	25.6	11.1	2.3	0.1773	2
4/19/2018 16:59			8	26.3	11.1	2.4	0.1623	2
4/19/2018 17:00			7.1	27.8	11	2.3	0.1543	2
4/19/2018 17:01			8.3	28.1	10.9	2.3	0.166	1.9
4/19/2018 17:02			7.9	28.1	10.6	2.3	0.1495	1.9
4/19/2018 17:03			7.3	27.7	10.3	2.2	0.1576	1.9
4/19/2018 17:04			7.6	27.9	10	2.2	0.1467	1.9
4/19/2018 17:05			6.8	27.6	9.8	2.2	0.1637	1.9
4/19/2018 17:06			8.5	28	9.5	2.1	0.1517	2
4/19/2018 17:07			8.9	28.3	9.3	2.1	0.1606	1.9
4/19/2018 17:08			8.7	29.1	9.1	2	0.1496	1.9
4/19/2018 17:09			8.3	29	8.9	2	0.1599	2
4/19/2018 17:10			8.9	28.8	8.9	2	0.1567	2
4/19/2018 17:11			8.4	27.9	9	2	0.1627	1.9
4/19/2018 17:12			7.8	26.8	9.1	2	0.1606	1.9
4/19/2018 17:13			8	26.3	9	2.1	0.1617	1.6
4/19/2018 17:14			7.3	26.2	8.8	2.1	0.1623	1.7
4/19/2018 17:15			7	25.8	8.5	2.1	0.1509	1.7

4/19/2018 17:16	5.8	25.6	8.4	2.2	0.161	1.8
4/19/2018 17:17	7.4	24.4	8.5	2.2	0.1508	1.9
4/19/2018 17:18	5	24.3	8.1	2.3	0.1572	1.9
4/19/2018 17:19	5.8	24.3	8.3	2.4	0.1478	2.1
4/19/2018 17:20	5.9	24.1	8.2	2.5	0.1708	2.1
4/19/2018 17:21	6.6	23.6	8.3	2.5	0.1622	2.1
4/19/2018 17:22	6.1	23.7	8.1	2.6	0.1623	2.2
4/19/2018 17:23	5.8	23.3	8.1	2.6	0.1576	2.2
4/19/2018 17:24	4.3	23.1	8.1	2.7	0.1549	2
4/19/2018 17:25	7.4	23.9	8.1	2.7	0.1586	1.9
4/19/2018 17:26	8.8	23.8	8	2.7	0.1455	1.9
4/19/2018 17:27	7.9	23.4	8	2.7	0.1594	2
4/19/2018 17:28		23.1	7.8	2.8	0.1464	2
4/19/2018 17:29	12.9	23.2	7.7	2.8	0.1642	2.1
4/19/2018 17:30	5.5	23.4	7.8	2.8	0.1492	2
4/19/2018 17:31		23.7	7.7	2.8	0.1613	2.1
4/19/2018 17:32		23.5	7.6	2.8	0.1528	2.1
4/19/2018 17:33	5.1	22.8	7.6	2.8	0.1572	2.1
4/19/2018 17:34		23.3	7.5	2.9	0.152	2.1
4/19/2018 17:35		23.5	7.5	2.9	0.1472	2.2
4/19/2018 17:36		23.4	7.5	2.9	0.1567	2.3
4/19/2018 17:37	5.8	23.6	7.5	2.9	0.1534	2.3
4/19/2018 17:38	5.9	23.6	7.3	2.9	0.1572	2.4
4/19/2018 17:39	6.5	22.6	7.1	2.9	0.1464	2.4
4/19/2018 17:40		23.2	7.1	2.9	0.1606	2.3
4/19/2018 17:41	5.6	23.4	7.1	2.9	0.1407	2.3
4/19/2018 17:42	5.6	23.6	7	2.8	0.159	2.4
4/19/2018 17:43	5.5	22.8	6.9	2.9	0.1449	2.4
4/19/2018 17:44		22.7	6.9	2.9	0.1581	2.4
4/19/2018 17:45	6.9	23.9	7	2.9	0.1439	2.4
4/19/2018 17:46	6.6	23.6	6.9	2.9	0.1511	2.2
4/19/2018 17:47	6.2	23.4	6.9	2.9	0.1487	2.2
4/19/2018 17:48	4.9	22.8	6.7	2.9	0.145	2.3
4/19/2018 17:49	6.3	22.9	6.7	2.9	0.1573	2.3
4/19/2018 17:50	6.3	23.2	6.8	2.9	0.1427	2.4
4/19/2018 17:51	7.8	23.6	6.7	2.9	0.1542	2.4
4/19/2018 17:52	6.7	23.7	6.8	2.9	0.1329	2.4
4/19/2018 17:53	7	23.6	6.8	2.8	0.1543	2.4
4/19/2018 17:54	4.4	22.8	6.6	2.9	0.1443	2.3
4/19/2018 17:55	5.7	22.9	6.7	2.9	0.1503	2.2
4/19/2018 17:56	6.9	23.3	6.7	2.9	0.137	2.2
4/19/2018 17:57	5.1	23.8	6.8	2.8	0.1457	2.2
4/19/2018 17:58	4.6	22.5	6.6	2.8	0.1393	2.2
4/19/2018 17:59	5	22.2	6.5	2.9	0.1535	2
4/19/2018 18:00	5	22.7	6.5	2.9	0.1428	2.1
4/19/2018 18:01	6	23	6.6	2.9	0.1539	2.1
4/19/2018 18:02	6.7	23.4	6.6	2.9	0.151	2.1

4/19/2018 18:03	5.3	22.8	6.6	2.9	0.1308	2
4/19/2018 18:04	5.9	22.9	6.5	2.9	0.1497	1.8
4/19/2018 18:05	5.9	23.2	6.6	2.9	0.1326	1.8
4/19/2018 18:06	5.6	23.1	6.5	3	0.1477	1.8
4/19/2018 18:07	4.6	23.2	6.5	2.9	0.1365	1.7
4/19/2018 18:08	5.2	22.8	6.5	2.9	0.1479	1.8
4/19/2018 18:09	5.9	22.8	6.5	2.9	0.1406	1.8
4/19/2018 18:10	3.5	22.1	6.5	3	0.1443	1.9
4/19/2018 18:11	5.6	23.1	6.7	3	0.1378	1.9
4/19/2018 18:12	5.7	22.9	6.9	3	0.1434	1.9
4/19/2018 18:13	6.1	22.7	6.8	3	0.1403	1.8
4/19/2018 18:14	6.1	22.7	7	3	0.1385	1.8
4/19/2018 18:15	5.4	22.9	6.9	3	0.1542	1.8
4/19/2018 18:16	5.1	22.3	7	3	0.1359	1.8
4/19/2018 18:17	5.1	22.8	7	3	0.1493	1.6
4/19/2018 18:18	5.4	22.7	7.1	3	0.1351	1.7
4/19/2018 18:19	6.5	22.5	7.1	3	0.1516	1.7
4/19/2018 18:20	5.2	22.9	7.3	2.9	0.1296	1.5
4/19/2018 18:21	6.1	23.1	7.2	2.9	0.1515	1.5
4/19/2018 18:22	5.2	21.7	7	3	0.1462	1.6
4/19/2018 18:23	5.1	21.3	6.9	3	0.1478	1.7
4/19/2018 18:24	4.8	21	7	3	0.1431	1.9
4/19/2018 18:25	5.7	21.6	6.9	3	0.1393	1.9
4/19/2018 18:26	3.5	21.5	7	3	0.1434	2
4/19/2018 18:27	5.7	22.3	6.9	3	0.1465	2
4/19/2018 18:28	6.6	21.9	7	3	0.1556	2.1
4/19/2018 18:29	6.3	23.6	7	3	0.138	2.2
4/19/2018 18:30	6.8	24.5	6.9	3	0.1477	2.2
4/19/2018 18:31	7.4	25	6.8	2.9	0.1323	2.2
4/19/2018 18:32	6.9	24.6	6.7	2.8	0.1409	2.2
4/19/2018 18:33	6.3	23.8	6.7	2.7	0.1253	2.2
4/19/2018 18:34	6.7	23.9	6.4	2.7	0.1415	2.3
4/19/2018 18:35	7.1	23.9	6.5	2.6	0.1366	2.2
4/19/2018 18:36	5	23	6.2	2.6	0.1426	2.2
4/19/2018 18:37	5.3	22.7	6.2	2.6	0.1405	2.3
4/19/2018 18:38	5.2	23.4	6.2	2.6	0.1377	2.3
4/19/2018 18:39	6.4	24	6.2	2.6	0.1427	2.3
4/19/2018 18:40	6.8	24.1	6.2	2.6	0.1327	2.4
4/19/2018 18:41	5.4	24.1	6.1	2.7	0.1414	2.5
4/19/2018 18:42	7	24.2	6	2.7	0.1292	2.4
4/19/2018 18:43	6.3	23.1	5.9	2.7	0.1462	2.2
4/19/2018 18:44	5.4	23.5	5.9	2.7	0.1428	2.3
4/19/2018 18:45	4.7	23.5	5.8	2.7	0.1343	2.4
4/19/2018 18:46	4.1	22.4	5.7	2.7	0.142	2.5
4/19/2018 18:47	6.7	22	6.1	2.8	0.1372	2.5
4/19/2018 18:48	5.4	21.3	6.3	2.8	0.1466	2.5
4/19/2018 18:49	5.4	21.9	6.2	2.9	0.1321	2.3

4/19/2018 18:50	4.4	22.3	6.1	2.9	0.1438	2.4
4/19/2018 18:51	4.7	22.6	6.2	2.9	0.1321	2.5
4/19/2018 18:52	5.4	22.3	6	2.9	0.1412	2.4
4/19/2018 18:53	4.6	22.2	6.2	3	0.134	2.4
4/19/2018 18:54	6.1	23	6	3	0.1385	2.5
4/19/2018 18:55	5.4	23.1	5.9	2.9	0.1366	2.4
4/19/2018 18:56	5.8	23.7	5.6	2.9	0.1329	2.5
4/19/2018 18:57	5.6	24.8	5.6	2.8	0.1356	2.6
4/19/2018 18:58	5.3	23.8	5.4	2.8	0.1342	2.6
4/19/2018 18:59	5.3	24	5.4	2.7	0.135	2.5
4/19/2018 19:00	4.8	24.8	5.4	2.7	0.1312	2.5
4/19/2018 19:01	6	24.4	5.3	2.6	0.1391	2.6
4/19/2018 19:02	6.5	24.1	5.2	2.6	0.1243	2.6
4/19/2018 19:03	6.6	24.4	5.1	2.5	0.1414	2.5
4/19/2018 19:04	6	24.8	5.2	2.5	0.1288	2.5
4/19/2018 19:05	5.9	24.9	5.1	2.5	0.141	2.5
4/19/2018 19:06	6.3	25.4	5.1	2.5	0.124	2.5
4/19/2018 19:07	5.9	24	5.1	2.4	0.1349	2.5
4/19/2018 19:08	5.9	23.5	5	2.4	0.1366	2.4
4/19/2018 19:09	5.3	24	5	2.5	0.1261	2.4
4/19/2018 19:10	5.4	23.1	5.1	2.5	0.1349	2.5
4/19/2018 19:11	4.1	21.9	5.6	2.6	0.1392	2.5
4/19/2018 19:12	4.7	21.6	5.8	2.6	0.1489	2.5
4/19/2018 19:13	4.8	21.8	5.8	2.7	0.1333	2.6
4/19/2018 19:14	4.3	22.3	5.7	2.8	0.1452	2.5
4/19/2018 19:15	3.9	22.5	5.7	2.9	0.1263	2.5
4/19/2018 19:16	6.3	22.8	5.7	2.9	0.1514	2.6
4/19/2018 19:17	5.2	22.3	5.8	3	0.1333	2.6
4/19/2018 19:18	4.6	22.7	5.7	3	0.1332	2.6
4/19/2018 19:19	5.3	23.3	5.6	2.9	0.1371	2.6
4/19/2018 19:20	6.8	24.7	5.6	2.9	0.1249	2.6
4/19/2018 19:21	6.3	24.8	5.4	2.8	0.1299	2.6
4/19/2018 19:22	4.9	24.8	5.4	2.7	0.122	2.5
4/19/2018 19:23	7	24.6	5.3	2.7	0.1349	2.5
4/19/2018 19:24	7.7	25.5	5.3	2.6	0.1196	2.6
4/19/2018 19:25	6.1	24.8	5.3	2.5	0.1267	2.6
4/19/2018 19:26	6.4	24.7	5.3	2.4	0.1314	2.5
4/19/2018 19:27	6	24.3	5.2	2.4	0.1246	2.5
4/19/2018 19:28	6	24.7	5.2	2.3	0.1413	2.5
4/19/2018 19:29	5.8	24.4	5.1	2.3	0.1232	2.5
4/19/2018 19:30	5.7	24.5	5.1	2.3	0.1376	2.5
4/19/2018 19:31	6.5	24.8	5.1	2.3	0.1239	2.5
4/19/2018 19:32	6.4	24	5.1	2.3	0.1368	2.5
4/19/2018 19:33	6.8	23	5.1	2.4	0.1248	2.6
4/19/2018 19:34	4.4	22	5.3	2.5	0.1342	2.4
4/19/2018 19:35	5.4	21.6	5.5	2.6	0.1396	2.4
4/19/2018 19:36	5.2	21.6	5.5	2.7	0.1316	2.4

4/19/2018 19:37	5.2	22.1	5.4	2.7	0.1395	2.5
4/19/2018 19:38	5.6	22.3	5.4	2.8	0.1223	2.5
4/19/2018 19:39	6	22.1	5.4	2.8	0.1465	2.6
4/19/2018 19:40	3.7	22.1	5.3	2.9	0.1272	2.5
4/19/2018 19:41	5.7	21.7	5.3	3	0.1401	2.5
4/19/2018 19:42	5.7	22	5.2	3	0.1221	2.5
4/19/2018 19:43	4.8	22.8	5.2	2.9	0.131	2.5
4/19/2018 19:44	6.2	23.3	5	2.9	0.1264	2.6
4/19/2018 19:45	6.9	24.2	4.9	2.8	0.1332	2.6
4/19/2018 19:46	5.9	24.5	4.9	2.8	0.1356	2.5
4/19/2018 19:47	5.2	23.9	4.8	2.7	0.1306	2.6
4/19/2018 19:48	6	25.1	4.8	2.6	0.137	2.7
4/19/2018 19:49	6.1	24.6	4.8	2.5	0.1179	2.6
4/19/2018 19:50	6.3	24.1	4.8	2.5	0.1382	2.5
4/19/2018 19:51	6.5	23.9	4.7	2.4	0.1235	2.5
4/19/2018 19:52	6.1	24.4	4.8	2.3	0.1272	2.5
4/19/2018 19:53	7.5	23.5	4.8	2.3	0.1242	2.4
4/19/2018 19:54	4.9	22.6	4.8	2.4	0.1265	2.4
4/19/2018 19:55	4.8	21.6	5.1	2.5	0.1266	2.3
4/19/2018 19:56	3.7	21.2	5.2	2.6	0.1323	2.3
4/19/2018 19:57	4.5	21.9	5.2	2.7	0.1354	2.2
4/19/2018 19:58	4.6	21.3	5.1	2.7	0.1279	2.3
4/19/2018 19:59	5.1	21.7	5.2	2.8	0.1304	2.3
4/19/2018 20:00	4.4	21.4	5.2	2.9	0.1254	2.4
4/19/2018 20:01	5.1	21.3	5.2	3	0.1442	2.3
4/19/2018 20:02	5.3	21.7	5.2	3.1	0.1259	2.3
4/19/2018 20:03	4.6	21.9	5.2	3.1	0.1416	2.3
4/19/2018 20:04	5.5	23.8	5.2	3	0.1323	2.4
4/19/2018 20:05	6	24.3	5.1	2.9	0.1259	2.4
4/19/2018 20:06	6.2	23.5	5.1	2.8	0.1337	2.4
4/19/2018 20:07	5.9	24	5	2.8	0.1168	2.4
4/19/2018 20:08	6.4	25.1	5.1	2.6	0.1353	2.3
4/19/2018 20:09	4.6	24.5	5	2.5	0.1226	2.3
4/19/2018 20:10	6.4	24.7	5	2.4	0.1328	2.3
4/19/2018 20:11	6.3	24.1	5	2.3	0.1212	2.4
4/19/2018 20:12	5.5	23.6	5	2.3	0.1293	2.5
4/19/2018 20:13	6.2	24.5	4.9	2.3	0.127	2.4
4/19/2018 20:14	6	23.1	4.9	2.3	0.1268	2.3
4/19/2018 20:15	5	21.8	5	2.3	0.1311	2.3
4/19/2018 20:16	6	22	5	2.4	0.1318	2.2
4/19/2018 20:17	4.9	21.8	5	2.5	0.1344	2.2
4/19/2018 20:18	5.4	22	5	2.6	0.1246	2.1
4/19/2018 20:19	4.9	21.6	5	2.7	0.1365	2
4/19/2018 20:20		22.4	4.9	2.7	0.1232	2
4/19/2018 20:21	5.8	21.7	4.9	2.8	0.133	2.1
4/19/2018 20:22	8	21.8	4.9	2.9	0.1239	2.1
4/19/2018 20:23		22.9	4.8	2.9	0.1259	2

4/19/2018 20:24		23.1	4.7	2.9	0.1315	2.1
4/19/2018 20:25	6.4	25.2	4.7	2.8	0.1273	2.1
4/19/2018 20:26	5.3	24.9	4.6	2.7	0.137	2.2
4/19/2018 20:27	6.2	25.1	4.6	2.7	0.1204	2.1
4/19/2018 20:28	7.6	25.8	4.5	2.6	0.1395	2.1
4/19/2018 20:29	7.1	25.8	4.5	2.5	0.1224	2.1
4/19/2018 20:30	6.5	25.2	4.5	2.4	0.1367	2.1
4/19/2018 20:31	6.5	24.9	4.5	2.3	0.1303	2.2
4/19/2018 20:32	7.9	24.8	4.4	2.2	0.1354	2.2
4/19/2018 20:33	7	25.2	4.4	2.2	0.1289	2.2
4/19/2018 20:34		24.1	4.4	2.2	0.1183	2.2
4/19/2018 20:35		21.6	4.7	2.2	0.136	2.2
4/19/2018 20:36		21.8	4.8	2.3	0.1175	2.1
4/19/2018 20:37		22	4.7	2.4	0.1302	2.2
4/19/2018 20:38	6.6	21.5	4.7	2.5	0.122	2.3
4/19/2018 20:39	3.3	21.1	4.8	2.6	0.1347	2.4
4/19/2018 20:40		21.1	4.8	2.7	0.1349	2.3
4/19/2018 20:41	4.7	21.6	4.8	2.8	0.1259	2.4
4/19/2018 20:42	7.8	21.9	4.7	2.9	0.1369	2.5
4/19/2018 20:43	5.1	22	4.7	3	0.1214	2.3
4/19/2018 20:44	4.3	21.4	4.7	3	0.1343	2.1
4/19/2018 20:45	3.8	21.2	4.7	3	0.1289	2.2
4/19/2018 20:46	5.7	22.9	4.6	3	0.1383	2.4
4/19/2018 20:47	5.4	24.5	4.5	2.9	0.1292	2.4
4/19/2018 20:48	6	24.6	4.5	2.8	0.1244	2.2
4/19/2018 20:49	7	24.3	4.5	2.7	0.1326	2.5
4/19/2018 20:50	6.7	25.1	4.4	2.6	0.1231	2.1
4/19/2018 20:51	7.9	25	4.4	2.5	0.1326	2
4/19/2018 20:52	6.6	24.3	4.4	2.4	0.1245	2
4/19/2018 20:53	6.1	24.9	4.4	2.3	0.1231	2.1
4/19/2018 20:54	6.4	24.6	4.4	2.3	0.1268	2.2
4/19/2018 20:55	5.6	24.7	4.4	2.2	0.1262	2.1
4/19/2018 20:56	6.7	24	4.3	2.2	0.1369	2.2
4/19/2018 20:57	3.6	23.4	4.3	2.2	0.1235	2.3
4/19/2018 20:58	4.7	21.4	4.6	2.4	0.1418	2.5
4/19/2018 20:59	4.1	21.3	4.6	2.4	0.1274	2.6
4/19/2018 21:00	4.5	21.6	4.6	2.5	0.1351	2.6
4/19/2018 21:01	4.9	21	4.6	2.6	0.13	2.6
4/19/2018 21:02	4.9	20.9	4.6	2.7	0.1362	2.8
4/19/2018 21:03	4.8	20.9	4.6	2.8	0.1382	2.4
4/19/2018 21:04	3.7	20.6	4.5	2.9	0.1203	2
4/19/2018 21:05	3.5	21.6	4.5	3	0.1396	2.1
4/19/2018 21:06	4.1	21.2	4.5	3	0.1235	2.2
4/19/2018 21:07	3.7	21.1	4.5	3	0.1397	2.4
4/19/2018 21:08	5.5	20.7	4.5	3	0.1349	2.5
4/19/2018 21:09	4.6	21.2	4.4	3	0.1306	2.6
4/19/2018 21:10	5.6	23.1	4.3	3	0.1433	2.4

4/19/2018 21:11	4.6	23.2	4.2	2.9	0.1278	2.6
4/19/2018 21:12	5.9	23.6	4.2	2.8	0.1352	2.7
4/19/2018 21:13	5.8	23.9	4.2	2.7	0.1265	2.8
4/19/2018 21:14	5.2	24.3	4.2	2.6	0.1331	2.8
4/19/2018 21:15	6.2	24.1	4.2	2.5	0.1265	2.8
4/19/2018 21:16	6.5	24.2	4.2	2.4	0.1351	2.9
4/19/2018 21:17	6.3	23.6	4.2	2.4	0.1334	2.9
4/19/2018 21:18	6.5	23.9	4.3	2.3	0.1343	2.9
4/19/2018 21:19	5.5	22.5	4.3	2.3	0.1365	2.8
4/19/2018 21:20	4.4	21	4.4	2.3	0.1289	2.8
4/19/2018 21:21	4.9	21.2	4.5	2.4	0.1354	2.7
4/19/2018 21:22	5.5	21.5	4.5	2.5	0.127	2.7
4/19/2018 21:23	6.5	20.8	4.5	2.6	0.1401	2.6
4/19/2018 21:24	4.5	20.7	4.5	2.7	0.1266	2.3
4/19/2018 21:25	4.5	20.4	4.5	2.8	0.1278	2.4
4/19/2018 21:26	4.6	20.6	4.5	2.9	0.1338	2.4
4/19/2018 21:27	4.5	20.7	4.5	3	0.1268	2.6
4/19/2018 21:28	5.7	20.9	4.5	3.1	0.1323	2.7
4/19/2018 21:29	4.1	20.8	4.5	3.1	0.132	2.9
4/19/2018 21:30	4.3	21.1	4.6	3.1	0.1297	2.1
4/19/2018 21:31	3.4	20.4	4.5	3.1	0.1367	1.4
4/19/2018 21:32	4.5	21.4	4.6	3.1	0.1295	1.3
4/19/2018 21:33	5.3	22.7	4.7	3	0.1401	1.3
4/19/2018 21:34	5	23	4.8	2.9	0.1211	1.3
4/19/2018 21:35	6.5	23.8	4.9	2.8	0.1374	1.3
4/19/2018 21:36	5.6	24	4.9	2.7	0.1191	1.4
4/19/2018 21:37	5.1	23	4.9	2.6	0.121	1.5
4/19/2018 21:38	6.6	23.5	4.9	2.5	0.1276	1.6
4/19/2018 21:39	5.1	23.4	4.9	2.4	0.1296	1.6
4/19/2018 21:40	4.2	23.1	4.8	2.3	0.1332	1.7
4/19/2018 21:41	4.3	22.3	4.8	2.3	0.1338	1.8
4/19/2018 21:42	5.2	20.8	4.7	2.3	0.1436	1.9
4/19/2018 21:43	5.1	20.9	4.7	2.4	0.1357	2
4/19/2018 21:44	4.9	20.5	4.7	2.5	0.1389	2.1
4/19/2018 21:45	4.7	20.7	4.7	2.6	0.1219	2.1
4/19/2018 21:46	4.9	20.2	4.6	2.7	0.1394	2.2
4/19/2018 21:47	4.5	20.5	4.6	2.8	0.1331	2.3
4/19/2018 21:48	4.3	20.7	4.6	2.9	0.1309	2.3
4/19/2018 21:49	4.9	21.1	4.6	2.9	0.135	2.4
4/19/2018 21:50	4.1	20.5	4.6	3	0.1344	2.4
4/19/2018 21:51	3.8	20.6	4.6	3	0.1407	2.4
4/19/2018 21:52	3.8	20.1	4.6	3	0.1244	2.5
4/19/2018 21:53	7.2	22.5	4.6	3	0.139	2.6
4/19/2018 21:54	5.7	23	4.7	2.9	0.1225	2.6
4/19/2018 21:55	6.1	22.5	4.7	2.8	0.1385	2.6
4/19/2018 21:56	5.1	23.6	4.7	2.7	0.1287	2.6
4/19/2018 21:57	5.3	23.5	4.7	2.6	0.1277	2.7

4/19/2018 21:58	5.9	23.1	4.8	2.5	0.1322	2.7
4/19/2018 21:59	5.8	23.2	4.8	2.4	0.1211	2.7
4/19/2018 22:00	3.8	21.7	4.7	2.4	0.1382	2.7
4/19/2018 22:01	4.4	20.6	4.6	2.4	0.128	2.5
4/19/2018 22:02	5.5	20.4	4.6	2.4	0.1322	2.3
4/19/2018 22:03	4.4	20.9	4.6	2.5	0.1465	1.8
4/19/2018 22:04	5.9	20.1	4.6	2.5	0.1264	1.7
4/19/2018 22:05	4.5	20.4	4.5	2.7	0.1392	1.7
4/19/2018 22:06	5.8	19.7	4.6	2.8	0.1246	1.7
4/19/2018 22:07	4.6	20.1	4.6	2.9	0.1364	1.8
4/19/2018 22:08	4	20	4.5	3	0.1382	1.8
4/19/2018 22:09	4.9	20.6	4.7	3.1	0.1289	1.8
4/19/2018 22:10	4.2	20.6	5	3.1	0.1462	1.9
4/19/2018 22:11	5.2	20.5	5	3.1	0.1315	2
4/19/2018 22:12	2.7	20.1	4.9	3.1	0.1288	2.1
4/19/2018 22:13	4.6	20.7	5.2	3.1	0.1218	2.1
4/19/2018 22:14	3.9	20.2	5.3	3	0.1417	2.1
4/19/2018 22:15	3.4	21.2	5.4	3	0.133	2.2
4/19/2018 22:16	3.9	21.6	6.1	2.9	0.1294	2.3
4/19/2018 22:17	5.6	21.3	6.2	2.8	0.1374	2.3
4/19/2018 22:18	5.3	21.4	6.5	2.8	0.1246	1.6
4/19/2018 22:19	4.4	22	6.8	2.7	0.1388	1.7
4/19/2018 22:20	5.6	21.8	6.9	2.6	0.1278	2
4/19/2018 22:21	5.6	21.9	6.8	2.5	0.1393	2.2
4/19/2018 22:22	4.3	20.8	6.7	2.5	0.1349	2.4
4/19/2018 22:23	4.7	20.1	5.3	2.6	0.1362	2.2
4/19/2018 22:24	4.4	19.8	5.4	2.6	0.1328	1.9
4/19/2018 22:25	2.5	19.6	5.7	2.7	0.1257	2.2
4/19/2018 22:26	3.5	19.7	5.4	2.7	0.1393	2.4
4/19/2018 22:27	2.7	19.9	5.1	2.8	0.1214	2.3
4/19/2018 22:28	3.5	19.6	5.1	2.9	0.1419	1.9
4/19/2018 22:29	4.1	19.9	5.2	3	0.119	2.2
4/19/2018 22:30	5.2	19.8	5.2	3.1	0.1393	2.4
4/19/2018 22:31	3.9	19.9	5.1	3.1	0.1359	2.5
4/19/2018 22:32	4.3	19.6	5.1	3.1	0.1307	2.6
4/19/2018 22:33	3.5	19.8	5.2	3.1	0.1321	2.7
4/19/2018 22:34	5.2	20	5.4	3.1	0.1271	2.7
4/19/2018 22:35	4	19.9	5.5	3.1	0.1385	2.8
4/19/2018 22:36	3.7	20.2	5.6	3.1	0.1188	2.2
4/19/2018 22:37	2.8	19.9	5.4	3.1	0.1349	2
4/19/2018 22:38	3.6	20.2	5.4	3	0.1287	2.4
4/19/2018 22:39	3.8	19.8	5.4	3	0.1313	2.7
4/19/2018 22:40	5.4	20.8	5.7	3	0.1298	2.8
4/19/2018 22:41	4.5	21.1	5.9	2.9	0.1254	2.9
4/19/2018 22:42	4.8	21.1	5.9	2.9	0.1396	2.9
4/19/2018 22:43	5.7	21.8	6.1	2.8	0.1249	2.4
4/19/2018 22:44	4.4	21.6	6.4	2.7	0.13	2.2

4/19/2018 22:45	5	20.9	6.4	2.7	0.1279	2.6
4/19/2018 22:46	6.2	21.8	6.4	2.6	0.128	2.8
4/19/2018 22:47	6	21.6	6.5	2.5	0.1321	2.9
4/19/2018 22:48	4.4	20.5	6.3	2.5	0.1238	2.7
4/19/2018 22:49	3	19.6	5.4	2.5	0.1418	2.7
4/19/2018 22:50	3.7	19.8	5.5	2.6	0.1263	2.9
4/19/2018 22:51	4.4	19.5	5.7	2.6	0.1362	2.7
4/19/2018 22:52	3.3	19.3	5.5	2.7	0.1257	2.7
4/19/2018 22:53	5.1	19.3	5.4	2.8	0.1358	2.9
4/19/2018 22:54	4	19.6	5.3	2.9	0.1322	2.9
4/19/2018 22:55	2.1	19.3	5.4	3	0.1291	2.9
4/19/2018 22:56	3	19.4	5.4	3.1	0.1393	2.9
4/19/2018 22:57	3.6	19.3	5.4	3.1	0.127	3
4/19/2018 22:58	3.7	19.4	5.4	3.1	0.1365	2.9
4/19/2018 22:59	5.1	19.4	5.4	3.2	0.1235	3
4/19/2018 23:00	4	19.6	5.4	3.2	0.1334	3
4/19/2018 23:01	4.9	19.6	5.4	3.2	0.1277	3
4/19/2018 23:02	4	20	5.6	3.1	0.1354	2.5
4/19/2018 23:03	5.2	19.8	5.5	3.1	0.1358	2.2
4/19/2018 23:04	4.9	19.8	5.4	3.1	0.1297	2.3
4/19/2018 23:05	4.3	20.6	5.5	3.1	0.1372	2.7
4/19/2018 23:06	3	19.6	5.5	3.1	0.124	2.9
4/19/2018 23:07	4.9	21.3	5.6	3.1	0.1403	2.7
4/19/2018 23:08	4.2	21.6	5.7	3	0.1265	2.6
4/19/2018 23:09	5.7	21.3	5.8	2.9	0.128	3
4/19/2018 23:10	4.7	21.9	5.7	2.8	0.1345	3.2
4/19/2018 23:11	5.5	21.8	5.7	2.7	0.1346	3.2
4/19/2018 23:12	5.6	21.7	5.7	2.6	0.1401	3.2
4/19/2018 23:13	5.7	22.1	5.6	2.5	0.1242	3.1
4/19/2018 23:14	4.7	21.8	5.6	2.5	0.1373	2.3
4/19/2018 23:15	5.1	20.7	5.5	2.4	0.1166	2.4
4/19/2018 23:16	4.2	19.8	5.3	2.4	0.1316	2.7
4/19/2018 23:17	4.7	19.8	5.3	2.5	0.1218	2.8
4/19/2018 23:18	4.4	19.8	5.3	2.5	0.1279	2.8
4/19/2018 23:19	4.4	19.2	5.3	2.6	0.1289	2.9
4/19/2018 23:20	4.4	19.3	5.2	2.7	0.1252	3
4/19/2018 23:21	4.8	19.4	5.2	2.8	0.1393	2.9
4/19/2018 23:22	2.2	19.6	5.2	2.9	0.1218	3
4/19/2018 23:23	4.1	19.4	5.1	3	0.142	3.1
4/19/2018 23:24	3.2	19.6	5.1	3	0.1219	3.1
4/19/2018 23:25	4.2	19.6	5.1	3	0.1384	3.2
4/19/2018 23:26	2.6	19.8	5.1	3.1	0.1304	3.2
4/19/2018 23:27	3.6	19.8	5.1	3.1	0.1241	3
4/19/2018 23:28	3.7	19.5	5	3.1	0.1423	3
4/19/2018 23:29	4.3	19.8	5	3	0.1252	3
4/19/2018 23:30	3.7	19.4	5	3	0.1356	3
4/19/2018 23:31	5	19.6	5	3	0.1263	3

4/19/2018 23:32	4.4	20.6	4.9	3	0.1271	2.6
4/19/2018 23:33	5.1	20.1	4.8	2.9	0.1343	2.3
4/19/2018 23:34	4.4	20.8	4.8	2.9	0.1274	2.6
4/19/2018 23:35	5.6	20.9	4.8	2.8	0.1339	2.7
4/19/2018 23:36	4.2	21.2	4.7	2.8	0.1244	2.8
4/19/2018 23:37	4.7	21.2	4.7	2.7	0.1381	2.9
4/19/2018 23:38	4.7	20.4	4.7	2.6	0.1178	3
4/19/2018 23:39	2.9	19.2	4.8	2.6	0.1397	3
4/19/2018 23:40	3.5	19.5	4.8	2.7	0.1263	3
4/19/2018 23:41	4.1	19.5	4.8	2.7	0.1347	3
4/19/2018 23:42	3.4	19.3	4.8	2.7	0.1292	3
4/19/2018 23:43	3.7	19.1	4.8	2.8	0.1201	3
4/19/2018 23:44	3.7	19.1	4.9	2.8	0.1381	3
4/19/2018 23:45	4.4	18.9	4.9	2.9	0.1205	3
4/19/2018 23:46	4	19	4.9	3	0.1325	3
4/19/2018 23:47	2.6	19.2	4.9	3	0.1294	3
4/19/2018 23:48	3.8	19.3	4.9	3	0.1247	3
4/19/2018 23:49	3.1	19.1	4.9	3.1	0.1368	3
4/19/2018 23:50	3.5	19.2	4.9	3.1	0.1201	2.9
4/19/2018 23:51	4.5	18.9	4.9	3.1	0.1352	2.8
4/19/2018 23:52		19.5	5	3.1	0.1179	2.9
4/19/2018 23:53	14.6	19.4	5	3	0.1336	2.9
4/19/2018 23:54		19.3	5	3	0.1253	2.8
4/19/2018 23:55	3.9	19	5	3	0.1312	2.7
4/19/2018 23:56	9.9	19	4.9	3	0.1318	2.8
4/19/2018 23:57		18.8	4.9	3	0.1189	2.7
4/19/2018 23:58	4.9	19.8	5	3	0.1335	2.7
4/19/2018 23:59	4	19.7	5.2	3	0.1259	2.7
4/20/2018 0:00	17.8	20.3	5.1	3	0.1378	2.7
4/20/2018 0:01		20.6	5.4	2.9	0.1281	2.7
4/20/2018 0:02	4.8	20.5	5.4	2.8	0.1239	2.7
4/20/2018 0:03	4.8	19.9	5.3	2.7	0.1332	2.6
4/20/2018 0:04	2.3	18.9	5	2.7	0.1167	2.7
4/20/2018 0:05	3.9	19.2	5	2.7	0.134	2.7
4/20/2018 0:06	4.1	19.1	5	2.7	0.1225	2.6
4/20/2018 0:07	4.1	18.8	5	2.7	0.1293	2.6
4/20/2018 0:08	4.7	19.2	4.9	2.8	0.1257	2.3
4/20/2018 0:09	5.1	18.9	4.9	2.9	0.1317	2.3
4/20/2018 0:10	4	19	4.9	2.9	0.1296	2.1
4/20/2018 0:11	4	18.9	4.9	3	0.125	2.2
4/20/2018 0:12	4	18.9	5	3.1	0.1374	2.2
4/20/2018 0:13	3.6	18.8	5	3.1	0.1241	2.1
4/20/2018 0:14	3.4	19	5.1	3.1	0.1374	2
4/20/2018 0:15	4.9	19.2	5.2	3.1	0.1213	2
4/20/2018 0:16	4.8	19	5.1	3	0.1316	2
4/20/2018 0:17	5.1	18.8	5.1	3	0.1266	2
4/20/2018 0:18	3.4	18.8	5	3	0.1298	2.1

4/20/2018 0:19	5.2	18.7	4.9	3	0.1364	2.1
4/20/2018 0:20	4.6	19.2	5.1	3	0.1229	2.2
4/20/2018 0:21	4.7	19.2	5.6	3	0.137	2.2
4/20/2018 0:22	3.4	19.6	5.5	2.9	0.1245	2.1
4/20/2018 0:23	3.7	18.8	5.1	2.9	0.1329	2.2
4/20/2018 0:24	3.7	18.8	5	3	0.1319	2.3
4/20/2018 0:25	3.9	18.9	5.1	3	0.1216	2.3
4/20/2018 0:26	1.3	18.7	5.2	3	0.1317	2.3
4/20/2018 0:27	3.5	18.7	5.2	3	0.1249	2.4
4/20/2018 0:28	4.1	18.8	5.1	3	0.1352	2.4
4/20/2018 0:29	2.6	18.8	5.1	3	0.1246	2.4
4/20/2018 0:30	3.6	18.7	5	3.1	0.1346	2.4
4/20/2018 0:31	4	18.6	5.1	3.1	0.1234	2.4
4/20/2018 0:32	3	18.5	5.1	3.1	0.1229	2.4
4/20/2018 0:33	3	18.9	5.1	3.2	0.1293	2.5
4/20/2018 0:34	4.2	18.8	5.3	3.2	0.1169	2.5
4/20/2018 0:35	3	18.8	5.6	3.1	0.1382	2.5
4/20/2018 0:36	4.4	19	5.7	3.1	0.122	2.6
4/20/2018 0:37	4.7	18.7	5.6	3.1	0.133	2.5
4/20/2018 0:38	4.3	18.9	5.8	3	0.1236	2.5
4/20/2018 0:39	3.2	18.4	5.5	3	0.1298	2.3
4/20/2018 0:40	3.6	18.8	5.5	3	0.1303	2.4
4/20/2018 0:41	2.8	18.4	5.5	3	0.1289	2.4
4/20/2018 0:42	3.9	18.8	5.6	3	0.1383	2.5
4/20/2018 0:43	3.7	18.7	5.8	3	0.122	2.5
4/20/2018 0:44	3.8	18.8	5.8	3	0.1383	2.5
4/20/2018 0:45	3.1	18.6	5.7	3.1	0.1303	2.3
4/20/2018 0:46	2.6	18.6	5.6	3.1	0.1335	2.2
4/20/2018 0:47	4.5	18.5	5.8	3.1	0.1218	2.4
4/20/2018 0:48	4	18.5	5.7	3.1	0.1288	2.5
4/20/2018 0:49	2.4	18.6	5.5	3.1	0.1379	2.5
4/20/2018 0:50	2.9	18.5	5.6	3.1	0.1233	2.5
4/20/2018 0:51	4.4	18.6	5.5	3.1	0.149	2
4/20/2018 0:52	3.7	18.3	5.4	3.1	0.122	1.8
4/20/2018 0:53	4.7	18.2	5.5	3.1	0.1371	1.8
4/20/2018 0:54	2.9	18.4	5.6	3.2	0.1258	1.8
4/20/2018 0:55	4.1	18.3	5.5	3.2	0.1328	1.9
4/20/2018 0:56	4.4	18.3	5.6	3.2	0.1291	1.8
4/20/2018 0:57	3.8	18.5	5.8	3.2	0.1182	1.8
4/20/2018 0:58	3.2	18.3	6	3.2	0.1322	2
4/20/2018 0:59	3.9	18.5	6.1	3.2	0.1206	2
4/20/2018 1:00	4.8	18.5	6.3	3.1	0.1309	2
4/20/2018 1:01	3.2	18.5	6.1	3.1	0.1215	2
4/20/2018 1:02	3.2	18.3	6	3.1	0.1339	2.2
4/20/2018 1:03	2.9	18.4	6	3	0.1257	2.4
4/20/2018 1:04	3.6	18.1	5.7	3	0.1281	2.5
4/20/2018 1:05	2	18.3	5.6	3	0.1317	2.5

4/20/2018 1:06	3.4	18.2	5.6	3	0.1198	2.4
4/20/2018 1:07	2.1	18.3	5.7	3.1	0.1388	2.3
4/20/2018 1:08	3.6	18.4	6.1	3.1	0.1222	1.9
4/20/2018 1:09	3.7	18.2	5.9	3.1	0.1392	1.7
4/20/2018 1:10	2.8	18	5.9	3.1	0.1209	1.6
4/20/2018 1:11	2.5	18	5.7	3.2	0.1339	1.6
4/20/2018 1:12	3.5	18.3	5.7	3.2	0.1305	1.6
4/20/2018 1:13	4.5	18.3	5.8	3.2	0.1224	1.8
4/20/2018 1:14	3.4	18.3	5.7	3.2	0.1373	1.6
4/20/2018 1:15	3.1	18.3	5.8	3.2	0.1286	1.5
4/20/2018 1:16	3.5	18.3	5.9	3.2	0.1339	1.4
4/20/2018 1:17	2.9	18.1	5.9	3.2	0.1267	1.3
4/20/2018 1:18	4	18.1	6	3.3	0.1385	1.4
4/20/2018 1:19	1.9	18.4	6.3	3.2	0.1358	1.5
4/20/2018 1:20	2.8	18.1	6.6	3.2	0.132	1.4
4/20/2018 1:21	1.6	18.2	6.6	3.2	0.134	1.4
4/20/2018 1:22	3	18.3	7	3.1	0.1281	1.3
4/20/2018 1:23	3.5	18.2	6.9	3.1	0.14	1.3
4/20/2018 1:24	3.9	18	6.6	3.1	0.1226	1.4
4/20/2018 1:25	3	18	6.7	3.1	0.1368	1.4
4/20/2018 1:26	3.1	18.2	6.3	3.1	0.1256	1.5
4/20/2018 1:27	2.5	17.8	6.3	3.1	0.1327	1.7
4/20/2018 1:28	2.8	18	6.5	3.1	0.133	1.8
4/20/2018 1:29	3.6	17.8	6.8	3.2	0.1277	1.9
4/20/2018 1:30	4.8	17.7	6.6	3.2	0.1419	1.9
4/20/2018 1:31	4	17.9	6.7	3.2	0.128	2
4/20/2018 1:32	2.1	18	6.5	3.3	0.1339	2.1
4/20/2018 1:33	4.5	18	6.7	3.3	0.1288	2
4/20/2018 1:34	2.7	17.8	6.6	3.3	0.1416	2.1
4/20/2018 1:35	3.1	18	6.9	3.3	0.1295	2.2
4/20/2018 1:36	3.4	18	6.7	3.3	0.1236	2.3
4/20/2018 1:37	3	17.7	6.9	3.3	0.1411	1.9
4/20/2018 1:38	2.6	17.9	7	3.3	0.1226	1.8
4/20/2018 1:39	3.6	17.9	7.2	3.3	0.1385	1.9
4/20/2018 1:40	3.2	17.7	7.2	3.3	0.129	2
4/20/2018 1:41	2.1	17.8	6.8	3.3	0.1426	2.1
4/20/2018 1:42	4.2	17.6	7	3.4	0.1379	2.1
4/20/2018 1:43	3.1	17.9	7	3.4	0.1351	2.2
4/20/2018 1:44	2.7	17.6	7.1	3.4	0.1426	2.2
4/20/2018 1:45	2.7	17.8	7.2	3.4	0.1306	2.2
4/20/2018 1:46	2.5	17.6	7.3	3.4	0.1414	2.2
4/20/2018 1:47	3.5	17.6	8.2	3.4	0.1252	2.2
4/20/2018 1:48	3.7	17.6	8.5	3.4	0.1405	2.3
4/20/2018 1:49	4	17.5	7.8	3.4	0.1245	2.4
4/20/2018 1:50	2.5	17.5	7.6	3.4	0.1361	2.4
4/20/2018 1:51	3.4	17.6	7.4	3.4	0.1335	2.3
4/20/2018 1:52	2.8	17.6	8	3.4	0.1326	2.3

4/20/2018 1:53	3.2	17.7	8.6	3.4	0.1421	2.1
4/20/2018 1:54	3.5	17.7	9.3	3.4	0.1327	2
4/20/2018 1:55	2.6	17.7	8.3	3.4	0.1445	1.9
4/20/2018 1:56	2.7	17.5	8	3.4	0.1286	1.9
4/20/2018 1:57	2.9	17.6	8.7	3.4	0.146	1.9
4/20/2018 1:58	3.5	17.7	8.2	3.5	0.1375	1.8
4/20/2018 1:59	2.5	17.7	8.2	3.5	0.1379	1.7
4/20/2018 2:00	2.5	17.5	7.8	3.4	0.1425	1.7
4/20/2018 2:01	2.6	17.7	8	3.4	0.1289	1.6
4/20/2018 2:02	4.3	17.8	8.1	3.5	0.146	1.4
4/20/2018 2:03	2.5	17.7	8.2	3.5	0.1331	1.5
4/20/2018 2:04	2.7	17.5	7.8	3.5	0.1398	1.5
4/20/2018 2:05	4.4	17.5	7.8	3.5	0.1417	1.6
4/20/2018 2:06	2.3	17.7	7.9	3.5	0.1336	1.6
4/20/2018 2:07	2.3	17.8	7.9	3.5	0.1443	1.6
4/20/2018 2:08	2.7	17.6	8.1	3.5	0.132	1.5
4/20/2018 2:09	2.1	17.5	8.1	3.5	0.1425	1.5
4/20/2018 2:10	2.4	17.9	8.1	3.6	0.133	1.5
4/20/2018 2:11	4.2	17.5	8.1	3.6	0.1402	1.5
4/20/2018 2:12	3	17.5	7.8	3.6	0.141	1.5
4/20/2018 2:13	4	17.5	7.9	3.6	0.1361	1.4
4/20/2018 2:14	4.2	17.4	7.9	3.6	0.1409	1.4
4/20/2018 2:15	3.4	18	7.8	3.6	0.134	1.4
4/20/2018 2:16	3.6	17.7	7.9	3.7	0.1397	1.4
4/20/2018 2:17	4.3	17.6	8.2	3.7	0.1394	1.3
4/20/2018 2:18	3.9	17.6	8	3.7	0.1423	1.4
4/20/2018 2:19	3.7	17.7	8.6	3.7	0.1573	1.4
4/20/2018 2:20	3.1	17.4	9.3	3.7	0.1442	1.5
4/20/2018 2:21	3.8	17.7	9	3.7	0.1527	1.5
4/20/2018 2:22	3.5	17.6	9.3	3.7	0.1489	1.5
4/20/2018 2:23	2.5	17.7	8.5	3.7	0.1563	1.4
4/20/2018 2:24	1.9	17.2	8.7	3.7	0.14	1.4
4/20/2018 2:25	2.7	17.6	8.5	3.6	0.1552	1.4
4/20/2018 2:26	2.5	17.6	9.1	3.6	0.1497	1.4
4/20/2018 2:27	3.9	17.7	10.3	3.6	0.162	1.3
4/20/2018 2:28	3.8	17.6	10.7	3.6	0.1662	1.4
4/20/2018 2:29	1.9	17.6	10.3	3.5	0.1583	1.5
4/20/2018 2:30	2.5	17.6	10.4	3.5	0.1693	1.5
4/20/2018 2:31	2.9	17.9	8.8	3.5	0.1489	1.5
4/20/2018 2:32	2.5	17.6	9.6	3.5	0.164	1.5
4/20/2018 2:33	3.1	17.4	8.9	3.5	0.145	1.5
4/20/2018 2:34	2.1	17.6	8.9	3.5	0.1527	1.4
4/20/2018 2:35	1.8	17.4	8.7	3.5	0.1534	1.4
4/20/2018 2:36	1.9	17.7	8.5	3.6	0.1423	1.4
4/20/2018 2:37	2.2	17.5	8.4	3.6	0.1491	1.4
4/20/2018 2:38	3.6	17.6	8.6	3.7	0.1445	1.5
4/20/2018 2:39	3.7	17.8	8.5	3.7	0.1542	1.5

4/20/2018 2:40	3.5	17.4	8.5	3.7	0.145	1.6
4/20/2018 2:41	2.2	17.5	8.5	3.8	0.1489	1.7
4/20/2018 2:42	3.7	17.6	8.6	3.8	0.1484	1.7
4/20/2018 2:43	4.8	17.8	8.6	3.8	0.1421	1.7
4/20/2018 2:44	2.8	17.5	8.6	3.8	0.1616	1.8
4/20/2018 2:45	2.7	17.3	8.6	3.8	0.1478	1.9
4/20/2018 2:46	2.8	17.4	8.6	3.8	0.1489	2.1
4/20/2018 2:47	3.5	17.5	8.5	3.8	0.1481	2
4/20/2018 2:48	2.7	17.6	8.5	3.8	0.1565	2.1
4/20/2018 2:49	2.8	17.7	8.5	3.8	0.1578	2.2
4/20/2018 2:50	1.9	17.7	8.6	3.8	0.1459	2.3
4/20/2018 2:51		17.7	8.6	3.9	0.1543	2.3
4/20/2018 2:52		17.7	8.6	3.9	0.1378	2.4
4/20/2018 2:53	12.5	17.3	8.6	3.9	0.1628	2.4
4/20/2018 2:54		17.7	8.5	3.9	0.1466	2.4
4/20/2018 2:55	0.3	17.4	8.7	3.9	0.1492	2.3
4/20/2018 2:56	2.2	17.6	8.5	3.9	0.1621	2.3
4/20/2018 2:57		17.8	8.6	3.9	0.1384	2.4
4/20/2018 2:58	3.5	17.6	8.6	3.9	0.1505	2.5
4/20/2018 2:59	3.1	17.4	8.6	3.9	0.1426	2.5
4/20/2018 3:00	3.7	17.4	8.5	3.9	0.155	2.5
4/20/2018 3:01	2.9	17.5	8.7	3.9	0.1484	2.5
4/20/2018 3:02	2	17.7	8.6	3.9	0.148	2.6
4/20/2018 3:03	3	17.4	8.6	3.9	0.1581	2.6
4/20/2018 3:04	3.8	17.5	8.6	3.9	0.1357	2.5
4/20/2018 3:05	3.7	17.7	8.6	3.9	0.1635	2.5
4/20/2018 3:06	3.7	17.5	8.9	3.9	0.1496	2.5
4/20/2018 3:07	3.5	17.8	9	3.9	0.1549	2.4
4/20/2018 3:08	3.4	17.8	8.7	3.8	0.1392	2.5
4/20/2018 3:09	3	17.6	8.9	3.8	0.1465	2.5
4/20/2018 3:10	4	17.9	9	3.8	0.1532	2.4
4/20/2018 3:11	3.8	17.9	9.7	3.7	0.1503	2.4
4/20/2018 3:12	3.6	18.1	9.9	3.6	0.1628	2.3
4/20/2018 3:13	4.1	18.1	10.2	3.5	0.1476	2.3
4/20/2018 3:14	3.1	18	10.1	3.4	0.1546	2.4
4/20/2018 3:15	2.6	18.2	10.2	3.3	0.1511	2.4
4/20/2018 3:16	3.2	18.3	10.2	3.2	0.1568	2.3
4/20/2018 3:17	3.5	17.6	10.1	3.1	0.1571	2.2
4/20/2018 3:18	2.8	17.6	10	3.1	0.1519	2.4
4/20/2018 3:19	3.2	17.7	8.8	3.1	0.1663	2.3
4/20/2018 3:20	3	17.5	9	3.2	0.1477	2
4/20/2018 3:21	2.6	17.7	9	3.2	0.1601	2
4/20/2018 3:22	2.1	17.7	8.7	3.3	0.1503	2.1
4/20/2018 3:23	2.5	17.6	8.5	3.5	0.1578	2.2
4/20/2018 3:24	2.2	17.5	8.6	3.6	0.1534	2.3
4/20/2018 3:25	2.5	17.5	8.8	3.7	0.1497	2.4
4/20/2018 3:26	2	17.5	8.6	3.8	0.164	2.4

4/20/2018 3:27	3.3	17.6	8.7	3.9	0.1491	2.4
4/20/2018 3:28	3.1	17.4	8.7	3.9	0.1537	2.2
4/20/2018 3:29	2	17.7	8.9	4	0.1522	1.8
4/20/2018 3:30	4.8	17.7	8.9	4	0.1623	1.5
4/20/2018 3:31	2.5	17.5	8.9	4	0.1599	1.4
4/20/2018 3:32	4.9	17.5	8.7	4	0.1522	1.4
4/20/2018 3:33	2.6	17.3	8.6	4.1	0.1591	1.4
4/20/2018 3:34	2.9	17.6	8.8	4.1	0.1428	1.4
4/20/2018 3:35	3.2	17.6	8.8	4.1	0.1578	1.4
4/20/2018 3:36	2.4	17.5	9	4.1	0.1509	1.5
4/20/2018 3:37	1.7	17.6	8.8	4.1	0.1542	1.5
4/20/2018 3:38	3.1	17.3	9	4.2	0.1501	1.5
4/20/2018 3:39	2.6	17.4	8.8	4.2	0.1426	1.5
4/20/2018 3:40	3.6	17.3	8.9	4.2	0.1553	1.6
4/20/2018 3:41	2.6	17.6	9	4.2	0.1507	1.5
4/20/2018 3:42	2.8	17.4	9	4.2	0.1508	1.6
4/20/2018 3:43	4	17.4	9.1	4.3	0.1534	1.6
4/20/2018 3:44	3.8	17.2	9.4	4.3	0.1509	1.7
4/20/2018 3:45	2.3	17.3	9.5	4.2	0.1558	1.8
4/20/2018 3:46	1.8	17.3	9.9	4.2	0.1472	1.9
4/20/2018 3:47	2.3	17.5	9.9	4.2	0.1588	2
4/20/2018 3:48	3.1	17.6	9.9	4.2	0.1498	2
4/20/2018 3:49	2.4	17	9.9	4.2	0.1584	2.2
4/20/2018 3:50	3.7	17.4	10.7	4.2	0.1508	2.3
4/20/2018 3:51	2.2	17.2	10.9	4.1	0.1519	2.4
4/20/2018 3:52	3	17.4	11.3	4	0.1593	2.4
4/20/2018 3:53	3.6	17.8	11.3	4	0.1457	2.5
4/20/2018 3:54	3.4	17.5	11.4	3.9	0.1581	2.5
4/20/2018 3:55	2.5	17.5	11	3.8	0.144	2.6
4/20/2018 3:56	2.8	17.6	10.8	3.7	0.1553	2.6
4/20/2018 3:57	2.6	17.6	9.3	3.7	0.1542	2.6
4/20/2018 3:58	3.2	17.5	9.5	3.7	0.1447	2.2
4/20/2018 3:59	3.7	17.2	9.4	3.7	0.1602	1.3
4/20/2018 4:00	2.1	17.4	9.3	3.8	0.1498	1.3
4/20/2018 4:01	3.7	17.7	9.1	3.9	0.1468	1.3
4/20/2018 4:02	3.8	17.3	9	4	0.1491	1.3
4/20/2018 4:03	3	17.7	9	4.1	0.153	1.4
4/20/2018 4:04	2.3	17.5	9	4.2	0.1533	1.5
4/20/2018 4:05	2.5	17.7	9	4.2	0.1538	1.7
4/20/2018 4:06	1.7	17.6	8.9	4.2	0.1579	1.9
4/20/2018 4:07	3.7	17.4	8.9	4.2	0.149	2
4/20/2018 4:08	3.5	17.7	8.8	4.2	0.1561	2.2
4/20/2018 4:09	1.6	17.6	8.8	4.2	0.15	2.2
4/20/2018 4:10	3.9	17.4	8.8	4.2	0.1602	2.4
4/20/2018 4:11	3.5	17.8	8.9	4.3	0.1552	2.3
4/20/2018 4:12	3.3	17.4	8.8	4.3	0.1607	2.2
4/20/2018 4:13	2.3	17.6	8.7	4.3	0.1641	1.7

4/20/2018 4:14	3.4	17.5	8.8	4.3	0.1465	1.7
4/20/2018 4:15	2.5	17.4	8.7	4.3	0.1557	1.9
4/20/2018 4:16	2.5	17.5	8.7	4.3	0.1438	2.1
4/20/2018 4:17	1.7	17.6	8.7	4.3	0.1536	2.2
4/20/2018 4:18	3.6	17.3	8.7	4.3	0.1505	2.3
4/20/2018 4:19	2.3	17.6	8.6	4.3	0.1569	2.4
4/20/2018 4:20	4	17.9	8.6	4.3	0.159	2.5
4/20/2018 4:21	3.9	17.6	8.6	4.3	0.1523	2.5
4/20/2018 4:22	4.1	17.4	8.6	4.3	0.1544	2.3
4/20/2018 4:23	2.1	17.6	8.5	4.3	0.1469	2.4
4/20/2018 4:24	3.1	17.5	8.6	4.3	0.1544	2.5
4/20/2018 4:25	1.8	17.8	8.5	4.3	0.1473	2.6
4/20/2018 4:26	2.2	17.6	8.6	4.2	0.1527	2.6
4/20/2018 4:27	2.7	17.5	8.5	4.2	0.1514	2.6
4/20/2018 4:28	3.7	17.3	8.5	4.2	0.1442	2.5
4/20/2018 4:29	2.9	17.7	8.6	4.2	0.16	1.6
4/20/2018 4:30	3.2	17.5	8.6	4.2	0.1402	1.5
4/20/2018 4:31	2.9	17.6	8.6	4.2	0.1599	1.6
4/20/2018 4:32	3.2	17.7	8.8	4.1	0.1532	1.7
4/20/2018 4:33	3.5	17.5	8.6	4.1	0.1476	1.6
4/20/2018 4:34	4.1	17.6	8.8	4	0.1474	1.5
4/20/2018 4:35	3.3	17.8	8.8	3.9	0.1388	1.5
4/20/2018 4:36	3.4	18	8.8	3.9	0.1603	1.5
4/20/2018 4:37	3	18	9.1	3.8	0.145	1.6
4/20/2018 4:38	2.7	17.9	9.3	3.6	0.1497	1.8
4/20/2018 4:39	2.9	17.9	9.3	3.5	0.1453	1.9
4/20/2018 4:40	3.2	18	9.2	3.4	0.144	2
4/20/2018 4:41	2.8	17.6	9	3.3	0.1558	2
4/20/2018 4:42	3.2	17.7	9	3.3	0.134	2.1
4/20/2018 4:43	3.2	17.5	8.4	3.3	0.15	2.2
4/20/2018 4:44	4.1	17.3	8.6	3.3	0.1399	2.2
4/20/2018 4:45	3.1	17.5	8.7	3.3	0.1466	2.2
4/20/2018 4:46	2.7	17.7	8.5	3.4	0.1581	2.3
4/20/2018 4:47	2.8	17.5	8.4	3.5	0.1408	2.4
4/20/2018 4:48	2.4	17.5	8.4	3.7	0.1541	2.4
4/20/2018 4:49	3.6	17.4	8.4	3.8	0.14	2.5
4/20/2018 4:50	4.1	17.4	8.5	3.9	0.1557	2.5
4/20/2018 4:51	3.4	17.2	8.5	4	0.146	2.6
4/20/2018 4:52	2.7	17.8	8.5	4	0.1511	2.6
4/20/2018 4:53	3.9	17.5	8.4	4	0.1536	2.6
4/20/2018 4:54	1.9	17.5	8.4	4.1	0.1449	2.7
4/20/2018 4:55	3.5	17.3	8.4	4.1	0.1565	2.6
4/20/2018 4:56	3.5	17.2	8.6	4.1	0.1446	2.7
4/20/2018 4:57	3	17.6	8.5	4.1	0.1563	2.6
4/20/2018 4:58	1.9	17.4	8.4	4.1	0.1481	2.7
4/20/2018 4:59	2.3	17.2	8.4	4.1	0.1467	2.6
4/20/2018 5:00	2.4	17.4	8.5	4.1	0.1568	2.6

4/20/2018 5:01	4.6	17.2	8.6	4.1	0.1328	2.6
4/20/2018 5:02	2.2	17.4	8.6	4.1	0.1564	2.5
4/20/2018 5:03	3	17.1	8.6	4.1	0.144	2.6
4/20/2018 5:04	3.2	17.3	8.6	4.1	0.1621	2.6
4/20/2018 5:05	5.4	17.1	8.5	4.1	0.1541	2.6
4/20/2018 5:06	3.6	17.3	8.6	4.2	0.1493	2.6
4/20/2018 5:07	4.5	17.3	8.7	4.2	0.1614	2.5
4/20/2018 5:08	2.5	17.3	8.7	4.2	0.1473	2.2
4/20/2018 5:09	2.5	17.1	8.9	4.2	0.1538	2.1
4/20/2018 5:10	3.8	17.1	8.9	4.2	0.1535	2.1
4/20/2018 5:11	2.5	17	8.8	4.2	0.1464	2.2
4/20/2018 5:12	3.2	17.2	9.2	4.2	0.1534	2.1
4/20/2018 5:13	3.5	17.1	9.5	4.2	0.1484	2.1
4/20/2018 5:14	1.6	16.9	9.9	4.2	0.1608	2.1
4/20/2018 5:15	2.4	17.3	9.9	4.2	0.1498	2.1
4/20/2018 5:16	2.8	17.1	9.7	4.3	0.1591	2.2
4/20/2018 5:17	2.9	16.9	10	4.3	0.164	2.3
4/20/2018 5:18	4.4	16.5	10.7	4.3	0.1581	2.4
4/20/2018 5:19	2.5	17.1	11.1	4.3	0.1693	2.4
4/20/2018 5:20	3.7	16.9	11.5	4.2	0.1446	2.5
4/20/2018 5:21	2.6	16.8	11.7	4.2	0.1621	2.7
4/20/2018 5:22	3.4	17	11.7	4.2	0.1568	2.7
4/20/2018 5:23	2.7	16.9	11.4	4.2	0.1632	2.7
4/20/2018 5:24	3.1	17.3	9.5	4.2	0.162	2.6
4/20/2018 5:25	3.1	17.2	9.5	4.2	0.15	2.7
4/20/2018 5:26	2.9	17	9.6	4.2	0.1641	2.6
4/20/2018 5:27	2.7	17.1	9.2	4.2	0.1495	2.7
4/20/2018 5:28	3.8	17.2	9	4.3	0.1617	2.6
4/20/2018 5:29	1.7	17.4	8.9	4.3	0.1584	2.7
4/20/2018 5:30	2.6	17.3	9.1	4.4	0.1471	2.7
4/20/2018 5:31	3.5	17.2	9.1	4.4	0.1592	2.7
4/20/2018 5:32	3.2	16.7	9.2	4.4	0.1538	2.7
4/20/2018 5:33	3.5	17.3	9.2	4.4	0.1622	2.7
4/20/2018 5:34	4.5	16.9	9.2	4.5	0.1539	2.6
4/20/2018 5:35	2.7	17.1	9.2	4.5	0.1503	2.6
4/20/2018 5:36	1.6	17.3	9.1	4.5	0.1599	2.7
4/20/2018 5:37	2.9	17.1	9.3	4.5	0.1436	2.7
4/20/2018 5:38	2.9	17.3	9.1	4.5	0.1601	2.6
4/20/2018 5:39	2.5	17.3	9	4.5	0.1471	2.7
4/20/2018 5:40	1.7	17.2	9.2	4.5	0.1532	2.7
4/20/2018 5:41	2.6	17.3	9.1	4.5	0.1561	2.5
4/20/2018 5:42	3.3	17.4	9.1	4.5	0.1478	2
4/20/2018 5:43	3.4	17.2	9.3	4.5	0.1684	2.1
4/20/2018 5:44	3.2	17.3	9.2	4.5	0.1542	2.2
4/20/2018 5:45	2.4	17.3	9.4	4.6	0.1591	2.3
4/20/2018 5:46	4	17.2	9.2	4.6	0.149	2.3
4/20/2018 5:47	1.7	17.3	9.4	4.6	0.1478	2.3

4/20/2018 5:48	2.6	17.1	9.1	4.6	0.1639	2.4
4/20/2018 5:49	2.1	16.9	9.1	4.6	0.1537	2.6
4/20/2018 5:50	2	17.1	9.1	4.6	0.1631	2.6
4/20/2018 5:51	2.8	17.4	9.3	4.6	0.1557	2.1
4/20/2018 5:52	1.8	17.2	9.4	4.6	0.1583	2
4/20/2018 5:53	3.6	17.3	9.3	4.6	0.1621	2.3
4/20/2018 5:54	2.6	17.1	9.7	4.6	0.1496	2.5
4/20/2018 5:55	2.4	17.2	10	4.6	0.1623	2.6
4/20/2018 5:56	3.1	17	10.2	4.6	0.1538	2.1
4/20/2018 5:57	3.4	16.9	10.6	4.6	0.1608	1.9
4/20/2018 5:58	2.6	17.2	10.6	4.5	0.1623	2.3
4/20/2018 5:59	2.8	17.1	10.1	4.5	0.1519	2.4
4/20/2018 6:00	3.4	17	10.6	4.5	0.1742	2.5
4/20/2018 6:01	2.4	16.9	10.7	4.5	0.1594	2.6
4/20/2018 6:02		16.9	11.7	4.6	0.1691	2.6
4/20/2018 6:03		16.8	12.2	4.6	0.1603	2.7
4/20/2018 6:04	3.6	16.8	12.6	4.6	0.1745	2.7
4/20/2018 6:05		17	12.5	4.7	0.1712	2.7
4/20/2018 6:06	3.1	16.9	12.3	4.8	0.1634	2.8
4/20/2018 6:07	3.2	17.1	10.1	4.9	0.1668	2.8
4/20/2018 6:08		17.1	10.1	4.9	0.1588	2.8
4/20/2018 6:09	4.5	17.2	10	5	0.1678	2.8
4/20/2018 6:10		17	9.8	5	0.1597	2.8
4/20/2018 6:11	3.1	17.3	9.8	5.1	0.1687	2.8
4/20/2018 6:12		17.1	9.9	5.1	0.1647	2.8
4/20/2018 6:13		17.3	9.7	5.2	0.1568	2.8
4/20/2018 6:14		17.3	9.8	5.2	0.1649	2.9
4/20/2018 6:15	3.2	17.1	9.7	5.2	0.1596	3
4/20/2018 6:16	3.1	16.9	9.7	5.3	0.1646	2.8
4/20/2018 6:17	3.1	17.1	9.7	5.4	0.1713	2.6
4/20/2018 6:18	2.8	17	9.7	5.5	0.1554	2.7
4/20/2018 6:19	2.7	17	9.8	5.5	0.1699	2.8
4/20/2018 6:20	2.5	17.2	9.7	5.6	0.1516	2.9
4/20/2018 6:21	2.1	17.2	9.8	5.7	0.1692	3
4/20/2018 6:22	2.7	17.2	9.7	5.8	0.1655	2.9
4/20/2018 6:23	2.5	16.8	9.9	5.9	0.1641	2.9
4/20/2018 6:24	3.2	17.1	9.8	6	0.1695	2.8
4/20/2018 6:25	2.8	17.1	9.9	6.1	0.1568	2.9
4/20/2018 6:26	3.9	17	9.8	6.2	0.1732	2.8
4/20/2018 6:27	3.1	17.1	9.8	6.3	0.1575	2.8
4/20/2018 6:28	3	17.1	9.9	6.4	0.1698	2.8
4/20/2018 6:29	2.6	16.9	9.9	6.4	0.1645	2.8
4/20/2018 6:30	2.9	17.2	9.9	6.5	0.1621	2.8
4/20/2018 6:31	2.2	17	9.8	6.6	0.1709	2.8
4/20/2018 6:32	2.9	16.9	9.8	6.6	0.1561	2.9
4/20/2018 6:33	1.9	17.4	9.8	6.7	0.1786	2.9
4/20/2018 6:34	1.5	17.5	9.7	6.7	0.1759	2.9

4/20/2018 6:35	1.7	17.4	9.8	6.8	0.159	2.8
4/20/2018 6:36	3.5	16.9	9.9	6.9	0.1823	2.6
4/20/2018 6:37	0.9	17.2	9.9	6.9	0.1629	2.7
4/20/2018 6:38	3.8	17	9.9	7	0.1747	2.8
4/20/2018 6:39	2.5	17.2	9.8	7	0.1635	2.8
4/20/2018 6:40	2.4	17.1	10.1	7.2	0.1654	2.8
4/20/2018 6:41	2.4	16.9	10.1	7.3	0.1671	2.8
4/20/2018 6:42	3.5	17	10.5	7.4	0.1573	2.9
4/20/2018 6:43	3.8	17	10.9	7.6	0.1606	2.9
4/20/2018 6:44	4.7	17.1	11	7.7	0.155	2.9
4/20/2018 6:45	2.8	16.8	11.2	7.9	0.1766	3
4/20/2018 6:46	3.6	17.2	10.5	8.1	0.1651	3
4/20/2018 6:47	2.3	17.1	10.7	8.2	0.1634	3
4/20/2018 6:48	3.5	17	10.7	8.3	0.1732	2.8
4/20/2018 6:49	3.6	16.9	11.7	8.5	0.1693	2.7
4/20/2018 6:50	2.9	17.2	11.6	8.7	0.1845	2.7
4/20/2018 6:51	2.1	16.6	11.6	8.9	0.1635	2.7
4/20/2018 6:52	3.8	17	11.6	9.1	0.1827	2.7
4/20/2018 6:53	2.6	16.9	11.6	9.2	0.1759	2.7
4/20/2018 6:54	2.3	17	10.8	9.3	0.1648	2.9
4/20/2018 6:55	2.6	17	10.7	9.4	0.1727	2.7
4/20/2018 6:56	2.9	17.3	10.3	9.4	0.1621	2.8
4/20/2018 6:57	3.4	17.2	10.2	9.3	0.1713	2.8
4/20/2018 6:58	1.6	16.9	10.2	9.2	0.1728	2.7
4/20/2018 6:59	3	17.1	10.2	9.1	0.1627	2.6
4/20/2018 7:00	4	17.2	10.2	8.9	0.174	2.5
4/20/2018 7:01	5.1	17.2	10	8.7	0.1581	2.5
4/20/2018 7:02	3.2	17.1	10	8.7	0.1764	2.4
4/20/2018 7:03	3.8	17.3	10	8.6	0.1594	2.3
4/20/2018 7:04	1.7	17.1	10	8.6	0.1811	2.2
4/20/2018 7:05	2.8	17	9.9	8.6	0.1736	1.8
4/20/2018 7:06	3.7	17.4	10	8.7	0.1632	1.6
4/20/2018 7:07	1.7	17.2	10.1	8.7	0.1779	1.7
4/20/2018 7:08	3	17	10	8.7	0.1642	1.8
4/20/2018 7:09	2.9	17.3	10	8.8	0.1793	2
4/20/2018 7:10	3.4	17.1	10.1	8.8	0.1735	2.1
4/20/2018 7:11	3.5	17	10	8.8	0.1712	2.2
4/20/2018 7:12	3.2	17.3	10.2	8.9	0.1764	2.3
4/20/2018 7:13	2.9	17.3	10.1	8.9	0.1677	2.3
4/20/2018 7:14	2.5	17.3	10.1	8.9	0.1862	2.3
4/20/2018 7:15	2.1	17.3	10.1	9	0.1678	2.2
4/20/2018 7:16	4.3	17.3	10	9	0.1824	2.3
4/20/2018 7:17	3.1	17.2	10.2	9	0.1741	2.4
4/20/2018 7:18	3	17.1	10.2	9.1	0.1779	2.4
4/20/2018 7:19	2.8	17.1	10.2	9.1	0.1877	2.4
4/20/2018 7:20	3.1	17	10.5	9.1	0.1704	2.6
4/20/2018 7:21	0.9	17.1	10.6	9.2	0.1854	2.6

4/20/2018 7:22			4.1	17.2	10.8	9.2	0.1747	2.6
4/20/2018 7:23			2.1	16.8	10.5	9.2	0.1834	2.5
4/20/2018 7:24			3.1	17.1	11.1	9.2	0.1821	2.6
4/20/2018 7:25	0.2	-2.2	2.5	17.2	11.6	9.2	0.1862	2.4
4/20/2018 7:26	0.3	-1.3	3.8	17.1	10.6	9.2	0.1816	1.7
4/20/2018 7:27	0.3	-0.7	3.6	17.4	10.5	9.2	0.2031	1.4
4/20/2018 7:28	0.3	-0.1	3.2	17.3	10.4	9.2	0.825	1.4
4/20/2018 7:29	0.3	-0.1	2.6	17.1	10.3	9.2	1.8438	1.4
4/20/2018 7:30	0.3	-0.3	3.7	17.2	10.1	9.2	-0.0324	1.5
4/20/2018 7:31	0.3	-0.2	3.1	17.2	10.1	9.3	-0.0347	1.5
4/20/2018 7:32	0.3	-0.2	4.5	17.1	10.1	9.3	-0.0528	1.4
4/20/2018 7:33	0.3	-0.1	3.3	17.2	10.1	9.3	-0.0404	1.4
4/20/2018 7:34	0.3	-0.4	2.6	17.6	10.2	9.3	-0.0547	1.4
4/20/2018 7:35	0.3	0.3	2.6	16.9	10.2	9.3	-0.0392	1.5
4/20/2018 7:36	0.3	0.3	2.6	17.2	10.2	9.3	0.0072	1.5
4/20/2018 7:37	0.3	-0.6	3.2	17.1	10.2	9.3		1.4
4/20/2018 7:38	0.3	-0.3	2.6	17	10.2	9.3		1.4
4/20/2018 7:39	0.2	0.4	2	17	10.3	9.3		1.4
4/20/2018 7:40	0.2	0.7	3.6	17.2	10.2	9.3		1.4
4/20/2018 7:41	0.2	-0.3	2.5	17.1	10.3	9.3		1.4
4/20/2018 7:42	0.2	-0.1	2.1	17.4	10.1	9.4		1.4
4/20/2018 7:43	0.2	-0.3	4.1	17	10.1	9.4		1.4
4/20/2018 7:44	0.2	0.5	3.5	17.2	10.2	9.5		1.4
4/20/2018 7:45	0.1	0.7	2.7	17.2	10.2	9.5		1.4
4/20/2018 7:46	0.2	0.6	2	17.2	10.2	9.5		1.4
4/20/2018 7:47	0.2	0.3	3.9	17.3	10.4	9.5		1.4
4/20/2018 7:48	0.2	0	3.1	17.3	10.4	9.6		1.4
4/20/2018 7:49	0.2	1.1	3.6	17.1	10.7	9.6	1.3452	1.5
4/20/2018 7:50	0.2	-0.2	3.4	19	10.9	9.6	0.2413	1.4
4/20/2018 7:51	0.2	0.2	2.2	16.2	10.4	9.6	0.2383	1.4
4/20/2018 7:52	0.2	0.3	1.8	16.5	10.4	9.6	0.2374	1.4
4/20/2018 7:53	0.2	-0.1	1	17.2	10.5	9.6	0.28	1.1
4/20/2018 7:54	0.2	-0.2	2	17.2	10.3	9.7	0.3073	0.1
4/20/2018 7:55	0.2	0.6	2.9	17	10.3	9.7	0.2978	0.6
4/20/2018 7:56	0.2	-0.2	3.2	17.5	12	17.5	0.324	1.5
4/20/2018 7:57	0.2	0.2	3.4	20.3	16.2	30.9	0.4619	1.5
4/20/2018 7:58	0.2	0.5	3	25.2	20.3	31.8	0.8331	1.5
4/20/2018 7:59	0.2	0.1	4.3	26.8	22.5	36.4	1.2143	1.6
4/20/2018 8:00	0.2	0.3	3.3	24.4	20.7	35.3	1.1302	1.6
4/20/2018 8:01	0.2	0	2.1	23.2	19.7	34	0.956	1.5
4/20/2018 8:02	0.2	0.4	2.7	23.3	19.7	34.2	0.9427	1.5
4/20/2018 8:03	0.3	1.1	3.5	22.7	19.3	34.2	0.8833	1.5
4/20/2018 8:04	0.2	0.4	2.9	22.5	19	34.1	0.8738	1.5
4/20/2018 8:05	0.2	0.6	3.1	22.1	18.7	34	0.8263	1.5
4/20/2018 8:06	0.2	0.4	2.2	22.3	18.8	33.9	0.8414	1.5
4/20/2018 8:07	0.1	0.3	4	21.9	18.7	33.8	0.8385	1.5
4/20/2018 8:08	0.1	0.5	4	21.7	18.2	33.5	0.7881	1.6

4/20/2018 8:09	0.1	0.5	3.9	21.3	17.9	33.3	0.7948	1.5
4/20/2018 8:10	0.2	0.8	2.4	21.6	17.8	32.8	0.7789	1.5
4/20/2018 8:11	0.2	0.2	3.4	21.3	17.8	32.5	0.7801	1.5
4/20/2018 8:12	0.2	0.2	2.5	20.9	17.6	32.2	0.7607	1.5
4/20/2018 8:13	0.2	0.3	3.6	20.2	16.9	31.7	0.7169	1.5
4/20/2018 8:14	0.1	-0.1	3.7	20	16.6	31.2	0.7121	1.5
4/20/2018 8:15	0.1	0.3	3.2	20	16.6	30.7	0.6914	1.5
4/20/2018 8:16	0.1	1	3.8	20.9	17.3	30.4	0.768	1.5
4/20/2018 8:17	0.1	0.3	2.9	20.7	17.2	30.3	0.7536	1.5
4/20/2018 8:18	0.2	0.1	2.7	20.9	17.2	30.3	0.7666	1.6
4/20/2018 8:19	0.2	-0.1	3.3	20.9	17.2	30.2	0.7718	1.6
4/20/2018 8:20	0.2	0.6	3.5	20.4	17	30.1	0.7411	1.5
4/20/2018 8:21	0.2	-0.3	3.3	20.3	16.8	30.1	0.7393	1.5
4/20/2018 8:22	0.2	0.3	2.9	20.3	16.7	30.3	0.7335	1.5
4/20/2018 8:23	0.2	0.1	4.1	19.8	16.6	30.4	0.7412	1.5
4/20/2018 8:24	0.2	0.1	3	19.9	16.2	30.4	0.7101	1.6
4/20/2018 8:25	0.2	1	2.8	19.8	15.9	30.1	0.6939	1.6
4/20/2018 8:26	0.2	0.3	3.2	19.6	16	29.9	0.6925	1.7
4/20/2018 8:27	0.2	0.8	3.2	19.1	15.9	29.6	0.6633	1.7
4/20/2018 8:28	0.2	0.3	4.3	19.5	15.9	29.3	0.691	1.7
4/20/2018 8:29	0.2	0.4	4.7	19.2	15.7	29	0.6655	1.7
4/20/2018 8:30	0.2	0.5	2.8	19.5	15.7	28.8	0.685	1.7
4/20/2018 8:31	0.2	0.2	2	19.4	15.6	28.6	0.6796	1.9
4/20/2018 8:32	0.2	-0.1	4.1	19.3	15.5	28.4	0.6769	1.9
4/20/2018 8:33	0.2	0.6	3.4	19.5	15.4	28.2	0.6826	1.7
4/20/2018 8:34	0.2	-0.1	3.9	19.5	15.4	28.2	0.6749	1.8
4/20/2018 8:35	0.2	0	4.5	19.2	15.4	28.1	0.6853	2
4/20/2018 8:36	0.2	0	3.6	19.3	15.2	28.1	0.667	2
4/20/2018 8:37	0.1	0.9	3.3	19.1	15.1	28.1	0.6731	1.6
4/20/2018 8:38	0.2	0.2	3.2	19.1	15.1	28	0.6699	1.5
4/20/2018 8:39	0.2	0.1	3	18.9	15	28	0.6622	1.5
4/20/2018 8:40	0.2	0.5	3.5	18.8	15	27.9	0.6651	1.5
4/20/2018 8:41	0.2	0.7	3.4	19.1	14.9	27.8	0.651	1.5
4/20/2018 8:42	0.2	0.5	3.3	18.9	14.8	27.8	0.6642	1.5
4/20/2018 8:43	0.2	-0.2	2.2	19	14.8	27.7	0.6494	1.6
4/20/2018 8:44	0.2	0.9	2.4	18.8	14.8	27.7	0.6632	1.6
4/20/2018 8:45	0.2	0.4	2.7	18.8	14.7	27.6	0.6499	1.6
4/20/2018 8:46	0.2	0.8	3.9	18.9	14.7	27.5	0.6534	1.6
4/20/2018 8:47	0.2	0.5	1.8	18.9	14.6	27.5	0.6653	1.6
4/20/2018 8:48	0.2	0.3	1.1	18.6	14.6	27.4	0.6337	1.6
4/20/2018 8:49	0.2	-0.2	4.3	18.6	14.5	27.4	0.6452	1.6
4/20/2018 8:50	0.1	0.9	3.6	18.8	14.4	27.4	0.6327	1.6
4/20/2018 8:51	0.1	-0.1	3.3	18.6	14.3	27.3	0.6362	1.7
4/20/2018 8:52	0.2	0.2	2.4	18.3	14.3	27	0.6248	1.7
4/20/2018 8:53	0.2	0.1	2	18.4	14.3	26.9	0.6011	1.6
4/20/2018 8:54	0.2	0.4	3.4	18.1	14.3	26.7	0.6078	1.6
4/20/2018 8:55	0.2	0.6	3.1	18.5	14.2	26.5	0.5886	1.6

4/20/2018 8:56	0.2	0.7	2.9	18.3	14.2	26.3	0.597	1.6
4/20/2018 8:57	0.1	0.1	3.2	18.5	14.1	26.2	0.605	1.7
4/20/2018 8:58	0.2	0.3	3.4	18.3	14	26	0.6072	1.9
4/20/2018 8:59	0.2	0.2		18	14	25.8	0.5891	2
4/20/2018 9:00	0.2	0.3		18.5	13.9	25.7	0.5953	2
4/20/2018 9:01	0.2	0.3		18.1	13.9	25.6	0.6127	1.9
4/20/2018 9:02	0.2	-0.2	2	18.2	13.8	25.6	0.5927	2
4/20/2018 9:03	0.2	-0.2	3.3	18.2	13.7	25.6	0.599	1.9
4/20/2018 9:04	0.2	0		18.2	13.7	25.5	0.5919	2
4/20/2018 9:05	0.2	0.2		18.5	13.7	25.4	0.5887	2
4/20/2018 9:06	0.2	0.7	2.2	18.2	13.7	25.3	0.5927	2
4/20/2018 9:07	0.2	0.8	3.9	18.2	13.7	25.2	0.5821	2
4/20/2018 9:08	0.2	0.1		17.6	14.8	24.9	0.534	2
4/20/2018 9:09	0.4	0.2	3.8	17.5	16.1	24.1	0.4894	2.2
4/20/2018 9:10	0.5	0.8	4.5	17.5	16.8	23.1	0.4588	1.9
4/20/2018 9:11	0.6	0.7	3.8	17.8	16.5	22.1	0.4597	2
4/20/2018 9:12	0.7	0.8	2.8	17.4	17.1	21	0.4413	2.2
4/20/2018 9:13	0.8	0.7	2.6	17.5	17.1	19.9	0.4471	2.3
4/20/2018 9:14	0.8	0.8	3.1	17.4	17.2	18.7	0.4199	2.4
4/20/2018 9:15	0.8	1.4	2.4	17.6	17.7	17.5	0.4169	2.4
4/20/2018 9:16	0.9	1.1	3.1	17.4	17.9	16.2	0.398	2.4
4/20/2018 9:17	0.9	1.2	2.9	17.2	18	15.4	0.4127	2.4
4/20/2018 9:18	0.8	0.9	2.4	17.4	18	14.9	0.4033	2.5
4/20/2018 9:19	0.9	1	3.3	17.6	18.3	14.3	0.3753	2.5
4/20/2018 9:20	1.1	1.3	2.2	17.5	18.7	13.5	0.3652	2.5
4/20/2018 9:21	1.4	1.7	2	17.6	18.9	12.8	0.3511	2.6
4/20/2018 9:22	1.5	2.1	3.8	18	18.9	12	0.3606	2.6
4/20/2018 9:23	1.5	2.1	3.2	17.7	18.9	11.4	0.3457	2.6
4/20/2018 9:24	1.5	1.4	1	17.4	18.9	10.8	0.3633	2.5
4/20/2018 9:25	1.3	1.1	2.7	17	18.8	10.5	0.3699	2.5
4/20/2018 9:26	1	1.4	1.8	17.2	18.8	10.1	0.3598	2.5
4/20/2018 9:27	0.8	0.5	2.8	16.9	18.8	9.8	0.3762	2.4
4/20/2018 9:28	0.7	0.8	2.8	19.2	22.9	18.4	0.4164	2.4
4/20/2018 9:29	0.6	1	3.3	25.8	30.3	36.9	0.6755	2.5
4/20/2018 9:30	0.5	0.6	3.3	33	33.5	46.2	0.918	2.6
4/20/2018 9:31	0.4	0.4	3.6	38.2	35.7	53.2	1.394	2.7
4/20/2018 9:32	0.4	0.6	4.2	39.4	37	53.2	1.7435	2.8
4/20/2018 9:33	0.4	0.4	3.8	40.8	38.2	60.4	1.9548	2.9
4/20/2018 9:34	0.4	0.2	3.7	39.6	37.1	60.6	1.9565	2.9
4/20/2018 9:35	0.4	0.2	5	40.4	37.1	60.1	1.9659	3
4/20/2018 9:36	0.4	0.8	3.4	38.5	36.7	59.7	1.9612	3
4/20/2018 9:37	0.4	0.9	2.9	37.6	35.8	59.1	1.9099	3
4/20/2018 9:38	0.3	0.4	4	37	34.9	58.2	1.8505	2.8
4/20/2018 9:39	0.3	0.5	3.5	36.6	34.9	57.7	1.9133	2.7
4/20/2018 9:40	0.2	0.2	3	37.4	35.6	57.1	1.9276	2.6
4/20/2018 9:41	0.2	0.1	3.7	36.8	35.1	56.4	1.8918	2.6
4/20/2018 9:42	0.2	1.2	2.8	35.1	34.1	56	1.8114	2.8

4/20/2018 9:43	0.2	0.1	2.7	35.8	33.7	55.7	1.8264	2.8
4/20/2018 9:44	0.2	0.1	4.5	34.9	33.6	55.3	1.7912	2.7
4/20/2018 9:45	0.2	0.7	3.8	34.1	32.9	54.7	1.7638	2.7
4/20/2018 9:46	0.2	0.6	5.6	34.1	32.6	54.7	1.7562	1.8
4/20/2018 9:47	0.2	0.5	3.6	33.8	33	54.4	1.7958	1.7
4/20/2018 9:48	0.1	0.6	4.7	33.7	32.8	54	1.796	1.9
4/20/2018 9:49	0.1	0.4	5.3	33.4	32.1	53.6	1.7469	2
4/20/2018 9:50	0.2	-0.3	3.8	33.6	32.5	53.5	1.796	2.4
4/20/2018 9:51	0.2	0.8	4	33.6	32.3	53.6	1.7793	2.5
4/20/2018 9:52	0.1	0.7	3.4	32.7	31.8	53.4	1.7681	2.7
4/20/2018 9:53	0.1	1.2	5.1	31.1	30.9	53.3	1.6895	2.7
4/20/2018 9:54	0.1	0.5	4	31.2	30.7	53	1.6858	2.7
4/20/2018 9:55	0.1	0.4	3.3	31	30.6	52.7	1.7091	2.8
4/20/2018 9:56	0.2	0.2	3.6	30.6	30.1	52.3	1.6802	2.8
4/20/2018 9:57	0.2	0.8	3.1	30.3	29.7	52.1	1.6697	2.9
4/20/2018 9:58	0.2	0	3.3	29.3	29.2	51.5	1.6183	2.9
4/20/2018 9:59	0.2	0.1	3.2	28.7	28.3	50.7	1.5921	2.9
4/20/2018 10:00	0.2	0.8	3.1	27.2	27.1	50	1.5353	3
4/20/2018 10:01	0.2	0	3.8	27	26.6	49.1	1.5184	3
4/20/2018 10:02	0.2	0.1	3.8	26.6	26.2	48.1	1.4718	2.9
4/20/2018 10:03	0.2	-0.2	3.9	27	26.7	47.4	1.5025	3
4/20/2018 10:04	0.2	0.7	3.6	26.2	25.8	46.3	1.4594	2.7
4/20/2018 10:05	0.2	-0.4	2.3	27.4	26.4	45.6	1.5032	1.9
4/20/2018 10:06	0.2	0.6	3.4	27.2	26.4	45	1.5131	2.1
4/20/2018 10:07	0.2	0.7	3.1	25.5	25.5	44.6	1.4486	2.3
4/20/2018 10:08	0.2	0.7	3	26.8	25.8	44.2	1.499	2.5
4/20/2018 10:09	0.2	0.5	4.4	26	25.4	43.9	1.4688	2.6
4/20/2018 10:10	0.2	0.7	3.4	25.8	25.1	43.9	1.4455	2.7
4/20/2018 10:11	0.2	0.7	4	26.2	25.1	43.7	1.4658	2.8
4/20/2018 10:12	0.2	0.5	3.1	26	25.1	43.7	1.4618	2.8
4/20/2018 10:13	0.2	0	4.1	25.6	24.9	43.7	1.46	2.6
4/20/2018 10:14	0.2	0.7	3	25.7	24.8	43.6	1.4678	2.6
4/20/2018 10:15	0.2	0.4	3.5	25.1	24.5	43.3	1.4503	2.6
4/20/2018 10:16	0.2	0.1	3.9	25.5	24.2	43.2	1.4613	2.3
4/20/2018 10:17	0.2	0.5	3.8	25	24.1	43.1	1.4262	1.9
4/20/2018 10:18	0.2	0.3	3.3	24.8	23.7	42.9	1.4305	2.1
4/20/2018 10:19	0.1	0.8	3.1	24.7	23.5	42.7	1.4153	2.3
4/20/2018 10:20	0.1	0.4	3.4	24.4	23.3	42.4	1.4018	2.4
4/20/2018 10:21	0.1	0.4	2.5	24.7	23.2	42.1	1.4118	2.5
4/20/2018 10:22	0.2	0.6	3.9	24.4	23.1	41.8	1.392	2.4
4/20/2018 10:23	0.2	1	3.9	24.8	23	41.4	1.418	2.4
4/20/2018 10:24	0.2	-0.1	4.4	24.3	22.9	41.3	1.3897	2.6
4/20/2018 10:25	0.2	0.1	2.9	24.1	22.7	41	1.4022	2.5
4/20/2018 10:26	0.2	0.9	3.3	23.7	22.2	40.8	1.3615	2.6
4/20/2018 10:27	0.1	1	4	23.9	22.2	40.7	1.3758	2.5
4/20/2018 10:28	0.1	0.4	3.7	23.8	22	40.5	1.3627	2.5
4/20/2018 10:29	0.1	0.2	3.7	23.2	21.6	40.4	1.3213	2.4

4/20/2018 10:30	0.1	0.7	4	23.2	21.3	40.1	1.3342	2.4
4/20/2018 10:31	0.1	0.5	3.4	23.1	21.2	39.8	1.3115	2.4
4/20/2018 10:32	0.1	0.3	3.9	23	20.9	39.3	1.2985	2.3
4/20/2018 10:33	0.1	0.3	1.9	22.7	20.5	38.8	1.258	2.2
4/20/2018 10:34	0.2	0.2	2.5	22	19.8	38.1	1.2282	2.3
4/20/2018 10:35	0.2	0.6	3.3	21.9	19.5	37.5	1.2077	2.3
4/20/2018 10:36	0.2	0.6	3	22.4	19.7	36.8	1.2097	2.3
4/20/2018 10:37	0.3	0.7	3	22.6	19.8	36.2	1.247	2.3
4/20/2018 10:38	0.3	0.2	1.8	22.5	19.7	35.9	1.2347	2.3
4/20/2018 10:39	0.2	0.6	3.1	22.1	19.4	35.5	1.2347	2.5
4/20/2018 10:40	0.2	0.8	3	21.7	19.2	35	1.1903	2.5
4/20/2018 10:41	0.2	0.4	2.4	21.9	18.9	34.6	1.193	2.3
4/20/2018 10:42	0.2	0.7	2.9	21.8	19.1	34.5	1.209	1.8
4/20/2018 10:43	0.3	0.7	2.8	21.6	18.8	34.3	1.1765	1.6
4/20/2018 10:44	0.3	0.4	3.5	21.4	19.1	34	1.1532	1.6
4/20/2018 10:45	0.4	1	3.6	20.8	19.2	33.4	1.0578	1.7
4/20/2018 10:46	0.6	1.4	4.8	20.5	19.2	32.2	1.0089	1.8
4/20/2018 10:47	0.8	1	2.1	20.1	19.3	30.6	0.9262	2
4/20/2018 10:48	0.9	1.2	2.9	20	19.3	28.8	0.887	2
4/20/2018 10:49	1.1	1.9	3.3	19.5	19.2	27.3	0.8184	2.1
4/20/2018 10:50	1.3	1.6	4.2	19.7	19	25	0.764	2
4/20/2018 10:51	1.5	2.3	4	19.7	19	22.5	0.729	1.9
4/20/2018 10:52	1.6	2.3	3.5	19.3	19	20.5	0.6877	2
4/20/2018 10:53	1.8	2.1	4.2	19.5	18.3	18	0.6403	2
4/20/2018 10:54	2	2.7	3.2	19.5	18.2	15.8	0.5994	2
4/20/2018 10:55	2.2	2.2	4.5	19.5	18.2	13.9	0.5966	2
4/20/2018 10:56	2.3	2.8	4.9	19.7	18.2	12.6	0.5493	2
4/20/2018 10:57	2.5	4	3.7	19.7	17.4	11	0.5242	2
4/20/2018 10:58	2.8	3.5	3.6	19.9	16.9	9.6	0.4945	2
4/20/2018 10:59	3.1	3.7	4.5	19.7	16.9	8.7	0.4894	2
4/20/2018 11:00	3.4	4	4.8	20.2	16.5	7.7	0.4736	2
4/20/2018 11:01	3.6	4.5	4.2	20.4	16.3	6.8	0.4487	2
4/20/2018 11:02	3.9	4.8	4.7	20.6	15.7	6.1	0.4288	2.1
4/20/2018 11:03	4.2	5.5	3.9	21	15.4	5.6	0.4008	1.9
4/20/2018 11:04	4.5	5.7	5.3	20.9	15.1	4.9	0.4081	1.7
4/20/2018 11:05	4.8	5.5	4.2	21.1	14.8	4.5	0.385	1.7
4/20/2018 11:06	4.9	5.1	5.2	21.1	14.7	4.2	0.3881	1.6
4/20/2018 11:07	5.1	5.9	4.5	21.6	14.4	3.8	0.3667	1.7
4/20/2018 11:08	5.4	6.2	3.6	21.8	13.9	3.5	0.355	1.8
4/20/2018 11:09	5.7	6.2	5.1	21.8	13.7	3.3	0.351	1.8
4/20/2018 11:10	6	6.7	5.9	22	13.2	3.1	0.3316	1.8
4/20/2018 11:11	6.3	7.6	4.7	22.6	12.9	2.9	0.3314	1.8
4/20/2018 11:12	6.7	8	4	23	12.5	2.7	0.304	1.7
4/20/2018 11:13	7	7.6	5.7	23.2	12.2	2.6	0.3148	1.8
4/20/2018 11:14	7.2	8.3	4.8	23	12	2.5	0.3034	1.9
4/20/2018 11:15	7.5	8.6	6.6	23.7	11.8	2.4	0.3017	1.9
4/20/2018 11:16	7.9	9.1	6.2	24	11	2.3	0.2774	1.9

4/20/2018 11:17	8	7.5	5.3	23.2	10.9	2.2	0.2817	1.8
4/20/2018 11:18	7.9	8.1	6.2	23	11.1	2.2	0.2986	1.7
4/20/2018 11:19	7.6	7.5	5.4	23.2	11	2.1	0.2819	1.6
4/20/2018 11:20	7.5	7.6	4.9	23.1	11.1	2.1	0.2988	1.6
4/20/2018 11:21	7.4	8.3	6.5	23.4	11.1	2.1	0.2845	1.8
4/20/2018 11:22	7.2	6.6	5.3	22.8	11.4	3.2	0.2999	1.9
4/20/2018 11:23	6.3	5.1	5.8	47.8	33	21.6	0.7527	2
4/20/2018 11:24	4.5	2.7	5.6	103.1	39.7	35.5	1.7243	2.4
4/20/2018 11:25	2.4	0.3	7.5	131.4	48.7	48.2	2.4055	2.6
4/20/2018 11:26	0.9	0	6.3	148.1	50.2	50.2	2.7525	2.7
4/20/2018 11:27	0.4	-0.1	7.6	157.7	51	52.8	3.0542	2.8
4/20/2018 11:28	0.3	-0.7	7.7	169.7	52.7	52.9	3.4273	2.7
4/20/2018 11:29	0.3	-0.3	7.2	175.6	51	53	3.6107	2.7
4/20/2018 11:30	0.3	0	6.7	163.4	48.8	53	3.5492	2.7
4/20/2018 11:31	0.3	-0.4	7.4	167.9	47.7	52.9	3.6672	2.8
4/20/2018 11:32	0.2	-0.8	5.2	166	48.9	53	3.8835	2.9
4/20/2018 11:33	0.2	-0.4	7.3	153.2	45.7	52.6	3.6937	2.8
4/20/2018 11:34	0.3	0.5	6.4	162.9	46.6	52.6	3.8953	2.8
4/20/2018 11:35	0.3	0.1	6.5	152.5	45.3	52.5	3.8273	2.9
4/20/2018 11:36	0.2	-0.3	6.8	161.6	46.1	52.3	4.0572	3
4/20/2018 11:37	0.2	-0.1	5.8	145.7	44.7	52.2	3.9023	3
4/20/2018 11:38	0.3	-0.1	7.6	143.2	42.2	51.8	3.795	3
4/20/2018 11:39	0.3	0.2	6.7	135.8	41.5	51.5	3.7773	3
4/20/2018 11:40	0.3	-0.1	5.6	134.8	41.5	50.9	3.8412	2.9
4/20/2018 11:41	0.3	0.4	5.9	128.2	40.2	50.6	3.7343	2.6
4/20/2018 11:42	0.3	0.1	5.6	123.2	38.6	50.1	3.6283	2.7
4/20/2018 11:43	0.3	-0.2	6.4	122.7	38.4	49.4	3.6852	2.9
4/20/2018 11:44	0.2	0	5	118.8	38.3	48.9	3.6477	3
4/20/2018 11:45	0.2	0.4	6.5	115.5	37.1	48.2	3.5892	3.1
4/20/2018 11:46	0.2	0.6	5.4	113.4	36.4	47.6	3.5415	3
4/20/2018 11:47	0.3	0.5	7.6	107.1	35	47	3.4445	2.8
4/20/2018 11:48	0.3	1	6.1	105.6	34.2	46.4	3.3948	2.4
4/20/2018 11:49	0.3	0.6	6.3	102.9	33.9	45.8	3.4238	2.4
4/20/2018 11:50	0.3	0.1	5.1	99.5	32.9	45.2	3.3413	2.4
4/20/2018 11:51	0.2	0.2	3.9	100.8	32.8	44.8	3.3783	2.5
4/20/2018 11:52	0.2	0.4	5.4	101.5	32.5	44.5	3.3742	2.6
4/20/2018 11:53	0.2	0.1	6.8	98.3	33.1	44.1	3.4362	2.8
4/20/2018 11:54	0.2	0.6	5.5	91.4	30.9	43.4	3.3097	2.8
4/20/2018 11:55	0.2	1.2	4.6	90.3	30	42.9	3.2647	2.8
4/20/2018 11:56	0.2	1	5.5	88.8	30.1	42.6	3.2187	2.6
4/20/2018 11:57	0.1	0.4	5.4	91.6	30.1	42.3	3.3135	2.7
4/20/2018 11:58	0.1	0.4	3.6	89	30	42	3.2908	2.8
4/20/2018 11:59	0.1	0.7	5.2	91.1	30.2	41.9	3.3357	2.8
4/20/2018 12:00	0.1	0.6	5	81.2	28.4	41.4	3.154	2.8
4/20/2018 12:01	0.1	0.7	4.9	80.9	27.8	40.8	3.1817	2.8
4/20/2018 12:02	0.1	0.9	4.8	80.1	27.5	40.3	3.1045	2.8
4/20/2018 12:03	0.1	0.9	4.8	81	27.4	40.1	3.1463	2.8

4/20/2018 12:04	0.1	0.6	4.8	75.7	26.7	39.8	3.0655	2.9
4/20/2018 12:05	0.1	0.4	5.8	79.3	27.1	39.5	3.1478	2.9
4/20/2018 12:06	0.1	0.2	4.2	76.6	26.8	39.2	3.103	2.9
4/20/2018 12:07	0.1	0.8	4.1	74.6	26	38.7	3.0498	3
4/20/2018 12:08	0.1	0.4	5.4	71.7	25.2	38.2	3.0468	2.9
4/20/2018 12:09	0.1	0	4.5	73.2	25.5	38.1	3.0504	2.8
4/20/2018 12:10	0.2	-0.1	4.7	70.9	24.9	37.9	3.0266	2.8
4/20/2018 12:11	0.2	1	4.2	72	24.8	37.7	3.0147	2.9
4/20/2018 12:12	0.1	0.4	3.3	69.2	24.5	37.5	3.007	2.9
4/20/2018 12:13	0.1	0.5	5.3	68	23.7	37.3	2.9348	2.8
4/20/2018 12:14	0.1	0.8	5.4	67.2	23.8	36.9	2.9672	2.9
4/20/2018 12:15	0.2	0.6	4.2	67.6	23.3	36.6	2.9533	2.9
4/20/2018 12:16	0.2	0	4.2	64	23.1	36.4	2.8983	2.8
4/20/2018 12:17	0.2	0.7		64.9	22.8	36.1	2.9337	2.9
4/20/2018 12:18	0.2	1.1	3.7	66.4	22.7	36	2.9486	2.7
4/20/2018 12:19	0.2	0	4.6	65.1	22.5	35.8	2.9465	2.7
4/20/2018 12:20	0.2	0.3		60.4	21.7	35.4	2.8043	2.8
4/20/2018 12:21	0.2	0.4		63.6	21.7	35.2	2.8917	2.8
4/20/2018 12:22	0.1	0	3.9	62.5	21.8	35.1	2.8938	2.9
4/20/2018 12:23	0.1	-0.1	4.4	59.9	21	34.9	2.8145	2.9
4/20/2018 12:24	0.1	0.9		56.5	20.4	34.6	2.7548	2.9
4/20/2018 12:25	0.2	0.8		54.5	19.6	34.2	2.6641	2.9
4/20/2018 12:26	0.2	0.4		54.5	19.6	33.8	2.7144	2.9
4/20/2018 12:27	0.3	0.3		53.2	19.1	33.2	2.651	2.8
4/20/2018 12:28	0.2	0.8	4.7	53.9	19.3	32.7	2.681	2.9
4/20/2018 12:29	0.2	-0.2	4.4	50.8	18.9	32.3	2.5753	2.9
4/20/2018 12:30	0.2	0.4		49.2	18.4	31.6	2.555	2.8
4/20/2018 12:31	0.2	1.1		49.5	18.3	31	2.5386	2.8
4/20/2018 12:32	0.2	1.1	5.3	50.5	18.2	30.5	2.5486	2.5
4/20/2018 12:33	0.2	1	5.2	51.4	18.5	30.3	2.6118	2.2
4/20/2018 12:34	0.2	0.7	4.2	48.9	18.2	30	2.5588	2.5
4/20/2018 12:35	0.2	1.2	4.9	49.7	17.7	29.7	2.5614	2.8
4/20/2018 12:36	0.1	0.8	4.3	48.3	17.5	29.5	2.5307	2.9
4/20/2018 12:37	0.1	0.5	4.1	48.3	17.6	29.4	2.5753	2.9
4/20/2018 12:38	0.2	0.6	4.5	46.7	16.8	29.2	2.5189	2.9
4/20/2018 12:39	0.2	0.2	4.6	46.8	16.8	29.1	2.5318	3
4/20/2018 12:40	0.2	0.3	2.8	47	16.8	29	2.5258	3
4/20/2018 12:41	0.2	0.1	1.7	45.8	16.7	28.7	2.4698	3
4/20/2018 12:42	0.2	0.9	3.2	43.8	16.7	28.4	2.4347	3
4/20/2018 12:43	0.2	1.5	3.8	44.3	16.2	28.1	2.4037	3.1
4/20/2018 12:44	0.3	1.2	4.5	40.1	16.5	27.4	2.2208	3
4/20/2018 12:45	0.5	1.4	3.9	34.5	16.7	24.5	2.0274	2.8
4/20/2018 12:46	1.5	6.8	3.7	21.2	12.4	9.7	1.1143	2.7
4/20/2018 12:47	3.3	8.6	4.5	21.3	9.6	5	0.761	2.6
4/20/2018 12:48	5.3	9.7	4.9	21.8	8.6	3.5	0.6348	2.3
4/20/2018 12:49	6.6	9.8	4.7	23.7	8.8	3.3	0.6373	2.4
4/20/2018 12:50	6.9	8.2	4	26.4	10.4	3.3	0.8344	2.4

4/20/2018 12:51	6.5	7.6	5.4	25.2	12.5	3.6	0.9658	2.3
4/20/2018 12:52	6.6	9	4.7	23.9	10	3.6	0.7114	2.4
4/20/2018 12:53	6.9	9.3	5.5	23.1	10.4	3.7	0.728	2.5
4/20/2018 12:54	7.4	9.5	4.8	24.8	10.6	3.8	0.7703	2.6
4/20/2018 12:55	7.5	10.3	5.4	23.9	9.7	3.8	0.656	2.4
4/20/2018 12:56	7.8	11.7	4.5	23.9	8.8	3.8	0.5979	2.3
4/20/2018 12:57	8.4	10.7	5.3	24.9	8.5	3.7	0.6133	2.3
4/20/2018 12:58	8.4	9.1	3.8	25.9	8.5	3.7	0.7469	2.5
4/20/2018 12:59	8	8.5	4.6	26.1	9.7	3.5	0.7504	2.5
4/20/2018 13:00	7.3	9.1	5.6	25.8	11	3.4	0.7713	2.5
4/20/2018 13:01	7.2	8.9	6.4	25.5	10.3	3.3	0.7143	2.6
4/20/2018 13:02	7.3	9.8	4.7	25.5	10	3.3	0.6936	2
4/20/2018 13:03	7.7	9.7	5.4	25.7	9.3	3.2	0.6421	1.7
4/20/2018 13:04	7.9	10.9	4.7	25.6	9.4	3.2	0.6496	1.9
4/20/2018 13:05	8.2	10.7	5.6	25.3	8.8	3.2	0.5941	2.1
4/20/2018 13:06	8.3	10.8	3.9	25.3	8.5	3.1	0.5646	2.1
4/20/2018 13:07	8.9	11.1	7	25.4	7.9	3	0.5416	2.1
4/20/2018 13:08	9.3	11.2	4.6	25.3	7.6	2.9	0.5066	2.4
4/20/2018 13:09	9.4	11.5	6.3	25	7.6	2.8	0.509	2.5
4/20/2018 13:10	9.7	13.1	6.6	25.5	6.8	2.7	0.4511	2.6
4/20/2018 13:11	10.1	12.4	7.3	25.6	6.6	2.5	0.4402	2.6
4/20/2018 13:12	10.5	11.8	5.2	25.5	6.6	2.5	0.4473	2.7
4/20/2018 13:13	10.7	12.6	6.5	25.6	6.4	2.4	0.4158	2.8
4/20/2018 13:14	11	13.2	6.9	26	6.1	2.3	0.4076	2.8
4/20/2018 13:15	11.5	14.4	5.7	26.8	5.6	2.2	0.3563	2.8
4/20/2018 13:16	11.9	14.2	6.2	26.3	5.6	2.1	0.3781	2.7
4/20/2018 13:17	12.2	15.2	10.1	34	5.4	2.1	0.3637	2.8
4/20/2018 13:18	13.3	18.7	11.7	25.7	5.4	2.1	0.342	2.8
4/20/2018 13:19	14.7	18.7	10.9	29	5.1	2	0.3348	2.2
4/20/2018 13:20	16.1	19.1	10	30.4	5	1.9	0.3192	1.4
4/20/2018 13:21	16.6	19.7	11	30.5	4.8	1.9	0.3193	1.4
4/20/2018 13:22	17	19.6	10.9	31.3	4.8	1.8	0.3034	1.4
4/20/2018 13:23	17.5	20	11	31	4.6	1.7	0.2971	1.5
4/20/2018 13:24	17.6	20	12.4	30.8	4.6	1.7	0.2893	1.7
4/20/2018 13:25	17.9	19.8	11.1	31.4	4.5	1.7	0.2707	1.8
4/20/2018 13:26	17.8	19.5	11.4	31.1	4.4	1.6	0.2826	2
4/20/2018 13:27	18.1	20.7	12.2	32.1	4.4	1.6	0.2712	2.1
4/20/2018 13:28	18.2	19.6	11.8	31.6	4.4	1.6	0.2744	2.2
4/20/2018 13:29	18	18.7	10.5	30.3	4.4	1.6	0.2784	2.3
4/20/2018 13:30	17.4	17.9	9.8	29.4	4.4	1.6	0.2671	2.4
4/20/2018 13:31	16.8	17.3	10.1	29.7	4.3	1.6	0.2681	2.2
4/20/2018 13:32	16.5	17	9.8	29.2	4.3	1.6	0.2558	2.2
4/20/2018 13:33	16.2	16.8	9.6	28.7	4.3	1.6	0.2751	2.3
4/20/2018 13:34	15.8	17.2	9.2	28.3	4.2	1.7	0.2481	2.5
4/20/2018 13:35	15.6	17	9.5	28.2	4.2	1.7	0.2561	2.5
4/20/2018 13:36	15.5	16.9	9.7	28.2	4.2	1.7	0.2313	2.6
4/20/2018 13:37	15.2	15.6	9.3	27	4.2	1.7	0.2461	2.7

4/20/2018 13:38	14.8	16.4	9.2	27.7	4.2	1.6	0.2442	2.6
4/20/2018 13:39	14.7	16.1	9.6	27.5	4.1	1.6	0.2428	1.7
4/20/2018 13:40	14.7	15.7	10	27.2	4.1	1.6	0.2405	1.4
4/20/2018 13:41	14.8	16.7	10.8	28	4.2	1.6	0.2199	1.4
4/20/2018 13:42	14.3	13.6	6.7	25.6	4.4	1.6	0.2435	1.5
4/20/2018 13:43	13.4	14.2	7.4	25.8	4.4	1.7	0.2309	1.5
4/20/2018 13:44	13	14.8	8.9	26.4	4.3	1.7	0.2495	1.6
4/20/2018 13:45	12.9	13.6	8	26	4.2	1.7	0.2309	1.7
4/20/2018 13:46	12.9	12.8	8.3	24.7	6.2	3.5	0.2439	1.7
4/20/2018 13:47	11.1	7.8	5.1	36.2	25.7	28.1	0.4921	1.9
4/20/2018 13:48	8	3.9	6.3	90.3	29.4	29.7	1.1848	2.1
4/20/2018 13:49	4.2	1.1	6.7	123.9	39.7	39.8	1.7638	2.4
4/20/2018 13:50	1.7	0.2	7.6	145.7	41.8	40.8	2.1438	2.6
4/20/2018 13:51	0.4	0.4	7	160.6	42	41.8	2.5083	2.8
4/20/2018 13:52	0.2	0.6	8.9	168.3	41.5	41.6	2.7907	2.8
4/20/2018 13:53	0.1	-1.1	6.4	166	40.8	41.5	2.8494	2.9
4/20/2018 13:54	0.1	-0.4	8.1	159.9	39.9	41.3	2.8595	2.9
4/20/2018 13:55	0.1	-0.4	6.8	156.3	39.2	41.1	2.9853	2.9
4/20/2018 13:56	0.1	-0.6	5.8	149.6	38	40.9	3.0288	2.9
4/20/2018 13:57	0.1	0.1	6.8	146.2	37.3	40.7	3.0618	2.6
4/20/2018 13:58	0.1	0.2	6.7	140.8	36.8	40.4	3.0714	2.5
4/20/2018 13:59	0.1	-0.1	5.3	134.1	36.8	40	3.0333	2.5
4/20/2018 14:00	0.1	0	6.9	131.3	35.5	39.5	3.0676	2.5
4/20/2018 14:01	0.1	0.4	5.2	127.5	33.8	39	3.0499	2.5
4/20/2018 14:02	0.1	0.2	5.7	119.9	32.6	38.4	2.9814	2.5
4/20/2018 14:03	0.2	0.6	6.1	109.5	30.9	36.1	2.8416	2.5
4/20/2018 14:04	0.3	0.1	5.2	99.8	28.2	30.4	2.6503	2.4
4/20/2018 14:05	0.3	0.4	5.7	98.1	28	31.3	2.6913	2.4
4/20/2018 14:06	0.4	-0.2	5.9	89.8	26.9	30.1	2.6293	2.4
4/20/2018 14:07	0.4	1.2	6.5	93.9	26.1	28.7	2.6298	2.3
4/20/2018 14:08	0.3	0	6	97.2	27.4	28.9	2.7506	2.4
4/20/2018 14:09	0.4	0.5	6.2	84.9	25.5	29	2.5658	2.4
4/20/2018 14:10	0.4	1.4	6.7	91.2	26	29.2	2.6946	2.4
4/20/2018 14:11	0.4	0.3	5.4	93.2	26.6	29.6	2.794	2.4
4/20/2018 14:12	0.3	0.6	5.7	93.9	27.2	30	2.8973	2.4
4/20/2018 14:13	0.3	0.4	5.7	88	25.6	30.3	3.0203	2.4
4/20/2018 14:14	0.3	0.3	6.6	89.5	25.8	30.7	3.1087	2.3
4/20/2018 14:15	0.3	0.7	5.2	88.2	25.4	31.1	3.0172	2.3
4/20/2018 14:16	0.3	0.3	4.4	88.4	25.5	31.2	2.9342	2.3
4/20/2018 14:17	0.2	0.1	4.8	85.3	25.1	31.4	2.873	2.2
4/20/2018 14:18	0.2	-0.4	5.3	83.9	24.5	31.5	2.8148	2.2
4/20/2018 14:19	0.2	0.2	3.9	82.8	24.1	31.5	2.7417	2.1
4/20/2018 14:20	0.3	0.8	6	83.2	24.2	31.3	2.7743	2.1
4/20/2018 14:21	0.3	0.3	5.7	82	24.2	31.2	2.749	2.1
4/20/2018 14:22	0.2	0.3	5.9	80.7	23.8	31	2.7457	2.1
4/20/2018 14:23	0.2	0.3	5.8	79.7	23.5	30.9	2.7008	2.1
4/20/2018 14:24	0.2	0.1	4.6	80	23.6	30.8	2.7285	2.1

4/20/2018 14:25	0.2	0.5	6.8	78.5	23.3	30.6	2.7008	2.1
4/20/2018 14:26	0.2	0.3	4.7	77.9	22.9	30.5	2.7037	2.1
4/20/2018 14:27	0.3	-0.1	6	76.2	22.5	30.5	2.6697	2.1
4/20/2018 14:28	0.2	0.8	3.5	74	22	30.3	2.6395	2.1
4/20/2018 14:29	0.2	0.5	5.7	73.4	21.7	30.1	2.6372	2.2
4/20/2018 14:30	0.2	0.1	4.2	71	21.2	29.9	2.59	2.2
4/20/2018 14:31	0.2	-0.1	5.4	66.5	20.3	29.6	2.498	2.1
4/20/2018 14:32	0.2	0.1	4.5	66.5	20	29.3	2.4848	2.1
4/20/2018 14:33	0.2	0.5	6.4	63.8	19.5	28.8	2.4655	2.1
4/20/2018 14:34	0.2	0.3	4.7	63.7	19.4	28.5	2.4455	2.1
4/20/2018 14:35	0.3	0.4	5.4	60.3	18.9	28	2.4168	2
4/20/2018 14:36	0.3	0.6	5.7	62.3	18.7	27.5	2.4167	2
4/20/2018 14:37	0.2	0.3	4.3	61.6	19	27.2	2.4693	2
4/20/2018 14:38	0.2	1.2	2.9	60.5	18.5	26.8	2.4072	2
4/20/2018 14:39	0.2	0.3	4.1	59.2	18.3	26.6	2.41	2
4/20/2018 14:40	0.2	0.4	5.2	59.5	17.8	26.4	2.426	2.1
4/20/2018 14:41	0.2	1	5	57.8	17.7	26.3	2.4163	2.1
4/20/2018 14:42	0.2	0.6	5.2	53.5	17	26.1	2.3185	2.1
4/20/2018 14:43	0.2	0.8	4.2	55.5	16.8	25.8	2.3202	2
4/20/2018 14:44	0.3	0.3	6	55.5	17.1	25.7	2.3563	2.1
4/20/2018 14:45	0.3	0.1	3.7	54.9	16.7	25.6	2.333	2.1
4/20/2018 14:46	0.3	1	4.4	54.7	16.6	25.5	2.3605	2.1
4/20/2018 14:47	0.2	0.5	4.9	54.6	16.5	25.3	2.3497	2.1
4/20/2018 14:48	0.2	1.3	4.3	54.5	16.6	25.2	2.371	2.1
4/20/2018 14:49	0.2	0.2	5.6	53.6	16.4	25	2.3405	2.1
4/20/2018 14:50	0.2	0.8	5	53.5	16.1	24.9	2.3325	2.2
4/20/2018 14:51	0.2	0.5	3.9	53.9	16.3	25	2.3283	2.1
4/20/2018 14:52	0.2	0.1	2.5	53.5	16.2	25	2.3318	2.1
4/20/2018 14:53	0.3	0.4	5.5	53.3	16	25	2.3162	2.1
4/20/2018 14:54	0.2	0.4	5.1	52.2	15.8	24.9	2.309	2
4/20/2018 14:55	0.2	0.7	3.9	51.8	15.5	24.8	2.3027	2.1
4/20/2018 14:56	0.2	0.5	3.4	51.1	15.3	24.7	2.2822	2.1
4/20/2018 14:57	0.2	0.3	6	49.8	15	24.6	2.2515	2.1
4/20/2018 14:58	0.2	0.6	3.8	49.5	14.8	24.4	2.2465	2.1
4/20/2018 14:59	0.2	0.8	3.9	48.6	14.7	24.3	2.2352	2
4/20/2018 15:00	0.2	0.5	3.5	45.8	14.7	24	2.1468	2
4/20/2018 15:01	0.2	0.5	2.8	42.3	14.1	23.6	2.0605	2.1
4/20/2018 15:02	0.3	1	3.7	41.5	13	23.1	1.9838	2.1
4/20/2018 15:03	0.3	0.7	3.7	41	12.7	22.6	1.1934	1.9
4/20/2018 15:04	0.3	0.9	3.9	39.9	12.4	22	0.1562	1.3
4/20/2018 15:05	0.3	0.6	3.7	36.7	11.6	21.4	0.1659	0.3
4/20/2018 15:06	0.3	1	3.5	35.1	11.1	20.5	0.1533	0
4/20/2018 15:07	0.3	1	4.1	39.8	11.7	19.7	0.1594	0
4/20/2018 15:08	0.5	2.6	3.2	33.6	11.4	19	0.1644	8.8
4/20/2018 15:09	1.8	6.9	4.7	28.6	9.6	17.8	0.1535	25
4/20/2018 15:10	3.6	9.8	5.8	27.3	9.2	16.1	0.1582	25.9
4/20/2018 15:11	5.6	10.5	6.6	27.7	8.6	14.2	0.1492	26.9

4/20/2018 15:12	7	12	6	28	8.5	12.4	0.1606	28.2
4/20/2018 15:13	8.1	12.4	6.5	27.8	7.9	10.5	0.1463	7.9
4/20/2018 15:14	9.1	14	6.3	28	7.4	8.7	0.1501	0.3
4/20/2018 15:15	10	14.4	7.5	28.6	7.1	6.9	0.1337	0.1
4/20/2018 15:16	10.3	12.5	7.1	28	7.5	5.3	0.1509	0.1
4/20/2018 15:17	9.3	9.1		28.6	9.3	4	0.1419	1
4/20/2018 15:18	8.1	8.8		27.4	8.6	3.4	0.1776	1.6
4/20/2018 15:19	7.6	11.3		26.2	8	3.2	0.5512	1.7
4/20/2018 15:20	8	10.2		25.7	7.3	3	0.6023	1.7
4/20/2018 15:21	8.7	11.4	6.6	25.7	7.1	2.9	0.5313	1.6
4/20/2018 15:22					7.3	2.9	0.5332	1.6
4/20/2018 15:23					6.6	2.9	0.4854	1.6
4/20/2018 15:24					6.5	2.9	0.4662	1.6
4/20/2018 15:25					6.2	2.8	0.4284	1.6
4/20/2018 15:26					6.3	2.6	0.4471	1.6
4/20/2018 15:27					5.8	2.5	0.3913	1.6
4/20/2018 15:28					5.6	2.4	0.3789	1.6
4/20/2018 15:29					5.4	2.2	0.3483	1.6
4/20/2018 15:30					5.1	2.1	0.315	1.6
4/20/2018 15:31					4.9	2	0.2952	1.6
4/20/2018 15:32					4.6	1.8	0.2882	1.6
4/20/2018 15:33					4.3	1.7	0.2515	1.6
4/20/2018 15:34					4.1	1.6	0.2472	1.6
4/20/2018 15:35					4	1.5	0.2172	1.5
4/20/2018 15:36					4.2	1.4	0.2334	1.5
4/20/2018 15:37					4.1	1.3	0.2126	1.5
4/20/2018 15:38					4.2	1.2	0.2253	1.6
4/20/2018 15:39					4.8	1.2	0.2383	1.5
4/20/2018 15:40					5	1.2	0.2501	1.6
4/20/2018 15:41					4.8	1.2	0.2659	1.6
4/20/2018 15:42					4.9	1.3	0.2645	1.6
4/20/2018 15:43					4.8	1.4	0.2385	1.6
4/20/2018 15:44					4.8	1.4	0.214	1.6
4/20/2018 15:45					4.9	1.5	0.264	1.6
4/20/2018 15:46					4.8	1.6	0.2803	1.6
4/20/2018 15:47					4.5	1.7	0.2642	1.6
4/20/2018 15:48					4.3	1.7	0.2503	1.6
4/20/2018 15:49					4	1.7	0.2124	1.5
4/20/2018 15:50					3.9	1.6	0.225	1.5
4/20/2018 15:51					3.8	1.6	0.2102	1.5
4/20/2018 15:52			8.8	30.8	3.9	1.6	0.2044	1.5
4/20/2018 15:53			8.3	29.8	4.1	1.6	0.1996	1.5
4/20/2018 15:54			8.7	29	4.2	1.5	0.1879	1.5
4/20/2018 15:55			6.8	27.5	4.3	1.5	0.1984	1.6
4/20/2018 15:56			9.1	27.6	4.3	1.5	0.194	1.6
4/20/2018 15:57			7.6	28.1	4.4	1.5	0.1932	1.6
4/20/2018 15:58			8	28.2	4.3	1.5	0.1673	1.7

4/20/2018 15:59	8	29.3	4.1	1.5	0.1654	1.7
4/20/2018 16:00	9	30	3.9	1.4	0.1674	1.7
4/20/2018 16:01	9.2	30.7	3.9	1.4	0.151	1.6
4/20/2018 16:02	9.1	30.1	3.8	1.4	0.1618	1.7
4/20/2018 16:03	7.5	29.7	3.7	1.3	0.1527	1.7
4/20/2018 16:04	8.8	28.5	3.7	1.3	0.1538	1.7
4/20/2018 16:05	7.7	27.9	3.8	1.3	0.1712	1.7
4/20/2018 16:06	8.5	28.1	3.9	1.2	0.1468	1.8
4/20/2018 16:07	6.6	28.7	3.8	1.2	0.1573	1.9
4/20/2018 16:08	7	28	3.7	1.3	0.1666	2
4/20/2018 16:09	7.9	28.6	3.8	1.3	0.1382	1.9
4/20/2018 16:10	9	28.6	3.8	1.3	0.1647	1.9
4/20/2018 16:11	8.7	29.9	3.6	1.3	0.1547	2.1
4/20/2018 16:12	8.2	30.2	3.4	1.3	0.1388	2.1
4/20/2018 16:13	9	29.7	3.4	1.3	0.1569	2.1
4/20/2018 16:14	8.8	29.7	3.5	1.3	0.1533	2.3
4/20/2018 16:15	8.7	29.5	3.4	1.3	0.1514	2.3
4/20/2018 16:16	7.5	28.8	3.5	1.3	0.1555	2.4
4/20/2018 16:17	7.2	28	3.5	1.3	0.1417	2.5
4/20/2018 16:18	10.4	28.7	3.4	1.3	0.1604	2.3
4/20/2018 16:19	10.2	30.3	3.2	1.3	0.148	1.7
4/20/2018 16:20	9.4	31.9	3.1	1.3	0.1407	1.8
4/20/2018 16:21	9.7	32.6	3	1.3	0.145	2
4/20/2018 16:22	10.1	31.1	3.1	1.2	0.1355	2.2
4/20/2018 16:23	8.9	30.5	3.3	1.2	0.1492	2.2
4/20/2018 16:24	8	28.6	3.4	1.2	0.1558	2.1
4/20/2018 16:25	8.9	27.5	3.5	1.2	0.1485	2.2
4/20/2018 16:26	8.8	28.6	3.6	1.2	0.1563	2.1
4/20/2018 16:27	6.4	26.1	3.6	1.3	0.1388	2.1
4/20/2018 16:28	8.1	26	3.7	1.3	0.154	2.1
4/20/2018 16:29	6.6	26.3	3.6	1.4	0.1497	2
4/20/2018 16:30	6.4	26.4	3.6	1.4	0.1485	2
4/20/2018 16:31	6.5	25.7	3.6	1.5	0.163	2.2
4/20/2018 16:32	7	25.7	3.7	1.5	0.1528	2.3
4/20/2018 16:33	7.6	24.8	3.8	1.6	0.1568	2.4
4/20/2018 16:34	6.5	24.4	3.8	1.6	0.1651	2.5
4/20/2018 16:35	6.5	24.8	3.7	1.7	0.1535	2.5
4/20/2018 16:36	6.5	24.4	3.7	1.7	0.1707	2.5
4/20/2018 16:37	6.5	24.5	3.7	1.8	0.1601	2
4/20/2018 16:38	5	24.1	3.7	1.8	0.1623	2
4/20/2018 16:39	7.3	24.7	3.6	1.9	0.1813	2.1
4/20/2018 16:40	7.8	24.7	3.6	1.9	0.1602	2.2
4/20/2018 16:41	6.5	24.2	3.6	1.9	0.1744	2.3
4/20/2018 16:42	7.9	24.2	3.6	2	0.162	2.5
4/20/2018 16:43	5.8	24.1	3.6	2	0.1684	2.6
4/20/2018 16:44	6.4	23.9	3.6	2	0.1708	2.7
4/20/2018 16:45	6.1	23.4	3.6	2	0.1664	2.8

4/20/2018 16:46	5.9	23.4	3.6	2.1	0.1816	2.7
4/20/2018 16:47	5.9	23.5	3.6	2.1	0.1767	2.1
4/20/2018 16:48	5.7	23.6	3.6	2.1	0.1723	2.1
4/20/2018 16:49	5.2	23.2	3.6	2.1	0.1786	2.3
4/20/2018 16:50	6.5	23	3.6	2.2	0.1616	2.5
4/20/2018 16:51	4	22.8	3.6	2.2	0.1761	2.4
4/20/2018 16:52	6.4	22.6	3.6	2.2	0.1799	2
4/20/2018 16:53	4.7	22.4	3.6	2.2	0.1681	2.1
4/20/2018 16:54	4.8	22.7	3.6	2.3	0.1785	2.4
4/20/2018 16:55	4.8	22.3	3.6	2.3	0.1644	2.6
4/20/2018 16:56	5.3	22.4	3.6	2.3	0.1775	2.5
4/20/2018 16:57	4.5	22.2	3.6	2.3	0.172	2.4
4/20/2018 16:58	5.1	22.1	3.6	2.3	0.1692	2.4
4/20/2018 16:59	4.5	21.8	3.6	2.4	0.1808	2.6
4/20/2018 17:00	6.2	21.9	3.6	2.4	0.1723	2.7
4/20/2018 17:01	5.9	22	3.6	2.4	0.1819	2.6
4/20/2018 17:02	5	22.1	3.6	2.4	0.1744	2.7
4/20/2018 17:03	4.4	21.5	3.6	2.5	0.1779	2.7
4/20/2018 17:04	4.7	21.5	3.4	2.5	0.1823	2.8
4/20/2018 17:05	5.9	21.6	3.4	2.5	0.1667	2.7
4/20/2018 17:06	4.7	21.4	3.4	2.5	0.1858	2.9
4/20/2018 17:07	4.4	21.3	3.4	2.5	0.1744	3.1
4/20/2018 17:08	4.9	21.6	3.4	2.5	0.1847	3.1
4/20/2018 17:09	3.3	21.3	3.4	2.5	0.1844	3.1
4/20/2018 17:10	5.1	21.3	3.4	2.5	0.1719	3.1
4/20/2018 17:11	3.2	21.1	3.4	2.6	0.1932	3.1
4/20/2018 17:12	5.1	20.9	3.4	2.6	0.1802	3.1
4/20/2018 17:13	4.5	20.9	3.4	2.6	0.1893	3.2
4/20/2018 17:14	5	21	3.4	2.6	0.1955	3.2
4/20/2018 17:15	4.4	20.8	3.4	2.6	0.1832	3.2
4/20/2018 17:16	4.9	20.9	3.4	2.7	0.1948	3.2
4/20/2018 17:17	4.5	20.6	3.4	2.7	0.1899	3.3
4/20/2018 17:18	3.7	20.6	3.4	2.7	0.1977	3.3
4/20/2018 17:19	4.5	20.6	3.4	2.7	0.1973	2.9
4/20/2018 17:20	4.3	20.8	3.4	2.7	0.1898	2.8
4/20/2018 17:21	3.8	20.7	3.3	2.7	0.2049	3
4/20/2018 17:22	4.2	20.7	3.4	2.8	0.1905	2.9
4/20/2018 17:23	4.1	20.7	3.4	2.8	0.21	2.9
4/20/2018 17:24	3.9	20.6	3.4	2.8	0.1966	2.8
4/20/2018 17:25	3.9	20.5	3.4	2.8	0.2013	2.9
4/20/2018 17:26	4	20.9	3.4	2.8	0.1985	2.7
4/20/2018 17:27	4	20.9	3.4	2.9	0.1977	2.8
4/20/2018 17:28	4.8	20.6	3.4	2.9	0.2098	3
4/20/2018 17:29	4.5	20.3	3.4	2.9	0.1919	3
4/20/2018 17:30	4.1	20.6	3.4	2.9	0.2052	3.1
4/20/2018 17:31	5	20.5	3.4	2.9	0.1962	3.2
4/20/2018 17:32	3.9	20.2	3.4	2.9	0.1998	3.2

4/20/2018 17:33	2.2	20.3	3.4	2.9	0.195	3.2
4/20/2018 17:34	3	20.6	3.4	2.9	0.1893	3.1
4/20/2018 17:35	4.8	20.6	3.4	2.9	0.2051	2.7
4/20/2018 17:36	3.8	20.4	3.4	2.9	0.1915	2.4
4/20/2018 17:37	4.1	20.5	3.4	2.9	0.2103	2.5
4/20/2018 17:38	3.9	20.5	3.4	3	0.1957	2.8
4/20/2018 17:39	2.9	20.7	3.4	3	0.2004	2.8
4/20/2018 17:40	3.8	20.5	3.4	3	0.1948	2.9
4/20/2018 17:41	3.7	20.6	3.4	3	0.1969	3
4/20/2018 17:42	3.6	20.2	3.4	3	0.198	3.1
4/20/2018 17:43	2.7	20.2	3.4	3	0.1903	3.1
4/20/2018 17:44	3	20.3	3.4	3	0.2039	3.1
4/20/2018 17:45	3	20.7	3.4	3	0.196	3.1
4/20/2018 17:46	4.1	20.5	3.3	3	0.1989	3.1
4/20/2018 17:47	4	20.3	3.3	3	0.1894	3.2
4/20/2018 17:48	3.3	20.5	3.4	3	0.1894	3.2
4/20/2018 17:49	2.7	20.4	3.4	3	0.1974	3.2
4/20/2018 17:50	3.3	20.6	3.4	3	0.1908	3.2
4/20/2018 17:51	3.5	20.2	3.3	3	0.1961	2.6
4/20/2018 17:52	2.8	20.3	3.3	3.1	0.1865	2.9
4/20/2018 17:53	2.9	20.5	3.3	3.1	0.2023	3.1
4/20/2018 17:54	2.6	20.5	3.3	3.1	0.1845	3.2
4/20/2018 17:55	3.9	20.5	3.3	3.1	0.1984	3.3
4/20/2018 17:56	2	20.6	3.3	3.1	0.1919	3.4
4/20/2018 17:57	2.4	20.6	3.3	3.2	0.1947	3.5
4/20/2018 17:58	3.2	20.3	3.3	3.2	0.1971	3.5
4/20/2018 17:59	3.9	20.6	3.4	3.2	0.1809	3.4
4/20/2018 18:00	3.9	20.4	3.3	3.2	0.2013	3.4
4/20/2018 18:01	4.6	20.5	3.4	3.2	0.1823	3.4
4/20/2018 18:02	3.1	20.8	3.3	3.2	0.1893	3.2
4/20/2018 18:03	1.8	20.7	3.3	3.2	0.1912	3.1
4/20/2018 18:04	2.9	20.4	3.3	3.2	0.1837	3.3
4/20/2018 18:05	3.4	20.7	3.2	3.2	0.2002	3.3
4/20/2018 18:06	3.5	20.7	3.2	3.3	0.1797	3.3
4/20/2018 18:07	3.2	20.4	3.2	3.3	0.1927	3.3
4/20/2018 18:08	2.8	20.6	3.2	3.3	0.1823	3.2
4/20/2018 18:09	2.6	20.8	3.2	3.3	0.1909	3.1
4/20/2018 18:10	3.5	20.6	3.2	3.3	0.1791	3.2
4/20/2018 18:11	4.5	20.8	3.2	3.3	0.1845	3.2
4/20/2018 18:12	4.3	20.3	3.2	3.3	0.1889	3.2
4/20/2018 18:13	1.6	20.5	3.2	3.2	0.1858	3.1
4/20/2018 18:14	3.5	20.7	3.2	3.2	0.1949	3.1
4/20/2018 18:15	4	20.8	3.2	3.2	0.1771	3.1
4/20/2018 18:16	4.1	20.6	3.2	3.2	0.1902	3.1
4/20/2018 18:17	2.4	20.5	3.2	3.1	0.1774	3.1
4/20/2018 18:18	2.9	20.7	3.1	3.2	0.1887	3.2
4/20/2018 18:19	4.3	20.9	3.1	3.1	0.1768	3.1

4/20/2018 18:20	4.7	20.9	3.2	3.1	0.1871	3.1
4/20/2018 18:21	2.9	20.8	3.1	3.2	0.1767	3
4/20/2018 18:22	2.3	20.7	3.1	3.2	0.1835	2.9
4/20/2018 18:23	3.1	20.7	3.2	3.2	0.1892	3.1
4/20/2018 18:24	3.7	20.9	3.2	3.3	0.1783	3.1
4/20/2018 18:25	1.9	20.9	3.2	3.3	0.1935	3
4/20/2018 18:26	3.2	20.6	3.2	3.3	0.1793	2.9
4/20/2018 18:27	4.5	20.8	3.2	3.3	0.1953	2.9
4/20/2018 18:28		20.6	3.1	3.3	0.1804	2.9
4/20/2018 18:29	2.6	20.7	3.2	3.3	0.1845	2.8
4/20/2018 18:30	3.1	20.8	3.2	3.3	0.1851	2.8
4/20/2018 18:31	2.5	20.7	3.2	3.3	0.185	2.9
4/20/2018 18:32	2.3	20.7	3.2	3.3	0.1874	2.8
4/20/2018 18:33	3.4	20.9	3.2	3.3	0.1678	2.7
4/20/2018 18:34	3	20.9	3.2	3.3	0.1903	2.8
4/20/2018 18:35	3.9	20.9	3.2	3.3	0.1812	2.9
4/20/2018 18:36	2.8	20.7	3.2	3.3	0.179	2.8
4/20/2018 18:37	3.3	21.2	3.2	3.3	0.1805	2.8
4/20/2018 18:38	3.8	20.7	3.2	3.3	0.1769	2.7
4/20/2018 18:39	4.3	20.6	3.2	3.3	0.1878	2.8
4/20/2018 18:40	3.9	20.9	3.2	3.3	0.1746	2.9
4/20/2018 18:41	3.4	20.9	3.2	3.3	0.1852	2.8
4/20/2018 18:42		20.7	3.2	3.3	0.175	2.5
4/20/2018 18:43		20.8	3.2	3.3	0.1847	2.6
4/20/2018 18:44		20.8	3.2	3.3	0.1833	2.8
4/20/2018 18:45		21	3.2	3.3	0.1873	2.8
4/20/2018 18:46	4.6	21.2	3.2	3.2	0.1815	2.8
4/20/2018 18:47	6.6	21.1	3.2	3.2	0.1764	2.8
4/20/2018 18:48		21	3.2	3.2	0.184	2.4
4/20/2018 18:49	2	21.2	3.2	3.2	0.1749	2.4
4/20/2018 18:50	3.2	20.9	3.2	3.2	0.1848	2.6
4/20/2018 18:51	3.9	21.2	3.2	3.2	0.175	2.7
4/20/2018 18:52	4.8	21	3.2	3.2	0.1905	2.7
4/20/2018 18:53	3.5	21.1	3.2	3.2	0.1726	2.9
4/20/2018 18:54	3.2	21	3.2	3.3	0.1813	3
4/20/2018 18:55	3.3	21	3.2	3.3	0.1791	3.1
4/20/2018 18:56	3.9	21.2	3.2	3.3	0.173	3.1
4/20/2018 18:57	3	20.9	3.2	3.3	0.1842	3.1
4/20/2018 18:58	3.1	21	3.2	3.4	0.1689	3.1
4/20/2018 18:59	2.2	21.1	3.2	3.4	0.1829	2.6
4/20/2018 19:00	3.5	21	3.2	3.4	0.1626	2.7
4/20/2018 19:01	3.1	20.7	3.2	3.4	0.189	3
4/20/2018 19:02	3.2	21	3.2	3.4	0.1709	3.2
4/20/2018 19:03	3.8	21.2	3.2	3.4	0.1795	3.1
4/20/2018 19:04	3.5	21	3.3	3.4	0.1786	3.2
4/20/2018 19:05	4	21.1	3.2	3.4	0.165	3.1
4/20/2018 19:06	2.6	21.3	3.1	3.3	0.1754	2.8

4/20/2018 19:07	1.8	21.1	3.1	3.3	0.1622	2.8
4/20/2018 19:08	3.2	21.1	3.1	3.3	0.18	3
4/20/2018 19:09	3.8	21	3.1	3.3	0.1671	3.2
4/20/2018 19:10	2.6	20.9	3.1	3.3	0.1725	3.2
4/20/2018 19:11	4.2	20.9	3.1	3.3	0.1741	3.2
4/20/2018 19:12	4.5	21.2	3.1	3.3	0.1753	3
4/20/2018 19:13	2.1	21.1	3.1	3.3	0.1784	2.8
4/20/2018 19:14	4.1	21.5	3.1	3.3	0.1651	3
4/20/2018 19:15	4.7	21.2	3.1	3.4	0.1805	3
4/20/2018 19:16	4	21.3	3.1	3.4	0.1702	3
4/20/2018 19:17	4.7	21.2	3.1	3.4	0.1753	2.8
4/20/2018 19:18	3	21	3.1	3.4	0.1704	2.7
4/20/2018 19:19	2.9	21	3.1	3.3	0.1746	2.6
4/20/2018 19:20	4.4	21.1	3.1	3.4	0.1739	2.2
4/20/2018 19:21	3.6	20.9	3.1	3.4	0.1596	2.4
4/20/2018 19:22	3	21.3	3.1	3.4	0.1808	2.6
4/20/2018 19:23	2.8	21.1	3.1	3.4	0.1652	2.7
4/20/2018 19:24	3	21.1	3.1	3.3	0.1784	2.8
4/20/2018 19:25	2.7	20.9	3.1	3.3	0.1655	2.7
4/20/2018 19:26	4.2	21.3	3.1	3.3	0.172	2.8
4/20/2018 19:27	3.9	21.4	3.1	3.3	0.1764	2.9
4/20/2018 19:28	3.3	21	3.1	3.3	0.1643	2.8
4/20/2018 19:29	2.2	21.3	3.1	3.3	0.1692	2.7
4/20/2018 19:30	2.6	21.5	3.1	3.3	0.1594	2.4
4/20/2018 19:31	3	21.1	3.1	3.3	0.1736	2
4/20/2018 19:32	3.4	21.3	3.1	3.3	0.163	2.1
4/20/2018 19:33	4	21.4	3.1	3.3	0.167	2.4
4/20/2018 19:34	2.9	21.4	3.1	3.3	0.1746	2.7
4/20/2018 19:35	4.4	21.5	3.1	3.3	0.1655	2.9
4/20/2018 19:36	4.3	21.2	3.1	3.3	0.1714	2.9
4/20/2018 19:37	4	21.5	3.1	3.3	0.1623	2.9
4/20/2018 19:38	4	21.6	3.2	3.3	0.1798	2.8
4/20/2018 19:39	4.2	21.3	3.1	3.3	0.1592	2.6
4/20/2018 19:40	4.4	21.4	3.2	3.3	0.1807	2.5
4/20/2018 19:41	3.5	21.3	3.1	3.3	0.1653	2.3
4/20/2018 19:42	4.2	21.3	3.2	3.3	0.1699	2.2
4/20/2018 19:43	4	21.3	3.2	3.3	0.169	2.4
4/20/2018 19:44	3.1	21.2	3.2	3.3	0.1645	2.6
4/20/2018 19:45	1.4	21.5	3.2	3.3	0.1702	2.8
4/20/2018 19:46	2.8	21.4	3.2	3.3	0.1557	2.8
4/20/2018 19:47	4	21.2	3.2	3.3	0.1785	2.6
4/20/2018 19:48	2.9	21.2	3.2	3.3	0.1604	2.3
4/20/2018 19:49	3.7	21.4	3.2	3.4	0.1719	2.3
4/20/2018 19:50	3.9	21.2	3.2	3.4	0.1653	2.5
4/20/2018 19:51	3.6	21.3	3.2	3.4	0.1659	2.6
4/20/2018 19:52	2.4	21.4	3.2	3.3	0.1751	2.8
4/20/2018 19:53	3.1	21.2	3.2	3.3	0.1604	2.8

4/20/2018 19:54	3.5	21.6	3.2	3.4	0.1744	2.8
4/20/2018 19:55	2.4	21.3	3.2	3.4	0.1545	2.9
4/20/2018 19:56	3	21	3.2	3.4	0.1785	3
4/20/2018 19:57	2.8	21.2	3.2	3.4	0.166	2.8
4/20/2018 19:58	3.6	21.1	3.2	3.3	0.1683	2
4/20/2018 19:59	2	21.5	3.2	3.4	0.1697	1.9
4/20/2018 20:00	3.8	21.4	3.2	3.4	0.1579	2
4/20/2018 20:01	4.2	21.2	3.2	3.4	0.1716	2.1
4/20/2018 20:02	4	21.3	3.2	3.4	0.16	2.3
4/20/2018 20:03	3.7	21.3	3.2	3.4	0.1727	2.4
4/20/2018 20:04	4.6	21.2	3.2	3.3	0.1618	2.5
4/20/2018 20:05	1.9	21.2	3.2	3.3	0.1638	2.7
4/20/2018 20:06	2.8	21.6	3.2	3.4	0.168	2.8
4/20/2018 20:07	3	21.1	3.1	3.4	0.1683	2.9
4/20/2018 20:08	4.3	21.4	3.1	3.4	0.171	2.9
4/20/2018 20:09	3.8	21.2	3.1	3.4	0.1588	2.9
4/20/2018 20:10	2.4	21.4	3.1	3.4	0.1725	2.8
4/20/2018 20:11	3.2	21.4	3.1	3.4	0.1586	2.8
4/20/2018 20:12	3.4	21.2	3.1	3.4	0.1768	2.7
4/20/2018 20:13	3	21.2	3.1	3.4	0.1642	2.7
4/20/2018 20:14	3	21.5	3.1	3.4	0.1613	2.8
4/20/2018 20:15	4.9	21.4	3.1	3.4	0.174	2.7
4/20/2018 20:16	3.3	21.3	3.1	3.4	0.1581	2.8
4/20/2018 20:17	3.7	21.2	3.1	3.3	0.1734	2.8
4/20/2018 20:18	3.6	21.3	3.1	3.3	0.1637	2.9
4/20/2018 20:19	3.4	21.4	3.1	3.3	0.1687	3
4/20/2018 20:20	3	21.3	3.1	3.3	0.1623	3
4/20/2018 20:21	4.2	21.4	3.1	3.3	0.156	2.9
4/20/2018 20:22	2.6	21.1	3.1	3.3	0.1722	2.8
4/20/2018 20:23	3.8	21.1	3.1	3.3	0.1556	2.8
4/20/2018 20:24	3.2	21.2	3.1	3.3	0.1671	2.9
4/20/2018 20:25	3.8	21.3	3.1	3.3	0.1538	3.1
4/20/2018 20:26	2.8	21.2	3.1	3.3	0.1662	3.2
4/20/2018 20:27	3.1	21.4	3.2	3.3	0.1622	3.1
4/20/2018 20:28	3.5	21.3	3.2	3.3	0.1545	3.1
4/20/2018 20:29	4.1	21.5	3.2	3.3	0.1718	3.1
4/20/2018 20:30	2.8	21.4	3.1	3.3	0.1577	3.1
4/20/2018 20:31	3	21	3.1	3.3	0.173	3
4/20/2018 20:32	3.4	21.4	3.1	3.4	0.1588	2.7
4/20/2018 20:33	3.1	21.5	3.1	3.3	0.1591	2.7
4/20/2018 20:34	4.3	21.4	3.1	3.3	0.1624	2.9
4/20/2018 20:35	3.9	21.4	3.2	3.3	0.1503	3.1
4/20/2018 20:36	3.3	21.5	3.1	3.3	0.1678	3.1
4/20/2018 20:37	2.8	21.4	3.2	3.3	0.1606	3.3
4/20/2018 20:38	3.6	21.5	3.1	3.3	0.1723	3.3
4/20/2018 20:39	3.2	21.4	3.1	3.3	0.1611	2.9
4/20/2018 20:40	2.8	21.6	3.2	3.3	0.17	2.4

4/20/2018 20:41	3.8	21.2	3.2	3.3	0.1614	2.5
4/20/2018 20:42	1.9	21.2	3.2	3.3	0.1681	2.7
4/20/2018 20:43	2.5	21.4	3.2	3.3	0.1698	2.9
4/20/2018 20:44	2.3	21.2	3.2	3.3	0.1604	2.8
4/20/2018 20:45	2.6	21.3	3.2	3.4	0.1699	2.3
4/20/2018 20:46	3.7	21.4	3.2	3.4	0.1604	2.2
4/20/2018 20:47	3.9	21.3	3.2	3.4	0.1734	2.1
4/20/2018 20:48	2.9	21.3	3.2	3.4	0.1624	1.8
4/20/2018 20:49	3.6	21.3	3.2	3.4	0.1682	1.7
4/20/2018 20:50	3	21.4	3.2	3.4	0.1657	1.8
4/20/2018 20:51	3.5	21.3	3.3	3.4	0.1534	1.9
4/20/2018 20:52	2.7	21.2	3.3	3.4	0.1753	2.1
4/20/2018 20:53	2.8	21.4	3.3	3.4	0.1537	2.3
4/20/2018 20:54	3.2	21.4	3.3	3.4	0.1669	2.4
4/20/2018 20:55	3.6	21.3	3.3	3.3	0.1571	2.3
4/20/2018 20:56	1.3	21.4	3.3	3.3	0.1643	2.1
4/20/2018 20:57	3.2	21.2	3.3	3.3	0.1607	2.5
4/20/2018 20:58	3.5	21.4	3.3	3.3	0.1633	2.7
4/20/2018 20:59	5	21.5	3.4	3.3	0.1653	3
4/20/2018 21:00	3.8	21.3	3.4	3.3	0.1502	3.2
4/20/2018 21:01	4.3	21.4	3.4	3.3	0.1694	3.4
4/20/2018 21:02	4	21.4	3.4	3.3	0.1618	3.2
4/20/2018 21:03	2.4	21.1	3.4	3.3	0.1701	2.9
4/20/2018 21:04	3.3	21.3	3.5	3.3	0.1678	3.2
4/20/2018 21:05	4.2	21.1	3.7	3.3	0.1618	3.3
4/20/2018 21:06	2.3	21.1	3.7	3.3	0.1639	3.3
4/20/2018 21:07	3.1	21.2	3.7	3.3	0.162	3.4
4/20/2018 21:08	2.4	21.4	3.5	3.3	0.168	3.2
4/20/2018 21:09	3.9	21	3.4	3.3	0.1546	2.4
4/20/2018 21:10	4.5	21.3	3.6	3.3	0.1743	2.6
4/20/2018 21:11	3.7	21.2	3.6	3.2	0.1565	2.4
4/20/2018 21:12	4.1	21.3	3.6	3.2	0.165	1.9
4/20/2018 21:13	3.8	21.5	3.5	3.3	0.165	2.2
4/20/2018 21:14	3.4	21.2	3.6	3.3	0.155	2.4
4/20/2018 21:15	2.8	21	3.6	3.3	0.1686	2.3
4/20/2018 21:16	3.4	21.3	3.6	3.3	0.1573	2.5
4/20/2018 21:17	3.9	21.3	3.7	3.3	0.1631	2.7
4/20/2018 21:18	2.4	21.2	3.6	3.3	0.1662	2.8
4/20/2018 21:19	3.6	21.1	3.7	3.3	0.1596	2.8
4/20/2018 21:20	4.3	21.2	3.6	3.3	0.17	2.8
4/20/2018 21:21	7.4	21.1	3.7	3.3	0.1595	2.8
4/20/2018 21:22	5.6	21.1	3.7	3.3	0.1695	2.7
4/20/2018 21:23	4	21.1	3.7	3.4	0.1533	2.7
4/20/2018 21:24	2.3	21.2	3.7	3.3	0.1728	2.8
4/20/2018 21:25	2.8	21.2	3.9	3.3	0.1582	2.9
4/20/2018 21:26	3.6	21.4	3.7	3.4	0.1657	3
4/20/2018 21:27	2.4	21.1	3.7	3.3	0.1612	2.9

4/20/2018 21:28	4.1	21.2	3.9	3.3	0.1594	3
4/20/2018 21:29	2.5	21	3.8	3.3	0.1667	2.9
4/20/2018 21:30		21	3.8	3.3	0.1579	2.7
4/20/2018 21:31		21.2	4	3.3	0.1695	2.7
4/20/2018 21:32		21.1	4	3.3	0.1538	2.6
4/20/2018 21:33	4.2	21	4.1	3.3	0.1647	2.6
4/20/2018 21:34		20.7	4	3.3	0.1647	2.9
4/20/2018 21:35	6.9	20.9	4.2	3.3	0.1617	3
4/20/2018 21:36		21.2	4.8	3.3	0.1698	3.2
4/20/2018 21:37	4.2	21.1	4.5	3.3	0.1581	3.2
4/20/2018 21:38	10.5	20.9	4.3	3.3	0.1695	3.2
4/20/2018 21:39	3.8	21	4.2	3.3	0.1634	3.2
4/20/2018 21:40	2.7	20.9	4.3	3.3	0.1685	3.1
4/20/2018 21:41	4.4	20.9	4.6	3.3	0.1586	3.1
4/20/2018 21:42	3.4	20.9	4.3	3.3	0.1734	3
4/20/2018 21:43	3.5	21	4.8	3.3	0.1679	2.5
4/20/2018 21:44	3.3	21	4.6	3.3	0.1582	2.6
4/20/2018 21:45	4.2	21	4.4	3.3	0.1635	2.7
4/20/2018 21:46	2.5	20.9	4.4	3.3	0.1574	2.9
4/20/2018 21:47	3.2	21.1	4.5	3.3	0.168	2.9
4/20/2018 21:48	2.6	21.2	4.4	3.3	0.1602	3
4/20/2018 21:49	3.4	21	4.3	3.3	0.1651	3
4/20/2018 21:50	4	21	4.4	3.3	0.1649	3
4/20/2018 21:51	5.1	21	4.4	3.3	0.1622	3.2
4/20/2018 21:52	3.1	21	4.4	3.3	0.1689	3.1
4/20/2018 21:53	3	21.1	4.5	3.3	0.1632	2.7
4/20/2018 21:54	1.8	21	4.4	3.3	0.1712	3.1
4/20/2018 21:55	4.7	21.4	4.5	3.3	0.1614	3.2
4/20/2018 21:56	4.3	20.9	4.5	3.3	0.1753	3
4/20/2018 21:57	3.3	21	4.5	3.3	0.1642	2.8
4/20/2018 21:58	3.3	21	4.5	3.3	0.1627	3
4/20/2018 21:59	2.6	21.1	4.5	3.3	0.1673	2.9
4/20/2018 22:00	2.7	21.2	4.4	3.3	0.1618	2.9
4/20/2018 22:01	3.1	20.9	4.4	3.3	0.1724	2.9
4/20/2018 22:02	4.8	21.1	4.4	3.3	0.1639	2.9
4/20/2018 22:03	3.8	20.9	4.5	3.3	0.1746	2.9
4/20/2018 22:04	3.2	20.9	4.5	3.3	0.1638	2.9
4/20/2018 22:05	3.8	21	4.5	3.3	0.167	2.8
4/20/2018 22:06	2.4	21.1	4.5	3.3	0.1653	2.7
4/20/2018 22:07	3.9	21.3	4.5	3.3	0.1591	2.7
4/20/2018 22:08	3.8	21.2	4.5	3.3	0.1704	2.4
4/20/2018 22:09	2.9	21	4.4	3.3	0.1577	1.9
4/20/2018 22:10	3.7	20.9	4.3	3.3	0.1672	1.9
4/20/2018 22:11	4.1	21	4.3	3.3	0.1605	2.1
4/20/2018 22:12	4.4	21.1	4.3	3.3	0.1639	2.3
4/20/2018 22:13	2	20.9	4.2	3.3	0.1739	2.4
4/20/2018 22:14	3.2	21.1	4.2	3.3	0.1577	2.6

4/20/2018 22:15	2.5	21	4.2	3.3	0.1778	2.5
4/20/2018 22:16	2.6	21	4.2	3.3	0.1621	2.8
4/20/2018 22:17	2.1	20.9	4.3	3.4	0.169	2.9
4/20/2018 22:18	5	21	4.3	3.4	0.1565	3
4/20/2018 22:19	3.7	20.9	4.2	3.4	0.1645	3.1
4/20/2018 22:20	3.2	21.2	4.2	3.4	0.1737	3.1
4/20/2018 22:21	2.9	20.9	4.2	3.4	0.1547	3.1
4/20/2018 22:22	4.4	21	4.2	3.4	0.1704	3.2
4/20/2018 22:23	2.6	21.1	4.2	3.4	0.1559	3.2
4/20/2018 22:24	2.9	21.2	4.2	3.4	0.1674	3.2
4/20/2018 22:25	3.8	20.8	4.2	3.4	0.1544	3.1
4/20/2018 22:26	3.1	21	4.2	3.4	0.1644	3.1
4/20/2018 22:27	2.4	21.1	4.3	3.4	0.1732	3
4/20/2018 22:28	3.4	20.9	4.3	3.4	0.1588	2.7
4/20/2018 22:29	3.4	20.8	4.3	3.4	0.1693	2.7
4/20/2018 22:30	3.8	20.9	4.4	3.4	0.1567	2.9
4/20/2018 22:31	4.3	20.8	4.5	3.3	0.1674	3
4/20/2018 22:32	4.2	20.9	4.4	3.3	0.1657	2.9
4/20/2018 22:33	2.9	20.8	4.4	3.3	0.1627	2.8
4/20/2018 22:34	2.8	20.7	4.4	3.3	0.1703	2.9
4/20/2018 22:35	4.5	21	4.5	3.3	0.1566	2.9
4/20/2018 22:36	4.1	20.7	4.6	3.3	0.1722	2.9
4/20/2018 22:37	4.2	20.5	4.6	3.3	0.1633	2.9
4/20/2018 22:38	3.7	20.8	4.5	3.3	0.1648	2.8
4/20/2018 22:39	2.3	20.7	4.7	3.3	0.1695	2.8
4/20/2018 22:40	4.7	20.8	4.6	3.3	0.1569	2.9
4/20/2018 22:41	2.3	20.7	4.6	3.4	0.1701	2.9
4/20/2018 22:42	3.1	20.9	4.5	3.4	0.1523	2.9
4/20/2018 22:43	2.9	20.7	4.5	3.4	0.1701	2.8
4/20/2018 22:44	1.9	20.7	4.5	3.4	0.1544	2.8
4/20/2018 22:45	2.8	20.9	4.5	3.4	0.1661	2.8
4/20/2018 22:46	3.4	20.7	4.6	3.4	0.1684	2.9
4/20/2018 22:47	4.3	20.9	4.6	3.4	0.1577	2.9
4/20/2018 22:48	3.5	20.7	4.5	3.4	0.1736	2.9
4/20/2018 22:49	3.3	20.7	4.6	3.4	0.158	3
4/20/2018 22:50	2.4	20.7	4.5	3.4	0.1712	2.8
4/20/2018 22:51	2.4	20.8	4.5	3.4	0.1549	2.7
4/20/2018 22:52	5.4	20.8	4.6	3.4	0.1691	2.7
4/20/2018 22:53	3.3	20.7	4.6	3.4	0.1639	2.7
4/20/2018 22:54	1.5	20.8	4.8	3.4	0.1628	2.7
4/20/2018 22:55	2.9	20.7	4.8	3.4	0.1687	2.8
4/20/2018 22:56	3	20.8	4.8	3.4	0.16	2.9
4/20/2018 22:57	2.3	20.7	4.8	3.4	0.1762	2.9
4/20/2018 22:58	3.1	20.9	4.6	3.4	0.1637	3
4/20/2018 22:59	3.2	20.7	4.8	3.4	0.1761	2.9
4/20/2018 23:00	2.7	20.5	5	3.4	0.1565	2.9
4/20/2018 23:01	3.1	20.8	5.1	3.4	0.1733	2.8

4/20/2018 23:02	3.4	20.8	4.9	3.4	0.1647	2.9
4/20/2018 23:03	4	20.6	4.8	3.4	0.1613	2.9
4/20/2018 23:04	2.8	20.6	4.8	3.4	0.175	3
4/20/2018 23:05	2.4	20.6	4.9	3.5	0.157	3
4/20/2018 23:06	3.4	20.7	4.9	3.4	0.1761	3
4/20/2018 23:07	3.7	20.7	4.8	3.4	0.1596	3.1
4/20/2018 23:08	3.3	20.7	4.8	3.5	0.1736	3.1
4/20/2018 23:09	2.2	20.7	4.8	3.5	0.1583	3
4/20/2018 23:10	3.4	20.7	4.7	3.5	0.1626	3
4/20/2018 23:11	2.3	20.6	4.8	3.5	0.1675	3
4/20/2018 23:12	4.9	20.7	4.7	3.5	0.1634	3
4/20/2018 23:13	4.4	20.8	4.8	3.5	0.1701	3.1
4/20/2018 23:14	2.9	20.6	4.8	3.5	0.1568	3.2
4/20/2018 23:15	4.5	20.6	4.9	3.5	0.1731	3.2
4/20/2018 23:16	4	20.6	5.2	3.4	0.1578	3.3
4/20/2018 23:17	2.9	20.4	5.2	3.4	0.1639	3.4
4/20/2018 23:18	3.5	20.5	4.7	3.4	0.1712	3.4
4/20/2018 23:19	3.1	20.7	4.9	3.4	0.1619	3.3
4/20/2018 23:20	4	20.6	5.1	3.4	0.1792	3.5
4/20/2018 23:21	2.4	20.6	5.3	3.4	0.1598	3.6
4/20/2018 23:22	2.4	20.4	5	3.4	0.1739	3.7
4/20/2018 23:23	3.5	20.4	5	3.4	0.1617	3.7
4/20/2018 23:24	3.2	20.5	5.2	3.4	0.1746	3.8
4/20/2018 23:25	2.6	20.5	4.9	3.4	0.1561	3.7
4/20/2018 23:26	1.9	20.7	4.9	3.4	0.1733	3.6
4/20/2018 23:27	2.8	20.6	5	3.4	0.1635	3.7
4/20/2018 23:28	3.9	20.5	4.9	3.4	0.1763	3.8
4/20/2018 23:29	3.8	20.5	4.9	3.5	0.1702	3.8
4/20/2018 23:30	4.4	20.6	4.9	3.4	0.1621	3.8
4/20/2018 23:31	4.1	20.4	4.9	3.5	0.1772	3.7
4/20/2018 23:32	3.6	20.6	4.9	3.5	0.157	3.8
4/20/2018 23:33	2.9	20.7	5	3.5	0.1763	3.8
4/20/2018 23:34	3.7	20.3	5	3.5	0.1615	3.8
4/20/2018 23:35	3.3	20.5	5.2	3.5	0.1714	3.8
4/20/2018 23:36	2.6	20.3	5.5	3.5	0.171	3.8
4/20/2018 23:37	4.2	20.3	5.2	3.5	0.1659	3.9
4/20/2018 23:38	3.5	20.4	5.2	3.5	0.1724	3.9
4/20/2018 23:39	2.7	20.4	5.4	3.5	0.1667	3.9
4/20/2018 23:40	1.6	20.5	5.4	3.5	0.1739	3.9
4/20/2018 23:41	2.6	20.5	5.8	3.4	0.1612	3.9
4/20/2018 23:42	2.9	20.4	5.5	3.5	0.1771	3.9
4/20/2018 23:43	3.9	20.4	5.5	3.5	0.1782	3.7
4/20/2018 23:44	3.3	20.2	5.9	3.5	0.1678	3.8
4/20/2018 23:45	4.3	20.1	5.7	3.5	0.1763	3.9
4/20/2018 23:46	3.8	20.3	5.7	3.5	0.1638	3.9
4/20/2018 23:47	2.7	20.3	5.8	3.5	0.1685	3.9
4/20/2018 23:48	4	20.3	5.7	3.5	0.1624	4

4/20/2018 23:49	0.9	20.2	5.6	3.6	0.166	4
4/20/2018 23:50	3.4	20.2	5.9	3.6	0.1572	4
4/20/2018 23:51	3.4	20.2	5.8	3.6	0.1718	4
4/20/2018 23:52	2.5	20.2	5.8	3.6	0.1656	4
4/20/2018 23:53	3.8	20.4	5.7	3.6	0.1673	3.8
4/20/2018 23:54	3.7	20	6	3.6	0.1745	3.8
4/20/2018 23:55	1.7	20	6.9	3.6	0.1716	3.9
4/20/2018 23:56	3.5	20	6.1	3.7	0.1784	3.9
4/20/2018 23:57	3.4	20	6.2	3.7	0.1651	3.8
4/20/2018 23:58	3.3	20	6.4	3.7	0.1842	3.9
4/20/2018 23:59	2.1	20	6.4	3.7	0.1625	3.9
4/21/2018 0:00	2.7	20.2	7	3.7	0.1789	4
4/21/2018 0:01	3.1	20.1	7.2	3.7	0.1764	3.8
4/21/2018 0:02	1.9	20.2	6.8	3.7	0.1731	3.3
4/21/2018 0:03	3.7	20.1	6.4	3.7	0.1818	3.6
4/21/2018 0:04	4.6	19.9	6.7	3.6	0.168	3.6
4/21/2018 0:05	2.6	19.9	6.8	3.6	0.1816	3.5
4/21/2018 0:06	3	20.2	6.7	3.6	0.165	3.7
4/21/2018 0:07	4.2	20.2	6.7	3.7	0.1859	3.8
4/21/2018 0:08	3.2	20.1	6.5	3.7	0.1805	3.8
4/21/2018 0:09	3	20.2	6.5	3.7	0.179	3.9
4/21/2018 0:10	3.1	20.1	6.5	3.7	0.1837	3.9
4/21/2018 0:11	2.9	20.2	6.5	3.7	0.175	4
4/21/2018 0:12	2.2	20.4	6.2	3.7	0.1844	4
4/21/2018 0:13	4.9	20	6.6	3.7	0.1781	3.9
4/21/2018 0:14	2.4	19.9	6.8	3.7	0.1943	4
4/21/2018 0:15	2.8	20.2	6.5	3.7	0.1667	4.1
4/21/2018 0:16	3.3	20.1	6.3	3.7	0.185	3.8
4/21/2018 0:17	2.9	20.3	6.2	3.8	0.1783	1.9
4/21/2018 0:18	1.4	19.9	6.3	3.8	0.1855	1.8
4/21/2018 0:19	1.8	20.3	6.5	3.7	0.1932	2.2
4/21/2018 0:20	3.3	20.1	6.5	3.7	0.1788	2.3
4/21/2018 0:21	2.4	20.1	6.3	3.7	0.1899	2.3
4/21/2018 0:22	4.4	20.3	6.4	3.7	0.179	2.3
4/21/2018 0:23	3.8	20.2	6.3	3.8	0.1893	2.5
4/21/2018 0:24	3.3	20.2	6.2	3.8	0.1866	2.5
4/21/2018 0:25	2.3	20.6	6.2	3.7	0.1885	2.6
4/21/2018 0:26	3.2	20.1	6.2	3.7	0.187	2.7
4/21/2018 0:27	2.6	20.2	6.2	3.7	0.1976	2.8
4/21/2018 0:28	3.7	20.2	6.1	3.7	0.1897	2.8
4/21/2018 0:29	3.3	20.3	6	3.7	0.185	2.7
4/21/2018 0:30	3	20.1	6	3.7	0.1893	2.7
4/21/2018 0:31	3.1	20.3	6	3.7	0.1752	2.7
4/21/2018 0:32	3.1	20.5	6.2	3.7	0.2005	2.7
4/21/2018 0:33	2.7	20.6	6.1	3.7	0.1844	2.7
4/21/2018 0:34	2.6	20.5	6	3.7	0.1888	2.9
4/21/2018 0:35	3.2	20.5	5.9	3.7	0.1776	3

4/21/2018 0:36	2.3	20.4	6	3.7	0.1903	3.1
4/21/2018 0:37	3	20.4	6	3.7	0.187	3.1
4/21/2018 0:38	2.8	20.4	5.9	3.7	0.182	3.1
4/21/2018 0:39	3.1	20.7	5.9	3.7	0.1938	3
4/21/2018 0:40	2.5	20.6	5.8	3.7	0.1834	3
4/21/2018 0:41	3.9	20.3	5.8	3.7	0.1896	3
4/21/2018 0:42	3.9	20.3	5.8	3.7	0.1847	3
4/21/2018 0:43	2.5	20.3	5.8	3.7	0.1951	3
4/21/2018 0:44		20.3	5.8	3.6	0.1889	2.9
4/21/2018 0:45		20.5	5.8	3.7	0.1914	3
4/21/2018 0:46	7.1	20.4	5.7	3.7	0.1874	3.1
4/21/2018 0:47	5.1	20.4	5.7	3.7	0.1782	3.1
4/21/2018 0:48	3.2	20.3	5.7	3.6	0.1921	3.1
4/21/2018 0:49	2.3	20.4	5.7	3.6	0.1754	3.1
4/21/2018 0:50	3.5	20.6	5.7	3.6	0.1905	3
4/21/2018 0:51	3.4	20.4	5.6	3.6	0.1797	3.1
4/21/2018 0:52	2.8	20.7	5.6	3.6	0.1903	3.2
4/21/2018 0:53		20.7	5.6	3.6	0.1774	3.2
4/21/2018 0:54		20.5	5.5	3.6	0.1795	3.1
4/21/2018 0:55		20.5	5.5	3.5	0.1781	3.2
4/21/2018 0:56		20.6	5.5	3.5	0.1799	3.2
4/21/2018 0:57		20.6	5.4	3.5	0.1939	3.2
4/21/2018 0:58		20.4	5.4	3.5	0.1758	3.2
4/21/2018 0:59	4	20.4	5.4	3.5	0.1886	3.2
4/21/2018 1:00		20.4	5.4	3.4	0.1725	3.3
4/21/2018 1:01	3.8	20.5	5.3	3.5	0.1839	3.3
4/21/2018 1:02	2.8	20.5	5.3	3.5	0.1773	3.3
4/21/2018 1:03		20.7	5.4	3.5	0.1857	3.3
4/21/2018 1:04	3.2	20.5	5.3	3.5	0.1819	3.3
4/21/2018 1:05	2.2	20.3	5.3	3.6	0.1814	3.3
4/21/2018 1:06	2.8	20.5	5.3	3.6	0.1907	3.3
4/21/2018 1:07	2.5	20.6	5.4	3.6	0.1755	3.4
4/21/2018 1:08	2.4	20.5	5.4	3.6	0.1877	3.4
4/21/2018 1:09	2.8	20.3	5.3	3.6	0.1762	3.4
4/21/2018 1:10	1.9	20.6	5.3	3.6	0.1879	3.5
4/21/2018 1:11	5	20.5	5.3	3.6	0.174	3.5
4/21/2018 1:12	2.2	20.5	5.3	3.6	0.1814	3.5
4/21/2018 1:13	2.1	20.3	5.2	3.6	0.1841	3.5
4/21/2018 1:14	2.9	20.3	5.1	3.5	0.1727	3.6
4/21/2018 1:15	3.8	20.3	5.2	3.5	0.1856	3.6
4/21/2018 1:16	2.9	20.1	5.2	3.5	0.1733	3.6
4/21/2018 1:17	3.7	20.5	5.2	3.5	0.1815	3.6
4/21/2018 1:18	2.1	20	5.2	3.5	0.174	3.7
4/21/2018 1:19	3	20.4	5.2	3.5	0.1867	3.2
4/21/2018 1:20	3.9	20.5	5.1	3.5	0.1824	2.4
4/21/2018 1:21	4.4	20.5	5.1	3.6	0.183	2.6
4/21/2018 1:22	2.5	20.2	5.3	3.5	0.1772	2.7

4/21/2018 1:23	2.3	20.4	5.2	3.5	0.1755	2.8
4/21/2018 1:24	2.2	20.4	5.2	3.5	0.1826	2.7
4/21/2018 1:25	2.4	20.4	5.3	3.5	0.1683	2.4
4/21/2018 1:26	3.5	20.2	5.3	3.6	0.1868	2.2
4/21/2018 1:27	2	20.2	5.3	3.6	0.1733	2.3
4/21/2018 1:28	2.7	20	5.4	3.5	0.1926	2.3
4/21/2018 1:29	2.9	20.4	5.5	3.5	0.1811	2.4
4/21/2018 1:30	2	20.1	5.3	3.5	0.1808	2.4
4/21/2018 1:31	2.1	20.1	5.4	3.5	0.182	2.4
4/21/2018 1:32	2.4	20.5	5.3	3.5	0.1758	2.6
4/21/2018 1:33	2.6	20.2	5.3	3.6	0.1885	2.7
4/21/2018 1:34	3.5	20.2	5.5	3.6	0.1748	2.7
4/21/2018 1:35	2.9	20.3	5.4	3.6	0.186	2.8
4/21/2018 1:36	3.5	19.8	5.5	3.6	0.1704	3
4/21/2018 1:37	3.4	20.1	5.6	3.6	0.1845	3
4/21/2018 1:38	2.6	20.1	5.8	3.6	0.1724	3
4/21/2018 1:39	3.7	19.7	5.8	3.6	0.1794	3.1
4/21/2018 1:40	2.6	20.1	6.1	3.6	0.1794	3
4/21/2018 1:41	3.3	19.9	5.6	3.6	0.1775	2.5
4/21/2018 1:42	3.3	20.2	6.1	3.6	0.1892	2
4/21/2018 1:43	2.9	20	6	3.6	0.1748	2.2
4/21/2018 1:44	1.4	20.1	6.2	3.6	0.1903	2.4
4/21/2018 1:45	1.9	20.2	6	3.6	0.1707	2.4
4/21/2018 1:46	1.4	20	6	3.7	0.1766	2.5
4/21/2018 1:47	1.5	20.1	6.4	3.7	0.1789	2.6
4/21/2018 1:48	2.8	19.8	6.4	3.7	0.1762	2.7
4/21/2018 1:49	2.8	20	6.5	3.7	0.1817	2.7
4/21/2018 1:50	3.3	20.2	6.1	3.7	0.1772	2.9
4/21/2018 1:51	2.5	19.8	6	3.7	0.1883	2.9
4/21/2018 1:52	3.7	20	6.2	3.8	0.1734	3
4/21/2018 1:53	3.6	19.9	6.1	3.8	0.1829	3
4/21/2018 1:54	2.8	19.8	6.7	3.8	0.1741	3
4/21/2018 1:55	2.8	19.8	6.8	3.8	0.1846	3
4/21/2018 1:56	2.8	19.9	6.1	3.8	0.1731	3.1
4/21/2018 1:57	2.1	20.1	6.5	3.9	0.1755	3
4/21/2018 1:58	4.2	20	6.1	3.9	0.1863	2.9
4/21/2018 1:59	2.8	19.9	5.9	3.8	0.1705	3
4/21/2018 2:00	2.1	20	6.4	3.8	0.1824	3
4/21/2018 2:01	1.9	19.8	6.7	3.8	0.1752	3
4/21/2018 2:02	2.9	19.8	6.8	3.8	0.1842	3
4/21/2018 2:03	2.4	20.2	6.8	3.7	0.1714	3
4/21/2018 2:04	3.1	19.8	6.4	3.8	0.1804	3.1
4/21/2018 2:05	2.9	20	6.3	3.8	0.1833	3.1
4/21/2018 2:06	2.6	19.7	6.6	3.8	0.1739	3.2
4/21/2018 2:07	4.3	19.9	6.4	3.8	0.1897	3.1
4/21/2018 2:08	3.4	20.1	6.4	3.8	0.1793	3.2
4/21/2018 2:09	3.1	19.8	6.4	3.8	0.1886	3.2

4/21/2018 2:10	2.3	19.9	6.4	3.8	0.174	3.2
4/21/2018 2:11	4.2	19.9	6.9	3.8	0.1927	2.9
4/21/2018 2:12	2.2	20	7	3.8	0.17	3
4/21/2018 2:13	3.3	20.2	6.9	3.8	0.1836	3.1
4/21/2018 2:14	2.1	19.9	6.2	3.8	0.1744	3
4/21/2018 2:15	2.8	20	6.2	3.8	0.184	3.1
4/21/2018 2:16	1.8	20.1	6.3	3.7	0.1816	3.2
4/21/2018 2:17	3.2	20	6.5	3.7	0.1747	3.4
4/21/2018 2:18	2.2	19.8	6.4	3.7	0.1935	3.4
4/21/2018 2:19	2.6	20	6.3	3.7	0.1711	3.5
4/21/2018 2:20	1.8	19.9	6.3	3.7	0.184	3.6
4/21/2018 2:21	1.5	20.1	6.2	3.7	0.1741	3.6
4/21/2018 2:22	1.9	20.1	6.2	3.7	0.1901	3.6
4/21/2018 2:23	2.3	20.1	6.2	3.7	0.1728	3.5
4/21/2018 2:24	3.6	20	6.2	3.7	0.1857	3.5
4/21/2018 2:25	2.8	20	6.3	3.7	0.1732	3.5
4/21/2018 2:26	5.2	19.9	6.3	3.7	0.1701	3.5
4/21/2018 2:27	3.9	20.1	6	3.7	0.1866	3.5
4/21/2018 2:28	2.6	20	6.1	3.6	0.17	3.5
4/21/2018 2:29	2.7	20.2	6	3.7	0.1886	3.5
4/21/2018 2:30	2.3	20	6	3.6	0.1708	3.6
4/21/2018 2:31	2.5	20.3	5.9	3.6	0.189	3.5
4/21/2018 2:32	3.3	20.2	5.9	3.7	0.1713	3.6
4/21/2018 2:33	2.3	20.1	6	3.7	0.1811	3.6
4/21/2018 2:34	1.5	20	6.2	3.7	0.179	3.7
4/21/2018 2:35	1.3	19.9	6.2	3.7	0.1765	3.6
4/21/2018 2:36	2.7	19.8	6	3.8	0.1766	3.1
4/21/2018 2:37	2.6	19.9	6.1	3.8	0.1744	3
4/21/2018 2:38	3.1	19.9	6	3.8	0.1841	3.1
4/21/2018 2:39	2.4	20	6.2	3.8	0.1677	3.3
4/21/2018 2:40	2.1	19.7	6.3	3.8	0.1876	3.5
4/21/2018 2:41	3	19.9	6	3.8	0.1689	3.6
4/21/2018 2:42	3.5	20	6.1	3.8	0.1778	3.6
4/21/2018 2:43	1.8	19.9	6.3	3.8	0.179	3.6
4/21/2018 2:44	1.6	19.7	7	3.8	0.1785	3.5
4/21/2018 2:45	1.8	20.1	6.6	3.8	0.1825	3.5
4/21/2018 2:46	2.4	19.9	6.3	3.8	0.1764	3.6
4/21/2018 2:47	3.8	19.9	6.3	3.8	0.1824	3.6
4/21/2018 2:48	3.8	19.7	6.5	3.9	0.1763	3.6
4/21/2018 2:49	1.9	19.9	6.7	3.9	0.1762	3.7
4/21/2018 2:50	3	20	6.9	3.9	0.1762	3.8
4/21/2018 2:51	1.9	19.7	7	3.9	0.1742	3.8
4/21/2018 2:52	1.1	19.6	6.8	3.9	0.181	3.8
4/21/2018 2:53	1.7	19.5	7.1	3.9	0.1708	3.8
4/21/2018 2:54	4.1	19.7	6.9	3.9	0.1849	3.8
4/21/2018 2:55	2.7	19.6	6.9	3.9	0.1637	3.8
4/21/2018 2:56	3.6	19.7	6.6	3.9	0.1891	3.8

4/21/2018 2:57	1.7	20	6.7	3.9	0.172	3.9
4/21/2018 2:58	2.6	19.8	7.1	3.9	0.1792	3.8
4/21/2018 2:59	3.1	19.6	6.9	3.9	0.1787	3.9
4/21/2018 3:00	1.8	19.6	6.7	3.9	0.1724	3.8
4/21/2018 3:01	1.7	19.8	6.6	3.9	0.1803	3.8
4/21/2018 3:02	1.8	19.5	6.7	3.9	0.1655	3.8
4/21/2018 3:03	3.9	19.7	6.6	3.9	0.1833	3.8
4/21/2018 3:04	2.4	19.7	6.5	3.9	0.1715	3.6
4/21/2018 3:05	2.8	19.8	6.8	3.9	0.1854	3
4/21/2018 3:06	4	20.1	6.9	3.9	0.1778	2.3
4/21/2018 3:07	1.5	19.7	6.8	3.9	0.1743	2.1
4/21/2018 3:08	2.5	19.7	7	3.9	0.1776	2.1
4/21/2018 3:09	2.3	19.9	6.9	3.9	0.1709	2.1
4/21/2018 3:10	2.8	19.8	6.8	4	0.1877	2
4/21/2018 3:11	3.6	19.7	6.7	4	0.1689	1.8
4/21/2018 3:12	3.8	20.1	6.6	4	0.1858	1.8
4/21/2018 3:13	3.2	19.7	6.6	4	0.1658	1.9
4/21/2018 3:14	2.9	19.9	6.7	4	0.1812	1.6
4/21/2018 3:15	1.9	19.8	6.6	4	0.1827	1.6
4/21/2018 3:16	3.7	19.6	6.6	4	0.1741	1.5
4/21/2018 3:17	2.8	19.8	6.5	4	0.1837	1.6
4/21/2018 3:18	2.7	19.9	6.4	4	0.1716	1.6
4/21/2018 3:19	1.8	19.8	6.5	4	0.1829	1.6
4/21/2018 3:20	3.3	20	6.5	4	0.1704	1.6
4/21/2018 3:21	2.8	19.8	6.4	4	0.1797	1.6
4/21/2018 3:22	4.5	19.7	6.6	4	0.1771	1.6
4/21/2018 3:23	3	19.8	6.6	4	0.1711	1.6
4/21/2018 3:24	3.7	19.9	6.4	4	0.1797	1.5
4/21/2018 3:25	2.5	20	6.4	3.9	0.168	1.5
4/21/2018 3:26	1.9	19.9	6.3	3.9	0.1867	1.5
4/21/2018 3:27	2.3	20	6.3	3.9	0.1709	1.5
4/21/2018 3:28	3.7	20.1	6.4	3.9	0.1881	1.5
4/21/2018 3:29	2.3	19.7	6.7	3.9	0.1712	1.6
4/21/2018 3:30	2.4	20	6.6	3.9	0.1799	1.6
4/21/2018 3:31	1	20	6.4	3.9	0.1701	1.5
4/21/2018 3:32	2.2	20	6.5	3.9	0.1765	1.5
4/21/2018 3:33	2.4	20	6.4	3.9	0.1769	1.5
4/21/2018 3:34		19.9	6.4	3.9	0.1676	1.5
4/21/2018 3:35	3.3	19.9	6.5	3.9	0.1824	1.5
4/21/2018 3:36	2.6	19.9	6.3	3.9	0.1605	1.5
4/21/2018 3:37	1.9	19.7	6.4	4	0.183	1.6
4/21/2018 3:38	3.3	19.8	6.7	4	0.1657	1.5
4/21/2018 3:39	2.3	19.6	6.9	3.9	0.177	1.5
4/21/2018 3:40	1.9	19.7	6.5	4	0.1767	1.5
4/21/2018 3:41	1.5	19.6	6.7	4	0.1651	1.5
4/21/2018 3:42	1.5	19.6	6.7	4	0.1836	1.5
4/21/2018 3:43	3.4	19.8	6.5	4	0.1728	1.5

4/21/2018 3:44		19.5	6.8	4	0.1791	1.5
4/21/2018 3:45	0.9	19.6	7.1	3.9	0.1648	1.6
4/21/2018 3:46		19.7	7.3	3.9	0.1806	1.5
4/21/2018 3:47	2.8	19.6	6.9	3.9	0.1652	1.5
4/21/2018 3:48		19.6	6.7	3.9	0.1716	1.6
4/21/2018 3:49	2.3	19.6	6.8	3.9	0.175	1.5
4/21/2018 3:50	3.6	19.6	6.6	3.9	0.1649	1.5
4/21/2018 3:51		20.1	6.8	4	0.1778	1.6
4/21/2018 3:52	1.9	20	6.6	4	0.1673	1.5
4/21/2018 3:53		19.6	6.6	4	0.1757	1.5
4/21/2018 3:54		19.5	6.6	4	0.1668	1.5
4/21/2018 3:55		19.8	6.9	4	0.1791	1.5
4/21/2018 3:56	2.7	19.7	6.8	4.1	0.1678	1.5
4/21/2018 3:57	3.5	19.7	7.1	4	0.1745	1.5
4/21/2018 3:58	2.4	19.7	7	4	0.1731	1.5
4/21/2018 3:59	3	19.7	6.8	4	0.1747	1.6
4/21/2018 4:00	2.7	19.4	7.3	4	0.1792	1.6
4/21/2018 4:01	1.7	19.6	7.1	4	0.167	1.6
4/21/2018 4:02	2.5	19.5	7.2	4	0.1814	1.6
4/21/2018 4:03	3.3	19.8	7.5	3.9	0.1668	1.6
4/21/2018 4:04	3.2	19.8	7.7	3.9	0.1769	1.6
4/21/2018 4:05	2.5	19.6	7.7	3.9	0.1718	1.6
4/21/2018 4:06	2.2	19.6	7.3	3.9	0.1687	1.5
4/21/2018 4:07	2.5	19.7	6.9	3.9	0.178	1.6
4/21/2018 4:08	1.7	19.6	7.1	3.9	0.164	1.6
4/21/2018 4:09	2.1	19.9	7	3.9	0.1818	1.6
4/21/2018 4:10	2.6	20	7	3.9	0.1597	1.6
4/21/2018 4:11	2.9	20	7	4	0.1797	1.6
4/21/2018 4:12	3.3	19.7	7.1	4	0.1664	1.6
4/21/2018 4:13	4.3	19.5	7.2	4	0.1696	1.6
4/21/2018 4:14	1.3	19.5	7.1	4	0.179	1.6
4/21/2018 4:15	3.9	19.5	7.1	4	0.163	1.6
4/21/2018 4:16	3.2	19.6	7.2	4	0.1826	1.6
4/21/2018 4:17	2.9	19.7	7.1	4	0.1636	1.6
4/21/2018 4:18	3.1	19.6	6.8	4	0.1781	1.6
4/21/2018 4:19	3.3	19.6	7	4	0.1635	1.6
4/21/2018 4:20	2.7	19.7	6.9	4	0.1761	1.6
4/21/2018 4:21	3.1	19.9	6.9	4	0.1674	1.6
4/21/2018 4:22	2.2	19.7	7.3	4	0.1677	1.6
4/21/2018 4:23	3.4	19.5	7	4	0.177	1.6
4/21/2018 4:24	2.4	19.7	6.8	4.1	0.1643	1.6
4/21/2018 4:25	2	19.7	7.1	4	0.1779	1.6
4/21/2018 4:26	2.2	19.5	7	4	0.1681	1.6
4/21/2018 4:27	2	19.3	7.4	4	0.177	1.6
4/21/2018 4:28	3.1	19.3	7.5	4	0.1669	1.6
4/21/2018 4:29	3.7	19.8	7.3	4	0.1673	1.6
4/21/2018 4:30	2.4	19.9	7.2	3.9	0.1708	1.6

4/21/2018 4:31	3.4	19.8	6.7	4	0.1583	1.6
4/21/2018 4:32	2.7	19.7	6.8	4	0.1741	1.7
4/21/2018 4:33	2.9	19.8	6.8	4	0.1659	1.7
4/21/2018 4:34	3.5	19.8	6.6	4	0.1732	1.7
4/21/2018 4:35	2.7	19.9	6.5	4	0.1731	1.7
4/21/2018 4:36	2	19.8	6.7	4	0.1695	1.7
4/21/2018 4:37	2.3	19.5	6.5	4.1	0.1757	1.7
4/21/2018 4:38	1.9	19.9	6.5	4.1	0.1657	1.7
4/21/2018 4:39	3.3	19.6	6.7	4.1	0.1775	1.7
4/21/2018 4:40	3.3	19.6	6.7	4.1	0.1568	1.7
4/21/2018 4:41	2.2	19.8	6.7	4.1	0.1693	1.6
4/21/2018 4:42	2.4	19.8	6.8	4.1	0.1735	1.7
4/21/2018 4:43	3.8	19.5	6.5	4.1	0.1651	1.7
4/21/2018 4:44	2.6	19.5	6.5	4.1	0.1774	1.7
4/21/2018 4:45	2.9	19.7	6.6	4.1	0.161	1.7
4/21/2018 4:46	0.9	19.3	6.8	4	0.1763	1.7
4/21/2018 4:47	1.9	19.7	6.5	4	0.1627	1.7
4/21/2018 4:48	3.4	19.6	6.5	4.1	0.1683	1.7
4/21/2018 4:49	2.9	19.6	6.6	4	0.1695	1.7
4/21/2018 4:50	3.5	19.7	6.3	4.1	0.1675	1.7
4/21/2018 4:51	2	19.8	6.4	4.1	0.1694	1.7
4/21/2018 4:52	2.5	19.9	6.3	4.1	0.1608	1.7
4/21/2018 4:53	2.8	20.1	6.3	4.1	0.175	1.7
4/21/2018 4:54	2.2	19.9	6.2	4.1	0.1599	1.8
4/21/2018 4:55	3.1	19.8	6.5	4.1	0.1751	1.8
4/21/2018 4:56	2.1	19.9	6.3	4.1	0.1691	1.7
4/21/2018 4:57	3.5	19.8	6.3	4.1	0.1681	1.7
4/21/2018 4:58	3.1	20	6.4	4.1	0.171	1.7
4/21/2018 4:59	1.9	19.7	6.3	4.1	0.1646	1.7
4/21/2018 5:00	2.3	19.7	6.3	4.1	0.1661	1.7
4/21/2018 5:01	3.4	19.7	6.6	4	0.1584	1.7
4/21/2018 5:02	2.7	19.6	6.7	4	0.1726	1.7
4/21/2018 5:03	2.6	19.5	6.4	4	0.1624	1.7
4/21/2018 5:04	2.1	19.6	6.7	4	0.1683	1.7
4/21/2018 5:05	3.4	19.7	6.5	3.9	0.165	1.7
4/21/2018 5:06	2.9	19.6	6.4	3.9	0.167	1.7
4/21/2018 5:07	4.2	19.6	6.3	3.9	0.1747	1.7
4/21/2018 5:08	3.4	19.5	6.3	3.9	0.1673	1.7
4/21/2018 5:09	5	19.9	6.2	4	0.1717	1.7
4/21/2018 5:10	2.1	19.9	6.1	4	0.1632	1.7
4/21/2018 5:11	2.6	19.6	6.2	4	0.1682	1.7
4/21/2018 5:12	3.1	19.8	6.3	4.1	0.1698	1.7
4/21/2018 5:13	3	19.9	6.1	4.1	0.1636	1.8
4/21/2018 5:14	2.5	19.8	6.3	4.1	0.1773	1.7
4/21/2018 5:15	2.5	19.7	6.4	4.1	0.1594	1.7
4/21/2018 5:16	2.8	19.7	6.4	4.1	0.175	1.7
4/21/2018 5:17	2.4	19.6	6.4	4.1	0.1583	1.8

4/21/2018 5:18	2.5	19.7	6.3	4.1	0.1763	1.8
4/21/2018 5:19	3	19.5	6.4	4.1	0.1642	1.8
4/21/2018 5:20	2.6	19.6	6.4	4.1	0.1705	1.8
4/21/2018 5:21	3.3	19.4	6.6	4	0.1653	1.8
4/21/2018 5:22	2.5	19.7	6.8	4	0.1644	1.8
4/21/2018 5:23	1.9	19.8	6.6	4	0.175	1.8
4/21/2018 5:24	2.8	19.7	6.8	4	0.1659	1.8
4/21/2018 5:25	3	19.6	6.7	4	0.1759	1.8
4/21/2018 5:26	4.1	19.7	6.2	4	0.1691	1.8
4/21/2018 5:27	2.9	19.7	6.2	4	0.1762	1.8
4/21/2018 5:28	3.5	19.8	6.2	4	0.1698	1.8
4/21/2018 5:29	3.6	19.7	6.3	4	0.1685	1.8
4/21/2018 5:30	4	19.3	6.4	4.1	0.1725	1.8
4/21/2018 5:31	2.3	19.8	6.2	4.1	0.1548	1.8
4/21/2018 5:32	3.3	19.8	6.1	4.1	0.1791	1.9
4/21/2018 5:33	3.6	19.9	6.1	4.2	0.1606	1.9
4/21/2018 5:34	2.4	19.6	6	4.2	0.171	1.8
4/21/2018 5:35	2.1	19.7	6.2	4.2	0.167	1.8
4/21/2018 5:36	2.7	19.6	6.2	4.2	0.1698	1.9
4/21/2018 5:37	4.1	19.9	6.3	4.2	0.1724	1.9
4/21/2018 5:38	3.3	19.6	6.3	4.2	0.1607	2
4/21/2018 5:39	3.3	19.7	6.1	4.2	0.176	2
4/21/2018 5:40	2.1	19.7	6.1	4.2	0.1634	2
4/21/2018 5:41	1.9	19.8	6	4.2	0.1698	2
4/21/2018 5:42	1.6	19.6	6	4.2	0.1745	2
4/21/2018 5:43	3.5	19.8	6	4.2	0.1693	2
4/21/2018 5:44	3.1	19.9	6	4.2	0.1693	2
4/21/2018 5:45	2	19.8	6.2	4.2	0.1712	2.1
4/21/2018 5:46	2.9	19.8	6	4.2	0.1766	2.2
4/21/2018 5:47	2.9	19.6	6.1	4.2	0.1581	2.1
4/21/2018 5:48	2.1	19.7	6.3	4.2	0.1696	2
4/21/2018 5:49	3.3	19.6	6.3	4.2	0.1658	2.1
4/21/2018 5:50	3.3	19.6	6.4	4.2	0.1665	2.2
4/21/2018 5:51	3.3	19.6	6.4	4.2	0.1761	2.2
4/21/2018 5:52	2	19.7	6.7	4.2	0.1607	2.1
4/21/2018 5:53	2.4	19.8	6.6	4.1	0.1741	2.2
4/21/2018 5:54	3.3	19.8	6.8	4.1	0.163	2.3
4/21/2018 5:55	2.3	19.8	6.4	4.1	0.1727	2.4
4/21/2018 5:56	2.9	19.5	6.2	4.1	0.1576	2.5
4/21/2018 5:57	3.3	19.7	6.1	4.1	0.1711	2.5
4/21/2018 5:58	3.2	19.8	6.1	4.2	0.1591	2.5
4/21/2018 5:59	2.9	19.6	6.3	4.2	0.1718	2.6
4/21/2018 6:00	2.3	19.7	6.1	4.2	0.1757	2.7
4/21/2018 6:01	3.3	19.3	6.8	4.3	0.1619	2.8
4/21/2018 6:02	2.5	19.5	6.8	4.3	0.1712	2.9
4/21/2018 6:03	1.4	19.5	6.5	4.3	0.1627	2.9
4/21/2018 6:04	2.9	19.4	6.4	4.3	0.1741	2.7

4/21/2018 6:05	2.8	19.9	6.1	4.3	0.1616	2.6
4/21/2018 6:06	1.5	19.5	6.1	4.3	0.1705	2.6
4/21/2018 6:07	2.6	19.6	6.3	4.3	0.1711	2.6
4/21/2018 6:08	0.9	19.4	6.1	4.3	0.1565	2.7
4/21/2018 6:09	2.8	19.8	6.3	4.3	0.1733	2.7
4/21/2018 6:10	3.2	19.8	6.3	4.4	0.159	2.7
4/21/2018 6:11	2.8	19.5	6.3	4.4	0.1768	2.7
4/21/2018 6:12	1	19.6	6.2	4.4	0.1664	2.7
4/21/2018 6:13	3.9	19.7	6.3	4.4	0.1692	2.7
4/21/2018 6:14	2.8	19.8	6.1	4.4	0.1657	2.6
4/21/2018 6:15	2.1	19.7	6.1	4.4	0.1642	2.6
4/21/2018 6:16	1.7	19.5	6.1	4.4	0.1769	2.6
4/21/2018 6:17	3.7	19.9	6.1	4.4	0.1563	2.7
4/21/2018 6:18	3.1	19.5	6.1	4.4	0.1756	2.7
4/21/2018 6:19	3.3	19.5	6.2	4.5	0.1633	2.7
4/21/2018 6:20	1	19.7	6.2	4.5	0.1736	2.6
4/21/2018 6:21	2.3	19.8	6.1	4.5	0.1768	2.5
4/21/2018 6:22	1.7	19.7	6.2	4.4	0.164	2.5
4/21/2018 6:23	1.9	19.7	6.2	4.4	0.1871	2.4
4/21/2018 6:24	3.2	19.8	6.2	4.4	0.1605	2.5
4/21/2018 6:25	2.1	19.6	6.3	4.4	0.1785	2.6
4/21/2018 6:26	2.1	19.7	6.2	4.4	0.1663	2.6
4/21/2018 6:27	3	19.8	6.2	4.4	0.1693	2.7
4/21/2018 6:28	1.2	19.6	6.4	4.4	0.1684	2.6
4/21/2018 6:29	2.5	19.5	6.4	4.4	0.1696	2.7
4/21/2018 6:30	3.1	19.5	6.6	4.4	0.1816	2.7
4/21/2018 6:31	1.6	19.4	6.9	4.4	0.1598	2.6
4/21/2018 6:32	3.2	19.5	6.9	4.3	0.1775	2.7
4/21/2018 6:33	2.6	19.6	7	4.3	0.1677	2.8
4/21/2018 6:34	2.9	19.5	7	4.2	0.179	2.7
4/21/2018 6:35	1.7	19.4	6.8	4.2	0.171	2.2
4/21/2018 6:36	3.1	19.4	7	4.1	0.166	2.2
4/21/2018 6:37	4.2	19.5	6.8	4.1	0.1767	2.3
4/21/2018 6:38	3.1	19.7	6.7	4.1	0.1654	2.1
4/21/2018 6:39	2.6	19.5	6.6	4.1	0.1761	2.2
4/21/2018 6:40	2.6	19.5	6.5	4.1	0.1661	2.3
4/21/2018 6:41	3.1	19.5	6.5	4.1	0.1765	2.5
4/21/2018 6:42	2.2	19.3	6.5	4.2	0.1671	2.5
4/21/2018 6:43	3.1	19.5	6.5	4.2	0.1705	2.6
4/21/2018 6:44	2.4	19.5	6.6	4.2	0.1776	2.5
4/21/2018 6:45	3.5	19.6	6.6	4.2	0.1664	2.5
4/21/2018 6:46	2.9	19.4	6.7	4.2	0.18	2.4
4/21/2018 6:47	1.9	19.5	6.6	4.2	0.161	2.2
4/21/2018 6:48	2.5	19.4	6.3	4.3	0.1736	2.3
4/21/2018 6:49	2.1	19.5	6.3	4.3	0.17	2.2
4/21/2018 6:50	2.6	19.5	6.2	4.4	0.169	2.2
4/21/2018 6:51	3.1	19.4	6.2	4.4	0.1755	2.2

4/21/2018 6:52	1	19.4	6.2	4.5	0.1634	2.2
4/21/2018 6:53	1.6	19.6	6.2	4.5	0.1716	2.1
4/21/2018 6:54	2.6	19.9	6.3	4.5	0.169	2.1
4/21/2018 6:55	2.5	19.6	6.2	4.5	0.1737	2.2
4/21/2018 6:56	4.4	19.7	6.4	4.5	0.1703	2.2
4/21/2018 6:57	2.9	19.6	6.5	4.5	0.163	2.3
4/21/2018 6:58	1.3	19.6	6.3	4.5	0.1771	2.1
4/21/2018 6:59	3.1	19.7	6.3	4.5	0.164	2.2
4/21/2018 7:00	2	19.8	6.5	4.5	0.1722	2.3
4/21/2018 7:01	2	19.4	6.3	4.5	0.1684	2.2
4/21/2018 7:02	3.6	19.7	6.3	4.5	0.1687	2
4/21/2018 7:03		19.5	6.4	4.5	0.177	1.9
4/21/2018 7:04		19.6	6.4	4.5	0.1722	1.9
4/21/2018 7:05	3.7	19.3	6.7	4.5	0.1768	2
4/21/2018 7:06		19.6	6.6	4.5	0.1609	1.9
4/21/2018 7:07	3	19.5	7.2	4.4	0.1718	1.9
4/21/2018 7:08	4.4	19.4	7.2	4.4	0.1665	1.9
4/21/2018 7:09		19.3	7.1	4.3	0.1674	2
4/21/2018 7:10	2.4	19.4	7.5	4.3	0.1783	2.1
4/21/2018 7:11	5.2	19.3	6.9	4.3	0.165	2.2
4/21/2018 7:12	3.1	19.4	7.2	4.2	0.1793	2.1
4/21/2018 7:13		19.4	7.2	4.2	0.1603	2.1
4/21/2018 7:14		19.5	6.8	4.2	0.1688	2.1
4/21/2018 7:15	2.5	19.6	6.6	4.2	0.1651	2.2
4/21/2018 7:16	5.3	19.4	6.6	4.3	0.1705	2.3
4/21/2018 7:17	3.2	19.4	6.6	4.3	0.1822	2.3
4/21/2018 7:18	3.1	19.8	6.6	4.3	0.163	2.3
4/21/2018 7:19	3.5	19.4	6.6	4.4	0.1734	2.3
4/21/2018 7:20	3	19.6	6.7	4.4	0.1557	2.2
4/21/2018 7:21	4	19.4	6.7	4.4	0.1774	2.2
4/21/2018 7:22	3.8	19.7	6.5	4.4	0.1701	2.2
4/21/2018 7:23	2	19.4	6.7	4.4	0.1677	2.1
4/21/2018 7:24	2.5	19.4	6.9	4.4	0.1763	2.2
4/21/2018 7:25	3.3	19.4	6.8	4.4	0.1616	2.2
4/21/2018 7:26	4	19.5	6.6	4.4	0.1701	1.9
4/21/2018 7:27	2.9	19.6	6.6	4.4	0.1647	1.8
4/21/2018 7:28	2.1	19.5	6.6	4.5	0.1826	1.9
4/21/2018 7:29	2.8	19.7	6.5	4.5	0.1676	1.9
4/21/2018 7:30	2.7	19.7	6.6	4.5	0.1643	1.9
4/21/2018 7:31	2.9	19.5	6.5	4.5	0.1693	2
4/21/2018 7:32	1.6	19.2	6.8	4.6	0.1607	1.9
4/21/2018 7:33	2	19.6	6.8	4.6	0.178	1.8
4/21/2018 7:34	1.6	19.5	6.7	4.6	0.1654	1.8
4/21/2018 7:35	3.2	19.3	6.7	4.6	0.1666	1.9
4/21/2018 7:36	1.4	19.5	6.7	4.6	0.1707	1.9
4/21/2018 7:37	2.9	19.4	6.6	4.6	0.1615	2
4/21/2018 7:38	2.8	19.5	6.8	4.6	0.1817	2

4/21/2018 7:39			1	19.4	6.6	4.6	0.1616	2.1
4/21/2018 7:40			3.1	19.6	6.5	4.6	0.1762	2.2
4/21/2018 7:41			3.7	19.4	6.6	4.6	0.1691	2.2
4/21/2018 7:42			2.5	19.3	6.6	4.6	0.1724	2.2
4/21/2018 7:43			2.2	19.3	6.9	4.6	0.185	2.2
4/21/2018 7:44			2.2	19.5	7.1	4.6	0.1588	2.3
4/21/2018 7:45			3.7	19.3	7.1	4.6	0.1755	2.3
4/21/2018 7:46			2.5	19.4	7.2	4.6	0.1666	2.3
4/21/2018 7:47			4.5	19.5	7.2	4.6	0.1808	2.3
4/21/2018 7:48			3.5	18.5	7.2	4.6	0.1681	2.3
4/21/2018 7:49			3.6	19.1	7.4	4.6	0.1686	2.3
4/21/2018 7:50			2.9	18.8	7.4	4.6	0.1744	2.2
4/21/2018 7:51			2.7	19	7.6	4.5	0.1637	2.2
4/21/2018 7:52			2.3	19.6	7.4	4.5	0.1748	2.2
4/21/2018 7:53			3.2	19.2	7.1	4.5	0.161	2.1
4/21/2018 7:54	0.5	-1.8	3.7	19.4	7.1	4.5	0.178	2.1
4/21/2018 7:55	0.4	-1.2	4.1	19.3	7.1	4.5	0.165	1.9
4/21/2018 7:56	0.4	-1.4	2.4	19.2	7	4.4	0.1658	2.1
4/21/2018 7:57	0.5	-1.1	3.7	19.2	7	4.4	0.1701	2.2
4/21/2018 7:58	0.5	-0.5	2.9	19.4	7	4.5	0.1559	2.3
4/21/2018 7:59	0.5	0.1	2.1	19.2	7.1	4.5	0.1764	2.3
4/21/2018 8:00	0.5	-0.4	2.6	19.2	7.1	4.5	0.1675	2.5
4/21/2018 8:01	0.5	0	3.6	19.2	6.8	4.5	0.1706	2.4
4/21/2018 8:02	0.4	0.5	2.8	19	6.7	4.5	0.1659	2.5
4/21/2018 8:03	0.4	0	3.2	19.3	6.6	4.5	0.1642	2.5
4/21/2018 8:04	0.4	4	13.9	44.3	9.4	4.7	0.1914	2.5
4/21/2018 8:05	0.4	6.5	59.3	186.7	62.6	14.1	6.221	3.5
4/21/2018 8:06	0.3	0.2	6.7	29.3	10.2	11.2	0.701	2.8
4/21/2018 8:07	0.3	0.1	7.9	33.6	12.6	15.7	0.667	2.6
4/21/2018 8:08	0.2	0.4	5.7	28.7	12.1	15.4	0.557	2.5
4/21/2018 8:09	0.1	0.1	5.5	27.4	12.1	15.5	0.5263	2.4
4/21/2018 8:10	0.2	0.4	5.3	25.9	11.7	15.5	0.4756	2.3
4/21/2018 8:11	0.2	0.7	5	25.7	11.6	15.5	0.4859	2.2
4/21/2018 8:12	0.2	0.7	5.3	25.3	11.5	15.5	0.4675	2.2
4/21/2018 8:13	0.2	-0.1	5.7	25.3	11.5	15.5	0.4764	1.9
4/21/2018 8:14	0.2	0.2	6	24.8	11.3	15.4	0.4563	1.9
4/21/2018 8:15	0.2	0.1	5.2	24	11	15.3	0.4446	1.9
4/21/2018 8:16	0.2	0.2	4.7	24.2	10.8	15.2	0.4316	1.9
4/21/2018 8:17	0.2	-0.1	5.4	23.6	10.7	15	0.4232	1.9
4/21/2018 8:18	0.3	0.6	4.4	24	10.7	14.8	0.4377	2
4/21/2018 8:19	0.3	0.8	4.2	23.9	10.7	14.6	0.4307	2
4/21/2018 8:20	0.2	0.2	5.1	23.8	10.5	14.5	0.4322	2.1
4/21/2018 8:21	0.3	-0.1	4.8	22.9	10.2	14.3	0.4191	2.2
4/21/2018 8:22	0.3	0.8	4.1	23	10	14	0.4114	2.2
4/21/2018 8:23	0.3	0.2	4.9	23.1	9.9	13.8	0.413	2.2
4/21/2018 8:24	0.3	0.7	2.5	23.2	9.9	13.7	0.4169	2.2
4/21/2018 8:25	0.3	1.4	4.2	23.1	9.8	13.5	0.4215	2.2

4/21/2018 8:26	0.3	-0.1	4.5	23.3	9.9	13.5	0.4136	2.3
4/21/2018 8:27	0.3	1.4	3.7	23	9.9	13.3	0.4212	2.3
4/21/2018 8:28	0.3	0.8	5.5	23	9.7	13.2	0.3973	2.3
4/21/2018 8:29	0.3	0.3	5.4	22.8	9.6	13.1	0.4046	2.3
4/21/2018 8:30	0.3	0.1	3.8	22.8	9.5	13.1	0.3982	2.3
4/21/2018 8:31	0.3	1.2	5.2	22.9	9.5	13.1	0.4025	2.3
4/21/2018 8:32	0.3	1.4	5.5	22.3	9.3	13	0.4026	2.4
4/21/2018 8:33	0.3	0.7	5	22.3	9.2	12.9	0.391	2.4
4/21/2018 8:34	0.3	0.3	5.2	22.3	9.3	12.9	0.4034	2.3
4/21/2018 8:35	0.2	0.5	4.6	21.8	8.9	12.7	0.3753	2.2
4/21/2018 8:36	0.2	0.1	4.1	22.2	9	12.6	0.3983	2.1
4/21/2018 8:37	0.2	0.6	4.8	22.6	9.2	12.5	0.3902	2.1
4/21/2018 8:38	0.2	1.2	4.4	22.1	9	12.4	0.3856	1.9
4/21/2018 8:39	0.3	0.6	4.5	22.2	9	12.3	0.3997	2
4/21/2018 8:40	0.3	0.9	4.4	22.1	9	12.3	0.3893	2.2
4/21/2018 8:41	0.3	0.5	4.6	22	8.8	12.3	0.3801	2.3
4/21/2018 8:42	0.3	0	6	22	8.7	12.3	0.3748	2.3
4/21/2018 8:43	0.3	0.3	4.5	21.9	8.5	12.2	0.3796	2.3
4/21/2018 8:44	0.3	0.5	4.6	21.3	8.5	12.2	0.3639	2.2
4/21/2018 8:45	0.4	0.4	4.1	21.5	8.3	12.1	0.3728	1.8
4/21/2018 8:46	0.3	0.5	5	21.4	8.2	11.9	0.37	1.6
4/21/2018 8:47	0.3	0.1	2.9	21.7	8.3	11.8	0.3684	1.6
4/21/2018 8:48	0.3	0.2	4.5	21.4	8.2	11.7	0.3745	1.6
4/21/2018 8:49	0.4	0.5	3.5	21.4	8	11.6	0.3535	1.6
4/21/2018 8:50	0.4	1.2	4.8	21.2	8.1	11.4	0.362	1.6
4/21/2018 8:51	0.4	0.7	4.2	21.1	7.9	11.3	0.3525	1.6
4/21/2018 8:52	0.4	0.2	5.2	21.2	7.9	11.2	0.3616	1.6
4/21/2018 8:53	0.4	1.1	4.1	21.2	7.8	11.1	0.353	1.6
4/21/2018 8:54	0.4	0.5	4	21.2	7.8	11	0.3507	1.6
4/21/2018 8:55	0.4	0.2	3.7	20.9	7.7	11	0.3587	1.6
4/21/2018 8:56	0.4	0.3	3.1	21.3	7.7	10.8	0.3456	1.6
4/21/2018 8:57	0.4	0.7	3.8	21	7.7	10.8	0.3557	1.6
4/21/2018 8:58	0.3	0.6	5.2	20.9	7.6	10.7	0.3364	1.7
4/21/2018 8:59	0.3	0.9	3.9	20.9	7.4	10.6	0.3353	1.6
4/21/2018 9:00	0.4	0.2	3.3	21	7.5	10.6	0.3457	1.6
4/21/2018 9:01	0.4	0.7	4.2	20.8	7.5	10.5	0.3386	1.7
4/21/2018 9:02	0.4	0.3	3.7	20.8	7.4	10.5	0.3519	1.7
4/21/2018 9:03	0.4	0.5	3.9	20.9	7.4	10.4	0.3355	1.7
4/21/2018 9:04	0.4	0.9	4.4	20.6	7.3	10.3	0.3341	1.6
4/21/2018 9:05	0.3	1.1	3.1	20.7	7.2	10.2	0.3283	1.7
4/21/2018 9:06	0.3	0.7	5.1	21	7.3	10.2	0.3315	1.7
4/21/2018 9:07	0.3	0.7	4.3	20.8	7.3	10.2	0.3388	1.7
4/21/2018 9:08	0.3	0.3	5.5	20.8	7.2	10.2	0.3257	1.7
4/21/2018 9:09	0.3	0.4	4	20.5	7.2	10.1	0.3291	1.7
4/21/2018 9:10	0.3	0.5	3.3	20.3	7.1	10.1	0.3152	1.7
4/21/2018 9:11	0.3	0.6	4	20.5	7.1	10	0.3405	1.6
4/21/2018 9:12	0.3	0.8	4.2	20.5	7.1	10	0.3284	1.6

4/21/2018 9:13	0.3	0.2	4.7	20.4	7	10	0.3273	1.7
4/21/2018 9:14	0.3	1.2	4.4	20.5	7	9.9	0.3349	1.7
4/21/2018 9:15	0.3	0.2	3.2	20.8	7	9.9	0.3213	1.7
4/21/2018 9:16	0.3	1.1	2.6	20.6	7	9.8	0.3329	1.7
4/21/2018 9:17	0.3	0.9	2.9	20.7	6.9	9.8	0.321	1.7
4/21/2018 9:18	0.3	-0.2	3.3	20.5	6.9	9.8	0.3303	1.7
4/21/2018 9:19	0.2	0.4	4.3	20.6	6.9	9.8	0.3241	1.7
4/21/2018 9:20	0.2	0.7	4	20.5	6.9	9.8	0.319	1.7
4/21/2018 9:21	0.3	0.6	3	20.3	7.1	9.7	0.3112	1.7
4/21/2018 9:22	0.4	2.1	3.2	20	7.8	9.5	0.2787	1.7
4/21/2018 9:23	0.6	1.7	4.6	19.8	8	9.2	0.2876	1.7
4/21/2018 9:24	0.8	1.2	2.7	19.8	8.3	8.8	0.2698	1.8
4/21/2018 9:25	0.9	0.6	3.8	19.7	8.4	8.4	0.2718	1.8
4/21/2018 9:26	1	1.4	3	19.9	8.7	7.9	0.2523	1.9
4/21/2018 9:27	1.1	1.6	5	19.4	9.1	7.5	0.238	2
4/21/2018 9:28	1.2	1.1	5.1	19.4	9.2	6.9	0.2468	2
4/21/2018 9:29	1.2	1.1	3.9	19.4	9.4	6.4	0.2324	1.9
4/21/2018 9:30	1.2	2.5	3.9	19.4	9.6	6.1	0.2405	1.9
4/21/2018 9:31	1.4	2.4	3.7	19.4	10	5.7	0.211	1.9
4/21/2018 9:32	1.6	2.8	4.2	19.7	10.2	5.3	0.2148	1.9
4/21/2018 9:33	1.7	2.2	1.4	19.2	10	5	0.2073	1.8
4/21/2018 9:34	1.8	2.6	4.3	19.8	10	4.7	0.2116	1.7
4/21/2018 9:35	1.8	2.4	2.9	19.2	10.6	4.5	0.2083	1.7
4/21/2018 9:36	1.8	2.6	3.1	19.5	10.6	4.3	0.201	1.7
4/21/2018 9:37	2	2.5	3.7	19.3	10.7	4.1	0.2071	1.8
4/21/2018 9:38	2	2.4	3.9	19.7	10.8	3.9	0.1911	1.9
4/21/2018 9:39	2.1	3	3.6	19.7	10.9	3.8	0.2015	1.8
4/21/2018 9:40	2.3	3	4.9	19.9	11	3.6	0.1846	1.7
4/21/2018 9:41	2.4	2.9	3.6	19.9	11.1	3.5	0.1948	1.8
4/21/2018 9:42	2.5	3	4	19.9	11.1	3.4	0.1886	1.9
4/21/2018 9:43	2.6	3.2	3.9	20.1	11	3.3	0.1858	2
4/21/2018 9:44	2.8	3.7	4.3	20.2	10.9	3.2	0.1928	2
4/21/2018 9:45	2.8	3.4	3	19.7	10.8	3.1	0.1844	2.1
4/21/2018 9:46	2.7	3.1	4.2	19.8	10.8	3.1	0.1954	2.2
4/21/2018 9:47	2.6	3.1	3.3	20	10.8	3	0.1828	2.2
4/21/2018 9:48	2.4	2.5	4.1	19.6	10.6	3	0.1972	2.1
4/21/2018 9:49	2.4	2.7	3.5	19.7	10.8	3	0.1806	2.1
4/21/2018 9:50	2.2	2.5	4.5	19.6	10.6	3	0.1854	2
4/21/2018 9:51	2.1	2.4	4.7	19.6	10.5	3	0.1814	2
4/21/2018 9:52	1.9	1.8	4	20.3	10.5	3.1	0.1919	2
4/21/2018 9:53	1.7	1.7	8.7	33.2	17	20	0.4884	2
4/21/2018 9:54	1.3	1.6	11.8	41.6	19.2	21	0.8031	2
4/21/2018 9:55	0.9	1	12.8	44.6	21.1	24.2	1.0109	2
4/21/2018 9:56	0.6	0.1	12.8	52.5	22.8	26.9	1.331	2.1
4/21/2018 9:57	0.4	0.3		49.2	23	27.6	1.3523	2.1
4/21/2018 9:58	0.3	0.6		48.3	22.4	27.5	1.3444	2
4/21/2018 9:59	0.2	0.7	19	50.2	22.6	27.4	1.3998	2.1

4/21/2018 10:00	0.2	0.5	13.2	49.7	23	27.5	1.4384	2.2
4/21/2018 10:01	0.2	0.8	15.2	49.8	22.8	27.5	1.4232	2.2
4/21/2018 10:02	0.2	0.6	13.8	48.9	22.9	27.5	1.4372	2.2
4/21/2018 10:03	0.2	0.9	14.6	47.1	22.3	27.5	1.3916	2
4/21/2018 10:04	0.2	0.2	14.1	46.7	21.8	27.4	1.3882	1.9
4/21/2018 10:05	0.2	0.6	14.1	45.8	21.6	27.3	1.3646	1.9
4/21/2018 10:06	0.2	0.5	12.3	45.3	21.6	27.2	1.3652	1.8
4/21/2018 10:07	0.1	0.5		44.8	21.2	27.1	1.3657	1.8
4/21/2018 10:08	0.2	1.2	12.5	43.7	20.6	26.9	1.3226	1.9
4/21/2018 10:09	0.2	0.6	13.6	43	20.4	26.6	1.3283	1.8
4/21/2018 10:10	0.2	0.4		42.9	20.3	26.3	1.3161	1.8
4/21/2018 10:11	0.2	0.6	14.2	41.8	20.2	26	1.3167	1.8
4/21/2018 10:12	0.2	0.3	12.7	40.9	19.6	25.8	1.2803	1.8
4/21/2018 10:13	0.2	0.3	12.6	40.9	19.4	25.6	1.2924	1.8
4/21/2018 10:14	0.2	0.8	12.5	40.4	19.3	25.4	1.2788	1.8
4/21/2018 10:15	0.3	0.2	10.5	38.7	18.8	25.1	1.2445	1.8
4/21/2018 10:16	0.3	0.4	11.3	38.9	18.2	24.8	1.2164	1.8
4/21/2018 10:17	0.3	0.7	12	37.8	18.3	24.6	1.2205	1.8
4/21/2018 10:18	0.3	0.8	11.6	37.7	17.9	24.4	1.2078	1.8
4/21/2018 10:19	0.3	0.6	11.8	37.5	18	24.2	1.2132	1.8
4/21/2018 10:20	0.3	0.1	10.7	37.5	17.8	24	1.2201	1.8
4/21/2018 10:21	0.2	0.6	10.8	36.1	17.8	23.7	1.1956	1.8
4/21/2018 10:22	0.2	0.4	10.9	36.3	17.7	23.5	1.1868	1.8
4/21/2018 10:23	0.3	0.5	10.5	35.2	16.9	23.3	1.17	1.9
4/21/2018 10:24	0.3	0.5	9.7	35.1	16.5	23.1	1.139	2
4/21/2018 10:25	0.3	0.7	11.1	34.9	16.5	22.9	1.1697	2
4/21/2018 10:26	0.3	0.2	9.9	34.2	16.2	22.6	1.1303	2
4/21/2018 10:27	0.3	1	10.7	34.3	16.1	22.4	1.1422	2.1
4/21/2018 10:28	0.3	1	10.7	33.4	15.9	22.1	1.1262	2.2
4/21/2018 10:29	0.3	0.4	9.7	32.9	15.7	21.9	1.123	2.3
4/21/2018 10:30	0.3	0.4	10.4	32.9	15.5	21.7	1.128	2.3
4/21/2018 10:31	0.2	1.3	9.4	32.9	15.2	21.4	1.0818	2.1
4/21/2018 10:32	0.2	0.8	9.6	32	15.2	21.2	1.1008	2
4/21/2018 10:33	0.2	1.3	10.3	32.2	14.9	21	1.0835	1.9
4/21/2018 10:34	0.2	1.2	10.5	31	14.7	20.8	1.0732	1.8
4/21/2018 10:35	0.2	0.9	10.5	31.3	14.5	20.6	1.0645	1.9
4/21/2018 10:36	0.2	0.3	9.3	30.9	14.3	20.4	1.039	1.9
4/21/2018 10:37	0.2	0.4	8.2	31.2	14.4	20.2	1.0847	1.9
4/21/2018 10:38	0.2	0.8	8.1	30.6	14.2	20	1.0547	1.9
4/21/2018 10:39	0.2	0.8	9.2	30.4	13.9	19.9	1.0443	1.9
4/21/2018 10:40	0.3	0.9	8	29.6	13.6	19.7	1.0157	1.8
4/21/2018 10:41	0.3	1.9	10.2	28.9	13.2	19.4	0.9828	1.8
4/21/2018 10:42	0.4	0.8	8.4	28.6	13	19.1	0.9698	1.9
4/21/2018 10:43	0.4	0.6	8.2	29.5	13	19	0.9889	1.8
4/21/2018 10:44	0.3	0.5	7.8	29.1	13.1	18.8	0.9998	1.9
4/21/2018 10:45	0.3	0.5	6.9	28.3	12.7	18.5	0.9568	1.9
4/21/2018 10:46	0.3	0.4	7.6	28	12.7	18.3	0.9746	1.9

4/21/2018 10:47	0.3	0.4	7.9	28.4	12.6	18.1	0.9582	1.8
4/21/2018 10:48	0.3	1	6.4	27.1	12.2	17.8	0.9242	1.8
4/21/2018 10:49	0.4	1.1	7.9	26.5	11.7	17.4	0.8893	1.9
4/21/2018 10:50	0.5	0.8	9.6	25.4	11.2	17.2	0.8358	1.9
4/21/2018 10:51	0.5	1.2	6.7	25.1	10.9	16.7	0.8222	1.9
4/21/2018 10:52	0.6	1.3	7.1	25.5	10.9	16.1	0.8244	2
4/21/2018 10:53	0.7	1.2	7.4	24.7	10.7	15.5	0.7963	2
4/21/2018 10:54	0.7	1.1	8.4	25.8	10.7	15.1	0.8063	2
4/21/2018 10:55	0.6	0.7	7.9	25.9	11.2	14.8	0.8624	2.1
4/21/2018 10:56	0.6	1.2	7.1	25.8	11.1	14.5	0.8432	2.1
4/21/2018 10:57	0.5	1.2	6.8	25.2	10.9	14.3	0.8142	2.2
4/21/2018 10:58	0.5	1.7	7.7	26.1	10.9	14.2	0.8493	2
4/21/2018 10:59	0.4	0.4	7.5	26.2	11.2	14.3	0.8623	2.1
4/21/2018 11:00	0.3	0.6	6.8	26.8	11.4	14.5	0.9108	2.2
4/21/2018 11:01	0.3	0.5	7	26.6	11.4	14.7	0.8987	2.3
4/21/2018 11:02	0.2	0	6.9	25	11.2	14.9	0.885	2.3
4/21/2018 11:03	0.3	1.7	8.1	24.9	10.5	14.8	0.8137	2.2
4/21/2018 11:04	0.3	1	6.8	24.7	10.4	14.7	0.8004	2
4/21/2018 11:05	0.5	1.1	7.8	24.5	10.3	14.6	0.8008	2
4/21/2018 11:06	0.5	0.7	5.6	24	10.1	14.5	0.7703	2.1
4/21/2018 11:07	0.5	1.1	6.8	24.5	10	14.3	0.7673	2.2
4/21/2018 11:08	0.5	1.2	5.6	24.3	10	14	0.7733	2.3
4/21/2018 11:09	0.5	1	6.7	24.8	10.1	13.7	0.7905	2.2
4/21/2018 11:10	0.8	1.7	5.8	23.2	9.6	13.2	0.6593	1.9
4/21/2018 11:11	1.1	2.3	6.9	22.9	9.6	12.6	0.6036	2
4/21/2018 11:12	1.5	2.2	6.7	23.7	9.4	11.9	0.5909	2
4/21/2018 11:13	1.5	2.4	7.1	22.9	9.9	11.4	0.5678	2
4/21/2018 11:14	1.5	3.3	5.1	23	10.5	10.8	0.6115	2
4/21/2018 11:15	1.4	2.5	4.9	22.8	10.6	10.2	0.5734	2
4/21/2018 11:16	1.5	1.9	5.8	22.7	10.4	9.5	0.5463	2.1
4/21/2018 11:17	1.6	2.4	4.3	21.9	10.4	8.8	0.5058	2.1
4/21/2018 11:18	1.8	3	5.5	21.9	10.4	7.9	0.4665	2.2
4/21/2018 11:19	2	3.2	5.6	21.4	10.3	7.3	0.443	2.2
4/21/2018 11:20	2.2	3.4	5.8	21.5	10.2	6.9	0.4158	2.2
4/21/2018 11:21	2.4	3.1	5.5	21.3	10.1	6.3	0.4166	2
4/21/2018 11:22	2.5	3.6	6.6	21.5	10.1	5.7	0.3856	1.9
4/21/2018 11:23	2.6	3.2	5.7	21.6	10.1	5.2	0.3873	1.9
4/21/2018 11:24	2.7	3.7	4.7	21.3	10	4.8	0.3593	2
4/21/2018 11:25	2.9	3.8	6.5	21.3	10	4.4	0.352	2.1
4/21/2018 11:26	3.1	4.1	6.5	21.3	9.9	4.1	0.3331	2.1
4/21/2018 11:27	3.2	4	6.5	21.4	9.8	3.8	0.3299	2.1
4/21/2018 11:28	3.4	4.6	5.4	21.5	9.5	3.6	0.3031	2.2
4/21/2018 11:29	3.5	4.4	4.6	21.4	9.5	3.4	0.3056	2.2
4/21/2018 11:30	3.7	4.5	5.6	21.7	9.3	3.2	0.2943	2.2
4/21/2018 11:31	4	5.2	6.1	21.8	9.1	3.1	0.2788	2.2
4/21/2018 11:32	4.2	5.1	6.7	22.1	9	2.9	0.2772	2.1
4/21/2018 11:33	4.5	5.7	6	22.4	8.8	2.8	0.2534	2.1

4/21/2018 11:34	4.7	6.5	6.4	22.7	8.7	2.7	0.2603	2.1
4/21/2018 11:35	5.1	6.7	5.5	22.8	8.5	2.5	0.2485	2.1
4/21/2018 11:36	5.4	6.5	6.3	23.2	8.1	2.5	0.2404	2.1
4/21/2018 11:37	5.6	6.5	5.6	22.7	8.3	2.4	0.2392	2.1
4/21/2018 11:38	5.9	7.9	7.6	23.5	8.1	2.3	0.2322	2.2
4/21/2018 11:39	6.1	7.5	6.4	23.9	7.9	2.3	0.2237	2.2
4/21/2018 11:40	6.5	7.5	7.6	24	7.8	2.2	0.223	2.2
4/21/2018 11:41	6.8	7.4	7.4	23.9	7.7	2.1	0.2295	2
4/21/2018 11:42	7.1	8.9	8.2	25.3	7.1	2.1	0.2065	2.1
4/21/2018 11:43	8.4	14	10	28.7	6.3	2.1	0.2073	2
4/21/2018 11:44	10.5	14.3	10.4	29.8	6	2	0.1726	2
4/21/2018 11:45	12.6	15.1	9.3	30.6	6.2	1.9	0.1814	2
4/21/2018 11:46	13.1	12.1	8.9	28	6.3	1.8	0.1901	2
4/21/2018 11:47	12	10.6	7.5	26.7	6.6	1.8	0.1952	2
4/21/2018 11:48	10.5	9.9	7.3	26.2	6.7	1.8	0.2067	2.1
4/21/2018 11:49	9.5	9.4	7	25.9	6.7	1.8	0.1861	2
4/21/2018 11:50	9.1	9.1	8.8	26	6.7	1.8	0.2001	2.1
4/21/2018 11:51	8.9	9.1	6.8	25.5	6.7	1.8	0.1896	2.1
4/21/2018 11:52	8.5	8	8.9	25.4	6.7	1.9	0.2079	2.2
4/21/2018 11:53	7.8	7.1	9.5	31.9	11.5	7.4	0.3264	2.1
4/21/2018 11:54	6.4	4.7	13	49.5	21.8	18.9	0.7136	2.3
4/21/2018 11:55	4.3	2.8	16.2	59	31.5	28.1	1.1576	2.4
4/21/2018 11:56	2.4	1.2	17.8	63.1	33.6	29.6	1.4353	2.5
4/21/2018 11:57	1.2	1.5	16.3	64.3	34.2	30.1	1.4902	2.4
4/21/2018 11:58	0.8	0.9	17.2	65.6	33.9	29.7	1.5655	2.5
4/21/2018 11:59	0.6	0.5	16.2	63.9	33.2	29.7	1.5843	2.5
4/21/2018 12:00	0.5	0.7	18.8	61.6	33	29.5	1.5637	2.5
4/21/2018 12:01	0.5	1	15.2	60.7	32.5	29.4	1.5635	2.5
4/21/2018 12:02	0.5	0.8	16.4	57.2	31.2	29.3	1.5133	2.6
4/21/2018 12:03	0.6	1.6	14.9	56.3	30.6	29	1.4652	2.4
4/21/2018 12:04	0.6	0.8	14.1	52.7	29	28.4	1.395	2.4
4/21/2018 12:05	0.6	1.5	13.6	53.9	28.7	27.9	1.4008	2.5
4/21/2018 12:06	0.6	0.9	15	52	28.5	27.4	1.3835	2.6
4/21/2018 12:07	0.6	1	14.6	49.9	27.8	26.8	1.3608	2.6
4/21/2018 12:08	0.6	1	13.3	50.1	27.8	26.2	1.359	2.5
4/21/2018 12:09	0.7	1.4	13.6	49.8	27.4	25.7	1.3532	2.4
4/21/2018 12:10	0.6	1.1	14.1	50.9	27.7	25.1	1.3822	2.4
4/21/2018 12:11	0.6	0.5	14.8	49.6	27.8	24.7	1.3918	2.4
4/21/2018 12:12	0.5	0.2	13.2	47.7	27.1	24.3	1.3423	2.5
4/21/2018 12:13	0.5	1.2	13.2	46.7	26.3	24.1	1.3212	2.3
4/21/2018 12:14	0.6	1.6	13.4	45.3	25.5	23.8	1.2903	2.1
4/21/2018 12:15	0.6	1.3	13.9	44.9	25.4	23.5	1.29	2.3
4/21/2018 12:16	0.6	0.5	13.3	44.3	25.1	23.3	1.2787	2.4
4/21/2018 12:17	0.7	1.6	11.8	43.5	24.4	23	1.2605	2.4
4/21/2018 12:18	0.6	1.1	11.2	44.1	25.1	22.8	1.3028	2.5
4/21/2018 12:19	0.6	0.8	12.1	43.3	24.6	22.5	1.2652	2.6
4/21/2018 12:20	0.5	0.9	12.4	42.5	24.5	22.2	1.2815	2.6

4/21/2018 12:21	0.5	1.8	12.8	41.7	23.8	21.9	1.234	2.6
4/21/2018 12:22	0.4	0.9	12.4	40.8	23.4	21.7	1.2367	2.6
4/21/2018 12:23	0.5	0.9	12.2	39.6	23.4	21.4	1.2067	2.5
4/21/2018 12:24	0.5	1.3	11.1	39.4	23.3	21.1	1.2063	2.3
4/21/2018 12:25	0.6	1.4	10.9	38.7	22.2	20.9	1.1847	2.4
4/21/2018 12:26	0.6	1.4	11.1	38.3	21.9	20.6	1.1742	2.6
4/21/2018 12:27	0.6	1.1	10.1	38.4	21.8	20.3	1.1731	2.6
4/21/2018 12:28	0.6	1	11.9	38.5	22	20.1	1.189	2.7
4/21/2018 12:29	0.6	1.2	12.2	37.8	21.4	19.8	1.1714	2.6
4/21/2018 12:30	0.5	1.7	10.7	37.3	21.2	19.7	1.157	2.6
4/21/2018 12:31	0.4	1.3	10.1	36.8	21.2	19.5	1.1553	2.6
4/21/2018 12:32	0.4	1.1	10.4	36.4	20.9	19.2	1.1567	2.6
4/21/2018 12:33	0.5	0.6	11.8	35.6	20.5	19.1	1.1275	2.6
4/21/2018 12:34	0.5	1.1	10	34.7	19.9	18.9	1.1047	2.6
4/21/2018 12:35	0.5	1.3	10.8	35.4	20	18.7	1.1133	2.6
4/21/2018 12:36	0.5	0.6	11.1	34.4	19.8	18.4	1.1142	2.6
4/21/2018 12:37	0.6	1.5	10.2	33.2	18.8	18.2	1.0475	2.7
4/21/2018 12:38	0.7	1.5	9.3	32.5	18.9	17.9	1.0458	2.7
4/21/2018 12:39	0.9	1.8	7.7	30.2	17.2	17.3	0.9651	2.6
4/21/2018 12:40	1.1	2.2	9.7	30.2	16.4	16.8	0.9273	2.6
4/21/2018 12:41	1.3	1.4	9.8	29.7	16.3	16.3	0.9263	2.6
4/21/2018 12:42	1.4	2.2	8.8	28.9	15.5	15.7	0.8697	2.7
4/21/2018 12:43	1.4	2.1	8.9	29.9	15.3	15	0.8889	2.7
4/21/2018 12:44	1.5	1.6	9.4	29.6	15.5	14.5	0.8836	2.7
4/21/2018 12:45	1.3	1.1	8.6	29.8	16	14	0.9246	2.8
4/21/2018 12:46	1.1	1.8	9.4	30.1	16.2	13.6	0.9344	2.8
4/21/2018 12:47	1	1.4	9	29.7	16.5	13.4	0.9517	2.4
4/21/2018 12:48	0.9	1	10.2	30.6	16.4	13.3	0.9527	2.6
4/21/2018 12:49	0.8	1.3	9.4	30.4	17	13.3	0.9749	2.8
4/21/2018 12:50	0.7	1.7	9.9	30.6	16.9	13.5	0.985	2.9
4/21/2018 12:51	0.5	0.7	9.2	30.4	17.2	13.7	0.9924	2.8
4/21/2018 12:52	0.4	0.7	7.9	30.6	16.9	13.9	0.9987	2.8
4/21/2018 12:53	0.3	0.8	9.7	30.7	17.1	14	0.9942	3
4/21/2018 12:54	0.3	1.1	9.6	30	16.7	14.1	0.988	2.9
4/21/2018 12:55	0.3	0.7	8	29.9	16.5	14.2	0.9693	2.7
4/21/2018 12:56	0.3	0.5	9.2	29.8	16.4	14.2	0.9721	2.7
4/21/2018 12:57	0.3	0.6	8	29.3	16.2	14.1	0.9684	2.7
4/21/2018 12:58	0.3	0.8	8.5	29.2	16	14.1	0.9472	2.4
4/21/2018 12:59	0.3	1.1	8.4	28.8	15.7	13.9	0.9415	2.4
4/21/2018 13:00	0.4	1.3	8	29.2	15.5	13.8	0.9327	2.3
4/21/2018 13:01	0.4	0.9	7.6	28.4	15.4	13.6	0.9347	2.4
4/21/2018 13:02	0.5	1.3	7.1	27.2	14.7	13.4	0.8716	2.5
4/21/2018 13:03	0.7	2	7.6	26.6	13.9	13	0.8355	2.1
4/21/2018 13:04	1.1	1.9	6.7	25.4	12.6	12.4	0.7117	1.7
4/21/2018 13:05	1.3	1.6	7.3	25.3	13	12	0.7512	1.6
4/21/2018 13:06	1.6	2.7	6.9	24.2	12.1	11.4	0.6622	1.6
4/21/2018 13:07	1.8	2.6	4.8	24.8	11.9	10.8	0.6557	1.6

4/21/2018 13:08	2	3.3	7.8	24.2	11.7	10.3	0.657	1.6
4/21/2018 13:09	2.8	6.3	6.7	26.6	10.9	9.5	0.5666	1.6
4/21/2018 13:10	3.9	6.9	7.3	27.2	11.5	8.8	0.5718	1.6
4/21/2018 13:11	5.2	7.9	8.6	26.9	10.6	8	0.4924	1.6
4/21/2018 13:12	5.9	7.2	8.4	27	10.4	7.5	0.4815	1.6
4/21/2018 13:13	6	7.1	8	26.6	10.8	7	0.5081	1.6
4/21/2018 13:14	6	8.2	8.2	26.7	10.3	6.3	0.457	1.6
4/21/2018 13:15	6.2	8.3	7.3	26.7	9.9	5.9	0.4313	1.6
4/21/2018 13:16	6.6	7.9	9	26.4	10	5.3	0.4197	1.6
4/21/2018 13:17	7.1	9	8.7	27.1	9.2	4.8	0.3881	1.5
4/21/2018 13:18	7.4	9.2	7.8	26.8	9.1	4.3	0.377	1.5
4/21/2018 13:19	7.7	9.9	8.5	27	8.6	4	0.3572	1.5
4/21/2018 13:20	8.2	10.8	9.4	27.6	8	3.7	0.3147	1.5
4/21/2018 13:21	9	11.9		28.3	7.4	3.3	0.3055	1.5
4/21/2018 13:22	9.7	11.9		28.1	7.3	3.1	0.2935	1.5
4/21/2018 13:23	10.1	11.5		28.3	7.2	2.8	0.2907	1.5
4/21/2018 13:24	10.2	12	9.4	28.3	7.2	2.6	0.282	1.5
4/21/2018 13:25	10.2	12		28.7	7.2	2.4	0.2648	1.5
4/21/2018 13:26	10.8	13.3	8.8	29	6.2	2.3	0.2489	1.5
4/21/2018 13:27	10.8	11.4	8.9	28.2	6.2	2.2	0.2672	1.5
4/21/2018 13:28	11	13	15.9	29.7	5.9	2.1	0.2576	1.5
4/21/2018 13:29	11.2	13		29.5	5.5	2	0.2277	1.5
4/21/2018 13:30	11.7	12.9	10.1	29.2	5.6	2	0.2435	1.5
4/21/2018 13:31	11.8	13.4		29.1	5.4	1.9	0.226	1.6
4/21/2018 13:32	11.9	14.1	10.4	29.6	5.2	1.9	0.2339	1.6
4/21/2018 13:33	12.3	14.7	9.9	30.1	4.8	1.9	0.208	1.6
4/21/2018 13:34	12.6	13.6	10.1	29.5	4.8	1.8	0.2096	1.5
4/21/2018 13:35	12.5	12.8	11.1	29.3	4.8	1.8	0.2209	1.5
4/21/2018 13:36	12.5	14.4	10.9	30.3	4.5	1.8	0.1933	1.5
4/21/2018 13:37	12.8	15.2	10.7	30.6	4.4	1.7	0.1965	1.5
4/21/2018 13:38	13.5	15.6	10.3	30.9	4.2	1.7	0.1796	1.5
4/21/2018 13:39	13.9	16.5	10.4	31.3	4.1	1.6	0.1928	1.5
4/21/2018 13:40	14.1	15.3	10.7	30.8	4.1	1.6	0.17	1.5
4/21/2018 13:41	14.2	15.2	9.7	30.8	4	1.6	0.1843	1.5
4/21/2018 13:42	14.5	15.9	11.3	31.8	3.8	1.5	0.1743	1.6
4/21/2018 13:43	14.7	15.6	10.5	31.5	3.8	1.5	0.1767	1.7
4/21/2018 13:44	14.9	16.1	10.4	31.5	3.7	1.5	0.1694	1.6
4/21/2018 13:45	14.9	15.7	10.3	31.5	3.7	1.5	0.168	1.5
4/21/2018 13:46	15	16.3	12.1	31.7	3.7	1.4	0.1759	1.5
4/21/2018 13:47	15.1	15.7	9.8	31.9	3.6	1.4	0.1558	1.5
4/21/2018 13:48	15.2	15.9	10.5	31.8	3.6	1.4	0.166	1.5
4/21/2018 13:49	15	15.4	10.6	31.5	3.6	1.4	0.1572	1.5
4/21/2018 13:50	14.7	15.6	10.6	31.7	3.6	1.4	0.169	1.5
4/21/2018 13:51	14.7	16.3	10.2	32.1	3.5	1.4	0.1583	1.6
4/21/2018 13:52	15.1	16.1	11.7	32.4	3.4	1.4	0.1569	1.6
4/21/2018 13:53	15.4	16.5	9.6	32.3	3.4	1.4	0.1597	1.6
4/21/2018 13:54	15.5	16.5	11.3	31.5	3.4	1.4	0.1525	1.6

4/21/2018 13:55	15.5	17.5	10.1	32.9	3.3	1.4	0.1576	1.7
4/21/2018 13:56	16.1	19.7	12.2	34	3.2	1.4	0.143	1.6
4/21/2018 13:57	17.2	20	13.6	34.4	3.2	1.4	0.1553	1.6
4/21/2018 13:58	18.2	20.6	13.2	34.6	3.2	1.4	0.1453	1.6
4/21/2018 13:59	18.6	19.8	12.5	35.1	3.2	1.3	0.1545	1.6
4/21/2018 14:00	18.3	18.3	12.2	33.1	3.3	1.3	0.1538	1.6
4/21/2018 14:01	18	19.5	11.3	34.4	3.2	1.3	0.1477	1.5
4/21/2018 14:02	17.9	19.7	12	34.2	3.1	1.3	0.1514	1.5
4/21/2018 14:03	17.8	19.1	11.8	34.2	3.2	1.3	0.1331	1.5
4/21/2018 14:04	18.3	20.5	12.1	35.1	3.1	1.3	0.1472	1.6
4/21/2018 14:05	18.4	20.3	12	35.6	3.1	1.3	0.1384	1.6
4/21/2018 14:06	19	20.4	12	35.3	3.2	1.3	0.1465	1.5
4/21/2018 14:07	19.2	20.1	12.1	35.5	3.1	1.3	0.1386	1.5
4/21/2018 14:08	18.6	17.1	11.4	33	3.2	1.3	0.1473	1.5
4/21/2018 14:09	17.7	16.5	10.2	32.4	3.2	1.3	0.1457	1.5
4/21/2018 14:10	16.5	16	11.6	32.1	3.2	1.3	0.1444	1.5
4/21/2018 14:11	15.7	15.1	10.6	31.4	3.2	1.4	0.1505	1.4
4/21/2018 14:12	15.2	15.9	9.5	31.6	3.2	1.4	0.1383	1.5
4/21/2018 14:13	14.8	15.5	10.6	31	3.2	1.5	0.1518	1.5
4/21/2018 14:14	14.5	15	10	30.9	3.2	1.5	0.1378	1.5
4/21/2018 14:15	13.8	12.5	9.4	29	4.6	1.9	0.1623	1.5
4/21/2018 14:16	12.1	8.4	6.9	33.1	14.4	14.2	0.3205	1.5
4/21/2018 14:17	8.8	4	5	32.5	33.7	33.8	0.5662	1.6
4/21/2018 14:18	5	2	4.3	41.8	40	41.5	0.8948	1.6
4/21/2018 14:19	2.3	2.1	4.5	46.9	42.9	46.2	1.2918	1.7
4/21/2018 14:20	1.2	0.7	5.2	45.9	43.5	48.2	1.4691	1.7
4/21/2018 14:21	1	1.1	4.6	43.2	40.5	44.6	1.4103	1.7
4/21/2018 14:22	1.3	2.7	4.9	44.1	35.7	40.3	1.4726	1.7
4/21/2018 14:23	1.6	3	5.5	42.4	35	36.9	1.5433	1.7
4/21/2018 14:24	2.1	3.6	5.4	42.6	32.6	35	1.5722	1.7
4/21/2018 14:25	2.3	2.8	5.9	43.2	32.4	34.9	1.9015	1.7
4/21/2018 14:26	2.2	2.5	4.5	42.2	32.4	36.1	1.9492	1.7
4/21/2018 14:27	1.9	1.5	4.7	42.6	34.4	39.6	2.0537	1.7
4/21/2018 14:28	1.7	2.7	5.3	39.6	32.7	35.3	1.732	1.7
4/21/2018 14:29	1.6	2	5.3	40.1	33.1	34.8	1.8662	1.7
4/21/2018 14:30	1.6	1.6	3.9	38.6	32.7	36.7	1.8843	1.7
4/21/2018 14:31	1.6	2.2	4.8	39.2	32.7	36.6	1.8177	1.7
4/21/2018 14:32	1.4	1.1	4.6	40.9	34.9	36.7	2.0037	1.7
4/21/2018 14:33	1	0.3	4.6	40.3	36.7	37	2.0937	1.7
4/21/2018 14:34	0.5	0.4	2.5	40.5	36.4	37.3	2.0628	1.7
4/21/2018 14:35	0.3	0.8	4.5	39.7	36.3	37.6	2.0617	1.7
4/21/2018 14:36	0.3	1	3.9	38.8	35.8	37.8	2.0198	1.7
4/21/2018 14:37	0.3	1.2	4.3	37.5	34.6	37.9	1.932	1.7
4/21/2018 14:38	0.4	1	4.6	36.8	33.7	37.8	1.9033	1.7
4/21/2018 14:39	0.4	1.3	4	36.3	33.7	38	1.8937	1.7
4/21/2018 14:40	0.5	0.5	3.9	35.5	32.5	37.8	1.829	1.7
4/21/2018 14:41	0.5	0.5	4.4	34.8	32.1	37.2	1.7943	1.7

4/21/2018 14:42	0.7	1.1	4.1	33.3	30.3	36.3	1.6825	1.7
4/21/2018 14:43	0.8	0.8	4.9	33.1	30.2	35.4	1.6912	1.7
4/21/2018 14:44	0.9	1.4	4.1	32.5	29.8	34.4	1.659	1.7
4/21/2018 14:45	0.9	1.3	4.9	32.5	29.1	33.4	1.6492	1.7
4/21/2018 14:46	0.8	0.6	3.2	31.5	29.1	32.7	1.6178	1.7
4/21/2018 14:47	1	1.4	2.9	29.4	26.7	31.6	1.4962	1.7
4/21/2018 14:48	1.2	1.5	4.4	29.3	25.3	30.4	1.423	1.7
4/21/2018 14:49	1.5	2.2	3.4	29.9	25.3	29.4	1.4387	1.6
4/21/2018 14:50	1.5	2	4.9	30	26.7	28.8	1.4927	1.6
4/21/2018 14:51	1.7	3	3.1	28	23.5	25.4	1.2867	1.6
4/21/2018 14:52	1.7	2.1	3.5	28.9	24	23.8	1.3743	1.7
4/21/2018 14:53	1.9	2.5	4.3	28.5	23.9	23.5	1.3433	1.7
4/21/2018 14:54	1.8	2.5	2.7	28.5	23.5	23.8	1.346	1.7
4/21/2018 14:55	1.7	2.1	4.3	28.2	23.8	23.2	1.3303	1.8
4/21/2018 14:56	1.5	2	3.9	28.9	24.9	23.4	1.424	1.8
4/21/2018 14:57	1.4	2.2	5.1	27.8	23.9	23.4	1.3393	1.8
4/21/2018 14:58	1.5	2	4.7	27.5	23	23.2	1.3125	1.9
4/21/2018 14:59	1.5	2	3	27.5	23.8	23.1	1.3518	1.9
4/21/2018 15:00	1.3	0.6	3.6	28	24.4	23.3	1.4008	1.8
4/21/2018 15:01	1	0.2	4.7	27.9	25.1	23.4	1.436	1.8
4/21/2018 15:02	0.7	0.8	2.6	27.9	25	23.5	1.4197	1.8
4/21/2018 15:03	0.6	1.3	3.1	27.4	25.1	23.7	1.441	1.8
4/21/2018 15:04	0.5	1	3.9	27.1	24.4	23.7	1.3942	1.7
4/21/2018 15:05	0.4	1.3	2.9	27.3	24.4	23.6	1.4225	1.7
4/21/2018 15:06	0.4	1.5	3.2	27	24	23.7	1.3743	1.7
4/21/2018 15:07	0.5	0.7	4.5	26.9	23.6	23.8	1.3753	1.7
4/21/2018 15:08	0.6	1.3	3.3	26.6	23.3	23.7	1.3462	1.7
4/21/2018 15:09	0.8	1.5	3.5	26	21.9	23.2	1.2428	1.8
4/21/2018 15:10	1	1.1	3.3	26.1	21.8	22.8	1.2527	1.8
4/21/2018 15:11	1.1	1.2	4.2	25.6	21.7	22.3	1.2268	1.8
4/21/2018 15:12	1.1	1.3	3.8	25.8	21.3	21.7	1.2455	1.7
4/21/2018 15:13	1	1.3	2.2	25.6	21.6	21.2	1.2395	1.8
4/21/2018 15:14	0.9	1	1.7	25.6	21.6	20.7	1.2597	1.9
4/21/2018 15:15	0.9	1.2	4.7	25.2	20.8	20.3	1.192	2
4/21/2018 15:16	1	1.8	3.8	24.7	20.2	19.7	1.1557	2.1
4/21/2018 15:17	1.3	2	4.6	24.6	18.8	19.1	1.084	2
4/21/2018 15:18	1.7	2.8	3.9	24.3	17.8	18.4	1.0244	2
4/21/2018 15:19	1.9	2.4	4.1	24.7	18.6	17.9	1.081	2.1
4/21/2018 15:20	2	2.7	5.2	24.5	18	17.4	1.0335	2.2
4/21/2018 15:21	2	2.3	4.7	24	17.2	16.8	1.0004	2.2
4/21/2018 15:22	1.9	2.6	3.9	24	18.1	16.3	1.0492	2.2
4/21/2018 15:23	1.9	2.3	4.2	24	18	15.8	1.0487	2.2
4/21/2018 15:24	1.7	1.8	3.4	24.5	17.9	15.4	1.0358	2
4/21/2018 15:25	1.5	1.6	3.5	24.1	18.4	15.2	1.0727	1.8
4/21/2018 15:26	1.4	1.2	3.9	24	18.3	15.2	1.067	1.9
4/21/2018 15:27	1.3	1.7	4	23.8	18.3	15.1	1.0553	2.1
4/21/2018 15:28	1.4	2.4	4.7	23.5	17.9	15	1.0335	2.2

4/21/2018 15:29	1.3	0.9	2.7	23.9	17.9	15	1.0588	2.3
4/21/2018 15:30	1.1	0.7	3.8	23.9	18.9	15.1	1.1083	2.3
4/21/2018 15:31	0.8	0.8	5	23.7	18.8	15.1	1.0862	2.3
4/21/2018 15:32	0.6	0.5	4	23.5	18.7	15.2	1.0927	2.3
4/21/2018 15:33	0.6	1.5	4.1	23.5	18.4	15.1	1.0747	2.2
4/21/2018 15:34	0.8	1.5	4.4	23.3	17.6	14.9	1.0271	2.1
4/21/2018 15:35	0.8	1.9	3.5	23.4	17.7	14.9	1.0312	2.2
4/21/2018 15:36	1	1.6	4.2	23.3	16.8	14.7	0.9633	2.3
4/21/2018 15:37	1.2	2	4.7	23.1	16.5	14.5	0.9489	2.3
4/21/2018 15:38	1.8	3.6	4.8	24.4	14.9	14.1	0.845	2.3
4/21/2018 15:39	3.9	10	7.5	27.5	11.6	13.2	0.6311	2.3
4/21/2018 15:40	6.6	10.9	6.7	28.4	10.5	12.1	0.5687	2.3
4/21/2018 15:41	9	10.9	8.5	28.5	10.5	11	0.5481	2.3
4/21/2018 15:42	10.2	11.8	8.7	28.5	9.6	10	0.4835	2.4
4/21/2018 15:43	10.7	12	7.8	29.4	9.5	8.8	0.4662	2.3
4/21/2018 15:44	11.4	13	8.8	29.3	9.1	7.6	0.4386	2.3
4/21/2018 15:45	12.2	14	10	31.1	8.5	6.4	0.3901	2.3
4/21/2018 15:46	13	14.7	10.5	31.2	7.9	5.3	0.3857	2.2
4/21/2018 15:47	13.9	14.5	9.7	31.5	7.4	4.3	0.332	2.2
4/21/2018 15:48	14.3	15.6	9.4	31.5	7.1	3.8	0.3395	2.3
4/21/2018 15:49	14.6	15.6	9.8	31.5	7	3.3	0.3197	2.3
4/21/2018 15:50	14.9	16.6	9.9	32.7	6.6	2.9	0.3158	2.4
4/21/2018 15:51	15.4	16.5	9.7	32.8	6.3	2.6	0.296	2.3
4/21/2018 15:52	16	17.4	10.5	33.1	5.8	2.3	0.2669	2.2
4/21/2018 15:53	16.5	17.7	10.3	33.7	5.7	2.1	0.2662	1.7
4/21/2018 15:54	17.5	19.6	13.1	35.1	5.4	2	0.2451	1.6
4/21/2018 15:55	18	18.6	11.8	34.4	5.1	1.9	0.2644	1.7
4/21/2018 15:56	18.3	18.8	10.9	34.1	4.8	1.8	0.229	1.7
4/21/2018 15:57	18.1	19.1	11	34.7	4.7	1.7	0.2417	1.7
4/21/2018 15:58	18.2	19.1	12.3	34.7	4.7	1.6	0.2305	1.7
4/21/2018 15:59	18	18	11.2	33.9	4.8	1.6	0.2417	1.7
4/21/2018 16:00	18.2	19.1	12.8	34.5	4.3	1.6	0.2011	1.7
4/21/2018 16:01	18.3	19	12.4	35.2	4.3	1.5	0.2242	1.7
4/21/2018 16:02	19	20.3	11.8	35.9	4	1.5	0.1963	1.8
4/21/2018 16:03	19.3	20.3	11.9	35.4	3.9	1.5	0.185	1.8
4/21/2018 16:04	19.2	18.2	11	34	4	1.5	0.2114	1.7
4/21/2018 16:05	18.3	16.9	11.3	33.2	4.2	1.5	0.2065	1.8
4/21/2018 16:06	17.3	15.9	11.1	32.1	4.3	1.5	0.2167	1.9
4/21/2018 16:07	16.2	15.8	10.5	31.6	4.4	1.5	0.2084	1.8
4/21/2018 16:08	15.5	15.5	11.3	31.8	4.4	1.5	0.2263	1.8
4/21/2018 16:09	14.9	14.4	8.5	31.3	4.4	1.6	0.2213	1.9
4/21/2018 16:10	14.5	13.6	9.1	29.9	6.3	2.7	0.2326	2
4/21/2018 16:11	11.9	5.5	4.1	27	37.8	35.5	0.4587	2.1
4/21/2018 16:12	7.5	2.1	4.9	42.1	49.2	79.8	1.0675	2.2
4/21/2018 16:13	3.3	1.6	5.4	58.6	49.2	85.2	1.3873	2.4
4/21/2018 16:14	1.1	1.6	6.5	81.2	54	99.7	1.8462	2.6
4/21/2018 16:15	0.8	0.9		91.9	53.7	97.9	2.3527	2.7

4/21/2018 16:16	0.7	0.8		96.1	55.1	104	2.5805	2.7
4/21/2018 16:17	0.5	0.8		101.3	55.8	104.5	2.9293	2.9
4/21/2018 16:18	0.5	1.1	7.8	99.1	52.5	97	2.9113	2.8
4/21/2018 16:19	0.4	0.4	10.3	98.1	52	97.3	3.0443	2.8
4/21/2018 16:20	0.5	0.6		91.5	48.2	91.1	2.884	2.8
4/21/2018 16:21	0.5	0.2		92.5	47.6	93.2	2.994	2.9
4/21/2018 16:22	0.5	0.5	14.7	85.1	47.5	91.9	3.2147	2.9
4/21/2018 16:23	0.5	0.2		85.9	45.7	90.1	3.1807	2.9
4/21/2018 16:24	0.5	1.4	5.6	81.4	44.8	85.6	3.2073	2.9
4/21/2018 16:25	0.5	0.9	7	82.5	45	88.2	3.318	2.9
4/21/2018 16:26	0.5	0.8	6.4	83.8	45.9	93.1	3.5028	2.9
4/21/2018 16:27	0.5	1.5	12.3	78.2	45.6	87.6	3.3389	2.6
4/21/2018 16:28	0.4	0.8	6.2	78.2	45.6	85.5	3.4208	2.5
4/21/2018 16:29	0.4	0.8	5.1	73.8	44.4	82.7	3.3228	2.5
4/21/2018 16:30	0.4	0.3	7.6	72.9	42.4	84.1	3.3628	2.6
4/21/2018 16:31	0.5	0.4	6.3	70.3	40.6	81.4	3.2603	2.5
4/21/2018 16:32	0.5	0.4	5.6	70.7	41.2	82.3	3.3356	2.5
4/21/2018 16:33	0.5	0.9	4.9	66.4	38.8	72.7	3.1132	2.5
4/21/2018 16:34	0.5	0.8	4.9	65.5	39.4	77.2	3.2183	2.5
4/21/2018 16:35	0.5	1.4	6.4	60.6	37.6	73.6	3.0273	2.6
4/21/2018 16:36	0.5	0.8	6.5	64.1	37.8	75.1	3.1247	2.6
4/21/2018 16:37	0.5	1	7	63.4	37.4	73.5	3.1101	2.7
4/21/2018 16:38	0.4	0.3	6.9	61.1	37.9	76.3	3.1607	2.6
4/21/2018 16:39	0.4	0.8	5.5	60.1	36.3	74.6	3.0248	2.7
4/21/2018 16:40	0.3	0.1	7	60.1	37	74.7	3.1136	2.6
4/21/2018 16:41	0.4	0.7	5.3	56.2	35.1	74.2	2.9398	2.7
4/21/2018 16:42	0.4	0.9	5.8	53.7	34.5	73.8	2.8913	2.7
4/21/2018 16:43	0.4	0	5.6	56.5	34.2	72.9	2.9033	2.6
4/21/2018 16:44	0.4	1.1	6.4	55.2	34.8	72.5	2.9263	2.6
4/21/2018 16:45	0.5	0.9	5	52.2	33.7	71.6	2.8378	2.8
4/21/2018 16:46	0.5	0.9	6.8	53.2	33.5	70.7	2.837	2.8
4/21/2018 16:47	0.5	0.1	5.2	50.1	32.8	69.7	2.7597	2.7
4/21/2018 16:48	0.5	0.9	6	53.5	33	68.7	2.8573	2.7
4/21/2018 16:49	0.5	0.5	5	47.2	31.8	63.6	2.6723	2.7
4/21/2018 16:50	0.5	0.3	5.9	47.6	31.2	62.3	2.6672	2.6
4/21/2018 16:51	0.6	1.5	4.6	45.3	29.5	56.5	2.4806	2.4
4/21/2018 16:52	0.6	1.2	4.5	47	30.2	60	2.5868	2.2
4/21/2018 16:53	0.6	0.2	5.4	43	29.3	57.6	2.4775	2.3
4/21/2018 16:54	0.6	0.6	4.6	41.2	28.1	55.9	2.4166	2.4
4/21/2018 16:55	0.7	1.3	4.2	39	26.5	49.3	2.1949	2.5
4/21/2018 16:56	0.8	1.3	6.1	39.1	26	46.5	2.1913	2.6
4/21/2018 16:57	0.8	1.5	6.5	39.9	26.3	47	2.2568	2.6
4/21/2018 16:58	0.8	1.4	5.2	40.5	26.9	47.7	2.3173	2.7
4/21/2018 16:59	0.7	0.3	4.6	39.1	26.4	48.2	2.2808	2.8
4/21/2018 17:00	0.6	1.6	5	39.6	26.1	48.7	2.2773	2.9
4/21/2018 17:01	0.6	1.7	4.2	40.7	26.9	49.3	2.3659	2.9
4/21/2018 17:02	0.5	0.7	3.6	41.3	27.3	50.3	2.3963	2.5

4/21/2018 17:03	0.4	0.7	4.8	42.4	27.8	51.5	2.4926	2.1
4/21/2018 17:04	0.3	0.1	5.7	41.8	27.7	52.4	2.4588	2
4/21/2018 17:05	0.3	0.8	4	41.4	27.3	53.5	2.4581	2
4/21/2018 17:06	0.2	0.6	4.3	40.5	27	53.9	2.4223	1.9
4/21/2018 17:07	0.2	0.5	4.8	40.1	26.5	54.2	2.3828	1.9
4/21/2018 17:08	0.3	1.1	4.3	40.1	26.3	54.5	2.3813	1.9
4/21/2018 17:09	0.2	0.6	4.7	39.7	26.3	54.6	2.3855	1.9
4/21/2018 17:10	0.2	0.7	4.8	39	26	54.5	2.3796	1.9
4/21/2018 17:11	0.1	1.5	4.7	38.1	25.4	54	2.2978	2
4/21/2018 17:12	0.2	1.1	5.5	37.4	24.9	53.1	2.2578	2
4/21/2018 17:13	0.3	0.8	4.1	35.7	24.3	52	2.1826	2
4/21/2018 17:14	0.4	0.6	4.6	36.4	24.1	50.9	2.1881	2
4/21/2018 17:15	0.4	0.8	5.3	36.4	23.9	50.2	2.1694	2
4/21/2018 17:16	0.4	1.4	6.1	34.6	23.5	49.2	2.1278	2.1
4/21/2018 17:17	0.4	1.7	3.3	34.7	23.1	48.1	2.0967	2.1
4/21/2018 17:18	0.4	0.2	3.6	34.8	23	47.3	2.0848	2.1
4/21/2018 17:19	0.4	0.9	4.4	32.8	22.1	46.1	1.9582	1.8
4/21/2018 17:20	0.5	1	4.4	31.1	21.4	44.8	1.8883	1.8
4/21/2018 17:21	0.7	1.7	4.1	30.6	19.8	35.8	1.7415	1.9
4/21/2018 17:22	0.8	1.3	4.4	30.5	20.7	37.2	1.8145	2
4/21/2018 17:23	0.9	1.9	4.6	29.8	19.3	32.2	1.6648	2.1
4/21/2018 17:24	0.9	1.5	4.5	29.7	19.9	35.8	1.7328	2.2
4/21/2018 17:25	1	2.2	3.8	28.5	18.9	35.6	1.6389	1.9
4/21/2018 17:26	0.9	1.7	4.2	30.5	19.3	35.2	1.7335	1.7
4/21/2018 17:27	0.8	1.6	4.1	32.3	20.7	35.5	1.8783	1.8
4/21/2018 17:28	0.6	0.5	5	29.8	20.3	35.4	0.6271	1.6
4/21/2018 17:29	0.5	0.8	4.9	30.4	20.2	35.4	0.0985	1.5
4/21/2018 17:30	0.6	0.9	4.1	29	20	35.1	0.1088	1.5
4/21/2018 17:31	0.6	1.6	3.8	29.6	19.5	34.8	0.0977	1.5
4/21/2018 17:32	0.8	1.3	3.9	27.3	18.6	34.3	0.1067	1.5
4/21/2018 17:33	1	2.2	3.7	23.4	16.6	21.2	0.096	1.5
4/21/2018 17:34	1.6	5	5.6	24.4	15.6	20.2	0.0991	1.5
4/21/2018 17:35	4	10.8	7.7	25.9	11.6	8.1	0.1024	1.5
4/21/2018 17:36	6.8	12.4	8.4	27.4	9.8	5.9	0.0955	1.5
4/21/2018 17:37	9.5	12.3	8	28	9.2	5.8	0.107	1.5
4/21/2018 17:38	10.6	13.7	9.4	28.8	8.8	5.6	0.0913	1.5
4/21/2018 17:39	11.5	14.3	8.2	29.9	7.7	5.3	0.1082	1.5
4/21/2018 17:40	12.7	16.8	11	31.3	7	5	0.0892	1.5
4/21/2018 17:41	14.1	18.6	11.7	32.8	6.2	4.6	0.101	1.5
4/21/2018 17:42	15.7	18.9	10.7	34.5	5.4	4.1	0.0995	1.5
4/21/2018 17:43	17.3	20.5	12	35.6	5.1	3.6	0.1027	1.5
4/21/2018 17:44	18.8	22	13.5	36.8	4.8	3.1	0.0989	1.5
4/21/2018 17:45	20.2	24.2	12.4	38.3	4.2	2.6	0.0957	1.5
4/21/2018 17:46	21.3	23.6	13.1	38.1	4.1	2.3	0.1059	1.5
4/21/2018 17:47	22	24.4	14.6	39.3	4	2	0.0765	1.5
4/21/2018 17:48	22.3	24.2	13.8	38.6	3.8	1.7	0.0921	1.5
4/21/2018 17:49	22.2	22.3	14.5	38.1	4.2	1.5	0.0845	1.5

4/21/2018 17:50	22.3	24	14.1	38.9	4.2	1.4	0.0907	1.5
4/21/2018 17:51	22.8	25.4	14.3	39.7	3.9	1.3	0.0893	1.5
4/21/2018 17:52	23.4	25	13.9	39.8	3.8	1.3	0.0936	1.5
4/21/2018 17:53	24.1	26.3	15.8	40.4	3.5	1.2	0.099	1.5
4/21/2018 17:54	24.5	26.1	15	40.7	3.4	1.2	0.0877	1.5
4/21/2018 17:55	25	26.2	15	41.2	3.4	1.1	0.1019	1.5
4/21/2018 17:56	25.2	26	13.3	40.5	3.3	1.1	0.0818	1.5
4/21/2018 17:57	24	21.8	11.8	37.7	4.2	1.1	0.0896	1.5
4/21/2018 17:58	22.8	21.1	12.7	36.6	4.7	1.1	0.0919	1.5
4/21/2018 17:59	20.1	17.6	12.2	34.2	5.9	1.2	0.0915	1.5
4/21/2018 18:00	17.7	14.2	9.7	30.8	6.3	1.3	0.0952	1.5
4/21/2018 18:01	14.9	13.6	8.8	30.2	6.9	1.5	0.0877	1.5
4/21/2018 18:02	13.3	13.1	9.5	29.3	6.8	1.7	0.102	1.5
4/21/2018 18:03	12.3	12.4	10.2	28.1	5.9	1.8	0.1974	2.4
4/21/2018 18:04	11.8	11.4	7.8	27.9	1.6	1.8	0.3317	2.6
4/21/2018 18:05	11.2	11.2	8	28	0.3	1.7	0.3254	2.4
4/21/2018 18:06	10.8	10.8	6.7	27.5	0.2	1.5	0.3402	2.3
4/21/2018 18:07	10.5	11	6.9	27.5	0.3	1.3	0.3535	2.1
4/21/2018 18:08	10.7	11.3	7.2	27.8	0.3	1.1	0.3348	2.2
4/21/2018 18:09	11.2	13.9	8.6	29.1	0.2	0.8	0.3237	2.4
4/21/2018 18:10	11.7	11.9	8.3	29.3	0.2	0.5	0.3956	2.4
4/21/2018 18:11	11.4	9.8	6.8	29.1	0.2	0.2	0.5146	2.2
4/21/2018 18:12	10.1	8.6	6.9	27.8	0.2	0.1	0.5648	2
4/21/2018 18:13	8.5	7.5	7.6	26.6	0.2	0.1	0.5054	2
4/21/2018 18:14	7.6	7.7	6.6	25.7	0.2	0.1	0.488	1.9
4/21/2018 18:15	7.4	8.8	5.6	25.5	0.2	0.1	0.3764	1.8
4/21/2018 18:16	7	6.3	6.4	24.3	0	0	0.4447	1.8
4/21/2018 18:17	6.9	6.7	6.9	24.5	0.2	51	0.4016	1.7
4/21/2018 18:18	6.7	7.1	5.7	24.8	0.1	99.6	0.3909	1.7
4/21/2018 18:19	6.7	5.7	6.1	23.8	0	100.7	0.3798	1.7
4/21/2018 18:20	6.4	6.5	5.7	24.3	0	96.8	0.4147	1.7
4/21/2018 18:21	6	7	6.3	23.9	2	41.4	0.385	1.7
4/21/2018 18:22			6.4	23.5	17.5	69.9	0.4236	1.7
4/21/2018 18:23			5	23.4	68.5	84.6	0.4006	1.7
4/21/2018 18:24			4.6	23.1	76.4	28	0.3742	1.7
4/21/2018 18:25			5.8	23.2	77.2	24.5	0.3578	1.7
4/21/2018 18:26			4	23.2	63.2	22.3	0.3448	1.7
4/21/2018 18:27			5	22.9	5.1	6.7	0.41	1.7
4/21/2018 18:28			6.1	23.4	6.1	3.2	0.3812	1.7
4/21/2018 18:29			7.1	23.4	6.1	3.3	0.4123	1.7
4/21/2018 18:30			6.2	23.1	6.1	2.9	0.3639	1.7
4/21/2018 18:31			5.8	23.4	6.1	2.9	0.3446	1.7
4/21/2018 18:32			5.2	23.6	5.8	2.9	0.3692	1.7
4/21/2018 18:33			6.1	23	6.1	2.9	0.3964	1.8
4/21/2018 18:34			5.7	23.3	6.1	2.9	0.391	1.8
4/21/2018 18:35			4.7	23	6.1	3	0.3911	1.8
4/21/2018 18:36			4.8	23.1	6	3	0.3912	1.9

4/21/2018 18:37	4.9	23.1	5.7	3	0.3491	1.8
4/21/2018 18:38	4.5	23.3	5.6	3	0.3547	1.8
4/21/2018 18:39	6.6	23.3	5.6	3	0.3293	1.9
4/21/2018 18:40	6.7	23.7	5.4	3	0.3201	1.9
4/21/2018 18:41	6.5	23.2	5.6	3	0.3411	2
4/21/2018 18:42	6.6	23.5	5.6	2.9	0.3391	2.1
4/21/2018 18:43	6.1	23.2	5.7	2.9	0.3366	2.1
4/21/2018 18:44	4.4	23	5.7	2.8	0.3356	2.1
4/21/2018 18:45	4.6	22.9	5.6	2.8	0.3449	2.1
4/21/2018 18:46	4.9	22.9	5.4	2.7	0.316	2.1
4/21/2018 18:47	4.4	22.9	5.4	2.7	0.3341	2.2
4/21/2018 18:48	5.6	23.2	5.4	2.7	0.3154	2.2
4/21/2018 18:49	5	22.8	5.2	2.7	0.3146	2.2
4/21/2018 18:50	5.3	23.2	5.4	2.7	0.3083	2.2
4/21/2018 18:51	5.5	22.8	5.2	2.7	0.3166	2.2
4/21/2018 18:52	5.5	23	5.3	2.6	0.3134	2.3
4/21/2018 18:53	4.8	22.7	5.1	2.6	0.3172	2.3
4/21/2018 18:54	6.3	22.5	5.2	2.6	0.3281	2.3
4/21/2018 18:55	5.7	22.7	5.1	2.7	0.3029	2.3
4/21/2018 18:56	5.6	23	4.9	2.7	0.3132	2.4
4/21/2018 18:57	4.3	22.5	5	2.7	0.3126	2.5
4/21/2018 18:58	5.3	22.7	5.1	2.7	0.3226	2.5
4/21/2018 18:59	4.1	22.5	4.9	2.7	0.2916	2.5
4/21/2018 19:00	4.6	22.8	5	2.7	0.3282	2.4
4/21/2018 19:01	5.1	22.5	5	2.7	0.3081	2.4
4/21/2018 19:02	4.6	22.5	5	2.7	0.3108	2.4
4/21/2018 19:03	5.3	22.8	5	2.8	0.3115	2.5
4/21/2018 19:04	4.5	22.7	5	2.8	0.3124	2.5
4/21/2018 19:05	5.7	22.4	5.1	2.8	0.3259	2.6
4/21/2018 19:06	4.6	22.7	5.2	2.8	0.3102	2.6
4/21/2018 19:07	4.2	22.3	5.2	2.8	0.3202	2.6
4/21/2018 19:08	4.3	22.6	5.2	2.9	0.2965	2.7
4/21/2018 19:09	3.5	22.7	5.1	2.9	0.312	2.7
4/21/2018 19:10	3.7	22.2	5.1	2.9	0.2984	2.7
4/21/2018 19:11	5.6	22.5	5.2	2.9	0.3218	2.6
4/21/2018 19:12	4.8	22.6	5.1	2.9	0.2989	2.6
4/21/2018 19:13	5	22.2	5.1	2.9	0.2951	2.6
4/21/2018 19:14	4.7	22.4	4.9	2.8	0.2893	2.6
4/21/2018 19:15	3.7	22.6	4.9	2.8	0.2837	2.6
4/21/2018 19:16	5.5	22.5	4.8	2.8	0.2884	2.6
4/21/2018 19:17	3.6	22.2	4.7	2.7	0.2751	2.7
4/21/2018 19:18	4.3	22.4	4.7	2.7	0.2872	2.7
4/21/2018 19:19	5.4	22.1	4.7	2.6	0.2735	2.7
4/21/2018 19:20	4	22.3	4.8	2.6	0.2888	2.6
4/21/2018 19:21	4.7	22.5	4.8	2.6	0.2934	2.6
4/21/2018 19:22	4.4	22.4	4.9	2.6	0.2974	2.6
4/21/2018 19:23	5.1	22.5	4.8	2.6	0.2883	2.6

4/21/2018 19:24		22.3	4.7	2.6	0.2815	2.6
4/21/2018 19:25	4.8	22.2	4.9	2.7	0.3074	2.7
4/21/2018 19:26	4.5	22.3	4.8	2.7	0.2733	2.8
4/21/2018 19:27	4.2	22.5	4.8	2.8	0.2868	2.8
4/21/2018 19:28	6.4	22.2	4.7	2.8	0.2715	2.8
4/21/2018 19:29	4.3	22.3	4.6	2.8	0.2868	2.8
4/21/2018 19:30	4.2	22.5	4.6	2.7	0.2774	2.9
4/21/2018 19:31	3.6	22.3	4.6	2.7	0.2861	2.9
4/21/2018 19:32	5.1	22.3	4.6	2.7	0.2707	2.9
4/21/2018 19:33	4.7	22.1	4.6	2.7	0.2719	3
4/21/2018 19:34	4.5	22	4.7	2.7	0.2897	2.8
4/21/2018 19:35	4.7	22.1	4.6	2.7	0.2734	2.8
4/21/2018 19:36	4.8	22.2	4.6	2.7	0.2844	2.9
4/21/2018 19:37	3.3	22.3	4.6	2.7	0.2689	2.9
4/21/2018 19:38	4.7	22.2	4.6	2.6	0.281	2.9
4/21/2018 19:39	6.4	22.2	4.6	2.7	0.2672	2.9
4/21/2018 19:40	4.2	22.2	4.7	2.7	0.2906	3
4/21/2018 19:41		21.9	4.7	2.7	0.2718	3.1
4/21/2018 19:42		22.2	4.7	2.7	0.2691	3.1
4/21/2018 19:43		22.3	4.7	2.7	0.2818	3.2
4/21/2018 19:44	9.5	22.3	4.7	2.7	0.2671	3.1
4/21/2018 19:45	4.8	22.2	4.6	2.7	0.2637	3.1
4/21/2018 19:46		22.7	4.6	2.7	0.2398	3.1
4/21/2018 19:47	4.2	22.4	4.5	2.7	0.2678	3.1
4/21/2018 19:48		22.7	4.5	2.6	0.2475	3.1
4/21/2018 19:49		23	4.6	2.6	0.2694	3.1
4/21/2018 19:50	4.2	23.2	4.7	2.5	0.235	3.1
4/21/2018 19:51	4.1	22.2	4.7	2.5	0.2824	3.1
4/21/2018 19:52		22.8	4.7	2.5	0.2425	2.9
4/21/2018 19:53	4	22.6	4.6	2.5	0.2428	2.9
4/21/2018 19:54	5.7	22.8	4.6	2.4	0.2365	2.9
4/21/2018 19:55	4.9	23	4.6	2.4	0.2298	2.8
4/21/2018 19:56	4.7	22.5	4.6	2.4	0.2584	2.9
4/21/2018 19:57	5.5	22.6	4.6	2.4	0.2217	2.9
4/21/2018 19:58	4.6	22.6	4.5	2.4	0.2451	3
4/21/2018 19:59	5.4	23.8	4.6	2.3	0.224	3.2
4/21/2018 20:00	5.2	23	4.7	2.3	0.265	3.2
4/21/2018 20:01	5.3	22.9	4.5	2.3	0.2356	3.1
4/21/2018 20:02	4.6	23.1	4.5	2.3	0.2276	2.9
4/21/2018 20:03	5	23.2	4.5	2.3	0.2383	2.9
4/21/2018 20:04	6	23.3	4.9	2.3	0.2498	2.9
4/21/2018 20:05	4.8	22.7	4.7	2.3	0.2504	3
4/21/2018 20:06	4.7	22.6	4.4	2.3	0.209	3.1
4/21/2018 20:07	5.6	23.2	4.4	2.3	0.2203	3.2
4/21/2018 20:08	5	23	4.4	2.3	0.2249	3.2
4/21/2018 20:09	4.5	22.8	4.4	2.3	0.2396	3.2
4/21/2018 20:10	4	22.6	4.4	2.3	0.2452	3.3

4/21/2018 20:11	5.4	23.3	4.3	2.3	0.2086	3.2
4/21/2018 20:12	5.6	23.8	4.4	2.2	0.2115	3
4/21/2018 20:13	5.5	23.2	4.3	2.2	0.209	3.1
4/21/2018 20:14	5.1	23.1	4.2	2.1	0.2399	3.3
4/21/2018 20:15	5.3	23.6	4.2	2.1	0.1993	3.4
4/21/2018 20:16	4.7	23.2	4.3	2.1	0.2256	3.2
4/21/2018 20:17	6.3	23.7	4.3	2.1	0.2284	3.1
4/21/2018 20:18	3.7	22.4	4.2	2.1	0.2363	3.1
4/21/2018 20:19	3.7	23.6	4.1	2.2	0.2232	3.1
4/21/2018 20:20	5.6	23.1	4.2	2.2	0.2136	3.1
4/21/2018 20:21	5.8	23.1	4	2.2	0.2143	3.1
4/21/2018 20:22	5.8	23	4.3	2.2	0.2113	3.3
4/21/2018 20:23	5	22.7	4.2	2.3	0.2422	3.4
4/21/2018 20:24	6.3	23.6	4	2.3	0.2187	3.5
4/21/2018 20:25	5.4	23.5	4	2.2	0.1928	3.5
4/21/2018 20:26	5	23.3	4.1	2.2	0.2494	3.5
4/21/2018 20:27	3.5	23.2	4.2	2.2	0.2314	3.6
4/21/2018 20:28	6	23.6	4	2.2	0.1969	3.5
4/21/2018 20:29	5.2	23.5	4.1	2.2	0.19	3.5
4/21/2018 20:30	4	22.9	4.2	2.2	0.2349	3.4
4/21/2018 20:31	4.6	22.6	4.2	2.2	0.2362	3.3
4/21/2018 20:32	4.5	22.6	4.2	2.3	0.243	3.4
4/21/2018 20:33	4.5	22.5	4.2	2.4	0.2421	3.5
4/21/2018 20:34	4.8	22.9	4.2	2.4	0.2171	3.6
4/21/2018 20:35	4.7	22.9	4.2	2.4	0.2379	3.6
4/21/2018 20:36	5	22.6	4.2	2.5	0.233	3.5
4/21/2018 20:37	4.2	22.4	4.2	2.6	0.2292	3.6
4/21/2018 20:38	4.8	22.4	4.2	2.6	0.2353	3.5
4/21/2018 20:39	4.9	22.9	4.3	2.5	0.2248	3.1
4/21/2018 20:40	5.3	22.6	4.1	2.5	0.2462	3.2
4/21/2018 20:41	4.8	22.4	4.1	2.5	0.2295	3.2
4/21/2018 20:42	4.6	22.4	4.2	2.6	0.2336	3.1
4/21/2018 20:43	4.2	22.3	4.3	2.5	0.2288	3.1
4/21/2018 20:44	4.2	22.5	4.3	2.5	0.2374	3.1
4/21/2018 20:45	3.9	22.3	4.3	2.6	0.2243	3.1
4/21/2018 20:46	5	22.4	4.3	2.6	0.2305	3
4/21/2018 20:47	4.3	22.1	4.2	2.6	0.2427	3.1
4/21/2018 20:48	4.4	22.4	4.2	2.6	0.231	3.1
4/21/2018 20:49	3.8	22.2	4.3	2.6	0.2385	3.2
4/21/2018 20:50	4	22	4.2	2.6	0.2263	3.2
4/21/2018 20:51	4	22.1	4.2	2.7	0.2475	3.3
4/21/2018 20:52	4.3	22.1	4.4	2.7	0.2352	3.5
4/21/2018 20:53	3.8	22	4.4	2.7	0.2364	3.5
4/21/2018 20:54	5.1	22.1	4.4	2.7	0.2338	3.6
4/21/2018 20:55	4.4	22.2	4.5	2.7	0.2366	3.6
4/21/2018 20:56	3.7	22.3	4.4	2.7	0.2412	3.6
4/21/2018 20:57	4	21.9	4.5	2.7	0.2225	3.7

4/21/2018 20:58	3.8	22.5	4.4	2.7	0.24	3.7
4/21/2018 20:59	5.6	22.2	4.6	2.7	0.2141	3.7
4/21/2018 21:00	5.4	22	4.6	2.7	0.2354	3.7
4/21/2018 21:01	4.3	21.9	4.5	2.6	0.2299	3.8
4/21/2018 21:02	3.8	22.4	4.5	2.6	0.2192	3.5
4/21/2018 21:03	3.4	22.2	4.6	2.6	0.2266	3.3
4/21/2018 21:04	4.3	22	4.6	2.6	0.2219	3.4
4/21/2018 21:05	2.9	22.1	4.5	2.6	0.2255	3.4
4/21/2018 21:06	3.3	21.8	4.5	2.6	0.2135	3.2
4/21/2018 21:07	3.9	22	4.7	2.6	0.2216	2.9
4/21/2018 21:08	4.7	22.1	4.6	2.6	0.2197	2.7
4/21/2018 21:09	4.3	22.1	4.6	2.7	0.2186	2.8
4/21/2018 21:10	6	22	4.5	2.7	0.2218	2.8
4/21/2018 21:11	6.2	22	4.5	2.7	0.2157	2.9
4/21/2018 21:12	3.8	22.2	4.5	2.7	0.2301	2.9
4/21/2018 21:13	4.8	22.2	4.4	2.7	0.2177	2.9
4/21/2018 21:14	5.2	21.8	4.4	2.7	0.2258	3
4/21/2018 21:15	4.8	22	4.5	2.7	0.2138	2.9
4/21/2018 21:16	4.8	21.8	4.5	2.7	0.2182	2.9
4/21/2018 21:17	3.4	22	4.5	2.7	0.2175	2.8
4/21/2018 21:18	5	21.9	4.5	2.7	0.2133	2.7
4/21/2018 21:19	3.8	21.8	4.5	2.7	0.2132	2.8
4/21/2018 21:20	4.5	22.2	4.5	2.7	0.199	3
4/21/2018 21:21	3.9	21.8	4.5	2.7	0.2218	2.9
4/21/2018 21:22	4	22	4.5	2.7	0.2061	3.1
4/21/2018 21:23	4.6	21.9	4.4	2.7	0.2193	3.2
4/21/2018 21:24	3.8	22	4.5	2.7	0.2061	3.2
4/21/2018 21:25	5.4	22.1	4.4	2.7	0.2122	3.2
4/21/2018 21:26	5.3	21.8	4.4	2.7	0.2107	3.2
4/21/2018 21:27	4.3	21.7	4.5	2.6	0.2095	3.2
4/21/2018 21:28	3.8	22	4.5	2.6	0.2184	3.3
4/21/2018 21:29	5.4	21.8	4.4	2.6	0.2003	3.3
4/21/2018 21:30	4.7	21.6	4.4	2.6	0.2171	3.4
4/21/2018 21:31	3.8	21.9	4.4	2.6	0.1996	3.3
4/21/2018 21:32	3.9	22.1	4.4	2.6	0.2099	2.9
4/21/2018 21:33	4.4	21.9	4.4	2.6	0.2065	3.2
4/21/2018 21:34	4.6	21.8	4.4	2.6	0.2027	2.9
4/21/2018 21:35	4.8	21.9	4.4	2.6	0.2125	2.2
4/21/2018 21:36	4.3	21.9	4.4	2.6	0.2015	2.2
4/21/2018 21:37	3.7	22	4.4	2.6	0.2093	2.4
4/21/2018 21:38	4.7	21.9	4.4	2.6	0.2	2.5
4/21/2018 21:39	4.5	22	4.4	2.6	0.2107	2.6
4/21/2018 21:40	5.4	22.1	4.4	2.6	0.2134	2.7
4/21/2018 21:41	3.8	21.8	4.4	2.6	0.2017	2.8
4/21/2018 21:42	4.9	21.5	4.4	2.6	0.2086	3.1
4/21/2018 21:43	4.5	21.8	4.4	2.6	0.1943	3.3
4/21/2018 21:44	5.2	22	4.4	2.6	0.2081	3.5

4/21/2018 21:45	4.5	21.7	4.4	2.6	0.188	3.6
4/21/2018 21:46	5.2	21.8	4.5	2.6	0.2014	3.5
4/21/2018 21:47	3.4	21.8	4.4	2.6	0.2073	3.5
4/21/2018 21:48	4.4	21.9	4.4	2.6	0.1992	3.6
4/21/2018 21:49	4.2	21.8	4.4	2.6	0.2006	3.7
4/21/2018 21:50	4.1	21.8	4.4	2.6	0.1898	3.8
4/21/2018 21:51	5.1	21.9	4.5	2.6	0.2026	3.9
4/21/2018 21:52	3.4	21.8	4.5	2.6	0.1922	3.7
4/21/2018 21:53	3	22	4.4	2.6	0.2015	3.4
4/21/2018 21:54	3.9	21.7	4.5	2.6	0.1968	3.2
4/21/2018 21:55	4.3	21.8	4.5	2.6	0.1997	3.2
4/21/2018 21:56	4.4	21.9	4.5	2.6	0.1994	3.5
4/21/2018 21:57	3.3	21.7	4.5	2.6	0.1906	3.6
4/21/2018 21:58	3.4	21.9	4.6	2.6	0.1969	3.7
4/21/2018 21:59	5.2	21.9	4.7	2.6	0.1848	3.8
4/21/2018 22:00	3.1	21.6	4.6	2.6	0.2047	3.8
4/21/2018 22:01	3.4	21.7	4.6	2.5	0.1939	3.9
4/21/2018 22:02	3.7	21.7	4.5	2.5	0.1969	3.8
4/21/2018 22:03	4.5	21.8	4.6	2.6	0.1981	3.8
4/21/2018 22:04	2.6	21.9	4.6	2.6	0.1873	3.8
4/21/2018 22:05	3	21.8	4.6	2.6	0.2006	3.9
4/21/2018 22:06	3.2	21.5	4.6	2.6	0.184	3.9
4/21/2018 22:07	4.4	21.8	4.6	2.6	0.1958	3.9
4/21/2018 22:08	3	21.6	4.6	2.6	0.1837	3.9
4/21/2018 22:09	3.9	21.8	4.7	2.6	0.1935	3.9
4/21/2018 22:10	4.6	21.7	4.7	2.6	0.1846	4
4/21/2018 22:11	4.7	21.9	4.7	2.6	0.192	4
4/21/2018 22:12	1.8	21.7	4.7	2.6	0.1939	4.1
4/21/2018 22:13	4.4	21.7	4.7	2.6	0.1886	4.1
4/21/2018 22:14	3.6	21.7	4.7	2.6	0.1976	4.1
4/21/2018 22:15	4.6	21.6	4.8	2.6	0.1855	4
4/21/2018 22:16	3.8	22	4.8	2.6	0.1946	3.9
4/21/2018 22:17		21.7	4.8	2.6	0.1853	3.9
4/21/2018 22:18	4.2	21.7	4.8	2.6	0.1903	3.9
4/21/2018 22:19	5.7	21.6	4.8	2.6	0.1855	4
4/21/2018 22:20	5	21.8	4.8	2.6	0.1835	4
4/21/2018 22:21	4.3	21.5	4.8	2.6	0.1849	4
4/21/2018 22:22	3.6	21.6	4.8	2.6	0.1791	4
4/21/2018 22:23	3.7	21.6	4.8	2.6	0.1893	4
4/21/2018 22:24	4.2	21.6	4.8	2.5	0.1735	4
4/21/2018 22:25	4.7	21.6	4.8	2.5	0.1933	3.9
4/21/2018 22:26	2.1	21.5	4.8	2.5	0.1799	3.8
4/21/2018 22:27	3.8	21.6	4.9	2.5	0.1871	3.9
4/21/2018 22:28	4.4	21.6	4.8	2.6	0.1765	4
4/21/2018 22:29	2.8	21.8	4.9	2.6	0.1787	4.1
4/21/2018 22:30	3.8	21.5	4.9	2.6	0.1826	4.3
4/21/2018 22:31	4.6	21.8	4.9	2.5	0.1778	3.4

4/21/2018 22:32	4.4	21.4	4.8	2.6	0.1821	2.1
4/21/2018 22:33	3.8	21.5	4.9	2.6	0.1698	2.3
4/21/2018 22:34		21.5	4.9	2.5	0.1864	2.3
4/21/2018 22:35		21.7	4.9	2.5	0.172	2.5
4/21/2018 22:36	3.7	21.3	4.9	2.5	0.1792	2.9
4/21/2018 22:37		21.5	4.9	2.5	0.1734	3.1
4/21/2018 22:38	4	21.6	4.9	2.5	0.1724	3.2
4/21/2018 22:39		21.5	4.9	2.5	0.1789	3.2
4/21/2018 22:40	3.4	21.4	4.9	2.5	0.1749	3.3
4/21/2018 22:41	3.5	21.6	4.9	2.5	0.1833	3.3
4/21/2018 22:42	7.1	21.6	4.9	2.5	0.1652	3.3
4/21/2018 22:43	3.4	21.6	4.9	2.5	0.1893	3.1
4/21/2018 22:44	7.7	21.5	4.9	2.5	0.17	3
4/21/2018 22:45	6.3	21.5	5	2.5	0.185	3.1
4/21/2018 22:46	3	21.5	5	2.5	0.1696	3
4/21/2018 22:47	3.7	21.2	5	2.5	0.1693	3
4/21/2018 22:48	4.9	21.3	5	2.5	0.1853	3.1
4/21/2018 22:49	4.1	21.4	5.1	2.5	0.1638	3.3
4/21/2018 22:50	4	21.4	5.2	2.5	0.1728	3.5
4/21/2018 22:51	4.5	21.4	5.1	2.5	0.1615	3.5
4/21/2018 22:52	4.1	21.4	5.2	2.5	0.1773	3.3
4/21/2018 22:53	4.4	21.5	5.2	2.5	0.1693	3.3
4/21/2018 22:54	2.6	21.4	5.2	2.5	0.1793	3.4
4/21/2018 22:55	3.5	21.3	5.3	2.5	0.1713	3.1
4/21/2018 22:56	3	21.3	5.3	2.5	0.1728	2.8
4/21/2018 22:57	3.6	21.1	5.4	2.5	0.1748	2.9
4/21/2018 22:58	4.2	21	5.4	2.5	0.1689	3
4/21/2018 22:59	3.7	21.2	5.5	2.5	0.1807	3.2
4/21/2018 23:00	4.4	21.2	5.7	2.5	0.1638	3.3
4/21/2018 23:01	5.2	21.1	5.5	2.5	0.1721	3.3
4/21/2018 23:02	2.8	21.1	5.6	2.5	0.1653	3.2
4/21/2018 23:03	2.6	21	5.5	2.5	0.1747	3.1
4/21/2018 23:04	2.8	20.9	5.6	2.5	0.1787	3.2
4/21/2018 23:05	3.5	21.1	5.6	2.5	0.1609	3.1
4/21/2018 23:06	3.2	21.2	5.6	2.5	0.1716	2.7
4/21/2018 23:07	4.9	20.9	5.7	2.5	0.1523	2.4
4/21/2018 23:08	4.5	20.8	5.7	2.5	0.1746	2.5
4/21/2018 23:09	4.6	21.1	5.7	2.5	0.1532	2.7
4/21/2018 23:10	3.7	20.9	5.7	2.5	0.1767	2.8
4/21/2018 23:11	3.6	21.1	5.7	2.5	0.1651	3
4/21/2018 23:12	3.2	21	5.8	2.5	0.1639	2.9
4/21/2018 23:13	3.7	20.9	5.8	2.5	0.1728	2.8
4/21/2018 23:14	3	21	5.8	2.5	0.1575	2.7
4/21/2018 23:15	3.5	21.2	5.8	2.5	0.1823	2.6
4/21/2018 23:16	3.5	21.1	5.8	2.5	0.1487	2.7
4/21/2018 23:17	3.7	21.1	5.9	2.5	0.1751	2.9
4/21/2018 23:18	5.1	21.3	5.9	2.5	0.1587	2.8

4/21/2018 23:19	4.5	21	5.9	2.5	0.1672	2.6
4/21/2018 23:20	3.9	20.9	5.9	2.5	0.1687	2.7
4/21/2018 23:21	2.8	20.9	5.8	2.5	0.158	2.8
4/21/2018 23:22	4.8	21	5.9	2.5	0.174	2.9
4/21/2018 23:23	3.9	21.1	5.9	2.5	0.1603	3
4/21/2018 23:24	3.1	20.9	5.9	2.5	0.1672	2.9
4/21/2018 23:25	4.3	21	5.9	2.5	0.1626	2.9
4/21/2018 23:26	3.9	20.7	6	2.5	0.164	2.8
4/21/2018 23:27	3	21	5.9	2.5	0.1626	2.8
4/21/2018 23:28	2.5	20.7	6	2.5	0.1485	2.7
4/21/2018 23:29	3.4	20.8	6	2.6	0.1716	2.7
4/21/2018 23:30	3.6	20.9	5.9	2.6	0.1563	2.7
4/21/2018 23:31	4.5	20.8	5.9	2.6	0.1712	2.8
4/21/2018 23:32	3.3	20.9	5.9	2.6	0.1547	2.9
4/21/2018 23:33	3.5	20.8	5.9	2.6	0.1676	2.9
4/21/2018 23:34	3.5	21	5.9	2.6	0.1465	2.9
4/21/2018 23:35	3.3	21	5.9	2.6	0.1639	2.9
4/21/2018 23:36	4.4	21.1	5.9	2.6	0.1645	2.9
4/21/2018 23:37	3.4	20.9	5.9	2.6	0.1473	2.9
4/21/2018 23:38	4.2	21	5.9	2.5	0.1664	3
4/21/2018 23:39	4.5	20.9	5.9	2.6	0.1494	3
4/21/2018 23:40	4.5	20.8	5.9	2.6	0.1588	3
4/21/2018 23:41	2.4	20.8	5.8	2.5	0.1527	3.1
4/21/2018 23:42	3.2	20.9	5.8	2.5	0.1602	3.1
4/21/2018 23:43	3.3	20.7	5.8	2.6	0.1532	3.1
4/21/2018 23:44	3.4	20.9	5.8	2.5	0.1559	2.9
4/21/2018 23:45	3.3	20.7	5.8	2.5	0.1649	2.9
4/21/2018 23:46	4.1	21	5.8	2.5	0.1461	3
4/21/2018 23:47	3.3	20.8	5.8	2.5	0.1664	3.1
4/21/2018 23:48	3.9	21.2	5.8	2.5	0.1472	3.2
4/21/2018 23:49	4.4	20.9	5.8	2.5	0.1593	3.3
4/21/2018 23:50	3.1	20.8	5.8	2.5	0.1541	3.4
4/21/2018 23:51	3.1	20.8	5.7	2.5	0.1635	3.5
4/21/2018 23:52	4.7	20.9	5.7	2.5	0.1558	3.1
4/21/2018 23:53	3.9	21	5.7	2.5	0.1502	2.4
4/21/2018 23:54	3.8	20.9	5.6	2.5	0.1616	2.6
4/21/2018 23:55	4.2	20.8	5.6	2.5	0.1401	2.9
4/21/2018 23:56	4.4	20.7	5.6	2.5	0.1645	3
4/21/2018 23:57	2.9	20.7	5.7	2.5	0.1441	3
4/21/2018 23:58	5.4	20.8	5.6	2.5	0.1581	2.9
4/21/2018 23:59	4.2	20.9	5.6	2.5	0.1539	2.6
4/22/2018 0:00	3.7	21	5.6	2.5	0.1566	2.5
4/22/2018 0:01	1.5	20.8	5.5	2.5	0.1559	2.5
4/22/2018 0:02	3.2	20.9	5.5	2.5	0.1529	2.6
4/22/2018 0:03	4.1	21	5.5	2.5	0.1541	2.7
4/22/2018 0:04	5	20.9	5.5	2.5	0.1474	2.7
4/22/2018 0:05	1.6	20.6	5.5	2.5	0.1573	2.6

4/22/2018 0:06	3.5	20.9	5.5	2.5	0.153	2.7
4/22/2018 0:07	2.2	21	5.4	2.5	0.1569	2.7
4/22/2018 0:08	4	20.9	5.4	2.5	0.1456	2.6
4/22/2018 0:09	4.9	20.7	5.4	2.5	0.1545	2.7
4/22/2018 0:10	4.8	20.9	5.4	2.5	0.1502	2.7
4/22/2018 0:11	4	20.8	5.3	2.5	0.1513	2.6
4/22/2018 0:12	3.6	20.9	5.4	2.5	0.152	2.7
4/22/2018 0:13	3.5	20.8	5.3	2.5	0.1469	2.7
4/22/2018 0:14	3.7	20.8	5.3	2.5	0.1556	2.7
4/22/2018 0:15	4.9	21	5.2	2.5	0.1401	2.8
4/22/2018 0:16	4.7	20.9	5.2	2.5	0.1452	2.8
4/22/2018 0:17	3.7	21	5.2	2.5	0.1492	3
4/22/2018 0:18	3.7	20.7	5.2	2.5	0.1465	3
4/22/2018 0:19	3.4	21	5.2	2.5	0.154	3
4/22/2018 0:20	3.6	20.8	5.2	2.5	0.1397	2.5
4/22/2018 0:21	3	21	5.1	2.5	0.149	2.5
4/22/2018 0:22	3.4	21	5.1	2.5	0.15	2.8
4/22/2018 0:23	4.8	21.1	5.1	2.5	0.1443	2.8
4/22/2018 0:24	4.4	20.9	5.1	2.5	0.1454	2.9
4/22/2018 0:25	3.5	20.8	5.1	2.5	0.1465	2.8
4/22/2018 0:26	3.3	20.8	5	2.5	0.1481	2.9
4/22/2018 0:27	4.4	20.9	4.9	2.5	0.1394	2.9
4/22/2018 0:28	3	20.9	4.9	2.5	0.1549	3
4/22/2018 0:29	3.2	21	4.9	2.5	0.1369	3
4/22/2018 0:30	3.7	20.9	5	2.5	0.1547	3
4/22/2018 0:31	3.4	20.7	4.9	2.5	0.1491	3
4/22/2018 0:32	4.2	20.7	4.9	2.5	0.1477	3.1
4/22/2018 0:33	5.1	21.1	4.9	2.5	0.1517	3.1
4/22/2018 0:34	4.4	20.9	4.8	2.5	0.1354	3.1
4/22/2018 0:35	3.9	21	4.8	2.5	0.1528	3
4/22/2018 0:36	4.8	20.8	4.7	2.4	0.1315	2.9
4/22/2018 0:37	4.4	21.2	4.7	2.4	0.1512	2.6
4/22/2018 0:38	4.4	20.9	4.7	2.4	0.129	2.6
4/22/2018 0:39	3.2	20.9	4.7	2.4	0.1436	2.6
4/22/2018 0:40	3.9	20.9	4.7	2.4	0.1419	2.4
4/22/2018 0:41	4	21	4.7	2.4	0.141	2.4
4/22/2018 0:42	3.6	21	4.7	2.4	0.15	2.6
4/22/2018 0:43	3.8	20.9	4.7	2.4	0.136	2.6
4/22/2018 0:44	3	21.1	4.6	2.4	0.1511	2.6
4/22/2018 0:45	3.4	21.1	4.6	2.4	0.13	2.8
4/22/2018 0:46	3.6	20.9	4.6	2.4	0.1532	2.8
4/22/2018 0:47	4.9	21.1	4.5	2.4	0.1334	2.7
4/22/2018 0:48	4.4	20.9	4.5	2.4	0.1522	2.7
4/22/2018 0:49	4.8	21.1	4.5	2.4	0.138	2.7
4/22/2018 0:50	3	21	4.4	2.4	0.1424	2.9
4/22/2018 0:51	4.8	21.2	4.5	2.4	0.1495	3
4/22/2018 0:52	2.3	21.2	4.5	2.4	0.1399	2.9

4/22/2018 0:53	4.1	21.2	4.4	2.4	0.1494	3
4/22/2018 0:54	4.3	21.3	4.4	2.4	0.1334	3
4/22/2018 0:55	4.4	21	4.4	2.4	0.1459	3.1
4/22/2018 0:56	3.9	21.2	4.3	2.4	0.1363	3.1
4/22/2018 0:57	5.4	21.2	4.3	2.4	0.1417	3.2
4/22/2018 0:58	4.6	20.9	4.3	2.4	0.1427	3.2
4/22/2018 0:59	4.8	21	4.3	2.4	0.1372	3.2
4/22/2018 1:00	3.2	20.9	4.3	2.4	0.1447	3.1
4/22/2018 1:01	4.3	21.4	4.3	2.4	0.1355	3.1
4/22/2018 1:02	4.2	20.9	4.2	2.4	0.1441	3
4/22/2018 1:03	4.1	21	4.2	2.4	0.1323	2.9
4/22/2018 1:04	3.4	21.2	4.2	2.4	0.1419	2.8
4/22/2018 1:05	4.3	21.2	4.2	2.4	0.1321	2.9
4/22/2018 1:06	4.6	21.2	4.2	2.3	0.1371	2.8
4/22/2018 1:07	3.6	21.1	4.2	2.3	0.1409	2.6
4/22/2018 1:08	5.1	21.4	4.1	2.3	0.1297	2.7
4/22/2018 1:09	2.8	20.8	4.1	2.4	0.1444	2.6
4/22/2018 1:10	3.9	20.7	4.2	2.4	0.1253	2.6
4/22/2018 1:11	3.8	21	4.2	2.4	0.1414	2.8
4/22/2018 1:12	4.1	21.1	4.2	2.4	0.1331	2.9
4/22/2018 1:13	3.3	20.9	4.1	2.4	0.1349	3.1
4/22/2018 1:14	4.7	21	4.1	2.3	0.1336	3.2
4/22/2018 1:15	4.1	20.8	4.1	2.3	0.1315	3.2
4/22/2018 1:16	2.9	20.9	4.1	2.4	0.138	3.1
4/22/2018 1:17	3.5	21	4.1	2.4	0.1274	3.1
4/22/2018 1:18	4.7	20.8	4.1	2.4	0.142	3.1
4/22/2018 1:19	2.6	21.2	4.1	2.4	0.1304	3.2
4/22/2018 1:20	3.4	21.1	4.1	2.4	0.1358	3.3
4/22/2018 1:21	5.1	21.1	4.1	2.4	0.1296	3.3
4/22/2018 1:22	4.2	21	4.1	2.4	0.1343	3.3
4/22/2018 1:23	3.2	20.8	4.1	2.4	0.139	3.3
4/22/2018 1:24	3.8	20.8	4.1	2.4	0.1241	2.8
4/22/2018 1:25	3.9	21	4.1	2.3	0.1372	2.8
4/22/2018 1:26	5.3	21	4.1	2.4	0.1222	3.1
4/22/2018 1:27	3.1	21	4	2.3	0.1342	3.2
4/22/2018 1:28	3.8	20.9	4	2.3	0.1257	3.2
4/22/2018 1:29	4.2	20.9	4.1	2.3	0.1348	3.2
4/22/2018 1:30	4.4	20.9	4.1	2.3	0.139	3.2
4/22/2018 1:31	4.3	20.6	4	2.3	0.1429	3.3
4/22/2018 1:32	4.6	21	4	2.3	0.142	3.4
4/22/2018 1:33	3.7	21.2	4	2.3	0.1274	3.4
4/22/2018 1:34	3.2	21.1	4	2.3	0.1414	3.4
4/22/2018 1:35	4.3	20.9	4	2.3	0.1237	3.4
4/22/2018 1:36	3.9	20.8	4	2.3	0.1349	3.4
4/22/2018 1:37	4	20.9	4	2.3	0.1261	3.4
4/22/2018 1:38	4.8	21	4	2.3	0.13	3.4
4/22/2018 1:39	3.3	21	4	2.3	0.1381	3.5

4/22/2018 1:40	3.7	20.9	4	2.3	0.1252	3.5
4/22/2018 1:41	4.6	21	4	2.3	0.1327	3.4
4/22/2018 1:42	5.4	21	4	2.3	0.1268	3.3
4/22/2018 1:43	3.7	21.1	4	2.3	0.1417	3.2
4/22/2018 1:44	3.4	21	4	2.3	0.1299	3
4/22/2018 1:45	3.4	21.1	4	2.4	0.126	2.9
4/22/2018 1:46	3.5	21	3.9	2.4	0.127	3.1
4/22/2018 1:47	4.1	21	3.9	2.3	0.1279	3.3
4/22/2018 1:48	3.8	21	3.9	2.3	0.131	3.2
4/22/2018 1:49		21.1	3.9	2.3	0.1286	3.1
4/22/2018 1:50	4	21	3.9	2.3	0.131	2.6
4/22/2018 1:51	2.9	20.8	3.9	2.3	0.1249	2.6
4/22/2018 1:52		21.1	3.9	2.3	0.1394	2.6
4/22/2018 1:53	7	20.9	3.9	2.3	0.1361	2.8
4/22/2018 1:54		21	3.9	2.3	0.1313	2.9
4/22/2018 1:55		21.1	3.9	2.3	0.1361	3
4/22/2018 1:56		21.2	3.9	2.3	0.1242	3.1
4/22/2018 1:57	4.3	21.2	3.9	2.3	0.1316	3
4/22/2018 1:58	9.3	20.8	3.9	2.3	0.128	2.9
4/22/2018 1:59		21	3.9	2.3	0.1355	2.9
4/22/2018 2:00		20.9	3.9	2.3	0.1257	2.9
4/22/2018 2:01		21.1	3.9	2.3	0.1271	3
4/22/2018 2:02		21	3.9	2.3	0.1376	3
4/22/2018 2:03	9.4	21	3.9	2.3	0.1162	2.9
4/22/2018 2:04	4	21	3.9	2.3	0.1405	2.9
4/22/2018 2:05	2.9	21.2	3.9	2.3	0.1219	3
4/22/2018 2:06	2.2	21.2	3.9	2.3	0.131	3.1
4/22/2018 2:07	3.9	21	3.9	2.3	0.1188	3.1
4/22/2018 2:08	3.9	21.1	3.9	2.3	0.1271	3.1
4/22/2018 2:09	3.1	21	3.9	2.3	0.1279	3.2
4/22/2018 2:10	3.9	21	3.8	2.3	0.1183	3.4
4/22/2018 2:11	5.2	21	3.9	2.3	0.1441	3.4
4/22/2018 2:12	4.8	21	3.9	2.3	0.1239	3.5
4/22/2018 2:13	3.6	21	3.9	2.3	0.1346	3.5
4/22/2018 2:14	3.9	21.1	3.8	2.3	0.1141	3.5
4/22/2018 2:15	3.6	20.9	3.8	2.3	0.1289	3.3
4/22/2018 2:16	2.5	20.9	3.8	2.3	0.1243	3.3
4/22/2018 2:17	4	21	3.8	2.3	0.1249	3.3
4/22/2018 2:18	4.3	20.8	3.8	2.3	0.1271	3.4
4/22/2018 2:19	3.6	20.9	3.8	2.3	0.1163	3.6
4/22/2018 2:20	3.7	21	3.8	2.3	0.1286	3.6
4/22/2018 2:21	3.4	20.8	3.8	2.3	0.1182	3.7
4/22/2018 2:22	3.3	20.7	3.8	2.3	0.1326	3.6
4/22/2018 2:23	1.6	21	3.8	2.3	0.1212	3.7
4/22/2018 2:24	3.6	20.8	3.8	2.3	0.127	3.7
4/22/2018 2:25	4.2	20.9	3.8	2.3	0.1319	3.7
4/22/2018 2:26	3.7	21.1	3.8	2.2	0.122	3.8

4/22/2018 2:27	3.9	20.8	3.8	2.3	0.1314	3.8
4/22/2018 2:28	3.6	20.9	3.8	2.3	0.1217	3.8
4/22/2018 2:29	2.8	20.6	3.8	2.3	0.1341	3.9
4/22/2018 2:30	4.6	20.8	3.8	2.3	0.1133	3.9
4/22/2018 2:31	4.4	20.9	3.8	2.3	0.1315	2.8
4/22/2018 2:32	3.8	20.9	3.8	2.3	0.1234	2.4
4/22/2018 2:33	3	20.9	3.8	2.3	0.1238	2.5
4/22/2018 2:34	4.6	20.7	3.8	2.3	0.1233	2.8
4/22/2018 2:35	4.2	20.5	3.8	2.3	0.1196	3.1
4/22/2018 2:36	4	20.6	3.8	2.3	0.1261	3.4
4/22/2018 2:37	5.7	20.8	3.8	2.3	0.1193	3.5
4/22/2018 2:38	3	20.5	3.8	2.3	0.131	3.6
4/22/2018 2:39	3.2	20.8	3.8	2.3	0.1119	3.6
4/22/2018 2:40	4	20.8	3.8	2.3	0.1144	3.6
4/22/2018 2:41	3.4	20.8	3.8	2.3	0.1259	3.7
4/22/2018 2:42	2.8	20.7	3.8	2.3	0.1158	3.8
4/22/2018 2:43	2.8	20.6	3.8	2.3	0.1274	3.7
4/22/2018 2:44	4.3	20.8	3.8	2.3	0.1107	3.7
4/22/2018 2:45	3.5	20.5	3.8	2.3	0.1315	3.8
4/22/2018 2:46	3	20.5	3.8	2.3	0.1204	3.8
4/22/2018 2:47	4.4	20.6	3.8	2.3	0.1255	2.7
4/22/2018 2:48	3.1	20.6	3.8	2.3	0.1295	2.2
4/22/2018 2:49	2.7	20.7	3.8	2.3	0.121	2.5
4/22/2018 2:50	4.3	20.7	3.8	2.3	0.1291	2.6
4/22/2018 2:51	4.6	20.8	3.8	2.3	0.1215	2.5
4/22/2018 2:52	4.1	20.7	3.9	2.3	0.1314	2.6
4/22/2018 2:53	4.2	20.6	3.8	2.3	0.1182	2.8
4/22/2018 2:54	3.5	20.5	3.9	2.3	0.1252	3
4/22/2018 2:55	3.7	20.8	3.8	2.3	0.1241	3.2
4/22/2018 2:56	4	20.5	3.8	2.3	0.1216	3.4
4/22/2018 2:57	2.7	20.6	3.9	2.3	0.1283	3.5
4/22/2018 2:58	3.3	20.6	3.9	2.3	0.116	3.6
4/22/2018 2:59	4.5	20.6	3.9	2.2	0.13	3.7
4/22/2018 3:00	3.8	20.6	3.8	2.2	0.1174	3.7
4/22/2018 3:01	4.3	20.5	3.8	2.3	0.1332	3.8
4/22/2018 3:02	3.5	20.7	3.9	2.3	0.1208	3.8
4/22/2018 3:03	3.6	20.6	3.9	2.3	0.1273	3.9
4/22/2018 3:04	3.3	20.4	3.9	2.3	0.1254	3.9
4/22/2018 3:05	3.1	20.5	3.9	2.3	0.1214	3.7
4/22/2018 3:06	3.1	20.5	3.9	2.3	0.125	3.6
4/22/2018 3:07	4.2	20.6	3.9	2.3	0.1169	3.7
4/22/2018 3:08	2.5	20.7	3.9	2.3	0.1242	3.8
4/22/2018 3:09	4.1	20.5	4	2.3	0.1107	3.9
4/22/2018 3:10	3.5	20.4	4	2.3	0.1302	3.9
4/22/2018 3:11	3.8	20.3	4	2.3	0.1162	3.7
4/22/2018 3:12	3.1	20.5	3.9	2.3	0.1262	3.5
4/22/2018 3:13	4	20.5	4.2	2.3	0.1161	3.4

4/22/2018 3:14	2.4	20.4	3.9	2.3	0.1195	3.6
4/22/2018 3:15	3.6	20.7	4	2.3	0.1254	3.8
4/22/2018 3:16	3.7	20.4	3.9	2.3	0.1233	3.9
4/22/2018 3:17	3.2	20.5	3.9	2.3	0.1282	3.9
4/22/2018 3:18	3.9	20.4	4	2.4	0.1152	3.8
4/22/2018 3:19	3.1	20.2	3.9	2.4	0.1182	3.8
4/22/2018 3:20	3.2	20.3	3.8	2.4	0.1172	3.9
4/22/2018 3:21	3.2	20.6	3.9	2.4	0.1151	3.9
4/22/2018 3:22	4.3	20.3	3.9	2.4	0.1299	3.8
4/22/2018 3:23	3.9	20.4	3.8	2.4	0.1132	3.7
4/22/2018 3:24	3.5	20.4	3.9	2.4	0.1179	3.7
4/22/2018 3:25	2.3	20.2	3.9	2.4	0.1184	3.8
4/22/2018 3:26	4.6	20.3	3.9	2.4	0.1284	3.6
4/22/2018 3:27	4.8	20.1	3.9	2.4	0.1273	3.7
4/22/2018 3:28	3.8	20.5	3.9	2.4	0.1199	3.8
4/22/2018 3:29	4.1	20.4	3.9	2.4	0.1275	3.9
4/22/2018 3:30	3.2	20.6	3.9	2.4	0.1122	3.9
4/22/2018 3:31	4.3	20.2	4	2.4	0.1276	3.8
4/22/2018 3:32	3.9	20.4	4	2.4	0.1193	3.8
4/22/2018 3:33	3	20.2	4	2.4	0.1242	3.7
4/22/2018 3:34	1.7	20	4	2.4	0.1213	3.6
4/22/2018 3:35	4.8	20.3	4	2.4	0.1177	3.7
4/22/2018 3:36	4.4	20.2	4	2.4	0.125	3.7
4/22/2018 3:37	2.2	20.2	4.1	2.4	0.1105	3.6
4/22/2018 3:38	2.7	20.2	4.1	2.4	0.1276	3.5
4/22/2018 3:39	3.3	20.3	4.1	2.4	0.1189	3.6
4/22/2018 3:40	4.4	20.4	4.1	2.4	0.1209	3.5
4/22/2018 3:41	3.6	20.1	4.1	2.4	0.1219	3.5
4/22/2018 3:42	4.2	20.1	4.1	2.4	0.1228	3.2
4/22/2018 3:43	4.2	20.4	4.1	2.4	0.1238	2.8
4/22/2018 3:44	4.1	20.3	4.1	2.4	0.1203	3.1
4/22/2018 3:45	3.5	20.1	4.1	2.4	0.1281	3.3
4/22/2018 3:46	3.2	20.4	4.2	2.4	0.114	3.5
4/22/2018 3:47	2.7	20.4	4.3	2.4	0.1295	3.6
4/22/2018 3:48	4.1	20.4	4.3	2.4	0.1171	3.7
4/22/2018 3:49	2.9	20.3	4.3	2.4	0.1198	3.8
4/22/2018 3:50	3.9	20.1	4.3	2.4	0.1238	3.8
4/22/2018 3:51	3.7	20.3	4.2	2.3	0.1085	3.8
4/22/2018 3:52	4	20.3	4.2	2.4	0.1233	3.8
4/22/2018 3:53	3.9	20.5	4.2	2.4	0.1127	3.8
4/22/2018 3:54	5.1	20.4	4.1	2.4	0.1238	3.8
4/22/2018 3:55	4.2	20.5	4.2	2.4	0.118	3.8
4/22/2018 3:56	3.9	20.2	4.1	2.4	0.1187	3.7
4/22/2018 3:57	3.4	20.3	4.1	2.4	0.1237	3.2
4/22/2018 3:58	3.6	20.2	4.1	2.4	0.1079	3.2
4/22/2018 3:59	2.9	20.3	4.1	2.4	0.1263	3.5
4/22/2018 4:00	3.1	20.1	4	2.4	0.1117	3.7

4/22/2018 4:01	3.1	20.1	4	2.4	0.1289	3.7
4/22/2018 4:02	2.6	20.2	4	2.4	0.1187	3.8
4/22/2018 4:03	3.6	20.4	4.1	2.4	0.1334	3.9
4/22/2018 4:04	6.3	20.2	4.1	2.4	0.1187	4
4/22/2018 4:05	4.5	20.1	4.1	2.4	0.1173	4.1
4/22/2018 4:06	3.8	20.2	4.1	2.4	0.1244	4.1
4/22/2018 4:07	2.7	20.2	4.1	2.4	0.1212	4.1
4/22/2018 4:08	4.3	20.3	4.1	2.4	0.1187	4.1
4/22/2018 4:09	4.6	20.6	4.1	2.4	0.1123	4.1
4/22/2018 4:10	4	20.2	4.1	2.4	0.1292	4.1
4/22/2018 4:11	2.3	20.4	4.1	2.4	0.1166	4.1
4/22/2018 4:12	3.1	20.4	4.1	2.4	0.119	4.1
4/22/2018 4:13	3.7	20.4	4.1	2.4	0.128	4.1
4/22/2018 4:14	3.9	20.4	4	2.4	0.1114	4.1
4/22/2018 4:15	3.6	20.4	4.1	2.3	0.1215	4.1
4/22/2018 4:16	2.4	20.5	4	2.3	0.1214	4.1
4/22/2018 4:17	3.1	20.5	4	2.3	0.1265	4.1
4/22/2018 4:18	3.3	20.5	4	2.3	0.1147	4.1
4/22/2018 4:19	3.4	20.2	3.9	2.3	0.1191	4.1
4/22/2018 4:20	4.1	20.2	3.9	2.3	0.1172	4.1
4/22/2018 4:21	3.2	20.2	3.9	2.3	0.1174	4.1
4/22/2018 4:22	4.8	20.2	3.8	2.3	0.1246	4.1
4/22/2018 4:23	4.1	20.3	3.8	2.4	0.1148	4
4/22/2018 4:24	2.9	20.1	3.8	2.4	0.1268	4
4/22/2018 4:25	4.6	20.3	3.8	2.4	0.1109	3.9
4/22/2018 4:26	3.4	20.1	3.8	2.4	0.1206	3.9
4/22/2018 4:27	4.6	20.2	3.8	2.4	0.1113	3.9
4/22/2018 4:28	2	20.3	3.8	2.4	0.1151	3.9
4/22/2018 4:29	3	20.7	3.8	2.4	0.1174	3.8
4/22/2018 4:30	2.7	20.7	3.8	2.4	0.1099	3.9
4/22/2018 4:31	4.4	20.2	3.8	2.4	0.1271	3.9
4/22/2018 4:32	2.9	20.3	3.8	2.4	0.1154	4.1
4/22/2018 4:33	3.7	20.1	3.8	2.4	0.1217	4.1
4/22/2018 4:34	1.5	20.4	3.8	2.4	0.1202	4.1
4/22/2018 4:35	4.2	20.4	3.8	2.4	0.1248	4
4/22/2018 4:36	3.3	20.6	3.8	2.4	0.1234	4
4/22/2018 4:37	3.4	20.3	3.7	2.4	0.1069	4.1
4/22/2018 4:38	3.7	20.4	3.7	2.4	0.1189	4.1
4/22/2018 4:39	3.3	20.5	3.7	2.4	0.1085	4
4/22/2018 4:40	4.7	20.5	3.7	2.4	0.1074	4
4/22/2018 4:41	3.7	20.4	3.7	2.3	0.1148	4
4/22/2018 4:42	4.1	20	3.7	2.4	0.1093	4
4/22/2018 4:43	3.4	20.4	3.7	2.4	0.1231	3.7
4/22/2018 4:44	3.4	20.2	3.7	2.4	0.1104	3.5
4/22/2018 4:45	3.4	20.5	3.6	2.4	0.1209	3.7
4/22/2018 4:46		20.6	3.6	2.4	0.117	3.9
4/22/2018 4:47		20.3	3.6	2.4	0.1134	3.9

4/22/2018 4:48	7.7	20.4	3.6	2.4	0.1178	4
4/22/2018 4:49		20.4	3.6	2.4	0.103	4.1
4/22/2018 4:50	6.8	20.2	3.6	2.4	0.1218	4.2
4/22/2018 4:51		20.3	3.6	2.4	0.1102	4.3
4/22/2018 4:52	4.2	20.2	3.6	2.4	0.1253	4.3
4/22/2018 4:53	4.4	20.3	3.6	2.4	0.1086	4.4
4/22/2018 4:54		20	3.6	2.4	0.1152	4.4
4/22/2018 4:55	4	20.1	3.6	2.4	0.1179	4.4
4/22/2018 4:56		20.3	3.6	2.4	0.1148	4.4
4/22/2018 4:57		20.2	3.7	2.4	0.1272	4.1
4/22/2018 4:58	4.3	20.5	3.7	2.4	0.1063	4.3
4/22/2018 4:59	2.1	20.4	3.7	2.3	0.1135	4.4
4/22/2018 5:00		20.5	3.7	2.3	0.1086	4.4
4/22/2018 5:01		20.4	3.7	2.3	0.1171	4.4
4/22/2018 5:02	2.7	20.4	3.6	2.3	0.1205	4.4
4/22/2018 5:03	4	20.2	3.6	2.3	0.1116	4.5
4/22/2018 5:04	2.9	20.3	3.6	2.3	0.1234	4.5
4/22/2018 5:05	4.3	20.3	3.6	2.3	0.1073	4.5
4/22/2018 5:06	3.9	20.3	3.6	2.3	0.117	4.4
4/22/2018 5:07	2.7	20.4	3.6	2.3	0.1112	4.4
4/22/2018 5:08	3.7	20.4	3.5	2.3	0.1113	4.1
4/22/2018 5:09	3.4	20.3	3.5	2.3	0.1141	2.4
4/22/2018 5:10	3.2	20	3.5	2.3	0.1071	2.3
4/22/2018 5:11	3.6	20.2	3.5	2.3	0.1193	2.5
4/22/2018 5:12	2.5	20.2	3.5	2.3	0.1043	2.9
4/22/2018 5:13	3.5	20.1	3.5	2.4	0.1203	3.3
4/22/2018 5:14	2.7	20.2	3.5	2.4	0.1099	3
4/22/2018 5:15	2.9	20.5	3.5	2.4	0.1199	2.3
4/22/2018 5:16	2.5	20.2	3.5	2.4	0.117	2.1
4/22/2018 5:17	4.3	20.3	3.6	2.4	0.1093	2.3
4/22/2018 5:18	2.5	19.9	3.6	2.4	0.1087	2.2
4/22/2018 5:19	3.4	20.1	3.5	2.4	0.1059	2.1
4/22/2018 5:20	5.8	20.3	3.6	2.4	0.1165	2.3
4/22/2018 5:21	3.1	20.1	3.6	2.4	0.1128	2.6
4/22/2018 5:22	3.6	20	3.5	2.3	0.118	2.6
4/22/2018 5:23	3.2	20.3	3.5	2.3	0.1167	2.1
4/22/2018 5:24	4.4	20	3.6	2.3	0.1067	2.5
4/22/2018 5:25	2.9	20.2	3.6	2.3	0.1198	2.9
4/22/2018 5:26	3.4	20	3.6	2.3	0.1017	3.1
4/22/2018 5:27	3.1	20.2	3.5	2.3	0.1225	3.4
4/22/2018 5:28	3.1	20.2	3.5	2.3	0.111	3.6
4/22/2018 5:29	4	20.1	3.5	2.3	0.1139	3.7
4/22/2018 5:30	2.7	20.2	3.4	2.3	0.1205	3.9
4/22/2018 5:31	4.6	20.2	3.4	2.3	0.1082	4
4/22/2018 5:32	4.3	20	3.4	2.3	0.1187	4
4/22/2018 5:33	4.8	20	3.4	2.3	0.1049	4
4/22/2018 5:34	3.1	20.1	3.5	2.4	0.1171	4.1

4/22/2018 5:35	3.4	19.9	3.6	2.4	0.1065	4.1
4/22/2018 5:36	5.1	20.2	3.6	2.4	0.119	3.9
4/22/2018 5:37	2.7	20	3.6	2.3	0.1186	3.4
4/22/2018 5:38	1.9	20.2	3.6	2.3	0.1075	3.4
4/22/2018 5:39	4.3	20.2	3.5	2.3	0.1221	3.8
4/22/2018 5:40	4.6	20.2	3.6	2.3	0.1052	4.2
4/22/2018 5:41	3.4	20	3.7	2.3	0.1127	4.1
4/22/2018 5:42	4.2	20.1	3.7	2.3	0.1092	4
4/22/2018 5:43	3.2	20.2	3.7	2.3	0.1164	4.1
4/22/2018 5:44	4.5	20	3.6	2.3	0.1177	4.3
4/22/2018 5:45	3	19.9	3.7	2.3	0.1043	4.3
4/22/2018 5:46	3.3	20.3	3.7	2.3	0.1223	4.1
4/22/2018 5:47	3.2	20	3.7	2.3	0.1019	4.2
4/22/2018 5:48	3.7	20.2	3.5	2.3	0.1188	4.3
4/22/2018 5:49	3.3	20	3.5	2.3	0.1069	4.4
4/22/2018 5:50	3.4	19.9	3.5	2.3	0.1148	4.5
4/22/2018 5:51	3.6	20	3.5	2.3	0.1079	4.5
4/22/2018 5:52	3	20.1	3.5	2.3	0.1102	4.5
4/22/2018 5:53	3.1	20	3.5	2.3	0.1205	4.6
4/22/2018 5:54	2.1	19.8	3.6	2.4	0.1057	4.6
4/22/2018 5:55	4.1	20	3.7	2.4	0.1117	4.6
4/22/2018 5:56	2.9	20	4	2.3	0.1136	4.6
4/22/2018 5:57	3.6	20	4	2.3	0.1122	4.6
4/22/2018 5:58	2.4	19.8	3.8	2.3	0.1187	4.6
4/22/2018 5:59	4.2	20	3.8	2.3	0.1079	4.7
4/22/2018 6:00	2.6	20.1	4	2.3	0.1192	4.7
4/22/2018 6:01	4.6	20.1	3.9	2.3	0.1029	4.8
4/22/2018 6:02	2.6	20	4	2.3	0.1151	4.8
4/22/2018 6:03	3.3	19.9	4.1	2.3	0.1148	4.7
4/22/2018 6:04	2.2	19.8	4	2.3	0.1139	4.8
4/22/2018 6:05	4.8	20	4.1	2.4	0.1095	4.7
4/22/2018 6:06	3.5	19.7	4.3	2.3	0.107	4.7
4/22/2018 6:07	2.5	20	4.2	2.3	0.1236	4.8
4/22/2018 6:08	3	19.8	4	2.4	0.1078	4.8
4/22/2018 6:09	2.5	20	3.9	2.4	0.1188	4.7
4/22/2018 6:10	3.5	19.8	3.8	2.4	0.1116	4.7
4/22/2018 6:11	3.3	19.7	3.9	2.4	0.114	4.8
4/22/2018 6:12	3.7	19.6	4.2	2.4	0.1192	4.8
4/22/2018 6:13	4.1	19.9	4.2	2.4	0.1079	4.8
4/22/2018 6:14	3	19.6	4.3	2.4	0.1229	4.8
4/22/2018 6:15	3.3	19.8	4.2	2.4	0.1087	4.8
4/22/2018 6:16	3.5	19.8	4.4	2.4	0.1169	4.8
4/22/2018 6:17	4	19.7	4.5	2.4	0.118	4.8
4/22/2018 6:18	3.5	19.8	4.5	2.3	0.115	4.6
4/22/2018 6:19	3.8	19.7	4.7	2.3	0.1194	3.5
4/22/2018 6:20	2.6	19.9	4.5	2.3	0.1122	2.8
4/22/2018 6:21	4.2	19.6	4.8	2.4	0.1284	3.1

4/22/2018 6:22	2.5	19.6	4.9	2.3	0.1151	3.6
4/22/2018 6:23	3.8	19.5	4.4	2.4	0.1214	3.9
4/22/2018 6:24	2.8	19.5	4.8	2.4	0.1074	4.1
4/22/2018 6:25	2.7	19.6	4.6	2.4	0.1181	4.3
4/22/2018 6:26	2.7	19.4	4.5	2.4	0.1217	4.3
4/22/2018 6:27	3.9	19.9	4.6	2.4	0.1049	4.4
4/22/2018 6:28	3.2	19.9	4.4	2.4	0.1162	4.5
4/22/2018 6:29	2.7	19.7	4.4	2.4	0.1131	4.6
4/22/2018 6:30	2.6	19.8	4.6	2.5	0.124	4.7
4/22/2018 6:31	2.9	19.5	4.9	2.5	0.1223	4.8
4/22/2018 6:32	2.5	20	4.9	2.5	0.114	4.9
4/22/2018 6:33	3.9	19.8	4.9	2.5	0.1279	4.9
4/22/2018 6:34	3.1	19.9	5	2.4	0.1149	4.9
4/22/2018 6:35	2.9	19.6	5.1	2.4	0.1206	4.9
4/22/2018 6:36	5.1	19.9	5.1	2.4	0.1193	4.9
4/22/2018 6:37	4.2	19.7	4.8	2.4	0.1159	4.8
4/22/2018 6:38	4.5	19.6	4.8	2.4	0.1179	4.7
4/22/2018 6:39	4.1	19.8	4.9	2.4	0.1111	4.7
4/22/2018 6:40	3.7	19.8	4.9	2.4	0.1181	4.7
4/22/2018 6:41	3.4	19.7	4.8	2.4	0.1113	4.6
4/22/2018 6:42	3.7	19.4	5	2.4	0.1152	4.4
4/22/2018 6:43	3.1	20	5	2.4	0.1218	3.6
4/22/2018 6:44	4.6	19.8	5	2.5	0.1084	4
4/22/2018 6:45	2.7	19.9	4.8	2.5	0.1162	4.5
4/22/2018 6:46	4.6	19.8	4.6	2.5	0.1158	4.7
4/22/2018 6:47	4	19.9	4.7	2.5	0.1223	4.7
4/22/2018 6:48	3.9	19.7	4.9	2.5	0.1155	4.7
4/22/2018 6:49	3.7	19.7	4.9	2.5	0.1104	4.7
4/22/2018 6:50	2.8	19.5	5	2.5	0.123	4.7
4/22/2018 6:51	2.5	19.7	4.9	2.5	0.1102	4.8
4/22/2018 6:52	4.5	19.8	5	2.5	0.1224	4.8
4/22/2018 6:53	4.5	19.8	5.2	2.4	0.1164	4.8
4/22/2018 6:54	3.4	20.2	5.1	2.4	0.1182	4.8
4/22/2018 6:55	3.6	19.7	5.1	2.4	0.124	4.6
4/22/2018 6:56	3.6	19.9	4.9	2.4	0.1181	4
4/22/2018 6:57	2.3	19.8	5.1	2.4	0.118	4.2
4/22/2018 6:58	3.4	19.9	5.2	2.4	0.1068	3.6
4/22/2018 6:59	4	19.9	5.1	2.4	0.1203	3
4/22/2018 7:00	4.2	20.2	5.2	2.4	0.1182	3.5
4/22/2018 7:01	3.3	19.7	5.1	2.4	0.1138	3.7
4/22/2018 7:02	3.7	19.9	4.9	2.5	0.1208	4.1
4/22/2018 7:03	2.6	19.7	4.9	2.4	0.1131	4.3
4/22/2018 7:04	4.8	19.8	4.9	2.4	0.1291	4.5
4/22/2018 7:05	2.9	20.1	5	2.4	0.114	4.6
4/22/2018 7:06	4	20	5.2	2.4	0.1137	4.7
4/22/2018 7:07	2.8	20	5.1	2.4	0.11	4.8
4/22/2018 7:08	4.1	19.8	5	2.4	0.1205	4.8

4/22/2018 7:09	3.8	19.9	5.1	2.5	0.1202	4.5
4/22/2018 7:10	3	19.9	5.1	2.5	0.1126	4.1
4/22/2018 7:11	2.7	19.7	5.3	2.5	0.1242	4.2
4/22/2018 7:12	2.5	19.7	5.2	2.5	0.108	4.4
4/22/2018 7:13	3.2	20	5.2	2.5	0.1219	4.3
4/22/2018 7:14	2.3	20.1	5.1	2.5	0.1134	4.4
4/22/2018 7:15	4.1	19.7	5.1	2.5	0.1088	4.5
4/22/2018 7:16	4.7	19.9	5.3	2.5	0.1188	4.6
4/22/2018 7:17	2.9	20	5.4	2.4	0.1106	4.5
4/22/2018 7:18	4	20.1	5.2	2.4	0.1224	4.5
4/22/2018 7:19	3.6	19.9	5.1	2.4	0.1111	4.4
4/22/2018 7:20	4.4	19.8	5.1	2.4	0.1174	3.8
4/22/2018 7:21	4.5	20	5.3	2.4	0.117	3
4/22/2018 7:22	2	19.9	5.1	2.4	0.1153	3.8
4/22/2018 7:23	3	20	5	2.4	0.1235	4.1
4/22/2018 7:24	2.2	20	5.1	2.4	0.1067	4.3
4/22/2018 7:25	3.3	19.7	5.2	2.4	0.1217	4.3
4/22/2018 7:26	2.3	19.7	5.2	2.4	0.1177	4.2
4/22/2018 7:27	2.4	19.6	5.1	2.5	0.1113	4.4
4/22/2018 7:28	3.4	19.8	5.1	2.5	0.1205	4.6
4/22/2018 7:29	2.7	19.9	5.4	2.5	0.1047	4.7
4/22/2018 7:30	4.3	19.5	5.3	2.5	0.1196	4.7
4/22/2018 7:31	3.6	19.6	5.5	2.5	0.1094	4.8
4/22/2018 7:32	4.4	19.9	5.2	2.5	0.1123	4.8
4/22/2018 7:33	2.7	19.8	5.4	2.5	0.1215	4.8
4/22/2018 7:34	2.8	19.8	5.3	2.5	0.1117	4.8
4/22/2018 7:35	4.5	19.8	5.7	2.4	0.1276	4.7
4/22/2018 7:36	3.5	19.9	5.2	2.4	0.1081	4.8
4/22/2018 7:37	3.8	19.9	5.4	2.4	0.1175	4.7
4/22/2018 7:38	2.3	19.7	5.3	2.4	0.1178	4.7
4/22/2018 7:39	1.9	19.6	5.1	2.4	0.1091	4.7
4/22/2018 7:40	3.6	19.7	5	2.4	0.1153	4.3
4/22/2018 7:41	3.1	19.7	5.2	2.4	0.1087	4.4
4/22/2018 7:42	2.3	19.7	5.4	2.4	0.1181	4.6
4/22/2018 7:43	3.3	20	5.3	2.5	0.1058	4.6
4/22/2018 7:44	3.1	19.6	5.2	2.5	0.1135	4.6
4/22/2018 7:45	3.2	19.8	5.2	2.5	0.1173	4.8
4/22/2018 7:46	3.4	19.5	5.2	2.5	0.1074	4.7
4/22/2018 7:47	3.2	19.8	5.4	2.5	0.1186	4.9
4/22/2018 7:48	2.9	19.9	5.3	2.5	0.1083	4.8
4/22/2018 7:49	3.7	19.8	5.5	2.5	0.1258	4.8
4/22/2018 7:50	3.4	19.6	5.7	2.5	0.1098	4.8
4/22/2018 7:51	3.3	19.7	5.7	2.5	0.1189	4.5
4/22/2018 7:52	2.5	19.6	5.5	2.5	0.1236	4.6
4/22/2018 7:53	4	19.6	5.4	2.5	0.1138	4.9
4/22/2018 7:54	2.6	19.6	5.6	2.5	0.1224	4.9
4/22/2018 7:55	3.7	19.8	5.8	2.5	0.1093	5

4/22/2018 7:56	3.3	19.7	5.8	2.5	0.1142	5
4/22/2018 7:57	3.1	19.8	5.7	2.5	0.1098	5.1
4/22/2018 7:58	3.3	19.8	5.6	2.5	0.1197	5.2
4/22/2018 7:59	2.8	19.7	5.7	2.5	0.115	5.2
4/22/2018 8:00	3.6	19.8	5.6	2.5	0.1132	5.2
4/22/2018 8:01	4.4	19.7	5.3	2.5	0.1238	5.1
4/22/2018 8:02		19.7	5.4	2.5	0.1062	5.1
4/22/2018 8:03		19.9	5.6	2.5	0.1258	5
4/22/2018 8:04		19.8	5.6	2.5	0.1124	5
4/22/2018 8:05	2.4	19.6	5.4	2.5	0.1157	4.8
4/22/2018 8:06	4.7	19.5	5.6	2.6	0.1229	4.9
4/22/2018 8:07		19.8	5.7	2.6	0.1091	5
4/22/2018 8:08	0.8	19.7	5.7	2.6	0.1159	5
4/22/2018 8:09		19.8	5.9	2.5	0.1114	5
4/22/2018 8:10	4.2	19.8	5.9	2.5	0.1249	4.9
4/22/2018 8:11	3.1	19.9	5.9	2.5	0.1241	5
4/22/2018 8:12		19.5	5.7	2.5	0.1173	5
4/22/2018 8:13		19.8	5.7	2.5	0.1224	5.1
4/22/2018 8:14	3.2	19.8	5.7	2.5	0.109	5.1
4/22/2018 8:15	2.8	19.6	5.6	2.5	0.1208	4.9
4/22/2018 8:16	3.5	19.6	5.6	2.5	0.1104	4.2
4/22/2018 8:17	7	19.5	5.5	2.5	0.1159	3.4
4/22/2018 8:18	3.3	19.6	5.6	2.5	0.1228	3.8
4/22/2018 8:19	4.3	19.7	5.5	2.5	0.1148	4.2
4/22/2018 8:20	3.8	19.7	5.5	2.6	0.1214	4.4
4/22/2018 8:21	2.3	19.4	5.5	2.5	0.1066	4.6
4/22/2018 8:22	4	19.7	5.8	2.5	0.1257	4.7
4/22/2018 8:23	3	19.8	5.7	2.5	0.1203	4.7
4/22/2018 8:24	4.5	19.8	5.7	2.5	0.1201	4.8
4/22/2018 8:25	3.8	19.8	5.5	2.5	0.1277	4.8
4/22/2018 8:26	2.7	19.7	5.4	2.5	0.108	4.8
4/22/2018 8:27	1.8	19.8	5.5	2.5	0.122	4.8
4/22/2018 8:28	3.1	19.7	5.4	2.6	0.1033	4.9
4/22/2018 8:29	2.9	19.6	5.5	2.6	0.1281	4.9
4/22/2018 8:30	1.2	19.8	5.5	2.6	0.1139	5
4/22/2018 8:31	1.7	19.6	5.4	2.6	0.1206	5
4/22/2018 8:32	3.1	19.7	5.5	2.7	0.1258	5
4/22/2018 8:33	3.1	19.7	5.8	2.6	0.1163	5.1
4/22/2018 8:34	4	19.7	5.9	2.6	0.1308	5.1
4/22/2018 8:35	2.6	19.6	5.9	2.6	0.1088	5.2
4/22/2018 8:36	4.7	19.6	5.6	2.6	0.1229	5.1
4/22/2018 8:37	3.2	19.4	5.7	2.6	0.1191	5.2
4/22/2018 8:38	4.5	19.7	5.8	2.6	0.1113	5.1
4/22/2018 8:39	4.5	19.6	5.8	2.6	0.115	5.2
4/22/2018 8:40	2.2	19.7	5.8	2.6	0.1174	5.2
4/22/2018 8:41	3.5	19.5	6	2.6	0.1186	5.2
4/22/2018 8:42	3.4	19.7	5.7	2.6	0.1084	5.3

4/22/2018 8:43	2.8	19.8	5.9	2.6	0.1231	5.2
4/22/2018 8:44	4	19.7	5.7	2.6	0.1166	5.2
4/22/2018 8:45	2.8	19.4	5.7	2.6	0.1222	5.3
4/22/2018 8:46	4	19.6	5.7	2.6	0.1176	5.3
4/22/2018 8:47	3.7	19.7	5.8	2.6	0.1112	5.3
4/22/2018 8:48	3.5	19.5	5.7	2.6	0.1244	5.2
4/22/2018 8:49	1.7	19.7	5.6	2.6	0.1198	5.2
4/22/2018 8:50	3	19.4	5.6	2.6	0.1185	5
4/22/2018 8:51	3.8	19.5	5.8	2.6	0.1243	5
4/22/2018 8:52	2.9	19.4	5.8	2.6	0.1139	5.1
4/22/2018 8:53	4	19.6	5.7	2.6	0.1326	5.2
4/22/2018 8:54	2.8	19.5	5.7	2.6	0.1088	5.3
4/22/2018 8:55	3.9	19.7	5.7	2.6	0.1236	5.3
4/22/2018 8:56	3.6	19.8	5.7	2.6	0.1128	5.3
4/22/2018 8:57	1.2	19.5	5.6	2.7	0.1216	5.3
4/22/2018 8:58	4.1	19.7	5.7	2.7	0.1225	5.2
4/22/2018 8:59	3.7	19.7	5.8	2.7	0.1134	5.2
4/22/2018 9:00	4.2	19.7	5.8	2.7	0.1256	5.1
4/22/2018 9:01	4	19.6	6.1	2.7	0.1129	5.1
4/22/2018 9:02	3.8	19.6	6.1	2.6	0.1254	4.9
4/22/2018 9:03	2.7	19.8	6	2.6	0.1188	4.8
4/22/2018 9:04	4	19.8	6	2.6	0.1218	4.7
4/22/2018 9:05	3.2	19.9	6.2	2.6	0.1267	4.6
4/22/2018 9:06	2.4	19.9	6	2.6	0.116	4.5
4/22/2018 9:07	1.9	19.7	5.9	2.6	0.1311	4.6
4/22/2018 9:08	2.8	19.8	5.9	2.6	0.1148	4.5
4/22/2018 9:09	3.8	19.7	5.8	2.6	0.1269	4.3
4/22/2018 9:10	3.1	19.6	5.8	2.6	0.1143	4.2
4/22/2018 9:11	3.5	19.8	5.8	2.6	0.1248	4.1
4/22/2018 9:12	4.7	19.7	5.7	2.6	0.1209	2.2
4/22/2018 9:13	3.9	19.8	5.8	2.6	0.1184	1.5
4/22/2018 9:14	2.9	19.9	5.8	2.6	0.1259	1.5
4/22/2018 9:15	3.5	19.9	5.8	2.6	0.1161	1.5
4/22/2018 9:16	3.3	19.9	5.8	2.6	0.128	1.5
4/22/2018 9:17	3.8	19.8	5.6	2.6	0.1153	1.5
4/22/2018 9:18	3.9	19.6	5.6	2.6	0.1195	1.6
4/22/2018 9:19	2.2	19.8	5.7	2.6	0.1259	1.6
4/22/2018 9:20	2.6	19.8	5.7	2.6	0.1144	1.6
4/22/2018 9:21	2.3	19.8	5.6	2.6	0.1314	1.6
4/22/2018 9:22	3.4	19.6	5.6	2.6	0.1149	1.6
4/22/2018 9:23	3.2	19.8	5.6	2.6	0.1225	1.6
4/22/2018 9:24	3	19.9	5.6	2.7	0.1279	1.8
4/22/2018 9:25	3.8	19.8	5.6	2.7	0.1139	2
4/22/2018 9:26	2.5	20	5.5	2.7	0.1328	2
4/22/2018 9:27	1.9	20	5.6	2.7	0.1191	1.9
4/22/2018 9:28	3.7	19.9	5.6	2.7	0.1259	1.7
4/22/2018 9:29	2.3	20	5.5	2.7	0.1235	2

4/22/2018 9:30	3.7	20.1	5.5	2.7	0.1265	2.1
4/22/2018 9:31	4	19.7	5.5	2.6	0.1231	2.1
4/22/2018 9:32	4	20.1	5.4	2.6	0.12	2.2
4/22/2018 9:33	3.2	20.2	5.5	2.6	0.1326	2.3
4/22/2018 9:34	4.3	19.9	5.4	2.6	0.1171	2.4
4/22/2018 9:35	3.4	20	5.4	2.6	0.1333	2.5
4/22/2018 9:36	3	20.1	5.3	2.6	0.1238	2.8
4/22/2018 9:37	3.4	20	5.3	2.6	0.1219	3.1
4/22/2018 9:38	3	19.9	5.3	2.5	0.128	3.2
4/22/2018 9:39	4	20	5.3	2.6	0.1204	3
4/22/2018 9:40	3.6	20	5.3	2.6	0.1301	3
4/22/2018 9:41	3.7	20	5.3	2.6	0.1205	3.2
4/22/2018 9:42	3.1	19.9	5.3	2.6	0.133	3.4
4/22/2018 9:43	2.9	19.8	5.2	2.6	0.1158	3.6
4/22/2018 9:44	3.4	19.9	5.2	2.6	0.1203	3.7
4/22/2018 9:45	4	20.1	5.2	2.6	0.1195	3.8
4/22/2018 9:46	3.8	19.6	5.2	2.6	0.1223	3.5
4/22/2018 9:47	2.9	19.8	5.2	2.6	0.1292	2.2
4/22/2018 9:48	4.1	19.9	5.2	2.6	0.1093	2.3
4/22/2018 9:49	2.9	20.1	5.2	2.6	0.1275	2.6
4/22/2018 9:50	2.5	20	5.2	2.6	0.117	2.8
4/22/2018 9:51	4.4	20.1	5.2	2.6	0.1232	2.7
4/22/2018 9:52	3.6	20.2	5.2	2.6	0.1247	2.7
4/22/2018 9:53	3.4	19.9	5.2	2.6	0.1123	2.7
4/22/2018 9:54	3.4	20	5.1	2.6	0.1261	2.8
4/22/2018 9:55	3.1	20.1	5.1	2.6	0.1097	3
4/22/2018 9:56	3.7	20.2	5.1	2.6	0.121	3.1
4/22/2018 9:57	3.3	20	5.1	2.6	0.121	3.2
4/22/2018 9:58	3.4	20.1	5.1	2.6	0.1269	3.3
4/22/2018 9:59	4.4	20.1	5	2.6	0.1183	3.3
4/22/2018 10:00	3.8	20.3	5	2.6	0.1192	3.1
4/22/2018 10:01	3	20.2	5	2.6	0.1267	2.9
4/22/2018 10:02	3.9	20.1	5	2.6	0.1145	3
4/22/2018 10:03	3.9	20.3	5	2.6	0.1243	2.9
4/22/2018 10:04	3.9	20	5	2.6	0.1158	3
4/22/2018 10:05	2.3	20	5	2.6	0.1176	3
4/22/2018 10:06	2.3	19.9	5	2.6	0.1287	3.2
4/22/2018 10:07	4.2	20.4	5	2.6	0.1148	3.2
4/22/2018 10:08	2.7	20	4.9	2.6	0.1334	3.3
4/22/2018 10:09	3.3	19.6	4.9	2.6	0.1187	3.3
4/22/2018 10:10	3.7	20	4.9	2.6	0.1261	3.2
4/22/2018 10:11	3.8	19.6	4.9	2.6	0.1201	3.2
4/22/2018 10:12	3.8	20.3	4.9	2.6	0.113	3.2
4/22/2018 10:13	2.8	19.9	4.9	2.6	0.1306	3.3
4/22/2018 10:14	4.7	20.3	4.8	2.6	0.1115	3.5
4/22/2018 10:15	3.1	20.2	4.8	2.6	0.122	3.7
4/22/2018 10:16	2.6	20.1	4.7	2.6	0.1217	3.8

4/22/2018 10:17	4.2	20	4.8	2.6	0.1246	2.9
4/22/2018 10:18	3.5	20	4.7	2.5	0.1328	2.4
4/22/2018 10:19	2.6	20.3	4.7	2.5	0.1138	2.7
4/22/2018 10:20	3.8	20.1	4.7	2.5	0.131	2.7
4/22/2018 10:21	4.4	20.2	4.7	2.5	0.1189	2.9
4/22/2018 10:22	2.6	19.8	4.7	2.5	0.1242	2.8
4/22/2018 10:23	2.2	19.9	4.7	2.6	0.1198	2.6
4/22/2018 10:24	3	20.4	4.7	2.6	0.1196	2.5
4/22/2018 10:25	4.7	20.2	4.6	2.6	0.1223	2.5
4/22/2018 10:26	3.8	20.3	4.6	2.6	0.1088	2.6
4/22/2018 10:27	4.2	19.7	4.6	2.6	0.131	2.8
4/22/2018 10:28	3	20.1	4.6	2.6	0.1148	2.4
4/22/2018 10:29	4	20.1	4.5	2.6	0.1229	2.1
4/22/2018 10:30	3.9	20.2	4.5	2.6	0.1274	2.4
4/22/2018 10:31	4.2	19.9	4.5	2.6	0.1185	2.5
4/22/2018 10:32	4.7	20.4	4.5	2.6	0.1257	2.4
4/22/2018 10:33	3.9	20.2	4.4	2.6	0.1125	2.3
4/22/2018 10:34	3.8	20.2	4.4	2.6	0.1256	2.4
4/22/2018 10:35	2.7	20.1	4.4	2.6	0.1235	2.3
4/22/2018 10:36	4.2	20.4	4.4	2.5	0.1211	2.3
4/22/2018 10:37	4.1	20.7	4.4	2.5	0.1303	2.3
4/22/2018 10:38	3.8	20.4	4.4	2.5	0.11	2.6
4/22/2018 10:39	3	20.4	4.3	2.6	0.1292	2.8
4/22/2018 10:40	3.1	20.5	4.3	2.5	0.1209	2.8
4/22/2018 10:41	3.1	20.4	4.3	2.5	0.1221	3
4/22/2018 10:42	3	20.3	4.3	2.5	0.125	2.9
4/22/2018 10:43	4.5	20.1	4.3	2.6	0.1156	3.1
4/22/2018 10:44	3.3	20.3	4.3	2.6	0.1337	3.1
4/22/2018 10:45	3	20	4.3	2.6	0.1172	3.2
4/22/2018 10:46	3.8	20.3	4.3	2.6	0.1204	3.2
4/22/2018 10:47	2.8	20.4	4.2	2.6	0.1197	3.3
4/22/2018 10:48	2.8	20.3	4.2	2.6	0.121	2.9
4/22/2018 10:49	4.2	20.6	4.2	2.6	0.1246	2.9
4/22/2018 10:50	4.7	20	4.3	2.6	0.1148	3.1
4/22/2018 10:51	4	20.2	4.2	2.5	0.1285	3.3
4/22/2018 10:52	3.1	20.7	4.2	2.5	0.1087	3.4
4/22/2018 10:53	4.5	20.6	4.2	2.5	0.126	3.4
4/22/2018 10:54		20.4	4.2	2.5	0.1128	3.1
4/22/2018 10:55		20.3	4.1	2.5	0.1208	2.1
4/22/2018 10:56	3.7	20.8	4.1	2.5	0.1316	2.2
4/22/2018 10:57	3.4	20.4	4.1	2.5	0.1116	2.5
4/22/2018 10:58	5.7	20.8	4.2	2.5	0.1239	2.7
4/22/2018 10:59	3.6	20.3	4.1	2.5	0.1162	2.7
4/22/2018 11:00	3.3	20.2	4.1	2.5	0.1248	2.9
4/22/2018 11:01		20.6	4.1	2.5	0.1204	3.1
4/22/2018 11:02	6.2	20.9	4.1	2.5	0.1206	3.1
4/22/2018 11:03		20.5	4.1	2.5	0.1243	3.3

4/22/2018 11:04	3.1	20.4	4.1	2.5	0.1121	3.3
4/22/2018 11:05		20.5	4.1	2.5	0.1261	3.3
4/22/2018 11:06	3.6	20.7	4.1	2.5	0.1129	3.2
4/22/2018 11:07		20.6	4	2.5	0.1241	3.4
4/22/2018 11:08	2.7	20.5	4	2.5	0.111	3.4
4/22/2018 11:09		20.9	4	2.5	0.1195	3.3
4/22/2018 11:10		21	4	2.5	0.1223	3.2
4/22/2018 11:11		20.7	4	2.5	0.1127	3.2
4/22/2018 11:12	3.3	20.8	4	2.5	0.1248	2.9
4/22/2018 11:13	3.8	21	3.9	2.5	0.1128	2.9
4/22/2018 11:14	3.5	20.9	3.9	2.5	0.1256	2.9
4/22/2018 11:15	2.2	20.5	3.9	2.5	0.112	3
4/22/2018 11:16	3.5	20.8	3.9	2.5	0.1211	3.1
4/22/2018 11:17	2.8	20.5	3.9	2.5	0.1213	3.1
4/22/2018 11:18	4.4	20.7	3.9	2.5	0.1177	3.1
4/22/2018 11:19	3.9	21	3.9	2.5	0.1206	3
4/22/2018 11:20	3	20.9	3.9	2.5	0.1112	2.7
4/22/2018 11:21	3	20.3	3.9	2.5	0.1337	2.6
4/22/2018 11:22	3.9	20.7	3.8	2.5	0.1137	2.6
4/22/2018 11:23	5.2	21	3.8	2.5	0.1281	2.6
4/22/2018 11:24	2.8	21	3.8	2.5	0.1168	2.7
4/22/2018 11:25	3.8	20.9	3.8	2.5	0.1166	2.7
4/22/2018 11:26	3	20.9	3.8	2.5	0.1235	2.6
4/22/2018 11:27	5.6	21.1	3.7	2.5	0.1154	2.8
4/22/2018 11:28	4.4	21.7	3.7	2.5	0.1319	2.9
4/22/2018 11:29	3.9	21.1	3.7	2.5	0.1152	2.9
4/22/2018 11:30	4.2	21.4	3.7	2.4	0.1266	3
4/22/2018 11:31	4	21.3	3.7	2.4	0.1172	3
4/22/2018 11:32	5.2	21.1	3.7	2.4	0.1168	2.9
4/22/2018 11:33	5.3	20.8	3.6	2.4	0.1209	3
4/22/2018 11:34	4.6	21.1	3.6	2.4	0.1164	2.8
4/22/2018 11:35	4.7	21.2	3.6	2.4	0.1277	2.8
4/22/2018 11:36	4.8	21.1	3.6	2.4	0.1156	2.8
4/22/2018 11:37	4.7	21	3.6	2.5	0.1253	2.8
4/22/2018 11:38	3.9	20.7	3.7	2.5	0.1184	2.9
4/22/2018 11:39	3.4	21.2	3.6	2.5	0.1162	2.9
4/22/2018 11:40	4.4	21.3	3.6	2.5	0.1272	2.8
4/22/2018 11:41	4	21	3.6	2.5	0.1111	3
4/22/2018 11:42	3.5	21	3.6	2.5	0.1288	2.9
4/22/2018 11:43	3	21.5	3.5	2.5	0.1108	2.6
4/22/2018 11:44	3.5	21.2	3.5	2.5	0.1272	2.3
4/22/2018 11:45	3.5	21.3	3.5	2.5	0.1187	2.3
4/22/2018 11:46	3.7	21.4	3.5	2.5	0.1223	2.4
4/22/2018 11:47	3.9	21.9	3.5	2.4	0.1228	2.4
4/22/2018 11:48	4.7	22.1	3.5	2.4	0.1073	2.4
4/22/2018 11:49	3.5	21.7	3.5	2.4	0.1301	2.6
4/22/2018 11:50	4.5	21.5	3.5	2.3	0.1098	2.6

4/22/2018 11:51	4.8	21.3	3.5	2.3	0.1284	2.7
4/22/2018 11:52	3.9	21.6	3.5	2.3	0.1239	2.8
4/22/2018 11:53	4	21.4	3.5	2.3	0.1207	2.9
4/22/2018 11:54	5.4	21.4	3.5	2.3	0.1252	3
4/22/2018 11:55	4.9	21.3	3.5	2.3	0.1113	3.2
4/22/2018 11:56	3.4	21.2	3.4	2.3	0.1275	3.2
4/22/2018 11:57	2.5	21	3.5	2.4	0.1178	3.2
4/22/2018 11:58	5	21.1	3.5	2.4	0.1276	3.2
4/22/2018 11:59	4.4	21.5	3.4	2.4	0.1234	3.3
4/22/2018 12:00	4.2	21.6	3.4	2.4	0.1237	3.3
4/22/2018 12:01	2.9	21.2	3.4	2.4	0.1253	3.4
4/22/2018 12:02	3.4	21.4	3.4	2.4	0.107	3.3
4/22/2018 12:03	3.9	21.5	3.4	2.5	0.1253	3.5
4/22/2018 12:04	4.8	21.2	3.3	2.4	0.1175	3.6
4/22/2018 12:05	4.5	21.4	3.3	2.4	0.1217	3.5
4/22/2018 12:06	5.1	21.2	3.3	2.4	0.1176	3.5
4/22/2018 12:07	6.3	21.6	3.3	2.4	0.1117	3.5
4/22/2018 12:08	5.2	21.7	3.3	2.4	0.1228	3.5
4/22/2018 12:09	3.9	21.8	3.3	2.4	0.1055	3.6
4/22/2018 12:10	5.1	21.7	3.2	2.4	0.1329	3.6
4/22/2018 12:11	3.9	21.5	3.2	2.4	0.1081	3.6
4/22/2018 12:12	5.1	21.4	3.2	2.4	0.1299	3.5
4/22/2018 12:13	2.6	21.4	3.2	2.4	0.1201	3.3
4/22/2018 12:14	3.8	21	3.2	2.4	0.1147	3.4
4/22/2018 12:15	4.7	21.8	3.2	2.4	0.1317	3.3
4/22/2018 12:16	3.5	21.4	3.2	2.4	0.1096	3.3
4/22/2018 12:17	3.5	21.3	3.2	2.4	0.1268	3.3
4/22/2018 12:18	3.4	21.6	3.2	2.4	0.1163	3.1
4/22/2018 12:19	4.7	21.9	3.1	2.4	0.1256	2.9
4/22/2018 12:20	4.4	21.7	3.1	2.4	0.1143	2.8
4/22/2018 12:21	3.5	21.2	3.1	2.4	0.1239	2.7
4/22/2018 12:22	3.3	21.5	3.1	2.4	0.1232	2.6
4/22/2018 12:23	4.1	21.6	3.1	2.4	0.1141	2.5
4/22/2018 12:24	5.7	21.6	3.1	2.4	0.1248	2.4
4/22/2018 12:25	3.6	21.7	3.1	2.4	0.1032	2.5
4/22/2018 12:26	5.8	21.7	3	2.4	0.1235	2.6
4/22/2018 12:27	4.1	21.7	3	2.4	0.1137	2.6
4/22/2018 12:28	5.2	21.7	3	2.4	0.1183	2.6
4/22/2018 12:29	4.5	21.6	3	2.4	0.1262	2.5
4/22/2018 12:30	3.5	21.7	3	2.4	0.1169	2.5
4/22/2018 12:31	3.4	21.7	3	2.4	0.1306	2.6
4/22/2018 12:32	3.7	21.3	3	2.4	0.1057	2.6
4/22/2018 12:33	3.6	21.2	3	2.4	0.1208	2.7
4/22/2018 12:34	3	21.6	2.9	2.4	0.1198	2.6
4/22/2018 12:35	3.8	21.7	2.9	2.4	0.1233	2.5
4/22/2018 12:36	4.5	21.3	2.9	2.4	0.1263	2.5
4/22/2018 12:37	5.8	21.1	3	2.4	0.1183	2.4

4/22/2018 12:38	4.9	21.6	3	2.4	0.1249	2.3
4/22/2018 12:39	5.6	21.8	2.9	2.4	0.1111	2.3
4/22/2018 12:40	4.5	21.7	2.9	2.4	0.1182	2.4
4/22/2018 12:41	3.8	21.6	2.9	2.4	0.1186	2.5
4/22/2018 12:42	4.2	21.7	2.9	2.4	0.1126	2.4
4/22/2018 12:43	3.6	21.5	2.9	2.4	0.1215	2.4
4/22/2018 12:44	4.4	21.3	2.9	2.4	0.1123	2.4
4/22/2018 12:45	5.2	21.6	2.8	2.4	0.125	2.4
4/22/2018 12:46	4.1	22	2.8	2.4	0.1087	2.4
4/22/2018 12:47	4.6	21.9	2.8	2.4	0.1264	2.3
4/22/2018 12:48	5.2	22.2	2.8	2.4	0.1195	2.3
4/22/2018 12:49	5.4	22.1	2.8	2.4	0.114	2.4
4/22/2018 12:50	5.4	22.1	2.8	2.3	0.1236	2.4
4/22/2018 12:51	5.3	22.2	2.8	2.3	0.1082	2.4
4/22/2018 12:52	3.6	21.6	2.8	2.3	0.1226	2.4
4/22/2018 12:53	4.1	21.8	2.8	2.3	0.1144	2.5
4/22/2018 12:54	4.4	21.4	2.8	2.3	0.1167	2.4
4/22/2018 12:55	4.9	22.1	2.8	2.3	0.1177	2.5
4/22/2018 12:56	2.9	21.4	2.8	2.3	0.1178	2.4
4/22/2018 12:57	4	21.5	2.8	2.4	0.1222	2.4
4/22/2018 12:58	4.1	21.7	2.8	2.4	0.1103	2.3
4/22/2018 12:59	4.6	22.1	2.8	2.4	0.1304	2.4
4/22/2018 13:00	4.2	22.1	2.8	2.4	0.1146	2.4
4/22/2018 13:01	3.3	21.7	2.8	2.4	0.1159	2.5
4/22/2018 13:02	4.2	21.6	2.7	2.4	0.114	2.7
4/22/2018 13:03	4.5	21.6	2.7	2.4	0.114	2.7
4/22/2018 13:04	4.3	21.2	2.8	2.4	0.1262	2.7
4/22/2018 13:05	4.7	21.4	2.7	2.4	0.107	2.8
4/22/2018 13:06	4.4	21.6	2.7	2.4	0.1206	2.9
4/22/2018 13:07	5.4	22	2.7	2.4	0.1067	3
4/22/2018 13:08	4.5	21.9	2.7	2.4	0.1239	2.8
4/22/2018 13:09	3.8	21.7	2.7	2.4	0.1116	2.9
4/22/2018 13:10	4.7	21.8	2.7	2.4	0.1149	3.1
4/22/2018 13:11	3.1	21.6	2.7	2.4	0.126	3.2
4/22/2018 13:12	4.4	21.6	2.6	2.3	0.1042	3.1
4/22/2018 13:13	3.9	21.7	2.6	2.3	0.1238	3.1
4/22/2018 13:14	4.7	22	2.6	2.3	0.1167	3.1
4/22/2018 13:15	5.1	21.9	2.6	2.3	0.1247	3.1
4/22/2018 13:16	3.9	22	2.6	2.3	0.1068	3
4/22/2018 13:17	3.8	21.5	2.6	2.3	0.119	3
4/22/2018 13:18	3.3	21.6	2.6	2.4	0.1228	2.9
4/22/2018 13:19	4.9	21.3	2.6	2.4	0.116	2.2
4/22/2018 13:20	4.1	22	2.6	2.4	0.1184	2.4
4/22/2018 13:21	5.2	21.5	2.6	2.4	0.1084	2.6
4/22/2018 13:22	5.4	22	2.6	2.4	0.119	2.9
4/22/2018 13:23	3.8	21.8	2.6	2.4	0.1081	3.1
4/22/2018 13:24	4.5	21.7	2.6	2.4	0.1176	3.1

4/22/2018 13:25	4	21.4	2.6	2.4	0.1155	3.2
4/22/2018 13:26	3.4	21.6	2.5	2.4	0.1114	3.2
4/22/2018 13:27	3.1	21.9	2.6	2.4	0.1182	3.2
4/22/2018 13:28	4	21.9	2.5	2.4	0.1078	3.4
4/22/2018 13:29	4.2	21.6	2.5	2.4	0.1201	3.5
4/22/2018 13:30	5.1	21.9	2.5	2.4	0.1087	3.6
4/22/2018 13:31	4.5	22	2.5	2.4	0.1182	3.6
4/22/2018 13:32	4.9	22.1	2.5	2.4	0.1147	3.7
4/22/2018 13:33	4.4	21.8	2.5	2.3	0.1229	3.8
4/22/2018 13:34	4.2	22	2.5	2.3	0.1197	3.8
4/22/2018 13:35	5.1	21.8	2.5	2.3	0.1164	3.9
4/22/2018 13:36	3.9	22.7	2.5	2.3	0.1191	3.8
4/22/2018 13:37	4.4	22.4	2.5	2.3	0.1105	3.8
4/22/2018 13:38	4.9	21.7	2.5	2.3	0.1223	3.8
4/22/2018 13:39	4.1	21.8	2.5	2.3	0.1201	3.7
4/22/2018 13:40	5.5	22.6	2.5	2.4	0.1153	3.7
4/22/2018 13:41	5.8	22.2	2.5	2.3	0.1209	3.8
4/22/2018 13:42	3.3	21.7	2.5	2.3	0.1133	3.8
4/22/2018 13:43	4	22	2.5	2.3	0.1251	3.6
4/22/2018 13:44	5.1	22.1	2.5	2.3	0.1213	3.5
4/22/2018 13:45	4.3	22.1	2.4	2.4	0.1224	3.2
4/22/2018 13:46	4.7	21.8	2.4	2.4	0.1131	3
4/22/2018 13:47	4.3	21.9	2.4	2.3	0.1155	2.7
4/22/2018 13:48	5.2	22.4	2.4	2.3	0.1053	2.9
4/22/2018 13:49	5	22.3	2.4	2.3	0.1136	2.9
4/22/2018 13:50	4.8	23	2.4	2.3	0.1243	2.9
4/22/2018 13:51	4.3	22.6	2.4	2.3	0.1095	2.7
4/22/2018 13:52	5.5	22.3	2.4	2.3	0.1306	2.4
4/22/2018 13:53	5.1	22.2	2.4	2.3	0.1085	2.2
4/22/2018 13:54	4.8	22.7	2.4	2.2	0.1223	2.4
4/22/2018 13:55	4.9	22.9	2.4	2.2	0.1191	2.5
4/22/2018 13:56	4.9	22.4	2.4	2.2	0.1146	2.7
4/22/2018 13:57	4.5	22.2	2.4	2.2	0.1237	2.2
4/22/2018 13:58	5.4	22.2	2.4	2.2	0.1153	1.6
4/22/2018 13:59	3.5	22.3	2.4	2.3	0.1245	1.5
4/22/2018 14:00	5.2	21.8	2.4	2.3	0.1145	1.6
4/22/2018 14:01	4.3	21.8	2.4	2.3	0.1233	1.9
4/22/2018 14:02	4.2	22	2.4	2.3	0.1105	2
4/22/2018 14:03	4.2	22.3	2.4	2.3	0.1138	1.8
4/22/2018 14:04	4	22.5	2.4	2.3	0.1206	1.8
4/22/2018 14:05	3	22.3	2.4	2.3	0.1143	1.9
4/22/2018 14:06	6.2	22.2	2.4	2.3	0.1243	2.1
4/22/2018 14:07	3.5	22.2	2.4	2.3	0.1085	2.3
4/22/2018 14:08	4.6	22	2.4	2.3	0.1223	2.2
4/22/2018 14:09	4	21.8	2.3	2.3	0.1156	2.3
4/22/2018 14:10	4.8	22.2	2.4	2.3	0.1207	2.3
4/22/2018 14:11	5.5	22.5	2.4	2.3	0.1177	2.3

4/22/2018 14:12	5.7	22.2	2.3	2.3	0.1071	2.2
4/22/2018 14:13	4.8	21.9	2.3	2.3	0.1202	2.1
4/22/2018 14:14	4.1	22.4	2.3	2.3	0.1047	2.2
4/22/2018 14:15		22	2.3	2.3	0.1268	2.3
4/22/2018 14:16		22.6	2.3	2.3	0.1137	2.3
4/22/2018 14:17	4.6	22.2	2.3	2.3	0.1246	2.4
4/22/2018 14:18		22.3	2.3	2.3	0.1215	2.4
4/22/2018 14:19		22.3	2.3	2.3	0.1155	2.5
4/22/2018 14:20	4.2	22.4	2.3	2.3	0.1115	2.4
4/22/2018 14:21		22.4	2.3	2.3	0.1084	2.5
4/22/2018 14:22	8.1	22.1	2.3	2.3	0.1239	2.7
4/22/2018 14:23		22.2	2.3	2.3	0.1156	2.8
4/22/2018 14:24	5	21.7	2.3	2.3	0.1226	2.6
4/22/2018 14:25	3.5	21.7	2.3	2.3	0.1127	2.8
4/22/2018 14:26	4.8	22	2.3	2.3	0.1152	2.9
4/22/2018 14:27		22	2.3	2.3	0.1228	2.9
4/22/2018 14:28		22.3	2.3	2.3	0.1022	2.9
4/22/2018 14:29		22	2.3	2.3	0.1169	2.9
4/22/2018 14:30		22.2	2.3	2.3	0.1079	3
4/22/2018 14:31	5.5	22.5	2.3	2.3	0.1153	2.8
4/22/2018 14:32	6.4	22.1	2.3	2.3	0.109	2.8
4/22/2018 14:33	5.7	22.2	2.3	2.3	0.1076	2.9
4/22/2018 14:34	3.7	22	2.2	2.3	0.1133	2.7
4/22/2018 14:35	5.1	22.6	2.3	2.2	0.1026	2.8
4/22/2018 14:36	4.5	21.9	2.2	2.2	0.119	2.8
4/22/2018 14:37	4.8	22.4	2.2	2.2	0.1005	2.7
4/22/2018 14:38	6.2	21.9	2.2	2.2	0.1139	2.6
4/22/2018 14:39	4.9	22.4	2.3	2.2	0.1196	2.7
4/22/2018 14:40	5.4	22.2	2.2	2.2	0.1	2.7
4/22/2018 14:41	5.2	22.1	2.2	2.2	0.1282	2.4
4/22/2018 14:42	4.6	22	2.2	2.2	0.1103	2.3
4/22/2018 14:43	5.5	22.6	2.2	2.2	0.1115	2.4
4/22/2018 14:44	4.3	22.4	2.2	2.2	0.1048	2.5
4/22/2018 14:45	3.4	22	2.2	2.2	0.1118	2.5
4/22/2018 14:46	5.5	22.2	2.2	2.2	0.1103	2.5
4/22/2018 14:47	6.1	22.6	2.2	2.2	0.1099	2.5
4/22/2018 14:48	3	22.5	2.2	2.2	0.1121	2.5
4/22/2018 14:49	5.3	21.5	2.2	2.2	0.0976	2.5
4/22/2018 14:50	2.7	21.9	2.2	2.2	0.1218	2.5
4/22/2018 14:51	4	21.8	2.2	2.2	0.1008	2.6
4/22/2018 14:52	4.9	22.3	2.2	2.2	0.1189	2.6
4/22/2018 14:53	4.7	22.3	2.2	2.3	0.1054	2.7
4/22/2018 14:54	4	22.3	2.3	2.3	0.1115	2.6
4/22/2018 14:55	4.3	22.4	2.3	2.3	0.1206	2.5
4/22/2018 14:56	4.8	22	2.3	2.3	0.1055	2.6
4/22/2018 14:57	4.5	22.2	2.3	2.3	0.1201	2.6
4/22/2018 14:58	3.9	22.2	2.3	2.3	0.1054	2.7

4/22/2018 14:59	3.9	22.1	2.3	2.3	0.1143	2.6
4/22/2018 15:00	4.3	22.8	2.3	2.3	0.1102	2.6
4/22/2018 15:01	3.7	22.6	2.3	2.3	0.1142	2.6
4/22/2018 15:02	4.5	22.2	2.3	2.3	0.1088	2.4
4/22/2018 15:03	5	22.1	2.3	2.3	0.1048	2.5
4/22/2018 15:04	4.9	22.4	2.3	2.3	0.1196	2.5
4/22/2018 15:05	4.9	22.5	2.3	2.3	0.0998	2.7
4/22/2018 15:06	4	22.1	2.3	2.3	0.1212	2.7
4/22/2018 15:07	4.5	22.3	2.3	2.2	0.1001	2.7
4/22/2018 15:08	3.6	22.2	2.3	2.2	0.1048	2.7
4/22/2018 15:09	4.4	22.4	2.3	2.2	0.1178	2.7
4/22/2018 15:10	4.4	22.2	2.3	2.2	0.1124	2.8
4/22/2018 15:11	4.8	22.5	2.4	2.2	0.1236	2.8
4/22/2018 15:12	5.1	22.1	2.3	2.2	0.1066	2.8
4/22/2018 15:13	4.3	21.8	2.2	2.2	0.118	2.7
4/22/2018 15:14	4.7	22.1	2.3	2.2	0.107	2.7
4/22/2018 15:15	3.8	21.7	2.3	2.2	0.1154	2.6
4/22/2018 15:16	5.4	22	2.3	2.3	0.1128	2.7
4/22/2018 15:17	5.6	22.4	2.3	2.3	0.1151	2.8
4/22/2018 15:18	4.4	22.2	2.3	2.3	0.1225	2.9
4/22/2018 15:19	4.3	22.1	2.3	2.3	0.1041	2.9
4/22/2018 15:20	4.2	21.9	2.3	2.3	0.1197	2.9
4/22/2018 15:21	4.5	22.1	2.3	2.3	0.1115	2.6
4/22/2018 15:22	3.7	22.2	2.3	2.3	0.1132	2.7
4/22/2018 15:23	4.5	22	2.3	2.3	0.1141	2.8
4/22/2018 15:24	6.1	22	2.3	2.3	0.1171	2.8
4/22/2018 15:25	4.8	22.6	2.3	2.3	0.1197	2.7
4/22/2018 15:26	5	22	2.3	2.3	0.1054	2.2
4/22/2018 15:27	3.6	22.1	2.2	2.3	0.1217	2.1
4/22/2018 15:28	3.8	21.9	2.2	2.3	0.1044	2.3
4/22/2018 15:29	4.2	21.9	2.2	2.2	0.1253	2.3
4/22/2018 15:30	3	22	2.2	2.2	0.1012	2.4
4/22/2018 15:31	5.4	22.1	2.2	2.2	0.1085	2.4
4/22/2018 15:32	5.1	22.3	2.2	2.2	0.1109	2.5
4/22/2018 15:33	4.9	22.7	2.2	2.2	0.1116	2.5
4/22/2018 15:34	5.3	22.7	2.3	2.2	0.1078	2.5
4/22/2018 15:35	4.6	22.1	2.2	2.2	0.0976	2.5
4/22/2018 15:36	4.5	21.7	2.2	2.3	0.1241	2.6
4/22/2018 15:37	4.7	21.8	2.2	2.2	0.098	2.7
4/22/2018 15:38	5	22	2.2	2.2	0.1206	2.6
4/22/2018 15:39	4.8	21.9	2.2	2.3	0.1035	2.5
4/22/2018 15:40	4.7	22.2	2.2	2.3	0.1153	2.5
4/22/2018 15:41	4.6	22.3	2.2	2.3	0.1054	2.4
4/22/2018 15:42	6.2	22.4	2.2	2.3	0.1093	2.2
4/22/2018 15:43	5	22.2	2.2	2.3	0.1049	2.3
4/22/2018 15:44	3.9	21.9	2.2	2.3	0.0988	2.3
4/22/2018 15:45	4.2	22	2.2	2.3	0.1131	2.4

4/22/2018 15:46	3.5	22.4	2.2	2.3	0.1008	2.4
4/22/2018 15:47	3.8	22.1	2.2	2.3	0.1111	2.5
4/22/2018 15:48	4.2	22.3	2.2	2.3	0.1023	2.4
4/22/2018 15:49	5	22.2	2.1	2.2	0.1157	2.4
4/22/2018 15:50	4.3	22	2.2	2.2	0.1123	2.4
4/22/2018 15:51	4.3	22.1	2.2	2.2	0.1064	2.3
4/22/2018 15:52	3.9	22.1	2.2	2.2	0.1172	2.2
4/22/2018 15:53	4.5	22.2	2.2	2.2	0.1039	2.2
4/22/2018 15:54	5	22.2	2.2	2.2	0.1129	2.2
4/22/2018 15:55	5.3	22.4	2.2	2.2	0.1051	2.2
4/22/2018 15:56	4.2	22.5	2.3	2.2	0.1154	2.3
4/22/2018 15:57	4.9	22	2.2	2.2	0.1053	2.4
4/22/2018 15:58	4.5	21.9	2.2	2.2	0.1141	2.4
4/22/2018 15:59	3.7	22	2.2	2.2	0.1161	2.5
4/22/2018 16:00	5.5	22.2	2.2	2.2	0.1051	2.6
4/22/2018 16:01	4.3	22.1	2.2	2.2	0.1163	2.6
4/22/2018 16:02	5.4	21.9	2.2	2.2	0.0993	2.6
4/22/2018 16:03	3.8	21.8	2.2	2.2	0.1177	2.2
4/22/2018 16:04	4.1	21.7	2.2	2.3	0.1012	1.8
4/22/2018 16:05	3.3	22.2	2.3	2.3	0.1182	1.9
4/22/2018 16:06	3.9	22	2.2	2.3	0.1064	2.2
4/22/2018 16:07	3.6	22.1	2.3	2.3	0.1099	2.4
4/22/2018 16:08	5	22.4	2.3	2.3	0.1209	2.4
4/22/2018 16:09	5.6	22	2.3	2.3	0.1073	2.5
4/22/2018 16:10	4.5	22.8	2.3	2.3	0.1138	2.6
4/22/2018 16:11	4.7	22.4	2.2	2.3	0.1037	2.7
4/22/2018 16:12	3.6	22.2	2.2	2.2	0.1145	2.5
4/22/2018 16:13	3.9	22.6	2.2	2.2	0.1003	2.2
4/22/2018 16:14	3.9	22.5	2.2	2.2	0.1118	2.3
4/22/2018 16:15	5.2	22.6	2.2	2.2	0.1128	2.3
4/22/2018 16:16	5	22.6	2.2	2.2	0.1039	2.4
4/22/2018 16:17	4.8	21.9	2.2	2.2	0.112	2.4
4/22/2018 16:18	5.1	22.8	2.3	2.2	0.1045	2.4
4/22/2018 16:19	5	22.9	2.2	2.2	0.1176	2.4
4/22/2018 16:20	4.8	23.2	2.3	2.2	0.1057	2.4
4/22/2018 16:21	4.1	23	2.2	2.1	0.1151	2.4
4/22/2018 16:22	5.8	22.7	2.2	2.1	0.1061	2.4
4/22/2018 16:23	5	22.9	2.2	2.1	0.1118	2.5
4/22/2018 16:24	4.1	22.6	2.1	2.1	0.1131	2.4
4/22/2018 16:25	4.1	22.8	2.2	2.1	0.1125	2.4
4/22/2018 16:26	5.1	22.6	2.2	2.1	0.1113	2.5
4/22/2018 16:27	5.7	22.4	2.2	2.1	0.1087	2.5
4/22/2018 16:28	6	22.3	2.2	2.1	0.1202	2.4
4/22/2018 16:29	4.4	22.6	2.2	2.1	0.1028	2.5
4/22/2018 16:30	4.1	22.2	2.2	2.1	0.117	2.4
4/22/2018 16:31	4.4	21.7	2.2	2.1	0.1027	2.5
4/22/2018 16:32	4.5	22.3	2.2	2.1	0.1155	2.5

4/22/2018 16:33	5.2	22.2	2.2	2.1	0.1136	2.5
4/22/2018 16:34	5	22.2	2.2	2.2	0.1059	2.6
4/22/2018 16:35	5.6	22.5	2.2	2.2	0.1163	2.6
4/22/2018 16:36	5.1	22.8	2.3	2.2	0.1106	2.7
4/22/2018 16:37	3.2	22.1	2.2	2.2	0.1146	2.7
4/22/2018 16:38	5.4	22.5	2.2	2.2	0.1148	2.7
4/22/2018 16:39	4.3	22.6	2.2	2.2	0.1082	2.7
4/22/2018 16:40	3.8	22.6	2.2	2.2	0.1142	2.8
4/22/2018 16:41	4.1	23.2	2.2	2.2	0.1019	2.8
4/22/2018 16:42	3.4	22.5	2.2	2.1	0.117	2.8
4/22/2018 16:43	3.4	22.2	2.2	2.1	0.102	2.8
4/22/2018 16:44	5.6	22.5	2.2	2.2	0.1151	2.8
4/22/2018 16:45	4.2	22.3	2.2	2.2	0.1085	2.7
4/22/2018 16:46	5	22.7	2.2	2.1	0.1127	2.6
4/22/2018 16:47	4.4	23	2.2	2.1	0.1076	2.6
4/22/2018 16:48	4.7	22.4	2.1	2.1	0.1118	2.6
4/22/2018 16:49	4.7	22.8	2.2	2.1	0.1124	2.8
4/22/2018 16:50	6.3	22.7	2.1	2.1	0.0963	3
4/22/2018 16:51	5.5	22.6	2.2	2.1	0.111	3
4/22/2018 16:52	4.6	22.3	2.2	2.1	0.1033	3
4/22/2018 16:53	4.4	22.5	2.2	2.1	0.1121	2.8
4/22/2018 16:54	4.8	22.8	2.1	2.1	0.1064	2.9
4/22/2018 16:55	3.5	22.3	2.1	2.1	0.1029	2.9
4/22/2018 16:56	4.4	22.4	2.2	2.2	0.1182	2.8
4/22/2018 16:57	5.7	22.7	2.2	2.2	0.1093	2.9
4/22/2018 16:58	3.4	21.9	2.2	2.2	0.1154	3
4/22/2018 16:59	5.4	22.1	2.2	2.2	0.1038	3.1
4/22/2018 17:00	5.6	22.5	2.3	2.2	0.1162	3
4/22/2018 17:01	4.9	22.6	2.3	2.2	0.1134	3
4/22/2018 17:02	4.9	22.6	2.3	2.2	0.1098	3.1
4/22/2018 17:03	5.9	22.6	2.3	2.2	0.1204	3
4/22/2018 17:04	4.8	23	2.3	2.2	0.0989	2.9
4/22/2018 17:05	5	23.1	2.3	2.2	0.1133	2.8
4/22/2018 17:06	5.5	22.3	2.3	2.2	0.1031	2.7
4/22/2018 17:07	5.7	22.3	2.3	2.2	0.1177	2.7
4/22/2018 17:08	4.4	22.7	2.3	2.2	0.1074	2.8
4/22/2018 17:09		22.8	2.3	2.1	0.1159	2.8
4/22/2018 17:10	5.5	22.5	2.3	2.1	0.1095	2.8
4/22/2018 17:11		22.6	2.3	2.2	0.1144	2.8
4/22/2018 17:12	3.8	22.5	2.3	2.2	0.1174	2.8
4/22/2018 17:13		22.6	2.3	2.2	0.1124	2.8
4/22/2018 17:14	4.6	22.6	2.3	2.2	0.1194	2.9
4/22/2018 17:15	4.9	22.7	2.3	2.2	0.1063	2.9
4/22/2018 17:16	3.2	22.7	2.3	2.2	0.1224	2.9
4/22/2018 17:17		22.3	2.3	2.2	0.1062	2.9
4/22/2018 17:18		22.5	2.3	2.2	0.1138	2.9
4/22/2018 17:19		22.7	2.3	2.2	0.1092	2.8

4/22/2018 17:20	4.8	22.6	2.3	2.2	0.1091	2.9
4/22/2018 17:21	5.3	22.5	2.3	2.2	0.1221	2.7
4/22/2018 17:22	4.3	22.7	2.3	2.2	0.1103	2.6
4/22/2018 17:23	8.5	22.8	2.3	2.2	0.1215	2.7
4/22/2018 17:24	5.2	22.6	2.3	2.2	0.1047	2.9
4/22/2018 17:25	5.4	22.8	2.4	2.1	0.1174	3
4/22/2018 17:26		23	2.4	2.1	0.1075	2.8
4/22/2018 17:27	5	22.9	2.4	2.1	0.12	2.7
4/22/2018 17:28	8.2	23.1	2.4	2.1	0.1087	3
4/22/2018 17:29	4.3	22.9	2.4	2.1	0.1142	3.1
4/22/2018 17:30	5.1	23.2	2.3	2.1	0.1102	3.2
4/22/2018 17:31	5.9	24.8	2.6	2.1	0.1112	3.1
4/22/2018 17:32	4.4	22.9	2.5	2	0.1145	3.2
4/22/2018 17:33	4.4	23.1	2.4	2	0.1022	3.3
4/22/2018 17:34	4.8	22.6	2.3	2	0.1194	3.3
4/22/2018 17:35	4.7	22.6	2.3	2	0.1035	3.2
4/22/2018 17:36	5.5	24	2.4	2	0.1181	3.1
4/22/2018 17:37	5.5	24.2	2.6	2	0.1095	3.1
4/22/2018 17:38	4.5	23.7	2.4	2	0.1166	3.3
4/22/2018 17:39	5.1	24.2	2.5	2	0.115	3.3
4/22/2018 17:40	4	23.6	2.5	2	0.1107	3.3
4/22/2018 17:41	4.8	22.5	2.3	2	0.1246	3.3
4/22/2018 17:42	5.7	22.4	2.3	2	0.1069	3.3
4/22/2018 17:43	5.1	23	2.3	2	0.1196	3.3
4/22/2018 17:44	6.3	23.3	2.4	2	0.1113	3.2
4/22/2018 17:45	5.4	24.3	2.5	2.1	0.1122	3.4
4/22/2018 17:46	5.4	22.7	2.4	2.1	0.1176	3.4
4/22/2018 17:47	5.7	23.2	2.3	2.1	0.1082	3.5
4/22/2018 17:48	5.4	23.5	2.5	2.1	0.1207	3.5
4/22/2018 17:49	3.2	22.6	2.3	2.1	0.0991	3.6
4/22/2018 17:50	4	22.6	2.3	2.1	0.1162	3.6
4/22/2018 17:51	4.5	23.7	2.4	2.1	0.1076	3.7
4/22/2018 17:52	4.6	22.9	2.4	2.1	0.1158	3.5
4/22/2018 17:53	5.2	22.6	2.4	2.1	0.1106	3.4
4/22/2018 17:54	4.8	22.2	2.3	2.1	0.1147	3.4
4/22/2018 17:55	4.7	22.5	2.3	2.1	0.1212	3.6
4/22/2018 17:56	5.5	22.3	2.3	2.1	0.105	3.8
4/22/2018 17:57	5.6		2.4	2.1	0.1199	3.7
4/22/2018 17:58	4.8	23	2.4	2.1	0.1065	3.6
4/22/2018 17:59	5.2	23.4	2.5	2.1	0.1173	3.5
4/22/2018 18:00	5.5	23.2	2.5	2.1	0.1149	2.2
4/22/2018 18:01	5.5	23.4	2.5	2.1	0.1049	1.9
4/22/2018 18:02	4.3	23.7	2.5	2.1	0.1184	1.9
4/22/2018 18:03	5.8	23.2	2.5	2.1	0.107	1.9
4/22/2018 18:04	5.3	23.6	2.5	2	0.1211	2
4/22/2018 18:05	6	24.4	2.5	2	0.1016	2
4/22/2018 18:06	5.3	23.7	2.5	2	0.1187	2.1

4/22/2018 18:07	5.6	23	2.5	2	0.1047	2.2
4/22/2018 18:08	4.3	22.5	2.4	2	0.1058	2.2
4/22/2018 18:09	3.5	22.7	2.4	2	0.1092	2.1
4/22/2018 18:10	5.6	22.8	2.4	2	0.1057	1.9
4/22/2018 18:11	3.7	22.7	2.4	2	0.1145	1.8
4/22/2018 18:12	5.4	23.3	2.4	2	0.1086	2
4/22/2018 18:13	5	23.8	2.5	2	0.1147	2
4/22/2018 18:14	4.4	23.1	2.4	2.1	0.1084	2.1
4/22/2018 18:15	5.1	23.6	2.4	2.1	0.1199	2.3
4/22/2018 18:16	5.8	23.9	2.5	2.1	0.1175	2.3
4/22/2018 18:17	4.7	22.7	2.4	2.1	0.1144	2.2
4/22/2018 18:18	4.5	23.1	2.4	2.1	0.1079	2.4
4/22/2018 18:19	4.9	22.6	2.4	2.1	0.0999	2.5
4/22/2018 18:20	5	22.6	2.4	2.1	0.1164	2.6
4/22/2018 18:21	4.9	22.8	2.4	2.1	0.1091	2.7
4/22/2018 18:22	4.7	22.7	2.4	2.2	0.1197	2.7
4/22/2018 18:23	6	22.7	2.4	2.2	0.1017	2.8
4/22/2018 18:24	4.7	23.3	2.5	2.2	0.1163	2.9
4/22/2018 18:25	5.2	23.2	2.5	2.2	0.1093	3.1
4/22/2018 18:26	5.9	23.8	2.5	2.2	0.1086	3.3
4/22/2018 18:27	6.6	23.4	2.5	2.1	0.1179	3.3
4/22/2018 18:28	4.6	23.3	2.5	2.1	0.1088	3.3
4/22/2018 18:29	4.4	23.9	2.5	2.1	0.1097	3.4
4/22/2018 18:30	5.9	23.7	2.5	2.1	0.1072	3.4
4/22/2018 18:31	5.8	24.4	2.5	2	0.1216	3.5
4/22/2018 18:32	6.8	24.5	2.4	2	0.1103	3.5
4/22/2018 18:33	4.3	24	2.4	2	0.114	3.4
4/22/2018 18:34	3.9	23.2	2.4	2	0.1021	3.5
4/22/2018 18:35	3.3	23.1	2.4	2	0.1171	3.4
4/22/2018 18:36	4.7	23.5	2.4	1.9	0.1121	3.5
4/22/2018 18:37	5.3	24.1	2.4	1.9	0.1135	3.4
4/22/2018 18:38	6.5	23.9	2.4	1.9	0.1152	3.4
4/22/2018 18:39	4.9	23.2	2.4	1.9	0.1162	3.4
4/22/2018 18:40	5.3	23.1	2.4	1.9	0.1091	3.4
4/22/2018 18:41	6.2	23.1	2.4	2	0.101	3.3
4/22/2018 18:42	4.4	23.2	2.4	2	0.1198	3.4
4/22/2018 18:43	5.5	22.9	2.4	2	0.0983	3.4
4/22/2018 18:44	4.1	23	2.4	2	0.117	3.5
4/22/2018 18:45	4.4	23.1	2.4	2	0.1056	3.3
4/22/2018 18:46	5.8	23.1	2.4	2	0.1098	3.3
4/22/2018 18:47	5.6	22.9	2.4	2.1	0.1153	3.3
4/22/2018 18:48	5.4	23.2	2.4	2.1	0.1032	2.5
4/22/2018 18:49	5.8	23	2.4	2.1	0.1134	1.9
4/22/2018 18:50	4.8	23	2.4	2.1	0.108	1.9
4/22/2018 18:51	4.4	23.2	2.4	2.1	0.1232	2
4/22/2018 18:52	4.3	23	2.4	2.1	0.1033	2.2
4/22/2018 18:53	4.7	23	2.4	2.1	0.1131	2.4

4/22/2018 18:54	4.1	23.2	2.4	2	0.1022	2.6
4/22/2018 18:55	4.9	23.7	2.4	2	0.1163	2.7
4/22/2018 18:56	4.4	23.8	2.4	2	0.1039	2.9
4/22/2018 18:57	5.6	24	2.4	2	0.1073	2.9
4/22/2018 18:58	6.3	24.4	2.4	2	0.1122	2.9
4/22/2018 18:59	5.6	24.2	2.5	1.9	0.1042	2.9
4/22/2018 19:00	5.8	24.1	2.5	1.9	0.1085	3
4/22/2018 19:01	5.5	24	2.5	1.9	0.1076	3.1
4/22/2018 19:02	5.6	24.1	2.5	1.9	0.114	3.2
4/22/2018 19:03	5.9	24.7	2.5	1.9	0.1063	3.3
4/22/2018 19:04	6.3	24.9	2.5	1.9	0.1078	3.3
4/22/2018 19:05	7.7	26	2.5	1.9	0.1141	3.4
4/22/2018 19:06	4.5	24.1	2.5	1.9	0.1027	3.4
4/22/2018 19:07	5.4	23.6	2.4	1.9	0.1218	3.5
4/22/2018 19:08	5.1	23.8	2.4	1.9	0.103	3.6
4/22/2018 19:09	4.6	23.1	2.4	1.9	0.1145	3.5
4/22/2018 19:10	5.8	23.6	2.4	1.9	0.0962	3.6
4/22/2018 19:11	4	23.7	2.4	1.9	0.1069	3.5
4/22/2018 19:12	4.9	23.8	2.4	1.9	0.1158	3.5
4/22/2018 19:13	5.2	23.3	2.4	1.9	0.1073	3.3
4/22/2018 19:14	6.4	23.2	2.4	2	0.1176	3.1
4/22/2018 19:15	4.8	23.4	2.4	2	0.1111	3.1
4/22/2018 19:16	5.6	23.5	2.4	2	0.1179	2.9
4/22/2018 19:17	3.8	23.5	2.4	2	0.0975	2.6
4/22/2018 19:18	5.9	23.3	2.4	2	0.1069	2.6
4/22/2018 19:19	6.2	23.5	2.4	2	0.1152	2.6
4/22/2018 19:20	5.8	23.2	2.4	2	0.1075	2.8
4/22/2018 19:21	5.5	23.3	2.4	2	0.112	2.8
4/22/2018 19:22	6.1	23.2	2.4	2	0.116	2.8
4/22/2018 19:23	4.5	23.4	2.4	2	0.1121	2.8
4/22/2018 19:24	5.3	23.5	2.4	2	0.1064	2.9
4/22/2018 19:25	4.8	23.3	2.4	2	0.1159	2.9
4/22/2018 19:26	5.5	23.5	2.4	2	0.1067	3
4/22/2018 19:27	6.1	23.4	2.4	2	0.1126	2.9
4/22/2018 19:28	4.7	23.3	2.4	2	0.1025	2.6
4/22/2018 19:29	5.1	23.2	2.5	2	0.1104	2.6
4/22/2018 19:30	5.5	23.3	2.5	2	0.1183	2.7
4/22/2018 19:31	6	23.2	2.5	2	0.0999	2.8
4/22/2018 19:32	6.3	23.3	2.5	2	0.1167	2.8
4/22/2018 19:33	4.8	23.8	2.7	2	0.1076	2.8
4/22/2018 19:34	4	24	2.8	2	0.114	2.8
4/22/2018 19:35	5.7	23.8	2.9	2	0.1038	2.8
4/22/2018 19:36	5.4	24.1	3	2	0.1058	2.8
4/22/2018 19:37	5.3	23.9	3	1.9	0.1184	2.9
4/22/2018 19:38	4.1	23.9	3.1	1.9	0.1	2.9
4/22/2018 19:39	5.7	24.1	3.1	1.9	0.1127	2.9
4/22/2018 19:40	6.2	23.8	3	1.9	0.1	2.6

4/22/2018 19:41	6.5	23.7	3	1.9	0.1209	2.3
4/22/2018 19:42	4.7	23.4	3.1	1.9	0.1034	2.2
4/22/2018 19:43	5.6	23.9	3.1	1.9	0.1161	2.1
4/22/2018 19:44	5.4	24.4	3.7	1.9	0.104	2.3
4/22/2018 19:45	4.7	23.2	3.1	1.9	0.1102	2.3
4/22/2018 19:46	3.4	23.2	3	1.9	0.1152	2.2
4/22/2018 19:47	4.7	23.6	3	2	0.1002	2.1
4/22/2018 19:48	5.6	23.5	3.1	2	0.1152	2
4/22/2018 19:49	5.7	23.2	3	2	0.0972	1.8
4/22/2018 19:50	4.4	23.3	3.1	2	0.1132	1.7
4/22/2018 19:51	5.1	23	3	2	0.1052	1.6
4/22/2018 19:52	4.7	23.1	3	2	0.1126	1.7
4/22/2018 19:53	4	23	3	2	0.0994	1.7
4/22/2018 19:54	5.9	23.2	3.1	2	0.1022	1.7
4/22/2018 19:55	5.3	23.1	3.1	2	0.1152	1.7
4/22/2018 19:56	3.7	23	3.1	2	0.0992	1.7
4/22/2018 19:57	4.5	23.3	3.1	2	0.115	1.7
4/22/2018 19:58	4.5	23.2	3.1	2	0.1007	1.7
4/22/2018 19:59	5.2	23.2	3.1	2	0.1017	1.7
4/22/2018 20:00	6.1	23.5	3.2	2	0.1067	1.7
4/22/2018 20:01	4.3	23.3	3.3	2	0.1014	1.7
4/22/2018 20:02	5.6	23.2	3.3	2	0.1133	1.6
4/22/2018 20:03	5.7	23.3	3.3	2	0.1006	1.5
4/22/2018 20:04	5.6	23.3	3.3	2	0.1113	1.5
4/22/2018 20:05	4.8	23.3	3.3	2	0.0996	1.5
4/22/2018 20:06	6.1	23.8	3.4	2	0.1091	1.5
4/22/2018 20:07	5.2	23.6	3.4	2	0.0999	1.6
4/22/2018 20:08	4.1	24.1	3.5	1.9	0.1048	1.6
4/22/2018 20:09	4.3	23.8	3.5	1.9	0.1078	1.7
4/22/2018 20:10	6	23.8	3.5	1.9	0.1019	1.7
4/22/2018 20:11	4.4	23.8	3.4	1.9	0.1103	1.7
4/22/2018 20:12	5.8	23.7	3.4	1.9	0.0994	1.7
4/22/2018 20:13	4.9	23.6	3.4	1.9	0.1077	1.7
4/22/2018 20:14	4.2	23.9	3.4	1.9	0.1011	1.7
4/22/2018 20:15	5.7	23.7	3.5	1.9	0.0987	1.4
4/22/2018 20:16	6.2	24	3.6	1.9	0.1064	1.5
4/22/2018 20:17	5.7	23.4	3.5	1.9	0.0958	1.5
4/22/2018 20:18	6.1	23.1	3.4	2	0.1062	1.5
4/22/2018 20:19	5.3	23.3	3.4	2	0.0971	1.4
4/22/2018 20:20	4	23.3	3.4	2	0.1102	1.5
4/22/2018 20:21	6	23.1	3.3	2	0.1011	1.5
4/22/2018 20:22	5.7	23.2	3.4	2	0.1088	1.5
4/22/2018 20:23	4.8	23.1	3.4	2	0.1008	1.4
4/22/2018 20:24	4.3	23	3.4	2	0.0963	1.4
4/22/2018 20:25		22.9	3.4	2	0.1142	1.4
4/22/2018 20:26	5.2	23.1	3.4	2	0.0986	1.4
4/22/2018 20:27		23.2	3.4	2	0.1046	1.4

4/22/2018 20:28	4.3	23	3.4	2	0.0985	1.4
4/22/2018 20:29	13.6	22.9	3.4	2	0.1005	1.4
4/22/2018 20:30	5.6	23.3	3.4	2	0.1019	1.4
4/22/2018 20:31	4.6	22.9	3.4	2	0.0974	1.4
4/22/2018 20:32	5.2	22.9	3.3	2	0.1084	1.5
4/22/2018 20:33		23	3.3	2	0.0976	1.5
4/22/2018 20:34		23	3.3	2	0.1097	1.5
4/22/2018 20:35		22.8	3.3	2	0.1031	1.5
4/22/2018 20:36	5.1	22.9	3.4	2	0.1034	1.5
4/22/2018 20:37		23.2	3.3	2	0.1035	1.5
4/22/2018 20:38	4.7	23.7	3.3	2	0.0997	1.5
4/22/2018 20:39	5.2	23.4	3.4	2	0.1133	1.5
4/22/2018 20:40	6.3	23.6	3.4	2	0.0947	1.4
4/22/2018 20:41		23.9	3.4	2	0.1094	1.5
4/22/2018 20:42	5.7	23.7	3.4	2	0.1045	1.5
4/22/2018 20:43		23.3	3.3	1.9	0.1056	1.5
4/22/2018 20:44		23.4	3.3	1.9	0.1055	1.5
4/22/2018 20:45	10.9	23.4	3.3	1.9	0.1011	1.5
4/22/2018 20:46	4.5	23.2	3.2	1.9	0.1189	1.5
4/22/2018 20:47	5.2	23.5	3.2	1.9	0.1047	1.5
4/22/2018 20:48	4.5	23.5	3.2	1.9	0.1142	1.5
4/22/2018 20:49	4.8	22.8	3.2	1.9	0.1074	1.6
4/22/2018 20:50	4.2	22.6	3.2	1.9	0.1064	1.6
4/22/2018 20:51	4	23.2	3.2	2	0.1198	1.5
4/22/2018 20:52	4.5	22.9	3.2	2	0.1012	1.5
4/22/2018 20:53	4.7	22.9	3.2	2	0.1127	1.5
4/22/2018 20:54	4.2	23.2	3.2	2	0.1081	1.5
4/22/2018 20:55	5.8	22.8	3.2	2	0.1077	1.5
4/22/2018 20:56	4.8	22.7	3.2	2	0.1077	1.5
4/22/2018 20:57	3.4	22.9	3.2	2	0.1109	1.6
4/22/2018 20:58	5.5	22.8	3.1	2	0.1127	1.6
4/22/2018 20:59	5.2	22.7	3.1	2	0.1012	1.6
4/22/2018 21:00	4.4	22.7	3.1	2	0.1094	1.6
4/22/2018 21:01	3.7	22.5	3.2	2	0.1078	1.7
4/22/2018 21:02	4.3	22.7	3.2	2	0.1105	1.7
4/22/2018 21:03	5	22.9	3.2	2	0.1129	1.7
4/22/2018 21:04	5.9	23.3	3.2	2	0.1042	1.7
4/22/2018 21:05	4.9	23.2	3.2	2	0.1169	1.8
4/22/2018 21:06	4.5	23.4	3.2	2	0.1023	1.8
4/22/2018 21:07	5.6	23.4	3.1	2	0.1167	1.7
4/22/2018 21:08	5.4	23.2	3.1	2	0.1062	1.8
4/22/2018 21:09	5.3	23	3.1	2	0.1044	1.8
4/22/2018 21:10	5.3	23.2	3.1	2	0.111	1.8
4/22/2018 21:11	5.5	23.3	3.1	2	0.1117	1.8
4/22/2018 21:12	6.2	23.3	3.1	2	0.1103	1.8
4/22/2018 21:13	6.4	23.9	3	1.9	0.1008	1.9
4/22/2018 21:14	5.1	23.7	3	1.9	0.1147	1.9

4/22/2018 21:15	4.5	23	3	1.9	0.107	1.9
4/22/2018 21:16	4.8	22.6	3	1.9	0.1144	1.9
4/22/2018 21:17	5.6	23.2	3	1.9	0.112	2
4/22/2018 21:18	4.5	22.8	3	1.9	0.1078	2
4/22/2018 21:19	5.1	22.7	3	1.9	0.1185	2.1
4/22/2018 21:20	4.7	22.3	3	1.9	0.102	2.1
4/22/2018 21:21	3.9	22.8	3	1.9	0.1179	2.1
4/22/2018 21:22	4.1	22.5	3	2	0.1025	2.1
4/22/2018 21:23	5.2	22.6	3	2	0.1152	2.2
4/22/2018 21:24	4.3	22.7	3	2	0.1074	2.1
4/22/2018 21:25	4.5	22.3	2.9	2	0.1047	2.2
4/22/2018 21:26	5.6	22.5	3	2	0.1135	2.1
4/22/2018 21:27	5.3	22.5	3	2	0.1001	2.1
4/22/2018 21:28	4.7	22.6	2.9	2	0.1151	2.1
4/22/2018 21:29	4	22.5	2.9	2.1	0.1102	2.3
4/22/2018 21:30	3.6	22.8	2.9	2.1	0.1155	2.3
4/22/2018 21:31	4.1	22.2	2.9	2.1	0.1111	2.3
4/22/2018 21:32	2.8	22.6	2.9	2.1	0.1025	2.3
4/22/2018 21:33	4.3	22.6	2.9	2.1	0.1149	2.3
4/22/2018 21:34	5	22.7	2.9	2.1	0.1043	2.2
4/22/2018 21:35	4.5	22.5	2.9	2.1	0.1179	2.2
4/22/2018 21:36	5.5	22.9	2.9	2.1	0.1036	2.2
4/22/2018 21:37	4.4	22.7	2.9	2	0.112	2.3
4/22/2018 21:38	4.6	22.7	2.9	2	0.1043	2.4
4/22/2018 21:39	6.1	22.7	2.8	2	0.1091	2.4
4/22/2018 21:40	5.3	22.5	2.8	2	0.1116	2.4
4/22/2018 21:41	4.3	22.5	2.8	2	0.1039	2.4
4/22/2018 21:42	5	22.5	2.8	2	0.1144	2.5
4/22/2018 21:43	4.1	23.4	2.8	2	0.1	2.6
4/22/2018 21:44	5.7	24.4	2.9	2	0.1159	2.5
4/22/2018 21:45	4.3	22.8	2.8	1.9	0.1068	2.5
4/22/2018 21:46	5.2	22.4	2.8	1.9	0.1064	2.5
4/22/2018 21:47	4.7	22.3	2.8	1.9	0.1115	2.6
4/22/2018 21:48	5.4	22.3	2.8	1.9	0.1033	2.6
4/22/2018 21:49	4.6	22.4	2.8	1.9	0.1142	2.7
4/22/2018 21:50	4.2	21.9	2.8	2	0.1045	2.7
4/22/2018 21:51	4.2	22.2	2.8	2	0.1198	2.8
4/22/2018 21:52	5.4	22.1	2.7	2	0.108	2.8
4/22/2018 21:53	5.7	22.2	2.7	2	0.1018	2.8
4/22/2018 21:54	4.8	22.1	2.7	2.1	0.1176	2.8
4/22/2018 21:55	4.2	22.2	2.7	2.1	0.097	2.7
4/22/2018 21:56	5.7	22.6	2.8	2.1	0.1121	2.7
4/22/2018 21:57	4	22.6	2.8	2	0.0986	2.8
4/22/2018 21:58	4.6	22.6	2.8	2	0.108	2.8
4/22/2018 21:59	4.5	22.5	2.8	2	0.1112	2.9
4/22/2018 22:00	4.5	22.3	2.8	2	0.1044	3
4/22/2018 22:01	5.1	22.4	2.8	2	0.1101	3

4/22/2018 22:02	4.7	22.5	2.9	2	0.1002	3
4/22/2018 22:03	4.1	22.5	2.9	2	0.1141	3.1
4/22/2018 22:04	5.2	22.2	2.9	2	0.0993	3.1
4/22/2018 22:05	4.6	22.3	2.9	2	0.1126	2.8
4/22/2018 22:06	4.6	22.7	3	2	0.1	2.5
4/22/2018 22:07	5.4	22.9	3	2	0.1134	2.5
4/22/2018 22:08	3.6	23.2	3.2	2	0.1024	2.5
4/22/2018 22:09	4.1	22.5	3.2	2	0.1051	2.5
4/22/2018 22:10	4.8	22.1	2.9	1.9	0.1158	2.7
4/22/2018 22:11	5.8	22.3	2.9	2	0.0962	2.9
4/22/2018 22:12	4.3	22.1	2.9	1.9	0.1178	2.9
4/22/2018 22:13	5.1	21.9	2.9	1.9	0.1055	3
4/22/2018 22:14	4.8	22	2.8	2	0.1076	3.1
4/22/2018 22:15	4.6	22	2.8	2	0.1101	3.2
4/22/2018 22:16	5.8	22	2.8	2	0.1021	3.2
4/22/2018 22:17	4.4	21.7	2.8	2.1	0.1177	3.2
4/22/2018 22:18	5.3	21.8	2.9	2.1	0.1021	3.3
4/22/2018 22:19	5	22	2.9	2.1	0.1221	3.4
4/22/2018 22:20	4.6	22.1	3	2.1	0.1027	3.4
4/22/2018 22:21	4.7	22.2	3.1	2.1	0.1127	3.3
4/22/2018 22:22	6	21.9	3	2.1	0.1051	3.3
4/22/2018 22:23	4	22.3	3.1	2	0.0996	3.4
4/22/2018 22:24	5.1	22.2	3	2	0.1128	3.3
4/22/2018 22:25	4.2	22.4	3.1	2	0.1016	3.3
4/22/2018 22:26	4.6	22.2	3.1	2	0.1108	3.4
4/22/2018 22:27	3.7	22.1	3	2	0.1028	3.5
4/22/2018 22:28	4.4	22.1	3	2	0.1123	3.5
4/22/2018 22:29	4.5	22.2	3	2	0.1101	3.5
4/22/2018 22:30	4.9	22	3	2	0.1069	3.3
4/22/2018 22:31	4.6	21.9	3	2	0.1173	3.4
4/22/2018 22:32	4.7	21.8	3	2	0.0984	3.5
4/22/2018 22:33	5.1	21.8	3	2	0.1111	3.3
4/22/2018 22:34	5.1	21.9	3.1	2	0.1018	3.2
4/22/2018 22:35	3.9	22	3	2	0.108	2.9
4/22/2018 22:36	3.4	21.9	3	2	0.1132	2.8
4/22/2018 22:37	3.7	21.9	3	2	0.1031	2.6
4/22/2018 22:38	5.3	21.9	3.1	2	0.1127	2.6
4/22/2018 22:39	4.2	22.2	3.1	2	0.0956	2.6
4/22/2018 22:40	4.7	22.2	3.1	2	0.113	2.5
4/22/2018 22:41	5	22.1	3.1	2	0.1008	2.6
4/22/2018 22:42	4.3	21.9	3	2	0.1062	2.6
4/22/2018 22:43	5.2	22	3	2	0.1104	2.6
4/22/2018 22:44	3.3	21.9	3	2	0.0974	2.7
4/22/2018 22:45	4.2	22.3	3.1	2	0.1188	2.7
4/22/2018 22:46	6	22.3	3.2	2	0.102	2.7
4/22/2018 22:47	4.9	22.1	3.3	2	0.1137	2.8
4/22/2018 22:48	3.6	21.6	3	2	0.1052	2.9

4/22/2018 22:49	3.2	21.6	3	2	0.1033	3.1
4/22/2018 22:50	4.3	21.6	3.1	2	0.1082	2.8
4/22/2018 22:51	4.3	21.4	3	2	0.1019	2.5
4/22/2018 22:52	3.9	21.7	3	2	0.1128	2.8
4/22/2018 22:53	4.1	21.6	3	2	0.0989	3
4/22/2018 22:54	3.4	21.4	3	2	0.1126	3
4/22/2018 22:55	4.5	21.6	3	2	0.1054	3.1
4/22/2018 22:56	3.8	21.6	3	2.1	0.1164	3.2
4/22/2018 22:57	3.8	21.3	3	2.1	0.1136	3.2
4/22/2018 22:58	4.7	21.6	3.1	2.1	0.1104	3.1
4/22/2018 22:59	3.8	21.8	3.2	2.1	0.1133	3.1
4/22/2018 23:00	4.4	21.6	3.3	2	0.1036	3.2
4/22/2018 23:01	4.5	22	3.3	2	0.113	3.2
4/22/2018 23:02	3.7	21.9	3.3	2	0.1015	3.2
4/22/2018 23:03	3.5	22.1	3.5	2	0.1101	3.3
4/22/2018 23:04	4.6	22	3.5	2	0.1076	3.3
4/22/2018 23:05	5.9	21.6	3.3	2	0.106	3.2
4/22/2018 23:06	3.2	21.5	3.3	2	0.1128	3.1
4/22/2018 23:07	3.5	21.5	3.4	2	0.1082	3.1
4/22/2018 23:08	4.5	21.9	3.4	2	0.1206	3.2
4/22/2018 23:09	4.2	22.3	3.7	1.9	0.0991	3.2
4/22/2018 23:10	4.5	21.6	3.5	1.9	0.1166	3.1
4/22/2018 23:11	4.4	21.4	3.3	2	0.109	3.3
4/22/2018 23:12	4.2	21.4	3.3	2	0.1111	3.4
4/22/2018 23:13	3.7	21.6	3.3	2	0.1062	3.4
4/22/2018 23:14	4.2	21.5	3.3	2	0.0995	3.4
4/22/2018 23:15	5.6	21.3	3.3	2	0.1104	3.4
4/22/2018 23:16	4.9	21.4	3.3	2	0.0945	3.4
4/22/2018 23:17	4.5	21.4	3.2	2	0.1178	3.2
4/22/2018 23:18	4.9	21.5	3.3	2	0.1032	3.2
4/22/2018 23:19	7.3	21.6	3.3	2	0.1136	3.3
4/22/2018 23:20		21.5	3.4	2	0.1075	3.5
4/22/2018 23:21	7.7	21.7	3.4	2	0.1078	3.5
4/22/2018 23:22	9.5	21.6	3.4	2	0.111	3.6
4/22/2018 23:23	4	22.2	3.4	2	0.1008	3.6
4/22/2018 23:24	3.7	21.5	3.3	2	0.1106	3.7
4/22/2018 23:25	4	21.5	3.3	2	0.0991	3.7
4/22/2018 23:26	4.6	21.4	3.3	2	0.1123	3.8
4/22/2018 23:27	4.7	21.6	3.3	2	0.104	3.8
4/22/2018 23:28		21.7	3.3	2	0.1076	3.5
4/22/2018 23:29		21.7	3.3	2	0.1091	3.6
4/22/2018 23:30	8.1	21.7	3.3	2	0.1095	3.8
4/22/2018 23:31	4.7	21.9	3.4	2	0.1112	3.6
4/22/2018 23:32		21.3	3.3	2	0.097	3.4
4/22/2018 23:33	5.3	21.7	3.3	2	0.1191	3.5
4/22/2018 23:34	5	21.5	3.3	2	0.1017	3.7
4/22/2018 23:35		21.8	3.2	2	0.1084	3.8

4/22/2018 23:36	4.4	21.5	3.2	2	0.1107	3.9
4/22/2018 23:37	4.8	21.6	3.2	2	0.1062	3.7
4/22/2018 23:38	4.8	21.2	3.2	2	0.1105	3.7
4/22/2018 23:39	4	21.3	3.2	2.1	0.0988	3.8
4/22/2018 23:40	5.9	21.4	3.3	2.1	0.1165	3.9
4/22/2018 23:41	4.8	21.9	3.3	2.1	0.0989	3.8
4/22/2018 23:42	5.8	21.7	3.3	2	0.1156	3.9
4/22/2018 23:43	3.4	21.2	3.2	2	0.1077	3.9
4/22/2018 23:44	3.1	21.5	3.2	2	0.105	4
4/22/2018 23:45	3.7	21.4	3.2	2	0.1022	3.9
4/22/2018 23:46	4.8	21.7	3.2	2	0.1015	3.9
4/22/2018 23:47	4.3	21.6	3.2	2	0.1166	3.8
4/22/2018 23:48	5.3	22.1	3.2	2	0.1003	3.7
4/22/2018 23:49	5.5	21.9	3.2	2	0.1138	3.6
4/22/2018 23:50	4.5	21.8	3.2	2	0.0974	3.3
4/22/2018 23:51	3.4	21.6	3.2	2	0.114	3.4
4/22/2018 23:52	4.8	21.6	3.2	2	0.101	3.4
4/22/2018 23:53	3.4	21.7	3.1	2	0.1127	3.5
4/22/2018 23:54	4.4	21.5	3.1	2	0.1145	3.6
4/22/2018 23:55	4.8	21.7	3.1	2	0.1029	3.6
4/22/2018 23:56	4.2	21.4	3.1	2	0.1146	3.7
4/22/2018 23:57	2.6	21.6	3.1	2	0.0985	3.8
4/22/2018 23:58	2.9	21.4	3.1	2	0.1069	3.7
4/22/2018 23:59	4.2	21.3	3.1	2	0.1015	3.6
4/23/2018 0:00	4	21.4	3.1	2	0.1073	3.7
4/23/2018 0:01	4.8	21.8	3.1	2	0.1078	3.7
4/23/2018 0:02	5.3	21.9	3.1	2	0.1034	3.6
4/23/2018 0:03	4.6	22.6	3.1	2	0.1115	3.6
4/23/2018 0:04	3.7	21.4	3.1	2	0.0983	3.6
4/23/2018 0:05	4.5	21.6	3.2	1.9	0.115	3.7
4/23/2018 0:06	4.3	21.6	3.1	1.9	0.1012	3.7
4/23/2018 0:07	4.6	21.8	3.1	1.9	0.1166	3.7
4/23/2018 0:08	3.6	21.8	3.1	1.9	0.1041	3.8
4/23/2018 0:09	4.4	22	3.2	1.9	0.1083	3.7
4/23/2018 0:10	3.3	21.4	3.1	1.9	0.1133	3.4
4/23/2018 0:11	4.3	21.6	3.1	1.9	0.1061	3.5
4/23/2018 0:12	4.5	21.5	3.1	2	0.1145	3.6
4/23/2018 0:13	3.4	21.7	3.1	2	0.1012	3.6
4/23/2018 0:14	3.9	21.5	3.1	2	0.1177	3.5
4/23/2018 0:15	3.3	21.8	3.1	2	0.1006	3.5
4/23/2018 0:16	2.4	21.4	3.1	2	0.112	3.5
4/23/2018 0:17	5.3	21.4	3	2	0.1041	3.6
4/23/2018 0:18	4.1	21.5	3	2	0.1098	3.7
4/23/2018 0:19	4.5	21.5	3.1	2	0.1147	3.7
4/23/2018 0:20	5.2	21.7	3.1	2	0.0931	3.7
4/23/2018 0:21	4.7	21.8	3.1	2	0.1082	3.7
4/23/2018 0:22	5.3	21.8	3.1	2	0.0952	3.8

4/23/2018 0:23	3.7	22.1	3.2	2	0.1111	3.8
4/23/2018 0:24	3.8	21.8	3.1	1.9	0.1045	3.9
4/23/2018 0:25	4.1	21.7	3.1	1.9	0.106	3.9
4/23/2018 0:26	3.3	21.5	3.1	1.9	0.0979	3.8
4/23/2018 0:27	5.1	21.4	3.1	1.9	0.1015	3.9
4/23/2018 0:28	3.8	21.8	3.2	1.9	0.1108	4
4/23/2018 0:29	4.5	21.3	3.1	1.9	0.101	3.9
4/23/2018 0:30	4.7	21.7	3.1	1.9	0.1105	3.9
4/23/2018 0:31	5.8	21.6	3.1	1.9	0.1048	4
4/23/2018 0:32	3.2	21.6	3	1.9	0.1115	4
4/23/2018 0:33	5.2	21.4	3.1	1.9	0.1053	3.8
4/23/2018 0:34	3.6	21.5	3	1.9	0.1071	3.7
4/23/2018 0:35	4.7	21.3	3	1.9	0.1155	3.8
4/23/2018 0:36	3.8	21.3	3	2	0.0974	3.9
4/23/2018 0:37	4	21.3	3	2	0.1098	3.9
4/23/2018 0:38	4	21.2	3.1	1.9	0.0949	4
4/23/2018 0:39	4.3	21.6	3.1	1.9	0.1071	3.9
4/23/2018 0:40	4	21.5	3.1	1.9	0.0974	4
4/23/2018 0:41	3.7	21.7	3.1	1.9	0.1049	3.9
4/23/2018 0:42	5.1	21.6	3.1	1.9	0.1071	2.8
4/23/2018 0:43	4.9	21.5	3.1	1.9	0.1066	1.5
4/23/2018 0:44	5.2	21.5	3.1	1.9	0.1178	1.6
4/23/2018 0:45	3.8	21.6	3.1	1.9	0.096	1.7
4/23/2018 0:46	4.7	21.4	3.1	1.9	0.1066	1.7
4/23/2018 0:47	4.1	21.6	3.2	2	0.1089	1.9
4/23/2018 0:48	3.6	21.4	3.2	1.9	0.102	2
4/23/2018 0:49	4.4	21.1	3.1	1.9	0.1043	2.2
4/23/2018 0:50	3.3	21.1	3.1	1.9	0.1009	2.3
4/23/2018 0:51	4.5	21.2	3.1	2	0.1187	2.5
4/23/2018 0:52	4.6	20.9	3.1	2	0.0933	2.6
4/23/2018 0:53	3.4	21.1	3.1	2	0.1141	2.2
4/23/2018 0:54	3.7	21	3	2	0.1046	2.3
4/23/2018 0:55	4.1	21.3	3.1	2	0.1059	2.6
4/23/2018 0:56	5	21.2	3.2	2	0.1136	2.7
4/23/2018 0:57	5.2	21.1	3.3	2	0.0967	2.7
4/23/2018 0:58	3.5	21.4	3.3	2	0.1084	2.7
4/23/2018 0:59	4.8	21.2	3.3	2	0.0984	2.3
4/23/2018 1:00	4.5	21.2	3.2	2	0.1141	2.1
4/23/2018 1:01	5.6	21.3	3.2	2	0.1047	2.1
4/23/2018 1:02	4.1	21.1	3.3	2	0.1027	2.1
4/23/2018 1:03	4.8	21	3.3	2	0.1057	2.1
4/23/2018 1:04	3.2	21.3	3.3	2	0.1039	2.1
4/23/2018 1:05	4	21.3	3.4	2	0.1135	2.2
4/23/2018 1:06	4	21.3	3.6	2	0.0992	2.1
4/23/2018 1:07	5	21.1	3.4	2	0.1132	2.1
4/23/2018 1:08	3.6	21.3	3.5	2	0.1007	2.3
4/23/2018 1:09	1.9	21.2	3.4	2	0.1048	2.4

4/23/2018 1:10	4.6	21.6	3.5	1.9	0.1031	2.3
4/23/2018 1:11	5.6	21.2	3.7	1.9	0.0979	1.6
4/23/2018 1:12	2.4	21.2	3.3	1.9	0.1061	1.6
4/23/2018 1:13	4.6	21	3.3	1.9	0.0942	1.5
4/23/2018 1:14	3.6	21.1	3.3	2	0.1155	1.6
4/23/2018 1:15	4.7	20.9	3.4	2	0.1022	1.7
4/23/2018 1:16	4.2	21	3.5	2	0.1074	1.9
4/23/2018 1:17	2.9	21.6	3.5	2	0.1098	2.2
4/23/2018 1:18	3.8	21.4	3.6	1.9	0.1047	2.4
4/23/2018 1:19	3.8	21.2	3.6	1.9	0.1147	2.6
4/23/2018 1:20	4.3	21	3.4	1.9	0.1032	2.7
4/23/2018 1:21	4.3	21.2	3.4	1.9	0.1122	2.7
4/23/2018 1:22	4.1	21.4	3.5	1.9	0.1003	2.8
4/23/2018 1:23	3.6	21.1	3.5	1.9	0.1085	2.7
4/23/2018 1:24	4.1	21.1	3.5	1.9	0.1045	2.8
4/23/2018 1:25	4.7	21	3.4	1.9	0.1005	2.8
4/23/2018 1:26	4.6	21.2	3.5	1.9	0.1152	2.9
4/23/2018 1:27	3.9	21.2	3.4	1.9	0.102	3.1
4/23/2018 1:28	3.6	21.1	3.3	1.9	0.1138	3.2
4/23/2018 1:29	3	21.2	3.4	1.9	0.1076	3.4
4/23/2018 1:30	3.5	20.8	3.4	2	0.113	3.4
4/23/2018 1:31	3.7	21	3.3	2	0.1037	3.5
4/23/2018 1:32	3.3	20.9	3.4	2	0.1005	3.5
4/23/2018 1:33	4.4	21.1	3.4	2	0.1105	3.5
4/23/2018 1:34	5.2	21.1	3.4	2	0.1029	3.6
4/23/2018 1:35	3.7	21	3.4	2	0.1146	3.6
4/23/2018 1:36	5.1	21.4	3.4	2	0.1073	3.7
4/23/2018 1:37	3.1	21	3.4	2	0.1176	3.7
4/23/2018 1:38	4.8	21.3	3.4	2	0.1035	3.7
4/23/2018 1:39	4.3	21.1	3.4	1.9	0.1076	3.5
4/23/2018 1:40	5.3	21.1	3.5	1.9	0.1091	3.5
4/23/2018 1:41	4.4	21	3.5	1.9	0.1082	3.3
4/23/2018 1:42	5.2	20.8	3.3	1.9	0.1165	2.8
4/23/2018 1:43	5.9	21.1	3.4	1.9	0.0964	3
4/23/2018 1:44	4.4	21.1	3.3	1.9	0.1119	3.1
4/23/2018 1:45	4.7	20.9	3.4	1.9	0.0975	3.3
4/23/2018 1:46	4.2	21.1	3.3	1.9	0.1136	3.4
4/23/2018 1:47	3.6	21.2	3.4	1.9	0.1044	3.5
4/23/2018 1:48	3.8	21	3.3	1.9	0.102	3.4
4/23/2018 1:49	4.6	20.8	3.3	2	0.1159	3.5
4/23/2018 1:50	5.2	20.9	3.4	1.9	0.1005	2.7
4/23/2018 1:51	4.5	21	3.4	1.9	0.118	2.5
4/23/2018 1:52	4.3	21.3	3.5	1.9	0.102	2.6
4/23/2018 1:53	2.9	21	3.3	1.9	0.1125	1.7
4/23/2018 1:54	4.3	20.8	3.3	1.9	0.1027	1.5
4/23/2018 1:55	4.1	21.2	3.3	1.9	0.1131	1.5
4/23/2018 1:56	3.9	20.8	3.4	1.9	0.1053	1.5

4/23/2018 1:57	4.5	20.9	3.3	1.9	0.1058	1.5
4/23/2018 1:58	3.1	21	3.4	1.9	0.1154	1.5
4/23/2018 1:59	4	21.1	3.4	1.9	0.0983	1.5
4/23/2018 2:00	2.8	20.9	3.4	1.9	0.1176	1.5
4/23/2018 2:01	4	21.1	3.4	1.9	0.0998	1.6
4/23/2018 2:02	3.8	21	3.3	1.9	0.1172	1.6
4/23/2018 2:03	4.3	21.3	3.4	1.9	0.1071	1.7
4/23/2018 2:04	3.2	20.9	3.4	1.9	0.1133	1.7
4/23/2018 2:05	3.6	20.7	3.4	1.9	0.1148	1.7
4/23/2018 2:06	2.9	20.6	3.4	1.9	0.1008	1.7
4/23/2018 2:07	3.4	21.2	3.4	1.9	0.1168	1.8
4/23/2018 2:08	3	20.8	3.5	1.9	0.094	1.9
4/23/2018 2:09	4.5	21.1	3.6	1.9	0.1181	1.9
4/23/2018 2:10	4.3	20.7	3.5	1.9	0.0997	2
4/23/2018 2:11	2.8	20.8	3.5	1.9	0.1214	2.1
4/23/2018 2:12	3.8	20.9	3.6	1.9	0.1107	2.1
4/23/2018 2:13	3.9	20.9	3.6	1.9	0.1091	2.2
4/23/2018 2:14	4.1	20.9	3.6	1.9	0.1112	2.3
4/23/2018 2:15	5.5	20.9	3.5	1.9	0.0969	2.3
4/23/2018 2:16	4.5	21	3.9	1.9	0.1148	2.2
4/23/2018 2:17	4.3	20.7	3.7	2	0.1008	2.3
4/23/2018 2:18	4.1	20.7	3.8	2	0.1175	2.3
4/23/2018 2:19	4.3	20.8	3.6	2	0.1027	2.5
4/23/2018 2:20	5.2	20.5	3.8	2	0.1122	2.6
4/23/2018 2:21	3.5	20.5	4	2	0.1082	2.8
4/23/2018 2:22	3.8	20.8	3.6	2	0.1084	2.9
4/23/2018 2:23	3.6	20.5	3.7	2	0.1203	2.7
4/23/2018 2:24	4.5	20.5	4.1	2	0.1087	2.7
4/23/2018 2:25	4.3	20.6	4.4	2	0.1169	2.7
4/23/2018 2:26	3.3	20.6	4.1	2	0.1024	2.9
4/23/2018 2:27	4.4	20.4	4.1	2	0.1164	2.9
4/23/2018 2:28	2.9	20.7	4	2	0.1109	2.9
4/23/2018 2:29	2.9	20.3	4.2	2	0.1115	2.9
4/23/2018 2:30	3.3	20.3	4.4	2	0.1158	2.8
4/23/2018 2:31	2.4	20.5	4.3	2	0.1046	2.8
4/23/2018 2:32	3.5	20.1	4.4	2	0.1113	2.7
4/23/2018 2:33	4.4	20.6	4.9	2	0.1028	2.7
4/23/2018 2:34	4.3	20.5	5.5	1.9	0.1137	2.5
4/23/2018 2:35	3.4	20.5	4.6	1.9	0.1071	2.6
4/23/2018 2:36	3.8	20.4	4.4	1.9	0.1146	2.6
4/23/2018 2:37	3.4	20.4	5.2	1.9	0.1055	2.6
4/23/2018 2:38	3.7	20.6	4.6	1.9	0.1076	2.7
4/23/2018 2:39	3.3	20.1	4.3	2	0.1194	2.8
4/23/2018 2:40	4.9	20.4	4.3	2	0.0991	2.8
4/23/2018 2:41	3.1	20.2	4.6	2	0.1206	2.8
4/23/2018 2:42	3.1	20.4	4.8	2	0.1105	2.9
4/23/2018 2:43		20.4	4.8	2	0.1144	2.9

4/23/2018 2:44	6.7	20.4	4.8	2	0.1181	2.8
4/23/2018 2:45	3.8	20.2	4.7	2.1	0.1116	3
4/23/2018 2:46	3.5	20.3	4.7	2.1	0.1226	3
4/23/2018 2:47	3.3	20.3	4.8	2	0.1084	2.8
4/23/2018 2:48	4.7	20.2	5.1	2	0.1204	2.6
4/23/2018 2:49		20.4	5.4	2	0.1148	2.5
4/23/2018 2:50		20.4	5.7	2	0.1143	2.5
4/23/2018 2:51	5	20.5	5	2	0.1288	2.5
4/23/2018 2:52	2.9	20.3	5.4	2	0.1115	2.3
4/23/2018 2:53	2.9	20.2	5	2	0.1241	2
4/23/2018 2:54		20	5.2	2	0.1131	2
4/23/2018 2:55		20.1	5.2	2	0.1218	2
4/23/2018 2:56	4.6	20.2	5.2	2	0.1152	2
4/23/2018 2:57	2.3	20.2	5.2	2	0.1123	2
4/23/2018 2:58	4.6	19.7	4.9	2	0.1182	2
4/23/2018 2:59	3.6	20.4	5.4	2.1	0.1113	2.1
4/23/2018 3:00	4.1	20.3	5.5	2.1	0.1237	2.2
4/23/2018 3:01	4.7	20.1	5.8	2.1	0.1138	2.1
4/23/2018 3:02	2.4	20	5.6	2.1	0.1204	2.1
4/23/2018 3:03	3.5	19.8	5.2	2.1	0.1187	2.1
4/23/2018 3:04	4.7	20.1	5.6	2.1	0.1149	2.1
4/23/2018 3:05	4.3	20.3	5.8	2.1	0.1202	2.1
4/23/2018 3:06	4.8	20	5.8	2.1	0.1179	2.1
4/23/2018 3:07	4.8	20.1	5.8	2.1	0.1296	2
4/23/2018 3:08	3.8	20.3	5.8	2.1	0.1212	1.7
4/23/2018 3:09	4.4	20.1	6	2.1	0.1176	1.6
4/23/2018 3:10	2.9	19.9	5.5	2.1	0.1242	1.8
4/23/2018 3:11	3.5	20	5.9	2.1	0.1077	1.9
4/23/2018 3:12	4.5	20.3	5.8	2.1	0.131	2.1
4/23/2018 3:13	2.4	19.9	5.4	2.1	0.1056	2.2
4/23/2018 3:14	4.3	20.2	5.5	2.1	0.1301	2.3
4/23/2018 3:15	3.3	20	5.9	2.1	0.1257	2.1
4/23/2018 3:16	3.2	20	5.8	2.2	0.1213	2.1
4/23/2018 3:17	3.2	20.2	5.7	2.2	0.124	2.2
4/23/2018 3:18	3.5	20.2	5.6	2.2	0.1222	2.1
4/23/2018 3:19	3.9	20.1	5.6	2.2	0.1253	1.6
4/23/2018 3:20	3.9	20.1	5.8	2.2	0.115	1.6
4/23/2018 3:21	3.4	19.9	6.1	2.2	0.1256	1.6
4/23/2018 3:22	3.3	19.6	6.2	2.2	0.1198	1.6
4/23/2018 3:23	3.6	20	5.9	2.2	0.1266	1.5
4/23/2018 3:24	4.2	20	6	2.2	0.1307	1.5
4/23/2018 3:25	4	20.1	6.1	2.2	0.1224	1.6
4/23/2018 3:26	3.1	19.8	5.8	2.2	0.1326	1.5
4/23/2018 3:27	3.2	20.1	5.8	2.1	0.1112	1.6
4/23/2018 3:28	3.6	20.1	5.9	2.2	0.1345	1.6
4/23/2018 3:29	3.4	19.8	5.8	2.2	0.1146	1.5
4/23/2018 3:30	3.2	19.6	5.7	2.2	0.1249	1.5

4/23/2018 3:31	3.1	20	5.9	2.2	0.1292	1.5
4/23/2018 3:32	4.3	20.2	6	2.2	0.1291	1.6
4/23/2018 3:33	4.1	20.4	6.3	2.2	0.1341	1.6
4/23/2018 3:34	3.8	20	6	2.2	0.1233	1.7
4/23/2018 3:35	3.6	20.1	5.9	2.2	0.1352	1.7
4/23/2018 3:36	3.3	20.1	5.9	2.2	0.1178	1.7
4/23/2018 3:37	2.5	19.9	6	2.2	0.1318	1.8
4/23/2018 3:38	3.4	20	6	2.2	0.1247	1.8
4/23/2018 3:39	5	20.1	5.9	2.2	0.1237	1.8
4/23/2018 3:40	3.5	19.7	6	2.2	0.1263	1.6
4/23/2018 3:41	5.6	20.4	6.1	2.2	0.1241	1.6
4/23/2018 3:42	3.9	20.1	6.4	2.2	0.1312	1.5
4/23/2018 3:43	3.4	19.8	6.3	2.2	0.1183	1.5
4/23/2018 3:44	3	20.1	5.9	2.2	0.1396	1.6
4/23/2018 3:45	4.7	19.7	5.9	2.2	0.1178	1.6
4/23/2018 3:46	4.2	20.1	6	2.2	0.1284	1.6
4/23/2018 3:47	4.5	19.8	6	2.2	0.1171	1.6
4/23/2018 3:48	4.8	19.9	5.9	2.2	0.1296	1.6
4/23/2018 3:49	3.7	20.1	6.3	2.2	0.128	1.6
4/23/2018 3:50	3.3	20.4	6.2	2.2	0.1316	1.6
4/23/2018 3:51	3.5	20	6	2.2	0.1306	1.6
4/23/2018 3:52	3.5	19.8	5.9	2.2	0.124	1.6
4/23/2018 3:53	4.2	20.1	6	2.2	0.1323	1.6
4/23/2018 3:54	2.8	20.2	6.2	2.2	0.1211	1.6
4/23/2018 3:55	4	19.9	6.3	2.2	0.1314	1.5
4/23/2018 3:56	3.4	20.1	6	2.2	0.1201	1.6
4/23/2018 3:57	3.9	20.2	6.2	2.2	0.1286	1.6
4/23/2018 3:58	2.4	19.8	6.2	2.2	0.1285	1.6
4/23/2018 3:59	3.7	20.1	6.2	2.2	0.1239	1.6
4/23/2018 4:00	4.5	20.2	5.9	2.2	0.1307	1.6
4/23/2018 4:01	2.6	20.1	6	2.2	0.1208	1.6
4/23/2018 4:02	2.9	20	6	2.2	0.1319	1.7
4/23/2018 4:03	3.9	19.8	5.9	2.2	0.1161	1.7
4/23/2018 4:04	4.7	20.1	6.2	2.2	0.1311	1.7
4/23/2018 4:05	2.7	19.9	6.2	2.2	0.1224	1.8
4/23/2018 4:06	3.2	20.1	6.1	2.2	0.1267	1.7
4/23/2018 4:07	3.7	19.9	6.1	2.2	0.1287	1.8
4/23/2018 4:08	1.8	19.8	6.2	2.3	0.1254	1.8
4/23/2018 4:09	3.5	19.9	6.3	2.2	0.1349	1.7
4/23/2018 4:10	3	19.9	6.5	2.2	0.1118	1.7
4/23/2018 4:11	3.5	19.8	6.5	2.2	0.1376	1.7
4/23/2018 4:12	3.1	20.1	6.7	2.2	0.1222	1.8
4/23/2018 4:13	5	19.9	6.6	2.2	0.1363	1.9
4/23/2018 4:14	3.4	19.7	6.2	2.2	0.1211	1.9
4/23/2018 4:15	2.1	19.7	6.5	2.2	0.131	1.9
4/23/2018 4:16	2.7	19.9	6.5	2.2	0.1262	1.9
4/23/2018 4:17	4.5	20	6.5	2.2	0.1176	1.9

4/23/2018 4:18	3.1	20	6.3	2.2	0.1257	2
4/23/2018 4:19	2.1	19.8	6.1	2.2	0.1222	2
4/23/2018 4:20	2.8	20	6.4	2.3	0.1384	2
4/23/2018 4:21	2.8	19.9	6.5	2.3	0.1298	2
4/23/2018 4:22	4.7	19.9	6.8	2.3	0.1347	2.1
4/23/2018 4:23	5.2	19.8	6.4	2.3	0.1257	2.1
4/23/2018 4:24	3.4	19.8	6.4	2.3	0.1239	2.2
4/23/2018 4:25	5.1	19.8	6.6	2.2	0.1326	2.2
4/23/2018 4:26	3.7	19.9	6.7	2.3	0.12	2.1
4/23/2018 4:27	2.9	19.9	6.7	2.2	0.1384	2.1
4/23/2018 4:28	4.3	19.9	6.5	2.2	0.1256	2.2
4/23/2018 4:29	4.3	20	6.7	2.2	0.1332	2.3
4/23/2018 4:30	3.3	19.6	6.6	2.3	0.1261	2.3
4/23/2018 4:31	3.3	20.1	6.8	2.3	0.1228	2.3
4/23/2018 4:32	3.9	20	6.5	2.3	0.1391	2.2
4/23/2018 4:33	2.8	20	6.7	2.3	0.1272	2.1
4/23/2018 4:34	5	19.9	6.5	2.3	0.1402	2
4/23/2018 4:35	4.5	19.7	6.4	2.3	0.1245	1.9
4/23/2018 4:36	4.2	19.5	6.7	2.3	0.1399	1.7
4/23/2018 4:37	4.6	19.6	7.3	2.3	0.1318	1.8
4/23/2018 4:38	3.2	19.9	6.7	2.3	0.1305	1.7
4/23/2018 4:39	2.9	19.9	6.7	2.3	0.13	1.6
4/23/2018 4:40	2.1	19.7	6.5	2.3	0.124	1.6
4/23/2018 4:41	2.7	19.9	6.7	2.3	0.1327	1.6
4/23/2018 4:42	2.8	19.5	6.9	2.3	0.1233	1.6
4/23/2018 4:43	2.5	19.8	7.3	2.3	0.1333	1.6
4/23/2018 4:44	2.9	19.8	6.7	2.3	0.12	1.6
4/23/2018 4:45	3.8	19.8	6.8	2.3	0.1355	1.6
4/23/2018 4:46	2.7	19.8	6.8	2.3	0.1273	1.6
4/23/2018 4:47	3.7	19.8	6.8	2.3	0.1251	1.6
4/23/2018 4:48	2.1	19.9	6.7	2.3	0.1366	1.6
4/23/2018 4:49	4.1	19.6	6.6	2.3	0.1193	1.7
4/23/2018 4:50	4.2	19.6	6.6	2.3	0.1346	1.7
4/23/2018 4:51	3.4	20	6.8	2.3	0.1205	1.7
4/23/2018 4:52	2.9	19.8	7.3	2.3	0.1402	1.8
4/23/2018 4:53	3.8	19.5	7	2.3	0.126	1.8
4/23/2018 4:54	2.6	19.4	7	2.3	0.1307	1.8
4/23/2018 4:55	4.4	19.7	6.7	2.3	0.1334	1.8
4/23/2018 4:56	2.5	19.7	7.3	2.3	0.1272	1.8
4/23/2018 4:57	3.3	19.6	7.4	2.3	0.1379	1.8
4/23/2018 4:58	3.8	19.5	7.6	2.3	0.1231	1.7
4/23/2018 4:59	3.8	19.6	7.5	2.3	0.13	1.7
4/23/2018 5:00	4.1	19.5	7.4	2.3	0.1301	1.7
4/23/2018 5:01	3.8	20	7.3	2.3	0.1345	1.8
4/23/2018 5:02	4.4	19.7	7.4	2.3	0.1331	1.8
4/23/2018 5:03	3	19.7	7.2	2.3	0.1294	1.8
4/23/2018 5:04	5.3	19.7	7.4	2.3	0.1412	1.9

4/23/2018 5:05	2.4	19.4	7.1	2.3	0.1279	1.9
4/23/2018 5:06	3	19.7	6.8	2.3	0.1388	1.9
4/23/2018 5:07	3.4	19.7	7.2	2.4	0.1263	1.9
4/23/2018 5:08	2.8	19.5	7.2	2.4	0.1343	1.8
4/23/2018 5:09	4.1	19.8	7.2	2.4	0.1325	1.8
4/23/2018 5:10	2.8	19.9	7.2	2.4	0.1263	1.8
4/23/2018 5:11	4.2	19.9	7.3	2.4	0.1337	1.8
4/23/2018 5:12	2.9	19.6	7.5	2.4	0.125	1.8
4/23/2018 5:13	3.8	19.4	7.4	2.4	0.1386	1.9
4/23/2018 5:14	3.8	19.5	7.3	2.4	0.1315	2.1
4/23/2018 5:15	2.8	19.4	7.6	2.4	0.1406	2.1
4/23/2018 5:16	3.4	19.5	7.7	2.5	0.1315	2.1
4/23/2018 5:17	2.4	19.3	8.2	2.5	0.1389	2.2
4/23/2018 5:18	2.7	19.3	8	2.5	0.1317	2.3
4/23/2018 5:19	3.3	19.4	7.6	2.5	0.1295	2.3
4/23/2018 5:20	3.9	19	7.7	2.5	0.1374	2.4
4/23/2018 5:21	2.8	19.3	7.6	2.6	0.1322	2.3
4/23/2018 5:22	4.3	19.3	7.8	2.6	0.1477	2.3
4/23/2018 5:23	4.3	19.3	8.7	2.6	0.129	2.4
4/23/2018 5:24	2.9	19.3	8.8	2.6	0.1397	2.4
4/23/2018 5:25	4.6	19.3	8.1	2.6	0.1339	2.5
4/23/2018 5:26	2.9	19.2	7.6	2.6	0.1289	2.6
4/23/2018 5:27	2.9	19.3	7.6	2.6	0.1519	2.5
4/23/2018 5:28		19.3	8	2.7	0.1301	2.6
4/23/2018 5:29	2.5	19.4	8.1	2.7	0.1391	2.5
4/23/2018 5:30	3.1	19.6	8.5	2.7	0.1298	1.9
4/23/2018 5:31	2.5	19.3	8.5	2.7	0.1425	1.8
4/23/2018 5:32	3.5	19.3	8.5	2.6	0.1364	1.8
4/23/2018 5:33	3.5	19.4	8.8	2.7	0.1302	1.9
4/23/2018 5:34	2	19.4	7.9	2.7	0.1381	2
4/23/2018 5:35		19.3	8	2.7	0.1297	2.1
4/23/2018 5:36		19.2	8	2.7	0.1413	2.2
4/23/2018 5:37	9.4	19.3	9.2	2.7	0.136	2.4
4/23/2018 5:38	5.6	19.1	9.8	2.7	0.1507	2.4
4/23/2018 5:39		19.2	9.2	2.7	0.1321	2.4
4/23/2018 5:40	7.8	19.2	8.6	2.7	0.144	2.4
4/23/2018 5:41	1.9	19.4	8.2	2.7	0.1368	2.5
4/23/2018 5:42	3.7	19.5	8.1	2.7	0.1266	2.6
4/23/2018 5:43		19.9	8.4	2.7	0.1461	2.6
4/23/2018 5:44	2.3	19.2	8.4	2.7	0.1337	2.7
4/23/2018 5:45	2.7	19.2	8.8	2.7	0.1359	2.6
4/23/2018 5:46		19.6	8.6	2.7	0.1263	2.6
4/23/2018 5:47		19.4	8.8	2.7	0.134	2.6
4/23/2018 5:48	4.4	19.4	8.8	2.8	0.1485	2.7
4/23/2018 5:49	3.4	19.2	8.2	2.8	0.1346	2.7
4/23/2018 5:50		19.3	8.2	2.8	0.1387	2.8
4/23/2018 5:51	1.5	19.6	8.3	2.8	0.1311	2.8

4/23/2018 5:52		19.6	8.6	2.8	0.146	2.8
4/23/2018 5:53	3.1	19.2	9.4	2.7	0.1416	2.9
4/23/2018 5:54	3.2	19.4	8.5	2.7	0.1444	3
4/23/2018 5:55	3.2	19.5	8.3	2.7	0.144	3
4/23/2018 5:56	3.2	19.6	8.2	2.7	0.1321	3.1
4/23/2018 5:57	3.4	19.5	8.3	2.7	0.1448	3.1
4/23/2018 5:58	3.7	19.4	8.4	2.7	0.1337	3.1
4/23/2018 5:59	3.6	19.4	8.7	2.7	0.1486	3.1
4/23/2018 6:00	2.7	19.5	8.5	2.7	0.1382	3.1
4/23/2018 6:01	3.8	19.5	8.6	2.7	0.1342	3.1
4/23/2018 6:02	4.1	19.6	8.7	2.7	0.1485	2.7
4/23/2018 6:03	1.9	19.5	9	2.7	0.1368	2.4
4/23/2018 6:04	3.1	19.3	8.7	2.7	0.1521	2.6
4/23/2018 6:05	4.1	19.4	8.6	2.7	0.1343	2.8
4/23/2018 6:06	3.6	19.6	8.4	2.7	0.1459	2.9
4/23/2018 6:07	3.7	19.5	8.4	2.7	0.1407	3
4/23/2018 6:08	2.2	19.1	8.9	2.7	0.1364	3
4/23/2018 6:09	3.9	19.2	8.9	2.7	0.1478	2.9
4/23/2018 6:10	2.8	19.5	9	2.7	0.1363	2.9
4/23/2018 6:11	3.2	19.2	8.7	2.7	0.1467	2.9
4/23/2018 6:12	3.2	19.2	8.3	2.8	0.1305	3
4/23/2018 6:13	3.4	19.3	8.4	2.8	0.1437	3
4/23/2018 6:14	3.7	19.4	9	2.8	0.1477	3.1
4/23/2018 6:15	3.3	18.9	9	2.9	0.1434	3.1
4/23/2018 6:16	1.6	19.1	9.3	2.9	0.1513	3
4/23/2018 6:17	3.8	19.3	9.5	2.9	0.1413	3
4/23/2018 6:18	3.8	19.4	9.3	3	0.1433	3
4/23/2018 6:19	2.9	19.1	9.3	3	0.1316	2.8
4/23/2018 6:20	3.1	19.3	9.2	3	0.1468	2.9
4/23/2018 6:21	3	19.1	9.2	3.1	0.1478	3
4/23/2018 6:22	3	18.7	9.1	3.1	0.1359	3.1
4/23/2018 6:23	3.3	19.2	10.1	3.2	0.1567	3
4/23/2018 6:24	2.2	19.1	9.7	3.3	0.1376	3.1
4/23/2018 6:25	2.3	19.3	9	3.3	0.1503	3
4/23/2018 6:26	3.2	19	9.7	3.3	0.1461	3
4/23/2018 6:27	3.4	19.1	9.7	3.4	0.1517	2.9
4/23/2018 6:28	3.6	19.3	9.3	3.4	0.1498	3
4/23/2018 6:29	3.9	19.2	9.5	3.4	0.137	3.1
4/23/2018 6:30	3.3	19.4	9.8	3.5	0.1514	3
4/23/2018 6:31	3.6	19.1	9.8	3.5	0.1459	3
4/23/2018 6:32	3.9	19.3	9.6	3.5	0.1548	3
4/23/2018 6:33	3.1	19.1	9.4	3.5	0.1482	3
4/23/2018 6:34	3.4	19.5	10	3.6	0.1434	3.1
4/23/2018 6:35	2.6	19	9.9	3.6	0.1559	2.7
4/23/2018 6:36	2.1	19.3	9.6	3.6	0.1431	2.5
4/23/2018 6:37	3.5	19.2	9.2	3.6	0.16	2.4
4/23/2018 6:38	2.3	19.1	9.4	3.6	0.1414	2.5

4/23/2018 6:39	3.1	18.9	9.7	3.6	0.1483	2.5
4/23/2018 6:40	1.3	19.1	9.7	3.5	0.1516	2.6
4/23/2018 6:41	2.5	19.2	9.5	3.5	0.1477	2.6
4/23/2018 6:42	2.9	19.3	9.5	3.5	0.1496	2.6
4/23/2018 6:43	2.4	19.1	9.7	3.5	0.1424	2.7
4/23/2018 6:44	3.8	19.2	9.6	3.5	0.1494	2.7
4/23/2018 6:45	3.8	19.1	9.5	3.5	0.1472	2.8
4/23/2018 6:46	3	19.2	9.9	3.5	0.1471	2.8
4/23/2018 6:47	3.2	19.2	9.6	3.5	0.1508	2.8
4/23/2018 6:48	3.3	19.4	9.7	3.5	0.142	2.8
4/23/2018 6:49	4.6	19.3	9.4	3.5	0.1516	2.7
4/23/2018 6:50	2	19.4	9.5	3.5	0.1455	2.5
4/23/2018 6:51	3.7	19.4	9.4	3.4	0.1403	2.3
4/23/2018 6:52	4.7	19.2	9.2	3.4	0.1415	2.3
4/23/2018 6:53	3.7	19.1	9.2	3.4	0.1514	2.3
4/23/2018 6:54	4	19.1	9.2	3.5	0.1522	2.4
4/23/2018 6:55	3.4	19	9.2	3.5	0.1346	2.5
4/23/2018 6:56	3.2	19.1	9.3	3.5	0.1564	2.5
4/23/2018 6:57	3	19.3	9.3	3.5	0.141	2.5
4/23/2018 6:58	2.9	19.3	9.4	3.5	0.1558	2.5
4/23/2018 6:59	2.9	19.1	9.5	3.4	0.1525	2.5
4/23/2018 7:00	3	19.1	9.3	3.4	0.1414	2.5
4/23/2018 7:01	3.4	19.3	9.3	3.4	0.1588	2.5
4/23/2018 7:02	3.7	19.2	9.1	3.4	0.1464	2.6
4/23/2018 7:03	2.1	19.4	9	3.4	0.1576	2.8
4/23/2018 7:04	4.3	19.6	9.1	3.5	0.1501	2.7
4/23/2018 7:05	3.4	19.3	9.3	3.5	0.1465	2.7
4/23/2018 7:06	3.6	19.2	9.1	3.5	0.1482	2.7
4/23/2018 7:07	1.5	19.2	8.9	3.5	0.142	2.7
4/23/2018 7:08	2.7	19.4	8.8	3.5	0.1558	2.7
4/23/2018 7:09	2.4	19.3	9	3.5	0.1398	2.7
4/23/2018 7:10	4.7	19.5	9	3.5	0.1509	2.5
4/23/2018 7:11	2.8	19.1	9	3.5	0.1495	2.5
4/23/2018 7:12	3.4	19.2	8.9	3.5	0.1449	2.5
4/23/2018 7:13	4.5	19.2	9	3.5	0.1525	2.5
4/23/2018 7:14	3.5	19	9	3.5	0.1502	2.5
4/23/2018 7:15	3.2	19.6	9	3.5	0.1568	2.4
4/23/2018 7:16	2.4	19.1	8.9	3.5	0.143	2.5
4/23/2018 7:17	2.9	19.3	8.9	3.6	0.1587	2.5
4/23/2018 7:18	3.1	19.2	8.9	3.6	0.1509	2.6
4/23/2018 7:19	3.9	19.5	8.9	3.6	0.1517	2.6
4/23/2018 7:20	3.5	19.2	9	3.6	0.1455	2.5
4/23/2018 7:21	4.5	19.1	9	3.6	0.1455	2.6
4/23/2018 7:22	3.4	19.2	9.2	3.6	0.1581	2.6
4/23/2018 7:23	3.3	20.5	9.1	3.6	0.1555	2.5
4/23/2018 7:24	2.4	20.2	8.9	3.6	0.1539	2.4
4/23/2018 7:25	2.5	16	8.9	3.6	0.1676	1.9

4/23/2018 7:26			1.9	18.3	8.9	3.6	0.1873	0.3
4/23/2018 7:27			3	18.9	8.8	3.6	0.2004	0
4/23/2018 7:28			2.7	19.4	8.9	3.6	0.192	1.4
4/23/2018 7:29			2.3	19.4	8.9	3.6	0.202	21
4/23/2018 7:30	0.2	-0.1	2.1	19.4	9	3.6	0.207	29.3
4/23/2018 7:31	0.2	-0.6	2.7	19.2	8.9	3.7	0.1995	29.7
4/23/2018 7:32	0.2	-0.4	2	19.5	9.1	3.7	0.2064	29.8
4/23/2018 7:33	0.3	0.5	3.5	19.2	9.2	3.6	0.1998	29.6
4/23/2018 7:34	0.3	0.2	1.6	19.3	9.1	3.6	0.209	23.3
4/23/2018 7:35	0.3	0.4	3.6	19.2	9	3.6	0.2147	2.4
4/23/2018 7:36	0.3	-0.4	3.7	19.3	9	3.6	0.2134	0.2
4/23/2018 7:37	0.2	-0.2	2.8	19.2	9.2	3.6	0.2201	0.1
4/23/2018 7:38	0.3	0.5	3.8	19.1	9	3.6	0.2102	0.1
4/23/2018 7:39	0.3	-0.1	2.7	19.5	9.1	3.6	0.2245	0.5
4/23/2018 7:40	0.3	0.3	3	19.2	9.1	3.6	0.1963	1.5
4/23/2018 7:41	0.3	-0.4	4	19.3	9	3.6	0.2105	1.6
4/23/2018 7:42	0.3	-0.3	3	19.2	9	3.6	0.2165	1.7
4/23/2018 7:43	0.3	-0.1	4	19.2	9	3.6	0.1672	1.6
4/23/2018 7:44	0.3	0.7	2.2	19.3	9	3.6	-0.0225	1.7
4/23/2018 7:45	0.3	0.1	2.8	19.2	9	3.6	-0.0439	1.7
4/23/2018 7:46	0.3	0.6	3.3	19.2	9.1	3.6	-0.0345	1.6
4/23/2018 7:47	0.4	0	3	19.3	9	3.6		1.7
4/23/2018 7:48	0.4	-0.1	3	19.3	9.1	3.7		1.7
4/23/2018 7:49	0.4	0.7	2.5	19	9	3.7		1.7
4/23/2018 7:50	0.4	1.4	3.5	19.2	9	3.7		1.7
4/23/2018 7:51	0.4	0.1	1.9	19.2	9.1	3.7		1.7
4/23/2018 7:52	0.4	-0.9	3.6	19	9.1	3.7		1.7
4/23/2018 7:53	0.4	-0.2	3	19.3	9.1	3.7		1.7
4/23/2018 7:54	0.4	0.2	4	19.4	9.2	3.8		1.7
4/23/2018 7:55	0.4	0.1	2.5	19.5	9.1	3.8		1.7
4/23/2018 7:56	0.3	-0.3	3.8	19.1	9.3	3.8		1.6
4/23/2018 7:57	0.3	0.5	2.4	19.2	10.4	3.8		1.7
4/23/2018 7:58	0.4	-0.4	3.5	19.2	10.6	3.8		1.6
4/23/2018 7:59	0.4	0.5	3.4	19.1	10.5	3.8		1.6
4/23/2018 8:00	0.4	0.8	3.7	19.2	10.4	3.8		1.6
4/23/2018 8:01	0.3	0.4	2.2	19.1	10.9	3.8		1.6
4/23/2018 8:02	0.3	0.9	2.6	19.3	10.9	3.9		1.6
4/23/2018 8:03	0.2	0.8	2.4	19.3	10.7	3.9		1.7
4/23/2018 8:04	0.2	-0.5	3.8	19.4	9.8	3.9		1.6
4/23/2018 8:05	0.2	-0.1	2.4	19.4	10.3	4		1.6
4/23/2018 8:06	0.2	-0.1	2.9	18.9	10.2	4		1.6
4/23/2018 8:07	0.2	-0.1	3.7	19	9.7	4		1.7
4/23/2018 8:08	0.2	0.4	2	19	10.4	4		1.6
4/23/2018 8:09	0.2	0.3	3.1	19.8	10.7	4.1		1.7
4/23/2018 8:10					10.3	4.1		1.6
4/23/2018 8:11					10	4.1		1.7
4/23/2018 8:12					10	4.1		1.7

4/23/2018 8:13					10.5	4.2		1.7
4/23/2018 8:14					10.5	4.2		1.7
4/23/2018 8:15					10.2	4.2		1.7
4/23/2018 8:16					10.2	4.2		1.7
4/23/2018 8:17					9.9	4.3		1.7
4/23/2018 8:18					11	4.3		1.7
4/23/2018 8:19					10.3	4.3		1.7
4/23/2018 8:20					10	4.3		1.7
4/23/2018 8:21					10.2	4.4		1.7
4/23/2018 8:22					9.8	4.4		1.7
4/23/2018 8:23					9.7	4.4		1.7
4/23/2018 8:24					9.7	4.4		1.7
4/23/2018 8:25					9.9	4.5		1.8
4/23/2018 8:26					9.8	4.5		1.8
4/23/2018 8:27					9.8	4.5		1.7
4/23/2018 8:28					9.9	4.5		1.7
4/23/2018 8:29					10.2	4.6		1.7
4/23/2018 8:30					9.9	4.6		1.7
4/23/2018 8:31					10.1	4.6		1.7
4/23/2018 8:32					10	4.7		1.7
4/23/2018 8:33					10.3	4.7		1.7
4/23/2018 8:34					9.7	4.7		1.7
4/23/2018 8:35					10	4.8		1.7
4/23/2018 8:36					9.9	4.8		1.7
4/23/2018 8:37					15.2	4.9		1.7
4/23/2018 8:38					0.6	4.6		1.7
4/23/2018 8:39					0	4.1		1.7
4/23/2018 8:40	0.3	0.1	3.1	25	0	3.4		1.7
4/23/2018 8:41	0.3	0.4	4.5	20.7	0	2.8		1.7
4/23/2018 8:42	0.2	0.2	3.3	19	0	2.3		1.7
4/23/2018 8:43	0.2	0	1.5	18.3	0	1.7		1.7
4/23/2018 8:44	0.2	0.2	3.4	18.1	0	1.1		1.7
4/23/2018 8:45	0.2	-0.2	2.9	17.6	0	0.5		1.7
4/23/2018 8:46	0.2	0.7	0.6	17.5	0	-0.1		1.7
4/23/2018 8:47	0.2	-0.4	2.9	17.6	0	-0.1		1.8
4/23/2018 8:48	0.3	0.1	3.1	17.3	0	-0.1		1.8
4/23/2018 8:49	0.2	0.9	3.5	17.1	0	0		1.7
4/23/2018 8:50	0.3	0.3	2.2	17.4	13.6	2.2	0.2326	1.7
4/23/2018 8:51	0.3	0.5	1.2	17.3	10.2	5.6	0.2447	1.7
4/23/2018 8:52	0.3	0.9	3.7	17.1	10.2	5.4	0.2443	1.7
4/23/2018 8:53	0.3	-0.4	4.4	17.2	10.1	5.4	0.2287	1.7
4/23/2018 8:54	0.2	1		17.3	10.2	5.5	0.2436	1.7
4/23/2018 8:55	0.2	0.4		17.3	10	5.5	0.2307	1.7
4/23/2018 8:56	0.2	0.6	9.9	17.2	10.2	5.5	0.2399	1.7
4/23/2018 8:57	0.2	0	3.2	17.4	10.4	5.5	0.2371	1.7
4/23/2018 8:58	0.2	0.9		19.4	22.1	37.3	0.3637	1.7
4/23/2018 8:59	0.2	0.5	3.2	23.4	29.8	74.3	0.734	1.8

4/23/2018 9:00	0.2	0.2	4	27.9	33.6	91.4	1.1598	1.8
4/23/2018 9:01	0.2	0.3		30.5	36	102.5	1.6108	1.9
4/23/2018 9:02	0.2	0.4	4.6	30.7	35.8	101.5	1.8801	1.9
4/23/2018 9:03	0.2	0.6	3.5	30.5	35.1	101.5	2.0143	1.9
4/23/2018 9:04	0.2	0.4	3.7	29.3	34.4	101.2	2.0781	1.9
4/23/2018 9:05	0.3	0	3.1	29.4	33.7	100.7	2.0783	1.9
4/23/2018 9:06	0.3	-0.3		28.7	33.1	100	2.0697	1.9
4/23/2018 9:07	0.2	0.5		28.3	32.4	99.1	2.0572	1.9
4/23/2018 9:08	0.2	0		27.9	32.3	98.1	2.0985	1.9
4/23/2018 9:09	0.3	0.4	2.5	28.1	32	96.9	2.0892	1.9
4/23/2018 9:10	0.3	0.4	3.2	27.3	31.6	96	2.0478	1.9
4/23/2018 9:11	0.3	0.4	3.3	27.2	31.1	94.6	2.0508	1.9
4/23/2018 9:12	0.2	0.1	2.9	26.6	30.9	93.3	1.9963	1.9
4/23/2018 9:13	0.1	0.2	2.4	26.3	30.9	92.2	1.9864	1.9
4/23/2018 9:14	0.1	-0.4	4.9	26.2	30.3	91	1.968	1.9
4/23/2018 9:15	0.1	0.1	3.9	25.6	29.4	89.9	1.9311	1.9
4/23/2018 9:16	0.1	0.9	4.3	25.2	29	88.6	1.9187	1.8
4/23/2018 9:17	0.1	0.9	4.1	25.1	28.6	87.7	1.8794	1.9
4/23/2018 9:18	0.1	0.6	3.9	24.9	28.3	86.5	1.8798	1.9
4/23/2018 9:19	0.1	0.6	4.6	24.3	27.8	85.2	1.8226	1.9
4/23/2018 9:20	0	0.7	3.7	24.5	27.6	84.1	1.8279	1.9
4/23/2018 9:21	0	0.4	4	24	27.2	83.1	1.8083	1.9
4/23/2018 9:22	0	0.2	4.7	23.9	27	82	1.7951	1.9
4/23/2018 9:23	0	0	4.1	23.9	26.7	81.1	1.7718	1.9
4/23/2018 9:24	0	0.3	4.1	23.9	26.5	80.2	1.7569	1.9
4/23/2018 9:25	0.1	0.1	1.9	23.7	26.2	79.4	1.7423	1.9
4/23/2018 9:26	0.1	0.4	3.5	23.4	25.8	78.5	1.7116	1.9
4/23/2018 9:27	0.1	0.5	3.8	23.2	25.5	77.7	1.701	1.8
4/23/2018 9:28	0.1	0.4	3.1	22.9	25.3	77	1.6846	1.8
4/23/2018 9:29	0.2	0.5	2.8	22.7	25	76	1.6626	1.9
4/23/2018 9:30	0.2	0.1	3.6	22.6	24.7	75.2	1.6503	1.8
4/23/2018 9:31	0.2	-0.5	2.7	22.7	24.5	74.2	1.6273	1.8
4/23/2018 9:32	0.2	0.4	3.8	22.5	24.2	73.5	1.6355	1.8
4/23/2018 9:33	0.1	0.2	2.8	22.2	24	72.6	1.6104	1.9
4/23/2018 9:34	0.1	0.6	3.1	22	23.8	71.9	1.592	1.8
4/23/2018 9:35	0.1	-0.1	3.3	22.1	23.6	71.2	1.601	1.9
4/23/2018 9:36	0.1	0.9	2.9	22.1	23.4	70.4	1.5805	1.9
4/23/2018 9:37	0.2	-0.1	3.5	21.6	23.1	69.8	1.5647	1.9
4/23/2018 9:38	0.2	0.5	4	21.5	22.9	69	1.5238	1.9
4/23/2018 9:39	0.1	0.1	2.3	21.6	22.7	68.4	1.5452	1.9
4/23/2018 9:40	0.1	-0.4	3.5	21.2	22.5	67.6	1.5075	1.9
4/23/2018 9:41	0.2	0.4	3.7	20.9	22.1	66.9	1.4868	1.9
4/23/2018 9:42	0.2	0.4	2.1	21.3	22	66.3	1.4979	1.9
4/23/2018 9:43	0.2	1	3.2	21	21.9	65.7	1.4958	1.9
4/23/2018 9:44	0.1	0.3	2.4	20.9	21.7	65.1	1.4674	1.9
4/23/2018 9:45	0.1	0.3	3.4	20.8	21.5	64.3	1.4617	1.9
4/23/2018 9:46	0.1	0.3	1.6	20.9	21.4	64	1.4788	1.8

4/23/2018 9:47	0.1	0.6	2.8	20.6	21.1	63.5	1.4296	1.9
4/23/2018 9:48	0.1	-0.4	3.8	20.6	21	63	1.4428	1.9
4/23/2018 9:49	0.1	0.2	2.6	21.1	20.8	62.5	1.4167	1.9
4/23/2018 9:50	0.1	0.8	3.1	20.6	20.7	62.1	1.4319	1.9
4/23/2018 9:51	0.1	0.4	3.3	20.7	20.5	61.5	1.4107	1.9
4/23/2018 9:52	0.1	-0.1	3.1	20.2	20.4	61	1.4093	1.9
4/23/2018 9:53	0.1	0.3	2.7	20.1	20.2	60.5	1.3733	1.8
4/23/2018 9:54	0.1	0.6	2.7	20	20	59.7	1.3519	1.9
4/23/2018 9:55	0.2	0.9	3.4	20.2	19.8	59.2	1.3548	2
4/23/2018 9:56	0.2	0.5	3.8	19.9	19.6	58.6	1.3356	2
4/23/2018 9:57	0.2	0.2	3	20.2	19.4	58.1	1.3241	2
4/23/2018 9:58	0.2	0.6	3.3	20.4	19.4	57.5	1.3392	1.9
4/23/2018 9:59	0.2	0.9	3.7	19.7	19.3	57.1	1.3089	1.9
4/23/2018 10:00	0.1	0.3	3.8	19.8	19.1	56.4	1.3198	1.9
4/23/2018 10:01	0.1	0.6	4.1	19.7	19	55.7	1.2858	1.8
4/23/2018 10:02	0.2	-0.1	2.4	19.7	18.8	55.5	1.302	1.8
4/23/2018 10:03	0.2	0.4	2.4	19.8	18.6	55.1	1.2878	1.9
4/23/2018 10:04	0.2	0.2	3.9	19.7	18.5	54.7	1.2587	1.9
4/23/2018 10:05	0.2	0.9	3.7	19.7	18.3	54.2	1.2726	2
4/23/2018 10:06	0.2	0.5	3.4	19.6	18.3	53.8	1.2678	1.9
4/23/2018 10:07	0.2	0.1	3.4	19.6	18.1	53.1	1.2348	1.8
4/23/2018 10:08	0.2	0.9	3	19.4	17.5	52.5	1.1888	1.8
4/23/2018 10:09	0.2	0.5	2.1	18.5	17.2	51.4	1.1086	1.7
4/23/2018 10:10	0.2	0.4	2.8	18.5	16.9	41.4	1.0035	1.8
4/23/2018 10:11	0.3	0.5	3.1	18.2	16.8	38.4	0.9643	1.8
4/23/2018 10:12	0.2	0.5	3	18.1	16.6	34.5	0.9125	1.8
4/23/2018 10:13	0.3	0.8	2.2	18.5	16.5	37.9	0.945	1.9
4/23/2018 10:14	0.3	0.4	2.6	18.5	16.5	39.4	0.9616	1.9
4/23/2018 10:15	0.3	0.8	3.2	18.8	16.7	39.9	1.0971	1.9
4/23/2018 10:16	0.3	1	2.1	18.8	17.5	40.3	1.0918	2
4/23/2018 10:17	0.4	0.8	3.5	18.7	17.9	40.1	1.0162	2
4/23/2018 10:18	0.5	0.8	3.8	18.4	18.3	39.4	0.912	2
4/23/2018 10:19	0.6	1.3	5.1	18.6	18.5	38	0.8237	2
4/23/2018 10:20	0.7	1.4	3.6	18.3	18.7	36.3	0.7697	2
4/23/2018 10:21	0.9	1.7	1.8	18.2	18.8	34.7	0.7495	2
4/23/2018 10:22	0.9	1.2	3.7	18.2	19	32.5	0.7184	2
4/23/2018 10:23	1	1.5	3.3	18.3	18.9	29.6	0.6605	2
4/23/2018 10:24	1.1	0.5	3.2	18.4	18.8	26.1	0.6154	1.9
4/23/2018 10:25	1.3	2.2	4	18.4	18.7	23.5	0.5922	1.9
4/23/2018 10:26	1.5	2.3	2	18.3	18.6	20.6	0.5494	1.9
4/23/2018 10:27	1.7	1.9	4.1	18.7	18.3	18.2	0.5244	1.9
4/23/2018 10:28	1.9	2.4	1.7	18.5	18.3	16.5	0.5106	1.9
4/23/2018 10:29	2.1	2.6	2.9	18.9	18.1	14.7	0.495	1.9
4/23/2018 10:30	2.3	2.3	3.7	19	18	12.9	0.4767	2
4/23/2018 10:31	2.6	3.6	3	19.4	17.6	11.5	0.4347	2
4/23/2018 10:32	2.9	4.3	3.3	19.5	17.1	10.3	0.4289	1.9
4/23/2018 10:33	3.2	4.1	4.2	19.8	17	9.1	0.4097	1.9

4/23/2018 10:34	3.4	3.8	4	20	16.7	8	0.3974	1.9
4/23/2018 10:35	3.6	4.5	3	20.2	15.9	7.3	0.3777	1.9
4/23/2018 10:36	4	5.2	4.8	20.7	15.8	6.4	0.3564	1.8
4/23/2018 10:37	4.4	5.8	4.6	21.4	15.3	5.7	0.3486	1.8
4/23/2018 10:38	4.8	5.2	3.4	21.1	14	5.1	0.3241	1.9
4/23/2018 10:39	4.9	4.1	4.2	20.8	14.7	4.6	0.3415	1.9
4/23/2018 10:40	4.8	4.8	3.3	20.7	14.8	4.3	0.3332	1.9
4/23/2018 10:41	4.3	4.6	3.8	20.6	14.8	3.9	0.3403	1.9
4/23/2018 10:42	4.1	4	4.8	20.3	15	3.7	0.3375	1.9
4/23/2018 10:43	3.8	3.7	2.9	19.9	17	4.8	0.3462	1.9
4/23/2018 10:44	2.9	1	3.4	25.8	49.7	48.2	0.6328	2
4/23/2018 10:45	1.8	1.9	3.2	39.3	65.4	112.9	1.157	2
4/23/2018 10:46	0.7	0.4	4.3	44.1	70.8	131.8	1.4983	2.1
4/23/2018 10:47	0.3	0.4	3.8	68.2	75.4	140.3	2.051	2.3
4/23/2018 10:48	0.2	0.3	4.5	76.4	75.2	141	2.6096	2.5
4/23/2018 10:49	0.2	0	4.4	86.9	76.6	147.5	3.2941	2.6
4/23/2018 10:50	0.2	0	4.1	87.5	74.3	145.2	3.8215	2.7
4/23/2018 10:51	0.2	-0.2	5	88.7	73.5	145.2	4.191	2.8
4/23/2018 10:52	0.3	0.1	4.1	83.8	69.7	138.4	4.2958	2.8
4/23/2018 10:53	0.3	0	3.3	83.2	68.9	139.5	4.5625	2.8
4/23/2018 10:54	0.3	-0.7	4.5	80.1	67.6	137.5	4.6507	2.8
4/23/2018 10:55	0.2	0.1	5	79.1	65.7	134.9	4.8365	2.7
4/23/2018 10:56	0.2	0.5	3.7	76.5	65.1	135.5	4.9322	2.8
4/23/2018 10:57	0.2	0.3	5.5	74.9	63.2	130.9	4.9903	2.7
4/23/2018 10:58	0.2	0.5	3.7	73.4	62.5	132.6	5.0852	2.8
4/23/2018 10:59	0.2	0.3	3.7	70.4	60.7	129.3	5.0133	2.8
4/23/2018 11:00	0.2	0.5	5.4	68.6	59.4	127.4	5.0417	2.7
4/23/2018 11:01	0.2	-0.1	4.7	66	58.1	127.1	4.9335	2.7
4/23/2018 11:02	0.3	0.3	4.6	65.3	57	126.7	5.0045	2.7
4/23/2018 11:03	0.3	0.2	4.3	63.5	56.1	126.2	5.017	2.7
4/23/2018 11:04	0.3	1	5.5	61.4	54.4	119.2	4.8968	2.7
4/23/2018 11:05	0.2	0.7	4.6	61	54.3	120.4	5.0015	2.7
4/23/2018 11:06	0.2	0.1	3.6	59.7	53.5	120	4.9952	2.7
4/23/2018 11:07	0.2	0.1	4.8	58.5	52.7	118.6	4.9693	2.6
4/23/2018 11:08	0.2	0.9	4.4	57.8	51.9	118.4	4.9412	2.6
4/23/2018 11:09	0.2	0.5	5.3	56.5	51.5	118.3	4.9403	2.6
4/23/2018 11:10	0.1	0.7	5.5	54.7	50	118.1	4.8487	2.6
4/23/2018 11:11	0.1	0.4	3.9	53.2	49	116.7	4.788	2.5
4/23/2018 11:12	0.1	0.2	4.6	50.8	47.6	111.5	4.6763	2.5
4/23/2018 11:13	0.2	-0.1	4.7	50.3	46.2	107.6	4.5945	2.4
4/23/2018 11:14	0.2	-0.2	4	50	45.7	109.4	4.6468	2.5
4/23/2018 11:15	0.2	0.3	3.9	50.4	45.7	109.6	4.6398	2.4
4/23/2018 11:16	0.1	0.3	4.4	47.9	45.1	109.4	4.526	2.4
4/23/2018 11:17	0.1	0.5	5.2	47.8	44.2	109	4.5257	2.4
4/23/2018 11:18	0.1	0.7	4.8	46.5	43.1	108.5	4.433	2.4
4/23/2018 11:19	0.1	0.6	5	44.4	41.8	104.4	4.3198	2.4
4/23/2018 11:20	0.1	0.8	4	44.3	40.7	98.7	4.2638	2.3

4/23/2018 11:21	0.1	0.6	4.8	43.7	40.8	101.1	4.3397	2.3
4/23/2018 11:22	0.1	0.3	5.3	41.4	38.9	99	4.0757	2.2
4/23/2018 11:23	0.1	0.3	4.9	42.1	39	97.8	4.224	2.3
4/23/2018 11:24	0.2	0.8	3.6	40.6	38.3	96	4.0813	2.3
4/23/2018 11:25	0.2	0.9	2.9	41.7	38.3	97.7	4.2002	2.3
4/23/2018 11:26	0.2	1.1	2.7	40.7	38.2	98.3	4.1587	2.4
4/23/2018 11:27	0.2	0.9	5.2	39.9	37.4	95.9	4.1065	2.4
4/23/2018 11:28	0.2	-0.1	3.3	40.6	37.6	95.5	4.1827	2.3
4/23/2018 11:29	0.2	0.8	4.8	39.2	36.8	95.6	4.0898	2.4
4/23/2018 11:30	0.2	0.4	4.8	37.6	35	94.9	3.9135	2.4
4/23/2018 11:31	0.2	0.2	4	38.4	35.3	94.6	4.0207	2.5
4/23/2018 11:32	0.2	0.5	4.3	36.3	34.3	93.2	3.8878	2.4
4/23/2018 11:33	0.3	0.4	3.3	35.4	32.8	86	3.7215	2.5
4/23/2018 11:34	0.3	1	3	36.3	33.3	90.3	3.8395	2.5
4/23/2018 11:35	0.3	0.4	4.1	35.8	32.9	87.6	3.8223	2.5
4/23/2018 11:36	0.3	0.1	2.9	35.4	32.3	85.7	3.7835	2.5
4/23/2018 11:37	0.3	-0.1	4.3	35	31.8	85.8	3.7653	2.4
4/23/2018 11:38	0.3	0.4	3.7	34.4	31.2	85.7	3.7058	2.5
4/23/2018 11:39	0.3	0.4	3.3	33.9	31.1	85.7	3.715	2.4
4/23/2018 11:40	0.2	0.7	2.5	33.4	30.3	84.4	3.6402	2.5
4/23/2018 11:41	0.3	0.8	4.4	33.8	29.9	83.3	3.6618	2.5
4/23/2018 11:42	0.3	0.5	4.1	32.4	29.5	81.3	3.5923	2.5
4/23/2018 11:43	0.3	0.7	5.2	31.8	28.7	76.8	3.4713	2.4
4/23/2018 11:44	0.3	0.7	2.6	31.8	28.3	75.7	3.4877	2.3
4/23/2018 11:45	0.3	0.6		31.9	28.6	76.3	3.519	2.3
4/23/2018 11:46	0.4	0.5	4.8	32.3	28.3	76.9	3.5647	2.2
4/23/2018 11:47	0.3	0.8		31.9	28.4	77.6	3.5712	2.2
4/23/2018 11:48	0.3	0.4		31	27.7	78.2	3.505	2.2
4/23/2018 11:49	0.3	1.3	3.8	30.7	27.1	78.4	3.4398	2.2
4/23/2018 11:50	0.3	0.1	3.5	30.6	26.8	78.7	3.4105	2.3
4/23/2018 11:51	0.3	0.3	4.3	30.3	26.2	78.7	3.3482	2.3
4/23/2018 11:52	0.3	0.4		28.9	25.3	70.5	3.1815	2.3
4/23/2018 11:53	0.3	0.4	4.2	29.9	25.7	73.5	3.343	2.3
4/23/2018 11:54	0.3	0.7	4.3	29.3	25.3	72.8	3.2815	2.3
4/23/2018 11:55	0.3	0.3	3.3	28.2	24.5	72.1	3.1852	2.2
4/23/2018 11:56	0.3	0.9	4.8	28.5	24	71.6	3.1002	2.1
4/23/2018 11:57	0.3	0.1	4.2	28.4	24.3	71.4	3.18	2
4/23/2018 11:58	0.3	0.4		28	23.8	71.2	3.1327	2
4/23/2018 11:59	0.3	0.8		27.1	23	70.6	3.021	2
4/23/2018 12:00	0.2	0.6		27.9	23.1	70.1	3.1192	2
4/23/2018 12:01	0.2	-0.2	5.1	27.9	23.3	69.9	3.1458	2
4/23/2018 12:02	0.2	-0.2		26.2	22.5	69.2	3.0157	2
4/23/2018 12:03	0.1	1.2	4.2	25.7	21.1	68	2.8402	2
4/23/2018 12:04	0.2	0.6	4.4	24.5	20.1	58.5	2.6037	1.9
4/23/2018 12:05	0.2	0.4	4.6	22	19.1	51.8	2.3463	1.9
4/23/2018 12:06	0.4	1	4.6	19.9	16.6	34.9	1.8527	1.8
4/23/2018 12:07	0.5	0.8	3.5	19.3	15.8	31.3	1.6755	1.9

4/23/2018 12:08	0.6	1.1	4	19	15.6	30.8	1.6203	1.9
4/23/2018 12:09	0.6	1.3	3.9	19.7	15	27.2	1.6613	1.9
4/23/2018 12:10	1	3.6	5	20.6	16.2	30.4	1.8518	1.9
4/23/2018 12:11	2.4	7.2	5.3	22.4	14.2	14	1.1713	1.8
4/23/2018 12:12	4.7	9.9	6.1	24	12.4	8.1	0.8439	1.7
4/23/2018 12:13	7.1	10.9	5.9	25.6	11.4	5.6	0.6697	1.6
4/23/2018 12:14	8.6	11.9	7.5	26.7	10.7	4.8	0.6252	1.6
4/23/2018 12:15	9.9	14.4	7.9	28.2	10.1	4.7	0.5198	1.6
4/23/2018 12:16	11.1	14.8	7.2	28.8	10	4.6	0.5338	1.6
4/23/2018 12:17	12	14.7	8.2	28.9	9.8	4.5	0.5108	1.6
4/23/2018 12:18	12.2	14.1	8.8	28.9	9.4	4.5	0.5466	1.6
4/23/2018 12:19	12.3	14.3	7.3	28.8	9.1	4.4	0.5327	1.6
4/23/2018 12:20	12.3	14.4	8.3	29.2	9.3	4.4	0.4931	1.6
4/23/2018 12:21	12.7	15.6	7.6	29.2	9.5	4.2	0.4603	1.6
4/23/2018 12:22	13	14.7	8.6	29.4	9.2	4.1	0.4537	1.6
4/23/2018 12:23	13.3	15.3	6.3	29.8	8.9	3.9	0.4767	1.6
4/23/2018 12:24	13.5	16	9.2	30.3	8.4	3.8	0.4342	1.6
4/23/2018 12:25	14.1	16.9	8.9	31.1	7.7	3.7	0.4052	1.6
4/23/2018 12:26	14.9	17.8	8.1	32	7.4	3.5	0.395	1.5
4/23/2018 12:27	15.9	19	9.6	32.9	6.8	3.2	0.3618	1.5
4/23/2018 12:28	16.6	18.8	9.3	32.7	6.5	2.9	0.3674	1.6
4/23/2018 12:29	16.8	20.2	8.8	33.5	6.7	2.8	0.3724	1.6
4/23/2018 12:30	17.2	19.8	11.3	33.6	6.5	2.6	0.3384	1.5
4/23/2018 12:31	17.7	20.7	10.4	34.9	6.1	2.4	0.308	1.5
4/23/2018 12:32	18.6	22	10.7	35.1	5.7	2.2	0.2844	1.5
4/23/2018 12:33	19.6	23.5	11.3	36	5.2	2	0.2772	1.5
4/23/2018 12:34	20.3	23.4	10	36.4	4.9	1.9	0.2586	1.5
4/23/2018 12:35	20.4	22	10.7	35.2	5.3	1.7	0.287	1.5
4/23/2018 12:36	20.1	21.9	11.8	34.9	5.7	1.7	0.2941	1.4
4/23/2018 12:37	19.4	21.4	11.1	34.8	5.6	1.6	0.2792	1.4
4/23/2018 12:38	19.3	22.7	10.4	35.4	5.5	1.5	0.2785	1.5
4/23/2018 12:39	20	24.2	11.8	36.8	4.9	1.4	0.2429	1.5
4/23/2018 12:40	21	24.3	12.5	37.4	4.4	1.3	0.2358	1.5
4/23/2018 12:41	22	24.5	13.1	37.8	4.1	1.3	0.24	1.5
4/23/2018 12:42	22.5	24.2	13.3	37.7	4	1.3	0.2275	1.6
4/23/2018 12:43	22.7	25	12.4	37.9	3.8	1.2	0.2385	1.6
4/23/2018 12:44	23.1	27	14.1	39.6	3.4	1.2	0.2053	1.7
4/23/2018 12:45	23.7	26	11.6	39.2	3.3	1.1	0.2122	1.6
4/23/2018 12:46	24.3	26	12.6	39.4	3.1	1	0.2031	1.7
4/23/2018 12:47	24.2	25.8	10.7	38.9	3.3	0.9	0.2039	1.7
4/23/2018 12:48	24	25.5	12.2	38.8	3.5	0.9	0.2032	1.8
4/23/2018 12:49	23.9	26.3	13.3	39	3.4	0.9	0.1929	1.8
4/23/2018 12:50	23.9	25.8	13.1	39	3.1	0.8	0.2063	1.8
4/23/2018 12:51	23.9	26.4	13.6	39.3	3.2	0.8	0.207	1.8
4/23/2018 12:52	24.2	26	11.8	39.1	3	0.8	0.2005	1.7
4/23/2018 12:53	23.1	21.1	9.1	35.1	4.2	0.8	0.2642	1.7
4/23/2018 12:54	21.2	19.3	9.5	33.8	4.9	0.8	0.2765	1.7

4/23/2018 12:55	19	19.8	9.1	33.7	4.6	0.9	0.2695	1.7
4/23/2018 12:56	17.9	17.1	9.4	36	4.4	1	0.2708	1.6
4/23/2018 12:57	17.9	21	12.6	31	4.2	1.1	0.2498	1.6
4/23/2018 12:58	18.2	20	12.9	33	4.3	1.1	0.2603	1.5
4/23/2018 12:59	18.6	20.3	12.6	33.2	4.2	1.2	0.2425	1.6
4/23/2018 13:00	18.3	18.9	12.7	32.5	4.3	1.3	0.2576	1.6
4/23/2018 13:01	17.7	17.9	11.1	32.2	4.4	1.3	0.2577	1.7
4/23/2018 13:02	17	17.2	12.3	31.9	4.5	1.4	0.2621	1.7
4/23/2018 13:03	16.4	17.5	10.2	31.5	4.5	1.4	0.2726	1.7
4/23/2018 13:04	15.9	16.6	10	30.6	4.5	1.4	0.2661	1.7
4/23/2018 13:05	15.4	15.4	10.6	30.1	4.6	1.4	0.2816	1.7
4/23/2018 13:06	13.9	10.1	7.6	26.7	14.3	15.3	0.3277	1.7
4/23/2018 13:07	10.9	6	4.8	27.2	33.4	34.6	0.5305	1.7
4/23/2018 13:08	6.7	2.4	3.2	29.8	46.8	50.8	0.8197	1.9
4/23/2018 13:09	3.1	0.5	5	39.4	60.5	66.8	1.275	2
4/23/2018 13:10	1.1	0.6	3.6	43.8	63.1	73.4	1.4872	2
4/23/2018 13:11	0.5	0.6	4.5	50.4	66.1	78.2	1.7472	2
4/23/2018 13:12	0.3	0.4	3.8	53.8	68	79.9	1.9473	1.9
4/23/2018 13:13	0.3	0.7	4.9	56.3	68.4	79.3	2.1132	1.8
4/23/2018 13:14	0.3	1.2	6.2	58.7	68.3	79.4	2.3247	1.9
4/23/2018 13:15	0.3	0.4	5.9	60.5	70.3	79.8	2.5993	1.9
4/23/2018 13:16	0.3	0	5.4	61.9	70.2	80.3	2.9118	2
4/23/2018 13:17	0.3	0.5	6.3	62	70.2	80.7	3.2268	2
4/23/2018 13:18	0.3	1.4	6.2	60.6	69.5	80.9	3.3775	2
4/23/2018 13:19	0.3	0.7	7.3	61.2	66	81	3.5421	2.1
4/23/2018 13:20	0.3	1.2	4.4	60.9	65.3	81.1	3.7715	2.1
4/23/2018 13:21	0.3	0.4	6.3	58.4	63.6	81.1	3.8244	2.1
4/23/2018 13:22	0.3	0	4.5	56.6	61.2	76.6	3.9758	2.1
4/23/2018 13:23	0.3	0.1	4.7	56.8	60.4	75.9	4.1522	2.2
4/23/2018 13:24	0.3	-0.2	5.3	55.1	59.9	74	4.2242	2.2
4/23/2018 13:25	0.3	0.6	6.5	53.9	58.3	73.9	4.2905	2.2
4/23/2018 13:26	0.3	0.2	6.3	53	57.7	73.8	4.4578	2.2
4/23/2018 13:27	0.3	0.3	5.3	51.1	56.8	73.5	4.5497	2.3
4/23/2018 13:28	0.3	0.2	5.2	50	55.4	73.2	4.5848	2.3
4/23/2018 13:29	0.3	0.2	4.7	48.9	54.6	72.7	4.5881	2.4
4/23/2018 13:30	0.3	0	5.8	47	53	72	4.5097	2.3
4/23/2018 13:31	0.3	0.4	6.3	45.6	51.7	71.1	4.5343	2.3
4/23/2018 13:32	0.4	0.8	5.1	46.1	50.8	70.2	4.5223	2.3
4/23/2018 13:33	0.3	0.1	5.7	45.2	51.3	69.3	4.6268	2.4
4/23/2018 13:34	0.4	0.1	5.4	43.3	49.7	68.2	4.4704	2.4
4/23/2018 13:35	0.4	0.2	6.1	43.2	49.1	67.2	4.4863	2.3
4/23/2018 13:36	0.4	0.5	2.5	41.9	48.3	66.3	4.4668	2.3
4/23/2018 13:37	0.4	0.5	5.7	40.9	46.9	63	4.3438	2.3
4/23/2018 13:38	0.4	1.3	5.4	40.4	46.5	60.6	4.3625	2.3
4/23/2018 13:39	0.4	0.4	3.7	39.8	45.7	60.4	4.3132	2.3
4/23/2018 13:40	0.4	0.6	5.1	39.9	45.1	60	4.3126	2.2
4/23/2018 13:41	0.4	1	3.7	38.7	45.1	60.6	4.3059	2.1

4/23/2018 13:42	0.4	0.1	6.9	38.5	44.1	60.4	4.2501	2.1
4/23/2018 13:43	0.4	0.3	4.7	37.3	43.6	60.1	4.2192	2
4/23/2018 13:44	0.3	0.8	3.6	36.1	42.4	59.6	4.0808	2
4/23/2018 13:45	0.4	0.9	4.4	35.3	40.8	54.7	3.9559	2
4/23/2018 13:46	0.4	0.3	3.7	36.2	41.3	55.5	4.072	2.1
4/23/2018 13:47	0.4	1	5.2	34.5	40.3	53.2	3.9298	2.1
4/23/2018 13:48	0.5	0.8	4.8	33.3	38.9	51.5	3.8232	2.2
4/23/2018 13:49	0.5	0.2	5	32.9	37.2	50.8	3.7081	2.1
4/23/2018 13:50	0.4	0.8	5.4	33.3	38.6	50.9	3.8368	2.1
4/23/2018 13:51	0.4	0.8	2.7	32.7	38	50.9	3.7836	2.1
4/23/2018 13:52	0.4	0.6	4.1	32.3	37.1	50.8	3.7041	2.1
4/23/2018 13:53	0.3	1.2	3.7	32	36.9	50.6	3.7238	2
4/23/2018 13:54	0.3	0.9	4.4	31.8	36.2	50.4	3.6543	2
4/23/2018 13:55	0.3	1.1	3.7	31.8	36	50.2	3.639	2
4/23/2018 13:56	0.3	0.7	4	30.7	35.8	49.9	3.6204	2
4/23/2018 13:57	0.4	1	5.7	29.4	34.1	48.1	3.4484	2
4/23/2018 13:58	0.4	0.4	5.2	29.3	33.5	44.9	3.4111	2
4/23/2018 13:59	0.5	1.3	3.7	29.6	32.7	44	3.3808	2
4/23/2018 14:00	0.5	0.6	3.9	29	33.1	43.9	3.3754	2.2
4/23/2018 14:01	0.5	-0.1	5.3	29.5	32.7	42.4	3.4109	2.2
4/23/2018 14:02	0.5	0.2	3.6	28.9	32.5	42.6	3.3672	2.1
4/23/2018 14:03	0.5	-0.1	3.2	28.7	32.1	42.7	3.3426	2
4/23/2018 14:04	0.4	0.8	3.4	28.2	31.5	42.7	3.2807	2.1
4/23/2018 14:05	0.4	0.8	4.8	27.7	31.3	42.6	3.2592	2.1
4/23/2018 14:06	0.5	1	3.5	27.1	29.8	42.1	3.1166	2.1
4/23/2018 14:07	0.5	0.5	3.5	27.7	30.5	42	3.201	2.1
4/23/2018 14:08	0.5	1	3.6	26.5	29.5	41.6	3.0866	2
4/23/2018 14:09	0.4	0.7	4.7	27.1	29.5	41.2	3.1286	2
4/23/2018 14:10	0.4	1.1	3	26.2	29	40.6	3.0588	2.1
4/23/2018 14:11	0.4	1.3	3.4	26.1	28.9	40	3.0567	2.1
4/23/2018 14:12	0.5	1	4.2	25.8	27.9	39.3	2.9663	2.1
4/23/2018 14:13	0.4	1	3.1	26.2	28.2	38.7	3.0188	2.1
4/23/2018 14:14	0.4	1.2	3.1	25.4	27.5	38.4	2.9294	2.1
4/23/2018 14:15	0.5	0.7	4.1	24.9	26.7	37.9	2.8472	2.1
4/23/2018 14:16	0.5	0.8	4.4	25.5	26.8	37.4	2.8839	2.2
4/23/2018 14:17	0.5	0.9	4.4	25	26.2	37	2.8083	2.1
4/23/2018 14:18	0.6	1	5.5	24.2	26.1	36.2	2.6689	2.2
4/23/2018 14:19	0.6	0.8	3.2	24.3	25.9	35.5	2.7606	2.2
4/23/2018 14:20	0.6	1.4	2.6	24	25.1	34.9	2.6982	2.2
4/23/2018 14:21	0.5	0.6	3.1	24.3	25.2	34.5	2.7392	2
4/23/2018 14:22	0.5	1.2	3.4	23.2	24.2	33.8	2.589	2
4/23/2018 14:23	0.6	1.7	4	23.6	23.1	32.9	2.4868	2
4/23/2018 14:24	0.6	0.5	4.4	23.4	23.8	32.4	2.5869	2.1
4/23/2018 14:25	0.7	1.2	3.5	22.5	22.1	31.4	2.3373	2.2
4/23/2018 14:26	0.7	1.3	3.4	22.3	21.7	30.5	2.3803	2.1
4/23/2018 14:27	0.8	1.2	2.1	21.3	20.7	29.4	2.2083	2.1
4/23/2018 14:28	1	2.3	4.8	21.3	19.5	28.3	2.0849	2.1

4/23/2018 14:29	1	1.3	3.9	22.3	21.1	27.4	2.2813	2.1
4/23/2018 14:30	1.1	2	4.5	20.8	19.7	26.1	2.0322	2
4/23/2018 14:31	1.1	1.2	3.6	20.6	19	25.3	1.9195	2
4/23/2018 14:32	1.1	1.4	3.3	20.1	19.1	24	1.9384	2.1
4/23/2018 14:33	1.1	2.5	3.8	19.9	17.8	22.7	1.7798	2.1
4/23/2018 14:34	1.4	2.7	4.4	19.7	15.5	15.8	1.5072	2.1
4/23/2018 14:35	1.8	2.9	4.2	19.6	16	14.4	1.5698	2
4/23/2018 14:36	3.1	9.7	6.8	23.2	13.5	11.5	1.2619	2
4/23/2018 14:37	6.6	15.9	11.6	28.1	9.1	4.5	0.7375	1.9
4/23/2018 14:38	11.1	18.7	10.6	30.8	7.4	3.9	0.5616	1.9
4/23/2018 14:39	15.4	21.7	13.7	33.1	6.3	3.6	0.4415	2
4/23/2018 14:40	17.6	21.4	13.6	33.8	5.3	3.3	0.413	2
4/23/2018 14:41	19	22.7	13.8	34.9	5.1	3	0.3686	2
4/23/2018 14:42	19.8	22.4	12.3	34.7	5.1	2.7	0.3953	2
4/23/2018 14:43	19.9	21.5	12.8	34.2	5.6	2.5	0.4137	2
4/23/2018 14:44	19.9	21.7	11	34.2	5.7	2.2	0.4171	1.9
4/23/2018 14:45	19.7	23.2	13.6	34.8	5.7	1.9	0.3824	1.8
4/23/2018 14:46	20.1	23.2	14.4	34.9	5.6	1.7	0.326	1.8
4/23/2018 14:47	20.9	23.8	14.4	36.1	5.4	1.5	0.32	1.7
4/23/2018 14:48	21.7	23.7	15.4	36.3	5.2	1.5	0.3228	1.6
4/23/2018 14:49	22	23.4	14.1	35.9	5	1.5	0.3336	1.6
4/23/2018 14:50	20.9	18.6	11.8	31.9	6.6	1.4	0.4257	1.7
4/23/2018 14:51	18.3	14.5	10.1	29.2	9.9	1.5	0.6388	1.9
4/23/2018 14:52	15.4	13.6	9.1	28.4	10.6	1.6	0.6523	2
4/23/2018 14:53	13.1	12.9	8.8	27.5	11.1	1.6	0.6879	2.1
4/23/2018 14:54	12.2	11.9	7.5	27	11.7	1.8	0.7385	2.1
4/23/2018 14:55	11.4	11.8	7.4	26.4	11.8	1.9	0.731	2.1
4/23/2018 14:56	10.6	10.8	8.6	25.8	11.7	2	0.7451	2.2
4/23/2018 14:57	9.8	9.3	7.2	24.4	12.6	3.1	0.7454	2.2
4/23/2018 14:58	7.9	3	5.2	22.2	39.1	38.9	0.911	2.2
4/23/2018 14:59	5	1.7	4.9	35.9	53.8	70	1.3548	2.2
4/23/2018 15:00	2.1	0.5	4.9	58.6	64.6	103.5	2.2398	2.4
4/23/2018 15:01	0.5	1.3	6.5	70.4	64.4	121.9	2.7005	2.5
4/23/2018 15:02	0.2	-0.2	5.8	76.6	65.1	126.3	3.0515	2.5
4/23/2018 15:03	0.2	0.6	6.7	88.6	65.6	128.4	3.5925	2.6
4/23/2018 15:04	0.2	-0.3	6.8	89.9	63.8	129.4	3.8613	2.7
4/23/2018 15:05	0.2	0.6		89.9	62.6	126.1	4.1377	2.8
4/23/2018 15:06	0.2	0.5		89	59.7	119.8	4.1718	2.8
4/23/2018 15:07	0.2	-0.9	7.1	87.6	59.3	120.2	4.358	2.8
4/23/2018 15:08	0.3	0.3		85.4	57.8	118	4.3478	2.8
4/23/2018 15:09	0.2	0.1	6.6	83	57	117.7	4.3978	2.7
4/23/2018 15:10	0.2	0.5	5.1	79.1	54.5	109.1	4.2922	2.6
4/23/2018 15:11	0.3	0.4	6.1	77.3	54.4	111.1	4.3575	2.6
4/23/2018 15:12	0.3	0.3	6.8	74.7	52.5	106.6	4.2832	2.5
4/23/2018 15:13	0.3	0.2	5.9	73.3	52.2	107.6	4.3443	2.5
4/23/2018 15:14	0.3	0.4		69.3	49.8	106.9	4.1833	2.5
4/23/2018 15:15	0.3	0.1	6.7	69.1	49.7	106.1	4.2508	2.4

4/23/2018 15:16	0.4	1.3	4.6	64.2	47.8	98.8	4.0715	2.4
4/23/2018 15:17	0.3	0.9		65.8	47.5	97.7	4.1585	2.4
4/23/2018 15:18	0.3	0		63.1	47.4	97.4	4.127	2.4
4/23/2018 15:19	0.3	0.4	5.3	59.8	47	94.4	4.0033	2.5
4/23/2018 15:20	0.3	0.6	5	61.4	46.9	94.5	4.0432	2.5
4/23/2018 15:21	0.3	0.3		56.6	44.4	93.3	3.9465	2.5
4/23/2018 15:22	0.3	0.8		57.5	42.9	90.9	3.8967	2.4
4/23/2018 15:23	0.3	0.9		56.6	43.6	91.1	3.9658	2.4
4/23/2018 15:24	0.2	0.6	3.9	54.4	42.4	89.2	3.8728	2.4
4/23/2018 15:25	0.2	0.4	4.3	52.2	41.2	88.9	3.7673	2.4
4/23/2018 15:26	0.3	0.8	5.9	50	39.3	87.9	3.5997	2.4
4/23/2018 15:27	0.3	1		49	39.3	84.2	3.6073	2.5
4/23/2018 15:28	0.3	0.1	3.9	49.6	38.5	79.7	3.6353	2.5
4/23/2018 15:29	0.3	0.9	5.5	47.9	38.5	81.4	3.5893	2.5
4/23/2018 15:30	0.3	1.1	9	46.4	37.3	78.4	3.5415	2.5
4/23/2018 15:31	0.3	0.6	6.1	45.9	37.2	79.2	3.5375	2.4
4/23/2018 15:32	0.3	0.6	4.7	46.2	37.1	79.2	3.5722	2.3
4/23/2018 15:33	0.3	0.4	4.4	42.5	35.2	74.1	3.3173	2.3
4/23/2018 15:34	0.3	0.6	5	42.4	34.5	73.4	3.3165	2.3
4/23/2018 15:35	0.4	0.8	4.8	41.4	33.8	71.7	3.2673	2.3
4/23/2018 15:36	0.3	1.3	5.5	42.8	34.4	73.6	3.3703	2.1
4/23/2018 15:37	0.3	1.2	5.6	41.1	34.2	71.6	3.3543	2.1
4/23/2018 15:38	0.3	0.4	4.7	36.6	31.4	66.1	3.0202	2
4/23/2018 15:39	0.4	1.1	4.6	35.3	29.1	57.7	2.8555	2
4/23/2018 15:40	0.5	1	4.6	34.3	29.1	57.4	2.852	1.9
4/23/2018 15:41	0.5	0.6	3.7	34.4	28.9	59.8	2.9078	2
4/23/2018 15:42	0.4	0.6	5.4	32.6	28.1	60	2.743	2
4/23/2018 15:43	0.4	1.3	5	31.2	26.7	54.6	2.588	2
4/23/2018 15:44	0.6	1.3	4.7	30.8	26.1	52.4	2.5642	2
4/23/2018 15:45	0.5	1.1	4.6	32.1	27	55.7	2.7568	2
4/23/2018 15:46	0.5	1.4	3.3	32.2	27.3	58	2.796	2
4/23/2018 15:47	0.3	0.6	5	34	27.5	58	2.8335	2
4/23/2018 15:48	0.3	1	3.7	36.1	29.7	58.7	3.0765	2.1
4/23/2018 15:49	0.2	0.6	4.6	35.8	29.5	59.5	3.0527	2.1
4/23/2018 15:50	0.2	0.6	4	35	29	60.4	3.0212	2.2
4/23/2018 15:51	0.2	0.3	3.7	34.7	28.6	61.1	3.034	2.2
4/23/2018 15:52	0.2	1	4.7	34.8	28.4	61.7	2.9948	2.2
4/23/2018 15:53	0.2	0.1	4.3	34.2	28.1	62.3	3.0088	2.2
4/23/2018 15:54	0.2	0.6	4.5	33.3	27.8	62.8	2.9462	2.2
4/23/2018 15:55	0.2	0.6	4.1	32.7	27.1	63	2.8767	2.2
4/23/2018 15:56	0.3	0.6	2.9	30.5	25.7	59.2	2.6938	2.2
4/23/2018 15:57	0.3	0.4	4.1	32.2	26.2	57.7	2.8187	2.2
4/23/2018 15:58	0.3	1.1	4	30.3	25.8	56.4	2.7247	2.2
4/23/2018 15:59	0.3	0.9	5.3	29.1	24.5	52.1	2.5788	2.1
4/23/2018 16:00	0.4	0.7	4.9	26.9	23.2	50.6	2.3947	2.1
4/23/2018 16:01	0.5	1.5	4.6	26.5	22.3	50	2.306	2.1
4/23/2018 16:02	0.6	1.6	3.5	25.9	21.7	49.2	2.2562	2.1

4/23/2018 16:03	0.7	1.4	3.7	26	22.1	48.7	2.2793	2.1
4/23/2018 16:04	0.7	1.7	4.4	24.9	20.5	47.4	2.1025	2.1
4/23/2018 16:05	0.8	1.6	3.5	25.9	20.7	46	2.1522	2.1
4/23/2018 16:06	0.8	1.1	4.2	28.1	22.7	45.5	2.463	2.2
4/23/2018 16:07	0.6	0.3	6.3	29.2	23.7	45.5	2.5945	2.2
4/23/2018 16:08	0.4	1.1	3.2	28.1	23.4	45.6	2.5213	2.2
4/23/2018 16:09	0.3	0.4	2.8	27.6	22.8	45.9	2.4935	2.3
4/23/2018 16:10	0.3	0.5	5.3	27.6	22.6	46.4	2.4613	2.3
4/23/2018 16:11	0.3	0.8	4.3	28	22.6	46.8	2.5092	2.3
4/23/2018 16:12	0.3	1	3.4	26.9	22.2	47.6	2.4202	2.3
4/23/2018 16:13	0.4	1.1	4.5	24.1	20.4	41.8	2.1108	2.3
4/23/2018 16:14	0.6	1.1	3.6	21.9	18.7	32	1.7573	2.3
4/23/2018 16:15	0.7	1	3.6	21.8	18.6	32.5	1.8018	2.2
4/23/2018 16:16	0.9	1.2	5	19.3	17.6	26	1.5123	2.1
4/23/2018 16:17	1	1.5	3.8	19.5	17.1	22.7	1.4433	2
4/23/2018 16:18	1.1	1.5	3.9	20	16.8	22.9	1.4408	2.1
4/23/2018 16:19	1.1	1.5	2.7	19.8	17	23.2	1.5042	2.2
4/23/2018 16:20	1.2	2.2	4.9	19.5	16.9	23.2	1.3655	2.2
4/23/2018 16:21	2.4	7	7.5	20.1	16.9	22.4	0.9529	2.1
4/23/2018 16:22	4.3	9.3	5.7	21.5	17.1	15	0.6849	1.9
4/23/2018 16:23	6.9	13.1	8.6	23.9	15	6	0.4852	1.8
4/23/2018 16:24	9	13.7	9.3	25.5	12	3.7	0.4112	1.7
4/23/2018 16:25	10.9	14.7	10.1	27	10.6	2.8	0.3843	1.6
4/23/2018 16:26	12.3	16.8	10.6	27.8	9.1	2.5	0.3494	1.6
4/23/2018 16:27	12.9	15.5	9.6	28.6	8.2	2.4	0.338	1.7
4/23/2018 16:28	12.1	9.9	10.9	29.2	7.7	2.4	0.3252	1.8
4/23/2018 16:29					7.5	2.3	0.3122	1.8
4/23/2018 16:30					8	2.3	0.3096	1.9
4/23/2018 16:31					8.7	2.2	0.3254	2
4/23/2018 16:32					9.5	2.3	0.3608	2
4/23/2018 16:33					10.4	2.3	0.3446	2.1
4/23/2018 16:34					11.5	2.3	0.4017	2.1
4/23/2018 16:35					10.6	2.4	0.3866	2.1
4/23/2018 16:36					9.4	2.4	0.3531	2.1
4/23/2018 16:37					8.3	2.4	0.3237	2
4/23/2018 16:38					7.3	2.3	0.29	2
4/23/2018 16:39					6.6	2.3	0.2781	2
4/23/2018 16:40					6.1	2.1	0.2566	2
4/23/2018 16:41					5.9	2	0.2558	2
4/23/2018 16:42					5.8	1.8	0.2422	2
4/23/2018 16:43					5.7	1.6	0.2481	2
4/23/2018 16:44					5.7	1.4	0.2539	2
4/23/2018 16:45					6.1	1.3	0.2489	2
4/23/2018 16:46					6	1.3	0.2428	2.1
4/23/2018 16:47					5.5	1.2	0.219	2.2
4/23/2018 16:48					5.6	1.2	0.2468	2.2
4/23/2018 16:49					6.1	1.2	0.259	2.2

4/23/2018 16:50	6.5	1.2	0.2771	2.3
4/23/2018 16:51	7.6	1.3	0.3718	2.3
4/23/2018 16:52	8.1	1.5	0.3864	2.5
4/23/2018 16:53	7.9	1.6	0.3774	2.6
4/23/2018 16:54	8.1	1.8	0.3733	2.6
4/23/2018 16:55	8	1.9	0.378	2.6
4/23/2018 16:56	8.3	2	0.3646	2.6
4/23/2018 16:57	9	2.1	0.3994	2.6
4/23/2018 16:58	8.8	2.2	0.3979	2.6
4/23/2018 16:59	8.9	2.3	0.4049	2.7
4/23/2018 17:00	8.7	2.2	0.3995	2.7
4/23/2018 17:01	8.4	2.2	0.3833	2.7
4/23/2018 17:02	8.2	2.2	0.3933	2.7
4/23/2018 17:03	8.1	2.2	0.382	2.7
4/23/2018 17:04	8	2.3	0.3831	2.8
4/23/2018 17:05	8	2.3	0.3695	2.7
4/23/2018 17:06	8	2.3	0.3959	2.1
4/23/2018 17:07	7.5	2.4	0.3765	2.1
4/23/2018 17:08	7.2	2.4	0.3753	2.5
4/23/2018 17:09	7.5	2.6	0.4195	2.7
4/23/2018 17:10	7.5	2.7	0.414	2.8
4/23/2018 17:11	7.5	2.8	0.4189	2.8
4/23/2018 17:12	7.4	2.9	0.4191	2.8
4/23/2018 17:13	7.5	3.1	0.4665	2.9
4/23/2018 17:14	7.4	3.3	0.4178	2.9
4/23/2018 17:15	7.2	3.4	0.4134	2.7
4/23/2018 17:16	7	3.4	0.4067	2.6
4/23/2018 17:17	7.1	3.5	0.4184	2.7
4/23/2018 17:18	7	3.5	0.3928	2.7
4/23/2018 17:19	6.9	3.4	0.3831	2.4
4/23/2018 17:20	7.1	3.4	0.4098	2.4
4/23/2018 17:21	7	3.4	0.3685	2.4
4/23/2018 17:22	7	3.3	0.3812	2.3
4/23/2018 17:23	6.5	3.2	0.3676	2.2
4/23/2018 17:24	6.2	3.2	0.3627	2.2
4/23/2018 17:25	6.1	3.1	0.3471	2.1
4/23/2018 17:26	6	3.1	0.3519	2
4/23/2018 17:27	6	3.1	0.3453	2
4/23/2018 17:28	5.9	3	0.3271	2.1
4/23/2018 17:29	5.8	3	0.328	2.2
4/23/2018 17:30	5.7	2.9	0.311	2.2
4/23/2018 17:31	5.7	2.9	0.3278	2.3
4/23/2018 17:32	5.6	2.8	0.3129	2.3
4/23/2018 17:33	5.6	2.8	0.3098	2.2
4/23/2018 17:34	5.5	2.8	0.3018	2.3
4/23/2018 17:35	6.2	2.8	0.3566	2.3
4/23/2018 17:36	6.9	2.8	0.378	2.3

4/23/2018 17:37	6.7	2.7	0.3464	2.2
4/23/2018 17:38	6.5	2.7	0.3436	2.2
4/23/2018 17:39	6.5	2.7	0.3363	2.1
4/23/2018 17:40	6.5	2.7	0.3575	2.1
4/23/2018 17:41	6.5	2.7	0.3254	2
4/23/2018 17:42	6.2	2.7	0.3275	2.1
4/23/2018 17:43	6	2.7	0.311	2.2
4/23/2018 17:44	5.5	2.6	0.2706	2.1
4/23/2018 17:45	5.3	2.6	0.2845	2.2
4/23/2018 17:46	5.3	2.5	0.2571	2.2
4/23/2018 17:47	5.1	2.5	0.2702	2.2
4/23/2018 17:48	5	2.5	0.2593	2.2
4/23/2018 17:49	5	2.4	0.2514	2.3
4/23/2018 17:50	4.9	2.3	0.2455	2.4
4/23/2018 17:51	4.9	2.3	0.2554	2.5
4/23/2018 17:52	4.9	2.3	0.2457	2.5
4/23/2018 17:53	5.3	2.3	0.2842	2.5
4/23/2018 17:54	6.1	2.3	0.3262	2.6
4/23/2018 17:55	5.8	2.4	0.2861	2.6
4/23/2018 17:56	5.9	2.4	0.3025	2.7
4/23/2018 17:57	6.1	2.4	0.3098	2.7
4/23/2018 17:58	6.1	2.5	0.3145	2.8
4/23/2018 17:59	6.2	2.6	0.3097	2.9
4/23/2018 18:00	6.2	2.6	0.3065	2.9
4/23/2018 18:01	6.1	2.7	0.3113	2.9
4/23/2018 18:02	6	2.7	0.266	2.9
4/23/2018 18:03	5.9	2.6	0.2626	3
4/23/2018 18:04	5.8	2.6	0.2473	3
4/23/2018 18:05	5.7	2.6	0.2503	2.9
4/23/2018 18:06	5.6	2.5	0.2412	2.9
4/23/2018 18:07	5.8	2.5	0.2301	2.9
4/23/2018 18:08	5.8	2.4	0.2413	2.9
4/23/2018 18:09	5.8	2.3	0.2299	2.9
4/23/2018 18:10	5.8	2.2	0.2333	2.8
4/23/2018 18:11	5.9	2.2	0.2096	2.6
4/23/2018 18:12	5.9	2.3	0.2726	2.5
4/23/2018 18:13	6.1	2.3	0.2736	2.5
4/23/2018 18:14	6.1	2.3	0.2744	2.5
4/23/2018 18:15	6.1	2.4	0.2887	2.5
4/23/2018 18:16	6.1	2.5	0.2892	2.6
4/23/2018 18:17	6.1	2.5	0.287	2.6
4/23/2018 18:18	6	2.6	0.276	2.7
4/23/2018 18:19	6	2.7	0.2926	2.7
4/23/2018 18:20	6	2.7	0.2874	2.8
4/23/2018 18:21	6	2.7	0.271	2.8
4/23/2018 18:22	6	2.7	0.2274	2.8
4/23/2018 18:23	6	2.6	0.2322	2.8

4/23/2018 18:24	5.7	2.5	0.2313	2.8
4/23/2018 18:25	5.5	2.5	0.2214	2.9
4/23/2018 18:26	5.4	2.4	0.2267	2.8
4/23/2018 18:27	5.4	2.3	0.2029	2.7
4/23/2018 18:28	5.4	2.2	0.2403	2.8
4/23/2018 18:29	5.3	2.1	0.1997	2.7
4/23/2018 18:30	5.3	2.2	0.2631	2.8
4/23/2018 18:31	5.5	2.2	0.2664	2.8
4/23/2018 18:32	5.4	2.3	0.263	2.7
4/23/2018 18:33	5.4	2.4	0.2675	2.8
4/23/2018 18:34	5.4	2.4	0.2571	2.7
4/23/2018 18:35	5.3	2.5	0.2773	2.5
4/23/2018 18:36	5.3	2.6	0.2681	2.5
4/23/2018 18:37	5.2	2.7	0.2548	2.5
4/23/2018 18:38	5.2	2.8	0.2673	2.6
4/23/2018 18:39	5.1	2.7	0.2459	2.6
4/23/2018 18:40	4.8	2.6	0.237	2.6
4/23/2018 18:41	4.7	2.6	0.2107	2.6
4/23/2018 18:42	4.7	2.5	0.2159	2.6
4/23/2018 18:43	4.6	2.3	0.1887	2.6
4/23/2018 18:44	4.5	2.2	0.2077	2.6
4/23/2018 18:45	4.5	2.1	0.2072	2.5
4/23/2018 18:46	4.5	2.1	0.2055	2.5
4/23/2018 18:47	4.8	2	0.2438	2.5
4/23/2018 18:48	4.9	2.1	0.235	2.5
4/23/2018 18:49	5	2.2	0.2574	2.4
4/23/2018 18:50	5	2.3	0.2381	2.4
4/23/2018 18:51	4.9	2.4	0.2533	2.4
4/23/2018 18:52	4.8	2.5	0.2448	2.5
4/23/2018 18:53	4.8	2.5	0.2455	2.4
4/23/2018 18:54	4.8	2.6	0.2475	2.4
4/23/2018 18:55	4.8	2.7	0.2349	2.4
4/23/2018 18:56	4.7	2.6	0.2346	2.5
4/23/2018 18:57	4.4	2.6	0.2127	2.5
4/23/2018 18:58	4.2	2.5	0.2116	2.5
4/23/2018 18:59	4.2	2.5	0.2033	2.4
4/23/2018 19:00	4.1	2.3	0.187	2.5
4/23/2018 19:01	4	2.2	0.1966	2.5
4/23/2018 19:02	4.1	2.1	0.1887	2.5
4/23/2018 19:03	4	2	0.2026	2.6
4/23/2018 19:04	4.3	2	0.2265	2.6
4/23/2018 19:05	4.5	2	0.2294	2.6
4/23/2018 19:06	4.5	2.1	0.2267	2.7
4/23/2018 19:07	4.6	2.1	0.2355	2.6
4/23/2018 19:08	4.7	2.3	0.2449	2.6
4/23/2018 19:09	4.6	2.4	0.2399	2.4
4/23/2018 19:10	4.5	2.5	0.2365	2.3

4/23/2018 19:11	4.4	2.6	0.2281	2.3
4/23/2018 19:12	4.5	2.6	0.231	2.3
4/23/2018 19:13	4.4	2.6	0.2284	2.3
4/23/2018 19:14	4.3	2.6	0.2135	2.2
4/23/2018 19:15	4.2	2.6	0.2196	2.1
4/23/2018 19:16	4	2.5	0.1918	2
4/23/2018 19:17	4	2.4	0.2004	2
4/23/2018 19:18	3.9	2.3	0.1785	2
4/23/2018 19:19	3.8	2.2	0.1778	2
4/23/2018 19:20	3.8	2.1	0.1877	2
4/23/2018 19:21	4.1	2	0.2064	2
4/23/2018 19:22	4.4	2	0.2308	2.1
4/23/2018 19:23	4.3	2	0.2121	2.1
4/23/2018 19:24	4.3	2.1	0.2317	2
4/23/2018 19:25	4.2	2.2	0.2306	2.1
4/23/2018 19:26	4	2.3	0.2137	2.1
4/23/2018 19:27	3.9	2.4	0.2254	2.1
4/23/2018 19:28	4	2.5	0.2173	2.1
4/23/2018 19:29	3.9	2.6	0.222	2.1
4/23/2018 19:30	3.9	2.6	0.221	2.1
4/23/2018 19:31	3.9	2.6	0.2013	2
4/23/2018 19:32	3.7	2.6	0.2102	1.9
4/23/2018 19:33	3.6	2.5	0.1886	1.9
4/23/2018 19:34	3.6	2.4	0.196	2
4/23/2018 19:35	3.5	2.3	0.171	2
4/23/2018 19:36	3.5	2.2	0.1862	2.2
4/23/2018 19:37	3.6	2.1	0.1984	2.2
4/23/2018 19:38	3.8	2.1	0.2209	2.3
4/23/2018 19:39	3.8	2.1	0.2216	2.3
4/23/2018 19:40	3.8	2.1	0.2113	2.4
4/23/2018 19:41	3.8	2.2	0.2202	2.5
4/23/2018 19:42	3.9	2.2	0.2195	2.5
4/23/2018 19:43	3.8	2.3	0.2152	2.5
4/23/2018 19:44	3.8	2.4	0.2182	2.4
4/23/2018 19:45	3.8	2.5	0.2054	2.5
4/23/2018 19:46	3.8	2.5	0.2172	2.6
4/23/2018 19:47	3.8	2.5	0.2091	2.6
4/23/2018 19:48	3.8	2.5	0.2045	2.6
4/23/2018 19:49	3.8	2.5	0.2089	2.6
4/23/2018 19:50	3.7	2.5	0.2013	2.5
4/23/2018 19:51	3.7	2.4	0.1998	2.6
4/23/2018 19:52	3.6	2.3	0.1874	2.8
4/23/2018 19:53	3.6	2.3	0.1889	2.8
4/23/2018 19:54	3.6	2.2	0.1879	2.8
4/23/2018 19:55	3.7	2.1	0.2002	2.8
4/23/2018 19:56	3.8	2.1	0.2191	2.9
4/23/2018 19:57	3.8	2.1	0.2049	3

4/23/2018 19:58	3.8	2.2	0.204	3
4/23/2018 19:59	3.8	2.2	0.2158	3.1
4/23/2018 20:00	3.8	2.3	0.1986	3.1
4/23/2018 20:01	3.8	2.4	0.2163	3.1
4/23/2018 20:02	3.7	2.5	0.1993	3.2
4/23/2018 20:03	3.7	2.6	0.2108	3.1
4/23/2018 20:04	3.7	2.6	0.2038	3.2
4/23/2018 20:05	3.7	2.6	0.2094	2.9
4/23/2018 20:06	3.7	2.6	0.2043	2.8
4/23/2018 20:07	3.7	2.6	0.1928	3
4/23/2018 20:08	3.7	2.5	0.1965	3.1
4/23/2018 20:09	3.7	2.4	0.1831	3.2
4/23/2018 20:10	3.7	2.3	0.1854	3.2
4/23/2018 20:11	3.7	2.3	0.1996	3.2
4/23/2018 20:12	3.7	2.3	0.2054	3.2
4/23/2018 20:13	3.8	2.3	0.2132	3.2
4/23/2018 20:14	3.7	2.3	0.1963	3.2
4/23/2018 20:15	3.7	2.3	0.205	3.2
4/23/2018 20:16	3.7	2.4	0.1983	3.1
4/23/2018 20:17	3.7	2.5	0.2044	3.2
4/23/2018 20:18	3.7	2.5	0.2053	3.1
4/23/2018 20:19	3.7	2.6	0.2043	3.2
4/23/2018 20:20	3.7	2.6	0.2153	3.3
4/23/2018 20:21	3.7	2.6	0.191	3.3
4/23/2018 20:22	3.7	2.7	0.2091	3.3
4/23/2018 20:23	3.7	2.7	0.1999	3.3
4/23/2018 20:24	3.7	2.6	0.1892	3.3
4/23/2018 20:25	3.7	2.6	0.196	3.3
4/23/2018 20:26	3.7	2.5	0.185	3.3
4/23/2018 20:27	3.5	2.5	0.2032	3.3
4/23/2018 20:28	3.5	2.4	0.2145	3.3
4/23/2018 20:29	3.5	2.4	0.1938	3.3
4/23/2018 20:30	3.5	2.4	0.2018	3.3
4/23/2018 20:31	3.5	2.4	0.1999	3.3
4/23/2018 20:32	3.5	2.4	0.208	3.2
4/23/2018 20:33	3.5	2.5	0.2037	3.3
4/23/2018 20:34	3.5	2.5	0.1989	3.4
4/23/2018 20:35	3.5	2.6	0.218	3.4
4/23/2018 20:36	3.5	2.6	0.2021	3.4
4/23/2018 20:37	3.5	2.6	0.1997	3.4
4/23/2018 20:38	3.6	2.6	0.204	3.3
4/23/2018 20:39	3.6	2.6	0.1861	3.3
4/23/2018 20:40	3.7	2.5	0.1927	3.4
4/23/2018 20:41	3.7	2.5	0.1807	3.4
4/23/2018 20:42	3.6	2.4	0.1917	3.4
4/23/2018 20:43	3.5	2.4	0.2054	3.4
4/23/2018 20:44	3.6	2.4	0.1922	3.4

4/23/2018 20:45	3.6	2.4	0.2067	3.4
4/23/2018 20:46	3.6	2.4	0.1935	3.5
4/23/2018 20:47	3.6	2.5	0.2115	3.5
4/23/2018 20:48	3.6	2.5	0.2043	3.4
4/23/2018 20:49	3.7	2.6	0.1898	3.5
4/23/2018 20:50	3.7	2.7	0.2014	3.6
4/23/2018 20:51	3.7	2.6	0.1828	3.6
4/23/2018 20:52	3.6	2.6	0.2062	3.6
4/23/2018 20:53	3.7	2.7	0.1941	3.6
4/23/2018 20:54	3.7	2.6	0.1881	3.6
4/23/2018 20:55	3.8	2.6	0.2001	3.7
4/23/2018 20:56	3.8	2.6	0.195	3.6
4/23/2018 20:57	3.6	2.6	0.2065	3
4/23/2018 20:58	3.7	2.6	0.1946	2.8
4/23/2018 20:59	3.9	2.6	0.1944	2.9
4/23/2018 21:00	3.9	2.6	0.2011	3
4/23/2018 21:01	3.8	2.6	0.1868	3.3
4/23/2018 21:02	3.8	2.6	0.2094	3.2
4/23/2018 21:03	3.9	2.6	0.1921	3.3
4/23/2018 21:04	3.8	2.7	0.2063	3.4
4/23/2018 21:05	3.9	2.6	0.1988	3.5
4/23/2018 21:06	4.1	2.6	0.1909	3.5
4/23/2018 21:07	3.9	2.6	0.2016	3.6
4/23/2018 21:08	4	2.6	0.1921	3.6
4/23/2018 21:09	4.1	2.6	0.2015	3.5
4/23/2018 21:10	4.2	2.6	0.1891	3.4
4/23/2018 21:11	4.3	2.6	0.1949	3.4
4/23/2018 21:12	3.9	2.6	0.2047	3.5
4/23/2018 21:13	3.9	2.6	0.1978	3.5
4/23/2018 21:14	4.3	2.6	0.2054	3.4
4/23/2018 21:15	4.1	2.6	0.1881	3.3
4/23/2018 21:16	4	2.6	0.1953	3.4
4/23/2018 21:17	4	2.6	0.2012	3.4
4/23/2018 21:18	4	2.6	0.1926	3.5
4/23/2018 21:19	3.9	2.7	0.2078	3.6
4/23/2018 21:20	4.2	2.7	0.1926	3.6
4/23/2018 21:21	4.1	2.7	0.2009	3.6
4/23/2018 21:22	4.2	2.7	0.2003	3.6
4/23/2018 21:23	4.1	2.7	0.1979	3.5
4/23/2018 21:24	4.2	2.7	0.196	3.5
4/23/2018 21:25	4.3	2.7	0.1881	3.5
4/23/2018 21:26	4.2	2.7	0.1947	3.5
4/23/2018 21:27	4.3	2.7	0.1886	3.5
4/23/2018 21:28	4.4	2.7	0.1906	3.4
4/23/2018 21:29	4.2	2.7	0.2044	3.3
4/23/2018 21:30	4.4	2.6	0.1861	3.4
4/23/2018 21:31	4.4	2.6	0.1958	3.4

4/23/2018 21:32	4.2	2.6	0.1912	3.4
4/23/2018 21:33	4.2	2.6	0.1965	3.2
4/23/2018 21:34	4.2	2.6	0.2037	3.2
4/23/2018 21:35	4.3	2.6	0.1915	3.3
4/23/2018 21:36	4.5	2.6	0.2029	3.2
4/23/2018 21:37	4.4	2.6	0.1889	3.3
4/23/2018 21:38	4.7	2.6	0.195	3.3
4/23/2018 21:39	4.2	2.6	0.1944	3.3
4/23/2018 21:40	4.5	2.6	0.1927	3.3
4/23/2018 21:41	4.8	2.6	0.1989	3.4
4/23/2018 21:42	5.3	2.6	0.188	3.4
4/23/2018 21:43	5	2.5	0.2046	3.4
4/23/2018 21:44	4.2	2.6	0.1946	3.4
4/23/2018 21:45	4.9	2.6	0.1953	3.5
4/23/2018 21:46	4.8	2.6	0.1915	3.4
4/23/2018 21:47	4.7	2.6	0.1972	3.3
4/23/2018 21:48	4.5	2.5	0.1949	3.3
4/23/2018 21:49	4.6	2.6	0.1917	3.3
4/23/2018 21:50	4.7	2.6	0.2027	3.3
4/23/2018 21:51	4.6	2.6	0.1925	3.3
4/23/2018 21:52	4.7	2.6	0.1984	3.3
4/23/2018 21:53	5.1	2.6	0.1955	3.3
4/23/2018 21:54	5	2.6	0.1963	3.3
4/23/2018 21:55	4.6	2.6	0.1988	3.3
4/23/2018 21:56	5.1	2.6	0.1887	3.2
4/23/2018 21:57	5	2.6	0.1998	3.2
4/23/2018 21:58	4.5	2.6	0.1765	3.2
4/23/2018 21:59	4.9	2.7	0.1968	3.3
4/23/2018 22:00	5.1	2.6	0.1965	3.3
4/23/2018 22:01	4.9	2.6	0.1903	3.3
4/23/2018 22:02	4.8	2.7	0.2	3.4
4/23/2018 22:03	4.7	2.7	0.1857	3.4
4/23/2018 22:04	4.9	2.7	0.1961	3.4
4/23/2018 22:05	4.9	2.7	0.1902	3.4
4/23/2018 22:06	4.9	2.7	0.1922	3.4
4/23/2018 22:07	4.9	2.7	0.1948	3.4
4/23/2018 22:08	4.9	2.7	0.1927	3.4
4/23/2018 22:09	4.8	2.7	0.1979	3.4
4/23/2018 22:10	4.8	2.7	0.1831	3.4
4/23/2018 22:11	5.2	2.7	0.2011	3.4
4/23/2018 22:12	4.8	2.7	0.1817	3.4
4/23/2018 22:13	4.7	2.7	0.1974	3.4
4/23/2018 22:14	5.2	2.7	0.189	3.4
4/23/2018 22:15	5.1	2.7	0.1837	3.4
4/23/2018 22:16	5.1	2.7	0.1942	3.4
4/23/2018 22:17	5	2.7	0.1828	3.3
4/23/2018 22:18	4.8	2.7	0.1979	3.3

4/23/2018 22:19	5	2.7	0.1835	3.4
4/23/2018 22:20	5.4	2.7	0.1902	3.4
4/23/2018 22:21	5.2	2.6	0.1897	3.4
4/23/2018 22:22	5.4	2.6	0.1735	3.3
4/23/2018 22:23	5.2	2.6	0.195	3.3
4/23/2018 22:24	4.9	2.7	0.1843	3.3
4/23/2018 22:25	4.9	2.7	0.1966	3.3
4/23/2018 22:26	5.1	2.7	0.1942	3.3
4/23/2018 22:27	5.1	2.7	0.1967	3.3
4/23/2018 22:28	5.1	2.7	0.1835	3.2
4/23/2018 22:29	5.2	2.7	0.1792	3.2
4/23/2018 22:30	5	2.7	0.1937	3.3
4/23/2018 22:31	4.8	2.7	0.1824	3.3
4/23/2018 22:32	5	2.7	0.1932	3.3
4/23/2018 22:33	4.9	2.7	0.1848	3.4
4/23/2018 22:34	5	2.7	0.1877	3.4
4/23/2018 22:35	5.1	2.7	0.198	3.3
4/23/2018 22:36	5	2.7	0.1889	3.3
4/23/2018 22:37	5.3	2.7	0.1957	3.3
4/23/2018 22:38	5.1	2.6	0.1885	3.4
4/23/2018 22:39	5.2	2.6	0.193	3.4
4/23/2018 22:40	5.1	2.6	0.1839	3.5
4/23/2018 22:41	5.1	2.6	0.1889	3.4
4/23/2018 22:42	5.2	2.6	0.1963	3.4
4/23/2018 22:43	5.5	2.5	0.1707	3.4
4/23/2018 22:44	5.3	2.5	0.1922	3.4
4/23/2018 22:45	5	2.5	0.179	3.3
4/23/2018 22:46	5.1	2.6	0.1923	2.9
4/23/2018 22:47	5.1	2.6	0.1814	2.9
4/23/2018 22:48	5.2	2.6	0.1817	2.9
4/23/2018 22:49	5.2	2.6	0.195	3
4/23/2018 22:50	5.3	2.6	0.179	3.2
4/23/2018 22:51	5.3	2.6	0.192	3.1
4/23/2018 22:52	5.3	2.7	0.1846	3.2
4/23/2018 22:53	5.4	2.6	0.1818	3.3
4/23/2018 22:54	5.2	2.6	0.1868	3.4
4/23/2018 22:55	5.3	2.6	0.1768	3.4
4/23/2018 22:56	5.6	2.6	0.1842	3.4
4/23/2018 22:57	5.4	2.6	0.1772	3.5
4/23/2018 22:58	5.2	2.6	0.1918	3.4
4/23/2018 22:59	5	2.6	0.1841	3.4
4/23/2018 23:00	4.9	2.6	0.1871	3.4
4/23/2018 23:01	5.1	2.6	0.1876	3.3
4/23/2018 23:02	5	2.6	0.185	3.3
4/23/2018 23:03	5.2	2.6	0.1878	3.3
4/23/2018 23:04	5.2	2.6	0.1813	3.3
4/23/2018 23:05	5.2	2.7	0.1873	3.3

4/23/2018 23:06	5.1	2.7	0.1838	3.3
4/23/2018 23:07	5.1	2.7	0.1821	3.3
4/23/2018 23:08	5	2.7	0.1887	3.3
4/23/2018 23:09	5	2.6	0.1763	3.4
4/23/2018 23:10	5.1	2.6	0.1886	3.3
4/23/2018 23:11	5.2	2.6	0.1731	3.3
4/23/2018 23:12	5	2.6	0.1887	3.3
4/23/2018 23:13	5	2.6	0.1794	3.3
4/23/2018 23:14	5	2.6	0.1853	3.2
4/23/2018 23:15	4.9	2.6	0.1862	3.2
4/23/2018 23:16	5	2.6	0.1787	3.2
4/23/2018 23:17	5	2.6	0.189	3.2
4/23/2018 23:18	5	2.6	0.1749	3.1
4/23/2018 23:19	5	2.7	0.1853	3
4/23/2018 23:20	4.9	2.7	0.1769	3.1
4/23/2018 23:21	4.9	2.7	0.1777	3.1
4/23/2018 23:22	4.9	2.7	0.1873	3.1
4/23/2018 23:23	4.9	2.7	0.1745	3.2
4/23/2018 23:24	4.9	2.7	0.1834	3
4/23/2018 23:25	5	2.6	0.1694	3.1
4/23/2018 23:26	4.9	2.6	0.1748	3.1
4/23/2018 23:27	4.8	2.6	0.1794	3.1
4/23/2018 23:28	4.8	2.6	0.1836	3.1
4/23/2018 23:29	4.8	2.6	0.187	3
4/23/2018 23:30	4.7	2.7	0.1737	3.1
4/23/2018 23:31	4.6	2.7	0.1865	3.2
4/23/2018 23:32	4.6	2.7	0.1774	3.2
4/23/2018 23:33	4.6	2.7	0.1881	3.1
4/23/2018 23:34	4.6	2.7	0.1771	2.7
4/23/2018 23:35	4.7	2.7	0.179	2.2
4/23/2018 23:36	4.6	2.7	0.1936	2.3
4/23/2018 23:37	4.6	2.7	0.1709	2.6
4/23/2018 23:38	4.6	2.7	0.1917	2.7
4/23/2018 23:39	4.6	2.7	0.1636	2.9
4/23/2018 23:40	4.6	2.6	0.1764	3
4/23/2018 23:41	4.6	2.6	0.1769	3
4/23/2018 23:42	4.5	2.6	0.1754	3.1
4/23/2018 23:43	4.6	2.5	0.1842	3.1
4/23/2018 23:44	4.6	2.5	0.168	3.2
4/23/2018 23:45	4.6	2.5	0.1823	3.2
4/23/2018 23:46	4.5	2.5	0.1695	3.2
4/23/2018 23:47	4.5	2.5	0.1813	3.3
4/23/2018 23:48	4.4	2.6	0.171	3.2
4/23/2018 23:49	4.4	2.6	0.167	3.2
4/23/2018 23:50	4.5	2.6	0.1821	3.2
4/23/2018 23:51	4.5	2.6	0.1647	3.1
4/23/2018 23:52	4.5	2.6	0.1856	3

4/23/2018 23:53	4.5	2.6	0.1714	3
4/23/2018 23:54	4.4	2.6	0.1826	3
4/23/2018 23:55	4.5	2.5	0.1775	3
4/23/2018 23:56	4.5	2.5	0.1734	3
4/23/2018 23:57	4.5	2.5	0.1737	3
4/23/2018 23:58	4.4	2.5	0.1628	2.9
4/23/2018 23:59	4.4	2.5	0.1808	2.8
4/24/2018 0:00	4.4	2.5	0.172	2.8
4/24/2018 0:01	4.3	2.5	0.1773	2.8
4/24/2018 0:02	4.4	2.5	0.1791	3
4/24/2018 0:03	4.4	2.6	0.1708	3
4/24/2018 0:04	4.5	2.6	0.1814	3.1
4/24/2018 0:05	4.6	2.6	0.1647	3.1
4/24/2018 0:06	4.7	2.6	0.1803	3
4/24/2018 0:07	4.9	2.6	0.168	3
4/24/2018 0:08	4.8	2.6	0.1755	3.1
4/24/2018 0:09	4.7	2.6	0.1776	3.1
4/24/2018 0:10	4.9	2.5	0.1694	3.1
4/24/2018 0:11	5.1	2.5	0.1767	3.1
4/24/2018 0:12	5.1	2.5	0.1594	2.6
4/24/2018 0:13	4.7	2.5	0.1854	2
4/24/2018 0:14	4.8	2.5	0.1664	2.1
4/24/2018 0:15	4.8	2.6	0.1783	2.3
4/24/2018 0:16	4.9	2.6	0.1726	2.6
4/24/2018 0:17	4.8	2.6	0.1685	2.9
4/24/2018 0:18	4.8	2.6	0.1729	2.8
4/24/2018 0:19	4.8	2.6	0.1628	3
4/24/2018 0:20	4.7	2.7	0.1768	3.1
4/24/2018 0:21	4.9	2.7	0.1669	3.2
4/24/2018 0:22	4.9	2.6	0.1753	3.2
4/24/2018 0:23	4.9	2.6	0.1779	3.2
4/24/2018 0:24	4.9	2.6	0.1596	3.2
4/24/2018 0:25	5	2.6	0.1795	3.1
4/24/2018 0:26	4.8	2.6	0.1651	3.1
4/24/2018 0:27	4.9	2.6	0.1777	3.1
4/24/2018 0:28	4.9	2.6	0.1757	3.1
4/24/2018 0:29	4.9	2.5	0.1632	3.1
4/24/2018 0:30	4.9	2.5	0.1817	3.1
4/24/2018 0:31	4.7	2.6	0.165	3.1
4/24/2018 0:32	4.6	2.6	0.1788	3
4/24/2018 0:33	4.6	2.6	0.1585	3
4/24/2018 0:34	4.6	2.6	0.1767	3
4/24/2018 0:35	4.5	2.6	0.1649	3
4/24/2018 0:36	4.6	2.5	0.1665	3.1
4/24/2018 0:37	4.5	2.6	0.1734	3.1
4/24/2018 0:38	4.5	2.5	0.1591	3.1
4/24/2018 0:39	4.5	2.5	0.1786	3.2

4/24/2018 0:40	4.3	2.5	0.1664	3.2
4/24/2018 0:41	4.4	2.5	0.1772	3.1
4/24/2018 0:42	4.4	2.5	0.1809	3.2
4/24/2018 0:43	4.3	2.5	0.1619	3.2
4/24/2018 0:44	4.3	2.6	0.1696	3.3
4/24/2018 0:45	4.3	2.6	0.158	3.3
4/24/2018 0:46	4.3	2.6	0.1763	3.3
4/24/2018 0:47	4.3	2.6	0.1655	3.3
4/24/2018 0:48	4.2	2.6	0.1655	3.3
4/24/2018 0:49	4.2	2.6	0.1728	3.3
4/24/2018 0:50	4.2	2.6	0.1553	3.3
4/24/2018 0:51	4.2	2.5	0.1749	3.3
4/24/2018 0:52	4.1	2.5	0.1601	3.3
4/24/2018 0:53	4.1	2.5	0.1683	3.2
4/24/2018 0:54	4.1	2.5	0.1609	3.1
4/24/2018 0:55	4.1	2.5	0.1707	3.1
4/24/2018 0:56	4	2.4	0.1735	3.1
4/24/2018 0:57	4	2.4	0.1597	3.2
4/24/2018 0:58	4	2.4	0.1639	3.2
4/24/2018 0:59	4	2.4	0.1618	3.2
4/24/2018 1:00	4	2.4	0.1703	3.1
4/24/2018 1:01	4	2.4	0.1655	3.1
4/24/2018 1:02	4	2.4	0.1668	3.1
4/24/2018 1:03	3.9	2.4	0.1691	3.1
4/24/2018 1:04	3.9	2.4	0.1626	3.1
4/24/2018 1:05	4	2.4	0.1695	3.1
4/24/2018 1:06	3.9	2.4	0.1548	3.2
4/24/2018 1:07	3.9	2.4	0.1661	3.2
4/24/2018 1:08	3.9	2.5	0.1601	3
4/24/2018 1:09	3.9	2.5	0.167	2.8
4/24/2018 1:10	3.9	2.5	0.1678	2.8
4/24/2018 1:11	3.9	2.5	0.1552	2.9
4/24/2018 1:12	3.9	2.5	0.1693	2.9
4/24/2018 1:13	3.9	2.5	0.1583	2.9
4/24/2018 1:14	3.9	2.5	0.1691	2.9
4/24/2018 1:15	3.9	2.5	0.1641	3
4/24/2018 1:16	3.9	2.5	0.1622	3
4/24/2018 1:17	3.9	2.5	0.1776	3
4/24/2018 1:18	3.9	2.5	0.1638	3
4/24/2018 1:19	3.8	2.5	0.1738	3
4/24/2018 1:20	3.8	2.4	0.1572	2.9
4/24/2018 1:21	3.8	2.4	0.1606	3
4/24/2018 1:22	3.8	2.4	0.1631	3
4/24/2018 1:23	3.8	2.5	0.1604	3
4/24/2018 1:24	3.8	2.5	0.1689	3.1
4/24/2018 1:25	3.8	2.5	0.154	3.1
4/24/2018 1:26	3.7	2.5	0.1635	3.1

4/24/2018 1:27	3.8	2.5	0.1468	3.1
4/24/2018 1:28	3.7	2.5	0.171	3.1
4/24/2018 1:29	3.7	2.5	0.1626	3.1
4/24/2018 1:30	3.8	2.5	0.1567	3.1
4/24/2018 1:31	3.8	2.5	0.1653	3.1
4/24/2018 1:32	3.7	2.5	0.1567	3.2
4/24/2018 1:33	3.6	2.5	0.1651	3.2
4/24/2018 1:34	3.6	2.5	0.1563	3.2
4/24/2018 1:35	3.6	2.5	0.1563	3.3
4/24/2018 1:36	3.6	2.5	0.1622	3.3
4/24/2018 1:37	3.6	2.5	0.1537	3.3
4/24/2018 1:38	3.6	2.5	0.1708	3.3
4/24/2018 1:39	3.6	2.5	0.1518	3.4
4/24/2018 1:40	3.7	2.5	0.1615	3.4
4/24/2018 1:41	3.7	2.5	0.1516	3.4
4/24/2018 1:42	3.7	2.5	0.1608	3.5
4/24/2018 1:43	3.6	2.5	0.1677	3.5
4/24/2018 1:44	3.6	2.6	0.1515	3.5
4/24/2018 1:45	3.7	2.6	0.1661	3.5
4/24/2018 1:46	3.6	2.6	0.156	3.5
4/24/2018 1:47	3.6	2.6	0.163	3.5
4/24/2018 1:48	3.6	2.6	0.1558	3.5
4/24/2018 1:49	3.6	2.6	0.1588	3.4
4/24/2018 1:50	3.6	2.6	0.1689	3.4
4/24/2018 1:51	3.8	2.6	0.153	3.4
4/24/2018 1:52	3.7	2.6	0.1617	3.5
4/24/2018 1:53	3.7	2.6	0.1589	3.5
4/24/2018 1:54	3.6	2.6	0.1617	3.5
4/24/2018 1:55	3.8	2.6	0.165	3.5
4/24/2018 1:56	3.7	2.6	0.1537	3.5
4/24/2018 1:57	3.7	2.6	0.1691	3.5
4/24/2018 1:58	3.8	2.6	0.1506	3.5
4/24/2018 1:59	3.8	2.6	0.1636	3.5
4/24/2018 2:00	3.8	2.6	0.1577	3.6
4/24/2018 2:01	3.7	2.6	0.1619	3.5
4/24/2018 2:02	3.7	2.6	0.1657	3.5
4/24/2018 2:03	3.7	2.6	0.1509	3.1
4/24/2018 2:04	3.6	2.6	0.1683	1.8
4/24/2018 2:05	3.6	2.6	0.1614	1.5
4/24/2018 2:06	3.7	2.7	0.1612	1.5
4/24/2018 2:07	3.7	2.7	0.162	1.5
4/24/2018 2:08	3.7	2.7	0.1638	1.5
4/24/2018 2:09	3.8	2.7	0.1605	1.6
4/24/2018 2:10	3.9	2.7	0.1566	1.5
4/24/2018 2:11	3.7	2.6	0.1658	1.5
4/24/2018 2:12	3.8	2.6	0.1538	1.5
4/24/2018 2:13	3.8	2.6	0.1623	1.5

4/24/2018 2:14	3.8	2.6	0.1534	1.6
4/24/2018 2:15	3.8	2.6	0.1675	1.5
4/24/2018 2:16	3.9	2.6	0.1635	1.5
4/24/2018 2:17	3.8	2.6	0.1539	1.5
4/24/2018 2:18	3.9	2.6	0.167	1.5
4/24/2018 2:19	3.9	2.7	0.1603	1.5
4/24/2018 2:20	3.9	2.7	0.1732	1.5
4/24/2018 2:21	3.9	2.7	0.1581	1.5
4/24/2018 2:22	4	2.6	0.1679	1.5
4/24/2018 2:23	4	2.6	0.1647	1.5
4/24/2018 2:24	4.1	2.6	0.1522	1.5
4/24/2018 2:25	4.2	2.6	0.1654	1.5
4/24/2018 2:26	4.3	2.6	0.1594	1.5
4/24/2018 2:27	4.3	2.6	0.1692	1.5
4/24/2018 2:28	4.2	2.5	0.1598	1.5
4/24/2018 2:29	4.2	2.5	0.1692	1.5
4/24/2018 2:30	4.3	2.5	0.1767	1.5
4/24/2018 2:31	4.3	2.5	0.1541	1.5
4/24/2018 2:32	4.3	2.5	0.1687	1.5
4/24/2018 2:33	4.6	2.4	0.1637	1.5
4/24/2018 2:34	4.5	2.4	0.1697	1.5
4/24/2018 2:35	4.2	2.5	0.1572	1.5
4/24/2018 2:36	4.5	2.5	0.1681	1.5
4/24/2018 2:37	4.2	2.5	0.1679	1.5
4/24/2018 2:38	4.2	2.5	0.1584	1.5
4/24/2018 2:39	4	2.5	0.171	1.5
4/24/2018 2:40	4	2.6	0.1561	1.5
4/24/2018 2:41	4.2	2.6	0.1762	1.5
4/24/2018 2:42	4.2	2.7	0.1618	1.6
4/24/2018 2:43	4.3	2.7	0.1594	1.6
4/24/2018 2:44	4.3	2.7	0.1728	1.6
4/24/2018 2:45	4.4	2.8	0.1589	1.6
4/24/2018 2:46	4.6	2.8	0.1763	1.6
4/24/2018 2:47	4.4	2.8	0.1613	1.6
4/24/2018 2:48	4.6	2.8	0.1675	1.6
4/24/2018 2:49	4.5	2.8	0.1654	1.6
4/24/2018 2:50	4.6	2.8	0.1572	1.6
4/24/2018 2:51	4.9	2.8	0.1715	1.6
4/24/2018 2:52	5	2.8	0.1614	1.6
4/24/2018 2:53	5.4	2.8	0.1737	1.6
4/24/2018 2:54	5.4	2.8	0.1611	1.6
4/24/2018 2:55	5.4	2.7	0.1709	1.6
4/24/2018 2:56	5.5	2.7	0.1688	1.6
4/24/2018 2:57	5.5	2.8	0.1647	1.7
4/24/2018 2:58	5.2	2.7	0.1789	1.7
4/24/2018 2:59	5.9	2.7	0.1742	1.6
4/24/2018 3:00	6.1	2.7	0.1837	1.6

4/24/2018 3:01	7	2.7	0.1797	1.6
4/24/2018 3:02	6.7	2.7	0.1733	1.7
4/24/2018 3:03	5.7	2.7	0.1803	1.6
4/24/2018 3:04	6.1	2.6	0.1716	1.6
4/24/2018 3:05	5.6	2.7	0.1807	1.6
4/24/2018 3:06	5.6	2.7	0.1684	1.6
4/24/2018 3:07	5.2	2.7	0.1817	1.6
4/24/2018 3:08	5.5	2.7	0.1696	1.6
4/24/2018 3:09	5.5	2.7	0.1684	1.7
4/24/2018 3:10	5.4	2.8	0.1683	1.6
4/24/2018 3:11	5.7	2.8	0.1692	1.7
4/24/2018 3:12	5.6	2.9	0.1722	1.7
4/24/2018 3:13	5.9	2.9	0.1647	1.7
4/24/2018 3:14	5.9	2.9	0.1848	1.7
4/24/2018 3:15	6.1	2.9	0.1707	1.7
4/24/2018 3:16	6.1	3	0.1732	1.7
4/24/2018 3:17	6	3	0.1767	1.7
4/24/2018 3:18	6	3	0.1614	1.7
4/24/2018 3:19	6.3	3	0.1806	1.7
4/24/2018 3:20	6.2	3	0.1732	1.7
4/24/2018 3:21	6.5	3.1	0.1726	1.7
4/24/2018 3:22	6.4	3.1	0.1763	1.7
4/24/2018 3:23	6.6	3.1	0.1744	1.7
4/24/2018 3:24	6.8	3.1	0.1789	1.7
4/24/2018 3:25	7.1	3.1	0.1704	1.7
4/24/2018 3:26	7.2	3.1	0.1856	1.7
4/24/2018 3:27	7.1	3.2	0.1716	1.7
4/24/2018 3:28	7.8	3.1	0.1727	1.7
4/24/2018 3:29	8.3	3.1	0.1781	1.7
4/24/2018 3:30	8.5	3.1	0.1697	1.8
4/24/2018 3:31	7.7	3.1	0.1791	1.8
4/24/2018 3:32	8.4	3.1	0.171	1.9
4/24/2018 3:33	8.4	3.1	0.1797	1.9
4/24/2018 3:34	8.5	3.1	0.1744	1.8
4/24/2018 3:35	8.1	3	0.1706	1.9
4/24/2018 3:36	7.2	3.1	0.1813	1.9
4/24/2018 3:37	6.8	3.1	0.1697	1.9
4/24/2018 3:38	6.6	3.1	0.1783	1.9
4/24/2018 3:39	7	3.1	0.1675	1.9
4/24/2018 3:40	6.9	3.1	0.1753	1.9
4/24/2018 3:41	6.7	3.1	0.1869	2
4/24/2018 3:42	7.1	3.2	0.1715	1.9
4/24/2018 3:43	6.9	3.2	0.1891	1.9
4/24/2018 3:44	6.7	3.2	0.1723	1.8
4/24/2018 3:45	6.9	3.2	0.1843	1.8
4/24/2018 3:46	7	3.1	0.1753	1.9
4/24/2018 3:47	7	3.2	0.1653	2

4/24/2018 3:48	7	3.2	0.1847	2
4/24/2018 3:49	7.2	3.2	0.1601	2
4/24/2018 3:50	6.9	3.1	0.1919	2.1
4/24/2018 3:51	7.1	3.2	0.1723	2.1
4/24/2018 3:52	7.2	3.1	0.1722	2.1
4/24/2018 3:53	7	3.2	0.174	2.1
4/24/2018 3:54	6.9	3.2	0.1762	2
4/24/2018 3:55	6.9	3.2	0.1803	2
4/24/2018 3:56	6.9	3.2	0.1667	2
4/24/2018 3:57	7	3.2	0.1843	2
4/24/2018 3:58	7	3.2	0.1712	2
4/24/2018 3:59	7.1	3.2	0.179	2
4/24/2018 4:00	7.2	3.2	0.173	2.1
4/24/2018 4:01	7.4	3.2	0.1665	2.1
4/24/2018 4:02	7.5	3.1	0.1814	2.1
4/24/2018 4:03	7.2	3.1	0.1623	2.1
4/24/2018 4:04	7.5	3.1	0.1799	2.1
4/24/2018 4:05	7.5	3	0.1623	2.1
4/24/2018 4:06	7.5	3	0.1796	2
4/24/2018 4:07	7.4	3	0.1804	1.9
4/24/2018 4:08	7.4	3	0.1726	2
4/24/2018 4:09	7.5	2.9	0.1788	2
4/24/2018 4:10	7.4	2.9	0.166	2
4/24/2018 4:11	6.9	2.9	0.1859	2
4/24/2018 4:12	6.8	3	0.1656	2.1
4/24/2018 4:13	6.8	3	0.1746	2.1
4/24/2018 4:14	6.8	3	0.1756	2.1
4/24/2018 4:15	6.8	3	0.1692	2.1
4/24/2018 4:16	6.7	3.1	0.1799	2.1
4/24/2018 4:17	6.7	3.1	0.1648	2.1
4/24/2018 4:18	6.7	3.2	0.1796	2.1
4/24/2018 4:19	6.6	3.2	0.1692	2.3
4/24/2018 4:20	6.8	3.2	0.1688	2.3
4/24/2018 4:21	6.6	3.2	0.1844	2.4
4/24/2018 4:22	6.6	3.1	0.1679	2.4
4/24/2018 4:23	6.6	3.1	0.1813	2.2
4/24/2018 4:24	6.6	3.1	0.172	2.2
4/24/2018 4:25	6.5	3.1	0.1719	2.2
4/24/2018 4:26	6.5	3.1	0.1743	2
4/24/2018 4:27	6.6	3.1	0.1687	2
4/24/2018 4:28	6.5	3.1	0.1862	2
4/24/2018 4:29	6.4	3.1	0.1625	2.1
4/24/2018 4:30	6.5	3.1	0.1785	2.1
4/24/2018 4:31	6.5	3.1	0.1648	2.1
4/24/2018 4:32	6.4	3.1	0.1769	2
4/24/2018 4:33	6.4	3.1	0.1748	1.9
4/24/2018 4:34	6.4	3	0.1683	1.9

4/24/2018 4:35	6.4	3	0.1819	1.9
4/24/2018 4:36	6.3	3	0.1662	1.9
4/24/2018 4:37	6.3	3	0.1776	2
4/24/2018 4:38	6.3	2.9	0.1719	2
4/24/2018 4:39	6.3	2.9	0.1683	2
4/24/2018 4:40	6.3	2.8	0.1756	2
4/24/2018 4:41	6.3	2.8	0.1607	2
4/24/2018 4:42	6.2	2.8	0.1733	1.9
4/24/2018 4:43	6.1	2.8	0.1696	1.9
4/24/2018 4:44	6.2	2.8	0.1712	1.9
4/24/2018 4:45	6	2.8	0.1733	1.9
4/24/2018 4:46	6	2.8	0.1729	1.9
4/24/2018 4:47	6	2.9	0.1796	1.9
4/24/2018 4:48	5.9	2.9	0.1661	1.9
4/24/2018 4:49	5.9	2.9	0.174	1.9
4/24/2018 4:50	5.9	3	0.1727	1.9
4/24/2018 4:51	5.9	3	0.1694	1.9
4/24/2018 4:52	5.9	3	0.1788	1.9
4/24/2018 4:53	5.8	3	0.1602	1.9
4/24/2018 4:54	5.8	3.1	0.1748	2
4/24/2018 4:55	5.8	3	0.1667	2
4/24/2018 4:56	5.7	3	0.1695	1.9
4/24/2018 4:57	5.7	3	0.1737	2
4/24/2018 4:58	5.7	3	0.1637	2
4/24/2018 4:59	5.7	3	0.1681	2
4/24/2018 5:00	5.7	3	0.1617	2
4/24/2018 5:01	5.7	3	0.1673	2.1
4/24/2018 5:02	5.7	3	0.1751	2.1
4/24/2018 5:03	5.6	2.9	0.1633	2.2
4/24/2018 5:04	5.5	2.9	0.1724	2.3
4/24/2018 5:05	5.5	2.9	0.1597	2.2
4/24/2018 5:06	5.6	2.8	0.1813	2.2
4/24/2018 5:07	5.5	2.8	0.1714	2.2
4/24/2018 5:08	5.6	2.8	0.1687	2.2
4/24/2018 5:09	5.5	2.7	0.1788	2.2
4/24/2018 5:10	5.5	2.7	0.1639	2.3
4/24/2018 5:11	5.5	2.6	0.1759	2.5
4/24/2018 5:12	5.5	2.6	0.1681	2.6
4/24/2018 5:13	5.4	2.6	0.1712	2.5
4/24/2018 5:14	5.3	2.6	0.1702	2.4
4/24/2018 5:15	5.3	2.6	0.1638	2.4
4/24/2018 5:16	5.3	2.7	0.1757	2.2
4/24/2018 5:17	5.3	2.7	0.1684	2.2
4/24/2018 5:18	5.3	2.8	0.1728	2.1
4/24/2018 5:19	5.3	2.8	0.1784	2.2
4/24/2018 5:20	5.3	2.8	0.1641	2.2
4/24/2018 5:21	5.2	2.9	0.1772	2.2

4/24/2018 5:22	5.2	2.9	0.1668	2.2
4/24/2018 5:23	5.2	2.9	0.1765	2.1
4/24/2018 5:24	5.1	2.9	0.1623	2.1
4/24/2018 5:25	5.1	2.9	0.169	2.2
4/24/2018 5:26	5.1	2.9	0.1716	2.2
4/24/2018 5:27	5.1	2.9	0.158	2.2
4/24/2018 5:28	5.1	2.9	0.1725	2.2
4/24/2018 5:29	5	2.9	0.1612	2.3
4/24/2018 5:30	5.1	2.9	0.1672	2.4
4/24/2018 5:31	5	2.9	0.1693	2.3
4/24/2018 5:32	5	2.9	0.1711	2.2
4/24/2018 5:33	5	2.9	0.1793	2.2
4/24/2018 5:34	5	2.8	0.1607	2.1
4/24/2018 5:35	5	2.8	0.1693	2
4/24/2018 5:36	5	2.8	0.1705	1.9
4/24/2018 5:37	4.9	2.7	0.1752	1.9
4/24/2018 5:38	4.9	2.7	0.1574	1.9
4/24/2018 5:39	5	2.7	0.1663	1.8
4/24/2018 5:40	5	2.7	0.1745	1.9
4/24/2018 5:41	5	2.6	0.1657	1.9
4/24/2018 5:42	4.9	2.6	0.1804	2
4/24/2018 5:43	5	2.6	0.1661	2
4/24/2018 5:44	5	2.6	0.1733	2
4/24/2018 5:45	4.9	2.6	0.1737	2.1
4/24/2018 5:46	4.9	2.6	0.1743	2.1
4/24/2018 5:47	4.8	2.7	0.1757	2.1
4/24/2018 5:48	4.8	2.7	0.1655	2.1
4/24/2018 5:49	4.8	2.7	0.1777	2.1
4/24/2018 5:50	4.8	2.8	0.1609	2.2
4/24/2018 5:51	4.9	2.8	0.1811	2.2
4/24/2018 5:52	4.9	2.8	0.1701	2.2
4/24/2018 5:53	4.9	2.9	0.1688	2.2
4/24/2018 5:54	4.9	2.9	0.1745	2.1
4/24/2018 5:55	5	2.9	0.1651	2.2
4/24/2018 5:56	5.2	2.9	0.1819	2.2
4/24/2018 5:57	5.1	2.9	0.1698	2.2
4/24/2018 5:58	5.3	2.9	0.175	2.3
4/24/2018 5:59	5.4	2.9	0.1825	2.3
4/24/2018 6:00	5.5	2.9	0.1668	2.3
4/24/2018 6:01	5.6	2.9	0.1763	2.2
4/24/2018 6:02	5.9	2.9	0.1687	2.1
4/24/2018 6:03	6.5	3	0.1783	2.1
4/24/2018 6:04	6.4	3	0.1787	2.2
4/24/2018 6:05	6.6	3	0.1728	2.1
4/24/2018 6:06	6.9	3	0.1838	2
4/24/2018 6:07	6.7	3.1	0.1762	2.1
4/24/2018 6:08	7	3.1	0.175	2.1

4/24/2018 6:09	6.8	3.1	0.185	2.2
4/24/2018 6:10	6.9	3.1	0.1765	2.2
4/24/2018 6:11	6.7	3.1	0.1844	2.3
4/24/2018 6:12	6.3	3.1	0.1757	2.5
4/24/2018 6:13	5.9	3.1	0.1745	2.4
4/24/2018 6:14	5.8	3.2	0.1841	2.4
4/24/2018 6:15	6	3.2	0.1675	2.3
4/24/2018 6:16	5.8	3.2	0.1786	2.3
4/24/2018 6:17	5.7	3.2	0.1731	2.3
4/24/2018 6:18	6	3.2	0.1797	2.3
4/24/2018 6:19	5.8	3.2	0.1906	2.3
4/24/2018 6:20	5.8	3.2	0.1693	2.4
4/24/2018 6:21	6	3.2	0.189	2.3
4/24/2018 6:22	6.2	3.2	0.1699	2.3
4/24/2018 6:23	5.9	3.2	0.1851	2.3
4/24/2018 6:24	6.1	3.2	0.1845	2.3
4/24/2018 6:25	6	3.2	0.1749	2.3
4/24/2018 6:26	6.4	3.2	0.1813	2.1
4/24/2018 6:27	6.5	3.2	0.1769	1.9
4/24/2018 6:28	6.6	3.2	0.1803	1.9
4/24/2018 6:29	6.9	3.3	0.1686	2
4/24/2018 6:30	6.8	3.3	0.1876	2
4/24/2018 6:31	7	3.3	0.1745	2.1
4/24/2018 6:32	7.2	3.3	0.1821	2
4/24/2018 6:33	6.9	3.3	0.1811	2
4/24/2018 6:34	7.4	3.3	0.178	2.1
4/24/2018 6:35	7.6	3.3	0.1868	2.1
4/24/2018 6:36	7.6	3.3	0.1752	2.2
4/24/2018 6:37	7.6	3.3	0.1822	2.3
4/24/2018 6:38	7.3	3.3	0.1855	2.3
4/24/2018 6:39	7.4	3.3	0.1762	2.3
4/24/2018 6:40	7.2	3.3	0.1828	2.3
4/24/2018 6:41	6.7	3.3	0.173	2.2
4/24/2018 6:42	6.5	3.4	0.1856	2.1
4/24/2018 6:43	6.6	3.4	0.1766	2.1
4/24/2018 6:44	6.7	3.4	0.1819	2.1
4/24/2018 6:45	6.6	3.4	0.1834	2.1
4/24/2018 6:46	6.3	3.5	0.1771	2.1
4/24/2018 6:47	6.6	3.5	0.1855	2.2
4/24/2018 6:48	6.8	3.5	0.1747	2.2
4/24/2018 6:49	6.9	3.5	0.1857	2.3
4/24/2018 6:50	6.6	3.5	0.1803	2.2
4/24/2018 6:51	6.6	3.5	0.1839	2.3
4/24/2018 6:52	6.9	3.5	0.1839	2.3
4/24/2018 6:53	7	3.5	0.1787	2.4
4/24/2018 6:54	7	3.5	0.182	2.5
4/24/2018 6:55	7.1	3.5	0.1806	2.6

4/24/2018 6:56					7	3.5	0.1884	2.5
4/24/2018 6:57					7	3.6	0.1796	2.4
4/24/2018 6:58					7.2	3.6	0.1857	2.3
4/24/2018 6:59					7.5	3.6	0.1888	2.4
4/24/2018 7:00					7.5	3.6	0.1737	2.4
4/24/2018 7:01					7.5	3.6	0.1906	2.5
4/24/2018 7:02					7.9	3.7	0.1879	2.4
4/24/2018 7:03					7.6	3.7	0.1912	2.4
4/24/2018 7:04					7.7	3.7	0.1956	2.4
4/24/2018 7:05					7.9	3.8	0.1912	2.3
4/24/2018 7:06					8.2	3.8	0.1995	2.2
4/24/2018 7:07					8.7	3.9	0.1859	2
4/24/2018 7:08					8.7	3.9	0.2009	2
4/24/2018 7:09					8.6	3.9	0.195	2
4/24/2018 7:10					8.1	4	0.1937	2
4/24/2018 7:11					7.5	4	0.1967	2
4/24/2018 7:12					7.2	4	0.1788	2
4/24/2018 7:13					7.4	4	0.2018	2
4/24/2018 7:14					7.3	4.1	0.1878	2.1
4/24/2018 7:15					7.3	4.1	0.2024	2
4/24/2018 7:16					7.5	4.1	0.1896	2
4/24/2018 7:17					7.4	4.1	0.1928	2
4/24/2018 7:18	0.2	-2.4	4.3	16.8	7.5	4.1	0.2007	2
4/24/2018 7:19	0.2	-2.5	3	16.9	7.6	4.1	0.1966	1.7
4/24/2018 7:20	0.2	-1.7	2.2	17.1	7.4	4.1	0.2323	1.6
4/24/2018 7:21	0.2	-0.7	2.3	16.8	7.8	4.1	0.2234	1.6
4/24/2018 7:22	0.3	-1	3.8	16.8	7.8	4.2	0.2256	1.6
4/24/2018 7:23	0.3	-0.3	2.9	17	7.9	4.2	0.2357	1.7
4/24/2018 7:24	0.3	-0.2	2	17	7.7	4.2	0.2262	1.6
4/24/2018 7:25	0.3	-0.4	3.1	17	7.8	4.2	0.2399	1.7
4/24/2018 7:26	0.3	-0.2	1.5	16.9	8.3	4.3	0.2235	1.6
4/24/2018 7:27	0.3	-0.7	3.9	17.1	8.2	4.3	0.2439	1.7
4/24/2018 7:28	0.4	0.4	2.5	17	8.4	4.4	0.2351	1.7
4/24/2018 7:29	0.4	-0.1	2.1	16.9	8.8	4.4	0.2354	1.7
4/24/2018 7:30	0.4	0.3	2.7	17.1	9	4.4	0.2439	1.7
4/24/2018 7:31	0.4	0.2	3.4	16.7	9	4.5	0.2312	1.7
4/24/2018 7:32	0.4	-0.1	3.5	16.8	9.5	4.5	0.2464	1.7
4/24/2018 7:33	0.4	0.1	2.6	16.8	9.1	4.5	0.1507	1.7
4/24/2018 7:34	0.4	-0.5	2.3	16.8	9	4.6	0.0061	1.7
4/24/2018 7:35	0.4	-0.2	2.1	16.8	8.5	4.5	0.0067	1.7
4/24/2018 7:36	0.4	0.2	3	17	8.3	4.5	-0.0025	1.7
4/24/2018 7:37	0.4	0.7	3.1	17.1	8.2	4.6	0.0328	1.7
4/24/2018 7:38	0.4	0.2	3.1	16.8	8.1	4.6	0.1693	1.7
4/24/2018 7:39	0.4	0.4	3.1	17.1	7.9	4.6	0.2115	1.7
4/24/2018 7:40	0.4	-0.1	2.1	17	7.9	4.6	0.2355	1.7
4/24/2018 7:41	0.4	-0.6	3.1	17.1	7.7	4.5	0.2316	1.7
4/24/2018 7:42	0.3	-0.4	2	17.2	7.8	4.6	0.2385	1.7

4/24/2018 7:43	0.3	-0.1	3.3	17	7.7	4.6	0.2231	1.7
4/24/2018 7:44	0.3	0.6	3.7	17.3	7.8	4.6	0.2391	1.7
4/24/2018 7:45	0.4	0.4	3.8	17.1	8.1	4.6	0.2311	1.7
4/24/2018 7:46	0.4	0	1.4	17.2	8	4.6	0.2319	1.7
4/24/2018 7:47	0.4	0	3.3	17.2	7.8	4.6	0.2347	1.7
4/24/2018 7:48	0.4	0.2	4.5	16.9	7.9	4.6	0.2235	1.7
4/24/2018 7:49	0.4	0.7	2.8	17.3	8.1	4.6	0.2381	1.7
4/24/2018 7:50	0.4	0.4	3.3	16.9	8.4	4.6	0.2315	1.7
4/24/2018 7:51	0.4	0.6	2.8	17.2	8.2	4.6	0.2346	1.7
4/24/2018 7:52	0.4	0.3	4	17	8.3	4.6	0.2198	1.7
4/24/2018 7:53	0.4	0.8	3.8	17.1	8.4	4.6	0.1999	1.6
4/24/2018 7:54	0.4	0.4	2.7	17	8.7	4.6	0.2173	1.6
4/24/2018 7:55	0.4	0.3	1.7	17.2	8.5	4.6	0.2045	1.6
4/24/2018 7:56	0.4	0	3	17.2	8.4	4.6	0.2051	1.7
4/24/2018 7:57	0.4	0.2	4	17	8.5	4.6	0.2029	1.6
4/24/2018 7:58	0.4	0.6	1.5	17.2	8.3	4.6	0.2074	1.6
4/24/2018 7:59	0.4	0.1	3	16.9	8.1	4.6	0.2094	1.6
4/24/2018 8:00	0.3	-0.2	3	17.2	8.3	4.6	0.2025	1.6
4/24/2018 8:01	0.3	0.5	2.5		8.2	4.5	0.2084	1.6
4/24/2018 8:02	0.3	-0.1	4.5		8.1	4.6	0.1945	1.6
4/24/2018 8:03	0.7	1.4	5.9		8.1	4.6	0.2059	1.6
4/24/2018 8:04	0.8	0.5	2.9		8.1	4.6	0.2035	1.7
4/24/2018 8:05	0.8	0.7	2.8	1.2	8.2	4.6	0.2024	1.6
4/24/2018 8:06	0.5	0.6	2.1	0.7	8.2	4.6	0.2094	1.6
4/24/2018 8:07	0.3	0.2	1.9	0.7	8	4.6	0.1932	1.6
4/24/2018 8:08	0.3	0.5	2.1	1.4	8	4.6	0.2183	1.6
4/24/2018 8:09	0.2	0.6	4.1	1.8	8	4.6	0.2187	1.6
4/24/2018 8:10	0.2	0.4	2.7	2	8	4.7	0.2037	1.6
4/24/2018 8:11	0.2	0.2	4	2.2	8	4.7	0.2138	1.6
4/24/2018 8:12	0.2	0.6	3.1	2.6	8	4.7	0.2051	1.6
4/24/2018 8:13	0.3	0.2	2.9	3	8.4	4.7	0.2127	1.6
4/24/2018 8:14	0.3	0.5	3.6	3	8.3	4.7	0.2049	1.6
4/24/2018 8:15	0.3	0.1	4.2	3.2	8.1	4.7	0.217	1.6
4/24/2018 8:16	0.3	0	2.2	3.4	8.2	4.6	0.2111	1.7
4/24/2018 8:17	0.3	0.1	2.8	3.3	8.1	4.6	0.2108	1.6
4/24/2018 8:18	0.3	0.6	2.7	3.4	7.8	4.6	0.2146	1.7
4/24/2018 8:19	0.3	0.6	2.6	3.8	7.6	4.6	0.2039	1.7
4/24/2018 8:20	0.2	0.6	2.9	3.6	7.7	4.6	0.2191	1.7
4/24/2018 8:21	0.3	0.1	3.8	3.9	7.7	4.6	0.2097	1.7
4/24/2018 8:22	0.3	0.7	3.7	5.7	7.7	4.7	0.223	1.7
4/24/2018 8:23	0.3	1.3	4.3	4.1	7.7	4.7	0.2086	1.7
4/24/2018 8:24	0.4	-0.2	3	4.3	7.7	4.7	0.217	1.7
4/24/2018 8:25	0.4	-0.1	3.6	4.3	7.8	4.8	0.2179	1.7
4/24/2018 8:26	0.5	0.8	2.9	4.2	7.8	4.8	0.2124	1.7
4/24/2018 8:27	0.5	0.8	3.9	4.6	7.9	4.8	0.2201	1.7
4/24/2018 8:28	0.6	0.4	3.5	4.3	7.9	4.7	0.2122	1.7
4/24/2018 8:29	0.6	0.9	1.6	4.7	7.9	4.7	0.2174	1.7

4/24/2018 8:30	0.6	0.8	3.5	4.6	7.9	4.7	0.219	1.7
4/24/2018 8:31	0.6	0.5	2.7	4.9	7.9	4.7	0.2186	1.8
4/24/2018 8:32	0.6	0.9	2.9	4.8	8	4.6	0.2192	1.7
4/24/2018 8:33	0.6	1	3.7	4.7	8	4.6	0.2138	1.7
4/24/2018 8:34	0.6	1.6	3.6	4.8	8	4.6	0.2187	1.8
4/24/2018 8:35	0.6	1.5	3.4	4.8	8	4.5	0.2154	1.8
4/24/2018 8:36	0.6	0.7	2.7	4.6	8.1	4.5	0.2269	1.8
4/24/2018 8:37	0.6	0.8	2.8	4.7	8.1	4.5	0.2137	1.8
4/24/2018 8:38	0.6	0.6	3.7	4.9	8.1	4.5	0.2186	1.8
4/24/2018 8:39	0.6	1.1	4.8	5	8.1	4.4	0.2175	1.8
4/24/2018 8:40	0.7	1.8	2.5	4.7	8.1	4.4	0.2186	1.8
4/24/2018 8:41	0.7	0.5	3.2	4.8	8.1	4.4	0.2239	1.8
4/24/2018 8:42	0.7	1.1	3.3	5	8.2	4.4	0.2095	1.8
4/24/2018 8:43	0.7	0.7	4.1	4.9	8.2	4.4	0.2203	1.8
4/24/2018 8:44	0.6	1.2	3.7	5	8.2	4.4	0.2126	1.8
4/24/2018 8:45	0.6	0.8	4.1	4.8	8.2	4.3	0.2166	1.8
4/24/2018 8:46	0.6	1.2	2.9	4.7	8.2	4.3	0.2059	1.8
4/24/2018 8:47	0.6	0.2	3.1	4.9	8.2	4.3	0.2105	1.8
4/24/2018 8:48	0.6	1	2.8	4.9	8.2	4.3	0.2179	1.9
4/24/2018 8:49	0.6	1.3	3.9	4.9	8.3	4.3	0.207	1.9
4/24/2018 8:50	0.6	0.6	4.3	5.2	8.3	4.2	0.2161	1.8
4/24/2018 8:51	0.6	0.6	3.2	5	8.3	4.2	0.2104	1.9
4/24/2018 8:52	0.7	0.9	4.7	4.9	8.3	4.2	0.2239	1.9
4/24/2018 8:53	0.8	0.8	2.7	5.1	8.3	4.2	0.2151	1.9
4/24/2018 8:54	0.8	1.3	2.6	5.1	8.3	4.2	0.2185	2
4/24/2018 8:55	0.8	0.7	4	5.1	8.3	4.2	0.2147	2
4/24/2018 8:56	0.8	0.1	3.7	5	8.3	4.1	0.2124	2
4/24/2018 8:57	0.8	0.5	2.4	5.3	8.3	4.1	0.2257	2
4/24/2018 8:58	0.8	1.2	3.5	4.9	8.3	4.1	0.2024	2.1
4/24/2018 8:59	0.8	1.3	3.2	5.1	8.3	4.1	0.2217	2
4/24/2018 9:00	0.8	0.7	3.1	4.9	8.3	4.1	0.2088	2
4/24/2018 9:01	0.8	1.5	3.7	5.2	8.3	4.1	0.2112	2
4/24/2018 9:02	0.8	1.2	3.1	4.9	8.3	4.1	0.2134	2.1
4/24/2018 9:03	0.9	1.5	3.4	5	8.3	4.1	0.2141	2.1
4/24/2018 9:04	0.9	0.9	4.2	4.9	8.3	4.1	0.2246	2.1
4/24/2018 9:05	0.9	0.6	4.5	5.1	8.3	4	0.2104	2.1
4/24/2018 9:06	0.9	1	3.3	5	8.3	4	0.2207	2.1
4/24/2018 9:07	0.9	0.8	3.6	5.2	8.3	4	0.2186	2.1
4/24/2018 9:08	0.9	0.8	4	5.3	8.3	4	0.2058	2.1
4/24/2018 9:09	1	1.5	4.4	5.2	8.3	4	0.2163	2.1
4/24/2018 9:10	0.9	1.3	4.3	5.2	8.2	4	0.2124	1.9
4/24/2018 9:11	0.9	1.1	2.7	5	8.3	4	0.2191	1.8
4/24/2018 9:12	0.9	1.5	5.1	5	8.3	4	0.1993	1.9
4/24/2018 9:13	0.9	1.1	3.6	5.1	8.2	4	0.2182	1.9
4/24/2018 9:14	0.9	1.7	2.8	5.3	8.2	3.9	0.2167	1.9
4/24/2018 9:15	1	1.2	3.9	5.1	8.1	3.9	0.2124	1.9
4/24/2018 9:16	1	0.8	2.4	5.2	8.1	3.9	0.2123	1.9

4/24/2018 9:17	1	1.1	3.2	5.3	8.2	3.9	0.2059	1.9
4/24/2018 9:18	1	1.7	3.9	5.4	8.1	3.8	0.2143	1.9
4/24/2018 9:19	1	0.8	3.6	5.2	8.1	3.8	0.2056	2
4/24/2018 9:20	1.1	1.6	3.2	5.2	8.1	3.8	0.2134	2
4/24/2018 9:21	1.1	1.8	3.4	5.4	8.1	3.8	0.2187	2.1
4/24/2018 9:22	1.1	1.6	2.8	5.1	8.1	3.8	0.2144	2.2
4/24/2018 9:23	1.1	1.4	3.5	5.2	8	3.8	0.2204	2.2
4/24/2018 9:24	1.1	1.5	3.1	5.4	8	3.7	0.2139	2.2
4/24/2018 9:25	1.2	1.7	2.1	5.4	8	3.7	0.2155	2.2
4/24/2018 9:26	1.2	1.5	2.6	5.5	8	3.7	0.2001	2.2
4/24/2018 9:27	1.2	0.9	3.6	5.5	8	3.7	0.2209	2.1
4/24/2018 9:28	1.2	0.9	3.8	5.3	7.9	3.6	0.21	2.1
4/24/2018 9:29	1.3	1.6	4.1	5.5	8	3.6	0.2233	2.1
4/24/2018 9:30	1.3	2	2.8	5.7	8	3.6	0.2172	2.1
4/24/2018 9:31	1.4	0.9	2.4	5.4	8.1	3.6	0.2011	2
4/24/2018 9:32	1.3	1.9	4	5.5	8	3.6	0.2175	2
4/24/2018 9:33	1.3	0.8	4.1	5.4	8	3.6	0.2129	2
4/24/2018 9:34	1.3	1.3	3.6	5.1	8	3.6	0.2143	2
4/24/2018 9:35	1.3	0.8	4.1	4.9	7.9	3.5	0.2192	2.1
4/24/2018 9:36	1.2	1.4	3	5.3	7.9	3.5	0.2029	2.2
4/24/2018 9:37	1.2	0.7	3	5.1	7.9	3.5	0.2218	2.2
4/24/2018 9:38	1.1	2.6	3	5.1	7.9	3.5	0.2008	2.1
4/24/2018 9:39	1.1	1	4.7	5.3	7.9	3.5	0.2116	2
4/24/2018 9:40	1.1	1.4	3.5	5.3	7.9	15.6	0.2896	2
4/24/2018 9:41	0.8	0.5	4.7	6.5	29.2	62.2	0.539	1.9
4/24/2018 9:42	0.6	1	7.6	9.8	39.5	107.6	1.1965	1.9
4/24/2018 9:43	0.3	0.7	8.2	11.4	43.8	120.2	1.7298	2.2
4/24/2018 9:44	0.2	0.2	8.5	14	44.6	123	2.3782	2.3
4/24/2018 9:45	0.2	0.9	9.1	14.5	44.2	122.6	2.6979	2.5
4/24/2018 9:46	0.2	-0.1	10.1	15.1	43.3	121.2	3.0046	2.5
4/24/2018 9:47	0.2	1.1	9.3	15	42.3	121.2	3.0894	2.6
4/24/2018 9:48	0.2	0.4	8.3	15.1	41.4	121	3.2103	2.6
4/24/2018 9:49	0.2	-0.3	9.1	14.9	40.6	120.7	3.3725	2.7
4/24/2018 9:50	0.2	-0.1	9.2	15.2	39.8	120.3	3.4988	2.7
4/24/2018 9:51	0.2	-0.1		14.6	38.4	119.4	3.4411	2.7
4/24/2018 9:52	0.2	0.3	17.2	14.4	37.9	118.3	3.496	2.7
4/24/2018 9:53	0.2	0.7	9.4	14.1	36.6	109.4	3.4568	2.6
4/24/2018 9:54	0.1	0.7	9	13.7	36	109.7	3.4301	2.7
4/24/2018 9:55	0.1	0.5	8.3	13.8	35.5	108.2	3.4214	2.7
4/24/2018 9:56	0.1	0.9		13.5	34.6	106.5	3.3596	2.7
4/24/2018 9:57	0.1	-0.2	8.3	13.5	33.8	106	3.2968	2.6
4/24/2018 9:58	0.1	0.4	8	13.6	33.2	105.4	3.2926	2.7
4/24/2018 9:59	0.1	0.2	6.9	13	32.4	104.5	3.2011	2.6
4/24/2018 10:00	0.1	0.2		13	31.8	103.4	3.1758	2.5
4/24/2018 10:01	0.1	1.3		12.7	31.2	102.5	3.1413	2.7
4/24/2018 10:02	0.1	0.6		12.5	30.9	99.6	3.1171	2.7
4/24/2018 10:03	0.1	-0.1	7.9	12.6	30.1	95.9	3.0794	2.7

4/24/2018 10:04	0.1	0.4		12.9	29.9	96	3.0533	2.7
4/24/2018 10:05	0.1	0.1	7.1	12.1	29.3	93.6	3.0113	2.8
4/24/2018 10:06	0.1	0.2	7.4	12.3	28.9	91	3.0023	2.8
4/24/2018 10:07	0.1	0.2	8.3	12	28.3	91	2.931	2.7
4/24/2018 10:08	0.1	0.4	7.9	12.2	27.9	91	2.9367	2.7
4/24/2018 10:09	0.1	0.9	6.8	12	27.7	91	2.9155	2.8
4/24/2018 10:10	0.1	0.3	7.7	11.9	27.3	90.8	2.9095	2.7
4/24/2018 10:11	0.1	-0.1	6.9	11.8	27.1	90.7	2.8893	2.8
4/24/2018 10:12	0.1	0.2	6.4	11.4	26	90.1	2.7871	3
4/24/2018 10:13	0.1	0.6	6.7	11.4	25.6	89.3	2.7384	3
4/24/2018 10:14	0.1	0.2	7.4	11.3	25.5	88.7	2.7725	3
4/24/2018 10:15	0.1	0.5	6.9	11.5	25.2	88	2.7538	3.2
4/24/2018 10:16	0.1	0.8	7.6	11.5	24.8	87.1	2.7143	3.2
4/24/2018 10:17	0.1	-0.2	5.4	11.1	24.4	86.1	2.7031	3.1
4/24/2018 10:18	0	0.4	5.9	10.7	24	85.4	2.6443	3.2
4/24/2018 10:19	0	0.2	7.2	11.3	23.8	84.4	2.6837	3.2
4/24/2018 10:20	0	0.2	7	10.5	23.3	83.6	2.6099	3.1
4/24/2018 10:21	0	0.6	6	11	23	83.2	2.6138	3.1
4/24/2018 10:22	0.1	0.5	7	10.8	22.8	82.7	2.6125	3
4/24/2018 10:23	0.1	0.4	5.6	10.6	22.2	81.4	2.5421	2.9
4/24/2018 10:24	0.1	0.5	6.2	10.8	21.8	80.4	2.5033	3
4/24/2018 10:25	0.1	0.8	6.7	10.7	21.5	79.6	2.4643	2.9
4/24/2018 10:26	0.1	0	5.9	10.6	21.3	78.7	2.4764	2.9
4/24/2018 10:27	0.1	0.4	6.4	10.1	21.1	77.7	2.4715	3
4/24/2018 10:28	0.1	0.1	6.8	10	20.4	76.7	2.3675	3
4/24/2018 10:29	0.2	0.2	6.6	10	19.9	75.3	2.3764	3
4/24/2018 10:30	0.2	0.3	6.7	10	20.3	74.3	2.4155	3
4/24/2018 10:31	0.2	-0.6	5.3	10.4	20	73.7	2.4062	3
4/24/2018 10:32	0.2	0	5.9	10	19.9	73.3	2.3982	3.1
4/24/2018 10:33	0.2	0.1	5.7	10	19.7	72.6	2.378	3.2
4/24/2018 10:34	0.2	0.3	5.9	10.1	19.5	72.2	2.3544	3
4/24/2018 10:35	0.2	0.5	4.8	10.2	19.3	71.8	2.3894	3
4/24/2018 10:36	0.2	0.2	4.3	9.6	18.9	71.4	2.3263	2.9
4/24/2018 10:37	0.2	-0.2	4	9.7	18.3	71	2.2322	3
4/24/2018 10:38	0.1	0.3	5.6	9.7	18.1	70.3	2.2549	3.1
4/24/2018 10:39	0.1	0.3	5.7	9.6	18.3	69.7	2.2482	3.2
4/24/2018 10:40	0.2	0.5	7.1	9.7	18	68.9	2.2557	3.1
4/24/2018 10:41	0.2	0.7	5.4	9.3	18	68.1	2.1776	3.2
4/24/2018 10:42	0.2	0.2	4.7	9.3	17.7	67.3	2.1934	3
4/24/2018 10:43	0.2	0.5	6.2	9.4	17.2	66.5	2.1478	3
4/24/2018 10:44	0.2	0	5.6	9.3	17.1	65.6	2.1623	2.8
4/24/2018 10:45	0.1	0.8	6.2	9.5	16.9	65.1	2.1667	2.9
4/24/2018 10:46	0.2	1.1	3.6	9.3	16.6	64.6	2.0819	3.1
4/24/2018 10:47	0.2	0.7	6.2	9.3	16.5	63.9	2.1175	3.1
4/24/2018 10:48	0.2	0.8	5.4	9	15.9	62.9	1.977	3.1
4/24/2018 10:49	0.2	0.1	5.4	9.1	16	62.1	2.0355	3
4/24/2018 10:50	0.2	0.3	6.3	9	16	61.6	2.0623	3

4/24/2018 10:51	0.2	0.6	4.8	9.1	15.8	60.9	2.0485	2.6
4/24/2018 10:52	0.2	0.5	5.5	9.1	15.7	60.2	2.0383	2.2
4/24/2018 10:53	0.2	0	4.6	9	15.5	59.7	2.0052	2.3
4/24/2018 10:54	0.2	0.5	4.7	8.7	15.2	59	1.9838	2.4
4/24/2018 10:55	0.2	0.3	3.9	8.9	15.2	58.6	1.9782	2.6
4/24/2018 10:56	0.2	0.7	5.4	8.8	14.9	58.1	1.9558	2.6
4/24/2018 10:57	0.2	0.2	5.4	9	14.6	57.8	1.8803	2.5
4/24/2018 10:58	0.2	0.8	3.9	8.5	14.6	57.1	1.8897	2.4
4/24/2018 10:59	0.3	0.5	4.9	8.4	14.2	56	1.8072	2.5
4/24/2018 11:00	0.3	0.2	5.2	8.3	14.4	55.4	1.8567	2.5
4/24/2018 11:01	0.3	0.4	5	8.3	14	54.6	1.8181	2.6
4/24/2018 11:02	0.3	0	5.9	8.5	14.2	54	1.8505	2.5
4/24/2018 11:03	0.3	-0.1	5	8.6	14.1	53.6	1.8563	2.4
4/24/2018 11:04	0.3	0.8	5.4	8.6	13.9	53.1	1.8235	2.4
4/24/2018 11:05	0.3	0.3	4.7	8.3	13.6	52.5	1.7655	2.4
4/24/2018 11:06	0.3	0.6	4.8	8.4	13.6	52	1.8123	2.4
4/24/2018 11:07	0.3	0.9	4.1	8.5	13.6	52	1.7885	2.1
4/24/2018 11:08	0.3	0.5	5	8.5	13.2	51.6	1.701	2.2
4/24/2018 11:09	0.3	0.7	4.5	8.2	13.1	51	1.7018	2.4
4/24/2018 11:10	0.3	0.4	4.7	8.2	13.2	50.4	1.7235	2.5
4/24/2018 11:11	0.3	1	5.2	8.1	13.8	49.8	1.6998	2.5
4/24/2018 11:12	0.4	0.8	5.6	8	13.6	48.8	1.5857	2.6
4/24/2018 11:13	0.4	0.7	4.4	8	13.7	48.1	1.6598	2.7
4/24/2018 11:14	0.5	0.8	4.5	8	14.2	47.1	1.5067	2.5
4/24/2018 11:15	0.5	0.5	4.6	8	15.6	45.8	1.5505	2.7
4/24/2018 11:16	0.6	0.6	5.1	8	16.2	44.4	1.5138	2.8
4/24/2018 11:17	0.6	0.7	5.1	8.1	16.8	43.3	1.4537	2.9
4/24/2018 11:18	0.6	1.1	4.9	7.8	17	42.1	1.4043	3
4/24/2018 11:19	0.6	0.5	3.8	7.9	17.5	40.6	1.4067	3.2
4/24/2018 11:20	0.7	1.3	5	8	17	30.6	1.2762	2.8
4/24/2018 11:21	1.1	2.5	5.1	9	15.4	27.7	1.157	2.4
4/24/2018 11:22	2.7	8.3	7.1	12.7	14.1	14.2	0.7577	2.3
4/24/2018 11:23	5.3	11.1	6.3	15.3	10.8	6.8	0.47	2.4
4/24/2018 11:24	8.1	13.8	9.9	16.8	9.5	6.4	0.3942	2.5
4/24/2018 11:25	10.3	15	10	17.6	8.5	5.9	0.342	2.4
4/24/2018 11:26	11.6	15.3	10.3	18.6	7.8	5.4	0.3131	2.3
4/24/2018 11:27	12.6	16.4	10.2	19.6	7.7	4.8	0.31	2.2
4/24/2018 11:28	13.6	16.6	11.9	19.9	7.2	4.3	0.2792	2.3
4/24/2018 11:29	14.3	17.3	11.4	20.5	6.9	3.7	0.288	2.4
4/24/2018 11:30	14.8	16.8	11.7	20	7.2	3.2	0.2834	2.4
4/24/2018 11:31	15	16.9	11.5	20.1	7.5	2.6	0.2821	2.4
4/24/2018 11:32	15.3	18.6	10.5	21.1	7.7	2.2	0.2649	2.4
4/24/2018 11:33	15.7	18.8	12.4	21.2	7.5	2	0.2567	2.5
4/24/2018 11:34	16.1	19.4	12.9	21.8	7.5	1.9	0.26	2.5
4/24/2018 11:35	16.7	19.1	12.6	22.1	7.2	1.8	0.2458	2.5
4/24/2018 11:36	17.3	19.4	13.9	23	7	1.7	0.2481	2.3
4/24/2018 11:37	17.4	17.1	10.6	20.8	10.9	1.7	0.3303	2.3

4/24/2018 11:38	15.9	11	7	15.2	59.6	3.9	0.7301	2.7
4/24/2018 11:39	12.2	8.8	6.8	13	82.4	10.2	1.2697	3.4
4/24/2018 11:40	8.9	7.8	6.9	12.6	74.5	9	1.1075	3.3
4/24/2018 11:41	6.7	6	7.7	11.5	81.7	10	1.2478	3.5
4/24/2018 11:42	5.9	5.2	6.3	10.5	81.7	10.4	1.2905	3.6
4/24/2018 11:43	4.8	3.5	4.9	9.1	101.5	27.6	1.3803	3.6
4/24/2018 11:44	3.1	1.1	5.5	8.6	140	95.6	1.837	3.6
4/24/2018 11:45	1.5	0.6	7.2	9.9	156.8	158.6	2.3348	3.6
4/24/2018 11:46	0.4	1.2	8.7	13.3	162.5	197.4	3.0128	3.7
4/24/2018 11:47	0.2	1.2	11.9	18.3	164.6	198.9	3.5432	4
4/24/2018 11:48	0.1	-0.2	12.2	21.4	163.1	199.6	4.3863	4.3
4/24/2018 11:49	0.2	0.6	12.5	21.2	162.7	206.6	4.5523	4.3
4/24/2018 11:50	0.1	1.1	12.4	21.9	161.3	205.7	4.8215	4.5
4/24/2018 11:51	0.1	0.1	13.1	21.6	156.8	198.8	5.0885	4.5
4/24/2018 11:52	0.1	0.6	13.3	21	155.5	191.1	5.3283	4.6
4/24/2018 11:53	0.1	0.6	12.3	21.9	162.2	194.9	5.797	4.8
4/24/2018 11:54	0.1	0.8	14.5	21.2	159	191.3	5.83	4.9
4/24/2018 11:55	0.1	0.7	11.4	21.5	165.1	194.3	6.133	5
4/24/2018 11:56	0.1	0.5	12.4	21.2	166.9	192.4	6.3493	5.2
4/24/2018 11:57	0.1	0.4	12.4	20.1	157.1	181.8	6.3102	5.1
4/24/2018 11:58	0.1	0.4	12.6	19.6	158.5	180.3	6.4057	5.1
4/24/2018 11:59	0.1	0.5	12.3	19.2	157.2	176.6	6.3872	5.2
4/24/2018 12:00	0.1	0	9.9	19.2	158.5	177.8	6.5733	5.2
4/24/2018 12:01	0.1	0.5	10.6	18.7	152.2	177.1	6.4018	5.2
4/24/2018 12:02	0.1	1.3	11.2	18.3	154.7	172.9	6.5347	5.2
4/24/2018 12:03	0.1	0.2	11.1	18.8	157.7	176.9	6.8058	5.4
4/24/2018 12:04	0.1	0.9	10.9	18.2	155.4	173.5	6.654	5.4
4/24/2018 12:05	0.2	0.7	10.3	17.9	155.4	176.8	6.8487	5.5
4/24/2018 12:06	0.2	1.2	10.5	17.2	150.8	167.6	6.684	5.5
4/24/2018 12:07	0.2	0.6	10.6	17	148	165.7	6.5433	5.5
4/24/2018 12:08	0.2	0.3	10.4	17.5	153.4	169.3	6.7448	5.6
4/24/2018 12:09	0.2	0.7	9.5	16.7	146.5	167.5	6.708	5.6
4/24/2018 12:10	0.2	0.2	9.6	16	137.1	158.2	6.1703	5.5
4/24/2018 12:11	0.2	0.1	9.8	16	143.9	157.4	6.481	5.7
4/24/2018 12:12	0.2	0.4	10.1	16.2	142	154.6	6.3872	5.7
4/24/2018 12:13	0.2	0.3	10.4	15.7	142.6	157.9	6.458	5.8
4/24/2018 12:14	0.2	0.5	8.9	15.7	143.7	155.9	6.5177	5.7
4/24/2018 12:15	0.2	0.8	9.6	15.3	136	148.5	6.1842	5.4
4/24/2018 12:16	0.2	0.7	8.1	15.1	135.6	152.1	6.2603	5.3
4/24/2018 12:17	0.2	1	7.6	14.4	128.6	142.5	5.989	5.1
4/24/2018 12:18	0.2	0.3	8.5	14.2	126.8	139.3	6.023	5
4/24/2018 12:19	0.2	0.4	7.5	13.8	122	138.1	5.848	4.9
4/24/2018 12:20	0.2	1.2	7.5	13.3	118.8	130.1	5.6822	4.9
4/24/2018 12:21	0.2	0.8	7.2	13.3	117.8	125.5	5.499	5
4/24/2018 12:22	0.3	0.7	8.8	12.9	115.7	122.9	5.3763	4.9
4/24/2018 12:23	0.3	-0.4	7.2	12.6	111.3	120.4	5.2028	4.8
4/24/2018 12:24	0.3	1.2	6	12.7	111.3	122.1	5.3492	4.8

4/24/2018 12:25	0.3	1.4	8.8	12	104	116	5.0835	4.8
4/24/2018 12:26	0.3	0.6	7.9	12.7	106.6	113.8	5.2142	4.9
4/24/2018 12:27	0.3	0.4	7.7	12.9	113.1	125.9	5.4595	5.1
4/24/2018 12:28	0.3	0.9	7.9	13.5	124.5	134.2	5.7367	5.3
4/24/2018 12:29	0.3	0.3	9.8	13.9	126.7	137.5	6.0148	5.5
4/24/2018 12:30	0.3	0.8	8.2	13.8	127.9	139	6.0848	5.7
4/24/2018 12:31	0.3	0.9	8.3	13.7	128.8	141.7	6.137	5.8
4/24/2018 12:32	0.3	0.3	8.7	13.3	126.4	141.6	6.0528	5.9
4/24/2018 12:33	0.3	0.5	8.5	13.4	124.7	140.9	6.0452	5.9
4/24/2018 12:34	0.2	0.7	8.7	13.4	123.5	140.3	5.9185	6
4/24/2018 12:35	0.2	0.8	7.6	13	120.5	136.9	5.8458	6
4/24/2018 12:36	0.2	1.2	7.6	13	117.6	133.2	5.7513	5.8
4/24/2018 12:37	0.2	0.6	8.4	12.2	111.8	124.5	5.4475	5.7
4/24/2018 12:38	0.2	0.6	8	11.6	103.9	116.1	5.2095	5.6
4/24/2018 12:39	0.2	0.5	7	11.6	102.3	114.6	5.2238	5.6
4/24/2018 12:40	0.2	0	8.4	11.9	98.8	111.4	5.1035	5.6
4/24/2018 12:41	0.2	0.7	6.1	11.4	95.8	107.2	4.9342	5.6
4/24/2018 12:42	0.2	0.8	6.3	11.2	97.5	103.8	4.7587	5.5
4/24/2018 12:43	0.2	0.5	6.8	12.3	98.7	103.6	5.145	5.8
4/24/2018 12:44	0.2	0.5	7.3	10.9	93	107.5	4.8193	5.7
4/24/2018 12:45	0.2	0.4		11.2	96.6	111.1	4.8338	5.7
4/24/2018 12:46	0.2	1	8.9	11.6	102.8	112.8	5.1395	5.9
4/24/2018 12:47	0.2	0.7		11.7	100.7	112.9	5.1528	5.9
4/24/2018 12:48	0.2	0.6	6.4	11.8	101.4	116.1	5.3115	6.1
4/24/2018 12:49	0.2	0.7	7.5	11.9	102.9	118.2	5.34	6.1
4/24/2018 12:50	0.1	0.4		11.6	105.2	119.5	5.4442	6.2
4/24/2018 12:51	0.1	1	6.6	11.8	104.7	120.4	5.451	6.2
4/24/2018 12:52	0.1	0.7	7	11.7	104.2	120.3	5.4365	6.2
4/24/2018 12:53	0.1	1		11.6	102.8	120.2	5.399	6.3
4/24/2018 12:54	0.2	1.2		11.7	101.3	120	5.3232	6.4
4/24/2018 12:55	0.2	0.5	6.2	11.2	97.2	112.7	5.1447	6.2
4/24/2018 12:56	0.2	-0.4	14.6	10.9	93.3	109	5.0285	6.1
4/24/2018 12:57	0.1	0.8		11.2	94.9	112.3	5.1205	6.1
4/24/2018 12:58	0.2	0.9	6.1	10.4	87	103.6	4.7338	5.8
4/24/2018 12:59	0.2	0.5	8.5	10.4	80.4	93.4	4.433	5.6
4/24/2018 13:00	0.2	0.6	4.9	10.5	84.4	98	4.6457	5.7
4/24/2018 13:01	0.2	0.4	6.9	9.7	73.2	89.6	4.2275	5.6
4/24/2018 13:02	0.3	1.5	5.3	9.3	65.8	81.8	3.8225	5.3
4/24/2018 13:03	0.4	1.2	3.7	9.1	58.6	69.7	3.194	5
4/24/2018 13:04	0.5	0.9	4.9	9.7	61.9	80.4	3.8288	5.2
4/24/2018 13:05	1.2	4.5	6.7	10.8	44.1	41.3	2.2535	4.1
4/24/2018 13:06	2.6	6.9	5.7	11.9	32.2	18.3	1.38	3.3
4/24/2018 13:07	4.6	9.2	7	13.2	22.8	9.9	0.931	3
4/24/2018 13:08	6.4	9.6	7.8	13.8	20.1	7.3	0.7677	2.8
4/24/2018 13:09	7.5	9.8	7.3	13.8	19.3	6.7	0.783	2.9
4/24/2018 13:10	8	10.1	7.6	14.5	19.1	6.8	0.7836	2.9
4/24/2018 13:11	8.2	10.4	7.4	14.7	18.5	6.8	0.8206	3

4/24/2018 13:12	8.6	12.3	8	15.5	16.9	6.8	0.7132	2.9
4/24/2018 13:13	8.9	11.5	7.8	15.6	17	6.8	0.7497	2.8
4/24/2018 13:14	9.2	11.9	9.5	15.9	16.5	6.8	0.7115	2.8
4/24/2018 13:15	9.4	11.6	9.1	15.9	16.9	6.7	0.738	2.7
4/24/2018 13:16	9.8	12.5	9.8	16.4	15.4	6.6	0.7742	2.5
4/24/2018 13:17	10.2	12.4	9.7	16.3	14.8	6.3	0.8134	2.4
4/24/2018 13:18	10.5	12	8.1	16.4	14.7	6.1	0.7408	2.4
4/24/2018 13:19	10.7	13.3	9.1	17	14.7	5.8	0.6619	2.4
4/24/2018 13:20	11.1	13.7	8.7	17.6	12.4	5.3	0.5402	2.4
4/24/2018 13:21	11.7	14.9	10.2	18.3	11.4	5	0.5088	2.6
4/24/2018 13:22	12.6	15.7	10	19.2	9.9	4.6	0.434	2.6
4/24/2018 13:23	13.1	15.5	10.8	18.7	9.9	4.1	0.4474	2.5
4/24/2018 13:24	13.4	15.8	10	19.3	9.8	3.7	0.4164	2.4
4/24/2018 13:25	13.6	16.2	9	19.4	10	3.4	0.403	2.4
4/24/2018 13:26	13.9	16.6	11.1	19.8	9.4	3.1	0.3978	2.4
4/24/2018 13:27	14.2	16.2	11.4	19.1	9.9	2.8	0.4194	2.4
4/24/2018 13:28	14.4	16.3	10.4	19.9	9.4	2.7	0.3976	2.4
4/24/2018 13:29	14.6	16.7	11	19.8	8.9	2.5	0.3743	2.4
4/24/2018 13:30	14.8	17.3	11.4	20.2	8.7	2.4	0.3689	2.1
4/24/2018 13:31	14.8	16.4	10.9	19.7	9.2	2.4	0.4085	2
4/24/2018 13:32	14.6	15.8	11.3	19.4	9.5	2.4	0.4352	2
4/24/2018 13:33	14.4	16.7	12.1	19.4	10.6	2.4	0.4536	2.1
4/24/2018 13:34	14.5	16.6	10.7	19.7	9.4	2.4	0.3975	2
4/24/2018 13:35	15.1	18.7	11.5	21.4	8.1	2.4	0.3508	2
4/24/2018 13:36	15.8	18.1	12.4	21.2	8.1	2.3	0.356	2
4/24/2018 13:37	16.3	17.9	12	21.4	8.2	2.3	0.3677	2
4/24/2018 13:38	16.5	18.8	10.5	21.2	7.6	2.3	0.341	2
4/24/2018 13:39	16.7	18.6	13.8	21.5	7.3	2.3	0.3295	2
4/24/2018 13:40	16.9	18.8	13.1	21.7	7	2.2	0.3139	2
4/24/2018 13:41	17	18.4	9.2	21.5	7.1	2.1	0.3089	2
4/24/2018 13:42	17.1	18.6	12.2	21.6	7.3	2	0.3234	2
4/24/2018 13:43	17	18.9	11.5	21.7	7.8	1.9	0.3084	2.1
4/24/2018 13:44	17	19.4	12	21.7	7.8	1.9	0.3161	2.1
4/24/2018 13:45	17.2	19.1	11.8	22.1	7.6	1.8	0.3036	2.2
4/24/2018 13:46	17.5	19.5	11.5	22.5	6.6	1.8	0.2886	2.2
4/24/2018 13:47	17.9	20.2	12.6	22.1	6.4	1.7	0.2898	2.3
4/24/2018 13:48	18.2	21.3	13.1	23.6	5.5	1.7	0.2514	2.4
4/24/2018 13:49	18.8	21.4	12.3	23.8	5.1	1.6	0.2444	2.5
4/24/2018 13:50	19	20.9	13.2	23.1	5.2	1.6	0.2506	2.5
4/24/2018 13:51	18.5	18.2	11.3	21.5	7.9	1.6	0.3321	2.7
4/24/2018 13:52	16.9	14.9	9.6	18.8	15.1	1.8	0.53	3.2
4/24/2018 13:53	14.9	14.3	10.7	18.1	18.3	2.1	0.6173	3.5
4/24/2018 13:54	13.5	14.9	9.9	18	17.5	2.4	0.5599	3.4
4/24/2018 13:55	13	13.9	10	17.5	16.5	2.6	0.5396	3.2
4/24/2018 13:56	12.7	13.6	8.8	16.7	15.2	2.8	0.5145	3.2
4/24/2018 13:57	12.5	13.4	8.9	17.2	15.5	3.1	0.5224	3.4
4/24/2018 13:58	12.3	13.6	8.2	16.8	16	3.4	0.524	3.4

4/24/2018 13:59	12	12.2	8.8	16.3	14.7	3.6	0.5118	3.4
4/24/2018 14:00	11.7	12.7	8.7	16.5	14.4	3.7	0.4911	3.4
4/24/2018 14:01	11.5	12.6	9.4	15.8	14	3.6	0.5094	3.3
4/24/2018 14:02	11.3	11.7	8.1	15.3	15.5	3.6	0.5498	3.5
4/24/2018 14:03	11.3	14	8.9	16.8	14.9	3.6	0.5533	3.5
4/24/2018 14:04	12.5	17.5	12.7	20	10.9	3.6	0.4579	3.1
4/24/2018 14:05	14.6	20.7	11.9	22.3	8.5	3.4	0.3569	2.7
4/24/2018 14:06	17.1	20.1	13.9	22.8	7.5	3.3	0.347	2.5
4/24/2018 14:07	18.4	20	12.9	22.8	7.4	3.1	0.3404	2.4
4/24/2018 14:08	18.8	20	12.8	22.8	7.2	2.9	0.3309	2.3
4/24/2018 14:09	19	21.3	13.5	23.6	6.8	2.7	0.3182	2.2
4/24/2018 14:10	19.6	22.4	14.4	24.6	5.9	2.5	0.2754	2.1
4/24/2018 14:11	20.3	23.2	13.4	24.9	5.3	2.2	0.2588	2
4/24/2018 14:12	21.1	22.7	14	24.9	5.1	1.9	0.2493	2
4/24/2018 14:13	21.3	22.4	15.2	24.6	5.2	1.7	0.2566	2
4/24/2018 14:14	21.4	22.6	13.4	25.1	5.1	1.7	0.2491	2.1
4/24/2018 14:15	21.4	22.9	14.9	25.1	5	1.6	0.2376	2.1
4/24/2018 14:16	21.5	23.3	14.9	25.3	4.8	1.5	0.2499	2.2
4/24/2018 14:17	21.5	23.1	13	24.8	4.9	1.4	0.2338	2.1
4/24/2018 14:18	21.4	23.1	15.5	25	4.9	1.3	0.2478	2.1
4/24/2018 14:19	21.5	24.4	14.9	25.7	4.7	1.3	0.232	2.1
4/24/2018 14:20	22.1	24.9	15.3	27	4.4	1.2	0.2179	2.2
4/24/2018 14:21	23	25.6	15.9	27.8	4.1	1.2	0.2104	2.2
4/24/2018 14:22	23.8	25.1	15.1	27.6	4	1.2	0.192	2.2
4/24/2018 14:23	23.6	22.7	12.9	24.6	5.4	1.2	0.2514	2.4
4/24/2018 14:24	21.9	19.2	13.9	21.8	10.1	1.3	0.3754	3
4/24/2018 14:25	19.7	18.8	11.9	21.8	11.7	1.5	0.3893	3.3
4/24/2018 14:26	18.1	18	11.1	21.2	11	1.7	0.3902	3.2
4/24/2018 14:27	17.2	17.2	11	20.2	11.1	2	0.3839	3.2
4/24/2018 14:28	15.3	10.2	7.3	14.6	28.1	14.3	0.5018	3.1
4/24/2018 14:29	11.6	5.7	6.1	11	38.8	28.5	0.7644	3.2
4/24/2018 14:30	6.8	1.4	5.2	10.3	46.6	37.4	1.2812	3.2
4/24/2018 14:31	3	1.8	5.7	10.4	49.9	39.9	1.5674	3.4
4/24/2018 14:32	1.2	0.9	4.1	10.6	50.1	39.1	1.7478	3.5
4/24/2018 14:33	0.5	0.5	7	10.8	49.3	39.1	1.8653	3.6
4/24/2018 14:34	0.4	0.5	5.4	10.5	48.6	39	1.8833	3.6
4/24/2018 14:35	0.4	0.2	5.4	10.4	47.5	38.9	1.9409	3.6
4/24/2018 14:36	0.4	-0.1	6.9	10.2	47.2	38.7	1.9759	3.7
4/24/2018 14:37	0.4	0.8	5.8	10.6	46.4	38.5	2.0035	3.6
4/24/2018 14:38	0.3	0.6	5.5	10.1	45.5	38.2	2.0009	3.5
4/24/2018 14:39	0.3	0.5	5.8	9.9	44.7	37.8	1.9731	3.6
4/24/2018 14:40	0.3	0.9	3.7	9.9	44.3	37.3	2.0078	3.6
4/24/2018 14:41	0.3	0.7	5	9.8	44.3	36.8	2.008	3.7
4/24/2018 14:42	0.3	1.1	4.5	9.7	43.4	36.4	2.0043	3.7
4/24/2018 14:43	0.3	0.2	4.9	9.9	43	35.9	1.9934	3.8
4/24/2018 14:44	0.3	0.8	5.1	9.8	43.3	35.5	2.0043	3.8
4/24/2018 14:45	0.3	0.6	5.3	9.7	43.2	35.1	1.9956	4

4/24/2018 14:46	0.3	0.7	4.3	9.5	43.1	34.8	1.9978	4
4/24/2018 14:47	0.3	0.7	5.7	9.7	42.4	34.5	1.9978	4.1
4/24/2018 14:48	0.3	0.4	4.6	9.7	41.9	34.1	1.965	4.1
4/24/2018 14:49	0.3	0.6	3.7	9.6	41.5	33.9	1.9591	4.2
4/24/2018 14:50	0.2	0.5	4.7	9.7	41.1	33.5	1.9423	4.3
4/24/2018 14:51	0.2	0.8	5.9	9.7	41	33.3	1.9557	4.3
4/24/2018 14:52	0.3	0.3	4	9.7	40.2	33	1.9183	4.3
4/24/2018 14:53	0.3	0.7	4.5	9.5	40.4	32.7	1.9195	4.3
4/24/2018 14:54	0.3	0.2	5.9	9.2	39.9	32.4	1.9136	4.2
4/24/2018 14:55	0.2	0.1	4.4	9.4	39.6	32	1.8954	4.3
4/24/2018 14:56	0.3	1	4.5	9.1	39	31.7	1.8646	4.3
4/24/2018 14:57	0.3	0.1	4.7	9.4	39.3	31.5	1.8903	4.2
4/24/2018 14:58	0.3	0.6	4.6	8.9	39	31.3	1.8894	4.1
4/24/2018 14:59	0.3	0.8	4.5	8.9	38.6	31	1.8754	4
4/24/2018 15:00	0.3	0.5	3.8	9.1	37.5	30.8	1.8169	4
4/24/2018 15:01	0.4	0.2	5.5	9	37.3	30.4	1.8119	4
4/24/2018 15:02	0.4	1.4	4.7	9	36.6	30	1.7794	4
4/24/2018 15:03	0.3	0.9	4.7	9	37.6	29.8	1.8533	4.2
4/24/2018 15:04	0.3	0.4	3.9	9	36.9	29.6	1.8064	4.2
4/24/2018 15:05	0.2	0.2	3.8	8.6	37.1	29.5	1.8308	4.3
4/24/2018 15:06	0.3	0.3	4.5	8.7	36.2	29.1	1.7769	4.3
4/24/2018 15:07	0.2	-0.1	4.2	8.8	36.2	28.8	1.7988	4.3
4/24/2018 15:08	0.2	0.8	3.8	8.9	35.8	28.7	1.7853	4.3
4/24/2018 15:09	0.2	0.4	4.1	8.7	35.1	28.4	1.7437	4.3
4/24/2018 15:10	0.3	0.9	3.6	8.5	35.4	28.3	1.7795	4.3
4/24/2018 15:11	0.3	1	3.4	8.6	35	28.1	1.7523	4.3
4/24/2018 15:12	0.3	0.3	4.1	8.6	35	27.9	1.772	4.3
4/24/2018 15:13	0.3	0.2	3.7	8.7	34.9	27.8	1.7683	4.2
4/24/2018 15:14	0.3	0.8	4.2	8.4	34.7	27.6	1.7672	4.2
4/24/2018 15:15	0.3	1.3	5	8.6	33.6	27.4	1.7145	4.1
4/24/2018 15:16	0.3	0.9	4.7	8.7	32.8	27.1	1.6685	4.1
4/24/2018 15:17	0.4	0.7	3.6	8.7	31.6	26.7	1.6117	4.1
4/24/2018 15:18	0.5	0.8	4.6	8.5	29.9	26.3	1.581	4
4/24/2018 15:19	0.6	0.9	3.9	8.9	28.9	25.6	1.496	3.9
4/24/2018 15:20	0.7	1.1	4.4	8.4	28.1	24.9	1.4913	3.9
4/24/2018 15:21	0.8	0.6	3	8.5	27.8	24.1	1.4773	4
4/24/2018 15:22	0.8	1.3	2.8	8.7	28.4	23.4	1.5002	4
4/24/2018 15:23	0.7	1.3	5.1	8.3	29.2	22.9	1.5725	4
4/24/2018 15:24	0.7	0.4	5.3	7.9	30.2	22.6	1.591	4.1
4/24/2018 15:25	0.6	0.8	4.9	8.2	29.5	22.4	1.5685	4.1
4/24/2018 15:26	0.5	0.9	4.4	8.1	30.1	22.3	1.6147	4.2
4/24/2018 15:27	0.5	1.3	2.9	8.2	29.2	22.4	1.5895	4.2
4/24/2018 15:28	0.4	0.5	4.5	8.2	29.5	22.6	1.625	4.2
4/24/2018 15:29	0.4	1.4	4	8.1	28.6	22.8	1.5673	4.2
4/24/2018 15:30	0.4	0.7	3.9	8	29.5	23	1.6133	4.2
4/24/2018 15:31	0.4	0.9	2.6	8	29.2	23.2	1.6045	4.3
4/24/2018 15:32	0.4	0.7	4.4	8.1	30.1	23.2	1.6183	4.3

4/24/2018 15:33	0.4	0.3	3.9	7.9	30	23.3	1.6277	4.3
4/24/2018 15:34	0.3	0.9	4.3	8	30.1	23.4	1.6218	4.2
4/24/2018 15:35	0.3	0.6	3.6	7.9	29.4	23.4	1.6142	4.2
4/24/2018 15:36	0.3	0.8	3.8	8.3	29	23.4	1.5858	4.1
4/24/2018 15:37	0.3	0.9	4.4	7.8	28.9	23.4	1.6027	4.1
4/24/2018 15:38	0.6	3.2	12.2	11.2	28.4	23.4	1.4465	4
4/24/2018 15:39	3.2	6.8	6.2	11.2	26.9	23.3	1.5352	4

USFS Missoula Chamber 2019

Date&Time	O3 NO-CL	O3 SL-UV	O3 UV-C	O3 UV-G	NO2-C	NO	CO	THC
4/15/2019 0:00	2.4	4.7	3.7733	2.1833	3.2565	17.5546	0.0557	1.7849
4/15/2019 0:01	2.4	4.7583	3.8427	2.1667	3.2661	17.7182	0.0698	1.7802
4/15/2019 0:02	2.4	4.7083	3.6295	1.65	3.2456	17.8667	0.0599	1.799
4/15/2019 0:03	2.4	5.1833	3.4703	2.3167	3.2587	17.7	0.0623	1.8106
4/15/2019 0:04	2.4	5.1333	3.8818	2.4333	3.2955	17.85	0.0675	1.7982
4/15/2019 0:05	2.4417	5	4.0138	2.2667	3.2715	18.1909	0.0509	1.7999
4/15/2019 0:06	2.5	4.1417	3.8568	1.7667	3.2659	18.41	0.0701	1.8183
4/15/2019 0:07	2.5	5.3	3.8643	2.2333	3.2708	18.5083	0.063	1.82
4/15/2019 0:08	2.4333	4.7167	3.9288	1.8667	3.2872	18.675	0.0653	1.8107
4/15/2019 0:09	2.4667	4.05	3.7363	1.8167	3.2938	18.525	0.0621	1.7904
4/15/2019 0:10	2.475	4.9833	3.9782	1.7833	3.2595	18.4182	0.0541	1.7946
4/15/2019 0:11	2.5	4.9083	3.814	2.75	3.2678	18.2182	0.0691	1.8343
4/15/2019 0:12	2.475	4.4417	3.8643	2.1667	3.2486	18.2833	0.056	1.8114
4/15/2019 0:13	2.4417	4.6	3.5945	2.15	3.2611	17.8727	0.0595	1.8232
4/15/2019 0:14	2.45	5.1917	3.9043	2.1333	3.2624	17.925	0.0702	1.8002
4/15/2019 0:15	2.475	4.2917	3.9752	2.45	3.2576	18.1818	0.0522	1.7944
4/15/2019 0:16	2.475	4.425	3.9165	1.75	3.2449	18.375	0.0683	1.781
4/15/2019 0:17	2.4667	4.875	3.8983	2.1	3.2184	18.3	0.0608	1.8131
4/15/2019 0:18	2.425	4.8917	3.5923	2.1167	3.2395	18.375	0.0607	1.8185
4/15/2019 0:19	2.475	4.9417	3.6193	2.2333	3.2321	18.49	0.065	1.8243
4/15/2019 0:20	2.4	4.925	3.7988	1.9667	3.2249	18.6091	0.0497	1.8206
4/15/2019 0:21	2.4	5.3167	3.827	2.1	3.214	18.7818	0.0624	1.8166
4/15/2019 0:22	2.4	4.3167	3.8112	2.0167	3.2145	18.9583	0.0557	1.8248
4/15/2019 0:23	2.4	4.7	3.6075	1.75	3.2496	19.0636	0.0653	1.8288
4/15/2019 0:24	2.3417	3.6333	4.0207	2.2167	3.2478	19.3583	0.0676	1.7988
4/15/2019 0:25	2.3083	4.9583	3.9507	1.7333	3.2603	19.7091	0.0525	1.7998
4/15/2019 0:26	2.325	5.0917	3.629	2.3333	3.2933	19.9	0.0636	1.8003
4/15/2019 0:27	2.4	5.6833	3.5235	2.0667	3.3088	19.8818	0.057	1.8133
4/15/2019 0:28	2.35	4.8083	3.5117	2.1667	3.296	19.8	0.0677	1.8057
4/15/2019 0:29	2.3	5.4083	3.9522	1.5	3.2935	19.87	0.066	1.8148
4/15/2019 0:30	2.3	5.2667	3.7313	1.8667	3.3302	19.9727	0.0545	1.8195
4/15/2019 0:31	2.3083	5.2667	3.4115	2.0167	3.3258	19.9636	0.0604	1.8263
4/15/2019 0:32	2.35	4.95	3.5447	1.7833	3.3519	19.8	0.0564	1.8359
4/15/2019 0:33	2.4	4.825	3.749	2.4333	3.3817	19.7083	0.0592	1.8154
4/15/2019 0:34	2.4	4.3167	3.5483	1.9333	3.3951	19.7	0.063	1.8162
4/15/2019 0:35	2.375	4.7667	3.7728	2.05	3.3491	19.7667	0.0523	1.8214
4/15/2019 0:36	2.2833	4.825	3.5052	1.65	3.3311	19.7	0.0695	1.7908
4/15/2019 0:37	2.1333	4.4083	3.1095	2.1	3.3208	19.7455	0.0617	1.8116
4/15/2019 0:38	2.0833	4.65	3.6687	2.3	3.3579	19.67	0.0583	1.8163
4/15/2019 0:39	2.0333	4.35	3.4013	1.8	3.3424	19.6	0.0631	1.824
4/15/2019 0:40	2.0833	4.6417	3.6363	1.6833	3.3717	19.7	0.0481	1.8099
4/15/2019 0:41	2.1333	4.65	3.4842	2.2333	3.3888	19.7	0.0658	1.8015
4/15/2019 0:42	2.2	5.2917	3.501	1.3167	3.3858	19.7	0.056	1.798
4/15/2019 0:43	2.1417	4.35	3.4145	1.45	3.3491	19.7	0.0636	1.8002
4/15/2019 0:44	2.125	5.0167	3.409	2.1167	3.3613	19.7727	0.0632	1.8098

4/15/2019 0:45	2.0917	4.475	3.2573	1.5833	3.3714	19.8091	0.0502	1.8226
4/15/2019 0:46	2.0417	4.5333	3.2767	1.3333	3.39	19.85	0.0673	1.808
4/15/2019 0:47	2.0833	5.15	3.5482	1.7	3.4208	19.9	0.0587	1.7947
4/15/2019 0:48	2.1	3.4917	3.1318	1.5667	3.4	20	0.0579	1.8058
4/15/2019 0:49	2.1	3.7917	3.0857	1.8667	3.4153	20.1	0.0616	1.8078
4/15/2019 0:50	2.1	4.45	3.0978	1.6667	3.4065	20.1727	0.049	1.8167
4/15/2019 0:51	2.0917	4.45	3.303	1.05	3.4348	20.2	0.0626	1.815
4/15/2019 0:52	2.0083	4.1833	3.2258	1.2333	3.4319	20.2333	0.0568	1.8234
4/15/2019 0:53	2	4.425	3.113	1.3333	3.4146	20.2546	0.0573	1.8363
4/15/2019 0:54	2	4.9417	3.4564	1.2333	3.4381	20.2182	0.064	1.8257
4/15/2019 0:55	2	4.2417	3.2129	1.45	3.4237	20.29	0.0526	1.8059
4/15/2019 0:56	2	3.875	3.3409	1.5167	3.476	20.2	0.0638	1.7754
4/15/2019 0:57	2	4.8333	3.4243	1.4833	3.4265	20.2333	0.0531	1.7795
4/15/2019 0:58	2	3.7167	3.1148	1.75	3.4495	20.3	0.0616	1.8232
4/15/2019 0:59	1.9667	4.025	3.3608	0.7	3.4656	20.2417	0.0639	1.8178
4/15/2019 1:00	1.9	4.0917	3.0713	1.5167	3.4253	20.2	0.0502	1.8329
4/15/2019 1:01	1.9083	4.4667	3.1968	1.8833	3.4765	20.2	0.058	1.82
4/15/2019 1:02	1.9667	4.0667	3.2523	1.4333	3.4937	20.2	0.0562	1.7973
4/15/2019 1:03	2	4.0417	3.1863	1.7	3.4873	20.2167	0.0524	1.8108
4/15/2019 1:04	2	4.55	3.443	2.0667	3.4366	20.3	0.0653	1.8249
4/15/2019 1:05	2	4.9167	3.5262	1.4167	3.465	20.3455	0.0539	1.8088
4/15/2019 1:06	2	4.1833	3.1218	1.4	3.4623	20.4	0.0629	1.8317
4/15/2019 1:07	2	4.3667	3.2769	1.9167	3.5003	20.4	0.0567	1.806
4/15/2019 1:08	2	4.5583	3.2698	1.0833	3.4448	20.4	0.0528	1.8079
4/15/2019 1:09	1.9417	4.6	3.0595	1.8667	3.4931	20.4	0.0626	1.8232
4/15/2019 1:10	1.9	3.95	3.385	2.0667	3.5033	20.5	0.0524	1.8093
4/15/2019 1:11	1.9	4.8583	3.2395	1.7167	3.5061	20.4667	0.0671	1.8226
4/15/2019 1:12	1.9	4.4667	3.0508	1.45	3.5048	19.7667	0.0625	1.8069
4/15/2019 1:13	1.9	3.9	3.1068	1.05	3.4797	18.81	0.0496	1.8058
4/15/2019 1:14	1.9	4.3333	3.191	1.0167	3.5169	17.7636	0.0673	1.7892
4/15/2019 1:15	1.85	4.3833	3.1741	1.4667	3.5323	17.6	0.0522	1.8165
4/15/2019 1:16	1.8917	4.4083	3.4669	1.7167	3.5284	17.5583	0.0626	1.8163
4/15/2019 1:17	1.825	4.3667	3.2136	1.35	3.5007	17.5273	0.0589	1.8052
4/15/2019 1:18	1.8	3.5583	2.9842	1.9167	3.4986	17.5	0.0481	1.7965
4/15/2019 1:19	1.8	4.3833	3.2616	1.4167	3.5252	16.8909	0.0598	1.7902
4/15/2019 1:20	1.8	5.1667	3.1624	1.6	3.5188	16.175	0.0532	1.7565
4/15/2019 1:21	1.8	4.4333	3.4567	2	3.5424	16.1	0.0539	1.8091
4/15/2019 1:22	1.8	4.5333	3.4062	1.4333	3.536	16.0167	0.0577	1.8391
4/15/2019 1:23	1.8833	4.35	3.2406	1.55	3.5355	16	0.0519	1.8194
4/15/2019 1:24	1.85	4.7583	3.1531	1.3167	3.4948	16.5	0.065	1.802
4/15/2019 1:25	1.8	4.3583	3.2963	1.75	3.506	17.2455	0.0521	1.7943
4/15/2019 1:26	1.8	4.2333	3.299	0.9667	3.5288	18.35	0.0525	1.7979
4/15/2019 1:27	1.8167	3.775	3.5092	1.2667	3.5071	18.625	0.0656	1.8044
4/15/2019 1:28	1.8083	3.95	2.9667	1.75	3.5293	18.5364	0.0525	1.7927
4/15/2019 1:29	1.8	4.575	3.1106	1.4333	3.4873	18.5182	0.0623	1.7834
4/15/2019 1:30	1.8	4.25	2.887	1.2167	3.4902	18.3091	0.0578	1.8176
4/15/2019 1:31	1.8	3.6667	3.1344	1.3333	3.4748	18.3667	0.055	1.8044

4/15/2019 1:32	1.8	5.0417	3.2507	1.6	3.5074	18.0727	0.0621	1.7969
4/15/2019 1:33	1.8	4.25	3.2233	1.7167	3.5113	17.53	0.0472	1.8168
4/15/2019 1:34	1.8083	4.2417	3.1158	1.1	3.5083	17.5	0.063	1.816
4/15/2019 1:35	1.875	3.875	3.0391	1.3667	3.5372	17.475	0.0582	1.8155
4/15/2019 1:36	1.9	3.875	2.9221	1.0333	3.5862	17.4455	0.0551	1.8214
4/15/2019 1:37	1.9583	4.8667	2.8566	1.0667	3.5572	17.5818	0.0661	1.8367
4/15/2019 1:38	1.9	4.75	2.9471	1.5833	3.5678	17.5273	0.052	1.8373
4/15/2019 1:39	1.9	4.1583	3.0837	1.55	3.5489	17.53	0.0651	1.8285
4/15/2019 1:40	1.9	3.525	3.1653	0.95	3.5392	17.3333	0.0555	1.8251
4/15/2019 1:41	1.825	4.0917	2.802	1.2	3.5523	17.1	0.0618	1.8388
4/15/2019 1:42	1.8	4.2417	3.0267	0.9	3.6279	17.1909	0.0582	1.8143
4/15/2019 1:43	1.8	4.3917	2.8441	1.2833	3.6251	17.3273	0.0522	1.8294
4/15/2019 1:44	1.8417	4.425	3.0998	1.4833	3.6707	17.7	0.0615	1.8115
4/15/2019 1:45	1.825	4.3583	2.8853	0.9167	3.6655	17.9444	0.0612	1.8044
4/15/2019 1:46	1.8417	4.25	2.9361	1.7	3.6554	17.725	0.0524	1.827
4/15/2019 1:47	1.8	4.375	3.2526	1.5667	3.7278	17.35	0.0656	1.8135
4/15/2019 1:48	1.8	4.4167	3.0965	1.35	3.7074	17.2455	0.0529	1.8235
4/15/2019 1:49	1.8	3.6917	2.8785	1.3667	3.7068	17	0.0611	1.8152
4/15/2019 1:50	1.8	4.125	3.1533	0.85	3.7029	17.075	0.0556	1.8185
4/15/2019 1:51	1.8	4.4167	3.056	0.9333	3.708	17.08	0.0583	1.8273
4/15/2019 1:52	1.7167	4.5167	3.1783	1.45	3.7223	17.0182	0.0617	1.8343
4/15/2019 1:53	1.7	3.9083	3.0804	1.3	3.6832	16.3	0.0547	1.8379
4/15/2019 1:54	1.6917	4.1583	2.9912	1.4667	3.6772	15.1125	0.0625	1.8326
4/15/2019 1:55	1.6833	4.65	3.1128	1.4	3.7273	13.85	0.0601	1.7983
4/15/2019 1:56	1.7	4.3167	3.2401	0.9167	3.7551	12.9	0.0577	1.7943
4/15/2019 1:57	1.6833	4.125	2.9727	1.4333	3.6918	12.18	0.0666	1.8199
4/15/2019 1:58	1.6167	4.425	2.8612	1.4167	3.7238	11.0818	0.0518	1.8321
4/15/2019 1:59	1.6	4.0333	2.9156	1.45	3.6993	10.29	0.0653	1.8401
4/15/2019 2:00	1.675	4.7333	2.9679	1.5833	3.6878	9.1364	0.0577	1.7984
4/15/2019 2:01	1.7	4.0667	3.2576	0.6667	3.6733	8.7556	0.0603	1.7922
4/15/2019 2:02	1.7	3.675	2.9623	0.9	3.6443	7.8818	0.062	1.8148
4/15/2019 2:03	1.7	4.325	3.0167	0.9333	3.6423	7.02	0.0437	1.7826
4/15/2019 2:04	1.7	4.1	2.7146	1.0167	3.6377	6.5818	0.0576	1.7916
4/15/2019 2:05	1.7727	4.025	2.748	1.3167	3.6343	6.3167	0.0505	1.8038
4/15/2019 2:06	1.7667	4.225	2.7498	1.1667	3.6118	6.1083	0.044	1.7629
4/15/2019 2:07	1.7583	4.6	2.8488	1.75	3.6178	5.8083	0.0523	1.7578
4/15/2019 2:08	1.8	4.0833	2.7777	1.2333	3.5848	5.7	0.0416	1.7567
4/15/2019 2:09	1.8	4.4417	2.9934	0.8	3.5873	5.55	0.0495	1.7427
4/15/2019 2:10	1.8	4.775	2.9339	1.0667	3.5771	6.075	0.0468	1.7427
4/15/2019 2:11	1.8083	4.0167	3.0387	1.9833	3.5571	6.8273	0.0373	1.7534
4/15/2019 2:12	1.8833	4.4667	3.1523	1.3333	3.5638	7.1667	0.0524	1.7343
4/15/2019 2:13	1.9	3.9083	3.0411	1.55	3.5798	6.94	0.0324	1.7119
4/15/2019 2:14	1.9917	4.65	3.3962	1.0167	3.6076	7.0583	0.0396	1.7339
4/15/2019 2:15	2.0167	4.55	3.2805	1.0167	3.6022	7.4636	0.0486	1.7234
4/15/2019 2:16	2.0917	4.5833	3.2213	1.2167	3.6113	7.47	0.0361	1.7444
4/15/2019 2:17	2.0333	4.3667	3.1075	1.8	3.6449	7.95	0.0494	1.7252
4/15/2019 2:18	2.05	4.125	3.1434	1.6667	3.6332	8.8818	0.0418	1.718

4/15/2019 2:19	1.9667	3.8833	2.9053	2.0333	3.5979	10.0909	0.0348	1.7422
4/15/2019 2:20	1.8917	4.175	3.279	1.3333	3.5661	11.2	0.0478	1.741
4/15/2019 2:21	1.85	3.7667	3.3576	1.3333	3.6129	12.3364	0.0412	1.7466
4/15/2019 2:22	1.9833	4.3583	2.9962	1.2667	3.6223	12.6091	0.0469	1.7363
4/15/2019 2:23	2	4.9833	3.1518	1.4333	3.623	12.9091	0.0459	1.7411
4/15/2019 2:24	2	4.425	3.1718	1.6333	3.623	13.47	0.0381	1.7948
4/15/2019 2:25	2	4.275	3.205	1.4	3.6081	14.46	0.0534	1.7954
4/15/2019 2:26	2	3.65	3.4793	1.0333	3.6045	14.125	0.0446	1.8115
4/15/2019 2:27	2	4.0167	3.2542	0.9167	3.5842	14.2	0.0567	1.8028
4/15/2019 2:28	2	4.5667	3.0375	1.8	3.5998	14.3556	0.0426	1.7903
4/15/2019 2:29	1.9583	4.25	3.0392	1.1333	3.588	14.65	0.0436	1.8027
4/15/2019 2:30	2.0083	5.1583	3.4632	0.9833	3.5873	14.2455	0.0517	1.8028
4/15/2019 2:31	2	4.0917	2.542	1.5833	3.5831	13.8	0.0443	1.814
4/15/2019 2:32	2.075	4.4917	3.2913	1.35	3.5878	13.7364	0.0542	1.7979
4/15/2019 2:33	2.1	4.4167	3.0928	1.25	3.6047	13.1909	0.0526	1.7773
4/15/2019 2:34	2.1	4.3167	3.0547	1.4333	3.5913	13.6917	0.0449	1.7757
4/15/2019 2:35	2.1333	3.9333	2.9188	1.8667	3.6072	13.8	0.0593	1.7614
4/15/2019 2:36	2.1	4.0083	3.2018	1.7833	3.5918	13.4	0.0513	1.7972
4/15/2019 2:37	2.1	4.9083	3.306	1.4667	3.5831	12.9667	0.0693	1.8082
4/15/2019 2:38	2.1167	4.4333	3.3672	1.3333	3.5907	12.6455	0.0596	1.7861
4/15/2019 2:39	2.1583	4.7667	3.453	1.9	3.5625	12.125	0.0509	1.7938
4/15/2019 2:40	2.2583	4.4583	3.2427	1.5833	3.5893	11.96	0.0615	1.7997
4/15/2019 2:41	2.225	3.625	3.2532	1.8333	3.5543	11.5	0.0492	1.7866
4/15/2019 2:42	2.3	4.6583	3.3203	1.7833	3.5749	11.78	0.0618	1.8067
4/15/2019 2:43	2.2417	4.15	3.0685	1.6	3.576	11.9833	0.0565	1.8191
4/15/2019 2:44	2.2	4.1083	2.9682	1.8	3.5589	11.79	0.0471	1.829
4/15/2019 2:45	2.1583	4.5667	3.3023	1.55	3.5758	12.4636	0.0672	1.8033
4/15/2019 2:46	2.15	4.7833	3.4245	1.6333	3.5608	12.5455	0.0551	1.793
4/15/2019 2:47	2.2	4.4417	3.136	1.3333	3.5573	11.69	0.0633	1.8199
4/15/2019 2:48	2.1833	4.4583	3.158	1.7333	3.5232	10.725	0.0623	1.8333
4/15/2019 2:49	2.1583	4.8167	2.9063	1.8833	3.5479	9.78	0.0478	1.8177
4/15/2019 2:50	2.125	3.8333	3.108	1.4167	3.5335	9.1	0.0673	1.8449
4/15/2019 2:51	2.1917	4.4333	3.346	2.1333	3.5186	9.01	0.0487	1.8217
4/15/2019 2:52	2.1	4.2917	3.2235	1.3	3.4982	8.1273	0.0604	1.8098
4/15/2019 2:53	2.1	4.025	2.8755	1.25	3.4855	7.2875	0.065	1.801
4/15/2019 2:54	2.1	4.7333	3.7923	1.2667	3.4888	6.1889	0.0544	1.8045
4/15/2019 2:55	2.1	4.5	3.2168	1.05	3.4637	5.2455	0.0565	1.8033
4/15/2019 2:56	2.1167	4.4833	3.4298	1.65	3.4698	4.54	0.0492	1.8346
4/15/2019 2:57	2.1083	4.6833	2.9355	1.9167	3.4532	3.2	0.0598	1.8189
4/15/2019 2:58	2.15	4.225	3.0493	2.2833	3.4636	1.9	0.0603	1.7895
4/15/2019 2:59	2.1583	4.4917	3.1107	1.9833	3.4626	1.475	0.0469	1.8211
4/15/2019 3:00	2.1	4.4667	3.3727	1.6833	3.4546	1.5	0.0561	1.8353
4/15/2019 3:01	2.1083	4.5167	3.1405	1.6833	3.4316	1.5	0.0501	1.7978
4/15/2019 3:02	2.1333	3.9167	3.6897	1.2833	3.4251	1.5	0.0572	1.81
4/15/2019 3:03	2.2	4.675	3.7397	2.0333	3.4186	1.5	0.0515	1.8156
4/15/2019 3:04	2.2667	4.625	3.7815	2.6333	3.3956	1.5	0.0439	1.808
4/15/2019 3:05	2.3083	4.6583	3.4962	2.25	3.3861	1.5	0.0612	1.8087

4/15/2019 3:06	2.3083	4.25	3.5843	2.1167	3.3691	1.4	0.0463	1.7969
4/15/2019 3:07	2.3083	4.625	3.4403	2.65	3.3566	1.4182	0.0558	1.7952
4/15/2019 3:08	2.4667	4.9833	3.865	2.6167	3.3473	1.4909	0.0544	1.7788
4/15/2019 3:09	2.5	4.375	3.5323	2.1	3.3749	1.4	0.0363	1.8168
4/15/2019 3:10	2.4083	4.35	3.2795	1.2667	3.384	1.4	0.0498	1.8177
4/15/2019 3:11	2.2	4.9583	3.2768	1.8333	3.3758	1.4	0.0453	1.8086
4/15/2019 3:12	2.1	4.7917	3.3225	1.9667	3.3801	1.4	0.0479	1.8269
4/15/2019 3:13	2.1083	5.0667	3.5765	1.75	3.3659	1.4	0.0538	1.8075
4/15/2019 3:14	2.2	5.225	3.3648	1.8833	3.3661	1.3167	0.0405	1.8018
4/15/2019 3:15	2.1909	4.6	3.122	2.0833	3.3524	1.3	0.0532	1.7927
4/15/2019 3:16	2.2083	5.0083	3.5772	1.9333	3.3608	1.3	0.0488	1.8053
4/15/2019 3:17	2.2167	5.2583	3.6588	2.0167	3.3554	1.3	0.0419	1.7875
4/15/2019 3:18	2.2417	4.6667	3.462	1.8167	3.3513	1.3	0.0524	1.785
4/15/2019 3:19	2.275	4.2417	3.4455	1.85	3.3475	1.3	0.0356	1.7776
4/15/2019 3:20	2.3	4.7167	3.8098	1.6	3.3435	1.2667	0.0477	1.7637
4/15/2019 3:21	2.4083	4.6917	3.7735	2.1833	3.3318	1.2364	0.0448	1.7468
4/15/2019 3:22	2.4917	4.2333	3.8842	1.5333	3.3348	1.2	0.0358	1.7649
4/15/2019 3:23	2.4917	4.25	3.5347	1.95	3.3117	1.3	0.0524	1.7979
4/15/2019 3:24	2.5	5.2167	3.7497	2.5	3.313	1.3	0.0434	1.7858
4/15/2019 3:25	2.575	5.2333	3.6315	2.3333	3.313	1.3	0.0443	1.7646
4/15/2019 3:26	2.7	5.0167	3.8388	1.8667	3.3156	1.3	0.048	1.7995
4/15/2019 3:27	2.8	5.2417	3.9835	1.9167	3.2964	1.3	0.0326	1.8106
4/15/2019 3:28	2.8	5.5667	3.8425	2.25	3.2956	1.3	0.0517	1.795
4/15/2019 3:29	2.825	5.7583	4.0367	2.1	3.2959	1.3	0.04	1.7948
4/15/2019 3:30	3.0167	5.675	4.3647	2.6	3.2693	1.29	0.046	1.7625
4/15/2019 3:31	3.1667	6.0833	4.3595	2.5833	3.256	1.2	0.0466	1.7634
4/15/2019 3:32	3.1083	5.4583	3.7782	2.2167	3.2589	1.2	0.037	1.8083
4/15/2019 3:33	3	5.8	3.876	2.6833	3.2417	1.2	0.0479	1.8135
4/15/2019 3:34	2.9167	4.9583	3.5005	2.3333	3.2263	1.2	0.0432	1.8228
4/15/2019 3:35	2.8667	4.95	3.6627	2.1333	3.2498	1.2	0.0431	1.8158
4/15/2019 3:36	2.75	4.575	3.9282	2.2167	3.243	1.2	0.0502	1.8186
4/15/2019 3:37	2.725	5.025	4.196	2.2167	3.2331	1.2	0.035	1.808
4/15/2019 3:38	2.7	5.7583	4.1483	2.3667	3.217	1.2	0.0548	1.7806
4/15/2019 3:39	2.6833	5.7583	3.6397	2.8333	3.1981	1.2	0.041	1.8124
4/15/2019 3:40	2.6667	5.1583	3.9007	2.5167	3.1964	1.1818	0.0511	1.8178
4/15/2019 3:41	2.7	4.6583	3.7795	2.0333	3.2141	1.1917	0.0502	1.8015
4/15/2019 3:42	2.6833	4.5	3.6655	1.7	3.2056	1.1	0.0396	1.7895
4/15/2019 3:43	2.55	5.425	3.6515	2.4333	3.1938	1.1833	0.0537	1.7975
4/15/2019 3:44	2.5	4.475	3.8642	2.5	3.1745	1.2	0.0402	1.805
4/15/2019 3:45	2.4909	4.6167	3.695	1.4167	3.1887	1.2	0.0479	1.7917
4/15/2019 3:46	2.425	4.8667	4.0012	2.15	3.1635	1.2	0.0504	1.7833
4/15/2019 3:47	2.5	4.9833	3.9355	2.6833	3.1363	1.2	0.0367	1.7944
4/15/2019 3:48	2.5	5.125	4.248	2.1667	3.1391	1.2	0.0505	1.7902
4/15/2019 3:49	2.5833	5.6667	4.0582	2.1	3.1299	1.2	0.0423	1.8088
4/15/2019 3:50	2.5	5.5167	3.9643	2.15	3.1139	1.2	0.0467	1.807
4/15/2019 3:51	2.5917	5.3333	4.1305	2.85	3.1006	1.1727	0.0543	1.8045
4/15/2019 3:52	2.7	4.9333	4.0005	2.55	3.0978	1.2	0.0416	1.7933

4/15/2019 3:53	2.7917	5.5583	3.7647	2.75	3.0896	1.2	0.052	1.8164
4/15/2019 3:54	2.8917	5.1667	4.3065	2.9	3.0973	1.2	0.0441	1.8219
4/15/2019 3:55	2.8583	5.0167	4.3217	2.75	3.0761	1.2	0.0515	1.8053
4/15/2019 3:56	2.9167	5.5083	4.5115	2.7667	3.0522	1.1	0.0529	1.7699
4/15/2019 3:57	3.0167	6.05	4.4766	3.1167	3.0633	1.1	0.0401	1.7701
4/15/2019 3:58	3.1667	4.7417	4.4489	2.9333	3.0518	1.1	0.0539	1.7789
4/15/2019 3:59	3.25	6.0833	4.2643	3.4667	3.0259	1.1	0.0501	1.7836
4/15/2019 4:00	3.275	5.45	4.279	3.3	3.0283	1.1	0.0516	1.7705
4/15/2019 4:01	3.2	5.575	4.1099	2.5	3.0307	1.1	0.0531	1.7849
4/15/2019 4:02	3.1	5.45	4.2888	3.1667	3.0287	1.1	0.0396	1.7985
4/15/2019 4:03	3.1	5.5667	4.1718	3.3	3.0199	1.1	0.0555	1.7965
4/15/2019 4:04	3.1417	5.8833	4.3283	3.3833	3.017	1.1	0.0421	1.7866
4/15/2019 4:05	3.2	5.3667	3.9555	2.45	3.0173	1.0917	0.0546	1.8069
4/15/2019 4:06	3.3417	6.1167	4.4663	2.5833	3.0057	1	0.0507	1.7963
4/15/2019 4:07	3.3417	5.325	4.1207	2.3667	2.986	1.04	0.045	1.7886
4/15/2019 4:08	3.2417	5.4833	4.1258	2.2167	3.0183	1.1	0.0551	1.8159
4/15/2019 4:09	3.15	5.1583	3.9603	2.1833	3.008	1.1	0.0428	1.8375
4/15/2019 4:10	3.0167	4.9167	4.1443	2.5667	2.9912	1.1	0.053	1.8267
4/15/2019 4:11	3.1	4.9333	4.3751	2.1167	2.9972	1.1	0.057	1.8321
4/15/2019 4:12	3.0583	5.0917	4.2775	2.5	2.9898	1.1	0.041	1.8245
4/15/2019 4:13	2.9833	4.6833	3.8012	2.2333	2.9799	1.1364	0.0585	1.8131
4/15/2019 4:14	2.9	5.225	4.0893	2.4333	2.9823	1.2	0.0459	1.8279
4/15/2019 4:15	2.925	5	4.0371	2.35	2.9657	1.1909	0.0473	1.8244
4/15/2019 4:16	3	5.3167	4.058	2.7167	2.9635	1.1	0.0549	1.8356
4/15/2019 4:17	3.0083	4.725	4.1267	2.4667	2.9397	1.1	0.0428	1.8579
4/15/2019 4:18	3	5.5833	4.2586	2.6167	2.9361	1.15	0.053	1.8225
4/15/2019 4:19	3	5.1583	4.312	2.2833	2.9289	1.2	0.0477	1.7893
4/15/2019 4:20	3	5.6417	4.5736	2.2333	2.9207	1.2	0.0498	1.8124
4/15/2019 4:21	3	5.575	4.3293	2.8667	2.9225	1.2	0.0538	1.8388
4/15/2019 4:22	3	5.7583	4.3103	2.5667	2.8948	1.2	0.0415	1.8042
4/15/2019 4:23	3.0167	5.3167	4.357	3.0167	2.8952	1.2	0.0557	1.7981
4/15/2019 4:24	3.1	5.4667	4.3154	3.0667	2.8668	1.225	0.0471	1.809
4/15/2019 4:25	3.0917	5.8417	4.1351	2.6667	2.8493	1.2	0.046	1.8036
4/15/2019 4:26	3.075	5.1917	4.6484	3.1	2.849	1.2	0.0528	1.8023
4/15/2019 4:27	3.0333	5.175	4.381	2.5167	2.8462	1.2	0.0393	1.7935
4/15/2019 4:28	3.075	5.5167	4.4388	2.65	2.8513	1.2545	0.0541	1.7895
4/15/2019 4:29	3	4.6917	4.1788	2.3833	2.85	1.3	0.0507	1.8085
4/15/2019 4:30	2.95	5.675	4.1194	2.4333	2.8737	1.3	0.0472	1.8226
4/15/2019 4:31	2.8333	5.925	4.0773	2.15	2.8616	1.2727	0.0554	1.8127
4/15/2019 4:32	2.7667	5.7667	3.8098	2.6	2.8809	1.275	0.0368	1.817
4/15/2019 4:33	2.7	5.175	4.0768	2.2333	2.8696	1.2455	0.0538	1.8369
4/15/2019 4:34	2.7	5.4917	3.7624	2.1333	2.8743	1.2	0.0488	1.8115
4/15/2019 4:35	2.6583	4.8333	3.8058	2.4333	2.8738	1.2	0.0394	1.8421
4/15/2019 4:36	2.6167	4.8917	3.507	2.35	2.8651	1.2	0.0503	1.8533
4/15/2019 4:37	2.525	4.8833	3.7575	2.7167	2.8608	1.2	0.0426	1.8187
4/15/2019 4:38	2.5333	5.0417	3.8291	1.4667	2.8518	1.2583	0.0488	1.8355
4/15/2019 4:39	2.5	5.2	3.8741	2.3667	2.8706	1.3	0.056	1.8561

4/15/2019 4:40	2.5667	4.525	3.8775	1.9	2.863	1.3	0.0407	1.8309
4/15/2019 4:41	2.5833	4.9417	3.8048	2.3167	2.8558	1.3	0.0542	1.844
4/15/2019 4:42	2.6	5.5083	3.7041	2.55	2.8541	1.3	0.0409	1.8316
4/15/2019 4:43	2.6	4.925	3.7919	1.4167	2.8516	1.3	0.0535	1.8165
4/15/2019 4:44	2.6	4.7083	3.9998	1.85	2.8404	1.3	0.0512	1.8176
4/15/2019 4:45	2.6	5.2333	3.7462	2.0667	2.8425	1.3	0.038	1.8252
4/15/2019 4:46	2.6	5.15	3.8569	2.3667	2.839	1.3	0.0564	1.8114
4/15/2019 4:47	2.6667	5.5833	3.9473	2.05	2.832	1.3	0.0357	1.8302
4/15/2019 4:48	2.7	4.9583	3.6899	2.0667	2.8268	1.325	0.0414	1.8281
4/15/2019 4:49	2.7	4.85	3.9077	2.35	2.8197	1.39	0.0496	1.8362
4/15/2019 4:50	2.8	4.9583	4.2199	2.6667	2.8094	1.3083	0.0424	1.8251
4/15/2019 4:51	2.875	5.5833	3.9894	2.2333	2.7984	1.3	0.0538	1.8173
4/15/2019 4:52	2.9	5.725	3.9652	2.8	2.7999	1.2636	0.0413	1.8284
4/15/2019 4:53	2.925	5.5667	4.4472	2.2833	2.7853	1.2	0.0425	1.8011
4/15/2019 4:54	3.05	5.4917	4.4249	2.2833	2.7806	1.2	0.0454	1.8144
4/15/2019 4:55	3.1091	6.3	4.3455	2.5833	2.7787	1.2	0.0416	1.7884
4/15/2019 4:56	3.1417	5.1917	4.4826	2.6	2.7527	1.2364	0.054	1.7847
4/15/2019 4:57	3.2	5.5833	4.4398	2.4	2.7584	1.2636	0.0369	1.7808
4/15/2019 4:58	3.2167	5.2917	4.4801	2.8	2.7568	1.2	0.0455	1.7923
4/15/2019 4:59	3.2667	5.7167	4.3022	2.75	2.7724	1.2	0.0448	1.797
4/15/2019 5:00	3.2	5.5	4.438	2.9	2.7654	1.2	0.0387	1.813
4/15/2019 5:01	3.2	5.7417	4.4566	3.05	2.7762	1.2	0.0477	1.7941
4/15/2019 5:02	3.2	5.525	4.5427	2.7	2.7919	1.2	0.0375	1.7746
4/15/2019 5:03	3.2	5.0417	4.3678	2.9333	2.7778	1.2	0.0457	1.7898
4/15/2019 5:04	3.1583	5.725	4.2146	2.3167	2.7819	1.2	0.0493	1.8427
4/15/2019 5:05	2.9333	5.275	3.9679	2.2667	2.7841	1.2	0.0355	1.8125
4/15/2019 5:06	2.8167	4.775	4.0047	2.4667	2.7918	1.2	0.0441	1.7895
4/15/2019 5:07	2.7	5.15	3.7632	2	2.7802	1.2	0.0428	1.7913
4/15/2019 5:08	2.7	5.35	4.1959	1.8833	2.7836	1.2	0.0481	1.8137
4/15/2019 5:09	2.6417	5.2	3.981	1.6667	2.7946	1.2	0.0475	1.8055
4/15/2019 5:10	2.6	4.9083	3.9147	2.3	2.8147	1.2	0.032	1.8033
4/15/2019 5:11	2.6333	4.7	3.8706	2.5167	2.8281	1.2	0.0509	1.8149
4/15/2019 5:12	2.6417	5.275	4.0674	2.65	2.8097	1.2	0.0411	1.7923
4/15/2019 5:13	2.65	5.8417	3.9207	2	2.8245	1.2	0.0409	1.7907
4/15/2019 5:14	2.6	5.25	3.71	1.8333	2.8107	1.2	0.0484	1.8087
4/15/2019 5:15	2.55	4.6833	3.55	1.9667	2.8119	1.2	0.036	1.8136
4/15/2019 5:16	2.5	4.6333	3.5461	1.95	2.8146	1.2	0.0511	1.7921
4/15/2019 5:17	2.5	5.1667	3.8873	1.8667	2.8289	1.2	0.0447	1.8035
4/15/2019 5:18	2.5667	5.2417	3.9762	2.2	2.8801	1.2	0.0333	1.7973
4/15/2019 5:19	2.6	4.7833	3.7828	2.3	2.8641	1.2	0.0499	1.8075
4/15/2019 5:20	2.6	5.35	3.8508	2.2833	2.921	1.2	0.0399	1.803
4/15/2019 5:21	2.6	5.1917	3.7433	2.2833	2.9489	1.2	0.0477	1.7948
4/15/2019 5:22	2.6833	4.725	4.0263	1.9333	3.0344	1.2	0.0468	1.7868
4/15/2019 5:23	2.7	5.0417	3.682	2.3	2.9642	1.2	0.0344	1.791
4/15/2019 5:24	2.6917	5.475	3.7372	2.3167	2.9864	1.2	0.0507	1.8043
4/15/2019 5:25	2.6	5.3167	3.6848	2.35	3.0191	1.2	0.0393	1.8332
4/15/2019 5:26	2.6	5.2833	3.963	2.1667	3.0456	1.2	0.0494	1.8198

4/15/2019 5:27	2.625	5.5417	4.113	2.2167	3.047	1.125	0.0434	1.8038
4/15/2019 5:28	2.7	4.875	4.1805	2.1833	3.0832	1.2	0.0382	1.7977
4/15/2019 5:29	2.7083	4.9917	4.3093	2.55	3.3318	1.2	0.0512	1.7894
4/15/2019 5:30	2.7083	4.5	3.9708	2.2667	3.2487	1.2	0.0407	1.7893
4/15/2019 5:31	2.7	5.4333	4.0665	2.5167	3.2993	1.2	0.0464	1.7998
4/15/2019 5:32	2.7	5.575	3.8753	2.0667	3.4026	1.2	0.0528	1.7924
4/15/2019 5:33	2.65	5.2417	4.0787	2.5833	3.4282	1.2	0.0467	1.818
4/15/2019 5:34	2.6	4.5417	3.7775	2.8167	3.396	1.1167	0.0536	1.8385
4/15/2019 5:35	2.4833	4.9	3.8155	1.8833	3.4021	1.2	0.0417	1.8267
4/15/2019 5:36	2.4	4.9167	3.7953	1.85	3.4241	1.2	0.0493	1.8364
4/15/2019 5:37	2.325	4.0833	3.6587	2.6333	3.4877	1.2	0.0495	1.8233
4/15/2019 5:38	2.2583	4.2333	3.6328	2.2833	3.4102	1.2	0.0418	1.8081
4/15/2019 5:39	2.1833	5.075	3.7178	2.15	3.3277	1.2	0.0564	1.8363
4/15/2019 5:40	2.1167	4.65	3.4972	1.9333	3.4653	1.2	0.0381	1.8223
4/15/2019 5:41	2.1	4.3583	3.3018	2.2167	3.5138	1.2	0.0488	1.8248
4/15/2019 5:42	2.1583	4.6583	3.2957	1.0833	3.3967	1.2	0.049	1.8277
4/15/2019 5:43	2.1083	4.625	3.2775	1.3833	3.4088	1.2	0.0371	1.8242
4/15/2019 5:44	2.1	4.7333	3.4532	1.6333	3.4651	1.2	0.0548	1.8293
4/15/2019 5:45	2.1	3.7083	3.0148	2.05	3.5727	1.2	0.0397	1.8029
4/15/2019 5:46	2.0333	4.525	3.3408	1.8833	3.6036	1.2	0.0434	1.8392
4/15/2019 5:47	2.075	4.1583	3.3452	1.55	3.6844	1.2	0.0566	1.8407
4/15/2019 5:48	2	4.575	3.0343	1.2333	3.7006	1.2	0.039	1.8451
4/15/2019 5:49	2.1	4.5333	3.1605	1.8167	3.8567	1.2	0.0556	1.845
4/15/2019 5:50	2.075	5.2333	3.2995	1.3667	3.9121	1.2	0.0415	1.8203
4/15/2019 5:51	2.0167	4.1333	3.25	1.3167	3.8889	1.2	0.0511	1.8198
4/15/2019 5:52	2	4.1333	3.2288	1.8833	3.9638	1.2	0.0455	1.8258
4/15/2019 5:53	2	3.825	3.177	1.2	4.1028	1.2	0.0383	1.8024
4/15/2019 5:54	2	4.2083	3.2687	1.3833	4.1831	1.2	0.0524	1.7894
4/15/2019 5:55	1.9818	4.075	3.3028	1.9167	4.4105	1.2	0.0516	1.772
4/15/2019 5:56	1.9333	4.3333	3.25	1.1667	4.3485	1.2	0.0449	1.7906
4/15/2019 5:57	1.9083	4.45	3.3663	1.45	4.4726	1.2	0.058	1.788
4/15/2019 5:58	1.9	3.675	3.226	1.4333	4.4813	1.2	0.0461	1.807
4/15/2019 5:59	1.8583	4.825	3.1065	1.4167	4.7998	1.2	0.0502	1.7968
4/15/2019 6:00	1.8	4.175	3.1622	1.5833	4.5798	1.2	0.0509	1.8023
4/15/2019 6:01	1.8333	4.1917	3.1673	1.4333	4.782	1.2	0.0414	1.7952
4/15/2019 6:02	1.8	4.1333	3.472	1.5167	4.9929	1.2182	0.0622	1.798
4/15/2019 6:03	1.8	3.9	3.2605	0.9	4.9978	1.3	0.0455	1.82
4/15/2019 6:04	1.75	3.8583	3.1618	1.35	5.0568	1.3	0.0602	1.8094
4/15/2019 6:05	1.7	4.5583	3.1413	1.65	5.1228	1.3	0.0516	1.8107
4/15/2019 6:06	1.7417	4.35	3.266	1.15	5.1131	1.3	0.0483	1.8127
4/15/2019 6:07	1.7083	4.175	3.2398	1.0167	5.1318	1.3	0.0581	1.8028
4/15/2019 6:08	1.6667	4.3417	3.0765	1.4167	4.9407	1.3	0.0451	1.7898
4/15/2019 6:09	1.6	4.125	3.0162	1.4833	5.1324	1.3	0.0562	1.813
4/15/2019 6:10	1.5917	3.8917	3.002	1.2833	5.0162	1.3727	0.0535	1.8367
4/15/2019 6:11	1.5583	4.175	2.9743	1.1167	5.1678	1.4	0.0436	1.8445
4/15/2019 6:12	1.5	3.875	1.7709	0.7833	5.0505	1.4	0.0575	1.8566
4/15/2019 6:13	1.5167	4.675	2.7255	1.0833	5.1826	1.4	0.0531	1.8287

4/15/2019 6:14	1.5333	3.85	2.9858	1.1167	4.9373	1.5	0.0554	1.8193
4/15/2019 6:15	1.5	4.4083	3.1898	1	5.0834	1.5	0.0643	1.8175
4/15/2019 6:16	1.5	3.6583	2.7603	0.7	5.2048	1.5	0.0465	1.8103
4/15/2019 6:17	1.5	4.025	2.8048	0.7	5.1449	1.5	0.0639	1.826
4/15/2019 6:18	1.5083	3.8667	2.8587	1.2333	5.3063	1.5	0.059	1.815
4/15/2019 6:19	1.475	4.275	2.571	1.0833	5.3126	1.5	0.0524	1.8153
4/15/2019 6:20	1.4	3.4417	2.8903	1.0167	5.3331	1.5	0.0636	1.8005
4/15/2019 6:21	1.3667	3.5	2.8622	0.8167	5.3426	1.6	0.0511	1.82
4/15/2019 6:22	1.3833	3.9333	2.94	1.3167	5.5795	1.6	0.0602	1.8306
4/15/2019 6:23	1.4	3.8167	2.5638	1.15	5.5188	1.6	0.0585	1.8335
4/15/2019 6:24	1.4583	3.75	2.6283	0.8167	5.5831	1.6	0.0568	1.8333
4/15/2019 6:25	1.5	4.0583	2.7443	1.0833	5.8826	1.6	0.0727	1.813
4/15/2019 6:26	1.5	4.2583	2.9233	0.9167	5.7904	1.6	0.0535	1.8254
4/15/2019 6:27	1.5	4.225	2.6597	1.0833	5.6236	1.6	0.0661	1.845
4/15/2019 6:28	1.5	4.1	2.6732	1.45	5.626	1.6	0.0642	1.8317
4/15/2019 6:29	1.5	3.9833	2.6295	1.5667	5.6702	1.6	0.0565	1.8193
4/15/2019 6:30	1.6	4.125	2.9307	1.7833	5.8963	1.6	0.0696	1.8271
4/15/2019 6:31	1.6	3.5833	2.8487	1.8167	6.0113	1.6	0.0601	1.8232
4/15/2019 6:32	1.6	4.3	2.3263	1.55	6.0428	1.6	0.0631	1.8213
4/15/2019 6:33	1.6583	3.7667	2.6297	0.85	6.1526	1.6	0.0712	1.8455
4/15/2019 6:34	1.7	3.8833	2.9218	1.6	6.395	1.6	0.066	1.8095
4/15/2019 6:35	1.7417	3.6417	3.0255	1.15	6.5003	1.5455	0.0789	1.7924
4/15/2019 6:36	1.8	3.3	2.7265	1.4333	6.3303	1.6	0.0737	1.8172
4/15/2019 6:37	1.7333	4.2083	2.7907	0.4667	5.9503	1.6	0.0574	1.8617
4/15/2019 6:38	1.6583	3.9583	2.5563	0.7333	6.0488	1.56	0.0732	1.8557
4/15/2019 6:39	1.55	3.75	2.569	0.7333	5.9483	1.5	0.0571	1.8478
4/15/2019 6:40	1.5917	4.0833	2.6987	1.3	5.9462	1.5	0.0694	1.8776
4/15/2019 6:41	1.525	3.4917	2.7373	0.9	5.9566	1.54	0.0656	1.8709
4/15/2019 6:42	1.4917	3.725	2.4912	0.3167	5.8381	1.5	0.0587	1.8429
4/15/2019 6:43	1.5	3.7833	2.7568	1.1333	5.8498	1.5	0.0745	1.8404
4/15/2019 6:44	1.4583	4.4583	2.5538	1.25	5.9174	1.5364	0.0629	1.841
4/15/2019 6:45	1.5	4.0833	2.538	0.7333	5.9862	1.56	0.0711	1.8305
4/15/2019 6:46	1.5	3.0167	2.5875	1	5.9544	1.59	-0.0046	1.8674
4/15/2019 6:47	1.5	4.2667	1.7172	1.1667	5.9223	1.6	-0.1031	1.8626
4/15/2019 6:48	1.5	3.4	2.1728	1.3167	6.0166	1.5778	-0.09	1.8448
4/15/2019 6:49	1.5	3.55	1.7414	0.5333	6.0197	1.5	-0.1022	1.8463
4/15/2019 6:50	1.5	3.0917	1.5898	0.5167	6.0108	1.5	-0.1053	1.8431
4/15/2019 6:51	1.525	3.675	2.422	1.3	5.9949	1.5	-0.0992	1.8473
4/15/2019 6:52	1.5417	2.7917	1.8028	1	6.0237	1.5	-0.1195	1.8399
4/15/2019 6:53	1.6	3.725	2.4492	1.5333	6.0718	1.5	-0.1012	1.8253
4/15/2019 6:54	1.6417	3.825	2.6477	1.0667	6.0522	1.5	-0.1045	1.8373
4/15/2019 6:55	1.7417	4.3333	2.6789	1.1167	6.1331	1.5	-0.1048	1.8335
4/15/2019 6:56	1.8	4.475	2.7705	1.3167	6.0983	1.4455	-0.0984	1.8215
4/15/2019 6:57	1.8333	3.3333	2.3223	0.8667	6.014	1.4	-0.1116	1.8333
4/15/2019 6:58	1.8167	3.9417	2.5293	0.9333	6.0001	1.4	-0.1004	1.8403
4/15/2019 6:59	1.7667	4.2583	2.8094	1.2	5.9914	1.3583	-0.1069	1.8483
4/15/2019 7:00	1.8833	4.3667	2.9107	1.8667	6.0236	1.3	-0.1101	1.8241

4/15/2019 7:01	2.025	4.0083	2.7663	1.0333	5.9634	1.3	-0.0993	1.8209
4/15/2019 7:02	2.1	3.55	3.1042	1	5.9322	1.3	-0.1129	1.8365
4/15/2019 7:03	2.1	4.0417	3.2452	1.3833	5.9349	1.3	-0.1153	1.8063
4/15/2019 7:04	2.1417	4.0167	3.3248	1.6167	5.8998	1.3	-0.1058	1.8292
4/15/2019 7:05	2.2917	4.1667	2.6173	1.5	5.8753	1.3	-0.1179	1.8153
4/15/2019 7:06	2.2636	4.725	2.5819	1.4667	5.8491	1.3	-0.1033	1.818
4/15/2019 7:07	2.2	4.675	2.1462	1.2333	5.8228	1.3	-0.1088	1.8101
4/15/2019 7:08	2.2	5.2833	2.9761	1.4833	5.8003	1.3	-0.1059	1.7688
4/15/2019 7:09	2.2	4.7917	2.84	1.4833	5.7693	1.3	-0.1087	1.782
4/15/2019 7:10	2.2	4.375	2.4635	1.8167	5.7278	1.3	-0.1074	1.8067
4/15/2019 7:11	2.1083	4.9	3.0714	1.4667	5.73	1.3	-0.1143	1.8143
4/15/2019 7:12	2.1667	4.7417	2.2218	2.0833	5.6909	1.3	-0.1035	1.8288
4/15/2019 7:13	2.2583	3.8917	3.4293	1.9833	5.6764	1.3	-0.117	1.8264
4/15/2019 7:14	2.3833	5.2833	3.0688	1.7667	5.6344	1.3	-0.108	1.8157
4/15/2019 7:15	2.3917	4.75	3.1488	1.75	5.6286	1.3	-0.1034	1.8123
4/15/2019 7:16	2.4	4.5417	3.6218	1.9833	5.579	1.3	-0.1175	1.8352
4/15/2019 7:17	2.3	4.6417	3.2647	0.9333	5.5732	1.3	-0.0991	1.8402
4/15/2019 7:18	2.2167	4.4583	2.574	1.85	5.6238	1.3	-0.1062	1.8347
4/15/2019 7:19	2.0167	4.5167	2.6839	1.35	5.6268	1.3	-0.1048	1.8396
4/15/2019 7:20	1.9333	3.5833	2.7366	1.7333	5.5913	1.3	-0.103	1.8173
4/15/2019 7:21	1.9083	4.025	2.9443	1.7833	5.5689	1.3	-0.1158	1.8543
4/15/2019 7:22	2.025	4.5167	2.8863	1.7333	5.5342	1.3	-0.0969	1.8321
4/15/2019 7:23	2.075	4.6583	3.1736	1.6833	5.5124	1.3	-0.111	1.8435
4/15/2019 7:24	2	4.6167	2.7106	1.3667	5.5227	1.3	-0.1107	1.8489
4/15/2019 7:25	1.9333	4.2	2.9219	0.95	5.5065	1.3636	-0.1051	1.8303
4/15/2019 7:26	1.9	4.5333	3.2138	0.95	5.4746	1.4	-0.1106	1.8475
4/15/2019 7:27	1.975	3.9083	2.4229	1.4833	5.4424	1.4	-0.1013	1.8523
4/15/2019 7:28	2.0583	4.325	3.576	0.9	5.364	1.4	-0.1114	1.8433
4/15/2019 7:29	2.1417	4.9083	3.3541	2.0667	5.37	1.36	-0.1059	1.8372
4/15/2019 7:30	2.2	5.2	3.0184	1.4833	5.3668	1.3	-0.1103	1.8419
4/15/2019 7:31	2.2583	4.775	3.2263	1.2333	5.2663	1.3	-0.1142	1.8391
4/15/2019 7:32	2.2667	4.3083	3.9337	1.9167	5.1788	1.3	-0.1006	1.798
4/15/2019 7:33	2.2917	3.7167	3.5524	2.1833	5.138	1.3	-0.1118	1.7838
4/15/2019 7:34	2.425	4.675	3.4986	2.3	5.0533	1.3	-0.1043	1.789
4/15/2019 7:35	2.4833	4.1583	3.7618	1.7333	5.0793	1.3	-0.1047	1.8002
4/15/2019 7:36	2.575	5.2917	3.6771	2.1	5.0389	1.3545	-0.1125	1.8158
4/15/2019 7:37	2.575	4.8333	2.9337	2.2167	4.9402	1.35	-0.0991	1.8203
4/15/2019 7:38	2.6333	5.1667	3.7063	2.2	4.9153	1.3	-0.1166	1.8275
4/15/2019 7:39	2.6667	4.975	3.4578	1.8333	4.916	1.3909	-0.1091	1.8425
4/15/2019 7:40	2.6	4.575	3.716	1.6167	4.9559	1.4	-0.1096	1.8714
4/15/2019 7:41	2.5083	4.6917	3.3115	1.5333	4.8855	1.4	-0.1182	1.8795
4/15/2019 7:42	2.5417	4.6917	3.573	2.3833	4.8234	1.4	-0.1042	1.8743
4/15/2019 7:43	2.6833	4.65	3.7387	2.2167	4.771	1.4	-0.1177	1.8761
4/15/2019 7:44	2.725	5.0167	3.5626	1.8667	4.7816	1.47	-0.1038	1.8837
4/15/2019 7:45	2.7333	4.5417	3.6613	1.3167	4.8003	1.5	-0.1102	1.8913
4/15/2019 7:46	2.6333	4.95	3.5613	1.8667	4.7479	1.5	-0.1062	1.8796
4/15/2019 7:47	2.7	4.9667	3.4952	2.35	4.7231	1.5	-0.103	1.8608

4/15/2019 7:48	2.7833	4.5	3.8328	2.0333	4.688	1.5917	-0.0905	1.8809
4/15/2019 7:49	2.7833	4.9083	3.7922	2.0167	4.7747	1.6	0.0284	1.9193
4/15/2019 7:50	2.65	5.0583	3.6981	1.5167	4.7993	1.58	0.0528	1.9368
4/15/2019 7:51	2.525	4.3583	3.6255	1.85	4.7685	1.5	0.0503	1.9438
4/15/2019 7:52	2.525	5.325	3.5088	1.9167	4.778	1.5	0.0734	1.9304
4/15/2019 7:53	2.6	5.1	3.7058	1.7667	4.6929	1.5	0.0616	1.8985
4/15/2019 7:54	2.6167	4.325	3.6249	2.15	4.6739	1.5	0.0623	1.9264
4/15/2019 7:55	2.725	5.0583	4.0908	2.2	4.6185	1.5	0.0691	1.8962
4/15/2019 7:56	2.7	4.8583	4.0284	2.5333	4.6367	1.5	0.0538	1.913
4/15/2019 7:57	2.7083	4.7583	3.6937	2.3	4.6469	1.4818	0.0623	1.9415
4/15/2019 7:58	2.7	4.3083	3.4672	2.2667	4.6735	1.4	0.0655	1.9107
4/15/2019 7:59	2.6583	4.7083	3.5934	2.2	4.7815	1.4	0.0601	1.9449
4/15/2019 8:00	2.525	4.375	5.4428	1.75	11.9458	2.81	0.177	1.9538
4/15/2019 8:01	2.1333	3.6333	22.6408	1.75	17.302	30.6364	0.5995	2.0342
4/15/2019 8:02	1.625	3.025	62.9158	1.4833	21.6758	51.5556	1.5095	2.1972
4/15/2019 8:03	1.025	3.95	175.8125	4.75	27.9135	58.2364	3.4332	2.6018
4/15/2019 8:04	0.6083	1.7833	229.5917	2.8	23.5273	49.9	4.0893	2.8461
4/15/2019 8:05	0.4	2.0667	161.8917	1.8167	26.8531	49.77	3.1932	2.7573
4/15/2019 8:06	0.3	2.5667	109.2917	1.1833	26.7293	62.0111	2.8257	2.61
4/15/2019 8:07	0.3	2.375	111.3417	1.65	26.6374	54.36	3.1281	2.6386
4/15/2019 8:08	0.25	2.6333	95.395	1.4167	22.9687	49.85	3.2437	2.6183
4/15/2019 8:09	0.275	2.025	84.0492	1.1	21.8684	46.6364	3.0653	2.5776
4/15/2019 8:10	0.275	2.775	80.49	0.5	21.3714	45.22	3.1324	2.5363
4/15/2019 8:11	0.3	2.9	78.935	0.3833	21.0668	44.7273	3.3584	2.525
4/15/2019 8:12	0.3	2.225	78.8183	0.6333	21.5919	48	3.4223	2.546
4/15/2019 8:13	0.2833	2.6583	80.1725	1.1	22.0416	47.3364	2.604	2.5472
4/15/2019 8:14	0.2	2.4167	75.3725	0.6333	21.2585	47.22	2.7562	2.5173
4/15/2019 8:15	0.225	2.7083	75.1692	0.7333	21.546	47.1909	2.6593	2.5399
4/15/2019 8:16	0.2333	2.5167	70.7117	0.25	20.7152	46.93	2.6636	2.5198
4/15/2019 8:17	0.2	2.4417	68.8317	0.4333	20.2013	46.6091	2.7653	2.4776
4/15/2019 8:18	0.1583	2.5417	62.3758	0.8333	19.2328	46.32	3.0227	2.47
4/15/2019 8:19	0.1	3.275	58.6983	0.1833	18.8048	46.0182	2.7588	2.4525
4/15/2019 8:20	0.1	2.8417	55.5333	0.55	18.24	45.4111	2.6919	2.3929
4/15/2019 8:21	0.1	2.0917	54.4842	0.2667	17.9673	44.9364	2.7381	2.3862
4/15/2019 8:22	0.1	2.25	50.7425	0.0333	17.5437	44.5	3.1545	2.4054
4/15/2019 8:23	0.1	2.7333	54.2192	0.25	18.0638	44.1091	2.9439	2.4611
4/15/2019 8:24	0.1	2.0417	53.8975	0.5167	17.9331	43.64	2.7713	2.4416
4/15/2019 8:25	0.1	2.2917	50.7275	-0.0667	17.3878	43.0909	2.5708	2.3782
4/15/2019 8:26	0.1	3.1167	51.5467	-0.0667	17.4069	42.5	2.4926	2.4044
4/15/2019 8:27	0.1	2.4083	55.9817	0.05	18.1617	41.99	2.5786	2.4313
4/15/2019 8:28	0.1417	2.0667	53.7933	0.2667	17.843	41.6333	2.5042	2.4614
4/15/2019 8:29	0.1167	2.6167	50.8233	-0.3167	17.4668	41.325	2.4511	2.4604
4/15/2019 8:30	0.1	2.6833	50.03	0.4833	17.398	40.8818	2.4062	2.4493
4/15/2019 8:31	0.1	2.3333	48.14	0.6	17.3653	40.2583	2.3482	2.4038
4/15/2019 8:32	0.1	2.4917	46.0033	0.0667	16.6425	40.1	2.3313	2.3713
4/15/2019 8:33	0.1833	2.6417	44.6767	0.15	16.1675	39.7667	2.2672	2.3813
4/15/2019 8:34	0.2	2.4333	42.9617	0.25	15.8478	39.3	2.2773	2.3699

4/15/2019 8:35	0.2	2.7083	39.9817	0.3	15.4326	38.81	2.1695	2.3543
4/15/2019 8:36	0.2	2.4417	42.2367	0.1333	15.6033	38.3	2.2241	2.3871
4/15/2019 8:37	0.25	3.1083	42.3	0.1667	15.5761	37.9	2.2268	2.389
4/15/2019 8:38	0.2667	2.3333	37.9833	-0.6833	14.8959	37.4444	2.0721	2.3647
4/15/2019 8:39	0.3	2	37.0233	-0.4333	14.6954	36.8182	2.0573	2.3661
4/15/2019 8:40	0.2917	2.0083	41.8883	-0.15	15.3891	36.27	2.2223	2.434
4/15/2019 8:41	0.25	2.925	42.4267	0.5	15.5423	35.9417	2.2642	2.4493
4/15/2019 8:42	0.2	2.2	41.2583	0.1667	15.3516	35.74	2.2492	2.4749
4/15/2019 8:43	0.2	2.1667	37.6217	-0.7333	14.6917	35.43	2.105	2.4241
4/15/2019 8:44	0.2	2.4667	36.9183	-0.2333	14.4553	35.0727	2.0832	2.3868
4/15/2019 8:45	0.2	2.4083	32.8983	-0.2667	13.8507	34.6667	1.9603	2.3466
4/15/2019 8:46	0.2417	2.8083	31.56	-0.1	13.4403	34.04	1.8933	2.3171
4/15/2019 8:47	0.3	2.975	31.155	-0.1167	13.3992	33.4545	1.897	2.2984
4/15/2019 8:48	0.275	3.1333	31.975	0.3833	13.4392	33.05	1.9273	2.333
4/15/2019 8:49	0.2	2.85	32.4767	-0.0167	13.5033	32.5917	1.9655	2.3613
4/15/2019 8:50	0.2	2.5333	32.0817	0.2167	13.4303	32.2909	1.9403	2.3538
4/15/2019 8:51	0.2417	2.5917	29.6033	0.2667	13.0097	31.9818	1.8443	2.3133
4/15/2019 8:52	0.2583	2.3083	30.6083	-0.0667	12.9355	31.4083	1.8762	2.3621
4/15/2019 8:53	0.2	2.6833	31.7733	-0.2	13.326	31	1.9733	2.4235
4/15/2019 8:54	0.2	2.1333	32.87	0	13.4253	30.62	2.0002	2.4215
4/15/2019 8:55	0.2	2.2417	32.8233	-0.2333	13.3599	30.35	1.9954	2.4336
4/15/2019 8:56	0.2	2.4583	31.0083	0.0167	13.0802	30.1909	1.9478	2.4043
4/15/2019 8:57	0.2417	2.4	28.2017	0.0333	12.5328	29.79	1.8307	2.3417
4/15/2019 8:58	0.3	3.1833	26.9183	0.2667	12.2243	29.5636	1.7463	2.315
4/15/2019 8:59	0.3	2.7583	26.9833	-0.4	12.2042	29.4167	1.7831	2.3368
4/15/2019 9:00	0.3	2	26.595	0.3833	12.0754	29.1909	1.7703	2.3378
4/15/2019 9:01	0.2083	2.275	26.735	-0.35	12.0789	28.8636	1.7741	2.3481
4/15/2019 9:02	0.2	3.0333	25.3617	-0.4167	11.8772	28.4636	1.7409	2.3108
4/15/2019 9:03	0.1333	2.6917	24.7783	-0.5	11.7033	28.0889	1.7037	2.3118
4/15/2019 9:04	0.1	2.325	24.9983	-0.3333	11.6833	27.9727	1.6897	2.3173
4/15/2019 9:05	0.1	2.0583	24.175	0.7833	11.4813	27.6222	1.6759	2.3087
4/15/2019 9:06	0.1	1.7	24.3517	-0.4333	11.4776	27.14	1.7058	2.3
4/15/2019 9:07	0.1	2.6	25.5767	-0.0833	11.6534	26.7083	1.7342	2.3389
4/15/2019 9:08	0.1	2.5167	26.285	-0.05	11.7653	26.3	1.7867	2.3783
4/15/2019 9:09	0.1167	2.525	25.285	-0.2167	11.5852	26.0583	1.7526	2.356
4/15/2019 9:10	0.1917	2.8833	25.7233	-0.2	11.6645	26.0273	1.7663	2.3612
4/15/2019 9:11	0.2	2.8333	24.735	0.3	11.4794	25.925	1.7215	2.3797
4/15/2019 9:12	0.2	2	24.4183	0.35	11.3754	25.7909	1.7278	2.3741
4/15/2019 9:13	0.1583	2.5667	22.56	0.1833	10.9913	25.6273	1.6097	2.3074
4/15/2019 9:14	0.1	2.625	22.0717	-0.0333	10.8108	25.41	1.6043	2.289
4/15/2019 9:15	0.1083	2.8417	22.665	-0.2833	10.9014	25.3091	1.644	2.3213
4/15/2019 9:16	0.1833	1.9833	20.9783	-0.75	10.6725	25.1091	1.565	2.3028
4/15/2019 9:17	0.2	2.5083	19.9467	-0.1333	10.4754	24.8455	1.5313	2.3031
4/15/2019 9:18	0.2	2.375	20.8083	0.2667	10.5407	24.62	1.556	2.3424
4/15/2019 9:19	0.1917	2.8167	20.235	0.3333	10.473	24.2727	1.5533	2.3607
4/15/2019 9:20	0.1917	2.5167	20.2883	0	10.4203	23.9625	1.5563	2.3561
4/15/2019 9:21	0.2	2.3583	20.7967	-0.2	10.4343	23.6727	1.5488	2.3308

4/15/2019 9:22	0.2	3.05	19.36	0.5667	10.2307	23.2455	1.494	2.3118
4/15/2019 9:23	0.2	2.8583	18.3733	0.55	9.9312	22.7818	1.4028	2.2664
4/15/2019 9:24	0.2583	2.4833	18.0983	0.2	10.3508	22.1636	1.3472	2.2468
4/15/2019 9:25	0.3833	2.7917	17.215	0.4667	11.005	21.5	1.2357	2.2375
4/15/2019 9:26	0.6417	3.2167	13.5583	0.9	10.7006	20.51	0.9794	2.0933
4/15/2019 9:27	0.9583	4.2667	13.205	1.0167	10.9116	19.3909	0.9184	2.0789
4/15/2019 9:28	1.325	4.2	12.195	1.35	10.5485	18.1091	0.7968	1.9752
4/15/2019 9:29	1.6583	5.0333	11.14	2.2167	10.1958	16.64	0.7106	1.9162
4/15/2019 9:30	2	4.6167	11.1917	2.55	10.2551	15.3455	0.6912	1.9505
4/15/2019 9:31	2.3083	5.475	10.4752	3.6167	10.292	13.95	0.6136	1.9308
4/15/2019 9:32	2.6	5.1417	10.7983	3.0667	10.2944	12.57	0.6141	1.8919
4/15/2019 9:33	2.825	6.7083	11.1833	3.75	9.9993	11.02	0.6004	1.9288
4/15/2019 9:34	3.05	7.8	11.2217	3.75	9.1625	9.4545	0.5379	1.8369
4/15/2019 9:35	3.4833	7.6	10.78	4.5333	8.0555	8.0556	0.4687	1.8081
4/15/2019 9:36	3.9333	8.1583	10.8117	4.8	8.1801	6.8	0.4574	1.7943
4/15/2019 9:37	4.25	8.5	10.6117	5.4333	7.9233	5.5636	0.4355	1.7697
4/15/2019 9:38	4.6	8.6667	10.5083	5.4	7.1927	4.6	0.3982	1.7518
4/15/2019 9:39	5.075	9.05	10.9517	6.2833	6.826	3.9333	0.3407	1.7345
4/15/2019 9:40	5.6833	10.2167	11.3833	6.5	6.3769	3.42	0.3323	1.7432
4/15/2019 9:41	6.175	10.2583	11.16	7.3	6.1135	3.17	0.3177	1.7841
4/15/2019 9:42	6.5833	10.2167	11.525	7.7333	6.1901	2.81	0.294	1.7525
4/15/2019 9:43	6.9167	10.8333	12.0017	7.6667	5.9339	2.54	0.2917	1.7748
4/15/2019 9:44	7.2583	10.9917	12.1817	8.65	5.6375	2.36	0.266	1.7326
4/15/2019 9:45	7.6	11.3667	12.145	8.5333	5.535	2.17	0.2547	1.7127
4/15/2019 9:46	7.725	10.8417	11.7017	7.7	5.8378	2.0182	0.2585	1.697
4/15/2019 9:47	7.8	11.25	12.17	8.9667	6.5654	1.9667	0.2297	1.6872
4/15/2019 9:48	8.0917	11.925	12.5867	9.6833	6.3738	1.85	0.2136	1.684
4/15/2019 9:49	8.675	12.725	13.0217	10.3833	5.791	1.7727	0.1983	1.6545
4/15/2019 9:50	9.575	13.3333	13.9083	11.7667	5.2282	1.6909	0.1707	1.6109
4/15/2019 9:51	10.2667	13.75	13.7017	11.1167	5.3225	1.6	0.1699	1.6188
4/15/2019 9:52	10.6833	14.2167	14.2117	11.1	5.1832	1.5636	0.1529	1.6241
4/15/2019 9:53	10.85	14.9417	13.805	11.9667	5.0158	1.4545	0.1464	1.5806
4/15/2019 9:54	10.9917	14.2667	13.3917	11.6167	4.5925	1.4	0.1519	1.5947
4/15/2019 9:55	11.1	14.1583	13.8542	11.3333	4.6787	1.4	0.1358	1.6135
4/15/2019 9:56	11.275	14.7417	14.8392	11.9167	4.5139	1.3182	0.1372	1.6069
4/15/2019 9:57	11.3917	14.3417	14.5767	12.0333	4.371	1.3	0.1305	1.5989
4/15/2019 9:58	11.625	14.7083	14.6133	12.6667	4.3511	1.2875	0.1269	1.5621
4/15/2019 9:59	11.8	15.625	15.0792	12.6667	4.3048	1.2	0.1202	1.5937
4/15/2019 10:00	11.975	15.0417	15.0267	12.6	4.2563	1.1444	0.1122	1.6191
4/15/2019 10:01	12.25	15.6583	14.2183	12.9667	4.1656	1.1	0.1126	1.6309
4/15/2019 10:02	12.4667	16.075	15.5983	13.7833	4.1041	1.1	0.1132	1.6642
4/15/2019 10:03	12.9083	16.8	16.0742	14.05	4.0511	1.1	0.0972	1.6544
4/15/2019 10:04	13.325	16.5917	16.0008	14.7	4.0366	1.1	0.1022	1.6348
4/15/2019 10:05	13.5667	16.7167	15.9075	14.35	4.0002	1.1	0.1005	1.6458
4/15/2019 10:06	13.5083	16.4083	15.9025	13.95	3.9797	1.1	0.0918	1.655
4/15/2019 10:07	13.3083	16.325	15.6142	13.5167	3.9778	1.1	0.1077	1.6555
4/15/2019 10:08	13.0417	15.8917	15.2483	13.55	3.9677	1.1	0.0946	1.6608

4/15/2019 10:09	12.7917	16.0333	15.18	13.15	3.9488	1.1	0.1019	1.6728
4/15/2019 10:10	12.5417	15.45	15.0258	12.3667	3.9673	1.1	0.1041	1.6641
4/15/2019 10:11	12.1667	14.5917	14.4367	12.0167	3.9647	1.1	0.1071	1.6852
4/15/2019 10:12	11.8833	14.1083	14.2875	12.0167	3.951	1.1	0.1157	1.7073
4/15/2019 10:13	11.5	13.6167	13.9883	11.5833	3.9223	1.1	0.0999	1.7193
4/15/2019 10:14	11.1333	13.3333	13.4908	10.7167	3.9018	1.1	0.1192	1.7288
4/15/2019 10:15	10.575	12.8833	12.8117	10.1167	3.9789	1.1583	0.128	1.7235
4/15/2019 10:16	9.85	11.2833	14.4933	9	11.4176	14.57	0.2271	1.7646
4/15/2019 10:17	8.575	8.8167	15.5692	6.7333	23.7007	42.7636	0.5457	1.8177
4/15/2019 10:18	6.75	6.6333	27.9792	3.85	34.2278	46.68	1.6436	1.9156
4/15/2019 10:19	4.1417	3.5083	59.4992	2.15	44.0584	78.52	4.8842	2.2686
4/15/2019 10:20	1.9917	2.6417	38.9633	1.05	44.0201	102.1333	5.9062	2.2918
4/15/2019 10:21	0.6333	1.7917	30.3992	0.0833	42.5418	97.2889	5.5848	2.2048
4/15/2019 10:22	0.325	3.1583	27.8042	0.4333	41.0215	89.4273	4.5212	2.121
4/15/2019 10:23	0.3417	2.9167	27.3083	0.8667	39.8598	81.73	4.0155	2.1093
4/15/2019 10:24	0.4	2.45	27.265	0.5167	41.5911	81.5333	3.7133	2.1298
4/15/2019 10:25	0.4	2.7583	27.595	1.15	42.1461	81.68	3.6015	2.1166
4/15/2019 10:26	0.35	2.6917	28.2233	0.3833	43.6343	83.8	3.333	2.1347
4/15/2019 10:27	0.3	2.675	27.0033	-0.2167	42.1973	83.7	3.2703	2.136
4/15/2019 10:28	0.2833	2.325	25.685	0.3167	41.3165	81.5	3.2082	2.117
4/15/2019 10:29	0.3	1.7917	24.9958	0.3	39.9417	81.1091	3.095	2.1043
4/15/2019 10:30	0.3	2.7667	23.2292	0.5667	37.8513	80.54	2.9362	2.0785
4/15/2019 10:31	0.3	3.3273	21.3417	-0.1333	35.1945	79.7	2.762	2.0816
4/15/2019 10:32	0.3	1.14	20.5642	1.0333	34.6	78.67	2.694	2.0748
4/15/2019 10:33	0.3	2.8833	20.0342	0.1167	34.6	77.3111	2.6167	2.0696
4/15/2019 10:34	0.3	2.4333	20.0225	-0.0667	34.4948	76.0444	2.588	2.0851
4/15/2019 10:35	0.3	2.1833	19.4867	0.2833	33.7078	74.8727	2.5838	2.0729
4/15/2019 10:36	0.3	2.7333	19.3908	0.4667	33.8546	73.6	2.5928	2.097
4/15/2019 10:37	0.3	2.4667	18.5708	0.2333	32.8778	72.1273	2.5375	2.0962
4/15/2019 10:38	0.3	2.9	18.8175	0.1667	32.858	70.68	2.5423	2.0985
4/15/2019 10:39	0.2583	2.7167	20.1175	0.2167	35.2319	69.8	2.7458	2.1226
4/15/2019 10:40	0.2	2.5667	19.5625	-0.0333	34.4267	68.7667	2.7078	2.1206
4/15/2019 10:41	0.1917	2.7	19.4608	0.1	33.9134	67.75	2.6547	2.1381
4/15/2019 10:42	0.1417	3.0667	19.2592	0.0667	33.5173	67.0818	2.6608	2.1288
4/15/2019 10:43	0.15	2.3833	18.1883	0.0333	32.9498	66.6546	2.601	2.1101
4/15/2019 10:44	0.2	2.65	17.32	-0.0167	31.6028	66.24	2.4808	2.1056
4/15/2019 10:45	0.2167	2.9	16.9417	0.1	30.9611	65.8818	2.4655	2.1194
4/15/2019 10:46	0.2667	2.5333	16.7275	-0.2	30.529	65.56	2.4357	2.1392
4/15/2019 10:47	0.3	2.5	16.1067	-0.25	29.6146	65	2.3557	2.0996
4/15/2019 10:48	0.325	2.5667	15.5883	-0.3833	28.993	64.2909	2.3242	2.1178
4/15/2019 10:49	0.3	2.7	16.025	-0.3333	29.7682	64	2.3975	2.1294
4/15/2019 10:50	0.3	3.25	14.7525	-0.1667	28.2681	63.5182	2.2795	2.1203
4/15/2019 10:51	0.3	2.6167	14.3767	-0.1333	27.5739	62.5333	2.2432	2.0983
4/15/2019 10:52	0.3	2.8333	14.54	0.0333	27.5799	61.31	2.224	2.1025
4/15/2019 10:53	0.275	2.3667	16.155	-0.2	29.8677	60.78	2.4595	2.1635
4/15/2019 10:54	0.2	2.6	15.8825	-0.1833	29.7121	60.2636	2.4432	2.178
4/15/2019 10:55	0.275	1.9667	15.3408	-0.3833	28.7931	59.58	2.3747	2.1488

4/15/2019 10:56	0.275	2.2833	14.9592	0.2833	28.3582	59.09	2.3503	2.1401
4/15/2019 10:57	0.25	2.2667	14.0333	0.1333	27.3253	58.49	2.2557	2.1341
4/15/2019 10:58	0.2	2.6667	13.895	0.0667	26.5618	57.96	2.2172	2.1388
4/15/2019 10:59	0.2	2.7	13.0667	-0.1	25.4887	57.3455	2.1303	2.105
4/15/2019 11:00	0.2	1.8667	12.7933	0.0167	24.7808	56.8	2.0698	2.086
4/15/2019 11:01	0.2333	2.8333	12.5308	-0.1667	25.0182	56.2	2.0965	2.0888
4/15/2019 11:02	0.2	2.3	13.0225	-0.2333	25.5218	55.65	2.1393	2.1154
4/15/2019 11:03	0.2	3.5	13.0167	-0.0667	25.2998	55.3091	2.1448	2.1148
4/15/2019 11:04	0.2	3.8667	12.3067	-0.4833	24.3865	54.8889	2.0698	2.1111
4/15/2019 11:05	0.1	2.9	12.1	-0.0167	24.1735	54.1909	2.0513	2.1252
4/15/2019 11:06	0.1	2.4667	11.6958	-0.4167	23.7074	53.12	2.0045	2.1104
4/15/2019 11:07	0.1	2.0333	12.6008	-0.4833	24.8608	52.4818	2.1325	2.1368
4/15/2019 11:08	0.1	2.2	13.1342	-0.1833	25.0765	51.9818	2.1595	2.161
4/15/2019 11:09	0.0333	2.1667	12.3667	0.1667	24.7784	51.6818	2.1339	2.1458
4/15/2019 11:10	0	2.5333	11.865	-0.5167	24.0438	51.4333	2.0768	2.1312
4/15/2019 11:11	0.0167	2.35	11.4083	-0.0833	23.4987	51.18	2.0243	2.1116
4/15/2019 11:12	0.1	2.75	11.0825	-0.55	22.3679	50.7727	1.9333	2.1009
4/15/2019 11:13	0.1	2.95	10.8083	0.1167	21.9937	50.5	1.9	2.0838
4/15/2019 11:14	0.1083	2.4333	10.6725	-0.9333	22.0578	50.05	1.9398	2.1118
4/15/2019 11:15	0.2	2.8667	10.1243	-0.55	21.5543	49.4727	1.871	2.102
4/15/2019 11:16	0.2	2.6833	10.2093	-0.15	21.5829	48.9727	1.8997	2.1194
4/15/2019 11:17	0.2	2.5667	10.5067	-0.05	21.7053	48.5455	1.9154	2.1387
4/15/2019 11:18	0.2	3.05	10.7658	-0.7833	21.9038	48.31	1.9329	2.1828
4/15/2019 11:19	0.2	2.3167	11.1092	-0.15	22.1297	48.0909	1.98	2.1713
4/15/2019 11:20	0.2	2.5667	11.1275	-0.8667	22.2079	47.71	1.9784	2.1922
4/15/2019 11:21	0.2	2.0667	10.7708	-0.2833	21.778	47.22	1.9358	2.1642
4/15/2019 11:22	0.2	2.7833	10.8817	-0.0833	21.5982	46.76	1.9508	2.1597
4/15/2019 11:23	0.25	2.6833	10.59	0.15	21.0679	46.3083	1.8943	2.1683
4/15/2019 11:24	0.3	3.0833	10.2583	-0.2667	20.6404	46.08	1.8559	2.1693
4/15/2019 11:25	0.3	2.7167	9.9033	-0.1833	20.2092	45.8111	1.8296	2.1458
4/15/2019 11:26	0.3167	2.4667	9.5377	-0.0167	19.7254	45.3333	1.7902	2.1527
4/15/2019 11:27	0.3	2.3333	9.435	-0.5	19.3951	45.0182	1.7723	2.1458
4/15/2019 11:28	0.3	2.2833	9.1382	-0.0833	19.5602	44.72	1.7813	2.1402
4/15/2019 11:29	0.2417	2.35	8.664	-0.2	19.7653	44.52	1.8083	2.1553
4/15/2019 11:30	0.225	2.6333	9.0603	-0.7333	19.4753	44.1818	1.7889	2.1649
4/15/2019 11:31	0.2	2.7667	9.1228	-0.15	19.1931	43.7727	1.7425	2.156
4/15/2019 11:32	0.2	1.9667	9.7273	0.2333	19.5434	43.2417	1.8064	2.1719
4/15/2019 11:33	0.175	2.1333	9.3938	0.4333	19.657	42.8	1.7614	2.1718
4/15/2019 11:34	0.1	2.2333	9.323	-0.7667	19.657	42.41	1.7892	2.1707
4/15/2019 11:35	0.125	1.9333	9.6535	-0.35	19.2369	41.9727	1.762	2.1762
4/15/2019 11:36	0.2	2.85	8.945	-0.5333	18.7235	41.6364	1.7336	2.1598
4/15/2019 11:37	0.2833	3.15	8.6317	-0.2833	17.4007	41.1	1.5913	2.1094
4/15/2019 11:38	0.6667	3.8667	8.7217	1.65	15.952	40.0455	1.3374	2.0582
4/15/2019 11:39	1.3667	5.8	8.2445	2.15	15.3542	38.83	1.1747	1.999
4/15/2019 11:40	2.075	5.0667	7.9867	2.8167	14.8708	36.9545	1.0386	1.9257
4/15/2019 11:41	2.6667	6.0833	8.0072	3.8	14.7373	35.0091	0.9024	1.8869
4/15/2019 11:42	3.2333	7.4833	8.6162	4.25	14.2793	32.5	0.8061	1.8481

4/15/2019 11:43	3.825	7.5333	8.6445	4.25	13.9961	30	0.7496	1.8256
4/15/2019 11:44	4.15	6.9	8.1633	4.1	15.1703	27.4	0.7465	1.779
4/15/2019 11:45	4.0917	7.4167	8.0525	4.2	16.6457	24.7909	0.7553	1.7852
4/15/2019 11:46	4.2545	8.3667	8.8457	5.6333	14.8799	22.2	0.6116	1.7373
4/15/2019 11:47	4.575	8.4	8.7255	5.6	14.3363	19.3	0.5719	1.7323
4/15/2019 11:48	4.9833	8.0833	8.4455	5.2667	15.0873	16.46	0.5555	1.7424
4/15/2019 11:49	5.225	8.8167	8.7745	6	15.1914	13.8818	0.4864	1.7007
4/15/2019 11:50	5.575	8.95	9.2432	6.5333	14.7878	11.58	0.4504	1.6892
4/15/2019 11:51	6.15	9.5167	9.199	6.9	14.4179	9.93	0.397	1.7015
4/15/2019 11:52	6.65	10.5833	9.855	7.4833	13.9582	8.4545	0.3834	1.689
4/15/2019 11:53	7	10.3167	9.6203	7.9167	13.5509	7.3	0.3754	1.6703
4/15/2019 11:54	7.5	12.1	11.4067	9.2167	12.6278	6.3667	0.3231	1.6254
4/15/2019 11:55	7.9333	11.6667	10.825	8.5333	12.7022	5.6364	0.3141	1.6393
4/15/2019 11:56	8.2167	11.0667	10.3413	8.2833	13.3382	4.94	0.3174	1.6863
4/15/2019 11:57	8.3583	11.4	10.2793	8.9667	12.5684	4.3545	0.2917	1.6569
4/15/2019 11:58	8.4167	11.5167	10.5133	8.9833	11.9708	3.84	0.2871	1.6468
4/15/2019 11:59	8.6417	11.8167	11.1	9.6833	11.7873	3.47	0.2764	1.6457
4/15/2019 12:00	9.2083	13.4167	11.87	10.5167	10.4878	3.1273	0.2489	1.6304
4/15/2019 12:01	9.85	13.3333	12.7383	10.8167	9.9375	2.8	0.2413	1.6137
4/15/2019 12:02	10.7667	15.1	13.7633	12.1167	9.2134	2.5333	0.2167	1.6034
4/15/2019 12:03	11.4667	15.05	13.975	13.0833	8.7424	2.3333	0.2087	1.5932
4/15/2019 12:04	11.9917	15.6	14.2733	12.75	8.6224	2.11	0.1825	1.6224
4/15/2019 12:05	12.3417	15.5	14.495	13.4667	8.3256	2	0.1791	1.613
4/15/2019 12:06	12.525	15.7667	14.315	12.6333	8.4339	1.89	0.1718	1.5858
4/15/2019 12:07	12.8167	17	14.795	13.5167	8.0352	1.8	0.1558	1.5954
4/15/2019 12:08	13.1083	16	14.9817	13.8	7.3959	1.8	0.1547	1.5993
4/15/2019 12:09	13.3417	16.5333	15.3267	14.4167	7.2937	1.7273	0.1469	1.5919
4/15/2019 12:10	13.4917	16	15.1283	14.0333	7.2249	1.7	0.1554	1.5706
4/15/2019 12:11	13.5583	16.8667	15.43	13.95	6.9298	1.6583	0.1575	1.5636
4/15/2019 12:12	13.7083	16.6333	15.735	14.6167	6.976	1.6	0.1422	1.5755
4/15/2019 12:13	13.9333	17.1167	15.4283	14.5333	7.0708	1.6	0.1479	1.6013
4/15/2019 12:14	14.4167	18.7	16.6883	15.8333	6.664	1.6	0.1239	1.5753
4/15/2019 12:15	14.8417	18.15	16.205	15.35	6.442	1.6	0.1205	1.5793
4/15/2019 12:16	15.2917	19.5	17.2183	16.3333	6.0556	1.6	0.1135	1.5768
4/15/2019 12:17	15.6833	19.1833	17.965	17.05	5.7503	1.6	0.0896	1.5636
4/15/2019 12:18	16.0333	19.05	17.8517	17.35	5.672	1.6	0.0995	1.5627
4/15/2019 12:19	16.2583	19.3	18.215	17.6833	5.4888	1.57	0.0987	1.5737
4/15/2019 12:20	16.1333	18.5667	17.3883	16.55	5.4438	1.5	0.0894	1.5798
4/15/2019 12:21	16	19.3333	17.8767	16.25	5.3852	1.5	0.1099	1.5738
4/15/2019 12:22	16.025	19.8667	18.14	17.6	5.0263	1.5	0.0894	1.5783
4/15/2019 12:23	16.2917	19.1667	18.4183	17.55	4.9012	1.43	0.1002	1.5746
4/15/2019 12:24	16.45	19.3	18.2633	16.4	4.8888	1.4	0.0958	1.5887
4/15/2019 12:25	16.5833	20.5667	18.855	17.7667	4.6943	1.3545	0.0802	1.5885
4/15/2019 12:26	16.8	20.75	18.8967	17.4667	4.5617	1.3	0.0854	1.5997
4/15/2019 12:27	16.7583	19.4167	17.53	15.9833	4.7144	1.2545	0.0879	1.5933
4/15/2019 12:28	16.5417	18.4667	17.8283	16.9167	4.5908	1.2	0.0905	1.594
4/15/2019 12:29	16.4167	19.9	18.2467	17.6667	4.4412	1.1909	0.089	1.5809

4/15/2019 12:30	16.8583	20.2167	18.8483	18.1167	4.2085	1.1	0.0658	1.567
4/15/2019 12:31	17.2	20.4833	19.2267	18.4833	4.0413	1.1	0.0651	1.5519
4/15/2019 12:32	17.3083	20.4167	18.8867	17.8667	4.0287	1.1	0.0757	1.5774
4/15/2019 12:33	17.1833	20.0667	18.455	17.9833	4.0213	1.0455	0.0688	1.5838
4/15/2019 12:34	16.7833	19.3167	17.8533	16	4.0464	1	0.085	1.5903
4/15/2019 12:35	16.3083	18.1667	17.2433	16.1333	4.047	1	0.071	1.5857
4/15/2019 12:36	15.6333	17.4833	16.345	15.2	4.0983	1	0.0839	1.6014
4/15/2019 12:37	15.0583	17.2667	15.7683	15.05	4.1599	1	0.0869	1.6158
4/15/2019 12:38	14.775	17.6833	16.405	15.5167	4.109	1	0.0691	1.6378
4/15/2019 12:39	14.4583	16.4167	15.43	14.5667	4.13	1	0.0911	1.653
4/15/2019 12:40	14.1583	16.0667	15.2917	13.5667	4.0932	1.09	0.0809	1.6552
4/15/2019 12:41	13.725	15.5167	15.3017	13.7167	4.0774	1.1	0.0838	1.6405
4/15/2019 12:42	13.7333	17.2	14.8667	14.8	4.0498	1.12	0.0895	1.6398
4/15/2019 12:43	13.7833	16.4833	15.405	13.6167	4.0195	1.2	0.0759	1.6634
4/15/2019 12:44	13.75	15.7667	14.9983	13.8167	4.0528	1.2	0.0894	1.7043
4/15/2019 12:45	13.2667	15.2833	14.2317	13.1667	3.995	1.2545	0.0862	1.691
4/15/2019 12:46	12.85	15	14.1067	12.5833	4.0064	1.3	0.081	1.7101
4/15/2019 12:47	12.35	15.4833	13.6117	12.4333	4.016	1.3	0.0885	1.7055
4/15/2019 12:48	11.9333	14.5167	13.1483	11.2833	3.9997	1.3	0.0779	1.7059
4/15/2019 12:49	11.475	14.1667	12.9233	10.9333	3.9798	1.35	0.093	1.723
4/15/2019 12:50	11.15	13.65	12.9517	11.1167	3.9908	1.4	0.0938	1.7783
4/15/2019 12:51	10.825	12.3667	12.3083	10.0167	3.9614	1.4	0.0833	1.75
4/15/2019 12:52	10.5583	12.3667	11.8483	10	3.9633	1.4	0.0951	1.7511
4/15/2019 12:53	10.3	12.6333	11.9967	10.6833	3.927	1.4364	0.0884	1.7468
4/15/2019 12:54	10.1917	12.25	11.9183	10.3167	3.939	1.5	0.0941	1.7517
4/15/2019 12:55	10.0667	12.5833	11.4567	9.6333	3.907	1.5	0.0925	1.7673
4/15/2019 12:56	9.8667	11.7	11.6267	9.8667	3.9174	1.55	0.0856	1.7858
4/15/2019 12:57	9.6583	12.0667	11.4733	9.7667	3.9014	1.52	0.095	1.8008
4/15/2019 12:58	9.5333	11.9	11.2983	9.3	3.8959	1.6	0.0865	1.7743
4/15/2019 12:59	9.4167	11.2	11.145	9.3167	3.8604	1.6	0.0945	1.7889
4/15/2019 13:00	9.35	12.0333	11.0433	9.1167	3.8758	1.6	0.0916	1.7923
4/15/2019 13:01	9.1	11	11.1858	7.8333	5.2933	1.72	0.1161	1.7989
4/15/2019 13:02	8.175	8.7	22.0225	6.7667	24.9019	2.76	0.429	1.8893
4/15/2019 13:03	6.3583	6.1833	28.9692	4.1667	27.7515	4.8	0.7709	1.9633
4/15/2019 13:04	4.1917	4.5667	44.8917	3.15	47.195	39.52	1.3979	2.0628
4/15/2019 13:05	2.3417	3.1167	61.6583	2.8667	57.9042	75.7546	2.5554	2.2667
4/15/2019 13:06	1.2636	3.0167	49.5925	1.6833	48.7396	75.0909	2.0688	2.2262
4/15/2019 13:07	0.6417	1.6833	46.0267	1.8333	49.65	65.2182	1.9583	2.2081
4/15/2019 13:08	0.4667	3.0833	55.7433	2.7333	56.9483	85.63	3.6688	2.4234
4/15/2019 13:09	0.4	1.9833	53.1142	1.4833	53.5118	87.9818	3.2865	2.4458
4/15/2019 13:10	0.4	3.15	48.8658	1.6667	49.3036	80.67	3.0058	2.3509
4/15/2019 13:11	0.4	3.0167	51.8058	1.4333	47.4847	75.9727	3.6295	2.4106
4/15/2019 13:12	0.3667	3.1	45.9392	0.6333	46.1112	66.1455	2.7435	2.3242
4/15/2019 13:13	0.3167	2.95	45.665	0.6	47.6963	69.14	2.5742	2.3283
4/15/2019 13:14	0.3	2.1333	45.2692	0.6167	47.1555	69.1818	2.7562	2.3197
4/15/2019 13:15	0.2583	2.8167	42.81	0.8667	44.946	65.98	2.7288	2.2886
4/15/2019 13:16	0.275	2.0667	40.2383	0.6	43.0673	63.9364	2.7018	2.2748

4/15/2019 13:17	0.3	2.5667	37.5275	0.4833	41.2727	63.6	2.5795	2.2173
4/15/2019 13:18	0.3	2.75	35.4967	0.5	39.5222	63.14	2.6503	2.2198
4/15/2019 13:19	0.3	2.2	33.7625	0.15	38.2628	62.5909	2.6798	2.2111
4/15/2019 13:20	0.2583	3.0333	32.8658	0.2667	37.2198	61.7333	2.7313	2.1967
4/15/2019 13:21	0.2	2.1833	33.4517	0.2833	38.1821	61.0909	2.6358	2.2378
4/15/2019 13:22	0.175	2.6	33.9642	0.7167	39.0863	60.5333	2.721	2.2733
4/15/2019 13:23	0.1	2.8667	31.9483	0.1167	37.7003	59.9083	2.6895	2.2462
4/15/2019 13:24	0.1333	3.2	31.1433	0.1667	36.6098	59.2083	2.6842	2.2278
4/15/2019 13:25	0.2	2.2833	30.4958	0.1833	36.9297	58.51	2.5462	2.2104
4/15/2019 13:26	0.2	2.8167	33.9558	0.5333	39.3031	58.0182	2.5162	2.2728
4/15/2019 13:27	0.15	2.2333	33.5208	0.4333	39.0727	57.5727	2.5145	2.2922
4/15/2019 13:28	0.1	2.3333	32.1475	0.35	38.084	57.03	2.4768	2.2873
4/15/2019 13:29	0.1	2.9333	31.5742	0.1833	37.789	56.64	2.4712	2.284
4/15/2019 13:30	0.2	2.7667	29.9117	-0.0833	36.0332	56.3167	2.3838	2.2682
4/15/2019 13:31	0.2083	2.6	27.8567	0.3667	34.6177	55.9455	2.323	2.2204
4/15/2019 13:32	0.2833	2.7833	26.6917	0.55	33.2756	55.5273	2.276	2.2117
4/15/2019 13:33	0.2	3.3667	27.3092	0.3667	33.7928	55.2	2.3005	2.2152
4/15/2019 13:34	0.2	3.2667	25.9217	-0.1667	32.8208	54.4333	2.1892	2.1883
4/15/2019 13:35	0.225	2.55	25.4092	0.2833	32.2949	53.7455	2.2285	2.2156
4/15/2019 13:36	0.3	2.8167	24.0842	0.15	32.284	53.0667	2.1547	2.2135
4/15/2019 13:37	0.3	3.5	23.8717	0.6333	32.0987	52.54	2.1422	2.2184
4/15/2019 13:38	0.2583	2.9	24.47	-0.35	31.7495	51.8083	2.177	2.2149
4/15/2019 13:39	0.2	3.4333	24.9775	-0.1	32.008	50.9583	2.1722	2.2217
4/15/2019 13:40	0.1833	2.2	25.11	-0.15	32.5183	50.2909	2.2175	2.2358
4/15/2019 13:41	0.125	2.0833	25.3817	-0.3667	32.3407	49.54	2.2098	2.2421
4/15/2019 13:42	0.1	2.6333	24.0325	0.15	31.4561	48.9833	2.1618	2.2627
4/15/2019 13:43	0.1917	2.2833	24.0408	-0.35	31.2836	48.68	2.1748	2.2383
4/15/2019 13:44	0.2	3.0333	22.7083	-0.2333	29.959	48.3	2.0587	2.2049
4/15/2019 13:45	0.2	2.5	22.4033	0.3833	29.6638	47.8333	2.0683	2.2116
4/15/2019 13:46	0.2	2.85	21.5608	0.15	29.0338	47.4909	2.0182	2.2064
4/15/2019 13:47	0.2	2	21.7025	0.1333	29.1273	47.15	2.0238	2.2159
4/15/2019 13:48	0.2917	2.4667	20.6875	-0.25	28.2385	46.775	1.9808	2.2366
4/15/2019 13:49	0.3	2.3333	20.1592	0.2333	27.8133	46.3417	1.9627	2.206
4/15/2019 13:50	0.3	1.7667	19.8908	0.0667	27.5289	45.8417	1.9513	2.1958
4/15/2019 13:51	0.3	2.2667	20.68	-0.1167	28.3065	45.4909	2.0222	2.229
4/15/2019 13:52	0.3	2.1333	20.4175	0.5333	27.9543	45.1083	1.977	2.2366
4/15/2019 13:53	0.3	3.05	19.7967	-0.05	27.4431	44.64	1.9612	2.2391
4/15/2019 13:54	0.3	2.5333	19.22	-0.1833	26.9205	44.0364	1.938	2.2332
4/15/2019 13:55	0.3667	1.7833	19.7258	0.35	27.2648	43.4917	1.9617	2.2205
4/15/2019 13:56	0.4	3.0667	19.985	-0.2333	27.6985	43.2	1.9945	2.2355
4/15/2019 13:57	0.4	2.0667	19.505	-0.2833	26.9117	43.1	1.9318	2.2742
4/15/2019 13:58	0.4	2.6833	19.1592	-0.0333	26.3178	42.775	1.912	2.2773
4/15/2019 13:59	0.4	3.3	17.66	0.1	25.4764	42.6083	1.8573	2.2656
4/15/2019 14:00	0.4	2.6833	17.0008	-0.1667	24.5913	42.2364	1.783	2.2651
4/15/2019 14:01	0.4333	2.4	17.8425	0.15	25.2503	42.0636	1.8538	2.2783
4/15/2019 14:02	0.5	2.8167	17.2467	0.5167	24.8818	41.83	1.8238	2.2642
4/15/2019 14:03	0.5	2.3	16.4508	-0.3667	23.9111	41.3636	1.766	2.2923

4/15/2019 14:04	0.5	2.0167	15.7692	0.1	23.1886	40.85	1.7167	2.2933
4/15/2019 14:05	0.5	2.4333	15.95	0	23.6231	40.275	1.7378	2.3046
4/15/2019 14:06	0.4917	2.5	16.4958	-0.1	23.9471	39.9455	1.791	2.3027
4/15/2019 14:07	0.4	2.8833	17.0767	-0.0333	24.1158	39.65	1.8082	2.2885
4/15/2019 14:08	0.4	2.7167	16.9608	-0.1167	24.2829	39.3417	1.8073	2.3038
4/15/2019 14:09	0.4	2.3333	17.1233	0.0833	24.4404	38.9833	1.8377	2.3064
4/15/2019 14:10	0.4	2.35	16.3992	-0.25	23.7329	38.7417	1.7722	2.3367
4/15/2019 14:11	0.4	3	16.1833	0.4	23.2569	38.3182	1.7578	2.3368
4/15/2019 14:12	0.4	2.6667	15.8717	-0.1	22.8648	38.1182	1.7382	2.3418
4/15/2019 14:13	0.4	2.7333	14.9475	0.2333	22.1096	37.78	1.6618	2.3245
4/15/2019 14:14	0.4	2.5333	14.6108	0.2	21.8261	37.2833	1.6657	2.3048
4/15/2019 14:15	0.4	3.0333	14.3383	-0.2	21.3835	36.8546	1.6347	2.2989
4/15/2019 14:16	0.4	2.3667	14.1517	-0.2	21.1506	36.46	1.5922	2.3278
4/15/2019 14:17	0.425	2.3	14.0883	-0.3833	21.1129	36.09	1.605	2.32
4/15/2019 14:18	0.4083	2.2167	13.4583	-0.65	20.8157	35.6083	1.5817	2.3288
4/15/2019 14:19	0.3333	2.2	13.9875	-0.3333	21.2987	35.2	1.6407	2.3694
4/15/2019 14:20	0.2333	2.6167	14.1858	0	21.1481	34.8	1.6253	2.348
4/15/2019 14:21	0.2	2.5333	13.3917	-0.2	20.3533	34.2364	1.56	2.3328
4/15/2019 14:22	0.1833	3.6833	14.0392	0.8	20.3604	33.6182	1.5708	2.3457
4/15/2019 14:23	0.1	3.2333	14.2408	0.2	21.0297	33.3182	1.6257	2.3857
4/15/2019 14:24	0.2	3.5833	12.7775	-0.25	19.5536	32.8455	1.5157	2.3506
4/15/2019 14:25	0.2917	3.1833	12.2108	0.7333	18.9761	32.2818	1.4087	2.2986
4/15/2019 14:26	0.6083	4.4167	12.305	1.15	18.3478	31.55	1.2848	2.2731
4/15/2019 14:27	1.375	5.8667	11.1283	3.3333	15.3376	30.3	0.9522	2.1613
4/15/2019 14:28	2.1	5.7	10.0315	2.85	15.3064	28.7417	0.8947	2.1331
4/15/2019 14:29	2.7667	7.3667	10.0352	3.3667	14.8515	27.1273	0.8237	2.1143
4/15/2019 14:30	3.175	7.2	9.997	4.1667	14.1976	25.3583	0.7148	2.0942
4/15/2019 14:31	3.85	8.55	10.2178	5.5833	13.7479	23.4333	0.6481	2.0563
4/15/2019 14:32	4.4333	7.6667	9.8453	5	13.6745	21.1167	0.6409	2.0341
4/15/2019 14:33	4.875	8.3167	10.2642	4.9833	13.3636	18.98	0.5916	2.053
4/15/2019 14:34	5.4	9.9333	11.1	7.2833	12.7168	16.8909	0.5407	2.0843
4/15/2019 14:35	6.0917	10.25	11.225	6.95	11.9658	14.5583	0.4768	2.0676
4/15/2019 14:36	6.6583	9.9333	11.195	7.75	11.9958	12.2091	0.4685	2.0684
4/15/2019 14:37	6.9917	10.6	11.395	7.8167	11.972	10.12	0.4544	2.0486
4/15/2019 14:38	7.3417	11.4667	12.1917	8.9667	11.5252	8.2	0.4032	2.0901
4/15/2019 14:39	7.825	11.2	12.1433	8.8833	10.6328	6.7545	0.3897	2.0589
4/15/2019 14:40	8.3917	12.25	12.4067	10.2667	9.9534	5.83	0.3619	2.0309
4/15/2019 14:41					9.1789	5.0111	0.3346	2.0044
4/15/2019 14:42							0.3338	1.988
4/15/2019 14:43							0.2916	1.9777
4/15/2019 14:44							0.2768	2.0052
4/15/2019 14:45							0.275	1.9449
4/15/2019 14:46							0.2281	1.9285
4/15/2019 14:47							0.2219	1.941
4/15/2019 14:48							0.2061	1.9414
4/15/2019 14:49							0.2243	1.9108
4/15/2019 14:50							0.1889	1.8963

4/15/2019 14:51	0.1696	1.8887
4/15/2019 14:52	0.1797	1.8715
4/15/2019 14:53	0.1573	1.8496
4/15/2019 14:54	0.1617	1.8396
4/15/2019 14:55	0.1685	1.8369
4/15/2019 14:56	0.1522	1.8294
4/15/2019 14:57	0.1552	1.8109
4/15/2019 14:58	0.1175	1.8163
4/15/2019 14:59	0.1356	1.8186
4/15/2019 15:00	0.1175	1.7578
4/15/2019 15:01	0.1055	1.7839
4/15/2019 15:02	0.1165	1.7449
4/15/2019 15:03	0.0953	1.7179
4/15/2019 15:04	0.1018	1.7172
4/15/2019 15:05	0.1013	1.7217
4/15/2019 15:06	0.0946	1.7168
4/15/2019 15:07	0.1367	1.7016
4/15/2019 15:08	0.1222	1.705
4/15/2019 15:09	0.113	1.6933
4/15/2019 15:10	0.1197	1.7029
4/15/2019 15:11	0.0966	1.6911
4/15/2019 15:12	0.1082	1.6575
4/15/2019 15:13	0.094	1.624
4/15/2019 15:14	0.0798	1.6315
4/15/2019 15:15	0.0973	1.6573
4/15/2019 15:16	0.0839	1.6683
4/15/2019 15:17	0.095	1.6534
4/15/2019 15:18	0.0942	1.6482
4/15/2019 15:19	0.0792	1.6468
4/15/2019 15:20	0.0822	1.6653
4/15/2019 15:21	0.0694	1.6578
4/15/2019 15:22	0.0857	1.6486
4/15/2019 15:23	0.0944	1.6163
4/15/2019 15:24	0.0863	1.6365
4/15/2019 15:25	0.0859	1.6431
4/15/2019 15:26	0.0815	1.6451
4/15/2019 15:27	0.076	1.6211
4/15/2019 15:28	0.0744	1.6013
4/15/2019 15:29	0.0592	1.6152
4/15/2019 15:30	0.0709	1.6443
4/15/2019 15:31	0.0633	1.6438
4/15/2019 15:32	0.0702	1.6562
4/15/2019 15:33	0.0697	1.6873
4/15/2019 15:34	0.0581	1.6576
4/15/2019 15:35	0.0713	1.6389
4/15/2019 15:36	0.0668	1.6273
4/15/2019 15:37	0.0607	1.6524

4/15/2019 15:38							0.0756	1.6758
4/15/2019 15:39							0.06	1.6713
4/15/2019 15:40							0.0649	1.6805
4/15/2019 15:41							0.0736	1.6674
4/15/2019 15:42							0.0608	1.7049
4/15/2019 15:43							0.073	1.7108
4/15/2019 15:44							0.0662	1.7137
4/15/2019 15:45							0.0677	1.7186
4/15/2019 15:46							0.0741	1.7259
4/15/2019 15:47							0.0657	1.7208
4/15/2019 15:48							0.0806	1.6676
4/15/2019 15:49							0.0612	1.6787
4/15/2019 15:50							0.0688	1.6943
4/15/2019 15:51							0.0684	1.7154
4/15/2019 15:52							0.0615	1.7199
4/15/2019 15:53							0.0749	1.71
4/15/2019 15:54							0.0651	1.7443
4/15/2019 15:55							0.0725	1.7299
4/15/2019 15:56							0.0758	1.7104
4/15/2019 15:57							0.0619	1.7318
4/15/2019 15:58							0.0737	1.7129
4/15/2019 15:59							0.0651	1.7405
4/15/2019 16:00							0.0766	1.7463
4/15/2019 16:01							0.0784	1.7309
4/15/2019 16:02							0.0548	1.7333
4/15/2019 16:03							0.0776	1.7064
4/15/2019 16:04							0.0651	1.7511
4/15/2019 16:05							0.07	1.753
4/15/2019 16:06							0.0751	1.7128
4/15/2019 16:07							0.0662	1.7334
4/15/2019 16:08							0.0783	1.744
4/15/2019 16:09							0.0687	1.7616
4/15/2019 16:10							0.0705	1.7861
4/15/2019 16:11							0.0756	1.7652
4/15/2019 16:12	5.8	5.4	5.5065	4.6333	3.2697	1.5333	0.07	1.7852
4/15/2019 16:13	5.8	5.4333	6.1535	4.7	3.2833	1.4364	0.077	1.807
4/15/2019 16:14	5.7583	5.3833	6.8013	5	3.303	1.5182	0.0639	1.7866
4/15/2019 16:15	5.7	5.3333	6.8278	4.9667	3.3208	1.69	0.0765	1.7848
4/15/2019 16:16	5.6417	4.8667	7.4093	4.9167	3.3179	1.6417	0.0787	1.7886
4/15/2019 16:17	5.5727	4.45	7.3403	4.3	3.3408	1.7818	0.0614	1.7696
4/15/2019 16:18	5.45	5.3667	7.5869	4.9667	3.3286	1.7167	0.0781	1.7718
4/15/2019 16:19	5.3333	4.65	7.6809	4.4333	3.3258	1.8	0.0664	1.7808
4/15/2019 16:20	5.3	4.5167	7.1639	5.25	3.3364	2.1	0.0686	1.7658
4/15/2019 16:21	5.3	4.7167	7.0398	4.6667	3.3178	2.1	0.0777	1.7511
4/15/2019 16:22	5.2167	5.4333	6.5433	4.35	3.3428	2.1	0.0622	1.7858
4/15/2019 16:23	5.15	4.7833	6.8215	4.6833	3.3623	2	0.0741	1.766
4/15/2019 16:24	5.025	4.4167	6.758	4.5	3.3589	2	0.065	1.7857

4/15/2019 16:25	4.8833	3.8833	7.0699	4.6833	3.3678	2	0.0691	1.7876
4/15/2019 16:26	4.7417	4.05	7.0151	4.3333	3.3626	1.9833	0.0748	1.7779
4/15/2019 16:27	4.625	4.1333	7.0106	3.8333	3.3639	1.9	0.0684	1.7738
4/15/2019 16:28	4.6	4.05	7.1472	4	3.3727	1.9	0.0792	1.7738
4/15/2019 16:29	4.5333	4.2833	6.8643	4.35	3.395	1.8636	0.0652	1.7614
4/15/2019 16:30	4.4833	4.0333	7.1051	4.05	3.3844	1.8	0.0696	1.7988
4/15/2019 16:31	4.4	3.7	6.6567	4.4167	3.3727	1.8	0.0758	1.8269
4/15/2019 16:32	4.325	3.1333	6.5868	4.1	3.3779	1.8	0.0638	1.7943
4/15/2019 16:33	4.225	3.8333	6.6686	3.65	3.3806	1.8	0.0711	1.8024
4/15/2019 16:34	4.2	4	6.7912	3.9667	3.3936	1.8	0.0588	1.8116
4/15/2019 16:35	4.125	4.0167	6.3593	4.1167	3.3847	1.8	0.0752	1.8217
4/15/2019 16:36	4.1	3.8167	6.3369	3.9	3.3768	1.86	0.0698	1.8106
4/15/2019 16:37	4.0167	3.55	6.3998	4.1333	3.3693	1.9	0.0624	1.8122
4/15/2019 16:38	4	3.85	6.4064	3.9833	3.3843	1.9	0.0755	1.822
4/15/2019 16:39	4	3.4833	6.519	3.6333	3.386	1.9	0.0657	1.8324
4/15/2019 16:40	4	3.6333	6.5358	3.1	3.3878	1.9	0.0729	1.8187
4/15/2019 16:41	3.9667	3.6333	6.2526	3.2167	3.4213	1.9	0.0605	1.8039
4/15/2019 16:42	3.9	3.1833	5.8823	3.6	3.4116	1.925	0.0678	1.7938
4/15/2019 16:43	3.8333	3.3	6.0292	3.5333	3.4128	1.9	0.0666	1.8003
4/15/2019 16:44	3.8	3.25	6.0818	3.05	3.4131	1.925	0.0613	1.8314
4/15/2019 16:45	3.8	3.2	6.105	3.0667	3.4068	1.9	0.0658	1.8138
4/15/2019 16:46	3.7	3.5667	5.8628	3.1833	3.3878	1.9	0.0629	1.8048
4/15/2019 16:47	3.7	3.2	5.7671	3.4	3.3979	1.9	0.0595	1.8077
4/15/2019 16:48	3.625	3.5167	5.9128	3.2833	3.3863	1.9	0.0729	1.8008
4/15/2019 16:49	3.55	3.3167	5.9443	3.3167	3.3811	1.8667	0.0558	1.8191
4/15/2019 16:50	3.45	2.85	5.9333	3.3667	3.3797	1.9	0.0763	1.8036
4/15/2019 16:51	3.3667	3.0167	5.8127	3.0667	3.3669	1.9	0.0668	1.7899
4/15/2019 16:52	3.3	2.8167	5.5291	3.2	3.3573	1.92	0.0563	1.782
4/15/2019 16:53	3.2	3.45	5.5158	2.6667	3.3628	1.9417	0.0672	1.8043
4/15/2019 16:54	3.2	2.1167	5.5174	3.1	3.3585	1.9727	0.0605	1.8256
4/15/2019 16:55	3.2	2.8833	5.4346	3.1333	3.3319	2	0.0638	1.8026
4/15/2019 16:56	3.2	2.9	5.539	2.6167	3.3495	2	0.0612	1.7855
4/15/2019 16:57	3.15	2.8333	5.2222	2.65	3.3413	2	0.0564	1.8002
4/15/2019 16:58	3.1667	2.8667	5.5329	2.95	3.313	2.0091	0.0682	1.7887
4/15/2019 16:59	3.1	2.4667	5.3013	2.5833	3.3238	2.0833	0.0561	1.799
4/15/2019 17:00	3.1	2.85	5.246	2.8667	3.3251	2	0.0656	1.8246
4/15/2019 17:01	3.0333	2.4	5.5625	2.35	3.32	2	0.0568	1.8469
4/15/2019 17:02	3	2.4167	5.3264	2.3333	3.3186	1.9727	0.0559	1.8249
4/15/2019 17:03	2.9667	2.6	5.0629	2.5667	3.314	1.9	0.0682	1.8118
4/15/2019 17:04	2.9	2.8	5.1107	2.1167	3.3062	1.9	0.0508	1.7738
4/15/2019 17:05	2.9	1.9167	5.4289	2.45	3.2938	1.9	0.0707	1.8249
4/15/2019 17:06	2.9	2.2833	5.138	2.15	3.2898	1.9	0.0587	1.8177
4/15/2019 17:07	2.9	2.2167	5.1247	2.35	3.2773	1.875	0.054	1.8033
4/15/2019 17:08	2.8	2.1	5.3834	1.95	3.2834	1.9	0.064	1.8012
4/15/2019 17:09	2.8083	2.4667	5.2406	2.65	3.2601	1.9	0.0513	1.7858
4/15/2019 17:10	2.8	2.7	5.2331	2.7833	3.2569	1.9	0.0622	1.7946
4/15/2019 17:11	2.8	3.0333	5.1618	2.3333	3.2578	1.9	0.0598	1.7905

4/15/2019 17:12	2.7333	1.7333	5.1132	2	3.2462	1.9333	0.0557	1.7958
4/15/2019 17:13	2.6417	2.1667	4.9497	2.55	3.2405	2	0.0668	1.8252
4/15/2019 17:14	2.5417	2.6	5.2926	2.05	3.2396	2	0.046	1.8234
4/15/2019 17:15	2.4333	1.8667	5.435	1.7333	3.223	2.0091	0.0617	1.7805
4/15/2019 17:16	2.3333	2.2333	5.0705	2.45	3.2235	2.1	0.0564	1.7864
4/15/2019 17:17	2.25	2.1833	5.0759	2.75	3.2147	2.1	0.0543	1.7988
4/15/2019 17:18	2.2	2.3667	4.8093	2.5833	3.2158	2.1	0.0633	1.8069
4/15/2019 17:19	2.2	1.8833	4.7108	2.1333	3.2015	2.1	0.0474	1.8007
4/15/2019 17:20	2.225	1.4333	5.1422	2	3.2021	2.1	0.0615	1.7904
4/15/2019 17:21	2.2	2.4	5.0803	2.3333	3.1862	2.1	0.0557	1.8104
4/15/2019 17:22	2.125	2.1667	4.8012	2.2333	3.1691	2.1	0.0571	1.7811
4/15/2019 17:23	2.1	2.3167	4.9781	1.8333	3.1686	2.1	0.0635	1.8158
4/15/2019 17:24	2.15	1.5	4.8256	1.8667	3.1471	2.1	0.0499	1.8422
4/15/2019 17:25	2.2	2.1167	4.9266	2.1333	3.1521	2.1	0.0551	1.8647
4/15/2019 17:26	2.2	1.1167	5.0728	1.8	3.1317	2.1	0.0544	1.9433
4/15/2019 17:27	2.2	1.7333	4.8637	1.8667	3.1283	2.1	0.0515	2.0994
4/15/2019 17:28	2.2	1.9167	5.0188	2.1667	3.1175	2.1	0.0637	2.2187
4/15/2019 17:29	2.2	2.0333	4.8043	2.0167	3.1175	2.1273	0.0489	2.1525
4/15/2019 17:30	2.1583	1.65	4.8527	2.5667	3.0976	2.1417	0.0578	2.1254
4/15/2019 17:31	2.175	2.0333	4.4528	1.4667	3.0953	2.2	0.0596	2.1337
4/15/2019 17:32	2.2	2.3667	4.6895	1.35	3.0986	2.2	0.0516	2.1576
4/15/2019 17:33	2.2917	1.4667	4.5447	1.5333	3.0908	2.2	0.0602	2.1398
4/15/2019 17:34	2.2667	1.4833	4.5517	1.7833	3.0773	2.2	0.0445	2.1159
4/15/2019 17:35	2.2	2.1167	4.7035	1.45	3.0753	2.2	0.0593	2.1089
4/15/2019 17:36	2.1167	1.7833	4.445	2.0667	3.0773	2.2	0.0629	2.1371
4/15/2019 17:37	2.1	1.8	4.693	1.5833	3.0743	2.2	0.0467	2.1255
4/15/2019 17:38	2.1	2.2167	4.8493	1.55	3.0646	2.2	0.0645	2.1484
4/15/2019 17:39	2.1	1.9667	4.7605	1.5833	3.0598	2.2	0.0524	2.14
4/15/2019 17:40	2.1	1.5667	4.6643	2.15	3.054	2.2	0.0582	2.1062
4/15/2019 17:41	2.1	1.75	4.6163	2.2167	3.0536	2.2	0.0579	2.1088
4/15/2019 17:42	2.0417	1.85	4.4388	1.5167	3.0503	2.2	0.054	2.1351
4/15/2019 17:43	2	1.7833	4.9888	1.8333	3.0439	2.1091	0.0714	2.0843
4/15/2019 17:44	2	1.9167	4.6095	1.8667	3.0527	2.1	0.0521	2.0635
4/15/2019 17:45	2	1.7	4.8103	2.0333	3.0328	2.1	0.0648	2.0966
4/15/2019 17:46	2.0333	1.4667	4.7138	2.2	3.0273	2.1	0.0619	2.104
4/15/2019 17:47	2.1	1.9167	4.7957	2.1167	3.0184	2.1	0.0496	2.1154
4/15/2019 17:48	2.1583	1.8833	4.5083	1.6333	3.0024	2.1	0.062	2.1294
4/15/2019 17:49	2.125	1.5333	4.6323	1.6333	3.029	2.1	0.0515	2.1249
4/15/2019 17:50	2.175	1.1833	4.6608	1.5167	3.0104	2.1	0.0613	2.1112
4/15/2019 17:51	2.1	1.3167	4.4365	1.5833	3.0117	2.15	0.0606	2.0814
4/15/2019 17:52	2.1083	1.4667	4.4052	1.7667	2.9923	2.1545	0.0528	2.0766
4/15/2019 17:53	2.1	2.0667	4.6628	1.45	2.9977	2.2	0.0705	2.13
4/15/2019 17:54	2.1	1.9333	4.611	1.2	2.9763	2.2	0.0505	2.1229
4/15/2019 17:55	2.1	2.0167	5.0258	1.4167	2.9807	2.2	0.0605	2.092
4/15/2019 17:56	2.0417	0.9833	4.6655	1.15	2.9789	2.2	0.0631	2.0758
4/15/2019 17:57	2.0917	1.5333	4.7268	1.9667	2.9643	2.2545	0.046	2.0804
4/15/2019 17:58	2.1	2.2	4.5967	1.5833	2.9708	2.2636	0.0573	2.0682

4/15/2019 17:59	2.2	1.9833	4.6328	1.3833	2.9398	2.3	0.0484	2.0521
4/15/2019 18:00	2.2667	2.2167	4.7272	0.8833	2.9353	2.2636	0.064	2.0653
4/15/2019 18:01	2.2	1.5833	4.452	1.2167	2.9466	2.2	0.0583	2.0636
4/15/2019 18:02	2.2	1.75	4.7643	1.35	2.934	2.2	0.0561	2.0784
4/15/2019 18:03	2.2	1.9	4.6622	1.5167	2.9345	2.2	0.0654	2.0843
4/15/2019 18:04	2.2	1.8667	4.5222	2.1333	2.9058	2.2	0.0544	2.0794
4/15/2019 18:05	2.2	1.5833	4.4232	1.8167	2.9266	2.2	0.0643	2.0939
4/15/2019 18:06	2.175	0.9167	4.3553	1.7333	2.8983	2.2	0.05	2.0723
4/15/2019 18:07	2.1	1.9167	4.4283	1.2667	2.8857	2.1083	0.0614	2.0853
4/15/2019 18:08	2.1	1.65	4.4982	1.7167	2.8825	2.1	0.063	2.073
4/15/2019 18:09	2.0917	1.9167	4.2442	1.6	2.8893	2.1	0.0555	2.0374
4/15/2019 18:10	2.0917	1.9	4.1407	2.15	2.8786	2.1	0.0688	2.0116
4/15/2019 18:11	2.0167	1.65	4.5105	1.15	2.8696	2.1	0.0559	2.0461
4/15/2019 18:12	2.1	1.2	4.4335	1.4667	2.8583	2.1	0.0665	2.0451
4/15/2019 18:13	2.1	2.0667	4.363	1.7167	2.8508	2.1	0.061	2.0736
4/15/2019 18:14	2.1833	2.2333	4.4467	2.0167	2.8568	2.1	0.0573	2.0861
4/15/2019 18:15	2.1667	2	4.6505	1.65	2.8439	2.1	0.0736	2.0397
4/15/2019 18:16	2.1	1.4333	4.5897	1.5833	2.833	2.1	0.0544	2.0058
4/15/2019 18:17	2.05	1.7333	4.2832	1.3833	2.8372	2.1	0.071	2.034
4/15/2019 18:18	2	1.85	4.5132	1.8667	2.8258	2.1	0.0503	2.0411
4/15/2019 18:19	1.975	1.5	4.3362	1.2167	2.8167	2.1	0.0586	2.0191
4/15/2019 18:20	1.9	1.8833	4.2465	1.4667	2.8037	2.1	0.0729	2.0005
4/15/2019 18:21	1.975	1.1833	4.1935	1.6833	2.8015	2.1	0.0487	2.0263
4/15/2019 18:22	2	1.8333	4.4462	1.5833	2.7946	2.1	0.069	2.0327
4/15/2019 18:23	2.0083	1.0333	4.4348	1.3667	2.8131	2.1	0.0546	2.0268
4/15/2019 18:24	2	2.15	4.1142	1.2833	2.7987	2.175	0.0591	2.0098
4/15/2019 18:25	1.9833	1.7167	4.4142	1.1833	2.7947	2.2	0.0634	2.0044
4/15/2019 18:26	1.9167	1.6667	4.533	1.2833	2.7776	2.1545	0.0562	2.0016
4/15/2019 18:27	1.9	1.05	4.104	1.5	2.7594	2.1	0.0655	2.0355
4/15/2019 18:28	1.9	1.3167	4.1792	1.2833	2.7685	2.1	0.0563	2.0365
4/15/2019 18:29	1.8	1.95	4.3055	1.7167	2.7605	2.1	0.0671	2.0285
4/15/2019 18:30	1.8	1.8333	4.1245	1.2	2.7507	2.19	0.0611	2.0068
4/15/2019 18:31	1.8	1.3667	4.0908	0.8333	2.7519	2.2	0.0535	1.9878
4/15/2019 18:32	1.8	2.2333	4.4433	1.95	2.7432	2.2	0.0689	1.9927
4/15/2019 18:33	1.7833	1.1667	4.4152	1.5333	2.7622	2.2	0.057	2.0243
4/15/2019 18:34	1.8	1.3833	4.3288	1.8	2.7553	2.2	0.055	2.0225
4/15/2019 18:35	1.8	0.9167	4.1397	1.0167	2.7521	2.12	0.0637	2.0185
4/15/2019 18:36	1.8	1.45	4.4808	1.7167	2.7338	2.1	0.0574	2.0275
4/15/2019 18:37	1.775	1.7	4.1582	1.3333	2.7468	2.1	0.0612	2.0308
4/15/2019 18:38	1.7417	1.7167	4.228	1	2.7413	2.1	0.0528	2.0608
4/15/2019 18:39	1.7167	0.9167	4.4	0.9833	2.7133	2.1	0.0632	2.0163
4/15/2019 18:40	1.7833	1.75	4.3532	1.15	2.7265	2.1	0.051	2.0089
4/15/2019 18:41	1.775	1.6167	4.495	1.3333	2.727	2.1	0.0594	2.0124
4/15/2019 18:42	1.7	1.4167	4.2258	1.0833	2.7268	2.1	0.0567	2.0129
4/15/2019 18:43	1.7	1.4167	4.1427	1.2667	2.7199	2.1	0.0506	2.0305
4/15/2019 18:44	1.7417	1.8	4.4768	1.05	2.7188	2.1	0.0658	2.0013
4/15/2019 18:45	1.8	1.75	4.3522	1.1167	2.7059	2.1	0.0498	2.0177

4/15/2019 18:46	1.8	1.7	4.2512	0.7333	2.6946	2.1	0.0615	2.0183
4/15/2019 18:47	1.8	1.8167	4.2257	0.8333	2.7039	2.1	0.0553	1.989
4/15/2019 18:48	1.8	1.3	4.2202	1.25	2.6983	2.1	0.0471	2.0131
4/15/2019 18:49	1.8	1.65	4.2172	1.2	2.7054	2.1	0.0641	1.9913
4/15/2019 18:50	1.8	0.8833	4.3012	0.9333	2.6705	2.1	0.048	1.9663
4/15/2019 18:51	1.8	1.5333	4.174	1.5667	2.6634	2.1	0.0668	1.9932
4/15/2019 18:52	1.8	0.6	4.0643	1.0667	2.6846	2.1	0.0559	2.0051
4/15/2019 18:53	1.8	1.4833	4.3077	1.5	2.6816	2.1	0.0524	2.0004
4/15/2019 18:54	1.7833	1.95	4.298	1.1833	2.6808	2.1	0.0622	1.986
4/15/2019 18:55	1.7167	1.3	4.2007	0.7667	2.6758	2.1	0.0521	1.9606
4/15/2019 18:56	1.6667	1.0833	4.1962	1.2667	2.6563	2.1	0.0609	1.958
4/15/2019 18:57	1.6583	1.35	4.0551	1.2833	2.6494	2.1	0.052	1.994
4/15/2019 18:58	1.6667	1.4	4.1437	1	2.6489	2.1	0.0555	1.9949
4/15/2019 18:59	1.7083	1.3167	4.1033	0.8667	2.6598	2.1	0.0624	1.9772
4/15/2019 19:00	1.7083	1.65	4.3653	1.3	2.6631	2.1	0.0506	1.9482
4/15/2019 19:01	1.7	0.9167	4.2173	0.7	2.6518	2.1	0.0633	1.9664
4/15/2019 19:02	1.7	1.4667	4.3866	1.65	2.6617	2.1	0.0569	1.9678
4/15/2019 19:03	1.7	0.6167	4.2973	1.2833	2.6737	2.1	0.0531	1.9454
4/15/2019 19:04	1.7	1.1	4.5401	1.2167	2.6598	2.2	0.0662	1.9504
4/15/2019 19:05	1.65	1.5	4.0398	1.1333	2.6612	2.2	0.0456	1.9749
4/15/2019 19:06	1.6	0.8333	4.2774	1.2667	2.6628	2.2	0.058	1.9779
4/15/2019 19:07	1.5917	1.5167	4.2076	1.0333	2.6505	2.2	0.0487	2.003
4/15/2019 19:08	1.5917	1.8667	4.3013	0.95	2.6665	2.2	0.0596	1.9646
4/15/2019 19:09	1.6	1	4.1228	0.9167	2.6472	2.1091	0.0497	1.9478
4/15/2019 19:10	1.6	1.2	4.196	0.5667	2.6572	2.1	0.0478	1.9503
4/15/2019 19:11	1.6667	1.25	4.0429	1.25	2.6683	2.1091	0.0612	1.97
4/15/2019 19:12	1.7	0.8667	3.975	1.05	2.6606	2.2	0.0465	1.9665
4/15/2019 19:13	1.6833	1.45	4.015	0.6833	2.6627	2.2	0.0632	1.9746
4/15/2019 19:14	1.7	0.95	4.2723	0.7333	2.6665	2.2	0.0435	1.9635
4/15/2019 19:15	1.7	0.5833	4.0487	0.8	2.6564	2.2	0.0538	1.9533
4/15/2019 19:16	1.7	1.0167	4.2402	1.5	2.6682	2.2	0.0565	1.9697
4/15/2019 19:17	1.7	1.4167	4.2731	1.55	2.6701	2.2	0.0494	1.9629
4/15/2019 19:18	1.6917	1.1833	4.1659	0.9	2.6598	2.2	0.0629	1.9818
4/15/2019 19:19	1.6	1.3	4.167	1.3833	2.6625	2.2	0.0472	1.9638
4/15/2019 19:20	1.6	0.7167	4.0957	1.7	2.6757	2.2	0.0541	1.9522
4/15/2019 19:21	1.6	1.3	4.2513	1.3833	2.6794	2.2	0.0524	1.9495
4/15/2019 19:22	1.5667	0.95	4.1129	0.7167	2.6748	2.2	0.0501	1.9482
4/15/2019 19:23	1.6	0.8667	3.8917	1.5	2.6779	2.2	0.0566	1.9451
4/15/2019 19:24	1.6	1.7333	4.1798	1.1167	2.682	2.2	0.0393	1.9714
4/15/2019 19:25	1.6	1.2333	4.0787	1.2	2.6758	2.2182	0.0569	1.9657
4/15/2019 19:26	1.6	1.05	3.9958	1.2667	2.6852	2.3	0.0529	1.9628
4/15/2019 19:27	1.6	1.3667	4.0374	1.05	2.6859	2.3	0.054	1.9653
4/15/2019 19:28	1.6	0.9333	4.0701	1.2	2.6718	2.3	0.0589	1.953
4/15/2019 19:29	1.6	0.6	3.9435	0.8167	2.6747	2.3	0.0497	1.9293
4/15/2019 19:30	1.5583	1.6667	4.048	1.5667	2.6858	2.3	0.0585	1.9215
4/15/2019 19:31	1.5667	0.75	3.9836	1.4167	2.7019	2.3	0.0459	1.9025
4/15/2019 19:32	1.525	0.8833	4.1097	0.9833	2.7062	2.3	0.0473	1.9159

4/15/2019 19:33	1.5	1.4333	3.9748	1.2167	2.723	2.3	0.0596	1.8953
4/15/2019 19:34	1.525	0.9667	4.0629	1.1167	2.6996	2.3636	0.04	1.9263
4/15/2019 19:35	1.6	2.0333	3.9617	1.3333	2.7004	2.4	0.0535	1.9698
4/15/2019 19:36	1.6	1.6167	3.7237	0.8833	2.6975	2.4	0.0379	1.9496
4/15/2019 19:37	1.5833	0.8833	3.8032	1.5	2.7135	2.4	0.0545	1.9123
4/15/2019 19:38	1.5833	1.2667	4.0514	1.1667	2.7088	2.4	0.0497	1.935
4/15/2019 19:39	1.5833	1.4667	4.06	1.3	2.7119	2.4	0.0453	1.953
4/15/2019 19:40	1.5833	1.7333	4.1441	0.9833	2.7286	2.4	0.0558	1.9533
4/15/2019 19:41	1.575	1.1167	3.9272	1.1667	2.7278	2.4	0.0427	1.9697
4/15/2019 19:42	1.6	0.6333	4.016	0.85	2.733	2.4	0.058	1.97
4/15/2019 19:43	1.6	0.9667	4.0707	1.2333	2.733	2.4	0.0471	1.9276
4/15/2019 19:44	1.6167	1.2667	3.8028	1.1	2.7597	2.4364	0.0426	1.9199
4/15/2019 19:45	1.6	0.85	4.0076	0.7	2.7576	2.5	0.0564	1.9463
4/15/2019 19:46	1.5167	1.1	3.6605	0.55	2.7758	2.5	0.0407	1.9578
4/15/2019 19:47	1.5	1.1167	3.7673	1.2333	2.8259	2.5	0.0571	1.9334
4/15/2019 19:48	1.55	1.4	4.1007	0.8833	2.8569	2.5167	0.0488	1.9143
4/15/2019 19:49	1.575	1.4333	3.9178	1.0667	2.8732	2.6	0.0461	1.9372
4/15/2019 19:50	1.5583	0.9	4.1235	1.2167	2.8458	2.6	0.0572	1.9541
4/15/2019 19:51	1.4917	1.4833	3.6522	0.7833	2.8443	2.6	0.0372	1.9293
4/15/2019 19:52	1.45	1.1833	3.9226	0.25	2.8783	2.6	0.0551	1.9255
4/15/2019 19:53	1.45	0.8667	4.0595	0.95	2.8976	2.6	0.0546	1.9176
4/15/2019 19:54	1.5	1.1167	3.6854	0.4833	2.9231	2.6	0.0521	1.9323
4/15/2019 19:55	1.5	0.9333	3.9176	1.3833	2.9443	2.6	0.0559	1.9119
4/15/2019 19:56	1.5	0.7333	3.8985	0.7167	2.9728	2.6	0.0397	1.8886
4/15/2019 19:57	1.5	1.2167	3.7529	0.75	2.9433	2.6	0.058	1.9409
4/15/2019 19:58	1.4	0.9333	3.8243	0.5667	2.9687	2.5545	0.0475	1.9436
4/15/2019 19:59	1.35	1	3.9729	0.55	2.9682	2.5	0.0489	1.9473
4/15/2019 20:00	1.3583	1.35	3.8898	0.6667	2.9991	2.5	0.0556	1.9363
4/15/2019 20:01	1.4	1.15	3.9452	0.7667	3.0283	2.5	0.0456	1.9188
4/15/2019 20:02	1.4	0.9833	3.9553	0.3167	2.9958	2.5	0.0541	1.9318
4/15/2019 20:03	1.4	0.7833	3.8698	1.2667	3.0195	2.5	0.0515	1.9364
4/15/2019 20:04	1.4	1.15	3.6223	1.15	3.0228	2.5	0.0532	1.953
4/15/2019 20:05	1.4	0.9	3.8026	1.0167	3.0398	2.5	0.0557	1.9363
4/15/2019 20:06	1.425	1.4167	3.7783	0.8	3.0554	2.5	0.0502	1.8797
4/15/2019 20:07	1.5	0.5	4.1355	0.9	3.0434	2.4167	0.0587	1.8943
4/15/2019 20:08	1.4167	0.2833	3.9175	0.9167	3.078	2.4	0.0489	1.9025
4/15/2019 20:09	1.4	0.65	3.6178	0.3833	3.0602	2.475	0.0514	1.9298
4/15/2019 20:10	1.45	1.1167	3.8652	0.4167	3.0471	2.5	0.0532	1.9248
4/15/2019 20:11	1.4333	1.6333	3.7303	0.8167	3.0762	2.4818	0.0454	1.911
4/15/2019 20:12	1.4	0.4167	3.5684	0.8	3.0903	2.4	0.0624	1.9172
4/15/2019 20:13	1.4583	0.6333	3.8597	1.1167	3.111	2.4	0.0477	1.9064
4/15/2019 20:14	1.4417	0.7833	3.7187	0.7167	3.1194	2.4	0.0567	1.8968
4/15/2019 20:15	1.4	0.65	3.5737	1.0667	3.1231	2.4	0.0501	1.9149
4/15/2019 20:16	1.4	1.3167	3.9113	0.5333	3.1659	2.4	0.0507	1.8728
4/15/2019 20:17	1.4167	1.1667	3.7252	0.8	3.1554	2.4	0.0659	1.8918
4/15/2019 20:18	1.4	0.55	3.8481	0.8	3.127	2.4	0.0438	1.9323
4/15/2019 20:19	1.4	0.5667	3.7674	0.8833	3.1572	2.4	0.0556	1.9418

4/15/2019 20:20	1.4	1.0333	3.837	1.05	3.1858	2.4	0.0532	1.9185
4/15/2019 20:21	1.4	1.05	3.5554	0.2833	3.1745	2.4	0.0564	1.9031
4/15/2019 20:22	1.4	0.0667	3.7104	0.8	3.1621	2.4	0.0563	1.8978
4/15/2019 20:23	1.3917	0.8667	3.8223	1	3.1667	2.4	0.0507	1.8922
4/15/2019 20:24	1.4	0.55	3.6812	0.4	3.1912	2.35	0.0647	1.9059
4/15/2019 20:25	1.375	0.8	3.7769	0.35	3.1848	2.3	0.0515	1.9078
4/15/2019 20:26	1.3	1.2833	4.0121	0.3833	3.1869	2.3	0.064	1.9028
4/15/2019 20:27	1.25	0.85	3.7405	0.5333	3.2092	2.3	0.0566	1.9038
4/15/2019 20:28	1.2	0.7667	3.7257	0.2	3.1838	2.3	0.0515	1.9061
4/15/2019 20:29	1.1917	1.0333	3.6623	1.05	3.2123	2.3	0.0639	1.9147
4/15/2019 20:30	1.1333	1.0333	3.529	0.9167	3.2049	2.3	0.0514	1.8979
4/15/2019 20:31	1.1583	0.75	3.6698	0.75	3.228	2.3273	0.0597	1.8737
4/15/2019 20:32	1.2	0.9167	3.7905	0.8833	3.2273	2.3091	0.0606	1.8883
4/15/2019 20:33	1.2917	0.65	3.74	0.7	3.2293	2.3	0.0528	1.902
4/15/2019 20:34	1.375	0.3667	3.9647	1.4667	3.2351	2.3	0.0623	1.8979
4/15/2019 20:35	1.4	0.9333	3.7387	1.35	3.2385	2.3	0.0493	1.922
4/15/2019 20:36	1.375	1.3667	3.7182	0.9833	3.2368	2.3	0.0661	1.8972
4/15/2019 20:37	1.2818	0.9667	3.7493	0.8167	3.2156	2.3909	0.05	1.8507
4/15/2019 20:38	1.3	1.15	3.9213	1.1833	3.2407	2.3273	0.0577	1.8803
4/15/2019 20:39	1.3	0.9333	3.8722	1.4833	3.1924	2.3273	0.061	1.897
4/15/2019 20:40	1.3	1.1667	3.7215	0.8667	3.2196	2.3	0.0484	1.9022
4/15/2019 20:41	1.2417	1.1167	3.8382	0.7167	3.2149	2.3	0.0661	1.9083
4/15/2019 20:42	1.2167	1.2833	3.6212	1.25	3.2033	2.3333	0.0546	1.9173
4/15/2019 20:43	1.3	0.65	3.7658	0.7333	3.217	2.3833	0.0632	1.9221
4/15/2019 20:44	1.3	0.5667	3.7127	1.1833	3.217	2.3455	0.0564	1.9113
4/15/2019 20:45	1.3917	0.4667	3.862	0.6333	3.2361	2.4	0.0603	1.8878
4/15/2019 20:46	1.4	1.0667	3.7578	0.3667	3.2207	2.4	0.0593	1.8833
4/15/2019 20:47	1.4	0.65	3.7388	0.6333	3.2248	2.4	0.0531	1.8948
4/15/2019 20:48	1.4	1.3167	3.8927	1.2833	3.2351	2.4	0.0598	1.8953
4/15/2019 20:49	1.3083	0.5333	3.8913	0.8	3.2303	2.4	0.0585	1.9019
4/15/2019 20:50	1.3	1.1333	3.87	1.2333	3.2518	2.4	0.0603	1.8809
4/15/2019 20:51	1.3	1.1333	3.775	0.85	3.2574	2.4	0.065	1.8376
4/15/2019 20:52	1.3	1.1833	3.9087	0.4333	3.2257	2.4	0.0494	1.8788
4/15/2019 20:53	1.3	0.7167	3.4875	1.15	3.2116	2.4	0.0617	1.8856
4/15/2019 20:54	1.275	0.5667	3.8023	0.65	3.2421	2.4	0.047	1.8658
4/15/2019 20:55	1.275	1.2833	3.8795	1.1167	3.26	2.4	0.0713	1.8619
4/15/2019 20:56	1.3	0.75	3.9077	0.7167	3.2438	2.4	0.0618	1.8505
4/15/2019 20:57	1.2417	0.85	3.741	0.9667	3.2496	2.4	0.055	1.8437
4/15/2019 20:58	1.2	0.35	3.7653	1.3333	3.2562	2.4	0.0637	1.8518
4/15/2019 20:59	1.2	0.3667	3.8115	1.3833	3.2515	2.4	0.042	1.893
4/15/2019 21:00	1.2	1.25	3.805	0.65	3.2412	2.4	0.0667	1.8797
4/15/2019 21:01	1.275	0.7833	3.5125	0.75	3.2838	2.4	0.055	1.8709
4/15/2019 21:02	1.275	1.6333	3.6185	1.05	3.2488	2.4	0.0537	1.8681
4/15/2019 21:03	1.3	0.55	3.6293	0.6333	3.2585	2.4	0.067	1.889
4/15/2019 21:04	1.3	1.0167	3.6685	1.0333	3.2838	2.4	0.0484	1.8873
4/15/2019 21:05	1.3	0	3.7695	1.1	3.2966	2.3667	0.0619	1.8793
4/15/2019 21:06	1.35	0.7	3.7383	0.65	3.2781	2.3	0.0577	1.8873

4/15/2019 21:07	1.4	0.7333	3.8252	0.5833	3.2845	2.3	0.0529	1.8783
4/15/2019 21:08	1.4	0.3667	3.9182	0.6167	3.2768	2.325	0.0572	1.8713
4/15/2019 21:09	1.4	0.9833	4.1175	1.1	3.262	2.3	0.0515	1.8485
4/15/2019 21:10	1.3583	1.1833	3.7127	1.1167	3.2927	2.3	0.0637	1.8657
4/15/2019 21:11	1.45	1.25	3.8852	0.5667	3.3184	2.3	0.0464	1.8601
4/15/2019 21:12	1.475	0.2	4.1402	1.1	3.3067	2.3	0.0587	1.8598
4/15/2019 21:13	1.4417	0.7833	3.8332	0.9167	3.2901	2.3	0.0545	1.842
4/15/2019 21:14	1.4333	0.8	3.7242	0.7	3.2998	2.3	0.0542	1.8486
4/15/2019 21:15	1.4167	0.7833	3.5872	0.55	3.3093	2.3	0.0597	1.8516
4/15/2019 21:16	1.4917	1.3667	4.0905	0.6	3.3313	2.3	0.0467	1.8626
4/15/2019 21:17	1.475	0.8833	3.848	0.9	3.3177	2.3	0.0677	1.858
4/15/2019 21:18	1.4364	0.5667	3.757	1.1333	3.3024	2.4	0.053	1.8868
4/15/2019 21:19	1.4583	1.3333	3.8733	1.3	3.2956	2.3417	0.0551	1.8782
4/15/2019 21:20	1.4	1.15	3.9622	0.7833	3.3007	2.3	0.0678	1.851
4/15/2019 21:21	1.4333	1.1	4.1648	1.05	3.3038	2.3	0.0512	1.827
4/15/2019 21:22	1.4	0.9833	3.8887	0.9333	3.3023	2.3545	0.0716	1.8228
4/15/2019 21:23	1.4	0.4	3.88	0.5	3.2719	2.3	0.0571	1.8423
4/15/2019 21:24	1.375	0.3667	3.7477	0.8333	3.313	2.3	0.0493	1.8438
4/15/2019 21:25	1.3083	1.25	3.6908	1.3	3.3218	2.3	0.0718	1.8523
4/15/2019 21:26	1.3083	1.2667	4.0055	0.7	3.3167	2.3	0.0569	1.8676
4/15/2019 21:27	1.3	1.3167	3.7037	1.2667	3.319	2.325	0.0621	1.8186
4/15/2019 21:28	1.3	1.2667	3.7112	1.2167	3.2965	2.3	0.0484	1.8658
4/15/2019 21:29	1.3167	0.95	3.6548	1.25	3.3298	2.3	0.055	1.8501
4/15/2019 21:30	1.3333	1.0667	3.8528	1.1833	3.3178	2.3	0.0657	1.8377
4/15/2019 21:31	1.375	1.1333	3.8312	0.5333	3.3285	2.3	0.0489	1.8694
4/15/2019 21:32	1.3083	1.05	3.7155	0.9	3.3176	2.4	0.0592	1.854
4/15/2019 21:33	1.3417	0.75	3.587	0.9	3.3313	2.4	0.0472	1.8641
4/15/2019 21:34	1.3333	1	3.4617	0.8333	3.3268	2.4	0.0454	1.8767
4/15/2019 21:35	1.35	1.1	3.426	1.0167	3.3363	2.4	0.0486	1.855
4/15/2019 21:36	1.3	0.55	3.3205	0.5333	3.3263	2.4	0.0472	1.8372
4/15/2019 21:37	1.3	1.45	3.5423	1.1833	3.3432	2.5	0.0648	1.8133
4/15/2019 21:38	1.325	0.6333	3.732	1.0167	3.3447	2.5	0.0438	1.8369
4/15/2019 21:39	1.3833	0.8167	3.5555	0.25	3.3331	2.5909	0.0622	1.858
4/15/2019 21:40	1.4	0.9833	3.8877	0.4833	3.3711	2.6	0.059	1.8554
4/15/2019 21:41	1.3583	0.8833	3.8187	0.7	3.3662	2.6	0.0438	1.8569
4/15/2019 21:42	1.3	1.0667	3.6877	0.9	3.3734	2.6167	0.0597	1.8663
4/15/2019 21:43	1.3	0.7333	3.7192	0.5167	3.3782	2.7	0.0454	1.8723
4/15/2019 21:44	1.3083	0.8833	3.5232	0.9333	3.3935	2.7	0.059	1.8536
4/15/2019 21:45	1.3	0.5	3.4675	0.6167	3.393	2.6833	0.0624	1.8688
4/15/2019 21:46	1.3	0.6833	3.6328	0.7	3.3932	2.6	0.0531	1.8473
4/15/2019 21:47	1.2667	1.3333	3.33	1.0833	3.4008	2.6	0.0598	1.8533
4/15/2019 21:48	1.2417	1.15	3.5802	0.8	3.4104	2.6	0.0439	1.8542
4/15/2019 21:49	1.2	0.6667	3.5995	0.9	3.4155	2.6	0.0633	1.8682
4/15/2019 21:50	1.2	1.1	3.525	1.2	3.4056	2.6	0.0555	1.8744
4/15/2019 21:51	1.2	1.25	3.5458	1.1167	3.4476	2.5667	0.0528	1.8693
4/15/2019 21:52	1.2	0.8833	3.6993	1.8333	3.4712	2.5	0.057	1.843
4/15/2019 21:53	1.2	0.8833	3.7423	1.1333	3.4709	2.5	0.0468	1.8398

4/15/2019 21:54	1.1833	1.1167	3.495	0.7333	3.4993	2.5	0.0663	1.8463
4/15/2019 21:55	1.1583	0.3	3.613	0.85	3.5148	2.5	0.054	1.8657
4/15/2019 21:56	1.1	0.4833	3.6544	0.85	3.4951	2.475	0.0542	1.8336
4/15/2019 21:57	1.1833	0.6833	3.6674	0.5	3.4803	2.5	0.0651	1.8212
4/15/2019 21:58	1.1917	1.2333	3.7743	0.3833	3.4956	2.4273	0.0519	1.8266
4/15/2019 21:59	1.2	0.8333	3.7847	1.0167	3.5564	2.4	0.0611	1.8338
4/15/2019 22:00	1.1583	1.35	3.6085	0.85	3.5059	2.4	0.0494	1.8447
4/15/2019 22:01	1.1	1.45	3.4512	0.7667	3.5427	2.4	0.0591	1.8293
4/15/2019 22:02	1.1	1.15	3.4224	0.85	3.5798	2.3636	0.0652	1.8525
4/15/2019 22:03	1.1833	1.3333	3.6902	0.8667	3.602	2.3182	0.0491	1.8119
4/15/2019 22:04	1.1333	0.55	3.5033	0.05	3.5398	2.4	0.0607	1.8424
4/15/2019 22:05	1.1333	0.4667	3.3622	0.45	3.601	2.4	0.0494	1.844
4/15/2019 22:06	1.1	0.2333	3.4717	0.4333	3.6671	2.4	0.0578	1.7921
4/15/2019 22:07	1.1333	0.8833	3.7524	0.15	3.6228	2.4	0.0585	1.7713
4/15/2019 22:08	1.2	0.2	3.2961	0.8167	3.687	2.4	0.0484	1.8053
4/15/2019 22:09	1.2333	0.7167	3.5055	0.6333	3.6724	2.3417	0.0662	1.7933
4/15/2019 22:10	1.275	0.3667	3.2868	1.1	3.7175	2.4	0.0514	1.8328
4/15/2019 22:11	1.2167	0.3167	3.502	0.3833	3.7189	2.3455	0.0567	1.8419
4/15/2019 22:12	1.2	1.2333	3.7076	0.45	3.6956	2.3	0.0462	1.8235
4/15/2019 22:13	1.175	1.3333	3.469	0.3	3.744	2.3	0.0551	1.8452
4/15/2019 22:14	1.1	1.4333	3.2723	0.6833	3.7721	2.3	0.0591	1.8354
4/15/2019 22:15	1.1	0.9167	3.2283	0.6167	3.862	2.3	0.0537	1.8253
4/15/2019 22:16	1.1	-0.0167	3.0718	0.1667	3.8414	2.3	0.0626	1.8264
4/15/2019 22:17	1.0667	0.7	3.6771	0.8667	4.0088	2.3	0.0449	1.8053
4/15/2019 22:18	1	0.5	3.3843	1.0333	4.0802	2.3	0.0582	1.7996
4/15/2019 22:19	1	0.8167	3.1744	0.6	4.0439	2.3	0.0547	1.8074
4/15/2019 22:20	0.9917	0.9333	3.297	0.15	4.0946	2.3	0.0561	1.8203
4/15/2019 22:21	0.9167	1.5333	3.4873	0.3333	4.1346	2.3	0.0669	1.8138
4/15/2019 22:22	0.8833	1.1	3.0844	0.5	4.219	2.3	0.0449	1.7891
4/15/2019 22:23	0.8083	0.5667	3.489	0.3833	4.2909	2.375	0.0588	1.8179
4/15/2019 22:24	0.8	0.6333	3.1655	0.05	4.2413	2.4	0.0534	1.8553
4/15/2019 22:25	0.8	0.75	3.0825	0.2	4.3485	2.4417	0.0553	1.8526
4/15/2019 22:26	0.8	0.3833	3.1273	0.0667	4.3292	2.43	0.0586	1.826
4/15/2019 22:27	0.8	0.25	2.8233	0.1833	4.5756	2.5	0.0477	1.8384
4/15/2019 22:28	0.8	0.1833	2.854	0.4333	4.5183	2.5	0.0731	1.8419
4/15/2019 22:29	0.8	0.35	3.1085	0.2667	4.5236	2.5	0.0439	1.8402
4/15/2019 22:30	0.8083	0.4833	3.0912	-0.2833	4.4519	2.575	0.049	1.8394
4/15/2019 22:31	0.8833	0.55	3.0686	0.45	4.7615	2.6	0.06	1.8358
4/15/2019 22:32	0.9	0.9167	3.2017	0.5	4.6808	2.6	0.0457	1.838
4/15/2019 22:33	0.875	0.5667	3.0012	0.1667	4.8631	2.6	0.0664	1.8503
4/15/2019 22:34	0.8333	0.7167	3.1218	0.2	4.8557	2.6	0.0552	1.8465
4/15/2019 22:35	0.8	0.6333	3.2296	0.7333	4.6585	2.6	0.0502	1.8605
4/15/2019 22:36	0.8	0.3167	2.8378	-0.1833	4.9491	2.6	0.0539	1.8279
4/15/2019 22:37	0.8	0.55	3.2048	0	5.0112	2.6	0.0445	1.8093
4/15/2019 22:38	0.8	0.55	3.1429	0.4	4.9562	2.6	0.0616	1.7949
4/15/2019 22:39	0.8	0.35	3.1312	0.4833	4.8551	2.6	0.0523	1.8308
4/15/2019 22:40	0.8	0.7333	3.275	0.3667	5.1525	2.6	0.064	1.8343

4/15/2019 22:41	0.825	0.1667	3.0104	-0.0333	5.1495	2.6	0.0496	1.8501
4/15/2019 22:42	0.825	-0.0833	3.0348	0.0833	4.9253	2.6	0.0566	1.856
4/15/2019 22:43	0.8	0.5333	3.2623	0.7333	5.219	2.6	0.0635	1.8557
4/15/2019 22:44	0.7167	-0.1	2.9744	0.3	5.2769	2.6	0.0474	1.8185
4/15/2019 22:45	0.7	0.6	3.075	0.1	5.3966	2.6	0.054	1.8223
4/15/2019 22:46	0.7	0.3667	2.8302	0.3	5.387	2.6	0.0571	1.8499
4/15/2019 22:47	0.7	0.6333	3.0459	0.4167	5.3761	2.6833	0.0554	1.8563
4/15/2019 22:48	0.7	0.2333	3.1289	0.6667	5.4388	2.7	0.0591	1.8402
4/15/2019 22:49	0.6083	-0.0333	3.0878	0.35	5.45	2.7	0.0488	1.837
4/15/2019 22:50	0.6	1.2	3.1557	0.2167	5.391	2.7	0.0596	1.833
4/15/2019 22:51	0.6	0.0167	2.9027	-0.0333	5.5798	2.6818	0.0495	1.8205
4/15/2019 22:52	0.6	0.2667	2.9695	-0.0833	5.5397	2.6	0.0552	1.8243
4/15/2019 22:53	0.6	0.4833	3.0553	0.15	5.5725	2.6	0.0608	1.825
4/15/2019 22:54	0.6	-0.2	2.9555	-0.2667	5.6044	2.6	0.0478	1.8198
4/15/2019 22:55	0.5917	0	2.9896	0.2	5.6144	2.6	0.0612	1.8117
4/15/2019 22:56	0.6	0.3	2.719	0.5333	5.5546	2.6273	0.0463	1.8101
4/15/2019 22:57	0.6	0.2667	2.5893	-0.3667	5.5626	2.6667	0.0589	1.8103
4/15/2019 22:58	0.5917	1.0667	2.853	0.05	5.5883	2.6	0.0622	1.8133
4/15/2019 22:59	0.5667	0.3667	3.0041	0.15	5.5951	2.6	0.0466	1.8281
4/15/2019 23:00	0.6	-0.2167	2.9613	0.1833	5.7236	2.5727	0.0675	1.7954
4/15/2019 23:01	0.6	0	2.6092	0.55	5.6783	2.6	0.0496	1.8278
4/15/2019 23:02	0.6	0.0667	2.8605	0.5833	5.7392	2.5091	0.0605	1.8238
4/15/2019 23:03	0.6	0.5667	2.6548	0.5333	5.6484	2.55	0.0528	1.8267
4/15/2019 23:04	0.7	0.3	3.0847	0.0667	5.6658	2.6	0.0521	1.8301
4/15/2019 23:05	0.7	-0.45	2.7888	0.3833	5.7267	2.6	0.0633	1.8119
4/15/2019 23:06	0.7	-0.0333	2.633	0.25	5.7234	2.6	0.0487	1.8045
4/15/2019 23:07	0.7	0.1833	2.913	-0.15	5.7309	2.6667	0.0609	1.7878
4/15/2019 23:08	0.6667	0.7833	2.926	0.5333	5.7663	2.6182	0.0535	1.7883
4/15/2019 23:09	0.625	0.1333	2.7746	0.6	5.7148	2.7	0.0562	1.829
4/15/2019 23:10	0.6167	0.5333	2.8708	0.2	5.7108	2.7	0.0606	1.8432
4/15/2019 23:11	0.6	-0.3833	2.927	0.4667	5.7693	2.7	0.053	1.8518
4/15/2019 23:12	0.7	0.0667	2.8668	0.2833	5.7569	2.7	0.0582	1.8666
4/15/2019 23:13	0.7	1.05	2.7987	0.3167	5.776	2.7	0.0532	1.8617
4/15/2019 23:14	0.7	0.35	2.6253	-0.1333	5.8388	2.66	0.0592	1.8258
4/15/2019 23:15	0.75	1.1833	2.8957	-0.2167	5.7406	2.7	0.0533	1.8259
4/15/2019 23:16	0.8	0.5833	2.6846	-0.1333	5.7689	2.7	0.0502	1.8256
4/15/2019 23:17	0.8	0.6	2.9263	-0.0833	5.8067	2.7	0.064	1.8168
4/15/2019 23:18	0.8	-0.1	2.6469	-0.05	5.7744	2.7	0.0517	1.8027
4/15/2019 23:19	0.8	0.5167	2.5998	0.35	5.8638	2.7	0.0642	1.8183
4/15/2019 23:20	0.8	0.7333	2.7678	0.15	5.8741	2.7	0.0551	1.8267
4/15/2019 23:21	0.8	0.6667	2.8625	-0.3	5.8324	2.7	0.0575	1.8126
4/15/2019 23:22	0.8	0.3833	2.9622	0.4	5.7869	2.775	0.0615	1.8208
4/15/2019 23:23	0.7167	-0.25	3.0114	0.8667	5.7729	2.8	0.0538	1.8206
4/15/2019 23:24	0.7	-0.6	2.6375	0.1333	5.8358	2.8	0.0643	1.8153
4/15/2019 23:25	0.6583	0.0167	2.9239	0.2	5.7988	2.8	0.0627	1.8343
4/15/2019 23:26	0.7	0.55	3.0028	0.0167	5.7843	2.82	0.0555	1.8063
4/15/2019 23:27	0.7	0.7667	2.8242	-0.1833	5.7869	2.8636	0.065	1.8085

4/15/2019 23:28	0.7	1.1333	2.7718	-0.2	5.7787	2.8	0.0495	1.8348
4/15/2019 23:29	0.6583	0.4333	2.9832	-0.05	5.773	2.8	0.0746	1.8329
4/15/2019 23:30	0.6	0.4167	2.78	0.4667	5.7543	2.8	0.0503	1.8105
4/15/2019 23:31	0.5833	0.4333	2.6642	0.1	5.7053	2.8	0.0551	1.8066
4/15/2019 23:32	0.5667	1.05	2.9568	0.0667	5.7361	2.8	0.0605	1.8083
4/15/2019 23:33	0.6	0.1167	3.0645	0.5667	5.7152	2.8	0.0499	1.8355
4/15/2019 23:34	0.6917	0.4833	3.0287	0.6833	5.7042	2.7182	0.0628	1.8418
4/15/2019 23:35	0.7	0.1833	3.1183	-0.1333	5.6665	2.7636	0.0581	1.8183
4/15/2019 23:36	0.7	0.3667	3.038	0.1833	5.6658	2.8	0.0608	1.8171
4/15/2019 23:37	0.7	0.2	3.0733	0.3167	5.641	2.8	0.0575	1.829
4/15/2019 23:38	0.7	0.3333	2.8088	0.15	5.6239	2.8	0.0496	1.8305
4/15/2019 23:39	0.7333	0.35	3.0542	0.0333	5.5983	2.8	0.0626	1.8182
4/15/2019 23:40	0.8	0.35	3.0642	0.2167	5.5554	2.8	0.0549	1.8308
4/15/2019 23:41	0.9	0.45	2.8422	0.3167	5.5572	2.8	0.0577	1.8202
4/15/2019 23:42	0.9	0.8833	2.9028	-0.0333	5.5385	2.8	0.0566	1.7999
4/15/2019 23:43	0.85	0.5167	2.8632	0.1	5.5175	2.8	0.0503	1.7928
4/15/2019 23:44	0.8	0.4	2.9853	0.3167	5.4848	2.8	0.0602	1.803
4/15/2019 23:45	0.8	0.9167	3.0587	0.5333	5.4763	2.825	0.0495	1.8185
4/15/2019 23:46	0.8	0.0833	3.332	0.3	5.457	2.87	0.0617	1.8156
4/15/2019 23:47	0.8167	0.05	2.9463	0.1	5.451	2.8091	0.0572	1.814
4/15/2019 23:48	0.8	0.3667	2.955	-0.0167	5.443	2.87	0.0526	1.8287
4/15/2019 23:49	0.8167	0.35	3.2003	0.4167	5.3827	2.8417	0.0607	1.8275
4/15/2019 23:50	0.8917	0.5	3.027	0.6667	5.3553	2.8	0.0473	1.8272
4/15/2019 23:51	0.925	0.2167	3.0532	1.1667	5.3423	2.8	0.0596	1.8108
4/15/2019 23:52	1	1.1333	2.966	0.2333	5.3233	2.8	0.0546	1.8384
4/15/2019 23:53	1	0.2333	3.1993	0.4667	5.3268	2.8	0.0573	1.8339
4/15/2019 23:54	0.9917	-0.0333	3.257	0.5333	5.2969	2.8	0.0605	1.8015
4/15/2019 23:55	0.9667	0.65	3.1003	0.9167	5.2705	2.8	0.0491	1.8179
4/15/2019 23:56	0.975	0.8	3.0905	0.2667	5.2304	2.8	0.0615	1.8348
4/15/2019 23:57	1	0.7333	2.9657	0.8333	5.1958	2.8	0.0562	1.8543
4/15/2019 23:58	0.9833	0.3667	3.2072	0.9667	5.2013	2.8	0.0507	1.8304
4/15/2019 23:59	0.975	0.1333	3.2892	0.55	5.1848	2.7727	0.0558	1.8113
4/16/2019 0:00	0.9917	0.3833	3.3832	0.6833	5.1533	2.7	0.0404	1.7923
4/16/2019 0:01	0.9417	-0.2667	3.2573	0.9333	5.1418	2.7	0.056	1.8121
4/16/2019 0:02	0.95	0.6667	3.2965	0.8667	5.1212	2.7	0.0478	1.8394
4/16/2019 0:03	1	0.55	3.2018	0.8333	5.0877	2.67	0.0468	1.8103
4/16/2019 0:04	1.0417	0.9667	3.1807	1.25	5.0874	2.6	0.054	1.8375
4/16/2019 0:05	1.0833	0.7333	3.108	0.2667	5.0857	2.6	0.0473	1.8177
4/16/2019 0:06	1.0833	1.3	3.2313	0.8333	5.0389	2.5091	0.0627	1.7813
4/16/2019 0:07	1.1	0.5167	3.283	0.4167	5.0215	2.5	0.0516	1.7997
4/16/2019 0:08	1.1833	1.25	3.2245	0.7	5.0134	2.5	0.0561	1.8276
4/16/2019 0:09	1.2	0.85	3.1603	0	5.0039	2.5	0.0551	1.8273
4/16/2019 0:10	1.2	0.5167	3.353	1.1333	4.9827	2.5	0.044	1.8017
4/16/2019 0:11	1.175	0.35	3.2675	0.4	4.9658	2.4545	0.0598	1.7993
4/16/2019 0:12	1.1333	0.2667	3.2842	1	4.9393	2.4	0.0509	1.7936
4/16/2019 0:13	1.1083	0.8167	3.2897	0.05	4.9326	2.4333	0.0499	1.7933
4/16/2019 0:14	1.1	0.5833	3.1998	1.0667	4.8943	2.48	0.0539	1.822

4/16/2019 0:15	1.05	0.2833	3.215	0.25	4.9224	2.4	0.0435	1.7894
4/16/2019 0:16	1	0.15	3.3263	0.45	4.883	2.4	0.0572	1.8102
4/16/2019 0:17	1	0.45	3.4703	1.1333	4.8783	2.4	0.043	1.8396
4/16/2019 0:18	1.05	0.35	3.3918	0.65	4.8411	2.4	0.0423	1.841
4/16/2019 0:19	1.1	0.9667	3.1667	1.05	4.8243	2.45	0.0533	1.8221
4/16/2019 0:20	1.1	1.15	3.0432	1.55	4.8295	2.5	0.0461	1.8114
4/16/2019 0:21	1.0417	0.6833	3.185	0.9333	4.8259	2.5	0.0535	1.7916
4/16/2019 0:22	1	0.5667	3.4425	1.1167	4.8215	2.5	0.0399	1.7896
4/16/2019 0:23	1	1.0333	3.3033	0.4667	4.8053	2.5	0.0565	1.799
4/16/2019 0:24	1	0.1333	3.199	1.2667	4.8126	2.5	0.0482	1.8049
4/16/2019 0:25	1	0.1333	3.3697	0.3	4.7928	2.5636	0.0465	1.7979
4/16/2019 0:26	1	0.2167	3.212	0.55	4.7888	2.5167	0.0591	1.7897
4/16/2019 0:27	1	0.2	2.9078	0.9333	4.7927	2.6	0.0377	1.7712
4/16/2019 0:28	0.9917	1.2333	3.2983	0.4833	4.7697	2.6	0.0536	1.787
4/16/2019 0:29	0.9	1.35	2.9845	0.5833	4.7643	2.6	0.0448	1.7911
4/16/2019 0:30	0.9	1.0667	3.3657	0.4833	4.7738	2.6	0.0515	1.7913
4/16/2019 0:31	0.9	0.3667	3	0.8833	4.7577	2.6	0.0588	1.8011
4/16/2019 0:32	0.9	0.9833	3.2412	0.5833	4.7563	2.6	0.0384	1.8206
4/16/2019 0:33	0.9	0.4167	3.204	0.5833	4.7512	2.6	0.0463	1.8087
4/16/2019 0:34	0.9417	0.55	3.1913	0.2167	4.7685	2.6	0.0446	1.805
4/16/2019 0:35	0.95	0.8833	3.0863	0.6	4.7454	2.6	0.0461	1.8068
4/16/2019 0:36	0.95	0.95	3.163	0.5667	4.7053	2.6	0.0478	1.8159
4/16/2019 0:37	1	0.3833	3.2698	0.5333	4.7165	2.6833	0.0403	1.8003
4/16/2019 0:38	1	0.8167	3.0297	1.0167	4.7075	2.675	0.0542	1.8172
4/16/2019 0:39	1	0.8333	3.2722	0.7	4.6937	2.7	0.0386	1.7987
4/16/2019 0:40	0.9917	0.4333	3.1872	0.6	4.6718	2.6583	0.05	1.8047
4/16/2019 0:41	0.9833	0.5333	3.1223	0.6333	4.67	2.6	0.0419	1.8205
4/16/2019 0:42	0.9333	0.5333	3.4277	0.9167	4.6535	2.6	0.047	1.8027
4/16/2019 0:43	1	0.4833	3.2355	0.7833	4.6339	2.6	0.0512	1.8011
4/16/2019 0:44	0.9917	0.9667	3.1652	0.6667	4.6473	2.6636	0.0342	1.8128
4/16/2019 0:45	1.075	0.6	3.3635	0	4.6261	2.7	0.046	1.8186
4/16/2019 0:46	1.0333	0.8167	2.9415	0.8833	4.6393	2.7	0.0551	1.8087
4/16/2019 0:47	1.1	0.7667	3.148	0.5667	4.6145	2.7	0.0475	1.8273
4/16/2019 0:48	1	0.4167	2.8368	1.2	4.615	2.7	0.0466	1.8095
4/16/2019 0:49	1	0.6167	3.0153	0.3333	4.6149	2.7	0.0342	1.8198
4/16/2019 0:50	1	1.1833	3.0083	0.1	4.6181	2.7	0.0436	1.7963
4/16/2019 0:51	1.0417	0.3167	2.926	0.25	4.5909	2.7	0.0413	1.7878
4/16/2019 0:52	1.025	0.4167	2.9715	0.2167	4.5742	2.7	0.039	1.8045
4/16/2019 0:53	1.0917	0.8167	2.9867	0.55	4.5806	2.7	0.0494	1.8194
4/16/2019 0:54	1.1	0.3667	3.1222	0.1667	4.5598	2.7917	0.039	1.8188
4/16/2019 0:55	1.1	1.0667	3.2513	0.4667	4.5797	2.78	0.0453	1.8077
4/16/2019 0:56	1.1	0.4167	2.9267	0.45	4.553	2.7	0.0454	1.8082
4/16/2019 0:57	1.0083	0.5	3.2368	0.6667	4.5323	2.7	0.0335	1.8086
4/16/2019 0:58	0.9833	0.6333	3.0719	0.1667	4.5514	2.7	0.0499	1.8058
4/16/2019 0:59	0.9583	0.8	2.961	0.0833	4.5754	2.7	0.0355	1.7918
4/16/2019 1:00	0.9917	0.0333	3.0626	0.25	4.5631	2.6364	0.043	1.7978
4/16/2019 1:01	1	0.4667	3.1796	0.45	4.5756	2.6	0.0451	1.8018

4/16/2019 1:02	1	0.5167	2.8879	-0.1833	4.5721	2.6	0.031	1.8035
4/16/2019 1:03	1	0.8	2.775	0.2833	4.5592	2.6	0.0466	1.8214
4/16/2019 1:04	0.9917	1.4	2.9606	0.6167	4.5578	2.5	0.0371	1.8153
4/16/2019 1:05	0.9083	0.8167	2.8331	0.2	4.555	2.5	0.0442	1.8145
4/16/2019 1:06	0.9	0.7333	2.8711	0.4167	4.5243	2.5	0.0422	1.8158
4/16/2019 1:07	0.8583	0.3333	2.9613	-0.1	4.5466	2.5	0.0379	1.8212
4/16/2019 1:08	0.8909	0.3833	2.8458	1.0333	4.545	2.5	0.0457	1.8269
4/16/2019 1:09	0.9333	0.2	2.8508	0.4167	4.5672	2.5	0.0343	1.8187
4/16/2019 1:10	0.975	0.3333	3.2348	0.3667	4.5442	2.4917	0.0374	1.8352
4/16/2019 1:11	0.9083	0.7167	3.0234	0.4833	4.5048	2.4727	0.0408	1.7944
4/16/2019 1:12	0.9917	0.5333	3.288	-0.05	4.5615	2.5	0.0377	1.8101
4/16/2019 1:13	1	0.0667	3.0424	0.6167	4.5522	2.5	0.0444	1.8103
4/16/2019 1:14	1	0.5167	2.7791	0.3167	4.5491	2.5	0.0325	1.834
4/16/2019 1:15	1	0.8167	3.0948	0.6833	4.5268	2.5727	0.0426	1.8287
4/16/2019 1:16	1	1.15	3.1926	-0.0833	4.538	2.6	0.0392	1.8321
4/16/2019 1:17	1	0.2333	3.1357	0.5833	4.5378	2.6	0.0381	1.8393
4/16/2019 1:18	1	0.7	3.0711	0.3333	4.5443	2.6	0.0475	1.8099
4/16/2019 1:19	1.0167	0.4667	3.2355	0.45	4.5288	2.6	0.0351	1.8165
4/16/2019 1:20	1.1	0.25	3.0302	0.6667	4.5213	2.6	0.041	1.8027
4/16/2019 1:21	1.1	-0.0167	3.1637	0.35	4.5411	2.6	0.0352	1.8184
4/16/2019 1:22	1.1	1.0333	3.0683	0.75	4.5252	2.6	0.0392	1.8136
4/16/2019 1:23	1.1	1.3833	3.071	0.6167	4.5263	2.6	0.0417	1.8278
4/16/2019 1:24	1.1083	0.7	3.0428	0.25	4.5294	2.6	0.031	1.8561
4/16/2019 1:25	1.1	0.4667	2.9499	0.2167	4.5311	2.6	0.0428	1.8515
4/16/2019 1:26	1.1417	0.9	2.9068	0.8	4.5494	2.6	0.0378	1.8238
4/16/2019 1:27	1.1	0.5333	3.0297	0.5167	4.4994	2.6	0.0382	1.7915
4/16/2019 1:28	1.1167	0.25	3.0293	1.1333	4.5127	2.5727	0.0489	1.8073
4/16/2019 1:29	1.1	0.5333	3.0253	1.1	4.5284	2.5	0.0265	1.8091
4/16/2019 1:30	1.1	0.9667	2.9225	0.3	4.514	2.5	0.0418	1.8133
4/16/2019 1:31	1.1	1.1333	2.9525	0.9	4.5094	2.5	0.0322	1.7978
4/16/2019 1:32	1.1	1.2	3.1124	0.5667	4.4993	2.5	0.033	1.8101
4/16/2019 1:33	1.1667	0.4833	2.9602	0.55	4.4919	2.5	0.0386	1.8258
4/16/2019 1:34	1.2	1.3667	3.0115	0.95	4.4696	2.46	0.0294	1.8136
4/16/2019 1:35	1.2	1.2	2.9843	1.2	4.4814	2.4	0.0427	1.8103
4/16/2019 1:36	1.2167	0.1167	3.0014	0.75	4.4758	2.4333	0.0306	1.8241
4/16/2019 1:37	1.2	0.7	3.0488	1.1667	4.4778	2.5	0.0359	1.8441
4/16/2019 1:38	1.1833	0.8667	2.8748	0.45	4.4753	2.48	0.0409	1.843
4/16/2019 1:39	1.1	-0.05	2.7282	0.9167	4.4298	2.4	0.0291	1.8192
4/16/2019 1:40	1.1167	0.4167	3.0622	0.7667	4.4408	2.4636	0.0372	1.8373
4/16/2019 1:41	1.2	0.75	2.8863	0.9667	4.4429	2.5	0.03	1.8539
4/16/2019 1:42	1.2083	0.9667	3.3547	0.5833	4.4472	2.5	0.0367	1.8448
4/16/2019 1:43	1.2083	0.3667	3.1673	0.5167	4.4363	2.5	0.0367	1.8007
4/16/2019 1:44	1.2	0.1333	3.1456	0.1833	4.4013	2.5	0.0292	1.8231
4/16/2019 1:45	1.1083	0.5333	3.0998	1.05	4.3777	2.5	0.0412	1.8546
4/16/2019 1:46	1.1	1.3167	3.0362	0.3833	4.3795	2.5	0.0336	1.8782
4/16/2019 1:47	1.1	0.4	3.0358	0.3667	4.3738	2.54	0.0379	1.8619
4/16/2019 1:48	1.1	0.65	3.0457	0.4833	4.3848	2.5	0.0427	1.8583

4/16/2019 1:49	1.1	0.6167	3.0273	0.9	4.385	2.5364	0.0288	1.8617
4/16/2019 1:50	1.1083	0.75	3.1723	0.8	4.3833	2.6	0.0385	1.8433
4/16/2019 1:51	1.1	-0.0333	3.1938	0.65	4.349	2.6	0.0283	1.8768
4/16/2019 1:52	1.0167	1.2167	2.7056	0.3333	4.3208	2.6	0.039	1.8479
4/16/2019 1:53	1	1.0833	2.8935	0.4	4.3354	2.5667	0.0334	1.8508
4/16/2019 1:54	0.9583	0.7667	3.0028	0.4	4.3032	2.5	0.0283	1.8613
4/16/2019 1:55	1	0.9833	3.1986	0.8333	4.2932	2.54	0.036	1.8489
4/16/2019 1:56	0.9	1.2833	2.9781	0.7	4.2768	2.6	0.0301	1.8523
4/16/2019 1:57	0.9833	0.8667	2.9703	0.1667	4.2922	2.6	0.0379	1.8292
4/16/2019 1:58	1	0.9667	3.0375	0.6333	4.268	2.6	0.0376	1.8384
4/16/2019 1:59	1	0.45	3.3103	0.9333	4.2668	2.6	0.0277	1.8526
4/16/2019 2:00	1	0.8833	3.0058	0.7333	4.2526	2.6364	0.0418	1.8604
4/16/2019 2:01	1	0.3167	3.4072	0.85	4.2582	2.6833	0.0273	1.8703
4/16/2019 2:02	0.9333	0.6	3.1813	0.4167	4.2355	2.6	0.0429	1.8758
4/16/2019 2:03	0.9	0.8833	3.1244	0.5333	4.2304	2.6167	0.0344	1.8774
4/16/2019 2:04	0.9	1.2333	2.8722	0.7333	4.235	2.7	0.0226	1.8735
4/16/2019 2:05	0.9083	0.8833	3.0826	0.3333	4.2091	2.7	0.0398	1.8618
4/16/2019 2:06	0.9083	0.4333	3.0233	0.1167	4.2025	2.7	0.027	1.8568
4/16/2019 2:07	0.9	0.7833	3.0798	0.7833	4.1875	2.7	0.0247	1.8539
4/16/2019 2:08	0.8917	-0.0167	2.9378	0.2667	4.1528	2.7	0.0357	1.8843
4/16/2019 2:09	0.8917	0.85	3.0194	0.4	4.1575	2.7	0.02	1.8597
4/16/2019 2:10	0.9	0.2	3.0407	0.6333	4.1236	2.7	0.0335	1.8386
4/16/2019 2:11	0.9	0.4667	3.1853	0.1333	4.1472	2.7	0.0332	1.8699
4/16/2019 2:12	0.9083	1.2833	2.8648	0.6333	4.1153	2.6917	0.0353	1.9074
4/16/2019 2:13	0.9	1.0333	2.9484	0.3	4.0978	2.6	0.0336	1.9048
4/16/2019 2:14	0.9	0.6	2.9356	0.2833	4.1108	2.6	0.0254	1.8879
4/16/2019 2:15	0.9	0.7333	2.9634	0.35	4.0862	2.6	0.0422	1.8749
4/16/2019 2:16	0.9	0.55	2.9499	0.6	4.0689	2.6	0.0358	1.8674
4/16/2019 2:17	0.9	0.3	2.9181	0.3167	4.0673	2.6	0.041	1.8916
4/16/2019 2:18	0.8	1.1167	2.9193	0.6167	4.0391	2.6	0.0333	1.887
4/16/2019 2:19	0.7667	1.2	3.0833	0.3833	4.0449	2.5083	0.0275	1.8786
4/16/2019 2:20	0.7917	1.1167	3.1042	0.7167	4.0188	2.5	0.0357	1.8964
4/16/2019 2:21	0.8	1.3333	3.0603	0.4667	4.0027	2.5	0.0263	1.8843
4/16/2019 2:22	0.8417	0.1833	2.5918	0.8167	3.9844	2.5	0.0361	1.8739
4/16/2019 2:23	0.9	0.2667	2.8808	1.2333	3.9791	2.5	0.0306	1.8852
4/16/2019 2:24	0.9	0.7667	2.9263	0.5	3.9535	2.4091	0.0273	1.8916
4/16/2019 2:25	0.9	0.2	2.8845	0.8333	3.9448	2.4	0.038	1.8757
4/16/2019 2:26	0.9	0.7	3.1528	0.6	3.9157	2.31	0.0272	1.8588
4/16/2019 2:27	0.9	1.1167	3.0355	0.4667	3.9053	2.3	0.0339	1.8399
4/16/2019 2:28	0.9	0.6167	2.794	0.8833	3.9208	2.3	0.0321	1.8451
4/16/2019 2:29	0.9	1.05	2.7908	0.6667	3.9023	2.3	0.0248	1.8397
4/16/2019 2:30	0.9	0.5333	2.9692	0.2	3.8991	2.3	0.0331	1.8553
4/16/2019 2:31	0.9	1.3	2.9472	0.4667	3.8845	2.3	0.0243	1.8863
4/16/2019 2:32	0.9417	1.0667	3.0818	0.8167	3.8907	2.3	0.0292	1.8746
4/16/2019 2:33	1.0083	0.8833	2.997	0.6	3.8879	2.3	0.0293	1.8524
4/16/2019 2:34	1.05	0.5667	3.0132	0.85	3.8839	2.375	0.0277	1.8384
4/16/2019 2:35	1.0083	0.25	3.216	0.6833	3.9146	2.4	0.0392	1.8725

4/16/2019 2:36	1	0.2833	3.1867	0.8	3.9122	2.4	0.0271	1.8708
4/16/2019 2:37	1	0.4833	3.11	0.1667	3.9575	2.4	0.0315	1.8548
4/16/2019 2:38	1.0417	0.65	2.938	0.3667	3.976	2.4	0.0317	1.8404
4/16/2019 2:39	1.025	0.8333	2.916	1.3833	4.0036	2.4727	0.027	1.8327
4/16/2019 2:40	0.9417	0.4333	2.877	0.4	4.0408	2.5	0.0443	1.8497
4/16/2019 2:41	0.9	0.5167	2.8345	1.0833	4.1218	2.5	0.0253	1.8469
4/16/2019 2:42	0.9417	0.2833	3.061	0.1	4.1299	2.5	0.034	1.8478
4/16/2019 2:43	0.9667	1.2333	3.0678	0.2833	4.1057	2.5	0.0219	1.846
4/16/2019 2:44	0.9	-0.4	2.6218	0.2	4.1803	2.5	0.0325	1.8595
4/16/2019 2:45	0.9	0.9333	2.7612	0.3167	4.1367	2.5	0.0373	1.8392
4/16/2019 2:46	0.8417	0.35	2.747	0.55	4.1803	2.5	0.0208	1.8419
4/16/2019 2:47	0.8	0.7833	2.5462	0.8333	4.2373	2.4273	0.0411	1.8581
4/16/2019 2:48	0.8	0.35	2.8137	0.7167	4.2293	2.5	0.0251	1.8513
4/16/2019 2:49	0.8	-0.1333	2.7278	0.25	4.2591	2.5	0.038	1.8433
4/16/2019 2:50	0.8	0.1167	2.6138	-0.2333	4.279	2.5	0.0356	1.8193
4/16/2019 2:51	0.8	0.7667	2.6607	0.6667	4.2798	2.5	0.0284	1.8214
4/16/2019 2:52	0.7917	0.2333	2.9213	0.5167	4.2863	2.5	0.0333	1.8345
4/16/2019 2:53	0.7583	0.8	2.742	0.4833	4.3329	2.5	0.0291	1.8181
4/16/2019 2:54	0.725	0.4167	2.5403	0.7333	4.457	2.5	0.0327	1.8441
4/16/2019 2:55	0.8	0.1	2.7097	-0.0167	4.4986	2.5	0.0396	1.8504
4/16/2019 2:56	0.8	0.3	2.9187	-0.6167	4.4782	2.5	0.0278	1.8787
4/16/2019 2:57	0.8	-0.0833	2.5058	0.2	4.5311	2.5	0.0331	1.8653
4/16/2019 2:58	0.8	0.8333	2.4997	0.1167	4.6693	2.5	0.0245	1.8303
4/16/2019 2:59	0.7917	0.65	2.6555	-0.2333	4.6273	2.5	0.0349	1.8358
4/16/2019 3:00	0.7	-0.05	2.3912	-0.05	4.6356	2.4333	0.0373	1.8381
4/16/2019 3:01	0.7	-0.05	2.6613	0.0333	4.7878	2.4	0.0234	1.8498
4/16/2019 3:02	0.7	0.4667	2.5505	-0.0833	4.9213	2.4	0.0388	1.8603
4/16/2019 3:03	0.6917	0.3333	2.857	0.1667	4.9043	2.4	0.0291	1.8535
4/16/2019 3:04	0.6	0.5167	2.4398	-0.15	4.7772	2.4	0.0311	1.8779
4/16/2019 3:05	0.6	0.35	2.6632	-0.2667	4.8173	2.5	0.0403	1.8438
4/16/2019 3:06	0.6	0.3167	2.658	-0.1667	4.9377	2.475	0.0269	1.834
4/16/2019 3:07	0.5333	0.4	2.7147	0.1667	5.041	2.5	0.0376	1.8484
4/16/2019 3:08	0.5333	0.25	2.4793	0.0667	5.1849	2.5	0.0279	1.857
4/16/2019 3:09	0.6	0.1167	2.4395	0.1333	5.1837	2.5333	0.0341	1.8248
4/16/2019 3:10	0.5583	-0.0167	2.3713	0.6333	5.0649	2.6	0.0386	1.8201
4/16/2019 3:11	0.5	0.3667	2.6378	0.0333	4.9548	2.6	0.025	1.8478
4/16/2019 3:12	0.5	0.0333	2.7902	0.35	5.0703	2.6	0.0386	1.8444
4/16/2019 3:13	0.4083	-0.2167	2.1677	-0.1333	5.1679	2.6833	0.0271	1.836
4/16/2019 3:14	0.4167	0.25	2.422	0.0833	5.2074	2.7	0.0258	1.8408
4/16/2019 3:15	0.4667	0.3667	2.253	-0.2833	5.3067	2.7	0.0385	1.8351
4/16/2019 3:16	0.5	-0.1167	2.3655	-0.1333	5.1535	2.7	0.0221	1.8283
4/16/2019 3:17	0.5	0.15	2.227	0.1833	5.1602	2.7	0.0397	1.8312
4/16/2019 3:18	0.5	0.1667	2.3327	-0.2167	5.1503	2.7	0.0294	1.8428
4/16/2019 3:19	0.5	-0.1833	2.375	-0.6167	5.195	2.7	0.0287	1.8678
4/16/2019 3:20	0.5	-0.5333	2.4408	-0.2833	5.2182	2.7	0.0322	1.8766
4/16/2019 3:21	0.5	0.5	2.2142	-0.2	5.2568	2.7	0.0266	1.8952
4/16/2019 3:22	0.5	-0.4	2.3965	-0.6667	5.0822	2.7	0.0423	1.8982

4/16/2019 3:23	0.5	0.0667	2.5597	-0.1	5.1645	2.7	0.0268	1.863
4/16/2019 3:24	0.5	-0.0167	2.3597	-0.5667	5.3935	2.7	0.0357	1.8537
4/16/2019 3:25	0.5	-0.4833	2.2107	-0.1333	5.3254	2.73	0.0322	1.856
4/16/2019 3:26	0.5	-0.5	2.5973	-0.0167	5.3581	2.7818	0.0231	1.8321
4/16/2019 3:27	0.5	0.2333	2.3762	-0.6333	5.2943	2.7727	0.0369	1.8369
4/16/2019 3:28	0.5	0.2	2.3353	-0.3333	5.3774	2.8	0.0273	1.83
4/16/2019 3:29	0.4667	0.55	2.5252	0.1333	5.3385	2.8	0.0334	1.8638
4/16/2019 3:30	0.4	0.1667	2.4155	-0.35	5.3591	2.8	0.0397	1.8673
4/16/2019 3:31	0.425	-0.4167	2.1588	-0.4167	5.3408	2.8	0.0264	1.8525
4/16/2019 3:32	0.45	-0.1	2.0105	-0.45	5.3834	2.7364	0.04	1.888
4/16/2019 3:33	0.4833	0.0833	2.5327	0.3	5.4347	2.7667	0.0244	1.8677
4/16/2019 3:34	0.4667	-0.1	2.3323	-0.2	5.3724	2.7545	0.037	1.8613
4/16/2019 3:35	0.5	-0.6	2.958	0.2	5.402	2.72	0.0374	1.8526
4/16/2019 3:36	0.5333	-0.05	2.4252	-0.3667	5.3797	2.7	0.0291	1.8593
4/16/2019 3:37	0.5417	0.3167	2.6645	-0.3833	5.3703	2.7583	0.0354	1.828
4/16/2019 3:38	0.5091	0.05	2.4503	-0.45	5.2835	2.71	0.0309	1.8268
4/16/2019 3:39	0.5	-0.3333	2.4337	0	5.3165	2.8	0.0376	1.844
4/16/2019 3:40	0.5	0.4833	2.508	0.3833	5.3931	2.8	0.0337	1.8594
4/16/2019 3:41	0.5667	-0.1667	2.2962	0.3	5.3378	2.8	0.032	1.8828
4/16/2019 3:42	0.6	0.0333	2.6397	0.1667	5.2835	2.8	0.0449	1.8741
4/16/2019 3:43	0.6	0.3333	2.4835	0.15	5.2749	2.8	0.0264	1.9103
4/16/2019 3:44	0.6	0.3667	2.5432	0.5	5.2251	2.8	0.0379	1.8958
4/16/2019 3:45	0.625	0	2.5357	0.7	5.2928	2.8	0.0406	1.8676
4/16/2019 3:46	0.7	0.1333	2.179	-0.5	5.2473	2.8	0.0286	1.8656
4/16/2019 3:47	0.7	0.2	2.4578	0.2833	5.2122	2.8	0.0322	1.8418
4/16/2019 3:48	0.7	-0.1	2.6163	1.2	5.2039	2.8	0.0243	1.8657
4/16/2019 3:49	0.7	0.25	2.2355	0.2833	5.1817	2.8	0.0329	1.8782
4/16/2019 3:50	0.6083	0.3167	2.466	-0.2833	5.1597	2.8	0.0292	1.8839
4/16/2019 3:51	0.6	0.4833	2.384	0.35	5.181	2.72	0.0258	1.8633
4/16/2019 3:52	0.6	0.8	2.4288	-0.0833	5.181	2.7	0.0424	1.8748
4/16/2019 3:53	0.6	0.5167	2.5465	0.7	5.1483	2.7	0.0229	1.8768
4/16/2019 3:54	0.6	0.2333	2.603	-0.25	5.0978	2.6909	0.0308	1.8628
4/16/2019 3:55	0.5833	0.7667	2.7148	0.8	5.0696	2.6	0.0299	1.8741
4/16/2019 3:56	0.5	1.2833	2.6495	0.2833	5.0218	2.6	0.028	1.8721
4/16/2019 3:57	0.5	-0.2167	2.6593	0.3333	5.0059	2.5917	0.0393	1.8905
4/16/2019 3:58	0.5	-0.1667	2.7798	0.4833	5.0043	2.5	0.0236	1.8559
4/16/2019 3:59	0.5	0.3667	2.3757	-0.1667	5.0058	2.56	0.0333	1.8533
4/16/2019 4:00	0.5	0.6333	2.6461	0.2833	4.9639	2.5727	0.0346	1.8663
4/16/2019 4:01	0.5583	0.4667	2.6125	0.3333	4.9575	2.5545	0.025	1.8871
4/16/2019 4:02	0.5083	0.3667	2.5558	0.3833	4.9196	2.6	0.0371	1.8967
4/16/2019 4:03	0.5	0.3667	2.3565	0.0167	4.8814	2.6	0.022	1.8463
4/16/2019 4:04	0.4917	0.8667	2.5088	0.75	4.837	2.6	0.03	1.8867
4/16/2019 4:05	0.5	0.8333	2.6707	0.7333	4.8305	2.6	0.0333	1.8648
4/16/2019 4:06	0.5	0.95	2.7444	0.4333	4.8108	2.6	0.0237	1.8758
4/16/2019 4:07	0.5	-0.4667	2.3863	0.5833	4.793	2.6	0.0314	1.8691
4/16/2019 4:08	0.5	0.4833	2.8592	0.35	4.775	2.68	0.0239	1.9104
4/16/2019 4:09	0.5	0.6667	2.2726	0.25	4.7788	2.7	0.0265	1.8955

4/16/2019 4:10	0.5833	0.0167	2.4901	0.2667	4.75	2.7	0.0385	1.8505
4/16/2019 4:11	0.6	0.5333	2.3768	0.3167	4.7568	2.7	0.0246	1.8548
4/16/2019 4:12	0.6917	0.35	2.4849	-0.1	4.7174	2.7	0.0371	1.871
4/16/2019 4:13	0.8	0.3	2.6818	0.1833	4.6684	2.7364	0.0311	1.8893
4/16/2019 4:14	0.8667	0.3333	2.7974	0.5167	4.6168	2.7818	0.0272	1.8745
4/16/2019 4:15	0.9	0.6667	2.6761	0.6667	4.5677	2.7	0.0305	1.864
4/16/2019 4:16	0.8	0.4667	2.6963	0.2833	4.5948	2.7	0.0165	1.8753
4/16/2019 4:17	0.8	0.15	2.755	0.1667	4.5655	2.7	0.0342	1.8703
4/16/2019 4:18	0.8	0.0333	2.7753	0.0333	4.5148	2.7	0.0213	1.8777
4/16/2019 4:19	0.8	0.2333	2.707	0.25	4.5289	2.7	0.0275	1.8793
4/16/2019 4:20	0.8083	0.3333	2.7759	0.1333	4.5236	2.6636	0.0229	1.8691
4/16/2019 4:21	0.8917	1.0333	2.8702	0.0833	4.4724	2.6417	0.0225	1.8805
4/16/2019 4:22	0.9167	0.75	3.1765	0.7167	4.4463	2.7	0.034	1.8753
4/16/2019 4:23	0.9417	0.6333	2.9064	0.3	4.4283	2.7	0.0201	1.8793
4/16/2019 4:24	0.9	0.4333	2.9518	0.35	4.3893	2.7	0.0286	1.8627
4/16/2019 4:25	0.9167	0.5	2.799	0.6167	4.3817	2.7	0.0283	1.8703
4/16/2019 4:26	0.9	0.1333	2.6626	0.6333	4.3709	2.66	0.0163	1.8496
4/16/2019 4:27	0.9	0.6167	2.8013	0.4333	4.372	2.7	0.0325	1.8307
4/16/2019 4:28	0.9	0.3333	2.9223	0.8	4.3171	2.7	0.0269	1.8263
4/16/2019 4:29	0.9667	1.0333	2.9517	0.5833	4.2818	2.7	0.0306	1.8391
4/16/2019 4:30	1	0.3	2.5958	0.2167	4.3043	2.7	0.0268	1.902
4/16/2019 4:31	1	0	2.7449	0.6	4.293	2.7	0.0222	1.8903
4/16/2019 4:32	1	1.2333	2.9541	0.6333	4.2544	2.7	0.0314	1.8892
4/16/2019 4:33	1	0.8833	2.8605	0.5667	4.2166	2.7545	0.0205	1.8884
4/16/2019 4:34	1	-0.05	2.6534	0.25	4.1857	2.7182	0.0251	1.9059
4/16/2019 4:35	1.0917	0.2667	2.9434	0.2	4.1914	2.7	0.0283	1.8749
4/16/2019 4:36	1.0333	0.0333	2.6478	0.3833	4.2069	2.7778	0.0164	1.8687
4/16/2019 4:37	1	1.0333	2.6708	0.4333	4.1688	2.8	0.0339	1.8838
4/16/2019 4:38	0.925	0.85	2.7869	0.5	4.1529	2.8	0.0221	1.8673
4/16/2019 4:39	0.9917	0.9667	2.6978	0.1	4.1118	2.8	0.0288	1.8436
4/16/2019 4:40	0.9583	0.65	2.6703	-0.0167	4.1133	2.78	0.0216	1.8588
4/16/2019 4:41	0.9	0.1	2.4795	-0.0167	4.1148	2.8	0.025	1.8519
4/16/2019 4:42	0.9417	0.3167	2.7517	-0.3	4.0813	2.8	0.0326	1.8532
4/16/2019 4:43	1	0.5167	2.8153	0.1	4.078	2.8	0.0181	1.8843
4/16/2019 4:44	1	0.75	2.6625	0.4667	4.0698	2.8	0.0328	1.8945
4/16/2019 4:45	1	0.5167	2.7369	-0.0667	4.0626	2.8	0.0284	1.8972
4/16/2019 4:46	0.975	0.8333	2.7545	0.05	4.0679	2.8	0.0238	1.8644
4/16/2019 4:47	1	0.3333	2.9475	0.6833	4.0671	2.8	0.0317	1.8758
4/16/2019 4:48	1	0.2833	2.7091	0.15	4.0509	2.7182	0.0159	1.8634
4/16/2019 4:49	0.9917	0.8333	2.7912	0.7	4.0434	2.7	0.0271	1.8438
4/16/2019 4:50	0.9	0.0167	2.7627	0.3	4.0598	2.7	0.0267	1.8601
4/16/2019 4:51	0.9	0.4167	2.5773	-0.1167	4.0635	2.7	0.023	1.8899
4/16/2019 4:52	0.8583	0.6167	2.6876	0.7667	4.066	2.7	0.0299	1.8688
4/16/2019 4:53	0.9	0.4167	2.5753	0.4167	4.066	2.75	0.0211	1.8601
4/16/2019 4:54	0.9	0.6833	2.2093	0.4667	4.0671	2.8	0.0323	1.8853
4/16/2019 4:55	0.8583	0.4167	2.833	0.75	4.0514	2.8	0.0232	1.8728
4/16/2019 4:56	0.8	0.1833	2.7158	0.3167	4.0735	2.83	0.0225	1.886

4/16/2019 4:57	0.8	0.6333	2.5387	0.75	4.0603	2.9	0.0317	1.9008
4/16/2019 4:58	0.7583	0.3333	2.7653	0.4667	4.0611	2.9	0.0182	1.9048
4/16/2019 4:59	0.7	1.25	2.7003	0.9333	4.1192	2.9	0.0294	1.9134
4/16/2019 5:00	0.7	0.1	2.6816	0.2167	4.0998	2.9	0.0221	1.9042
4/16/2019 5:01	0.7	0.1333	2.7744	0.25	4.0994	2.9	0.023	1.8823
4/16/2019 5:02	0.7	0.7	2.6787	0.6333	4.1094	2.9	0.0241	1.8633
4/16/2019 5:03	0.7	0.2	2.6633	0.5333	4.1462	2.9	0.0168	1.886
4/16/2019 5:04	0.675	0.0333	2.4618	0.95	4.1303	2.9	0.0194	1.876
4/16/2019 5:05	0.6	0.2667	2.6931	0.4	4.1401	2.9	0.023	1.8475
4/16/2019 5:06	0.6	0.2333	2.4718	-0.05	4.1492	2.8636	0.0061	1.8673
4/16/2019 5:07	0.6417	0.4167	2.7154	0.9167	4.2118	2.8	0.0239	1.8669
4/16/2019 5:08	0.7	0.65	2.5723	0.0833	4.247	2.8	0.0125	1.8396
4/16/2019 5:09	0.65	0.1667	2.5081	0.4	4.229	2.8	0.0068	1.8471
4/16/2019 5:10	0.6	0.4	2.5536	0.3167	4.1976	2.8	0.0125	1.8793
4/16/2019 5:11	0.6	0.45	2.5763	0.4833	4.236	2.7833	0.0057	1.8348
4/16/2019 5:12	0.7	0.25	2.2355	0.5167	4.3059	2.7	0.0104	1.8404
4/16/2019 5:13	0.7	0.7833	2.5837	0.05	4.2338	2.7	0.013	1.8454
4/16/2019 5:14	0.7	0.7	2.4343	0.0833	4.2485	2.72	0.0008	1.8254
4/16/2019 5:15	0.6917	0.0667	2.6639	-0.3167	4.3025	2.8	0.0177	1.8259
4/16/2019 5:16	0.6917	0.1333	2.6307	0.5667	4.2776	2.8	-0.0039	1.8337
4/16/2019 5:17	0.6417	0.2833	2.6509	0.55	4.2698	2.7182	0.0096	1.8151
4/16/2019 5:18	0.625	0.4167	2.5966	0.1	4.3103	2.7	0.0049	1.794
4/16/2019 5:19	0.6	-0.0167	2.7354	0.3667	4.3167	2.7	-0.0014	1.8134
4/16/2019 5:20	0.6917	0.6833	2.6433	0.2333	4.4373	2.7	0.0115	1.8472
4/16/2019 5:21	0.6083	-0.5167	2.7218	0.0667	4.3839	2.7	0.0019	1.8408
4/16/2019 5:22	0.625	-0.45	2.4301	0.2	4.3102	2.7	-0.0003	1.822
4/16/2019 5:23	0.6	0.0333	2.1844	-0.1167	4.4684	2.7	0.008	1.7987
4/16/2019 5:24	0.6	-0.25	2.6567	-0.0333	4.4695	2.7	-0.0032	1.7887
4/16/2019 5:25	0.6	0.1333	2.5323	0.3167	4.3508	2.7	0.0019	1.8077
4/16/2019 5:26	0.6	-0.5	2.5443	0.45	4.3492	2.7	0.0063	1.8424
4/16/2019 5:27	0.5917	-0.2667	2.5098	0	4.4857	2.6182	-0.0059	1.8337
4/16/2019 5:28	0.6	0.5	2.8092	0.0167	4.5084	2.7	0.0033	1.805
4/16/2019 5:29	0.6	0.05	2.8288	0.1833	4.5403	2.7	-0.0051	1.8071
4/16/2019 5:30	0.6	0.4	2.811	0.5667	4.6338	2.7	0.0029	1.8048
4/16/2019 5:31	0.6	-0.4333	2.6698	-0.05	4.6959	2.7	0.0107	1.8101
4/16/2019 5:32	0.6	0.15	2.6145	0.4333	4.701	2.7	-0.0045	1.8053
4/16/2019 5:33	0.6	0.4667	2.3485	0.25	4.6316	2.7	0.0125	1.82
4/16/2019 5:34	0.6	0.3333	2.2063	0.0167	4.6708	2.7	0.0114	1.839
4/16/2019 5:35	0.5333	0.2333	2.3757	-0.1667	4.5724	2.72	0.0046	1.8543
4/16/2019 5:36	0.5083	-0.1	2.4258	-0.1	4.6723	2.8	0.0059	1.8307
4/16/2019 5:37	0.6	0.3	2.4023	-0.35	4.709	2.8	0.0101	1.8235
4/16/2019 5:38	0.6	0.2167	2.4598	-0.2167	4.6396	2.8	0	1.8463
4/16/2019 5:39	0.6	0.5333	2.825	-0.05	4.7778	2.8	0.0155	1.8454
4/16/2019 5:40	0.6167	0.15	2.9223	0.5	4.8163	2.8	0.0046	1.8348
4/16/2019 5:41	0.675	0.9167	2.5687	-0.3	4.6031	2.8	0.0148	1.8599
4/16/2019 5:42	0.7	-0.15	2.6153	-0.25	4.7969	2.8	0.0197	1.8147
4/16/2019 5:43	0.7	0.6	2.3948	0.4167	4.7221	2.8545	0.0062	1.8217

4/16/2019 5:44	0.7	-0.3333	2.5008	0.1167	4.731	2.9	0.0221	1.8067
4/16/2019 5:45	0.7	-0.4	2.6272	-0.2833	4.7959	2.9	0.0127	1.789
4/16/2019 5:46	0.7	-0.35	2.4268	0.55	4.7826	2.9	0.0126	1.8108
4/16/2019 5:47	0.625	-0.2667	2.2885	0.2167	4.7043	2.9	0.0211	1.8328
4/16/2019 5:48	0.6	0.4667	2.4858	0.1333	4.7794	2.8091	0.0116	1.8303
4/16/2019 5:49	0.6	0.5167	2.4717	0.2667	4.7735	2.8	0.026	1.8226
4/16/2019 5:50	0.6	0.4667	2.4308	0	4.7213	2.8	0.02	1.8267
4/16/2019 5:51	0.5833	-0.7833	2.6685	-0.0667	4.6641	2.8	0.0163	1.8586
4/16/2019 5:52	0.5917	0.15	2.8153	0.1167	4.7063	2.8	0.0311	1.8767
4/16/2019 5:53	0.6	0.3167	2.7763	-0.25	4.7523	2.84	0.0206	1.869
4/16/2019 5:54	0.6	0.8167	2.2668	-0.0333	4.752	2.88	0.0299	1.865
4/16/2019 5:55	0.6	0.5833	2.5245	-0.3	4.7458	2.8	0.0258	1.8483
4/16/2019 5:56	0.6	0	2.5923	-0.8167	4.7513	2.8	0.0201	1.8459
4/16/2019 5:57	0.6	1.05	2.4077	-0.4167	4.7698	2.8	0.0281	1.8642
4/16/2019 5:58	0.6	-0.0833	2.5788	0.2833	4.721	2.8	0.0213	1.8668
4/16/2019 5:59	0.6	0.4333	2.2298	-0.1167	4.7758	2.8	0.031	1.8688
4/16/2019 6:00	0.5583	0.35	2.3238	-0.6	4.7703	2.8091	0.0269	1.886
4/16/2019 6:01	0.525	0.3167	2.1825	0.0333	4.7752	2.9	0.0196	1.8765
4/16/2019 6:02	0.6	0.3167	2.5993	-0.2333	4.7839	2.84	0.0373	1.8556
4/16/2019 6:03	0.5	0.35	2.3815	-0.3833	4.6969	2.8333	0.0199	1.8788
4/16/2019 6:04	0.5	0.4	2.1512	-0.05	4.7282	2.8	0.0299	1.8902
4/16/2019 6:05	0.5	-0.25	2.1675	0.1833	4.7361	2.8	0.0325	1.8655
4/16/2019 6:06	0.5	0.1667	2.4467	-0.25	4.7271	2.8	0.0214	1.8995
4/16/2019 6:07	0.5	1.2167	2.3687	-0.4667	4.749	2.8	0.0339	1.9122
4/16/2019 6:08	0.525	0.2	2.1695	-0.2333	4.7233	2.7545	0.0182	1.9133
4/16/2019 6:09	0.5833	-0.2333	1.6011	-0.8333	4.7164	2.7	0.0306	1.9001
4/16/2019 6:10	0.5833	0.0333	2.7618	-0.3	4.7052	2.7	0.03	1.8903
4/16/2019 6:11	0.5333	-0.2667	2.6367	-0.2167	4.6724	2.7	0.0154	1.9047
4/16/2019 6:12	0.5	0.7	2.4338	-0.0167	4.7658	2.7	0.0349	1.8772
4/16/2019 6:13	0.5	-0.3333	2.7842	-0.0667	4.7352	2.6545	0.0256	1.8732
4/16/2019 6:14	0.5	0.8667	2.5032	0.05	4.7487	2.6	0.0305	1.8764
4/16/2019 6:15	0.5	1.3167	2.4682	-0.1833	4.7494	2.6	0.0326	1.8553
4/16/2019 6:16	0.5333	0.2167	2.6803	0.3333	4.7348	2.6	0.021	1.8909
4/16/2019 6:17	0.55	0.2	2.639	0.1667	4.7493	2.69	0.033	1.9189
4/16/2019 6:18	0.6	-0.0833	2.679	0.1833	4.7089	2.7	0.0242	2.0195
4/16/2019 6:19	0.6	0.1667	2.5055	0.15	4.7065	2.7	0.0346	2.2355
4/16/2019 6:20	0.6	0.0167	2.7955	0.0833	4.6935	2.7636	0.0283	2.2263
4/16/2019 6:21	0.6	0.7333	2.4265	0.2667	4.7372	2.8	0.0226	2.2253
4/16/2019 6:22	0.6833	0.0833	2.5797	0.05	4.7208	2.8778	0.0312	2.1783
4/16/2019 6:23	0.6	0.3833	2.4502	0.05	4.6758	2.9	0.0292	2.1743
4/16/2019 6:24	0.6	-0.0833	2.8422	-0.3333	4.6511	2.9	0.0204	2.2118
4/16/2019 6:25	0.5083	-0.2333	2.6025	-0.25	4.6367	2.9	0.0313	2.2415
4/16/2019 6:26	0.5	0.45	2.6107	-0.2833	4.6423	2.9545	0.0225	2.2064
4/16/2019 6:27	0.5	0.2	2.3767	-0.35	4.5976	3	0.0253	2.2246
4/16/2019 6:28	0.5083	0.6167	2.132	0.3	4.5869	2.9545	0.0326	2.2183
4/16/2019 6:29	0.55	0.25	2.6448	0.1333	4.633	2.9	0.0161	2.2173
4/16/2019 6:30	0.5833	0.0333	2.6288	0.4667	4.6381	2.9	0.0323	2.1806

4/16/2019 6:31	0.5833	0.2	2.414	-0.3167	4.5782	2.9	0.0209	2.2142
4/16/2019 6:32	0.5917	0.1167	2.2775	0.1833	4.5928	2.9	0.0291	2.1967
4/16/2019 6:33	0.5667	0.4667	2.311	0.8167	4.5818	2.9	0.0303	2.1813
4/16/2019 6:34	0.55	0.55	0.9554	-0.1833	4.5781	2.8818	0.0224	2.1796
4/16/2019 6:35	0.5	0.0333	2.2888	0.2667	4.6188	2.8	0.0317	2.171
4/16/2019 6:36	0.5	0.4167	2.2687	0	4.5857	2.8	0.0216	2.1817
4/16/2019 6:37	0.5083	0.0333	2.6505	-0.1167	4.6124	2.8	0.0324	2.1623
4/16/2019 6:38	0.6	0.1167	2.331	0.4167	4.599	2.8	0.0315	2.1608
4/16/2019 6:39	0.5667	0.15	2.304	0.8333	4.6196	2.8	0.0218	2.1432
4/16/2019 6:40	0.5	0.55	2.2725	-0.1833	4.5894	2.7636	0.0301	2.1422
4/16/2019 6:41	0.5	-0.1167	2.5657	0.55	4.5761	2.8	0.0145	2.1477
4/16/2019 6:42	0.5167	-0.1333	2.8195	0.0833	4.6539	2.8	0.0196	2.1205
4/16/2019 6:43	0.5	0.15	2.5835	-0.0833	4.5883	2.77	0.0224	2.1424
4/16/2019 6:44	0.5	0.2667	2.6025	0.6	4.5479	2.7727	0.0186	2.1683
4/16/2019 6:45	0.5	0.1833	2.582	0.0333	4.5988	2.8	0.0253	2.1481
4/16/2019 6:46	0.5	-0.1333	2.6155	0.0833	4.6249	2.8	0.0204	2.094
4/16/2019 6:47	0.55	-0.1	2.7485	-0.2667	4.6448	2.8	0.0196	2.1013
4/16/2019 6:48	0.575	0.5167	2.7507	0.3333	4.6647	2.8	0.0266	2.1155
4/16/2019 6:49	0.4818	0.6167	2.4717	0.0667	4.6085	2.8417	0.0127	2.1552
4/16/2019 6:50	0.4	0	2.5008	0.1667	4.6189	2.9	0.0277	2.1448
4/16/2019 6:51	0.4	0.4667	2.5243	0.3833	4.6963	2.9	0.0238	2.1328
4/16/2019 6:52	0.4	0.2333	2.41	0.2833	4.7302	2.9	0.0126	2.1469
4/16/2019 6:53	0.4	0.0833	2.4708	-0.0667	4.7618	2.9	0.032	2.1666
4/16/2019 6:54	0.4	0.5833	2.5935	0	4.7318	2.9	0.0128	2.1803
4/16/2019 6:55	0.4	-0.1833	2.2952	0.4667	4.729	2.9	0.0238	2.191
4/16/2019 6:56	0.4083	0.0167	1.4176	0.6167	4.7631	2.9	0.0204	2.1691
4/16/2019 6:57	0.4917	0.0167	2.4725	-0.0833	4.8728	2.9	0.0171	2.1747
4/16/2019 6:58	0.4917	0.15	2.5048	0.0333	4.8691	2.9	0.0243	2.1548
4/16/2019 6:59	0.4833	0.3167	2.0462	-0.4167	4.8453	2.9364	0.0138	2.1896
4/16/2019 7:00	0.4	-0.4333	2.4473	0.3167	4.8536	3	0.0232	2.2159
4/16/2019 7:01	0.4	0	3.6408	0.2667	6.0911	3.0273	0.0558	2.2124
4/16/2019 7:02	0.3417	-0.1833	7.7654	-0.4833	11.2048	3.9909	0.1458	2.2507
4/16/2019 7:03	0.3	0.0833	62.1383	2.1833	23.1506	22.53	2.0544	2.5108
4/16/2019 7:04	0.1583	-0.3333	77.6592	0.9667	25.5948	60.1	2.9442	2.7388
4/16/2019 7:05	0.05	-0.05	58.8192	0.9	22.2955	52.8182	2.8778	2.6251
4/16/2019 7:06	-0.0167	-0.6667	54.5658	0.6333	21.1675	51.2833	2.8921	2.6197
4/16/2019 7:07	0	-0.15	52.1917	0.55	20.3511	50.9909	2.93	2.6183
4/16/2019 7:08	0	-0.55	46.7175	0.2667	19.2968	46.1818	2.6719	2.589
4/16/2019 7:09	0.0833	-1	45.63	-0.2	19.3596	47.3909	2.5348	2.5678
4/16/2019 7:10	0.0667	-0.3333	42.2433	-0.2667	18.7288	41.8167	1.9468	2.5212
4/16/2019 7:11	0.1	-0.5167	41.0733	0.0667	18.7808	42.3636	1.8758	2.5207
4/16/2019 7:12	0.15	0.4167	43.95	-0.15	19.3065	42.8727	1.821	2.5545
4/16/2019 7:13	0.2	-0.3833	43.2892	0.4	19.3529	43.8909	1.7957	2.5862
4/16/2019 7:14	0.2	-0.8667	41.9267	-0.2833	19.0685	42.9818	1.7564	2.5964
4/16/2019 7:15	0.2	-0.4667	40.7433	0.3833	18.6704	42.1455	1.7053	2.5568
4/16/2019 7:16	0.1917	-0.4833	38.3792	0.0667	18.375	42.1091	1.6733	2.544
4/16/2019 7:17	0.2	-0.2	37.6533	-0.15	17.9915	42.05	1.6303	2.531

4/16/2019 7:18	0.1417	0.1833	34.9183	0.3833	17.3388	41.7333	1.534	2.5053
4/16/2019 7:19	0.1083	-0.45	33.8133	-0.0833	17.0491	41.39	1.5266	2.519
4/16/2019 7:20	0.2	-0.6167	31.6008	-0.25	16.4975	40.975	1.4334	2.479
4/16/2019 7:21	0.2	-0.4833	31.0333	183.2	16.2569	40.5	1.4434	2.4764
4/16/2019 7:22	0.2	-0.8667	28.3492	21.65	15.7758	39.875	1.3368	2.4451
4/16/2019 7:23	0.1083	-0.3167	27.7925	183.0167	15.608	39.2546	1.3833	2.4474
4/16/2019 7:24	0.1583	-0.2167	29.3183	0.3833	15.7831	38.5909	1.4037	2.4994
4/16/2019 7:25	0.2	-0.4	30.0267	-0.15	15.9042	38.1583	1.4426	2.5027
4/16/2019 7:26	0.2	-0.5	29.4275	-0.3667	16.086	37.7	1.4814	2.5118
4/16/2019 7:27	0.1333	0.05	30.9442	137.8167	16.1224	37.35	1.4968	2.527
4/16/2019 7:28	0.1	-0.05	30.3108	137.6	15.9977	36.9909	1.5046	2.5341
4/16/2019 7:29	0.1	-0.2833	28.2075	219.48	15.6527	36.5818	1.4424	2.5082
4/16/2019 7:30	0.1	-0.2167	27.41	-0.41	15.2656	36.2546	1.3952	2.4495
4/16/2019 7:31	0.1	-0.15	25.8575	-0.3833	15.0037	35.98	1.3578	2.4547
4/16/2019 7:32	0.1	-0.4	23.5933	138.8	14.5093	35.4636	1.2838	2.4386
4/16/2019 7:33	0.0833	0.0333	22.6858	70.1333	14.2313	35.125	1.2824	2.4399
4/16/2019 7:34	0.1	-0.1167	22.2867	298.8	14.0378	34.78	1.2402	2.4063
4/16/2019 7:35	0.1	-0.55	22.2533	80.1917	13.9646	34.4833	1.251	2.4161
4/16/2019 7:36	0.1	-0.45	22.5325	3.0273	13.9876	34.3364	1.2795	2.465
4/16/2019 7:37	0.1	-0.4167	23.1	32.3917	13.9761	34.0364	1.2925	2.4721
4/16/2019 7:38	0.1	0.35	23.9375	208.5583	14.1363	33.71	1.3368	2.4851
4/16/2019 7:39	0.1	-0.5167	23.4383	65.3917	14.0926	33.4364	1.3362	2.4698
4/16/2019 7:40	0.1	0.1	23.0383	124.8	13.9955	33.1	1.3144	2.5008
4/16/2019 7:41	0.1	-0.3833	22.9658	0.8455	13.8116	32.7083	1.2997	2.5156
4/16/2019 7:42	0.1	0.05	21.3392	1.6636	13.5496	32.4364	1.2553	2.4804
4/16/2019 7:43	0.1	-0.0833	19.1733	1.5875	13.1168	32.0583	1.1823	2.4327
4/16/2019 7:44	0.1	-0.1833	20.0675	0.4	13.0735	31.825	1.1998	2.4368
4/16/2019 7:45	0.1	0.1333	19.2242	-0.3667	12.8888	31.5333	1.1721	2.4395
4/16/2019 7:46	0.1	0.0833	17.8475	-0.0167	12.6459	31.2636	1.1404	2.423
4/16/2019 7:47	0.1	-0.0667	18.535	-0.1875	12.6605	31.0667	1.1663	2.4257
4/16/2019 7:48	0.1	-0.1167	18.8433	0.4455	12.6388	30.8417	1.1835	2.4132
4/16/2019 7:49	0.1167	-0.6667	18.5783	-0.1667	12.5876	30.625	1.1913	2.4583
4/16/2019 7:50	0.1667	-0.95	17.49	-0.4917	12.2517	30.25	1.1193	2.3878
4/16/2019 7:51	0.1833	-0.4167	17.9342	-0.4833	12.3384	29.6909	1.1483	2.4293
4/16/2019 7:52	0.1167	-0.4333	18.9775	0.2917	12.477	29.4	1.1803	2.4748
4/16/2019 7:53	0.1	0.0167	18.4933	-0.3417	12.4125	29.1364	1.1861	2.4671
4/16/2019 7:54	0.1	0	18.8858	-0.3917	12.4284	28.9417	1.1901	2.4886
4/16/2019 7:55	0.05	-0.0667	17.6983	0.2583	12.2356	28.73	1.1387	2.4591
4/16/2019 7:56	0.0917	0.05	15.9675	-0.2083	12.219	28.5364	1.0817	2.3944
4/16/2019 7:57	0.1	-0.6833	15.6092	0.2167	12.101	28.2	1.043	2.3908
4/16/2019 7:58	0.1	-0.55	15.5867	-0.55	11.5297	28.0182	1.0579	2.3811
4/16/2019 7:59	0.1	0.0333	13.8667	-0.5	11.3748	27.8182	1.0318	2.3633
4/16/2019 8:00	0.1	-0.0667	14.7625	0.0667	11.3556	27.4818	1.0344	2.4109
4/16/2019 8:01	0.1	-0.15	14.7333	-0.9667	11.3578	27.13	1.0661	2.4331
4/16/2019 8:02	0.0417	-0.3833	14.9125	-0.45	11.3325	26.9273	1.0467	2.424
4/16/2019 8:03	0	-0.3833	15.3908	-0.45	11.424	26.78	1.0806	2.4251
4/16/2019 8:04	0	-0.0667	16.3733	-0.4167	11.4923	26.5727	1.1046	2.4856

4/16/2019 8:05	0	-0.1167	15.6458	-0.1333	11.3124	26.3364	1.0798	2.4908
4/16/2019 8:06	0	0.55	14.9825	-0.0333	11.1783	26.0167	1.0599	2.4451
4/16/2019 8:07	0	-0.1167	15.2458	-0.6833	11.109	25.7727	1.0489	2.4278
4/16/2019 8:08	0.1	0.25	14.4842	0	10.938	25.6083	1.0279	2.4247
4/16/2019 8:09	0.1	0.2833	13.77	0.1333	10.7224	25.4583	0.9912	2.4003
4/16/2019 8:10	0.1167	-0.2833	12.8442	-0.6167	10.6119	25.25	0.9727	2.4091
4/16/2019 8:11	0.15	-0.65	13.2358	-0.1833	10.4588	25.07	0.9667	2.3941
4/16/2019 8:12	0.2	-0.7333	12.5883	-0.7667	10.3461	24.9083	0.9416	2.3937
4/16/2019 8:13	0.2	-0.6	12.3417	-0.3583	10.3089	24.69	0.9536	2.3968
4/16/2019 8:14	0.2417	-0.2	12.4358	-0.2917	10.1933	24.3909	0.9331	2.3685
4/16/2019 8:15	0.3	-0.4	12.0925	-0.4417	10.2189	24.2273	0.9346	2.4186
4/16/2019 8:16	0.3	-0.0833	12.5092	-0.0667	10.3109	23.9083	0.9577	2.4286
4/16/2019 8:17	0.3	-0.6667	12.9742	-0.125	10.3716	23.6636	0.9723	2.4364
4/16/2019 8:18	0.3	0.2833	13.1992	-0.2667	10.3613	23.375	0.9965	2.477
4/16/2019 8:19	0.3	0.6	12.9442	-0.1417	10.2961	23.2364	0.9735	2.4638
4/16/2019 8:20	0.2917	0.1333	12.3192	-0.6167	10.1672	23.0417	0.955	2.4533
4/16/2019 8:21	0.2417	-0.4667	12.3783	-0.15	10.0017	22.8583	0.9422	2.4033
4/16/2019 8:22	0.3	0.0333	11.5533	-0.4	9.7426	22.6636	0.8735	2.3463
4/16/2019 8:23	0.3333	0.0167	10.5867	0.075	9.4928	22.4182	0.8322	2.3102
4/16/2019 8:24	0.5667	0.9	9.9227	-0.325	9.2483	22.0364	0.7779	2.2742
4/16/2019 8:25	1.0083	1.4333	9.9188	1.5667	9.3178	21.425	0.6732	2.1915
4/16/2019 8:26	1.7583	2.6833	9.2802	2.6333	9.2282	20.54	0.5547	2.0774
4/16/2019 8:27	2.625	3.6167	9.3067	3.9	9.4283	19.5182	0.4773	1.9921
4/16/2019 8:28	3.525	5.0833	9.4383	4.5833	9.1902	18.2	0.4056	1.9384
4/16/2019 8:29	4.1364	4.55	9.632	4.5167	9.5307	16.72	0.4031	1.9042
4/16/2019 8:30	4.4167	4.85	9.5202	3.9417	10.2543	15.3583	0.4208	1.9107
4/16/2019 8:31	4.6583	6.2	10.6	5.4833	9.6025	13.8417	0.3724	1.8168
4/16/2019 8:32	4.85	6.1	9.8785	4.8583	9.9084	12.2833	0.3502	1.7952
4/16/2019 8:33	5.3833	7.35	10.1248	6.5167	9.3098	10.7583	0.3249	1.7657
4/16/2019 8:34	5.7583	6.8833	10.9317	7.2833	9.1392	9.3	0.3045	1.7368
4/16/2019 8:35	6.625	7.8167	11.3075	8.4333	8.1448	7.8583	0.2313	1.6962
4/16/2019 8:36	7.4333	9.2167	10.9303	9.0167	7.669	6.5	0.2192	1.6748
4/16/2019 8:37	8.175	9.1167	11.9767	9.0333	7.2234	5.375	0.205	1.6906
4/16/2019 8:38	8.5583	9.6333	12.305	9.4833	6.9764	4.4833	0.1791	1.6693
4/16/2019 8:39	8.7333	9.45	12.4167	9.55	7.2066	3.7667	0.1978	1.6717
4/16/2019 8:40	8.8333	8.9	12.36	9.5	7.214	3.2833	0.1802	1.6948
4/16/2019 8:41	8.85	9.3	11.9667	9.35	7.145	2.9417	0.1863	1.664
4/16/2019 8:42	9.05	9.8667	12.9483	10.425	6.6349	2.6083	0.1694	1.6652
4/16/2019 8:43	9.5	10.9333	12.6133	10.4583	6.1321	2.26	0.1435	1.6611
4/16/2019 8:44	10.2167	11.4	13.8567	11.8833	5.6723	2.0364	0.14	1.6531
4/16/2019 8:45	10.9167	12.45	14.1017	11.9	5.319	1.86	0.1217	1.6351
4/16/2019 8:46	11.4917	12.9	15.1267	12.9083	4.9722	1.6636	0.104	1.6051
4/16/2019 8:47	12.7167	16.4	18.9733	17.7	4.7997	1.5333	0.1025	1.6281
4/16/2019 8:48	14.8833	19.2167	20.11	19.5417	4.8009	1.45	0.0926	1.6628
4/16/2019 8:49	16.725	18.6	20.55	18.95	4.9196	1.3727	0.0943	1.6429
4/16/2019 8:50	17.8167	19.1	20.9367	19.5417	4.798	1.3	0.0887	1.6419
4/16/2019 8:51	17.975	19.7	21.1983	19.95	4.5795	1.3	0.0755	1.6069

4/16/2019 8:52	18.3167	19	20.6133	19.15	4.5773	1.2583	0.0749	1.614
4/16/2019 8:53	18.25	18.05	20.0367	18.5083	4.5933	1.2	0.0853	1.6063
4/16/2019 8:54	18.2	19.0833	21.1867	19.9417	4.6025	1.2	0.0601	1.599
4/16/2019 8:55	18.05	18.2833	20.5767	18.9	4.4983	1.2	0.0741	1.6295
4/16/2019 8:56	18.175	19	19.5983	19.5	4.3765	1.1364	0.0698	1.6345
4/16/2019 8:57	18.55	20.2333	21.3833	21.4	4.364	1.1	0.0631	1.5803
4/16/2019 8:58	19.5083	20.6167	22.6017	21.7	4.2877	1.1	0.0632	1.5812
4/16/2019 8:59	20.4667	21.4333	23.1833	22.6417	3.8186	1.1	0.0442	1.5568
4/16/2019 9:00	20.8	20.3167	21.51	21.6333	3.8033	1.0333	0.0598	1.5671
4/16/2019 9:01	20.5083	20.6	21.6267	21.5417	3.8632	1.1	0.0558	1.5706
4/16/2019 9:02	20.3083	21.4833	23.25	22.9083	3.6352	1	0.045	1.5691
4/16/2019 9:03	20.35	20.9167	21.0717	21.5583	3.6359	1	0.0589	1.5718
4/16/2019 9:04	20.4917	20.75	22.7867	21.35	3.5478	1	0.044	1.5525
4/16/2019 9:05	20.5417	20.7	22.7367	21.4667	3.5011	1	0.0477	1.5525
4/16/2019 9:06	20.5667	21.15	22.4833	21.85	3.4922	1.0091	0.0513	1.5986
4/16/2019 9:07	20.6667	21.8833	23.005	22.125	3.4627	1.1	0.0379	1.6063
4/16/2019 9:08	20.6917	22.0833	23.2533	23.35	3.3918	1.1	0.0543	1.5934
4/16/2019 9:09	21.2273	22.5667	23.91	22.175	3.2119	1.1	0.033	1.6255
4/16/2019 9:10	21.55	21.7167	23.0867	22.5833	3.2123	1.1	0.0345	1.6134
4/16/2019 9:11	21.9	21.6333	23.2817	22.7	3.1385	1.1	0.0349	1.6043
4/16/2019 9:12	21.6833	21.3333	22.985	22.4667	3.1516	1.13	0.0197	1.5968
4/16/2019 9:13	21.5333	21.0667	23.2333	22.3583	3.1418	1.15	0.0421	1.6135
4/16/2019 9:14	20.925	20.4667	22.0733	20.3583	3.1328	1.1833	0.0348	1.6108
4/16/2019 9:15	20.15	19.7667	21.045	19.475	3.1711	1.2	0.0362	1.6389
4/16/2019 9:16	19.3167	19	20.9083	19.2333	3.2085	1.2	0.0452	1.6413
4/16/2019 9:17	18.55	17.9167	19.6633	18.05	3.2521	1.2556	0.0415	1.6506
4/16/2019 9:18	17.7083	16.3833	18.4467	16.9083	3.2889	1.3	0.0458	1.6828
4/16/2019 9:19	16.675	15.6	17.5367	16.8167	3.2751	1.3	0.0489	1.6822
4/16/2019 9:20	15.95	15.9833	17.6583	16.1167	3.2583	1.3	0.0356	1.6672
4/16/2019 9:21	15.5667	15.2833	17.2717	16.025	3.2589	1.31	0.0536	1.7016
4/16/2019 9:22	15.2833	14.9	17.0867	14.4083	3.2344	1.4	0.0397	1.7206
4/16/2019 9:23	15.1333	15	16.9867	16.6	3.2293	1.4	0.0416	1.7211
4/16/2019 9:24	15.05	15.5167	16.99	15.7083	3.2295	1.4	0.0494	1.7118
4/16/2019 9:25	15.0583	15	17.1783	15.4667	3.2408	1.4636	0.0415	1.7185
4/16/2019 9:26	14.8417	14.8833	17.09	15.3417	3.2191	1.5	0.047	1.7377
4/16/2019 9:27	14.525	14.7833	16.465	14.5	3.2281	1.5	0.046	1.741
4/16/2019 9:28	14.15	13.6	15.825	14.025	3.2378	1.5	0.05	1.7276
4/16/2019 9:29	13.525	12.45	14.835	12.7917	3.2389	1.5	0.0468	1.7533
4/16/2019 9:30	12.825	11.5167	14.2283	12.0333	3.3755	1.5167	0.0445	1.7869
4/16/2019 9:31	11.9667	11.1167	25.8867	17.1167	18.8076	2.3455	0.4996	1.8516
4/16/2019 9:32	10.45	9.5333	66.5467	14.1583	25.2095	29.0833	1.361	2.129
4/16/2019 9:33	8.6333	6.95	66.0517	13.6583	25.556	16.8583	1.4678	2.0888
4/16/2019 9:34	5.775	3.1667	92.55	12.9917	40.6261	36.2917	2.4758	2.4057
4/16/2019 9:35	3.375	3.05	126.55	16.8583	48.7722	54.4545	3.43	2.5893
4/16/2019 9:36	1.275	1.1167	137.9833	15.7917	52.3662	65.3546	3.6638	2.7461
4/16/2019 9:37	0.5917	1.8667	136.0333	14.8583	50.6512	62.6417	3.3935	2.6952
4/16/2019 9:38	0.35	2.45	132.0167	15.05	53.465	63.05	3.3078	2.7045

4/16/2019 9:39	0.3	2.6833	138.3	16.225	52.6458	65.975	3.7893	2.7844
4/16/2019 9:40	0.3	1.5167	145.6833	15.9917	51.2682	65.5545	4.5322	2.8485
4/16/2019 9:41	0.3417	2.3833	133.8167	14.825	48.1631	59.6273	4.44	2.8233
4/16/2019 9:42	0.3667	2.85	126.0667	14.3583	47.1486	58.4364	4.1733	2.7482
4/16/2019 9:43	0.3	2.1167	121.8833	14.55	46.2514	58.3556	3.9297	2.7238
4/16/2019 9:44	0.2833	1.65	129.9	14.7417	45.2194	58.06	4.916	2.7773
4/16/2019 9:45	0.2417	2.5667	124.2333	14.0083	45.9911	58.075	4.5332	2.7712
4/16/2019 9:46	0.2	1.6	116.4	12.9083	43.9229	57.92	4.391	2.7367
4/16/2019 9:47	0.2	1.65	111.7833	13.0833	42.7324	57.7167	4.294	2.6878
4/16/2019 9:48	0.2	2.1	110.6167	13.4583	42.9853	57.4583	4.0897	2.6996
4/16/2019 9:49	0.2	1.2833	105.9833	12.725	41.2406	57.25	4.3073	2.6661
4/16/2019 9:50	0.2	1.4833	103.9333	12.7583	41.0265	56.9	4.1792	2.6428
4/16/2019 9:51	0.25	1.4667	102.7	13.0417	41.2931	56.6364	4.0075	2.6553
4/16/2019 9:52	0.3	1.8667	98.5467	12.7583	40.3038	56.05	3.818	2.6397
4/16/2019 9:53	0.3	1.3	96.9067	12.2333	40.0286	55.6818	3.849	2.6568
4/16/2019 9:54	0.4	1.5667	91.4217	12.2333	38.0898	55.1818	3.773	2.6059
4/16/2019 9:55	0.4	1.95	90.1217	12.0333	38.0033	54.6091	3.623	2.5702
4/16/2019 9:56	0.3583	1.4333	89.7767	11.6	37.8091	54.0833	3.6475	2.5943
4/16/2019 9:57	0.3	1.7	87.9992	11.5083	37.5971	53.4091	3.5923	2.6312
4/16/2019 9:58	0.3	2.1167	87.4425	11.9333	37.619	52.875	3.6152	2.6501
4/16/2019 9:59	0.3	1.9167	85.98	11.85	37.5558	52.45	3.554	2.6659
4/16/2019 10:00	0.3167	1.55	85.3392	11.675	36.6968	51.9182	3.5793	2.6338
4/16/2019 10:01	0.3667	1.3667	83.9567	11.1417	36.2049	51.5273	3.5457	2.6109
4/16/2019 10:02	0.4	0.9667	79.1933	10.8583	34.3051	51.0167	3.4022	2.5808
4/16/2019 10:03	0.4	1.05	75.0433	10.725	33.2497	50.3455	3.3065	2.5649
4/16/2019 10:04	0.4	1.0667	76.1	11	33.5945	49.9455	3.3712	2.561
4/16/2019 10:05	0.3917	1.4333	71.8383	10.225	32.011	49.3091	3.1857	2.5247
4/16/2019 10:06	0.3083	1.5833	74.7408	10.6667	32.7224	48.7667	3.3118	2.5728
4/16/2019 10:07	0.3	1.3	74.5125	10.35	33.0136	48.52	3.3767	2.6048
4/16/2019 10:08	0.3	1.4167	70.0525	10.05	31.7096	48.1818	3.221	2.5491
4/16/2019 10:09	0.3	1.1833	68.2458	9.7167	30.6782	47.5667	3.1618	2.5069
4/16/2019 10:10	0.3	1.5667	66.2375	10.0583	30.4396	47.0636	3.1537	2.5401
4/16/2019 10:11	0.3	1	66.3625	9.875	30.0608	46.5833	3.1398	2.5495
4/16/2019 10:12	0.2083	0.8333	66.105	9.5333	29.9748	46.1727	3.1745	2.5123
4/16/2019 10:13	0.2	0.3833	64.6833	9	29.7177	45.6917	3.1572	2.5121
4/16/2019 10:14	0.2	0.4667	62.5217	9.45	28.7011	45.26	3.0557	2.5039
4/16/2019 10:15	0.2	1.0167	64.9958	9.3417	29.3398	44.9583	3.157	2.5241
4/16/2019 10:16	0.1667	1.0667	62.8092	9.4333	28.5788	44.6111	3.08	2.5136
4/16/2019 10:17	0.1667	0.7333	55.1408	8.375	26.5323	44.0636	2.8693	2.454
4/16/2019 10:18	0.2	1.2	55.4117	8.4083	26.1238	43.5833	2.8675	2.437
4/16/2019 10:19	0.1917	1.6833	58.1117	9.0167	26.9423	42.9364	2.9612	2.4801
4/16/2019 10:20	0.1417	0.6667	58.6542	9.3583	27.0806	42.42	3.0087	2.5188
4/16/2019 10:21	0.125	1.4833	54.8717	8.8583	25.9158	42.08	2.8812	2.4899
4/16/2019 10:22	0.2	1.15	53.2783	8.1083	25.1501	41.6182	2.8287	2.4677
4/16/2019 10:23	0.2	1.1333	51.2117	8.15	24.6653	41.1091	2.7962	2.4973
4/16/2019 10:24	0.2	0.8	50.0717	7.0917	24.3962	40.64	2.7712	2.4758
4/16/2019 10:25	0.1333	1.3167	54.0333	7.8917	25.111	40.1818	2.8895	2.4876

4/16/2019 10:26	0.1	0.8833	52.9292	7.775	24.8707	39.89	2.8608	2.4994
4/16/2019 10:27	0.1917	0.9667	51.1317	8.5583	24.2899	39.4364	2.7832	2.4567
4/16/2019 10:28	0.2	0.1167	52.4542	7.9	24.4688	39.1364	2.83	2.4691
4/16/2019 10:29	0.1917	1	52.3442	7.2333	24.2943	39	2.8197	2.4904
4/16/2019 10:30	0.1	0.8	50.9583	7.6583	24.0043	38.9182	2.825	2.4876
4/16/2019 10:31	0.1	0.45	50.4967	7.675	23.6084	38.8417	2.7865	2.5034
4/16/2019 10:32	0.1	1.1167	49.8992	7.325	23.4067	38.6455	2.7837	2.4972
4/16/2019 10:33	0.1667	1.4167	46.6983	6.75	22.3496	38.3583	2.6677	2.454
4/16/2019 10:34	0.2	0.25	45.1583	6.8833	21.6858	38.13	2.6012	2.4598
4/16/2019 10:35	0.2167	0.5	46.27	7.0083	21.8786	37.825	2.6532	2.4747
4/16/2019 10:36	0.2833	1.5833	46.0583	7.25	21.9623	37.6455	2.669	2.4913
4/16/2019 10:37	0.2333	0.85	43.8325	6.7667	21.2343	37.35	2.5885	2.4859
4/16/2019 10:38	0.3	0.6	43.3142	6.8167	20.9841	36.8273	2.5803	2.4917
4/16/2019 10:39	0.3	0.55	41.8533	6.225	20.3694	36.475	2.5067	2.4376
4/16/2019 10:40	0.3	1.05	41.3783	6.775	20.2922	36.02	2.5206	2.4098
4/16/2019 10:41	0.3	0.2833	42.2667	6.6083	20.4506	35.5583	2.5396	2.4245
4/16/2019 10:42	0.3	0.5	42.3225	6.3417	20.4313	35.0818	2.5473	2.4673
4/16/2019 10:43	0.3	1.05	43.8567	6.1667	20.798	34.85	2.6245	2.5242
4/16/2019 10:44	0.3	1.1333	42.9408	5.9667	20.4755	34.4545	2.5852	2.5218
4/16/2019 10:45	0.3	0.4	43.3067	6.4583	20.4977	34.1455	2.5995	2.5308
4/16/2019 10:46	0.3167	0.8833	41.7508	6.1583	19.9568	34.025	2.5327	2.5087
4/16/2019 10:47	0.3083	0.5333	40.7217	5.7833	19.4108	33.7546	2.4761	2.5048
4/16/2019 10:48	0.3333	0.65	37.64	6.1333	18.472	33.36	2.3717	2.4569
4/16/2019 10:49	0.4	0.5667	36.3442	5.5083	18.057	32.9636	2.3282	2.4347
4/16/2019 10:50	0.4	1.1333	36.425	5.65	17.9372	32.56	2.326	2.402
4/16/2019 10:51	0.4667	0.9333	34.0042	5.5333	17.2414	32.2546	2.2119	2.3713
4/16/2019 10:52	0.8667	3.9167	30.9558	6.825	15.9575	31.5667	1.9429	2.2813
4/16/2019 10:53	1.8583	5.5333	27.4108	8.2167	15.6893	30.4917	1.6118	2.1559
4/16/2019 10:54	3.0333	5.9833	25.0958	8.5667	15.5322	29.0909	1.3848	2.0543
4/16/2019 10:55	4.1667	6.95	22.7875	8.8167	15.8081	27.3583	1.1993	1.9783
4/16/2019 10:56	5.0083	7.6333	20.7533	9.7333	15.2485	25.3667	1.0254	1.8575
4/16/2019 10:57	5.3333	6.5333	21.885	8.1333	16.5518	23.5091	1.2651	1.8903
4/16/2019 10:58	5.625	8.05	20.4708	8.825	17.0415	21.5273	1.2516	1.8621
4/16/2019 10:59	5.675	7.9333	20.0883	9.3667	17.065	19.5546	1.1316	1.8198
4/16/2019 11:00	6.3667	9.1167	19.9492	11.1417	16.9928	17.5546	0.9441	1.7678
4/16/2019 11:01	7.2083	9.8833	19.6275	11.425	14.7228	15.4273	0.8529	1.7255
4/16/2019 11:02	8.2417	12.0167	19.9042	12.9333	13.4263	13.3546	0.7685	1.6767
4/16/2019 11:03	9.2583	12.3833	20.3442	14.1917	12.5836	11.35	0.6642	1.6347
4/16/2019 11:04	10.3083	14.2667	20.1633	15.1833	11.2576	9.45	0.5616	1.5918
4/16/2019 11:05	11.1	13.9667	19.9608	14.8167	10.7836	7.975	0.5607	1.5888
4/16/2019 11:06	11.2917	13.3667	19.8958	14.3	11.0223	6.8636	0.5896	1.5978
4/16/2019 11:07	11.2083	13	19.3275	13.9917	10.6128	6.05	0.574	1.5603
4/16/2019 11:08	11.6083	16.1833	20.9067	17.025	9.7828	5.3778	0.4834	1.5293
4/16/2019 11:09	12.4583	15.45	20.9083	16.75	8.9247	4.6364	0.435	1.506
4/16/2019 11:10	13.2417	16.15	20.6567	17.0167	8.5546	4.05	0.4116	1.5212
4/16/2019 11:11	13.975	17.1333	21.0233	17.8833	7.9423	3.55	0.3684	1.5109
4/16/2019 11:12	14.5417	18.2667	21.4667	17.95	7.4521	3.0778	0.3331	1.4778

4/16/2019 11:13	15.0667	17.8167	21.2583	18.6417	7.0381	2.7636	0.3177	1.4522
4/16/2019 11:14	15.425	17.8833	21.0775	19.2917	6.7662	2.4727	0.2822	1.4448
4/16/2019 11:15	15.725	18.9	21.7742	19.0333	6.5223	2.3083	0.2559	1.4643
4/16/2019 11:16	15.9333	17.9833	21.5075	18.8667	6.4466	2.2	0.2691	1.5152
4/16/2019 11:17	16.0667	18.8833	21.8492	19.475	6.1616	2.0917	0.2414	1.4781
4/16/2019 11:18	16.3167	19.2833	21.9617	19.2167	5.8262	1.95	0.2204	1.4513
4/16/2019 11:19	16.5833	19.2833	21.6025	19.7833	5.6728	1.8333	0.2252	1.4608
4/16/2019 11:20	16.9417	19.5833	22.1567	20.275	5.2509	1.7667	0.1855	1.4467
4/16/2019 11:21	17.1167	18.8	21.6833	20.1167	5.1162	1.7	0.1856	1.4473
4/16/2019 11:22	17.4833	19.7833	21.9058	21.075	4.9144	1.6	0.1691	1.4298
4/16/2019 11:23	17.8833	21.2667	22.875	21.9167	4.5995	1.55	0.129	1.4047
4/16/2019 11:24	17.875	19.4167	21.0667	19.7083	4.9758	1.5	0.1594	1.4267
4/16/2019 11:25	17.6167	18.9333	21.6442	19.3667	5.3478	1.5167	0.1616	1.4378
4/16/2019 11:26	16.8417	17.4833	19.8975	17.85	7.1732	1.6	0.1146	1.4143
4/16/2019 11:27	15.9083	15.3667	18.15	15.725	9.4444	1.75	0.1276	1.4065
4/16/2019 11:28	15.0917	15.5833	18.155	16.0167	9.8155	1.8444	0.1217	1.3898
4/16/2019 11:29	14.4417	15.45	18.2867	16.5667	9.1798	1.9167	0.1256	1.4021
4/16/2019 11:30	14.7667	17.55	18.8133	18.0833	8.4373	2	0.0958	1.3727
4/16/2019 11:31	15.4	17.15	19.2483	18.35	7.8775	2	0.0917	1.3534
4/16/2019 11:32	16.1167	18.1333	19.9617	18.925	7.4024	2	0.0682	1.3707
4/16/2019 11:33	16.4667	16.3333	19.025	18.575	7.2513	2	0.0852	1.3705
4/16/2019 11:34	16.375	17.6333	19.385	17.9417	7.1441	2	0.0868	1.3884
4/16/2019 11:35	16.425	17.6667	19.7683	18.55	6.7563	2	0.0668	1.3946
4/16/2019 11:36	16.4917	18.25	19.8767	18.875	6.5919	2	0.07	1.3971
4/16/2019 11:37	16.6333	17.8167	19.34	18.9917	6.5303	2	0.0737	1.4244
4/16/2019 11:38	16.8	18.9167	20.555	19.3833	6.3508	1.9364	0.0643	1.3933
4/16/2019 11:39	17.0667	17.8667	20.24	18.075	6.1818	1.8	0.0686	1.408
4/16/2019 11:40	17.2417	18.6333	20.2383	19.3333	6.0207	1.67	0.0667	1.4322
4/16/2019 11:41	17.5083	19.4333	20.7967	19.9583	5.6865	1.5273	0.0469	1.419
4/16/2019 11:42	17.7083	19.1167	20.505	19.4	5.6367	1.4364	0.0502	1.4181
4/16/2019 11:43	17.9417	18.2333	20.4833	19.3167	5.6465	1.4	0.0521	1.4267
4/16/2019 11:44	18.05	19.2833	20.7267	18.8417	5.4553	1.4	0.0466	1.3728
4/16/2019 11:45	18.25	19.5667	21.3617	20.2167	5.189	1.4	0.0462	1.3348
4/16/2019 11:46	18.2417	18.1333	20.6767	19.2083	5.3532	1.4	0.049	1.3676
4/16/2019 11:47	18.0167	18.8167	20.425	19.7167	5.3753	1.375	0.0387	1.391
4/16/2019 11:48	17.9833	19.1	21.32	19.975	5.0607	1.3	0.0335	1.3833
4/16/2019 11:49	18.3333	20.0667	21.7383	20.6333	4.8994	1.3	0.0349	1.3872
4/16/2019 11:50	18.8667	20.5333	21.995	20.7167	4.6978	1.3	0.0234	1.383
4/16/2019 11:51	19.0917	19.3667	21.5217	19.5	4.6208	1.3	0.0315	1.3564
4/16/2019 11:52	18.875	18.65	21.135	19.5583	4.6683	1.2083	0.0358	1.3385
4/16/2019 11:53	18.65	19.6667	21.515	20.1583	4.6018	1.2	0.0223	1.3373
4/16/2019 11:54	18.75	19.9667	21.9083	20.4833	4.357	1.2	0.0302	1.3289
4/16/2019 11:55	18.9667	19.9167	21.9233	20.925	4.2584	1.2	0.0337	1.3519
4/16/2019 11:56	19.4167	21.05	22.7317	21.375	4.0346	1.2	0.0153	1.3405
4/16/2019 11:57	19.5833	20.3833	22.16	20.8333	3.986	1.2	0.025	1.3528
4/16/2019 11:58	19.6917	20.15	21.59	20.5583	3.9324	1.2	0.0255	1.3508
4/16/2019 11:59	19.8	21.0167	22.6833	21.9	3.7604	1.2	0.0071	1.3776

4/16/2019 12:00	19.8917	21.55	22.4533	21.5833	3.744	1.2	0.0174	1.3758
4/16/2019 12:01	20.075	20.8167	22.665	22	3.7435	1.2	0.0167	1.3702
4/16/2019 12:02	19.9083	20.9	22.3	21.2083	3.6365	1.2	0.0077	1.3648
4/16/2019 12:03	19.975	21.85	23.07	21.2917	3.4788	1.2	0.0167	1.3857
4/16/2019 12:04	20.0417	21.5833	21.915	21.5167	3.4596	1.1417	0.0138	1.3945
4/16/2019 12:05	20.3333	21.9833	22.9217	21.3667	3.4363	1.1455	0.0062	1.3656
4/16/2019 12:06	20.125	20.0167	21.9283	20.4833	3.5033	1.2	0.0314	1.3723
4/16/2019 12:07	20.05	21	22.1717	20.4167	3.3761	1.13	0.0181	1.3591
4/16/2019 12:08	20.0417	21.6833	23.3467	21.6083	3.2849	1.1	0.0079	1.3663
4/16/2019 12:09	20.7417	22.5	24.0317	23.1667	3.1416	1.1	0.0169	1.3593
4/16/2019 12:10	21.2833	21.8167	23.57	22.3167	3.1686	1.1	0.0089	1.3568
4/16/2019 12:11	21.3	21.25	22.21	21.2917	3.2673	1.1	0.0024	1.3868
4/16/2019 12:12	20.85	21.6	22.3033	21.2833	3.3378	1.04	0.0163	1.3872
4/16/2019 12:13	20.2	19.7833	21.685	19.75	3.3543	1.0417	0.0061	1.3611
4/16/2019 12:14	19.7833	20.7	21.5667	20.2667	3.3718	1	0.0134	1.3611
4/16/2019 12:15	19.2917	20.4667	21.6667	19.675	3.3735	1	0.0153	1.3843
4/16/2019 12:16	18.8333	18.2167	20.2817	18.3667	3.3869	1	0.0127	1.3945
4/16/2019 12:17	18.3167	18.3167	19.7567	18.45	3.374	1	0.0179	1.4108
4/16/2019 12:18	17.9	17.7667	20.1467	18.4917	3.3978	1	0.0232	1.4238
4/16/2019 12:19	17.5833	17.6167	19.7367	18.7833	3.3887	1	0.0103	1.4285
4/16/2019 12:20	17.2917	17.4333	19.6517	17.6	3.42	1.025	0.0273	1.4226
4/16/2019 12:21	16.8833	16.5333	18.8667	16.8833	3.4016	1.1	0.0233	1.4369
4/16/2019 12:22	16.275	15.8667	18.37	16.5583	3.4247	1.1091	0.0175	1.4784
4/16/2019 12:23	15.75	15.4	17.9633	15.75	3.4283	1.2	0.0275	1.5183
4/16/2019 12:24	15.075	14.5	17.3133	15.0083	3.4238	1.2	0.0295	1.5178
4/16/2019 12:25	14.65	14.85	17.2217	14.7833	3.4414	1.2	0.0166	1.5281
4/16/2019 12:26	14.2083	13.9167	17	14.2833	3.4403	1.3	0.0337	1.5413
4/16/2019 12:27	13.7417	12.8167	15.8817	13.7667	3.4526	1.3	0.0322	1.5333
4/16/2019 12:28	13.2167	13.35	15.7017	13.2833	3.4533	1.3636	0.0344	1.5278
4/16/2019 12:29	12.4833	11.8333	14.9917	11.975	3.4348	1.4	0.036	1.5701
4/16/2019 12:30	11.8818	11.8333	14.765	11.8083	3.4649	1.4364	0.027	1.6048
4/16/2019 12:31	11.225	10.15	14.2067	11.3917	7.8793	1.6909	0.072	1.6353
4/16/2019 12:32	10.2833	8.9167	16.8517	10.0417	13.3783	2.9	0.3469	1.6763
4/16/2019 12:33	8.7083	6.25	14.8067	7.15	16.3833	4.5083	0.4079	1.672
4/16/2019 12:34	6.1333	1.9833	19.5583	5.0833	33.3388	39.1833	1.6547	1.8085
4/16/2019 12:35	3.4333	-0.1	24.4317	4.45	38.4332	60.9091	3.107	1.9493
4/16/2019 12:36	1.35	0.3667	24.8967	4.225	39.363	60.4167	2.9599	1.9698
4/16/2019 12:37	0.5	0.2833	24.5167	4.35	39.7067	57.1455	1.8882	1.9656
4/16/2019 12:38	0.3167	0.3667	24.3567	4.0417	39.7673	55.9364	1.7275	1.9648
4/16/2019 12:39	0.3	0.2	23.065	3.425	38.4929	55.66	1.665	1.9443
4/16/2019 12:40	0.3	0.6833	22.7417	3.6167	37.9726	53.51	1.6312	1.9248
4/16/2019 12:41	0.3	0.7333	22.18	3.5167	37.1812	52.54	1.5415	1.9071
4/16/2019 12:42	0.35	0.1667	20.425	3.2167	34.0006	48.0727	1.4042	1.8834
4/16/2019 12:43	0.4	-0.1333	20.0817	3.3417	34.5438	49.37	1.438	1.9169
4/16/2019 12:44	0.4	-0.0333	19.8817	3.2917	34.2323	48.79	1.4052	1.9578
4/16/2019 12:45	0.375	0.55	19.5133	3.025	34.0094	48.8667	1.4042	1.9528
4/16/2019 12:46	0.325	0.2833	18.88	3.35	33.0423	48.16	1.3628	1.9463

4/16/2019 12:47	0.3	0.1	18.1467	2.9833	31.9572	48.0091	1.2962	1.9696
4/16/2019 12:48	0.375	0.7	18.1183	3.15	32.2629	47.8833	1.3492	1.9933
4/16/2019 12:49	0.3667	0.0833	18.2017	2.975	32.0735	47.7273	1.3277	1.9875
4/16/2019 12:50	0.2917	-0.4	18.7533	2.625	33.3076	47.75	1.3947	1.9635
4/16/2019 12:51	0.2	-0.7	18.7833	2.9	33.5938	47.8	1.4075	2.0086
4/16/2019 12:52	0.1417	-0.15	18.8283	2.8	32.8888	47.8	1.3738	2.0226
4/16/2019 12:53	0.1	0.3833	18.34	2.4167	32.9696	47.7	1.3938	2.0312
4/16/2019 12:54	0.1583	0.2333	17.7133	2.3833	31.5888	47.5	1.3323	2.026
4/16/2019 12:55	0.2583	0.4833	16.54	2.475	30.4208	47.2091	1.2742	1.995
4/16/2019 12:56	0.3833	0.0667	16.5283	2.625	29.8351	46.8727	1.2728	1.9668
4/16/2019 12:57	0.4	-0.5833	16.4817	2.7	30.2338	46.5417	1.2805	1.9803
4/16/2019 12:58	0.3167	-0.4	15.58	1.825	29.2059	46.1	1.2438	1.9817
4/16/2019 12:59	0.2667	-0.0333	15.9083	2.3833	29.0187	45.7727	1.2553	1.9796
4/16/2019 13:00	0.2333	0.2833	15.5292	1.725	28.5638	45.5	1.218	1.9952
4/16/2019 13:01	0.2	0.2	15.2942	2.1583	28.079	45.0583	1.2127	2.0158
4/16/2019 13:02	0.2	-0.1167	15.6008	1.9667	28.079	44.63	1.254	2.0496
4/16/2019 13:03	0.2	-0.3667	15.6733	1.7333	28.2152	44.0889	1.2213	2.0163
4/16/2019 13:04	0.2	0.3167	15.7608	1.675	28.9963	43.6818	1.276	2.0277
4/16/2019 13:05	0.2083	-0.25	15.9217	1.9583	29.0863	43.3727	1.2693	2.0268
4/16/2019 13:06	0.3	0.2	15.5775	1.875	28.2014	43.0667	1.235	2.0408
4/16/2019 13:07	0.3167	-0.2833	14.9475	2.1083	27.9298	42.8455	1.2335	2.0724
4/16/2019 13:08	0.4	1.1833	15.0342	1.775	27.1157	42.6455	1.1905	2.0783
4/16/2019 13:09	0.4	-0.2333	14.6033	1.8167	26.4883	42.4273	1.183	2.0601
4/16/2019 13:10	0.5	-0.0167	13.9725	1.7083	25.9844	42.2	1.1603	2.0498
4/16/2019 13:11	0.5	-0.1833	13.7583	1.7833	25.5629	41.9091	1.1515	2.0697
4/16/2019 13:12	0.5	-0.4667	13.4858	1.9	24.8138	41.4727	1.1363	2.0489
4/16/2019 13:13	0.425	-0.4167	13.5942	1.7333	24.9391	41.1818	1.1318	2.0738
4/16/2019 13:14	0.4	-0.0667	13.6817	1.6917	25.25	40.9818	1.1543	2.1096
4/16/2019 13:15	0.3917	-0.6167	13.3292	1.6167	24.7648	40.65	1.1417	2.0983
4/16/2019 13:16	0.375	0.05	13.0333	1.55	24.265	40.1	1.1032	2.0884
4/16/2019 13:17	0.3917	-0.3	13.7908	1.525	25.2228	39.7	1.1583	2.1188
4/16/2019 13:18	0.3417	-0.2833	13.9708	1.4167	25.6447	39.4	1.1738	2.1504
4/16/2019 13:19	0.3	0.0333	13.815	1.1	25.2112	39.1364	1.1562	2.1428
4/16/2019 13:20	0.3	-0.2667	13.685	1.7417	25.0035	38.9083	1.1612	2.1547
4/16/2019 13:21	0.2917	-0.65	13.5533	1.2167	24.5526	38.6636	1.1302	2.1119
4/16/2019 13:22	0.2833	-0.2167	12.9008	1.225	23.6883	38.3182	1.1028	2.0732
4/16/2019 13:23	0.3	-0.2167	12.3733	1.125	22.7563	38	1.0728	2.067
4/16/2019 13:24	0.3	0.4667	12.2883	1.625	22.6946	37.6583	1.0665	2.0722
4/16/2019 13:25	0.35	-0.7833	11.6717	1.1833	22.0341	37.5	1.0503	2.0828
4/16/2019 13:26	0.3	0.6167	11.5167	1.425	21.5902	37.0417	1.0317	2.0948
4/16/2019 13:27	0.25	0.3333	12.0025	1.075	22.0102	36.6818	1.0508	2.1084
4/16/2019 13:28	0.2	0.2333	12.0983	0.925	22.5625	36.5818	1.078	2.1076
4/16/2019 13:29	0.2	0.3833	11.4858	1.1833	21.4606	36.3	1.0106	2.1195
4/16/2019 13:30	0.2	0.3167	11.6575	1.2417	21.22	35.825	1.0207	2.1173
4/16/2019 13:31	0.2	0.4333	11.9092	1.025	21.6653	35.5333	1.0437	2.1711
4/16/2019 13:32	0.2	0.1	11.8317	1.5667	21.2226	35.0167	1.015	2.179
4/16/2019 13:33	0.1417	0.3667	11.6642	1.3417	21.275	34.6364	1.0293	2.169

4/16/2019 13:34	0.1417	-0.1833	11.6692	1.2917	21.7151	34.475	1.0468	2.163
4/16/2019 13:35	0.125	-0.3333	11.7392	1.6417	21.3187	34.4	1.035	2.1522
4/16/2019 13:36	0.1833	0.2	11.3125	0.6583	21.1154	34.325	1.0355	2.137
4/16/2019 13:37	0.25	0	11.3417	1.1833	20.6219	34.1	1.0028	2.1108
4/16/2019 13:38	0.3167	0.0667	11.0075	1.5083	20.3342	33.95	1.0044	2.1353
4/16/2019 13:39	0.325	0.1667	11.4475	1.2583	20.1562	33.86	1.0022	2.1194
4/16/2019 13:40	0.3182	-0.35	10.9392	0.3333	19.7806	33.6583	0.9768	2.1501
4/16/2019 13:41	0.4	-0.0167	10.5342	0.9917	19.1128	33.3222	0.9668	2.1285
4/16/2019 13:42	0.4167	-0.1833	10.1188	0.725	18.6992	33.0917	0.9224	2.0654
4/16/2019 13:43	0.5	-0.1167	10.1338	1.3667	18.5808	32.6111	0.9453	2.0886
4/16/2019 13:44	0.5	-0.4667	10.1686	1.0667	18.4328	32.3091	0.9312	2.1278
4/16/2019 13:45	0.425	-0.4667	10.4825	1.2167	19.3113	32.0455	0.9665	2.1823
4/16/2019 13:46	0.375	-0.15	10.7783	0.7333	19.3058	31.8583	0.9824	2.181
4/16/2019 13:47	0.3	-0.1833	10.8092	1.125	19.4478	31.67	0.9801	2.1979
4/16/2019 13:48	0.2333	-0.3667	11.1233	0.875	19.4762	31.4917	0.9933	2.1758
4/16/2019 13:49	0.2	0.45	10.6867	0.8333	19.1705	31.39	0.9852	2.1636
4/16/2019 13:50	0.2	-0.0833	10.3733	1.125	18.4999	31.175	0.9343	2.1771
4/16/2019 13:51	0.2917	-0.2167	10.5292	1.1167	18.0823	30.93	0.9327	2.2007
4/16/2019 13:52	0.3083	0.35	10.3659	0.8917	17.7829	30.65	0.9218	2.2031
4/16/2019 13:53	0.3917	0.3667	9.6837	0.9833	17.1105	30.41	0.8824	2.1576
4/16/2019 13:54	0.4	0.5833	9.8814	1.4667	16.7348	30.1273	0.8734	2.125
4/16/2019 13:55	0.7417	1.3667	10.2975	2.9583	15.4979	29.825	0.7772	2.0636
4/16/2019 13:56	1.75	4.2833	10.9133	4.9083	14.6786	29.3091	0.7083	1.9992
4/16/2019 13:57	3.5083	7.15	13.3067	7.6667	13.936	28.2	0.5807	1.9458
4/16/2019 13:58	5.9083	10.1667	14.8625	10.9667	13.3833	26.6583	0.4729	1.8712
4/16/2019 13:59	8.3333	12.3833	16.0767	12.9333	12.6673	24.66		1.7956
4/16/2019 14:00	10.3333	12.5	16.7467	13.925	12.4258	22.65		1.7584
4/16/2019 14:01	11.4917	13.35	17.0617	14.1	12.1867	20.4455		1.7071
4/16/2019 14:02	12.0583	14.1667	17.4375	13.625	11.292	18.5		1.67
4/16/2019 14:03	12.675	14.95	18.1425	15.85	11.292	16.575		1.6341
4/16/2019 14:04	13.6083	15.5333	18.4042	16.4917	10.9968	14.4833		1.5903
4/16/2019 14:05	14.3583	15.5	18.5167	16.5417	10.8906	12.4667		1.58
4/16/2019 14:06	15.2417	17.6833	19.6992	18.4083	9.8483	10.2917		1.5609
4/16/2019 14:07	15.8	17.1667	20.0308	18.125	9.2479	8.44		1.8953
4/16/2019 14:08	16.8417	19.9	21.2367	20.825	8.2677	6.6364		2.2143
4/16/2019 14:09	18.05	20.85	22.8908	21.55	7.1358	5.24		1.8578
4/16/2019 14:10	18.8909	20.7167	22.4183	21	6.9991	4.27		1.7063
4/16/2019 14:11	19.6583	20.9833	22.9458	21.775	6.4513	3.6636		1.6123
4/16/2019 14:12	19.975	21.9333	23.61	22.275	6.2135	3.1545		1.6138
4/16/2019 14:13	20.2917	21.5333	22.3758	21.3083	6.0229	2.7833		1.5644
4/16/2019 14:14	20.65	22.1333	23.4317	22.6417	5.709	2.4455		1.5517
4/16/2019 14:15	21.1083	23.25	24.93	23.2667	5.27	2.1083		1.5153
4/16/2019 14:16	21.875	23.0333	25.3908	23.7833	5.0455	1.9091		1.4868
4/16/2019 14:17	22.1333	23.4667	24.4492	23.7	4.7432	1.6833		1.4702
4/16/2019 14:18	22.3167	22.9667	24.3525	24.2667	4.3919	1.5583		1.4588
4/16/2019 14:19	22.8083	25.5333	26.2267	26.65	3.9634	1.4455		1.4456
4/16/2019 14:20	23.4917	24.2	26.1325	24.8	3.8253	1.3583		1.4305

4/16/2019 14:21	23.6417	24.2	25.39	24.5417	4.0018	1.3	1.4447
4/16/2019 14:22	23.5583	23.5667	25.2658	24.6417	3.7161	1.2455	1.4453
4/16/2019 14:23	22.8833	23.5667	24.3667	23.1917	4.2149	1.2	1.435
4/16/2019 14:24	22.6917	23.6667	24.7017	23.7833	4.2585	1.18	1.4441
4/16/2019 14:25	22.6417	24.7667	25.825	26.05	4.0391	1.1	1.437
4/16/2019 14:26	23.6583	26.7167	27.3308	27.0583	3.4763	1.0833	1.4204
4/16/2019 14:27	24.4417	26.1167	26.8658	26.5	3.2608	1	1.4015
4/16/2019 14:28	25.0833	26.8	27.4758	27.0583	3.1135	1.025	1.3919
4/16/2019 14:29	25.2333	26.7	27.6217	27.3167	3.0221	1	1.4094
4/16/2019 14:30	25.5	26.7167	27.9783	26.775	2.9012	1	1.4013
4/16/2019 14:31	25.5667	26.45	27.6167	26.8833	2.8705	1	1.3748
4/16/2019 14:32	25.6	25.8667	27.5217	26.25	2.7648	1	1.3643
4/16/2019 14:33	25.5167	25.6833	27.1017	25.8583	2.7325	1	1.3538
4/16/2019 14:34	25.0417	24.7	25.6733	24.9833	2.9305	1	1.3663
4/16/2019 14:35	24.8167	26.4667	27.5233	27.2167	3.0229	1	1.3588
4/16/2019 14:36	24.6333	25.5167	26.76	25.675	3.0133	1	1.3644
4/16/2019 14:37	24.6083	25.2833	25.8117	25.5667	2.9516	1	1.3882
4/16/2019 14:38	24.1917	25.7833	26.1783	26.275	2.9097	1	1.3983
4/16/2019 14:39	24.65	26.8	28.3983	26.925	2.4978	1	1.3701
4/16/2019 14:40	24.9833	25.6	27.1567	25.6583	2.6405	0.9545	1.3907
4/16/2019 14:41	25.5083	27.0667	26.8	26.4833	2.5119	0.9	1.3742
4/16/2019 14:42	25.3917	26.3333	27.7133	27.2583	2.4119	0.9	1.3693
4/16/2019 14:43	25.5083	26.3833	27.7883	26.6667	2.3376	0.9	1.3604
4/16/2019 14:44	25.3417	25.6667	27.445	26.9333	2.4038	0.9	1.3631
4/16/2019 14:45	25.0167	25.0167	26.8467	26.0417	2.5727	0.85	1.3555
4/16/2019 14:46	24.8167	25.6667	26.695	25.3667	2.5577	0.8	1.3451
4/16/2019 14:47	24.925	26.75	27.715	26.925	2.5813	0.8	1.3417
4/16/2019 14:48	25.1167	25.8	27.3983	26.4833	2.5462	0.8	1.3658
4/16/2019 14:49	25.2667	26.5	26.8283	26.4583	2.7408	0.8	1.3596
4/16/2019 14:50	25.0636	25.4667	26.2033	25.825	2.7174	0.74	1.3632
4/16/2019 14:51	25.1083	26	26.735	26.4083	2.5093	0.8	1.3263
4/16/2019 14:52	25.2583	26.9167	28.0267	27.5583	2.4238	0.8	1.3088
4/16/2019 14:53	25.9333	27.7833	28.275	28.2417	2.3089	0.8	1.3218
4/16/2019 14:54	26.4	26.8333	27.9017	28.3083	2.3247	0.7	1.3389
4/16/2019 14:55	26.6917	27.3667	28.2333	27.6167	2.3943	0.7	1.375
4/16/2019 14:56	26.5083	27.0833	28.3817	27.225	2.3729	0.7	1.3543
4/16/2019 14:57	26.2333	26.7	27.775	26.4833	2.2998	0.7	1.3402
4/16/2019 14:58	25.9917	26	27.4867	26.7667	2.327	0.7	1.3243
4/16/2019 14:59	25.8083	26.2833	27.7317	27	2.3304	0.7	1.3215
4/16/2019 15:00	25.7917	26.6333	27.8683	27.075	2.3403	0.7	1.3257
4/16/2019 15:01	25.8167	26.7667	27.8983	27.2333	2.3624	0.7	1.3493
4/16/2019 15:02	25.7417	27	27.4933	26.75	2.2565	0.7364	1.354
4/16/2019 15:03	25.7083	26.95	27.8717	27.9167	2.3094	0.8	1.3073
4/16/2019 15:04	25.8	27	27.8783	27.525	2.31	0.8	1.3051
4/16/2019 15:05	26.2167	28.0167	28.4933	28.7083	2.1603	0.8	1.2899
4/16/2019 15:06	26.4417	26.15	27.9083	26.9083	1.9961	0.8	1.2853
4/16/2019 15:07	26.3167	25.9833	27.6167	27.125	2.0471	0.8	1.3094

4/16/2019 15:08	25.775	25.8667	26.97	26.025	2.0523	0.8		1.3574
4/16/2019 15:09	25.1583	25.6333	26.145	25.9917	2.0824	0.8		1.3722
4/16/2019 15:10	24.6333	25.0833	25.9033	24.9	2.1018	0.825		1.371
4/16/2019 15:11	24.025	23.8667	25.0017	24.075	2.1301	0.8455		1.3553
4/16/2019 15:12	23.3917	22.8167	24.6967	23.6333	2.1638	0.9		1.415
4/16/2019 15:13	22.65	21.8833	23.82	23.525	2.205	0.9		1.4261
4/16/2019 15:14	22	22.05	23.47	22.3083	2.2124	0.9556		1.4113
4/16/2019 15:15	21.4667	21.8333	23.0967	21.575	2.2386	1		1.404
4/16/2019 15:16	20.9333	20.7	22.485	21.2667	2.2786	1		1.4049
4/16/2019 15:17	20.4167	19.8833	22.325	21.1333	2.2748	1.0909		1.4212
4/16/2019 15:18	19.9	19.25	21.2017	20.4167	2.3047	1.1455		1.4566
4/16/2019 15:19	19.3583	19.0333	21.3783	19.375	2.3298	1.2		1.4814
4/16/2019 15:20	18.8333	18.7833	20.6333	19.1167	2.3611	1.23		1.4828
4/16/2019 15:21	18.3833	18.2167	20.2867	18.45	2.3817	1.3		1.4518
4/16/2019 15:22	17.875	17.9167	18.88	17.2833	2.3956	1.34		1.4718
4/16/2019 15:23	17.275	17.0833	19.1517	17.225	2.4323	1.4		1.4784
4/16/2019 15:24	16.525	15.7167	17.925	16.2417	2.4618	1.4083		1.4773
4/16/2019 15:25	15.9667	15.2833	18.1567	15.7333	2.4876	1.5		1.4908
4/16/2019 15:26	15.2667	13.8167	16.77	14.0167	2.5658	1.5583		1.5069
4/16/2019 15:27	14.6583	14.05	16.5633	13.9167	2.5579	1.6		1.531
4/16/2019 15:28	14.075	13.35	16.4567	13.875	2.5673	1.6		1.5094
4/16/2019 15:29	13.6917	13.45	15.9783	13.6583	2.6043	1.6909		1.5197
4/16/2019 15:30	13.3667	12.6667	15.3733	13.4667	2.5918	1.7		1.5323
4/16/2019 15:31	12.8667	12.1167	15.3133	12.7	2.6292	1.7		1.5294
4/16/2019 15:32	12.5083	12.0333	14.8617	12.1	2.6222	1.8		1.5196
4/16/2019 15:33	12.0667	11.5333	14.5767	12.1917	2.6449	1.8		1.5268
4/16/2019 15:34	11.6583	11.0667	14.3317	11.8083	2.6683	1.8		1.541
4/16/2019 15:35	11.3	10.0833	13.7567	10.8667	2.6689	1.8		1.5646
4/16/2019 15:36	10.9	10.6167	13.6667	10.6917	2.6831	1.8		1.5805
4/16/2019 15:37	10.875	10.9667	13.9717	11.1417	2.6367	1.8273		1.591
4/16/2019 15:38	10.7417	9.7667	13.865	9.9333	2.6776	1.8		1.5924
4/16/2019 15:39	10.6083	9.15	14.0333	9.4167	2.7096	1.8182		1.5933
4/16/2019 15:40	10.325	9.4667	12.185	10.1167	2.7174	1.88	0.2147	1.588
4/16/2019 15:41	10.15	10.05	12.8683	9.3167	2.6888	1.9	0.2184	1.6293
4/16/2019 15:42	9.825	8.8167	12.2867	9.2583	2.7389	1.9	0.2101	1.5973
4/16/2019 15:43	9.4167	8.8	11.9167	8.7083	2.7491	1.9	0.2233	1.5774
4/16/2019 15:44	8.9833	9.0333	11.6133	9.2333	2.7632	1.9	0.2143	1.5925
4/16/2019 15:45	8.9	9.15	11.8117	9.2	2.7344	1.91	0.2232	1.6045
4/16/2019 15:46	8.9833	8.9333	11.8133	8.8917	2.7177	2	0.2309	1.5974
4/16/2019 15:47	8.9917	8.45	11.72	8.675	2.6953	2	0.2199	1.6071
4/16/2019 15:48	8.8917	8.45	11.5433	8.675	2.7186	2.03	0.2277	1.609
4/16/2019 15:49	8.7917	7.95	11.4717	8.55	2.6968	2.1	0.2323	1.6063
4/16/2019 15:50	8.7	7.45	10.9517	8	2.7056	2.1	0.2225	1.6342
4/16/2019 15:51	8.5667	7.2833	10.98	7.7917	2.6988	2.1455	0.228	1.6583
4/16/2019 15:52	8.2833	7.7667	10.53	7.7583	2.7088	2.2	0.2181	1.645
4/16/2019 15:53	8.1083	7.0167	10.9083	7.6667	2.6983	2.2	0.227	1.6383
4/16/2019 15:54	7.95	7.8	10.6767	7.525	2.6856	2.2	0.2232	1.6152

4/16/2019 15:55	7.8833	7.3167	10.845	7.7833	2.7024	2.2364	0.22	1.6311
4/16/2019 15:56	7.7083	6.5333	10.445	6.975	2.712	2.3	0.2288	1.5944
4/16/2019 15:57	7.6083	7.35	9.9177	6.9167	2.6858	2.3	0.2254	1.6066
4/16/2019 15:58	7.5583	7.2167	10.3105	7.0167	2.6783	2.4	0.219	1.6442
4/16/2019 15:59	7.3917	6.8167	9.9328	6.575	2.7118	2.4	0.2315	1.6313
4/16/2019 16:00	7.225	7.25	9.7285	6.8583	2.6957	2.3917	0.2251	1.6106
4/16/2019 16:01	7.0583	6.6167	10.0749	6.725	2.6976	2.3	0.2211	1.6455
4/16/2019 16:02	7	5.9	9.7222	6.2333	2.7137	2.3273	0.2248	1.6568
4/16/2019 16:03	6.9	5.8833	9.7613	7.2333	2.6947	2.4	0.2132	1.6428
4/16/2019 16:04	6.8417	6.3167	9.5123	6.1417	2.6861	2.4	0.2234	1.6423
4/16/2019 16:05	6.775	6.45	9.6549	6.275	2.685	2.4	0.2207	1.6221
4/16/2019 16:06	6.5917	5.4167	9.6217	6.125	2.6888	2.4	0.2194	1.6476
4/16/2019 16:07	6.325	5.2167	9.5522	5.6167	2.6733	2.43	0.2335	1.6557
4/16/2019 16:08	6.1167	6.1	9.2533	6.475	2.6823	2.4	0.2105	1.6402
4/16/2019 16:09	5.9833	5.5	9.1489	5.6417	2.6831	2.4	0.2287	1.6415
4/16/2019 16:10	5.8417	5.1667	9.2211	5.25	2.6949	2.4	0.2327	1.6366
4/16/2019 16:11	5.7333	4.9167	8.8378	5.4583	2.6943	2.4	0.2172	1.6423
4/16/2019 16:12	5.5583	5.4667	8.8398	5.8167	2.6861	2.4364	0.2312	1.6418
4/16/2019 16:13	5.45	4.95	8.8051	5.7833	2.6816	2.5	0.2216	1.6434
4/16/2019 16:14	5.2667	5.0833	8.746	4.8	2.6941	2.5	0.2205	1.6556
4/16/2019 16:15	5.1333	4.9167	8.4103	4.65	2.6838	2.5	0.2273	1.6713
4/16/2019 16:16	4.975	4.9	8.4273	4.6083	2.6747	2.5	0.2152	1.6842
4/16/2019 16:17	4.875	4.6167	8.2182	4.425	2.6783	2.5	0.2241	1.6538
4/16/2019 16:18	4.8	4.05	7.9262	4.4417	2.6923	2.5	0.2237	1.6756
4/16/2019 16:19	4.65	5	7.94	4.6333	2.7047	2.5	0.2135	1.6762
4/16/2019 16:20	4.55	4.1833	8.0345	4.6583	2.6938	2.5	0.2304	1.6663
4/16/2019 16:21	4.4583	4.05	7.8694	4.3917	2.7147	2.51	0.22	1.6878
4/16/2019 16:22	4.4	4.5833	8.0644	4.4667	2.6893	2.6	0.2354	1.6938
4/16/2019 16:23	4.35	4.2833	7.7147	4.3333	2.7061	2.6	0.2279	1.6555
4/16/2019 16:24	4.3	3.75	7.5718	3.8917	2.694	2.55	0.2182	1.6413
4/16/2019 16:25	4.2667	3.5	7.5804	3.875	2.7125	2.5818	0.2333	1.6356
4/16/2019 16:26	4.175	3.5667	7.2293	3.3583	2.7298	2.6	0.223	1.6553
4/16/2019 16:27	4.0833	3.5833	7.4504	3.0917	2.7119	2.6	0.2244	1.668
4/16/2019 16:28	3.9667	3.6167	7.3261	3.2083	2.7312	2.6333	0.2337	1.6963
4/16/2019 16:29	3.9	3.4667	7.2539	4.3167	2.7153	2.7	0.2193	1.7033
4/16/2019 16:30	3.8818	2.9333	7.2072	3.8667	2.7278	2.7	0.2326	1.7026
4/16/2019 16:31	3.8583	3.95	7.0978	3.875	2.7268	2.7	0.2265	1.6982
4/16/2019 16:32	3.8333	3.2833	7.3061	3.9667	2.7072	2.7	0.2189	1.6999
4/16/2019 16:33	3.7917	3.1	7.0903	3.3583	2.7263	2.7727	0.2313	1.7325
4/16/2019 16:34	3.75	3.4833	7.0615	3.2083	2.7205	2.8	0.227	1.724
4/16/2019 16:35	3.6833	2.4667	6.9813	3.8417	2.7194	2.8	0.2232	1.7013
4/16/2019 16:36	3.6	3.3	6.9445	3.0333	2.7111	2.8	0.2306	1.6963
4/16/2019 16:37	3.5667	2.2667	6.764	2.9667	2.7044	2.8	0.2213	1.6801
4/16/2019 16:38	3.525	3.2667	6.9463	3.2583	2.7313	2.8	0.2203	1.6678
4/16/2019 16:39	3.5	3.3	6.8475	2.8583	2.7105	2.8	0.2245	1.703
4/16/2019 16:40	3.4	3.6	6.6844	2.4333	2.7122	2.8	0.2113	1.7323
4/16/2019 16:41	3.325	3.4167	6.8153	2.7667	2.7213	2.8	0.2228	1.6929

4/16/2019 16:42	3.3	2.8333	6.4712	2.7	2.7166	2.8	0.2232	1.654
4/16/2019 16:43	3.2917	2.7	6.6728	2.6083	2.7028	2.8	0.2146	1.6889
4/16/2019 16:44	3.225	2.8667	6.4853	2.5083	2.6888	2.8	0.2239	1.7019
4/16/2019 16:45	3.2083	2.55	6.4986	2.8833	2.7025	2.8	0.2147	1.6993
4/16/2019 16:46	3.1083	1.8833	6.575	2.8917	2.6989	2.8	0.2212	1.6963
4/16/2019 16:47	3.1	2.6667	6.6319	2.8833	2.7197	2.8	0.2186	1.6945
4/16/2019 16:48	3.0167	2.7333	5.6657	1.7167	2.7057	2.82	0.2014	1.6997
4/16/2019 16:49	2.9917	2.1833	6.5672	2.1583	2.6956	2.8727	0.2186	1.7045
4/16/2019 16:50	2.9333	2.5333	6.3773	2.05	2.6893	2.8	0.2142	1.7069
4/16/2019 16:51	2.9	2.3833	6.2181	2.625	2.7123	2.8	0.2146	1.7027
4/16/2019 16:52	2.8667	3.1833	6.2766	2.2667	2.7043	2.8	0.2284	1.6876
4/16/2019 16:53	2.7333	2.4833	6.3495	2.7583	2.6749	2.8	0.208	1.6822
4/16/2019 16:54	2.7	2.3167	6.2292	2.2583	2.6896	2.8	0.2144	1.6904
4/16/2019 16:55	2.7083	1.6667	6.2594	2.175	2.7201	2.8	0.2185	1.6798
4/16/2019 16:56	2.8	1.7	6.1798	1.9167	2.7193	2.8	0.2048	1.6799
4/16/2019 16:57	2.7417	2.3833	6.1333	1.5917	2.7073	2.79	0.2195	1.6642
4/16/2019 16:58	2.7167	2.35	5.908	2.2917	2.7054	2.7	0.2131	1.6515
4/16/2019 16:59	2.625	2.4167	5.7802	1.8917	2.7069	2.7	0.2085	1.6865
4/16/2019 17:00	2.5917	1.75	5.7749	2.3	2.7003	2.625	0.2165	1.6862
4/16/2019 17:01	2.5083	2.3833	5.9275	1.5917	2.6968	2.6	0.2064	1.6964
4/16/2019 17:02	2.5	2.0333	6.0233	1.9583	2.6995	2.6	0.2165	1.7107
4/16/2019 17:03	2.5	1.6333	6.0033	2.625	2.7013	2.6	0.2149	1.7191
4/16/2019 17:04	2.5	1.75	5.8725	2.525	2.7202	2.6083	0.2067	1.7356
4/16/2019 17:05	2.4	1.9833	5.9583	2.0083	2.7009	2.6091	0.2233	1.7408
4/16/2019 17:06	2.4	2.3	6.1122	2.325	2.695	2.6	0.213	1.7168
4/16/2019 17:07	2.4083	1.55	5.8847	2.2417	2.6949	2.6333	0.2116	1.7268
4/16/2019 17:08	2.4833	2.0333	5.1989	2	2.7055	2.6833	0.2189	1.7236
4/16/2019 17:09	2.5	2.2833	5.9211	2.2583	2.7026	2.7	0.2141	1.7045
4/16/2019 17:10	2.525	2.6667	6.1494	2.3833	2.7164	2.7	0.2145	1.7135
4/16/2019 17:11	2.5333	2.5667	5.964	1.35	2.7266	2.7636	0.2199	1.7197
4/16/2019 17:12	2.5	1.3667	5.7104	1.975	2.7188	2.8	0.2128	1.757
4/16/2019 17:13	2.4333	1.9667	6.0568	1.4	2.7087	2.8	0.2236	1.7511
4/16/2019 17:14	2.4	1.9167	5.7415	1.575	2.7083	2.8	0.2109	1.7222
4/16/2019 17:15	2.425	1.75	6.1508	2.2667	2.7016	2.8	0.2099	1.7024
4/16/2019 17:16	2.5167	2.45	5.9893	2.0917	2.7009	2.7727	0.2251	1.7283
4/16/2019 17:17	2.5083	2.15	5.8349	2.1417	2.6968	2.8	0.2082	1.7405
4/16/2019 17:18	2.4583	1.9833	5.9547	1.9333	2.7083	2.8	0.2161	1.7478
4/16/2019 17:19	2.325	1.6	5.7956	1.8167	2.6894	2.8	0.218	1.73
4/16/2019 17:20	2.325	1.4333	5.5507	2.1833	2.678	2.8	0.2057	1.6768
4/16/2019 17:21	2.35	1.8167	5.823	2	2.6776	2.8	0.2184	1.6932
4/16/2019 17:22	2.3167	1.9167	5.9529	1.9667	2.6795	2.8	0.2141	1.6913
4/16/2019 17:23	2.275	2.0667	5.7807	2.0667	2.6632	2.83	0.2078	1.6869
4/16/2019 17:24	2.275	1.4667	5.9231	1.7	2.6709	2.9	0.22	1.7189
4/16/2019 17:25	2.25	1.2167	5.721	1.8083	2.688	2.9	0.2114	1.7372
4/16/2019 17:26	2.225	1.5833	5.7503	2.0083	2.6703	2.9	0.2134	1.705
4/16/2019 17:27	2.2	1.3	5.3983	2.275	2.6618	2.9	0.2219	1.7177
4/16/2019 17:28	2.1833	1.95	5.7667	1.6333	2.6703	2.9	0.2064	1.7393

4/16/2019 17:29	2.1	1.55	5.7245	1.9083	2.6552	2.9	0.2178	1.7514
4/16/2019 17:30	2.0333	1.4667	5.7998	2.15	2.6583	2.9	0.2093	1.7176
4/16/2019 17:31	2.1	1.65	5.7912	1.7333	2.659	2.9	0.2118	1.7179
4/16/2019 17:32	2.1917	2	5.7917	0.9083	2.6468	2.9	0.2198	1.7208
4/16/2019 17:33	2.2083	2.2167	6.0212	2.375	2.6369	2.9	0.2046	1.7223
4/16/2019 17:34	2.2083	1.7	5.784	2.4	2.6478	2.9	0.2234	1.7293
4/16/2019 17:35	2.1417	1.2333	5.7948	1.7583	2.6472	2.86	0.2135	1.724
4/16/2019 17:36	2.1583	1.3	5.9402	1.6833	2.6433	2.8	0.2099	1.7033
4/16/2019 17:37	2.1417	1.45	5.8923	1.6583	2.6378	2.8	0.2231	1.7345
4/16/2019 17:38	2.1333	1.7333	5.7427	1.3083	2.6388	2.8	0.2143	1.7578
4/16/2019 17:39	2.1	1.9667	5.8583	1.5917	2.6383	2.8	0.2163	1.7525
4/16/2019 17:40	2.0083	1.9833	5.7793	1.4	2.6327	2.85	0.2197	1.7335
4/16/2019 17:41	2.0545	1.4	5.5353	1.5917	2.6353	2.9	0.2133	1.7456
4/16/2019 17:42	2.0083	1.2667	5.7742	1.7167	2.6176	2.9	0.2234	1.7836
4/16/2019 17:43	2	1.6	5.6077	1.6	2.6334	2.9	0.2116	1.7742
4/16/2019 17:44	1.9417	1.3	5.5938	1.3	2.6377	2.9909	0.219	1.7453
4/16/2019 17:45	1.9167	1.7333	5.4513	1.825	2.6233	3	0.2249	1.7255
4/16/2019 17:46	1.9	1.4333	5.5002	1.2083	2.6208	3	0.2074	1.7365
4/16/2019 17:47	1.9	1.4167	5.4913	1.5417	2.6168	3	0.2163	1.7367
4/16/2019 17:48	1.9	1.3333	5.645	1.775	2.6057	3	0.2113	1.7583
4/16/2019 17:49	1.9	1.4	5.647	1.45	2.6072	3	0.2117	1.7621
4/16/2019 17:50	1.9	1.65	5.4745	1.575	2.6053	3	0.2183	1.762
4/16/2019 17:51	1.9	1.3333	5.4608	2.075	2.6006	3	0.2099	1.7532
4/16/2019 17:52	1.825	0.8833	5.4057	1.9333	2.5893	3	0.2165	1.72
4/16/2019 17:53	1.8	1.2333	5.5848	1.7083	2.5885	2.975	0.2213	1.722
4/16/2019 17:54	1.825	1.3833	5.3628	1.5333	2.5686	2.9	0.2122	1.7548
4/16/2019 17:55	1.85	1.9333	5.149	1.2167	2.5788	2.9	0.2155	1.7418
4/16/2019 17:56	1.9167	2.1	5.6672	1.8833	2.5823	2.9	0.2004	1.7333
4/16/2019 17:57	1.9833	1.5667	5.499	1.6333	2.5734	2.9	0.221	1.7602
4/16/2019 17:58	2	1.5667	5.6478	1.2417	2.5809	2.9	0.2176	1.7622
4/16/2019 17:59	2	1.25	5.8877	1.4917	2.5609	2.8909	0.2077	1.7931
4/16/2019 18:00	1.9917	0.8667	5.6347	1.375	2.5465	2.8	0.222	1.7869
4/16/2019 18:01	2	1.3833	5.5525	1.75	2.5565	2.8	0.2107	1.7878
4/16/2019 18:02	2	1.75	5.868	1.7167	2.5393	2.8	0.2156	1.7784
4/16/2019 18:03	2.05	1.4333	5.8288	1.8	2.5366	2.8	0.2186	1.7663
4/16/2019 18:04	2.1	1.45	5.5167	1.725	2.5305	2.8	0.2093	1.7883
4/16/2019 18:05	2.075	1.5667	5.6918	1.6583	2.535	2.8	0.2241	1.8036
4/16/2019 18:06	2	2.1333	5.6372	1.725	2.5359	2.8	0.2155	1.7959
4/16/2019 18:07	1.9917	1.4	5.4543	1.8333	2.537	2.8	0.2136	1.7918
4/16/2019 18:08	2	1.5833	5.6838	1.8417	2.5324	2.8	0.2175	1.803
4/16/2019 18:09	2	2.1	5.4937	1.3833	2.5283	2.8	0.2152	1.8035
4/16/2019 18:10	2	1.7833	5.8675	1.75	2.5278	2.8818	0.2222	1.804
4/16/2019 18:11	2	2.05	5.693	1.8833	2.5088	2.9	0.2149	1.8214
4/16/2019 18:12	2.0417	1.5	5.678	1.825	2.5009	2.9	0.2127	1.8183
4/16/2019 18:13	2.1	1.7	5.603	2.15	2.4902	2.9364	0.2219	1.7778
4/16/2019 18:14	2.0917	2.2	5.7655	1.45	2.4998	2.9444	0.2084	1.7849
4/16/2019 18:15	2.1	2.0667	5.5887	1.6667	2.4848	2.9	0.2205	1.8108

4/16/2019 18:16	2.1083	1.5833	5.8452	2.2	2.4834	2.9	0.2137	1.8048
4/16/2019 18:17	2.1833	1.95	5.7943	2.2667	2.4987	2.9	0.2087	1.7996
4/16/2019 18:18	2.2	1.6333	5.738	2.1	2.4803	2.9	0.2278	1.8012
4/16/2019 18:19	2.25	1.3667	6.0483	1.5333	2.4612	2.9	0.2137	1.7959
4/16/2019 18:20	2.3	1.6333	6.0322	2.075	2.4764	2.9	0.2255	1.7736
4/16/2019 18:21	2.3	2.1167	5.9218	1.8833	2.4604	2.9	0.2119	1.7553
4/16/2019 18:22	2.3	1.7	5.6683	1.9833	2.4588	2.9	0.2152	1.7758
4/16/2019 18:23	2.3	1.7667	5.774	2.15	2.4461	2.8727	0.2252	1.7936
4/16/2019 18:24	2.3833	2.35	5.5577	2.1333	2.4633	2.87	0.209	1.7818
4/16/2019 18:25	2.4	2.45	5.8252	1.5833	2.4469	2.8	0.2179	1.7735
4/16/2019 18:26	2.4333	1.7833	5.8125	1.625	2.4569	2.83	0.2129	1.7736
4/16/2019 18:27	2.4	2.0333	5.9288	1.7	2.4379	2.9	0.2133	1.7808
4/16/2019 18:28	2.4	2.3833	5.7957	1.9333	2.4453	2.9	0.2252	1.804
4/16/2019 18:29	2.4	1.9833	5.941	1.6333	2.4572	2.9	0.21	1.7887
4/16/2019 18:30	2.4	2.0833	5.9347	2.3833	2.4474	2.9	0.2208	1.78
4/16/2019 18:31	2.4083	1.5667	5.9018	2.3	2.4359	2.9	0.2131	1.8001
4/16/2019 18:32	2.4833	1.7333	6.1683	2.2417	2.42	2.9	0.2098	1.8193
4/16/2019 18:33	2.5	2.1	5.6438	1.5417	2.4396	2.9182	0.2285	1.7963
4/16/2019 18:34	2.5	1.7167	5.6217	2.025	2.4369	3	0.2126	1.7968
4/16/2019 18:35	2.4167	2.3167	5.7595	1.8083	2.4358	3	0.2111	1.7903
4/16/2019 18:36	2.4	1.7667	5.615	1.9167	2.44	3	0.2186	1.8228
4/16/2019 18:37	2.425	2.2167	5.762	2.2333	2.4182	3	0.2047	1.8022
4/16/2019 18:38	2.5	2.4667	5.95	2.1417	2.4147	3	0.2244	1.7707
4/16/2019 18:39	2.5083	1.5333	5.8648	2.0167	2.4364	3	0.213	1.7741
4/16/2019 18:40	2.5083	2.2333	5.7597	1.8167	2.3939	3	0.2163	1.7903
4/16/2019 18:41	2.4091	2.1833	6.0282	2.2167	2.3989	3	0.2241	1.7999
4/16/2019 18:42	2.4	1.7167	5.6503	1.7167	2.3971	3	0.2095	1.8013
4/16/2019 18:43	2.4	2.5833	6.0198	2.2917	2.3852	3	0.2218	1.815
4/16/2019 18:44	2.4	1.65	5.8988	1.925	2.4094	2.9889	0.2146	1.8294
4/16/2019 18:45	2.35	1.8167	5.629	1.425	2.3998	2.9	0.2143	1.834
4/16/2019 18:46	2.3167	2.2667	5.4708	1.8417	2.3981	2.9	0.222	1.8386
4/16/2019 18:47	2.2833	1.6	5.8603	1.7667	2.3803	2.9	0.2123	1.8066
4/16/2019 18:48	2.2083	2.3667	5.7618	1.2833	2.37	2.9	0.2169	1.7966
4/16/2019 18:49	2.1167	2.1167	5.7707	1.725	2.4014	2.9	0.2122	1.8063
4/16/2019 18:50	2.1	1.9167	5.9347	1.8833	2.3778	2.9	0.2161	1.7788
4/16/2019 18:51	2.1	2.55	5.6968	1.775	2.3824	2.9	0.2185	1.8107
4/16/2019 18:52	2.1	1.7667	5.6703	1.8917	2.3853	2.9	0.2072	1.8387
4/16/2019 18:53	2.0667	1.2167	5.6385	1.725	2.3779	2.9	0.2173	1.8255
4/16/2019 18:54	2.0917	1.8333	5.6652	1.5583	2.3597	2.9	0.2093	1.8147
4/16/2019 18:55	2.1583	2.0833	5.4255	1.8083	2.3592	2.8667	0.214	1.81
4/16/2019 18:56	2.125	1.5333	5.2917	1.6417	2.3661	2.9	0.2199	1.8093
4/16/2019 18:57	2.1417	2.1167	5.4543	1.8167	2.3563	2.9	0.2048	1.8015
4/16/2019 18:58	2.1583	2.2333	5.743	1.5333	2.3689	2.9	0.2186	1.8104
4/16/2019 18:59	2.1833	1.8667	5.497	1.75	2.3638	2.9	0.2093	1.8223
4/16/2019 19:00	2.1167	2.3	5.6044	1.2167	2.3625	2.9	0.2164	1.8293
4/16/2019 19:01	2.1	2.3667	5.994	1.4417	2.354	2.9	0.2171	1.8216
4/16/2019 19:02	2.0417	1.6333	5.8453	2.125	2.3617	2.9	0.206	1.8204

4/16/2019 19:03	2.0167	1.3	5.6845	1.5417	2.3589	2.9	0.2201	1.8113
4/16/2019 19:04	2.0667	1.9167	5.6817	2.3333	2.3285	2.9	0.2039	1.8411
4/16/2019 19:05	2.1	1.5	5.2748	1.6833	2.3543	2.9	0.2143	1.8571
4/16/2019 19:06	2.1	1.8	5.6812	2.3417	2.3447	2.9	0.2196	1.8174
4/16/2019 19:07	2.1	1.9333	5.2696	2.2167	2.344	2.9	0.207	1.7958
4/16/2019 19:08	2.1	1.95	5.6498	2.15	2.348	2.9	0.2169	1.7972
4/16/2019 19:09	2.1	2.15	5.7297	2.3	2.3484	2.9	0.2093	1.8237
4/16/2019 19:10	2.1	2.2833	5.4136	1.9417	2.3661	2.9	0.2168	1.8202
4/16/2019 19:11	2.1167	1.8667	5.716	1.925	2.3761	2.9	0.2125	1.8092
4/16/2019 19:12	2.15	1.1333	5.4628	1.725	2.3656	2.9	0.2084	1.8249
4/16/2019 19:13	2.2	1.6833	5.7879	1.4083	2.3816	2.9	0.2149	1.8073
4/16/2019 19:14	2.1417	1.6833	5.3663	2.0417	2.3951	2.9	0.199	1.7978
4/16/2019 19:15	2.1	1.4167	5.4805	1.55	2.4052	2.9	0.2189	1.8255
4/16/2019 19:16	2.0833	0.8667	5.3671	1.55	2.4118	2.9	0.2125	1.8363
4/16/2019 19:17	2.05	1.6667	5.3448	2.1167	2.4292	2.9	0.2113	1.8431
4/16/2019 19:18	2.0417	2.3	5.1858	2.2583	2.4309	2.9	0.2123	1.833
4/16/2019 19:19	2	1.0333	5.6697	1.0917	2.413	2.9	0.1987	1.8278
4/16/2019 19:20	1.9833	1.5	5.4096	2.1917	2.4325	2.9	0.216	1.8402
4/16/2019 19:21	1.9167	1.0333	5.18	1.5833	2.4154	2.9	0.2073	1.8363
4/16/2019 19:22	1.9417	1.15	5.7774	1.4083	2.4574	2.9	0.2045	1.8161
4/16/2019 19:23	1.9083	1.6333	5.2667	1.7	2.4635	2.9167	0.2154	1.7997
4/16/2019 19:24	1.9	1	5.3111	1.1917	2.4682	3	0.2008	1.8259
4/16/2019 19:25	1.9333	2.0833	5.272	1.3333	2.4854	3	0.2109	1.823
4/16/2019 19:26	2	1.1333	5.3764	1.6	2.4758	3	0.2052	1.7999
4/16/2019 19:27	2	1.8333	5.5316	1.775	2.4999	3	0.1997	1.8168
4/16/2019 19:28	1.925	1.8833	5.497	0.975	2.5134	3	0.212	1.8186
4/16/2019 19:29	1.9	1.2833	5.375	1.7	2.5053	3	0.1996	1.8073
4/16/2019 19:30	1.9	1.6667	5.3505	1.3417	2.5251	3	0.2089	1.8398
4/16/2019 19:31	1.9	1.4333	5.3502	1.3083	2.5285	3	0.2018	1.831
4/16/2019 19:32	1.8417	1.4833	5.2003	1.7667	2.543	3	0.203	1.8099
4/16/2019 19:33	1.8	1.8833	5.2565	1.9917	2.5581	3	0.2068	1.8013
4/16/2019 19:34	1.8	1.8	5.2858	1.1167	2.5576	3	0.2037	1.8216
4/16/2019 19:35	1.8	2.0833	5.2226	1.3333	2.5668	3	0.2013	1.8136
4/16/2019 19:36	1.8	1.9167	5.2782	1.5833	2.5678	3	0.2148	1.8478
4/16/2019 19:37	1.8	0.7333	5.2069	1.475	2.559	2.96	0.1968	1.8114
4/16/2019 19:38	1.8	0.7833	5.2869	1.5417	2.5991	2.9	0.2101	1.8162
4/16/2019 19:39	1.8	1.05	5.2009	1.8083	2.5842	2.9	0.1997	1.7994
4/16/2019 19:40	1.8	1.6167	5.1413	1.575	2.5794	2.9	0.2018	1.8078
4/16/2019 19:41	1.7917	1.4833	4.772	1.4333	2.5979	2.9	0.2039	1.8133
4/16/2019 19:42	1.7583	0.8	5.1776	1.3333	2.599	2.9	0.1951	1.8033
4/16/2019 19:43	1.7667	1.4333	5.1615	1.8417	2.6018	2.9	0.2097	1.8076
4/16/2019 19:44	1.75	1.95	5.1947	0.9083	2.6143	2.9	0.1943	1.7823
4/16/2019 19:45	1.7	1.6167	5.2809	1.05	2.6323	2.8667	0.2073	1.8088
4/16/2019 19:46	1.6917	1.65	5.2235	1.4833	2.6424	2.8	0.2077	1.8418
4/16/2019 19:47	1.6	1.7	4.763	1.3417	2.6492	2.8	0.1941	1.841
4/16/2019 19:48	1.6	1.4667	4.9572	0.7417	2.6463	2.8	0.2088	1.8408
4/16/2019 19:49	1.65	0.9333	5.256	1.6917	2.6434	2.8	0.1896	1.8298

4/16/2019 19:50	1.7	0.8833	5.1911	1.6	2.6222	2.8	0.2044	1.7873
4/16/2019 19:51	1.6833	1.7833	5.3955	1.475	2.6555	2.8	0.203	1.7845
4/16/2019 19:52	1.675	1.4667	4.9448	1.95	2.6359	2.8	0.195	1.805
4/16/2019 19:53	1.7	1.7667	5.4123	1.25	2.6287	2.8	0.2066	1.7989
4/16/2019 19:54	1.7	1.4	5.1993	1.025	2.6579	2.8	0.1923	1.8135
4/16/2019 19:55	1.7	1.6333	5.1324	1.0583	2.6863	2.8	0.2052	1.8185
4/16/2019 19:56	1.7083	1.75	5.0318	1.3917	2.6781	2.8	0.1962	1.8118
4/16/2019 19:57	1.8	1.2	5.0712	0.8	2.6784	2.8636	0.1969	1.8097
4/16/2019 19:58	1.775	1.0333	5.1666	1.6833	2.6888	2.84	0.2011	1.8028
4/16/2019 19:59	1.7	1.6333	4.9707	0.725	2.6874	2.9	0.1917	1.8073
4/16/2019 20:00	1.7	0.85	5.4086	0.9167	2.6995	2.9	0.2012	1.7722
4/16/2019 20:01	1.6917	1.5333	5.1066	1.6417	2.7034	2.9	0.1993	1.7681
4/16/2019 20:02	1.6833	1.6167	4.9003	1.9417	2.7086	2.9	0.1982	1.7808
4/16/2019 20:03	1.7	1.3	5.1528	1.55	2.7453	2.9	0.2016	1.8218
4/16/2019 20:04	1.7	0.9833	5.1364	1.0833	2.7451	2.9	0.1924	1.8439
4/16/2019 20:05	1.725	1.0167	4.9812	1.2167	2.7275	2.9	0.2058	1.8304
4/16/2019 20:06	1.7	1.0167	4.9848	1.3583	2.7292	2.9	0.1987	1.8386
4/16/2019 20:07	1.7583	1.6	4.7094	1.175	2.7451	2.9	0.1939	1.8073
4/16/2019 20:08	1.8	1.4833	4.9917	1.3917	2.7867	2.9	0.1971	1.8087
4/16/2019 20:09	1.8	1.8833	4.7713	0.9417	2.788	2.9	0.1915	1.8042
4/16/2019 20:10	1.8	1.55	4.9348	1.3167	2.7869	2.9	0.2018	1.8218
4/16/2019 20:11	1.7167	1.4833	4.9819	1.0583	2.7668	2.9	0.1949	1.8179
4/16/2019 20:12	1.7	1.6667	4.8388	0.9583	2.7679	2.9	0.1893	1.8008
4/16/2019 20:13	1.6583	1.4	4.9897	1.1833	2.8058	2.9	0.2081	1.8128
4/16/2019 20:14	1.7	1.4333	4.979	1.375	2.8158	2.9	0.186	1.791
4/16/2019 20:15	1.7	1.4667	5.0371	1.5833	2.7868	2.9	0.1976	1.8009
4/16/2019 20:16	1.7	0.7	4.8913	1.325	2.7922	2.9	0.1908	1.8114
4/16/2019 20:17	1.625	1.2333	4.9144	1.7833	2.8133	2.9	0.1919	1.8143
4/16/2019 20:18	1.6	1.3333	4.8175	0.9917	2.8106	2.9	0.2004	1.7972
4/16/2019 20:19	1.6	0.7833	4.7245	1.7667	2.8334	2.9	0.18	1.7835
4/16/2019 20:20	1.6	1.9833	4.8623	1.5	2.84	2.9	0.1983	1.776
4/16/2019 20:21	1.6083	0.7333	4.7134	1.5083	2.8224	2.9	0.1977	1.7811
4/16/2019 20:22	1.625	1.2	4.8092	1.575	2.8444	2.9	0.1929	1.7764
4/16/2019 20:23	1.6	1.7833	4.6794	1.9667	2.8462	2.9	0.2061	1.8092
4/16/2019 20:24	1.6	1.4667	4.6902	0.9	2.8667	2.94	0.1904	1.8126
4/16/2019 20:25	1.6	1.7833	4.8268	1.2583	2.8507	3	0.2027	1.8101
4/16/2019 20:26	1.6	1.4167	4.934	0.5833	2.8427	3	0.1935	1.8188
4/16/2019 20:27	1.5583	1.1167	4.8923	1.2667	2.8558	3	0.1864	1.8173
4/16/2019 20:28	1.5	1.1333	4.827	0.9917	2.8573	3	0.2034	1.8246
4/16/2019 20:29	1.5	1.2833	4.9403	1.1083	2.8702	3	0.1905	1.8363
4/16/2019 20:30	1.5	1.2167	4.7428	1.0417	2.8772	3	0.1897	1.836
4/16/2019 20:31	1.5	0.95	4.6857	1.0917	2.8688	3.0636	0.2003	1.8354
4/16/2019 20:32	1.5083	0.8833	4.738	1.0667	2.8783	3	0.1836	1.8242
4/16/2019 20:33	1.5833	1.1833	5.0248	0.9417	2.8881	3	0.1972	1.7965
4/16/2019 20:34	1.5167	0.85	4.7767	0.9	2.8668	3	0.1926	1.7875
4/16/2019 20:35	1.5	1.35	4.733	1.0583	2.8788	3	0.1925	1.7731
4/16/2019 20:36	1.5	1.15	4.7685	1.225	2.8864	3	0.1971	1.8278

4/16/2019 20:37	1.525	1.7667	4.7468	1.5	2.8936	2.9727	0.1764	1.825
4/16/2019 20:38	1.55	1.1667	4.7788	1	2.8907	2.9	0.2003	1.8021
4/16/2019 20:39	1.6	1.1	4.683	0.95	2.8827	2.9	0.1985	1.7987
4/16/2019 20:40	1.6	1.8833	4.5788	0.7333	2.8971	2.9	0.1946	1.8348
4/16/2019 20:41	1.6	1.1833	4.6302	0.8	2.9098	2.9	0.2008	1.8296
4/16/2019 20:42	1.6	1.6	4.6112	0.9	2.9294	2.9	0.1875	1.8095
4/16/2019 20:43	1.6167	0.9167	4.6815	1.45	2.9105	2.9	0.1969	1.8013
4/16/2019 20:44	1.7	1.2	4.8618	0.9833	2.9088	2.9	0.1883	1.8021
4/16/2019 20:45	1.6583	0.8	4.5392	1.05	2.9048	2.9	0.1933	1.8337
4/16/2019 20:46	1.575	1.65	4.98	1.3417	2.8993	2.9	0.1997	1.7963
4/16/2019 20:47	1.5	1.4833	4.694	0.8167	2.9095	2.9	0.1896	1.7878
4/16/2019 20:48	1.5	0.8	4.617	1.2333	2.9162	2.9	0.2015	1.796
4/16/2019 20:49	1.5	0.6	4.7027	1.2417	2.9053	2.9	0.1901	1.8313
4/16/2019 20:50	1.4917	1.05	4.504	1.2917	2.9148	2.9	0.1932	1.8282
4/16/2019 20:51	1.5	1.4833	4.6817	1.075	2.9008	2.9	0.1945	1.8317
4/16/2019 20:52	1.5	0.8	4.6697	1.0417	2.905	2.9	0.1849	1.8068
4/16/2019 20:53	1.6	1.1167	4.4698	1.1333	2.9062	2.95	0.2038	1.8014
4/16/2019 20:54	1.6	1.05	4.8193	1.1	2.9078	2.9364	0.1856	1.7901
4/16/2019 20:55	1.575	0.45	4.8068	0.8583	2.9088	2.9636	0.1881	1.7846
4/16/2019 20:56	1.4833	0.9	4.5572	1.2833	2.9103	2.9	0.1982	1.7847
4/16/2019 20:57	1.4	0.8667	4.6252	0.3667	2.9113	2.9	0.1865	1.8276
4/16/2019 20:58	1.3917	1.2333	4.6833	1.0083	2.9226	2.9	0.1961	1.8219
4/16/2019 20:59	1.4167	1.65	4.8052	0.7083	2.9124	2.9	0.1944	1.8193
4/16/2019 21:00	1.5	1.4833	4.8657	1.4167	2.9093	2.9	0.1938	1.8251
4/16/2019 21:01	1.5091	1.4333	4.6688	1.625	2.9093	2.9	0.1963	1.803
4/16/2019 21:02	1.5583	1.8167	4.5362	1.325	2.8999	2.8636	0.1795	1.8039
4/16/2019 21:03	1.5917	1.0167	4.4275	1.2167	2.9214	2.8545	0.2011	1.8205
4/16/2019 21:04	1.6	1.3333	4.9723	0.7	2.9203	2.8	0.1985	1.7853
4/16/2019 21:05	1.6	1.2333	4.6752	1.2583	2.9153	2.8	0.1929	1.8013
4/16/2019 21:06	1.6	1.0833	4.8782	1.2333	2.904	2.8	0.1992	1.8215
4/16/2019 21:07	1.6	0.8167	4.5463	0.9417	2.9198	2.8	0.1863	1.8218
4/16/2019 21:08	1.6	1.3	4.6022	0.9667	2.9198	2.8	0.1967	1.8343
4/16/2019 21:09	1.525	1.3833	4.4943	0.9917	2.9103	2.8	0.1962	1.8413
4/16/2019 21:10	1.5	1.1667	4.4788	1.4917	2.9	2.8	0.1948	1.8398
4/16/2019 21:11	1.5833	1.3333	4.7637	1.325	2.8998	2.8	0.2015	1.8169
4/16/2019 21:12	1.6	1.8167	4.8765	1.1583	2.9073	2.8	0.1826	1.812
4/16/2019 21:13	1.6	1.1833	4.4393	0.5833	2.92	2.8	0.1963	1.8071
4/16/2019 21:14	1.6	0.5833	4.387	0.6833	2.907	2.8	0.201	1.8011
4/16/2019 21:15	1.6	1.5833	4.5175	1.0667	2.9219	2.8	0.1838	1.8219
4/16/2019 21:16	1.6	1.1167	4.5167	0.9917	2.9174	2.8182	0.2002	1.823
4/16/2019 21:17	1.6	1.1833	4.7043	1.4333	2.9336	2.85	0.1882	1.8243
4/16/2019 21:18	1.5417	0.5167	4.7302	1.0167	2.942	2.9	0.1943	1.8136
4/16/2019 21:19	1.5333	0.9667	4.8272	1.1417	2.918	2.9	0.2003	1.8355
4/16/2019 21:20	1.5	1.1667	4.5135	1.1	2.9296	2.9	0.1886	1.8318
4/16/2019 21:21	1.4083	1.1333	4.5248	0.8	2.9295	2.9	0.2002	1.8144
4/16/2019 21:22	1.4417	1.75	4.5815	1.1	2.9443	2.9	0.2009	1.8176
4/16/2019 21:23	1.5	1.5667	4.5235	1.2083	2.9429	2.9	0.1922	1.8406

4/16/2019 21:24	1.5	1.3833	4.4642	0.7167	2.9352	2.9	0.2013	1.867
4/16/2019 21:25	1.4667	0.6833	4.7937	1.1	2.9357	2.9	0.1901	1.8358
4/16/2019 21:26	1.4	1.25	4.6342	1.575	2.9226	2.9	0.1938	1.8311
4/16/2019 21:27	1.4167	1.8	4.7485	1.1833	2.9429	2.9545	0.1995	1.84
4/16/2019 21:28	1.4083	1.7667	4.6752	1.0083	2.9463	2.9636	0.1886	1.8197
4/16/2019 21:29	1.4833	0.95	4.5617	1.1667	2.9397	2.9	0.2015	1.8395
4/16/2019 21:30	1.4	0.95	4.4617	0.55	2.9339	2.9636	0.1884	1.8523
4/16/2019 21:31	1.4	1.4167	4.5818	1.175	2.9278	3	0.1955	1.8495
4/16/2019 21:32	1.4	1.75	4.9495	1.525	2.9323	3	0.2003	1.849
4/16/2019 21:33	1.4	1.1667	4.4637	1.3417	2.9454	3	0.1898	1.8281
4/16/2019 21:34	1.45	1	4.554	1.225	2.9441	2.9727	0.2042	1.8325
4/16/2019 21:35	1.4	0.8333	4.487	1.425	2.9467	2.9636	0.1895	1.8197
4/16/2019 21:36	1.4	0.6833	4.7678	1.175	2.9503	2.925	0.2012	1.8069
4/16/2019 21:37	1.4	0.9833	4.7312	1.45	2.9667	2.9909	0.2032	1.8436
4/16/2019 21:38	1.4	1.05	4.527	1.375	2.9483	3	0.1916	1.8515
4/16/2019 21:39	1.4583	0.6667	4.6977	1.2083	2.939	2.9455	0.2054	1.8763
4/16/2019 21:40	1.4583	0.9333	4.713	0.75	2.9542	2.9083	0.1829	1.8335
4/16/2019 21:41	1.5	1.3667	5.0763	1.375	2.9578	3	0.2016	1.8281
4/16/2019 21:42	1.5	1.1667	4.5892	1.025	2.9683	3	0.1931	1.8214
4/16/2019 21:43	1.5	1.1167	4.555	1.1417	2.9622	2.9727	0.1913	1.8398
4/16/2019 21:44	1.5	1.0833	4.5053	1.1417	2.9489	2.9	0.2017	1.8484
4/16/2019 21:45	1.55	1.6833	4.7585	1.2667	2.9642	2.9	0.1856	1.8123
4/16/2019 21:46	1.6583	1.3	4.3655	0.95	2.9827	2.9	0.2033	1.813
4/16/2019 21:47	1.6167	1.2167	4.678	1.4917	2.96	2.9	0.1996	1.8129
4/16/2019 21:48	1.6	1.3167	4.3045	0.9167	2.9779	2.9	0.1907	1.8125
4/16/2019 21:49	1.625	1.0333	4.4738	1.0667	2.9862	2.9	0.2022	1.7954
4/16/2019 21:50	1.7	1.1333	4.4335	0.675	2.9711	2.9	0.1937	1.7996
4/16/2019 21:51	1.7	0.7167	4.5322	0.6167	2.9719	2.9	0.2055	1.8018
4/16/2019 21:52	1.6917	1.3167	4.5683	1.2083	2.9731	2.9	0.2019	1.8263
4/16/2019 21:53	1.675	1.45	4.6217	1.3417	2.9875	2.9	0.1986	1.8618
4/16/2019 21:54	1.7	1.1333	4.5663	1.4083	2.9926	2.9	0.2057	1.8353
4/16/2019 21:55	1.775	1.7	4.6825	0.7833	2.9707	2.9	0.1888	1.8248
4/16/2019 21:56	1.8167	1.15	4.3582	1.3833	2.9888	2.9	0.2053	1.8225
4/16/2019 21:57	1.8	0.8333	4.5527	0.55	2.9933	2.95	0.2038	1.8053
4/16/2019 21:58	1.775	1.45	4.2937	1.5667	2.9833	3	0.1979	1.8364
4/16/2019 21:59	1.725	1.4667	4.5251	1.0333	2.9698	3	0.2067	1.8508
4/16/2019 22:00	1.7	1	4.3611	0.6833	2.9945	3	0.1912	1.8342
4/16/2019 22:01	1.7	0.7667	4.4911	1.2333	2.9665	3	0.2054	1.8182
4/16/2019 22:02	1.7833	0.7667	4.4454	1.05	2.9898	3	0.2014	1.8182
4/16/2019 22:03	1.75	1.1667	4.2638	1.4917	2.9782	3	0.2012	1.8153
4/16/2019 22:04	1.8	1.3333	4.5932	1.225	3.0058	3	0.2074	1.8226
4/16/2019 22:05	1.7333	0.8667	4.2225	0.8833	2.9896	3	0.1989	1.8072
4/16/2019 22:06	1.7	1.3667	4.241	1.2083	2.9829	3	0.1996	1.8138
4/16/2019 22:07	1.7583	0.8833	4.6355	1.0667	3.0083	3	0.2107	1.8394
4/16/2019 22:08	1.7333	1.1333	4.4752	0.8667	2.9991	3	0.1903	1.8572
4/16/2019 22:09	1.7	1.85	4.4904	0.725	2.9856	2.9545	0.2061	1.8332
4/16/2019 22:10	1.7	0.95	4.4529	0.975	3.0014	2.9	0.1957	1.8359

4/16/2019 22:11	1.7	0.8667	4.3703	0.3667	3.001	2.9	0.2087	1.8074
4/16/2019 22:12	1.7	1.2667	4.226	0.3917	3.001	2.9	0.2026	1.8013
4/16/2019 22:13	1.7	1.5333	4.5636	0.7833	3.0101	2.9	0.1951	1.7811
4/16/2019 22:14	1.675	1.0833	4.46	0.5833	3.0126	2.8818	0.2124	1.8061
4/16/2019 22:15	1.6833	1.15	4.5039	0.9167	2.9842	2.84	0.188	1.8431
4/16/2019 22:16	1.7	1.4833	4.3241	0.7833	3.0263	2.9	0.2039	1.8382
4/16/2019 22:17	1.7	1.3667	4.2715	1.125	3.0194	2.9	0.2033	1.8236
4/16/2019 22:18	1.7	0.7	4.1971	0.9083	3.0133	2.9	0.199	1.8449
4/16/2019 22:19	1.7	0.75	4.3755	1	3.0185	2.9	0.2055	1.8407
4/16/2019 22:20	1.6583	0.7333	4.5418	1.0333	2.9943	2.9	0.1941	1.81
4/16/2019 22:21	1.6	0.75	4.6821	0.7667	3.0069	2.9	0.2033	1.7893
4/16/2019 22:22	1.5667	0.9167	4.265	1.4417	3.0128	2.9	0.1966	1.7953
4/16/2019 22:23	1.575	0.3	4.52	0.9583	3.0132	2.9	0.2012	1.8138
4/16/2019 22:24	1.6	1.7167	4.1873	1.6083	3.0013	2.9	0.2055	1.8073
4/16/2019 22:25	1.6	1.2833	4.3009	1.325	3.0078	2.9	0.1923	1.8254
4/16/2019 22:26	1.6	1.0667	4.4625	1.4083	3.0029	2.9	0.2079	1.823
4/16/2019 22:27	1.6083	1.6833	4.3671	1.6	2.9998	2.9	0.1955	1.8105
4/16/2019 22:28	1.7	1.1833	4.6095	1.3083	2.9846	2.9	0.1981	1.8198
4/16/2019 22:29	1.7	1.2667	4.2793	1.15	2.989	2.9	0.2064	1.8188
4/16/2019 22:30	1.625	1.3333	4.7179	1.6	2.9769	2.9	0.1918	1.819
4/16/2019 22:31	1.6	1.8167	4.5093	1.4833	2.9861	2.9	0.2074	1.8078
4/16/2019 22:32	1.6	1.95	4.5957	1.25	2.9783	2.99	0.2005	1.8245
4/16/2019 22:33	1.6	0.7667	4.2694	0.8417	2.9844	2.93	0.195	1.8364
4/16/2019 22:34	1.7	1.3667	4.4113	1.075	2.9679	2.9	0.1997	1.8276
4/16/2019 22:35	1.6583	0.95	4.3028	0.9833	2.975	2.9	0.194	1.8101
4/16/2019 22:36	1.6833	1.5833	4.4538	1.5917	2.9659	2.9	0.202	1.8289
4/16/2019 22:37	1.625	0.8333	4.4723	1.4917	2.9834	2.9556	0.1965	1.8283
4/16/2019 22:38	1.6417	1.55	4.592	1.6833	2.9729	2.9818	0.1923	1.8553
4/16/2019 22:39	1.6833	1.0333	4.6545	1.2167	2.9665	2.9091	0.204	1.8353
4/16/2019 22:40	1.7	1.8167	4.6006	0.9583	2.9691	2.92	0.1862	1.8036
4/16/2019 22:41	1.8	0.6333	4.2422	0.9833	2.9382	3	0.2049	1.7948
4/16/2019 22:42	1.75	1.25	4.4411	-0.075	2.9544	3	0.1959	1.7908
4/16/2019 22:43	1.7	1.2333	4.5765	1.4417	2.9498	2.9727	0.202	1.8248
4/16/2019 22:44	1.6833	1.55	4.6061	0.875	2.9259	2.9364	0.1997	1.8441
4/16/2019 22:45	1.6083	0.9	4.4528	0.75	2.9383	3	0.197	1.7973
4/16/2019 22:46	1.6	1.2333	4.3675	0.95	2.9306	3	0.2018	1.7961
4/16/2019 22:47	1.6	1.3833	4.4287	1.2083	2.9296	3.01	0.1894	1.8318
4/16/2019 22:48	1.6833	1.5	4.7153	1.35	2.9053	3.0091	0.1937	1.8154
4/16/2019 22:49	1.7	1.9833	4.5627	1.8833	2.9195	3	0.2	1.8323
4/16/2019 22:50	1.6917	0.9167	4.4524	1.5167	2.9068	3	0.1886	1.8084
4/16/2019 22:51	1.6083	1.2833	4.5133	1.2167	2.9185	3	0.203	1.8323
4/16/2019 22:52	1.5917	0.95	4.4436	1.65	2.9116	3	0.1977	1.8296
4/16/2019 22:53	1.6	1.2333	4.4379	1.225	2.8941	3	0.1958	1.8082
4/16/2019 22:54	1.575	1.0833	4.5563	0.975	2.9036	3	0.1983	1.7926
4/16/2019 22:55	1.5917	0.6	4.6046	1.0833	2.892	3	0.1871	1.8145
4/16/2019 22:56	1.5583	1.1667	4.5543	1.3833	2.8746	3	0.2019	1.8236
4/16/2019 22:57	1.6	1.2333	4.1152	1.4917	2.8734	3	0.1864	1.8368

4/16/2019 22:58	1.6	1.9333	4.3306	0.7583	2.8808	2.93	0.1986	1.8297
4/16/2019 22:59	1.6	0.7	4.369	1.4083	2.8715	2.9	0.1988	1.833
4/16/2019 23:00	1.6	1.2	4.3001	1.0917	2.8594	2.9	0.195	1.868
4/16/2019 23:01	1.65	1.0333	4.3714	1.575	2.8493	2.9	0.2068	1.8385
4/16/2019 23:02	1.7	1.8	4.5123	1.725	2.8365	2.9	0.191	1.8504
4/16/2019 23:03	1.6917	0.2667	4.2231	1.2083	2.8428	2.9	0.2	1.8624
4/16/2019 23:04	1.6167	1.35	4.2743	1.4167	2.844	2.9	0.1968	1.8433
4/16/2019 23:05	1.6417	1.8	4.4178	1.3833	2.8243	2.9	0.1905	1.8535
4/16/2019 23:06	1.7	1.5833	4.2918	1.4083	2.8234	2.9	0.2039	1.8278
4/16/2019 23:07	1.7167	1.0667	4.4285	1.1583	2.8301	2.9	0.1876	1.8203
4/16/2019 23:08	1.725	1.2333	4.4106	0.6083	2.8116	2.9	0.1974	1.8223
4/16/2019 23:09	1.7	0.7833	4.4753	0.8083	2.8208	2.8545	0.2005	1.7976
4/16/2019 23:10	1.7	1.7333	4.4663	1.3	2.8263	2.8364	0.1986	1.8068
4/16/2019 23:11	1.7	1.1833	4.5558	1.1917	2.814	2.9	0.2015	1.8283
4/16/2019 23:12	1.7167	1.4	4.5188	1.0083	2.811	2.9	0.1907	1.8121
4/16/2019 23:13	1.7	1.3167	4.466	0.7833	2.811	2.9	0.189	1.7953
4/16/2019 23:14	1.7	2.1	4.0857	1.325	2.7988	2.9545	0.201	1.835
4/16/2019 23:15	1.725	1.1	4.4175	1.0583	2.7748	3	0.1904	1.8394
4/16/2019 23:16	1.8	1.4333	4.5066	1.4417	2.7723	2.9818	0.1976	1.8065
4/16/2019 23:17	1.7417	1.7	4.3399	1.1083	2.7921	3	0.1807	1.81
4/16/2019 23:18	1.6917	1.8833	4.2833	0.975	2.7846	3	0.2051	1.7913
4/16/2019 23:19	1.6833	1.5	4.5519	1.3083	2.7793	3	0.1997	1.7966
4/16/2019 23:20	1.625	2.0333	4.2776	1.45	2.7863	3	0.1968	1.8033
4/16/2019 23:21	1.6	1.4	4.2878	1.4417	2.7734	3	0.2033	1.805
4/16/2019 23:22	1.5333	1.1333	4.2501	0.5	2.786	3	0.1833	1.8112
4/16/2019 23:23	1.5	1.1833	4.4511	1.35	2.8016	3	0.2067	1.8356
4/16/2019 23:24	1.5	1.4333	4.34	0.7	2.8046	2.9636	0.1959	1.8337
4/16/2019 23:25	1.55	1.4333	4.3821	1.0917	2.7963	2.9	0.1957	1.8178
4/16/2019 23:26	1.5917	1.6167	4.4713	0.9083	2.7893	2.9	0.2011	1.8223
4/16/2019 23:27	1.6	1.8167	4.4497	0.875	2.7971	2.9	0.1873	1.8368
4/16/2019 23:28	1.5333	1.55	4.3173	0.65	2.7954	2.9	0.2012	1.8455
4/16/2019 23:29	1.5167	1.2	4.4905	0.7	2.8064	2.8455	0.189	1.8235
4/16/2019 23:30	1.5	0.8	4.4455	1	2.8246	2.8	0.1995	1.8162
4/16/2019 23:31	1.5	0.5833	4.3482	1.3667	2.8323	2.8	0.1927	1.825
4/16/2019 23:32	1.5	1.2	4.2487	1.4417	2.8434	2.8	0.19	1.8404
4/16/2019 23:33	1.5	0.6333	4.1563	1.25	2.832	2.8	0.1995	1.8198
4/16/2019 23:34	1.5	1.2333	4.5268	1.4	2.8264	2.8	0.1872	1.8214
4/16/2019 23:35	1.5	1.7667	4.204	1.6	2.8517	2.8	0.1915	1.8273
4/16/2019 23:36	1.5	1.8333	4.1727	1.4417	2.864	2.8	0.1952	1.8433
4/16/2019 23:37	1.4833	1.3833	4.4952	1.325	2.8698	2.8	0.1916	1.8535
4/16/2019 23:38	1.4417	1.45	4.4945	1.3667	2.8635	2.8	0.1978	1.8646
4/16/2019 23:39	1.5	1.3167	4.5038	1.6417	2.8794	2.8	0.1864	1.8438
4/16/2019 23:40	1.5	1.1167	4.5362	1.3	2.8602	2.8	0.1953	1.8501
4/16/2019 23:41	1.5	1.1667	4.4292	0.6083	2.8772	2.8	0.1906	1.8344
4/16/2019 23:42	1.5583	1.4833	4.5758	1.2833	2.9078	2.8	0.1889	1.8311
4/16/2019 23:43	1.6	1.6833	4.3575	1.1917	2.8755	2.8	0.1947	1.8202
4/16/2019 23:44	1.6	0.5333	4.4747	1.125	2.8879	2.8	0.186	1.8231

4/16/2019 23:45	1.6	1.8167	4.5218	1.65	2.9062	2.8	0.2054	1.8261
4/16/2019 23:46	1.6	1.4667	4.325	0.9083	2.8923	2.8	0.1929	1.8457
4/16/2019 23:47	1.6	1.2	4.3678	1.2333	2.8736	2.8	0.1964	1.8656
4/16/2019 23:48	1.6	1.7	4.2725	1.0667	2.8923	2.8	0.1915	1.8691
4/16/2019 23:49	1.6	1.05	4.1178	0.9917	2.8805	2.8	0.1947	1.8612
4/16/2019 23:50	1.6	1.3667	4.1753	0.9833	2.8624	2.8	0.2022	1.8376
4/16/2019 23:51	1.5727	1.3333	4.221	0.7583	2.8919	2.8	0.1904	1.8373
4/16/2019 23:52	1.5083	1.9333	4.643	1.2167	2.9013	2.77	0.204	1.8368
4/16/2019 23:53	1.5	1.45	4.4408	1.3167	2.9088	2.7727	0.187	1.8317
4/16/2019 23:54	1.5	1.1167	4.1753	0.95	2.9051	2.78	0.1915	1.8438
4/16/2019 23:55	1.5167	1.4	4.2808	0.775	2.9098	2.7	0.1991	1.8618
4/16/2019 23:56	1.6	2.0667	4.3818	1.5667	2.9053	2.7	0.193	1.8637
4/16/2019 23:57	1.6	1.0333	4.3795	0.875	2.9058	2.7545	0.2017	1.8235
4/16/2019 23:58	1.5917	2.1167	4.2047	1.2083	2.8883	2.8	0.1841	1.8251
4/16/2019 23:59	1.5083	1.45	4.3773	0.725	2.8869	2.8	0.1948	1.8082
4/17/2019 0:00	1.5083	1.25	4.2667	1.4833	2.8708	2.8	0.1954	1.8188
4/17/2019 0:01	1.5417	1.4333	4.3127	1.075	2.8833	2.8	0.1904	1.8017
4/17/2019 0:02	1.6	1.7667	4.4177	1.8	2.8872	2.8	0.1929	1.8197
4/17/2019 0:03	1.6	0.7833	3.9472	1.2167	2.8713	2.87	0.1876	1.8227
4/17/2019 0:04	1.6417	0.9667	4.432	1.5417	2.8741	2.9	0.2047	1.8407
4/17/2019 0:05	1.6333	1.3167	4.5135	1.175	2.8728	2.9	0.1889	1.8229
4/17/2019 0:06	1.7	1.1167	4.3513	1.1083	2.8828	2.9	0.1821	1.7893
4/17/2019 0:07	1.6167	0.8167	4.5823	2.0083	2.8853	2.9	0.1951	1.7908
4/17/2019 0:08	1.6	0.5667	4.3328	1.05	2.8771	2.9	0.1853	1.805
4/17/2019 0:09	1.6	1.2333	4.4657	1.3833	2.8748	2.9	0.1984	1.8205
4/17/2019 0:10	1.6	1.2833	4.4532	1.1333	2.8678	2.88	0.1877	1.8416
4/17/2019 0:11	1.625	1.1833	4.2185	0.8417	2.8706	2.89	0.1947	1.8449
4/17/2019 0:12	1.6	1.2833	4.4018	1.0583	2.8713	2.8	0.2007	1.8391
4/17/2019 0:13	1.6	1.5167	4.324	1.0917	2.8585	2.8	0.1815	1.8076
4/17/2019 0:14	1.6	1.6667	4.4117	1.55	2.859	2.8	0.1981	1.7798
4/17/2019 0:15	1.6	1.5	4.4295	1.1667	2.8554	2.8	0.1906	1.8181
4/17/2019 0:16	1.55	1.2333	4.2253	0.7917	2.8617	2.8	0.1951	1.8298
4/17/2019 0:17	1.45	1.4	4.4815	1.1083	2.8633	2.8	0.1976	1.8345
4/17/2019 0:18	1.4583	1.6833	4.4998	1.35	2.8714	2.73	0.1827	1.8186
4/17/2019 0:19	1.5	1.3667	4.4112	1.125	2.8728	2.8	0.1968	1.8223
4/17/2019 0:20	1.5833	1.1333	4.4723	1	2.8481	2.8	0.1834	1.8118
4/17/2019 0:21	1.5167	1.6	4.4242	1.375	2.8556	2.8	0.1968	1.8213
4/17/2019 0:22	1.5	1.3833	4.2522	1.025	2.8716	2.8	0.1854	1.8273
4/17/2019 0:23	1.5	1.0167	4.5457	0.9833	2.8573	2.8	0.187	1.8436
4/17/2019 0:24	1.5083	1.3833	4.3838	1.25	2.8428	2.8	0.2004	1.8203
4/17/2019 0:25	1.5833	1.0333	4.5413	0.7083	2.8609	2.8	0.1899	1.812
4/17/2019 0:26	1.6	1.2333	4.3077	0.7083	2.8488	2.8	0.1925	1.8208
4/17/2019 0:27	1.6	1.3167	4.5617	1.0583	2.8596	2.8	0.1902	1.8189
4/17/2019 0:28	1.6167	1.2167	4.3097	1.1917	2.852	2.8917	0.1806	1.8206
4/17/2019 0:29	1.7	1.5167	4.3955	1.2833	2.8395	2.9	0.1968	1.846
4/17/2019 0:30	1.6083	0.9167	4.3365	1.3667	2.8279	2.9	0.1915	1.8426
4/17/2019 0:31	1.6	1.2333	4.2718	1.1417	2.8123	2.8	0.1875	1.8507

4/17/2019 0:32	1.6167	1.55	4.3852	1.675	2.8287	2.8	0.1934	1.8264
4/17/2019 0:33	1.7	1.45	4.4553	1.2167	2.8207	2.8	0.1849	1.8087
4/17/2019 0:34	1.7	0.6667	3.922	0.85	2.8058	2.8273	0.1999	1.8108
4/17/2019 0:35	1.675	1.7667	4.3812	1.7583	2.8092	2.85	0.1867	1.8098
4/17/2019 0:36	1.6	1.1667	4.3363	1.1417	2.8013	2.9	0.1919	1.8259
4/17/2019 0:37	1.6583	1.5333	4.4938	1.3667	2.789	2.9	0.1955	1.8368
4/17/2019 0:38	1.7	1.1333	4.3932	0.975	2.7906	2.91	0.1812	1.8306
4/17/2019 0:39	1.7	2.0333	4.3998	1.075	2.7815	2.95	0.1999	1.8408
4/17/2019 0:40	1.7	1.2167	4.4442	1.3167	2.7692	2.84	0.1813	1.8336
4/17/2019 0:41	1.65	1.5167	4.3002	0.7417	2.7689	2.8	0.1894	1.8202
4/17/2019 0:42	1.6	1.5333	4.5013	1.2333	2.7667	2.8	0.1907	1.8209
4/17/2019 0:43	1.5333	1.5667	4.3202	0.95	2.7591	2.9	0.1854	1.8011
4/17/2019 0:44	1.5	1.2667	4.471	0.8417	2.753	2.9	0.1962	1.8148
4/17/2019 0:45	1.5	1.4333	4.246	1.4417	2.7603	2.9	0.1839	1.8254
4/17/2019 0:46	1.5	1.3667	4.4533	1.175	2.7403	2.9	0.1853	1.8309
4/17/2019 0:47	1.5	1.3	4.6437	1.6333	2.7228	2.9	0.1931	1.7953
4/17/2019 0:48	1.6	0.7333	4.5817	0.8833	2.7161	2.8222	0.1794	1.8268
4/17/2019 0:49	1.6	1.3167	4.2118	1.4833	2.7172	2.8	0.1995	1.8253
4/17/2019 0:50	1.5083	1.8833	4.379	1.1417	2.7133	2.8	0.1819	1.8203
4/17/2019 0:51	1.5083	1.5	4.458	1.375	2.701	2.8	0.1872	1.8078
4/17/2019 0:52	1.5273	1.7167	4.7153	1.125	2.7086	2.84	0.1927	1.7998
4/17/2019 0:53	1.575	1.95	4.4325	1.1333	2.7	2.9	0.181	1.7916
4/17/2019 0:54	1.5833	1.7	4.433	1.05	2.7021	2.9	0.1923	1.8273
4/17/2019 0:55	1.5	1.3167	4.6328	0.875	2.6782	2.8273	0.1825	1.8261
4/17/2019 0:56	1.5083	1.35	4.4355	1.3333	2.689	2.8	0.1881	1.815
4/17/2019 0:57	1.525	1.4667	4.5225	1.0333	2.6703	2.8	0.1902	1.8298
4/17/2019 0:58	1.625	1.2333	4.5893	1.75	2.6597	2.8	0.1829	1.822
4/17/2019 0:59	1.7	1.4667	4.427	1.3083	2.6577	2.8	0.194	1.8443
4/17/2019 1:00	1.7	1.2	4.5511	1.3833	2.6574	2.8333	0.1817	1.8123
4/17/2019 1:01	1.7	0.9	4.5733	1.1083	2.661	2.9	0.1924	1.7967
4/17/2019 1:02	1.6833	1.6	4.4608	1.325	2.6498	2.9	0.1931	1.7921
4/17/2019 1:03	1.6	0.6667	4.3735	1.3417	2.6467	2.9	0.186	1.7872
4/17/2019 1:04	1.6583	1.75	4.5258	1.5083	2.6471	2.9	0.1963	1.8031
4/17/2019 1:05	1.7417	0.95	4.4523	1.1917	2.6482	2.9	0.183	1.7866
4/17/2019 1:06	1.7833	1.3333	4.4319	1.3583	2.6354	2.9	0.1854	1.8202
4/17/2019 1:07	1.725	0.85	4.4218	1.025	2.6453	3	0.1829	1.8033
4/17/2019 1:08	1.7	1.8667	4.5357	1.15	2.6454	3	0.1804	1.7913
4/17/2019 1:09	1.7	1.4667	4.6078	1.3083	2.6526	3.0364	0.1927	1.8058
4/17/2019 1:10	1.7167	1.6167	4.4787	1.3083	2.6683	3.1	0.1805	1.7923
4/17/2019 1:11	1.775	1.5833	4.4299	1.5667	2.674	3.1111	0.1871	1.8293
4/17/2019 1:12	1.8	1.2	4.5193	1.825	2.6917	3.2	0.1868	1.8376
4/17/2019 1:13	1.8	1.2167	4.5088	1.525	2.6963	3.2	0.1816	1.8191
4/17/2019 1:14	1.8	1.7833	4.6193	1.6417	2.7079	3.2083	0.1902	1.8129
4/17/2019 1:15	1.7083	1.4167	4.4293	1	2.711	3.3	0.178	1.8328
4/17/2019 1:16	1.7	1.45	4.4583	1.3667	2.7163	3.3	0.1965	1.8044
4/17/2019 1:17	1.7	1.45	4.4052	1.2583	2.7217	3.25	0.1832	1.8105
4/17/2019 1:18	1.7	1.45	4.53	2.0167	2.7328	3.2	0.1815	1.8153

4/17/2019 1:19	1.725	1.6	4.3225	1.6833	2.7452	3.2	0.1953	1.7891
4/17/2019 1:20	1.7083	1.4	4.6587	1.05	2.7214	3.15	0.1817	1.7916
4/17/2019 1:21	1.7167	1.9333	4.0055	1.4167	2.723	3.1	0.1945	1.8063
4/17/2019 1:22	1.75	1.1	4.2806	1.4167	2.7466	3.1	0.1867	1.7974
4/17/2019 1:23	1.7167	2.0167	4.6095	1.3083	2.7423	3.0364	0.1865	1.8037
4/17/2019 1:24	1.7417	1.6167	4.5393	1.3833	2.7523	2.92	0.1917	1.8013
4/17/2019 1:25	1.75	1.2	4.3086	0.9167	2.7656	2.9	0.1754	1.8118
4/17/2019 1:26	1.8	1.5667	4.2956	1.6083	2.7586	2.9	0.1992	1.7923
4/17/2019 1:27	1.7		4.4507	1.5167	2.7917	2.9	0.1805	1.7835
4/17/2019 1:28	1.625		4.4888	1.2833	2.7948	2.91	0.1879	1.7857
4/17/2019 1:29	1.6	1.15	4.3317	1.4	2.7926	3	0.194	1.8081
4/17/2019 1:30	1.6		4.4444	1.55	2.7908	3	0.1835	1.8212
4/17/2019 1:31	1.6417		4.539	1.4833	2.787	3	0.1938	1.7886
4/17/2019 1:32	1.7		4.4923	0.8667	2.7757	3.0364	0.184	1.7973
4/17/2019 1:33	1.7		4.1588	1.65	2.8158	3.0333	0.1908	1.7795
4/17/2019 1:34	1.7		4.4002	1.325	2.8149	3	0.1894	1.7905
4/17/2019 1:35	1.7		4.3335	1.4083	2.7721	3.0667	0.1777	1.7846
4/17/2019 1:36	1.7		4.4452	1.8917	2.7775	3.1	0.1934	1.7857
4/17/2019 1:37	1.7083		4.1714	1.1333	2.8063	3.0091	0.1804	1.8119
4/17/2019 1:38	1.7		4.0778	1.8583	2.8119	3.1	0.1848	1.7939
4/17/2019 1:39	1.6917		4.3111	1.1583	2.815	3.1556	0.1905	1.7943
4/17/2019 1:40	1.6833		4.4073	1.5833	2.8239	3.625	0.1813	1.8235
4/17/2019 1:41	1.7		4.2462	0.9667	2.8052	3.93	0.1896	1.8393
4/17/2019 1:42	1.6417		4.3438	0.7333	2.79	3.975	0.1735	1.8127
4/17/2019 1:43	1.6		4.3223	1.4833	2.8184	4	0.1955	1.813
4/17/2019 1:44	1.6		4.4308	1.5	2.8133	4	0.1873	1.8138
4/17/2019 1:45	1.7		4.3263	1.3	2.8132	3.94	0.1786	1.799
4/17/2019 1:46	1.7		4.4763	1.3417	2.7946	4	0.1946	1.7754
4/17/2019 1:47	1.7333		4.2463	1.1417	2.8218	4	0.1763	1.7905
4/17/2019 1:48	1.8		4.2771	1.0333	2.8393	3.96	0.1851	1.7982
4/17/2019 1:49	1.8		4.4031	0.6833	2.8099	3.99	0.1869	1.8079
4/17/2019 1:50	1.8	1.3143	4.3838	1.1	2.79	3.9167	0.181	1.8326
4/17/2019 1:51	1.8	1.22	4.6122	1.15	2.8152	3.94	0.19	1.8418
4/17/2019 1:52	1.8	1.5167	4.3343	1.5417	2.798	3.5833	0.1728	1.8463
4/17/2019 1:53	1.7083	1.825	4.2746	0.8833	2.799	3.1111	0.1927	1.8163
4/17/2019 1:54	1.7	0.9	4.3528	1.2	2.8138	3	0.1837	1.789
4/17/2019 1:55	1.7	0.8583	4.2416	1.3583	2.806	3	0.1886	1.8232
4/17/2019 1:56	1.7	1.2833	4.431	1.0917	2.8017	3	0.1962	1.8284
4/17/2019 1:57	1.65	1.1667	4.0841	1.0083	2.792	3	0.1786	1.8183
4/17/2019 1:58	1.6583	1.525	4.4518	0.8417	2.7953	2.9182	0.1877	1.8463
4/17/2019 1:59	1.7	0.8917	4.4108	0.7917	2.8029	2.9182	0.1824	1.8284
4/17/2019 2:00	1.7	1.5	4.4878	0.675	2.8099	3	0.1799	1.7808
4/17/2019 2:01	1.675	1.5167	4.3893	0.9583	2.7938	3	0.1922	1.7958
4/17/2019 2:02	1.6364	0.9917	4.3584	1.6083	2.8208	3	0.1783	1.818
4/17/2019 2:03	1.6	1.375	4.0043	1.6167	2.7973	2.9083	0.1859	1.8157
4/17/2019 2:04	1.6	1.5917	4.101	0.9	2.8218	2.9455	0.187	1.8273
4/17/2019 2:05	1.5417	1.5917	4.1909	1.1917	2.8265	2.94	0.1804	1.8135

4/17/2019 2:06	1.5083	1.275	4.0486	0.8917	2.8339	2.9	0.1884	1.7962
4/17/2019 2:07	1.5	1.1583	4.0274	1	2.8178	2.9	0.1808	1.8235
4/17/2019 2:08	1.5	0.6583	4.1786	1.475	2.8458	2.9	0.1897	1.8268
4/17/2019 2:09	1.5	1.2333	3.9003	0.8833	2.8533	2.9	0.1864	1.8284
4/17/2019 2:10	1.5333	1	4.0446	1.1083	2.8437	2.96	0.1799	1.8247
4/17/2019 2:11	1.5	1.4083	4.0563	1.2667	2.8673	3.1	0.1893	1.7855
4/17/2019 2:12	1.45	1.4333	3.8182	0.8083	2.8603	3.05	0.175	1.7931
4/17/2019 2:13	1.4083	1.6417	4.4013	1.1417	2.874	3.03	0.1875	1.8043
4/17/2019 2:14	1.4167	1.3333	4.3605	1.275	2.8967	3.1	0.1785	1.8001
4/17/2019 2:15	1.4917	1.6417	4.218	0.9	2.8975	3.1	0.1846	1.7897
4/17/2019 2:16	1.425	0.9833	4.2041	0.6167	2.904	3.0778	0.1916	1.8168
4/17/2019 2:17	1.3917	1.1333	4.0149	1.2667	2.905	3	0.1767	1.8309
4/17/2019 2:18	1.3	1.6167	3.7353	1	2.8862	3.04	0.188	1.8175
4/17/2019 2:19	1.3	1.4917	4.0933	0.5833	2.8908	3.1	0.1783	1.7963
4/17/2019 2:20	1.3	1.5833	4.1263	1.2333	2.9022	3.1	0.1866	1.8023
4/17/2019 2:21	1.3917	1.4083	4.223	1.4583	2.9134	3.1	0.1842	1.7843
4/17/2019 2:22	1.4	1.4917	3.9737	0.85	2.9071	3.0364	0.1781	1.8138
4/17/2019 2:23	1.45	0.825	4.2504	1.075	2.9238	2.9	0.1907	1.7998
4/17/2019 2:24	1.4	1.45	4.0143	1.1583	2.9058	2.8417	0.1787	1.7997
4/17/2019 2:25	1.4	1.375	4.2019	1.1417	2.9193	2.8667	0.1822	1.8085
4/17/2019 2:26	1.4	1.6917	4.3203	1.05	2.9079	2.825	0.184	1.8103
4/17/2019 2:27	1.4167	0.825	4.3969	1.3417	2.924	2.8	0.1786	1.8124
4/17/2019 2:28	1.475	1.2083	4.2343	1.25	2.9162	2.8	0.1901	1.8291
4/17/2019 2:29	1.475	1.175	4.1753	1.325	2.9075	2.8	0.1746	1.8162
4/17/2019 2:30	1.425	-0.2667	3.9902	1.1333	2.8893	2.8	0.1845	1.8118
4/17/2019 2:31	1.4	0.9583	4.196	1.275	2.9285	2.7182	0.1852	1.7852
4/17/2019 2:32	1.4909	1.3083	4.2067	1.5667	2.9167	2.7	0.1821	1.7639
4/17/2019 2:33	1.4417	1.0417	4.0682	1.6667	2.9167	2.7	0.187	1.77
4/17/2019 2:34	1.45	1.7	4.1337	1.3417	2.9353	2.7	0.1721	1.7742
4/17/2019 2:35	1.475	1.3667	4.216	1.25	2.9391	2.7	0.189	1.8255
4/17/2019 2:36	1.55	0.9583	3.9767	1.0417	2.9258	2.7	0.1763	1.8254
4/17/2019 2:37	1.5167	1.1	4.1488	1.1333	2.9257	2.7	0.18	1.8163
4/17/2019 2:38	1.5	0.625	3.9555	1.2833	2.9205	2.7	0.1861	1.7954
4/17/2019 2:39	1.4333	0.575	3.9682	1.3167	2.9053	2.7	0.1784	1.7933
4/17/2019 2:40	1.4917	1.0083	4.2388	0.65	2.8958	2.7	0.1882	1.8024
4/17/2019 2:41	1.4833	1.3917	4.1355	0.7083	2.9016	2.7	0.1782	1.8046
4/17/2019 2:42	1.5	1.15	4.0217	0.825	2.8934	2.7	0.181	1.7985
4/17/2019 2:43	1.5	0.3167	4.2558	1.05	2.9083	2.73	0.1863	1.8035
4/17/2019 2:44	1.5	0.95	4.1565	1.0083	2.8992	2.7333	0.1732	1.7902
4/17/2019 2:45	1.5667	1.325	3.9583	0.25	2.9316	2.7	0.1867	1.7784
4/17/2019 2:46	1.575	1.2583	3.9988	1.0667	2.9046	2.7	0.1814	1.7763
4/17/2019 2:47	1.5	1.1083	3.8647	1.9583	2.8864	2.7	0.1806	1.7748
4/17/2019 2:48	1.5	1.0833	3.7825	1.3	2.9026	2.7545	0.1899	1.7988
4/17/2019 2:49	1.4083	0.9	3.838	0.9167	2.8884	2.8	0.1785	1.8305
4/17/2019 2:50	1.4	1.475	4.0143	0.9667	2.8827	2.825	0.1892	1.7958
4/17/2019 2:51	1.4	0.9083	4.311	1.3083	2.8814	3.0091	0.1773	1.8024
4/17/2019 2:52	1.4	1.3417	4.0397	1.5417	2.8976	3.5083	0.1789	1.8127

4/17/2019 2:53	1.4583	1.4833	4.2922	0.65	2.8843	3.8909	0.1838	1.8137
4/17/2019 2:54	1.5	0.5417	4.0635	0.9917	2.887	3.9	0.1714	1.8051
4/17/2019 2:55	1.5	1.475	3.9943	0.9917	2.8692	3.9	0.1845	1.7941
4/17/2019 2:56	1.4833	1.25	4.2235	1.0083	2.8773	3.9	0.1736	1.7729
4/17/2019 2:57	1.4167	1.375	4.114	1.0333	2.8613	3.9	0.1841	1.7818
4/17/2019 2:58	1.4833	1.1	4.183	1.1667	2.865	3.9	0.1865	1.7779
4/17/2019 2:59	1.5	0.925	4.2627	1.0667	2.8697	4.17	0.1771	1.8068
4/17/2019 3:00	1.5	1.2083	4.2905	1.3083	2.8622	4.1636	0.1927	1.8008
4/17/2019 3:01	1.4667	1.35	4.122	1.0167	2.8667	4.1182	0.179	1.7624
4/17/2019 3:02	1.475	0.5167	3.9837	0.7833	2.8557	4.1545	0.1861	1.7533
4/17/2019 3:03	1.4083	1.25	4.2423	1.025	2.8483	4.0273	0.1837	1.7871
4/17/2019 3:04	1.4	0.8667	4.1992	1.0083	2.8453	3.64	0.1771	1.8103
4/17/2019 3:05	1.4	1.075	3.965	1.1833	2.8524	3.11	0.1848	1.8063
4/17/2019 3:06	1.4	1.6167	3.973	0.6917	2.8476	3.0111	0.1762	1.7878
4/17/2019 3:07	1.4	1.2833	4.0295	0.95	2.8535	3.18	0.182	1.7734
4/17/2019 3:08	1.4	1.1	3.9992	0.8167	2.8423	3.2	0.1882	1.7404
4/17/2019 3:09	1.4	1.0167	4.1507	0.5833	2.826	3.2	0.1722	1.7514
4/17/2019 3:10	1.4	0.4667	3.9567	1.1	2.8342	3.2	0.1884	1.787
4/17/2019 3:11	1.4	1.525	4.1633	0.8583	2.8406	3.0818	0.1748	1.7945
4/17/2019 3:12	1.325	1.3417	4.0562	1.2	2.8337	3.02	0.1866	1.7998
4/17/2019 3:13	1.3417	1.2917	4.169	0.5417	2.8276	3.1	0.1857	1.7731
4/17/2019 3:14	1.3	0.8667	3.954	0.65	2.8078	3.0182	0.1752	1.783
4/17/2019 3:15	1.3583	0.9167	4.1947	0.95	2.8087	3	0.1895	1.7928
4/17/2019 3:16	1.3167	1.3167	3.895	0.65	2.833	3	0.1728	1.8121
4/17/2019 3:17	1.3	1.3917	4.1162	1.3333	2.836	3.06	0.183	1.7981
4/17/2019 3:18	1.3083	1.4083	4.2763	1.1583	2.8371	3.1	0.1854	1.7948
4/17/2019 3:19	1.3833	1.425	4.0727	0.8667	2.8437	3.0091	0.1782	1.8048
4/17/2019 3:20	1.4	0.9083	3.8418	0.8833	2.8677	2.9636	0.1921	1.7826
4/17/2019 3:21	1.35	1.025	4.1365	0.5917	2.8691	2.9	0.175	1.7778
4/17/2019 3:22	1.3833	1.4917	4.5567	1.5833	2.9046	2.9	0.1885	1.786
4/17/2019 3:23	1.4	1.4	4.1722	1.025	2.8936	2.9	0.1807	1.8087
4/17/2019 3:24	1.4	1.3	3.8408	0.5833	2.9053	2.9	0.179	1.8153
4/17/2019 3:25	1.375	1.0417	3.8285	0.2917	2.9211	2.9	0.1839	1.8208
4/17/2019 3:26	1.3	0.8583	3.9125	0.95	2.9267	2.9545	0.1765	1.7864
4/17/2019 3:27	1.3167	0.5167	4.3018	1.2583	2.9813	3.1091	0.187	1.7922
4/17/2019 3:28	1.3083	1.1583	3.8603	1.0333	2.9622	3.3	0.1794	1.8104
4/17/2019 3:29	1.3583	0.85	4.0822	0.925	3.0383	3.3	0.1782	1.7861
4/17/2019 3:30	1.4	1.6833	3.9943	0.8667	3.079	3.3	0.1881	1.7705
4/17/2019 3:31	1.4	0.6167	4.3678	0.1667	3.0411	3.3818	0.1756	1.7827
4/17/2019 3:32	1.45	0.725	3.6942	0.9417	3.0479	3.5	0.188	1.8025
4/17/2019 3:33	1.4	0.8	3.8832	1.1167	3.0742	3.5	0.1795	1.8058
4/17/2019 3:34	1.4083	1.375	3.7763	0.8083	3.1043	3.5	0.1784	1.7618
4/17/2019 3:35	1.4333	0.85	4.0913	0.6083	3.094	3.6	0.1857	1.7748
4/17/2019 3:36	1.4083	0.8667	4.2487	0.8167	3.1001	3.8	0.1709	1.7916
4/17/2019 3:37	1.4	1.0167	3.8892	0.9917	3.1318	3.8	0.1894	1.7684
4/17/2019 3:38	1.4	1.2167	3.9907	1.5917	3.1138	3.74	0.1734	1.7614
4/17/2019 3:39	1.4	0.65	3.9273	0.5083	3.1447	3.56	0.1805	1.7516

4/17/2019 3:40	1.3833	0.6667	3.8068	0.6417	3.1141	3.47	0.1852	1.7666
4/17/2019 3:41	1.325	0.7917	4.0077	0.925	3.1098	3.53	0.1735	1.8228
4/17/2019 3:42	1.3727	0.6917	3.9223	1.0833	3.2272	3.6	0.1863	1.7798
4/17/2019 3:43	1.3417	1.1333	3.9507	0.725	3.1636	3.5545	0.1759	1.7826
4/17/2019 3:44	1.3	1.15	3.8678	0.4333	3.1893	3.4	0.1848	1.7933
4/17/2019 3:45	1.3	0.8833	4.0693	0.4167	3.2093	3.4	0.1873	1.7783
4/17/2019 3:46	1.3	0.6167	4.0165	0.8583	3.2041	3.4	0.1754	1.7673
4/17/2019 3:47	1.3667	1.0417	4.2735	1.025	3.2133	3.38	0.1926	1.779
4/17/2019 3:48	1.3	1.05	4.0088	1.5917	3.2334	3.2091	0.1808	1.7778
4/17/2019 3:49	1.3	1.125	3.8227	1.0583	3.2136	3.1727	0.1804	1.7663
4/17/2019 3:50	1.3	1.125	3.9585	0.8333	3.1945	3.14	0.1869	1.7814
4/17/2019 3:51	1.3	0.6	4.3668	0.625	3.2151	3.2	0.1714	1.7893
4/17/2019 3:52	1.2917	1.0167	3.9433	0.8	3.2154	3.28	0.1896	1.8247
4/17/2019 3:53	1.2167	0.7333	3.8693	1.15	3.2376	3.3909	0.1848	1.7905
4/17/2019 3:54	1.3	1.1167	3.7895	0.5917	3.267	3.3	0.181	1.7583
4/17/2019 3:55	1.3	1.0333	3.8858	0.7417	3.2168	3.3	0.1876	1.7744
4/17/2019 3:56	1.3	1.3833	4.0002	0.875	3.2147	3.3	0.175	1.7794
4/17/2019 3:57	1.3	1.1333	4.0633	0.85	3.2155	3.3	0.183	1.7823
4/17/2019 3:58	1.3	0.3167	3.7708	0.5	3.2199	3.3	0.1824	1.7564
4/17/2019 3:59	1.2917	0.95	3.8538	0.7917	3.2373	3.33	0.185	1.7446
4/17/2019 4:00	1.3	1.025	4.0883	0.675	3.2242	3.4667	0.1853	1.7659
4/17/2019 4:01	1.2333	1.1083	3.9188	0.95	3.2088	3.5	0.1763	1.7796
4/17/2019 4:02	1.2	1.4333	4.0098	0.925	3.2348	3.5545	0.1867	1.7525
4/17/2019 4:03	1.2	0.7167	4.0738	1.1917	3.2303	3.6	0.1771	1.7843
4/17/2019 4:04	1.225	0.325	3.8233	0.45	3.2058	3.4917	0.1779	1.8101
4/17/2019 4:05	1.3	1.2167	4.0229	1.2583	3.1992	3.3	0.1869	1.8058
4/17/2019 4:06	1.3	1.3333	4.2658	0.925	3.2113	3.3	0.1722	1.8156
4/17/2019 4:07	1.3917	1.0333	4.2219	0.55	3.2097	3.3	0.1833	1.7869
4/17/2019 4:08	1.3167	0.8167	3.6683	0.95	3.1972	3.45	0.1807	1.8104
4/17/2019 4:09	1.3	0.4667	3.2714	1	3.2209	3.5	0.1779	1.7848
4/17/2019 4:10	1.3083	1.0417	3.9216	0.6833	3.2016	3.5	0.185	1.7814
4/17/2019 4:11	1.3833	0.8917	3.8203	1.2083	3.2178	3.5	0.1669	1.7983
4/17/2019 4:12	1.4	2.225	3.8553	0.8417	3.2016	3.36	0.1871	1.8208
4/17/2019 4:13	1.4	0.9417	3.7998	1.3333	3.2097	3.32	0.1731	1.7989
4/17/2019 4:14	1.4	0.4583	3.9164	0.6417	3.1874	3.4	0.1821	1.8025
4/17/2019 4:15	1.4	0.9	4.1371	0.9	3.1879	3.45	0.1912	1.7849
4/17/2019 4:16	1.3917	1.325	4.0653	1.1583	3.1795	3.5556	0.1779	1.7764
4/17/2019 4:17	1.3917	1.175	4.0707	0.6833	3.1838	3.53	0.1889	1.7683
4/17/2019 4:18	1.375	1.5917	3.8826	0.8833	3.177	3.5	0.1842	1.7713
4/17/2019 4:19	1.3	1.6083	4.0907	0.95	3.1754	3.5273	0.1836	1.7886
4/17/2019 4:20	1.3	1.2583	3.7018	0.7167	3.1566	3.48	0.1909	1.8069
4/17/2019 4:21	1.3	1.1583	4.0178	0.5583	3.1731	3.4	0.1703	1.8173
4/17/2019 4:22	1.2583	0.4917	3.9771	0.7167	3.1701	3.325	0.1876	1.8088
4/17/2019 4:23	1.2	0.8417	4.1347	0.4917	3.1689	3.38	0.182	1.77
4/17/2019 4:24	1.2	0.7917	4.0548	1.5417	3.1583	3.44	0.1796	1.7766
4/17/2019 4:25	1.2333	1.0333	4.1321	1.025	3.1653	3.57	0.186	1.7609
4/17/2019 4:26	1.25	1.0667	3.9162	0.5167	3.1806	3.5818	0.1772	1.7726

4/17/2019 4:27	1.25	1.5583	4.1153	1.0833	3.1491	3.59	0.1865	1.763
4/17/2019 4:28	1.2833	1.575	4.1458	0.7333	3.1575	3.4727	0.1778	1.7811
4/17/2019 4:29	1.2333	1.575	3.9427	0.4583	3.1511	3.41	0.1782	1.807
4/17/2019 4:30	1.2833	1.3833	3.9966	0.3667	3.1661	3.5	0.1876	1.8123
4/17/2019 4:31	1.3	0.75	4.0126	0.7167	3.1639	3.6556	0.1754	1.8121
4/17/2019 4:32	1.3	0.8833	4.1133	1.2417	3.1627	3.6	0.1904	1.824
4/17/2019 4:33	1.3	1.3333	4.0518	0.725	3.1578	3.6917	0.1835	1.7946
4/17/2019 4:34	1.25	0.925	3.9724	0.775	3.1581	3.7	0.1793	1.8075
4/17/2019 4:35	1.3	1.0667	3.9911	1.125	3.1282	3.7	0.1923	1.8344
4/17/2019 4:36	1.2167	0.775	3.7693	0.9167	3.1449	3.75	0.1789	1.8353
4/17/2019 4:37	1.1833	0.675	4.1089	0.7917	3.1417	4.0182	0.1813	1.8258
4/17/2019 4:38	1.1	0.7917	4.1056	0.475	3.1378	4.1917	0.1884	1.8005
4/17/2019 4:39	1.1	1.175	3.9768	1.0833	3.1418	4.3	0.1833	1.8044
4/17/2019 4:40	1.125	0.8167	3.8351	1.2667	3.135	4.3545	0.1866	1.7899
4/17/2019 4:41	1.2167	1.275	3.8673	0.6833	3.1415	4.5818	0.171	1.7843
4/17/2019 4:42	1.3	1.2333	3.9264	0.8	3.1582	4.6273	0.1891	1.7757
4/17/2019 4:43	1.4	0.8083	3.8672	0.9917	3.1426	4.6	0.1853	1.7763
4/17/2019 4:44	1.3083	1.0667	3.8533	1.15	3.1354	4.7273	0.1818	1.7855
4/17/2019 4:45	1.3	1.2583	3.9866	1.2	3.1327	4.8909	0.1893	1.7978
4/17/2019 4:46	1.3	0.0917	4.1197	1.0833	3.1316	4.94	0.1704	1.8023
4/17/2019 4:47	1.3167	1.3833	3.8877	1.1167	3.1282	5.0583	0.1902	1.8013
4/17/2019 4:48	1.3417	1.2083	3.9056	1.1917	3.1283	5.1583	0.1772	1.8103
4/17/2019 4:49	1.325	0.4583	4.0753	1.1167	3.1151	5.1818	0.1788	1.8059
4/17/2019 4:50	1.3	1.3833	3.9703	0.9917	3.1157	5	0.1914	1.8234
4/17/2019 4:51	1.3	1.5833	3.7059	1.1167	3.1193	5.0455	0.1774	1.8008
4/17/2019 4:52	1.275	0.65	3.8784	1.45	3.108	5.19	0.1908	1.7808
4/17/2019 4:53	1.3	0.5083	4.1603	1.0917	3.1183	5.0818	0.1792	1.7831
4/17/2019 4:54	1.3	0.675	3.832	1.1083	3.0989	5.1455	0.1804	1.7913
4/17/2019 4:55	1.3	1.4833	3.8104	1.0417	3.1101	5.2	0.184	1.8313
4/17/2019 4:56	1.2833	0.9	3.899	1.175	3.1023	5.12	0.1754	1.8107
4/17/2019 4:57	1.2	0.8833	3.9674	1.2083	3.1145	5.2	0.1905	1.7875
4/17/2019 4:58	1.2	1.575	4.0481	0.8	3.108	5.5273	0.1777	1.7943
4/17/2019 4:59	1.2	1.2583	3.7511	0.8917	3.1038	5.5	0.1827	1.8265
4/17/2019 5:00	1.175	1.2	3.6465	1.3333	3.0996	5.6273	0.1849	1.8294
4/17/2019 5:01	1.1	0.7917	3.7373	1.2	3.1263	5.5444	0.1762	1.8364
4/17/2019 5:02	1.1	1.2333	3.8635	0.65	3.1391	5.72	0.1854	1.807
4/17/2019 5:03	1.1	0.4917	4.0326	1.0667	3.1918	5.86	0.1676	1.8248
4/17/2019 5:04	1.1	0.9417	3.8116	1.2	3.1791	5.9	0.1735	1.8123
4/17/2019 5:05	1.0833	0.8417	3.6637	1.0333	3.2317	5.9	0.181	1.787
4/17/2019 5:06	1	0.7167	3.6698	0.3917	3.26	5.7636	0.1634	1.794
4/17/2019 5:07	1	0.7167	3.6553	0.675	3.2742	5.5727	0.1773	1.7868
4/17/2019 5:08	0.975	0.65	3.5753	0.375	3.2473	5.6	0.1708	1.7696
4/17/2019 5:09	1	0.85	3.8296	0.6083	3.3504	5.4273	0.1699	1.772
4/17/2019 5:10	1	1.25	3.7193	1.1167	3.3207	4.99	0.1769	1.7735
4/17/2019 5:11	1	1.1583	3.6549	0.0667	3.3514	5.0909	0.1549	1.7529
4/17/2019 5:12	1.0167	0.1417	3.566	0.8083	3.3125	5.1	0.1678	1.783
4/17/2019 5:13	1.1	0.725	3.6973	-0.0583	3.3923	5.075	0.1618	1.7787

4/17/2019 5:14	1.15	0.5083	3.6052	0.9333	3.4303	5	0.1556	1.7522
4/17/2019 5:15	1.1833	1.1667	3.6111	0.3083	3.4823	4.9	0.1649	1.7448
4/17/2019 5:16	1.1583	0.25	3.3627	-0.0667	3.4218	5	0.1613	1.7291
4/17/2019 5:17	1.1667	0.825	3.5778	0.5167	3.4618	5	0.1614	1.7186
4/17/2019 5:18	1.1917	0.3	3.3283	0.4917	3.4462	5	0.1586	1.7315
4/17/2019 5:19	1.2	0.625	3.4418	0.7333	3.455	4.925	0.1527	1.7243
4/17/2019 5:20	1.1	0.7583	3.7951	0.3583	3.4573	4.9	0.1642	1.7196
4/17/2019 5:21	1.1	1.0833	3.6081	1.075	3.505	4.9	0.1474	1.7096
4/17/2019 5:22	1.1	0.65	3.6984	0.675	3.4468	4.9	0.1541	1.7538
4/17/2019 5:23	1.1167	0.45	3.6974	-0.25	3.4868	4.8667	0.1637	1.768
4/17/2019 5:24	1.1917	0.475	3.6833	0.525	3.4939	4.7636	0.1481	1.7506
4/17/2019 5:25	1.2	0.1917	3.4664	0.625	3.5831	4.8	0.154	1.7559
4/17/2019 5:26	1.2	0.2333	3.6856	0.2667	3.6208	4.6	0.1616	1.719
4/17/2019 5:27	1.2	0.7333	3.7838	0.725	3.5888	4.38	0.1478	1.7365
4/17/2019 5:28	1.1583	1.4	3.246	0.6	3.566	4.0091	0.1609	1.7407
4/17/2019 5:29	1.1	0.3333	3.2891	0.4417	3.6072	4.0182	0.1551	1.7258
4/17/2019 5:30	1.1	0.825	3.6415	0.575	3.6308	4.3083	0.1471	1.7303
4/17/2019 5:31	1.0583	0.3667	3.6257	0.375	3.6098	4.6727	0.161	1.7294
4/17/2019 5:32	1	0.95	3.769	0.5667	3.5903	4.7	0.1527	1.7493
4/17/2019 5:33	0.9083	0.1083	3.6197	0.7833	3.5191	4.8091	0.1587	1.7738
4/17/2019 5:34	0.9	0.075	3.4922	0.4	3.5872	4.9727	0.1592	1.7661
4/17/2019 5:35	0.9	0.7333	3.588	0.625	3.5621	4.9091	0.1524	1.7553
4/17/2019 5:36	0.9	0.2833	3.6233	0.125	3.5938	4.9182	0.1652	1.769
4/17/2019 5:37	0.8	0.4583	3.529	0.15	3.5803	4.9833	0.154	1.7584
4/17/2019 5:38	0.8	0.2917	3.4262	0.075	3.5821	5.2818	0.1581	1.7717
4/17/2019 5:39	0.8	0.5333	3.6212	0.0917	3.6354	5.4917	0.1695	1.7865
4/17/2019 5:40	0.8	0.1083	3.8382	0.4167	3.6361	5.975	0.1516	1.7807
4/17/2019 5:41	0.8	0.1833	3.5063	-0.0667	3.5756	6.4091	0.1678	1.755
4/17/2019 5:42	0.7833	-0.1333	3.4388	0.3417	3.6218	6.63	0.1659	1.7729
4/17/2019 5:43	0.7917	0.2667	3.7732	0.325	3.6589	6.2909	0.1595	1.7692
4/17/2019 5:44	0.8	1.1333	3.4702	0.3833	3.701	6.4818	0.1776	1.7778
4/17/2019 5:45	0.7417	0.55	3.752	0.1917	3.6581	6.5583	0.1661	1.8002
4/17/2019 5:46	0.7	-0.2667	3.6908	0.2	3.6895	6.3545	0.1741	1.8201
4/17/2019 5:47	0.7417	0.1917	3.6633	0.0833	3.7461	6.3909	0.1817	1.8143
4/17/2019 5:48	0.8	-0.0417	3.7183	0.4833	3.6753	6.3455	0.1569	1.8077
4/17/2019 5:49	0.8417	0.425	3.5258	0.2917	3.7081	6.3455	0.168	1.7794
4/17/2019 5:50	0.8	1.0417	3.4695	0.7417	3.6893	6.025	0.17	1.8075
4/17/2019 5:51	0.825	0.3333	3.6872	0.225	3.7397	5.8909	0.1678	1.803
4/17/2019 5:52	0.8	0.6833	3.3825	0.0667	3.6953	5.55	0.1768	1.7901
4/17/2019 5:53	0.725	0.2333	3.5562	-0.15	3.6562	5.1909	0.173	1.8149
4/17/2019 5:54	0.7	0.2833	3.553	0.1583	3.6942	4.8455	0.1791	1.8068
4/17/2019 5:55	0.7	0.7667	3.5307	0.7333	3.7205	4.8455	0.1769	1.8331
4/17/2019 5:56	0.7	0.7417	3.3643	0.0667	3.6766	5.0083	0.1739	1.8017
4/17/2019 5:57	0.7	0.7167	3.5815	0.4	3.7717	4.7273	0.1878	1.7948
4/17/2019 5:58	0.7167	0.8417	4.3532	-0.2083	3.7471	4.9455	0.1682	1.7975
4/17/2019 5:59	0.7083	0.325	3.4127	0.6167	3.7381	5.18	0.1835	1.8268
4/17/2019 6:00	0.7	0.55	3.582	0.3917	3.7053	5.6	0.1804	1.8442

4/17/2019 6:01	0.6917	0.1	3.5478	0.1667	3.7973	5.9833	0.1748	1.7949
4/17/2019 6:02	0.7	0.3333	3.6247	0.65	3.8181	6.1	0.1845	1.8083
4/17/2019 6:03	0.7	0.1417	3.502	-0.1917	3.8067	6.1455	0.1755	1.8
4/17/2019 6:04	0.7	0.75	3.6443	-0.0583	3.797	6.175	0.1801	1.7964
4/17/2019 6:05	0.7	-0.1333	3.7155	0.475	3.7701	6.425	0.1779	1.811
4/17/2019 6:06	0.6	0.2	3.4013	0.8167	3.7509	6.5667	0.1717	1.7927
4/17/2019 6:07	0.5417	0.6833	3.6247	0.2417	3.8028	6.875	0.1833	1.8255
4/17/2019 6:08	0.6	0.1583	3.4932	0.1167	3.8509	6.7727	0.1697	1.8487
4/17/2019 6:09	0.6	0.2167	3.5053	0.3667	3.8817	7.0833	0.1826	1.8416
4/17/2019 6:10	0.6	-0.025	3.4778	0.3833	3.8106	7.4455	0.1837	1.835
4/17/2019 6:11	0.6	0.825	3.3592	0.3	3.8592	7.5	0.1743	1.8282
4/17/2019 6:12	0.6	0.6333	3.3408	-0.0167	4.0053	7.4917	0.1865	1.8001
4/17/2019 6:13	0.6	0	3.7592	0.275	4.0082	7.57	0.1705	1.7862
4/17/2019 6:14	0.7	-0.325	3.456	0.0583	4.1038	7.7545	0.185	1.7959
4/17/2019 6:15	0.7	0.3917	3.7302	0.4333	4.2117	7.9455	0.1758	1.7973
4/17/2019 6:16	0.725	0.5333	3.5147	0.5417	4.1599	8.4083	0.1779	1.8208
4/17/2019 6:17	0.7	0.0417	3.5568	0.1333	4.0884	8.9583	0.1885	1.8293
4/17/2019 6:18	0.7	0.6917	3.4653	0.25	4.1004	9.4182	0.1683	1.827
4/17/2019 6:19	0.7	0.6333	3.4937	-0.1	4.1421	9.0364	0.1841	1.8252
4/17/2019 6:20	0.7	0.475	3.2418	0.5	4.094	9.0917	0.1773	1.8524
4/17/2019 6:21	0.7417	0.8333	3.3693	0.7833	4.094	9.0583	0.1752	1.8308
4/17/2019 6:22	0.7667	0.2917	3.5167	0.7583	4.1337	9.0583	0.1835	1.8229
4/17/2019 6:23	0.8	-0.1167	3.508	0.25	4.1611	9.19	0.1758	1.8433
4/17/2019 6:24	0.8	0.25	2.7133	0.1083	4.0801	9.2333	0.1854	1.8826
4/17/2019 6:25	0.7833	0.5	3.4218	0.2833	4.1022	9.45	0.175	1.8729
4/17/2019 6:26	0.7833	-0.1417	3.3703	0.9917	4.133	9.1083	0.1743	1.8591
4/17/2019 6:27	0.725	0.1583	2.901	0.3	4.1483	8.87	0.1841	1.8506
4/17/2019 6:28	0.7417	0.65	3.6338	0.3833	4.1933	8.6545	0.1652	1.8651
4/17/2019 6:29	0.775	0	3.745	0.475	4.0445	8.3818	0.1855	1.882
4/17/2019 6:30	0.725	1.0583	3.8373	0.2583	3.9997	7.64	0.1741	1.879
4/17/2019 6:31	0.7	-0.2667	3.2922	-0.075	3.9988	7.6091	0.1823	1.879
4/17/2019 6:32	0.7	-0.5333	0.7059	-0.175	4.1012	7.4833	0.1834	1.889
4/17/2019 6:33	0.7	0.0583	3.1143	0.175	4.1863	7.5583	0.1712	1.8528
4/17/2019 6:34	0.7	0.8917	3.5063	0.6917	4.1868	7.4333	0.1828	1.8373
4/17/2019 6:35	0.7	0.5833	3.6647	0.3167	4.2385	7.65	0.1775	1.8387
4/17/2019 6:36	0.7	0.5167	3.4152	0.475	4.2098	7.45	0.174	1.8544
4/17/2019 6:37	0.7	0.3083	3.5738	0.1583	4.2258	6.83	0.1872	1.8597
4/17/2019 6:38	0.7833	0.6	3.4487	1.0083	4.1901	6.4818	0.1698	1.8528
4/17/2019 6:39	0.8	-0.1333	3.6457	1.1167	4.1598	6.8083	0.18	1.8655
4/17/2019 6:40	0.7917	0.1833	3.386	0.6167	4.1437	7.1545	0.1736	1.8541
4/17/2019 6:41	0.7167	-0.15	3.4037	0.15	4.1126	6.8909	0.1759	1.8271
4/17/2019 6:42	0.7167	0.0083	3.5138	0.2917	4.1665	6.8	0.1838	1.8084
4/17/2019 6:43	0.7917	0.675	3.455	0.95	4.1464	6.925	0.1694	1.8217
4/17/2019 6:44	0.7583	1	3.7857	0.1333	4.1711	6.97	0.1772	1.8314
4/17/2019 6:45	0.7	0.7417	3.4712	0.3	4.1422	6.775	0.1772	1.8305
4/17/2019 6:46	0.7	0.0667	3.6285	0.3167	4.1299	6.7364	0.1731	1.8363
4/17/2019 6:47	0.7	0.1083	3.3223	0.0833	4.1243	6.25	0.1851	1.8724

4/17/2019 6:48	0.7	0.4583	3.6997	0.8917	4.1242	6.5636	0.1717	1.8643
4/17/2019 6:49	0.8	0.1333	3.7257	0.175	4.1617	6.6	0.1747	1.8177
4/17/2019 6:50	0.7833	0.6583	3.0822	0.225	4.1411	6.6273	0.1814	1.871
4/17/2019 6:51	0.8	0.2833	3.1693	0.0417	4.0826	6.6	0.1705	1.8935
4/17/2019 6:52	0.8	0.3417	4.5522	0.875	4.0964	6.1	0.1784	1.9117
4/17/2019 6:53	0.8833	0.4417	3.5527	-0.075	4.1074	6	0.1738	1.8749
4/17/2019 6:54	0.9	0.4917	3.4262	0.3	4.0787	6.09	0.1697	1.8641
4/17/2019 6:55	0.8667	0.575	3.3458	-0.0333	4.0741	5.8636	0.1786	1.8913
4/17/2019 6:56	0.8	0.325	3.4123	-0.25	4.0708	5.875	0.1694	1.9009
4/17/2019 6:57	0.8	0.1833	3.4748	0.2417	4.0679	6.1273	0.1757	1.8849
4/17/2019 6:58	0.8	0.2167	3.4162	0.3333	4.069	6.2364	0.1775	1.9197
4/17/2019 6:59	0.85	0.7667	3.7981	0.4167	4.0598	6.29	0.1644	1.9135
4/17/2019 7:00	0.9	1.5333	3.7558	0.0917	4.1783	6.1083	0.1828	1.9288
4/17/2019 7:01	0.8167	0.25	6.4563	-0.2083	10.6962	7.1	0.342	1.9719
4/17/2019 7:02	0.7833	-0.475	7.5183	0.4417	5.9533	7.825	0.4175	1.9607
4/17/2019 7:03	0.6667	0.075	23.17	1.4083	11.9914	12.0636	1.5044	2.1108
4/17/2019 7:04	0.5	-0.175	44.4317	1.05	19.9498	55.2417	3.3209	2.3638
4/17/2019 7:05	0.3417	-0.0917	35.0158	0.9083	19.8977	61.2636	2.6458	2.2521
4/17/2019 7:06	0.3	-0.1083	33.78	0.15	18.9163	47.6167	2.1056	2.2229
4/17/2019 7:07	0.3	-0.1083	30.2375	0.0583	18.2657	44.3455	2.0063	2.2282
4/17/2019 7:08	0.2333	-0.3167	27.9625	0.2583	17.9096	48.1417	1.8575	2.2128
4/17/2019 7:09	0.1917	-0.0583	27.56	-0.05	17.9583	47.675	1.7615	2.2401
4/17/2019 7:10	0.1333	-0.1417	27.1742	-0.2083	18.0035	44.0727	1.6782	2.2025
4/17/2019 7:11	0.2	-0.3583	25.6775	-0.0583	17.5378	51.6833	1.5345	2.1946
4/17/2019 7:12	0.2	-0.5917	24.035	-0.1	16.8738	43.7083	1.427	2.1898
4/17/2019 7:13	0.2	-0.9583	23.3567	-0.2833	16.6248	41.0583	1.3958	2.2226
4/17/2019 7:14	0.2	0.4167	21.7658	0.025	16.0123	45.6455	1.3482	2.2139
4/17/2019 7:15	0.2	-0.6417	22.4242	-0.0083	16.1929	45.825	1.3724	2.2012
4/17/2019 7:16	0.2	-0.5333	21.77	-0.1583	16.268	47.0273	1.3743	2.2292
4/17/2019 7:17	0.2	0.125	21.4817	0.1167	16.0124	47.0727	1.3583	2.2211
4/17/2019 7:18	0.275	-0.325	20.4608	-0.15	15.7056	47.1833	1.3352	2.1853
4/17/2019 7:19	0.3	0.2167	21.3425	0.525	16.1097	47.1909	1.4025	2.2094
4/17/2019 7:20	0.3	-0.0417	20.8683	-0.3583	15.8503	46.9917	1.3644	2.2032
4/17/2019 7:21	0.2583	-0.4917	19.8517	-0.1167	15.888	46.7182	1.3233	2.1788
4/17/2019 7:22	0.2083	-0.6583	19.6967	0.1417	15.888	46.3818	1.3197	2.209
4/17/2019 7:23	0.2917	0.1333	18.97	0.1417	15.4833	46.075	1.2909	2.2368
4/17/2019 7:24	0.275	-0.25	18.685	-0.2167	14.9128	46.09	1.2988	2.2349
4/17/2019 7:25	0.2083	0.125	17.8133	-0.2917	14.6731	45.7333	1.263	2.2239
4/17/2019 7:26	0.2083	-0.3083	17.9067	0.4083	14.6149	44.8818	1.2935	2.2066
4/17/2019 7:27	0.2	-0.8167	16.8708	0.0833	14.277	44.0546	1.2593	2.1878
4/17/2019 7:28	0.2	-0.5083	17.9942	-0.125	14.59	43.1909	1.2944	2.2446
4/17/2019 7:29	0.2	-0.25	17.0392	0.2	14.2638	42.5727	1.2684	2.2472
4/17/2019 7:30	0.2	-0.2667	15.9442	-0.5833	13.7357	42.6636	1.2169	2.2493
4/17/2019 7:31	0.2833	-0.0167	15.9517	0.1083	13.6913	42.3273	1.2195	2.233
4/17/2019 7:32	0.3	-0.1333	15.6908	0	13.5437	41.6833	1.2086	2.1956
4/17/2019 7:33	0.3083	-0.2583	15.7458	0.3583	13.5729	41.04	1.2125	2.2206

4/17/2019 7:34	0.3	-0.2333	15.3258	0.0833	13.5298	40.75	1.2208	2.2064
4/17/2019 7:35	0.3	-0.0417	15.5075	-0.1083	13.4654	40.5333	1.2146	2.2175
4/17/2019 7:36	0.2583	-0.2917	14.7808	0	13.1743	40.2	1.1813	2.2171
4/17/2019 7:37	0.1917	-1.1083	14.5183	-0.2917	13.1218	39.4	1.1903	2.2493
4/17/2019 7:38	0.1333	-0.25	14.9217	-0.9333	13.2263	39.7583	1.2017	2.2413
4/17/2019 7:39	0.1	-0.325	14.645	-0.3083	13.1861	40.13	1.1988	2.2121
4/17/2019 7:40	0.1	-0.4167	14.1933	-0.4583	12.9609	40.375	1.1807	2.2241
4/17/2019 7:41	0.1	0.2583	13.4367	-0.6417	12.6481	40.35	1.1386	2.2141
4/17/2019 7:42	0.1	0	13.1567	-0.2583	12.4895	40.1083	1.1316	2.1912
4/17/2019 7:43	0.1	0.225	13.3375	-0.1833	12.5859	39.15	1.1565	2.2223
4/17/2019 7:44	0.1	0.0167	12.8775	-0.675	12.2896	38.7333	1.1138	2.2234
4/17/2019 7:45	0.1	-0.3917	12.4842	-0.4333	12.1343	39.13	1.1077	2.1744
4/17/2019 7:46	0.075	0.2417	12.935	-0.6083	12.2829	39.3583	1.1258	2.1828
4/17/2019 7:47	0	-0.3083	13.6292	-0.7417	12.4217	39.3091	1.1574	2.1973
4/17/2019 7:48	0	-0.125	12.1583	-0.1917	12.0603	38.775	1.1109	2.1858
4/17/2019 7:49	0	-0.0417	11.9725	-0.2	11.8891	38.56	1.075	2.184
4/17/2019 7:50	0	-0.4917	12.2908	-0.225	12.0168	38.5818	1.1063	2.22
4/17/2019 7:51	0	-0.7417	12.6133	-0.3417	11.9948	38.46	1.1079	2.2508
4/17/2019 7:52	0	-0.55	11.9808	-0.5167	11.7873	38.425	1.0771	2.2343
4/17/2019 7:53	0	-0.3917	11.3025	-0.3583	11.7333	38.6091	1.0907	2.2348
4/17/2019 7:54	0	-0.2833	11.9042	-0.2083	11.7408	38.35	1.079	2.2543
4/17/2019 7:55	0.05	-0.6917	10.945	-0.525	11.4294	38.17	1.0441	2.2353
4/17/2019 7:56	0.1	-1.0417	11.1492	-0.4	11.4206	38.6333	1.0642	2.2306
4/17/2019 7:57	0.1	-1.0417	11.0158	-0.525	11.3493	38.7818	1.0486	2.2278
4/17/2019 7:58	0.1	-0.7583	10.9042	-0.4417	11.2982	38.4182	1.0462	2.2051
4/17/2019 7:59	0.1	-0.1833	10.7242	-0.85	11.308	37.83	1.0303	2.1994
4/17/2019 8:00	0.1	-0.625	11.1692	-0.125	11.2942	37.2909	1.04	2.238
4/17/2019 8:01	0.1167	0.1333	10.72	-0.1667	11.1613	37.55	1.0383	2.2129
4/17/2019 8:02	0.125	-0.8333	10.3263	-0.6917	10.9313	37.3909	1.0081	2.1867
4/17/2019 8:03	0.1	-0.1167	10.0204	-0.6667	10.9883	37.04	0.9977	2.2069
4/17/2019 8:04	0.1	-0.0583	10.0448	-0.3833	10.9638	36.92	1.0143	2.2285
4/17/2019 8:05	0.1	0.0667	10.4569	0.0583	11.0163	36.4273	1.034	2.2647
4/17/2019 8:06	0.1	-0.3	10.0346	0.0333	10.8663	35.475	0.9992	2.2435
4/17/2019 8:07	0.1	-0.3917	9.5583	-0.925	10.7478	35.075	0.9763	2.2057
4/17/2019 8:08	0.1	-0.3583	9.6263	-0.4583	10.6499	34.8	0.9834	2.2242
4/17/2019 8:09	0.1	-0.3667	9.7648	-0.725	10.7409	34.2	1.0007	2.2426
4/17/2019 8:10	0.1	-1.175	10.018	-0.7	10.7086	33.575	0.9762	2.2527
4/17/2019 8:11	0.1	-0.0083	9.4824	-0.7	10.5967	33.6917	0.9393	2.2055
4/17/2019 8:12	0.1	-0.6333	9.2255	-0.5333	10.4697	34.06	0.9552	2.1839
4/17/2019 8:13	0.1	-0.175	9.01	-0.6833	10.5065	33.4167	0.9341	2.1863
4/17/2019 8:14	0.1	0.0583	8.8021	-0.45	10.4214	32.8417	0.9497	2.1961
4/17/2019 8:15	0.1	-0.3083	8.9828	-0.3833	10.493	32.7	0.9366	2.1924
4/17/2019 8:16	0.1	-0.0667	8.6903	-0.5	10.3533	32.4667	0.9299	2.1838
4/17/2019 8:17	0.1	-0.1083	8.4718	-0.8417	10.4131	32.0546	0.928	2.2165
4/17/2019 8:18	0.1	-0.15	8.7912	-0.3667	10.3538	31.85	0.9346	2.2453
4/17/2019 8:19	0.1	0.4583	8.242	-0.2667	10.2202	31.7182	0.9159	2.2176
4/17/2019 8:20	0.1	-0.4583	7.4074	-0.375	10.2916	31.4167	0.8966	2.1811

4/17/2019 8:21	0.1	-0.6417	7.9803	-0.7333	10.1293	31.3546	0.8879	2.1759
4/17/2019 8:22	0.1	-0.7333	7.4218	-0.15	10.1733	31.2583	0.8886	2.1828
4/17/2019 8:23	0.15	0.0583	6.8734	-0.2667	10.174	30.86	0.824	2.1494
4/17/2019 8:24	0.225	-0.625	7.3247	0.3833	10.7288	30.1909	0.7985	2.1392
4/17/2019 8:25	0.4417	0.1	6.0839	0.4167	14.1566	29.2182	0.6743	2.0251
4/17/2019 8:26	0.55	0.3667	5.5891	0.05	14.34	28.7417	0.6429	1.9538
4/17/2019 8:27	0.7917	0.5833	4.5972	0.575	15.4318	27.7364	0.5814	1.8892
4/17/2019 8:28	0.95	1.0333	5.1363	0.4667	16.2148	26.0917	0.5489	1.8304
4/17/2019 8:29	1.1583	0.6417	4.841	0.7167	16.3568	24.47	0.531	1.8293
4/17/2019 8:30	1.2	1.025	4.622	0.6	16.7018	23.8083	0.5246	1.8037
4/17/2019 8:31	1.225	0.8	4.7487	0.9083	17.0158	22.9917	0.4985	1.7683
4/17/2019 8:32	1.325	1.3833	4.6177	1.6833	17.3833	21.875	0.4496	1.748
4/17/2019 8:33	1.4583	1.2917	4.4438	1.4333	17.8317	20.8833	0.4439	1.7441
4/17/2019 8:34	1.6917	1.2333	4.4657	1.8083	18.3129	19.8091	0.4128	1.7277
4/17/2019 8:35	1.8	1.15	4.1612	1.2	18.3665	18.7636	0.3883	1.6943
4/17/2019 8:36	1.8833	2.2083	3.901	1.5833	18.8938	18.5083	0.3864	1.6943
4/17/2019 8:37	1.9	1.9667	4.1653	1.5083	19.3507	18.9417	0.3649	1.6863
4/17/2019 8:38	2.0333	1.9083	4.0663	1.475	19.8486	19.03	0.3539	1.6578
4/17/2019 8:39	2.0167	1.075	4.6702	2.2167	19.9568	18.825	0.3589	1.6414
4/17/2019 8:40	2.0917	2.2917	3.8608	1.1417	20.518	19.1083	0.3293	1.6452
4/17/2019 8:41	2.0083	2.3	3.538	1.6667	21.1328	19.55	0.3255	1.6378
4/17/2019 8:42	2	1.925	3.4442	1.5417	21.4959	19.42	0.3221	1.618
4/17/2019 8:43	1.9333	2.0167	3.3365	1.8083	21.6103	19.7182	0.3066	1.6158
4/17/2019 8:44	1.9	1.4083	3.458	1.7833	21.7433	20.0182	0.3148	1.6405
4/17/2019 8:45	1.9167	1.325	3.2528	1.5083	21.812	19.8636	0.3079	1.6456
4/17/2019 8:46	1.9917	2.1167	3.3272	1.7333	22.0028	20.0083	0.2939	1.6223
4/17/2019 8:47	2	1.625	3.3318	1.6417	21.9083	20.23	0.3104	1.6208
4/17/2019 8:48	2.0417	1.5167	3.579	1.8833	21.8522	20.09	0.289	1.6009
4/17/2019 8:49	2.1	1.8	3.5585	2.0167	21.5936	19.7546	0.2893	1.5908
4/17/2019 8:50	2.1417	2.3667	3.6678	1.875	21.3193	19.4546	0.2872	1.5841
4/17/2019 8:51	2.1917	2.0083	3.4503	2.15	21.2048	19.525	0.2701	1.5531
4/17/2019 8:52	2.1417	2.0167	3.6847	1.7083	20.8035	19.4091	0.2832	1.5723
4/17/2019 8:53	2.2167	2.2	3.64	1.625	20.4574	18.7636	0.2667	1.5403
4/17/2019 8:54	2.3	1.6	3.6757	2.4583	20.2287	18.475	0.2588	1.5178
4/17/2019 8:55	2.45	1.8667	3.7232	2.0167	20.0615	18.1909	0.2625	1.5134
4/17/2019 8:56	2.575	2.4167	4.22	2.5667	19.897	17.9	0.244	1.5045
4/17/2019 8:57	2.6667	2.025	4.23	2.4167	19.4673	17.8818	0.2491	1.5288
4/17/2019 8:58	2.8	2.55	4.0763	2.275	19.1265	17.6818	0.2478	1.5115
4/17/2019 8:59	2.8417	2.4167	4.0725	2.1417	18.7908	17.4818	0.2303	1.4993
4/17/2019 9:00	2.8	1.8583	4.042	2.7667	18.6328	17.2417	0.2482	1.5587
4/17/2019 9:01	2.7	2.4167	3.7985	2.1417	18.8229	16.9182	0.2461	1.5685
4/17/2019 9:02	2.5	1.7333	3.8438	1.7083	19.0264	16.8417	0.2431	1.5556
4/17/2019 9:03	2.325	2.2	3.4713	1.625	18.8085	16.36	0.2581	1.562
4/17/2019 9:04	2.1417	1.2167	3.4748	0.8917	18.5969	15.9667	0.2443	1.5793
4/17/2019 9:05	1.9833	1.7583	3.287	1.7833	18.5947	16.2667	0.2535	1.5681
4/17/2019 9:06	1.825	1.35	3.3153	1	18.4873	16.6667	0.2594	1.5867
4/17/2019 9:07	1.7917	0.825	3.1977	1.2667	18.3743	16.8091	0.2477	1.6048

4/17/2019 9:08	1.7	1.4583	3.1312	0.8667	18.3028	17.2	0.2647	1.6037
4/17/2019 9:09	1.6333	1.4667	3.217	0.4	18.2227	17.95	0.2531	1.6124
4/17/2019 9:10	1.5	0.9917	3.2415	0.6917	17.9633	17.8818	0.2574	1.6285
4/17/2019 9:11	1.4	1.5167	2.9318	0.3167	17.6482	17.17	0.2622	1.6714
4/17/2019 9:12	1.3	0.6917	2.9752	0.4	17.6053	16.3167	0.2606	1.6688
4/17/2019 9:13	1.2545	0.5833	2.871	0.8917	17.6341	15.5667	0.2626	1.6965
4/17/2019 9:14	1.2	1.175	3.0868	0.4	17.5455	14.5583	0.2666	1.6942
4/17/2019 9:15	1.125	0.7833	2.9722	0.0333	17.4542	14.1	0.2526	1.692
4/17/2019 9:16	1.0417	0.275	4.0725	0.575	20.1175	14.175	0.3195	1.7291
4/17/2019 9:17	0.8917	1	25.44	2.2417	34.7918	45.57	0.8513	1.8553
4/17/2019 9:18	0.7167	-0.0333	20.2733	0.9667	33.5911	56.0917	0.7687	1.9113
4/17/2019 9:19	0.4833	0.15	28.4767	1.7333	49.2285	61.3818	1.5541	1.9828
4/17/2019 9:20	0.2917	0	52.9333	1.8167	54.4937	76.925	2.434	2.2053
4/17/2019 9:21	0.0833	-0.4417	91.6717	3.8917	57.376	109.6417	5.3535	2.588
4/17/2019 9:22	-0.025	-0.4833	77.75	2.2583	59.0193	112	5.7778	2.6795
4/17/2019 9:23	-0.0833	-0.8083	64.0817	1.3667	58.8373	116.5727	5.4355	2.6653
4/17/2019 9:24	-0.0167	-0.6583	57.2683	1	58.706	119.0167	5.5	2.4768
4/17/2019 9:25	0	-0.4	51.6467	0.6333	55.1194	111.8333	4.3455	2.3534
4/17/2019 9:26	0	-0.2667	51.5517	-0.25	52.8059	115.875	5.052	2.3331
4/17/2019 9:27	0	-0.5583	50.6317	0.0333	53.442	102.3636	5.1875	2.3406
4/17/2019 9:28	0	-0.4917	48.7333	0.0417	52.8147	100.8167	3.7662	2.3108
4/17/2019 9:29	0.0167	-0.9083	48.2383	0.6917	52.5917	97.2364	4.1358	2.3156
4/17/2019 9:30	0.0917	-0.75	47.7317	0.0417	52.1529	101.8667	4.115	2.324
4/17/2019 9:31	0.0917	-0.1	44.5717	0.5917	50.3105	104.0083	3.7648	2.2952
4/17/2019 9:32	0.1	-0.7333	44.8717	0.6667	50.1548	100.6182	3.8037	2.2989
4/17/2019 9:33	0.1	-1.0583	41.7517	0.4583	47.7456	98.42	3.4042	2.2733
4/17/2019 9:34	0.1667	0.1167	41.7717	0.5333	47.9272	98.6909	3.3835	2.2547
4/17/2019 9:35	0.2	0.275	42.9733	0.8917	49.0726	99.1583	3.7188	2.2717
4/17/2019 9:36	0.2	-0.4083	42.695	0.15	49.1096	99.5182	3.4415	2.295
4/17/2019 9:37	0.2	-0.7083	40.265	0.0583	47.0065	98.8917	3.2658	2.2952
4/17/2019 9:38	0.275	-0.3333	38.765	0.1	45.8708	98.2417	3.2632	2.2866
4/17/2019 9:39	0.3083	-0.7333	38.5	0.7917	45.8989	97.7333	3.335	2.3089
4/17/2019 9:40	0.3667	-1	38.3283	-0.0083	45.6353	97.2455	3.1947	2.3135
4/17/2019 9:41	0.325	-0.9167	35.8417	-0.0333	43.8443	96.4	3.0018	2.2851
4/17/2019 9:42	0.3	-0.55	37.3383	0.775	44.6816	95.6455	3.0463	2.3003
4/17/2019 9:43	0.3	-0.4917	35.7067	0.5667	43.8696	95.0364	2.9838	2.2759
4/17/2019 9:44	0.3	-0.2667	34.175	0.3167	42.322	94.0091	2.8632	2.2473
4/17/2019 9:45	0.3	0.1167	33.465	0.325	41.7789	92.8	2.8773	2.2771
4/17/2019 9:46	0.3	-0.1917	32.4767	0.0917	40.563	90.9182	2.7953	2.3091
4/17/2019 9:47	0.3	-0.6167	32.605	0.325	40.5501	89.47	2.8033	2.2998
4/17/2019 9:48	0.3	-0.25	31.865	0.0333	39.928	87.5273	2.792	2.2658
4/17/2019 9:49	0.3	0.2083	34.465	0.3333	42.1529	87.25	2.9263	2.3024
4/17/2019 9:50	0.3	-0.525	32.945	-0.5083	40.6003	86.8818	2.8567	2.3046
4/17/2019 9:51	0.3	0.05	29.8317	-0.025	37.4736	86.1455	2.6688	2.2302
4/17/2019 9:52	0.3	-0.1833	30.08	0.225	38.0726	85.175	2.7227	2.2475
4/17/2019 9:53	0.2583	-0.2417	28.73	0.1833	37.1508	84.3727	2.6932	2.2469
4/17/2019 9:54	0.2	0.1917	30.03	0.7833	37.9575	83.8083	2.7602	2.2721

4/17/2019 9:55	0.2	-0.0417	29.2717	0.1167	36.4409	82.9818	2.67	2.2858
4/17/2019 9:56	0.2	-0.1	27.9533	-0.0083	35.8798	82.0833	2.6495	2.2903
4/17/2019 9:57	0.2	-0.3167	28.6483	0.125	36.8787	81.5833	2.7313	2.2917
4/17/2019 9:58	0.1167	-0.4333	28.3217	-0.2917	36.1486	81.2636	2.6657	2.2753
4/17/2019 9:59	0.1	-0.6417	27.0333	-0.3833	34.8578	80.875	2.6058	2.2403
4/17/2019 10:00	0.1	-0.3667	26.2642	-0.45	34.1899	80.4	2.5605	2.2818
4/17/2019 10:01	0.1	-0.65	26.2567	0.2917	34.1258	79.7455	2.5995	2.2638
4/17/2019 10:02	0	-0.4333	26.1008	0.1917	33.9261	78.88	2.5788	2.2763
4/17/2019 10:03	0	-0.4417	26.1592	0.2667	33.732	78.5167	2.5727	2.2862
4/17/2019 10:04	0	-0.4417	25.1542	0.2833	33.1503	78.0818	2.5567	2.2804
4/17/2019 10:05	0.0667	-0.1833	26.3183	0.4917	33.428	77.9273	2.5787	2.315
4/17/2019 10:06	0.0917	-0.5	26.2167	-0.0083	33.459	77.675	2.6018	2.3245
4/17/2019 10:07	0.0167	-1.0917	24.5525	0.0667	32.3823	77.2417	2.5375	2.2899
4/17/2019 10:08	0	0.2083	25.085	0.225	32.2335	76.8833	2.5235	2.3059
4/17/2019 10:09	0	-0.425	24.0958	-0.8083	31.1533	76.325	2.4743	2.2835
4/17/2019 10:10	0	0.25	22.9142	-0.1833	30.4713	75.5182	2.442	2.2562
4/17/2019 10:11	0.05	-0.775	23.8283	-0.2083	31.2895	74.95	2.5027	2.2607
4/17/2019 10:12	0.1	0.15	23.0675	0.3167	30.2909	74.55	2.4258	2.29
4/17/2019 10:13	0.1	-0.0917	22.6025	0.0083	29.9579	74.0727	2.4158	2.2985
4/17/2019 10:14	0.1	-0.3083	22.4425	0.05	29.5588	73.5083	2.4148	2.2698
4/17/2019 10:15	0.1	-0.1833	23.2517	0.375	29.9518	72.9818	2.4608	2.2946
4/17/2019 10:16	0.1417	-0.25	23.1767	-0.375	30.1018	72.6833	2.4687	2.3298
4/17/2019 10:17	0.1667	-0.3083	22.4533	-0.3333	29.3583	72.275	2.4199	2.3286
4/17/2019 10:18	0.1583	-0.0667	21.5567	0.075	28.8576	71.6583	2.3775	2.3402
4/17/2019 10:19	0.2	0.0333	21.465	-0.2417	28.3872	71.1364	2.3649	2.3288
4/17/2019 10:20	0.2	-0.0833	20.8217	-0.3	27.8611	70.675	2.348	2.3201
4/17/2019 10:21	0.1583	0.0917	21.3358	-0.0833	28.3149	70.4455	2.3727	2.3458
4/17/2019 10:22	0.1	0.15	22.1458	-0.15	28.1793	70.3167	2.3834	2.332
4/17/2019 10:23	0.1	-0.6083	21.1858	-0.2833	27.9924	70.0909	2.3646	2.3285
4/17/2019 10:24	0.1	-0.6083	20.3642	-0.0083	27.4035	69.825	2.3164	2.3077
4/17/2019 10:25	0.1	-0.65	20.1842	-0.2917	27.307	69.4909	2.3023	2.2959
4/17/2019 10:26	0.0333	-0.625	20.1275	-0.1333	27.1828	69.2091	2.2699	2.3135
4/17/2019 10:27	0.025	-0.3917	19.7875	-0.5667	26.2318	68.8417	2.2649	2.3382
4/17/2019 10:28	0.0167	-0.1417	19.1633	-0.8417	25.643	68.15	2.2241	2.3193
4/17/2019 10:29	0	-0.6	19.28	-0.1667	25.5725	67.5	2.2241	2.3083
4/17/2019 10:30	-0.025	-0.025	19.7008	0.375	25.8469	67.0455	2.269	2.3054
4/17/2019 10:31	-0.1	-0.2833	19.145	0.0833	25.3181	66.7182	2.2351	2.3147
4/17/2019 10:32	-0.0667	-0.4	19.3142	-0.4083	25.6199	66.5	2.267	2.319
4/17/2019 10:33	-0.1	-0.3	19.01	-0.0667	25.2753	66.1917	2.2453	2.3456
4/17/2019 10:34	-0.0417	-0.5667	18.78	-0.2417	24.8957	65.725	2.1975	2.3562
4/17/2019 10:35	-0.0583	-0.5917	18.2092	0.2417	24.2205	65.0545	2.1829	2.3273
4/17/2019 10:36	-0.1	-0.6417	18.9433	0.0167	24.6053	64.5364	2.2159	2.3713
4/17/2019 10:37	-0.1	-0.475	17.9133	-0.4083	23.7321	64.1417	2.1343	2.3588
4/17/2019 10:38	-0.0417	-0.1667	15.6542	-0.5417	21.9354	63.4	1.9616	2.2593
4/17/2019 10:39	0.025	-0.0083	16.0075	0.075	21.698	62.35	1.8689	2.2123
4/17/2019 10:40	0.275	0.7	13.8692	0.3667	20.6827	60.9727	1.6244	2.1076
4/17/2019 10:41	0.5167	1.175	12.795	0.5917	20.3086	59.1083	1.4973	2.0487

4/17/2019 10:42	0.7833	1.1417	11.6208	1.1667	19.7957	56.8818	1.3663	1.9919
4/17/2019 10:43	0.8917	0.6583	10.3392	0.9083	18.8334	54.275	1.2568	1.9428
4/17/2019 10:44	1.0667	1.1917	9.6973	1.675	18.533	51.2818	1.1171	1.9176
4/17/2019 10:45	1.275	1.9417	8.7536	1.425	17.9481	48.0833	1.0395	1.8803
4/17/2019 10:46	1.4833	1.2333	8.5752	1.85	18.1573	44.5182	0.9805	1.855
4/17/2019 10:47	1.5917	1.7583	8.1673	1.5917	18.1754	40.9583	0.899	1.8182
4/17/2019 10:48	1.75	2.425	7.468	1.6167	17.5542	37.1364	0.8435	1.7778
4/17/2019 10:49	2.0167	3.0667	7.5673	2.7583	16.8065	33.1667	0.7492	1.7556
4/17/2019 10:50	2.2667	2.625	7.2653	3.2	16.6271	29.1556	0.7072	1.7248
4/17/2019 10:51	2.4583	2.475	6.9849	2.5667	16.2649	25.8583	0.6931	1.7403
4/17/2019 10:52	2.6667	3.5083	7.2459	3.4917	15.7361	22.6909	0.6236	1.7392
4/17/2019 10:53	2.9833	3.45	7.2803	3.475	15.1986	19.8667	0.5876	1.7283
4/17/2019 10:54	3.275	3.7917	7.3459	3.2667	15.0588	17.2167	0.584	1.7207
4/17/2019 10:55	3.3083	3.2	7.1545	2.925	15.0307	15.1727	0.5702	1.7267
4/17/2019 10:56	3.325	3.9	7.6978	3.2583	14.6196	13.3083	0.5566	1.7076
4/17/2019 10:57	3.4667	4.1167	6.8629	3.975	14.2046	11.58	0.5195	1.6734
4/17/2019 10:58	3.85	5.0333	7.5813	4.2417	13.4365	10.4167	0.4803	1.6615
4/17/2019 10:59	4.3417	6.2333	7.7613	5.5083	12.7887	9.21	0.4468	1.643
4/17/2019 11:00	4.75	5.775	7.8481	5.4	12.3148	8.2	0.4148	1.6718
4/17/2019 11:01	4.9917	5.675	7.8464	5.1833	12.0475	7.3636	0.4191	1.6498
4/17/2019 11:02	5.0083	5.2083	7.962	5.725	11.8445	6.7	0.4021	1.6605
4/17/2019 11:03	5.2583	6.2583	8.2402	6.2667	11.2888	6.0333	0.3809	1.6642
4/17/2019 11:04	5.5167	6.9417	8.5531	6.6	11.0416	5.5364	0.3676	1.626
4/17/2019 11:05	5.8	6.1167	8.4038	5.8417	10.6875	5.1083	0.343	1.619
4/17/2019 11:06	5.9333	6.225	8.8434	6.9417	10.3854	4.6909	0.3594	1.6063
4/17/2019 11:07	6.1917	6.9333	9.0024	7.2	10.0673	4.3417	0.334	1.5926
4/17/2019 11:08	6.3917	7.2	9.2101	7.7083	9.8515	4.0091	0.319	1.6019
4/17/2019 11:09	6.8167	7.8833	9.6923	7.2917	9.3683	3.7583	0.3096	1.5855
4/17/2019 11:10	7.225	8.6583	9.9874	8.4167	9.0901	3.45	0.2887	1.6172
4/17/2019 11:11	7.625	7.8333	9.8342	8.225	8.9463	3.2583	0.2977	1.6158
4/17/2019 11:12	7.8	8.7833	9.9816	8.25	8.6098	3.1182	0.2878	1.5689
4/17/2019 11:13	7.9	9.375	10.3907	8.7917	8.3688	2.9667	0.2714	1.546
4/17/2019 11:14	7.9083	8.4167	10.0143	8.0583	8.3274	2.8182	0.2865	1.5703
4/17/2019 11:15	8.1083	8.875	10.7842	8.8417	8.1367	2.7083	0.2539	1.5795
4/17/2019 11:16	8.2333	9.35	10.7717	9.025	7.871	2.6	0.2633	1.5733
4/17/2019 11:17	8.55	9.3083	11.025	8.85	7.736	2.5455	0.2495	1.57
4/17/2019 11:18	8.6167	9.05	10.8	8.7083	7.6102	2.4583	0.2487	1.5544
4/17/2019 11:19	8.8	9.475	11.7058	9.6917	7.3408	2.3545	0.2555	1.5553
4/17/2019 11:20	8.9583	9.2333	11.205	9.8667	7.1591	2.3	0.2329	1.5783
4/17/2019 11:21	9.2083	9.25	11.505	10.5	7.0691	2.2545	0.246	1.5603
4/17/2019 11:22	9.375	10.2417	12.6458	10.675	6.8587	2.2	0.2413	1.5405
4/17/2019 11:23	9.5	10.4667	11.7833	10.5167	6.6646	2.1	0.2298	1.5843
4/17/2019 11:24	9.6167	10.625	12.1608	10.7667	6.5405	2.0727	0.2439	1.5907
4/17/2019 11:25	9.7167	9.6083	11.8483	10.3	6.4243	2	0.23	1.5883
4/17/2019 11:26	9.85	9.4167	12.135	10.7833	6.436	1.9545	0.235	1.5448
4/17/2019 11:27	9.9583	10.4417	12.4392	10.8167	6.4001	1.9	0.2295	1.5581
4/17/2019 11:28	10.0667	10.4417	12.1075	10.1333	5.9253	1.86	0.2259	1.5665

4/17/2019 11:29	10.3083	11.7	12.7417	11.65	5.6831	1.8	0.2254	1.5699
4/17/2019 11:30	10.4	10.65	12.5517	11.0083	5.631	1.7273	0.2184	1.5655
4/17/2019 11:31	10.5583	10.5583	12.7333	10.675	5.5543	1.7182	0.2231	1.5548
4/17/2019 11:32	10.5083	10.8083	13.0983	11.1917	5.5024	1.7	0.2205	1.5584
4/17/2019 11:33	10.5333	11.025	12.785	11.0333	5.3817	1.7	0.2145	1.534
4/17/2019 11:34	10.6	11.1333	13.2167	11.4917	5.336	1.7	0.2202	1.5076
4/17/2019 11:35	10.75	11.1667	13.185	11.1833	5.2208	1.7	0.1996	1.5203
4/17/2019 11:36	10.8	11.5417	13.3433	11.6583	5.2513	1.7	0.213	1.5524
4/17/2019 11:37	10.7917	11.1167	12.9283	10.9	5.1428	1.6917	0.2169	1.5665
4/17/2019 11:38	10.7333	10.875	13.1067	10.825	5.0618	1.6273	0.1969	1.5278
4/17/2019 11:39	10.7333	11.05	13.175	11.3917	5.0191	1.6636	0.2123	1.5289
4/17/2019 11:40	11.0917	12.2167	13.965	12.05	4.8123	1.6	0.1992	1.5396
4/17/2019 11:41	11.2833	11.1083	13.4917	11.5167	4.7749	1.6	0.1891	1.5334
4/17/2019 11:42	11.4583	12.0333	13.6233	12.3083	4.7454	1.6	0.2004	1.5296
4/17/2019 11:43	11.35	11.8917	13.7983	12.3917	4.6822	1.5182	0.1959	1.5265
4/17/2019 11:44	11.475	12.25	13.9567	12.4583	4.7113	1.5	0.2005	1.53
4/17/2019 11:45	11.3667	12.0583	12.97	11.6333	4.9519	1.5273	0.1978	1.539
4/17/2019 11:46	11.0833	10.7083	12.45	10.7833	6.6981	1.6273	0.1932	1.529
4/17/2019 11:47	10.525	9.8667	11.705	10.0583	7.7193	1.7909	0.1988	1.5483
4/17/2019 11:48	10.1083	10.1417	11.9067	10.1417	8.1426	1.9182	0.1905	1.5451
4/17/2019 11:49	9.9	9.325	11.3133	10.4417	8.2023	2	0.1971	1.5328
4/17/2019 11:50	9.8083	10.1833	11.6783	9.5333	8.1643	2.0833	0.1927	1.5318
4/17/2019 11:51	9.8167	10.4083	11.9033	10.3917	8.3251	2.1364	0.1822	1.5164
4/17/2019 11:52	9.825	10.0167	11.9517	10.7167	8.3382	2.2	0.1974	1.5497
4/17/2019 11:53	9.8818	10.5083	11.8517	9.9083	8.3647	2.2273	0.184	1.5447
4/17/2019 11:54	9.825	10.275	11.7533	9.7	8.2825	2.3	0.1749	1.5587
4/17/2019 11:55	9.85	10.1333	11.8933	9.7167	8.1973	2.3	0.1925	1.5683
4/17/2019 11:56	9.9083	10.4667	12.09	9.9083	8.0298	2.38	0.1773	1.5568
4/17/2019 11:57	10.1917	10.775	12.3	10.7417	7.8262	2.3417	0.1951	1.5472
4/17/2019 11:58	10.425	10.8833	12.5583	10.6833	7.7322	2.2545	0.1898	1.5364
4/17/2019 11:59	10.6417	11.0667	12.3233	11.575	7.5773	2.125	0.1812	1.5275
4/17/2019 12:00	10.7917	10.9167	13.4967	10.8417	7.2793	1.9833	0.1931	1.529
4/17/2019 12:01	10.9167	10.7583	12.6317	11.0917	7.1015	1.8818	0.1826	1.5208
4/17/2019 12:02	11	11.7083	12.9417	11.425	6.9243	1.8	0.1862	1.5496
4/17/2019 12:03	11.2833	12.0833	13.865	12.675	6.6652	1.77	0.1937	1.5224
4/17/2019 12:04	11.5583	11.7083	13.7583	12.0333	6.5487	1.7	0.1763	1.5071
4/17/2019 12:05	11.8333	12.8167	13.94	12.8167	6.4078	1.7	0.1922	1.5081
4/17/2019 12:06	12.05	13.125	14.455	12.7167	6.2381	1.675	0.1837	1.5323
4/17/2019 12:07	12.0167	11.3083	13.3333	11.8833	6.2876	1.6333	0.1799	1.5433
4/17/2019 12:08	12.1417	13.1667	14.4583	13.2917	6.2245	1.6636	0.1895	1.5249
4/17/2019 12:09	12.175	12.25	14.5633	13.1333	5.9993	1.6273	0.1752	1.5521
4/17/2019 12:10	12.4083	12.95	14.9717	13.5417	5.7989	1.7	0.1861	1.5376
4/17/2019 12:11	12.55	12.625	14.5033	12.9833	5.7342	1.7	0.1876	1.5205
4/17/2019 12:12	12.5583	13.1333	14.94	12.9583	5.6277	1.7	0.1684	1.5203
4/17/2019 12:13	12.725	12.875	14.8817	13.6667	5.4703	1.7	0.1863	1.5243
4/17/2019 12:14	13.0833	13.5917	15.6333	14.4583	5.2805	1.68	0.1929	1.5218
4/17/2019 12:15	13.3583	13.2333	15.0833	13.4667	5.1832	1.6	0.1852	1.539

4/17/2019 12:16	13.4	13.3083	14.7383	13.5417	5.213	1.6333	0.1832	1.5101
4/17/2019 12:17	13.1083	13.6167	15.1083	13.3667	5.2788	1.6	0.1825	1.5094
4/17/2019 12:18	12.875	13.0583	14.7117	12.8833	5.2453	1.6	0.1859	1.5396
4/17/2019 12:19	12.65	12.4583	14.385	12.6083	5.245	1.6	0.1832	1.5588
4/17/2019 12:20	12.3833	12.35	14.315	12.75	5.2298	1.5	0.1779	1.5784
4/17/2019 12:21	11.9833	11.2333	13.5117	11.8333	5.1942	1.5	0.193	1.599
4/17/2019 12:22	11.6167	12.3	13.4583	11.7917	5.1771	1.5	0.1753	1.6018
4/17/2019 12:23	11.3	11.0417	12.9717	11.4083	5.1673	1.5	0.1868	1.608
4/17/2019 12:24	11.2	11.15	13.4733	11.675	5.1752	1.5	0.1841	1.6219
4/17/2019 12:25	11.0333	10.5	12.71	10.325	5.1389	1.4667	0.1785	1.6214
4/17/2019 12:26	10.775	10.0167	12.4867	10.4167	5.0925	1.5	0.191	1.5928
4/17/2019 12:27	10.375	10.0833	12.2783	10.15	5.086	1.5	0.1793	1.6186
4/17/2019 12:28	10.075	9.8917	11.8583	9.6667	5.0846	1.5	0.1899	1.6247
4/17/2019 12:29	9.825	9.1333	11.8383	9.775	5.0506	1.5	0.1858	1.6201
4/17/2019 12:30	9.55	8.775	11.6767	9.4167	5.292	1.5636	0.1827	1.6323
4/17/2019 12:31	8.9417	8.2667	24.1417	9.0917	17.9022	2.3545	0.4853	1.7018
4/17/2019 12:32	7.8333	5.0583	59.5633	8.1667	30.0363	33.3182	1.3087	1.9547
4/17/2019 12:33	6.25	3.6667	48.36	5.2833	35.0073	33.08	1.0937	1.9537
4/17/2019 12:34	4.1833	1.3083	138.7983	6.4167	58.8372	73.6182	4.0187	2.4443
4/17/2019 12:35	2.35	2.9167	285.25	8.8667	63.0909	93.45	6.3338	3.2149
4/17/2019 12:36	1	-0.3083	433.1833	7.6917	66.9568	96.9818	9.1535	4.2088
4/17/2019 12:37	0.4583	-0.6417	519.2167	5.4667	64.9378	96.3	10.2722	4.783
4/17/2019 12:38	0.2917	0.0833	363.0667	3.1	58.8214	70.7	6.5412	4.1053
4/17/2019 12:39	0.2	-0.6833	531.1166	6.2083	77.2038	90.2546	9.4265	4.8385
4/17/2019 12:40	0.2167	-1.2	298.75	1.3167	61.7385	101.4727	6.1472	4.1228
4/17/2019 12:41	0.2667	-0.15	335.3833	3.5917	66.9634	89.4091	7.6832	3.9253
4/17/2019 12:42	0.3	-0.425	314.55	2.275	58.6864	91.8727	7.2455	4.0662
4/17/2019 12:43	0.3	-0.7	247.0833	0.7333	60.494	79.6167	5.775	3.7503
4/17/2019 12:44	0.3	0.925	224.4833	2.2917	58.7158	90.3273	6.4983	3.7182
4/17/2019 12:45	0.3	-0.3333	265.15	2.7917	61.2142	90.3583	9.4848	4.1119
4/17/2019 12:46	0.2917	0.3917	243.0833	2.0083	57.9138	80.5727	8.3543	4.0243
4/17/2019 12:47	0.2	-0.3833	227.7333	1.375	55.0442	79.8909	8.0005	3.8727
4/17/2019 12:48	0.2083	-0.1	209.3	1.15	53.6489	80.0917	7.3613	3.6262
4/17/2019 12:49	0.2	-0.6833	206.4	1.55	53.8118	82.025	8.1677	3.6032
4/17/2019 12:50	0.2	-0.275	193.85	1.45	51.8665	78.5455	7.9073	3.5182
4/17/2019 12:51	0.2	0.0917	185.0667	1.15	50.7941	76.3833	7.6068	3.4762
4/17/2019 12:52	0.1833	0.4583	183.4667	1.0583	49.9538	76.4083	7.9632	3.4908
4/17/2019 12:53	0.1	0.325	180.7333	1.375	48.7133	76.2667	8.2288	3.5053
4/17/2019 12:54	0.1	-0.3833	178.25	1.0417	48.0391	76.2417	7.854	3.4658
4/17/2019 12:55	0.1	-0.1667	178.35	1.0333	48.0399	76.475	8.3863	3.4743
4/17/2019 12:56	0.1	-0.4333	156.9333	0.975	45.4279	76.0167	6.4442	3.3123
4/17/2019 12:57	0.175	-0.3917	151.4667	1.025	44.8328	75.3818	6.4222	3.2283
4/17/2019 12:58	0.2	-0.2333	153.55	0.8833	45.3558	74.9667	6.4263	3.2469
4/17/2019 12:59	0.1	0.5583	155.175	1.875	45.7946	74.6909	6.3513	3.2679
4/17/2019 13:00	0.1	0.175	151.0417	1.0833	44.8634	74.47	6.5528	3.2464
4/17/2019 13:01	0.1	-0.2417	141.7583	0.8333	43.0659	73.8364	6.2028	3.235
4/17/2019 13:02	0.0917	-0.175	147.5583	1.7083	43.7299	73.3546	6.1368	3.2353

4/17/2019 13:03	0	-0.35	146.75	0.8083	43.8858	73.1667	6.8698	3.2413
4/17/2019 13:04	0	-0.0917	141.45	0.75	42.4197	72.8	6.3933	3.1678
4/17/2019 13:05	0	-0.8167	140.5083	0.3	42.1006	72.1364	6.1208	3.1777
4/17/2019 13:06	0	0.0917	139.3833	0.6583	41.8248	71.56	6.079	3.174
4/17/2019 13:07	0	0.7917	139.3167	0.65	41.7628	70.8273	6.052	3.1715
4/17/2019 13:08	0	0.4	129.075	0.4	39.5796	70.23	5.6903	3.1383
4/17/2019 13:09	0.025	-0.4833	128.325	0.8417	39.0597	69.975	5.6462	3.1247
4/17/2019 13:10	0.1	-0.0583	126.9833	0.5083	38.7111	69.6546	5.6343	3.1148
4/17/2019 13:11	0.1	-0.525	125.2167	0.5667	38.3921	69.1556	5.6622	3.1005
4/17/2019 13:12	0.05	0.2917	121.1917	0.425	37.2447	68.2667	5.4618	3.0601
4/17/2019 13:13	0	-0.1333	121.7583	0.1417	37.3575	67.7167	5.4963	3.071
4/17/2019 13:14	0	0.4083	113.7667	0.4917	35.3461	67.16	5.199	2.9953
4/17/2019 13:15	0	-0.3417	115.2833	0.275	35.6263	66.1546	5.2713	3.0301
4/17/2019 13:16	0.1	0.3083	114.3417	0.7583	35.3406	65.3909	5.2773	2.9995
4/17/2019 13:17	0.05	0.4417	111.4917	-0.1833	34.7328	64.55	5.1707	2.9765
4/17/2019 13:18	0.075	-0.0083	107.2833	0.35	33.6651	63.63	5.0395	2.9508
4/17/2019 13:19	0	0.4083	104.8667	0.525	33.046	62.6	4.9272	2.9226
4/17/2019 13:20	0	-0.6167	112.2417	0.9917	34.6687	62.2	5.2197	2.9913
4/17/2019 13:21	0	-0.475	99.3617	0.7	32.1312	61.725	4.8348	2.9137
4/17/2019 13:22	0.0917	0.15	102.8467	0.525	32.066	61.02	4.8827	2.9445
4/17/2019 13:23	0.1	0.2667	106.1167	0.4	32.8848	60.4818	5.0083	2.9991
4/17/2019 13:24	0.0636	-0.1417	101.5683	0.2917	31.8606	60.04	4.8785	2.9231
4/17/2019 13:25	0	0.1667	100.3717	-0.1667	31.3001	59.5909	4.8555	2.9326
4/17/2019 13:26	0.0083	0.3167	97.2808	0.1083	30.6911	59	4.7865	2.904
4/17/2019 13:27	0.0833	-0.1667	91.8667	0.1333	29.6606	58.52	4.6322	2.8828
4/17/2019 13:28	0.1	0.525	93.3358	0.15	29.508	57.84	4.6503	2.8835
4/17/2019 13:29	0.1083	-0.5083	92.4917	-0.05	29.5086	57.2727	4.6402	2.8679
4/17/2019 13:30	0.1167	0.2667	94.105	0.5417	29.5015	56.91	4.7058	2.8928
4/17/2019 13:31	0.15	-0.0333	92.6075	0.0417	28.8909	56.7	4.6558	2.8721
4/17/2019 13:32	0.15	-0.15	89.8267	-0.225	28.2471	56.1083	4.5727	2.8506
4/17/2019 13:33	0.175	0.525	89.8033	-0.125	27.9568	55.46	4.5803	2.849
4/17/2019 13:34	0.1833	0.3917	88.7983	-0.1	27.6712	55.0917	4.5913	2.8744
4/17/2019 13:35	0.1333	0.4417	86.035	0.4167	27.1711	54.5083	4.4855	2.8771
4/17/2019 13:36	0.1	0.675	82.9825	0.1333	26.4308	53.9	4.4052	2.834
4/17/2019 13:37	0.1	0.9083	82.2625	0.75	26.1388	53.3667	4.3648	2.8603
4/17/2019 13:38	0.1	-0.125	83.5325	0.3083	26.2708	52.96	4.4128	2.8572
4/17/2019 13:39	0.1	-0.4833	79.9725	-0.2083	25.4928	52.6364	4.3337	2.8306
4/17/2019 13:40	0.1	0.625	78.9642	-0.3417	25.0162	52.2167	4.2888	2.8077
4/17/2019 13:41	0.1	0.425	80.2275	0.3667	25.2231	51.8818	4.3538	2.8048
4/17/2019 13:42	0.1083	-0.175	82.6592	0.65	25.7583	51.6545	4.4517	2.7971
4/17/2019 13:43	0.1583	0.1083	80.9208	0.3583	25.2448	51.3833	4.3947	2.7863
4/17/2019 13:44	0.1167	0.0083	78.3133	0.5917	24.6077	51.06	4.3172	2.8003
4/17/2019 13:45	0.2	-0.5417	76.6217	0.0167	24.1076	50.7	4.2418	2.7548
4/17/2019 13:46	0.1083	0.2833	74.7125	0.1083	23.6858	50.3636	4.2012	2.7785
4/17/2019 13:47	0.1	-0.025	73.1242	0.425	23.2443	50.0417	4.1537	2.7697
4/17/2019 13:48	0.0833	0.125	74.3042	0.3167	23.3749	49.7667	4.1855	2.7539
4/17/2019 13:49	0.1	-0.075	72.6025	0.325	22.98	49.5	4.1513	2.7464

4/17/2019 13:50	0.1	0.3667	71.445	0.0167	22.4823	49.2083	4.0637	2.7168
4/17/2019 13:51	0.1	-0.6	67.5792	0.25	21.7079	48.7818	3.9478	2.7221
4/17/2019 13:52	0.1	-0.1917	61.6667	0.7667	20.4203	48.1909	3.7142	2.6511
4/17/2019 13:53							3.8817	2.6709
4/17/2019 13:54							3.6905	2.5976
4/17/2019 13:55							3.2235	2.5273
4/17/2019 13:56							2.827	2.4141
4/17/2019 13:57							2.4518	2.2943
4/17/2019 13:58							2.0967	2.1553
4/17/2019 13:59							2.0047	2.105
4/17/2019 14:00							1.7608	2.0328
4/17/2019 14:01							1.5403	1.9723
4/17/2019 14:02							1.439	1.9329
4/17/2019 14:03							1.3445	1.8943
4/17/2019 14:04							1.2295	1.8586
4/17/2019 14:05							1.1245	1.82
4/17/2019 14:06							1.0142	1.7983
4/17/2019 14:07							0.9631	1.779
4/17/2019 14:08							0.8895	1.988
4/17/2019 14:09							0.825	1.9858
4/17/2019 14:10							0.7531	1.8608
4/17/2019 14:11							0.7067	1.7765
4/17/2019 14:12							0.6636	1.7399
4/17/2019 14:13							0.6084	1.7345
4/17/2019 14:14							0.5705	1.6906
4/17/2019 14:15							0.5368	1.6683
4/17/2019 14:16							0.5	1.6713
4/17/2019 14:17							0.492	1.6729
4/17/2019 14:18							0.4665	1.6439
4/17/2019 14:19							0.4218	1.5997
4/17/2019 14:20							0.4009	1.5915
4/17/2019 14:21							0.3797	1.6052
4/17/2019 14:22							0.3711	1.6119
4/17/2019 14:23							0.3589	1.5799
4/17/2019 14:24							0.3335	1.5713
4/17/2019 14:25							0.3252	1.5497
4/17/2019 14:26							0.3062	1.5413
4/17/2019 14:27							0.2813	1.5351
4/17/2019 14:28							0.2857	1.5152
4/17/2019 14:29							0.2695	1.4988
4/17/2019 14:30							0.2461	1.5079
4/17/2019 14:31							0.2484	1.4838
4/17/2019 14:32							0.2264	1.4763
4/17/2019 14:33							0.2305	1.4793
4/17/2019 14:34							0.2373	1.484
4/17/2019 14:35							0.2363	1.4751
4/17/2019 14:36							0.2393	1.4745

4/17/2019 14:37	0.2521	1.4682
4/17/2019 14:38	0.2448	1.4767
4/17/2019 14:39	0.2427	1.4551
4/17/2019 14:40	0.2307	1.4557
4/17/2019 14:41	0.217	1.4602
4/17/2019 14:42	0.2189	1.4448
4/17/2019 14:43	0.2069	1.4335
4/17/2019 14:44	0.221	1.4405
4/17/2019 14:45	0.2318	1.4357
4/17/2019 14:46	0.2222	1.4351
4/17/2019 14:47	0.2116	1.4438
4/17/2019 14:48	0.2133	1.4516
4/17/2019 14:49	0.197	1.4485
4/17/2019 14:50	0.1987	1.4428
4/17/2019 14:51	0.2036	1.4058
4/17/2019 14:52	0.1782	1.4188
4/17/2019 14:53	0.1904	1.4462
4/17/2019 14:54	0.1839	1.4207
4/17/2019 14:55	0.1769	1.408
4/17/2019 14:56	0.1806	1.4459
4/17/2019 14:57	0.1732	1.4575
4/17/2019 14:58	0.1674	1.4798
4/17/2019 14:59	0.1773	1.481
4/17/2019 15:00	0.1634	1.4387
4/17/2019 15:01	0.1654	1.4352
4/17/2019 15:02	0.1726	1.4438
4/17/2019 15:03	0.1635	1.4545
4/17/2019 15:04	0.163	1.4508
4/17/2019 15:05	0.1672	1.4293
4/17/2019 15:06	0.1948	1.4126
4/17/2019 15:07	0.3184	1.4048
4/17/2019 15:08	0.2703	1.4005
4/17/2019 15:09	0.2514	1.4118
4/17/2019 15:10	0.2455	1.4398
4/17/2019 15:11	0.2202	1.4429
4/17/2019 15:12	0.214	1.4412
4/17/2019 15:13	0.2146	1.4109
4/17/2019 15:14	0.1928	1.4346
4/17/2019 15:15	0.2022	1.4123
4/17/2019 15:16	0.1986	1.4088
4/17/2019 15:17	0.192	1.4298
4/17/2019 15:18	0.1939	1.4441
4/17/2019 15:19	0.1893	1.4334
4/17/2019 15:20	0.1759	1.4265
4/17/2019 15:21	0.1784	1.4344
4/17/2019 15:22	0.181	1.4395
4/17/2019 15:23	0.1694	1.441

4/17/2019 15:24	0.1771	1.4441
4/17/2019 15:25	0.1671	1.4438
4/17/2019 15:26	0.1627	1.4467
4/17/2019 15:27	0.1701	1.4309
4/17/2019 15:28	0.1563	1.4235
4/17/2019 15:29	0.154	1.4501
4/17/2019 15:30	0.1566	1.4479
4/17/2019 15:31	0.1493	1.4384
4/17/2019 15:32	0.1501	1.4573
4/17/2019 15:33	0.1533	1.4295
4/17/2019 15:34	0.1445	1.4243
4/17/2019 15:35	0.1528	1.4563
4/17/2019 15:36	0.1485	1.4556
4/17/2019 15:37	0.1368	1.4454
4/17/2019 15:38	0.1467	1.4301
4/17/2019 15:39	0.1472	1.4148
4/17/2019 15:40	0.14	1.4043
4/17/2019 15:41	0.1453	1.4133
4/17/2019 15:42	0.1369	1.4313
4/17/2019 15:43	0.1408	1.4332
4/17/2019 15:44	0.1731	1.4355
4/17/2019 15:45	0.1454	1.4005
4/17/2019 15:46	0.1456	1.4133
4/17/2019 15:47	0.1561	1.4188
4/17/2019 15:48	0.1806	1.417
4/17/2019 15:49	0.1817	1.4227
4/17/2019 15:50	0.1683	1.4321
4/17/2019 15:51	0.1531	1.4543
4/17/2019 15:52	0.174	1.4646
4/17/2019 15:53	0.1773	1.4521
4/17/2019 15:54	0.1646	1.448
4/17/2019 15:55	0.1745	1.4469
4/17/2019 15:56	0.1689	1.4714
4/17/2019 15:57	0.1659	1.4838
4/17/2019 15:58	0.1721	1.5037
4/17/2019 15:59	0.1676	1.5135
4/17/2019 16:00	0.1724	1.5352
4/17/2019 16:01	0.1766	1.5237
4/17/2019 16:02	0.1656	1.5485
4/17/2019 16:03	0.1833	1.5543
4/17/2019 16:04	0.1793	1.5226
4/17/2019 16:05	0.1712	1.543
4/17/2019 16:06	0.1862	1.5754
4/17/2019 16:07	0.1708	1.5633
4/17/2019 16:08	0.1781	1.545
4/17/2019 16:09	0.186	1.571
4/17/2019 16:10	0.177	1.5643

4/17/2019 16:11							0.1854	1.5522
4/17/2019 16:12							0.1804	1.5219
4/17/2019 16:13							0.1727	1.5353
4/17/2019 16:14							0.1867	1.5648
4/17/2019 16:15							0.1762	1.5883
4/17/2019 16:16							0.1815	1.6154
4/17/2019 16:17							0.1878	1.625
4/17/2019 16:18							0.1791	1.6108
4/17/2019 16:19							0.1827	1.6063
4/17/2019 16:20	7.3833	6.6167	11.6275	6.625	2.6238	1.6091	0.1769	1.5939
4/17/2019 16:21	7.1667	6.8833	11.1967	7.0667	2.6423	1.7	0.1771	1.5942
4/17/2019 16:22	7.0083	7.2	10.9775	6.675	2.6358	1.7	0.1873	1.5851
4/17/2019 16:23	6.8667	7.0833	11.1058	6.2	2.6448	1.7	0.1775	1.6087
4/17/2019 16:24	6.6583	6.65	10.9042	6.7083	2.6257	1.7	0.1898	1.6126
4/17/2019 16:25	6.5083	6.55	10.6208	5.8333	2.6137	1.7818	0.1922	1.6475
4/17/2019 16:26	6.3417	5.8667	10.5425	6.3833	2.6273	1.8	0.1794	1.6519
4/17/2019 16:27	6.2167	5.6	10.5083	5.95	2.6538	1.8	0.1944	1.6243
4/17/2019 16:28	5.9909	5.65	10.2683	5.5333	2.6433	1.8	0.1831	1.5946
4/17/2019 16:29	5.7917	5.3	10.0144	5.375	2.6308	1.8	0.1876	1.6321
4/17/2019 16:30	5.6167	5.2833	9.8299	5.2667	2.6483	1.8	0.1925	1.665
4/17/2019 16:31	5.45	5.4667	9.7609	4.8417	2.6383	1.8	0.1789	1.6696
4/17/2019 16:32	5.275	4.9333	9.3123	5.5	2.64	1.9	0.1889	1.6768
4/17/2019 16:33	5.0333	4.6	9.3328	4.7	2.6197	1.9	0.1804	1.6808
4/17/2019 16:34	4.85	4.25	9.0749	4.5667	2.5413	1.9909	0.1847	1.6471
4/17/2019 16:35	4.675	3.3333	8.7911	4.7083	2.5251	1.975	0.1841	1.6697
4/17/2019 16:36	4.5583	3.9833	8.4343	3.9667	2.5387	1.9636	0.1734	1.6458
4/17/2019 16:37	4.4083	3.5167	7.9298	3.775	2.5324	2	0.184	1.646
4/17/2019 16:38	4.2583	3.0333	7.7608	3.6917	2.5304	2.0273	0.179	1.6657
4/17/2019 16:39	4	3.3167	7.6268	3.2833	2.5359	2.1	0.1729	1.6798
4/17/2019 16:40	3.775	2.8333	7.4808	3.55	2.5668	2.1167	0.1859	1.6761
4/17/2019 16:41	3.5333	3.2167	7.1678	3.3083	2.5595	2.2	0.1708	1.6676
4/17/2019 16:42	3.3583	3.6	6.9417	2.95	2.5598	2.3	0.1785	1.6693
4/17/2019 16:43	3.275	3.1	6.9445	2.8	2.5466	2.3333	0.182	1.6588
4/17/2019 16:44	3.2	2.3	6.881	2.1833	2.5578	2.4	0.1735	1.6901
4/17/2019 16:45	3.175	2.8333	6.8543	2.475	2.5644	2.4	0.1796	1.6895
4/17/2019 16:46	3.1	2.85	6.929	2.125	2.5578	2.4	0.178	1.6907
4/17/2019 16:47	3.1	2.4	6.6213	2.6167	2.5648	2.4182	0.1769	1.6885
4/17/2019 16:48	3.0083	2.0667	6.5548	2.2667	2.5484	2.5	0.1836	1.7087
4/17/2019 16:49	2.9083	2.4833	6.5018	2.3	2.5578	2.5	0.1613	1.6983
4/17/2019 16:50	2.8667	2.7833	6.4978	3.125	2.5498	2.5	0.1806	1.6917
4/17/2019 16:51	2.8	2.1	6.7035	2.125	2.5525	2.54	0.177	1.7286
4/17/2019 16:52	2.8	3.1333	6.6724	2.225	2.5923	2.55	0.1738	1.7378
4/17/2019 16:53	2.8	2.6667	6.6242	2.2417	2.5875	2.5	0.1733	1.7271
4/17/2019 16:54	2.8	2.1667	6.3459	2.85	2.5726	2.5	0.1693	1.7211
4/17/2019 16:55	2.8	1.7	6.7119	2.5917	2.5601	2.5	0.1735	1.7343
4/17/2019 16:56	2.7083	2.2333	6.7199	2.375	2.5788	2.5	0.1741	1.7258
4/17/2019 16:57	2.7	2.8	6.7388	2.7333	2.6045	2.5	0.1637	1.7221

4/17/2019 16:58	2.6909	2.3333	6.7408	2.7083	2.5958	2.5	0.1805	1.6935
4/17/2019 16:59	2.7	2.2833	6.4881	2.7667	2.6065	2.5	0.1603	1.6908
4/17/2019 17:00	2.7917	2.85	6.6843	3.2333	2.6176	2.5	0.1842	1.6893
4/17/2019 17:01	2.7833	2.15	6.4518	2.0417	2.6148	2.5	0.1737	1.7045
4/17/2019 17:02	2.7333	2.3333	6.4821	2.1417	2.6269	2.5	0.1705	1.7064
4/17/2019 17:03	2.75	2.4833	6.3542	2.4333	2.6458	2.5	0.1816	1.7205
4/17/2019 17:04	2.7	2.05	6.6373	1.9583	2.6373	2.5	0.1674	1.7599
4/17/2019 17:05	2.7	1.7833	6.4409	1.725	2.6203	2.5	0.177	1.7468
4/17/2019 17:06	2.7	2.5333	6.6183	1.85	2.6158	2.5091	0.1828	1.7122
4/17/2019 17:07	2.7	2.2	6.4422	2.0083	2.6202	2.6	0.1658	1.7258
4/17/2019 17:08	2.7	2.65	6.2643	2.1917	2.6314	2.6	0.1793	1.725
4/17/2019 17:09	2.6167	3.0333	6.5125	2.575	2.639	2.6	0.1693	1.7142
4/17/2019 17:10	2.625	3.0333	6.837	2.575	2.6463	2.6	0.1725	1.733
4/17/2019 17:11	2.6667	2.3667	6.7909	2.3	2.6511	2.6	0.181	1.7464
4/17/2019 17:12	2.6917	2.6833	6.6278	2.5667	2.6587	2.6	0.1699	1.755
4/17/2019 17:13	2.65	2.4833	6.7638	1.8417	2.6328	2.6	0.1783	1.75
4/17/2019 17:14	2.6	2.4167	6.5648	2.2917	2.6221	2.6	0.1739	1.7587
4/17/2019 17:15	2.5083	2.4167	6.3379	1.6917	2.6039	2.6	0.173	1.7361
4/17/2019 17:16	2.5	1.6333	6.7239	1.6917	2.6531	2.6	0.1806	1.6999
4/17/2019 17:17	2.5	2.3667	6.7058	2.25	2.6633	2.6	0.1728	1.7293
4/17/2019 17:18	2.6083	2.35	6.6377	2.0417	2.6632	2.6	0.1784	1.7251
4/17/2019 17:19	2.8333	3.3167	7.1924	2.75	2.6707	2.5333	0.1779	1.7062
4/17/2019 17:20	3.1583	3.1667	7.6647	3.4417	2.6403	2.5	0.162	1.7033
4/17/2019 17:21	3.4333	3.1667	7.3239	3.1917	2.6383	2.5	0.1761	1.6996
4/17/2019 17:22	3.5	3.6	7.5734	3.7333	2.6489	2.5	0.1685	1.6937
4/17/2019 17:23	3.5	3.3333	7.4396	3.3333	2.6623	2.5	0.1695	1.6924
4/17/2019 17:24	3.5583	3.0333	7.3225	3.275	2.6534	2.41	0.1792	1.7215
4/17/2019 17:25	3.55	3.7833	7.2361	3.3	2.6503	2.4	0.1622	1.7413
4/17/2019 17:26	3.6	3.1667	7.3679	3.3167	2.6338	2.4	0.174	1.6947
4/17/2019 17:27	3.6	3.75	7.3524	3.2417	2.626	2.38	0.1696	1.6462
4/17/2019 17:28	3.7182	4.2667	7.8214	3.6917	2.652	2.3	0.1688	1.6476
4/17/2019 17:29	3.7917	3.4667	7.573	4.0167	2.6351	2.3	0.175	1.6287
4/17/2019 17:30	3.8333	3.95	7.4296	2.9083	2.6364	2.3	0.1649	1.6433
4/17/2019 17:31	3.8		7.3477	3.175	2.6426	2.2083	0.1756	1.6835
4/17/2019 17:32	3.8	4.1833	7.6473	3.575	2.6104	2.2	0.163	1.703
4/17/2019 17:33	3.9167	3.4667	7.9042	3.1667	2.604	2.2	0.1626	1.7003
4/17/2019 17:34	4.075	4.6333	7.9133	3.825	2.5953	2.2	0.1694	1.6673
4/17/2019 17:35	4.1	4.7667	8.0473	3.55	2.5393	2.1667	0.1597	1.6523
4/17/2019 17:36	4.05		7.5587	3.3	2.5438	2.1583	0.1691	1.6864
4/17/2019 17:37	3.9833	3.9667	7.6017	3.2917	2.5278	2.1	0.1715	1.7018
4/17/2019 17:38	3.9667	3.95	7.8042	3.5417	2.531	2.1	0.1631	1.6941
4/17/2019 17:39	3.9833	3.0167	7.4573	3.7333	2.5347	2.1	0.1764	1.7033
4/17/2019 17:40	3.8917	3.7	7.279	3.3667	2.5281	2.1	0.1554	1.7294
4/17/2019 17:41	3.6417	3.2	7.1438	2.4667	2.5268	2.2	0.1687	1.7535
4/17/2019 17:42	3.5667		7.4578	3.275	2.5085	2.2	0.17	1.7093
4/17/2019 17:43	3.5		7.5307	3.0833	2.5278	2.2	0.1606	1.693
4/17/2019 17:44	3.5		7.349	3.2583	2.5325	2.2	0.1756	1.6959

4/17/2019 17:45	3.425		7.317	2.8583	2.5268	2.2	0.1587	1.7015
4/17/2019 17:46	3.4083		7.6278	3.325	2.5388	2.24	0.1702	1.6971
4/17/2019 17:47	3.375		7.0363	2.975	2.5165	2.3	0.1672	1.7211
4/17/2019 17:48	3.3333		6.9405	2.9	2.5212	2.3	0.1622	1.7332
4/17/2019 17:49	2.9833		6.766	2.4833	2.5039	2.3364	0.1795	1.7495
4/17/2019 17:50	2.8		6.7328	2.3833	2.5032	2.3727	0.1598	1.7558
4/17/2019 17:51	2.575		6.6937	2.6417	2.4808	2.4	0.1747	1.7353
4/17/2019 17:52	2.6		7.004	2.3083	2.4857	2.4	0.1631	1.7299
4/17/2019 17:53	2.6		6.634	1.9917	2.4818	2.4	0.1662	1.7473
4/17/2019 17:54	2.6917		6.6595	2.2583	2.4857	2.4	0.1755	1.7407
4/17/2019 17:55	2.7		6.7942	1.7083	2.491	2.4	0.1597	1.7403
4/17/2019 17:56	2.675		6.459	2.5167	2.4771	2.4	0.1713	1.7296
4/17/2019 17:57	2.65		6.832	2.1167	2.4729	2.4	0.1712	1.7293
4/17/2019 17:58	2.6		6.9353	2.4	2.4813	2.4	0.1634	1.7343
4/17/2019 17:59	2.6		6.7025	2.3583	2.4883	2.4083	0.1756	1.7195
4/17/2019 18:00	2.6	4.2417	6.8027	2.4333	2.4688	2.5	0.1592	1.7287
4/17/2019 18:01	2.6	3.9917	6.8843	2.6	2.4273	2.5	0.1744	1.7302
4/17/2019 18:02	2.6	6.7667	6.1585	1.8417	2.4608	2.5	0.1653	1.6831
4/17/2019 18:03	2.6167	8.7833	6.7287	1.8667	2.4464	2.5	0.1689	1.7022
4/17/2019 18:04	2.675	4.2714	6.5515	2.325	2.4723	2.53	0.1784	1.7173
4/17/2019 18:05	2.7	4.2429	6.7503	2.1333	2.4431	2.5	0.161	1.7317
4/17/2019 18:06	2.7167	2.3625	6.5588	2.3	2.4328	2.525	0.1767	1.7064
4/17/2019 18:07	2.7	2.1909	6.3858	2.7	2.4195	2.5	0.1626	1.6948
4/17/2019 18:08	2.725	2.9167	6.6267	1.9083	2.4043	2.5	0.1691	1.6994
4/17/2019 18:09	2.6917	1.9083	6.4167	2.0917	2.4034	2.5	0.1787	1.7345
4/17/2019 18:10	2.6583	1.8667	6.6227	2.95	2.4012	2.5	0.1622	1.7336
4/17/2019 18:11	2.5167	2.0917	6.4592	2.0333	2.4415	2.5	0.1857	1.7283
4/17/2019 18:12	2.4417	1.425	6.2627	2.3833	2.4314	2.5	0.1688	1.735
4/17/2019 18:13	2.3417	1.975	6.3645	2.3333	2.4233	2.5	0.1712	1.7501
4/17/2019 18:14	2.3333	2.375	6.2097	2.4583	2.4187	2.5	0.1754	1.7573
4/17/2019 18:15	2.325	2.0583	6.215	2.5583	2.4086	2.5	0.168	1.778
4/17/2019 18:16	2.4	2.0833	6.2863	2.0083	2.4332	2.5	0.179	1.7641
4/17/2019 18:17	2.4167	2.3333	6.3512	2.375	2.4106	2.5182	0.1662	1.7618
4/17/2019 18:18	2.4	1.6167	6.3897	1.9333	2.4155	2.5667	0.1806	1.7551
4/17/2019 18:19	2.375	1.9667	6.2193	1.625	2.3967	2.6	0.1741	1.7465
4/17/2019 18:20	2.25	1.9833	6.091	1.6333	2.4423	2.6	0.1709	1.7315
4/17/2019 18:21	2.175	2.575	6.3145	1.35	2.4451	2.7	0.1783	1.7786
4/17/2019 18:22	2.0583	1.7583	6.3995	1.775	2.4429	2.7	0.1637	1.7768
4/17/2019 18:23	2	1.9583	6.346	1.7	2.4114	2.7	0.1751	1.7901
4/17/2019 18:24	2	1.9667	6.4408	1.85	2.4328	2.7	0.1689	1.7704
4/17/2019 18:25	2	1.6	6.4683	1.15	2.4551	2.7	0.1764	1.7216
4/17/2019 18:26	1.975	1.4083	6.532	1.8333	2.4428	2.7	0.1806	1.7033
4/17/2019 18:27	1.9833	1.6417	6.5222	1.75	2.4567	2.7	0.1686	1.7253
4/17/2019 18:28	2	1.2	6.399	1.9417	2.4751	2.7333	0.1761	1.7153
4/17/2019 18:29	2.075	1.9083	6.2978	1.3333	2.4513	2.8	0.1731	1.7113
4/17/2019 18:30	2.025	2.5583	6.1887	1.5167	2.4372	2.8	0.1766	1.7476
4/17/2019 18:31	2	1.5333	6.4235	1.7917	2.4265	2.7273	0.1809	1.7671

4/17/2019 18:32	1.9167	1.0417	6.6285	1.9083	2.4265	2.7	0.164	1.7428
4/17/2019 18:33	1.9917	1.2917	6.2497	1.6833	2.4174	2.7	0.1777	1.7568
4/17/2019 18:34	2	2.0333	6.2695	1.5667	2.412	2.7	0.1707	1.7352
4/17/2019 18:35	1.9667	1.6667	6.2233	1.8333	2.4109	2.7	0.1718	1.778
4/17/2019 18:36	1.9	1.1917	6.645	1.475	2.3755	2.67	0.1765	1.7335
4/17/2019 18:37	1.9	1.0833	6.3323	1.625	2.387	2.7	0.1672	1.7094
4/17/2019 18:38	1.9	1.8167	6.297	1.7333	2.3927	2.7	0.1784	1.6768
4/17/2019 18:39	1.9333	1.9917	6.423	1.5833	2.3826	2.7	0.1645	1.7173
4/17/2019 18:40	1.9667	1.6583	6.5153	1.9	2.3773	2.6364	0.1666	1.7063
4/17/2019 18:41	1.9	1.9667	6.332	1.5417	2.3889	2.6	0.1787	1.6953
4/17/2019 18:42	1.8583	1.55	6.45	1.8333	2.3935	2.6	0.1663	1.689
4/17/2019 18:43	1.8083	2.1	6.6807	1.6167	2.4043	2.6	0.1774	1.6964
4/17/2019 18:44	1.85	1.875	6.5538	1.4583	2.3943	2.6	0.1659	1.7249
4/17/2019 18:45	1.8917	1.8333	6.5393	1.8417	2.3874	2.6	0.178	1.6956
4/17/2019 18:46	1.8667	1.0833	6.3247	1.1083	2.4068	2.6	0.181	1.7146
4/17/2019 18:47	1.8	1.875	6.1733	1.3917	2.375	2.6083	0.1662	1.7093
4/17/2019 18:48	1.8	1.55	6.506	1.5417	2.3819	2.7	0.1772	1.7244
4/17/2019 18:49	1.8	1.425	6.485	1.65	2.3741	2.7	0.1677	1.7505
4/17/2019 18:50	1.8	1.5667	6.5333	1.1083	2.3683	2.7	0.1733	1.7449
4/17/2019 18:51	1.8083	1.55	6.2433	1.525	2.3794	2.7	0.1729	1.7222
4/17/2019 18:52	1.8667	1.3417	6.6482	1.5917	2.3671	2.75	0.1737	1.7144
4/17/2019 18:53	1.8917	1.3417	6.5915	1.5583	2.3807	2.8	0.1766	1.7338
4/17/2019 18:54	1.9	1.1167	6.8288	1.525	2.3585	2.8	0.166	1.7182
4/17/2019 18:55	1.85	1.775	6.2422	1.6583	2.3616	2.8	0.177	1.7069
4/17/2019 18:56	1.8	1.9917	6.2307	2.0583	2.369	2.8	0.1663	1.7202
4/17/2019 18:57	1.775	1.7083	6.4202	1.275	2.3574	2.8	0.1636	1.7163
4/17/2019 18:58	1.8	1.6167	6.3423	1.4917	2.377	2.7091	0.1732	1.7012
4/17/2019 18:59	1.8	1.6583	6.4162	1.9583	2.3468	2.7	0.1585	1.713
4/17/2019 19:00	1.775	2.025	6.5571	1.5333	2.363	2.7	0.175	1.7024
4/17/2019 19:01	1.7	1.525	6.2023	1.6583	2.3553	2.7	0.1648	1.7048
4/17/2019 19:02	1.7	1.5667	6.5132	2.4	2.3564	2.7	0.1635	1.7273
4/17/2019 19:03	1.775	1.6333	6.4763	1.9167	2.3468	2.7	0.1744	1.6978
4/17/2019 19:04	1.8	1.6833	6.3536	1.5333	2.3446	2.7	0.1556	1.7121
4/17/2019 19:05	1.8	1.6167	6.5599	1.5667	2.3464	2.6167	0.1711	1.7123
4/17/2019 19:06	1.725	1.1833	6.414	1.3333	2.3254	2.6	0.1632	1.7015
4/17/2019 19:07	1.7	1.5833	6.3029	1.15	2.3353	2.6	0.1628	1.6866
4/17/2019 19:08	1.6364	1.3833	6.2945	1.1083	2.3388	2.6	0.1662	1.7028
4/17/2019 19:09	1.6083	1.9167	6.3241	1.0917	2.3181	2.6	0.1507	1.7243
4/17/2019 19:10	1.6083	1.3833	6.2808	1.2167	2.3264	2.6909	0.1695	1.7634
4/17/2019 19:11	1.6	1.5	6.6071	0.9833	2.3335	2.7	0.1557	1.7767
4/17/2019 19:12	1.6	0.9417	6.5385	0.8667	2.3213	2.7	0.1562	1.7292
4/17/2019 19:13	1.5333	0.8167	6.2906	1.6083	2.3111	2.7	0.1631	1.7138
4/17/2019 19:14	1.5	2	6.4079	1.15	2.3083	2.7	0.1518	1.7163
4/17/2019 19:15	1.5667	1.7583	6.5267	1.55	2.3134	2.7	0.155	1.7298
4/17/2019 19:16	1.65	1.4833	6.4457	1.5	2.3173	2.7	0.1616	1.7462
4/17/2019 19:17	1.7083	1.675	6.4889	1.4917	2.3135	2.75	0.1521	1.7606
4/17/2019 19:18	1.7917	1.25	6.4227	1.5083	2.2964	2.8	0.1544	1.7739

4/17/2019 19:19	1.8	1.3417	6.1578	1.4	2.2928	2.8	0.1437	1.7686
4/17/2019 19:20	1.8	1.5917	6.2418	1.2167	2.2901	2.8	0.1525	1.7468
4/17/2019 19:21	1.725	2	6.0335	0.5667	2.2818	2.8	0.1537	1.7483
4/17/2019 19:22	1.7	1.55	6.5311	1.5583	2.281	2.8	0.143	1.7542
4/17/2019 19:23	1.7	1.2417	6.2904	1.125	2.2948	2.8	0.161	1.7528
4/17/2019 19:24	1.7	1.3833	6.2759	2.2417	2.2736	2.8	0.1488	1.7468
4/17/2019 19:25	1.7	1.5583	6.4731	2.2917	2.2748	2.8	0.1483	1.7589
4/17/2019 19:26	1.6917	1.2	6.4615	1.4333	2.2908	2.8	0.1581	1.7725
4/17/2019 19:27	1.6833	1.1167	6.6675	1.6	2.2755	2.8	0.1479	1.7696
4/17/2019 19:28	1.7	0.975	6.2993	1.375	2.3013	2.8	0.1609	1.7046
4/17/2019 19:29	1.7	0.9083	6.3889	0.8917	2.298	2.8	0.1512	1.7183
4/17/2019 19:30	1.625	0.975	6.4156	0.8083	2.2785	2.8	0.1486	1.7408
4/17/2019 19:31	1.625	1.525	6.0474	1.6083	2.2979	2.8	0.1557	1.7398
4/17/2019 19:32	1.6917	1.1667	6.2368	1.3083	2.3104	2.8	0.1426	1.6964
4/17/2019 19:33	1.6333	1.075	6.25	0.8917	2.3182	2.8	0.1535	1.7143
4/17/2019 19:34	1.6167	1.6667	6.3854	1.4167	2.3088	2.8	0.1495	1.6961
4/17/2019 19:35	1.575	1.325	6.2812	1.05	2.312	2.9	0.1395	1.7305
4/17/2019 19:36	1.5083	1.5583	6.3743	0.5333	2.3111	2.9	0.1575	1.7201
4/17/2019 19:37	1.5667	1.525	6.3364	1.1667	2.2496	2.9	0.1487	1.7346
4/17/2019 19:38	1.6	1.8083	6.551	1.2417	2.2282	2.9	0.15	1.7436
4/17/2019 19:39	1.6	1.2417	6.4568	1.05	2.2175	2.8727	0.1572	1.7213
4/17/2019 19:40	1.6083	1.9917	6.2973	1.325	2.2355	2.8	0.1427	1.7049
4/17/2019 19:41	1.6	1.8167	6.5956	1.5	2.2242	2.8	0.1544	1.6881
4/17/2019 19:42	1.6	0.775	6.5203	1.025	2.2175	2.7455	0.1483	1.6628
4/17/2019 19:43	1.6	1.1167	6.4095	1.15	2.2136	2.75	0.1474	1.6816
4/17/2019 19:44	1.6	0.875	6.6548	1.25	2.2149	2.7	0.1563	1.7068
4/17/2019 19:45	1.5833	1.375	6.521	1.725	2.2343	2.7	0.1439	1.7231
4/17/2019 19:46	1.6	0.625	6.498	1.4083	2.2175	2.7	0.1522	1.7267
4/17/2019 19:47	1.6	0.5083	6.2868	1.3417	2.2114	2.7	0.1593	1.7063
4/17/2019 19:48	1.5667	1.125	6.4094	1.3333	2.2033	2.7	0.1427	1.7125
4/17/2019 19:49	1.525	1.4333	6.8118	1.5167	2.2165	2.7	0.1587	1.6779
4/17/2019 19:50	1.5	0.875	6.4552	1.4917	2.2105	2.7	0.1453	1.6772
4/17/2019 19:51	1.5417	1.2917	6.4003	1.375	2.217	2.7	0.1564	1.6558
4/17/2019 19:52	1.6333	1	6.2108	1.3417	2.2144	2.7	0.1543	1.6733
4/17/2019 19:53	1.7	1.0083	6.5481	1.1583	2.2168	2.7	0.148	1.684
4/17/2019 19:54	1.7	1.4333	6.2384	1.2917	2.2293	2.7	0.1568	1.6843
4/17/2019 19:55	1.65	1.325	6.0441	1.2833	2.2151	2.7	0.1483	1.7108
4/17/2019 19:56	1.5917	1.2667	6.3551	1.375	2.2313	2.7455	0.1553	1.6963
4/17/2019 19:57	1.5917	1.5917	6.6144	1.0667	2.215	2.7455	0.1562	1.7074
4/17/2019 19:58	1.6	1.3583	6.2196	0.9167	2.2268	2.7	0.1479	1.7078
4/17/2019 19:59	1.6083	1.5333	6.4271	1.5333	2.2336	2.7	0.1638	1.7081
4/17/2019 20:00	1.6667	1.425	6.4796	1.3	2.2355	2.7	0.1549	1.7099
4/17/2019 20:01	1.6667	1.2667	6.2377	1.1833	2.2293	2.7	0.1622	1.7171
4/17/2019 20:02	1.6583	0.7	6.7284	0.9167	2.2196	2.7	0.1608	1.6672
4/17/2019 20:03	1.6	1.25	6.2863	1.375	2.215	2.7583	0.1473	1.7006
4/17/2019 20:04	1.5917	1.0667	6.6723	1.125	2.2317	2.7	0.1637	1.7088
4/17/2019 20:05	1.5417	1.425	6.4903	0.8417	2.2278	2.7909	0.1548	1.7355

4/17/2019 20:06	1.6	1.8	6.2484	1.2	2.2153	2.8	0.1606	1.7581
4/17/2019 20:07	1.625	1.7333	6.1936	0.8333	2.2309	2.8	0.16	1.7627
4/17/2019 20:08	1.7	1.9	6.536	1.4833	2.2193	2.8	0.1494	1.7647
4/17/2019 20:09	1.7	1.9667	6.404	1.375	2.2241	2.85	0.169	1.7691
4/17/2019 20:10	1.7083	1.5167	6.2123	1.3083	2.2148	2.9	0.1603	1.7566
4/17/2019 20:11	1.8	1.3667	6.1384	1.55	2.2333	2.9	0.1576	1.7379
4/17/2019 20:12	1.8833	2.0417	6.469	1.3	2.2487	2.8545	0.1675	1.7523
4/17/2019 20:13	1.9417	1.1333	6.3769	1.6417	2.2296	2.8273	0.1487	1.743
4/17/2019 20:14	2	1.9833	6.2568	1.6	2.2393	2.9	0.165	1.7342
4/17/2019 20:15	1.9833	1.4333	6.2758	1.4	2.2369	2.8083	0.1612	1.7185
4/17/2019 20:16	1.9667	2.1583	6.4202	1.3833	2.2342	2.8	0.1593	1.7222
4/17/2019 20:17	1.9	2.0917	6.4037	1.475	2.2459	2.8	0.1694	1.7358
4/17/2019 20:18	1.875	1.525	6.2088	0.8333	2.2228	2.8	0.1528	1.7671
4/17/2019 20:19	1.8583	1.425	6.2366	1.7333	2.2236	2.8	0.1609	1.7458
4/17/2019 20:20	1.9083	1.6917	6.4996	1.9	2.2129	2.76	0.1556	1.7208
4/17/2019 20:21	1.9	1.6583	6.5283	1.55	2.2251	2.7	0.1604	1.7208
4/17/2019 20:22	1.8583	1.375	6.4351	1.425	2.2169	2.7	0.1643	1.7305
4/17/2019 20:23	1.8	1.3917	6.0613	1.3167	2.2371	2.7	0.1479	1.7574
4/17/2019 20:24	1.8	1.2833	6.3497	2	2.2243	2.7	0.1699	1.7664
4/17/2019 20:25	1.8	1.5083	6.3451	1.2	2.2055	2.6333	0.1605	1.7619
4/17/2019 20:26	1.8917	1.7	6.4507	2.0917	2.2318	2.6	0.1556	1.7584
4/17/2019 20:27	1.9	1.5333	6.2662	1.825	2.2079	2.6	0.1664	1.7666
4/17/2019 20:28	1.9	1.6583	6.1524	0.8583	2.2294	2.6	0.1543	1.7465
4/17/2019 20:29	1.8333	1.0917	6.2064	1.1	2.2274	2.6909	0.1633	1.7577
4/17/2019 20:30	1.825	1.2667	6.2089	1.6917	2.225	2.7	0.154	1.7678
4/17/2019 20:31	1.8	1.525	6.0132	1.8917	2.214	2.7	0.1537	1.7482
4/17/2019 20:32	1.7333	1.0667	6.2245	1.2	2.2177	2.6583	0.1647	1.7525
4/17/2019 20:33	1.8	1.2	6.3985	0.775	2.2055	2.6	0.1552	1.7468
4/17/2019 20:34	1.8167	1.725	6.3398	1.4583	2.2013	2.6636	0.1603	1.7479
4/17/2019 20:35	1.9583	1.7583	6.192	2.2833	2.2118	2.65	0.1542	1.7298
4/17/2019 20:36	2.0083	1.675	6.5503	1.775	2.225	2.7	0.1541	1.7316
4/17/2019 20:37	2.0083	0.875	6.8952	1.2333	2.225	2.7	0.1648	1.7915
4/17/2019 20:38	1.9	1.375	5.8642	1.6333	2.1767	2.7	0.1492	1.8169
4/17/2019 20:39	1.8333	1.6667	6.0055	1.4917	2.1564	2.7	0.1593	1.7529
4/17/2019 20:40	1.875	1.6333	5.9962	2.0667	2.1807	2.7	0.1524	1.7365
4/17/2019 20:41	2	1.2917	6.1598	1.875	2.1664	2.7	0.165	1.7187
4/17/2019 20:42	2	1.7083	6.2013	1.4333	2.161	2.7	0.1665	1.7197
4/17/2019 20:43	1.9667	1.55	6.0948	1.9083	2.1808	2.7	0.1512	1.6997
4/17/2019 20:44	1.8917	1.9833	6.0648	1.3833	2.1617	2.7	0.1659	1.7105
4/17/2019 20:45	1.8	1.8583	6.67	1.2917	2.1674	2.7455	0.1484	1.7113
4/17/2019 20:46	1.8	2.1833	6.1607	1.25	2.1759	2.8	0.1618	1.7244
4/17/2019 20:47	1.8	1.3583	6.2838	1.575	2.1953	2.8	0.1646	1.7202
4/17/2019 20:48	1.7833	1.0167	6.3695	1.9917	2.1688	2.8	0.1567	1.7212
4/17/2019 20:49	1.7	1.1083	6.3082	1.6333	2.1881	2.8	0.1649	1.7678
4/17/2019 20:50	1.6833	1.625	6.1815	1.6167	2.1975	2.8	0.1483	1.7609
4/17/2019 20:51	1.6833	1.3	6.0317	0.9917	2.1839	2.8	0.1682	1.7783
4/17/2019 20:52	1.7	1.4083	5.9173	1.5	2.1878	2.8	0.1599	1.7846

4/17/2019 20:53	1.7	1.0917	6.2068	1.5333	2.1752	2.8	0.1623	1.7522
4/17/2019 20:54	1.7167	1.2667	5.823	1.2417	2.2022	2.8	0.1636	1.7396
4/17/2019 20:55	1.8	1.2583	6.0307	1.8333	2.1947	2.8	0.1481	1.7693
4/17/2019 20:56	1.8	1.2083	5.9373	1.45	2.2108	2.8	0.1675	1.7585
4/17/2019 20:57	1.8	1.6667	5.9523	1.4167	2.2182	2.8	0.1569	1.7263
4/17/2019 20:58	1.8	1.5917	6.01	0.8417	2.2148	2.8	0.1606	1.7342
4/17/2019 20:59	1.7333	1.0167	6.1057	1.3	2.2287	2.8	0.1671	1.7269
4/17/2019 21:00	1.6	0.9917	5.8445	1.1667	2.194	2.8	0.1578	1.7771
4/17/2019 21:01	1.625	1.4583	6.1688	1.0083	2.2429	2.8417	0.1675	1.7634
4/17/2019 21:02	1.6417	1.1167	5.8535	0.8583	2.2473	2.9	0.1524	1.7493
4/17/2019 21:03	1.65	0.925	5.834	1.0833	2.2482	2.9	0.161	1.7658
4/17/2019 21:04	1.6	1.0333	5.8755	1.4583	2.2543	2.9	0.1649	1.7406
4/17/2019 21:05	1.7	1.275	5.7362	0.7083	2.3082	2.9	0.15	1.7473
4/17/2019 21:06	1.75	1.95	6.3232	1.95	2.3269	2.9	0.1679	1.7185
4/17/2019 21:07	1.775	1.0667	6.2178	1.3	2.2843	2.8083	0.1572	1.711
4/17/2019 21:08	1.7667	1.2417	6.0817	0.825	2.3283	2.8	0.1619	1.7208
4/17/2019 21:09	1.675	1.6	6.0558	0.6917	2.2599	2.8	0.1659	1.7663
4/17/2019 21:10	1.6	1.7167	6.0098	1.4167	2.2566	2.8	0.1488	1.7957
4/17/2019 21:11	1.6	1.3417	6.0857	1.5917	2.2685	2.8	0.1648	1.804
4/17/2019 21:12	1.6	1.15	5.8298	1.4917	2.3336	2.8	0.1557	1.7955
4/17/2019 21:13	1.5917	0.6667	5.9308	1.1917	2.3134	2.7222	0.1654	1.7945
4/17/2019 21:14	1.4667	1.3583	5.7637	0.8833	2.3147	2.7	0.1679	1.7735
4/17/2019 21:15	1.4083	1.425	5.6888	0.8583	2.3712	2.7	0.1589	1.7621
4/17/2019 21:16	1.5	1.6667	5.9678	1.025	2.4005	2.7	0.168	1.7983
4/17/2019 21:17	1.5	1	6.0463	0.7417	2.3976	2.7	0.1494	1.8016
4/17/2019 21:18	1.5	1.4333	5.9553	0.675	2.4046	2.7	0.1623	1.8053
4/17/2019 21:19	1.4	1.05	5.7512	0.6333	2.3732	2.7273	0.1615	1.7978
4/17/2019 21:20	1.4	0.6417	5.7315	0.475	2.3857	2.8	0.1662	1.792
4/17/2019 21:21	1.4	0.4	5.7603	0.7917	2.3685	2.8	0.1632	1.8009
4/17/2019 21:22	1.4	0.675	5.4568	0.8083	2.4022	2.8	0.1469	1.8022
4/17/2019 21:23	1.3917	1.125	5.9835	0.9417	2.4078	2.8	0.1655	1.8
4/17/2019 21:24	1.3917	0.6833	5.9128	1.2417	2.4539	2.8	0.1545	1.7808
4/17/2019 21:25	1.3417	0.55	5.698	0.8167	2.4328	2.8	0.1589	1.7841
4/17/2019 21:26	1.3	0.9667	5.6805	1.1083	2.4805	2.8	0.1661	1.7796
4/17/2019 21:27	1.2917	1.15	5.748	0.8417	2.4407	2.8	0.1526	1.8082
4/17/2019 21:28	1.3	0.45	5.7148	1.05	2.4328	2.8	0.1692	1.8468
4/17/2019 21:29	1.3083	0.85	5.7343	1.1667	2.4465	2.8	0.1507	1.8199
4/17/2019 21:30	1.3	0.775	5.52	1.05	2.4521	2.8	0.1721	1.8004
4/17/2019 21:31	1.3	0.7583	5.8893	1.0333	2.495	2.8444	0.154	1.768
4/17/2019 21:32	1.3	0.8583	5.5887	1.3333	2.4884	2.8273	0.1511	1.7753
4/17/2019 21:33	1.3	0.7083	5.9818	1.0167	2.5398	2.88	0.1704	1.7969
4/17/2019 21:34	1.3	0.6583	5.6817	1.2917	2.5437	2.8583	0.1571	1.7851
4/17/2019 21:35	1.2917	1.1167	5.6317	0.7417	2.516	2.8	0.1652	1.7915
4/17/2019 21:36	1.3	1.025	5.7163	0.5333	2.5266	2.8	0.1543	1.7805
4/17/2019 21:37	1.3	1.0917	5.7405	0.5083	2.521	2.88	0.1648	1.7737
4/17/2019 21:38	1.2583	1.8167	5.6508	0.625	2.521	2.9	0.1615	1.8115
4/17/2019 21:39	1.2167	0.6417	5.4507	0.7333	2.5073	2.9	0.1591	1.7837

4/17/2019 21:40	1.2	0.8917	5.9872	0.825	2.5142	2.9	0.1667	1.7799
4/17/2019 21:41	1.2	-0.0417	5.8412	0.8667	2.522	2.9	0.1594	1.7799
4/17/2019 21:42	1.2	1.3	5.689	0.4333	2.5295	2.9	0.1563	1.7726
4/17/2019 21:43	1.2	1.4333	5.6058	0.7417	2.5458	2.9	0.1626	1.7648
4/17/2019 21:44	1.2	1	5.4005	0.375	2.5698	2.9	0.1533	1.7976
4/17/2019 21:45	1.2	0.6167	6.0123	0.925	2.5774	2.9	0.1631	1.8005
4/17/2019 21:46	1.2	1.4	5.6103	0.8917	2.5819	2.9	0.1501	1.8158
4/17/2019 21:47	1.2	0.9417	5.7625	0.5917	2.6011	2.9	0.1633	1.8228
4/17/2019 21:48	1.2	0.6583	5.633	1.325	2.6222	2.9	0.1534	1.8181
4/17/2019 21:49	1.2	1.225	5.666	0.8	2.6361	2.9	0.1549	1.7957
4/17/2019 21:50	1.2	0.8583	5.5558	0.4917	2.6559	2.9	0.1684	1.7771
4/17/2019 21:51	1.2	1.2833	5.5008	1.7417	2.6565	2.9	0.1488	1.7765
4/17/2019 21:52	1.2667	0.7917	5.3555	0.6833	2.6612	2.9	0.163	1.7967
4/17/2019 21:53	1.3	1.05	5.8	0.9917	2.6611	2.9	0.1513	1.8078
4/17/2019 21:54	1.2917	1.1583	5.3728	0.9583	2.6924	2.9	0.1571	1.7974
4/17/2019 21:55	1.2083	0.6333	5.094	1.0333	2.6755	2.9	0.1616	1.7928
4/17/2019 21:56	1.1917	0.1917	5.4975	0.6417	2.7083	2.8	0.1464	1.7796
4/17/2019 21:57	1.1	0.8583	5.4088	0.7167	2.7106	2.8	0.1648	1.7829
4/17/2019 21:58	1.1	1.025	5.6432	0.9833	2.7268	2.8	0.1579	1.7843
4/17/2019 21:59	1.1	1.275	5.5877	1.2417	2.7268	2.8	0.1578	1.7708
4/17/2019 22:00	1.1	1.0167	5.334	0.9083	2.7428	2.8	0.1575	1.7925
4/17/2019 22:01	1.0833	1.2	5.7193	0.9	2.7781	2.8	0.1504	1.7913
4/17/2019 22:02	1.0667	0.625	5.4742	0.575	2.7772	2.8	0.1676	1.779
4/17/2019 22:03	1.0167	0.9667	5.5104	0.8417	2.7851	2.8	0.1577	1.7743
4/17/2019 22:04	1	0.9	5.4007	0.8333	2.7843	2.8	0.1561	1.7755
4/17/2019 22:05	1	0.425	5.3811	0.4833	2.8133	2.75	0.1646	1.7703
4/17/2019 22:06	1	0.575	5.399	0.8833	2.7953	2.7	0.1555	1.7937
4/17/2019 22:07	1	1.2917	5.3877	0.7917	2.7914	2.7	0.1664	1.8063
4/17/2019 22:08	0.9667	0.8083	5.2963	1.0917	2.8011	2.7	0.1623	1.7981
4/17/2019 22:09	0.9	0.6167	5.3906	0.7667	2.7984	2.7	0.162	1.7834
4/17/2019 22:10	0.9	0.75	5.2918	0.875	2.813	2.7	0.1618	1.7583
4/17/2019 22:11	0.95	0.1	5.4208	0.6583	2.8188	2.7	0.1545	1.7873
4/17/2019 22:12	1	0.3917	5.4471	0.3333	2.8178	2.7	0.1689	1.7983
4/17/2019 22:13	0.9667	1.1	5.2898	0.55	2.8359	2.7	0.1552	1.7859
4/17/2019 22:14	0.925	0.8167	5.2897	1.0833	2.8373	2.7	0.1629	1.7743
4/17/2019 22:15	1	1.3667	5.3973	0.825	2.8426	2.7	0.1575	1.7743
4/17/2019 22:16	1.0083	0.85	5.3743	0.35	2.8197	2.7	0.1605	1.7681
4/17/2019 22:17	1.1	-0.4167	5.3818	0.75	2.8392	2.8	0.1624	1.7911
4/17/2019 22:18	1.0167	1.2583	5.2694	0.5583	2.8666	2.8	0.152	1.7916
4/17/2019 22:19	1	0.175	5.2488	0.8667	2.8511	2.8	0.1652	1.7963
4/17/2019 22:20	1	1	5.3018	0.8167	2.854	2.8	0.1589	1.7861
4/17/2019 22:21	1.0167	0.3417	5.2403	0.8583	2.8808	2.8	0.1573	1.7671
4/17/2019 22:22	1.1	0.5583	5.2876	0.7583	2.8738	2.8	0.1619	1.7573
4/17/2019 22:23	1.1	0.8417	5.4222	0.1083	2.9071	2.8	0.1516	1.7915
4/17/2019 22:24	1.1	0.525	5.3766	0.5583	2.8942	2.9	0.1636	1.7914
4/17/2019 22:25	1.1	1.7917	5.3379	0.725	2.9132	2.9	0.1591	1.7925
4/17/2019 22:26	1.1	0.9167	5.2043	0.5667	2.9317	2.9	0.1608	1.7945

4/17/2019 22:27	1.0917	0.3583	5.6596	0.5	2.9334	2.875	0.1592	1.7928
4/17/2019 22:28	1	1.0917	4.9958	0.8833	2.9459	2.8273	0.1519	1.8163
4/17/2019 22:29	1	0.1417	5.3102	0.55	2.9503	2.825	0.1701	1.8223
4/17/2019 22:30	1.0583	0.75	5.5677	0.7583	2.9385	2.85	0.1477	1.8313
4/17/2019 22:31	1.1	1.4917	5.4286	1.025	2.9644	2.9	0.1574	1.8255
4/17/2019 22:32	1.0583	1.6833	5.3993	0.4917	2.97	2.9	0.165	1.8098
4/17/2019 22:33	1	1.0083	5.19	0.2667	2.9801	2.9	0.1516	1.8088
4/17/2019 22:34	1	0.3333	5.6037	0.0083	2.958	2.9	0.162	1.8161
4/17/2019 22:35	1.0583	0.5667	5.7206	0.5167	3.0149	2.9	0.1523	1.7841
4/17/2019 22:36	1.0583	1.0167	5.2738	0.05	3.0053	2.9	0.1578	1.7793
4/17/2019 22:37	1.0583	0.975	5.1947	0.6083	3.0034	2.9	0.1638	1.7923
4/17/2019 22:38	1	0.45	5.2786	0.6417	3.0053	2.9	0.1551	1.8041
4/17/2019 22:39	1	0.4333	5.14	0.5333	3.005	2.9	0.1692	1.7924
4/17/2019 22:40	1	0.6	5.3709	0.1833	2.9903	2.99	0.1477	1.7997
4/17/2019 22:41	1	1.4917	5.0169	1.1333	2.9647	2.9273	0.1601	1.8101
4/17/2019 22:42	1	0.625	5.2796	0.3	2.9616	3	0.1577	1.7991
4/17/2019 22:43	0.9833	0.8417	4.9836	0.3917	2.9843	3	0.1611	1.7754
4/17/2019 22:44	0.9083	0.2417	5.2933	0.5083	2.9738	3	0.1724	1.7803
4/17/2019 22:45	0.9	1.0083	5.0735	0.55	2.9893	3	0.1457	1.7748
4/17/2019 22:46	0.875	0.575	5.055	0.0917	3.0358	3	0.1524	1.7878
4/17/2019 22:47	0.9	0.75	5.1664		3.0008	3	0.1571	1.7823
4/17/2019 22:48	0.9	0.5833	4.868	0.71	3.0148	3	0.1624	1.7999
4/17/2019 22:49	0.8667	0.3583	5.0405	0.6	2.9898	3.0273	0.1642	1.8145
4/17/2019 22:50	0.8	0.7	5.1244	0.2833	3.041	3.0333	0.1559	1.8052
4/17/2019 22:51	0.8	0.4333	4.9987	0.65	3.0363	3	0.1672	1.8108
4/17/2019 22:52	0.8	0.55	4.9995		3.0494	3	0.1558	1.7848
4/17/2019 22:53	0.8083	0.6083	4.8264		3.0414	3	0.164	1.7973
4/17/2019 22:54	0.9	0.1917	5.1692		3.087	3	0.1616	1.8096
4/17/2019 22:55	0.9	0.4167	4.9272		3.0649	3	0.1564	1.8023
4/17/2019 22:56	0.9	0.325	4.776		3.0751	3	0.1627	1.7963
4/17/2019 22:57	0.9	0.1583	4.9526		3.0762	3	0.1535	1.7693
4/17/2019 22:58	0.8917	0.8083	4.5646		3.0997	3.0833	0.1561	1.7883
4/17/2019 22:59	0.8583	0.5333	5.0742		3.1044	3.0727	0.1629	1.8188
4/17/2019 23:00	0.8083	0.3583	5.2989		3.0779	3.0833	0.1489	1.829
4/17/2019 23:01	0.8917	1.0083	5.2075		3.1365	3	0.161	1.7881
4/17/2019 23:02	0.825	0.6417	4.931		3.0938	3.0417	0.152	1.7789
4/17/2019 23:03	0.8417	0.7167	5.1194		3.0788	3.1	0.1631	1.8069
4/17/2019 23:04	0.8	0.8417	5.0921		3.1008	3.1	0.1628	1.791
4/17/2019 23:05	0.8917	0.4917	4.8723	0.2	3.0919	3.1	0.1516	1.8088
4/17/2019 23:06	0.9333	0.9083	5.0972	0.8286	3.1357	3.1	0.164	1.817
4/17/2019 23:07	0.9917	0.1833	4.9958	0.625	3.1291	3.1	0.1479	1.8068
4/17/2019 23:08	1	1.2917	5.2349	0.5	3.1358	3.0417	0.1651	1.7944
4/17/2019 23:09	0.9818	0.6167	4.7514	0.6111	3.1339	3	0.1581	1.7953
4/17/2019 23:10	0.975	1.1833	4.7055	0.2636	3.1307	3	0.1565	1.7907
4/17/2019 23:11	0.9167	0.4083	5.2526	0.75	3.1438	3	0.1637	1.8145
4/17/2019 23:12	0.9	0.5167	4.9732	0.8833	3.1201	3	0.1565	1.8074
4/17/2019 23:13	0.9167	0.5	5.0039	0.7833	3.1308	3	0.1613	1.7753

4/17/2019 23:14	0.9083	0.6583	5.0194	0.8167	3.1313	3	0.1616	1.7689
4/17/2019 23:15	0.925	0.5417	4.9363	0.1667	3.1106	3	0.1561	1.7691
4/17/2019 23:16	0.9083	0.475	4.7053	0.7667	3.1244	3	0.1684	1.7728
4/17/2019 23:17	0.9	0.5167	4.5318	0.1333	3.1305	3	0.1507	1.7966
4/17/2019 23:18	0.8583	-0.125	5.2296	0.3833	3.1198	3	0.1657	1.8221
4/17/2019 23:19	0.8	0.6583	4.5492	0	3.1234	3.0364	0.1578	1.8301
4/17/2019 23:20	0.8583	0.575	5.4593	0.3167	3.1433	3.1	0.1547	1.8297
4/17/2019 23:21	0.9	0.6083	4.5531	0.5167	3.1281	3.1	0.1608	1.8018
4/17/2019 23:22	0.9	0.675	4.6147	0.2333	3.1467	3.0727	0.1462	1.7793
4/17/2019 23:23	0.85	0.0083	4.7938	1.0167	3.1364	3	0.1638	1.7449
4/17/2019 23:24	0.825	0.4083	5.1865	0.6333	3.1318	3.025	0.1533	1.7693
4/17/2019 23:25	0.8	0.925	4.9961	-0.05	3.1407	3.0417	0.1586	1.7893
4/17/2019 23:26	0.8	1.1417	5.0154	0.3333	3.1538	3	0.1619	1.8115
4/17/2019 23:27	0.8	0.9	4.5488	-0.2167	3.1473	3	0.1498	1.8313
4/17/2019 23:28	0.8	-0.0083	5.107	0.4667	3.1735	3	0.1601	1.7842
4/17/2019 23:29	0.8333	0.55	4.7067	0.4833	3.1742	3	0.155	1.812
4/17/2019 23:30	0.8167	1.15	4.9319	0.4833	3.1528	3	0.1631	1.8151
4/17/2019 23:31	0.8	0.8333	5.0998	0.2333	3.1368	3	0.1576	1.8213
4/17/2019 23:32	0.8	0.3667	4.9945	0.6667	3.1691	2.9667	0.1469	1.7993
4/17/2019 23:33	0.8	0.475	4.8975	0.05	3.1873	2.9	0.1654	1.7932
4/17/2019 23:34	0.8667	0.4333	4.8902	0.2833	3.1931	2.9	0.1527	1.7973
4/17/2019 23:35	0.9	0.025	4.5522	0.3833	3.1844	2.91	0.1603	1.7741
4/17/2019 23:36	0.9	0.625	5.0353	0.7	3.1589	2.925	0.164	1.789
4/17/2019 23:37	0.8583	0.2167	4.805	0.4167	3.1627	2.9	0.1457	1.815
4/17/2019 23:38	0.8	0.3083	4.9052	0.1667	3.1912	2.9	0.1613	1.8007
4/17/2019 23:39	0.8	0.35	5.0163	0.35	3.1572	2.9	0.1493	1.7798
4/17/2019 23:40	0.8	0.625	4.7997	0.1	3.159	2.9	0.1593	1.7648
4/17/2019 23:41	0.8	0.725	4.5998	0.7667	3.1475	2.9	0.1604	1.7864
4/17/2019 23:42	0.8	0.8583	5.1657	0.9333	3.1117	2.9	0.1502	1.7663
4/17/2019 23:43	0.8	1.075	4.8547	0.3	3.1124	2.875	0.1645	1.7713
4/17/2019 23:44	0.8	0.6833	4.849	0.2	3.0744	2.85	0.1564	1.8008
4/17/2019 23:45	0.8	0.6583	4.9198	-0.25	3.0906	2.8364	0.163	1.8057
4/17/2019 23:46	0.8	0.4	4.6633	0.0333	3.0907	2.8	0.1609	1.7783
4/17/2019 23:47	0.8	0.825	4.6467	0.2333	3.0961	2.8	0.1487	1.7875
4/17/2019 23:48	0.8	0.575	4.9152	0.3833	3.0861	2.8	0.1622	1.7972
4/17/2019 23:49	0.8917	0.65	4.5522	-0.1667	3.1061	2.8	0.1508	1.7993
4/17/2019 23:50	0.8833	0.9667	4.8183	0.0833	3.0848	2.8	0.1556	1.7802
4/17/2019 23:51	0.8833	0.475	4.7733	0.25	3.0742	2.8	0.1627	1.77
4/17/2019 23:52	0.9	0.2667	4.7345	0.8	3.0589	2.8	0.152	1.7862
4/17/2019 23:53	0.9	0.8417	4.754	0.15	3.0643	2.8	0.1662	1.7992
4/17/2019 23:54	0.9	0.7083	4.9623	0.4333	3.0865	2.9	0.1562	1.8108
4/17/2019 23:55	0.925	0.0583	4.9972	0.7667	3.0833	2.9	0.156	1.8149
4/17/2019 23:56	1	-0.2167	4.9262	0.4333	3.1053	2.9	0.1634	1.8203
4/17/2019 23:57	0.95	0.0167	4.8005	0.4	3.0743	2.9	0.1485	1.8231
4/17/2019 23:58	0.9	0.1083	4.7733	0.5167	3.0777	2.9	0.1574	1.8009
4/17/2019 23:59	0.9167	0.7667	4.858	-0.0333	3.105	2.9	0.1583	1.7908
4/18/2019 0:00	1	1.0917	4.963	0.6167	3.11	2.9833	0.1448	1.7834

4/18/2019 0:01	1	1.15	4.9758	1.0333	3.1648	3	0.1626	1.7771
4/18/2019 0:02	0.9417	0.4417	4.7403	0.1167	3.1529	3.0182	0.1489	1.7826
4/18/2019 0:03	0.9	0.575	4.6837	0.5667	3.1839	3.0636	0.1649	1.758
4/18/2019 0:04	0.9	0.2	4.6055	-0.2	3.1692	3.1	0.154	1.7694
4/18/2019 0:05	0.9	0.3083	4.6938	0.1167	3.1608	3.075	0.1494	1.8059
4/18/2019 0:06	0.9	0.225	4.5393	0.6	3.2217	3	0.1622	1.821
4/18/2019 0:07	0.8417	0.2	4.6753	0.2667	3.2103	3	0.1491	1.8182
4/18/2019 0:08	0.8	0.425	5.0648	0.0333	3.2495	3	0.1558	1.8304
4/18/2019 0:09	0.8	0.3583	4.6558	-0.0167	3.2383	3	0.1551	1.8188
4/18/2019 0:10	0.7667	0.6083	4.534	0.3833	3.264	3.0636	0.1475	1.7889
4/18/2019 0:11	0.7	0.5	4.7975	0.2333	3.3404	3.1	0.1658	1.7643
4/18/2019 0:12	0.7	0.2417	4.6937	0.5333	3.356	3.1	0.1509	1.7728
4/18/2019 0:13	0.7	0.0333	4.7822	0.0167	3.2915	3.07	0.1566	1.7993
4/18/2019 0:14	0.6833	0.5833	4.6007	0.3167	3.3692	3	0.1578	1.7866
4/18/2019 0:15	0.6	0.725	4.5558	0.3167	3.4027	3	0.1555	1.7793
4/18/2019 0:16	0.6	-0.1083	4.6698	-0.0333	3.4127	3	0.161	1.7779
4/18/2019 0:17	0.5917	0.125	4.5928	0.3333	3.4272	3	0.146	1.8015
4/18/2019 0:18	0.6	0.5917	4.8463	0.6167	3.4213	3	0.1638	1.8134
4/18/2019 0:19	0.6	0.025	4.6	-0.2	3.4419	3	0.1601	1.7941
4/18/2019 0:20	0.6	0.7417	4.6928	0.2833	3.5377	3	0.1574	1.7753
4/18/2019 0:21	0.5417	-0.1583	4.4913	-0.15	3.5267	3	0.163	1.7845
4/18/2019 0:22	0.5	-0.125	4.7442	0.2	3.5998	3	0.1501	1.7637
4/18/2019 0:23	0.5	0.8583	4.5248	0.3667	3.5389	3	0.1675	1.8205
4/18/2019 0:24	0.5	0.7833	4.5733	0.4833	3.5413	3	0.1584	1.8107
4/18/2019 0:25	0.5	0.8583	3.8198	-0.1	3.5457	3	0.1553	1.7975
4/18/2019 0:26	0.5	0.0667	4.7347	0.5833	3.5672	3	0.1663	1.7868
4/18/2019 0:27	0.55	0.4167	4.6143	0.3333	3.6377	3	0.1556	1.7964
4/18/2019 0:28	0.6	-0.2417	4.3385	0.3	3.6713	3	0.1707	1.7902
4/18/2019 0:29	0.6	0.8583	4.4522	0.8167	3.6539	3	0.1644	1.8263
4/18/2019 0:30	0.625	0.3417	4.3168	0.55	3.7146	3	0.1528	1.8398
4/18/2019 0:31	0.65	0.225	4.3627	-0.3167	3.743	3	0.1671	1.8129
4/18/2019 0:32	0.7	0.2083	4.736	-0.1	3.7373	3	0.1536	1.8069
4/18/2019 0:33	0.6417	0.0333	4.2447	-0.45	3.7058	3	0.1628	1.8018
4/18/2019 0:34	0.6083	-0.175	4.4452	-0.0333	3.7266	3	0.1496	1.8234
4/18/2019 0:35	0.6	0.3667	4.27	0.0333	3.7746	3	0.1633	1.8134
4/18/2019 0:36	0.6	1.0667	4.5088	0.1167	3.7509	3	0.1709	1.8087
4/18/2019 0:37	0.6333	0.1667	4.3653	0.0667	3.8508	3	0.1519	1.8027
4/18/2019 0:38	0.7	0.25	4.27	0.0167	3.8682	3	0.1678	1.7938
4/18/2019 0:39	0.7	0.6167	4.5172	-0.2167	3.8255	3.0091	0.1545	1.7783
4/18/2019 0:40	0.7	-0.6167	3.4375	-0.0833	3.8965	3.02	0.1661	1.7568
4/18/2019 0:41	0.7	-0.3167	3.3295	-0.1667	3.903	3	0.1622	1.777
4/18/2019 0:42	0.7	0.5583	4.8568	-0.25	3.8995	3	0.1574	1.7932
4/18/2019 0:43	0.6917	-0.1917	4.7455	0.3	3.8406	3	0.1676	1.7993
4/18/2019 0:44	0.6	0.175	3.921	0.3	3.8413	3	0.1556	1.8018
4/18/2019 0:45	0.6083	0.3583	4.5743	-0.2667	3.8345	3	0.1585	1.8155
4/18/2019 0:46	0.6417	0.025	4.6848	-0.3167	3.8798	3	0.1613	1.8167
4/18/2019 0:47	0.6333	-0.225	4.0365	-0.55	3.8636	3	0.1576	1.8078

4/18/2019 0:48	0.6	0.3417	3.25	0.0833	3.8913	3	0.1713	1.7798
4/18/2019 0:49	0.6	0.3417	4.109	0.6333	3.9174	3	0.1559	1.8021
4/18/2019 0:50	0.6	0.5417	4.0238	0.15	3.8856	3	0.1595	1.815
4/18/2019 0:51	0.6	0.625	4.4558	-0.2	3.8634	3	0.1649	1.8008
4/18/2019 0:52	0.6	0.35	4.1933	-0.05	3.9012	3	0.1477	1.7976
4/18/2019 0:53	0.6	0.5833	4.1398	-0.3167	3.8495	3	0.1677	1.8075
4/18/2019 0:54	0.5333	0.5667	4.54	-0.2833	3.9019	3	0.16	1.8258
4/18/2019 0:55	0.5	0.0667	4.4863	-0.2833	3.8945	3	0.1609	1.8061
4/18/2019 0:56	0.5	0.35	5.0468	-0.0833	3.869	3	0.1674	1.8295
4/18/2019 0:57	0.5	0.4167	4.191	0.3	3.8771	3	0.1511	1.8467
4/18/2019 0:58	0.5	-0.1917	4.236	-0.25	3.842	3	0.1677	1.8139
4/18/2019 0:59	0.5083	0.0667	4.227	0.1667	3.8529	3	0.1614	1.7744
4/18/2019 1:00	0.5917	-0.2833	4.0298	0.6167	3.8846	3	0.1628	1.8065
4/18/2019 1:01	0.6	-0.1833	4.2213	-0.0333	3.8802	3	0.1669	1.8311
4/18/2019 1:02	0.6	-0.0333	5.2283	0.1667	3.8482	3	0.152	1.8013
4/18/2019 1:03	0.6	0.275	4.0244	0.4833	3.8485	3	0.1651	1.8092
4/18/2019 1:04	0.6	-0.1583	4.3152	-0.1167	3.8633	3	0.1593	1.8199
4/18/2019 1:05	0.6	0.5583	4.3923	-0.1333	3.866	3	0.159	1.8228
4/18/2019 1:06	0.6	0.3333	4.0008	0.2	3.8636	3.0167	0.165	1.8311
4/18/2019 1:07	0.6	0.3083	4.3843	-0.3333	3.8566	3.1	0.1503	1.8302
4/18/2019 1:08	0.6	0.3417	4.3955	0.15	3.8796	3.1	0.1652	1.8466
4/18/2019 1:09	0.625	-0.0083	4.5413	0.0833	3.8688	3.1	0.156	1.8448
4/18/2019 1:10	0.7	0.35	4.639	0.5167	3.8841	3.1091	0.1576	1.8168
4/18/2019 1:11	0.7	0.725	4.3537	0.1667	3.8663	3.1778	0.1641	1.8388
4/18/2019 1:12	0.7	0.925	3.9903	-0.3833	3.8478	3.2	0.1515	1.8309
4/18/2019 1:13	0.7	0.7083	3.9163	-0.3167	3.8368	3.2	0.1721	1.8019
4/18/2019 1:14	0.7	0.1333	4.5145	0.0667	3.8301	3.2	0.1461	1.8
4/18/2019 1:15	0.7	0.1667	4.2265	0.5167	3.8231	3.2	0.17	1.7867
4/18/2019 1:16	0.7	0.1167	4.0581	-0.0167	3.8233	3.15	0.1602	1.7943
4/18/2019 1:17	0.7083	0.0667	4.4617	1.0833	3.8128	3.2	0.1564	1.805
4/18/2019 1:18	0.7167	0.8	4.0926	0.0667	3.8062	3.2	0.1676	1.8145
4/18/2019 1:19	0.7091	0.9417	4.3143	0.2	3.7875	3.2	0.1517	1.8208
4/18/2019 1:20	0.7583	-0.05	4.2708	0.1333	3.7901	3.2	0.1627	1.829
4/18/2019 1:21	0.7083	0.125	4.141	0.0333	3.7912	3.2	0.1691	1.8333
4/18/2019 1:22	0.7	0.3	4.0227	0.35	3.7722	3.2	0.1551	1.8339
4/18/2019 1:23	0.6833	0.0917	4.3295	0.0333	3.7795	3.1	0.1707	1.7969
4/18/2019 1:24	0.675	0.3417	4.4241	0.1667	3.7468	3.1	0.1559	1.8063
4/18/2019 1:25	0.7	0.0333	4.2511	-0.3	3.7498	3.1	0.1662	1.8188
4/18/2019 1:26	0.7	0.4	4.2352	0.05	3.7437	3.1	0.1655	1.8201
4/18/2019 1:27	0.7	-0.2417	4.6584	0.2167	3.7493	3.1	0.1511	1.801
4/18/2019 1:28	0.7	0.4917	4.3918	-0.45	3.7556	3.1	0.1677	1.8168
4/18/2019 1:29	0.7	0.0667	4.274	-0.1	3.7286	3.1	0.1552	1.8258
4/18/2019 1:30	0.7083	0.45	4.3492	0.2333	3.7414	3.0182	0.1619	1.8002
4/18/2019 1:31	0.7	0.3	4.467	-0.3167	3.7374	3	0.1568	1.8116
4/18/2019 1:32	0.7	0.6	4.4516	0.2833	3.7419	3	0.1603	1.7966
4/18/2019 1:33	0.675	0.0333	4.1899	-0.1333	3.7654	3	0.1649	1.765
4/18/2019 1:34	0.6667	0.1417	4.1653	0.4667	3.746	3	0.1508	1.788

4/18/2019 1:35	0.65	-0.3833	3.9859	0.6833	3.7466	3	0.1626	1.8031
4/18/2019 1:36	0.525	-0.1417	3.881	-0.0667	3.7549	3	0.1614	1.7906
4/18/2019 1:37	0.5083	0.25	4.0931	0.3833	3.7832	2.9545	0.1541	1.8533
4/18/2019 1:38	0.5	0.275	4.2718	0.6667	3.785	3	0.1632	1.8408
4/18/2019 1:39	0.5167	0.55	3.8088	0.2333	3.815	3	0.1534	1.8262
4/18/2019 1:40	0.5	-0.35	3.5981	0.4667	3.8183	3	0.1614	1.7967
4/18/2019 1:41	0.5	0.0833	3.9941	0.5333	3.8218	3.0364	0.1583	1.7705
4/18/2019 1:42	0.5167	0.55	4.2974	0.45	3.824	3.1	0.1549	1.7743
4/18/2019 1:43	0.5833	0.7417	4.0161	0.1167	3.8164	3.1	0.1671	1.7564
4/18/2019 1:44	0.5917	0.125	4.0563	-0.6833	3.7762	3.1	0.1479	1.7672
4/18/2019 1:45	0.6	0.3917	3.981	-0.3167	3.7638	3.1	0.1628	1.7913
4/18/2019 1:46	0.6	0.475	4.35	0.15	3.7736	3.1091	0.1595	1.8002
4/18/2019 1:47	0.525	0.1167	4.0461	-0.0333	3.77	3.2	0.1554	1.8168
4/18/2019 1:48	0.5	-0.1167	4.5797	0.6	3.7739	3.2	0.17	1.8088
4/18/2019 1:49	0.5	0.125	4.0363	0.25	3.7643	3.2	0.1501	1.8199
4/18/2019 1:50	0.5	0.2167	4.2282	0.1	3.7955	3.2	0.1648	1.7979
4/18/2019 1:51	0.4833	-0.1	4.1183	0.1	3.8007	3.1273	0.1544	1.7998
4/18/2019 1:52	0.5	0.025	4.1228	0.2833	3.7824	3.1	0.1605	1.7843
4/18/2019 1:53	0.5417	0.3167	4.1285	0.05	3.8113	3.1	0.1592	1.7713
4/18/2019 1:54	0.6	0.7417	4.4165	-0.05	3.8026	3.0818	0.1549	1.7955
4/18/2019 1:55	0.5083	0.2333	4.1352	0	3.8103	3	0.1628	1.8368
4/18/2019 1:56	0.5	0.1417	3.9494	0.65	3.7778	3	0.1534	1.8548
4/18/2019 1:57	0.55	0.3167	3.6352	-0.0833	3.8111	3	0.1517	1.827
4/18/2019 1:58	0.6	-0.3167	4.533	0.15	3.7637	3	0.1644	1.8222
4/18/2019 1:59	0.6	0.1667	3.5765	0.1333	3.7566	3	0.1573	1.7999
4/18/2019 2:00	0.6	0.1167	3.8538	-0.3333	3.7833	3	0.1739	1.8027
4/18/2019 2:01	0.6	-0.075	3.9569	0.25	3.7823	3	0.1552	1.8246
4/18/2019 2:02	0.6	0.0917	4.798	-0.3333	3.7566	3	0.1573	1.8146
4/18/2019 2:03	0.6	-0.025	4.0374	-0.0833	3.7748	3	0.1615	1.8047
4/18/2019 2:04	0.6	0.3083	4.1263	-0.0333	3.7618	3	0.1493	1.8339
4/18/2019 2:05	0.6	0.0417	3.7462	0.25	3.7603	3.0727	0.1618	1.8328
4/18/2019 2:06	0.6	-0.6417	3.6882	0.05	3.7401	3.1182	0.1558	1.8234
4/18/2019 2:07	0.6	-0.0083	3.9982	0.1333	3.7616	3.2	0.1533	1.8333
4/18/2019 2:08	0.5583	0.2167	3.8749	0.1833	3.7268	3.2	0.1636	1.855
4/18/2019 2:09	0.6	0.2667	4.2388	0.3667	3.7538	3.2	0.1505	1.8179
4/18/2019 2:10	0.6	0.2417	4.0217	-0.2667	3.7494	3.2	0.1607	1.8163
4/18/2019 2:11	0.6	0.8917	4.3538	-0.3	3.7568	3.2909	0.1644	1.8148
4/18/2019 2:12	0.5167	0.1917	4.1903	-0.1	3.7558	3.2889	0.1516	1.7988
4/18/2019 2:13	0.5	0.2833	4.0002	-0.4833	3.7402	3.28	0.1655	1.7794
4/18/2019 2:14	0.5	1.1167	3.7814	-0.5	3.7435	3.3	0.1504	1.8111
4/18/2019 2:15	0.5	0.0083	3.8792	-0.1	3.7663	3.27	0.1612	1.8371
4/18/2019 2:16	0.5	0.6333	3.9566	-0.1667	3.7725	3.2	0.1612	1.8307
4/18/2019 2:17	0.5	0.2	4.4617	-0.1833	3.7509	3.2	0.1577	1.8336
4/18/2019 2:18	0.5	0.3333	3.8347	-0.55	3.7743	3.2	0.1666	1.8017
4/18/2019 2:19	0.5	0.35	4.0957	-0.2	3.7681	3.1364	0.1562	1.7997
4/18/2019 2:20	0.5	0.5667	3.5909	-0.1	3.7959	3.2	0.1631	1.8105
4/18/2019 2:21	0.5167	-0.0917	3.9137	-0.4333	3.7966	3.2	0.1605	1.7969

4/18/2019 2:22	0.5667	-0.0833	4.0759	0.2333	3.8012	3.12	0.1565	1.801
4/18/2019 2:23	0.6	0.0583	4.027	-0.3167	3.8317	3.1	0.1709	1.8327
4/18/2019 2:24	0.6	0	3.4241	0.0667	3.8455	3.1	0.1511	1.8416
4/18/2019 2:25	0.6	0.525	3.358	-0.6	3.8533	3.1	0.1609	1.8369
4/18/2019 2:26	0.5167	0.5083	3.7423	-0.5	3.8482	3.1	0.164	1.8458
4/18/2019 2:27	0.5	0.6667	4.0208	-0.2167	3.8613	3.1	0.1574	1.82
4/18/2019 2:28	0.4833	0.3167	4.0155	0.1667	3.8528	3.1	0.1643	1.8088
4/18/2019 2:29	0.4	-0.0167	3.495	-0.1833	3.9209	3.1	0.15	1.7972
4/18/2019 2:30	0.4	0.075	3.7431	0.3	3.9077	3.1	0.1632	1.8052
4/18/2019 2:31	0.4167	-0.6583	3.8938	-0.1	3.8845	3.1	0.1607	1.7933
4/18/2019 2:32	0.45	-0.3417	3.9528	-0.5167	3.9181	3.1636	0.1572	1.8195
4/18/2019 2:33	0.425	0.225	4.0593	0	3.973	3.16	0.1666	1.8403
4/18/2019 2:34	0.4083	-0.025	3.6313	0.0833	4.0198	3.2	0.1503	1.819
4/18/2019 2:35	0.4	-0.15	3.8107	-0.4167	4.009	3.2	0.159	1.8328
4/18/2019 2:36	0.4	-0.2417	4.1592	-0.2667	4.0035	3.2	0.1618	1.8313
4/18/2019 2:37	0.4	-0.0583	3.7308	0.0167	3.9976	3.2	0.1539	1.8533
4/18/2019 2:38	0.3667	-0.3167	3.7865	-0.0167	4.0373	3.2	0.1669	1.8634
4/18/2019 2:39	0.3	-0.3583	3.7713	-0.1833	4.0524	3.2	0.1513	1.8498
4/18/2019 2:40	0.3	0.4833	3.844	-0.25	4.0643	3.2	0.1677	1.8548
4/18/2019 2:41	0.3	-0.2667	4.0137	-0.4833	4.0897	3.28	0.1641	1.8238
4/18/2019 2:42	0.3	-0.45	3.7147	-0.1667	4.1	3.3	0.153	1.8211
4/18/2019 2:43	0.2	0.4417	3.329	0.15	4.112	3.3	0.1689	1.8358
4/18/2019 2:44	0.2	-0.2333	3.8558	0.2333	4.1199	3.3	0.1525	1.8505
4/18/2019 2:45	0.2417	0.175	3.8208	-0.15	4.1531	3.3	0.1648	1.8386
4/18/2019 2:46	0.3	0.4917	4.347	-0.3167	4.1704	3.3	0.1601	1.837
4/18/2019 2:47	0.3	0.55	3.6827	-0.3667	4.249	3.3	0.1577	1.8041
4/18/2019 2:48	0.3	-0.65	3.4087	-0.5333	4.2854	3.3	0.1706	1.8163
4/18/2019 2:49	0.3	-0.0083	3.5677	-0.75	4.2785	3.3	0.1546	1.844
4/18/2019 2:50	0.3	0.3083	4.222	-0.4167	4.3123	3.3	0.1636	1.8339
4/18/2019 2:51	0.3	-0.7667	2.7827	-0.5	4.3827	3.3	0.1651	1.8289
4/18/2019 2:52	0.3	0.2333	3.3598	0.1333	4.389	3.3	0.1595	1.8533
4/18/2019 2:53	0.2833	-0.1	3.3482	-0.55	4.4746	3.3	0.1713	1.8626
4/18/2019 2:54	0.2	-0.075	3.2532	-0.35	4.5214	3.3	0.1565	1.8471
4/18/2019 2:55	0.2	0.5917	3.1015	-0.5167	4.5372	3.3	0.1646	1.8317
4/18/2019 2:56	0.2	-0.2667	3.4937	-0.3667	4.4983	3.3	0.1611	1.8252
4/18/2019 2:57	0.2	-0.2167	3.7802	-0.0833	4.6198	3.3	0.1635	1.8364
4/18/2019 2:58	0.2	-0.1	3.586	0	4.6556	3.3	0.1727	1.8407
4/18/2019 2:59	0.2	0.1417	3.1063	0.0667	4.7505	3.3	0.158	1.8013
4/18/2019 3:00	0.2	-0.4833	3.4078	-0.3667	4.6877	3.3	0.1682	1.7878
4/18/2019 3:01	0.1417	-0.45	3.5877	-0.25	4.8283	3.33	0.1619	1.8064
4/18/2019 3:02	0.1083	0.05	3.919	0.2167	4.8748	3.4	0.1637	1.8281
4/18/2019 3:03	0.1917	-0.1167	3.0772	-0.35	4.8993	3.4273	0.1673	1.836
4/18/2019 3:04	0.2	-0.7917	3.2482	-0.05	4.9372	3.5	0.155	1.8188
4/18/2019 3:05	0.2	-0.4167	3.0995	0.3167	4.9868	3.5	0.1706	1.8278
4/18/2019 3:06	0.2	0.0333	3.4047	-0.3	4.9443	3.53	0.1612	1.8363
4/18/2019 3:07	0.1917	-0.5083	2.9272	-0.3333	5.0103	3.6	0.1605	1.7935
4/18/2019 3:08	0.2	-0.3167	3.4512	-0.4	4.9232	3.6	0.1715	1.8098

4/18/2019 3:09	0.2	0.1417	3.1173	0	5.0123	3.6	0.1571	1.8168
4/18/2019 3:10	0.2	0.325	2.9077	0.05	5.146	3.6	0.1651	1.8345
4/18/2019 3:11	0.2	0.2083	3.2445	-0.4833	5.059	3.6091	0.1645	1.8345
4/18/2019 3:12	0.2083	-0.925	3.9383	-0.4333	5.2373	3.7	0.1654	1.8203
4/18/2019 3:13	0.2	-0.9167	3.3273	-0.3833	5.2372	3.7	0.1739	1.8303
4/18/2019 3:14	0.2	-0.8667	2.9738	-0.4167	5.2979	3.7	0.1607	1.8477
4/18/2019 3:15	0.125	-0.0917	3.0995	-0.35	5.2618	3.7	0.1665	1.8476
4/18/2019 3:16	0.125	-0.5417	3.3725	-0.3167	5.339	3.7667	0.1634	1.8605
4/18/2019 3:17	0.125	0.275	3.4028	0.0667	5.2819	3.8	0.1587	1.854
4/18/2019 3:18	0.1	-0.25	3.2407	-0.7167	5.2744	3.8	0.1693	1.8431
4/18/2019 3:19	0.1	-0.2583	3.437	-0.05	5.3631	3.8	0.1556	1.8534
4/18/2019 3:20	0.1167	-0.55	3.7913	0.0833	5.4129	3.85	0.1703	1.8459
4/18/2019 3:21	0.1	0.1417	3.349	-0.8167	5.5255	3.9	0.1763	1.8441
4/18/2019 3:22	0.1	-0.1583	3.1328	-0.45	5.5758	3.9	0.1622	1.8235
4/18/2019 3:23	0.1	0.2417	3.389	-0.0167	5.4309	3.94	0.1736	1.8352
4/18/2019 3:24	0.1	-0.55	3.202	-0.2667	5.7407	4	0.1606	1.8191
4/18/2019 3:25	0.1	-0.2583	3.073	-0.5333	5.6543	4	0.1733	1.7998
4/18/2019 3:26	0.1	-0.3667	3.3237	-0.6333	5.5819	4	0.1659	1.7942
4/18/2019 3:27	0.1	0.2	3.079	0.55	5.6178	4	0.165	1.8333
4/18/2019 3:28	0.1	-0.4917	2.9937	-0.9667	5.6026	4	0.1744	1.8417
4/18/2019 3:29	0.1	0.125	3.4278	-0.2333	5.5925	4.01	0.1624	1.8788
4/18/2019 3:30	0.1	-0.2417	3.54	-0.3333	5.5738	4.1	0.167	1.886
4/18/2019 3:31	0.1	-1.5083	3.247	-0.6333	5.5519	4.1	0.1703	1.8473
4/18/2019 3:32	0.1833	0.475	2.7493	-0.6	5.737	4.1	0.1616	1.8164
4/18/2019 3:33	0.2	0.125	3.2558	-0.45	5.7647	4.1	0.1728	1.8184
4/18/2019 3:34	0.2	-0.35	3.1667	-0.5	5.7405	4.125	0.1582	1.8361
4/18/2019 3:35	0.15	-0.2417	3.0272	-0.3333	5.8671	4.2	0.1746	1.8395
4/18/2019 3:36	0.2	-0.025	3.3468	-0.1833	5.8978	4.2	0.1702	1.873
4/18/2019 3:37	0.2	-0.3417	3.025	-0.5833	5.8584	4.2	0.1674	1.8783
4/18/2019 3:38	0.2	-0.4167	2.966	-0.5667	5.803	4.2273	0.1734	1.8564
4/18/2019 3:39	0.2	-0.0333	2.9082	-0.7333	5.8981	4.2818	0.1612	1.8647
4/18/2019 3:40	0.2	0.1917	3.1433	-0.1833	5.9469	4.3	0.1786	1.855
4/18/2019 3:41	0.2	-0.725	3.2835	-0.2667	6.0015	4.3889	0.1611	1.8657
4/18/2019 3:42	0.2	-0.45	3.3993	-0.6	5.9596	4.4	0.1737	1.8718
4/18/2019 3:43	0.2083	0.0917	3.0245	0.1667	5.9873	4.4	0.1773	1.8715
4/18/2019 3:44	0.2	-0.4667	3.3032	-0.2667	5.979	4.4333	0.1653	1.8306
4/18/2019 3:45	0.2	0.0583	2.8082	-0.5833	5.9784	4.5	0.1776	1.7933
4/18/2019 3:46	0.2	-0.675	3.0153	-0.8667	5.9899	4.5	0.1671	1.806
4/18/2019 3:47	0.2	-0.5583	2.9513	-0.6167	6.0053	4.56	0.1799	1.8105
4/18/2019 3:48	0.2	-0.0583	2.9008	0.2167	6.0624	4.6	0.1746	1.8013
4/18/2019 3:49	0.2	-0.1667	3.6597	-0.1167	6.1423	4.6	0.1641	1.7913
4/18/2019 3:50	0.2	-0.4167	3.3418	-0.15	6.0603	4.5727	0.177	1.7985
4/18/2019 3:51	0.2	-0.5583	3.0498	-0.75	6.1778	4.6	0.1704	1.8329
4/18/2019 3:52	0.2	-0.5333	3.1632	-0.65	6.1	4.6	0.1776	1.8626
4/18/2019 3:53	0.2	-0.7833	3.0447	-0.2	6.1183	4.6	0.1742	1.8526
4/18/2019 3:54	0.2	-0.9667	2.7368	-0.55	6.1494	4.6	0.165	1.847
4/18/2019 3:55	0.2	-0.55	3.0728	-0.45	6.2352	4.62	0.183	1.8413

4/18/2019 3:56	0.2	-0.625	3.1128	-0.1667	6.1984	4.7	0.172	1.8645
4/18/2019 3:57	0.2	-0.5667	3.2372	-0.15	6.2076	4.7	0.1742	1.8514
4/18/2019 3:58	0.2	-0.5583	3.1057	0.1	6.2038	4.65	0.1765	1.8115
4/18/2019 3:59	0.1727	-0.45	3.131	-0.3833	6.2063	4.7	0.1681	1.8263
4/18/2019 4:00	0.175	-0.475	2.9647	-0.6	6.2431	4.7	0.1797	1.8314
4/18/2019 4:01	0.1	-0.2	3.1353	-0.3333	6.2563	4.7	0.1669	1.8334
4/18/2019 4:02	0.1	0.125	3.1174	-0.6	6.1875	4.7833	0.1768	1.7999
4/18/2019 4:03	0.1	-0.2167	3.0293	-0.3333	6.2156	4.8	0.18	1.8258
4/18/2019 4:04	0.1	0.1167	3.1177	-0.6333	6.3217	4.8	0.168	1.8506
4/18/2019 4:05	0.1	0.475	3.3218	-0.5333	6.2386	4.8	0.1815	1.8562
4/18/2019 4:06	0.1	-0.5333	2.7883	-0.3833	6.3072	4.8	0.1625	1.8432
4/18/2019 4:07	0.1	-1.425	3.0785	-0.1167	6.2162	4.8	0.1808	1.8409
4/18/2019 4:08	0.1	-0.2333	2.9412	0.1667	6.3178	4.9	0.1754	1.8538
4/18/2019 4:09	0.1	0.25	3.061	0.0667	6.3013	4.9	0.1625	1.863
4/18/2019 4:10	0.1	-0.1667	3.0017	-0.1167	6.3053	4.9	0.186	1.8629
4/18/2019 4:11	0.0333	-0.225	3.0445	-0.6667	6.2263	4.9	0.1707	1.844
4/18/2019 4:12	0	-0.025	3.2804	0.0333	6.2167	4.9	0.1774	1.8683
4/18/2019 4:13	0.0083	-0.5167	3.0238	-0.1167	6.3292	4.9	0.1753	1.8546
4/18/2019 4:14	0.1	-0.1583	3.0989	-0.4333	6.3718	4.9	0.1765	1.8368
4/18/2019 4:15	0.1417	-0.7	2.9543	-0.2167	6.3698	4.9	0.1759	1.8618
4/18/2019 4:16	0.125	-0.15	3.2778	-0.1333	6.3678	4.9	0.1694	1.846
4/18/2019 4:17	0.1	-1.0917	2.9801	-0.1833	6.3744	4.9	0.183	1.8527
4/18/2019 4:18	0.1	-0.5	2.858	-1.5833	6.433	4.9	0.1795	1.8531
4/18/2019 4:19	0.1	-0.925	2.819	0.0667	6.4534	4.9	0.1691	1.8593
4/18/2019 4:20	0.1833	0.25	3.0973	-0.25	6.5245	4.9	0.1824	1.8572
4/18/2019 4:21	0.2	-0.5417	3.1026	-0.5667	6.5349	4.9	0.1713	1.8422
4/18/2019 4:22	0.2	-0.725	2.9028	-0.4167	6.4801	4.9	0.1826	1.8624
4/18/2019 4:23	0.2	-0.9083	3.0637	-0.8	6.455	4.9182	0.1708	1.8743
4/18/2019 4:24	0.2	0.0583	3.0035	-0.7167	6.5083	5	0.1712	1.8404
4/18/2019 4:25	0.2	-0.575	3.2364	-0.6833	6.559	5.0455	0.179	1.8054
4/18/2019 4:26	0.2	-0.4333	2.6673	-0.9833	6.5233	5.1	0.1658	1.8393
4/18/2019 4:27	0.2083	-0.2667	3.2216	-0.3333	6.6124	5.2	0.1832	1.8486
4/18/2019 4:28	0.2	-0.3917	2.434	-0.6	6.6355	5.2167	0.1723	1.8594
4/18/2019 4:29	0.2333	0.1167	2.5863	-0.25	6.5568	5.3	0.1725	1.872
4/18/2019 4:30	0.2167	-0.3	3.0913	-0.3	6.5593	5.3727	0.1789	1.8714
4/18/2019 4:31	0.2	0.025	2.7178	-0.8667	6.5684	5.4	0.1667	1.8638
4/18/2019 4:32	0.1667	-0.0667	3.2084	-0.7167	6.617	5.4417	0.1809	1.8625
4/18/2019 4:33	0.1583	0.5583	3.3618	-0.7167	6.6513	5.5	0.1721	1.8683
4/18/2019 4:34	0.2	0.4583	3.3562	-1.2	6.6176	5.5833	0.177	1.8615
4/18/2019 4:35	0.2	-0.8333	3.0184	-0.8833	6.6726	5.6	0.176	1.872
4/18/2019 4:36	0.2	-0.225	3.1449	-0.5	6.6509	5.6455	0.1677	1.8653
4/18/2019 4:37	0.1667	-1.075	3.0835	-0.45	6.6788	5.7	0.1811	1.8463
4/18/2019 4:38	0.1	-0.25	2.7008	-0.4833	6.6863	5.7	0.1785	1.8718
4/18/2019 4:39	0.1	-0.7833	2.9412	-0.6833	6.8088	5.7455	0.1724	1.8848
4/18/2019 4:40	0.1	-0.025	2.7135	-0.5	6.6794	5.8	0.1818	1.886
4/18/2019 4:41	0.1333	-0.0833	2.1047	-0.3167	6.7007	5.8	0.1668	1.8855
4/18/2019 4:42	0.1	-0.25	2.6687	-0.8167	6.7717	5.8	0.1726	1.874

4/18/2019 4:43	0.0583	-0.35	2.435	-0.9333	6.7254	5.8	0.1735	1.8765
4/18/2019 4:44	0	-0.2667	3.0919	-0.9333	6.7401	5.8091	0.1729	1.8825
4/18/2019 4:45	0	-0.625	2.5408	-0.1167	6.739	5.9	0.1782	1.8976
4/18/2019 4:46	0.1	-0.25	3.0582	-0.4	6.738	5.9	0.1646	1.8717
4/18/2019 4:47	0.1	-0.7667	2.9315	-0.3167	6.6333	5.9	0.1798	1.8693
4/18/2019 4:48	0.025	0.2	3.1408	-0.6167	6.5998	5.9	0.1761	1.8861
4/18/2019 4:49	0	-1.2083	2.9005	-0.5667	6.6209	5.9	0.1725	1.8963
4/18/2019 4:50	0	-0.5583	2.8355	-0.8167	6.6064	5.9	0.1846	1.8868
4/18/2019 4:51	0	0.175	2.9073	-0.9167	6.6113	5.9	0.1699	1.9019
4/18/2019 4:52	0	-0.7333	3.1035	-1.1167	6.6318	5.92	0.1772	1.8904
4/18/2019 4:53	0.0667	0.3667	2.9363	-0.7833	6.6527	6	0.1743	1.8773
4/18/2019 4:54	0.1	-0.6583	3.1132	-0.4833	6.6839	6	0.1705	1.8766
4/18/2019 4:55	0.1	0.1167	2.7872	-0.55	6.6998	6	0.1792	1.8695
4/18/2019 4:56	0.075	-0.4417	3.0927	-0.8167	6.6206	6.0667	0.1679	1.8913
4/18/2019 4:57	0	-0.3417	3.016	-0.1167	6.6674	6.1	0.1786	1.8858
4/18/2019 4:58	0	-0.6	3.0613	-0.5	6.6201	6.1091	0.1756	1.8918
4/18/2019 4:59	0	-0.1917	3.1911	-0.7833	6.6843	6.2	0.1688	1.8807
4/18/2019 5:00	0.0833	-0.2083	2.7053	-0.2333	6.689	6.2	0.182	1.8712
4/18/2019 5:01	0.1	0.0833	2.8836	-0.6	6.6027	6.24	0.1651	1.8903
4/18/2019 5:02	0.1	0.05	2.8786	-0.4833	6.6577	6.3	0.1719	1.8603
4/18/2019 5:03	0.1	-0.25	3.1113	-0.9167	6.7227	6.3	0.179	1.8538
4/18/2019 5:04	0.0917	-0.2333	2.7937	-0.6333	6.6446	6.3818	0.1616	1.8484
4/18/2019 5:05	0.0833	-0.3	3.2422	-0.4667	6.6199	6.4	0.174	1.8568
4/18/2019 5:06	0.1	-0.6167	3.0729	-0.5667	6.6427	6.4	0.1631	1.8213
4/18/2019 5:07	0.1	-0.4583	3.1243	-0.4333	6.6463	6.4	0.1638	1.8073
4/18/2019 5:08	0.125	-0.8417	2.8342	-0.1667	6.611	6.4	0.1663	1.8638
4/18/2019 5:09	0.1	-0.2833	2.7703	-0.3667	6.6404	6.4455	0.1558	1.8556
4/18/2019 5:10	0.1833	-0.5167	3.1286	-1.2833	6.6388	6.4909	0.1652	1.8311
4/18/2019 5:11	0.1583	-0.55	3.1344	0.2667	6.6639	6.4	0.1562	1.7903
4/18/2019 5:12	0.15	-0.2333	2.9181	-0.6167	6.6228	6.4	0.156	1.8026
4/18/2019 5:13	0.2	-0.1	3.0527	0.2333	6.6671	6.45	0.156	1.8107
4/18/2019 5:14	0.2917	-0.1167	3.2444	-0.0667	6.6898	6.4	0.1493	1.8012
4/18/2019 5:15	0.3	-0.3	3.3859	-0.9667	6.6933	6.4	0.1591	1.8232
4/18/2019 5:16	0.2417	0.5583	2.8969	-0.5667	6.7173	6.4	0.1593	1.8258
4/18/2019 5:17	0.2	-0.5333	2.9528	-0.1833	6.712	6.4	0.1462	1.8005
4/18/2019 5:18	0.2	-0.2167	3.1983	-0.5833	6.7329	6.4	0.1577	1.8009
4/18/2019 5:19	0.2	-0.0667	2.8923	-0.2833	6.7128	6.4	0.1514	1.8255
4/18/2019 5:20	0.2	0.225	3.1632	-0.7	6.6834	6.4	0.1478	1.8224
4/18/2019 5:21	0.2	-0.35	3.1578	-0.2333	6.6598	6.4	0.1583	1.7972
4/18/2019 5:22	0.2	-0.125	2.9318	-0.6667	6.681	6.3909	0.145	1.8127
4/18/2019 5:23	0.2167	-0.3583	3.1334	-0.45	6.6433	6.3	0.1469	1.8008
4/18/2019 5:24	0.225	-0.725	2.9245	-0.2167	6.6591	6.3	0.1528	1.8058
4/18/2019 5:25	0.2417	-0.6	3.1935	-0.4	6.6853	6.3	0.1424	1.7913
4/18/2019 5:26	0.275	-0.3	2.9311	-0.1333	6.6337	6.2636	0.1545	1.7883
4/18/2019 5:27	0.3	-0.275	2.962	-0.3167	6.6273	6.3	0.1483	1.8003
4/18/2019 5:28	0.3	-0.3	2.9247	-0.1	6.636	6.3	0.1466	1.7956
4/18/2019 5:29	0.3	-0.1083	2.7205	-0.1167	6.6109	6.2909	0.1534	1.8288

4/18/2019 5:30	0.3	-0.5083	2.7115	-0.6333	6.6336	6.2	0.1435	1.794
4/18/2019 5:31	0.3	-0.15	2.9367	-0.3667	6.6013	6.1083	0.1536	1.779
4/18/2019 5:32	0.3	-0.2167	2.9447	-0.1333	6.5639	6.1	0.1536	1.8131
4/18/2019 5:33	0.3	-0.0667	2.8108	-0.8167	6.5722	6.1	0.1461	1.8378
4/18/2019 5:34	0.3	-0.6917	3.1375	-1.1833	6.6398	6.1	0.16	1.8343
4/18/2019 5:35	0.3	-0.7	2.8427	-0.6667	6.6304	6.1	0.1527	1.807
4/18/2019 5:36	0.3	0.3583	2.9633	-0.1833	6.5953	6.1	0.1544	1.8122
4/18/2019 5:37	0.3667	0.175	2.9652	-0.75	6.5742	6.1	0.1648	1.8573
4/18/2019 5:38	0.4	-0.1833	2.779	-0.2	6.5343	6.1	0.1499	1.8401
4/18/2019 5:39	0.4	-1.1417	2.7888	-0.25	6.57	6	0.1705	1.8396
4/18/2019 5:40	0.3417	-0.0083	2.8443	-0.9	6.5395	6	0.1637	1.8472
4/18/2019 5:41	0.3	0.1	3.0593	-0.5833	6.5324	6	0.155	1.8594
4/18/2019 5:42	0.3	-0.375	2.9215	-0.5833	6.551	6	0.1682	1.8498
4/18/2019 5:43	0.3	-0.1167	2.8057	-0.7333	6.5467	6	0.1623	1.8543
4/18/2019 5:44	0.2833	0	2.8572	-0.0833	6.5468	6	0.1639	1.8464
4/18/2019 5:45	0.225	-0.1583	2.9798	-0.0333	6.5567	6	0.1692	1.8483
4/18/2019 5:46	0.2167	0.4	3.1042	-0.1167	6.555	6	0.1604	1.8335
4/18/2019 5:47	0.2	-0.275	2.8878	-0.65	6.555	6	0.1702	1.8568
4/18/2019 5:48	0.2	-0.2333	2.9185	-0.3667	6.5287	6	0.1713	1.8606
4/18/2019 5:49	0.2417	0.4167	3.0353	-0.2333	6.4777	5.9273	0.1686	1.8462
4/18/2019 5:50	0.3	-0.925	2.9282	-0.25	6.4824	5.9182	0.1772	1.8641
4/18/2019 5:51	0.2583	-0.3333	3.129	-0.35	6.478	5.9	0.1703	1.877
4/18/2019 5:52	0.2	-0.2917	3.0808	-0.4667	6.5033	5.9	0.1743	1.8598
4/18/2019 5:53	0.2	-0.6917	2.5578	-0.2167	6.4831	5.9	0.1666	1.8556
4/18/2019 5:54	0.2	-0.4417	2.8665	-0.2	6.4801	5.9	0.1628	1.8558
4/18/2019 5:55	0.2	0.05	2.7515	0.05	6.4987	5.9	0.171	1.8768
4/18/2019 5:56	0.2	-0.2417	2.9185	-0.6333	6.4778	5.9	0.1733	1.887
4/18/2019 5:57	0.2	0.725	2.8493	-0.45	6.4768	5.9	0.1758	1.8926
4/18/2019 5:58	0.2	-0.45	2.8982	-0.5667	6.4725	5.9	0.186	1.878
4/18/2019 5:59	0.2	-0.1333	2.8688	-0.8167	6.4682	5.9	0.1708	1.8497
4/18/2019 6:00	0.2	-0.8083	2.8515	-0.2833	6.4773	5.9	0.1734	1.8261
4/18/2019 6:01	0.2	-0.225	2.7525	-0.05	6.5096	5.9	0.1779	1.8608
4/18/2019 6:02	0.2	-0.275	2.8173	-0.6167	6.4683	5.9333	0.1707	1.8864
4/18/2019 6:03	0.2	0.1333	2.8842	-0.3167	6.4923	6	0.1847	1.8638
4/18/2019 6:04	0.1333	0.0083	2.697	-0.4333	6.5443	6.0182	0.1772	1.8428
4/18/2019 6:05	0.1417	0.4667	3.1083	-1.1333	6.5321	6.1	0.1756	1.8521
4/18/2019 6:06	0.1667	0.7	2.6753	-1	6.5848	6.1	0.1863	1.8643
4/18/2019 6:07	0.1	0.1833	2.5143	-0.6167	6.6572	6.1667	0.1756	1.8723
4/18/2019 6:08	0.1	0.0083	2.6325	-0.6333	6.5919	6.2	0.1869	1.8787
4/18/2019 6:09	0.1	0.1833	2.6037	-0.45	6.656	6.2583	0.1791	1.9039
4/18/2019 6:10	0.1	-0.5333	2.9132	-0.7	6.6988	6.3364	0.178	1.8665
4/18/2019 6:11	0.1	0.0583	2.7013	-0.4167	6.7348	6.42	0.1868	1.847
4/18/2019 6:12	0.1	-0.2	3.0158	0.0167	6.7468	6.5	0.1764	1.8785
4/18/2019 6:13	0.1	0.0333	2.513	0.0833	6.7909	6.5818	0.1898	1.8903
4/18/2019 6:14	0.1	0.0833	2.3878	-0.5167	6.8367	6.6	0.1868	1.9018
4/18/2019 6:15	0.1	-0.3833	2.966	-0.45	6.8408	6.7	0.1831	1.9095
4/18/2019 6:16	0.1833	-0.6917	2.0735	-1.2667	6.9178	6.7364	0.1891	1.9288

4/18/2019 6:17	0.2	-0.6	2.735	-0.3833	7.0818	6.8364	0.182	1.8693
4/18/2019 6:18	0.2	-0.8417	2.6975	-0.2	7.0031	6.9	0.1903	1.8638
4/18/2019 6:19	0.2	-0.1667	2.5145	-0.3167	6.9331	7	0.1894	1.8866
4/18/2019 6:20	0.2	-0.2083	2.6495	-0.6667	6.9942	7.0111	0.185	1.911
4/18/2019 6:21	0.15	-0.4	2.7813	-0.1167	6.9951	7.1	0.1878	1.8871
4/18/2019 6:22	0.1667	-0.075	3.0277	-0.5833	7.0115	7.17	0.1776	1.8653
4/18/2019 6:23	0.2	-0.9583	2.7447	-0.3	7.0317	7.2	0.1842	1.8813
4/18/2019 6:24	0.2	-0.1417	2.6658	-0.45	7.0537	7.2909	0.1948	1.8741
4/18/2019 6:25	0.2	-0.725	2.7747	-0.55	7.0161	7.35	0.1779	1.8828
4/18/2019 6:26	0.1667	0.4667	2.49	-0.0333	7.0883	7.4143	0.1898	1.8873
4/18/2019 6:27	0.2	-1.35	2.5193	-0.65	7.1188	7.5	0.1897	1.8963
4/18/2019 6:28	0.2	-0.175	2.3722	-0.9	7.1458	7.6167	0.1853	1.843
4/18/2019 6:29	0.2	-0.0167	2.764	-0.1833	7.1438	7.71	0.1977	1.8534
4/18/2019 6:30	0.1727	-0.2167	2.5565	-0.35	7.1539	7.8273	0.1831	1.8772
4/18/2019 6:31	0.1167	0.7917	3.467	-0.3333	7.1921	7.9667	0.1911	1.8872
4/18/2019 6:32	0.1	-0.1	2.6718	-0.8	7.2221	8.0545	0.1954	1.8812
4/18/2019 6:33	0.1	-0.1167	2.445	-0.4667	7.2359	8.2182	0.1825	1.863
4/18/2019 6:34	0.1	-0.6833	2.5032	-0.9167	7.2388	8.3364	0.196	1.8665
4/18/2019 6:35	0.075	-0.825	2.3683	-0.5167	7.2889	8.4818	0.1862	1.8734
4/18/2019 6:36	0.1	-0.2	2.9363	-0.4667	7.2845	8.6455	0.1913	1.8821
4/18/2019 6:37	0.0583	-0.9667	2.6637	-0.2333	7.3267	8.8	0.2032	1.9038
4/18/2019 6:38	0.075	-0.075	2.7548	-1.4667	7.3421	8.975	0.1848	1.908
4/18/2019 6:39	0	0.125	2.575	-0.4833	7.4017	9.1545	0.1948	1.9237
4/18/2019 6:40	0	-0.6417	2.7035	-0.6333	7.4189	9.35	0.1984	1.9008
4/18/2019 6:41	0	-0.2333	2.5315	-0.6333	7.4676	9.5818	0.1918	1.891
4/18/2019 6:42	0.05	-0.3083	2.618	-0.6333	7.4843	9.8	0.2101	1.8798
4/18/2019 6:43	0.1	-0.075	2.579	-0.4	7.4513	9.9727	0.1848	1.8848
4/18/2019 6:44	0.1	-0.6417	2.4655	-0.5167	7.4897	10.175	0.1987	1.8853
4/18/2019 6:45	0.1	0.2083	2.327	-0.3167	7.6805	10.3917	0.2086	1.8648
4/18/2019 6:46	0.1	-0.325	2.5408	-0.2833	7.6362	10.6364	0.1997	1.8699
4/18/2019 6:47	0.1	-0.4833	2.661	-0.45	7.633	10.9167	0.208	1.8348
4/18/2019 6:48	0.1	-0.2917	2.634	-0.7833	7.633	11.2167	0.2036	1.8582
4/18/2019 6:49	0.1083	-0.8667	2.3213	-0.7167	7.7121	11.5583	0.218	1.8633
4/18/2019 6:50	0.2	-0.1083	2.4877	-0.65	7.6857	11.9091	0.2155	1.8781
4/18/2019 6:51	0.2	0.05	2.5582	-0.7833	7.7327	12.2455	0.2081	1.9195
4/18/2019 6:52	0.2	-0.0583	2.1105	-0.4333	7.6808	12.5636	0.2189	1.9051
4/18/2019 6:53	0.2	-0.4583	2.614	-1.1667	7.6722	12.9083	0.2171	1.9132
4/18/2019 6:54	0.2	-0.5167	2.5503	-0.85	7.6951	13.1556	0.2069	1.9005
4/18/2019 6:55	0.2	-0.2083	2.4827	-0.8333	7.6163	13.5182	0.2198	1.9369
4/18/2019 6:56	0.2	-0.25	2.516	-0.6333	7.6287	13.8583	0.2128	1.9358
4/18/2019 6:57	0.2083	-0.6	2.6388	-0.7333	7.6546	14.2091	0.2217	1.9613
4/18/2019 6:58	0.2	-0.15	2.5185	-0.4333	7.6879	14.3909	0.2201	1.9618
4/18/2019 6:59	0.2	0.0167	2.492	-0.9333	7.6353	14.5667	0.2123	1.9323
4/18/2019 7:00	0.2	-0.375	2.9518	-0.65	7.5523	14.71	0.2215	1.9646
4/18/2019 7:01	0.2	-0.2667	4.533	-0.8	11.4062	15.6583	0.2756	1.9765
4/18/2019 7:02	0.2	0.1667	6.1736	-0.35	17.3954	22.31	0.4835	1.9822
4/18/2019 7:03	0.1333	-0.725	12.3898	-0.4	20.8208	77.5167	2.2104	2.1276

4/18/2019 7:04	0.1	-0.1167	10.473	-1.2	18.321	59.2727	1.5613	2.0699
4/18/2019 7:05	0.1833	-0.425	11.8783	-0.65	20.452	58.4583	1.6664	2.0873
4/18/2019 7:06	0.15	-0.1	11.03	-0.35	19.5597	59.2909	1.6267	2.0745
4/18/2019 7:07	0.1167	-0.0417	10.8042	-0.1333	19.0188	59.1909	1.5759	2.0764
4/18/2019 7:08	0.1	-0.2583	10.9308	-0.6	19.3429	59.68	1.4281	2.0831
4/18/2019 7:09	0.1	-0.1333	10.7517	-0.0833	19.2928	58.8583	1.3754	2.0928
4/18/2019 7:10	0.1	-0.1583	10.6483	-0.05	18.8466	57.4455	1.2916	2.0928
4/18/2019 7:11	0.1	-0.25	10.5292	0.3333	18.6068	56.4917	1.2513	2.1105
4/18/2019 7:12	0.1	0.675	10.3875	-0.3	18.462	56.9	1.2423	2.1159
4/18/2019 7:13	0.1	0.0833	10.3825	-0.95	18.3258	56.9417	1.2354	2.0952
4/18/2019 7:14	0.1	-0.85	10.7175	-0.9333	18.5328	57.58	1.2528	2.1041
4/18/2019 7:15	0.1	-0.175	10.2264	-0.55	18.4353	57.3167	1.2653	2.1244
4/18/2019 7:16	0.1	-0.675	9.2743	-0.3833	17.6995	57.2636	1.2022	2.1188
4/18/2019 7:17	0.1	-0.7583	9.1526	-0.9	17.6736	57.1833	1.1899	2.1328
4/18/2019 7:18	0.1	-0.2833	8.9361	-0.8	17.3628	57.0636	1.1689	2.1077
4/18/2019 7:19	0.1	-0.1833	9.1047	-0.6833	17.6804	56.9273	1.2028	2.1151
4/18/2019 7:20	0.175	-0.8667	9.3039	-0.4833	17.6826	56.8182	1.2289	2.1487
4/18/2019 7:21	0.2	-0.4667	8.7714	-0.95	17.2908	56.7333	1.1843	2.1543
4/18/2019 7:22	0.1583	-0.1	8.9307	-0.2167	17.1683	56.6182	1.1737	2.1541
4/18/2019 7:23	0.1	0.175	8.9344	-0.2167	17.289	56.5333	1.2006	2.1611
4/18/2019 7:24	0.1	-0.1667	8.3079	0.0667	16.987	56.36	1.1685	2.1727
4/18/2019 7:25	0.1	0.3583	8.6842	-0.4167	16.7473	56.1833	1.1599	2.1743
4/18/2019 7:26	0.1	-0.6	8.2771	-0.7333	16.7146	56.0091	1.1547	2.1634
4/18/2019 7:27	0.1	0.3583	8.3652	0.2833	16.7789	55.85	1.1589	2.1553
4/18/2019 7:28	0.0417	-0.3833	8.3344	-0.6	16.6618	55.7636	1.174	2.1473
4/18/2019 7:29	0.0333	-0.3833	7.6171	-0.1167	16.2088	55.6583	1.1333	2.1227
4/18/2019 7:30	0.1	-0.05	7.4836	-0.1833	16.0663	55.58	1.1288	2.1162
4/18/2019 7:31	0.1917	-0.125	7.6293	-0.4167	16.0329	55.4917	1.1303	2.1029
4/18/2019 7:32	0.2	-1.05	7.8574	-0.5167	16.2209	55.3182	1.135	2.1218
4/18/2019 7:33	0.1	-0.1667	7.7553	-0.4167	16.1228	55.2083	1.1378	2.1507
4/18/2019 7:34	0.0333	0.15	7.5409	-0.45	15.8473	55.13	1.1187	2.1598
4/18/2019 7:35	0	-0.4917	7.6499	-1.1167	15.6679	54.9364	1.0928	2.1598
4/18/2019 7:36	0	-1.2167	7.1747	-0.5667	15.5378	54.71	1.1021	2.1625
4/18/2019 7:37	0	-0.5417	6.9656	0.0667	15.4398	54.5333	1.0739	2.1778
4/18/2019 7:38	0.075	0.5583	7.4048	-0.8167	15.4796	54.49	1.0947	2.1943
4/18/2019 7:39	0.1	-0.6167	7.0914	-0.8667	15.3301	54.3667	1.0779	2.173
4/18/2019 7:40	0.1	-0.4	6.9314	-0.4333	15.116	54.2	1.0548	2.1583
4/18/2019 7:41	0.1	-0.5917	6.8348	-0.15	14.9902	54.0455	1.067	2.1618
4/18/2019 7:42	0.1	-0.1083	7.0158	-0.8833	15.0357	53.9091	1.0614	2.159
4/18/2019 7:43	0.1	-0.7333	6.92	0.0833	14.9043	53.7546	1.0543	2.1649
4/18/2019 7:44	0.1	-0.2417	6.5593	-0.4833	14.8573	53.68	1.0647	2.1407
4/18/2019 7:45	0.1	-0.7167	6.5841	-0.7833	14.6076	53.48	1.033	2.1064
4/18/2019 7:46	0.1	-0.2	6.0294	-0.0167	14.4923	53.15	1.0162	2.1249
4/18/2019 7:47	0.0917	-0.3333	6.4081	-0.8833	14.5468	53	1.0502	2.1687
4/18/2019 7:48	0.1	-1.125	6.4029	-0.3167	14.5261	52.9	1.0337	2.143
4/18/2019 7:49	0.025	-0.1917	6.3923	-0.1333	14.525	52.8417	1.0178	2.1495
4/18/2019 7:50	0.0917	0.0917	6.2968	-0.4333	14.3358	52.625	1.02	2.1734

4/18/2019 7:51	0.1	-0.4833	6.2263	-0.3667	14.1035	52.4909	1.0192	2.166
4/18/2019 7:52	0.1	-0.175	6.3071	-0.7	14.122	52.325	1.0242	2.1728
4/18/2019 7:53	0.1	0.1667	6.0434	-0.15	14.0395	52.15	1.0003	2.185
4/18/2019 7:54	0.1083	-0.7917	6.2278	-0.2	13.888	52.025	1.0069	2.2107
4/18/2019 7:55	0.125	0.0417	6.2008	0.0333	13.7618	51.81	0.988	2.1986
4/18/2019 7:56	0.1333	-0.6083	5.8016	-0.1833	13.6863	51.525	0.9817	2.1582
4/18/2019 7:57	0.1	-0.7833	5.7972	0.0333	13.7399	51.36	0.9958	2.1906
4/18/2019 7:58	0.1	0.1417	5.7835	-0.0667	13.7268	51.275	0.9875	2.1998
4/18/2019 7:59	0.15	0.0333	6.0604	-0.1333	13.5951	51.12	0.9833	2.1722
4/18/2019 8:00	0.1917	0.05	5.8306	-0.2833	13.4743	50.9545	0.9814	2.142
4/18/2019 8:01	0.2	-0.5583	5.7458	-0.2333	13.3503	50.7417	0.9434	2.1431
4/18/2019 8:02	0.1167	-0.55	5.6492	-0.65	13.3209	50.4636	0.9657	2.1606
4/18/2019 8:03	0.1	-0.5917	5.7193	-0.5333	13.3232	50.1833	0.9599	2.17
4/18/2019 8:04	0.025	-0.65	5.7587	-0.5333	13.243	49.9583	0.9548	2.2118
4/18/2019 8:05	0.075	-0.675	5.4778	-0.45	13.0698	49.7	0.9634	2.1905
4/18/2019 8:06	0.1	-0.2417	5.4653	0.2833	13.1015	49.5333	0.9327	2.2212
4/18/2019 8:07	0.1583	0.2833	5.2809	-0.8833	12.9728	49.4	0.9292	2.1926
4/18/2019 8:08	0.2	-0.3667	5.5574	-0.7333	13.0373	49.2091	0.9332	2.17
4/18/2019 8:09	0.2	-0.35	5.6424	-0.75	12.9759	49.0636	0.9348	2.1784
4/18/2019 8:10	0.2	-0.5667	5.5923	-0.45	12.8267	48.9182	0.9389	2.1835
4/18/2019 8:11	0.2	-0.45	5.3311	-0.9833	12.7282	48.6583	0.9136	2.2165
4/18/2019 8:12	0.1417	0.1083	4.9132	-0.6	12.7224	48.35	0.9102	2.1988
4/18/2019 8:13	0.2	0.3417	4.8531	-0.4	12.6659	48.1667	0.9046	2.1749
4/18/2019 8:14	0.1833	0.05	4.9585	-0.25	12.594	48.0455	0.911	2.1919
4/18/2019 8:15	0.125	-0.75	5.1633	-0.95	12.5693	47.8818	0.9026	2.2144
4/18/2019 8:16	0.1	0.1167	4.9277	-0.55	12.5375	47.7445	0.9064	2.2188
4/18/2019 8:17	0.1	-0.375	5.046	-0.4167	12.4482	47.5182	0.8888	2.1843
4/18/2019 8:18	0.0917	-0.675	4.7905	-0.2667	12.4078	47.2546	0.8925	2.2309
4/18/2019 8:19	0.075	-0.4417	4.8823	-0.95	12.4322	47.05	0.8906	2.2483
4/18/2019 8:20	0.1	-0.6333	4.9502	-0.7167	12.4058	46.9625	0.8856	2.2943
4/18/2019 8:21	0.1417	-0.1833	4.9242	-0.4167	12.321	46.7273	0.8924	2.2647
4/18/2019 8:22	0.1917	-0.3833	4.8865	-0.1333	12.3435	46.54	0.8704	2.2309
4/18/2019 8:23	0.2	-1.4417	4.7797	-0.5833	12.2178	46.3546	0.8676	2.2529
4/18/2019 8:24	0.2	0.2833	5.0358	-0.5667	12.1768	46.28	0.8755	2.2669
4/18/2019 8:25	0.2	-0.4	4.9983	-0.4833	12.1655	46.1091	0.8622	2.2728
4/18/2019 8:26	0.2	-0.6417	4.9142	-0.8833	12.1339	45.8727	0.8566	2.2675
4/18/2019 8:27	0.2	-0.3583	4.733	-0.25	12.0862	45.6091	0.852	2.2808
4/18/2019 8:28	0.15	-0.3167	4.7206	-1.2833	12.0953	45.4083	0.839	2.2988
4/18/2019 8:29	0.1	-0.8333	4.4893	-0.4833	11.973	45.275	0.8429	2.2638
4/18/2019 8:30	0.1083	-0.2583	4.6014	-0.7667	12.0008	45.125	0.8469	2.2303
4/18/2019 8:31	0.1583	0.1833	4.784	0	12.0172	44.9545	0.8267	2.2089
4/18/2019 8:32	0.1	-0.5583	4.7077	-1.1833	11.8188	44.8091	0.8463	2.2367
4/18/2019 8:33	0.1	-0.6917	4.3248	-0.7333	11.8953	44.61	0.8091	2.2403
4/18/2019 8:34	0.1	-0.15	4.1032	-0.2167	12.0828	44.4091	0.7977	2.2468
4/18/2019 8:35	0.2	0.225	4.4275	-0.45	13.4549	43.9273	0.7577	2.2838
4/18/2019 8:36	0.2	-0.6	4.5347	-0.7167	12.5138	43.6455	0.8058	2.274
4/18/2019 8:37	0.3	-0.025	4.342	-0.4833	14.0811	43.1273	0.7484	2.2597

4/18/2019 8:38	0.2833	-0.325	4.0223	-0.5333	15.0998	42.4167	0.7009	2.2331
4/18/2019 8:39	0.3333	0.3	3.9487	0.0333	16.0312	41.5	0.652	2.2148
4/18/2019 8:40	0.4273	0.175	3.5223	0.2333	17.116	40.375	0.6252	2.1908
4/18/2019 8:41	0.5	0.1333	3.2798	0.2833	17.7269	39.12	0.5815	2.1591
4/18/2019 8:42	0.5417	0.45	3.4322	-0.0167	18.0313	37.6727	0.582	2.149
4/18/2019 8:43	0.5417	0.175	3.0832	0.45	19.1546	36.22	0.5303	2.139
4/18/2019 8:44	0.525	0.3917	2.6138	-0.1	19.6757	34.4636	0.5078	2.1267
4/18/2019 8:45	0.6	-0.1167	2.8333	0.7	20.3156	32.65	0.4869	2.1088
4/18/2019 8:46	0.6	0.25	2.8633	0.2	20.5143	30.725	0.4777	2.109
4/18/2019 8:47	0.7	-0.2333	2.806	-0.05	20.9998	28.8636	0.4504	2.0864
4/18/2019 8:48	0.7	0.95	2.9775	0.2167	21.4051	26.95	0.4504	2.0819
4/18/2019 8:49	0.7	0.5333	2.8548	0.4667	21.6276	25.0083	0.4109	2.0422
4/18/2019 8:50	0.75	0.7417	3.0143	0	21.633	23.31	0.4062	2.0249
4/18/2019 8:51	0.8	0.9167	2.597	0.4667	21.7587	21.9091	0.4113	2.0551
4/18/2019 8:52	0.8	0.4917	2.5968	0.7167	22.2108	20.4364	0.3843	2.0535
4/18/2019 8:53	0.8	0.875	2.4667	0.35	22.4546	19.2583	0.3863	2.0339
4/18/2019 8:54	0.8333	1.225	2.6548	0.7833	22.5865	18	0.3615	2.0118
4/18/2019 8:55	0.925	1.2083	2.4977	1	22.6354	16.9	0.362	1.9768
4/18/2019 8:56	1	1.4833	2.7447	1.05	22.7858	15.8455	0.3583	1.9801
4/18/2019 8:57	1.075	1.0667	2.7315	1.15	22.7777	15	0.342	1.9701
4/18/2019 8:58	1.1	1.3417	2.9933	1.0167	22.8636	14.2091	0.3525	1.9515
4/18/2019 8:59	1.2	1.2167	2.89	1.0833	22.9023	13.5273	0.354	1.9767
4/18/2019 9:00	1.2917	1.95	2.574	0.7667	22.8268	12.8667	0.3382	1.9382
4/18/2019 9:01	1.3333	1.8417	3.0948	1.1	22.8543	12.275	0.3398	1.941
4/18/2019 9:02	1.3	1.3083	2.856	1.05	22.8558	11.7546	0.3385	1.9243
4/18/2019 9:03	1.3	0.8583	2.9672	0.8333	22.9454	11.2182	0.3266	1.9062
4/18/2019 9:04	1.375	1.4333	2.8605	1	23.0833	10.7	0.3306	1.8825
4/18/2019 9:05	1.4	0.975	2.831	0.9333	23.0863	10.1546	0.3212	1.8821
4/18/2019 9:06	1.3083	1.225	2.6145	1.05	23.1196	9.775	0.3162	1.8935
4/18/2019 9:07	1.125	1.4083	2.3062	0.55	23.1134	9.4	0.3226	1.9132
4/18/2019 9:08	0.9083	0.8583	2.455	0.5667	23.0658	9.0364	0.3226	1.922
4/18/2019 9:09	0.7417	0.3417	2.2273	-0.0167	23.1243	8.7455	0.3229	1.9548
4/18/2019 9:10	0.6273	-0.0083	2.076	0.0167	23.0622	8.45	0.3302	1.9678
4/18/2019 9:11	0.5917	-0.0167	2.131	0.6667	22.9778	8.2273	0.3193	1.9677
4/18/2019 9:12	0.5	0.4333	1.972	-0.35	22.8778	8.0556	0.335	1.976
4/18/2019 9:13	0.4333	0.0583	1.8473	-0.2833	22.8623	7.8636	0.3354	1.9727
4/18/2019 9:14	0.4	-0.3	1.9015	-0.0167	22.7746	7.7455	0.3273	1.9857
4/18/2019 9:15	0.4	-0.3583	2.012	-0.6833	22.9078	7.65	0.3381	2.0014
4/18/2019 9:16	0.3167	0.125	3.6877	-0.0167	28.5683	8.39	0.3791	2.0469
4/18/2019 9:17	0.2833	-0.0167	34.2	0.9667	45.3118	13.7833	0.9802	2.2928
4/18/2019 9:18	0.2	-0.3667	44.4133	1.4167	60.2518	61.04	1.2877	2.3973
4/18/2019 9:19	0.2	-0.2583	53.1483	1.6667	62.3835	77.0364	1.4945	2.4399
4/18/2019 9:20	0.1833	0.6417	65.7567	1.95	66.5143	83.3636	1.7597	2.5385
4/18/2019 9:21	0.2	-0.225	68.71	1.9	68.8968	89.0583	1.8787	2.5971
4/18/2019 9:22	0.2	-0.4083	75.1917	1.8333	70.8758	93.4	2.1312	2.6716
4/18/2019 9:23	0.2	-0.1917	76.2667	1.75	71.3281	95.175	2.213	2.7153
4/18/2019 9:24	0.1083	0.3833	86.35	2.7833	73.9398	105.8909	3.0753	2.9111

4/18/2019 9:25	0.1	0.1917	92.5133	1.6833	75.1908	119.375	4.32	3.2035
4/18/2019 9:26	0.1	-0.3333	85.625	1.4833	73.6572	112.5091	3.611	2.9829
4/18/2019 9:27	0.1917	0.5333	80.4667	1.8333	70.0694	107.7833	4.0732	2.9807
4/18/2019 9:28	0.2	0.5417	78.99	1.55	69.2136	105.4727	3.9338	2.9738
4/18/2019 9:29	0.2	-0.2	76.8717	2.0833	68.2656	107.7917	4.1407	2.9601
4/18/2019 9:30	0.2	-0.7167	74.1967	1.5833	66.9718	107.4455	4.5538	2.9933
4/18/2019 9:31	0.2	-0.3167	72.1967	1.1333	65.6195	107.1583	4.2872	2.95
4/18/2019 9:32	0.2	-0.4667	71.9733	0.9	65.4157	106.8917	4.2457	2.9519
4/18/2019 9:33	0.2	-0.175	72.005	0.5167	65.5986	106.6364	4.3127	2.9527
4/18/2019 9:34	0.1583	-0.2583	70.7433	1.1667	64.8307	106.2667	4.3168	2.9363
4/18/2019 9:35	0.1	-0.0667	68.13	0.8833	63.756	105.9636	4.2885	2.938
4/18/2019 9:36	0.1083	0.1833	67.4483	1.1333	63.1215	105.5273	4.0717	2.9476
4/18/2019 9:37	0.1	0.0667	67.3533	1.2167	62.918	105.1545	4.0683	2.9622
4/18/2019 9:38	0.1	-0.3583	65.635	0.6167	62.1266	104.7545	4.233	3.0069
4/18/2019 9:39	0.1083	-0.4833	63.0833	1.1	60.074	104.1455	4.2263	2.9747
4/18/2019 9:40	0.1545	0.175	63.0367	0.5833	60.2646	103.65	4.4423	2.9677
4/18/2019 9:41	0.1	-0.2417	61.8867	1.5167	59.5696	103.1167	4.2445	2.9294
4/18/2019 9:42	0.1	-0.6167	61.1683	0.6833	59.0364	102.5818	4.1098	2.9533
4/18/2019 9:43	0.1	0.1	60.7383	0.6833	58.6699	102.3083	4.1357	2.9625
4/18/2019 9:44	0.1083	-0.125	57.755	0.8167	56.7449	101.8333	4.1029	2.923
4/18/2019 9:45	0.1	-1.1083	56.3683	0.9167	55.8288	101.1818	3.9323	2.8968
4/18/2019 9:46	0.0167	-0.15	55.2883	0.2667	54.8368	100.45	4.0219	2.8716
4/18/2019 9:47	0.0167	0.2417	54.9033	0.3167	54.5348	99.8546	4.1231	2.8988
4/18/2019 9:48	0.1	-0.4167	54.2967	1.0333	54.1026	99.2546	4.0158	2.9408
4/18/2019 9:49	0.1333	-0.0083	53.27	0.5167	53.5674	98.68	3.9599	2.9108
4/18/2019 9:50	0.2	-0.425	51.9783	-0.0333	52.8048	97.9364	3.9593	2.9092
4/18/2019 9:51	0.2	-0.4083	51.2867	0.5667	52.833	97.45	3.9004	2.9009
4/18/2019 9:52	0.175	-0.5917	50.4867	0.7167	52.7053	96.8	3.9775	2.9013
4/18/2019 9:53	0.0917	-0.5333	49.8317	0.15	50.9916	96	3.9063	2.9063
4/18/2019 9:54	0	0.1417	49.3583	0.5333	50.1502	95.3333	3.8579	2.9107
4/18/2019 9:55	0	0.0167	48.535	0.0833	49.7073	94.7667	3.8063	2.9256
4/18/2019 9:56	0	-0.1833	47.39	0.05	48.6338	94.0583	3.7753	2.8792
4/18/2019 9:57	0.0083	-0.275	47.76	0.8	49.1748	93.57	3.7977	2.8859
4/18/2019 9:58	0.1	-0.05	46.0317	0.5667	47.4668	93.2	3.7099	2.8843
4/18/2019 9:59	0.1	0.2167	45.875	0.8333	47.4834	92.66	3.7147	2.906
4/18/2019 10:00	0.1083	0.625	45.6583	0.3	47.3009	92.0583	3.6823	2.8999
4/18/2019 10:01	0.1	0.4	44.2492	0.6	46.4521	91.46	3.6398	2.8704
4/18/2019 10:02	0.1	0.6833	44.09	0.75	46.1523	90.9333	3.6477	2.8375
4/18/2019 10:03	0.1	0.05	43.2	0.2833	45.4539	90.24	3.5993	2.8681
4/18/2019 10:04	0.1	-0.3917	42.7517	0.5167	44.9617	89.525	3.5845	2.8786
4/18/2019 10:05	0.1	0.1083	42.9642	0.3167	44.9945	89.11	3.6019	2.8724
4/18/2019 10:06	0.1	-0.4833	40.4767	0.0833	43.3009	88.425	3.4647	2.8613
4/18/2019 10:07	0.0083	-0.325	41.3192	-0.3333	43.1463	87.7364	3.5027	2.8764
4/18/2019 10:08	0.0083	0.0083	40.7067	0.1333	42.9492	87.1083	3.4828	2.8914
4/18/2019 10:09	0.0417	-0.2417	40.8433	0.5333	43.0922	86.64	3.5099	2.9243
4/18/2019 10:10	0.0667	0.15	39.89	0.05	42.3376	86.1273	3.4733	2.9039
4/18/2019 10:11	0.0583	-0.2333	38.8725	0.0667	41.3932	85.66	3.4156	2.8812

4/18/2019 10:12	0.1	-0.1417	37.4967	0.4833	40.4111	85.02	3.3544	2.8575
4/18/2019 10:13	0.1	-0.25	38.26	-0.05	40.9553	84.4455	3.4036	2.8793
4/18/2019 10:14	0.1083	0.025	37.3775	0.05	40.0196	83.8	3.3373	2.8705
4/18/2019 10:15	0.1	0.2083	36.7208	0.15	39.5953	83.2182	3.3327	2.8818
4/18/2019 10:16	0.1333	-0.0583	36.9708	0.3	39.2141	82.7667	3.2968	2.869
4/18/2019 10:17	0.1	-0.3167	36.0308	0.5667	38.8688	82.2	3.2826	2.8541
4/18/2019 10:18	0.1	-0.3667	35.7425	0.2833	38.4894	81.6	3.2789	2.8621
4/18/2019 10:19	0.1	-0.4167	34.9692	-0.1	37.954	81.19	3.2387	2.8283
4/18/2019 10:20	0.1182	-0.1833	34.3292	0.1333	37.4966	80.6083	3.2313	2.8611
4/18/2019 10:21	0.1333	-0.4667	34.905	0.4833	37.4783	80.0455	3.2275	2.8437
4/18/2019 10:22	0.1	-0.2083	34.1742	0.0333	36.8128	79.3667	3.1974	2.8174
4/18/2019 10:23	0.1667	-0.4917	33.4492	0.2333	36.4118	78.7091	3.1734	2.8065
4/18/2019 10:24	0.2	-0.2	33.8608	0.4667	36.5163	78.2083	3.1733	2.8314
4/18/2019 10:25	0.2	-0.225	33.3167	0.5667	35.7479	77.6091	3.1403	2.8357
4/18/2019 10:26	0.2	0.2167	33.0642	0.5167	35.6039	77.0833	3.1175	2.8303
4/18/2019 10:27	0.2	0.1917	32.6708	0	35.3618	76.65	3.1144	2.8612
4/18/2019 10:28	0.1083	-0.4667	32.1975	0.4167	34.8846	76.3083	3.1164	2.8573
4/18/2019 10:29	0.1	-0.4583	31.8142	0.1833	34.3804	75.79	3.0668	2.8315
4/18/2019 10:30	0.1	-0.025	30.9175	0.4	33.9335	75.2667	3.0482	2.8198
4/18/2019 10:31	0.1583	-0.05	30.9125	0.4167	33.5969	74.7333	3.0272	2.8591
4/18/2019 10:32	0.2	-0.6083	30.9433	0.2667	33.7478	74.35	3.0452	2.8718
4/18/2019 10:33	0.175	0.1583	30.805	0.9333	33.5591	73.9583	3.0398	2.86
4/18/2019 10:34	0.1333	0.6583	30.2475	0.6	33.1276	73.5583	3.004	2.8309
4/18/2019 10:35	0.125	0.1417	29.2817	-0.0333	32.1097	73.0364	2.9377	2.8416
4/18/2019 10:36	0.1083	-0.7333	29.5633	-0.6833	32.1401	72.62	2.9658	2.8493
4/18/2019 10:37	0.1	-0.4167	28.4567	0.35	31.6513	72.1833	2.9021	2.8211
4/18/2019 10:38	0.1667	0.2417	25.8167	0.3833	29.6138	71.3818	2.7289	2.7559
4/18/2019 10:39	0.2	-0.2833	24.2617	-0.2833	28.1952	70.1455	2.6166	2.6818
4/18/2019 10:40	0.2	0.1833	25.62	-0.3333	29.4743	69.15	2.6709	2.7258
4/18/2019 10:41	0.2	-0.2083	23.2892	-0.6	29.5918	67.9	2.4573	2.6325
4/18/2019 10:42	0.25	0.3	20.3142	0.1833	28.8116	65.8917	2.1355	2.4744
4/18/2019 10:43	0.5	1.6833	18.0908	1.35	27.223	63.2667	1.8555	2.364
4/18/2019 10:44	1.2583	3.25	14.325	2.2	22.6918	59.6667	1.3482	2.106
4/18/2019 10:45	1.8667	2.8167	13.6267	2.3167	22.9888	55.9091	1.3155	2.061
4/18/2019 10:46	2.15	2.7	13.0133	1.7667	23.7971	51.6091	1.319	2.0612
4/18/2019 10:47	2.0417	3.0333	12.3008	2.1667	22.907	47.7	1.2048	2.0442
4/18/2019 10:48	2.2167	4.0583	11.5042	3.0333	21.8503	43.1636	1.0647	1.9858
4/18/2019 10:49	2.6833	3.525	11.0033	3.45	20.9896	38.6917	0.978	1.9015
4/18/2019 10:50	2.9333	3.6917	10.76	2.9667	20.9469	34.5455	1.1024	1.9284
4/18/2019 10:51	3.0833	3.0583	10.6942	3.7833	21.0792	30.8909	1.0131	1.8976
4/18/2019 10:52	3.2333	3.6667	10.2228	3.85	20.905	26.7083	0.9231	1.8662
4/18/2019 10:53	3.525	4.45	9.6612	4.15	20.714	22.78	0.8664	1.8629
4/18/2019 10:54	4.05	5.0917	10.0748	5.3667	18.6739	19.4917	0.7952	1.8316
4/18/2019 10:55	4.3583	4.9333	10.067	5.0667	18.1642	16.8667	0.7528	1.8305
4/18/2019 10:56	4.9833	6.8917	10.65	6.4	16.7801	15.3167	0.6768	1.7718
4/18/2019 10:57	5.45	6.175	10.3665	6.0833	16.238	14.1	0.6442	1.7578
4/18/2019 10:58	6.0833	7.65	11.0775	8.5833	15.2371	12.7091	0.5822	1.7368

4/18/2019 10:59	6.4917	7.5583	10.7	7.85	14.7696	11.4583	0.5762	1.705
4/18/2019 11:00	6.8917	7.9	11.1517	8.4	14.3416	10.31	0.5383	1.6878
4/18/2019 11:01	7.2167	8.0167	11.085	8.2	13.4799	9.6273	0.4989	1.6573
4/18/2019 11:02	7.575	8.85	11.9025	8.7833	13.1593	8.9364	0.494	1.6453
4/18/2019 11:03	8.025	10.1917	12.4133	9.4	12.2757	8.1167	0.4477	1.6634
4/18/2019 11:04	8.5167	9.425	11.885	9.3667	11.7084	7.61	0.414	1.6658
4/18/2019 11:05	8.7583	8.6917	11.8967	8.9167	11.6586	7.2333	0.4226	1.6348
4/18/2019 11:06	9.125	10.5833	12.5508	10.1833	11.3059	6.6182	0.3889	1.6432
4/18/2019 11:07	9.2917	10.375	12.7558	10.5833	10.7888	6.1	0.3839	1.6368
4/18/2019 11:08	9.625	11	13.0175	11.15	10.4267	5.6727	0.365	1.6357
4/18/2019 11:09	9.7667	10.7167	13.0958	11.35	10.0228	5.6	0.3417	1.6066
4/18/2019 11:10	10.0333	12.3667	13.6492	12.1333	9.4326	5.6583	0.3385	1.6092
4/18/2019 11:11	10.2917	11.7333	13.4125	11.4	9.0958	5.8556	0.3213	1.6237
4/18/2019 11:12	10.6917	12.9167	13.9758	12.2667	8.9336	5.8167	0.3032	1.6157
4/18/2019 11:13	10.8583	11.9833	13.4333	12.4167	8.7051	5.64	0.3118	1.6166
4/18/2019 11:14	11.1917	12.7833	14.3925	13.25	8.4358	5.5182	0.2793	1.6122
4/18/2019 11:15	11.3	12.1167	14.37	12.8667	8.3573	5.5818	0.2877	1.6061
4/18/2019 11:16	11.65	13.175	14.7867	13.1833	8.1646	5.5818	0.2669	1.5823
4/18/2019 11:17	11.8917	13.7083	14.8158	13.4833	7.9766	5.4182	0.2578	1.5678
4/18/2019 11:18	12.2333	12.4	14.9225	13.4833	7.6647	5.325	0.2588	1.5823
4/18/2019 11:19	12.3583	13.2583	15.0642	13.5167	7.5839	5.35	0.2463	1.6143
4/18/2019 11:20	12.5417	14.2333	15.7467	14.15	7.3135	5.125	0.2489	1.6232
4/18/2019 11:21	12.8667	14.4667	16.0158	14.8833	7.0091	4.7727	0.2321	1.5763
4/18/2019 11:22	13.2667	14.65	16.3592	15.1833	6.8507	4.4167	0.2233	1.5956
4/18/2019 11:23	13.725	14.6833	16.6875	15.6167	6.6197	3.9	0.2299	1.6308
4/18/2019 11:24	13.9667	15.8583	16.6767	16.0833	6.4414	3.6583	0.2211	1.5869
4/18/2019 11:25	14.2	15.0833	16.9625	15.3333	6.3126	3.6273	0.2122	1.578
4/18/2019 11:26	14.3583	16.0417	16.7925	15.6333	6.2064	3.6917	0.2206	1.6005
4/18/2019 11:27	14.6417	16.05	17.1925	16.2333	5.9799	3.5091	0.1957	1.6118
4/18/2019 11:28	14.9167	16.0417	17.9283	17.0167	5.8291	3.2667	0.2031	1.5758
4/18/2019 11:29	15.2083	15.8667	17.8633	16.4833	5.696	3	0.1979	1.5568
4/18/2019 11:30	15.4333	15.775	17.935	17.3833	5.4981	3	0.1918	1.5588
4/18/2019 11:31	15.5333	16.0417	17.6233	16.8333	5.3571	2.9	0.2002	1.5798
4/18/2019 11:32	15.7417	17.0417	18.2983	17.35	5.2206	2.9636	0.1791	1.6046
4/18/2019 11:33	15.8667	16.525	17.87	17.15	5.0753	2.9333	0.1878	1.5864
4/18/2019 11:34	16.1167	17.6083	18.4783	17.2167	5.0568	2.85	0.1961	1.5713
4/18/2019 11:35	16.2917	16.9	18.4317	17.4667	4.8768	2.83	0.1718	1.5624
4/18/2019 11:36	16.4083	16.9667	18.5867	17.5333	4.8556	2.9417	0.1858	1.5893
4/18/2019 11:37	16.575	16.55	18.965	17.95	4.775	3.0545	0.1748	1.5678
4/18/2019 11:38	16.8667	17.1833	19.0483	18	4.5933	3.0917	0.1745	1.552
4/18/2019 11:39	17.3	18.675	19.6533	18.45	4.5544	3	0.1764	1.5394
4/18/2019 11:40	17.5833	17.7417	20.2083	18.5	4.5578	3.025	0.1627	1.5688
4/18/2019 11:41	17.6417	17.675	19.6817	18.0167	4.529	3.17	0.1783	1.5755
4/18/2019 11:42	17.5917	17.9083	19.69	18.9	4.4349	3.2	0.1704	1.5668
4/18/2019 11:43	17.45	18.05	19.5283	18.5833	4.3994	3.2636	0.164	1.5662
4/18/2019 11:44	17.425	17.45	19.405	18.9833	4.4087	3.4167	0.1774	1.5952
4/18/2019 11:45	17.2583	17.075	19.2183	18.0333	4.4301	3.5091	0.1608	1.5904

4/18/2019 11:46	17.2833	17.8917	24.09	19	4.3677	3.7455	0.1715	1.5618
4/18/2019 11:47	17.4167	18.0667	19.5833	18.2833	4.3355	4.2091	0.1691	1.5733
4/18/2019 11:48	17.6417	18.5167	19.875	19.2167	4.3463	4.475	0.166	1.5414
4/18/2019 11:49	17.7	17.3333	19.2583	17.95	4.4121	4.7833	0.174	1.5631
4/18/2019 11:50	17.6333	17.7083	19.06	18.3167	4.8538	4.8636	0.1625	1.5574
4/18/2019 11:51	17.4167	17.575	18.8083	17.9	5.0621	5.0583	0.156	1.5793
4/18/2019 11:52	17.175	17.3833	18.8167	18.3333	4.9142	5.3	0.175	1.5705
4/18/2019 11:53	17.2	18.5917	19.2517	18.3	4.904	5.2667	0.1594	1.5966
4/18/2019 11:54	17.35	18.3667	19.3367	18.75	4.8947	5.2	0.1778	1.5645
4/18/2019 11:55	17.625	17.725	19.2717	18.1333	4.6508	5.1833	0.1625	1.5996
4/18/2019 11:56	17.6833	18.3667	19.405	18.5167	4.5125	5.1833	0.1543	1.5907
4/18/2019 11:57	17.9917	18.475	20.1033	19.4167	4.4411	5.3083	0.1647	1.6052
4/18/2019 11:58	18.2833	17.7917	20.3033	20.0167	4.4133	5.325	0.1546	1.5893
4/18/2019 11:59	18.5083	18.1417	20.23	19.3833	4.3027	5	0.1577	1.5842
4/18/2019 12:00	18.625	19.0833	21.32	19.85	4.1831	4.73	0.1675	1.5743
4/18/2019 12:01	18.9091	19.825	20.745	19.9333	4.1369	4.6636	0.158	1.6009
4/18/2019 12:02	18.9417	18.1333	19.935	17.6	4.1213	4.6818	0.1636	1.6018
4/18/2019 12:03	19.1917	19.8167	21.2367	20.55	3.9373	4.7833	0.1485	1.5781
4/18/2019 12:04	19.2	19.5417	20.5883	20.0667	3.9303	4.9083	0.1468	1.5666
4/18/2019 12:05	19.5	19.2917	20.5867	19.8833	3.9037	5.2	0.1634	1.5711
4/18/2019 12:06	19.525	20.0667	21.29	20.7	3.8005	5.4	0.1506	1.5652
4/18/2019 12:07	19.7333	20.4	21.6117	20.55	3.7918	5.3	0.1647	1.5699
4/18/2019 12:08	19.775	20.2917	21.3117	20.7833	3.7332	5.3	0.1601	1.5483
4/18/2019 12:09	19.525	19.1583	20.3833	19.8667	3.782	5.2417	0.154	1.5336
4/18/2019 12:10	19.1417	18.4833	20.3083	19.5667	3.7817	5.1455	0.1639	1.5528
4/18/2019 12:11	18.75	18.8667	19.7283	18.9833	3.7689	5.1375	0.1539	1.5881
4/18/2019 12:12	18.325	17.7667	19.3617	18.2167	3.7736	4.98	0.1569	1.6154
4/18/2019 12:13	17.8083	17.6	19.2833	17.5167	3.7725	4.8091	0.1673	1.6038
4/18/2019 12:14	17.3583	17.5417	19.015	17.4	3.7768	4.62	0.1548	1.6084
4/18/2019 12:15	17.0167	16.775	18.925	17.1833	3.7595	4.425	0.164	1.6295
4/18/2019 12:16	16.6583	15.725	18.2783	15.9167	3.7637	4.1	0.1498	1.6206
4/18/2019 12:17	16.125	15.6083	17.7167	16.0167	3.7489	3.725	0.1599	1.6111
4/18/2019 12:18	15.5833	15.5083	17.3867	14.8667	3.7657	3.75	0.1661	1.6413
4/18/2019 12:19	15.0167	14.925	16.7817	14.75	3.7458	4.0167	0.1523	1.6281
4/18/2019 12:20	14.6833	14.5917	16.5633	14.8167	3.7365	4.19	0.1626	1.6669
4/18/2019 12:21	14.4333	14.3917	16.51	14.25	3.7423	4.1818	0.1626	1.6739
4/18/2019 12:22	14.2167	14	16.2017	14.0333	3.7332	4.14	0.157	1.6718
4/18/2019 12:23	13.925	13.3667	16.0033	14.25	3.7324	4.0083	0.1728	1.6887
4/18/2019 12:24	13.6667	13.5667	15.8633	13.2167	3.7271	4	0.1579	1.6888
4/18/2019 12:25	13.2917	12.475	14.925	13.1	3.713	4.0545	0.1741	1.8627
4/18/2019 12:26	12.8	11.7333	14.795	13	3.6755	4.1364	0.1582	2.2353
4/18/2019 12:27	12.475	12.0667	14.9483	12.85	3.6925	4.15	0.1657	1.9711
4/18/2019 12:28	12.3	12.0417	14.5433	12.3333	3.7004	4.3917	0.1742	1.7239
4/18/2019 12:29	12.2583	12.1	14.3383	11.9833	3.6998	4.3333	0.1594	1.6856
4/18/2019 12:30	12.075	11.7917	14.3517	12.1	3.6854	4.2833	0.1617	1.7519
4/18/2019 12:31	11.8917	11.65	14.4817	11.9	3.8043	4.0455	0.1621	1.7599
4/18/2019 12:32	10.8417	7	11.7017	6.6	19.8688	13.3667	0.2296	1.7043

4/18/2019 12:33	8.225	3.3583	15.3233	3.9167	36.9454	50.1364	0.5979	1.7013
4/18/2019 12:34	4.75	1.0417	28.19	2.2667	49.7672	69.725	1.0496	1.8683
4/18/2019 12:35	1.925	0.1583	55.1783	2.7333	54.5188	99.4917	1.5912	2.2742
4/18/2019 12:36	0.6667	-0.15	80.1133	4.5333	62.4233	127.0364	5.1365	2.5489
4/18/2019 12:37	0.1583	0.2333	72.5233	1.5333	52.1678	148.4909	4.229	2.4722
4/18/2019 12:38	0.0167	0.3167	78.175	1.5667	51.4883	162.1182	3.7842	2.5968
4/18/2019 12:39	0	-0.7083	75.2117	1.6167	50.0793	152.2	3.442	2.6132
4/18/2019 12:40	0	-0.75	72.2783	1.2	49.3006	158.14	3.9048	2.575
4/18/2019 12:41	0.0583	-0.2	69.5033	1.1667	48.5114	139.2091	3.5507	2.5711
4/18/2019 12:42	0.1	-0.4	68.9633	1.0333	47.9929	158.23	3.6498	2.5227
4/18/2019 12:43	0.1	0.0667	68.14	0.8167	47.4738	148.75	3.3525	2.5398
4/18/2019 12:44	0.1	-0.1333	66.955	1.2333	47.0542	138.5909	3.2148	2.5255
4/18/2019 12:45	0.1	-0.6333	66.135	0.6	46.5694	111.4333	3.197	2.5059
4/18/2019 12:46	0.2	0.05	65.2767	0.3833	46.2134	86.1417	3.2705	2.5106
4/18/2019 12:47	0.2	-0.0167	64.8683	0.6333	45.719	78.1273	3.2528	2.5282
4/18/2019 12:48	0.2	-0.3917	63.695	0.7	45.1941	77.2818	3.1932	2.5123
4/18/2019 12:49	0.2	-0.1167	62.4517	1.2	44.6074	92.3636	3.2245	2.4991
4/18/2019 12:50	0.2	-0.8417	60.895	0.7333	43.9241	78.3	3.1362	2.4949
4/18/2019 12:51	0.2	-0.025	59.6817	0.3667	43.2904	79.075	2.9518	2.5083
4/18/2019 12:52	0.2	-0.5083	58.7917	0.8333	42.8149	76.01	2.8832	2.4978
4/18/2019 12:53	0.2833	-0.575	57.6717	0.9	42.2309	75.875	2.8492	2.4824
4/18/2019 12:54	0.2917	-0.3917	56.89	0.95	42.026	75.9	2.8565	2.4752
4/18/2019 12:55	0.225	-0.6833	55.9683	0.7833	42.0148	76.4167	2.8008	2.475
4/18/2019 12:56	0.225	-0.2333	55.2233	0.5333	41.2436	76.45	2.809	2.4729
4/18/2019 12:57	0.3	-0.9083	54.7033	0.7667	40.7846	77.1818	2.78	2.4591
4/18/2019 12:58	0.3	-0.6	53.7267	0.6167	40.0366	76.9727	2.7355	2.4349
4/18/2019 12:59	0.3	-0.3083	52.8083	0.4	39.6418	77.1273	2.6935	2.4315
4/18/2019 13:00	0.2667	-0.575	51.8733	0.4833	39.0371	77.825	2.678	2.4446
4/18/2019 13:01	0.2	-0.4583	51.5433	0.2333	38.7825	78.3455	2.6705	2.454
4/18/2019 13:02	0.2	-0.2833	50.0025	0.75	38.0643	79.0818	2.6235	2.4279
4/18/2019 13:03	0.2	-0.6833	49.5567	0.4833	37.7785	79.9167	2.629	2.3998
4/18/2019 13:04	0.2	0.2	48.4358	0.6833	37.0846	80.5455	2.5863	2.4035
4/18/2019 13:05	0.1667	-0.0667	47.4342	-0.05	36.5893	82	2.5695	2.3989
4/18/2019 13:06	0.1	0.475	47.2933	0.4333	36.5096	82.9636	2.5702	2.3916
4/18/2019 13:07	0.075	-0.4583	46.795	-0.0667	36.0823	81.375	2.5447	2.4043
4/18/2019 13:08	0	-0.5417	45.8075	0.4167	35.6637	81.0636	2.5468	2.4071
4/18/2019 13:09	-0.0333	-0.6167	46.1683	-0.3	35.3587	80.6583	2.5277	2.386
4/18/2019 13:10	-0.0583	-0.175	44.935	0.0167	35.0006	80.6455	2.5123	2.3893
4/18/2019 13:11	0	0.2667	44.1225	-0.1833	34.3875	80.0727	2.4883	2.3741
4/18/2019 13:12	0.0167	-0.6667	44.0592	-0.2333	34.1449	79.7455	2.463	2.3794
4/18/2019 13:13	0.0833	-0.65	42.71	0.1	33.9456	78.7455	2.4723	2.386
4/18/2019 13:14	0.0917	0.675	42.7917	0.15	33.387	78.6636	2.439	2.4103
4/18/2019 13:15	0.0583	0.2333	42.2792	0.1	33.0954	78.5909	2.4262	2.4124
4/18/2019 13:16	0.05	0.5083	42.9433	0.5167	32.9147	78.85	2.436	2.3833
4/18/2019 13:17	0	0.025	41.3475	0.1667	32.8038	78.06	2.4065	2.392
4/18/2019 13:18	-0.0917	0.3083	40.4725	0.1333	32.2502	77.8273	2.4092	2.3929
4/18/2019 13:19	0	-0.5917	39.3725	-0.1667	31.9114	78.1727	2.3772	2.3774

4/18/2019 13:20	0	-0.0667	39.0425	-0.4667	31.3197	78.49	2.3353	2.3576
4/18/2019 13:21	0.1	-0.2333	38.7183	0.2167	31.1486	78.2667	2.3492	2.3557
4/18/2019 13:22	0.0917	-0.2667	38.4125	-0.5333	30.7896	77.2818	2.3255	2.3607
4/18/2019 13:23	0.1	-0.4083	38.1325	-0.0833	30.6649	77.4727	2.3217	2.3718
4/18/2019 13:24	0.1	0.025	37.5217	0.4	30.3934	78.6909	2.3143	2.3657
4/18/2019 13:25	0.1	-0.5167	36.7517	-0.3333	29.7805	80.0833	2.269	2.368
4/18/2019 13:26	0.1583	-0.5833	36.3658	-0.4167	29.3643	81.0182	2.2643	2.3384
4/18/2019 13:27	0.1667	-0.05	36.0767	0.2	29.2304	79.8833	2.2497	2.3285
4/18/2019 13:28	0.2	-0.1083	35.7358	0.3667	29.0485	77.88	2.2547	2.3136
4/18/2019 13:29	0.1	-0.1	35.2317	-0.2167	28.6698	77.1083	2.2328	2.2891
4/18/2019 13:30	0.1917	-0.6667	34.59	-0.2833	28.1762	77.275	2.1913	2.3149
4/18/2019 13:31	0.2	-0.35	34.6467	0	28.0358	78.92	2.2055	2.3323
4/18/2019 13:32	0.2	-0.05	34.5325	0.05	28.0365	80.8636	2.1905	2.3268
4/18/2019 13:33	0.1583	-0.5667	33.665	-0.1833	27.5486	81.2091	2.1693	2.2822
4/18/2019 13:34	0.175	-0.4083	33.3167	-0.3	27.1531	81.41	2.1583	2.2953
4/18/2019 13:35	0.175	-0.625	33.2058	-0.6	27.1123	81.6364	2.1527	2.2958
4/18/2019 13:36	0.2	-0.2917	32.5492	-0.3333	26.7309	79.1	2.1477	2.2794
4/18/2019 13:37	0.1333	-0.6	31.5175	-0.35	26.3111	76.1182	2.1025	2.2828
4/18/2019 13:38	0.1167	-0.375	31.5283	-0.1	25.9715	73.5417	2.0887	2.2874
4/18/2019 13:39	0.1	-0.1917	31.1792	-0.4333	25.8709	72.6455	2.0901	2.3166
4/18/2019 13:40	0.1	0.3417	31.1083	-0.05	25.7551	72.1333	2.0751	2.3285
4/18/2019 13:41	0.1	0.2	30.9425	-0.0167	25.5547	71.0636	2.0798	2.3153
4/18/2019 13:42	0.1	-0.4167	31.1825	0.1333	25.2408	68.8667	2.0528	2.3007
4/18/2019 13:43	0.0917	-0.1167	29.805	0.2333	24.8789	66.31	2.0301	2.3173
4/18/2019 13:44	0.075	-0.2917	29.4792	0.35	24.6193	63.45	2.0174	2.3158
4/18/2019 13:45	0	-0.6167	29.1542	-0.55	24.6141	60.3667	2.0141	2.3057
4/18/2019 13:46	0	-0.0333	29.0908	-0.3333	24.4841	58.8167	2.021	2.2818
4/18/2019 13:47	0.0417	0.1	28.9075	0.2833	24.2093	57.5091	2.0042	2.2824
4/18/2019 13:48	0.1	-0.1083	28.3067	-0.7833	23.8625	56.8	1.971	2.2773
4/18/2019 13:49	0.1	-0.225	28.56	-0.1667	23.7108	57.175	1.9817	2.2673
4/18/2019 13:50	0.1	0.1167	28.5217	-0.1	23.5373	57.1917	1.956	2.2641
4/18/2019 13:51	0.1	0.0167	27.9708	-0.0333	23.1763	55.59	1.9422	2.3005
4/18/2019 13:52	0.1	-0.3333	26.3008	-0.1667	22.4113	54.6	1.8576	2.2364
4/18/2019 13:53	0.1	0.05	24.7858	-0.45	21.9829	54.1333	1.7745	2.2638
4/18/2019 13:54	0.15	-0.4167	24.5392	-0.0667	22.8141	52.8818	1.7428	2.213
4/18/2019 13:55	0.4583	0.7583	21.5992	1	22.988	51.3583	1.5127	2.1254
4/18/2019 13:56	0.8083	1.3583	19.9225	0.7833	22.988	49.2636	1.419	2.0628
4/18/2019 13:57	1.1917	1.7583	18.4858	1.1	23.0788	48.02	1.2942	2.032
4/18/2019 13:58	1.5417	2.6333	17.6683	2.25	22.7835	45.9636	1.1621	1.9663
4/18/2019 13:59	2.0333	3.1167	15.1158	2.65	22.0823	43.36	1.3269	1.8714
4/18/2019 14:00	2.5667	3.7917	15.285	2.95	21.2826	41.2833	1.0015	1.9061
4/18/2019 14:01	3.225	5.325	15.1033	4.5	19.6861	38.0091		1.8305
4/18/2019 14:02	3.9333	5.1333	14.5325	5.3333	18.3664	34.43		1.7221
4/18/2019 14:03	4.5833	5.4667	14.1367	5.3833	17.8153	31.5727		1.5123
4/18/2019 14:04	5.0667	5.8	13.6292	6.0333	17.3782	29.0636		0.8849
4/18/2019 14:05	5.55	6.875	13.77	7.4167	16.2963	26.18		1.5907
4/18/2019 14:06	6.2333	7.5583	13.665	8.65	15.2751	23.5636		1.7158

4/18/2019 14:07	7.0417	8.7417	13.6925	8.3833	14.5637	20.9182	1.6826
4/18/2019 14:08	7.6917	9.3083	14.1517	9.3333	13.9383	18.45	1.6898
4/18/2019 14:09	8.15	9.4417	13.7133	8.8833	13.7452	17.0083	1.7098
4/18/2019 14:10	8.4083	9.2333	13.7608	9.4333	13.2083	16.0167	1.6873
4/18/2019 14:11	8.775	10.35	14.2708	10.8833	12.6791	15.2167	1.7143
4/18/2019 14:12	9.1583	10.1583	13.7817	10.4333	12.142	13.7111	1.6897
4/18/2019 14:13	9.5667	11.5083	14.2683	11.75	11.356	12.8273	1.6692
4/18/2019 14:14	10.1167	12.15	14.5933	12.0667	10.3063	12.4636	1.6529
4/18/2019 14:15	10.5083	11.3	14.0192	11.85	10.3074	12.0364	1.6606
4/18/2019 14:16	11.0083	12.7	15.2292	12.7167	9.5508	11.5	1.6546
4/18/2019 14:17	11.3167	12.925	14.7183	13.1	9.1428	11.4909	1.6378
4/18/2019 14:18	11.775	13.1583	15.5558	13.0833	8.7771	10.9455	1.631
4/18/2019 14:19	12.0833	13.2917	15.5208	13.6833	8.8143	10.7	1.6061
4/18/2019 14:20	12.2583	13.3167	15.4858	13.2333	9.3383	10.22	1.6868
4/18/2019 14:21	12.2818	13.05	13.8108	12.15	9.6697	9.525	1.6713
4/18/2019 14:22	12.3	13.2833	14.93	13.4167	9.4961	9	1.6958
4/18/2019 14:23	12.3417	13.575	15.1608	13.4333	9.2433	8.5667	1.7053
4/18/2019 14:24	12.6833	14.3583	14.8417	14.2	8.6172	8.4	1.7042
4/18/2019 14:25	13.0083	14.2667	15.67	14.95	8.1644	8.15	1.6815
4/18/2019 14:26	13.4917	15.5583	15.8475	15.15	7.6523	8.1	1.6499
4/18/2019 14:27	13.85	14.6833	16.53	15.5167	7.4274	8.4455	1.6438
4/18/2019 14:28	14.175	14.2833	16.6933	15.7667	7.2129	8.71	1.645
4/18/2019 14:29	14.475	15.3333	17.2975	15.7	6.8958	8.825	1.6164
4/18/2019 14:30	14.775	16.1417	17.8317	16.7	6.6783	9.1091	1.6367
4/18/2019 14:31	15.0417	16.85	17.2525	16.2833	6.5434	9.1818	1.6475
4/18/2019 14:32	15.2	15.9083	17.2833	16.1333	6.4681	9.14	1.6134
4/18/2019 14:33	15.2	15.9667	17.555	16.25	6.4268	9.2727	1.5942
4/18/2019 14:34	15.2833	16.5417	17.425	16.1333	6.3017	9.3273	1.5877
4/18/2019 14:35	15.3833	16.3667	17.1433	15.9833	6.0868	9.375	1.5917
4/18/2019 14:36	15.525	16.4833	17.67	16.75	5.8181	9.6	1.5948
4/18/2019 14:37	15.7	16.8083	18.1167	17.4333	5.56	9.7417	1.6036
4/18/2019 14:38	16.0667	17.2667	18.5517	17.9167	5.3395	9.71	1.5661
4/18/2019 14:39	16.4833	17.5	18.2467	17.3833	5.0444	9.3667	1.5768
4/18/2019 14:40	16.6417	17.3083	18.63	17.2	4.8815	8.9818	1.5689
4/18/2019 14:41	16.7833	18.45	19.0767	18.1167	4.7168	8.8833	1.5601
4/18/2019 14:42	16.9917	18.4417	19.2317	18.5167	4.5767	8.8	1.5698
4/18/2019 14:43	17.2167	18.35	19.425	18.2667	4.4805	8.7545	1.5972
4/18/2019 14:44	17.3417	18.175	19.6083	18.1333	4.3307	9.2917	1.5358
4/18/2019 14:45	17.4667	18.075	19.5267	18.3333	4.2238	9.4273	1.5357
4/18/2019 14:46	17.6833	18.4583	19.7717	18.2667	4.0339	9.51	1.5483
4/18/2019 14:47	17.7917	18.2083	19.5067	18.95	4.0125	9.575	1.5841
4/18/2019 14:48	17.9583	18.9333	19.99	19.5	3.977	9.53	1.6098
4/18/2019 14:49	18	18.1917	19.3817	18.4333	4.0016	9.625	1.6118
4/18/2019 14:50	17.8167	17.0917	18.9333	17.4167	4.0388	9.7818	1.5901
4/18/2019 14:51	17.3333	17.425	18.1867	16.7333	4.0338	10.1167	1.5982
4/18/2019 14:52	16.8333	16.775	17.705	17.25	3.9902	10.3091	1.5964
4/18/2019 14:53	16.4667	16.5833	18.975	17.1167	3.9257	10.3333	1.5702

4/18/2019 14:54	16.3	15.8	16.4283	16.25	3.8728	10.3556	1.5529
4/18/2019 14:55	16.0833	15.625	16.7067	17.2333	3.7731	10.3583	1.5946
4/18/2019 14:56	16.0083	16.125	17.7733	16.2167	3.727	10.1546	1.5975
4/18/2019 14:57	15.95	15.8417	17.61	16.25	3.727	10.0667	1.5835
4/18/2019 14:58	15.8833	16.55	17.81	16.3167	3.6993	9.95	1.5924
4/18/2019 14:59	15.7583	15.575	17.5133	15.7	3.7186	9.7167	1.5601
4/18/2019 15:00	15.6167	15.925	17.3083	16.4667	3.7495	9.6	1.5356
4/18/2019 15:01	15.5182	16.0833	17.28	16.5	3.7116	9.575	1.5754
4/18/2019 15:02	15.4333	14.9667	16.6883	15.9833	3.8063	9.3273	1.5699
4/18/2019 15:03	15.2667	15.7083	16.9517	15.0333	4.1302	8.925	1.5193
4/18/2019 15:04	15.15	15.8	16.665	15.25	4.2108	8.4273	1.4888
4/18/2019 15:05	15.1	15.025	16.805	16.1167	4.1762	7.9	1.5477
4/18/2019 15:06	15.1	15.3	16.6633	15.3167	4.0918	7.5091	1.5538
4/18/2019 15:07	15.1583	15.575	16.6867	15.2	3.9988	7.45	1.5061
4/18/2019 15:08	15.0917	15.2833	16.7267	15.7333	3.9478	7.35	1.5287
4/18/2019 15:09	15.1083	15.2583	16.5583	15.7	3.95	7.1833	1.5176
4/18/2019 15:10	15.2083	15.7	17.3633	16.2167	4.0245	7.05	1.5453
4/18/2019 15:11	15.2083	15.4917	16.6733	15.5	4.0246	7.05	1.5313
4/18/2019 15:12	15.275	16.1	16.7633	15.5833	3.9572	6.975	1.5362
4/18/2019 15:13	15.1833	15.55	16.7933	15.4833	3.9983	7.0545	1.5315
4/18/2019 15:14	15.2	15.3833	16.7733	15.8833	4.078	6.9667	1.5326
4/18/2019 15:15	15.1917	15.0833	16.8833	15.8333	4.068	6.95	1.5334
4/18/2019 15:16	15.1417	15.4583	16.66	15.7833	4.0273	7.1909	1.5321
4/18/2019 15:17	15.1083	15.5167	16.6383	15.75	3.9766	7.4	1.5728
4/18/2019 15:18	15.125	15.725	17.16	15.95	3.906	7.3909	1.5626
4/18/2019 15:19	15.2333	15.7417	16.5267	15.6667	3.8503	7.225	1.562
4/18/2019 15:20	15.2	15.55	17.0683	15.2333	3.8019	6.8333	1.5262
4/18/2019 15:21	15.275	15.9583	16.7333	15.6	3.7442	6.7727	1.5464
4/18/2019 15:22	15.3083	15.025	16.9883	15.6167	3.7358	6.64	1.5376
4/18/2019 15:23	15.4	14.9583	16.9467	15.4167	3.7885	6.5583	1.5313
4/18/2019 15:24	15.3667	15.5	17.0083	15.4833	3.8262	6.45	1.5321
4/18/2019 15:25	15.3417	15.575	17.2867	16.3667	3.7852	6.3	1.5245
4/18/2019 15:26	15.3917	15.3083	16.9217	16.2333	3.7452	6.3	1.5379
4/18/2019 15:27	15.3	15.4667	17.0833	15.8667	3.7353	6.5	1.4599
4/18/2019 15:28	15.2917	15.4667	16.8417	16.45	3.7534	6.34	1.3035
4/18/2019 15:29	15.2917	15.575	17.0283	16.1167	3.7817	6.2417	0.5119
4/18/2019 15:30	15.3333	14.9833	17.0517	15.6167	3.8289	6.41	-0.131
4/18/2019 15:31	15.3727	15.3667	16.855	15.9667	3.8107	6.3	-0.1571
4/18/2019 15:32	15.3083	15.475	16.8433	15.6333	3.79	6.3818	0.503
4/18/2019 15:33	15.2	14.4583	16.7767	15.45	3.788	6.3417	1.4173
4/18/2019 15:34	15.1	14.8833	16.9683	15.6833	3.8008	6.675	0.431
4/18/2019 15:35	15	15.2417	16.915	15.45	3.9359	6.8833	-0.0098
4/18/2019 15:36	15.0833	14.7917	16.5817	14.9333	4.1298	7.02	-0.0198
4/18/2019 15:37	14.9833	14.7167	16.6983	14.75	4.1444	7.425	-0.0213
4/18/2019 15:38	15	15.375	17.2117	14.95	4.0874	7.4917	0.1014
4/18/2019 15:39	15.025	14.6583	16.9567	15.7333	4.003	7.2583	1.213
4/18/2019 15:40	15.1333	14.95	16.6517	16.1167	3.9691	7.0545	1.4841

4/18/2019 15:41	15.1833	14.4	16.91	16.15	3.9947	7.0333		1.4891
4/18/2019 15:42	15.025	14.4	16.3417	15.5	4.0405	7		1.4936
4/18/2019 15:43	14.7917	14.1333	15.9317	15.2167	4.0548	7.1091		1.5218
4/18/2019 15:44	14.5417	-0.74	16.085	14.5	4.0842	7.4		1.5266
4/18/2019 15:45	14.5833	14.4167	16.0883	15	4.1395	7.72		1.5357
4/18/2019 15:46	14.7083	14.9	16.2783	14.9667	4.0968	7.85		1.5167
4/18/2019 15:47	14.875	14.4667	16.3817	15.05	4.1569	7.6727		1.5056
4/18/2019 15:48	14.8333	14.1833	15.9133	14.9667	4.2269	7.675		1.5043
4/18/2019 15:49	14.8083	13.9167	16.0367	14.3833	4.2454	7.5909		1.5211
4/18/2019 15:50	14.725	14.8667	16.2267	15.3667	4.2347	7.5083		1.5158
4/18/2019 15:51	14.7417	14.2333	15.995	15.1833	4.2852	7.6273		1.4738
4/18/2019 15:52	14.6833	14.4	15.7467	14.7333	4.3582	8		1.4806
4/18/2019 15:53	14.4917	13.8667	15.5133	14.25	4.3523	8.2909		1.4928
4/18/2019 15:54	14.025	12.4167	14.8267	13.9833	4.295	8.4182		1.5213
4/18/2019 15:55	13.4	12.3667	14.3033	12.25	4.2678	8.6833		1.524
4/18/2019 15:56	12.7917	12.35	13.53	11.6833	4.2798	8.9167		1.5152
4/18/2019 15:57	12.35	11.1167	14.045	12.2167	4.2995	8.7091		1.472
4/18/2019 15:58	12.0333	11.5	13.5267	11.4333	4.3	8.3833		1.5288
4/18/2019 15:59	11.7167	11.3167	13.4767	11.7167	4.2837	8.17		1.6418
4/18/2019 16:00	11.4083	11.55	13.1917	10.8167	4.2451	8.1636		3.0845
4/18/2019 16:01	11.0917	10.3833	12.4817	11.0833	4.2375	7.99		22.6425
4/18/2019 16:02	10.875	11.0167	12.78	11.3667	4.2174	7.9583		28.7683
4/18/2019 16:03	10.65	10.15	12.4167	10.3833	4.2367	7.9545		29.1892
4/18/2019 16:04	10.3833	9.8333	12.1108	9.3333	4.2308	7.9273		29.385
4/18/2019 16:05	10.1417	10.3	12.2892	9.8	4.2071	7.8818		26.0758
4/18/2019 16:06	10.0083	10.3	12.51	10.4333	4.2042	7.8917		5.817
4/18/2019 16:07	10	10	11.9925	10.1333	4.1839	7.7167		1.5125
4/18/2019 16:08	9.85	8.9667	11.8092	9.8667	4.1803	7.4833		1.4997
4/18/2019 16:09	9.65	8.9833	11.6625	9.9833	4.1727	7.49		1.5029
4/18/2019 16:10	9.4833	9.3833	11.5008	9.35	4.1532	7.7909	0.1641	1.4964
4/18/2019 16:11	9.2	8.5667	11.105	9.3	4.1284	8.2545	0.1731	1.5013
4/18/2019 16:12	9	8.3	11.1175	8.5333	4.1194	8.6818	0.1874	1.4988
4/18/2019 16:13	8.8	8.5	11.1367	9.05	4.1036	9.1273	0.1711	1.4999
4/18/2019 16:14	8.825	8.9833	10.9475	8.8833	4.0944	9.2417	0.1824	1.5108
4/18/2019 16:15	8.6583	8.7	11.0758	8.4167	4.0893	9.58	0.1693	1.5173
4/18/2019 16:16	8.6333	8.35	11.3675	8.9667	4.0918	9.6818	0.1789	1.519
4/18/2019 16:17	8.625	8.25	11.4917	8.9833	4.0947	10.0273	0.1861	1.4959
4/18/2019 16:18	8.6333	8.4833	10.8308	8.4	4.0713	10.3182	0.1699	1.4857
4/18/2019 16:19	8.3667	7.4833	10.1225	7.55	4.0456	10.9	0.1852	1.502
4/18/2019 16:20	7.9667	6.8	10.1708	7.6333	4.056	11.4778	0.1707	1.5183
4/18/2019 16:21	7.6083	6.9333	10.1258	7.6833	4.0121	11.9091	0.1795	1.5093
4/18/2019 16:22	7.3083	5.95	9.6153	6.9833	3.9967	12.3222	0.1794	1.5053
4/18/2019 16:23	6.975	5.65	9.2307	6.5333	3.9753	12.8182	0.1681	1.4905
4/18/2019 16:24	6.6583	6.4333	9.2332	6.6167	3.9366	13.07	0.1756	1.4942
4/18/2019 16:25	6.7	6.9	9.8994	7.3833	3.9565	13.05	0.18	1.5097
4/18/2019 16:26	6.8167	6.6667	9.8462	6.6167	3.9297	13.29	0.1783	1.5197
4/18/2019 16:27	6.975	7.0167	9.7588	6.0667	3.9408	13.6	0.18	1.5077

4/18/2019 16:28	6.7	5.9667	9.0702	6.0833	3.9125	13.82	0.1709	1.5161
4/18/2019 16:29	6.6333	6.5333	9.5072	6.7667	3.9251	14.1636	0.1788	1.5402
4/18/2019 16:30	6.4417	5.85	9.3414	6.1167	3.8982	14.4636	0.1728	1.5385
4/18/2019 16:31	6.4917	6.4667	9.0796	6.3667	3.8736	14.7167	0.1766	1.5281
4/18/2019 16:32	6.35	5.8333	9.8224	6.5833	3.8811	14.9417	0.1873	1.5012
4/18/2019 16:33	6.425	6.5667	10.1623	6.2	3.8858	15.1909	0.1666	1.5009
4/18/2019 16:34	6.25	5.2667	9.0621	5.0333	3.8418	15.4333	0.175	1.5059
4/18/2019 16:35	6.0917	5.5167	8.6623	5.35	3.8339	15.5091	0.1747	1.5043
4/18/2019 16:36	5.95	4.9833	8.8788	5.8667	3.8567	15.475	0.171	1.5305
4/18/2019 16:37	6.0583	6.1333	9.082	6.5167	3.8576	15.575	0.1822	1.5077
4/18/2019 16:38	6.1417	5.5167	9.0643	6.4333	3.8254	15.6333	0.167	1.5275
4/18/2019 16:39	6.1083	5.65	8.8752	6.3	3.8097	15.6	0.1748	1.5489
4/18/2019 16:40	6.075	5.45	8.8178	6.2833	3.7932	16.0455	0.1752	1.5361
4/18/2019 16:41	5.9909	5.2	8.7439	5.9	3.7866	16.1333	0.1675	1.5231
4/18/2019 16:42	5.9583	6.25	8.8208	5.9667	3.7899	16.3455	0.1841	1.5092
4/18/2019 16:43	5.8167	5.3333	8.4056	5.5167	3.7362	16.1818	0.1611	1.5298
4/18/2019 16:44	5.5833	5.2667	8.3093	4.8833	3.6337	15.8667	0.1806	1.5406
4/18/2019 16:45	5.2333	4.6667	7.7143	5.0667	3.6751	15.7833	0.1715	1.5224
4/18/2019 16:46	5	4.7667	8.1179	5.4	3.6707	16.075	0.1726	1.5345
4/18/2019 16:47	5.0833	5.2833	8.5518	5.2167	3.7309	16.3	0.1803	1.5103
4/18/2019 16:48	5.2417	4.8167	8.2858	5.55	3.7328	16.4909	0.1716	1.525
4/18/2019 16:49	5.3083	5.05	7.9567	4.9333	3.7091	17.0364	0.1782	1.5328
4/18/2019 16:50	5.0917	4.2667	7.8734	4.35	3.6082	17.4333	0.1755	1.547
4/18/2019 16:51	4.9667	4.7333	8.0754	5.05	3.6643	17.8727	0.1673	1.5418
4/18/2019 16:52	5.025	4.6	8.0088	5.0333	3.6601	18	0.185	1.5405
4/18/2019 16:53	5.1	5.4667	8.5712	5.0167	3.641	18.09	0.1663	1.5337
4/18/2019 16:54	5.025	4.55	7.8886	4.4833	3.6048	18.1364	0.1816	1.5353
4/18/2019 16:55	4.825	4.2	8.2483	4.45	3.465	18.3273	0.1711	1.5489
4/18/2019 16:56	4.6917	4.5333	7.7501	4.55	3.5516	18.8182	0.1661	1.5803
4/18/2019 16:57	4.6	3.6333	8.8186	4.85	3.5663	19.17	0.1792	1.5533
4/18/2019 16:58	4.5833	4.7833	7.6643	4.3667	3.5546	19.2	0.1615	1.5395
4/18/2019 16:59	4.5417	4.2	7.6627	4.3333	3.558	19.2	0.1784	1.5411
4/18/2019 17:00	4.6	4.3667	7.6993	4.3667	3.5671	19.2	0.172	1.545
4/18/2019 17:01	4.6	4.65	7.7208	4.3167	3.5872	19.2	0.1645	1.541
4/18/2019 17:02	4.4667	3.4333	7.2077	4.5333	3.4844	19.2	0.1773	1.5555
4/18/2019 17:03	4.3417	4.3333	7.3973	4.1167	3.488	19.2546	0.1637	1.5808
4/18/2019 17:04	4.2833	4.0167	7.2938	4	3.5347	19.3	0.1707	1.5675
4/18/2019 17:05	4.3	3.5333	7.5644	3.9333	3.5122	19.3833	0.1769	1.5495
4/18/2019 17:06	4.3	3.9667	7.2947	3.8	3.5274	19.4909	0.1663	1.5498
4/18/2019 17:07	4.3	4.3667	7.2858	4.2667	3.5098	19.5727	0.1797	1.5585
4/18/2019 17:08	4.3	3.7333	7.2118	3.8667	3.4985	19.6	0.1633	1.561
4/18/2019 17:09	4.25	3.9333	7.2844	3.6667	3.5108	19.6	0.1716	1.5508
4/18/2019 17:10	4.2	3.5667	7.1982	4.45	3.4713	19.6818	0.1688	1.5433
4/18/2019 17:11	4.1917	3.7	7.1692	3.65	3.4739	19.7417	0.1647	1.5499
4/18/2019 17:12	4.1	3.6667	6.9638	3.7	3.5086	19.8	0.1798	1.5515
4/18/2019 17:13	4.0417	3.7167	7.2991	3.6833	3.4658	19.8583	0.1584	1.5549
4/18/2019 17:14	4.0083	3.8667	7.1806	3.3333	3.4835	19.9	0.1744	1.5712

4/18/2019 17:15	4	3.55	7.3013	3.65	3.4731	19.9	0.1687	1.5673
4/18/2019 17:16	4	3.6167	7.1043	3.4833	3.482	19.8917	0.1618	1.5708
4/18/2019 17:17	3.8917	3.4167	7.2291	2.85	3.4438	19.8167	0.1805	1.5856
4/18/2019 17:18	3.8	3.4	6.9514	3.5	3.4603	19.9	0.1614	1.5856
4/18/2019 17:19	3.8	3.7667	7.2814	2.9167	3.4765	19.8727	0.1716	1.5777
4/18/2019 17:20	3.8167	3.0167	7.1723	3.1	3.4531	19.8	0.1638	1.6078
4/18/2019 17:21	3.7833	3.6167	7.2541	3.1167	3.4323	19.8667	0.1579	1.5983
4/18/2019 17:22	3.6917	3.7	7.0401	3.1333	3.4148	19.88	0.1751	1.5814
4/18/2019 17:23	3.6	2.9167	6.7337	3.8833	3.3974	19.8	0.1611	1.5794
4/18/2019 17:24	3.5833	3.1167	6.8843	3.2	3.356	19.8	0.1766	1.5778
4/18/2019 17:25	3.45	3.3333	7.5488	3.0167	3.343	19.775	0.1749	1.5664
4/18/2019 17:26	3.4083	3	6.7634	2.8167	3.3359	19.8	0.1649	1.5665
4/18/2019 17:27	3.4	3.0833	6.7753	2.75	3.3504	19.8833	0.1756	1.5851
4/18/2019 17:28	3.4	2.4	7.0972	3.0667	3.3547	19.9	0.1523	1.5681
4/18/2019 17:29	3.3833	2.85	6.7337	2.6333	3.3369	19.9917	0.1771	1.5691
4/18/2019 17:30	3.3417	2.9	6.7308	2.8333	3.3461	20	0.1662	1.5873
4/18/2019 17:31	3.3	3.4	6.7653	2.6167	3.3562	20	0.1563	1.5718
4/18/2019 17:32	3.275	1.9	6.6628	2.8167	3.3206	20	0.1732	1.5678
4/18/2019 17:33	3.1667	2.25	6.7185	2.9833	3.2922	20.05	0.1588	1.5712
4/18/2019 17:34	3.1		6.5717	2.3	3.272	20	0.1705	1.5829
4/18/2019 17:35	3.0417		6.6482	2.15	3.2666	20.1	0.1732	1.5992
4/18/2019 17:36	3.0167	2.7	6.686	2.6333	3.2581	20.1	0.162	1.58
4/18/2019 17:37	3.0417	2.3833	6.3718	2.8833	3.2797	20.2	0.1746	1.565
4/18/2019 17:38	3.0833	2.0333	6.85	2.8	3.2941	20.2	0.1618	1.5653
4/18/2019 17:39	3.0333		6.4333	2.0167	3.2605	20.2583	0.1696	1.5629
4/18/2019 17:40	2.95	2.45	6.384	2.4833	3.2268	20.1	0.1661	1.5748
4/18/2019 17:41	2.8833	2.4167	6.5558	2.1167	3.2193	19.9727	0.1608	1.5873
4/18/2019 17:42	2.8333	2.4	6.3763	1.8667	3.1986	19.9	0.1763	1.5881
4/18/2019 17:43	2.8083	2.5167	6.5003	2.35	3.193	19.925	0.1581	1.5688
4/18/2019 17:44	2.8	2.6167	6.6985	2.3667	3.1892	20	0.1662	1.586
4/18/2019 17:45	2.8		6.3267	2.75	3.2264	20	0.1651	1.5878
4/18/2019 17:46	2.875		6.5503	3.1167	3.2495	20	0.1651	1.5883
4/18/2019 17:47	2.9		6.499	2.25	3.1985	19.9583	0.1739	1.5862
4/18/2019 17:48	2.9		6.3098	1.8333	3.182	19.875	0.161	1.5886
4/18/2019 17:49	2.8417		6.2217	2.6	3.1876	19.7364	0.1733	1.5582
4/18/2019 17:50	2.8		6.6257	2.6333	3.2026	19.7	0.1632	1.5507
4/18/2019 17:51	2.8		6.481	2.5833	3.2213	19.7333	0.1632	1.5768
4/18/2019 17:52	2.8		6.1122	2.9	3.2007	19.8182	0.164	1.5965
4/18/2019 17:53	2.8		6.3015	2.45	3.1497	19.9	0.1543	1.5845
4/18/2019 17:54	2.775		6.149	2.3167	3.1538	19.9	0.1654	1.5778
4/18/2019 17:55	2.6583		6.4652	2.7667	3.1503	19.98	0.1627	1.5758
4/18/2019 17:56	2.6		6.21	2.4333	3.1388	19.8417	0.1619	1.5746
4/18/2019 17:57	2.5167		6.2905	2.3	3.0798	19.5909	0.164	1.5982
4/18/2019 17:58	2.4667		6.3017	1.2167	3.0944	19.0917	0.1541	1.6007
4/18/2019 17:59	2.4		6.3513	2.2333	3.0718	18.5364	0.1684	1.6053
4/18/2019 18:00	2.4		6.2518	2.0833	3.075	18.4	0.1565	1.5923
4/18/2019 18:01	2.4		6.2795	2.3833	3.089	18.2727	0.1539	1.582

4/18/2019 18:02	2.4583	3.5167	5.9837	1.7167	3.1214	18.2	0.171	1.5937
4/18/2019 18:03	2.4667		6.1577	1.6167	3.1105	17.9455	0.1528	1.6164
4/18/2019 18:04	2.3333		6.0407	1.9167	3.1205	17.2273	0.1688	1.6012
4/18/2019 18:05	2.2167		5.9507	2	3.0554	16.6636	0.1604	1.5975
4/18/2019 18:06	2.2	1.7714	6.1227	1.85	3.0948	16.175	0.1562	1.5933
4/18/2019 18:07	2.2	1.3667	5.9838	1.7333	3.0828	16.1	0.1686	1.5897
4/18/2019 18:08	2.2167	2.225	5.8667	2.35	3.0807	16.125	0.1547	1.5648
4/18/2019 18:09	2.2	1.7667	5.8252	1.8833	3.0884	16.075	0.164	1.5788
4/18/2019 18:10	2.2	2.4417	5.932	1.4833	3.1145	16.0667	0.1613	1.5995
4/18/2019 18:11	2.2083	2.125	6.0613	1.7667	3.0827	15.8417	0.1542	1.6137
4/18/2019 18:12	2.2	2.2417	5.9982	1.7667	3.0608	15.5583	0.1658	1.5959
4/18/2019 18:13	2.2	2.375	6.2128	1.3167	3.0505	15.4364	0.1552	1.5976
4/18/2019 18:14	2.1	1.3917	6.1943	1.7	3.0429	15.5	0.1656	1.6146
4/18/2019 18:15	2.1	1.5667	6.0743	1.65	3.0517	15.5727	0.1677	1.6175
4/18/2019 18:16	2.1	1.9667	6.0248	1.8333	3.0685	16.0417	0.1599	1.6195
4/18/2019 18:17	2.1	1.9833	5.906	1.2	3.06	16.2818	0.167	1.6408
4/18/2019 18:18	2.1	2	6.0218	2.1833	3.0401	16.4833	0.1605	1.6423
4/18/2019 18:19	2.0417	0.975	6.1743	1.2167	3.0104	16.1273	0.1588	1.6213
4/18/2019 18:20	2	1.4667	5.7503	1.6833	3.0273	15.7	0.1633	1.6177
4/18/2019 18:21	2.0333	1.2917	5.9558	1.3667	3.0427	15.52	0.1491	1.5997
4/18/2019 18:22	2.1	1.6417	6.0675	1.4667	3.0198	15.7	0.1596	1.5812
4/18/2019 18:23	2.1	1.025	5.8538	1.65	3.0174	16.3273	0.1527	1.5953
4/18/2019 18:24	2.0667	1.8833	5.8945	0.9833	3.0043	17.025	0.1636	1.5853
4/18/2019 18:25	2	1.625	6.0118	1.1833	2.9833	17.3455	0.1566	1.5834
4/18/2019 18:26	2	1.6583	6.0647	1.7667	2.9688	17.3167	0.1465	1.5866
4/18/2019 18:27	1.9917	1.3	5.8402	1.05	2.9404	16.94	0.1641	1.6167
4/18/2019 18:28	1.9167	1.6583	5.9805	1.0167	2.9787	16.65	0.1563	1.617
4/18/2019 18:29	1.875	2.225	5.8605	1.4167	2.9938	16.4546	0.156	1.6507
4/18/2019 18:30	1.8	1.7	5.8382	1.3167	2.943	16.5	0.1586	1.6373
4/18/2019 18:31	1.8	1.8667	5.7122	1.55	2.9542	16.6545	0.1438	1.6053
4/18/2019 18:32	1.8	0.95	5.9302	1	2.9472	16.7455	0.1616	1.6158
4/18/2019 18:33	1.775	1.6917	5.7838	1.1	2.9319	16.96	0.1568	1.6189
4/18/2019 18:34	1.7583	1.1833	6.0395	1.0667	2.8938	17.0909	0.152	1.624
4/18/2019 18:35	1.7	1.1333	5.8688	1.65	2.9114	16.7182	0.1653	1.6241
4/18/2019 18:36	1.7	0.7917	5.7348	0.6	2.8979	16.4546	0.1474	1.6205
4/18/2019 18:37	1.7	1.4417	5.7462	0.9167	2.9083	16.3833	0.165	1.6091
4/18/2019 18:38	1.7	1.6917	5.6335	1.1167	2.9183	16.575	0.163	1.6303
4/18/2019 18:39	1.6833	1.0583	5.5813	1.9667	2.8845	16.675	0.1519	1.6488
4/18/2019 18:40	1.6667	1.2167	5.667	1.25	2.8863	17	0.1612	1.6325
4/18/2019 18:41	1.6833	1.3333	5.7032	0.6667	2.9029	17.47	0.1464	1.6003
4/18/2019 18:42	1.7	0.95	5.8382	0.9167	2.8768	17.7	0.1579	1.6022
4/18/2019 18:43	1.7167	1.1417	5.6378	1.0167	2.8534	17.6083	0.1612	1.6309
4/18/2019 18:44	1.7167	1.25	5.8688	1.0333	2.8453	17.3333	0.1505	1.6124
4/18/2019 18:45	1.6583	0.925	5.9182	1.0333	2.8037	16.8818	0.1618	1.6199
4/18/2019 18:46	1.6	0.4083	5.6575	1.15	2.8142	16.4333	0.1497	1.6147
4/18/2019 18:47	1.6	1.0417	5.7908	1.15	2.8444	16.3667	0.1589	1.6221
4/18/2019 18:48	1.6	1.2667	5.8123	1.2167	2.846	16.05	0.1508	1.6155

4/18/2019 18:49	1.6	0.6667	6.5492	1.5	2.8134	15.84	0.1525	1.6146
4/18/2019 18:50	1.6	1.6417	5.5998	0.8667	2.8113	15.6636	0.1699	1.6028
4/18/2019 18:51	1.5833	1.4167	5.7823	1.3167	2.8284	15.6083	0.149	1.6378
4/18/2019 18:52	1.6	1.2083	5.9033	1.6333	2.8204	15.2583	0.1574	1.6631
4/18/2019 18:53	1.6	1.1833	5.715	1.35	2.8223	15.05	0.1499	1.64
4/18/2019 18:54	1.6	1.6667	5.8223	1.25	2.7943	14.7909	0.1455	1.634
4/18/2019 18:55	1.6	1.375	5.5773	0.85	2.84	14.7833	0.1548	1.6149
4/18/2019 18:56	1.6	1.6	5.768	1.3667	2.831	15.1818	0.1487	1.6078
4/18/2019 18:57	1.5917	1.3333	5.4443	1	2.8199	15.7455	0.1592	1.6169
4/18/2019 18:58	1.5	1.4333	5.5367	0.7	2.8153	16.15	0.1524	1.6155
4/18/2019 18:59	1.5	1.125	5.5523	0.9333	2.8051	16.6556	0.1521	1.6088
4/18/2019 19:00	1.5	1.0333	5.5097	0.6833	2.7847	16.9917	0.1586	1.6146
4/18/2019 19:01	1.5	0.6583	5.637	0.7667	2.779	16.9556	0.1423	1.628
4/18/2019 19:02	1.4167	0.5917	5.519	0.7333	2.7739	16.6364	0.1569	1.611
4/18/2019 19:03	1.4	0.7167	5.4902	0.8	2.7808	16.1	0.1496	1.6054
4/18/2019 19:04	1.3333	0.7	5.601	0.65	2.7924	16.0583	0.1472	1.6275
4/18/2019 19:05	1.3917	0.6083	5.4782	0.7667	2.7783	15.8667	0.154	1.6024
4/18/2019 19:06	1.4	0.7833	5.5248	0.75	2.7461	15.725	0.1429	1.5913
4/18/2019 19:07	1.4417	1.45	5.3927	0.7167	2.7442	15.7909	0.1563	1.6267
4/18/2019 19:08	1.4167	0.9667	5.6727	1.05	2.6863	15.6	0.154	1.6608
4/18/2019 19:09	1.3417	0.9333	5.6276	0.7	2.7145	15.1182	0.1441	1.6567
4/18/2019 19:10	1.2917	1.5	5.781	1.2833	2.7515	14.8727	0.1558	1.6364
4/18/2019 19:11	1.275	0.7417	5.4835	0.7667	2.7649	14.65	0.1433	1.6382
4/18/2019 19:12	1.3	1.3667	5.4081	1.3333	2.7383	14.5909	0.1546	1.6212
4/18/2019 19:13	1.3	0.8917	5.7181	1.3667	2.6964	15.0636	0.1515	1.6121
4/18/2019 19:14	1.3	0.975	5.9614	1.5333	2.7287	15.375	0.1478	1.6347
4/18/2019 19:15	1.3167	1.0417	5.4828	1.0833	2.7431	16.0364	0.1583	1.6185
4/18/2019 19:16	1.375	0.65	5.5928	0.8	2.7296	16.3	0.1453	1.6633
4/18/2019 19:17	1.3167	1.225	5.346	0.1667	2.7093	16.34	0.1554	1.6518
4/18/2019 19:18	1.3	1.0833	5.5936	0.2833	2.7248	16.65	0.1489	1.6727
4/18/2019 19:19	1.3	0.6167	5.5018	0.7	2.7034	16.8364	0.1455	1.6289
4/18/2019 19:20	1.3	0.6417	5.4658	0.3	2.7349	16.9417	0.1564	1.6121
4/18/2019 19:21	1.3083	0.8917	5.6548	0.4833	2.6932	16.925	0.1466	1.6434
4/18/2019 19:22	1.2417	1.2	5.5955	0.7333	2.6649	17.1583	0.163	1.6454
4/18/2019 19:23	1.2	0.6667	5.3349	0.6	2.6647	16.84	0.1423	1.6406
4/18/2019 19:24	1.2417	0.6833	5.4088	1.05	2.6615	16.9546	0.1571	1.6598
4/18/2019 19:25	1.3	0.8583	5.1243	0.85	2.6618	16.33	0.1572	1.6518
4/18/2019 19:26	1.3	0.9417	5.5421	1.0167	2.7084	16.0667	0.1463	1.6538
4/18/2019 19:27	1.3	1.7667	5.4492	0.25	2.6694	15.8167	0.1668	1.6495
4/18/2019 19:28	1.3	1.3333	5.3498	1.5	2.6758	15.85	0.1466	1.6581
4/18/2019 19:29	1.3	0.3083	5.2923	0.7333	2.7032	16.0833	0.1548	1.6625
4/18/2019 19:30	1.3167	0.775	5.1433	0.75	2.7058	16.2583	0.1544	1.635
4/18/2019 19:31	1.375	0.7	5.2675	0.7167	2.6943	15.9917	0.1467	1.6431
4/18/2019 19:32	1.3727	0.65	5.2684	0.4333	2.6698	15.7818	0.1588	1.6601
4/18/2019 19:33	1.325	0.4	5.6362	0.8333	2.677	15.7083	0.1527	1.6678
4/18/2019 19:34	1.3	0.3333	5.144	0.7333	2.6793	15.8182	0.1589	1.6532
4/18/2019 19:35	1.3	0.1333	5.1393	0.2	2.68	16.0083	0.1645	1.6471

4/18/2019 19:36	1.25	0.3417	5.6975	0.9333	2.705	16.1818	0.1496	1.6292
4/18/2019 19:37	1.2	0.2667	5.3316	0.4333	2.7048	16.65	0.1651	1.6501
4/18/2019 19:38	1.2	0.9917	5.3086	0.3833	2.6828	17.2	0.1502	1.6611
4/18/2019 19:39	1.2	0.325	5.0782	0.5833	2.6968	17.5818	0.1592	1.6623
4/18/2019 19:40	1.2	0.45	5.2453	1	2.7063	17.7546	0.1647	1.6602
4/18/2019 19:41	1.2	0.1333	5.4464	0.5167	2.7221	17.81	0.1468	1.6422
4/18/2019 19:42	1.2167	0.3	5.6266	0.45	2.6863	17.9182	0.17	1.6511
4/18/2019 19:43	1.2333	0.3667	5.5349	0.8	2.6992	18.2778	0.1431	1.6568
4/18/2019 19:44	1.2	0.275	5.2808	0.65	2.7027	18.3818	0.1553	1.6997
4/18/2019 19:45	1.125	0.425	5.205	0.2333	2.7021	18.6727	0.1557	1.6753
4/18/2019 19:46	1.1	0.5333	5.1044	0.3667	2.7156	19.0167	0.1566	1.6516
4/18/2019 19:47	1.1	0.775	5.3413	-0.3333	2.701	19.2909	0.1589	1.6456
4/18/2019 19:48	1.1	1.5583	4.9393	0.2833	2.7146	19.0455	0.1438	1.6289
4/18/2019 19:49	1.1	-0.0917	5.4404	0.5333	2.7113	18.82	0.1566	1.6338
4/18/2019 19:50	1.1	0.1083	5.2589	0.4833	2.7008	18.5167	0.1591	1.6169
4/18/2019 19:51	1.1	1.1917	5.1682	0.6667	2.7143	18.4546	0.149	1.6475
4/18/2019 19:52	1.1	1.2167	5.3743	0.1667	2.7272	18.4583	0.1524	1.6629
4/18/2019 19:53	1.1	0.6167	5.2884	0.9667	2.7331	18.5	0.1464	1.6623
4/18/2019 19:54	1.1	0.8167	5.2733	0.2667	2.7018	18.5	0.1497	1.6621
4/18/2019 19:55	1.1167	0.2833	5.3769	0.8667	2.7388	18.59	0.1601	1.6608
4/18/2019 19:56	1.1833	0.75	5.172	0.6	2.7546	18.9167	0.1474	1.6753
4/18/2019 19:57	1.1833	0.225	5.2096	0.6333	2.7317	19.2455	0.1582	1.6678
4/18/2019 19:58	1.125	0.2417	5.0386	0.5667	2.7442	19.36	0.1367	1.6493
4/18/2019 19:59	1.1	0.3167	5.0506	0.6833	2.7717	19.4727	0.1528	1.6442
4/18/2019 20:00	1.1917	0.35	4.9361	0.5667	2.7498	20	0.1515	1.6399
4/18/2019 20:01	1.2	1.6167	5.0008	0.55	2.7645	20.5455	0.1475	1.6503
4/18/2019 20:02	1.2	1.2417	5.3322	-0.1333	2.782	20.9333	0.1629	1.6565
4/18/2019 20:03	1.175	1.175	5.3908	0.55	2.7859	21.0167	0.1507	1.6561
4/18/2019 20:04	1.2	0.8167	5.1398	0.5	2.8431	21.1917	0.1521	1.6405
4/18/2019 20:05	1.175	0.4583	5.1157	0.7	2.8608	21.32	0.1494	1.6268
4/18/2019 20:06	1.1917	-0.3167	4.8015	0.3667	2.8701	21.4182	0.1456	1.6368
4/18/2019 20:07	1.1167	0.4167	5.1823	0.5833	2.8238	21.5417	0.1517	1.6449
4/18/2019 20:08	1.1417	0.0917	4.9803	0.75	2.8851	21.625	0.1405	1.6413
4/18/2019 20:09	1.15	1.1833	4.951	0.25	2.8955	21.7364	0.155	1.6601
4/18/2019 20:10	1.1	0.7333	5.0838	0.4667	2.8788	21.8	0.1476	1.6881
4/18/2019 20:11	1.1	-0.0833	5.1021	0.1167	2.8827	21.8	0.1466	1.6853
4/18/2019 20:12	1.1	0.8667	4.6658	0.25	2.8943	21.8	0.1553	1.6605
4/18/2019 20:13	1.1	0.7083	4.9437	-0.1	2.9055	21.8182	0.1398	1.6662
4/18/2019 20:14	1.0333	0.3	5.1696	0.2	2.8978	21.9	0.1545	1.66
4/18/2019 20:15	1	0.1167	5.0093	0.0833	2.9222	21.9167	0.1468	1.6428
4/18/2019 20:16	1	0.425	4.9065	-0.1333	2.9075	21.9091	0.1471	1.6787
4/18/2019 20:17	1	0.375	5.2659	0.1333	2.9134	21.9546	0.1594	1.6958
4/18/2019 20:18	1	0.2167	4.9781	0.1667	2.9311	22.025	0.1416	1.6733
4/18/2019 20:19	1	0.6	4.7168	0.0167	2.9443	22	0.1519	1.6318
4/18/2019 20:20	1	0.2333	5.3801	0.4833	2.919	22	0.1535	1.6401
4/18/2019 20:21	1	0.7833	5.0886	0.0167	2.943	21.9636	0.1499	1.6363
4/18/2019 20:22	1	0	5.0108	-0.1	2.9369	21.8583	0.1601	1.6538

4/18/2019 20:23	1	0.5583	4.9928	1.0167	2.9534	21.8273	0.1389	1.6632
4/18/2019 20:24	1	-0.05	5.1204	0.2333	2.9702	21.9	0.1586	1.6853
4/18/2019 20:25	1	0.5583	5.0038	0.4667	2.9639	21.8636	0.1505	1.6841
4/18/2019 20:26	1	-0.025	4.7588	0.25	2.9443	21.8167	0.1464	1.684
4/18/2019 20:27	0.9	0.8	4.6315	0.4667	2.9679	21.9	0.1538	1.7008
4/18/2019 20:28	0.9	0.2917	4.6016	0.55	2.9682	21.8727	0.1434	1.6836
4/18/2019 20:29	0.8333	0.15	4.8231	0.55	2.9744	21.7833	0.1506	1.6783
4/18/2019 20:30	0.8833	-0.0417	4.9204	0.0333	2.9594	21.725	0.1506	1.6957
4/18/2019 20:31	0.8667	0.6667	4.7585	0.7833	2.9722	21.7	0.1409	1.6904
4/18/2019 20:32	0.8273	0.675	5.0029	0.4	2.9699	21.7	0.158	1.6838
4/18/2019 20:33	0.8083	0.65	4.7892	0.4667	2.9572	21.7818	0.1427	1.6739
4/18/2019 20:34	0.8	0.4417	4.9765	0.3167	2.9678	21.925	0.1588	1.6434
4/18/2019 20:35	0.725	-0.75	5.0363	0.7667	3.0013	21.9	0.1502	1.6268
4/18/2019 20:36	0.7	0.275	5.0443	-0.2833	2.9942	21.9	0.1477	1.6524
4/18/2019 20:37	0.7	0.1583	4.7735	0.0333	2.9787	21.9	0.1519	1.6733
4/18/2019 20:38	0.7	-0.0583	4.558	0.4	2.9633	21.9	0.1399	1.6803
4/18/2019 20:39	0.7	-0.0667	4.903	-0.2333	2.9814	21.9	0.1593	1.6722
4/18/2019 20:40	0.7	0.025	4.7705	-0.1833	2.982	21.9083	0.1487	1.6746
4/18/2019 20:41	0.6833	0.3833	4.709	0.3	2.9827	22.05	0.1473	1.6646
4/18/2019 20:42	0.6	0.5333	4.9557	-0.0167	2.9863	22.1	0.1563	1.6635
4/18/2019 20:43	0.5583	0.6083	4.7548	-0.3667	2.9965	22.1818	0.1422	1.6663
4/18/2019 20:44	0.5	0.6167	5.0158	0.3	2.9973	22.1417	0.1605	1.665
4/18/2019 20:45	0.5	0.4667	4.8245	0.1	3.0103	22.1	0.1445	1.6506
4/18/2019 20:46	0.5167	0.4667	4.5852	0.4167	3.0129	22.1	0.1538	1.6407
4/18/2019 20:47	0.6	0.625	4.5013	-0.0167	2.9827	22.1583	0.1557	1.6482
4/18/2019 20:48	0.6	0.4167	4.9107	0.0333	2.9747	22.2	0.1436	1.6702
4/18/2019 20:49	0.6	0.325	4.7982	-0.4667	2.9923	22.2917	0.1554	1.6676
4/18/2019 20:50	0.6167	1.2167	4.8555	0.25	3.0239	22.2	0.1436	1.6754
4/18/2019 20:51	0.6	0.3	5.2103	0.2667	2.9879	22.2	0.1479	1.672
4/18/2019 20:52	0.6	-0.3167	4.9472	0.0333	3.006	22.2909	0.1527	1.6736
4/18/2019 20:53	0.6	0.5333	4.5532	-0.3833	2.9992	22.275	0.1459	1.6933
4/18/2019 20:54	0.6167	0.7083	5.1862	0.2167	2.9858	22.19	0.153	1.6848
4/18/2019 20:55	0.6417	0.0333	4.8018	-0.0667	2.993	21.85	0.1414	1.6878
4/18/2019 20:56	0.6333	0.0167	4.7745	-0.05	3.0155	21.1	0.1516	1.6772
4/18/2019 20:57	0.6917	0.45	4.8503	0.0333	2.9942	20.5182	0.1491	1.6599
4/18/2019 20:58	0.6583	0.4917	4.885	0.2833	3.0193	20.3364	0.1398	1.6458
4/18/2019 20:59	0.6333	0.95	4.7522	-0.2833	2.9765	19.9417	0.1513	1.6692
4/18/2019 21:00	0.65	0.0667	4.4103	0.2833	3.0148	19.8455	0.1404	1.7076
4/18/2019 21:01	0.7	0.325	4.7445	-0.2667	3.0062	19.7909	0.1494	1.6903
4/18/2019 21:02	0.7	-0.0167	4.4695	-0.2167	2.9941	19.7	0.1507	1.6678
4/18/2019 21:03	0.7417	-0.5333	4.7352	0.1833	2.99	19.6818	0.1439	1.6713
4/18/2019 21:04	0.7	-0.3917	4.8818	0.3333	2.9901	19.6	0.1532	1.6953
4/18/2019 21:05	0.7	0.175	4.5982	0	3.0141	19.6	0.1349	1.7051
4/18/2019 21:06	0.6333	-0.075	4.7778	0.3667	3.0203	19.6	0.1541	1.6928
4/18/2019 21:07	0.6	0.25	4.6783	-0.25	3.0081	19.8	0.1472	1.6863
4/18/2019 21:08	0.6417	-0.35	4.8015	-0.1	3.0233	20.4833	0.1416	1.6894
4/18/2019 21:09	0.6917	0.2083	4.7068	0.05	3.0431	21.1333	0.1555	1.674

4/18/2019 21:10	0.7	-0.1167	4.724	-0.5667	3.0413	21.2818	0.1434	1.6838
4/18/2019 21:11	0.7	0.6583	5.0088	-0.3	3.0238	21.6818	0.1532	1.6741
4/18/2019 21:12	0.7	0.3083	4.8647	0.5833	3.0266	21.8	0.1472	1.6739
4/18/2019 21:13	0.7	-0.45	4.4973	0	3.0455	21.8917	0.1472	1.6924
4/18/2019 21:14	0.7	0.0083	4.7598	0.4	3.0468	22.0091	0.1509	1.6968
4/18/2019 21:15	0.6	-0.35	4.9083	-0.0167	3.0438	22.1	0.1433	1.6778
4/18/2019 21:16	0.6	-0.4917	4.5745	-0.1667	3.0481	22.1	0.1602	1.6585
4/18/2019 21:17	0.6417	-0.4917	4.6632	0.05	3.0633	22.1	0.147	1.6743
4/18/2019 21:18	0.7	0.0917	4.8665	0.5	3.0701	22.1	0.1478	1.6534
4/18/2019 21:19	0.7	0.0667	4.6742	-0.2	3.0839	22.1167	0.1537	1.6744
4/18/2019 21:20	0.7	1.1083	4.589	0	3.0518	22.2	0.1445	1.7017
4/18/2019 21:21	0.6833	-0.375	4.519	-0.3	3.0706	22.2636	0.1595	1.7277
4/18/2019 21:22	0.6667	0.0583	4.758	-0.6667	3.0789	22.3818	0.1441	1.7024
4/18/2019 21:23	0.6	0.175	4.7143	0.3667	3.0713	22.4364	0.1511	1.7051
4/18/2019 21:24	0.6	0.0833	4.569	-0.4667	3.0807	22.5	0.1474	1.6664
4/18/2019 21:25	0.6	0.1833	4.5978	-0.0833	3.0884	22.5	0.1539	1.6622
4/18/2019 21:26	0.525	-0.1917	4.486	-0.2	3.1054	22.4273	0.152	1.6676
4/18/2019 21:27	0.5417	0.2	4.7572	-0.15	3.1173	22.4818	0.1423	1.6801
4/18/2019 21:28	0.575	0.3417	5.0638	-0.2	3.0966	22.5667	0.1547	1.6773
4/18/2019 21:29	0.5	0.0667	4.6745	-0.2833	3.1207	22.6	0.1514	1.6819
4/18/2019 21:30	0.5	-0.1417	4.4838	-0.1333	3.1094	22.63	0.1455	1.6843
4/18/2019 21:31	0.5	-0.8083	4.6157	0.1333	3.098	22.7	0.1594	1.6957
4/18/2019 21:32	0.5	0.2417	4.7127	-0.3	3.1153	22.7	0.1413	1.6703
4/18/2019 21:33	0.5	-0.0833	4.7968	0.15	3.1294	22.625	0.1538	1.6778
4/18/2019 21:34	0.5	-0.325	4.595	0	3.1261	22.6	0.1471	1.6763
4/18/2019 21:35	0.475	-0.1583	4.435	-0.3333	3.141	22.6636	0.1436	1.7115
4/18/2019 21:36	0.4	-0.0667	4.6627	-0.0333	3.151	22.6556	0.155	1.7173
4/18/2019 21:37	0.4	0.45	4.995	-0.0833	3.1514	22.6083	0.1388	1.7399
4/18/2019 21:38	0.4	-0.3083	4.5228	0.6	3.1244	22.7	0.1556	1.7469
4/18/2019 21:39	0.4	-0.55	4.5023	0.0833	3.1452	22.68	0.1456	1.7168
4/18/2019 21:40	0.4	0.2167	4.5598	0.4333	3.1695	22.7	0.1533	1.706
4/18/2019 21:41	0.3333	0.5583	4.7572	0.1	3.1579	22.7	0.1532	1.6851
4/18/2019 21:42	0.4	-0.1917	4.7557	0.85	3.1655	22.7	0.1405	1.6979
4/18/2019 21:43	0.4	0.1833	4.7037	0	3.1569	22.72	0.1579	1.7173
4/18/2019 21:44	0.4	0.1583	4.4162	-0.1333	3.1816	22.8	0.1529	1.7141
4/18/2019 21:45	0.4	-0.1167	4.7048	-0.0833	3.2039	22.8	0.1501	1.724
4/18/2019 21:46	0.4	-0.1083	4.761	-0.7667	3.2304	22.8	0.1527	1.7318
4/18/2019 21:47	0.4	-0.0167	4.5473	-0.0833	3.2564	22.8	0.1423	1.7258
4/18/2019 21:48	0.4	0.3583	4.536	0.0167	3.2441	22.8	0.1584	1.7068
4/18/2019 21:49	0.4	0.2	4.6757	-0.15	3.2584	22.8	0.1488	1.7126
4/18/2019 21:50	0.4167	0.075	4.667	-0.65	3.2788	22.8	0.1535	1.7413
4/18/2019 21:51	0.4	0.3667	4.5813	-0.4	3.3103	22.8364	0.1466	1.7335
4/18/2019 21:52	0.4	0.35	4.6797	0.0667	3.3143	22.8	0.1495	1.7388
4/18/2019 21:53	0.4	-0.025	4.4142	-0.0833	3.3246	22.85	0.1548	1.714
4/18/2019 21:54	0.3833	-0.1083	4.6062	-0.7	3.3463	22.9	0.1389	1.7279
4/18/2019 21:55	0.35	-0.1917	4.4157	-0.2	3.3583	22.8167	0.1561	1.7131
4/18/2019 21:56	0.3583	-0.0417	4.3492	-0.2833	3.3788	22.8	0.153	1.6941

4/18/2019 21:57	0.3	-0.2667	4.5585	-0.1333	3.3942	22.8	0.1494	1.7083
4/18/2019 21:58	0.3	-0.1167	4.3843	0.2333	3.4186	22.8	0.1597	1.7003
4/18/2019 21:59	0.3	-0.7917	4.4878	-0.4333	3.4341	22.8	0.142	1.7236
4/18/2019 22:00	0.3	-0.6083	4.4848	-0.3833	3.4793	22.825	0.1609	1.7153
4/18/2019 22:01	0.3	0.25	4.3453	-0.7667	3.4508	22.9	0.1468	1.6968
4/18/2019 22:02	0.3333	0.1583	4.4481	-0.35	3.4616	22.95	0.1477	1.6833
4/18/2019 22:03	0.4	0.1583	4.4613	-0.3	3.5088	23.0091	0.1586	1.6889
4/18/2019 22:04	0.4583	-0.25	4.256	-0.55	3.51	23.1	0.1459	1.7212
4/18/2019 22:05	0.4	0.1583	4.3268	-0.1	3.51	23.1636	0.1583	1.7538
4/18/2019 22:06	0.4	-0.5583	4.2842	0.25	3.5739	23.2	0.1503	1.7439
4/18/2019 22:07	0.4	-0.2417	4.4493	-0.3	3.5811	23.2	0.1532	1.748
4/18/2019 22:08	0.4	-0.4667	4.4675	-0.0333	3.5682	23.2455	0.1488	1.7243
4/18/2019 22:09	0.4	-0.325	4.7166	-0.3333	3.618	23.27	0.1465	1.6857
4/18/2019 22:10	0.4	-0.0917	4.5206	-0.2167	3.6129	23.3583	0.1532	1.6986
4/18/2019 22:11	0.4	-0.5083	4.4408	0.2167	3.6293	23.47	0.1397	1.7075
4/18/2019 22:12	0.4917	-0.3083	4.5613	-0.3333	3.6488	23.5	0.1506	1.696
4/18/2019 22:13	0.45	-0.4	4.4884	-0.8333	3.6389	23.3909	0.1538	1.7017
4/18/2019 22:14	0.4	-0.0417	4.599	-0.2333	3.7242	23.3	0.1534	1.7271
4/18/2019 22:15	0.3167	-0.4417	4.3437	0.0833	3.6602	23.3	0.1577	1.7168
4/18/2019 22:16	0.3	-0.75	4.4133	-0.1167	3.6692	23.35	0.139	1.7338
4/18/2019 22:17	0.3167	-0.3083	4.2844	0.0167	3.6779	23.4	0.1527	1.7086
4/18/2019 22:18	0.3917	0.0833	4.2576	-0.7667	3.646	23.4	0.1429	1.7128
4/18/2019 22:19	0.4	-0.2917	4.3222	-0.1667	3.6877	23.4455	0.152	1.7388
4/18/2019 22:20	0.325	0.675	4.3467	-0.7	3.6858	23.5	0.154	1.7168
4/18/2019 22:21	0.3417	0.4583	4.6369	-0.4833	3.6802	23.5	0.1497	1.7123
4/18/2019 22:22	0.3	0.25	4.4593	-0.2833	3.677	23.5	0.1585	1.6936
4/18/2019 22:23	0.3	-0.0417	4.4808	-0.05	3.6743	23.45	0.1489	1.6991
4/18/2019 22:24	0.3	0.05	4.1605	-0.3833	3.6811	23.4	0.1518	1.6923
4/18/2019 22:25	0.3	-0.4583	4.2551	-0.45	3.6898	23.52	0.1585	1.7298
4/18/2019 22:26	0.325	0.575	4.4128	-0.4	3.7013	23.6	0.1508	1.6934
4/18/2019 22:27	0.3	0.1917	4.2246	-0.3667	3.7293	23.6	0.1593	1.717
4/18/2019 22:28	0.3	-0.8	4.3347	-0.4833	3.72	23.5833	0.1452	1.737
4/18/2019 22:29	0.3	-0.3667	4.3959	-0.4167	3.7744	23.53	0.1563	1.6998
4/18/2019 22:30	0.3083	-0.8583	4.2956	-0.4833	3.7706	23.6	0.1454	1.6877
4/18/2019 22:31	0.4	-0.025	4.5439	0.0167	3.7857	23.6	0.1541	1.6927
4/18/2019 22:32	0.4	0.2583	4.1266	-0.1333	3.786	23.5091	0.1563	1.7128
4/18/2019 22:33	0.4	-0.35	4.2702	-0.4	3.8377	23.5167	0.1419	1.7354
4/18/2019 22:34	0.4	-0.75	4.0333	-0.3	3.7947	23.53	0.1577	1.7088
4/18/2019 22:35	0.4083	-0.3083	4.1642	-0.6	3.828	23.6	0.1438	1.7158
4/18/2019 22:36	0.4333	-1.0917	4.4271	-0.2333	3.7902	23.6364	0.1527	1.7113
4/18/2019 22:37	0.425	-0.6	4.288	-0.7167	3.7986	23.7	0.1557	1.7216
4/18/2019 22:38	0.45	-0.55	4.2003	-0.4	3.8009	23.7	0.152	1.73
4/18/2019 22:39	0.4	-0.075	4.2319	-0.6167	3.7778	23.7	0.1579	1.7121
4/18/2019 22:40	0.375	-0.2167	4.3923	-0.6667	3.8062	23.8	0.1419	1.705
4/18/2019 22:41	0.3	-0.2	4.3026	-0.0333	3.8161	23.8	0.1587	1.6919
4/18/2019 22:42	0.3	0.35	4.5674	-0.45	3.8027	23.9	0.1506	1.7055
4/18/2019 22:43	0.3	-0.3083	4.6876	0.45	3.7949	23.9	0.1512	1.7223

4/18/2019 22:44	0.3	-0.6333	4.5653	0.05	3.8148	23.9	0.1549	1.6927
4/18/2019 22:45	0.275	0.1833	4.3229	-0.0833	3.8226	23.9	0.1475	1.7098
4/18/2019 22:46	0.225	0.1833	4.2685	-0.15	3.8352	23.97	0.1623	1.7012
4/18/2019 22:47	0.2	0.0333	4.5083	-0.2333	3.8081	23.82	0.1463	1.7208
4/18/2019 22:48	0.2	-0.35	4.2374	-0.6833	3.796	23.8	0.1547	1.719
4/18/2019 22:49	0.2	-0.0917	4.4613	0.1167	3.7999	23.73	0.155	1.7386
4/18/2019 22:50	0.2	-0.75	4.3901	0.05	3.8105	23.5909	0.1498	1.7097
4/18/2019 22:51	0.2	-0.4417	4.3286	-0.5333	3.7957	23.4167	0.1606	1.7103
4/18/2019 22:52	0.2	0.75	4.0355	-0.2	3.7963	23.3	0.1448	1.6953
4/18/2019 22:53	0.3	0.4583	4.2132	-0.25	3.8198	23.3	0.1535	1.7139
4/18/2019 22:54	0.2167	-0.175	4.324	0.15	3.8323	23.3	0.15	1.7396
4/18/2019 22:55	0.2	-0.2083	4.2898	-0.2667	3.8067	23.3	0.1513	1.7411
4/18/2019 22:56	0.125	-0.1917	4.3288	-0.3167	3.8115	23.3364	0.1598	1.7244
4/18/2019 22:57	0.1	-0.25	4.436	0.2	3.7978	23.4	0.1434	1.7202
4/18/2019 22:58	0.1083	-0.1833	4.6008	-0.2	3.7823	23.36	0.1581	1.7153
4/18/2019 22:59	0.1833	-0.125	4.4063	0.3333	3.798	23.3273	0.1395	1.6947
4/18/2019 23:00	0.2	-0.7	4.1928	-0.25	3.809	23.4	0.1592	1.6937
4/18/2019 23:01	0.2167	0.05	4.1408	-0.1	3.8189	23.4	0.1496	1.6988
4/18/2019 23:02	0.2917	-0.15	4.4018	0.0667	3.8108	23.5	0.1493	1.6881
4/18/2019 23:03	0.3	0.0917	4.4304	-0.35	3.8099	23.6667	0.1622	1.7113
4/18/2019 23:04	0.3	-0.1833	4.1955	-0.3333	3.8093	23.7818	0.1387	1.7043
4/18/2019 23:05	0.3083	-0.2667	4.4083	-0.4	3.804	23.8273	0.1544	1.7344
4/18/2019 23:06	0.4	0.25	4.3663	-0.4	3.804	23.9	0.1488	1.7641
4/18/2019 23:07	0.4	0.225	3.9547	-0.1833	3.8108	23.9	0.1566	1.7388
4/18/2019 23:08	0.4	-0.35	4.2163	-0.4167	3.7878	23.9	0.1599	1.7093
4/18/2019 23:09	0.4	-0.7583	4.1273	-0.5	3.7888	23.9	0.1512	1.6929
4/18/2019 23:10	0.4	-0.1417	4.2361	-0.25	3.8	23.9091	0.1544	1.7077
4/18/2019 23:11	0.3667	-0.1667	3.9808	-0.2167	3.8061	24	0.14	1.7123
4/18/2019 23:12	0.3182	-0.0083	4.2958	-0.6167	3.7832	24.01	0.1576	1.7058
4/18/2019 23:13	0.3	-0.7167	4.3185	-0.0333	3.7894	24.1	0.1564	1.733
4/18/2019 23:14	0.3667	-0.725	4.2643	-0.3667	3.7826	24.1	0.1492	1.7503
4/18/2019 23:15	0.4	-0.3917	4.3093	-0.4	3.7897	24.1273	0.1568	1.7266
4/18/2019 23:16	0.375	-0.9417	4.3627	0.0167	3.778	24.2	0.1403	1.7279
4/18/2019 23:17	0.3	-1.0667	4.0273	-0.5167	3.7693	24.2	0.1584	1.6826
4/18/2019 23:18	0.3	-0.075	4.3564	-0.05	3.7733	24.1273	0.1504	1.6898
4/18/2019 23:19	0.3	-0.3	4.0257	0.1	3.7776	24.0546	0.1479	1.7208
4/18/2019 23:20	0.3	-0.5583	4.1798	-0.4667	3.7714	24.05	0.1552	1.7307
4/18/2019 23:21	0.3	-0.2167	4.0675	-0.0167	3.7492	24.1	0.1376	1.7378
4/18/2019 23:22	0.275	-0.0083	4.7977	-0.3333	3.7487	24.0727	0.1593	1.7135
4/18/2019 23:23	0.2	0.1	4.3538	-0.1333	3.7516	24.1	0.1428	1.6912
4/18/2019 23:24	0.2833	-0.325	4.4539	0.05	3.7454	24.1	0.1526	1.6968
4/18/2019 23:25	0.3	-0.1083	4.4124	-0.0333	3.736	24.1	0.1505	1.7326
4/18/2019 23:26	0.3	-0.5083	4.5058	-0.65	3.7278	24.1	0.1399	1.717
4/18/2019 23:27	0.3	0.15	4.0522	-0.35	3.7198	24.03	0.1525	1.693
4/18/2019 23:28	0.3	-0.9	3.8482	-0.4	3.7134	24	0.1414	1.6795
4/18/2019 23:29	0.3	-0.3917	4.1044	-0.4667	3.7173	23.9909	0.1554	1.6768
4/18/2019 23:30	0.35	-0.5667	4.1027	-0.6167	3.6996	23.9182	0.1509	1.7278

4/18/2019 23:31	0.3	-0.375	4.2068	0.05	3.7098	23.9111	0.1516	1.7577
4/18/2019 23:32	0.3833	-0.5167	4.1418	0.1667	3.7203	23.9	0.1571	1.7556
4/18/2019 23:33	0.3083	0.0583	3.7422	-0.5	3.6992	23.9	0.1389	1.7391
4/18/2019 23:34	0.4	-0.2583	4.1443	-0.2833	3.6784	23.9	0.1578	1.6926
4/18/2019 23:35	0.4	0.1417	4.135	-0.3833	3.6694	23.9	0.1458	1.6826
4/18/2019 23:36	0.45	-0.4417	4.5423	-0.25	3.6826	23.9	0.149	1.7171
4/18/2019 23:37	0.4917	-0.1167	4.1568	-0.7667	3.6734	23.9	0.1574	1.7242
4/18/2019 23:38	0.5	-0.4833	4.1513	-0.6167	3.6783	23.8091	0.1398	1.7126
4/18/2019 23:39	0.5	-0.45	4.1557	-0.3333	3.6409	23.625	0.1525	1.7283
4/18/2019 23:40	0.4917	-0.775	4.1477	0.0667	3.6325	23.6	0.1459	1.6792
4/18/2019 23:41	0.4083	-0.1917	3.9442	-0.2333	3.649	23.5636	0.1516	1.6723
4/18/2019 23:42	0.4	-0.0417	4.2155	-0.35	3.647	23.5	0.1512	1.6628
4/18/2019 23:43	0.3	-0.8083	4.1825	-0.0333	3.6554	23.4333	0.1501	1.6874
4/18/2019 23:44	0.3	0.0833	4.3568	-0.7333	3.639	23.2091	0.1598	1.6996
4/18/2019 23:45	0.3417	-0.6	4.5113	-0.0333	3.6254	23.07	0.1441	1.6903
4/18/2019 23:46	0.4	0.1083	4.2768	-0.0833	3.5888	22.8182	0.1522	1.7015
4/18/2019 23:47	0.4	0.2833	4.414	0.4833	3.599	22.5917	0.152	1.7056
4/18/2019 23:48	0.3417	-0.7167	4.2432	-0.25	3.6163	22.45	0.1421	1.7177
4/18/2019 23:49	0.3	-0.3	4.1398	-0.5833	3.5967	22.2	0.1575	1.7329
4/18/2019 23:50	0.3	0	4.2517	-0.4667	3.6161	21.9778	0.1444	1.744
4/18/2019 23:51	0.3667	-0.1667	4.184	0.3833	3.5961	21.8833	0.1525	1.7151
4/18/2019 23:52	0.3833	-0.5083	4.3183	-0.2833	3.5649	22	0.1453	1.7187
4/18/2019 23:53	0.35	-0.15	4.2925	-0.1667	3.5931	22	0.1458	1.6963
4/18/2019 23:54	0.3	-0.6	4.5662	-0.5167	3.5877	21.6889	0.1583	1.6582
4/18/2019 23:55	0.3083	0.1833	4.1932	-0.25	3.5493	21.2727	0.1377	1.6837
4/18/2019 23:56	0.325	-0.3833	4.0758	-0.3	3.559	21.18	0.1509	1.712
4/18/2019 23:57	0.3917	-0.2167	3.9517	-0.1333	3.5639	21.2	0.1493	1.7195
4/18/2019 23:58	0.4	0.05	4.2233	0.1	3.5591	21.14	0.1491	1.7129
4/18/2019 23:59	0.4	-0.175	4.0967	-0.4167	3.5427	21.0583	0.1541	1.752
4/19/2019 0:00	0.4	0.0917	4.082	-0.0167	3.5323	21.01	0.1397	1.7408
4/19/2019 0:01	0.5	-0.25	4.3808	0.15	3.5744	20.9	0.1535	1.7104
4/19/2019 0:02	0.5	-0.5583	4.4002	0.0333	3.5619	21	0.1475	1.7212
4/19/2019 0:03	0.5	-0.1667	4.2247	-0.1	3.53	21	0.1502	1.7388
4/19/2019 0:04	0.4417	-0.8083	4.1333	0.05	3.5443	21	0.1478	1.7278
4/19/2019 0:05	0.4	0	4.1297	-0.1833	3.5313	20.91	0.1383	1.718
4/19/2019 0:06	0.4	0.1333	4.2298	-0.7667	3.523	20.65	0.152	1.7041
4/19/2019 0:07	0.4	0.1583	4.4198	0.0333	3.523	20.8182	0.1409	1.6947
4/19/2019 0:08	0.4	-0.3	3.7985	0.45	3.5095	20.9546	0.1535	1.6882
4/19/2019 0:09	0.4	-0.025	4.0183	-0.05	3.5019	21.09	0.1441	1.7105
4/19/2019 0:10	0.4	-0.1333	4.393	-0.4333	3.4687	21.1167	0.1419	1.7243
4/19/2019 0:11	0.4	-0.125	4.3255	0.0667	3.4593	21	0.151	1.7218
4/19/2019 0:12	0.4	-0.5833	4.0763	0.1333	3.4963	20.9909	0.1338	1.7368
4/19/2019 0:13	0.375	-0.0417	4.2715	-0.3167	3.5078	21.0273	0.1507	1.715
4/19/2019 0:14	0.3	-0.4917	3.9788	0	3.4998	21.0636	0.1368	1.7203
4/19/2019 0:15	0.3	0.175	4.0943	-0.6667	3.5088	21.0333	0.152	1.7063
4/19/2019 0:16	0.3	0.4417	4.0053	-0.4	3.5107	20.8546	0.1511	1.692
4/19/2019 0:17	0.3	-0.1333	3.8187	-0.4833	3.5288	20.9	0.1434	1.7019

4/19/2019 0:18	0.3	-0.025	3.9403	-0.5	3.5089	21.0546	0.1513	1.7044
4/19/2019 0:19	0.3	-0.4083	4.3148	-0.1	3.4943	21.3556	0.1372	1.6927
4/19/2019 0:20	0.3	0.4167	4.1142	0.2833	3.5392	21.39	0.1527	1.6928
4/19/2019 0:21	0.3	0.0917	4.1862	-0.5167	3.5556	21.38	0.1396	1.7063
4/19/2019 0:22	0.3	0.1	3.9258	-0.1833	3.5181	21.0778	0.1376	1.7226
4/19/2019 0:23	0.3	0.025	4.186	0.1	3.4921	21.0636	0.151	1.7201
4/19/2019 0:24	0.3	-0.125	4.6157	-0.4167	3.5664	21.2909	0.1336	1.7052
4/19/2019 0:25	0.3083	0.4917	4.175	0.5333	3.755	21.4182	0.1501	1.6927
4/19/2019 0:26	0.4	-0.2083	4.0655	-0.95	3.5908	21.5	0.1314	1.699
4/19/2019 0:27	0.4333	-0.6667	4.131	0.15	3.7068	21.6455	0.1394	1.7259
4/19/2019 0:28	0.4417	-0.3333	4.058	-0.3333	3.6124	22	0.1454	1.73
4/19/2019 0:29	0.4	0.0083	4.1753	-0.3167	3.5827	21.7182	0.1425	1.7255
4/19/2019 0:30	0.375	-0.8167	4.2602	0.2833	3.5763	21.4	0.1611	1.7141
4/19/2019 0:31	0.375	-0.8083	4.2	0.1167	3.6734	21.43	0.1417	1.6998
4/19/2019 0:32	0.3083	-0.275	4.1783	-0.3333	3.668	21.2167	0.1442	1.689
4/19/2019 0:33	0.3667	-1.1917	4.0065	-0.15	3.7093	21.3	0.1428	1.6818
4/19/2019 0:34	0.325	-0.6333	4.1162	0.1167	3.661	21.5364	0.145	1.706
4/19/2019 0:35	0.35	-0.35	3.9813	-0.2	3.6156	22.0182	0.1558	1.7003
4/19/2019 0:36	0.3	-0.525	4.257	-0.6	3.6813	22	0.1291	1.6948
4/19/2019 0:37	0.3083	-0.3833	4.2435	-0.2167	3.6491	21.76	0.1502	1.6884
4/19/2019 0:38	0.3	-0.3	4.141	-0.1667	3.6325	21.49	0.1466	1.6851
4/19/2019 0:39	0.35	-0.6	3.9895	-0.9	3.7047	21.3273	0.1427	1.6802
4/19/2019 0:40	0.3167	-0.1167	4.3487	-0.8	3.7189	21.2	0.1573	1.6697
4/19/2019 0:41	0.4	-0.9333	4.1558	0.0167	3.7641	21.2546	0.1392	1.6851
4/19/2019 0:42	0.4	-0.05	4.115	-0.9667	3.7267	21.55	0.1478	1.7028
4/19/2019 0:43	0.3917	-0.0417	3.8367	-0.8333	3.7058	21.3	0.1367	1.7007
4/19/2019 0:44	0.3083	-0.2	3.9025	-0.1	3.6851	21.4182	0.1503	1.7179
4/19/2019 0:45	0.3	-0.4083	4.0202	-0.6167	3.7196	21.08	0.1471	1.7226
4/19/2019 0:46	0.35	-0.0667	3.8765	-1.0167	3.6976	20.9	0.1317	1.698
4/19/2019 0:47	0.4	-0.5083	3.8835	-0.95	3.7213	20.7546	0.1501	1.6981
4/19/2019 0:48	0.4	-0.25	3.8088	-0.1833	3.7472	20.8	0.1439	1.7144
4/19/2019 0:49	0.3917	-0.5	3.9782	-0.3667	3.7393	20.84	0.1574	1.7165
4/19/2019 0:50	0.4	0.3167	3.9205	-0.5	3.7983	21.1	0.1391	1.7218
4/19/2019 0:51	0.4	-0.575	3.707	-0.8	3.7991	21.1	0.1448	1.7053
4/19/2019 0:52	0.4	-0.125	3.9265	-0.9	3.7825	21.2818	0.1528	1.7256
4/19/2019 0:53	0.4	-0.35	3.7473	-0.4667	3.8922	20.96	0.1339	1.7246
4/19/2019 0:54	0.4	0.05	3.951	0.2	3.7668	20.6182	0.1442	1.7025
4/19/2019 0:55	0.3667	-0.3083	4.0525	-0.0833	3.8358	20.58	0.1397	1.7216
4/19/2019 0:56	0.4	-0.5417	3.7537	-0.3167	3.9193	20.37	0.1421	1.7163
4/19/2019 0:57	0.4	0.1	3.8673	-0.15	3.8583	20.07	0.1486	1.701
4/19/2019 0:58	0.4	-0.0667	3.7917	0.2833	3.9675	19.6833	0.1367	1.7271
4/19/2019 0:59	0.4	-0.175	3.6787	-0.1833	3.9011	19.4182	0.1477	1.7191
4/19/2019 1:00	0.4	-0.1833	3.9912	0.0833	3.8708	19.15	0.1358	1.7224
4/19/2019 1:01	0.4	-0.3667	3.9125	-0.45	3.8473	19.08	0.1515	1.7087
4/19/2019 1:02	0.4	0.5167	4.1514	-0.5833	3.9997	18.8818	0.1417	1.6883
4/19/2019 1:03	0.4	0.125	3.7018	-0.7333	4.1118	18.1636	0.1404	1.7148
4/19/2019 1:04	0.4	-0.6083	3.8476	-0.6833	4.0423	17.7444	0.1518	1.7078

4/19/2019 1:05	0.4	-0.5333	3.6743	-0.6	4.0734	17.3364	0.1375	1.7068
4/19/2019 1:06	0.4	-0.225	3.8276	-0.3167	3.9512	17.5091	0.145	1.7142
4/19/2019 1:07	0.4833	0.1167	3.9151	-0.3	4.078	17.72	0.132	1.6968
4/19/2019 1:08	0.4167	-0.1333	3.6863	0.25	4.086	17.7636	0.1444	1.7135
4/19/2019 1:09	0.4	-0.0167	3.7848	-0.1	4.0795	17.9909	0.1474	1.743
4/19/2019 1:10	0.35	-0.0917	3.8197	-0.2	4.0962	18.0909	0.1429	1.7581
4/19/2019 1:11	0.3	-0.9583	3.6991	-0.5333	4.1738	17.8273	0.1512	1.7346
4/19/2019 1:12	0.3	-0.1417	3.8234	-0.3333	4.1728	17.4818	0.1349	1.7216
4/19/2019 1:13	0.3	-0.3917	3.7	-0.4167	4.1433	16.8818	0.1403	1.7475
4/19/2019 1:14	0.2917	-0.375	3.5913	-0.5167	4.2569	16.72	0.1455	1.7432
4/19/2019 1:15	0.2333	0.475	3.8793	0	4.2243	17.5	0.1373	1.7257
4/19/2019 1:16	0.2	-0.275	3.6003	-0.0167	4.3008	17.9417	0.1539	1.7234
4/19/2019 1:17	0.1667	-0.6583	3.7629	-0.4667	4.2446	18.06	0.1374	1.7089
4/19/2019 1:18	0.175	0.4167	4.0053	-0.5167	4.2424	18.31	0.14	1.7203
4/19/2019 1:19	0.2167	-0.3167	3.9863	-0.3333	4.3557	18.39	0.1495	1.7302
4/19/2019 1:20	0.3	-0.075	4.0873	-0.4667	4.1959	18.1583	0.1437	1.72
4/19/2019 1:21	0.2167	0.0083	3.4196	-0.4167	4.1272	17.66	0.1553	1.7114
4/19/2019 1:22	0.1333	-0.3167	3.9851	-0.0167	4.1458	17.9111	0.1376	1.7066
4/19/2019 1:23	0.1	0.225	3.9217	-0.3167	4.1429	18.43	0.1456	1.7249
4/19/2019 1:24	0.1	-0.3833	3.8516	-0.1	4.2529	19.0111	0.143	1.7148
4/19/2019 1:25	0.1667	0.0667	3.8384	-0.4833	4.2866	19.68	0.1371	1.6965
4/19/2019 1:26	0.2	-0.1917	3.9918	0.25	4.2755	19.7182	0.1518	1.7285
4/19/2019 1:27	0.2	-0.65	3.8529	-0.3667	4.3233	19.3909	0.1353	1.7327
4/19/2019 1:28	0.1917	0.15	3.7494	-0.1	4.4049	19.08	0.1505	1.6911
4/19/2019 1:29	0.1917	-0.475	3.8148	-0.2667	4.3739	19.2273	0.1441	1.7293
4/19/2019 1:30	0.2083	-0.4917	3.9491	-0.8	4.3394	19.2889	0.1419	1.7243
4/19/2019 1:31	0.225	0.2083	3.8208	-0.5	4.3709	19.4546	0.1531	1.7125
4/19/2019 1:32	0.2	-0.425	3.5498	-0.2167	4.3601	19.56	0.1393	1.6928
4/19/2019 1:33	0.2083	-0.6	3.6628	-0.2833	4.4054	20.55	0.1513	1.7248
4/19/2019 1:34	0.2	0.25	3.7563	-0.6	4.3337	20.9444	0.1399	1.6912
4/19/2019 1:35	0.2083	0.1083	3.8088	-0.7167	4.2973	20.8546	0.1478	1.6811
4/19/2019 1:36	0.2	-0.3667	3.9825	-0.5	4.422	20.9	0.15	1.7039
4/19/2019 1:37	0.2	-0.3333	3.3513	-0.2167	4.3666	21.0091	0.1354	1.7035
4/19/2019 1:38	0.2333	-0.3417	3.8158	-0.0167	4.3348	21.1091	0.1468	1.7023
4/19/2019 1:39	0.3	-0.4583	3.8293	-0.1667	4.3368	21.0727	0.1383	1.6928
4/19/2019 1:40	0.3	-0.825	3.6069	-0.6167	4.2768	21.4	0.1479	1.6638
4/19/2019 1:41	0.3	0.65	3.7967	-0.55	4.3463	21.2417	0.1452	1.712
4/19/2019 1:42	0.3	0.15	3.9349	-1.0167	4.3396	21.3364	0.1453	1.7117
4/19/2019 1:43	0.3	0.4917	3.8182	-0.0667	4.2683	21.06	0.1527	1.6993
4/19/2019 1:44	0.3	-0.025	3.856	-0.4167	4.3111	20.8636	0.134	1.6998
4/19/2019 1:45	0.3	-0.4833	3.6735	-0.4333	4.2835	20.06	0.1532	1.7068
4/19/2019 1:46	0.3	-0.1083	3.5498	-0.45	4.3213	19.1667	0.1402	1.6964
4/19/2019 1:47	0.3	-0.5833	3.7946	-0.6333	4.2818	18.9818	0.1436	1.702
4/19/2019 1:48	0.3	-0.1667	3.6963	-0.2167	4.2659	18.7909	0.1478	1.6984
4/19/2019 1:49	0.2833	0.35	3.6885	-0.4167	4.27	18.79	0.132	1.6958
4/19/2019 1:50	0.2	-0.1833	3.8496	-0.5	4.26	18.65	0.1519	1.709
4/19/2019 1:51	0.175	-0.3	3.5491	-0.8833	4.2343	18.8	0.13	1.7287

4/19/2019 1:52	0.1917	-0.575	3.8364	-0.25	4.2514	18.7556	0.1444	1.725
4/19/2019 1:53	0.2	-0.7167	3.9153	-0.5	4.2628	18.8636	0.1416	1.7222
4/19/2019 1:54	0.2	-0.65	4.0102	-0.2667	4.2407	18.7364	0.1401	1.7105
4/19/2019 1:55	0.2083	0.25	4.0463	0.05	4.2356	18.5455	0.1499	1.7121
4/19/2019 1:56	0.2917	-0.4	3.4667	-0.1667	4.2496	18.57	0.1333	1.6848
4/19/2019 1:57	0.3	-0.1583	3.9313	-0.35	4.247	19.2	0.1507	1.6968
4/19/2019 1:58	0.2083	-0.1667	3.9288	-0.2	4.2291	20.0778	0.1384	1.7068
4/19/2019 1:59	0.2	-0.125	3.8783	-0.0667	4.2203	20.14	0.1415	1.683
4/19/2019 2:00	0.2	-0.125	3.7233	-0.4167	4.2133	20.3	0.1406	1.7071
4/19/2019 2:01	0.1083	-0.0417	3.7025	-0.6333	4.1998	20.4364	0.1354	1.7248
4/19/2019 2:02	0.1667	-0.8667	3.856	-0.4167	4.2073	20.4546	0.1505	1.7245
4/19/2019 2:03	0.2	-0.0083	4.1934	-1.5	4.2686	20.55	0.1334	1.7206
4/19/2019 2:04	0.225	-0.4417	3.6979	-0.5667	4.2648	20.7727	0.15	1.7159
4/19/2019 2:05	0.2	-0.5583	3.8068	-0.4667	4.2502	21	0.1413	1.7163
4/19/2019 2:06	0.2	-0.6917	3.4636	-0.3	4.2334	21.13	0.1438	1.7383
4/19/2019 2:07	0.275	-0.425	3.5872	-0.7667	4.2848	21.49	0.1485	1.7593
4/19/2019 2:08	0.3	-0.4833	3.8137	0.1833	4.2365	21.76	0.1342	1.761
4/19/2019 2:09	0.25	-0.3167	3.6804	-0.3667	4.234	21.4	0.1548	1.7611
4/19/2019 2:10	0.2083	-0.4417	4.0133	-0.15	4.245	21.5364	0.1449	1.7587
4/19/2019 2:11	0.2167	-0.1333	3.5729	-0.2	4.3093	21.8111	0.1444	1.7361
4/19/2019 2:12	0.3	-0.2	3.6707	-0.7333	4.264	21.74	0.1467	1.7203
4/19/2019 2:13	0.3	-0.3083	3.4873	-0.8	4.2962	21.43	0.1366	1.7033
4/19/2019 2:14	0.2917	-0.525	3.9913	-0.0667	4.3624	20.5818	0.1534	1.7162
4/19/2019 2:15	0.2667	-0.4667	3.5669	-0.1333	4.4195	20.42	0.1385	1.7178
4/19/2019 2:16	0.2667	-0.8583	3.6899	-0.1167	4.4136	20.34	0.1536	1.7465
4/19/2019 2:17	0.2083	-0.35	3.4078	0.25	4.4772	20.43	0.1484	1.7283
4/19/2019 2:18	0.275	-1.0083	3.8017	-0.5833	4.4985	20.26	0.1467	1.7013
4/19/2019 2:19	0.3	-0.7833	3.7431	-0.75	4.4447	20	0.1522	1.7083
4/19/2019 2:20	0.3	-0.2833	3.5495	-0.45	4.455	19.8455	0.1384	1.7328
4/19/2019 2:21	0.3	-0.3417	3.4292	0.2	4.5227	20.1818	0.1557	1.7253
4/19/2019 2:22	0.3	-0.4833	3.5672	-0.5	4.5032	20.1818	0.1394	1.7262
4/19/2019 2:23	0.3	-0.4333	3.6103	-0.8	4.5835	20.1091	0.1501	1.6988
4/19/2019 2:24	0.3	-0.75	3.4936	-0.7167	4.5545	20.2182	0.1463	1.6878
4/19/2019 2:25	0.2417	0.2583	3.6621	-0.35	4.5478	20.39	0.1448	1.7102
4/19/2019 2:26	0.2	-0.3667	3.5599	-0.5333	4.5849	20.9778	0.1495	1.7051
4/19/2019 2:27	0.15	0.1583	3.7209	-0.1833	4.663	21.7917	0.1358	1.6822
4/19/2019 2:28	0.1833	0.1417	3.5829	-0.15	4.5878	21.9778	0.1546	1.6929
4/19/2019 2:29	0.2	-0.1417	3.4838	-0.4167	4.7153	22.1	0.141	1.737
4/19/2019 2:30	0.2333	-0.675	3.3727	-0.1833	4.7863	22.3818	0.15	1.7355
4/19/2019 2:31	0.2833	-0.4083	3.6987	-0.6333	4.755	22.5	0.1504	1.7328
4/19/2019 2:32	0.3	-1.025	3.2905	0.05	4.9003	22.7091	0.1415	1.7369
4/19/2019 2:33	0.3	-0.7667	3.4678	-0.6333	4.839	22.97	0.1606	1.7563
4/19/2019 2:34	0.3	0.0583	3.6278	-0.3	4.7746	22.93	0.1436	1.7159
4/19/2019 2:35	0.225	-0.1083	3.5113	-0.4167	4.7849	22.9182	0.1606	1.6978
4/19/2019 2:36	0.2	0.2417	3.4377	-0.75	4.7973	22.9818	0.1507	1.7014
4/19/2019 2:37	0.2	-0.1417	3.5827	-0.0833	4.8438	22.91	0.1383	1.7245
4/19/2019 2:38	0.2	-0.275	3.2998	-0.2167	4.732	23	0.1545	1.7313

4/19/2019 2:39	0.2	-0.3667	3.2993	0.2833	4.8201	23.0167	0.1367	1.717
4/19/2019 2:40	0.2	0.2	3.4245	-0.0333	4.738	22.8273	0.1549	1.704
4/19/2019 2:41	0.2583	-0.2917	3.411	-0.6	4.8501	22.5667	0.1503	1.7063
4/19/2019 2:42	0.3	-0.375	3.467	-0.4167	4.9694	22.39	0.1545	1.7285
4/19/2019 2:43	0.3	-0.0667	3.3788	-0.1167	4.9709	22.4364	0.1497	1.7265
4/19/2019 2:44	0.275	-0.7917	3.3835	-0.65	4.8719	22.5	0.1433	1.7251
4/19/2019 2:45	0.2	-0.15	3.3987	-0.5	4.8409	22.5182	0.1565	1.7459
4/19/2019 2:46	0.1583	0.1417	3.5493	0.1667	4.8918	22.6222	0.146	1.7103
4/19/2019 2:47	0.2	-0.0333	3.2853	-0.45	5.0346	22.7273	0.1513	1.7198
4/19/2019 2:48	0.2	-0.1917	3.5057	-0.4833	5.0564	22.5182	0.1573	1.7395
4/19/2019 2:49	0.2	-0.0083	3.4528	-1.3833	5.0897	22.9	0.1451	1.7283
4/19/2019 2:50	0.1917	-0.5167	3.3452	-0.9167	5.2149	23	0.1611	1.716
4/19/2019 2:51	0.2	-0.6583	3.5372	-0.4833	5.0599	23.0889	0.1445	1.7326
4/19/2019 2:52	0.2	0.0667	3.4122	-0.6833	4.8923	23.4364	0.1515	1.7272
4/19/2019 2:53	0.2	0.2333	3.228	-0.5167	5.0673	23.71	0.1521	1.7299
4/19/2019 2:54	0.1833	0.0583	3.6082	-0.35	5.035	24	0.1477	1.7507
4/19/2019 2:55	0.1	-0.0833	4.0903	-0.85	5.0608	24.1333	0.1621	1.7541
4/19/2019 2:56	0.1083	-0.9083	3.4227	-0.6167	5.0873	24.2273	0.1483	1.7271
4/19/2019 2:57	0.1667	-0.8333	3.4238	-0.3333	5.12	24.3556	0.1565	1.717
4/19/2019 2:58	0.2	-0.5833	3.3922	-0.25	5.0852	24.4	0.1625	1.7332
4/19/2019 2:59	0.2	-0.7583	3.4118	0.0167	5.0548	24.42	0.1449	1.735
4/19/2019 3:00	0.2	-0.9083	3.7813	-0.2667	5.1269	24.6636	0.1607	1.7176
4/19/2019 3:01	0.2	-0.3167	3.5748	-0.85	5.2273	24.7	0.1492	1.7162
4/19/2019 3:02	0.2	-0.1083	3.4763	-0.2833	5.2203	24.6182	0.1568	1.7431
4/19/2019 3:03	0.2	-0.75	3.1478	-0.6167	5.3995	24.6667	0.1575	1.7406
4/19/2019 3:04	0.2	-0.7	3.3927	-0.4833	5.3384	24.6	0.1469	1.7238
4/19/2019 3:05	0.2	-0.8833	3.7465	-0.6333	5.1892	24.6909	0.162	1.7168
4/19/2019 3:06	0.2	-0.3	3.5013	-0.8	5.132	24.72	0.1477	1.7007
4/19/2019 3:07	0.2	-0.575	3.354	-0.5667	5.1734	24.8	0.1603	1.7078
4/19/2019 3:08	0.2	-1.3167	3.3197	-1.0333	5.2158	24.8	0.1508	1.7099
4/19/2019 3:09	0.2	-0.9583	3.3423	-0.5667	5.2656	24.7182	0.1556	1.7158
4/19/2019 3:10	0.2	-0.1417	3.4803	-0.5	5.267	24.67	0.1597	1.7193
4/19/2019 3:11	0.2	-1.1833	3.3927	-0.8667	5.2912	24.75	0.149	1.7322
4/19/2019 3:12	0.2	-0.4	3.5928	-0.4667	5.3293	24.8	0.1593	1.7315
4/19/2019 3:13	0.2	-0.1917	3.4948	-0.6333	5.1129	24.7727	0.1569	1.7381
4/19/2019 3:14	0.15	-0.325	3.6222	-0.3167	5.2343	24.87	0.1509	1.6974
4/19/2019 3:15	0.2	-0.4083	3.3665	-0.7833	5.3583	24.9455	0.1607	1.7159
4/19/2019 3:16	0.2	-0.5167	3.6205	-0.3833	5.3911	25.0111	0.1532	1.7326
4/19/2019 3:17	0.2	-0.2917	3.351	-0.1833	5.0902	25.01	0.1573	1.7174
4/19/2019 3:18	0.2	0.075	3.293	-0.45	5.2469	25.0375	0.1521	1.7225
4/19/2019 3:19	0.2	-0.7167	3.2983	-0.8833	5.3343	25.03	0.1593	1.743
4/19/2019 3:20	0.2	-0.5	3.2138	-0.6	5.3539	25.1444	0.1599	1.729
4/19/2019 3:21	0.2	-0.4167	3.116	-1.1	5.29	25.2	0.1524	1.714
4/19/2019 3:22	0.2083	0.1417	3.291	-0.5667	5.2841	25.3	0.1641	1.6998
4/19/2019 3:23	0.2	0.225	3.7173	-0.3167	5.1911	25.27	0.1489	1.7172
4/19/2019 3:24	0.2	-0.275	3.4193	-0.6667	5.2772	25.22	0.1585	1.7063
4/19/2019 3:25	0.2	-0.7333	3.4062	-0.8167	5.2782	25.34	0.1545	1.7407

4/19/2019 3:26	0.1333	-0.6667	3.5005	-0.3167	5.2657	25.4	0.1497	1.7362
4/19/2019 3:27	0.1	-0.5833	3.619	-0.55	5.2558	25.4	0.1625	1.7397
4/19/2019 3:28	0.125	-0.075	3.3008	-0.15	5.2172	25.4	0.1416	1.7568
4/19/2019 3:29	0.2	-0.9083	3.2383	-0.3	5.2091	25.4	0.1633	1.7695
4/19/2019 3:30	0.2	-0.4583	3.4318	-0.25	5.157	25.4	0.1572	1.7714
4/19/2019 3:31	0.1917	-0.5833	3.4078	-0.3	5.183	25.4	0.1578	1.7495
4/19/2019 3:32	0.1083	-1.0667	3.2567	-0.5833	5.156	25.4	0.1608	1.7422
4/19/2019 3:33	0.1	-0.4917	3.2677	-0.9667	5.0955	25.4	0.1481	1.7477
4/19/2019 3:34	0.0917	-1.1667	3.4965	-0.3	5.0969	25.4	0.1635	1.7493
4/19/2019 3:35	0.1	-0.7083	3.4322	-0.8667	5.0888	25.4455	0.1485	1.7485
4/19/2019 3:36	0.1	-0.5083	3.661	-0.9833	5.0734	25.59	0.1546	1.7552
4/19/2019 3:37	0.0667	-0.3833	3.2922	-0.5833	5.1298	25.5583	0.1578	1.7459
4/19/2019 3:38	0	-0.6083	3.3967	-0.2167	5.0761	25.5	0.1514	1.747
4/19/2019 3:39	0	-0.0083	3.5423	-0.3667	5.0588	25.5364	0.1652	1.7248
4/19/2019 3:40	0	-0.175	3.707	-0.0333	5.0341	25.6	0.1479	1.7185
4/19/2019 3:41	0	-0.0667	3.5673	0.1333	4.9909	25.6	0.1599	1.715
4/19/2019 3:42	0	-0.5917	3.3077	-0.5333	5.04	25.6182	0.1536	1.7441
4/19/2019 3:43	0.0455	-0.5583	3.547	-0.1667	5.0197	25.7	0.1522	1.7361
4/19/2019 3:44	0.1	-0.5167	3.75	-0.6667	4.9894	25.7727	0.1585	1.7493
4/19/2019 3:45	0.1	-0.65	3.2118	-0.9333	4.9958	25.78	0.1486	1.7479
4/19/2019 3:46	0.1	-0.2417	3.246	-0.2	5.0335	25.7	0.1611	1.7503
4/19/2019 3:47	0.1	-0.625	3.4193	-0.3333	4.9867	25.4818	0.1515	1.7699
4/19/2019 3:48	0.1	-0.5917	3.4813	-0.4	4.9872	25.2	0.1597	1.7491
4/19/2019 3:49	0.1	-1.1833	3.6288	-0.65	5.0193	25.1	0.1575	1.7391
4/19/2019 3:50	0.1083	-0.3167	3.2442	-0.4333	5.0155	25.0818	0.1465	1.7214
4/19/2019 3:51	0.2	-0.725	3.5005	0.15	5.0311	25	0.1617	1.7187
4/19/2019 3:52	0.2	-0.4333	3.0588	-0.1833	4.9635	24.98	0.1472	1.7369
4/19/2019 3:53	0.2	-0.9583	3.3702	-0.9167	4.9083	24.9	0.1566	1.7298
4/19/2019 3:54	0.2	0	3.4647	-0.05	4.9254	24.9	0.1561	1.745
4/19/2019 3:55	0.2	-0.4917	3.222	-0.5167	4.9917	24.8444	0.1558	1.7406
4/19/2019 3:56	0.2	-0.7917	3.3368	-0.3833	5.0866	24.8	0.1604	1.7353
4/19/2019 3:57	0.2	-0.3833	3.5563	0.2333	5.1957	24.8	0.1423	1.7432
4/19/2019 3:58	0.1583	-0.1417	3.2958	-0.45	5.2415	24.9	0.1606	1.7334
4/19/2019 3:59	0.1167	-0.2167	3.365	-0.35	5.1411	24.9667	0.158	1.7424
4/19/2019 4:00	0.1917	-0.8167	3.2547	0.1333	5.1361	25.1909	0.1505	1.7422
4/19/2019 4:01	0.2	-0.55	3.1795	-0.7333	5.1709	25.3333	0.1584	1.7263
4/19/2019 4:02	0.2	-0.825	3.5008	-0.6667	5.1651	25.4273	0.1528	1.7157
4/19/2019 4:03	0.1	-0.4667	3.4764	-0.6167	5.2261	25.5	0.1635	1.771
4/19/2019 4:04	0.1	-1.1167	3.7338	-0.4333	5.1344	25.5	0.1512	1.7518
4/19/2019 4:05	0.1	0.1583	3.3514	-0.2167	5.2033	25.5455	0.1607	1.7471
4/19/2019 4:06	0.1	0.0917	3.7249	-0.5667	5.1559	25.5	0.1625	1.7746
4/19/2019 4:07	0.1	0.0417	3.3417	-0.75	5.1122	25.5	0.153	1.7464
4/19/2019 4:08	0.1	-0.2917	3.395	-0.5833	5.0492	25.5	0.1619	1.7393
4/19/2019 4:09	0.1	-0.3	3.2062	-0.3333	5.0615	25.5	0.1549	1.7491
4/19/2019 4:10	0.1	-0.8083	3.4198	-0.4167	5.1138	25.56	0.1649	1.7292
4/19/2019 4:11	0.1	-0.425	2.8398	-0.5833	5.113	25.6833	0.1581	1.7258
4/19/2019 4:12	0.0167	-0.2167	3.2813	-0.2333	5.1228	25.7667	0.1519	1.741

4/19/2019 4:13	0	-0.4	3.6515	-0.2833	5.2015	25.8182	0.1667	1.7257
4/19/2019 4:14	0	-0.775	3.0998	-0.15	5.217	25.9	0.1492	1.7151
4/19/2019 4:15	0	-0.1833	3.2947	-0.5667	5.1939	25.9417	0.1637	1.7018
4/19/2019 4:16	0	-0.9667	3.318	-0.8167	5.2927	26.0833	0.1476	1.7058
4/19/2019 4:17	0	0.1667	3.3008	-0.5	5.2623	26.07	0.1609	1.704
4/19/2019 4:18	0	-0.825	3.1893	-0.4667	5.151	26.1545	0.158	1.7124
4/19/2019 4:19	0	-0.4083	3.4218	-0.0333	5.1687	26.2	0.1526	1.7076
4/19/2019 4:20	0	-0.275	3.6055	-0.2167	5.0021	26.2182	0.1657	1.71
4/19/2019 4:21	0	-0.475	3.3104	-0.2667	5.1476	26.3	0.1477	1.7271
4/19/2019 4:22	-0.0167	-0.0167	2.8794	-0.75	5.1957	26.3273	0.162	1.7523
4/19/2019 4:23	-0.1	-0.0583	3.452	-0.9167	4.9706	26.2167	0.1511	1.7668
4/19/2019 4:24	-0.1	0.25	3.1042	-0.6167	5.1564	26.2	0.1565	1.7425
4/19/2019 4:25	-0.1	-0.6917	3.4273	-0.5	5.1948	26.15	0.1636	1.7177
4/19/2019 4:26	-0.0417	0.025	3.149	-0.4333	5.2379	26.1	0.1545	1.7406
4/19/2019 4:27	0	-0.6667	3.6033	-0.2167	5.2531	26	0.1643	1.7525
4/19/2019 4:28	0.0083	-0.2583	3.2784	-0.9667	5.1501	26	0.155	1.7443
4/19/2019 4:29	0.0083	-0.9417	3.543	-0.7833	5.2053	26	0.1622	1.7478
4/19/2019 4:30	0	-0.2833	3.4179	-0.6833	5.2511	25.8	0.1657	1.7358
4/19/2019 4:31	0	0.125	3.1327	-0.8333	5.1759	25.7917	0.1512	1.7289
4/19/2019 4:32	0	-0.5917	3.2608	-0.8333	5.1536	25.7	0.161	1.7369
4/19/2019 4:33	0.0667	-0.3	3.4867	-1.2	5.2473	25.6333	0.1494	1.7235
4/19/2019 4:34	0.0583	-0.0583	3.3046	-0.3333	5.1957	25.65	0.1628	1.7199
4/19/2019 4:35	0.1	-0.3667	3.2187	-0.35	5.2168	25.72	0.1545	1.7203
4/19/2019 4:36	0.0167	-0.15	3.4032	-0.8333	5.2142	25.7167	0.1503	1.7273
4/19/2019 4:37	0.0333	-0.5	3.2757	-0.3333	5.2473	25.7727	0.1635	1.7241
4/19/2019 4:38	0.0333	-0.2917	3.198	-0.2667	5.1911	25.8455	0.1466	1.7027
4/19/2019 4:39	0.1	-0.4167	3.1228	-0.2	5.2887	26	0.164	1.7228
4/19/2019 4:40	0.1	-0.45	3.1202	-1.0167	5.224	26.0727	0.1538	1.7179
4/19/2019 4:41	0.1	0.2	3.1219	-0.45	5.1039	26.1091	0.1547	1.7432
4/19/2019 4:42	0.1	-0.425	3.3556	0.0667	5.1693	26.3273	0.157	1.7259
4/19/2019 4:43	0.1	0.1333	3.2802	-0.5	5.1253	26.2273	0.1518	1.7063
4/19/2019 4:44	0.0583	-0.9417	3.4492	-0.7333	5.029	26.3	0.1671	1.7269
4/19/2019 4:45	0.0917	-0.1333	3.0448	-0.55	5.2011	26.39	0.1488	1.7293
4/19/2019 4:46	0.1	0.5583	3.2213	-0.8167	5.1466	26.3833	0.1639	1.7535
4/19/2019 4:47	0.1833	-0.3583	3.2344	-0.3	5.1418	26.3273	0.1551	1.7489
4/19/2019 4:48	0.175	0.0167	3.374	-1.05	5.2534	26.4727	0.1623	1.7363
4/19/2019 4:49	0.1	-0.225	3.281	-0.3	5.0506	26.5	0.1711	1.7332
4/19/2019 4:50	0.1	-0.3917	3.654	-0.1667	5.0104	26.5	0.1418	1.7348
4/19/2019 4:51	0.1	-0.2667	3.328	-0.9	5.1188	26.475	0.1659	1.7294
4/19/2019 4:52	0.1	0.025	3.3054	-0.75	5.0806	26.4	0.1539	1.7584
4/19/2019 4:53	0.1	-0.65	3.4778	-0.2667	5.0667	26.4	0.1587	1.7399
4/19/2019 4:54	0.1	0.525	5.105	-0.6333	5.068	26.3727	0.162	1.7391
4/19/2019 4:55	0.1	-0.575	1.6426	-0.55	5.0694	26.5417	0.1539	1.7309
4/19/2019 4:56	0.1083	-0.2333	3.1677	-0.2	4.9901	26.6	0.1652	1.739
4/19/2019 4:57	0.1	-0.6417	3.3675	-0.5667	4.9466	26.6	0.1476	1.7336
4/19/2019 4:58	0.1	-0.7417	3.2123	0.05	5.0013	26.6	0.1522	1.7163
4/19/2019 4:59	0.1	-0.35	3.3263	-0.5333	4.9546	26.6917	0.1477	1.7503

4/19/2019 5:00	0.1	-0.8417	3.2918	-0.2	4.9549	26.6583	0.153	1.7576
4/19/2019 5:01	0.1	-0.1	3.3738	-0.8667	4.9624	26.5727	0.1589	1.7545
4/19/2019 5:02	0.15	-0.5583	3.3359	-1.1167	4.88	26.5	0.1483	1.7483
4/19/2019 5:03	0.2	0.0917	3.3149	-1.15	4.8776	26.5	0.1614	1.7155
4/19/2019 5:04	0.2	-0.1667	3.5239	-0.2	4.8798	26.5	0.1448	1.7023
4/19/2019 5:05	0.2	-0.35	3.5278	-0.5333	4.891	26.5	0.1489	1.7179
4/19/2019 5:06	0.2	-0.1167	3.1488	-0.4	4.9058	26.5333	0.153	1.719
4/19/2019 5:07	0.2	-0.925	3.5456	-0.3	4.8713	26.5	0.144	1.728
4/19/2019 5:08	0.1917	-0.7333	3.466	0	4.8563	26.5	0.1516	1.7393
4/19/2019 5:09	0.1	-0.8833	3.7508	0.2	4.8707	26.41	0.141	1.7218
4/19/2019 5:10	0.1667	-0.7417	3.8248	-0.1167	4.8678	26.4778	0.1541	1.7163
4/19/2019 5:11	0.2	-0.35	3.4079	-0.05	4.9063	26.45	0.1517	1.7082
4/19/2019 5:12	0.2	-0.3167	3.581	-0.8833	4.901	26.5	0.1474	1.7218
4/19/2019 5:13	0.175	-0.625	3.7637	-0.2167	4.8987	26.5273	0.1528	1.7244
4/19/2019 5:14	0.1	-0.425	3.2915	-0.35	4.8548	26.6	0.1408	1.7113
4/19/2019 5:15	0.175	-1.025	3.4053	-0.2167	4.8504	26.6	0.1557	1.7176
4/19/2019 5:16	0.1583	-0.5667	3.6177	-0.5333	4.8792	26.6	0.1419	1.7318
4/19/2019 5:17	0.15	-0.5	3.3163	-0.0167	4.8412	26.6	0.1461	1.7401
4/19/2019 5:18	0.1	-0.5583	3.4873	-0.5167	4.7901	26.6	0.1432	1.7026
4/19/2019 5:19	0.0917	-0.775	3.5134	-0.2833	4.7615	26.67	0.1322	1.6973
4/19/2019 5:20	0.0167	-0.5	3.6468	-0.2833	4.8726	26.7	0.1542	1.6963
4/19/2019 5:21	0.0083	-0.5167	3.696	-1.0333	4.8224	26.78	0.1396	1.6783
4/19/2019 5:22	0	0	3.2504	-0.45	4.8589	26.8	0.14	1.6973
4/19/2019 5:23	0	-0.0833	3.3526	-0.7	4.8158	26.84	0.1429	1.6987
4/19/2019 5:24	0	-0.225	3.1128	-0.3	4.932	26.9	0.1339	1.713
4/19/2019 5:25	0	0.1333	3.3693	-1.05	4.9429	26.9	0.1461	1.7031
4/19/2019 5:26	0	-1.2917	3.3948	-0.85	4.9877	26.9182	0.1343	1.7252
4/19/2019 5:27	0	0.2333	3.3823	-0.5	5.0113	26.9546	0.1309	1.7497
4/19/2019 5:28	0	-0.1667	3.4397	-0.3667	4.9679	26.9636	0.1481	1.7399
4/19/2019 5:29	0	-0.6333	3.6111	-0.7333	4.9365	26.92	0.1295	1.7143
4/19/2019 5:30	0	-0.6167	3.2607	0.0167	4.8851	26.9364	0.1412	1.6845
4/19/2019 5:31	0	-0.2833	3.2318	-0.25	4.8331	26.9	0.1438	1.6709
4/19/2019 5:32	0.0167	-0.275	3.167	-0.2167	4.919	26.9273	0.1377	1.6964
4/19/2019 5:33	0.05	-0.85	3.338	-0.55	4.9105	27	0.1478	1.7103
4/19/2019 5:34	0.0417	-0.2083	3.8778	-0.5833	4.8208	27.025	0.1313	1.6922
4/19/2019 5:35	0.0833	0.175	3.6553	-0.4	4.9211	27.1	0.1455	1.6912
4/19/2019 5:36	0.0667	-0.95	3.5478	-0.2	4.9995	27.1	0.1379	1.6703
4/19/2019 5:37	0.1	-0.0417	3.1095	-0.6167	4.9218	27.1	0.1328	1.687
4/19/2019 5:38	0.075	0.15	3.6328	-0.0167	4.7898	27.1	0.1433	1.7026
4/19/2019 5:39	0.1	-0.1417	3.4168	-0.6833	4.8031	27.1	0.1292	1.7125
4/19/2019 5:40	0.1	-0.2917	3.3483	-0.2	4.9486	27.1	0.1436	1.6776
4/19/2019 5:41	0.1	0.0167	3.3852	-0.5667	4.8804	27.1182	0.139	1.6732
4/19/2019 5:42	0.175	-0.8167	3.0278	-0.65	4.7658	27.1667	0.1277	1.6725
4/19/2019 5:43	0.1917	-0.5	3.5367	-0.75	4.8643	27.2	0.1452	1.6831
4/19/2019 5:44	0.2	-0.55	3.6317	-0.5	4.9223	27.225	0.13	1.6879
4/19/2019 5:45	0.2	-0.0083	3.2837	-0.7833	4.9371	27.2636	0.1411	1.6569
4/19/2019 5:46	0.2	-1.075	3.8078	-0.8833	4.8848	27.3	0.1392	1.6906

4/19/2019 5:47	0.1917	-0.3833	3.4915	-1.4167	4.922	27.2636	0.1302	1.7001
4/19/2019 5:48	0.1	-0.5833	3.386	-1.25	4.8504	27.3	0.1404	1.6895
4/19/2019 5:49	0.1	0.1333	3.4733	-0.9	4.8838	27.3	0.1365	1.6977
4/19/2019 5:50	0.1	-0.525	3.4192	-0.3167	4.9138	27.3	0.1379	1.6852
4/19/2019 5:51	0.1	0.0667	3.5385	-0.8	4.8991	27.3	0.14	1.6901
4/19/2019 5:52	0.1	-0.2417	3.4762	-0.4	4.9298	27.3	0.1276	1.6828
4/19/2019 5:53	0.1083	0.0083	3.4318	-0.6	4.9092	27.3546	0.1346	1.684
4/19/2019 5:54	0.1	-0.6667	3.4728	-0.2	4.9584	27.4	0.1315	1.6871
4/19/2019 5:55	0.1667	0.475	3.7415	-0.5667	4.9983	27.48	0.1363	1.687
4/19/2019 5:56	0.1	-0.2583	3.3777	-0.0667	5.138	27.5	0.138	1.6918
4/19/2019 5:57	0.1	-0.9167	3.3598	-0.2833	5.1633	27.575	0.1321	1.6848
4/19/2019 5:58	0.1	-0.1333	3.4315	-1.0167	5.1851	27.6	0.1415	1.6891
4/19/2019 5:59	0.1583	-0.55	3.462	-0.8333	5.1886	27.6667	0.1412	1.6944
4/19/2019 6:00	0.2	-0.15	3.5833	-0.7333	5.3173	27.725	0.1356	1.7075
4/19/2019 6:01	0.2	-0.6417	3.3875	-0.1	5.2677	27.8333	0.1434	1.6834
4/19/2019 6:02	0.175	-0.725	3.4897	-0.2833	5.3213	27.9917	0.1323	1.6813
4/19/2019 6:03	0.1	-0.3	3.417	-0.3	5.3382	28.1545	0.1445	1.6794
4/19/2019 6:04	0.1	-0.4	3.517	-0.1667	5.1469	28.3333	0.1395	1.7018
4/19/2019 6:05	0.1	0.3167	3.2598	-0.1667	5.1759	28.3833	0.1293	1.698
4/19/2019 6:06	0.1583	-0.425	3.4768	-1.1	5.1724	28.4727	0.1439	1.691
4/19/2019 6:07	0.1	-0.5417	3.459	-0.5333	5.24	28.5	0.1317	1.6646
4/19/2019 6:08	0.1	-0.4583	3.4382	-0.3167	5.2333	28.5583	0.1297	1.7012
4/19/2019 6:09	0.1	-0.5917	4.2123	0.4833	5.2202	28.5273	0.1478	1.7168
4/19/2019 6:10	0.1583	-0.4667	2.8882	-0.1333	5.2258	28.3273	0.1318	1.7154
4/19/2019 6:11	0.2	-0.1917	3.4465	-0.5	5.1513	28.6167	0.1403	1.9805
4/19/2019 6:12	0.2	-0.0583	3.3155	-0.7833	5.0794	29.1091	0.1425	3.4963
4/19/2019 6:13	0.2	-0.65	3.1382	-0.2667	5.089	29.5833	0.1356	1.9262
4/19/2019 6:14	0.2	-0.2667	3.2062	-0.2	5.0888	29.93	0.1426	1.7124
4/19/2019 6:15	0.125	-0.0083	3.3328	-0.6333	5.1873	30.125	0.1308	1.6968
4/19/2019 6:16	0.1	-0.825	3.1577	-0.5	5.0263	30.21	0.1422	1.6785
4/19/2019 6:17	0.1	-0.6333	3.1842	-0.7333	4.9573	30.3583	0.1404	1.707
4/19/2019 6:18	0.075	-0.3167	3.1138	-0.25	5.0218	30.3333	0.1386	1.7177
4/19/2019 6:19	0.1	-0.3167	3.3672	-0.6167	5.0729	30.375	0.1448	1.6928
4/19/2019 6:20	0.125	-0.825	3.4148	-0.05	5.1324	30.4	0.1354	1.6823
4/19/2019 6:21	0.2	-0.3583	3.4348	-0.2333	5.0442	30.4333	0.1428	1.6974
4/19/2019 6:22	0.2	0.175	3.4157	-0.4	5.0315	30.7546	0.1441	1.6832
4/19/2019 6:23	0.2	-0.0167	3.1888	-0.0333	5.0172	30.6583	0.1311	1.6833
4/19/2019 6:24	0.2	0.0917	3.2832	-0.3833	5.0733	30.22	0.1492	1.6722
4/19/2019 6:25	0.2	-0.1333	3.546	-0.3333	5.0628	29.6417	0.1392	1.6882
4/19/2019 6:26	0.2	-0.3833	3.5205	-0.1667	5.0498	29.2364	0.1416	1.7012
4/19/2019 6:27	0.2	0.4333	3.3917	-0.8	5.0514	28.9546	0.1464	1.692
4/19/2019 6:28	0.1917	-0.5	2.9805	-0.6667	5.1054	28.8	0.1345	1.7144
4/19/2019 6:29	0.1083	0.0833	3.2017	-1.1167	5.1282	28.8	0.1472	1.714
4/19/2019 6:30	0.1	0.2167	3.4033	-0.6	5.1683	28.8	0.1441	1.7158
4/19/2019 6:31	0.1	-0.575	3.4578	-0.6333	5.1395	28.8818	0.1363	1.7388
4/19/2019 6:32	0.1	0.0333	3.555	-0.6333	5.168	28.9	0.1468	1.7369
4/19/2019 6:33	0.1909	-0.3083	3.0633	-0.6333	5.2176	28.9	0.1346	1.7383

4/19/2019 6:34	0.2	-0.2667	3.305	-0.7	5.1038	28.94	0.1429	1.7102
4/19/2019 6:35	0.175	0.225	3.3128	-0.3167	5.1148	29	0.1466	1.7099
4/19/2019 6:36	0.1	-0.4333	3.2468	-0.05	5.1323	29.025	0.1364	1.7419
4/19/2019 6:37	0.075	0.4083	3.4225	-0.7333	5.0327	29.1333	0.1443	1.7349
4/19/2019 6:38	0	-0.25	3.084	-1.3667	5.074	29.25	0.1423	1.7024
4/19/2019 6:39	0	-0.2333	3.1637	-1.2333	5.0018	29.3	0.1358	1.7107
4/19/2019 6:40	0.0917	-0.1333	3.7438	-0.9	5.0585	29.3182	0.1508	1.734
4/19/2019 6:41	0.1	0.15	3.1368	0.1333	5.2012	29.4	0.133	1.7101
4/19/2019 6:42	0	-0.125	3.2697	-0.7333	5.1605	29.4	0.1499	1.7056
4/19/2019 6:43	0	0.0167	3.3948	-0.1333	5.1635	29.4833	0.1448	1.6873
4/19/2019 6:44	0	-0.275	3.1208	-0.5	5.2331	29.5636	0.1379	1.6761
4/19/2019 6:45	0	0.1667	3.2668	-0.5333	5.1406	29.6727	0.1492	1.7065
4/19/2019 6:46	0	-0.1333	3.1687	-0.2	5.1575	29.825	0.1415	1.7305
4/19/2019 6:47	0	0.175	3.1032	0.0833	5.3127	29.9	0.1403	1.7441
4/19/2019 6:48	0.0083	-0.5667	3.2102	-0.4	5.2901	29.9818	0.1542	1.9099
4/19/2019 6:49	0.05	-0.7083	3.1272	-0.9	5.292	30.0333	0.1378	1.9646
4/19/2019 6:50	0.1	-0.3833	3.423	-0.4	5.3679	30.1	0.1505	1.852
4/19/2019 6:51	0.0583	-0.0417	3.3753	-0.3	5.3896	30.1909	0.1486	1.7655
4/19/2019 6:52	0	-0.1417	3.2628	-0.0833	5.3458	30.2636	0.1411	1.7493
4/19/2019 6:53	0	-0.4	3.1295	-0.1667	5.3463	30.2	0.1479	1.7535
4/19/2019 6:54	0	-0.0667	3.3597	-0.2667	5.3561	30.2636	0.144	1.8297
4/19/2019 6:55	0.075	-0.0083	3.399	-0.45	5.3496	30.3	0.149	1.7911
4/19/2019 6:56	0.1	-0.8167	3.2587	-0.1333	5.3623	30.3	0.1535	1.8165
4/19/2019 6:57	0.1	0.4083	3.2342	-0.2667	5.3767	30.3	0.1378	1.8343
4/19/2019 6:58	0.1	-0.1667	2.9933	-0.5833	5.4018	30.3	0.1518	1.7791
4/19/2019 6:59	0.1	-0.525	3.2208	-0.7167	5.4018	30.2917	0.1445	1.8113
4/19/2019 7:00	0.0417	-0.4417	3.2082	-0.65	5.3803	30.15	0.1447	1.8225
4/19/2019 7:01	0.0667	-0.0417	3.1198	-1.1167	5.3663	30.2	0.1534	1.8168
4/19/2019 7:02	0.0333	-0.2583	3.1052	-0.6333	5.3433	30.25	0.1442	1.7862
4/19/2019 7:03	0.0083	-0.3667	3.2983	-0.4833	5.3862	30.0727	0.1543	1.7852
4/19/2019 7:04	0.0917	-0.5333	3.1442	-1.0833	5.3731	29.6	0.1533	1.8188
4/19/2019 7:05	0.0083	0.1083	3.2076	-0.5833	5.3896	29.6	0.1418	1.8271
4/19/2019 7:06	0.0917	-0.4667	3.1275	-0.3333	5.3899	29.675	0.1589	1.8598
4/19/2019 7:07	0.0583	0.5833	3.4818	-0.2833	5.3961	29.7222	0.1509	1.8171
4/19/2019 7:08	0.1	0.1583	3.1265	-0.5667	5.416	29.8	0.1536	1.8003
4/19/2019 7:09	0.1	0.0083	3.1583	-0.4167	5.4115	29.8546	0.1583	1.8074
4/19/2019 7:10	0.1167	0.0333	3.4458	-0.7333	5.4473	29.9	0.1447	1.8125
4/19/2019 7:11	0.1917	0.4333	2.8987	-0.7833	5.4293	29.9	0.1581	1.7915
4/19/2019 7:12	0.2	0.4667	2.881	-0.9833	5.4053	30.04	0.1566	1.8122
4/19/2019 7:13	0.2	-0.4917	3.2951	-0.3	5.3774	30.1727	0.1454	1.7926
4/19/2019 7:14	0.2	-0.8167	3.4588	-0.45	5.398	30.1727	0.1563	1.8273
4/19/2019 7:15	0.2	0.3	3.3101	-0.5333	5.398	30.2636	0.1483	1.8634
4/19/2019 7:16	0.2	-0.0583	3.2758	-0.2667	5.3933	30.8364	0.1518	1.8345
4/19/2019 7:17	0.1917	-0.3333	3.2969	-0.3	5.4809	31.1636	0.1567	1.8568
4/19/2019 7:18	0.1833	0.3667	4.0137	-0.65	6.2731	31.45	0.1733	1.887
4/19/2019 7:19	0.175	0.3	7.2578	0.25	8.4864	32.1	0.288	2.0027
4/19/2019 7:20	0.2	0.3083	11.1419	1.4167	11.8373	33.6818	0.4329	2.1414

4/19/2019 7:21	0.2	-0.3333	14.9567	2.0667	12.8174	35.4455	0.762	2.1303
4/19/2019 7:22	0.2	-0.0833	16.0092	2.1	13.3944	37.8	0.7685	1.997
4/19/2019 7:23	0.2	-0.025	17.3633	2.2	14.2763	40.3818	1.0225	1.9586
4/19/2019 7:24	0.2	0.55	16.5	2.6	14.2878	43.2833	1.0466	1.9489
4/19/2019 7:25	0.2	-0.0333	16.6625	2.45	14.3499	46.425	1.0069	1.9623
4/19/2019 7:26	0.2	-0.3583	16.2658	2.25	14.1955	49.3091	0.9215	1.9756
4/19/2019 7:27	0.2	-0.45	15.765	2.4667	13.9312	52.4364	0.897	1.9668
4/19/2019 7:28	0.175	0.8417	15.7425	2.4	13.7736	55.125	0.873	1.9939
4/19/2019 7:29	0.2	0.5667	15.5067	2.6333	13.8283	57.9667	0.8922	1.964
4/19/2019 7:30	0.1083	-0.1917	14.5325	1.8667	13.5383	60.575	0.8667	1.9897
4/19/2019 7:31	0.1083	-0.175	15.1258	2.5833	13.5878	62.88	0.8706	1.9978
4/19/2019 7:32	0.1	0.3417	14.9367	2.1333	13.479	64.4167	0.8764	1.9838
4/19/2019 7:33	0.1	0.4333	14.7658	1.4667	13.4272	65.3091	0.8758	2.0161
4/19/2019 7:34	0.1917	-0.4917	14.9667	2.3167	13.2375	65.8667	0.8528	2.0071
4/19/2019 7:35	0.2	0.475	14.0267	1.8	13.2119	66.0545	0.8711	2.0259
4/19/2019 7:36	0.225	-0.1333	14.1317	2.1	13.1373	65.8273	0.8627	2.0198
4/19/2019 7:37	0.3	0.2167	14.1342	2	13.0344	65.45	0.8578	2.0135
4/19/2019 7:38	0.2917	0.15	13.7167	1.7333	12.8308	65.0273	0.8484	2.0039
4/19/2019 7:39	0.2	-0.5917	12.9192	1.8833	12.5759	64.625	0.8178	2.0298
4/19/2019 7:40	0.2	0.025	12.8225	1.8	12.5463	64.3182	0.8383	2.0475
4/19/2019 7:41	0.2	0.2917	12.71	1.7667	12.514	64	0.8188	2.055
4/19/2019 7:42	0.1917	0.2167	12.3517	1.35	12.3822	63.5455	0.8178	2.0306
4/19/2019 7:43	0.1333	0.2417	12.6542	1.5167	12.3167	63.3	0.8185	2.0546
4/19/2019 7:44	0.2	0.2417	12.7117	1.3	12.3166	62.9583	0.8167	2.054
4/19/2019 7:45	0.2	0.2	11.9425	1	12.0872	62.5455	0.8127	2.0899
4/19/2019 7:46	0.2	-0.1167	11.7775	1.2667	11.9785	62.18	0.7929	2.0988
4/19/2019 7:47	0.2	-0.3083	11.7433	1.4667	11.9073	61.85	0.7986	2.0893
4/19/2019 7:48	0.2	-0.0417	11.8567	1.25	11.9161	61.5167	0.8073	2.0651
4/19/2019 7:49	0.1833	0.0583	11.9192	0.9	11.7978	61.1727	0.7856	2.0776
4/19/2019 7:50	0.1	-0.1167	11.5367	1.3	11.6992	60.8333	0.7964	2.0539
4/19/2019 7:51	0.1	0.1083	11.4175	1.0333	11.6431	60.4909	0.7846	2.0538
4/19/2019 7:52	0.1	-0.1167	11.1467	1.15	11.5189	60.1818	0.7749	2.0528
4/19/2019 7:53	0.0333	0.15	10.9925	0.8833	11.4358	59.7364	0.7832	2.0452
4/19/2019 7:54	0	-0.625	11.0492	1.25	11.3564	59.2583	0.7634	2.0788
4/19/2019 7:55	0	0.3	10.9633	1	11.3015	59	0.7667	2.1073
4/19/2019 7:56	0	0.05	10.4223	0.7667	11.2185	58.6	0.7573	2.1055
4/19/2019 7:57	0	-0.3333	10.8067	1.6667	11.1453	58.17	0.746	2.1159
4/19/2019 7:58	0	0.0667	10.5917	1.9167	11.0726	57.9909	0.7662	2.0868
4/19/2019 7:59	0	0.5083	10.2692	1.55	10.9574	57.8546	0.7412	2.099
4/19/2019 8:00	0	0.3917	10.485	1.8	10.9036	57.6182	0.7425	2.0651
4/19/2019 8:01	0	0.4083	10.2942	1.3667	10.8233	57.3455	0.7512	2.0778
4/19/2019 8:02	0	0.2	9.8287	0.65	10.7694	57.0636	0.7278	2.0889
4/19/2019 8:03	0	0.4167	9.422	1.1	10.6781	56.8727	0.7392	2.1453
4/19/2019 8:04	0.05	0.6083	9.7747	0.7667	10.6024	56.6636	0.7174	2.1034
4/19/2019 8:05	0.0667	0.05	9.7508	1.0833	10.5503	56.4	0.7167	2.0904
4/19/2019 8:06	0.1	-0.0083	9.7736	0.9167	10.5043	56.4167	0.7193	2.0918
4/19/2019 8:07	0.1	-0.3333	9.1148	1.0833	10.4288	56.21	0.6936	2.109

4/19/2019 8:08	0.1	0	9.3142	1.0333	10.368	55.8	0.7109	2.111
4/19/2019 8:09	0.1	-0.1333	9.2113	0.9333	10.317	55.8	0.6974	2.088
4/19/2019 8:10	0.1	-0.425	9.6504	1.35	10.248	55.6667	0.6937	2.1079
4/19/2019 8:11	0.175	0.3	9.2872	0.9333	10.182	55.4818	0.6965	2.1085
4/19/2019 8:12	0.1917	-0.0917	8.9992	1.0833	10.132	55.2083	0.6833	2.0914
4/19/2019 8:13	0.1833	0.0583	9.3067	0.6	10.0874	55.0417	0.7004	2.0812
4/19/2019 8:14	0.1273	0.2917	9.1402	0.7	10.0278	54.875	0.6982	2.1212
4/19/2019 8:15	0.1167	0.3083	8.8317	0.7833	9.9932	54.7	0.6745	2.1228
4/19/2019 8:16	0.175	0.3	9.2748	0.6333	9.993	54.6167	0.6795	2.11
4/19/2019 8:17	0.2	-0.2417	8.8208	0.3333	9.9303	54.5818	0.6783	2.1461
4/19/2019 8:18	0.2	0.1333	8.7683	0.7667	9.8	54.55	0.6766	2.126
4/19/2019 8:19	0.2	0.0333	8.672	0.55	9.7465	54.4455	0.6697	2.0703
4/19/2019 8:20	0.2	0.0417	8.5807	0.9667	9.6759	54.5167	0.656	2.0817
4/19/2019 8:21	0.2	-0.1667	8.551	0.3833	9.6388	54.3455	0.6658	2.1443
4/19/2019 8:22	0.2	0.2417	8.8098	0.25	9.6075	54.2273	0.6629	2.1623
4/19/2019 8:23	0.2	0.0583	8.4807	0.35	9.5192	53.9917	0.6468	2.1509
4/19/2019 8:24	0.2083	-0.25	8.4143	0.7167	9.5023	53.5083	0.6558	2.1256
4/19/2019 8:25	0.2	-0.35	8.1239	1.0333	9.4746	53.125	0.6416	2.1458
4/19/2019 8:26	0.2	0.3167	8.383	0.5833	9.4093	52.95	0.6474	2.1502
4/19/2019 8:27	0.1333	0.1	8.3869	0.45	9.355	52.7833	0.6455	2.1607
4/19/2019 8:28	0.1	-0.2333	7.8508	0.5	9.2482	52.5917	0.6339	2.1533
4/19/2019 8:29	0.1	-0.0167	8.1248	0.8667	9.2222	52.3333	0.6322	2.116
4/19/2019 8:30	0.1	-0.075	7.8533	1.2	9.1893	52.125	0.6246	2.0774
4/19/2019 8:31	0.1	-0.1	7.9272	0.45	9.145	51.9455	0.6259	2.1167
4/19/2019 8:32	0.1	0.0667	7.8257	0.4667	9.1138	51.575	0.6278	2.1394
4/19/2019 8:33	0.1	-0.4083	7.6268	0.3667	9.0774	51.275	0.6111	2.1178
4/19/2019 8:34	0.1	-1.1167	7.6403	0.7	9.0219	50.9909	0.629	2.1197
4/19/2019 8:35	0.1	-0.3333	7.8425	0.8	8.9924	50.7727	0.6067	2.1179
4/19/2019 8:36	0.1	0.475	7.3622	0.5667	8.9362	50.5273	0.6051	2.1118
4/19/2019 8:37	0.1	-0.2333	7.1268	0.4833	8.943	50.5727	0.5884	2.1165
4/19/2019 8:38	0.1583	-0.1167	6.9293	0.45	9.1063	50	0.5592	2.0963
4/19/2019 8:39	0.2667	0.3	6.5302	0.8667	9.4736	49.2818	0.5204	2.0319
4/19/2019 8:40	0.4167	0.7583	5.9413	0.8	9.8113	48.6833	0.4716	1.9642
4/19/2019 8:41	0.575	0.8	5.8413	0.9833	10.0425	48.1	0.4383	1.9187
4/19/2019 8:42	0.6333	0.7167	5.3277	1.15	10.2637	47.2417	0.4202	1.9016
4/19/2019 8:43	0.7	0.4417	5.2418	0.85	10.5285	46.3	0.38	1.8332
4/19/2019 8:44	0.7	0.9083	4.7113	0.8833	10.6271	45.3	0.3671	1.7533
4/19/2019 8:45	0.725	0.8833	4.6122	1.25	10.7749	44.5091	0.3649	1.7534
4/19/2019 8:46	0.8	1.6583	4.3363	1.4667	10.8584	43.1909	0.3286	1.7178
4/19/2019 8:47	0.875	1.175	4.4745	0.5	10.9519	41.7818	0.3233	1.7116
4/19/2019 8:48	0.9833	1.35	4.5187	0.5167	10.933	40.6091	0.2998	1.6771
4/19/2019 8:49	1.1	1.15	4.7592	1.7833	10.9153	39.4182	0.2922	1.6743
4/19/2019 8:50	1.3333	1.55	4.6648	2.3	10.7571	38.1	0.2862	1.6583
4/19/2019 8:51	1.7	2.2417	5.125	2.8667	10.7405	37.1364	0.2609	1.6613
4/19/2019 8:52	2.1083	2.6333	5.4747	3.0167	10.7139	35.7	0.2586	1.6378
4/19/2019 8:53	2.475	3.15	4.3473	2.5167	10.7918	34.0727	0.2482	1.5834
4/19/2019 8:54	2.8727	3.9	4.652	3.65	10.779	32.3583	0.2322	1.5556

4/19/2019 8:55	3.0917	3.6583	5.5765	3.8333	10.7888	31.13	0.2337	1.5693
4/19/2019 8:56	3.3167	3.6417	5.3678	3.65	10.811	30.15	0.2226	1.5778
4/19/2019 8:57	3.3917	3.6333	5.3648	3.6167	10.7795	29.2273	0.232	1.5448
4/19/2019 8:58	3.4833	3.9833	5.7575	3.5333	10.637	28.3583	0.2249	1.5249
4/19/2019 8:59	3.5667	4.025	5.874	3.8167	10.4559	27.7	0.2111	1.5258
4/19/2019 9:00	3.7583	4.3917	5.787	4.4833	10.3401	27.0182	0.2153	1.5212
4/19/2019 9:01	3.85	4.1417	6.058	4.15	10.4042	26.2546	0.2095	1.5049
4/19/2019 9:02	3.95	4.525	6.1587	4.1333	10.0973	25.6091	0.205	1.5141
4/19/2019 9:03	4.1167	3.625	5.975	4.8333	9.9474	25.0909	0.2081	1.4904
4/19/2019 9:04	4.2167	4.075	6.1353	4.5	9.8372	24.725	0.1898	1.4965
4/19/2019 9:05	4.35	4.4333	6.4217	4.3833	9.7375	24.4091	0.1984	1.4893
4/19/2019 9:06	4.4083	5.4	6.583	4.8667	9.5497	24.5833	0.1995	1.4444
4/19/2019 9:07	4.5333	5.5667	6.5727	5.25	9.4188	24.4091	0.1839	1.4741
4/19/2019 9:08	4.7333	5.4417	6.7698	5.2167	9.3196	23.9636	0.1932	1.4755
4/19/2019 9:09	4.75	5	6.495	4.6667	9.4303	23.5546	0.1883	1.4699
4/19/2019 9:10	4.8083	4.825	6.8097	5.05	9.3938	23.2091	0.18	1.4424
4/19/2019 9:11	4.8	5.075	6.6797	5.1833	9.3292	22.925	0.1927	1.4573
4/19/2019 9:12	4.8583	5.7333	6.8218	5.1667	9.2699	22.56	0.1707	1.4808
4/19/2019 9:13	4.8583	5.4833	6.675	5.5833	9.2299	22.25	0.1815	1.4371
4/19/2019 9:14	4.9333	5.5	6.6925	5.1333	9.1243	21.8818	0.1831	1.413
4/19/2019 9:15	4.9917	5.425	6.7242	5.2167	9.0518	21.4546	0.1692	1.4199
4/19/2019 9:16	5.0083	5.775	6.713	5.2833	9.2188	21.13	0.1692	1.4007
4/19/2019 9:17	4.7833	4.225	5.8408	4.3333	9.211	20.5083	0.1784	1.4231
4/19/2019 9:18	4.5	3.9	10.8542	4.2333	9.3003	19.3	0.164	1.405
4/19/2019 9:19	4.175	3.45	5.825	3.7833	9.5584	17.83	0.1801	1.3816
4/19/2019 9:20	3.9833	3.875	5.4578	4.2167	9.5953	16.7727	0.1816	1.3985
4/19/2019 9:21	3.75	3.9583	5.404	3.2	9.63	15.4333	0.1743	1.4388
4/19/2019 9:22	3.5083	3.6167	5.0502	3.4333	9.6513	14.3417	0.1834	1.4613
4/19/2019 9:23	3.2667	3.9833	5.455	3.0833	9.6892	13.1273	0.1742	1.4688
4/19/2019 9:24	3.0818	3.5667	5.0498	3.4167	9.702	11.7818	0.1737	1.5198
4/19/2019 9:25	2.9	3.3167	5.1288	2.5667	9.6708	10.4909	0.1931	1.5171
4/19/2019 9:26	2.775	3.1917	4.9295	2.1667	9.6988	9.7545	0.1719	1.4971
4/19/2019 9:27	2.7	2.75	5.3108	2.8	9.7173	9.33	0.1817	1.5239
4/19/2019 9:28	2.6167	2.45	4.9953	2.0167	9.6908	8.3917	0.1853	1.5498
4/19/2019 9:29	2.575	1.9333	4.7188	1.8833	9.6881	7.34	0.1787	1.5834
4/19/2019 9:30	2.4667	2.1417	4.6855	1.7333	9.6366	6.9833	0.1892	1.583
4/19/2019 9:31	2.3333	2.3917	4.6717	2.5333	9.8926	7.19	0.1948	1.6203
4/19/2019 9:32	2.0583	1.8417	4.6218	1.5833	12.9564	8.4455	0.2306	1.685
4/19/2019 9:33	1.6417	1.2083	7.8423	1.1167	19.7528	33.7667	0.4454	1.7672
4/19/2019 9:34	1.0833	3.4083	48.8983	11.8	36.6443	64.56	1.1831	1.9851
4/19/2019 9:35	0.525	0.9417	66.8417	11.8667	42.5527	87.7917	1.6033	2.2073
4/19/2019 9:36	0.175	1.1	58.675	9.3	42.2381	89.2182	1.5173	2.2703
4/19/2019 9:37	0.025	3.1667	85.1833	13.7167	45.8639	107.0833	1.9867	2.6604
4/19/2019 9:38	0	0.8167	136.1167	22.0167	51.4184	158.3	4.2579	2.6089
4/19/2019 9:39	-0.0667	1.3917	124.2333	18.15	49.6476	142.4	4.4733	2.5702
4/19/2019 9:40	-0.0583	2.5417	126.1333	17.6167	48.967	145.28	3.9071	2.594
4/19/2019 9:41	0	2.0917	123.4833	17.4167	47.1376	155.7818	4.1474	2.6313

4/19/2019 9:42	0.0083	1.6917	121.4667	16.5667	46.1563	160.89	4.592	2.657
4/19/2019 9:43	0	1.7833	118.1	16.3167	45.1604	175.7167	4.5455	2.699
4/19/2019 9:44	0.05	1.8083	117.3667	16.0333	44.9156	189.8364	5.0766	2.6829
4/19/2019 9:45	0.075	1.975	114.1667	15.9333	43.9293	182.35	4.3792	2.6647
4/19/2019 9:46	0.1	1.875	113.8833	15.75	44.0245	186.7182	4.9768	2.6244
4/19/2019 9:47	0.0167	2.2	109.5667	15.25	42.843	186.7455	4.1804	2.6438
4/19/2019 9:48	0	1.4583	108.4167	15.4333	42.5368	182.7273	4.6915	2.6673
4/19/2019 9:49	0	1.2667	105.3833	15.1667	41.5604	176.7333	4.2781	2.6319
4/19/2019 9:50	0	2.125	102.2	15.1167	40.6443	174.8333	3.8128	2.6279
4/19/2019 9:51	0	2.1583	100.6167	14.7167	40.1484	174.0833	3.8459	2.6263
4/19/2019 9:52	0	1.9833	99.4183	14.7	39.6995	173.2455	3.9258	2.6067
4/19/2019 9:53	0.0917	1.7	97.925	14.0167	39.3162	172.36	3.8619	2.6278
4/19/2019 9:54	0.0818	1.0667	93.755	14.15	38.1484	171.06	3.5853	2.6383
4/19/2019 9:55	0.0417	1.9083	93.4667	14.1333	37.8753	169.4417	3.5496	2.6023
4/19/2019 9:56	0.0083	1.5167	91.8017	13.8167	37.481	167.7	3.564	2.5914
4/19/2019 9:57	0.0833	1.8167	90.2517	13.55	36.9073	165.4636	3.4638	2.612
4/19/2019 9:58	0.1	0.6417	88.0317	13.3	36.1867	163.23	3.459	2.6035
4/19/2019 9:59	0.1	1.2667	87.8783	13.3667	35.8549	161.0091	3.4951	2.5892
4/19/2019 10:00	0.1	1.3167	85.7883	13.1	35.3293	158.67	3.3359	2.5687
4/19/2019 10:01	0.1	1.375	84.7667	12.95	34.8431	156.0364	3.2904	2.5884
4/19/2019 10:02	0.15	1.3	82.4742	12.4	33.8484	153.1778	3.2051	2.5891
4/19/2019 10:03	0.1083	1.2167	82.0525	12.0667	33.8006	151	3.1999	2.5684
4/19/2019 10:04	0.1417	1.6167	75.5267	12	33.0039	148.8	3.1304	2.5778
4/19/2019 10:05	0.2	1.8	78.9008	12.6833	32.6159	146.4	3.1259	2.5903
4/19/2019 10:06	0.2	1.4	77.0283	12.4833	31.9896	143.4583	3.0806	2.576
4/19/2019 10:07	0.2	1.6	76.0833	12.0167	31.5798	140.7273	3.0728	2.5563
4/19/2019 10:08	0.2083	1.675	74.9967	12.0333	31.1768	139.0091	3.0448	2.546
4/19/2019 10:09	0.2083	1.5417	74.4517	11.8333	30.8128	137.4182	3.0198	2.5493
4/19/2019 10:10	0.2	1.6667	72.91	11.5667	30.2311	135.8083	3.0048	2.6041
4/19/2019 10:11	0.2083	0.8917	72.6017	11.3167	30.1879	134.44	2.9931	2.5368
4/19/2019 10:12	0.2667	1.2083	71.4617	11.55	29.6747	133.1364	2.9648	2.5191
4/19/2019 10:13	0.3	1.875	69.3767	11.4833	28.9686	131.55	2.9258	2.5684
4/19/2019 10:14	0.3	1.5167	68.6258	11.1833	28.5946	130.3364	2.8891	2.5823
4/19/2019 10:15	0.2917	1.7167	67.78	10.6833	28.4298	128.7636	2.8902	2.5263
4/19/2019 10:16	0.2583	1.3833	67.0942	10.5167	27.8693	127.3364	2.8608	2.5066
4/19/2019 10:17	0.1833	1.1833	65.9675	10.0833	27.4882	126.32	2.8318	2.5139
4/19/2019 10:18	0.1	1.5083	62.4067	9.7833	27.463	125.69	2.8244	2.5093
4/19/2019 10:19	0.1	1.275	63.4558	10.4333	27.217	125.54	2.7953	2.516
4/19/2019 10:20	0.1083	1.675	63.5958	10.4667	26.4036	123.72	2.7795	2.5235
4/19/2019 10:21	0.1833	1.1667	63.1308	10.35	26.0902	122.125	2.778	2.5365
4/19/2019 10:22	0.1917	1.5083	61.6733	10.6667	25.6033	121.23	2.7394	2.5433
4/19/2019 10:23	0.1583	1.075	60.315	9.65	25.3177	120.0333	2.7255	2.5221
4/19/2019 10:24	0.1091	0.175	60.2517	9.5667	25.0288	118.9182	2.6991	2.5111
4/19/2019 10:25	0.1	1.15	55.6542	9.1833	24.5596	117.7909	2.6673	2.5256
4/19/2019 10:26	0.0333	0.575	58.4825	9.7333	24.4533	116.7556	2.6726	2.4984
4/19/2019 10:27	0.0333	1.7667	57.9492	9.25	23.9487	116.03	2.6298	2.4957
4/19/2019 10:28	0.05	1.1833	57.81	9.2167	23.8105	115.29	2.6321	2.5015

4/19/2019 10:29	0.0917	1.2667	56.8108	9.8667	23.4711	114.3636	2.6144	2.4793
4/19/2019 10:30	0.1	1.7667	56.2133	9.4833	23.2568	113.3818	2.5919	2.4608
4/19/2019 10:31	0.1	0.8333	55.235	9.4167	22.8861	112.49	2.5804	2.4557
4/19/2019 10:32	0.1	1.55	54.64	8.8167	22.5681	112.4889	2.552	2.4746
4/19/2019 10:33	0.1	0.925	54.1092	8.4333	22.289	112.2727	2.5336	2.4615
4/19/2019 10:34	0.1	1.1417	52.9033	8.6	21.925	110.9	2.5198	2.4523
4/19/2019 10:35	0.1	1.125	52.8833	8.45	21.7307	109.6727	2.4793	2.4403
4/19/2019 10:36	0.1	1.0417	52.0358	8.35	21.3688	108.7909	2.4841	2.4483
4/19/2019 10:37	0.1	0.825	51.4492	8.3333	21.128	108.0455	2.4586	2.5003
4/19/2019 10:38	0.1	0.5417	51.1183	8.65	20.9093	107.3364	2.4458	2.4718
4/19/2019 10:39	0.1	0.975	49.9842	8.0167	20.5686	106.4417	2.4332	2.4472
4/19/2019 10:40	0.1	1.2083	49.4325	8.1167	20.3651	105.43	2.3967	2.4495
4/19/2019 10:41	0.15	0.9583	48.7367	8.4167	20.0403	104.6364	2.3939	2.4888
4/19/2019 10:42	0.15	0.825	48.3717	7.8667	19.8295	103.8	2.3736	2.4632
4/19/2019 10:43	0.2	0.8583	47.725	7.8833	19.5623	103.1091	2.3528	2.4342
4/19/2019 10:44	0.2	0.9	47.0333	8	19.3298	102.3	2.3469	2.4578
4/19/2019 10:45	0.2	1.3	46.895	7.25	19.0609	101.4167	2.3139	2.4433
4/19/2019 10:46	0.2	0.575	46.5608	6.9333	18.8452	100.9778	2.3078	2.4185
4/19/2019 10:47	0.1917	0.5583	45.705	7.4667	18.6426	100.6833	2.3002	2.4244
4/19/2019 10:48	0.1667	1.0417	45.2992	6.7833	18.4723	99.8273	2.2752	2.4093
4/19/2019 10:49	0.2	1.1833	45.0967	6.9167	18.2959	98.7667	2.2761	2.4008
4/19/2019 10:50	0.1667	0.5083	44.2508	6.55	17.9596	97.7583	2.2461	2.3981
4/19/2019 10:51	0.1083	0.4417	43.5467	6.4667	17.7288	97.23	2.2357	2.4221
4/19/2019 10:52	0.1	0.3417	42.425	7	17.5894	96.95	2.2225	2.4407
4/19/2019 10:53	0.1167	0.8083	42.015	7.1667	17.2983	96.1909	2.1796	2.4283
4/19/2019 10:54	0.1917	0.1667	41.9225	7.1833	17.0864	95.475	2.1855	2.4341
4/19/2019 10:55	0.1667	0.875	40.3667	6.9	16.7077	94.61	2.1297	2.4123
4/19/2019 10:56	0.1833	-0.1917	40.235	6.8167	16.5347	93.3273	2.1128	2.3999
4/19/2019 10:57	0.2	0.2833	39.2633	6.35	16.1893	92.54	2.1004	2.434
4/19/2019 10:58	0.2	1.4833	37.5575	5.8333	15.7003	91.6455	2.0322	2.4033
4/19/2019 10:59	0.2	0.7167	37.6158	6.3667	15.6647	90.8556	2.0416	2.3506
4/19/2019 11:00	0.1	0.6417	36.5242	5.9167	15.3903	90.1636	2.0017	2.3388
4/19/2019 11:01	0.1	0.5917	34.5383	5.7	14.8364	89.3	1.9047	2.3372
4/19/2019 11:02	0.1	0.35	34.6042	6.15	14.8032	88.4818	1.9299	2.2823
4/19/2019 11:03	0.1	0.025	32.8883	5.6333	14.4265	87.4	1.855	2.3139
4/19/2019 11:04	0.1	0.4667	33.8325	5.85	14.4734	86.1727	1.9048	2.2882
4/19/2019 11:05	0.0583	1.15	32.2333	5.35	14.0331	85.1091	1.8503	2.2618
4/19/2019 11:06	0	0.625	31.4217	5.2	13.7717	83.9364	1.7949	2.2601
4/19/2019 11:07	0	0.8083	31.2792	5.4833	13.7411	82.89	1.8119	2.245
4/19/2019 11:08	0	0.4667	31.13	4.8833	13.7298	82.5182	1.7867	2.2033
4/19/2019 11:09	-0.025	0.2417	30.6742	4.0833	13.6992	81.72	1.7542	2.1824
4/19/2019 11:10	-0.1	0.175	28.85	4.95	13.3957	80.8818	1.7044	2.152
4/19/2019 11:11	-0.05	0.3167	28.2067	4.15	13.4378	79.73	1.653	2.1533
4/19/2019 11:12	0	0.5417	26.0558	4.1167	13.3913	78.5182	1.5642	2.1634
4/19/2019 11:13	0.0917	0.075	24.1975	3.7667	13.3486	76.4636	1.4803	2.138
4/19/2019 11:14	0.1	0.3667	22.5758	3.2	13.4001	75.14	1.4182	2.0968
4/19/2019 11:15	0.1	0.6333	22.1575	3.7167	13.8583	73.8	1.3992	2.0708

4/19/2019 11:16	0.2	0.5	21.375	3.55	14.161	72.4455	1.3297	2.0192
4/19/2019 11:17	0.2	0.3667	19.76	3.4167	14.1673	70.8833	1.2682	1.9751
4/19/2019 11:18	0.2	0.8	19.2183	3.7167	14.3334	69.2	1.2405	1.9821
4/19/2019 11:19	0.1583	0.7333	16.9358	3.3333	14.359	67.3833	1.1488	1.9668
4/19/2019 11:20	0.1	0.3833	16.9417	2.25	14.3235	65.46	1.1173	1.9102
4/19/2019 11:21	0.1	0.2333	15.9925	2.8667	14.0973	63.6	1.0642	1.8817
4/19/2019 11:22	0.1	-0.2	15.4858	2.2833	13.2035	61.43	0.9684	1.8726
4/19/2019 11:23	0.1	0.5083	14.1793	2.8833	13.9983	59.3273	1.0006	1.8798
4/19/2019 11:24	0.1833	0.3583	11.4552	2.7	13.713	57.5727	0.9345	1.8691
4/19/2019 11:25	0.2	0.9083	13.39	3.2667	13.8503	56.43	0.9152	1.8388
4/19/2019 11:26	0.2417	1.175	12.5492	2.6167	13.7993	55.0727	0.885	1.8193
4/19/2019 11:27	0.3	1.0167	12.22	2.5167	13.5412	53.4818	0.8329	1.8073
4/19/2019 11:28	0.3417	1.5083	12.0483	2.7167	13.832	51.8417	0.8447	1.7879
4/19/2019 11:29	0.4167	0.6583	11.0592	2.2167	13.0226	50.3364	0.7671	1.7647
4/19/2019 11:30	0.55	1.5583	11.115	2.6333	13.3509	48.9455	0.768	1.7688
4/19/2019 11:31	0.6917	1.05	10.9025	2.5833	13.0896	47.8182	0.7329	1.7478
4/19/2019 11:32	0.775	1.55	10.3693	2.05	12.6578	46.42	0.6809	1.7443
4/19/2019 11:33	0.875	1.6417	10.4987	2.1333	13.0058	44.8	0.6931	1.704
4/19/2019 11:34	0.9583	1.575	10.1597	2.7667	12.4484	43.7727	0.6427	1.6989
4/19/2019 11:35	1.05	1.625	9.6445	2.55	12.3643	42.4818	0.6229	1.6871
4/19/2019 11:36	1.1583	1.6417	9.7067	2.6333	12.0602	41.37	0.6122	1.6918
4/19/2019 11:37	1.2667	1.7	9.4277	2.5333	11.7388	40.4636	0.5718	1.6545
4/19/2019 11:38	1.3583	1.6417	8.9957	2.6	11.0009	39.53	0.5443	1.6403
4/19/2019 11:39	1.4583	1.7417	9.1643	2.3833	10.9618	38.6364	0.5363	1.6468
4/19/2019 11:40	1.5833	2.4667	8.683	2.2667	10.6313	37.68	0.5147	1.6442
4/19/2019 11:41	1.6667	2.225	8.6995	2.6833	10.1128	36.7667	0.493	1.6323
4/19/2019 11:42	1.825	2.6	8.8262	2.95	10.2304	35.99	0.4788	1.6392
4/19/2019 11:43	1.9083	2.8	8.1157	3.3333	9.6274	35.2182	0.4676	1.6177
4/19/2019 11:44	2	2.4583	0.7838	2.55	9.1454	34.43	0.4422	1.6104
4/19/2019 11:45	2.025	3.3917	8.2502	3.2833	9.0443	34.1	0.4335	1.6233
4/19/2019 11:46	2.1	3.1167	8.3323	2.7667	8.7463	33.4111	0.4278	1.6283
4/19/2019 11:47	2.2	3.3167	8.6005	3.4833	8.6521	32.9364	0.4128	1.6117
4/19/2019 11:48	2.3	2.825	8.3442	3.3667	8.1988	32.52	0.4035	1.5788
4/19/2019 11:49	2.425	3.0167	8.3338	3.6667	8.0351	31.8909	0.3946	1.5698
4/19/2019 11:50	2.5583	3.3417	8.2978	3.9833	7.6299	31.3444	0.3646	1.5772
4/19/2019 11:51	2.7583	3.2333	8.6552	4.3	7.4965	30.9273	0.3772	1.5766
4/19/2019 11:52	2.875	3.5917	8.5157	4.3667	7.1232	30.47	0.3391	1.5486
4/19/2019 11:53	3	3.9	8.4542	4.05	6.8523	30.0636	0.3402	1.5418
4/19/2019 11:54	3.1167	4.5917	8.8368	4.2833	6.8903	29.54	0.3477	1.5301
4/19/2019 11:55	3.275	5.0833	9.4695	5.0667	6.821	29.2273	0.3362	1.5535
4/19/2019 11:56	3.4833	5.075	8.9853	4.3833	6.5108	28.98	0.3311	1.5784
4/19/2019 11:57	3.675	5.3417	9.3252	5.4333	6.3708	28.7364	0.3101	1.5885
4/19/2019 11:58	3.875	4.9	9.462	5.2	6.2588	28.4091	0.3117	1.571
4/19/2019 11:59	4.0333	5.0083	9.403	5.5333	6.1087	28.06	0.3054	1.55
4/19/2019 12:00	4.1	4.5417	9.0307	4.9333	5.8358	27.82	0.2795	1.5613
4/19/2019 12:01	4.1	4.7417	8.7173	4.6333	5.7816	27.5091	0.2919	1.5293
4/19/2019 12:02	4.1	5.9083	8.7835	4.8833	5.7555	27.3	0.2769	1.4988

4/19/2019 12:03	4.1833	5.275	8.6632	5.1167	5.6171	27.07	0.2722	1.5077
4/19/2019 12:04	4.2	5.175	8.9833	4.7	5.5212	26.9273	0.2784	1.4917
4/19/2019 12:05	4.1833	4.7917	8.8965	4.9333	5.4124	26.7818	0.2576	1.4956
4/19/2019 12:06	4.2	5.1417	8.729	5.4	5.346	26.7636	0.2672	1.5271
4/19/2019 12:07	4.2167	5.075	8.7073	4.8667	5.2459	26.6455	0.2567	1.5183
4/19/2019 12:08	4.2	5.2917	8.8667	4.75	5.1353	26.4546	0.2445	1.5339
4/19/2019 12:09	4.1083	4.7333	8.4355	5.2333	5.0911	26.3	0.2463	1.5151
4/19/2019 12:10	4	4.85	8.1873	4.5	5.0163	26.24	0.2293	1.5195
4/19/2019 12:11	3.9417	4.6417	8.43	5.1	4.999	26.25	0.2468	1.5037
4/19/2019 12:12	3.9417	3.875	8.3363	4.7167	4.9066	26.1417	0.2369	1.5093
4/19/2019 12:13	3.975	4.2417	8.0748	4.8667	4.8585	26.05	0.2378	1.5123
4/19/2019 12:14	3.9	3.55	8.5605	4.1833	4.8023	25.8	0.2342	1.5327
4/19/2019 12:15	3.7917	3.9	7.6753	4.6	4.7401	25.6818	0.2213	1.555
4/19/2019 12:16								
4/19/2019 12:17								
4/19/2019 12:18								
4/19/2019 12:19								
4/19/2019 12:20								
4/19/2019 12:21								
4/19/2019 12:22								
4/19/2019 12:23								
4/19/2019 12:24								
4/19/2019 12:25								
4/19/2019 12:26								
4/19/2019 12:27								
4/19/2019 12:28								
4/19/2019 12:29								
4/19/2019 12:30								
4/19/2019 12:31								
4/19/2019 12:32								
4/19/2019 12:33								
4/19/2019 12:34								
4/19/2019 12:35								
4/19/2019 12:36								
4/19/2019 12:37								
4/19/2019 12:38								
4/19/2019 12:39								
4/19/2019 12:40								
4/19/2019 12:41								
4/19/2019 12:42								
4/19/2019 12:43								
4/19/2019 12:44								
4/19/2019 12:45								
4/19/2019 12:46								
4/19/2019 12:47								
4/19/2019 12:48								
4/19/2019 12:49								

4/19/2019 12:50
4/19/2019 12:51
4/19/2019 12:52
4/19/2019 12:53
4/19/2019 12:54
4/19/2019 12:55
4/19/2019 12:56
4/19/2019 12:57
4/19/2019 12:58
4/19/2019 12:59
4/19/2019 13:00
4/19/2019 13:01
4/19/2019 13:02
4/19/2019 13:03
4/19/2019 13:04
4/19/2019 13:05
4/19/2019 13:06
4/19/2019 13:07
4/19/2019 13:08
4/19/2019 13:09
4/19/2019 13:10
4/19/2019 13:11
4/19/2019 13:12
4/19/2019 13:13
4/19/2019 13:14
4/19/2019 13:15
4/19/2019 13:16
4/19/2019 13:17
4/19/2019 13:18
4/19/2019 13:19
4/19/2019 13:20
4/19/2019 13:21
4/19/2019 13:22
4/19/2019 13:23
4/19/2019 13:24
4/19/2019 13:25
4/19/2019 13:26
4/19/2019 13:27
4/19/2019 13:28
4/19/2019 13:29
4/19/2019 13:30
4/19/2019 13:31
4/19/2019 13:32
4/19/2019 13:33
4/19/2019 13:34
4/19/2019 13:35
4/19/2019 13:36

4/19/2019 13:37				
4/19/2019 13:38				
4/19/2019 13:39				
4/19/2019 13:40				
4/19/2019 13:41				
4/19/2019 13:42				
4/19/2019 13:43	3.4958	217.86	0.1048	-0.9474
4/19/2019 13:44	3.7839	217.45	0.0803	-0.9474
4/19/2019 13:45	4.3859	215.825	0.0275	-0.9474
4/19/2019 13:46	4.2029	193.88	-0.014	-0.9474
4/19/2019 13:47	4.4991	160.7455	-0.0157	-0.9474
4/19/2019 13:48	4.8341	140.71	-0.0143	-0.9474
4/19/2019 13:49	4.8922	122.3364	0.0383	-0.9474
4/19/2019 13:50	4.8413	105.5889	0.072	-0.9474
4/19/2019 13:51	4.7456	90.5	0.1225	-0.9474
4/19/2019 13:52	4.5997	77.9667	0.1622	-0.9474
4/19/2019 13:53	4.4684	67.225	0.1809	-0.9474
4/19/2019 13:54	4.3233	58.5818	0.2123	-0.9474
4/19/2019 13:55	4.1562	50.3182	0.2352	-0.9474
4/19/2019 13:56	4.0794	44.35	0.2439	-0.9474
4/19/2019 13:57	4.0779	38.8417	0.2644	-0.9474
4/19/2019 13:58	4.2224	34.175	0.2769	-0.9474
4/19/2019 13:59	4.3493	32.9091	0.2718	-0.9474
4/19/2019 14:00	4.4759	32.2556	0.2928	-0.9474
4/19/2019 14:01	4.487	31.68	0.2876	-0.9474
4/19/2019 14:02				-0.9474
4/19/2019 14:03				-0.9474
4/19/2019 14:04				-0.9474
4/19/2019 14:05				-0.9474
4/19/2019 14:06				-0.9474
4/19/2019 14:07				-0.9474
4/19/2019 14:08				-0.9474
4/19/2019 14:09				-0.9474
4/19/2019 14:10				-0.9474
4/19/2019 14:11				2.2812
4/19/2019 14:12				4.9658
4/19/2019 14:13				5.1653
4/19/2019 14:14				5.1883
4/19/2019 14:15				4.6255
4/19/2019 14:16				2.9514
4/19/2019 14:17				2.082
4/19/2019 14:18				1.8998
4/19/2019 14:19				2.2381
4/19/2019 14:20				1.7579
4/19/2019 14:21				1.7059
4/19/2019 14:22				1.6033
4/19/2019 14:23				1.649

4/19/2019 14:24	1.611
4/19/2019 14:25	1.6457
4/19/2019 14:26	1.6456
4/19/2019 14:27	1.6553
4/19/2019 14:28	1.6578
4/19/2019 14:29	1.6323
4/19/2019 14:30	1.6128
4/19/2019 14:31	1.638
4/19/2019 14:32	1.6376
4/19/2019 14:33	1.6388
4/19/2019 14:34	1.6068
4/19/2019 14:35	1.5991
4/19/2019 14:36	1.6047
4/19/2019 14:37	1.6257
4/19/2019 14:38	1.619
4/19/2019 14:39	1.6158
4/19/2019 14:40	1.6568
4/19/2019 14:41	1.6395
4/19/2019 14:42	1.6428
4/19/2019 14:43	1.6521
4/19/2019 14:44	1.6392
4/19/2019 14:45	1.6254
4/19/2019 14:46	1.6482
4/19/2019 14:47	1.6679
4/19/2019 14:48	1.6535
4/19/2019 14:49	1.6252
4/19/2019 14:50	1.6238
4/19/2019 14:51	1.6388
4/19/2019 14:52	1.632
4/19/2019 14:53	1.6256
4/19/2019 14:54	1.6217
4/19/2019 14:55	1.6323
4/19/2019 14:56	1.6562
4/19/2019 14:57	1.6248
4/19/2019 14:58	1.632
4/19/2019 14:59	1.6208
4/19/2019 15:00	1.6239
4/19/2019 15:01	1.6375
4/19/2019 15:02	1.6366
4/19/2019 15:03	1.6182
4/19/2019 15:04	1.5888
4/19/2019 15:05	1.5801
4/19/2019 15:06	1.6024
4/19/2019 15:07	1.6155
4/19/2019 15:08	1.6049
4/19/2019 15:09	1.5997
4/19/2019 15:10	1.6181

4/19/2019 15:11	1.639
4/19/2019 15:12	1.658
4/19/2019 15:13	1.643
4/19/2019 15:14	1.6303
4/19/2019 15:15	1.605
4/19/2019 15:16	1.6023
4/19/2019 15:17	1.6273
4/19/2019 15:18	1.6162
4/19/2019 15:19	1.613
4/19/2019 15:20	1.5981
4/19/2019 15:21	1.6237
4/19/2019 15:22	1.6271
4/19/2019 15:23	1.6157
4/19/2019 15:24	1.6268
4/19/2019 15:25	1.6293
4/19/2019 15:26	1.6264
4/19/2019 15:27	1.6362
4/19/2019 15:28	1.6422
4/19/2019 15:29	1.6638
4/19/2019 15:30	1.6486
4/19/2019 15:31	1.6611
4/19/2019 15:32	1.6644
4/19/2019 15:33	1.6462
4/19/2019 15:34	1.6568
4/19/2019 15:35	1.6348
4/19/2019 15:36	1.6437
4/19/2019 15:37	1.6359
4/19/2019 15:38	1.633
4/19/2019 15:39	1.6421
4/19/2019 15:40	1.6668
4/19/2019 15:41	1.6407
4/19/2019 15:42	1.6388
4/19/2019 15:43	1.6304
4/19/2019 15:44	1.6546
4/19/2019 15:45	1.6575
4/19/2019 15:46	1.6133
4/19/2019 15:47	1.6192
4/19/2019 15:48	1.6388
4/19/2019 15:49	1.6283
4/19/2019 15:50	1.6028
4/19/2019 15:51	1.6551
4/19/2019 15:52	1.6499
4/19/2019 15:53	1.6439
4/19/2019 15:54	1.6528
4/19/2019 15:55	1.6428
4/19/2019 15:56	1.6516
4/19/2019 15:57	1.6577

4/19/2019 15:58	1.8583	-0.05	4.8564	-0.4167	2.545	6.5917	0.2375	1.6708
4/19/2019 15:59	1.35	0.25	5.0758	-0.3	2.5633	6.3091	0.2187	1.655
4/19/2019 16:00	1	0.6333	5.393	0.35	2.5904	6.1833	0.2204	1.6552
4/19/2019 16:01	1	0.3417	4.3798	0.05	2.612	6.3333	0.1986	1.6532
4/19/2019 16:02	1	0.45	4.4272	-0.35	2.6626	7.65	0.2131	1.6311
4/19/2019 16:03	1	0.725	4.8252	-0.3833	2.6584	14.45	0.1008	1.6487
4/19/2019 16:04	1	-0.3	5.1393	0.15	2.668	14.4	-0.0002	1.6488
4/19/2019 16:05	1	0.6083	4.4316	0	2.6879	12.8	0.0189	1.661
4/19/2019 16:06	1	0.3917	3.5953	0.05	2.9801	12.9909	-0.0023	1.6545
4/19/2019 16:07	1	-0.0083	5.0263	-0.0333	3.0208	13.4889	0.0089	1.6438
4/19/2019 16:08	1.0417	0.3167	4.9415	-0.0167	3.0403	13.6546	0.0038	1.6589
4/19/2019 16:09	1.0417	0.4083	4.5024	0.05	3.0684	13.3818	0.0015	1.632
4/19/2019 16:10	1.0667	0.5083	4.3768	-0.3667	3.0696	13.15	-0.001	1.6258
4/19/2019 16:11	1.1	0.2	4.9173	0.4	3.108	12.9222	-0.0056	1.6382
4/19/2019 16:12	1.1917	0.1917	4.6179	-0.1333	3.1016	12.6667	0.0802	1.6539
4/19/2019 16:13	1.2	0.6417	4.7312	-0.1333	3.1413	12.4364	0.1206	1.6672
4/19/2019 16:14	1.1583	0.1083	5.0631	-0.8167	3.1303	12.5	0.0038	1.6661
4/19/2019 16:15	1.125	0.1667	4.9335	-0.0333	3.1509	12.5091	0.0062	1.6787
4/19/2019 16:16	1.1583	0.0417	4.7645	-168.3333	3.1639	12.53	-0.0053	1.6723
4/19/2019 16:17	1.1417	0.95	4.5123	-0.3167	3.1613	12.71	0.0116	1.6711
4/19/2019 16:18	1.125	0.2583	4.1763	0.4167	3.211	13.57	-0.0054	1.6796
4/19/2019 16:19	1.1	0.7583	4.9268	0.25	3.1993	13.6182	0.0025	1.6775
4/19/2019 16:20	1.1	0.4167	4.7417	-0.05	3.2055	12.8909	0.003	1.6748
4/19/2019 16:21	1.1	0.5667	4.4023	-0.4	3.1925	12.825	-0.011	1.6796
4/19/2019 16:22	1.1167	0.2583	4.6443	0.2833	3.2114	13.22	0.0185	1.6488
4/19/2019 16:23	1.1917	-0.125	4.3298	0.6167	3.2378	14	-0.0095	1.6777
4/19/2019 16:24	1.2	1.0667	5.0148	-0.1	3.2173	14.1727	-0.0046	1.6643
4/19/2019 16:25	1.2	0.6083	5.1754	-0.2	3.2401	14.3167	-0.0031	1.6519
4/19/2019 16:26	1.2	-0.35	4.0319	-0.2833	3.2538	14.3417	-0.0199	1.6863
4/19/2019 16:27	1.1667	0.3083	4.8634	-0.05	3.2783	14.375	-0.0008	1.7167
4/19/2019 16:28	1.1167	0.4	4.2257	0.1333	3.2863	14.4333	-0.0099	1.6852
4/19/2019 16:29	1.1167	0.325	4.9378	0.0333	3.2973	14.45	0.0012	1.6921
4/19/2019 16:30	1.1	0.0583	4.6075	-0.3	3.3015	13.5818	-0.0066	1.6815
4/19/2019 16:31	1.125	0.2333	4.6493	-0.0167	3.3268	13.1818	-0.0142	1.6564
4/19/2019 16:32	1.1667	0.6333	5.2176	-0.2	3.366	13.4833	-0.0079	1.6614
4/19/2019 16:33	1.1583	0.3	5.1426	-0.3167	3.3615	14.36	0.1332	1.6551
4/19/2019 16:34	1.1	0.3083	4.9934	-0.0167	3.3949	15.25	0.1618	1.664
4/19/2019 16:35	1.0583	0.7917	5.0108	-0.1333	3.3706	15.0546	0.1791	1.6801
4/19/2019 16:36	1	-0.2	4.8267	-0.1333	3.3896	15.2	0.1886	1.6698
4/19/2019 16:37	1	0.7083	4.7526	0.6333	3.3945	15.67	0.1914	1.6632
4/19/2019 16:38	1	0.1167	4.9292	0.6	3.4002	15.9455	0.1866	1.6614
4/19/2019 16:39	1.0167	1.05	5.1396	0.4167	3.4082	16.28	0.1944	1.6614
4/19/2019 16:40	1.025	0.375	4.8842	-0.2	3.4043	17.06	0.1847	1.6632
4/19/2019 16:41	1.1	0.6667	4.7054	0.9167	3.4505	17.39	0.1835	1.6862
4/19/2019 16:42	1.1	0.0917	4.6628	0.2167	3.427	18.2	0.1918	1.6939
4/19/2019 16:43	1.1167	-0.475	4.935	-0.2667	3.4018	18.4909	0.1757	1.6723
4/19/2019 16:44	1.1	0.1667	4.79	-0.25	3.429	18.7273	0.1897	1.6421

4/19/2019 16:45	1.1	-0.1833	4.7631	0.2667	3.4318	18.8	0.1845	1.6681
4/19/2019 16:46	1.0833	0.0583	5.1503	-0.2	3.4378	18.45	0.1773	1.6467
4/19/2019 16:47	1	0.475	4.8441	0.1167	3.4095	18.6273	0.1917	1.6558
4/19/2019 16:48	1	-0.675	5.0546	0.2	3.4115	19.62	0.1824	1.6717
4/19/2019 16:49	1	-0.1583	4.8208	0.2333	3.4252	20.0273	0.1871	1.6832
4/19/2019 16:50	1.0583	0.1	4.777	-0.3333	3.4239	20.7	0.1812	1.6548
4/19/2019 16:51	1.1	0.225	4.679	-0.0167	3.4218	20.8727	0.1758	1.6413
4/19/2019 16:52	1.1	-0.5417	3.9568	-0.5	3.4265	20.7333	0.185	1.6562
4/19/2019 16:53	1.1	0.575	4.9274	0.0333	3.4355	20.98	0.1611	1.6577
4/19/2019 16:54	1.0917	0.575	4.8479	0.3333	3.432	20.7667	0.1821	1.6414
4/19/2019 16:55	1.075	-0.3	4.8157	-0.0333	3.4149	20.2455	0.1738	1.6406
4/19/2019 16:56	1.0083	-0.1083	4.8892	0.1167	3.4148	19.775	0.1723	1.6481
4/19/2019 16:57	1	0.3583	4.734	-0.3167	3.4114	19.6	0.1762	1.6297
4/19/2019 16:58	1.0333	0.625	4.8021	-0.3167	3.4305	19.3	0.1753	1.6168
4/19/2019 16:59	1.025	0.85	4.6707	0.6667	3.4083	18.86	0.1891	1.6401
4/19/2019 17:00	1	-0.075	4.721	0.15	3.4008	18.1545	0.1745	1.6693
4/19/2019 17:01	0.9833	-0.3333	4.6043	0.6	3.3768	17.86	0.1722	1.6888
4/19/2019 17:02	0.9333	-0.05	4.4932	0.6667	3.369	17.4083	0.1806	1.6643
4/19/2019 17:03	0.9083	0.3833	4.7174	0.5333	3.3585	17.5546	0.1732	1.6851
4/19/2019 17:04	0.9	-0.2	4.6993	0.4167	3.3449	18.1333	0.1812	1.718
4/19/2019 17:05	0.8083	-0.1583	4.641	-0.65	3.347	18.2364	0.1652	1.7196
4/19/2019 17:06	0.8	0	5.0075	-0.1	3.35	18.1727	0.1667	1.6766
4/19/2019 17:07	0.8	-0.1917	5.0807	-0.4	3.4523	18.7917	0.1791	1.6768
4/19/2019 17:08	0.8	-0.1583	4.892	0.1833	3.4815	19.425	0.1708	1.6648
4/19/2019 17:09	0.8	0.1083	4.9955	-0.0667	3.5079	19.4909	0.1764	1.6758
4/19/2019 17:10	0.8417	-0.2083	4.7488	0.0667	3.4828	20.2636	0.1694	1.6823
4/19/2019 17:11	0.8667	-0.3833	5.0423	-0.55	3.4846	21	0.1672	1.6694
4/19/2019 17:12	0.9	-0.1083	4.8158	-0.0833	3.4828	21.7727	0.1831	1.6553
4/19/2019 17:13	0.9	0.3333	4.7722	-0.1667	3.4608	22.3273	0.1651	1.6419
4/19/2019 17:14	0.9	-0.1833	4.8195	-0.05	3.4741	23.2818	0.177	1.6675
4/19/2019 17:15	0.9	0.6583	4.6987	-0.1	3.4535	23.56	0.1798	1.6818
4/19/2019 17:16	0.9	0.15	4.9307	-0.2667	3.4758	23.1	0.1726	1.692
4/19/2019 17:17	0.9	0.5167	4.8955	0.0833	3.4465	23.27	0.1736	1.7084
4/19/2019 17:18	0.9	-0.6833	5.0153	0.3	3.447	23.8	0.1563	1.6876
4/19/2019 17:19	0.9	-0.6167	4.8582	-0.3667	3.4298	23.9546	0.1723	1.6673
4/19/2019 17:20	0.8833	0.3583	5.1465	-0.5833	3.4411	24.4182	0.173	1.6787
4/19/2019 17:21	0.9	0.225	4.8788	-0.1333	3.4582	25.2333	0.1618	1.6564
4/19/2019 17:22	0.9167	-0.325	4.7927	-0.6333	3.4395	25.6182	0.1828	1.6888
4/19/2019 17:23	1	0.1667	4.8827	-0.4333	3.4581	25.7455	0.1634	1.6798
4/19/2019 17:24	1	0.2333	4.7977	-0.3333	3.4367	26.0273	0.1778	1.705
4/19/2019 17:25	1	-0.2	4.7913	-0.2833	3.4361	26.0455	0.1689	1.6948
4/19/2019 17:26	1	-0.3083	4.8007	0.3333	3.4309	25.8667	0.1665	1.7038
4/19/2019 17:27	1	-0.1	4.858	-0.05	3.4418	25.8091	0.1836	1.7148
4/19/2019 17:28	0.9333	-0.0083	4.858	-0.0167	3.4483	26.3727	0.177	1.6926
4/19/2019 17:29	0.8833	-0.0417	5.065	-0.9167	3.4452	26.6818	0.1828	1.7217
4/19/2019 17:30	0.8083	-0.1583	4.7912	-0.1	3.4585	26.54	0.1782	1.7068
4/19/2019 17:31	0.8	0.075	4.9297	0.0833	3.4288	26.7667	0.1714	1.7086

4/19/2019 17:32	0.8	-0.475	4.7083	-0.85	3.4355	26.9333	0.1748	1.7409
4/19/2019 17:33	0.8	-0.3167	4.7665	-0.5667	3.42	27.0909	0.168	1.7227
4/19/2019 17:34	0.7917	-0.2333	5.0343	0.1	3.4213	27.2364	0.1768	1.6833
4/19/2019 17:35	0.8	-0.6333	5.1002	-0.25	3.4301	27.4273	0.1727	1.692
4/19/2019 17:36	0.8	0.1083	4.6938	0.0833	3.4168	27.2917	0.1666	1.7168
4/19/2019 17:37	0.8083	-0.6417	4.9978	-0.0667	3.4149	27.2091	0.1799	1.7112
4/19/2019 17:38	0.8167	0.0583	4.9523	0.1	3.4161	27.2636	0.1655	1.6969
4/19/2019 17:39	0.8	0.0417	4.7315	-0.6	3.4018	27.4182	0.1732	1.6971
4/19/2019 17:40	0.8	-0.4	4.8895	-0.3333	3.4016	27.475	0.1715	1.6892
4/19/2019 17:41	0.8	0.2333	5.0948	-0.4333	3.3949	27.4091	0.1642	1.6996
4/19/2019 17:42	0.8	0.2667	4.827	-0.2	3.4394	26.9333	0.1787	1.6835
4/19/2019 17:43	0.8	0.425	4.7912	-0.05	3.4168	26.68	0.1676	1.7082
4/19/2019 17:44	0.8	-0.05	4.7823	-0.25	3.4152	26.4083	0.1733	1.7285
4/19/2019 17:45	0.8	-0.5	4.8658	-0.3667	3.3813	25.7546	0.1718	1.7348
4/19/2019 17:46	0.8	-0.2833	4.7622	-0.0667	3.3955	25.025	0.171	1.6934
4/19/2019 17:47	0.8	-0.0167	4.8463	-0.4667	3.3828	24.3182	0.1796	1.6807
4/19/2019 17:48	0.8	0.125	4.927	-0.3333	3.3858	24.1417	0.1695	1.7084
4/19/2019 17:49	0.8833	-0.575	4.9207	-0.1	3.3826	24.2909	0.1701	1.7098
4/19/2019 17:50	0.9	0.5333	4.955	0.3167	3.3785	24.3364	0.173	1.7013
4/19/2019 17:51	0.9667	-0.0667	4.9195	-0.25	3.3817	24.3833	0.1732	1.7124
4/19/2019 17:52	0.9667	-0.65	4.8247	-0.5	3.3553	24.3	0.1746	1.7168
4/19/2019 17:53	0.9583	-0.175	4.6537	-0.5833	3.3766	24.4182	0.1719	1.7023
4/19/2019 17:54	0.9	0.2583	5.0028	-0.3	3.35	25.1	0.1765	1.7098
4/19/2019 17:55	0.9	-0.25	4.9865	0.0667	3.3697	25.4455	0.1624	1.7042
4/19/2019 17:56	0.9	0.2333	5.0285	0.1167	3.3807	26.1091	0.1731	1.7043
4/19/2019 17:57	0.95	0.45	5.2808	0.1667	3.3757	26.65	0.1833	1.7128
4/19/2019 17:58	1	0.5	4.9818	0.0833	3.3898	27.4556	0.1642	1.7227
4/19/2019 17:59	1	-0.2	4.8998	-0.6833	3.3594	28.1583	0.1797	1.7184
4/19/2019 18:00	1	-0.15	4.8458	-0.1333	3.37	28.6909	0.17	1.7006
4/19/2019 18:01	1	0.375	4.9803	0.0833	3.3624	27.85	0.1767	1.6891
4/19/2019 18:02	1	0.0333	4.775	0.5667	3.3534	26.65	0.1765	1.6948
4/19/2019 18:03	1	0.6167	4.7067	0.5333	3.3664	25.575	0.1684	1.7268
4/19/2019 18:04	1.0083	-0.0417	4.6402	0.1	3.3513	24.05	0.1855	1.7306
4/19/2019 18:05	1.0083	0.375	4.7373	-0.4	3.3607	22.3273	0.1711	1.7056
4/19/2019 18:06	1	0.025	4.9312	-0.3667	3.361	20.7546	0.173	1.7009
4/19/2019 18:07	1	-0.2833	5.1237	0.2167	3.3608	19.075	0.1798	1.7163
4/19/2019 18:08	1	-0.0333	4.854	-0.3333	3.423	17.2818	0.1699	1.7199
4/19/2019 18:09	0.9833	0.1167	5.0175	-0.05	3.4522	15.55	0.1755	1.7343
4/19/2019 18:10	0.9	0.4667	5.145	-0.35	3.4578	13.9	0.175	1.7338
4/19/2019 18:11	0.9	0.1417	4.7338	-0.45	3.4507	12.6364	0.1799	1.7148
4/19/2019 18:12	0.8417	-0.4167	4.7928	-0.6167	3.459	11.0583	0.1732	1.7151
4/19/2019 18:13	0.8	-0.4083	4.7598	0.15	3.4492	10.2546	0.1635	1.7021
4/19/2019 18:14	0.8	0.125	4.9032	0.3833	3.4628	10.2546	0.1794	1.7019
4/19/2019 18:15	0.8	-0.0417	4.9157	-0.5333	3.4489	10.3	0.1677	1.7126
4/19/2019 18:16	0.7583	-0.1917	4.7575	0.0667	3.4582	10.9333	0.1779	1.7148
4/19/2019 18:17	0.7	-0.0083	4.9862	-0.15	3.447	11.9182	0.1749	1.7244
4/19/2019 18:18	0.7	0.025	4.835	-0.2	3.4393	13.1182	0.1653	1.7333

4/19/2019 18:19	0.7	0.3083	4.7633	0.1333	3.4557	13.8417	0.1801	1.7137
4/19/2019 18:20	0.7	0.225	4.8125	-0.3667	3.4273	15.275	0.1721	1.7161
4/19/2019 18:21	0.65	0.1083	4.8793	0.1667	3.4513	16.9	0.1817	1.7153
4/19/2019 18:22	0.6667	-0.3	5.255	0.2333	3.4475	18.6	0.1749	1.6994
4/19/2019 18:23	0.6	0.125	4.8012	-0.25	3.4443	20.0546	0.1701	1.705
4/19/2019 18:24	0.6	0.125	4.7318	-0.6	3.4485	21.7364	0.1851	1.7004
4/19/2019 18:25	0.6	-0.025	4.635	-0.5333	3.4272	23.2091	0.1648	1.7058
4/19/2019 18:26	0.675	0.6667	4.7558	0.1167	3.4386	24.0364	0.178	1.7127
4/19/2019 18:27	0.7	0.5	4.671	0.05	3.4132	24.7917	0.1785	1.7282
4/19/2019 18:28	0.7	0.0667	4.6645	0.05	3.4139	25.4364	0.1708	1.7502
4/19/2019 18:29	0.7	-0.1833	4.6892	-0.55	3.4113	26.3	0.1788	1.7126
4/19/2019 18:30	0.7917	0.1917	4.8352	-0.45	3.4052	26.725	0.1648	1.6885
4/19/2019 18:31	0.8167	0.0167	4.5792	-0.0167	3.4106	27.65	0.1788	1.6875
4/19/2019 18:32	0.9	-0.1417	5.0502	-0.3167	3.4103	28.175	0.1745	1.7104
4/19/2019 18:33	0.8083	0.275	4.7913	-0.5667	3.403	28.5636	0.1754	1.7378
4/19/2019 18:34	0.8	-0.0833	4.7584	-0.3833	3.3799	28.6	0.1797	1.6958
4/19/2019 18:35	0.8	0.2333	5.1547	-0.55	3.3788	28.5182	0.1659	1.6907
4/19/2019 18:36	0.8	0.25	5.0932	-0.3833	3.3747	28.35	0.1786	1.7232
4/19/2019 18:37	0.8	0.0417	4.7896	-0.35	3.4036	28.4	0.1766	1.7185
4/19/2019 18:38	0.7667	-0.5417	4.8015	-1.05	3.3771	28.7333	0.1706	1.7293
4/19/2019 18:39	0.75	0.0333	4.8426	0.0167	3.3869	29.3667	0.1748	1.7238
4/19/2019 18:40	0.7833	-0.5083	4.6495	-0.15	3.3875	29.7455	0.1649	1.7413
4/19/2019 18:41	0.75	-0.4417	4.8835	-0.5667	3.3483	29.8727	0.1808	1.734
4/19/2019 18:42	0.8	-0.625	5.1207	-0.25	3.3605	29.9	0.1717	1.7318
4/19/2019 18:43	0.8	0.0833	4.8435	0.5667	3.3696	29.9417	0.1729	1.7377
4/19/2019 18:44	0.775	-0.2167	4.6341	-0.35	3.3566	30	0.1795	1.7168
4/19/2019 18:45	0.7	-0.125	4.6544	0.0333	3.3443	30.1	0.1668	1.7183
4/19/2019 18:46	0.6917	0.2917	4.5054	-0.3833	3.3266	30.1	0.175	1.718
4/19/2019 18:47	0.6083	-0.0083	4.6812	0.0833	3.3547	30.1182	0.1695	1.7208
4/19/2019 18:48	0.675	-0.2917	4.5422	-0.0333	3.3482	30.3727	0.1702	1.7368
4/19/2019 18:49	0.7	0.3083	4.8416	-0.2833	3.3303	30.4546	0.1738	1.7404
4/19/2019 18:50	0.6917	-0.4083	4.852	-0.35	3.3424	30.5667	0.1614	1.7494
4/19/2019 18:51	0.6333	0.3333	4.762	0.1167	3.3434	30.6	0.184	1.7233
4/19/2019 18:52	0.6	0.0583	4.7083	-0.25	3.3367	30.6	0.1695	1.7101
4/19/2019 18:53	0.6	0.3083	4.9948	-0.8	3.3208	30.6	0.1764	1.7423
4/19/2019 18:54	0.6583	0.45	4.7253	0.5	3.3108	30.6364	0.1738	1.7619
4/19/2019 18:55	0.7	0.3167	4.9465	-0.4833	3.3018	30.7	0.1672	1.779
4/19/2019 18:56	0.7	0.15	4.6632	-0.35	3.3129	30.7	0.1778	1.7554
4/19/2019 18:57	0.7	-0.2417	5.0272	-0.0667	3.2947	30.6636	0.1616	1.7516
4/19/2019 18:58	0.7	-0.3583	4.6708	-0.7667	3.2811	30.6909	0.183	1.7713
4/19/2019 18:59	0.7	0.0583	4.8272	-0.4667	3.2943	30.8	0.1614	1.7385
4/19/2019 19:00	0.7	0.5	4.7973	-0.0333	3.2763	30.8	0.1674	1.731
4/19/2019 19:01	0.7167	-0.075	4.5178	-0.2833	3.2651	30.8	0.1745	1.7529
4/19/2019 19:02	0.8	-0.1833	4.7828	0.0667	3.2639	30.8	0.1641	1.7693
4/19/2019 19:03	0.8364	0.4	4.7998	-0.15	3.2647	30.8	0.1752	1.7478
4/19/2019 19:04	0.8333	0.3167	4.7008	-0.4333	3.2514	30.8	0.1626	1.7609
4/19/2019 19:05	0.8	-0.0333	4.6878	-0.2	3.2568	30.8	0.1683	1.7767

4/19/2019 19:06	0.8	0.1917	5.0719	-0.1	3.2498	30.8	0.1776	1.7603
4/19/2019 19:07	0.8	0.0333	5.0033	-0.3667	3.256	30.8	0.1572	1.7403
4/19/2019 19:08	0.8	-0.6333	4.536	-0.8	3.256	30.8	0.1883	1.746
4/19/2019 19:09	0.8	0.6	4.8106	-0.0833	3.2058	30.8	0.1665	1.7368
4/19/2019 19:10	0.8	0.475	4.7371	0.0333	3.1812	30.8	0.1692	1.7315
4/19/2019 19:11	0.8	-0.0417	4.9255	-0.2	3.1603	30.7333	0.1774	1.7256
4/19/2019 19:12	0.7833	-0.1333	4.3692	-0.4167	3.1718	30.6667	0.165	1.7422
4/19/2019 19:13	0.7917	-0.2083	4.7842	-1	3.167	30.6083	0.1758	1.745
4/19/2019 19:14	0.7	0.2583	4.7871	0.0333	3.1515	30.6	0.1587	1.7678
4/19/2019 19:15	0.7	-0.3167	4.7156	-0.1333	3.1427	30.6545	0.1747	1.7453
4/19/2019 19:16	0.7	-0.6583	4.8	-0.4667	3.1405	30.7364	0.1796	1.7603
4/19/2019 19:17	0.7	-0.5083	4.835	-0.3667	3.1593	30.8	0.1601	1.7359
4/19/2019 19:18	0.6167	-0.1583	4.6686	0.05	3.1315	30.8	0.1869	1.7477
4/19/2019 19:19	0.5417	0.1583	4.7659	-0.3667	3.1297	30.8	0.1563	1.737
4/19/2019 19:20	0.5083	-0.5417	4.7375	-0.3333	3.1475	30.8917	0.1709	1.7576
4/19/2019 19:21	0.6	-0.95	4.7243	-0.0667	3.1256	30.9727	0.1717	1.7666
4/19/2019 19:22	0.6	-0.3417	4.8176	-0.95	3.1293	31.03	0.1595	1.7424
4/19/2019 19:23	0.6	-0.0667	4.818	-0.5333	3.1318	31.075	0.1716	1.7278
4/19/2019 19:24	0.6	0.1583	4.5239	-0.75	3.147	31.1	0.1624	1.7315
4/19/2019 19:25	0.5167	-0.05	4.7986	-0.1333	3.142	31.0083	0.165	1.7341
4/19/2019 19:26	0.5	-0.4333	4.6424	-0.3833	3.1484	31.1	0.1666	1.762
4/19/2019 19:27	0.6	-0.0417	4.7069	-0.9167	3.1476	31.0083	0.1586	1.7557
4/19/2019 19:28	0.6167	0.4167	4.7864	0.05	3.1308	31	0.1796	1.7473
4/19/2019 19:29	0.625	0.3667	4.8066	0.0833	3.1321	31	0.1538	1.7444
4/19/2019 19:30	0.6	-0.25	4.9696	0.05	3.1218	31	0.1741	1.7539
4/19/2019 19:31	0.5917	-0.0333	4.9573	-0.3333	3.129	31	0.1641	1.7649
4/19/2019 19:32	0.5917	-0.5167	4.8033	0.0667	3.1415	30.99	0.1612	1.7688
4/19/2019 19:33	0.5909	-0.15	4.7803	-0.25	3.1118	30.9	0.1753	1.7538
4/19/2019 19:34	0.5083	-0.1083	4.7513	-0.4667	3.1305	30.9	0.1591	1.7406
4/19/2019 19:35	0.5	-0.2083	4.7062	-0.6167	3.1078	30.85	0.1744	1.7143
4/19/2019 19:36	0.5667	-0.05	4.4004	-0.5	3.1271	30.71	0.1735	1.7613
4/19/2019 19:37	0.6	0.2583	4.614	-0.5667	3.1272	30.6417	0.1622	1.7604
4/19/2019 19:38	0.6917	0.1417	4.7137	0.1167	3.1198	30.6818	0.1709	1.7507
4/19/2019 19:39	0.7	-0.6333	4.6671	-0.1	3.1152	30.7	0.1528	1.764
4/19/2019 19:40	0.7	-0.5	4.7885	-0.6	3.1287	30.7	0.1754	1.7655
4/19/2019 19:41	0.7	0.0417	4.8768	-0.4	3.1223	30.7	0.1666	1.7481
4/19/2019 19:42	0.7	-0.1917	4.8908	-0.6833	3.1089	30.6636	0.1683	1.744
4/19/2019 19:43	0.7	-0.5833	4.6791	-0.25	3.1176	30.6182	0.1843	1.7521
4/19/2019 19:44	0.7583	-0.525	4.5025	-0.45	3.1056	30.6	0.1586	1.7441
4/19/2019 19:45	0.7583	-0.4417	4.7603	-0.8833	3.1241	30.6	0.175	1.7433
4/19/2019 19:46	0.8	-0.1	4.7838	-0.7833	3.1222	30.6	0.1639	1.7543
4/19/2019 19:47	0.8	-0.1083	4.8719	0.1333	3.0825	30.6182	0.1722	1.7683
4/19/2019 19:48	0.85	-0.2	4.9958	-0.4	3.0991	30.725	0.1773	1.7653
4/19/2019 19:49	0.9	-0.7417	4.6119	-0.7167	3.0981	31	0.1638	1.7503
4/19/2019 19:50	0.875	-0.4333	4.636	0.15	3.0929	31	0.1794	1.7671
4/19/2019 19:51	0.8083	-1.025	4.9594	-0.1833	3.0974	31	0.1691	1.7513
4/19/2019 19:52	0.8	-0.4417	4.8931	-0.5333	3.1146	31.0091	0.1703	1.7306

4/19/2019 19:53	0.875	0.575	4.7874	-0.35	3.0978	31.1	0.1768	1.7505
4/19/2019 19:54	0.9	0.35	4.8885	-0.0167	3.1015	31.1727	0.1549	1.7621
4/19/2019 19:55	0.85	-0.3083	4.7193	-0.4333	3.0955	31.2	0.1775	1.7577
4/19/2019 19:56	0.8	-0.2583	4.6478	-0.3333	3.0893	31.2	0.1615	1.7445
4/19/2019 19:57	0.8	-0.275	4.8033	-0.5667	3.0922	31.2	0.1799	1.7334
4/19/2019 19:58	0.8	-0.5833	4.7378	-0.4	3.0835	31.25	0.1792	1.7473
4/19/2019 19:59	0.8167	0.4417	4.8494	-0.5667	3.0936	31.1583	0.1645	1.7487
4/19/2019 20:00	0.8	-0.3083	4.678	-0.3	3.0823	31.1	0.1744	1.7833
4/19/2019 20:01	0.8	-0.4083	4.6294	-0.6	3.0913	31.1364	0.1664	1.7854
4/19/2019 20:02	0.775	-0.1417	4.7487	-0.35	3.0923	31.0818	0.1761	1.7461
4/19/2019 20:03	0.7909	-0.3833	4.759	-0.4333	3.0825	31.0909	0.165	1.7501
4/19/2019 20:04	0.7667	-0.2167	4.5485	-0.8333	3.0996	31.1	0.1605	1.7448
4/19/2019 20:05	0.75	-0.1833	4.6332	-0.1333	3.0791	31.1	0.1724	1.7489
4/19/2019 20:06	0.7	-0.0333	4.5775	-0.5833	3.0875	31.0833	0.1612	1.7355
4/19/2019 20:07	0.7	-0.4667	4.7475	-0.4333	3.0954	31	0.1778	1.7324
4/19/2019 20:08	0.7	0.0167	4.5127	-0.95	3.0786	31.0091	0.1641	1.7718
4/19/2019 20:09	0.7	-0.625	4.6988	-0.0667	3.079	31.1	0.1621	1.7546
4/19/2019 20:10	0.7	-0.3583	4.849	-0.9	3.0417	31.1667	0.1774	1.7353
4/19/2019 20:11	0.6333	-0.575	4.8503	-1.05	2.9976	31.175	0.1668	1.7363
4/19/2019 20:12	0.6	0.2333	4.5092	-0.5	2.9769	31.2818	0.1714	1.7877
4/19/2019 20:13	0.625	-0.4917	4.9533	-0.8667	2.985	31.25	0.1629	1.7673
4/19/2019 20:14	0.6333	-0.5583	4.9165	-0.8667	2.9933	31.3	0.1686	1.7463
4/19/2019 20:15	0.675	-0.1833	4.6915	-0.4667	2.9904	31.3	0.1744	1.7432
4/19/2019 20:16	0.7	0.4917	4.8075	-0.35	2.9796	31.3	0.1674	1.7403
4/19/2019 20:17	0.7	-0.5583	4.9785	-0.4333	2.9908	31.3	0.1763	1.7532
4/19/2019 20:18	0.7	-0.2333	4.9078	0	2.9939	31.3	0.1707	1.7303
4/19/2019 20:19	0.7	-0.3833	4.7307	-0.8833	2.9837	31.3636	0.1694	1.7314
4/19/2019 20:20	0.6333	-0.225	4.8308	-0.15	2.9989	31.3833	0.1684	1.7493
4/19/2019 20:21	0.6	0.025	5.4417	-0.3167	2.9941	31.3	0.1619	1.7431
4/19/2019 20:22	0.6	0.2083	3.759	-0.15	3.01	31.3917	0.1761	1.7293
4/19/2019 20:23	0.6	-0.4	4.9308	-0.2667	3.0032	31.3833	0.1708	1.7246
4/19/2019 20:24	0.6	-0.5417	4.8978	0.4667	2.9953	31.4	0.1675	1.7551
4/19/2019 20:25	0.55	0.1333	4.7283	-0.3833	2.9863	31.4	0.1752	1.7555
4/19/2019 20:26	0.5167	-0.15	4.6938	-0.7167	2.9906	31.4	0.164	1.7672
4/19/2019 20:27	0.5417	0.3167	3.3807	-0.1833	2.9805	31.4	0.1771	1.7794
4/19/2019 20:28	0.5333	-0.6	5.031	-0.2833	2.9729	31.375	0.1691	1.7897
4/19/2019 20:29	0.5083	0.3667	4.8127	-0.5833	2.9786	31.3	0.1696	1.7463
4/19/2019 20:30	0.5	0.5917	5.0722	-0.2667	2.9918	31.3	0.1738	1.7594
4/19/2019 20:31	0.5	-0.3833	4.4922	-0.5333	2.9818	31.3	0.1644	1.732
4/19/2019 20:32	0.5	-0.225	4.7002	-0.7	2.9965	31.2	0.168	1.7414
4/19/2019 20:33	0.4667	0.25	4.8868	-0.25	2.9942	31.2	0.1575	1.7558
4/19/2019 20:34	0.4167	0.3667	4.9157	0.3333	2.9899	31.2	0.1671	1.7308
4/19/2019 20:35	0.4	-0.0833	4.73	-0.7	2.991	31.2	0.1633	1.7458
4/19/2019 20:36	0.45	0.1833	4.8033	-0.8333	2.9876	31.175	0.1565	1.7565
4/19/2019 20:37	0.4417	-0.175	4.6088	0.1333	2.9958	31.1727	0.174	1.7338
4/19/2019 20:38	0.5	0.8417	4.9213	-0.3667	3.0013	31.1417	0.1619	1.747
4/19/2019 20:39	0.5	0.4417	4.7513	-0.05	2.9903	31.1273	0.1695	1.7434

4/19/2019 20:40	0.5	-0.0083	4.6463	-0.2167	3.0156	31.0546	0.1662	1.756
4/19/2019 20:41	0.5	-0.4583	4.668	-0.4	3.025	30.9636	0.166	1.7328
4/19/2019 20:42	0.5	0.0833	4.818	0.3667	2.9919	30.9333	0.1706	1.7249
4/19/2019 20:43	0.5	-0.0417	4.5732	-0.65	3.0039	30.8818	0.1604	1.742
4/19/2019 20:44	0.55	-0.675	4.6285	-0.35	3.0128	30.7273	0.1722	1.7333
4/19/2019 20:45	0.6	0.3833	4.8113	-0.2167	3.0121	30.7818	0.1702	1.7453
4/19/2019 20:46	0.6	-0.15	4.712	-0.8333	3.0029	30.8	0.1639	1.7433
4/19/2019 20:47	0.6	-0.2917	4.6925	-0.7333	3.0317	30.72	0.1706	1.7563
4/19/2019 20:48	0.6	-0.3417	5.0007	-0.5	3.0252	30.7364	0.1527	1.7372
4/19/2019 20:49	0.6333	-0.0083	4.4872	-0.1833	3.0071	30.6182	0.1723	1.7085
4/19/2019 20:50	0.7	0.2583	4.6482	-0.2833	3.0248	30.675	0.1644	1.7356
4/19/2019 20:51	0.7	0.2333	4.6817	-0.1833	3.0367	30.5583	0.1665	1.7303
4/19/2019 20:52	0.7	-0.025	4.7648	0.45	3.0339	30.6273	0.1717	1.7124
4/19/2019 20:53	0.7	-0.3667	4.6672	-0.1833	3.0022	30.7	0.1575	1.7304
4/19/2019 20:54	0.7	-0.3583	4.5587	-0.1333	2.9911	30.7546	0.1676	1.7308
4/19/2019 20:55	0.7	0.0917	4.5437	-0.6333	3.0193	30.8455	0.1523	1.731
4/19/2019 20:56	0.7	-0.0083	4.6512	-0.1167	2.9996	31	0.168	1.7524
4/19/2019 20:57	0.7083	-0.2	4.6488	-0.5833	3.0233	31	0.1651	1.7343
4/19/2019 20:58	0.8	0.6667	4.6868	-0.0667	3.0231	30.9	0.1615	1.7537
4/19/2019 20:59	0.8	-0.025	4.7852	-0.4	3.0296	30.9	0.166	1.7393
4/19/2019 21:00	0.8	0.1	4.741	-0.9167	3.0215	30.9091	0.1542	1.7348
4/19/2019 21:01	0.8	-0.0417	4.583	-0.4667	3.0076	31.08	0.1697	1.7193
4/19/2019 21:02	0.8	0.0583	4.6773	-0.7167	3.0083	31.0364	0.1625	1.7192
4/19/2019 21:03	0.8	0.0083	4.6298	-0.2667	3.0004	31.1455	0.1665	1.7155
4/19/2019 21:04	0.8	-0.0833	4.6588	-0.5667	3.0303	31.2	0.1612	1.7447
4/19/2019 21:05	0.8	0.175	4.545	-0.3	3.01	31.2	0.1528	1.7428
4/19/2019 21:06	0.8083	-0.2917	4.623	-0.2667	3.0166	31.2	0.1788	1.7265
4/19/2019 21:07	0.8167	-0.625	4.5435	-0.9333	3.0093	31.2	0.1491	1.7288
4/19/2019 21:08	0.8417	-0.3	4.605	-0.75	2.9967	31.2	0.1618	1.7237
4/19/2019 21:09	0.8583	0.3167	4.3525	-0.5333	2.9947	31.2	0.1687	1.7317
4/19/2019 21:10	0.8	-0.6333	4.7247	-0.5167	2.995	31.2917	0.1617	1.7201
4/19/2019 21:11	0.8	-0.1167	4.4457	-0.55	2.9893	31.2	0.1718	1.713
4/19/2019 21:12	0.8	-0.325	4.564	-0.7167	2.949	31.2	0.1504	1.7195
4/19/2019 21:13	0.8	-0.6417	4.6938	-0.0167	2.9517	31.2	0.1699	1.7314
4/19/2019 21:14	0.8	0.325	4.5908	-0.5167	2.9613	31.2	0.1498	1.745
4/19/2019 21:15	0.7667	-0.7667	4.3993	-0.2667	2.9599	31.2	0.1659	1.7246
4/19/2019 21:16	0.7	-0.025	4.72	-0.55	2.9387	31.2	0.1735	1.7113
4/19/2019 21:17	0.7	-0.2167	4.833	-0.1167	2.9439	31.2	0.1465	1.7393
4/19/2019 21:18	0.7	0.1	4.8573	-0.4833	2.9383	31.2833	0.1675	1.7138
4/19/2019 21:19	0.7	-0.125	4.4702	-0.3333	2.9343	31.2583	0.1485	1.7157
4/19/2019 21:20	0.6917	-0.05	4.8563	-0.4667	2.9304	31.2	0.1674	1.7123
4/19/2019 21:21	0.7	-0.1417	4.5085	-0.2833	2.9317	31.2	0.1583	1.7284
4/19/2019 21:22	0.6917	-0.425	4.5283	-0.0333	2.9301	31.2167	0.1583	1.7431
4/19/2019 21:23	0.6083	-0.3	4.6378	-0.1833	2.9268	31.325	0.1735	1.7291
4/19/2019 21:24	0.6	0.0167	4.6677	-0.15	2.9263	31.4	0.1535	1.7271
4/19/2019 21:25	0.6	-0.3667	4.7755	-0.6333	2.9245	31.3455	0.1699	1.7374
4/19/2019 21:26	0.6	-0.5083	4.62	-0.75	2.9204	31.39	0.1596	1.7259

4/19/2019 21:27	0.625	-0.475	4.6407	-0.7333	2.9195	31.4	0.1651	1.7318
4/19/2019 21:28	0.65	0.5833	4.7787	-0.4833	2.9053	31.4	0.1619	1.7401
4/19/2019 21:29	0.5583	-0.925	4.9018	-0.35	2.9018	31.3833	0.1497	1.7071
4/19/2019 21:30	0.475	0.0167	4.7338	-0.65	2.9026	31.275	0.1726	1.7166
4/19/2019 21:31	0.4	-0.25	4.6505	-0.8	2.9078	31.2091	0.1578	1.7348
4/19/2019 21:32	0.4	-0.2	4.7808	0.25	2.9002	31.3	0.1597	1.7595
4/19/2019 21:33	0.4	-0.275	4.8415	0.05	2.8794	31.2455	0.1618	1.7461
4/19/2019 21:34	0.4	-0.3083	4.6356	0.1833	2.8755	31.1583	0.1538	1.7291
4/19/2019 21:35	0.4	0.325	4.7391	0.3167	2.8864	31.1	0.1699	1.7494
4/19/2019 21:36	0.4	-0.4833	4.4278	-0.4333	2.8745	31.1	0.1533	1.7571
4/19/2019 21:37	0.4083	-0.15	4.4133	0.1	2.8902	31.1	0.1629	1.7561
4/19/2019 21:38	0.5	0.1583	4.3693	-0.2167	2.8732	31.0667	0.1671	1.7523
4/19/2019 21:39	0.5	0.275	4.4738	-0.4167	2.8733	31.1	0.1612	1.7368
4/19/2019 21:40	0.5	-0.3583	4.6076	0.0167	2.8631	31.1333	0.1615	1.7334
4/19/2019 21:41	0.5	0.225	4.8338	0.2333	2.8713	31.2182	0.1464	1.7477
4/19/2019 21:42	0.5333	0.15	4.6494	-0.65	2.8628	31.325	0.1612	1.721
4/19/2019 21:43	0.5917	0.1583	4.7271	-0.1	2.8452	31.4	0.1584	1.7332
4/19/2019 21:44	0.5	-0.25	4.5058	-0.1333	2.8595	31.4	0.1629	1.7518
4/19/2019 21:45	0.5	0.2083	4.5697	-0.25	2.8418	31.4	0.1634	1.75
4/19/2019 21:46	0.4917	-0.275	4.5179	-0.5	2.8483	31.4	0.1606	1.7147
4/19/2019 21:47	0.575	-0.625	4.5438	0.0833	2.8549	31.4	0.1626	1.7328
4/19/2019 21:48	0.6083	-0.2583	4.6106	-0.35	2.8386	31.375	0.1518	1.7363
4/19/2019 21:49	0.7	-0.125	4.468	-0.85	2.8422	31.4	0.1618	1.7638
4/19/2019 21:50	0.6167	-0.3583	4.6058	-0.2333	2.8388	31.5	0.1598	1.7698
4/19/2019 21:51	0.6833	-0.5083	4.5957	0.1667	2.8494	31.5	0.1542	1.7367
4/19/2019 21:52	0.7	-0.7417	4.3696	-0.3833	2.8373	31.5	0.1673	1.7403
4/19/2019 21:53	0.7	-0.2083	4.5956	-0.6333	2.8348	31.4182	0.1505	1.7468
4/19/2019 21:54	0.7	-0.3333	4.5904	-0.45	2.8358	31.3667	0.1732	1.7559
4/19/2019 21:55	0.7	-0.475	4.7638	-0.15	2.836	31.3	0.1595	1.7388
4/19/2019 21:56	0.6583	0.1917	4.497	-0.9167	2.8192	31.3	0.1656	1.7486
4/19/2019 21:57	0.6	-0.3917	4.7317	0.6	2.8142	31.25	0.1647	1.7361
4/19/2019 21:58	0.6	0.05	4.3287	-0.3833	2.8128	31.2167	0.1545	1.7335
4/19/2019 21:59	0.5833	0.3333	4.6218	-0.2667	2.8109	31.3727	0.168	1.7244
4/19/2019 22:00	0.6	-0.1833	4.4522	-0.6667	2.8143	31.4	0.1522	1.7087
4/19/2019 22:01	0.6	-0.5083	4.517	-0.3167	2.817	31.4	0.1667	1.745
4/19/2019 22:02	0.6	-0.075	4.7345	-0.0667	2.8155	31.3	0.1529	1.7533
4/19/2019 22:03	0.6	-0.2917	4.9045	-0.2	2.8158	31.2	0.1565	1.7658
4/19/2019 22:04	0.6	-0.4083	4.5662	-0.4333	2.8379	31.1091	0.1606	1.7493
4/19/2019 22:05	0.6	0	4.7273	-0.35	2.8058	31.08	0.1519	1.7228
4/19/2019 22:06	0.6	0.55	4.6913	0.1667	2.7936	31.0583	0.1704	1.7255
4/19/2019 22:07	0.6	-0.85	4.4646	-0.5	2.7993	31.13	0.1569	1.7391
4/19/2019 22:08	0.6	-0.55	4.6764	-0.0667	2.7937	31.1583	0.1643	1.7113
4/19/2019 22:09	0.6	-0.0333	4.8538	-0.4167	2.795	31.2	0.1692	1.7039
4/19/2019 22:10	0.65	-0.4917	4.7635	0.2833	2.7989	31.2	0.1551	1.7302
4/19/2019 22:11	0.65	-0.375	4.4333	-0.1667	2.798	31.1083	0.169	1.7413
4/19/2019 22:12	0.7	-0.0417	4.5031	-0.0667	2.7816	30.7091	0.1511	1.7113
4/19/2019 22:13	0.7	-0.5083	4.8868	-0.3667	2.7355	30.3833	0.1692	1.7343

4/19/2019 22:14	0.6833	-0.225	4.7874	-0.0333	2.7501	29.8273	0.1551	1.7374
4/19/2019 22:15	0.6167	-0.8917	4.6603	-0.45	2.7353	28.5	0.1625	1.7446
4/19/2019 22:16	0.7	0.575	4.4966	-0.4	2.7335	27.0636	0.1655	1.7359
4/19/2019 22:17	0.7	-0.525	4.5802	-0.7667	2.7428	25.6333	0.1512	1.7295
4/19/2019 22:18	0.7	0.15	4.6737	0.0333	2.7558	24.6091	0.1662	1.72
4/19/2019 22:19	0.7	-0.0667	4.7302	0.0167	2.7634	23.9	0.1457	1.7397
4/19/2019 22:20	0.7	-0.625	4.6354	-0.3	2.7421	23.3	0.1615	1.7448
4/19/2019 22:21	0.7	-0.375	4.6976	-0.0833	2.7343	21.9583	0.1623	1.7544
4/19/2019 22:22	0.6667	0.275	4.7095	-0.25	2.7369	21.0091	0.1565	1.7625
4/19/2019 22:23	0.6167	-0.2167	4.9299	-0.4667	2.7581	20.5636	0.1576	1.7561
4/19/2019 22:24	0.65	0.0667	4.8338	-0.3833	2.7544	20.3818	0.1502	1.7499
4/19/2019 22:25	0.7	-0.4833	4.4819	-0.8167	2.7368	20.1364	0.1643	1.7448
4/19/2019 22:26	0.7	-0.2417	4.5148	-0.4167	2.7367	20.1833	0.1512	1.7298
4/19/2019 22:27	0.7	-0.1833	4.6282	0.1667	2.7274	20.9833	0.1596	1.7426
4/19/2019 22:28	0.7	0	4.5952	-0.6667	2.7464	22.5546	0.1617	1.7413
4/19/2019 22:29	0.65	0.4667	4.8033	-0.2667	2.7361	23.91	0.155	1.7361
4/19/2019 22:30	0.6083	-0.075	4.5683	-0.9	2.7468	25.0727	0.1641	1.7345
4/19/2019 22:31	0.7	-0.3583	4.7317	-0.45	2.7273	25.8182	0.141	1.7379
4/19/2019 22:32	0.725	-0.15	4.4919	-0.5167	2.7389	26.1636	0.1675	1.7368
4/19/2019 22:33	0.7	0.3667	4.8715	0.0167	2.7436	27.3455	0.1604	1.7526
4/19/2019 22:34	0.7	-0.525	4.6843	-0.0333	2.7328	28.5273	0.1561	1.771
4/19/2019 22:35	0.7	-0.3083	4.4517	-0.2833	2.7451	29.1	0.1719	1.78
4/19/2019 22:36	0.7	0.1667	4.6637	-0.4167	2.7325	29.37	0.1491	1.7614
4/19/2019 22:37	0.7	-0.2333	4.7342	-0.6833	2.7238	29.525	0.1653	1.7308
4/19/2019 22:38	0.6833	-0.65	4.7089	-0.7	2.7308	29.44	0.1525	1.7321
4/19/2019 22:39	0.6833	0.1583	4.7835	-0.4167	2.7423	29.2833	0.1628	1.7322
4/19/2019 22:40	0.7	-0.2417	4.512	-0.2	2.7402	29.3	0.1569	1.7518
4/19/2019 22:41	0.7	-0.3417	4.5638	-0.8167	2.7378	29.1333	0.1458	1.7511
4/19/2019 22:42	0.6583	-0.3417	4.6593	-0.1333	2.7409	28.6167	0.1662	1.756
4/19/2019 22:43	0.6333	-0.25	4.7149	-0.2	2.7329	28.1833	0.1522	1.7501
4/19/2019 22:44	0.7	-0.475	4.6474	-0.5	2.7486	27.5	0.1602	1.7704
4/19/2019 22:45	0.7333	-0.7583	4.6949	-0.2	2.7398	26.9667	0.1619	1.7603
4/19/2019 22:46	0.8	-0.5167	4.6888	-0.4833	2.7484	26.5182	0.155	1.7714
4/19/2019 22:47	0.8	-0.25	4.4558	-0.7333	2.7251	26.3636	0.1645	1.7585
4/19/2019 22:48	0.8	-0.2667	4.5872	0.1	2.7367	26.2	0.1409	1.7479
4/19/2019 22:49	0.8	0.0417	4.6746	-0.0333	2.7532	26.3833	0.1626	1.7597
4/19/2019 22:50	0.8	-0.0917	4.7179	0.0333	2.7621	26.2556	0.1583	1.7574
4/19/2019 22:51	0.8	-0.45	4.8492	-0.2	2.7275	26.3917	0.1571	1.7507
4/19/2019 22:52	0.8	0.6	4.7453	-0.3833	2.7153	26.2	0.1627	1.7478
4/19/2019 22:53	0.8	0.225	4.7307	-0.25	2.7229	26.2	0.1491	1.76
4/19/2019 22:54	0.7917	0.3667	4.7083	-0.2667	2.7293	26.6182	0.1602	1.7548
4/19/2019 22:55	0.7	0.2	4.7089	-0.45	2.7383	26.9	0.1542	1.7143
4/19/2019 22:56	0.7333	0.0333	4.6312	0.1167	2.7515	27.3583	0.1553	1.7173
4/19/2019 22:57	0.8	-0.1417	4.6263	-0.75	2.7291	27.9273	0.1512	1.7294
4/19/2019 22:58	0.8	-0.6333	4.483	-0.0833	2.7276	28.425	0.1618	1.7551
4/19/2019 22:59	0.775	-0.5333	4.8026	-0.4667	2.7156	28.5364	0.1633	1.766
4/19/2019 23:00	0.7	-0.2917	4.7693	-0.5	2.7317	28.5455	0.1595	1.7652

4/19/2019 23:01	0.675	-0.575	4.8136	-0.3167	2.7116	28.4083	0.1608	1.784
4/19/2019 23:02	0.6	-0.2583	4.7566	-0.4	2.7271	28.7	0.1577	1.7709
4/19/2019 23:03	0.6833	-0.6917	4.59	-0.7833	2.6981	29.2917	0.1574	1.7635
4/19/2019 23:04	0.65	-0.9417	4.8025	-0.4333	2.7234	29.42	0.168	1.7597
4/19/2019 23:05	0.6583	-0.0917	4.7407	-0.0167	2.7087	29.3	0.1477	1.778
4/19/2019 23:06	0.6	-0.775	4.3977	-0.6333	2.7033	29.4	0.1612	1.7686
4/19/2019 23:07	0.6	0.1417	4.7723	-0.6667	2.7093	29.4583	0.1486	1.7715
4/19/2019 23:08	0.65	-0.0167	4.7832	-0.3667	2.6962	29.52	0.1629	1.7648
4/19/2019 23:09	0.6167	-0.6	4.6923	-0.2167	2.7023	29.6	0.1651	1.7583
4/19/2019 23:10	0.6	-0.0417	4.6977	-0.0667	2.682	29.57	0.1495	1.7549
4/19/2019 23:11	0.6	-0.5917	4.8155	-0.3	2.6879	29.5333	0.1656	1.7417
4/19/2019 23:12	0.6	-0.075	4.7467	-0.1667	2.687	29.625	0.1473	1.731
4/19/2019 23:13	0.6	-0.3167	4.6255	-0.5167	2.6815	29.8083	0.1636	1.7396
4/19/2019 23:14	0.6	-0.3167	4.8425	-0.3667	2.6665	29.9	0.1555	1.7373
4/19/2019 23:15	0.6	-0.2417	4.7597	-0.65	2.6423	29.8727	0.1605	1.7433
4/19/2019 23:16	0.6	0.0667	4.6342	-0.15	2.6384	29.975	0.16	1.7222
4/19/2019 23:17	0.6	-1.1167	4.6705	-0.5667	2.6288	30.3	0.1489	1.7746
4/19/2019 23:18	0.6333	-0.325	4.7947	-0.2833	2.6449	30.3	0.1645	1.7641
4/19/2019 23:19	0.7	-0.1583	4.6958	-0.0333	2.6363	30.325	0.1461	1.7467
4/19/2019 23:20	0.6083	0.1	4.9817	-0.7	2.6352	30.4417	0.1421	1.7324
4/19/2019 23:21	0.6	-0.2917	4.9643	0.0667	2.6404	30.5273	0.1641	1.7449
4/19/2019 23:22	0.6	0.0833	4.8465	-0.3	2.6568	30.5917	0.1327	1.7603
4/19/2019 23:23	0.6	-0.7833	4.8322	-0.25	2.6578	30.5417	0.1556	1.742
4/19/2019 23:24	0.6	0.3083	4.7013	-0.3833	2.6354	30.65	0.1487	1.7313
4/19/2019 23:25	0.6	0.1083	4.829	0.1833	2.6336	30.6727	0.1511	1.7661
4/19/2019 23:26	0.6583	0.2083	4.8817	-0.45	2.6514	30.8	0.1464	1.7735
4/19/2019 23:27	0.625	-0.4333	4.6567	-0.4333	2.6255	30.8	0.1405	1.7534
4/19/2019 23:28	0.6	0.0333	4.7317	0.0667	2.6495	30.8091	0.1595	1.7541
4/19/2019 23:29	0.6	-0.2167	4.7375	-0.6667	2.6263	30.9	0.1484	1.7579
4/19/2019 23:30	0.6	0.0917	4.7507	0.3333	2.6333	30.9	0.1519	1.7374
4/19/2019 23:31	0.6	-0.2333	4.5145	-0.4333	2.6282	30.9	0.1588	1.7199
4/19/2019 23:32	0.6917	-0.1083	4.835	-0.1333	2.648	30.99	0.1431	1.7468
4/19/2019 23:33	0.7	-0.075	4.8333	-0.35	2.6365	31	0.164	1.7668
4/19/2019 23:34	0.7	-0.9583	4.727	-0.1333	2.6259	31.02	0.1349	1.7672
4/19/2019 23:35	0.7	-0.1	4.7807	-0.3667	2.6233	31.0583	0.1601	1.7648
4/19/2019 23:36	0.7	0.0083	4.8835	0.1167	2.6278	31.0818	0.1617	1.7727
4/19/2019 23:37	0.6417	-0.1	4.6098	-0.4167	2.6449	31.1667	0.149	1.776
4/19/2019 23:38	0.675	0.1833	4.6572	-0.25	2.6013	31.2273	0.1589	1.7807
4/19/2019 23:39	0.7	-0.3333	4.4382	-0.45	2.5913	31.35	0.143	1.7673
4/19/2019 23:40	0.7	-0.5	4.966	0.0167	2.6188	31.2546	0.139	1.7868
4/19/2019 23:41	0.625	0.3167	5.1808	-0.3833	2.6297	31.175	0.1557	1.7915
4/19/2019 23:42	0.6	0.1083	4.7828	-0.2833	2.6146	30.9182	0.1351	1.7583
4/19/2019 23:43	0.6	-0.65	4.8232	0.1667	2.6155	30.8818	0.1605	1.7524
4/19/2019 23:44	0.6182	-0.4917	4.4705	-0.0333	2.621	30.8	0.1509	1.7467
4/19/2019 23:45	0.6	-0.2	4.5828	-0.3333	2.6212	30.8727	0.1621	1.7706
4/19/2019 23:46	0.6	0.1	4.8432	-0.3667	2.6022	30.8364	0.1479	1.7678
4/19/2019 23:47	0.6583	-0.3333	4.8613	-0.45	2.6081	30.8	0.15	1.7842

4/19/2019 23:48	0.7	-0.1917	5.1363	0.1833	2.6043	30.8	0.159	1.7778
4/19/2019 23:49	0.7	0.0167	4.8217	-0.55	2.594	30.8	0.1283	1.7751
4/19/2019 23:50	0.6417	-0.3083	4.4785	-0.5167	2.5943	30.8	0.155	1.7797
4/19/2019 23:51	0.6	-0.3917	4.7963	-0.3	2.5988	30.73	0.1555	1.7711
4/19/2019 23:52	0.55	-0.2833	4.7752	-0.1333	2.5967	30.7273	0.1483	1.7463
4/19/2019 23:53	0.6	-0.625	4.8735	-0.9333	2.5861	30.7	0.1682	1.7643
4/19/2019 23:54	0.6	0.8333	4.8198	-1.1667	2.5835	30.8	0.1374	1.7521
4/19/2019 23:55	0.6	0.975	4.614	-0.3833	2.5909	30.9818	0.1532	1.7552
4/19/2019 23:56	0.6	-0.55	4.6958	-0.95	2.5864	31.0273	0.1502	1.7824
4/19/2019 23:57	0.6167	-0.1083	4.6538	-0.5	2.5878	30.85	0.1506	1.7224
4/19/2019 23:58	0.7	0.0833	4.623	-0.4667	2.5755	30.8	0.1606	1.728
4/19/2019 23:59	0.7417	-0.3083	5.3715	-0.3667	2.5741	30.8818	0.1442	1.712
4/20/2019 0:00	0.8	-0.5667	4.934	-0.5667	2.5748	30.9	0.1602	1.7153
4/20/2019 0:01	0.8	-0.875	4.8057	-0.4333	2.5662	30.9	0.1398	1.7439
4/20/2019 0:02	0.775	0.275	4.638	-0.45	2.5724	30.8182	0.1476	1.7565
4/20/2019 0:03	0.7083	-0.15	4.907	0.0667	2.568	30.8	0.153	1.7474
4/20/2019 0:04	0.7	-0.2667	4.811	-0.2833	2.5575	30.8	0.1392	1.7548
4/20/2019 0:05	0.7	-0.475	4.9973	-0.2167	2.5638	30.8636	0.1547	1.778
4/20/2019 0:06	0.7083	-0.2167	4.9962	-0.4833	2.5615	30.925	0.1512	1.7459
4/20/2019 0:07	0.7	-0.2583	4.817	-0.1167	2.5638	30.7546	0.1537	1.754
4/20/2019 0:08	0.7917	-0.0333	4.9417	-0.0667	2.5573	30.6	0.1598	1.7546
4/20/2019 0:09	0.8	0.3833	4.7918	-0.2833	2.5453	30.6818	0.1414	1.7358
4/20/2019 0:10	0.8	0.125	4.7503	-0.0833	2.5403	30.7	0.1598	1.7404
4/20/2019 0:11	0.8	-0.3667	4.8527	-0.1167	2.5447	30.7	0.1439	1.7389
4/20/2019 0:12	0.8	-0.5	4.771	0.2333	2.5421	30.6667	0.1587	1.7503
4/20/2019 0:13	0.8	-0.75	4.9197	-0.1333	2.547	30.6	0.1485	1.7331
4/20/2019 0:14	0.8	-0.3083	4.9152	-0.45	2.5468	30.6	0.1487	1.7602
4/20/2019 0:15	0.8	-0.225	5.0092	-0.3167	2.508	30.7	0.1479	1.7603
4/20/2019 0:16	0.7	-0.1667	4.8288	-0.1333	2.4701	30.7	0.1401	1.7553
4/20/2019 0:17	0.7833	-0.6	4.8538	-0.9167	2.4643	30.7917	0.152	1.7483
4/20/2019 0:18	0.8	-0.3	4.9397	-0.65	2.4773	30.8	0.1388	1.7571
4/20/2019 0:19	0.8	-1.35	4.7623	-0.3167	2.4765	30.8546	0.1578	1.7644
4/20/2019 0:20	0.8	-0.55	4.6937	-0.5167	2.4688	30.9917	0.1515	1.7523
4/20/2019 0:21	0.8	-0.0667	4.9242	-0.5833	2.4783	31	0.1477	1.7523
4/20/2019 0:22	0.8	-0.3833	4.7695	-0.75	2.4813	31.0455	0.1603	1.7173
4/20/2019 0:23	0.8	0.3833	4.8695	-0.8833	2.4845	31	0.1418	1.7224
4/20/2019 0:24	0.8	-0.125	4.9103	0	2.4666	31	0.1419	1.7653
4/20/2019 0:25	0.8	0.0167	4.8813	-0.1667	2.4871	31.1	0.1576	1.7587
4/20/2019 0:26	0.8	0.1	5.0583	-0.5333	2.4805	31.1	0.1359	1.753
4/20/2019 0:27	0.8	-0.0083	4.588	-0.8	2.4838	31.1727	0.1568	1.7549
4/20/2019 0:28	0.7833	-0.1917	4.6028	0.15	2.4638	31.125	0.1459	1.7536
4/20/2019 0:29	0.7333	-0.2333	4.4297	-0.3	2.4572	31.1273	0.1531	1.7667
4/20/2019 0:30	0.7	-0.2	4.7068	-0.75	2.4513	31.0833	0.1572	1.761
4/20/2019 0:31	0.7	0.0667	4.371	-0.2167	2.4745	31.0917	0.1421	1.7677
4/20/2019 0:32	0.6583	-0.075	4.826	0.1	2.4383	31.1	0.1566	1.784
4/20/2019 0:33	0.6	0.0167	4.6718	-0.2667	2.4626	31.1	0.15	1.7672
4/20/2019 0:34	0.6	-0.2083	4.5513	-0.0667	2.4821	31.0273	0.1575	1.7319

4/20/2019 0:35	0.5083	-0.3583	4.6938	-0.05	2.4841	31.04	0.1497	1.7316
4/20/2019 0:36	0.5	0.1167	4.5878	-0.5833	2.4731	31	0.1431	1.7553
4/20/2019 0:37	0.5167	-0.4333	4.8979	-0.2333	2.4596	30.9636	0.1541	1.7734
4/20/2019 0:38	0.6	-0.4333	4.6313	-0.5833	2.454	30.8417	0.1463	1.7704
4/20/2019 0:39	0.6583	-0.25	4.8595	-0.45	2.4668	30.8	0.1537	1.7718
4/20/2019 0:40	0.7	-0.15	4.6833	-0.5	2.4538	30.8	0.1518	1.7888
4/20/2019 0:41	0.7	-0.2417	4.9913	-0.4833	2.4754	30.8	0.1466	1.7869
4/20/2019 0:42	0.6333	-0.025	4.7266	-0.5667	2.459	30.8	0.1586	1.7791
4/20/2019 0:43	0.6	0.4333	4.815	-0.5667	2.4786	30.8182	0.1372	1.7684
4/20/2019 0:44	0.55	-0.4	4.9398	-0.5	2.4495	30.9	0.1592	1.7846
4/20/2019 0:45	0.6	-1.2333	4.555	-0.4833	2.4569	30.93	0.1363	1.7765
4/20/2019 0:46	0.6917	0.1667	4.6454	-0.25	2.4507	30.9727	0.1579	1.7504
4/20/2019 0:47	0.7	0.1833	4.9168	-0.5167	2.4693	31	0.1484	1.744
4/20/2019 0:48	0.7	-0.1583	4.3504	-0.0667	2.4558	30.9727	0.1497	1.7364
4/20/2019 0:49	0.7	-0.275	4.5993	0.1667	2.4598	31.0455	0.1515	1.7453
4/20/2019 0:50	0.7167	-0.7583	4.5878	-0.4667	2.4571	31.1545	0.1367	1.7722
4/20/2019 0:51	0.8	-0.2083	4.7464	-0.55	2.4439	31.2364	0.1415	1.7608
4/20/2019 0:52	0.8	-0.5917	4.7413	-0.85	2.4439	31.3	0.1507	1.7399
4/20/2019 0:53	0.8	0.15	4.8218	-0.6167	2.4471	31.3	0.1479	1.7425
4/20/2019 0:54	0.8	0.3417	4.8918	-0.2167	2.44	31.37	0.1635	1.7701
4/20/2019 0:55	0.8	0.425	4.8504	0.1	2.4278	31.3417	0.1385	1.7692
4/20/2019 0:56	0.8	-0.0333	4.8066	-0.1833	2.4309	31.28	0.1582	1.7728
4/20/2019 0:57	0.7667	0.3667	4.6255	-0.6167	2.4316	31.1083	0.1461	1.7613
4/20/2019 0:58	0.7	-0.9667	4.9547	-0.1667	2.4206	31.0546	0.1482	1.7364
4/20/2019 0:59	0.7	-0.1833	5.0083	-0.3833	2.4414	30.8182	0.1493	1.7523
4/20/2019 1:00	0.7	-0.6417	4.894	-0.1	2.4226	30.87	0.1438	1.7532
4/20/2019 1:01	0.7	-0.375	4.6827	-0.0667	2.4375	30.8455	0.1487	1.7557
4/20/2019 1:02	0.7	-0.3333	4.6195	-0.6833	2.4336	30.8	0.1384	1.7511
4/20/2019 1:03	0.7	0.0833	4.8688	-0.6667	2.4045	30.8	0.1493	1.7545
4/20/2019 1:04	0.7	0.1333	4.7174	-0.6667	2.4306	30.7444	0.1424	1.7665
4/20/2019 1:05	0.7	-0.325	4.8623	-0.7	2.4216	30.7	0.1408	1.7541
4/20/2019 1:06	0.6083	-0.4667	4.6765	-0.1667	2.4114	30.7	0.1564	1.747
4/20/2019 1:07	0.6	-0.3833	4.8596	-0.55	2.3899	30.7636	0.1404	1.7523
4/20/2019 1:08	0.5917	-0.05	4.6758	-0.2333	2.3799	30.8	0.1546	1.7574
4/20/2019 1:09	0.5083	-0.225	4.5958	-0.65	2.4052	30.8833	0.1456	1.7711
4/20/2019 1:10	0.5	-0.375	4.8999	-0.6	2.4222	30.9636	0.1424	1.7763
4/20/2019 1:11	0.5	-0.2833	4.79	-0.1167	2.4184	31.1818	0.1534	1.7759
4/20/2019 1:12	0.6	-0.1833	4.6395	-0.4833	2.3953	31.2	0.1374	1.8021
4/20/2019 1:13	0.6	-0.3917	4.6073	-0.7167	2.4174	31.2	0.1479	1.7841
4/20/2019 1:14	0.6	0.3583	4.6284	-0.6167	2.423	31.2	0.135	1.7808
4/20/2019 1:15	0.6	-0.2583	4.8074	-1	2.4231	31.2	0.156	1.7646
4/20/2019 1:16	0.6	-0.5167	4.6251	-0.6667	2.3769	31.2	0.1506	1.7593
4/20/2019 1:17	0.6	-0.0333	4.7127	-0.8	2.3414	31.1545	0.1372	1.782
4/20/2019 1:18	0.6	-0.4167	4.5861	-0.1167	2.3578	31.1	0.1583	1.7828
4/20/2019 1:19	0.6583	-0.4333	4.8435	-0.35	2.3482	31.1	0.1311	1.7904
4/20/2019 1:20	0.7	-0.6917	4.7719	-0.3833	2.3597	31.1	0.1519	1.7738
4/20/2019 1:21	0.7	-0.125	4.7083	0.2667	2.3679	31.1	0.1362	1.7945

4/20/2019 1:22	0.7	-0.0917	4.9119	-0.55	2.3676	31.1	0.1472	1.7582
4/20/2019 1:23	0.7	0.2833	4.5927	0.0833	2.3396	31.1	0.1526	1.7679
4/20/2019 1:24	0.7	-0.975	4.4653	0.25	2.3563	31.17	0.1368	1.786
4/20/2019 1:25	0.7	0.0833	4.9021	-0.1667	2.3498	31.1818	0.1496	1.8024
4/20/2019 1:26	0.7	-0.175	4.7293	-0.45	2.3496	31.2	0.1372	1.7798
4/20/2019 1:27	0.7	-0.6917	4.6362	-0.1167	2.3441	31.21	0.1555	1.771
4/20/2019 1:28	0.7	-0.3417	4.753	-0.5667	2.3331	31.2333	0.1482	1.786
4/20/2019 1:29	0.7	-0.15	4.9243	-0.2333	2.3368	31.2583	0.145	1.7913
4/20/2019 1:30	0.7	0.8	4.839	-0.1	2.3388	31.4	0.1511	1.7837
4/20/2019 1:31	0.65	0.125	4.7509	-0.15	2.3333	31.4	0.1383	1.7862
4/20/2019 1:32	0.6	-0.9	4.8208	-0.7333	2.335	31.4	0.1531	1.8117
4/20/2019 1:33	0.6	0.525	4.7502	-0.4667	2.3002	31.4	0.1402	1.7898
4/20/2019 1:34	0.6	-0.0917	4.7297	-0.7	2.3234	31.4	0.148	1.7821
4/20/2019 1:35	0.6	-0.0667	4.7582	-0.7833	2.3172	31.32	0.1464	1.7784
4/20/2019 1:36	0.5333	0.1333	4.7846	-0.3833	2.3143	31.3	0.1526	1.7922
4/20/2019 1:37	0.5	0.075	4.7798	-0.6333	2.338	31.3	0.1542	1.7883
4/20/2019 1:38	0.4417	-0.3667	4.9029	-0.5833	2.3467	31.4	0.1396	1.7549
4/20/2019 1:39	0.4333	-0.25	4.9177	-0.25	2.3426	31.3546	0.1577	1.7683
4/20/2019 1:40	0.5	0.075	4.8239	-0.45	2.3019	31.3	0.1383	1.7797
4/20/2019 1:41	0.55	-0.3833	4.7014	-0.45	2.326	31.3	0.148	1.7982
4/20/2019 1:42	0.6	0.825	4.5472	-0.6167	2.329	31.3	0.1566	1.799
4/20/2019 1:43	0.6	0.0167	4.6651	0.1167	2.3357	31.3	0.1321	1.7985
4/20/2019 1:44	0.6	-0.0333	4.7233	-0.5333	2.3191	31.3	0.1469	1.8043
4/20/2019 1:45	0.6	0.4667	4.7713	0.0833	2.2958	31.3	0.1415	1.7809
4/20/2019 1:46	0.5333	-0.05	4.9492	-0.75	2.3204	31.4	0.1578	1.7949
4/20/2019 1:47	0.5333	0.0167	4.8378	-0.4	2.3008	31.4	0.1425	1.8029
4/20/2019 1:48	0.525	0.2667	4.9386	-0.1667	2.3401	31.4	0.1448	1.8032
4/20/2019 1:49	0.6	-0.775	4.7453	-0.5167	2.3116	31.3167	0.1503	1.783
4/20/2019 1:50	0.625	0.5917	4.7768	-0.0833	2.3144	31.3	0.1432	1.7358
4/20/2019 1:51	0.7	-0.0583	4.7734	-0.4833	2.3238	31.3	0.1498	1.7342
4/20/2019 1:52	0.7	0.7583	4.7963	-0.75	2.3128	31.37	0.138	1.7832
4/20/2019 1:53	0.7	-0.575	4.6481	-0.7	2.3109	31.4	0.1506	1.7853
4/20/2019 1:54	0.7	-0.2417	4.5284	-0.5667	2.3043	31.4	0.1408	1.7689
4/20/2019 1:55	0.725	-0.2583	4.7534	-0.6	2.3041	31.4	0.1407	1.7623
4/20/2019 1:56	0.7	-0.675	4.8474	-0.6833	2.3123	31.3455	0.1504	1.8063
4/20/2019 1:57	0.7	-0.5667	4.9059	-0.6167	2.3114	31.3	0.1397	1.7997
4/20/2019 1:58	0.7333	-0.2167	4.8242	-0.6167	2.2931	31.3	0.1548	1.7683
4/20/2019 1:59	0.8	-0.25	4.997	-0.1333	2.3018	31.2727	0.1454	1.7683
4/20/2019 2:00	0.8	-0.0417	4.6587	-0.75	2.3041	31.2091	0.1484	1.7827
4/20/2019 2:01	0.8	0.175	4.6768	-0.2833	2.2991	31.3	0.149	1.7826
4/20/2019 2:02	0.8	-0.5417	4.886	-0.8667	2.2973	31.3	0.1399	1.7968
4/20/2019 2:03	0.8	0.6083	4.9414	-0.1833	2.2978	31.3	0.1548	1.7779
4/20/2019 2:04	0.8	-0.3333	4.8467	0.0833	2.2917	31.28	0.1421	1.7897
4/20/2019 2:05	0.8167	-0.775	4.8282	-0.5833	2.276	31.26	0.1526	1.797
4/20/2019 2:06	0.8	-0.175	4.7805	-0.2333	2.3146	31.15	0.1314	1.7499
4/20/2019 2:07	0.775	0.2083	4.9202	-0.5667	2.2932	31.1	0.1458	1.7492
4/20/2019 2:08	0.7167	-0.025	4.8398	-0.2333	2.2869	31.1	0.145	1.7513

4/20/2019 2:09	0.7	0.1	4.7377	-0.1667	2.32	31.19	0.1406	1.7735
4/20/2019 2:10	0.7	-0.7333	5.0572	-0.6	2.2913	31.2	0.1567	1.82
4/20/2019 2:11	0.7	-0.525	4.793	-0.1667	2.3008	31.2	0.1396	1.8178
4/20/2019 2:12	0.7	0.25	5.1022	-0.8667	2.2971	31.2	0.1538	1.8151
4/20/2019 2:13	0.7	-0.725	4.7167	-0.3167	2.2818	31.1727	0.143	1.7785
4/20/2019 2:14	0.7833	0.1167	4.771	-0.2667	2.2738	31.2	0.1389	1.8057
4/20/2019 2:15	0.8	-0.2167	5.0543	-0.4333	2.263	31.2	0.1518	1.7907
4/20/2019 2:16	0.8	-0.4833	4.535	-0.5833	2.2631	31.1333	0.1375	1.7792
4/20/2019 2:17	0.7	-0.4167	4.7757	-0.2667	2.2456	31.1364	0.152	1.808
4/20/2019 2:18	0.6	-0.425	4.6213	-0.0833	2.2768	31.1818	0.1403	1.7999
4/20/2019 2:19	0.575	-0.1417	4.8512	-0.4167	2.2673	31.2	0.1508	1.7683
4/20/2019 2:20	0.525	0.4583	4.5127	-0.45	2.2796	31.2	0.1484	1.7543
4/20/2019 2:21	0.6	0.05	4.8043	0	2.2523	31.1	0.1316	1.7807
4/20/2019 2:22	0.5833	-0.6833	4.7605	-0.5	2.2599	31.09	0.1458	1.7826
4/20/2019 2:23	0.6	-0.3667	4.6238	-0.7333	2.2859	31.0636	0.1437	1.7869
4/20/2019 2:24	0.6	-0.025	4.5497	-0.5167	2.2894	31.1	0.1449	1.7696
4/20/2019 2:25	0.5917	-0.5417	4.73	-0.3833	2.2847	31.1273	0.1506	1.7845
4/20/2019 2:26	0.5833	0.3	4.7008	-0.3167	2.2575	31.1273	0.1303	1.788
4/20/2019 2:27	0.6	0.0083	4.7657	-0.5	2.2845	31	0.1489	1.7731
4/20/2019 2:28	0.6	0.025	4.7323	-0.8333	2.2692	31	0.1394	1.7583
4/20/2019 2:29	0.6667	-0.6333	4.6825	-0.7667	2.247	30.93	0.1472	1.7808
4/20/2019 2:30	0.6083	-0.2667	4.8313	-0.1667	2.277	30.9	0.1415	1.771
4/20/2019 2:31	0.7	-0.65	5.029	-0.6333	2.2488	30.9	0.1428	1.7878
4/20/2019 2:32	0.6833	-0.0167	4.6187	-0.6167	2.2478	30.9	0.1521	1.7732
4/20/2019 2:33	0.7	0.2833	4.4985	-0.2	2.2539	30.9	0.1339	1.7723
4/20/2019 2:34	0.6917	0.1	4.6152	-0.3167	2.2541	30.9	0.1503	1.7803
4/20/2019 2:35	0.7	-0.2167	4.5842	-0.5333	2.2449	30.96	0.1373	1.7854
4/20/2019 2:36	0.7	-0.175	4.7172	-0.4167	2.2408	30.975	0.1399	1.7503
4/20/2019 2:37	0.7	-0.575	4.7085	-0.05	2.2558	31	0.1401	1.7551
4/20/2019 2:38	0.7	-0.2333	4.6305	-0.6833	2.2487	31.02	0.1395	1.7493
4/20/2019 2:39	0.7333	-0.6417	4.6433	-0.3	2.2653	31.0455	0.1496	1.7642
4/20/2019 2:40	0.8	-0.2333	4.8332	0.5	2.2727	31	0.134	1.7391
4/20/2019 2:41	0.8	-0.2667	4.9257	-0.6833	2.248	31.0818	0.1536	1.7252
4/20/2019 2:42	0.7833	-0.0583	5.03	-0.4167	2.2428	31.0818	0.1422	1.7701
4/20/2019 2:43	0.7333	0.0083	4.8373	-0.2333	2.2433	30.9273	0.138	1.7774
4/20/2019 2:44	0.7083	0.1417	4.6483	-0.6667	2.248	30.9	0.143	1.7546
4/20/2019 2:45	0.7167	0.4917	4.6593	-0.1833	2.2448	30.9	0.1392	1.7478
4/20/2019 2:46	0.7	0	4.7703	-0.6667	2.2518	30.8778	0.144	1.7806
4/20/2019 2:47	0.7	-0.7917	4.8842	-1.15	2.2343	30.8364	0.1352	1.788
4/20/2019 2:48	0.7	0.15	4.7238	-0.5333	2.2623	30.82	0.1455	1.7815
4/20/2019 2:49	0.7	-0.8	4.5577	-0.05	2.2417	30.7273	0.1437	1.7747
4/20/2019 2:50	0.7	0.275	4.8088	-0.45	2.2476	30.7	0.149	1.8015
4/20/2019 2:51	0.7	-0.15	4.6538	-0.6833	2.2449	30.7182	0.1468	1.7842
4/20/2019 2:52	0.7	-1.1333	4.8977	-0.45	2.2427	30.78	0.1306	1.7709
4/20/2019 2:53	0.7083	0.325	4.6145	0.3167	2.2556	30.73	0.16	1.795
4/20/2019 2:54	0.7182	-0.3333	4.5185	-0.65	2.2478	30.7364	0.1378	1.7627
4/20/2019 2:55	0.7	-0.15	4.7023	-0.2167	2.2356	30.88	0.1508	1.7608

4/20/2019 2:56	0.7	-0.425	4.771	-0.4333	2.2253	31	0.1491	1.7685
4/20/2019 2:57	0.7	-0.3417	4.8173	-0.3333	2.2415	30.9	0.1417	1.7344
4/20/2019 2:58	0.7	-0.175	4.8658	-0.4167	2.2587	30.9	0.1559	1.7633
4/20/2019 2:59	0.7	-0.5583	4.8912	-0.4833	2.2402	30.9909	0.1411	1.7604
4/20/2019 3:00	0.7	-0.275	4.6457	-0.65	2.2251	31	0.1492	1.7615
4/20/2019 3:01	0.7333	0.2	5.0272	-0.3	2.2398	31	0.1473	1.7591
4/20/2019 3:02	0.7167	-0.5333	4.6802	-0.0333	2.2513	30.9583	0.1382	1.7583
4/20/2019 3:03	0.6333	-0.3833	4.6087	-0.3833	2.2296	30.8364	0.1542	1.7846
4/20/2019 3:04	0.6	0.025	4.7667	-0.1	2.2546	30.7091	0.1266	1.7751
4/20/2019 3:05	0.6	-0.3	4.7225	0.2667	2.2315	30.59	0.158	1.7798
4/20/2019 3:06	0.6	-0.35	4.6518	-0.2667	2.2428	30.4273	0.1449	1.7581
4/20/2019 3:07	0.6	-0.0583	4.7418	-0.2833	2.2523	30.4	0.1439	1.7522
4/20/2019 3:08	0.6	0.1417	4.9975	-0.15	2.2423	30.4	0.1461	1.8
4/20/2019 3:09	0.625	-0.2083	4.7322	-0.0667	2.2503	30.325	0.1373	1.8073
4/20/2019 3:10	0.7	0.175	4.6077	-0.5833	2.2206	30.3	0.1504	1.802
4/20/2019 3:11	0.7	0.2167	4.7227	-0.85	2.2502	30.3	0.1373	1.804
4/20/2019 3:12	0.6833	-0.3333	4.5765	-0.3	2.2405	30.2455	0.154	1.7752
4/20/2019 3:13	0.6	0.2417	4.9193	-0.4333	2.2379	30.2	0.141	1.7913
4/20/2019 3:14	0.6	-0.9167	4.7847	-0.4667	2.2556	30.2	0.1489	1.7978
4/20/2019 3:15	0.6	0.025	4.9662	-0.4	2.2538	30.2889	0.1509	1.7843
4/20/2019 3:16	0.6	-0.7	4.7673	0.0167	2.262	30.47	0.1439	1.7739
4/20/2019 3:17	0.6	-0.1833	5.0275	-0.9833	2.262	30.52	0.1483	1.7738
4/20/2019 3:18	0.6083	-0.2	4.7358	-0.3	2.2439	30.78	0.1362	1.7688
4/20/2019 3:19	0.6917	-0.7917	4.8238	-0.05	2.248	30.8556	0.1519	1.7913
4/20/2019 3:20	0.6833	0.075	4.3112	-1.0333	2.2448	30.9	0.1478	1.7806
4/20/2019 3:21	0.6833	-0.2667	4.691	0.0667	2.2415	30.9	0.148	1.7722
4/20/2019 3:22	0.625	0.15	4.413	0.0333	2.2379	30.94	0.1538	1.7735
4/20/2019 3:23	0.6	-0.2417	4.4867	-0.4667	2.2602	30.94	0.131	1.7808
4/20/2019 3:24	0.6	-0.0417	4.6723	-0.35	2.2613	30.9	0.155	1.7774
4/20/2019 3:25	0.6167	-0.5667	4.7123	-0.5	2.2573	30.98	0.1368	1.7764
4/20/2019 3:26	0.7	0	4.7278	-0.1333	2.2589	31	0.1412	1.7738
4/20/2019 3:27	0.7	-0.125	4.6368	-0.3333	2.2598	30.9364	0.141	1.7948
4/20/2019 3:28	0.675	0.0167	4.4407	-0.7	2.2729	30.9	0.1452	1.8107
4/20/2019 3:29	0.6583	0.25	4.7293	-0.1833	2.2782	30.9	0.145	1.7852
4/20/2019 3:30	0.6667	-0.45	4.9767	-0.0333	2.2688	30.9	0.1447	1.7873
4/20/2019 3:31	0.6	0.1	4.9653	-0.2667	2.2572	30.9	0.1559	1.7706
4/20/2019 3:32	0.6333	-0.025	4.7973	-0.35	2.2625	30.9	0.1412	1.7922
4/20/2019 3:33	0.6333	-0.2167	4.4818	-0.8667	2.2659	30.9	0.1538	1.8043
4/20/2019 3:34	0.7	-0.5417	4.5349	-0.45	2.257	30.96	0.142	1.788
4/20/2019 3:35	0.7	0.0667	4.6525	-0.4833	2.2533	31.01	0.1386	1.8041
4/20/2019 3:36	0.7	-0.7833	4.8999	-0.6667	2.2702	31.15	0.1535	1.8069
4/20/2019 3:37	0.65	-0.575	4.7189	-0.2667	2.2536	31.2	0.137	1.7834
4/20/2019 3:38	0.6	-0.55	4.5846	-0.6333	2.2538	31.2	0.1531	1.7571
4/20/2019 3:39	0.6	-0.4	4.6863	-0.45	2.2698	31.2	0.1365	1.7743
4/20/2019 3:40	0.6	-0.35	4.4196	-0.6167	2.2723	31.3	0.1522	1.7878
4/20/2019 3:41	0.6	0.0333	4.6088	-0.3333	2.2908	31.22	0.1408	1.7528
4/20/2019 3:42	0.6	-0.2667	4.8112	-0.55	2.2642	31.2	0.1414	1.7716

4/20/2019 3:43	0.6	-0.125	4.5398	0	2.2871	31.22	0.1522	1.7719
4/20/2019 3:44	0.6	-0.6917	4.6418	-0.7833	2.2813	31.3	0.1312	1.7604
4/20/2019 3:45	0.6	-0.325	4.7368	0.1333	2.2792	31.2455	0.1482	1.7414
4/20/2019 3:46	0.6	-0.8833	4.6418	-0.5667	2.2893	31.2	0.1376	1.7433
4/20/2019 3:47	0.55	-0.3833	4.6228	-0.1833	2.2713	31.1091	0.1457	1.7556
4/20/2019 3:48	0.525	0.3	4.5723	-0.7	2.2734	30.91	0.1493	1.7854
4/20/2019 3:49	0.5	-1.0667	4.5938	-0.35	2.2871	30.8	0.1408	1.7812
4/20/2019 3:50	0.5167	0.0917	4.4918	-0.7833	2.311	30.7727	0.1554	1.7924
4/20/2019 3:51	0.5167	-0.3167	4.737	-0.4667	2.298	30.7091	0.1331	1.7931
4/20/2019 3:52	0.5333	-0.4583	4.3898	-0.4	2.2851	30.75	0.1502	1.7885
4/20/2019 3:53	0.6	-0.6583	4.2649	-0.5	2.3348	30.8	0.1447	1.8148
4/20/2019 3:54	0.6	-0.6917	4.4389	0.1833	2.2699	30.78	0.1422	1.7933
4/20/2019 3:55	0.6583	-0.3333	4.4877	-0.7833	2.3	30.63	0.1515	1.7688
4/20/2019 3:56	0.6583	-1.1583	4.4663	-0.65	2.2966	30.4364	0.1396	1.7888
4/20/2019 3:57	0.6917	-0.1	4.2884	0	2.3098	30.4	0.1462	1.7677
4/20/2019 3:58	0.6917	-0.225	4.8511	-0.3833	2.3044	30.4	0.1403	1.795
4/20/2019 3:59	0.7	-0.4417	4.6253	-0.5	2.3368	30.4	0.1403	1.7998
4/20/2019 4:00	0.6167	-0.8417	4.7106	-0.3833	2.3025	30.4909	0.147	1.7947
4/20/2019 4:01	0.6	0.15	4.4993	-0.5333	2.2969	30.6	0.1332	1.8196
4/20/2019 4:02	0.6	-0.55	4.5068	-0.3	2.3196	30.6	0.1477	1.8395
4/20/2019 4:03	0.6	0.2667	4.2073	-0.2833	2.2982	30.6	0.1316	1.811
4/20/2019 4:04	0.6333	-0.2417	4.5025	-0.7167	2.3059	30.5818	0.1512	1.7872
4/20/2019 4:05	0.6	-0.2667	4.3959	-0.4167	2.2822	30.53	0.1378	1.7821
4/20/2019 4:06	0.625	-0.0417	4.4056	-0.4333	2.303	30.6545	0.1403	1.7753
4/20/2019 4:07	0.6083	0.3667	4.7197	-0.5167	2.3233	30.8636	0.1517	1.7731
4/20/2019 4:08	0.6583	0.0333	4.3376	-0.7333	2.3233	30.9455	0.1315	1.7734
4/20/2019 4:09	0.7	-0.075	4.7443	-0.6	2.3196	31.1182	0.1432	1.7709
4/20/2019 4:10	0.7	-0.125	4.729	-0.5	2.3175	31.2	0.1344	1.7809
4/20/2019 4:11	0.7	-0.3417	4.918	-0.5333	2.3287	31.3	0.1433	1.7925
4/20/2019 4:12	0.6417	0.2917	4.3903	-0.1333	2.3132	31.3	0.1416	1.8071
4/20/2019 4:13	0.6	-0.5917	4.7326	-0.75	2.3091	31.4	0.1374	1.8103
4/20/2019 4:14	0.6	-0.9083	4.1988	-0.1333	2.3371	31.4	0.1492	1.7667
4/20/2019 4:15	0.6333	-0.975	4.3039	-0.5833	2.3017	31.4091	0.1341	1.7443
4/20/2019 4:16	0.7	0.1417	4.4126	0.0167	2.3157	31.52	0.1489	1.7703
4/20/2019 4:17	0.7	-0.4583	4.4139	-0.1667	2.299	31.6	0.1365	1.7828
4/20/2019 4:18	0.65	-0.8833	4.4028	-0.3333	2.299	31.5546	0.1381	1.7561
4/20/2019 4:19	0.6	-0.1583	4.3532	-0.1833	2.302	31.5	0.1416	1.7473
4/20/2019 4:20	0.6417	-0.625	4.5797	-0.05	2.3361	31.5917	0.135	1.7768
4/20/2019 4:21	0.65	-0.6167	4.6536	-0.0833	2.3113	31.67	0.1416	1.7753
4/20/2019 4:22	0.7	-0.1333	4.6118	-0.8167	2.3215	31.67	0.1304	1.7998
4/20/2019 4:23	0.75	0.3167	4.1664	-0.45	2.3094	31.7	0.1406	1.7993
4/20/2019 4:24	0.7917	-0.375	4.5464	-0.2667	2.2991	31.7455	0.1446	1.7808
4/20/2019 4:25	0.7833	-0.2	4.3334	-0.5333	2.3053	31.8	0.1405	1.7792
4/20/2019 4:26	0.725	-0.7167	4.1381	-0.6667	2.275	31.8	0.1461	1.8139
4/20/2019 4:27	0.7	-0.175	4.557	-0.25	2.2958	31.8	0.1329	1.8187
4/20/2019 4:28	0.7	-0.2417	4.8098	-0.3667	2.287	31.8	0.1463	1.7968
4/20/2019 4:29	0.6917	-0.5833	4.4107	-0.9333	2.2932	31.76	0.1296	1.7763

4/20/2019 4:30	0.6833	-0.3	4.5686	-0.6333	2.2823	31.7	0.1399	1.7871
4/20/2019 4:31	0.65	-0.075	4.4951	-0.8667	2.2985	31.6778	0.1422	1.8059
4/20/2019 4:32	0.6083	0.025	4.5803	-0.25	2.3023	31.6	0.1372	1.8066
4/20/2019 4:33	0.6083	-0.8917	4.3212	-0.6	2.289	31.5455	0.1508	1.8148
4/20/2019 4:34	0.6	0.4917	4.3946	-0.4333	2.3196	31.5	0.1292	1.8136
4/20/2019 4:35	0.6	-0.6833	4.5123	-0.1333	2.2903	31.4455	0.1458	1.8388
4/20/2019 4:36	0.6	0.3667	4.6354	-0.6	2.2999	31.4	0.1421	1.8076
4/20/2019 4:37	0.6	0.2917	4.4087	-0.8833	2.2853	31.4	0.135	1.8102
4/20/2019 4:38	0.6	-0.5583	4.4474	-0.4167	2.2895	31.4	0.1418	1.7936
4/20/2019 4:39	0.6	-0.35	4.5712	0.2333	2.2981	31.4	0.1323	1.8008
4/20/2019 4:40	0.6417	-0.425	4.248	0.0667	2.2715	31.3818	0.1489	1.7802
4/20/2019 4:41	0.6167	-0.5917	4.5081	-0.25	2.2702	31.3364	0.1324	1.7813
4/20/2019 4:42	0.6	-0.375	4.6007	0.2333	2.2525	31.4	0.1458	1.7904
4/20/2019 4:43	0.5333	-0.625	4.2767	-0.55	2.3035	31.43	0.1405	1.7985
4/20/2019 4:44	0.5417	-0.4	4.7573	-0.3667	2.2888	31.5	0.1384	1.7972
4/20/2019 4:45	0.6	-0.5417	4.5711	-0.2667	2.2969	31.5	0.1453	1.8035
4/20/2019 4:46	0.6	-0.4083	4.6266	-0.1833	2.2908	31.5	0.1293	1.7993
4/20/2019 4:47	0.6	-0.1083	4.8335	-0.2833	2.2811	31.5	0.1417	1.7755
4/20/2019 4:48	0.5583	-0.2667	4.6378	-0.3167	2.2913	31.5	0.1319	1.8093
4/20/2019 4:49	0.6	-0.175	4.6838	-0.3167	2.2713	31.48	0.1397	1.8285
4/20/2019 4:50	0.6	-0.2833	4.6089	-0.1833	2.2833	31.38	0.1471	1.8153
4/20/2019 4:51	0.6917	-0.55	4.505	-0.6667	2.2587	31.33	0.127	1.8121
4/20/2019 4:52	0.7	-0.4333	4.4533	-0.7833	2.2833	31.3455	0.1512	1.799
4/20/2019 4:53	0.7	-0.6667	4.2211	-0.35	2.2738	31.3	0.1246	1.7912
4/20/2019 4:54	0.7	0.0833	4.35	-0.85	2.2648	31.3	0.1407	1.7988
4/20/2019 4:55	0.7	-0.3	4.6133	-0.6667	2.2769	31.2636	0.1382	1.7974
4/20/2019 4:56	0.7	-1.1333	4.5836	0	2.2753	31.15	0.138	1.796
4/20/2019 4:57	0.6667	-0.2083	4.6493	-1.2667	2.2728	31.1	0.1463	1.7903
4/20/2019 4:58	0.6	0.3667	4.4615	-0.3833	2.2826	31.1	0.1346	1.766
4/20/2019 4:59	0.6583	-0.0167	4.3793	-0.8333	2.2692	31.0727	0.1466	1.7813
4/20/2019 5:00	0.6333	-0.1667	4.4761	-0.7167	2.2702	31.02	0.1323	1.7824
4/20/2019 5:01	0.7	-0.4833	4.6865	-0.3667	2.276	31.1	0.1467	1.7945
4/20/2019 5:02	0.6167	-0.4417	4.4325	-0.1833	2.2884	31.1	0.1349	1.7984
4/20/2019 5:03	0.6333	-0.2083	4.724	-0.5167	2.2885	31.1	0.1438	1.8204
4/20/2019 5:04	0.6083	0.3833	4.462	-0.4833	2.3145	31.0273	0.1427	1.7802
4/20/2019 5:05	0.6	0.3667	4.5717	-0.3333	2.2792	30.9909	0.1343	1.7805
4/20/2019 5:06	0.6	-0.1583	4.4727	0.0167	2.288	30.8546	0.1455	1.7971
4/20/2019 5:07	0.6	-0.2667	4.5948	-0.4833	2.2737	30.8	0.1378	1.809
4/20/2019 5:08	0.6	-0.0417	4.8242	-0.3333	2.2712	30.8091	0.1401	1.7986
4/20/2019 5:09	0.6	-0.8	4.7168	-0.75	2.2793	30.9	0.1411	1.8137
4/20/2019 5:10	0.5833	-0.275	4.6157	-0.25	2.288	30.9	0.1277	1.8081
4/20/2019 5:11	0.5	-0.5167	4.6327	-0.0833	2.3013	30.9	0.1472	1.7839
4/20/2019 5:12	0.5	0.15	4.4538	-0.3833	2.2646	30.9	0.137	1.7818
4/20/2019 5:13	0.5	-0.1333	4.5893	-0.2	2.2767	30.8833	0.1441	1.7925
4/20/2019 5:14	0.5	-0.2667	4.8618	-0.2833	2.2864	30.8	0.137	1.7809
4/20/2019 5:15	0.4917	-0.925	4.7267	-0.0167	2.2836	30.8417	0.1381	1.7898
4/20/2019 5:16	0.5	-0.5333	4.6075	-0.8	2.2989	30.9273	0.1445	1.8308

4/20/2019 5:17	0.5	-0.475	4.5598	-0.9333	2.2973	31.0091	0.1298	1.815
4/20/2019 5:18	0.5	-0.575	4.782	-0.6	2.3045	31.15	0.1423	1.8043
4/20/2019 5:19	0.5	-0.8417	4.5755	-0.3167	2.305	31.24	0.1306	1.8074
4/20/2019 5:20	0.5	0.0583	4.5382	-0.5167	2.3119	31.3455	0.1379	1.8254
4/20/2019 5:21	0.5	-0.3167	4.5463	-0.7	2.3197	31.4	0.1458	1.8039
4/20/2019 5:22	0.5	-0.3833	4.6947	-0.1833	2.2947	31.3182	0.1329	1.8025
4/20/2019 5:23	0.5083	-0.275	4.8017	-0.6667	2.2905	31.3273	0.1488	1.8057
4/20/2019 5:24	0.6	-0.2333	4.5742	-0.0833	2.3026	31.3	0.1281	1.8239
4/20/2019 5:25	0.6	0.5917	4.6878	-0.7	2.3003	31.3	0.1487	1.825
4/20/2019 5:26	0.6	-0.95	4.731	-0.2333	2.2962	31.37	0.1333	1.8061
4/20/2019 5:27	0.6	0.075	4.6838	-0.4833	2.3101	31.4	0.137	1.8027
4/20/2019 5:28	0.6	-0.3333	4.7915	-0.25	2.3054	31.4	0.1466	1.7865
4/20/2019 5:29	0.6	-0.0667	4.6822	-0.8	2.3143	31.3091	0.1342	1.7935
4/20/2019 5:30	0.6083	0.05	4.4973	-0.7667	2.313	31.3	0.1433	1.8013
4/20/2019 5:31	0.6	-0.25	4.6667	-0.2833	2.2992	31.3	0.1317	1.7804
4/20/2019 5:32	0.6	-0.5083	4.5902	-0.7333	2.3036	31.2273	0.1435	1.7856
4/20/2019 5:33	0.6	-0.4083	4.6918	0.1333	2.3113	31.1667	0.1434	1.8218
4/20/2019 5:34	0.6	-0.5083	4.4252	-0.3167	2.3183	31.16	0.1315	1.8278
4/20/2019 5:35	0.6	0.275	4.6657	0.0333	2.2933	31.1	0.1435	1.8093
4/20/2019 5:36	0.6	0.4167	4.4988	0.0167	2.3173	31.1909	0.1362	1.8059
4/20/2019 5:37	0.6	-0.5583	4.525	-0.8333	2.3168	31.2	0.1438	1.7876
4/20/2019 5:38	0.5667	-0.3083	4.3925	-0.1333	2.3381	31.0546	0.1334	1.7858
4/20/2019 5:39	0.5	-0.6667	4.4165	-0.1167	2.3273	31	0.1452	1.777
4/20/2019 5:40	0.5	-1.0917	4.4498	-0.6667	2.3082	30.8818	0.1414	1.7954
4/20/2019 5:41	0.475	0.1917	4.5275	-1.15	2.3421	30.73	0.1335	1.8127
4/20/2019 5:42	0.5417	0.1167	4.6908	-0.25	2.3294	30.7	0.1493	1.8107
4/20/2019 5:43	0.6	-0.1083	4.489	-0.7	2.328	30.7	0.1342	1.7933
4/20/2019 5:44	0.6	-0.525	4.4083	-0.25	2.3049	30.6273	0.1463	1.8028
4/20/2019 5:45	0.6	-0.5917	4.854	0.05	2.3405	30.6	0.1272	1.792
4/20/2019 5:46	0.6083	-1.0833	4.4147	-0.45	2.3231	30.6	0.1411	1.7898
4/20/2019 5:47	0.6	-0.025	4.5898	-0.25	2.3314	30.5778	0.141	1.7782
4/20/2019 5:48	0.6	-0.0667	4.4473	0.1167	2.3503	30.5	0.1327	1.7995
4/20/2019 5:49	0.6	-1.5583	4.4318	-0.35	2.3447	30.5	0.1482	1.801
4/20/2019 5:50	0.6	-0.5167	4.4458	-0.8333	2.3454	30.53	0.131	1.8096
4/20/2019 5:51	0.6667	-0.5083	4.4105	-0.7	2.3523	30.5546	0.142	1.8243
4/20/2019 5:52	0.7	-0.0667	4.3488	-0.1	2.3683	30.5636	0.1398	1.8135
4/20/2019 5:53	0.6583	-0.25	4.6418	-0.8	2.3329	30.7455	0.1399	1.7992
4/20/2019 5:54	0.6	-0.5083	4.4773	-0.4333	2.3295	30.7727	0.1416	1.7946
4/20/2019 5:55	0.6	-0.3833	4.5012	-0.5	2.3418	30.7	0.1269	1.8098
4/20/2019 5:56	0.6083	-0.15	4.5663	-0.5667	2.3355	30.8273	0.1466	1.8098
4/20/2019 5:57	0.7	0.5417	4.393	-0.5333	2.3489	30.9	0.1294	1.7988
4/20/2019 5:58	0.7	-0.775	4.2977	-0.4667	2.3438	30.9727	0.144	1.8143
4/20/2019 5:59	0.7	-0.475	4.3947	-0.8667	2.3664	31.05	0.1416	1.7896
4/20/2019 6:00	0.7	-0.6083	4.557	-0.45	2.3446	31.2	0.133	1.8085
4/20/2019 6:01	0.6833	-0.0833	4.4415	-0.8667	2.3149	31.2	0.1427	1.8236
4/20/2019 6:02	0.6	-0.2083	4.3895	-0.2	2.3308	31.2	0.1283	1.8293
4/20/2019 6:03	0.6	-0.425	4.3717	-0.1833	2.3212	31.3	0.1409	1.8091

4/20/2019 6:04	0.6833	0.1833	4.489	-1	2.3429	31.4	0.1296	1.8057
4/20/2019 6:05	0.7	-0.0333	4.4245	-0.6333	2.3556	31.5	0.1351	1.807
4/20/2019 6:06	0.6583	-0.6583	4.4555	-0.8333	2.364	31.4273	0.1357	1.8325
4/20/2019 6:07	0.6583	0.6833	4.3205	-0.5667	2.3178	31.5	0.1329	1.7937
4/20/2019 6:08	0.7	-0.55	4.5452	-0.8167	2.3658	31.4182	0.1491	1.7578
4/20/2019 6:09	0.7	0.65	4.2548	-1	2.3582	31.4	0.1352	1.7828
4/20/2019 6:10	0.7	-0.5167	4.6075	-0.3833	2.3528	31.3455	0.1431	1.7805
4/20/2019 6:11	0.7	-0.275	4.389	-0.6833	2.3588	31.3	0.1397	1.7907
4/20/2019 6:12	0.7	-0.8833	4.4488	-0.2167	2.3458	31.34	0.1297	1.7908
4/20/2019 6:13	0.7	-0.0417	4.4973	-0.95	2.3519	31.4	0.1484	1.8033
4/20/2019 6:14	0.7	-0.0917	4.525	-1.3167	2.3556	31.4	0.13	1.8004
4/20/2019 6:15	0.7	-0.8583	4.5753	-0.2167	2.3498	31.4	0.1438	1.7713
4/20/2019 6:16	0.7	-0.1	4.47	-0.25	2.3426	31.4	0.1355	1.8057
4/20/2019 6:17	0.7583	-0.8167	4.3777	-0.3	2.3467	31.32	0.1281	1.7998
4/20/2019 6:18	0.8	-0.8167	4.463	-0.4167	2.3641	31.3636	0.1501	1.7985
4/20/2019 6:19	0.7417	-0.0333	4.4638	-0.1333	2.3639	31.4	0.13	1.8026
4/20/2019 6:20	0.6667	0.1917	4.559	-0.4	2.362	31.4546	0.1432	1.7824
4/20/2019 6:21	0.6083	-0.3167	4.5118	-0.0667	2.3455	31.5	0.1324	1.7604
4/20/2019 6:22	0.6	-0.9333	4.3968	-0.6	2.3431	31.5	0.1406	1.7578
4/20/2019 6:23	0.6167	-0.6667	4.4148	-0.9167	2.3272	31.5	0.1388	1.796
4/20/2019 6:24	0.6	0.3583	4.3083	-0.4	2.3368	31.5	0.1283	1.8274
4/20/2019 6:25	0.6	-0.2	4.3648	-0.4667	2.3499	31.5	0.1483	1.8082
4/20/2019 6:26	0.6	0.0333	4.5883	-0.55	2.3401	31.45	0.1328	1.7848
4/20/2019 6:27	0.6	-0.6833	4.373	-0.65	2.3557	31.5	0.1361	1.7799
4/20/2019 6:28	0.6	-0.6417	4.2548	-0.4667	2.3428	31.4091	0.1405	1.7919
4/20/2019 6:29	0.6	-0.3	4.1787	0.4167	2.3599	31.4	0.1331	1.806
4/20/2019 6:30	0.6583	-0.2667	4.5912	-0.3833	2.349	31.43	0.1392	1.8125
4/20/2019 6:31	0.7	-0.3417	4.4663	-0.4667	2.3418	31.4	0.1254	1.7646
4/20/2019 6:32	0.7	-0.35	4.5447	0.2	2.3528	31.3727	0.1464	1.7933
4/20/2019 6:33	0.7	-1.0333	4.4563	-0.5333	2.3376	31.3	0.1261	1.7902
4/20/2019 6:34	0.6333	0.2917	4.4251	-0.4333	2.3591	31.3	0.1413	1.8077
4/20/2019 6:35	0.6455	0.2333	4.2678	-0.5	2.3761	31.3	0.1343	1.7875
4/20/2019 6:36	0.6167	-0.45	4.2707	0.0167	2.3982	31.2	0.1359	1.7682
4/20/2019 6:37	0.6	-0.75	4.4191	0.0333	2.3851	31.2273	0.1427	1.7773
4/20/2019 6:38	0.6	-0.3417	4.4646	0.2167	2.3883	31.2	0.1304	1.7868
4/20/2019 6:39	0.65	-0.2	4.4663	-0.2	2.3579	31.2	0.1439	1.7578
4/20/2019 6:40	0.7	-0.6583	4.4209	-0.8	2.3853	31.175	0.1274	1.7319
4/20/2019 6:41	0.7	0.0833	4.5979	-0.3167	2.3592	31.1545	0.1408	1.7474
4/20/2019 6:42	0.7	0.5667	4.4163	-0.4167	2.3512	31.1	0.1392	1.7373
4/20/2019 6:43	0.7	0.1167	4.4849	-0.25	2.3526	30.97	0.1373	1.7616
4/20/2019 6:44	0.7	-0.125	4.4671	-0.3	2.3681	30.8909	0.1462	1.7668
4/20/2019 6:45	0.7	-0.3333	4.5446	-0.95	2.376	30.8	0.1139	1.7603
4/20/2019 6:46	0.7	-0.4083	4.8023	-0.1333	2.371	30.9	0.1357	1.7467
4/20/2019 6:47	0.7	-0.6	4.3392	0.1333	2.376	30.9091	0.1402	1.7487
4/20/2019 6:48	0.7	0.2583	4.3433	-0.05	2.3807	31	0.1429	1.7787
4/20/2019 6:49	0.7	-0.1083	4.6033	-0.2667	2.3668	30.97	0.1411	1.7948
4/20/2019 6:50	0.7	-0.4	4.4709	-0.2	2.3942	30.93	0.1355	1.7989

4/20/2019 6:51	0.7	-0.2667	4.4421	-0.2333	2.3715	30.9636	0.1466	1.807
4/20/2019 6:52	0.7	0.275	4.5398	-0.3	2.371	31	0.1284	1.794
4/20/2019 6:53	0.7	-0.175	4.6232	-0.2167	2.373	31	0.1354	1.7659
4/20/2019 6:54	0.7	0.1833	4.4269	0.1333	2.3853	31.0091	0.1246	1.807
4/20/2019 6:55	0.7	0.5917	4.3633	-0.3167	2.3908	31.12	0.1381	1.8146
4/20/2019 6:56	0.7	-0.0667	4.3528	-0.7833	2.3669	31.28	0.1424	1.8058
4/20/2019 6:57	0.7	-0.7917	4.6333	-0.3667	2.3878	31.4	0.1298	1.7846
4/20/2019 6:58	0.7	-0.0833	4.7007	-0.4	2.3761	31.32	0.14	1.8058
4/20/2019 6:59	0.7	0.6917	4.5742	-0.85	2.3825	31.37	0.1344	1.8027
4/20/2019 7:00	0.7	-0.6167	4.7316	-0.2667	2.3977	31.4	0.1349	1.8093
4/20/2019 7:01	0.7	-1.0833	4.5068	0.05	2.4068	31.46	0.1456	1.8178
4/20/2019 7:02	0.7	-0.75	4.5738	-0.8167	2.4062	31.5	0.1355	1.8113
4/20/2019 7:03	0.7	0.325	4.5132	0.05	2.3925	31.5	0.1497	1.7878
4/20/2019 7:04	0.7	0.2417	4.6215	-0.4	2.4017	31.5083	0.1321	1.777
4/20/2019 7:05	0.7	-0.12	4.4288	-0.64	2.405	31.6	0.1389	1.7598
4/20/2019 7:06	0.6583	-0.0167	4.5511	-0.45	2.4227	31.6	0.1399	1.7596
4/20/2019 7:07	0.7	-0.1833	4.5487	-0.45	2.4631	31.6	0.1333	1.7663
4/20/2019 7:08	0.7	-0.26	4.3526	0.04	2.423	31.41	0.1503	1.7926
4/20/2019 7:09	0.7	-1.1167	4.3854	-0.4167	2.4377	31.0273	0.1272	1.8057
4/20/2019 7:10	0.7	0.5583	4.5258	-0.1667	2.4448	30.9909	0.1432	1.8138
4/20/2019 7:11	0.6917	0.6	4.6163	-0.5333	2.443	30.9	0.1379	1.8119
4/20/2019 7:12	0.7	-0.3833	4.732	-0.1167	2.4573	30.8	0.1319	1.8486
4/20/2019 7:13	0.7	0.0917	4.2986	-0.3333	2.4601	30.6545	0.1429	1.8574
4/20/2019 7:14	0.7667	-0.5583	4.3721	-0.5167	2.443	30.56	0.1306	1.8339
4/20/2019 7:15	0.8	-0.3	4.4453	-0.25	2.4419	30.2546	0.1435	1.8366
4/20/2019 7:16	0.8083	-0.0917	4.4981	-0.35	2.4338	29.8182	0.1352	1.8051
4/20/2019 7:17	0.8	-0.3	4.2823	-0.5333	2.4503	28.5273	0.1384	1.8311
4/20/2019 7:18	0.8	-0.7167	4.3405	-0.5	2.4347	26.85	0.1423	1.8723
4/20/2019 7:19	0.8	-0.2	4.4974	-0.1167	2.4405	24.9636	0.1267	1.8723
4/20/2019 7:20	0.8167	-0.3417	4.3756	-0.3167	2.4336	23.2273	0.1469	1.8513
4/20/2019 7:21	0.8667	-0.0667	4.3181	-0.4167	2.434	21.5818	0.1334	1.8813
4/20/2019 7:22	0.8	-0.05	4.5549	-0.5	2.4338	19.9	0.1373	1.8652
4/20/2019 7:23	0.8333	-1	4.3735	-0.5	2.4172	18.0182	0.1497	1.8574
4/20/2019 7:24	0.8	-0.7167	4.5662	-0.8333	2.4458	16.23	0.1313	1.8578
4/20/2019 7:25	0.8	-0.2583	4.4315	-0.5333	2.4493	14.2546	0.1479	1.8553
4/20/2019 7:26	0.8	-0.6917	4.359	-0.85	2.4548	12.4546	0.1278	1.8973
4/20/2019 7:27	0.8	0.0833	4.3431	-1.15	2.4781	10.6909	0.1446	1.8953
4/20/2019 7:28	0.8	-0.45	4.2437	0.2	2.4582	9.27	0.1378	1.8985
4/20/2019 7:29	0.8	-0.8667	4.5423	0.1333	2.4364	8.2545	0.1348	1.8893
4/20/2019 7:30	0.7083	-0.3167	4.4541	-0.2833	2.4196	8.0727	0.148	1.8768
4/20/2019 7:31	0.7	-0.95	4.5167	-0.6667	2.4343	7.9	0.1326	1.8982
4/20/2019 7:32	0.7	-0.4667	4.4105	-0.2167	3.2274	8.0818	0.153	1.9106
4/20/2019 7:33	0.6417	-0.5417	6.2947	0.2333	8.3419	9.4583	0.2246	2.0504
4/20/2019 7:34	0.6167	0.1333	10.4369	0.6667	15.861	23.08	0.377	2.1413
4/20/2019 7:35	0.6167	-0.2417	14.6725	2.0833	19.027	55.5636	0.5155	2.2207
4/20/2019 7:36	0.6333	-0.2167	15.9392	1.8333	19.5575	58.75	1.2155	2.2165
4/20/2019 7:37	0.6	0.05	17.1133	2.75	20.1046	63.9	0.9971	2.0815

4/20/2019 7:38	0.6	-0.4333	18.265	2.7	20.3269	66.5583	1.0237	2.0798
4/20/2019 7:39	0.6	0.4333	17.6225	2.1667	19.3141	62.55	0.8663	2.0828
4/20/2019 7:40	0.5667	0.4833	16.8358	2.2833	18.6261	60.29	0.824	2.0792
4/20/2019 7:41	0.525	-0.2667	16.7958	2.8167	18.5479	60.2	0.8063	2.084
4/20/2019 7:42	0.575	-0.2083	16.7525	1.9833	18.4059	60.3917	0.824	2.0862
4/20/2019 7:43	0.5917	0.0917	16.345	2.9	18.1991	59.9364	0.7824	2.1135
4/20/2019 7:44	0.6	-0.3917	15.3633	2.2167	17.3444	58.1167	0.7578	2.12
4/20/2019 7:45	0.6	-0.225	15.5825	2.6833	17.4035	55.8167	0.7444	2.096
4/20/2019 7:46	0.6167	0.2167	15.6042	2.0833	17.2247	57.3818	0.7469	2.114
4/20/2019 7:47	0.675	0.7167	15.4433	1.9333	16.9703	57.2917	0.75	2.0732
4/20/2019 7:48	0.7	-0.525	14.7917	1.8333	16.4561	57.1545	0.7041	2.0642
4/20/2019 7:49	0.7	0.3417	14.9758	1.8333	16.5106	57.1	0.7345	2.0942
4/20/2019 7:50	0.7	-0.5833	14.5667	1.9333	16.4287	57.0583	0.7183	2.1002
4/20/2019 7:51	0.7	0.3	14.3567	1.2333	16.0487	56.9167	0.6988	2.0898
4/20/2019 7:52	0.7	0.3167	13.8392	1.95	15.3596	56.5636	0.6972	2.0858
4/20/2019 7:53	0.6833	0.15	13.9025	2.2833	15.5026	56.2167	0.6898	2.1029
4/20/2019 7:54	0.7	0.575	13.5492	1.7833	15.2698	55.9	0.6962	2.0989
4/20/2019 7:55	0.7	0.4667	13.6742	1.6	15.211	55.6818	0.6894	2.1071
4/20/2019 7:56	0.7	0.675	13.3925	2.1	14.9086	55.4167	0.6868	2.1033
4/20/2019 7:57	0.7	0.7583	13.4317	1.85	14.8241	55.0909	0.6921	2.1058
4/20/2019 7:58	0.7	0.0833	13.0883	1.75	14.2979	54.7083	0.6515	2.1362
4/20/2019 7:59	0.7	0.8417	12.675	1.3	14.2122	54.39	0.6803	2.1373
4/20/2019 8:00	0.7	0.0667	12.955	1.7833	14.0915	54	0.6564	2.15
4/20/2019 8:01	0.7	0.0667	12.645	2.3167	13.8529	53.64	0.6712	2.1198
4/20/2019 8:02	0.7	-0.25	12.4692	1.7	13.5796	53.2	0.6601	2.1038
4/20/2019 8:03	0.6583	0.4917	12.1708	1.9333	13.4093	52.71	0.6566	2.1307
4/20/2019 8:04	0.6	0.225	12.1517	2.15	13.2513	52.375	0.6526	2.0908
4/20/2019 8:05	0.5917	-0.3083	12.0383	1.3667	12.9588	52.0273	0.6272	2.0932
4/20/2019 8:06	0.5917	0.4083	11.9967	1.8833	13.0172	51.7182	0.6505	2.108
4/20/2019 8:07	0.575	0.1583	11.6867	1.65	12.7989	51.425	0.6314	2.0865
4/20/2019 8:08	0.6	0.3583	11.6883	1.9833	12.6947	50.9727	0.6282	2.0773
4/20/2019 8:09	0.6	-0.3167	11.7467	1.5667	12.6007	50.65	0.7004	2.0807
4/20/2019 8:10	0.6	-0.4583	11.3733	1.1833	12.4349	50.39	0.6945	2.1031
4/20/2019 8:11	0.6	0.7417	11.545	0.9833	12.2358	50.1	0.7113	2.0888
4/20/2019 8:12	0.6	0.5417	11.2067	1.4167	12.1909	49.8636	0.6862	2.0808
4/20/2019 8:13	0.6	-0.075	11.0517	1.2833	12.0632	49.5636	0.6963	2.0704
4/20/2019 8:14	0.6	-0.6167	11.0967	1.5833	11.8747	49.2667	0.6905	2.1066
4/20/2019 8:15	0.6	-0.1	11	1.1667	11.6536	49.0546	0.6705	2.1122
4/20/2019 8:16	0.6	-0.2167	11.0533	1.1	11.3455	48.8	0.6767	2.141
4/20/2019 8:17	0.5833	0.1417	10.6317	1.1833	11.4182	48.76	0.6634	2.1182
4/20/2019 8:18	0.5	0.3	10.6283	1.1667	11.0908	48.4417	0.6595	2.1256
4/20/2019 8:19	0.5	0.075	10.5633	1.2	10.9433	48.0909	0.6549	2.1283
4/20/2019 8:20	0.5	-0.0333	10.4217	1.1833	10.9134	47.9636	0.6565	2.1368
4/20/2019 8:21	0.5	-0.2583	10.23	1.2167	10.7227	47.67	0.6563	2.1475
4/20/2019 8:22	0.5417	-0.5667	10.3733	1.1667	10.701	47.3167	0.6395	2.0841
4/20/2019 8:23	0.5	0.175	10.3567	1.3333	10.6725	46.9091	0.6408	2.0755
4/20/2019 8:24	0.5	0.6083	10.0907	0.95	10.4296	46.6417	0.6367	2.0631

4/20/2019 8:25	0.5	-0.3583	9.967	1.6167	10.2966	46.3	0.6337	2.0818
4/20/2019 8:26	0.5417	-0.1917	9.9052	1.5333	10.1168	46.05	0.6353	2.1283
4/20/2019 8:27	0.55	0.3917	9.8048	0.9333	9.9935	45.73	0.6297	2.1051
4/20/2019 8:28	0.5	-0.075	10.0585	1.2167	10.0322	45.6333	0.6314	2.1085
4/20/2019 8:29	0.5	-0.2083	9.7957	0.7833	9.8751	45.35	0.6359	2.1088
4/20/2019 8:30	0.4333	0.575	9.6645	0.6333	9.6523	45.1	0.6222	2.1253
4/20/2019 8:31	0.4	-0.6333	9.8735	0.6833	9.6013	44.9182	0.6224	2.1495
4/20/2019 8:32	0.4	0.3333	9.4947	0.8833	9.3714	44.7	0.6055	2.1331
4/20/2019 8:33	0.4	0.95	9.4915	1.1833	9.2777	44.3	0.6148	2.1018
4/20/2019 8:34	0.4	0.1417	9.5833	1.35	9.1673	44.0417	0.6082	2.1415
4/20/2019 8:35	0.4	0.1833	9.286	0.8333	9.0355	43.8364	0.5951	2.1298
4/20/2019 8:36	0.4	-0.1417	9.1068	1.25	8.9085	43.62	0.607	2.1433
4/20/2019 8:37	0.4	-0.025	9.3087	0.8833	8.8502	43.425	0.592	2.1427
4/20/2019 8:38	0.4	-0.0833	9.0912	0.55	8.7542	43.1818	0.5986	2.0993
4/20/2019 8:39	0.45	-0.125	9.5707	0.7333	8.7442	42.9583	0.6067	2.0873
4/20/2019 8:40	0.5	0.125	9.1252	0.1833	8.6559	43.0273	0.5923	2.1203
4/20/2019 8:41	0.5	-0.1417	8.9165	0.8667	8.4978	43.3083	0.5989	2.1188
4/20/2019 8:42	0.5	-0.05	8.8587	0.65	8.3648	43.0455	0.5789	2.1328
4/20/2019 8:43	0.5	-0.9167	8.8357	0.4167	8.1988	42.725	0.5745	2.1214
4/20/2019 8:44	0.5	-0.1667	8.896	0.6333	8.2748	42.5182	0.5948	2.105
4/20/2019 8:45	0.6	-0.1917	8.6843	0.35	8.0969	42.325	0.575	2.0878
4/20/2019 8:46	0.6	-0.475	8.5985	1.3	7.9417	42.1455	0.592	2.099
4/20/2019 8:47	0.6	-0.2667	8.5368	0.7667	7.9063	41.9083	0.5737	2.1148
4/20/2019 8:48	0.5	0.3083	8.6805	0.8667	7.867	41.6818	0.5718	2.0996
4/20/2019 8:49	0.5	0.025	8.3625	0.5667	7.7349	41.4455	0.5714	2.0983
4/20/2019 8:50	0.5	0.2333	8.302	1.0333	7.5983	41.1333	0.5515	2.1042
4/20/2019 8:51	0.575	0.1917	8.173	0.9167	7.4419	40.7727	0.5546	2.1137
4/20/2019 8:52	0.6	0.5583	8.2065	0.8167	7.4374	40.3727	0.5512	2.0992
4/20/2019 8:53	0.6	-0.4667	8.2167	1.0667	7.4071	39.4083	0.548	2.0842
4/20/2019 8:54	0.6	0.2167	8.2378	0.5833	7.3782	39.1909	0.5393	2.0962
4/20/2019 8:55	0.6	0.3333	8.0388	0.5667	7.2006	38.9333	0.487	2.1238
4/20/2019 8:56	0.575	-0.35	8.0295	1.4667	7.1505	38.5833	0.4891	2.1189
4/20/2019 8:57	0.6167	0.1083	7.957	1.2833	7.501	38.1545	0.4666	1.8898
4/20/2019 8:58	0.85	1.175	7.6493	1.45	8.1169	37.1818	0.4294	1.7413
4/20/2019 8:59	1.125	1.1	7.5508	1.8	8.6122	36.275	0.4066	1.7303
4/20/2019 9:00	1.4917	1.6583	7.3072	1.65	9.0239	35.4167	0.3784	1.6719
4/20/2019 9:01	1.9417	3.2667	7.4913	2.7833	9.3265	35.0273	0.334	1.5926
4/20/2019 9:02	2.35	2.2083	7.3997	3.2167	9.8501	34.1636	0.3385	1.5744
4/20/2019 9:03	2.65	2.4917	7.0665	2.8667	10.4463	33.4667	0.329	1.5788
4/20/2019 9:04	2.525	1.6167	6.2698	2.4833	10.8653	32.9909	0.3343	1.5808
4/20/2019 9:05	2.4417	2.025	6.438	2.3333	11.4079	32.35	0.3227	1.5304
4/20/2019 9:06	2.4917	2.7667	6.4117	3.0333	11.7061	32.0455	0.3042	1.5684
4/20/2019 9:07	2.7333	3.1083	6.6637	3.45	11.8817	31.5833	0.2879	1.5883
4/20/2019 9:08	3.05	2.7167	6.6668	3.3833	11.6798	31.2	0.269	1.5251
4/20/2019 9:09	3.2917	3.55	6.7238	3.4833	11.7836	30.2833	0.2839	1.4635
4/20/2019 9:10	3.5917	4.1167	7.1638	3.7333	11.8629	29.4364	0.2828	1.427
4/20/2019 9:11	3.8583	4.1583	7.0932	4.25	11.9722	28.5455	0.2755	1.4464

4/20/2019 9:12	4.15	4.5833	7.6678	4.25	12.1281	28.0445	0.2805	1.415
4/20/2019 9:13	4.3667	4.475	7.4862	4.75	12.1304	27.1273	0.2536	1.4366
4/20/2019 9:14	4.675	5.0083	8.021	5.3833	11.9252	26.2909	0.2362	1.4447
4/20/2019 9:15	4.9	5.4	7.8793	6.0667	11.7783	26.1	0.2365	1.4263
4/20/2019 9:16	5.1417	5.0333	8.0432	5.95	11.6437	25.0455	0.2088	1.4543
4/20/2019 9:17	5.3333	5.3083	8.0315	5.8833	11.47	24.58	0.2115	1.4233
4/20/2019 9:18	5.65	6.7167	8.9228	7.5667	10.8677	23.75	0.211	1.4085
4/20/2019 9:19	5.975	6.5	8.758	6.8833	10.6161	22.85	0.2021	1.4013
4/20/2019 9:20	6.3417	7.4417	8.9722	7.4	10.3326	21.6667	0.2121	1.4046
4/20/2019 9:21	6.5167	6.8583	8.9535	7.2667	10.35	20.96	0.2144	1.3855
4/20/2019 9:22	6.8083	7.175	9.2102	7.2833	10.0785	20.8636	0.1813	1.396
4/20/2019 9:23	7.1167	7.55	9.7528	8.1833	10.035	20.6455	0.1913	1.3695
4/20/2019 9:24	7.5333	8.3667	10.0007	8.5833	9.9577	20.2417	0.1832	1.3832
4/20/2019 9:25	7.975	9.2083	10.2113	8.5333	9.1305	19.6545	0.161	1.3996
4/20/2019 9:26	8.275	9.9333	10.985	9.6167	8.6925	19.1667	0.1773	1.3868
4/20/2019 9:27	8.6917	9.5833	11.005	10.15	8.2419	18.6333	0.1614	1.3608
4/20/2019 9:28	8.9583	9.1	10.975	9.1	8.2273	18.2818	0.1556	1.3651
4/20/2019 9:29	9.275	10.0833	11.6717	10.3667	7.8259	17.5333	0.166	1.3579
4/20/2019 9:30	9.5083	9.9083	11.6083	10.3	7.6598	16.7333	0.1478	1.3763
4/20/2019 9:31	9.6833	9.8167	11.495	10.1167	7.6541	16.225	0.157	1.3818
4/20/2019 9:32	9.7	9.8583	11.4033	9.9167	7.5105	15.8909	0.1666	1.4253
4/20/2019 9:33	9.4667	9.6	11.1617	9.5667	7.5877	15.6083	0.1611	1.4281
4/20/2019 9:34	9.1917	8.5667	10.845	9.0333	7.5413	15.2417	0.1682	1.4181
4/20/2019 9:35	8.8833	8.5917	10.4625	8.4833	7.5425	14.9167	0.1699	1.4066
4/20/2019 9:36	8.5917	8.7667	10.2275	8.5333	7.4998	14.77	0.1593	1.4412
4/20/2019 9:37	8.3833		9.8118	8.6333	7.5172	14.6364	0.1669	1.4292
4/20/2019 9:38	8.1417		9.7168	8	7.4708	14.625	0.1675	1.4343
4/20/2019 9:39	7.9417		9.4882	7.7167	7.4442	14.5455	0.1597	1.42
4/20/2019 9:40	7.6833		9.3533	7.2	7.4259	14.325	0.1749	1.4202
4/20/2019 9:41	7.4583		9.2207	7.15	7.4097	14.2818	0.1676	1.4353
4/20/2019 9:42	7.1917		9.0427	6.4333	7.3788	14.5546	0.1675	1.426
4/20/2019 9:43	6.9833		8.7557	7.4333	7.3367	14.4455	0.1759	1.4353
4/20/2019 9:44	6.825		8.8442	6.25	7.3152	14.6083	0.1581	1.4779
4/20/2019 9:45	6.6583		8.729	6.1	7.3058	14.6727	0.175	1.4988
4/20/2019 9:46	6.4818		8.5959	6.4167	7.2689	14.9167	0.177	1.5
4/20/2019 9:47	6.2583		8.098	5.9333	8.2103	15.4818	0.1799	1.5123
4/20/2019 9:48	5.7333	8.4167	11.9995	5.8833	11.5518	16.2364	0.2314	1.6909
4/20/2019 9:49	4.8667	9.0083	25.93	9.0333	24.8806	17.78	0.3604	1.9832
4/20/2019 9:50	3.5917	9.6667	45.8017	14	35.3703	31.4546	0.5707	1.98
4/20/2019 9:51	2.325	8.7	70.8525	19.9167	47.7448	76.2	0.9103	2.1606
4/20/2019 9:52	1.2833	10.3667	94.4867	26.4333	53.9497	96.4364	1.1791	2.2818
4/20/2019 9:53	0.7167	6.1583	110.4833	29.5	58.4774	113.2833	1.5385	2.4584
4/20/2019 9:54	0.5167	5.8583	146.8417	38.45	61.5984	138	3.7405	2.4818
4/20/2019 9:55	0.4833	7.25	156.4333	38.7167	59.7995	154.6917	4.2258	2.5347
4/20/2019 9:56	0.5	8.3667	137.9667	34.5167	58.4803	154.6909	3.5436	2.5297
4/20/2019 9:57	0.5	10.0833	134.7667	34.45	57.9718	155.2364	3.8027	2.523
4/20/2019 9:58	0.5	4.9333	125.5917	32.9167	56.7471	148.9182	3.2849	2.513

4/20/2019 9:59	0.5417	4.0571	120.45	32.0167	55.7309	151.1455	3.1614	2.5061
4/20/2019 10:00	0.5	4.3909	125.1083	33.0333	55.4703	154.1182	3.5884	2.4993
4/20/2019 10:01	0.5	4.8667	119.3	31.25	54.4693	147.15	3.3658	2.4915
4/20/2019 10:02	0.5083	4.5	120.15	31.45	53.7625	136.5636	3.5473	2.4824
4/20/2019 10:03	0.6	4.1667	108.7167	30.05	52.5603	138.1417	3.064	2.5117
4/20/2019 10:04	0.6	4.25	103.4083	30.1667	51.6147	144.79	2.7643	2.494
4/20/2019 10:05	0.5833	3.9333	102.9833	29.6667	51.4873	134.0455	2.8123	2.472
4/20/2019 10:06	0.5	4.4167	98.9417	29.2667	50.5873	133.525	2.6418	2.5145
4/20/2019 10:07	0.5	4.1	96.7875	29.0167	49.7227	128.9	2.5836	2.5051
4/20/2019 10:08	0.5167	3.7167	93.5317	28.3333	48.9108	135.95	2.5804	2.4844
4/20/2019 10:09	0.6	4.4833	92.0217	27.4667	48.4924	133.375	2.593	2.4795
4/20/2019 10:10	0.6	4.0167	87.3875	27.1	46.3048	119.1917	2.4815	2.4718
4/20/2019 10:11	0.6	3.6	85.13	27.0167	45.2563	110.9083	2.3878	2.4618
4/20/2019 10:12	0.5667	3.8333	85.0808	26.3667	45.2091	113.0667	2.4458	2.4905
4/20/2019 10:13	0.5	4.1167	83.5042	25.8667	44.4765	112.9545	2.44	2.491
4/20/2019 10:14	0.5	3.8667	82.8367	25.5833	44.1287	115	2.443	2.4486
4/20/2019 10:15	0.5167	3.7167	80.9967	25.3667	43.1289	117.4583	2.4053	2.4433
4/20/2019 10:16	0.6	3.9167	80.2758	24.65	42.8713	117.3583	2.3477	2.4266
4/20/2019 10:17	0.6	3.5	80.0483	25.1	42.5519	116.2091	2.4012	2.4296
4/20/2019 10:18	0.6	3.45	78.0117	24.7	41.6078	115.6818	2.3752	2.4588
4/20/2019 10:19	0.6	3.5167	75.1783	24.2	40.3578	115.1545	2.337	2.46
4/20/2019 10:20	0.5833	3.05	74.955	23.95	39.8403	114.2636	2.3148	2.4742
4/20/2019 10:21	0.6	3.3167	74.82	23.4333	39.7208	113.3167	2.314	2.4737
4/20/2019 10:22	0.6	3.6167	74.1275	23.1833	39.5298	112.7182	2.3347	2.4533
4/20/2019 10:23	0.6	4.0667	73.9967	22.95	39.3568	112.25	2.32	2.4625
4/20/2019 10:24	0.6583	3.1667	73.2642	23.1167	39.203	111.9	2.3018	2.4443
4/20/2019 10:25	0.7	3.45	70.7	22.4	39.1521	110.97	2.2483	2.4518
4/20/2019 10:26	0.7	3.1	69.8417	22.4833	37.3417	109.8417	2.2237	2.4268
4/20/2019 10:27	0.7	3.4833	68.2783	21.7	36.1853	109.5444	2.179	2.4217
4/20/2019 10:28	0.7	3.2833	67.7583	21.0333	35.839	107.9583	2.2047	2.4337
4/20/2019 10:29	0.6333	2.75	66.825	22.1333	35.2197	107.2909	2.2185	2.4465
4/20/2019 10:30	0.6	3.0333	64.4583	20.7833	34.196	108.0917	2.1885	2.4502
4/20/2019 10:31	0.6	3.1667	64.6683	21	34.1752	108.51	2.1435	2.4819
4/20/2019 10:32	0.6	3.1167	64.6742	20.7167	33.9628	109.1167	2.175	2.4593
4/20/2019 10:33	0.6	3.3333	64.5725	20.5333	33.9118	108.8727	2.1775	2.4439
4/20/2019 10:34	0.6	3.0333	62.6117	19.7167	32.9917	109.475	2.1308	2.4618
4/20/2019 10:35	0.6	2.0833	61.4083	19.6833	32.2288	109.32	2.1997	2.4137
4/20/2019 10:36	0.6	3.2333	60.7958	19.45	31.7395	108.5167	2.1495	2.4351
4/20/2019 10:37	0.6333	2.3833	59.4467	19.2833	31.3543	108.1333	2.0495	2.4709
4/20/2019 10:38	0.7	3.6833	59.3925	18.9167	31.1403	108.4667	2.0575	2.4408
4/20/2019 10:39	0.6417	2.85	59.4267	19.3833	30.6893	107.2545	2.046	2.4353
4/20/2019 10:40	0.6	2.8667	58.9458	18.6667	30.2942	108.15	2.0718	2.428
4/20/2019 10:41	0.6	2.25	57.6525	18.15	29.8718	108.4818	2.0067	2.4322
4/20/2019 10:42	0.6	2.1333	56.7425	18.2	29.2039	107.725	2.0298	2.4352
4/20/2019 10:43	0.5833	2.4333	57.0483	18.1333	29.2723	107.3167	2.108	2.3866
4/20/2019 10:44	0.575	2.2333	55.8725	18.0833	28.5583	106.5273	2.049	2.3762
4/20/2019 10:45	0.5833	2.4333	55.4067	18.05	28.3411	106.9417	2.01	2.4165

4/20/2019 10:46	0.5167	2.9833	54.9125	17.8333	27.831	107.1818	1.9948	2.4343
4/20/2019 10:47	0.525	1.6833	54.6208	17.4333	27.7559	106.8	1.9253	2.4073
4/20/2019 10:48	0.5	2.2833	54.1858	16.6333	27.4554	106.9333	1.9848	2.4207
4/20/2019 10:49	0.5	2.2667	53.5883	16.45	27.0331	107.1333	2.0025	2.4503
4/20/2019 10:50	0.5	2.1333	52.8258	16.4167	26.6138	107.225	1.982	2.4111
4/20/2019 10:51	0.5	2.2167	52.0325	16.8833	26.0457	108.0667	1.902	2.4293
4/20/2019 10:52	0.5	2.4167	52.1017	16.4	25.9621	107.925	1.9315	2.4303
4/20/2019 10:53	0.4667	2.6167	50.22	16.2833	25.1356	107.5167	1.9477	2.4243
4/20/2019 10:54	0.5	2.15	50.9742	16.55	25.322	107.6111	1.9122	2.4243
4/20/2019 10:55	0.5	2	49.0492	16.0833	24.3388	106.6167	1.9162	2.421
4/20/2019 10:56	0.5	1.6833	49.3367	15.3667	24.3311	105.5571	1.8795	2.474
4/20/2019 10:57	0.4917	1.95	48.4408	15.1833	23.8572	104.8286	1.8812	2.4304
4/20/2019 10:58	0.5	2.35	48.435	15.1833	23.6889	104.0455	1.8973	2.3922
4/20/2019 10:59	0.5	1.8333	46.8242	15.1667	22.9688	103.1333	1.7835	2.4
4/20/2019 11:00	0.6	2.2333	48.08	14.9	23.3812	101.62	1.8562	2.3984
4/20/2019 11:01	0.6	1.8833	46.9908	14.5167	22.8531	101.275	1.8047	2.3854
4/20/2019 11:02	0.6	1.5667	46.8258	14.8167	22.6832	100.3167	1.82	2.355
4/20/2019 11:03	0.6	2.0167	46.01	14.1333	22.2017	84.275	1.8263	2.3636
4/20/2019 11:04	0.5583	0.9333	46.18	14.7667	22.311	80.6364	1.8603	2.3906
4/20/2019 11:05	0.5	1.6167	45.0483	13.8833	21.6335	98.7273	1.8468	2.4377
4/20/2019 11:06	0.5167	2.5833	43.5017	13.5	20.9735	98.625	1.768	2.4313
4/20/2019 11:07	0.5333	1.6833	44.2083	14.0167	21.1353	82.4364	1.7388	2.4088
4/20/2019 11:08	0.5	1.9833	44.1167	14.1	21.0127	58.48	1.7417	2.415
4/20/2019 11:09	0.5	1.1333	43.6617	13.6333	20.6615	62.5182	1.6595	2.44
4/20/2019 11:10	0.5083	1.4333	40.005	12.1833	19.2058	59.9364	1.6055	2.4198
4/20/2019 11:11	0.6	1.3667	37.6167	11.9	18.5049	62.2667	1.5022	2.443
4/20/2019 11:12	0.6	1.3333	38.3067	11.85	19.4863	58.6455	1.4948	2.2991
4/20/2019 11:13	0.6917	1.4	34.0067	10.05	19.6686	62.5667	1.3972	2.1009
4/20/2019 11:14	0.8417	2.6167	29.7283	9.6833	20.2384	76.2818	1.2402	1.9428
4/20/2019 11:15	1.0583	1.8833	27.23	9.65	20.4194	72.03	1.1473	1.889
4/20/2019 11:16	1.4	3.6	24.0333	9.9833	20.3872	71.3182	1.0291	1.7751
4/20/2019 11:17	1.7417	2.8833	22.135	9.3167	20.1763	70.6546	0.9475	1.8334
4/20/2019 11:18	2.15	4.0667	20.07	9.2667	19.8628	69.8083	0.8799	1.8552
4/20/2019 11:19	2.55	4.3333	18.125	9.1667	19.5372	68.55	0.7905	1.8138
4/20/2019 11:20	3.0167	5.05	17.11	9.75	19.1268	66.8364	0.7447	1.7971
4/20/2019 11:21	3.4667	5.5667	15.8467	9.05	18.6144	64.5273	0.6884	1.7854
4/20/2019 11:22	4.175	7.25	15.2433	9.8	17.1402	62.4	0.6289	1.7432
4/20/2019 11:23	4.7667	7.0333	14.685	9.8667	16.858	60.24	0.616	1.6934
4/20/2019 11:24	5.2917	7.4167	14.5817	9.85	16.4216	57.8583	0.5734	1.6827
4/20/2019 11:25	5.6917	7.55	14.6483	10.7667	15.97	55.2727	0.5223	1.6535
4/20/2019 11:26	6.1833	9.2167	14.1033	11.0833	15.9474	52.6917	0.4976	1.6297
4/20/2019 11:27	6.9	9.8	14.05	12.1167	13.926	49.8083	0.4427	1.575
4/20/2019 11:28	7.425	10.05	14.3833	12.2833	12.6968	47.1727	0.4451	1.5341
4/20/2019 11:29	7.9917	10.2	14.47	12.2833	11.8497	44.55	0.4141	1.4997
4/20/2019 11:30	8.3167	10.8833	14.4267	12	11.3888	42.2364	0.4003	1.5097
4/20/2019 11:31	8.825	11.8333	14.5867	13.8	10.6453	40.2917	0.3912	1.4998
4/20/2019 11:32	9.4583	12.6833	14.6917	13.9833	9.7894	38.4909	0.3495	1.4755

4/20/2019 11:33	9.9583	12.2667	15.075	14.0667	9.5953	37.35	0.3523	1.4603
4/20/2019 11:34	10.5167	13.65	15.6867	15.25	8.8595	36.11	0.3222	1.4475
4/20/2019 11:35	11.0333	15.3	15.835	15.95	8.334	34.9273	0.2958	1.4734
4/20/2019 11:36	11.775	14.7333	16.1617	15.8667	7.7408	33.525	0.2985	1.4295
4/20/2019 11:37	12.4167	15.6333	16.58	16.3167	7.2573	32.4182	0.2718	1.4243
4/20/2019 11:38	12.875	15.7167	16.9833	16.65	6.9331	31.2417	0.2614	1.4466
4/20/2019 11:39	13.2667	16.3667	17.525	17.0333	6.6181	30.35	0.2634	1.4388
4/20/2019 11:40	13.6333	17.45	17.2533	17.3333	6.189	29.35	0.2363	1.4387
4/20/2019 11:41	14.15	17.3167	17.695	18.4	5.8495	28.42	0.2362	1.4311
4/20/2019 11:42	14.6583	17.1833	18.51	18.6167	5.6183	27.5417	0.2274	1.4103
4/20/2019 11:43	15.1	17.1167	18.2317	18.4	5.3893	26.7364	0.2111	1.4148
4/20/2019 11:44	15.3583	17.2167	18.5317	18.15	5.2266	26.0667	0.2266	1.4077
4/20/2019 11:45	15.6417	18.2333	18.6933	19.0667	4.991	25.4273	0.1993	1.373
4/20/2019 11:46	15.8917	18.65	18.965	18.9833	4.8371	24.7546	0.2003	1.3718
4/20/2019 11:47	16.25	18.75	18.8183	19.3167	4.6966	23.9273	0.2056	1.3678
4/20/2019 11:48	16.4333	18.7	19.1283	19.5	4.5846	23.3167	0.1823	1.3768
4/20/2019 11:49	16.65	19.4	19.26	19.2833	4.4157	22.7636	0.1919	1.3963
4/20/2019 11:50	16.85	19.0667	19.4317	19.6833	4.2615	21.9727	0.1766	1.3731
4/20/2019 11:51	16.8667	18.7167	18.6467	18.85	4.2502	21.3667	0.175	1.3479
4/20/2019 11:52	16.9917	19.3833	19.255	18.9167	4.0567	20.7818	0.1857	1.331
4/20/2019 11:53	17.0083	18.85	19.505	19.1833	3.9807	20.0182	0.1662	1.3403
4/20/2019 11:54	17.2	18.7	19.52	19.9	3.8799	19.2364	0.1625	1.346
4/20/2019 11:55	17.425	19.4667	19.5867	19.55	3.8063	18.5917	0.1732	1.3468
4/20/2019 11:56	17.775	19.6167	19.9283	20.15	3.7403	17.8727	0.1534	1.3371
4/20/2019 11:57	17.9917	19.7833	20.04	20.05	3.7401	17.0333	0.1716	1.3319
4/20/2019 11:58	18.15	19.3833	20.09	20.6	3.7113	16.4091	0.1658	1.2768
4/20/2019 11:59	18.375	20.1833	20.3467	20.7667	3.6559	16.1727	0.1504	1.3483
4/20/2019 12:00	18.575	20.2333	20.4367	20.7833	3.619	16.1167	0.1514	1.5057
4/20/2019 12:01	18.5333	19.5167	19.67	19.3	3.6209	16.075	0.1627	1.3346
4/20/2019 12:02	18.3917	20.8667	20.29	20.1	3.5997	16.225	0.1508	1.3327
4/20/2019 12:03	18.3417	19.9667	20.4283	20.5333	3.5753	16.2667	0.1575	1.3235
4/20/2019 12:04	18.45	19.75	20.4283	20.5167	3.5563	16.1833	0.1418	1.3181
4/20/2019 12:05	18.65	20.9833	20.75	20.7333	3.4636	16.4833	0.148	1.3152
4/20/2019 12:06	18.9333	21.2167	21.235	21.4167	3.3959	16.975	0.1567	1.3223
4/20/2019 12:07	19.2167	21.1833	20.9067	21.2333	3.3667	17.2167	0.1379	1.3237
4/20/2019 12:08	19.475	20.8833	21.655	21.6333	3.3296	17.42	0.1541	1.3116
4/20/2019 12:09	19.5833	21.0667	21.6067	21.2	3.2758	18.0333	0.146	1.3439
4/20/2019 12:10	19.8583	22.2167	21.5283	21.4167	3.2658	18.375	0.1448	1.3355
4/20/2019 12:11	19.9333	21.2667	21.165	21.6833	3.2784	18.5	0.1529	1.3277
4/20/2019 12:12	20.025	22.2333	21.635	21.6833	3.2583	18.425	0.1414	1.3333
4/20/2019 12:13	20.0583	21.9833	21.8483	21.6333	3.2316	18.3917	0.1397	1.3275
4/20/2019 12:14	20.125	21.7667	21.8817	21.5667	3.2697	18.3	0.1419	1.3106
4/20/2019 12:15	20.275	22.1167	21.86	22.2167	3.406	18.2083	0.1314	1.2815
4/20/2019 12:16	20.2417	22.3667	22.085	22.1833	3.4851	18.3	0.1391	1.3143
4/20/2019 12:17	20.1917	21.2833	21.035	21.3333	3.4733	18.3	0.1421	1.335
4/20/2019 12:18	20.5333	23.6667	23.975	23.9333	3.4845	18.21	0.132	1.4538
4/20/2019 12:19	21.1	23.6	23.6583	24.1333	3.5116	18.125	0.1478	1.3703

4/20/2019 12:20	21.5667	23.4	23.1883	22.6167	3.528	17.92	0.1389	1.3535
4/20/2019 12:21	21.3417	21.2	22.8883	21.7833	3.5298	17.775	0.1358	1.3768
4/20/2019 12:22	20.7417	21.5667	22.205	21.35	3.5192	17.6909	0.1457	1.3679
4/20/2019 12:23	20.2	20.65	21.795	21.3333	3.5114	17.95	0.1333	1.3759
4/20/2019 12:24	19.675	20.5	21.4883	20.1833	3.5284	18.2091	0.1385	1.4022
4/20/2019 12:25	19.2	19.4	21.34	19.7167	3.5239	18.4667	0.1583	1.4099
4/20/2019 12:26	18.75	18.8333	20.39	19.0333	3.52	18.6545	0.1431	1.4168
4/20/2019 12:27	18.3	18.4667	20.2583	18.6667	3.52	18.8917	0.1594	1.4408
4/20/2019 12:28	17.85	18.2167	19.7633	18	3.5288	19.1909	0.1477	1.4643
4/20/2019 12:29	17.3333	17.2	18.8833	17.0833	3.5369	19.4091	0.1552	1.4661
4/20/2019 12:30	16.7667	16.1333	18.9767	16.9167	4.1623	19.7818	0.168	1.4889
4/20/2019 12:31	15.4583	13.4167	19.3617	14.45	10.3617	20.7583	0.1833	1.5921
4/20/2019 12:32	13.3417	10.85	26.845	17.1833	18.4366	22.825	0.2676	1.636
4/20/2019 12:33	9.9083	10.3	72.0842	37.1	41.9259	58.0546	0.5601	2.0204
4/20/2019 12:34	6.2167	6.7833	106.5167	47.8833	50.545	100.7667	0.8771	2.1803
4/20/2019 12:35	3.125	7.05	107.65	46.9167	52.4961	112.5091	0.9263	2.3038
4/20/2019 12:36	1.4167	15.9667	127.1667	55.8333	57.7426	119.9909	1.1184	2.2322
4/20/2019 12:37	0.9083	5.7	203.1083	98	69.8939	148.87	2.9492	2.4438
4/20/2019 12:38	0.725	6.8333	164.2667	66.1	60.8141	189.19	2.0094	2.4833
4/20/2019 12:39	0.6	7	149.9583	64.0333	55.1678	160.4917	1.5508	2.5055
4/20/2019 12:40	0.5333	7.8	163.8583	67.9167	59.9967	162.7	1.7538	2.5243
4/20/2019 12:41	0.5	8.3167	160.9917	67.7833	59.3742	162.7333	1.7843	2.5327
4/20/2019 12:42	0.5	7.5333	159.3417	66.35	58.8049	158.3364	1.8192	2.5299
4/20/2019 12:43	0.5	7.2	156.4833	65.9833	58.151	157.4182	1.9022	2.5313
4/20/2019 12:44	0.5083	7.25	153.5667	65.2667	57.3261	155.8727	1.886	2.5168
4/20/2019 12:45	0.5	7.15	150.5917	64.1667	56.3963	155.725	1.9203	2.5324
4/20/2019 12:46	0.5	6.9667	147.05	62.9667	55.6173	154.3833	1.9423	2.4956
4/20/2019 12:47	0.4833	7.65	143.375	61.9667	54.6303	152.0818	1.9327	2.4883
4/20/2019 12:48	0.5	7.3167	140.8917	61.1	53.8684	150.1333	1.9488	2.5038
4/20/2019 12:49	0.5	7.7833	138.0417	59.75	53.1251	149.4909	1.9743	2.4912
4/20/2019 12:50	0.5	6.75	135.1333	59.0333	52.193	148.8583	1.9689	2.4957
4/20/2019 12:51	0.5	7	132.175	57.4	51.3068	148.13	1.9928	2.4808
4/20/2019 12:52	0.5	6.8833	129.8583	56.9333	50.667	147.4364	1.9569	2.4921
4/20/2019 12:53	0.5	6.3833	127.6083	55.7333	49.9515	146.4111	1.9644	2.5014
4/20/2019 12:54	0.5	6.5167	125.1333	54.85	49.2645	145.5667	1.986	2.5022
4/20/2019 12:55	0.5	6.5	123.6417	55.0167	48.7068	144.3727	1.9831	2.4909
4/20/2019 12:56	0.5333	6.5167	121.6083	53.0333	48.0683	143.3273	1.9615	2.4877
4/20/2019 12:57	0.6	5.25	119.4167	53	47.3608	141.35	1.9785	2.4582
4/20/2019 12:58	0.6	6.3333	117.575	52.8333	46.7808	140.1583	1.9628	2.4978
4/20/2019 12:59	0.6	6.2167	115.8	52.2833	46.1558	138.82	1.9383	2.5187
4/20/2019 13:00	0.55	5.8	113.6583	50.9333	45.4798	137.6182	1.9498	2.5157
4/20/2019 13:01	0.5833	5.7167	111.9	50	44.685	136.075	1.9192	2.5068
4/20/2019 13:02	0.6	6	110.5917	49.8167	44.1977	135.3909	1.9184	2.4888
4/20/2019 13:03	0.6	5.6167	109.2833	48.8667	43.7033	134.6727	1.8801	2.483
4/20/2019 13:04	0.65	5.85	107.5917	47.9167	43.0853	133.9273	1.8991	2.4562
4/20/2019 13:05	0.625	5.7833	106.0917	47.5667	42.4627	133	1.8739	2.4696
4/20/2019 13:06	0.6	5.6833	104.275	46.65	41.9632	132.15	1.8373	2.4481

4/20/2019 13:07	0.6	4.8833	102.9	45.7833	41.421	131.0909	1.8688	2.4416
4/20/2019 13:08	0.6	5.8333	101.4833	46	40.9094	130.0583	1.8267	2.4559
4/20/2019 13:09	0.6333	5.3167	99.9542	45.8333	40.2538	129.7636	1.8412	2.4368
4/20/2019 13:10	0.7	5.3167	98.1658	45.05	39.5944	129.1	1.7996	2.467
4/20/2019 13:11	0.7	4.9	97.5075	43.9833	39.2406	128.0909	1.8065	2.4539
4/20/2019 13:12	0.725	5.4167	96.0592	43.3833	38.7543	127.01	1.8196	2.4448
4/20/2019 13:13	0.7	5.4833	94.5742	42.8667	38.2311	125.925	1.8364	2.4508
4/20/2019 13:14	0.65	5.3167	93.5583	42.3333	37.8993	124.875	1.8345	2.4583
4/20/2019 13:15	0.6	4.6667	92.5583	42.1833	37.3383	123.8364	1.8091	2.4677
4/20/2019 13:16	0.5417	4.4833	91.3408	41.1167	36.8248	122.6333	1.7813	2.4503
4/20/2019 13:17	0.6	5.3333	89.7542	40.4167	36.2199	121.425	1.7631	2.4103
4/20/2019 13:18	0.6	5.1667	89.095	40.05	35.8625	120.2182	1.7692	2.4535
4/20/2019 13:19	0.6	4.7167	87.5383	38.9833	35.4305	119.2727	1.7594	2.4564
4/20/2019 13:20	0.5917	4.9833	86.7717	39.0333	35.0094	118.2667	1.763	2.4294
4/20/2019 13:21	0.5333	4.6833	85.7242	39.0167	34.6037	117.0727	1.7443	2.4023
4/20/2019 13:22	0.5	4.4833	84.6383	38.3167	34.1422	115.9083	1.7288	2.3825
4/20/2019 13:23	0.5	4.7167	83.4333	38.3333	33.6228	114.9364	1.69	2.3996
4/20/2019 13:24	0.5	4.3	82.6517	37.4667	33.2838	113.9417	1.6867	2.3905
4/20/2019 13:25	0.5	4.1667	81.6517	36.8333	32.8906	112.9833	1.6729	2.392
4/20/2019 13:26	0.5	3.9333	80.9775	36.4	32.482	112.1909	1.6875	2.3783
4/20/2019 13:27	0.5	3.6333	79.83	35.85	32.302	111.4	1.6709	2.3967
4/20/2019 13:28	0.5	4.2667	78.98	35.5833	32.302	110.675	1.698	2.4012
4/20/2019 13:29	0.5	3.95	78.225	34.9833	31.7685	109.97	1.6919	2.3989
4/20/2019 13:30	0.5	3.9167	77.5117	35	30.8812	109.2091	1.6633	2.3928
4/20/2019 13:31	0.5167	4.0167	76.3225	34.6667	30.5824	108.52	1.6563	2.3727
4/20/2019 13:32	0.5	4.5167	75.3975	34.0167	30.2125	107.9364	1.6518	2.3814
4/20/2019 13:33	0.575	4.0333	74.6317	33.75	29.8896	107.33	1.6576	2.3765
4/20/2019 13:34	0.6	4.35	73.9142	33.5667	29.5146	106.7333	1.6524	2.371
4/20/2019 13:35	0.6	3.6667	73.2658	33.1	29.149	106.03	1.6348	2.396
4/20/2019 13:36	0.6	3.9833	72.1275	32.4167	28.8404	105.4091	1.6298	2.4175
4/20/2019 13:37	0.6	3.6833	71.7067	32.2667	28.5528	104.7545	1.6327	2.4089
4/20/2019 13:38	0.6	3.7167	71.4175	32.1833	28.3216	104.1667	1.616	2.4232
4/20/2019 13:39	0.6167	4.4	70.2983	31.6833	27.9749	103.5455	1.599	2.4023
4/20/2019 13:40	0.7	3.4833	69.5942	30.95	27.59	102.8	1.6364	2.4085
4/20/2019 13:41	0.6	3.6667	69.0492	30.8667	27.2868	102.2091	1.5843	2.4101
4/20/2019 13:42	0.6	3.7	68.1467	30.3833	26.9737	101.8417	1.59	2.4015
4/20/2019 13:43	0.6	3.45	67.5933	29.95	26.5784	101.2917	1.616	2.3914
4/20/2019 13:44	0.6	3.5833	66.7558	30.1333	26.3341	100.7091	1.5764	2.3935
4/20/2019 13:45	0.575	3.4167	65.9942	29.95	26.0604	100.15	1.5877	2.3881
4/20/2019 13:46	0.5	3.2167	65.4942	29.1	25.7121	99.4273	1.5762	2.4218
4/20/2019 13:47	0.5	2.65	64.5758	28.7667	25.3226	98.675	1.4838	2.4417
4/20/2019 13:48	0.5	3.4667	64.3525	29.4333	25.1302	98.0273	1.4761	2.4187
4/20/2019 13:49	0.5	3.15	63.585	29.0833	24.8924	97.3333	1.5117	2.3778
4/20/2019 13:50	0.45	3.1167	63.3083	28.4333	24.6703	96.7	1.5361	2.412
4/20/2019 13:51	0.4333	2.9	62.6192	27.9833	24.3814	96.05	1.4571	2.4039
4/20/2019 13:52	0.5	3.9	62.175	28.4833	24.0902	95.4833	1.4496	2.3978
4/20/2019 13:53	0.525	2.8667	59.1133	26.4	23.2148	94.925	1.4053	2.382

4/20/2019 13:54	0.6	3.9	55.9183	26.3333	22.0106	94	1.3041	2.3848
4/20/2019 13:55	0.6	3.05	54.6317	24.9833	22.2915	93.0417	1.3359	2.4145
4/20/2019 13:56	0.6636	3.1667	46.4567	21.8	21.7838	91.8091	1.1928	2.1476
4/20/2019 13:57	1.025	4.9167	34.9942	19.65	20.0658	89.8	0.9656	1.9115
4/20/2019 13:58	1.3833	4.0667	36.8825	18.9833	21.7789	87.8727	0.9915	1.8385
4/20/2019 13:59	1.7917	4.15	32.2583	17.6167	21.1328	85.3333	0.875	1.9863
4/20/2019 14:00	1.9833	5.0333	29.3917	16.5833	20.5942	82.5364	0.8189	1.9029
4/20/2019 14:01	2.2833	5.8333	26.8658	15.9667	20.1084	79.5727	0.7695	1.8448
4/20/2019 14:02	2.6667	6.3	24.0258	15.3833	19.0524	76.1909	0.6836	1.7852
4/20/2019 14:03	3.075	6.3667	22.2008	15.6167	18.0295	72.525	0.6385	1.7533
4/20/2019 14:04	3.5167	7.2833	21.0617	14.6667	17.3992	68.8444	0.5911	1.7498
4/20/2019 14:05	3.95	7.5	19.8167	14.9333	16.3546	64.7	0.5433	1.7063
4/20/2019 14:06	4.45	8.35	18.5483	14.8	14.9808	60.7273	0.5008	1.6584
4/20/2019 14:07	4.9167	8.6333	18.3317	13.9333	14.7118	56.8333	0.484	1.6367
4/20/2019 14:08	5.425	10.8833	17.3417	14.85	13.2593	53.1222	0.4401	1.5994
4/20/2019 14:09	5.975	11.1167	16.8967	15.4333	11.9999	49.85	0.4131	1.5617
4/20/2019 14:10	7.0083	13.3333	15.4933	16.8333	9.5375	44.3	0.3172	1.5112
4/20/2019 14:11	8.0083	13.7833	15.365	16.8167	8.8038	28.3	0.316	1.5035
4/20/2019 14:12	8.9333	15.0167	15.3817	17	8.2515	26.5429	0.2822	1.4905
4/20/2019 14:13	9.3333	13.9833	15.3267	16.6833	7.9824	27.88	0.2852	1.5148
4/20/2019 14:14	9.6167	14.7667	15.955	16.95	7.7543	28.8455	0.2876	1.4883
4/20/2019 14:15	9.9833	14.95	15.925	17.25	7.1652	28.6182	0.2583	1.467
4/20/2019 14:16	10.3583	15.2	15.92	17	6.8745	28.3455	0.2452	1.4624
4/20/2019 14:17	10.7667	15.7667	15.8717	17.3833	6.5698	28.0167	0.2569	1.4449
4/20/2019 14:18	10.8833	14.9833	16.1933	17.3667	6.4	27.62	0.2252	1.4381
4/20/2019 14:19	11.2167	15.9667	16.2017	18.0333	5.9058	27.12	0.2306	1.4284
4/20/2019 14:20	11.5167	16.2667	16.3417	17.1667	5.7548	26.6	0.2227	1.3716
4/20/2019 14:21	11.9333	16.5667	16.7667	18.2333	5.4998	26.025	0.2029	1.394
4/20/2019 14:22	12.2167	16.25	16.5917	17.7667	5.1696	25.3333	0.2126	1.3823
4/20/2019 14:23	12.475	16.6833	16.6633	17.9833	5.1127	24.72	0.1925	1.3864
4/20/2019 14:24	12.775	17.7	17.5383	18.9167	4.7676	24.0333	0.1819	1.4
4/20/2019 14:25	13.175	17.8167	17.5033	18.5167	4.5593	23.33	0.1844	1.3687
4/20/2019 14:26	13.5455	18.7167	17.42	18.45	4.4784	22.5667	0.1742	1.3623
4/20/2019 14:27	13.8417	18.2667	17.6667	19.0333	4.4558	22	0.1754	1.3649
4/20/2019 14:28	14.0917	18.25	17.8267	19.1	4.3706	21.4	0.1674	1.3576
4/20/2019 14:29	14.3583	19.0333	18.115	19.1167	4.371	20.9636	0.1575	1.3248
4/20/2019 14:30	14.6417	18.55	18.24	19.1167	4.2266	20.5091	0.1705	1.3222
4/20/2019 14:31	14.75	18.9167	18.1433	19.2667	3.8399	20.125	0.1582	1.317
4/20/2019 14:32	14.7417	18.5333	17.5667	18.9333	3.7739	19.7455	0.1554	1.3112
4/20/2019 14:33	14.85	19.3	18.2783	20.2	3.6888	19.3917	0.1603	1.3374
4/20/2019 14:34	15.0167	18.2667	18.5633	19.6333	3.6083	19.2636	0.1544	1.3383
4/20/2019 14:35	15.0833	17.8333	18.425	18.6	3.627	19.075	0.1618	1.3185
4/20/2019 14:36	15.05	19.0667	18.0417	19.4167	3.5469	18.85	0.1486	1.3173
4/20/2019 14:37	15.0583	18.4333	18.6383	19.25	3.4259	18.7	0.1298	1.3151
4/20/2019 14:38	15.1333	18.1667	17.6333	18.9667	3.4287	18.6182	0.148	1.2919
4/20/2019 14:39	15.2583	19.0167	18.81	19.7	3.3793	18.6	0.148	1.3073
4/20/2019 14:40	15.4083	19.8333	18.2333	19.35	3.3948	18.65	0.144	1.2894

4/20/2019 14:41	15.95	20.3333	19.34	20.1667	3.376	18.625	0.1416	1.3065
4/20/2019 14:42	16.3167	19.6667	19.1583	20.3167	3.3426	18.3273	0.1425	1.3054
4/20/2019 14:43	16.7	20.7667	19.665	20.5833	3.2925	18.3	0.1222	1.301
4/20/2019 14:44	16.7583	19.3833	18.71	19.8	3.3297	18.3	0.134	1.3109
4/20/2019 14:45	16.5917	19.0833	18.635	19.3667	3.352	18.3	0.133	1.2951
4/20/2019 14:46	16.2583	19.35	18.1183	18.4333	3.3308	18.2546	0.1338	1.3051
4/20/2019 14:47	15.8083	17.6	17.88	17.8333	3.3938	18.2	0.1455	1.291
4/20/2019 14:48	15.4417	17.45	17.8017	17.8833	3.4693	18.2546	0.125	1.299
4/20/2019 14:49	15.4083	18.1667	18.0517	18.9833	3.4342	18.2417	0.1345	1.3368
4/20/2019 14:50	15.625	18.55	18.4367	19.1333	3.3793	18.2727	0.1281	1.3401
4/20/2019 14:51	16.0417	18.6667	18.5433	19.6	3.356	18.1909	0.1255	1.3381
4/20/2019 14:52	16.5	20.0167	19.5933	20.2667	3.4214	18.1	0.1241	1.3218
4/20/2019 14:53	16.7583	19.5167	19.28	19.5	3.3486	18.0333	0.124	1.2913
4/20/2019 14:54	17.15	19.8833	19.7433	20.0667	3.2451	18.26	0.1179	1.2991
4/20/2019 14:55	17.4	20.5	20.3717	20.9667	3.2293	18.2667	0.1281	1.301
4/20/2019 14:56	17.7636	20.65	20.2167	21.35	3.279	18.2	0.1129	1.2892
4/20/2019 14:57	18.0667	21.3333	20.37	21.4	3.2032	18.1455	0.1236	1.309
4/20/2019 14:58	18.225	22.0167	20.43	21	3.0649	18.1	0.1148	1.3215
4/20/2019 14:59	18.3167	20.6667	20.4017	21.3333	3.2527	18.1	0.257	1.315
4/20/2019 15:00	18.025	19.95	19.68	19.9667	4.575	18.1364	0.5126	1.2763
4/20/2019 15:01	17.875	20.0667	20.2833	20.9333	4.5822	18.2	0.2943	1.2916
4/20/2019 15:02	17.9333	20.4167	20.6617	21.6	4.186	18.2	0.2114	1.2898
4/20/2019 15:03	18.425	21.7167	20.7233	21.65	3.8726	18.1364	0.1983	1.3154
4/20/2019 15:04	18.5917	20.8667	20.5133	20.6667	3.8921	18.1	0.1725	1.2778
4/20/2019 15:05	18.6333	21.55	20.6317	21.05	3.8248	18.1	0.1765	1.2652
4/20/2019 15:06	18.6167	21.35	20.98	21.8167	3.6455	18.0167	0.1541	1.3049
4/20/2019 15:07	19.05	21.9667	21.8517	22.45	3.4975	18	0.1426	1.3039
4/20/2019 15:08	19.1917	21.65	21.42	22.3333	3.5222	18	0.1609	1.297
4/20/2019 15:09	19.5833	22.7667	22.2783	23.2	3.3064	17.9364	0.1469	1.3104
4/20/2019 15:10	19.7833	22.7	22.5133	22.8167	3.2362	17.9	0.1456	1.2759
4/20/2019 15:11	20.1917	22.6167	22.1883	23.25	3.201	17.8727	0.1531	1.259
4/20/2019 15:12	20.1667	22.3833	22.0117	21.5167	3.1949	17.625	0.1461	1.2718
4/20/2019 15:13	20.075	21.75	22.1467	22.45	3.0998	17.3546	0.141	1.2898
4/20/2019 15:14	19.9167	22.8333	21.745	22.6167	3.0603	17.1818	0.1487	1.3038
4/20/2019 15:15	20.0083	22.2167	21.775	22.3833	3.0428	17.0909	0.1349	1.2868
4/20/2019 15:16	20.125	23.05	22.2433	22.9167	3.2413	16.975	0.14	1.2888
4/20/2019 15:17	20.45	22.8167	22.365	22.5167	3.4908	16.9	0.1429	1.2818
4/20/2019 15:18	20.2917	21.4167	21.4683	21.4667	3.5172	16.9	0.1229	1.2777
4/20/2019 15:19	20.2667	22.9833	22.4567	22.6	3.5189	16.9	0.1412	1.2924
4/20/2019 15:20	20.2083	23.15	22.2717	23.2	3.4696	16.85	0.1283	1.2913
4/20/2019 15:21	20.6417	22.6833	22.4817	23.6667	3.329	16.8	0.1284	1.2974
4/20/2019 15:22	20.8083	23.3	22.735	24.2833	3.1799	16.7583	0.1381	1.2619
4/20/2019 15:23	21.0667	22.8667	23.5833	23.4833	3.0869	16.7	0.1229	1.2854
4/20/2019 15:24	21.2667	23.1667	22.74	23.8833	3.1327	16.7083	0.1257	1.294
4/20/2019 15:25	21.3333	22.9833	22.7567	23.4167	3.118	16.8	0.1273	1.2773
4/20/2019 15:26	20.8	20.9833	21.5133	21.6167	3.21	16.7083	0.1073	1.2463
4/20/2019 15:27	20.0833	21.2833	21.0967	20.3667	3.2306	16.7	0.1305	1.25

4/20/2019 15:28	19.3	20.0333	20.63	20.05	3.2226	16.7	0.1349	1.277
4/20/2019 15:29	18.7167	19.45	19.9167	19.3333	3.2022	16.7455	0.1225	1.3138
4/20/2019 15:30	18.0583	18.1833	19.165	18.6667	3.203	16.7	0.1429	1.3188
4/20/2019 15:31	17.4833	19.1833	19.14	19.0833	3.2038	16.7091	0.1256	1.306
4/20/2019 15:32	16.9083	17.2167	18.505	18.2	3.2046	16.8	0.1253	1.3152
4/20/2019 15:33	16.5083	17.9333	18.5192	17.6833	3.2039	16.9182	0.1349	1.2948
4/20/2019 15:34	16.075	16.9833	18.0742	16.6833	3.2049	17.125	0.1121	1.3019
4/20/2019 15:35	15.7083	16.4333	17.5275	16.9333	3.2024	17.3636	0.1327	1.3196
4/20/2019 15:36	15.3167	15.4667	17.255	16.2167	3.1997	17.625	0.1346	1.3323
4/20/2019 15:37	14.9667	15.2167	16.8267	15.9167	3.1901	17.81	0.1279	1.3339
4/20/2019 15:38	14.6417	15.3833	16.7242	15.3667	3.1738	17.8917	0.1318	1.3263
4/20/2019 15:39	14.2667	14.4167	16.2992	14.2667	3.197	18.2364	0.1312	1.3393
4/20/2019 15:40	13.875	14.3	15.9583	14.5333	3.1854	18.6083	0.1254	1.3471
4/20/2019 15:41	13.5667	13.5833	15.8992	14.4333	3.1851	18.925	0.1395	1.3446
4/20/2019 15:42	13.2833	13.9667	15.1583	14.5667	3.203	19.2667	0.1266	1.3563
4/20/2019 15:43	13.0333	13.0833	15.255	13.4	3.1892	19.6	0.1337	1.3413
4/20/2019 15:44	12.8083	13.1833	15.1158	13.1	3.1732	19.85	0.1444	1.3514
4/20/2019 15:45	12.5917	12.7667	14.7925	12.3167	3.1872	20.1909	0.1265	1.3536
4/20/2019 15:46	12.3167	12.5167	14.685	12.0833	3.2005	20.4583	0.1397	1.3652
4/20/2019 15:47	11.9083	11.7667	14.0733	11.7167	3.1996	20.7455	0.1342	1.3823
4/20/2019 15:48	11.6417	11.8667	14.0442	11.7333	3.1989	21.0083	0.139	1.3798
4/20/2019 15:49	11.3917	11.2667	13.505	11.6333	3.1814	21.2833	0.1473	1.4016
4/20/2019 15:50	11.1333	10.5333	13.6367	10.7333	3.1903	21.7727	0.1291	1.4129
4/20/2019 15:51	10.8667	10.9	12.9333	10.5	3.2048	21.9917	0.1361	1.4129
4/20/2019 15:52	10.5083	10.2833	12.8292	10.6833	3.2085	22.1636	0.1381	1.4062
4/20/2019 15:53	10.2583	10.25	12.5733	9.9333	3.2032	22.2833	0.1296	1.4138
4/20/2019 15:54	10.0167	9.2333	12.3175	9.6167	3.2037	22.2833	0.1454	1.4082
4/20/2019 15:55	9.85	10	11.9033	9.6333	3.2193	22.375	0.1368	1.4167
4/20/2019 15:56	9.625	9.7167	11.9792	9.35	3.2262	22.55	0.1437	1.4225
4/20/2019 15:57	9.3167	9.1167	11.7208	8.25	3.2356	22.7182	0.1386	1.4173
4/20/2019 15:58	9.0667	8.0833	11.5592	8.6667	3.2443	22.8583	0.1327	1.4255
4/20/2019 15:59	8.8	8.5	11.4208	8.2667	3.2393	22.7083	0.1439	1.3773
4/20/2019 16:00	8.6167	7.7333	11.0775	8.0833	3.2467	22.7727	0.1346	1.3895
4/20/2019 16:01	8.45	8.7167	10.66	7.8833	3.2315	22.8833	0.1296	1.4369
4/20/2019 16:02	8.2917	8.5667	10.5575	7.2333	3.2468	22.9917	0.1469	1.4409
4/20/2019 16:03	8.1333	6.9667	10.6292	7.5333	3.2474	23.1	0.1328	1.4602
4/20/2019 16:04	7.9333	7.35	10.5342	7.5	3.2417	23.2333	0.1447	1.4692
4/20/2019 16:05	7.7	6.7333	10.2308	6.75	3.2649	23.3727	0.1434	1.4677
4/20/2019 16:06	7.4833	6.5833	9.9121	6.7833	3.2703	23.625	0.1301	1.4697
4/20/2019 16:07	7.2417	6.6333	9.9269	6.6333	3.2454	23.9273	0.1447	1.4477
4/20/2019 16:08	7.0833	6.6	9.9231	6.6167	3.2557	24.0167	0.1384	1.432
4/20/2019 16:09	6.925	6.3167	9.6682	5.9167	3.2653	24.14	0.1335	1.4357
4/20/2019 16:10	6.7917	5.6833	9.6031	6.1833	3.2636	24.225	0.1502	1.4407
4/20/2019 16:11	6.65	5.75	9.3595	6.4667	3.2718	24.5273	0.1306	1.4426
4/20/2019 16:12	6.475	5.0667	9.1678	5.7333	3.27	24.7	0.1398	1.4669
4/20/2019 16:13	6.2667	5.95	8.9988	4.9333	3.2653	24.8182	0.1416	1.4847
4/20/2019 16:14	6.0167	5.1667	8.6877	4.6333	3.2575	24.9083	0.1318	1.4556

4/20/2019 16:15	5.775	5.4167	8.6299	5.6833	3.2511	25.0167	0.1415	1.4656
4/20/2019 16:16	5.6083	5.3667	8.4909	5.5333	3.2638	25.1667	0.1351	1.4756
4/20/2019 16:17	5.4917	5.2167	8.4538	5.3833	3.2617	25.25	0.1332	1.4758
4/20/2019 16:18	5.3833	4.9833	8.5164	4.95	3.2448	25.3	0.1358	1.5018
4/20/2019 16:19	5.2667	5.1	8.4551	4.7333	3.2602	25.3	0.1317	1.5058
4/20/2019 16:20	5.1917	5.1	8.3691	4.5	3.2573	25.3833	0.1428	1.4938
4/20/2019 16:21	5.0833	4.5333	8.117	5.2333	3.2508	25.4083	0.135	1.4896
4/20/2019 16:22	4.95	3.7833	8.077	4.1167	3.2385	25.47	0.1366	1.4862
4/20/2019 16:23	4.8333	4.2333	7.8858	3.9333	3.2397	25.5	0.1395	1.4821
4/20/2019 16:24	4.7417	3.5333	7.8167	4.05	3.2313	25.52	0.1247	1.4744
4/20/2019 16:25	4.5917	3.9	7.7308	3.9333	3.241	25.6	0.1368	1.4713
4/20/2019 16:26	4.3917	3.9167	7.397	3.7167	3.232	25.6	0.1377	1.4393
4/20/2019 16:27	4.2583	3.3	7.6528	3.8	3.2366	25.6	0.1349	1.4458
4/20/2019 16:28	4.1364	3.9833	7.6885	4.0667	3.2113	25.6	0.1496	1.4536
4/20/2019 16:29	4.025	3.7833	7.4571	3.85	3.2356	25.6818	0.131	1.4574
4/20/2019 16:30	3.925	2.9167	7.3004	3.5667	3.2391	25.7	0.1452	1.4752
4/20/2019 16:31	3.8167	3.4	7.2932	3.3167	3.238	25.8	0.1344	1.4988
4/20/2019 16:32	3.7833	3.75	7.3105	2.8667	3.2247	25.85	0.1275	1.4767
4/20/2019 16:33	3.7	3.8667	7.1539	2.9167	3.1749	26	0.1402	1.4689
4/20/2019 16:34	3.6417	3.2333	7.3076	2.8	3.1661	26.1417	0.1277	1.5045
4/20/2019 16:35	3.5083	2.0333	7.0208	2.7167	3.1599	26.2917	0.1354	1.4774
4/20/2019 16:36	3.3667	3.5	6.9023	3.5167	3.1545	26.4091	0.1363	1.4773
4/20/2019 16:37	3.25	3.7333	6.746	2.35	3.1405	26.53	0.1274	1.4794
4/20/2019 16:38	3.1167	2.9167	7.0618	2.4667	3.1314	26.5727	0.148	1.4927
4/20/2019 16:39	2.9667	2.7333	6.6779	2.35	3.1439	26.6455	0.1278	1.488
4/20/2019 16:40	2.8333	2.95	6.5822	2.7	3.1415	26.7636	0.1394	1.496
4/20/2019 16:41	2.8	3.1167	6.5164	2.15	3.1516	26.8	0.1409	1.5209
4/20/2019 16:42	2.8	2.35	6.3037	2.3	3.1223	26.8	0.1356	1.5108
4/20/2019 16:43	2.7167	2.3833	6.323	2.3833	3.1305	26.9	0.1394	1.5132
4/20/2019 16:44	2.6417	2.35	6.3546	2	3.1456	26.9636	0.1373	1.4812
4/20/2019 16:45	2.6333	1.3	6.3682	2.5	3.1305	26.9818	0.143	1.4874
4/20/2019 16:46	2.65	1.3	6.3437	1.9	3.1293	26.9273	0.1327	1.5066
4/20/2019 16:47	2.5917	1.85	6.186	2.3333	3.1319	26.9	0.1294	1.5063
4/20/2019 16:48	2.5	1.7667	6.5372	2.2167	3.121	26.9	0.1401	1.5155
4/20/2019 16:49	2.45	2.4	6.2509	2.2	3.126	26.97	0.1292	1.5133
4/20/2019 16:50	2.4	2.3	6.0548	1.8167	3.112	26.9417	0.133	1.5051
4/20/2019 16:51	2.35	1.7167	6.0767	2.1	3.1239	26.9455	0.1365	1.5227
4/20/2019 16:52	2.3	1.1167	6.0134	1.8667	3.1079	26.9546	0.1267	1.5323
4/20/2019 16:53	2.275	1.5333	6.1563	1.8167	3.1183	26.92	0.1459	1.5562
4/20/2019 16:54	2.2	1.9667	5.8544	1.1667	3.1243	26.9909	0.1267	1.5795
4/20/2019 16:55	2.2	1.25	6.2536	1.4167	3.1221	27.0417	0.132	1.5548
4/20/2019 16:56	2.2	1.5167	6.1733	1.3	3.1135	27.1	0.1449	1.5428
4/20/2019 16:57	2.225	1.1333	5.9365	1.2167	3.1015	27.1	0.1251	1.5441
4/20/2019 16:58	2.2667	1.0167	6.0783	1.1	3.1106	27.1	0.1305	1.5031
4/20/2019 16:59	2.2333	1.3333	6.3143	1.1167	3.1091	27.1083	0.1326	1.4868
4/20/2019 17:00	2.1417	0.95	5.8185	1.2833	3.1212	27.19	0.1244	1.5155
4/20/2019 17:01	2.075	0.85	5.6238	0.7167	3.1173	27.1	0.1323	1.516

4/20/2019 17:02	2	0.95	5.9429	0.9833	3.1074	27.1	0.1145	1.5059
4/20/2019 17:03	1.9833	1.0333	5.8293	1.3333	3.0768	27.1545	0.1369	1.5385
4/20/2019 17:04	1.8917	1.1833	5.6558	1.2833	3.0792	27.2	0.1294	1.5474
4/20/2019 17:05	1.8417	1.1333	5.9728	1.4167	3.0854	27.3	0.1249	1.54
4/20/2019 17:06	1.8583	1.2	5.6998	1.2	3.1008	27.3917	0.1391	1.5344
4/20/2019 17:07	1.9	0.9333	5.7427	1.3167	3.0872	27.3833	0.1266	1.5043
4/20/2019 17:08	1.9	0.6833	5.4797	0.55	3.0639	27.3	0.1356	1.5503
4/20/2019 17:09	1.8833	1.05	5.6952	1.0167	3.0753	27.4091	0.1418	1.5472
4/20/2019 17:10	1.8167	0.1667	5.6955	0.7333	3.0874	27.5	0.1268	1.5598
4/20/2019 17:11	1.7667	0.9333	5.7047	1.4667	3.0846	27.5	0.1376	1.5678
4/20/2019 17:12	1.7	0.8667	5.6327	1.15	3.0623	27.575	0.1218	1.5421
4/20/2019 17:13	1.6667	0.4333	5.5472	1.05	3.0728	27.6083	0.1243	1.5231
4/20/2019 17:14	1.6667	1.0167	5.3788	0.6333	3.0563	27.7417	0.138	1.5288
4/20/2019 17:15	1.7	1.7	5.4992	0.5667	3.0526	27.8	0.1154	1.5649
4/20/2019 17:16	1.7	-0.0333	5.5555	0.5333	3.0543	27.8	0.1369	1.5896
4/20/2019 17:17	1.7	0.4333	5.3995	0.4167	3.0454	27.8	0.1279	1.6272
4/20/2019 17:18	1.65	0.5667	5.6065	0.6167	3.0299	27.8	0.1387	1.6033
4/20/2019 17:19	1.6	0.5	5.4217	1	3.0273	27.8	0.146	1.6076
4/20/2019 17:20	1.525	0.1833	5.4707	0.7167	3.036	27.8273	0.1286	1.5857
4/20/2019 17:21	1.5	0.0833	5.309	0.4	3.0268	27.9	0.14	1.5632
4/20/2019 17:22	1.5583	1.15	5.5895	0.15	3.0353	27.9	0.135	1.5703
4/20/2019 17:23	1.5583	0.95	5.4373	0.5	3.0305	28.0333	0.1284	1.562
4/20/2019 17:24	1.5167	0.0167	5.3807	0.5	3.0038	28.12	0.1369	1.5553
4/20/2019 17:25	1.4667	0.0667	5.3677	0.4833	3.0089	28.2364	0.1227	1.5494
4/20/2019 17:26	1.4	0.4667	5.3655	-0.1333	3.0233	28.3167	0.1349	1.5318
4/20/2019 17:27	1.4	0.3167	5.3282	0.3	3.0012	28.4	0.1304	1.5508
4/20/2019 17:28	1.4	0.9333	5.4563	0.1	2.9804	28.4667	0.1267	1.5633
4/20/2019 17:29	1.4	0.4167	5.2383	0.5333	2.9808	28.5833	0.1375	1.5692
4/20/2019 17:30	1.3083	0.1833	5.4268	0.1833	2.966	28.6364	0.1252	1.5799
4/20/2019 17:31	1.3	0.4333	5.4792	0.9167	2.9849	28.7	0.1254	1.5783
4/20/2019 17:32	1.2833	1.1333	5.4635	0.1667	2.991	28.7	0.1294	1.5954
4/20/2019 17:33	1.2083	0.1167	5.3928	0.7	2.9893	28.8	0.1206	1.5928
4/20/2019 17:34	1.2083	0.0333	5.3505	0.1833	2.9791	28.74	0.1318	1.6024
4/20/2019 17:35	1.2	0.1	5.1137	0.1	2.9623	28.8	0.1281	1.5664
4/20/2019 17:36	1.2	0.4833	5.1927	0.0167	2.957	28.8	0.1316	1.553
4/20/2019 17:37	1.2	0.3167	5.3445	0.4333	2.9311	28.7667	0.1384	1.5562
4/20/2019 17:38	1.1333	0.5833	5.3922	0.3833	2.9388	28.7	0.1191	1.5589
4/20/2019 17:39	1.1833	0.6333	5.267	0.5833	2.9614	28.7	0.1309	1.5888
4/20/2019 17:40	1.1667	0.6833	5.305	0.3333	2.9449	28.7	0.133	1.5908
4/20/2019 17:41	1.2	0.5167	5.2882	0.3833	2.9544	28.7	0.114	1.5778
4/20/2019 17:42	1.2083	0.0833	5.4617	0.0167	2.9312	28.6727	0.1332	1.587
4/20/2019 17:43	1.2833	-0.1	5.3147	0.4333	2.9508	28.7	0.1202	1.6218
4/20/2019 17:44	1.2	0.5333	5.2257	0.3333	2.9364	28.7	0.1269	1.5971
4/20/2019 17:45	1.1583	0.3833	5.3848	0.75	2.9205	28.8	0.1374	1.5716
4/20/2019 17:46	1.1083	-0.0833	5.222	0.6833	2.9194	28.84	0.1196	1.5683
4/20/2019 17:47	1.1833	0.1333	5.0317	0.5167	2.9253	28.8636	0.1304	1.5698
4/20/2019 17:48	1.2	-0.0667	5.0505	0.0167	2.8918	28.8	0.1278	1.5988

4/20/2019 17:49	1.2	0.1	5.1455	-0.7667	2.8897	28.8	0.1236	1.5688
4/20/2019 17:50	1.2	0.0833	5.3987	0.5333	2.8867	28.8455	0.1313	1.577
4/20/2019 17:51	1.2	0.4	5.31	0.6833	2.8822	28.9	0.1295	1.5988
4/20/2019 17:52	1.175	0.2667	5.2075	-0.05	2.8701	28.9	0.1303	1.5839
4/20/2019 17:53	1.1	0.45	4.8905	0.25	2.8722	28.9546	0.1388	1.5972
4/20/2019 17:54	1.1167	0.5833	5.2577	-0.0333	2.8668	29	0.1204	1.5895
4/20/2019 17:55	1.1	-0.2667	5.3172	-0.2833	2.8678	28.93	0.1379	1.5838
4/20/2019 17:56	1.1167	0.3833	5.193	0.4833	2.8638	28.9	0.1195	1.5961
4/20/2019 17:57	1.0833	-0.1667	5.453	-0.0833	2.879	28.9	0.1295	1.5993
4/20/2019 17:58	1.025	-0.0833	5.4337	-0.1833	2.8422	28.8727	0.1325	1.5769
4/20/2019 17:59	1	-0.2167	5.089	0.8333	2.8551	28.8091	0.1243	1.5851
4/20/2019 18:00	1	-0.3333	5.1715	0.15	2.8639	28.9	0.1304	1.5942
4/20/2019 18:01	0.9333	0.0667	5.2375	0.35	2.8568	28.9	0.1211	1.5763
4/20/2019 18:02	0.9	-0.2667	5.5602	0.0333	2.8485	28.8182	0.1297	1.5928
4/20/2019 18:03	0.9	0.1	5.5253	0.5333	2.8305	28.8	0.1431	1.6015
4/20/2019 18:04	0.9	0.45	5.2152	0.5167	2.7995	28.7583	0.1236	1.5936
4/20/2019 18:05	0.9	0.65	5.1662	0.5167	2.8173	28.79	0.1271	1.5993
4/20/2019 18:06	0.8917	0.4	5.2302	-0.1667	2.8183	28.7	0.1355	1.6245
4/20/2019 18:07	0.8333	-0.2833	5.1457	0.2	2.8408	28.7	0.1154	1.6199
4/20/2019 18:08	0.8	-0.1667	5.106	-0.1833	2.8232	28.7	0.1415	1.6319
4/20/2019 18:09	0.8	0.4833	5.1448	0.2833	2.8039	28.7546	0.1176	1.6137
4/20/2019 18:10	0.8	0.4167	5.1772	0.2667	2.8102	28.8	0.1263	1.5985
4/20/2019 18:11	0.8	-0.25	5.262	0.5333	2.8252	28.9	0.1282	1.6023
4/20/2019 18:12	0.8	0.6	5.3928	0	2.8206	28.8167	0.1219	1.6071
4/20/2019 18:13	0.8	0.0833	5.0952	0.2667	2.8263	28.8364	0.1362	1.6381
4/20/2019 18:14	0.8	-0.8167	5.208	0.5833	2.8183	28.9	0.118	1.6282
4/20/2019 18:15	0.85	-0.3333	5.3387	-0.2	2.8181	28.9	0.131	1.6183
4/20/2019 18:16	0.9	-0.2	5.5497	0.1667	2.8331	28.9455	0.1285	1.6
4/20/2019 18:17	0.8417	-0.5167	5.5772	-0.1833	2.8198	29	0.1215	1.636
4/20/2019 18:18	0.8	0.5667	5.4917	0.1667	2.8143	29	0.1323	1.6363
4/20/2019 18:19	0.8	0.4833	5.0677	-0.6667	2.8118	29.0818	0.1233	1.5773
4/20/2019 18:20	0.825	0.4	5.2768	-0.5333	2.8245	29.1	0.1293	1.5968
4/20/2019 18:21	0.9	-0.3667	5.2257	0.0333	2.8412	29.1273	0.1245	1.5863
4/20/2019 18:22	0.9	0.1167	5.165	0.1333	2.8253	29.1167	0.1223	1.6125
4/20/2019 18:23	0.9167	0.1833	5.234	-0.2667	2.8305	29.1	0.1315	1.6214
4/20/2019 18:24	0.9417	-0.2333	5.5852	0.2667	2.8546	29.1	0.1261	1.637
4/20/2019 18:25	1	-0.2	5.4288	0.0667	2.8511	29.1	0.1321	1.6554
4/20/2019 18:26	0.9417	0.1667	5.1148	0.0833	2.8498	29.0833	0.1313	1.6486
4/20/2019 18:27	1	-0.1333	5.154	-0.2333	2.8485	29	0.1185	1.6148
4/20/2019 18:28	1	0.4667	5.2577	0.0167	2.8645	29	0.1285	1.5783
4/20/2019 18:29	1	-0.05	5.172	-0.1333	2.8581	29	0.1238	1.578
4/20/2019 18:30	1	0.1	5.317	0.05	2.8498	29	0.1192	1.5835
4/20/2019 18:31	1	0.6667	5.0717	-0.5833	2.8578	29	0.1253	1.6133
4/20/2019 18:32	0.9667	-0.1833	4.9873	0.1667	2.8418	29	0.1183	1.6166
4/20/2019 18:33	0.9	-0.5167	4.9893	-0.25	2.842	29	0.1291	1.6373
4/20/2019 18:34	0.9	0.3333	4.9482	0.3833	2.8417	29	0.1264	1.6233
4/20/2019 18:35	0.9	0.05	5.1378	-0.2333	2.8486	29	0.1272	1.6268

4/20/2019 18:36	0.9	-0.6333	5.0892	-0.5167	2.8458	29.03	0.1342	1.6379
4/20/2019 18:37	0.9	-0.0667	5.1576	-0.5	2.8498	29.1	0.1212	1.6078
4/20/2019 18:38	0.9	0.75	5.1246	-0.2167	2.8482	29.1	0.1281	1.5888
4/20/2019 18:39	0.9083	0.7333	5.1214	-0.6	2.8498	29.1091	0.1253	1.6134
4/20/2019 18:40	0.9	0.5	5.2346	0.05	2.851	29.2	0.1216	1.6193
4/20/2019 18:41	0.9	-0.0667	5.2979	-0.6167	2.8382	29.2	0.1366	1.6257
4/20/2019 18:42	0.9	0.0667	5.275	-0.0167	2.8446	29.2	0.1229	1.6426
4/20/2019 18:43	0.9083	0.15	5.1624	-0.25	2.8285	29.2	0.1313	1.6454
4/20/2019 18:44	1	0.45	5.2029	0.15	2.8358	29.2	0.1378	1.6478
4/20/2019 18:45	0.9083	-0.6167	5.3808	-0.2333	2.8427	29.2818	0.1141	1.6393
4/20/2019 18:46	0.9	0	5.4077	-0.25	2.8281	29.3	0.1263	1.62
4/20/2019 18:47	0.9	-0.25	5.1703	0.7167	2.8402	29.3182	0.1318	1.6242
4/20/2019 18:48	0.9	-0.4167	5.1685	0.1	2.8124	29.3636	0.1176	1.6326
4/20/2019 18:49	0.9	0.0333	5.0643	0.05	2.8068	29.4	0.1369	1.6669
4/20/2019 18:50	0.9	0.2167	5.1663	0.0167	2.8302	29.4	0.1217	1.6596
4/20/2019 18:51	0.9	0.4167	5.0913	0.0667	2.8255	29.4	0.1315	1.6735
4/20/2019 18:52	0.95	-0.1667	5.3048	-0.6667	2.8138	29.4	0.1347	1.6639
4/20/2019 18:53	0.9583	0.3333	5.2993	-0.8833	2.8272	29.4667	0.124	1.6487
4/20/2019 18:54	0.9	0.0667	4.925	-0.0833	2.8161	29.5	0.1393	1.6248
4/20/2019 18:55	0.8833	-0.2833	5.0347	0.1167	2.7969	29.5	0.1225	1.6165
4/20/2019 18:56	0.8833	-0.6	5.0903	-0.05	2.7817	29.5	0.1245	1.6503
4/20/2019 18:57	0.9	0.0833	5.0143	-0.1333	2.785	29.5	0.1261	1.6613
4/20/2019 18:58	0.9	0.1333	4.8068	-0.0667	2.796	29.6	0.1169	1.6486
4/20/2019 18:59	0.8333	-0.6833	4.9494	-0.2667	2.7954	29.6	0.1359	1.6517
4/20/2019 19:00	0.8	-0.1167	5.4877	-0.35	2.7694	29.6	0.1181	1.6934
4/20/2019 19:01	0.8	0.1333	4.9826	-0.6333	2.762	29.525	0.1267	1.669
4/20/2019 19:02	0.8	0.3833	4.943	-0.3333	2.7745	29.5	0.1324	1.7022
4/20/2019 19:03	0.8	-0.4167	5.2967	-0.0333	2.7688	29.5	0.123	1.6836
4/20/2019 19:04	0.8	-0.1	5.1809	-0.4667	2.7506	29.5	0.1274	1.6662
4/20/2019 19:05	0.8	0.05	5.3999	0.1333	2.7418	29.4636	0.1303	1.6658
4/20/2019 19:06	0.8	0.0333	5.3157	-0.1833	2.757	29.3364	0.1169	1.6682
4/20/2019 19:07	0.8	-0.3	5.0847	0.4333	2.7575	29.375	0.1331	1.6453
4/20/2019 19:08	0.8083	-0.1333	5.0076	0.0833	2.7566	29.2909	0.1186	1.6219
4/20/2019 19:09	0.9	0.0667	5.3465	-0.0667	2.7362	29.2	0.1195	1.6516
4/20/2019 19:10	0.825	-0.4	5.2195	0.0667	2.7431	29.1833	0.1304	1.6487
4/20/2019 19:11	0.8	-0.1833	5.0608	-0.9	2.7523	29.1	0.1132	1.6662
4/20/2019 19:12	0.8	0.3667	5.1258	-0.3667	2.7473	29	0.1357	1.6673
4/20/2019 19:13	0.8083	-0.4833	5.072	-0.2833	2.7456	28.9182	0.1224	1.6351
4/20/2019 19:14	0.825	-0.1833	4.9827	0.2	2.7381	28.9	0.1233	1.6284
4/20/2019 19:15	0.8917	-0.35	5.2555	-0.3167	2.7524	28.9	0.1374	1.632
4/20/2019 19:16	0.9	-0.4	5.1514	-0.5	2.7391	28.8167	0.115	1.6279
4/20/2019 19:17	0.8917	0.7	5.0163	-0.3	2.7371	28.8	0.1333	1.6406
4/20/2019 19:18	0.8	-0.25	5.1375	-0.1333	2.7246	28.875	0.1211	1.6267
4/20/2019 19:19	0.8	-0.7167	5.0912	0.05	2.7312	28.9	0.1196	1.6327
4/20/2019 19:20	0.7833	0.55	5.0303	0.1	2.7201	28.9	0.1337	1.6546
4/20/2019 19:21	0.7917	-0.2167	5.3693	-0.85	2.7068	28.9833	0.1172	1.6929
4/20/2019 19:22	0.8	-0.7333	5.2613	-0.15	2.7192	29	0.1222	1.684

4/20/2019 19:23	0.8	-0.0667	5.17	-0.4667	2.7111	29.0546	0.1299	1.6946
4/20/2019 19:24	0.8	0.0667	5.1652	-0.5333	2.7103	29.1	0.1157	1.673
4/20/2019 19:25	0.8	-0.0167	5.2376	-0.5667	2.6731	29.1	0.1247	1.6823
4/20/2019 19:26	0.8	-0.3833	5.1278	-0.3333	2.6789	29.1	0.121	1.6432
4/20/2019 19:27	0.8	0.3	5.1981	-0.7333	2.6975	29.0333	0.122	1.6373
4/20/2019 19:28	0.8	-0.1	5.3451	-0.2833	2.6966	29.1	0.1293	1.6308
4/20/2019 19:29	0.8417	0.8333	5.0503	-0.5333	2.6942	29.1636	0.1116	1.6498
4/20/2019 19:30	0.85	0.8667	5.0707	-0.55	2.7035	29.075	0.1285	1.6673
4/20/2019 19:31	0.8667	-0.7667	5.0978	-0.05	2.7022	29	0.1239	1.667
4/20/2019 19:32	0.85	-0.7167	5.2275	-0.4	2.6978	29	0.1183	1.645
4/20/2019 19:33	0.8083	-0.3167	5.2215	0.1	2.6933	28.9417	0.1331	1.6649
4/20/2019 19:34	0.8	-0.1833	4.9868	-0.25	2.687	28.8636	0.1128	1.6529
4/20/2019 19:35	0.8167	-0.7333	5.2281	-0.0833	2.6868	28.8	0.1289	1.6315
4/20/2019 19:36	0.8	0.4333	5.2083	0.1167	2.6864	28.8	0.125	1.6263
4/20/2019 19:37	0.8	-0.5167	5.0833	-0.7	2.6926	28.7	0.1228	1.6498
4/20/2019 19:38	0.8083	0.0167	5.1503	-0.3667	2.6985	28.6583	0.126	1.6746
4/20/2019 19:39	0.8833	-0.95	4.8893	-0.1833	2.6881	28.18	0.12	1.6591
4/20/2019 19:40	0.8	-0.5167	5.2363	-0.6333	2.7077	27.1	0.1143	1.6636
4/20/2019 19:41	0.8583	-0.5	5.0144	0	2.6988	25.5083	0.1286	1.6811
4/20/2019 19:42	0.8	-0.35	4.7893	-0.7667	2.6958	23.7083	0.1162	1.6539
4/20/2019 19:43	0.8	0.1333	5.0936	0.3333	2.7067	21.9546	0.1317	1.6571
4/20/2019 19:44	0.8	-0.5667	5.3113	-0.2833	2.7108	20.2455	0.1145	1.666
4/20/2019 19:45	0.8333	-0.6667	5.1173	0.1167	2.6969	18.4833	0.1264	1.6634
4/20/2019 19:46	0.8583	-1.2	4.8963	-0.35	2.6993	16.7455	0.1315	1.6597
4/20/2019 19:47	0.8583	0.4833	5.0819	-0.05	2.7177	14.9091	0.1169	1.6481
4/20/2019 19:48	0.8583	0.0167	5.1802	-0.5	2.696	13.15	0.1315	1.6862
4/20/2019 19:49	0.8417	-0.7333	4.9624	0.1	2.7172	11.4833	0.1236	1.6909
4/20/2019 19:50	0.8	-0.1667	5.0573	-0.0833	2.683	9.75	0.1171	1.6492
4/20/2019 19:51	0.7833	-0.4667	5.1522	-0.7167	2.69	8.3909	0.1314	1.6315
4/20/2019 19:52	0.7	-0.0833	4.9824	0.2167	2.697	7.5091	0.1139	1.6428
4/20/2019 19:53	0.7	-0.3167	5.1111	-0.7667	2.6894	7.1364	0.1247	1.6519
4/20/2019 19:54	0.7	-0.1167	4.8897	-0.8	2.6898	7.1	0.1193	1.639
4/20/2019 19:55	0.7	0	5.2075	-0.4833	2.6849	7.1	0.1189	1.6432
4/20/2019 19:56	0.6917	0.3	4.7739	-0.4167	2.6902	7.1	0.1308	1.6595
4/20/2019 19:57	0.6833	0.4	5.0447	-0.7833	2.6645	7.1	0.1171	1.671
4/20/2019 19:58	0.6833	-0.2	5.0606	-0.6167	2.6753	7.1	0.1203	1.6898
4/20/2019 19:59	0.6583	0.4167	5.1422	-0.6167	2.6838	7.1	0.1198	1.6993
4/20/2019 20:00	0.6083	-0.45	5.0348	-0.0167	2.679	7.1	0.1136	1.6845
4/20/2019 20:01	0.6	0.25	4.7973	-0.0667	2.6778	7.1	0.1219	1.6713
4/20/2019 20:02	0.6	0.0667	4.9719	-0.4333	2.6945	7.1	0.113	1.6688
4/20/2019 20:03	0.6	0.2	5.1238	0.1	2.6824	7.1667	0.1213	1.6871
4/20/2019 20:04	0.6583	-0.0167	5.015	0	2.6934	7.2417	0.1237	1.6523
4/20/2019 20:05	0.7	-0.0167	5.0427	0.2833	2.6866	7.2	0.113	1.6388
4/20/2019 20:06	0.7	-0.55	4.8575	-0.25	2.6807	7.2	0.1268	1.6546
4/20/2019 20:07	0.7	-0.3167	5.1188	-0.2667	2.6656	7.3	0.1227	1.6812
4/20/2019 20:08	0.6833	-0.0667	5.0617	0.0833	2.6642	7.3	0.1133	1.6594
4/20/2019 20:09	0.6583	-0.2667	4.8718	-0.2167	2.6707	7.275	0.1255	1.6523

4/20/2019 20:10	0.675	-0.1	4.872	-0.5	2.6716	7.2	0.1148	1.6537
4/20/2019 20:11	0.7	-0.5333	4.9443	-0.25	2.6592	7.2	0.124	1.6706
4/20/2019 20:12	0.7	0.05	4.7805	-0.3167	2.6582	7.2	0.1289	1.6669
4/20/2019 20:13	0.7	0.5667	5.02	0.1333	2.6524	7.2182	0.1129	1.6669
4/20/2019 20:14	0.7	0.2	4.9283	-0.3333	2.6733	7.3	0.129	1.6703
4/20/2019 20:15	0.7	-0.6667	4.857	-0.2167	2.6718	7.2364	0.1114	1.6644
4/20/2019 20:16	0.8	-0.55	4.866	-0.4833	2.646	7.1	0.1245	1.6409
4/20/2019 20:17	0.8	0.1	5.004	0.2333	2.6607	7.1	0.1207	1.6694
4/20/2019 20:18	0.8	0.3167	5.0427	-0.3167	2.6683	7.0182	0.1206	1.6683
4/20/2019 20:19	0.8	-0.3	5.237	-0.05	2.6546	7	0.129	1.6663
4/20/2019 20:20	0.8	-0.3167	5.144	-0.1833	2.6363	7	0.1088	1.6649
4/20/2019 20:21	0.8	0.0167	4.8538	-0.2167	2.6482	7	0.1237	1.6873
4/20/2019 20:22	0.8	0.9333	5.3307	-0.0833	2.6404	7	0.1239	1.6613
4/20/2019 20:23	0.7917	-0.0667	5.0978	0.35	2.6421	7	0.1121	1.6713
4/20/2019 20:24	0.75	0.4167	5.0107	-0.05	2.6336	7	0.1262	1.663
4/20/2019 20:25	0.7667	0.2333	5.3325	-0.6667	2.6384	7	0.1262	1.6443
4/20/2019 20:26	0.7833	-0.85	5.169	0.1167	2.6597	6.99	0.1257	1.6573
4/20/2019 20:27	0.8	-0.5833	4.9887	0.3833	2.6484	6.9	0.1309	1.6601
4/20/2019 20:28	0.8	-0.5167	4.9078	-0.4333	2.6314	6.9	0.1216	1.676
4/20/2019 20:29	0.8333	-0.15	4.9707	-0.3833	2.6528	6.9	0.1322	1.6979
4/20/2019 20:30	0.8917	-0.8333	5.1477	0.0667	2.6531	6.9909	0.1245	1.6844
4/20/2019 20:31	0.9	-0.6167	5.0923	-0.0667	2.6305	7	0.1232	1.695
4/20/2019 20:32	0.85	0.3833	5.0857	0.1	2.6434	7	0.1303	1.6711
4/20/2019 20:33	0.8	0.1	4.9088	0.2333	2.6448	7	0.1212	1.6807
4/20/2019 20:34	0.8	-0.35	4.9952	-0.0333	2.6143	7	0.127	1.6977
4/20/2019 20:35	0.8	0.1	4.7723	-0.4	2.632	7	0.1277	1.6888
4/20/2019 20:36	0.8	-0.1167	4.7643	-0.1167	2.632	7	0.1135	1.6808
4/20/2019 20:37	0.875	-0.1	5.013	-0.1167	2.6153	7	0.1311	1.6763
4/20/2019 20:38	0.85	0.9167	5.0317	-0.3167	2.6047	7	0.1247	1.6677
4/20/2019 20:39	0.8	0.3	4.9857	-0.3167	2.6088	7	0.1259	1.6833
4/20/2019 20:40	0.8	-0.15	4.956	0.1167	2.6159	7.0333	0.1208	1.6669
4/20/2019 20:41	0.8	-0.0667	4.943	0.2333	2.6238	7.1	0.1087	1.6778
4/20/2019 20:42	0.8	0.2333	4.9562	-0.4167	2.6282	7.1	0.1226	1.6453
4/20/2019 20:43	0.8	0.55	4.7813	-0.1333	2.6225	7.1	0.1261	1.657
4/20/2019 20:44	0.8	-0.1167	5.2092	-0.3167	2.6294	7.1	0.1143	1.6658
4/20/2019 20:45	0.8	-0.3667	4.8967	0.05	2.6254	7.1	0.1272	1.6603
4/20/2019 20:46	0.8667	-0.0167	4.7913	-0.65	2.6091	7.1	0.1139	1.6596
4/20/2019 20:47	0.9	-0.15	4.804	-0.4	2.5947	7.1	0.1223	1.6455
4/20/2019 20:48	0.9	-0.35	4.7233	-0.2333	2.6042	7.1	0.1237	1.6713
4/20/2019 20:49	0.9	0.85	5.0177	-0.1833	2.6155	7.1	0.1149	1.6648
4/20/2019 20:50	0.9	0.1167	5.1348	-0.3667	2.6155	7.1	0.1334	1.6586
4/20/2019 20:51	0.9	-0.1333	5.0848	0.2333	2.6338	7.1	0.1186	1.6727
4/20/2019 20:52	0.9	-0.3167	5.2497	0.0833	2.6173	7.1	0.1185	1.6577
4/20/2019 20:53	0.9	-0.2167	4.6617	0.0833	2.6067	7.1	0.121	1.6691
4/20/2019 20:54	0.9	0.05	4.9713	-0.05	2.6111	7.1	0.1138	1.673
4/20/2019 20:55	0.8083	0	4.9462	-0.55	2.6038	7.0364	0.1262	1.6949
4/20/2019 20:56	0.8	-0.0667	4.9525	0.0333	2.6065	7	0.1284	1.697

4/20/2019 20:57	0.8	-0.05	4.9955	-0.2	2.6045	7	0.1179	1.6927
4/20/2019 20:58	0.875	0.0667	4.8633	-0.1	2.6213	7	0.1334	1.7112
4/20/2019 20:59	0.9	-0.25	4.7747	-0.1	2.6127	7	0.1198	1.6853
4/20/2019 21:00	0.9	-0.2833	5.1182	-0.25	2.5931	7	0.1327	1.6751
4/20/2019 21:01	0.8	0.0667	4.9935	-0.2167	2.6082	7	0.1261	1.6604
4/20/2019 21:02	0.8	0.15	4.8852	-0.8	2.623	7	0.1198	1.6718
4/20/2019 21:03	0.8	-0.3	4.7128	-0.8167	2.6263	7	0.1318	1.6609
4/20/2019 21:04	0.8	-0.45	4.7935	0.4667	2.6069	7	0.1164	1.664
4/20/2019 21:05	0.8	0.6	4.637	0.25	2.6157	7	0.1296	1.6788
4/20/2019 21:06	0.8	0.1333	4.8983	-0.2333	2.6203	7	0.1317	1.6623
4/20/2019 21:07	0.7917	-0.1833	4.7257	0.1667	2.6328	7	0.1201	1.6708
4/20/2019 21:08	0.725	0.1833	4.8637	-0.35	2.6172	7	0.1312	1.685
4/20/2019 21:09	0.75	-0.2667	4.7773	-0.2667	2.6044	7.1	0.1244	1.6748
4/20/2019 21:10	0.7	0	4.968	-0.3833	2.6265	7.1	0.12	1.6775
4/20/2019 21:11	0.7	-0.3833	4.6863	-0.65	2.6183	7.1	0.1331	1.6865
4/20/2019 21:12	0.7	0.4333	4.7482	-0.2833	2.6292	7.1	0.1163	1.6812
4/20/2019 21:13	0.7	0.6167	4.9987	-0.6667	2.6411	7.1	0.1318	1.6804
4/20/2019 21:14	0.7	0.0333	4.6328	-0.1833	2.6414	7.1	0.1238	1.6799
4/20/2019 21:15	0.7	0.35	4.7107	0.0667	2.6407	7.1	0.1227	1.6843
4/20/2019 21:16	0.75	-0.0667	4.759	0.0167	2.6309	7.1	0.1362	1.6978
4/20/2019 21:17	0.8	-0.0333	4.9363	-0.2	2.633	7.0182	0.1208	1.7142
4/20/2019 21:18	0.8167	-0.65	4.965	-0.25	2.6348	7.0917	0.1243	1.7118
4/20/2019 21:19	0.8333	0.15	4.618	0.2	2.6455	7.075	0.1303	1.7029
4/20/2019 21:20	0.85	0.1833	4.9302	-0.2667	2.6705	7.1	0.1218	1.687
4/20/2019 21:21	0.8	0.2833	5.022	0.1	2.6957	7.0333	0.1365	1.6635
4/20/2019 21:22	0.8	0.2	4.8818	-0.4	2.6803	7	0.1211	1.6706
4/20/2019 21:23	0.8	0.1833	5.097	-0.1	2.6734	7	0.1232	1.6576
4/20/2019 21:24	0.8	0.45	4.831	0.8	2.6641	7	0.1376	1.6878
4/20/2019 21:25	0.8	0.1667	4.7702	-0.2833	2.7272	6.9083	0.1304	1.6896
4/20/2019 21:26	0.825	0.1	4.7215	-0.3833	2.7168	6.9	0.1329	1.69
4/20/2019 21:27	0.8833	0.0333	4.9025	-0.35	2.6986	6.8667	0.1277	1.6986
4/20/2019 21:28	0.9	-0.4833	4.5977	-0.25	2.7318	6.84	0.1222	1.6963
4/20/2019 21:29	0.975	-0.3333	4.609	0.3167	2.7217	6.9	0.1393	1.6733
4/20/2019 21:30	1	-0.0667	4.9772	0.2	2.7244	6.9	0.1261	1.6673
4/20/2019 21:31	0.9917	0.3667	4.6278	0.3333	2.7161	6.9	0.133	1.6738
4/20/2019 21:32	1	-0.4333	4.9485	0.1833	2.7118	6.9	0.1306	1.6786
4/20/2019 21:33	1	0.25	5.003	0.1833	2.7248	6.8667	0.1252	1.6745
4/20/2019 21:34	1.0167	-0.3167	4.7747	0.2833	2.7339	6.8	0.1365	1.6739
4/20/2019 21:35	1	0.2667	4.8293	-0.3	2.7303	6.8909	0.1243	1.6837
4/20/2019 21:36	1	-0.2167	4.845	-0.1333	2.727	6.9	0.1278	1.6832
4/20/2019 21:37	1	0.3167	4.8613	-0.05	2.727	6.9	0.1401	1.7033
4/20/2019 21:38	1	0.1167	4.8473	-0.4333	2.7412	6.9	0.122	1.7213
4/20/2019 21:39	1.0833	0.4667	4.9099	-0.0667	2.7248	6.9	0.1396	1.7276
4/20/2019 21:40	1.1	-0.05	4.9475	-0.5833	2.737	6.9	0.1281	1.7264
4/20/2019 21:41	1.1	-0.5833	4.6704	0.45	2.7354	6.9	0.136	1.7225
4/20/2019 21:42	1.0917	-0.2	4.9098	-0.1	2.7314	6.9	0.1375	1.7274
4/20/2019 21:43	1.0333	0.2667	4.6576	0.0667	2.7282	6.9	0.1267	1.7446

4/20/2019 21:44	1	-0.8167	4.8243	-0.0667	2.7576	6.9	0.1351	1.752
4/20/2019 21:45	1	0.05	4.6824	-0.15	2.734	6.9	0.1314	1.7409
4/20/2019 21:46	0.9833	-0.55	4.6301	0.15	2.7267	6.9636	0.1282	1.735
4/20/2019 21:47	0.9	0.1667	4.6003	0.3667	2.7236	6.9	0.1387	1.7053
4/20/2019 21:48	0.9	-0.35	4.6527	-0.1833	2.7251	6.9	0.1284	1.7059
4/20/2019 21:49	0.8583	-0.4667	4.5855	0.1167	2.7303	6.9	0.1412	1.6975
4/20/2019 21:50	0.9	-0.2167	4.9078	0	2.7318	6.9	0.1318	1.6969
4/20/2019 21:51	0.9	-0.3167	4.6945	-0.7167	2.741	6.9	0.137	1.6768
4/20/2019 21:52	0.9	0.3667	4.8118	-0.35	2.7143	6.9	0.1394	1.6881
4/20/2019 21:53	0.9	-0.3	4.9123	-0.4333	2.7056	6.9	0.1315	1.6927
4/20/2019 21:54	0.9	-0.2667	4.9751	0.3833	2.699	6.9	0.1379	1.6986
4/20/2019 21:55	0.8583	0.6	4.7922	0.0333	2.7043	6.9	0.1318	1.7121
4/20/2019 21:56	0.8	0.2833	4.5072	-0.0167	2.7131	6.9	0.1349	1.7054
4/20/2019 21:57	0.7917	-0.0167	4.3498	-0.0167	2.7182	6.9	0.1368	1.7073
4/20/2019 21:58	0.8	0.1	4.5018	-0.6	2.7151	6.9	0.1281	1.7156
4/20/2019 21:59	0.8	0.4167	4.8738	-0.0667	2.7176	6.9	0.1302	1.7124
4/20/2019 22:00	0.8	-0.3167	4.6472	-0.3167	2.7245	6.9	0.1358	1.7288
4/20/2019 22:01	0.8	0.8833	4.5825	-0.0667	2.7251	6.9	0.1264	1.7178
4/20/2019 22:02	0.8	-0.1167	4.6991	-0.3833	2.7046	6.9	0.1385	1.7231
4/20/2019 22:03	0.8	0.3333	4.5021	-0.2833	2.7132	6.9	0.1215	1.6959
4/20/2019 22:04	0.85	0.4167	4.5236	0	2.6863	6.9364	0.1412	1.7025
4/20/2019 22:05	0.9	-0.3667	4.7204	-0.4667	2.6962	7	0.1264	1.7132
4/20/2019 22:06	0.8917	-0.0667	4.6298	-0.2	2.6938	7	0.1294	1.6954
4/20/2019 22:07	0.8583	-0.1167	4.6327	-0.1833	2.6935	7	0.1361	1.7102
4/20/2019 22:08	0.8	0.1667	4.7475	-0.1667	2.7161	7	0.1236	1.7272
4/20/2019 22:09	0.8	0.25	4.7368	-0.2333	2.7013	7	0.1338	1.7222
4/20/2019 22:10	0.8333	-0.7833	4.6585	-0.55	2.69	7	0.1289	1.7284
4/20/2019 22:11	0.825	-0.0833	4.8678	0.05	2.6783	7	0.1323	1.7665
4/20/2019 22:12	0.8083	-0.3333	4.5713	-0.6	2.6658	7.0273	0.1406	1.7764
4/20/2019 22:13	0.75	0.1833	4.4198	-0.45	2.6525	7.0091	0.1201	1.7713
4/20/2019 22:14	0.7	0.0167	4.4575	-0.8333	2.6597	7.1	0.1303	1.7643
4/20/2019 22:15	0.7583	-0.1833	4.4998	-0.2	2.656	7.1	0.1309	1.7618
4/20/2019 22:16	0.8083	0.1	4.3406	-0.5	2.6548	7.1	0.129	1.7654
4/20/2019 22:17	0.9	-0.2	4.5983	-0.2	2.6646	7.1	0.1378	1.7386
4/20/2019 22:18	0.9	-0.1167	4.6805	-0.2833	2.6828	7.0364	0.1251	1.7485
4/20/2019 22:19	0.875	-0.7667	4.7313	-0.5667	2.6553	7	0.1389	1.7335
4/20/2019 22:20	0.8167	0.1167	4.5266	-0.2833	2.6608	7	0.1387	1.7574
4/20/2019 22:21	0.8083	-0.2167	4.6688	-0.55	2.6581	7	0.1295	1.7468
4/20/2019 22:22	0.8	-0.5	4.6113	-0.3333	2.6246	7	0.1362	1.7377
4/20/2019 22:23	0.8	-0.45	4.3672	-0.2667	2.6292	7	0.1219	1.7229
4/20/2019 22:24	0.825	-0.4167	4.6104	-0.6167	2.655	7	0.1336	1.7301
4/20/2019 22:25	0.9	0.1	4.7832	-0.8	2.6541	7	0.1306	1.7518
4/20/2019 22:26	0.9	-0.3	4.5003	-0.8167	2.6453	7	0.1284	1.7386
4/20/2019 22:27	0.9	0.5167	4.4905	-0.5167	2.6384	6.9727	0.1339	1.7368
4/20/2019 22:28	0.9	0.2167	4.5423	-0.5333	2.6236	6.93	0.1299	1.7406
4/20/2019 22:29	0.8667	0.15	4.4875	-0.4	2.6168	6.9	0.1368	1.732
4/20/2019 22:30	0.8417	-0.0167	4.3805	-0.4333	2.5858	6.9364	0.1287	1.7372

4/20/2019 22:31	0.8	-0.0167	4.0986	-0.6333	2.6066	7	0.1304	1.7526
4/20/2019 22:32	0.8	-0.0667	4.3848	8.0167	2.6108	7	0.1337	1.7443
4/20/2019 22:33	0.7417	-0.1667	4.5172	-0.5333	2.5951	7	0.1185	1.7333
4/20/2019 22:34	0.7	0.0833	4.3886	-0.4083	2.5865	7	0.1308	1.7498
4/20/2019 22:35	0.65	0.0167	4.5508	0.0167	2.5955	7	0.1244	1.7271
4/20/2019 22:36	0.6	-0.2	4.3806	-0.125	2.5849	7	0.1287	1.7278
4/20/2019 22:37	0.6083	-0.5833	4.4418	0.2083	2.6002	7	0.134	1.7093
4/20/2019 22:38	0.6	-0.5167	4.3443	0.7583	2.601	7	0.1251	1.7353
4/20/2019 22:39	0.575	0.05	4.5678		2.5809	7	0.1437	1.7328
4/20/2019 22:40	0.5333	-0.2167	4.3269		2.5558	7.0182	0.1233	1.753
4/20/2019 22:41	0.575	-0.15	4.4664		2.5488	7.1	0.1215	1.7679
4/20/2019 22:42	0.6	0.05	4.3658		2.5481	7.1	0.1319	1.7146
4/20/2019 22:43	0.6	-0.2833	4.4442		2.5348	7.1	0.1272	1.7226
4/20/2019 22:44	0.6	0.0667	4.663		2.5393	7.0727	0.1358	1.7321
4/20/2019 22:45	0.6	0.1833	4.6082		2.5198	7.1	0.1332	1.7425
4/20/2019 22:46	0.5167	-0.5167	4.3486	1	2.5218	7.0083	0.1209	1.762
4/20/2019 22:47	0.5	0.0167	4.4009	-0.0833	2.5258	7.1	0.1334	1.7403
4/20/2019 22:48	0.5417	-0.05	4.4469	-0.1	2.5077	7.0333	0.1178	1.7414
4/20/2019 22:49	0.6	1.1167	4.581	-0.2375	2.5136	7	0.1392	1.7335
4/20/2019 22:50	0.6	-0.0167	4.0764	0.0286	2.5075	7.0583	0.1247	1.7243
4/20/2019 22:51	0.6	0.0333	4.2773	-0.625	2.513	7.0091	0.1272	1.742
4/20/2019 22:52	0.6	-0.2	4.4817	1.4833	2.4997	7	0.1364	1.7378
4/20/2019 22:53	0.6	0.0167	4.5723	1.3333	2.4917	7.04	0.123	1.7403
4/20/2019 22:54	0.6	-0.9833	4.5284	0.6833	2.4824	7.0583	0.1323	1.7438
4/20/2019 22:55	0.6	-0.3167	4.3476	-0.2333	2.4968	7	0.1239	1.7343
4/20/2019 22:56	0.6	-0.6333	4.4784	-0.1	2.4768	7.0909	0.1306	1.718
4/20/2019 22:57	0.6	-0.4667	4.4292	0.2583	2.4717	7.025	0.1365	1.7045
4/20/2019 22:58	0.6583	-0.8333	4.5108	0.05	2.4814	7.1	0.1166	1.7464
4/20/2019 22:59	0.7	-0.1333	4.3833	-0.0417	2.4623	7.1	0.1382	1.7569
4/20/2019 23:00	0.7	-0.2167	4.7068	0.1667	2.4618	7.1	0.1253	1.7419
4/20/2019 23:01	0.65	-0.2667	4.3875	-0.3333	2.4483	7.1	0.1202	1.7548
4/20/2019 23:02	0.6167	-0.2	4.6224	-0.0333	2.4493	7.1	0.1317	1.7491
4/20/2019 23:03	0.6167	-0.3833	4.3568	-0.65	2.4462	7.1	0.1173	1.7682
4/20/2019 23:04	0.6	-0.9833	4.5352	-0.1833	2.4482	7.1	0.133	1.7627
4/20/2019 23:05	0.6	-0.25	4.4423	0.25	2.4366	7.1	0.121	1.7551
4/20/2019 23:06	0.65	-0.55	4.6848	-0.2833	2.4538	7.1	0.1177	1.7769
4/20/2019 23:07	0.7	-0.6833	4.2903	-0.25	2.4382	7.1	0.1253	1.7582
4/20/2019 23:08	0.7	-1.15	4.6028	-0.5	2.4366	7.1	0.1118	1.736
4/20/2019 23:09	0.7	-0.8	4.3375	-0.625	2.4375	7.1818	0.1378	1.7354
4/20/2019 23:10	0.6917	-0.3833	4.3955	-0.5333	2.4102	7.1091	0.1293	1.7352
4/20/2019 23:11	0.6	-0.15	4.5157	-0.825	2.4104	7.1	0.1219	1.7394
4/20/2019 23:12	0.6	-0.4167	4.349	0.025	2.4188	7.1833	0.1319	1.7329
4/20/2019 23:13	0.6	0	4.3373	-0.3583	2.4193	7.2	0.1185	1.7279
4/20/2019 23:14	0.6	0.0667	4.3068	-0.2083	2.4302	7.2	0.1287	1.7253
4/20/2019 23:15	0.5083	-1.3	4.4603	-0.1583	2.4022	7.3	0.1169	1.7452
4/20/2019 23:16	0.5	0.4	4.4355	-0.3167	2.3928	7.3	0.1231	1.7471
4/20/2019 23:17	0.5	-0.4167	4.251	-0.075	2.3778	7.3	0.1305	1.7451

4/20/2019 23:18	0.5	-0.4167	4.3983	-0.1667	2.3909	7.3	0.1173	1.7207
4/20/2019 23:19	0.5	-0.35	4.411	0.1583	2.3853	7.3	0.1284	1.7327
4/20/2019 23:20	0.5	-0.1833	4.2658	-0.5167	2.3748	7.3	0.1196	1.7292
4/20/2019 23:21	0.5	-0.3	4.2198	0.0167	2.381	7.3	0.121	1.7353
4/20/2019 23:22	0.5583	-0.5667	4.2855	-0.6333	2.3723	7.3	0.1307	1.7388
4/20/2019 23:23	0.6	0.5833	4.2732	-0.4417	2.3746	7.3	0.1107	1.7506
4/20/2019 23:24	0.6	-0.0333	4.4993	-0.375	2.3684	7.2333	0.1263	1.7612
4/20/2019 23:25	0.5417	-0.5667	4.5172	-0.6833	2.3604	7.2	0.128	1.7671
4/20/2019 23:26	0.5	0.35	4.3505	-0.05	2.3522	7.2	0.1203	1.7216
4/20/2019 23:27	0.5667	-0.95	4.4713	-0.2417	2.3523	7.1364	0.1285	1.7112
4/20/2019 23:28	0.6	-0.55	4.8537	0.2167	2.3569	7.1818	0.1189	1.7223
4/20/2019 23:29	0.5833	-0.0667	4.5145	-0.175	2.3355	7.19	0.1157	1.7353
4/20/2019 23:30	0.5	-0.6833	4.4635	-0.5167	2.3447	7.1	0.1287	1.7261
4/20/2019 23:31	0.575	-0.8667	4.5795	-0.1667	2.3348	7.1	0.1198	1.7386
4/20/2019 23:32	0.6083	-0.7667	4.271	-0.3667	2.3394	7.1	0.1216	1.763
4/20/2019 23:33	0.7	-0.1167	4.319	-0.8917	2.3346	7.1	0.1179	1.7493
4/20/2019 23:34	0.7	0.3833	4.396	-0.2167	2.3232	7.1	0.1339	1.7543
4/20/2019 23:35	0.7	-0.45	4.3988	-0.3917	2.336	7.1	0.1293	1.7525
4/20/2019 23:36	0.7	-0.1667	4.1857	-0.4333	2.3201	7.1	0.117	1.7276
4/20/2019 23:37	0.7667	-0.3833	4.442	-0.5167	2.322	7.1	0.1304	1.7477
4/20/2019 23:38	0.8	0.25	4.4178	0.1417	2.2903	7.1	0.1171	1.741
4/20/2019 23:39	0.8	0	4.5522	-0.6333	2.29	7.125	0.119	1.7467
4/20/2019 23:40	0.725	-0.8167	4.4813	-0.725	2.277	7.1	0.1314	1.7948
4/20/2019 23:41	0.7	0.05	4.3323	-0.4667	2.2608	7.1	0.1105	1.8058
4/20/2019 23:42	0.7	-1.05	4.5738	-0.4167	2.2733	7.1	0.1291	1.7837
4/20/2019 23:43	0.7	-0.35	4.3025	-1.0167	2.2808	7.1	0.1199	1.7604
4/20/2019 23:44	0.7	-0.1333	4.4935	-0.05	2.2571	7.1091	0.1238	1.7459
4/20/2019 23:45	0.7417	-0.8	4.6262	-0.3	2.2421	7.2	0.1265	1.7823
4/20/2019 23:46	0.7417	-0.2333	4.694	-0.775	2.2677	7.2	0.1124	1.8104
4/20/2019 23:47	0.8	0.2333	4.5512	-0.1917	2.2417	7.2	0.1293	1.7827
4/20/2019 23:48	0.8	-0.15	4.4237	-0.2	2.2523	7.2	0.12	1.7727
4/20/2019 23:49	0.8	0.0167	4.0203	-0.3583	2.2513	7.2	0.1187	1.7628
4/20/2019 23:50	0.7583	0.2333	4.1603	-0.175	2.2478	7.2	0.1228	1.7621
4/20/2019 23:51	0.7	-0.3333	4.1332	-0.0917	2.2344	7.2	0.115	1.7743
4/20/2019 23:52	0.65	0.0833	4.5882	-0.5167	2.2308	7.2	0.1261	1.781
4/20/2019 23:53	0.6	-0.3167	4.4963	-0.3917	2.2436	7.15	0.1185	1.8003
4/20/2019 23:54	0.625	-0.4667	4.6002	-0.6833	2.2303	7.2	0.1257	1.8205
4/20/2019 23:55	0.7	-0.7333	4.245	-0.5417	2.2369	7.2	0.1226	1.7664
4/20/2019 23:56	0.6583	-0.45	4.5082	0.425	2.2186	7.1583	0.1188	1.7457
4/20/2019 23:57	0.6	-0.0167	4.3877	-0.375	2.2224	7.1	0.1348	1.7568
4/20/2019 23:58	0.6	-0.6167	4.4813	-0.9167	2.2358	7.1	0.1238	1.7395
4/20/2019 23:59	0.65	-0.45	4.2627	-0.3083	2.2251	7.1	0.1191	1.7711
4/21/2019 0:00	0.6583	-0.2167	4.5007	-0.85	2.2179	7.1	0.1266	1.7548
4/21/2019 0:01	0.6	0.4167	4.5278	-0.1833	2.2056	7.125	0.113	1.7426
4/21/2019 0:02	0.55	-0.5333	4.375	-0.175	2.2055	7.1909	0.1225	1.7632
4/21/2019 0:03	0.5	-0.3833	4.2343	-0.4167	2.1962	7.1	0.1209	1.7824
4/21/2019 0:04	0.5	0.0833	4.6253	0.3667	2.1911	7.1	0.1177	1.776

4/21/2019 0:05	0.5	0.05	4.563	-0.475	2.1849	7.1083	0.131	1.7758
4/21/2019 0:06	0.5	-0.2333	4.5615	-0.8333	2.1771	7.2	0.1182	1.7868
4/21/2019 0:07	0.5	-0.4667	4.4753	0	2.1858	7.2	0.1185	1.7671
4/21/2019 0:08	0.55	-0.3167	4.3988	-0.4	2.1872	7.2167	0.121	1.7711
4/21/2019 0:09	0.6	-0.0167	4.5173	-0.6083	2.1862	7.3	0.1132	1.7838
4/21/2019 0:10	0.6	0.1833	4.8308	-0.4083	2.1713	7.3	0.125	1.7776
4/21/2019 0:11	0.6	-0.2167	4.7802	-0.675	2.1583	7.3	0.1124	1.7633
4/21/2019 0:12	0.6	-0.75	4.46	-0.4833	2.1747	7.3	0.1256	1.7652
4/21/2019 0:13	0.6	-0.2833	4.3633	-0.5167	2.1691	7.3	0.1199	1.7739
4/21/2019 0:14	0.6	-0.3167	4.426	-0.3333	2.1582	7.3	0.1125	1.7881
4/21/2019 0:15	0.6	-0.1667	4.4257	0.0417	2.1694	7.3	0.127	1.7902
4/21/2019 0:16	0.6	-0.2833	4.39	0.0417	2.179	7.3	0.1157	1.8149
4/21/2019 0:17	0.6	-0.25	4.4313	0.0583	2.1623	7.3	0.1186	1.7794
4/21/2019 0:18	0.6	-0.0667	4.8583	-0.1167	2.1438	7.3	0.1266	1.7899
4/21/2019 0:19	0.6333	-0.4833	4.8185	0.0333	2.1413	7.3	0.1062	1.7973
4/21/2019 0:20	0.7	-0.35	4.6258	0.0667	2.1272	7.225	0.1213	1.7833
4/21/2019 0:21	0.7	-0.4167	4.3793	-0.1333	2.1168	7.2	0.1234	1.768
4/21/2019 0:22	0.7333	-0.35	4.4297	-1.0417	2.1089	7.2667	0.1178	1.772
4/21/2019 0:23	0.7	-0.3167	4.6202	-0.7417	2.1205	7.2091	0.1256	1.7868
4/21/2019 0:24	0.6833	-0.5333	4.5062	-0.925	2.1236	7.2	0.1075	1.8057
4/21/2019 0:25	0.6	0.25	4.2923	-0.2833	2.1118	7.2	0.1279	1.8106
4/21/2019 0:26	0.5667	-0.3833	4.589	-0.2917	2.1254	7.2917	0.1205	1.8059
4/21/2019 0:27	0.5	-0.4	4.668	-0.3417	2.1067	7.3	0.1185	1.8007
4/21/2019 0:28	0.5667	-0.45	4.8048	-0.275	2.1123	7.3	0.1304	1.7646
4/21/2019 0:29	0.6167	-0.3667	4.329	-0.5	2.1121	7.3	0.1163	1.8022
4/21/2019 0:30	0.7	-0.6667	4.6052	-0.3583	2.0939	7.3	0.1308	1.7856
4/21/2019 0:31	0.7	-0.25	4.5757	-0.7167	2.1057	7.3	0.1253	1.7707
4/21/2019 0:32	0.7	-0.1167	4.6155	-0.575	2.0839	7.3	0.1148	1.7709
4/21/2019 0:33	0.6417	-0.85	4.8105	-0.7333	2.1127	7.3667	0.13	1.7636
4/21/2019 0:34	0.6	-0.85	4.7304	-0.05	2.0803	7.4	0.1061	1.758
4/21/2019 0:35	0.5583	-0.6667	4.6461	-0.6417	2.1088	7.4	0.117	1.7573
4/21/2019 0:36	0.5	-0.7333	4.683	-0.05	2.101	7.4	0.1277	1.8028
4/21/2019 0:37	0.5	-0.1167	4.6288	-0.5833	2.0853	7.4	0.1135	1.7991
4/21/2019 0:38	0.5917	-0.6	4.5518	-0.4083	2.0979	7.4	0.1297	1.7824
4/21/2019 0:39	0.6	0.15	4.5594	-0.3833	2.1064	7.4	0.1125	1.8004
4/21/2019 0:40	0.6	-0.25	4.6688	-0.7917	2.109	7.4	0.1231	1.8148
4/21/2019 0:41	0.6	0.2	4.8693	-0.0667	2.1053	7.45	0.1275	1.7762
4/21/2019 0:42	0.6583	-0.15	4.6358	-0.2583	2.0822	7.5	0.111	1.7865
4/21/2019 0:43	0.7333	-0.4	4.6425	-0.325	2.0885	7.5	0.123	1.8188
4/21/2019 0:44	0.8	-0.4833	4.5033	-0.2417	2.0925	7.5	0.1167	1.8277
4/21/2019 0:45	0.7917	0.5333	4.8241	-0.025	2.0801	7.5	0.1117	1.8305
4/21/2019 0:46	0.7417	-0.6	4.5049	-0.5333	2.0727	7.5	0.1205	1.8014
4/21/2019 0:47	0.7083	-0.3333	4.3591	-0.5667	2.0739	7.5636	0.1146	1.8129
4/21/2019 0:48	0.7	-0.45	4.3813	-0.0083	2.0619	7.6	0.1241	1.8613
4/21/2019 0:49	0.7	0.0333	4.7051	-0.1417	2.084	7.6	0.1162	1.8135
4/21/2019 0:50	0.7	-0.0667	4.5496	-0.6417	2.0632	7.6	0.1216	1.7857
4/21/2019 0:51	0.7583	-0.1	4.5205	-0.85	2.0644	7.6	0.1259	1.7735

4/21/2019 0:52	0.8	0.2333	4.4521	-0.7667	2.0671	7.6	0.11	1.7863
4/21/2019 0:53	0.8	0.3	4.8563	0.1833	2.0581	7.6	0.1324	1.822
4/21/2019 0:54	0.8	-0.5833	4.4743	-0.825	2.0628	7.6	0.1175	1.8174
4/21/2019 0:55	0.8	-0.45	4.6091	-1.025	2.0609	7.5333	0.1149	1.8008
4/21/2019 0:56	0.8	-0.6833	4.7117	-0.325	2.062	7.5	0.1183	1.8092
4/21/2019 0:57	0.8	-0.3	4.7214	-0.6583	2.0576	7.5	0.1106	1.8391
4/21/2019 0:58	0.8	-0.9333	4.5498	-0.1333	2.0517	7.5	0.1271	1.8125
4/21/2019 0:59	0.8	-0.2833	4.422	-0.5333	2.0559	7.5	0.1117	1.7852
4/21/2019 1:00	0.8	-0.3333	4.7758	-0.075	2.064	7.5083	0.1167	1.7983
4/21/2019 1:01	0.8	-0.75	4.7721	-0.5917	2.0675	7.5636	0.1324	1.8313
4/21/2019 1:02	0.8	0.25	4.6767	-0.5167	2.0549	7.5	0.1117	1.841
4/21/2019 1:03	0.7417	-0.4667	4.5428	-0.6333	2.0628	7.5	0.1267	1.8293
4/21/2019 1:04	0.7833	-0.3667	4.6671	-0.0583	2.0482	7.5	0.1195	1.8406
4/21/2019 1:05	0.775	-0.2333	4.6033	-0.425	2.0416	7.5167	0.1152	1.8208
4/21/2019 1:06	0.8	0.45	4.4247	-0.625	2.0436	7.5091	0.1233	1.8121
4/21/2019 1:07	0.85	0.0833	4.212	-0.5667	2.0353	7.5	0.1111	1.8218
4/21/2019 1:08	0.9	-0.25	4.8194	-0.925	2.0334	7.5	0.1267	1.8129
4/21/2019 1:09	0.8417	-0.1833	4.328	-0.6083	2.0433	7.5	0.1188	1.8324
4/21/2019 1:10	0.7917	-0.7833	4.6506	-0.35	2.0485	7.5	0.1124	1.8476
4/21/2019 1:11	0.7	-0.1167	4.5648	-0.6583	2.0478	7.5	0.1258	1.8163
4/21/2019 1:12	0.7	-0.15	4.5321	-0.675	2.0453	7.5	0.1118	1.8268
4/21/2019 1:13	0.7	-0.15	4.7275	-0.25	2.0536	7.4818	0.1148	1.8117
4/21/2019 1:14	0.675	-0.45	4.5576	0.0417	2.034	7.4364	0.1219	1.8317
4/21/2019 1:15	0.7	-0.2167	4.7598	-0.5667	2.0443	7.4182	0.11	1.8357
4/21/2019 1:16	0.7167	-0.3167	4.7708	-0.0583	2.0303	7.5	0.1258	1.8663
4/21/2019 1:17	0.8	-0.6167	4.4185	-0.425	2.0419	7.5	0.1211	1.8588
4/21/2019 1:18	0.8	-0.7167	4.4688	-0.3667	2.0371	7.4417	0.1223	1.8223
4/21/2019 1:19	0.7333	-0.15	4.5588	-0.6333	2.0193	7.5	0.1244	1.8158
4/21/2019 1:20	0.7	-0.15	4.9228	-0.7333	2.0416	7.4455	0.1105	1.8369
4/21/2019 1:21	0.7	-0.3	4.7454	-0.275	2.0263	7.4	0.1221	1.8323
4/21/2019 1:22	0.65	-0.4333	5.1413	-0.5	2.0374	7.4	0.1182	1.8343
4/21/2019 1:23	0.675	-0.4833	4.7065	0.0583	2.0308	7.4	0.1137	1.8599
4/21/2019 1:24	0.7	-0.3667	4.4823	-0.4083	2.028	7.4	0.1292	1.855
4/21/2019 1:25	0.7	-0.55	4.6498	-0.525	2.019	7.4	0.1104	1.8595
4/21/2019 1:26	0.7	-0.3833	4.6715	-0.4083	2.0223	7.4	0.1241	1.8535
4/21/2019 1:27	0.7	-0.2	4.5472	-0.4417	2.047	7.4	0.1189	1.8778
4/21/2019 1:28	0.7	0.6833	4.437	-0.2667	2.0353	7.4	0.1228	1.8503
4/21/2019 1:29	0.7	-1.3833	4.7473	-0.3667	2.0419	7.4	0.1253	1.8593
4/21/2019 1:30	0.65	-0.1833	4.6978	-0.525	2.0414	7.4	0.109	1.8633
4/21/2019 1:31	0.6	-0.2333	4.3736	0.3083	2.0411	7.4	0.1257	1.8747
4/21/2019 1:32	0.6	-0.9833	4.7022	0.3583	2.0158	7.4	0.1184	1.8473
4/21/2019 1:33	0.5917	-0.3333	4.511	0.0083	2.0182	7.4	0.1059	1.8481
4/21/2019 1:34	0.5	-0.3167	4.459	-0.4	2.0341	7.4	0.1273	1.847
4/21/2019 1:35	0.5	-1.0333	4.5471	-0.2917	2.0433	7.4	0.1145	1.8513
4/21/2019 1:36	0.5	0.3333	4.6703	-0.475	2.0259	7.4	0.1271	1.8437
4/21/2019 1:37	0.5083	-0.7667	4.3418	-0.3417	2.0043	7.4	0.1176	1.8444
4/21/2019 1:38	0.6	-0.5333	4.2135	-0.275	2.0316	7.4	0.1142	1.8544

4/21/2019 1:39	0.55	-0.2167	4.6923	-0.2583	2.0145	7.4	0.1303	1.843
4/21/2019 1:40	0.5	-0.55	4.5408	-0.225	2.0114	7.4	0.1105	1.8691
4/21/2019 1:41	0.5	-0.35	4.9441	-0.85	2.017	7.4	0.1226	1.8641
4/21/2019 1:42	0.575	-0.6	4.6507	-0.6	2.0206	7.4	0.1208	1.8548
4/21/2019 1:43	0.6	0.4167	4.5278	-0.0417	2.0308	7.45	0.1158	1.8777
4/21/2019 1:44	0.625	-0.55	4.6114	-0.9083	2.0168	7.4	0.1245	1.8525
4/21/2019 1:45	0.65	-0.1333	4.4787	-0.8167	2.0345	7.5	0.1156	1.8668
4/21/2019 1:46	0.6	-0.0833	4.5892	-0.8333	2.0378	7.5	0.1264	1.8663
4/21/2019 1:47	0.6583	0.5667	4.4774	-0.55	2.0299	7.5	0.1194	1.8948
4/21/2019 1:48	0.675	-0.8	4.4594	-0.1833	2.0584	7.5	0.1133	1.9028
4/21/2019 1:49	0.6167	-0.3333	4.7746	-0.2	2.0413	7.59	0.1298	1.9003
4/21/2019 1:50	0.6	-0.4833	4.5236	-0.1583	2.0539	7.6	0.1133	1.8924
4/21/2019 1:51	0.6	-0.4167	4.6133	-0.3417	2.0338	7.6	0.1271	1.8781
4/21/2019 1:52	0.6	-0.6	4.5941	-0.3917	2.0338	7.6667	0.127	1.8827
4/21/2019 1:53	0.6	-0.2833	4.79	-0.2	2.0524	7.6182	0.1126	1.8882
4/21/2019 1:54	0.6	-0.3667	4.7403	0.1917	2.0323	7.6	0.1253	1.8928
4/21/2019 1:55	0.6	0.15	4.7746	-0.1333	2.0501	7.6	0.1104	1.8943
4/21/2019 1:56	0.6	-0.4333	4.4798	-0.5917	2.0682	7.6	0.1206	1.883
4/21/2019 1:57	0.6	-0.0833	4.626	-0.275	2.0558	7.6	0.125	1.9103
4/21/2019 1:58	0.5455	0.05	4.7268	-0.4167	2.0583	7.6	0.1139	1.8817
4/21/2019 1:59	0.5833	-0.9333	4.8595	-0.8833	2.0473	7.6	0.1223	1.8937
4/21/2019 2:00	0.525	-0.4	4.7469	-0.5667	2.0843	7.6273	0.121	1.8873
4/21/2019 2:01	0.5	-0.5833	4.5969	-0.025	2.074	7.7	0.1214	1.8749
4/21/2019 2:02	0.525	-0.0833	4.6159	-0.15	2.0833	7.7	0.1222	1.9098
4/21/2019 2:03	0.575	-0.7333	4.6822	-0.3167	2.0558	7.7	0.1083	1.9063
4/21/2019 2:04	0.6	-0.05	4.9742	-0.675	2.0552	7.7	0.1303	1.9041
4/21/2019 2:05	0.525	0.3	4.8945	-0.5083	2.0413	7.7	0.1128	1.9084
4/21/2019 2:06	0.5	-0.3667	4.7973	-0.7167	2.041	7.7	0.1253	1.9178
4/21/2019 2:07	0.5	-1.05	4.8785	-0.9	2.0498	7.7	0.1207	1.8894
4/21/2019 2:08	0.5417	-0.4833	4.6097	-0.5417	2.0601	7.7	0.1144	1.8993
4/21/2019 2:09	0.6	0.0333	4.586	-0.325	2.0643	7.7	0.1238	1.8913
4/21/2019 2:10	0.5917	-0.6833	4.6468	-0.9083	2.049	7.7	0.1131	1.9185
4/21/2019 2:11	0.5917	-0.8833	4.6842	-0.8	2.0731	7.7	0.1246	1.925
4/21/2019 2:12	0.5833	-0.5	4.7692	-0.2667	2.0493	7.75	0.122	1.9361
4/21/2019 2:13	0.6	-0.1833	4.6992	-0.6	2.0493	7.7455	0.111	1.9163
4/21/2019 2:14	0.6	-1.0833	4.502	-0.7167	2.0532	7.7	0.1249	1.8792
4/21/2019 2:15	0.6	-0.25	4.4548	-0.4917	2.0665	7.7	0.1103	1.875
4/21/2019 2:16	0.65	0.2333	4.4943	-0.475	2.0954	7.7	0.1169	1.8911
4/21/2019 2:17	0.6917	-0.1667	4.6132	-0.4417	2.0712	7.7	0.1283	1.896
4/21/2019 2:18	0.7	-1.2333	4.9483	-0.2	2.0554	7.7	0.1057	1.9263
4/21/2019 2:19	0.7	-0.0167	4.5787	-0.1417	2.0483	7.7	0.1206	1.9049
4/21/2019 2:20	0.6583	0.3333	4.6367	-0.225	2.0896	7.7	0.1279	1.8838
4/21/2019 2:21	0.6333	-0.4833	4.9033	-0.75	2.0669	7.7	0.1105	1.9232
4/21/2019 2:22	0.7	-0.65	4.643	-0.7917	2.0853	7.675	0.1285	1.9298
4/21/2019 2:23	0.7	-0.05	4.54	-0.4917	2.0631	7.6	0.1081	1.9642
4/21/2019 2:24	0.6667	-0.4833	4.8533	-0.4333	2.0836	7.6	0.124	1.9633
4/21/2019 2:25	0.6	-0.2	4.6907	-0.6	2.0708	7.5091	0.1195	1.9598

4/21/2019 2:26	0.5917	-0.4167	4.8833	-0.425	2.0704	7.5	0.1149	1.9677
4/21/2019 2:27	0.5167	-0.4	4.549	-0.0417	2.0855	7.5	0.1172	1.9436
4/21/2019 2:28	0.6	-0.4167	4.7063	-0.2	2.0903	7.5	0.1131	1.9603
4/21/2019 2:29	0.6	-0.6167	4.5837	-0.125	2.0956	7.5	0.1253	1.9383
4/21/2019 2:30	0.6	-0.95	4.5337	-0.2083	2.0949	7.6	0.1226	1.9428
4/21/2019 2:31	0.6	-0.6167	4.3527	-0.5083	2.0941	7.575	0.1182	1.9439
4/21/2019 2:32	0.6	0.0833	4.574	-0.4667	2.1058	7.5667	0.1282	1.936
4/21/2019 2:33	0.6167	-0.3167	4.5058	-0.4833	2.0947	7.6	0.1166	1.9378
4/21/2019 2:34	0.6917	-0.6667	4.687	-0.2917	2.1003	7.6	0.1161	1.9362
4/21/2019 2:35	0.7	-0.1833	4.7067	-0.825	2.0923	7.6	0.1284	1.913
4/21/2019 2:36	0.7	-0.1833	4.7725	-0.0333	2.098	7.6	0.1054	1.9213
4/21/2019 2:37	0.7	-0.3333	4.809	-0.2667	2.0792	7.6	0.1338	1.9378
4/21/2019 2:38	0.7	0.1	4.3623	-0.2333	2.0989	7.65	0.1116	1.9195
4/21/2019 2:39	0.7083	0.1	4.4332	-0.5667	2.1066	7.6455	0.1193	1.9838
4/21/2019 2:40	0.8	0.05	4.4837	-0.4167	2.0942	7.6583	0.1233	1.9771
4/21/2019 2:41	0.8	0.0833	4.6628	-0.225	2.1103	7.6	0.1085	1.95
4/21/2019 2:42	0.8	-0.2167	4.5455	-0.675	2.111	7.6	0.1242	1.9462
4/21/2019 2:43	0.8	-0.5667	4.6883	-0.2417	2.1115	7.6	0.1136	1.9531
4/21/2019 2:44	0.8417	-0.1333	4.6638	-0.7083	2.1288	7.6	0.1274	1.9586
4/21/2019 2:45	0.8917	-0.5167	4.7245	0.2583	2.0916	7.6	0.124	1.9633
4/21/2019 2:46	0.8083	-0.2333	4.6087	0.1333	2.1137	7.6	0.1102	1.9505
4/21/2019 2:47	0.8	-0.45	4.7872	-0.75	2.1109	7.6	0.1215	1.9311
4/21/2019 2:48	0.8	0.3333	4.7337	0.0333	2.1049	7.5091	0.1242	1.9353
4/21/2019 2:49	0.775	0	4.3953	0.0917	2.1143	7.5727	0.1188	1.9358
4/21/2019 2:50	0.7417	-0.5333	4.4765	-0.1167	2.1225	7.6	0.129	1.9856
4/21/2019 2:51	0.7583	-0.4333	4.584	-0.075	2.1156	7.6	0.1123	1.9684
4/21/2019 2:52	0.7583	-0.8333	4.7787	-0.4	2.0927	7.6	0.1203	1.9803
4/21/2019 2:53	0.7	-0.3833	4.575	-0.6917	2.119	7.6	0.1239	1.9718
4/21/2019 2:54	0.7	-0.5333	4.4477	-0.425	2.1459	7.6	0.1108	1.9907
4/21/2019 2:55	0.7	-0.8	4.5807	-0.5167	2.1441	7.6	0.1285	1.9901
4/21/2019 2:56	0.7	0.0833	4.881	-0.5417	2.1451	7.6	0.1092	1.995
4/21/2019 2:57	0.7	0.7	4.6863	-0.1333	2.1343	7.6	0.1265	1.9623
4/21/2019 2:58	0.7	-0.35	4.7475	-1.0167	2.1408	7.6	0.1225	1.933
4/21/2019 2:59	0.7	-0.5	4.7708	-0.3	2.1403	7.6	0.1232	1.9619
4/21/2019 3:00	0.7	-0.4	4.6068	-0.5	2.1418	7.6	0.1296	1.9333
4/21/2019 3:01	0.7	-0.05	4.6423	-0.2583	2.1269	7.6	0.1152	1.952
4/21/2019 3:02	0.7	0.05	4.6572	-0.6833	2.1458	7.6545	0.124	1.9628
4/21/2019 3:03	0.7	-0.3833	4.5588	-0.3083	2.1503	7.6364	0.1193	1.9673
4/21/2019 3:04	0.7	0.0833	4.636	-0.15	2.1547	7.6	0.1143	1.9625
4/21/2019 3:05	0.6917	0.0333	4.4308	-0.6167	2.1732	7.6	0.1231	1.9511
4/21/2019 3:06	0.65	-0.0833	4.6893	-0.2917	2.1242	7.6	0.1168	1.9565
4/21/2019 3:07	0.6	-0.3833	4.7553	0.4167	2.1364	7.6	0.1239	1.9643
4/21/2019 3:08	0.6	-0.2333	4.504	-0.6833	2.1802	7.6	0.1135	1.9488
4/21/2019 3:09	0.6917	-0.1667	4.7145	-0.2583	2.1528	7.6	0.1165	1.9916
4/21/2019 3:10	0.7	-0.2333	4.499	0.1667	2.1375	7.6	0.1243	1.9771
4/21/2019 3:11	0.7167	0.2167	4.4563	0	2.1524	7.6	0.1128	1.9823
4/21/2019 3:12	0.7	-0.15	4.5032	-0.55	2.1563	7.6	0.1275	1.9862

4/21/2019 3:13	0.7	0.1	4.5027	-0.625	2.1326	7.6	0.1118	2.0067
4/21/2019 3:14	0.7	-0.1667	4.7183	-0.275	2.1368	7.6	0.1222	2.0026
4/21/2019 3:15	0.7	-0.0833	4.7217	-0.2167	2.1503	7.6	0.1192	1.9883
4/21/2019 3:16	0.7	-0.7333	4.4338	-0.6667	2.1489	7.6	0.1131	1.9829
4/21/2019 3:17	0.7	-0.1667	4.614	-0.5667	2.1374	7.6	0.1226	1.9818
4/21/2019 3:18	0.7083	-0.6167	4.4712	-0.5333	2.1508	7.65	0.1185	2.0028
4/21/2019 3:19	0.7083	-0.5667	4.8575	-0.3833	2.1738	7.7	0.1177	1.9873
4/21/2019 3:20	0.7	-0.3167	4.792	-0.3	2.1393	7.7	0.1286	1.9671
4/21/2019 3:21	0.7	-0.7	4.5585	-0.2333	2.1415	7.7182	0.1116	1.9834
4/21/2019 3:22	0.7	-0.0333	4.8177	-0.7417	2.1281	7.7909	0.1188	1.9951
4/21/2019 3:23	0.7	-0.9333	4.5757	-0.3583	2.1462	7.7	0.1149	1.9867
4/21/2019 3:24	0.7	-0.4833	4.812	0.125	2.1453	7.7	0.1121	2.012
4/21/2019 3:25	0.675	-0.5167	4.5915	-0.3667	2.1137	7.7	0.1298	2.0287
4/21/2019 3:26	0.7	-0.0333	4.791	-0.4	2.134	7.7	0.106	2.0287
4/21/2019 3:27	0.7	-0.4333	4.7038	-0.475	2.1405	7.7	0.1151	1.9713
4/21/2019 3:28	0.6417	-0.5	4.5962	-0.8417	2.1449	7.7	0.1287	1.9551
4/21/2019 3:29	0.6333	-0.5167	4.3607	-0.325	2.1245	7.7	0.1167	1.9584
4/21/2019 3:30	0.675	-0.3833	4.4333	-0.7667	2.1348	7.6182	0.1122	1.9667
4/21/2019 3:31	0.7417	0.1	4.5643	-0.2417	2.1432	7.6	0.1161	2.0093
4/21/2019 3:32	0.8	-0.3833	4.6112	0.1	2.1327	7.6	0.1126	2.0134
4/21/2019 3:33	0.75	-0.5	4.6578	0.0667	2.1331	7.6	0.1195	2.0058
4/21/2019 3:34	0.7167	0.6333	4.439	-0.6667	2.1158	7.5909	0.1095	1.978
4/21/2019 3:35	0.7	-0.5333	4.5116	-0.0083	2.1569	7.5	0.122	2.0038
4/21/2019 3:36	0.7	-0.2833	4.7603	-0.85	2.1229	7.5	0.1232	1.9734
4/21/2019 3:37	0.6667	-0.0667	4.8106	-0.4917	2.1389	7.5	0.1152	1.9757
4/21/2019 3:38	0.6	-0.4667	4.7403	-0.2667	2.1474	7.5	0.1309	1.9783
4/21/2019 3:39	0.6	-0.2333	4.4311	-1.1083	2.1443	7.575	0.1141	1.9851
4/21/2019 3:40	0.6	-0.1167	4.687	0.275	2.1638	7.6	0.125	1.995
4/21/2019 3:41	0.5833	0.4167	4.7467	-0.8917	2.1248	7.6	0.1191	1.9873
4/21/2019 3:42	0.5417	-0.3167	4.5539	-0.3833	2.1564	7.6	0.1134	2.0188
4/21/2019 3:43	0.6	-0.4167	4.6143	0.0667	2.16	7.6	0.12	1.997
4/21/2019 3:44	0.6167	-0.6167	4.6283	-0.4333	2.1608	7.5818	0.1078	2.0078
4/21/2019 3:45	0.7	-0.25	4.698	-0.425	2.1693	7.525	0.1227	1.99
4/21/2019 3:46	0.6083	0.0667	4.9607	-0.4833	2.1833	7.6	0.1205	1.9989
4/21/2019 3:47	0.6	-0.2833	4.6463	-0.7083	2.1627	7.6273	0.1094	1.992
4/21/2019 3:48	0.6	-0.2	4.7395	-0.6917	2.1424	7.7	0.1251	2.0162
4/21/2019 3:49	0.5583	-0.5167	4.5226	-0.5833	2.1398	7.7	0.1118	2.0329
4/21/2019 3:50	0.5	-0.2333	4.6643	-0.1833	2.1451	7.7	0.1158	2.0508
4/21/2019 3:51	0.5	0.1	4.5628	-0.1917	2.149	7.7	0.1193	2.0474
[truncated: 679,120 more chars]
